# A revision of the Old World Black Nightshades (Morelloid clade of *Solanum* L., Solanaceae)

**DOI:** 10.3897/phytokeys.106.21991

**Published:** 2018-07-25

**Authors:** Tiina Särkinen, Peter Poczai, Gloria E. Barboza, Gerard M. van der Weerden, Maria Baden, Sandra Knapp

**Affiliations:** 1 Royal Botanic Garden Edinburgh, 20A Inverleith Row, Edinburgh EH3 5LR, United Kingdom; 2 Botany Unit, Finnish Museum of Natural History, University of Helsinki, P.O. Box 7, FI-00014 Helsinki, Finland; 3 Instituto Multidisciplinario de Biología Vegetal (CONICET-Universidad Nacional de Córdoba), Casilla de Correo 495, 5000 Córdoba, Argentina; 4 Experimental Garden, Radboud University, Faculty of Science Box 49, P.O. Box 9010, 6500 Nijmegen, The Netherlands; 5 Max-Planck Odense Center on the Biodemography of Aging and Department of Biology, University of Southern Denmark, Campusvej 55, DK-5230 Odense M, Denmark; 6 Department of Life Sciences, Natural History Museum, Cromwell Road, London SW7 5BD, United Kingdom

**Keywords:** Africa, Asia, Australia, Eurasia, Europe, Madagascar, Pacific, polyploidy, *Solanum nigrum* complex, supervegetables, weeds

## Abstract

The Morelloid clade, also known as the black nightshades or “Maurella” (Morella), is one of the 10 major clades within *Solanum* L. The pantropical clade consists of 75 currently recognised non-spiny herbaceous and suffrutescent species with simple or branched hairs with or without glandular tips, with a centre of distribution in the tropical Andes. A secondary centre of diversity is found in Africa, where a set of mainly polyploid taxa occur. A yet smaller set of species is found in Australasia and Europe, including *Solanum nigrum* L., the type of the genus *Solanum*. Due to the large number of published synonyms, combined with complex morphological variation, our understanding of species limits and diversity in the Morelloid clade has remained poor despite detailed morphological studies carried out in conjunction with breeding experiments. Here we provide the first taxonomic overview since the 19^th^ century of the entire group in the Old World, including Africa, Asia, Australia, Europe and islands of the Pacific. Complete synonymy, morphological descriptions, distribution maps and common names and uses are provided for all 19 species occurring outside the Americas (i.e. Africa, Asia, Australia, Europe and islands of the Pacific). We treat 12 species native to the Old World, as well as 7 taxa that are putatively introduced and/or invasive in the region. The current knowledge of the origin of the polyploid species is summarised. A key to all of the species occurring in the Old World is provided, together with line drawings and colour figures to aid identification both in herbaria and in the field. Preliminary conservation assessments are provided for all species.

## Introduction

*Solanum* L., with approximately 1,400 species, is one of the largest genera of flowering plants (Frodin 2004). The genus poses a taxonomic challenge not only due to its large size, but also due to the 6,931 published names, many of which are associated with the cultivated and widespread species of the genus, including the potato (*S. tuberosum* L.), tomato (*S. lycopersicum* L.) and the eggplant (*S. melongena* L.). Recent taxonomic and molecular systematic efforts (http://www.solanaceaesource.org) have identified major clades within *Solanum* (e.g. Weese and Bohs 2007), clarified relationships and the monophyly of previously recognised morphological sections (e.g. Stern et al. 2011) and provided taxonomic revisions for major clades with keys for species identification (e.g. Knapp 2013).

**Figure 1.**
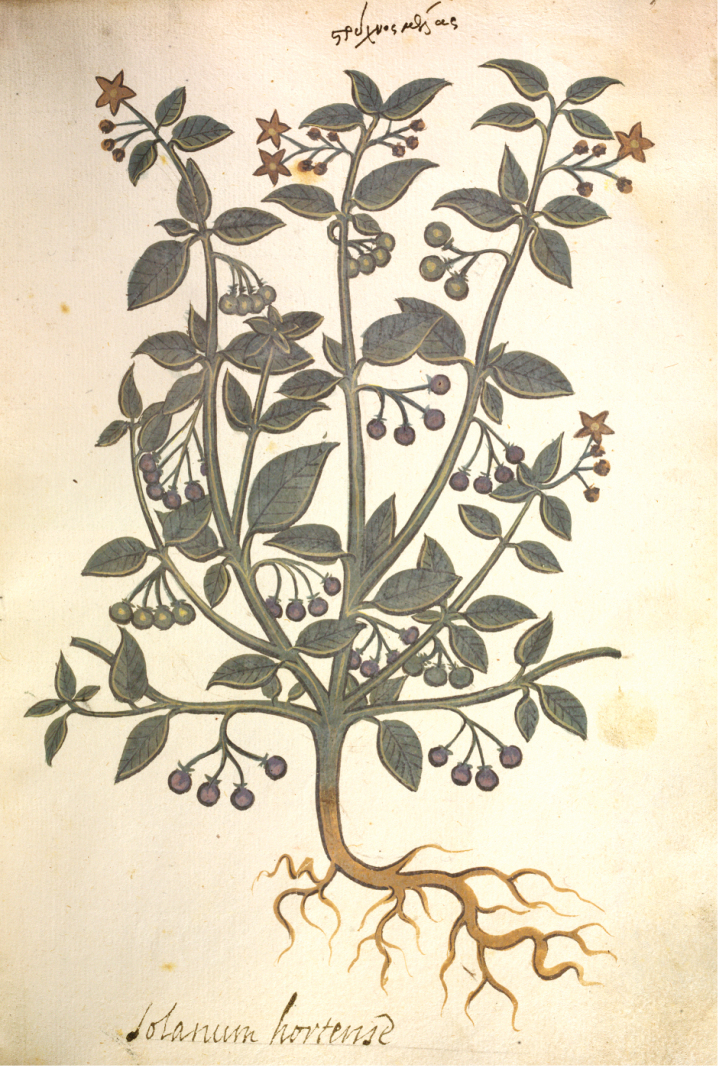
“Solanum Hortense” from Joseph Banks' handcoloured copy of Dioscorides *De Materia Medica*; the flowers were originally painted white but have turned brown because a lead paint was used. This copy dates from the 15^th^ century. Copyright The Trustees of the Natural History Museum, London. Reproduced with permission.

**Figure 2.**
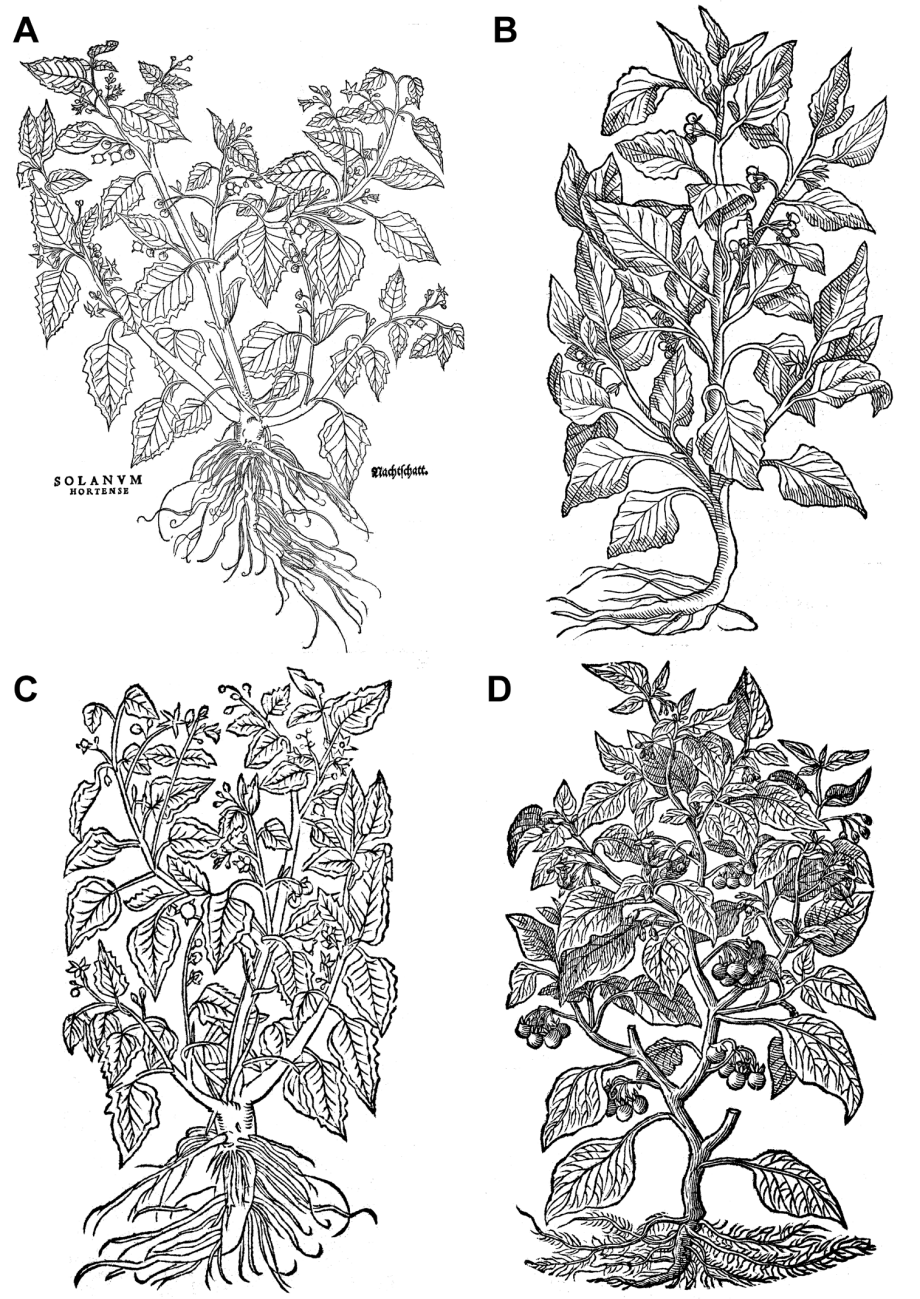
“Solanum Hortense” as illustrated in early herbals **A** Fuchs (1542) *Neu Kreuterbuch* **B** Matthioli (1544) *Di Pedacio Dioscoride Anazarbeo* **C** Dodoens (1563) *Crüydeboeck* **D** Dodoens (1583) *Stirpium historiae pemptades sex*. Courtesy of the Library of the Natural History Museum, London.

**Figure 3.**
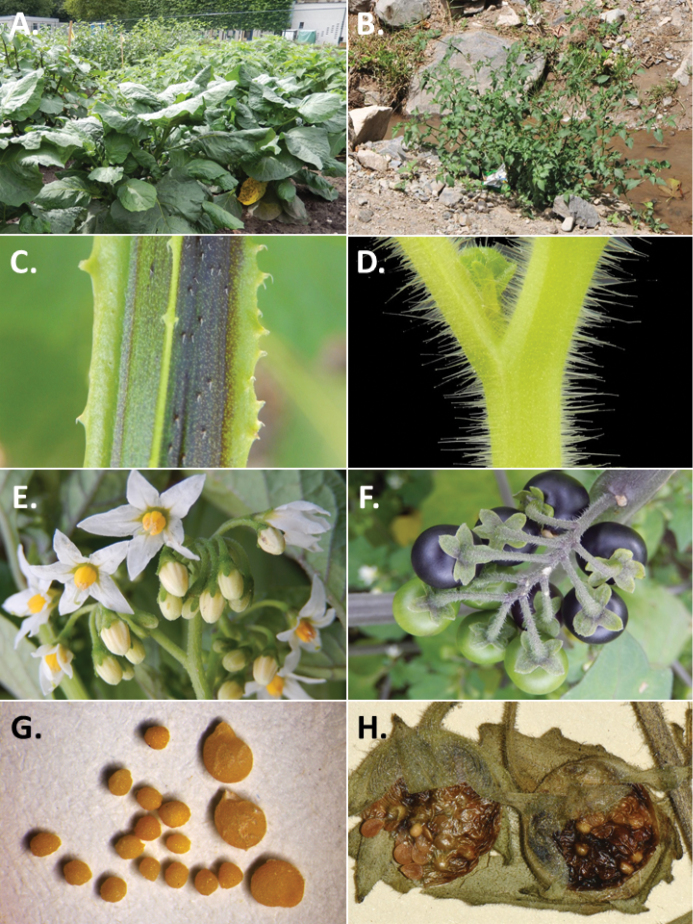
Morphology of the Old World species of the Morelloid clade of *Solanum* **A** Many species are cultivated for leaves and fruits (*S. scabrum*, NIJ994750012) **B** Most species show preference of disturbed, dry habitats in the wild (*S. americanum*, Knapp et al. 10210) **C** Strongly ridged and often purple coloured stems present in many species (*S. tarderemotum*, NIJ 2010-5) **D** Glandular hairs present in some species of the Morelloid clade (*S. memphiticum*, A34750473) **E** Typical stellate, white flowers with spreading or reflexed corolla lobes (*S. tarderemotum*, A14750151) **F** Fleshy round berries that are black, green, yellow, orange or red depending on the species (*S. nigrum*, A44750150) **G** Stone cells (also known as sclerotic granules or brachysclerids) are found in the fruits of most species of the Morelloid clade and are round in shape as compared to the tear-drop shaped or ellipsoid seeds (*S. umalilaense*, A24750133) **H** Stone cells are often easy to see in herbarium specimens (e.g. *S. sarrachoides*, *Blom s.n.* BM001207745). Photos by S. Knapp and G. van der Weerden.

The Morelloid clade of *Solanum*, also known as the black nightshades or “Maurella” (Morella), is amongst the 10 robustly supported major clades within *Solanum* (Bohs 2005; Weese and Bohs 2007). This group, that includes the type species of the genus, *S. nigrum* L., has a long taxonomic history dating back to the early herbals (Figures 1–2). The group has traditionally been considered difficult, due in part to the weedy nature of its species and its worldwide distribution. The clade comprises 75 currently recognised non-spiny herbaceous and suffrutescent species with simple or branched hairs with or without glandular tips (glandular or eglandular) and inflorescences usually arising from the internodes (Figure 3; Särkinen et al. 2015a). Ploidy level varies from diploid to hexaploid within the group (e.g. Edmonds and Chweya 1997; Moyetta et al. 2013; see Table 1), in part contributing to the difficulties in its taxonomy. While taxonomic revisions of the smaller New World sections within the Morelloids have recently been published (del Vitto and Petenatti 1999; Barboza 2003; Barboza and Hunziker 2005), the group in its entirety has not been revised since the 19^th^ century (Dunal 1852).

**Table 1.** Chromosome counts for species of the Morelloid clade of *Solanum*. For references, see individual species treatments.

**Species**	**Haploid chromosome number**
*Solanum alpinum* Zoll. & Moritzi	–
*Solanum americanum* Mill.	12 (2n=2x=24)
*Solanum chenopodioides* Lam.	12 (2n=2x=24)
*Solanum furcatum* Dunal	36 (2n=6x=72)
*Solanum hirtulum* A.Rich.	–
*Solanum memphiticum* J.F.Gmel.	24 (2n=4x=48)
*Solanum nigrum* L.	36 (2n=6x=72)
*Solanum nitidibaccatum* Bitter	12 (2n=2x=24)
*Solanum opacum* A.Braun & C.D.Bouché	36 (2n=6x=72)
*Solanum palitans* C.V.Morton	12 (2n=2x=24)
*Solanum pseudospinosum* C.H.Wright	24 (2n=4x=48)
*Solanum pygmaeum* Cav.	12 (2n=2x=24, some supernumerary anomalies, see species description)
*Solanum retroflexum* Dunal	24 (2n=4x=48)
*Solanum sarrachoides* Sendtn.	12 (2n=2x=24)
*Solanum scabrum* Mill.	36 (2n=6x=72)
*Solanum tarderemotum* Bitter	24 (2n=4x=48)
*Solanum triflorum* Nutt.	12 (2n=2x=24)
*Solanum umalilaense* Manoko	24 (2n=4x=48)
*Solanum villosum* Mill.	24 (2n=4x=48)

General overviews of Black nightshade taxonomy and systematics have been published (Edmonds 1977, 1978, 1979a; Chakrabarti and Sharma 1994), including geographically focused taxonomic treatments for South America (Edmonds 1972), North America (Schilling 1981), Australia (Henderson 1974), Africa (Edmonds and Chweya 1997; Olet 2004; Manoko 2007; Edmonds 2012), Europe (Wessely 1960) and the Indian subcontinent (Schilling and Andersen 1990) and detailed cytological, molecular and morphological studies (Saarisalo-Taubert 1967; Venkateswarlu and Rao 1972; Edmonds 1982, 1983, 1984a; Ganapathi and Rao 1986a, 1986b, 1986c, 1986d, 1987a, 1987b; Nandhini and Paramaguru 2014). These studies have greatly enhanced our understanding of the complex morphological and ploidy level variation present in the group, but much taxonomic work remains within the Morelloids especially in South America, where more than half of the known Morelloid species are found (Barboza et al. 2013).

**Figure 4.**
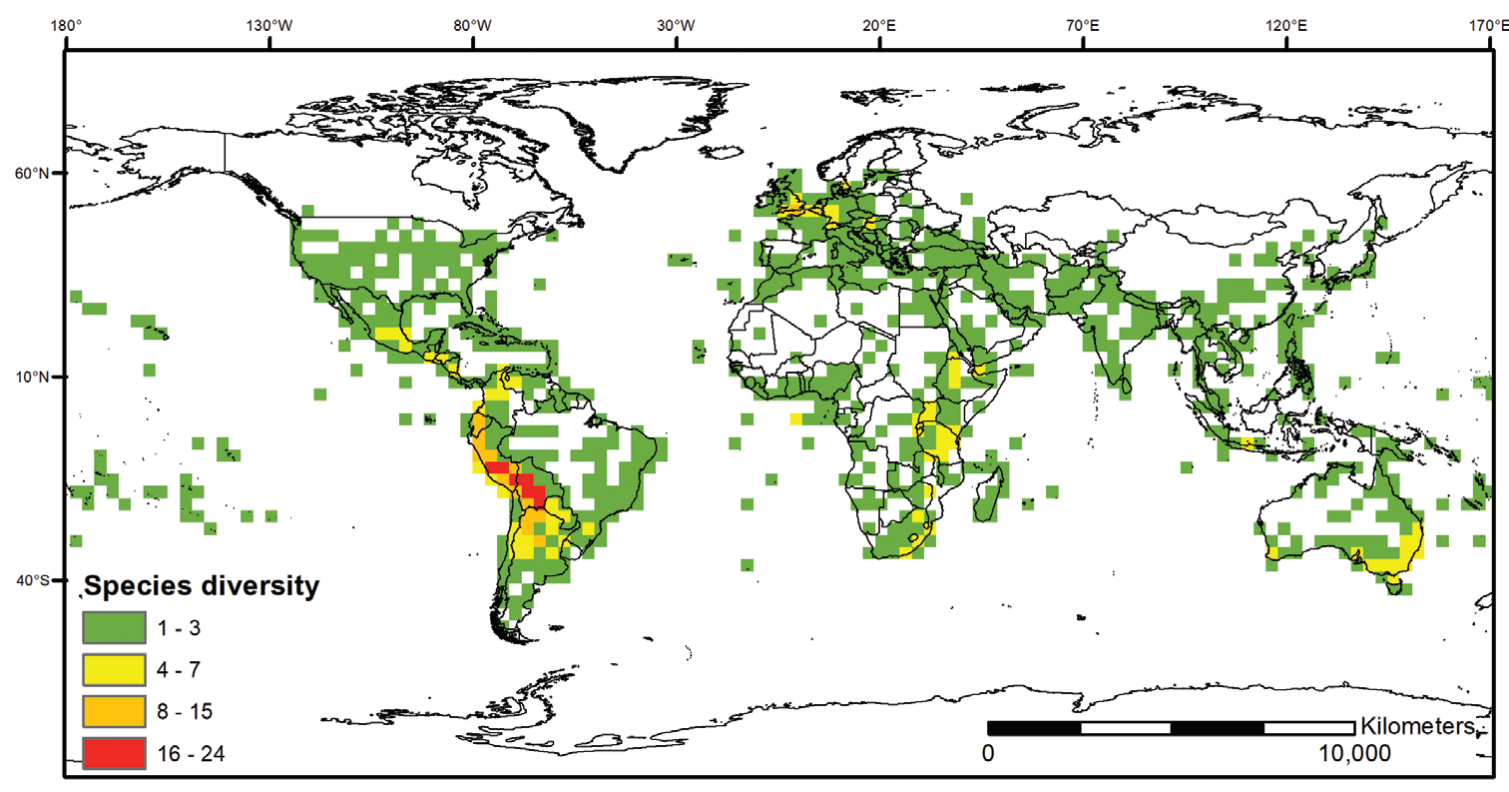
Distribution of the Morelloid clade of *Solanum*, showing number of species per 3 degree grid cells (ca. 300 km^2^) based on native and non-native records.

Within the Old World, Africa is a centre of species diversity with a total of 12 species (10 of them native), with fewer species found in Australasia (Figure 4; Tables 2–6). Many of the Old World species have spread to the New World with humans, such as *S. nigrum*, *S. scabrum* and *S. villosum*, while five species currently established in the Old World are widespread weeds originally native to South America but have been taken in the reverse direction, often as contaminants of wool; *S. chenopodioides*, *S. nitidibaccatum*, *S. pygmaeum*, *S. sarrachoides* and *S. triflorum* (Tables 2–6; Särkinen et al. 2015a).

An overview of the entire section in the Old World, including a revision of the nomenclature and typification of the more than 370 names associated with these taxa, has never been done but is needed in order to provide identification tools for these difficult and morphologically very similar species. Here we provide a taxonomic revision of all 19 species of the Morelloid clade (black nightshades) occurring in the Old World based on a detailed morphological study; we include native and introduced taxa. This is part of our molecular systematic and taxonomic work focusing on producing a monographic treatment of the entire Morelloid clade which has to date focused on understanding species diversity and delimitation in the New World (e.g. Barboza et al. 2013; Särkinen et al. 2013, 2015a, b).

## History, taxonomy and relationships of the Morelloid clade

The European species of black nightshades were known to the ancient Greeks and Romans, who used them medicinally. In the 1^st^ century AD, Pliny the Elder (Riley 1855) called the plant “cucubalus, strumus or strychnon” [translated from the Greek] and documented its use against stings, wounds and lumbago. Dioscorides, whose *De Materia Medica* was the primary source for much of medicine in Europe until after the Middle Ages, illustrated the plant (Figure 1) and advocated it for its astringent and cooling properties. Later European herbals, building upon the work of Dioscorides (e.g. Fuchs 1542, 1543; Matthioli 1544; Dodoens 1554, 1563) all treated *S. nigrum* L. as “Solanum Hortense” and recognised it as a native plant and distinct from others which they considered similar plants such as “Halicacabum [Solanum Halicacabum]” (*Alkekengi officinarum* Moench), “Solanum somniferum” (*Withania somnifera* (L) Pauq.) or “Solanum furiosum” (probably *Atropa bella-donna* L.). Fuchs (1542) listed French (Morelle) and German (Nachtschatten) common names, along with the name used in medicine “Solatrum”, stating that the name “Nigrum” given to this plant came from the berries and not from the leaves. It is clear that authors in continental Europe recognised the different fruit colours as the same plant; Matthioli (1544) describes the fruit as “fructu rotundo, uiridi, qui post maturitatem nigricat, aut fuluefcit” (round green fruit, black or yellowish-brown at maturity). Most hand-coloured editions of these early works (e.g. Fuchs 1543; Dodoens 1554) have the berries painted black and the plants in the woodcuts are identifiable as *S. nigrum* with the sepals more or less appressed to the berries (see species treatments). Dodoens (1583) described the fruit more clearly (“per initia virentes, maturae veròe, aut nigricantes, aut rubentes, aut luteo coloris” [at the beginning green, when mature black, or red, or yellow coloured]) and his illustration is clearly of *S. villosum* with its characteristic strongly reflexed and longer sepals in fruit (Figure 2).

The first herbal written in English was by John Gerard. He (Gerard 1597: 338–340) extensively treated the Garden Nightshade (as “Solanum Hortense” = *S. nigrum*) and documented its uses (“…Physicians do worthily use it, and that seldom as a nourishment, but always as a medicine”) and alluded to the confusion of nightshades by earlier authors (“Apuleis, amongst the confused names of Nightshade, who comprehending all the kinds of Nightshade together in one chapter, being so many, hath strangely and absurdly confounded their names.”). He suggested that it was safe and not poisonous – “Neither the juice hereof nor any other part is usually given inwardly, yet it may without any danger.”

By the mid-15^th^ century, black nightshades were already becoming confused with the more toxic deadly nightshade (*Atropa bella-donna*). In his Complete herbal, first published in 1653, Nicholas Culpeper called his “Common nightshade” a “cold Saturnine plant” and warned “Have a care you mistake not the deadly nightshade for this [black nightshade]; if you know it not, then you may let them both alone” (Culpeper 1814). He described its weedy nature well; “It groweth wild in this kingdom, and in rubbish, the common paths and sides of hedges, in fields, and also in gardens without any planting.” Black nightshades were given the common name “Petty Morel” (from the French petite morelle) and *Atropa* was called “Great Morel” (Grieve 1931). Nightshades’ reputation for being poisonous developed over the succeeding centuries in Europe, despite the use of these plants in local cultures across their range (see Uses below).

Linnaeus (1753) broadly circumscribed *S. nigrum* and divided it into six infraspecific taxa, many of which were based on the plates in Dillenius’s *Hortus Elthamensis* (Dillenius 1732). He recognised the European *S. nigrum* (as var. vulgare), *S. villosum* (as S. nigrum var. villosum), *S. americanum* (as S. nigrum var. patulum) and included the African cultivated species *S. scabrum* (as S. *nigrum* var. guineenense); but he had not seen material of other species treated here (see individual species treatments for details). He clearly recognised all these taxa as very similar and as variants of a worldwide species; his diagnosis reads “Habitat in Orbis totius, cultis” [Habitat in all the world, cultivated]. He also noted many of these looked like mixtures (“Tot varietates β, γ, δ, ε, ζ videntur esse hybridae proles”). In the *Species plantarum* (Linnaeus 1753), he did not cite many of the works based on non-European plants (e.g. Piso 1648; Rheede von Draakestein 1689), which he had previously cited in *Hortus cliffortianus* (Linnaeus 1737) and, in the Clifford herbarium (BM), only specimens of *S. nigrum*, *S. scabrum* and *S. villosum* are preserved.

In the sixth edition of his *Gardener’s dictionary*, Philip Miller used Linnaean binomials for the first time (see Stearn 1974). In this work, he (Miller 1768) described seven members of the Morelloid clade, five of these as new names (*S. villosum* Mill., *S. luteum* Mill., *S. rubrum* Mill., *S. americanum* Mill., *S. scabrum* Mill.). He did not recognise infraspecific taxa, but also did not indicate he was raising Linnaeus’s varieties to species level.

Lamarck (1794) recognised seven taxa, including some not known to either Miller or Linnaeus, such as *S. radicans* L.f. and *S. corymbosum* Jacq. (members of the clade belonging to the Radicans group, see Särkinen et al. 2015a). He additionally described *S. chenopodioides* Lam., from material said to be from “île de France” (Mauritius, but see species description) and *S. triangulare* Lam. based in part on an illustration from Rumphius (1750, = *S. scabrum*). Some of these early authors re-used epithets (e.g. *villosum* used by both Miller and Lamarck), but it is not clear whether they were referring to earlier names or not; the principle of priority had not yet become established for botanical naming (see Knapp et al. 2004).

The name for the Morelloid clade is derived from Dunal’s (1813: 119) un-ranked group “Maurella” that included herbaceous or subherbaceous species with entire leaves. He included 15 species, all of which are still considered members of the clade. In his *Solanorum synopsis* (Dunal 1816), he maintained this group, adding to it taxa described by himself and others, most of which (with the exception of *S. quadrangulare* Thunb. = *S. africanum* Mill., a member of the Dulcamaroid clade, see Knapp 2013) are still considered related. Dumortier (1827) used this group for his conspectus of the Belgian species, but with Dunal’s spelling changed to “Morella”. In general, the concept of Morella was narrow and included only those species later recognised as members of Solanum sect. Solanum, but did not include species now recognised as part of the more broadly defined group (Bohs 2005; Weese and Bohs 2007; Särkinen et al. 2013, 2015a). In the *Prodromus*, Dunal (1852) paid little attention to earlier names and erected an entirely new framework for *Solanum* mostly composed of *gradi ambigui* (names of ambiguous rank). Morella, however, was one of the names he continued to use. Within it, Dunal (1852) recognised two groups based on the inflorescence position, “Morellae spuriae” (six spp.) and “Morellae verae” (54 spp.). Circumscription of “Morella” remained obscure and loose during most of the 19^th^ and 20^th^ centuries, with many herbaceous non-spiny taxa treated as members of the group, resulting in many names associated with the Morelloid clade. Many of these names do not belong to the clade as now recognised based on phylogenetic data (Bohs 2005; Weese and Bohs 2007). An extreme example of this is *S. stipuloideum* Rusby from the morphologically distinct Potato clade (Spooner and Knapp 2013), which was assigned to Solanum sect. Solanum in the original description (Rusby 1907).

The very numerous treatments of these species in European floristic works (see species descriptions) usually did not specifically treat these taxa as belonging to infrageneric groups. Often these species were the only solanums treated in these floristic works, and they were often called “black nightshades” (e.g. Opiz 1843). Von Wettstein (1895) followed Dunal’s scheme in his treatment of the European species, as did Dammer (1906) and Bitter (1917, 1919) for Africa and Asia. Bitter (1917) was the first to explicitly recognise the group at the sectional level. Following the rules on use of autonyms, Seithe (1962) was the first to re-name the group containing the type species of the genus (*S. nigrum*) Solanum section Solanum; she also recognised sects. *Campanulisolanum* Bitter, *Chamaesarachidium* Bitter and *Episarcophyllum* Bitter (all groups confined to the New World, see Barboza 2003; Barboza and Hunziker 2005), now considered part of the larger Morelloid clade (Särkinen et al. 2015a). Her sectional names were followed by Danert (1967, 1970) with little change. D’Arcy (1972) lectotypified the infrageneric groupings in *Solanum* and provides an overview of the history of these infrageneric names.

Within the Morelloids, four well-supported clades have been recognised based on a detailed molecular phylogenetic study (Särkinen et al. 2015a). Prior to the use of molecular characters in phylogenetic analysis (e.g. Bohs 2005; Weese and Bohs 2007), the Morelloid clade thus defined was not recognised as a natural group. These clades loosely correspond to the previously recognised morphological sections: (1) the Radicans clade which comprises four of the species (but does not include the type species, *S. triflorum* Nutt.) of Solanum sect. Parasolanum A.Child (Bohs 2005); (2) the Episarcophyllum clade that includes most species of Solanum section Episarcophyllum Bitter; (3) the Chamaesarachidium clade that includes two species of Solanum section Chamaesarachidium Bitter; and finally the largest group (4) the Black nightshade clade, that includes all species of the traditional Solanum sect. Solanum. The first three clades are restricted to the New World, while most species of the Black nightshade clade occur in the New World but with a secondary centre of diversity in the Old World that is treated in this monograph.

The number of taxa included in this clade has not been clear, in part because it contains many widespread and morphologically variable species and has always been considered difficult. Although Edmonds (1972) suggested estimates of species richness had been exaggerated in the group and provided relatively low estimates of species numbers, our ongoing work in the group shows the clade includes ca. 75 species that are mostly restricted to South America (Särkinen et al. 2015a). Nineteen black nightshade species are found in the Old World, of which twelve are native there (four narrowly endemic) and not found in the Americas (Table 2; Särkinen et al. 2015a); the other seven are introductions from the New World.

**Table 2.** Status and general distribution of Old World members of the Morelloid clade. Narrowly endemic taxa are shown in boldface Type.

**Species**	**Status in the Old World**	**Distribution**
***Solanum alpinum* Zoll. & Moritzi**	**Native**	**Indonesia**
*Solanum americanum* Mill.	Native?	Circumtropical
*Solanum chenopodioides* Lam.	Introduced	Southern South America; sporadically introduced elsewhere
*Solanum furcatum* Dunal	Introduced	Southern South America; sporadically introduced elsewhere
***Solanum hirtulum* A.Rich.**	**Native**	**Ethiopia**
*Solanum memphiticum* J.F.Gmel.	Native	Eastern Africa
*Solanum nigrum* L.	Native	Europe, northern Africa, Eurasia and southeast Asia; sporadically introduced elsewhere
*Solanum nitidibaccatum* Bitter	Introduced	Southern South America; widely introduced elsewhere
*Solanum opacum* A.Braun & C.D.Bouché	Native	Australasia and the Pacific
*Solanum palitans* C.V.Morton	Introduced	Southern South America; sporadically introduced elsewhere
***Solanum pseudospinosum* C.H.Wright**	**Native**	**Cameroon, Equatorial Guinea (Bioko)**
*Solanum pygmaeum* Cav.	Introduced	Southern South America; sporadically introduced elsewhere
*Solanum retroflexum* Dunal	Native	Southern Africa; sporadically introduced elsewhere from cultivation (see species discussion)
*Solanum sarrachoides* Sendtn.	Introduced	Southern South America; sporadically introduced elsewhere
*Solanum scabrum* Mill.	Native	Africa; introduced through cultivation elsewhere
*Solanum tarderemotum* Bitter	Native	Africa
*Solanum triflorum* Nutt.	Introduced	Southern South America and North America; introduced elsewhere
***Solanum umalilaense* Manoko**	**Native**	**Tanzania**
*Solanum villosum* Mill.	Native	Europe, northern Africa, Eurasia and Africa; sporadically introduced elsewhere

Numerical taxonomic studies have been undertaken in order to resolve species relationships, parental origin of polyploids and species delimitation in these species (Soria and Heiser 1961; Heiser et al. 1965; Edmonds 1978), but the power of these methods has remained limited due to the complex and often overlapping morphological variation between the closely related species. Species of black nightshades show large amounts of morphological variation, especially in growth form, leaf morphology and indumentum.

Modern regional taxonomic treatments of the Old World members of the Morelloid clade have been done for Europe (Hawkes and Edmonds 1972), India (Ganapathi and Rao 1986a, 1988; Schilling and Andersen 1990), Africa and Madagascar (Bitter 1912a, 1912b, 1913a, 1921, 1923; Jaeger 1985; Bukenya and Hall 1988; Bukenya 1993; Bukenya and Carasco 1995; Edmonds and Chweya 1997) and Australia (Henderson 1974). Edmonds and Chweya (1997) remains the most thorough account of the black nightshades in the Old World, but includes only some of the species known to occur in Africa and does not treat species from Asia or those known from Australasia and the Pacific. Regional floristic treatments for regions of Africa have recently been published with detailed descriptions of local morphological variation (Edmonds 2005, 2006a, b, 2012). Schilling and Andersen (1990) clarified some of the nomenclatural problems and issues relating to misapplication of names in the Indian subcontinent in relation to the most commonly occurring species in the region.

The Old World species of the Morelloid clade do not form a monophyletic group. Phylogenetic analysis using plastid DNA sequence data (Särkinen et al. 2015a; T. Särkinen et al. in prep.) indicates that the native Old World polyploid species (*S. hirtulum*, *S. memphiticum*, *S. nigrum*, *S. opacum*, *S. retroflexum*, *S. scabrum*, *S. tarderemotum*, *S. villosum*, *S. umalilaense*) form a distinct group, to which *S. alpinum* does not belong. Polyploids from Africa are not each other’s closest relatives. Other species occurring in the Old World are members of distinct South American clades.

## Morphology

### Habit and stems

Members of the Morelloid clade are either herbs or shrubs; species occurring in the Old World range from annual (e.g. *S. triflorum*) to short-lived perennials (e.g. *S. retroflexum*, *S. tarderemotum*) and most are herbaceous, although some species can develop woody bases and appear to be somewhat shrubby (e.g. *S. villosum*). Stems are usually weak and occasionally somewhat scrambling, but can reach 2+ m in height. Plants of all species usually have herbaceous upper stems, even if the base is woody. The stems can be hollow (drying flattened, e.g. *S. tarderemotum*) or solid (e.g. *S. americanum*, *S. villosum*); this can be a useful character for identification of herbarium specimens.

Sympodial growth is characteristic of Solanaceae, giving the stems a typical “zig-zag” appearance; details of sympodial structure have proved useful for infrageneric classification within *Solanum* (Child and Lester 1991; Knapp 2002a). Vegetative growth is initially monopodial, but with the onset of flowering becomes sympodial. The inflorescence is developmentally terminal and stem continuation is initiated in the axil of the leaf below each inflorescence. Each lateral shoot with alternate leaves arranged in a 1/3 phyllotaxic spiral and a terminal inflorescence is termed a sympodial unit. In some cases, when the axes of sympodial units are fused, the inflorescences appear to originate laterally from the middle of an internode; when growth of the axes is suppressed, the leaves appear paired (geminate) at a node (Danert 1958). All of the members of the Morelloid clade have difoliate sympodial units with leaves sometimes strongly paired (geminate) at the nodes and the inflorescences often arise internodally through axis fusion (Danert 1958, 1967). Occasionally inflorescences appear to be opposite the geminate leaves (e.g. *S. hirtulum*) especially on very young shoots (e.g. *S. americanum*).

“Spinose” processes are common on herbaceous stems in many species of black nightshades. They usually occur along the angles of upper parts of larger stems and are often decurrent from leaf bases. These are not true prickles, like those found in the “spiny” solanums (Leptostemonum clade, see Knapp and Vorontsova 2016) but are similar in that they are outgrowths of the epidermis and are usually associated with trichomes as the enlarged basal portions of stem trichomes that have fallen off. They have been used to differentiate species in this group, but these structures are variable within species where they do occur and even within stems on a single plant. In addition, they often change markedly in appearance when plants are pressed and dried. Their absence, however, can be diagnostic when combined with other characters.

### Leaves

Species of the Morelloid clade have simple leaves that are generally elliptic or ovate in outline. *Solanum retroflexum* has a distinctive rhomboid leaf shape and *S. scabrum* leaves are usually broadly ovoid with long petioles. As with other vegetative characters in this group, leaf morphology can be extremely variable within a species or even in a single plant. Many of the infraspecific names in *S. nigrum* are based on variation in leaf morphology, particularly with respect to lobing of the margins.

Leaf margins vary from entire to quite deeply sinuate and lobed. Most populations of *S. triflorum* have deeply pinnatifid leaves, but a wing of leaf blade is always present along the midrib. Other species have variously entire or toothed margins and often the teeth occur only in the basal half to third of the leaf blade. The leaf blades are usually somewhat decurrent on to the petiole and the leaf base is cuneate to attenuate. Leaf apices are acute to attenuate, but vary considerably within species.

Petiole length to some extent is related to leaf size and, on individual plants, larger leaves always have longer petioles. As *Solanum scabrum* tends to have long petioles with little decurrent leaf blade tissue, this character can be helpful in distinguishing it from *S. nigrum* or from the sympatric *S. tarderemotum*.

### Pubescence

Trichomes in species of the Morelloid clade are simple or branched (e.g. *S. pallidum* Rusby of the Andes), but never stellate (Seithe 1962; Roe 1971). Old World species have only simple trichomes, these being usually 1-6-celled and uniseriate. Occasionally the trichome base is enlarged with the lowermost cell much larger than more distal cells and these enlarged bases persist as “pseudospines” on stems (see above). Much importance has been placed on differences in density of pubescence as a taxonomic character (e.g. in Europe the densely pubescent plants of *S. nigrum* sometimes recognised infraspecifically as var. or subsp. schultesii in European floras), but pubescence within taxa is continuously variable and apparently also related to environment, with plants growing in sunny sites more densely pubescent.

The presence or absence of glandular trichomes has also been previously treated as taxonomically significant (see Edmonds 1977, 1979b), with glandular and eglandular morphotypes of the European species *S. nigrum* and *S. villosum* being treated as separate subspecies or varieties (see Edmonds 2012). Manoko (2007; Manoko et al. 2008) showed that this character did not correspond to monophyletic groups in his AFLP analyses of *S. nigrum* or *S. villosum*. Several species amongst the Old World black nightshades have both glandular and eglandular populations and individuals (e.g. *S. alpinum*, *S. nigrum*, *S. tarderemotum*, *S. villosum*). Seithe (1962, 1979) showed that in most *Solanum* species, glandular trichomes are found on cotyledons and hypocotyls of seedlings and are lost as plants mature; she suggested that species with glandular trichomes were more “primitive”. It is equally probable that the retention of glandular tips on trichomes is a simple paedomorphic character and that it has little taxonomic significance if not correlated with other characteristics. Modern developmental work has not been undertaken with morelloid trichomes, but work has been done with the glandular trichomes of tomatoes and their relatives (e.g. Bergau et al. 2015). These studies show that these trichomes are architecturally very invariable and suggest they play a role in pest defence through release of metabolites in response to insect contact. Local ecological and herbivore pressures may also play a role in the presence or absence of glandular trichomes in the morelloids; this may help explain the highly heterogenous distributions of glandular and eglandular individuals in the polymorphic species in this group.

### Inflorescences

As with all species of *Solanum*, the inflorescence of members of the Morelloid clade is developmentally terminal and later overtopped by the leading axillary shoot so that it appears lateral. The basic structure is an unbranched or variously branched scorpoid cyme. Most members of the black nightshades have unbranched (simple) or merely furcate (once-branched) inflorescences, but in populations cultivated for fruit (e.g. *S. scabrum*, *S. umalilaense*, *S. villosum*), complex branching can occur, presumably through human selection for higher fruit production. Populations of *S. americanum* from Hainan Island in southern China with highly branched inflorescences have been described as a distinct species (*S. merrillianum*), but we consider these to be variants of the common *S. americanum* (see description of *S. americanum*). The degree of inflorescence branching in species of the group may also depend upon plant or inflorescence age (e.g. *S. tarderemotum*). In all *Solanum* species, the inflorescence expands from the tip producing flowers in a proliferating manner (Lippman et al. 2008).

All members of the group have distinct peduncles, usually somewhat longer than the distal flower-bearing portion, but inflorescence length and flower number vary both between and within species. Many species in the group have what are termed “sub-umbellate” inflorescences, where the flower-bearing rhachis is very short and the pedicels are all very closely spaced and congested at the very tip of the inflorescence. We use the term sub-umbelliform in the species descriptions for the extreme cases of this flower clustering. This inflorescence is not a true umbel, but is described as such in much previous literature, usually as an umbellate or subumbellate cyme (e.g. D’Arcy and Rakotozafy 1994; Edmonds and Chweya 1997; Edmonds 2012). Both peduncles and pedicels usually have pubescence that is similar to that of the stems and leaves or somewhat reduced distally.

### Pedicels

Pedicels in flower are usually deflexed or spreading, but this can be very difficult to see in herbarium specimens. In fruit, pedicels are usually somewhat pendent from the weight of the berry, but are strongly (e.g. *S. chenopodioides*, *S. tarderemotum*) or weakly (e.g. *S. nigrum*) deflexed in some species. *Solanum tarderemotum* has the pedicels in fruit strongly bent at the base in an acute angle relative to the rhachis; this is a useful character for identification of herbarium specimens. Other species have markedly spreading pedicels in fruit (e.g. *S. memphiticum*). The abscission zone at the pedicel base in members of the Morelloid clade is at the very base and, if and when pedicels fall, the scars are generally flush with the rhachis. Pedicel persistence with fruit ripening is an important species character in this group. Ripe berries either fall or are taken from the plant with the pedicel still attached (e.g. *S. opacum*, *S. tarderemotum*) or the berry falls and the pedicel is left behind (e.g. *S. americanum*, *S. nigrum*, *S. villosum*). The presence of old pedicels can be useful for identification of non-flowering herbarium specimens.

### Calyces

The calyx in all members of the Morelloid clade is 5-merous and synsepalous. The calyx tube is generally conical or occasionally somewhat elongate (e.g. *S. memphiticum*) and the lobes are extremely variable in size and shape from minute and deltate (e.g. *S. umalilaense*) to long-triangular (e.g. *S. pseudospinosum*). The position of the calyx lobes in fruit is an important identification character; they can be strongly reflexed (e.g. *S. americanum*, *S. villosum*), spreading (e.g. *S. nigrum*, *S. tarderemotum*) or appressed to the berry (e.g. *S. opacum*). The calyces of the weedy introduced species *S. nitidibaccatum* and *S. sarrachoides* are accrescent in fruit with the calyx lobes expanding to envelop almost the entire berry (several other New World members of the group also have accrescent calyces, see Särkinen and Knapp 2016).

### Corollas

In common with most other species of *Solanum*, members of the Morelloid clade have 5-merous sympetalous corollas that are variously stellate. Floral mutants are often observed, where 4-6-merous corollas can occur on individual plants that are otherwise 5-merous (e.g. *S. scabrum*). Colour is generally white or pale violet-tinged, but anthocyanin pigmentation can vary depending on environmental growth conditions. In most species at least, some individuals (collections) with purple or violet flowers have been recorded. *Solanum villosum* often has distinctive dark purple coloration on the abaxial midvein of each corolla lobe. At the base of the corolla tube, there is a ring or irregular area of differently coloured tissue usually referred to as the “eye”. In the species of the Morelloid clade, this is usually yellow or greenish-yellow and, in some species, the eye has darker brown or blackish-purple margins (e.g. *S. nitidibaccatum*). The colours of the eye usually disappear in herbarium specimens and are rarely noted on labels. This eye is usually similar in texture to the rest of the corolla and not shiny as occurs in the Dulcamaroid clade (see Knapp 2013)

Corollas in the Morelloid clade are stellate to deeply stellate and corolla lobes are deltate to long-triangular. *Solanum tarderemotum* has deeply stellate corollas, with reflexed corolla lobes, while *S. memphiticum* has corollas with the lobes approximately the same length as the tubular portion and the lobes are not strongly reflexed at anthesis. These characters, particularly those of the degree to which corolla lobes are reflexed, can be very difficult to see in herbarium specimens. As is seen in many other groups of solanums (e.g. Dulcamaroid clade, ANS clade, see Knapp 2013; Knapp and Vorontsova 2016) where flowers last more than one day, the corolla lobes vary in the degree to which they are reflexed through the life of the flower. Lobes often are spreading on day one, become reflexed to strongly reflexed on subsequent days and, as the flower ages, become spreading again.

Corollas of members of the Morelloid clade are usually very small, in fact representing the tiniest flowers of any *Solanum*. Corolla diameter varies from 4–20 mm; *S. nitidibaccatum* has the smallest corollas and *S. hirtulum* and *S. furcatum* the largest in the group of species occurring in the Old World. Adaxial lobe surfaces are usually glabrous, while abaxial corolla lobe surfaces are variously papillate, with longer simple uniseriate trichomes on the margins and tips.

### Androecium

The stamens of members of the Morelloid clade are ellipsoid and equal to very slightly unequal in size and length. The filament tube and filaments are variously pubescent adaxially. Most populations of *S. memphiticum* have completely glabrous filaments, but this is not completely consistent within the species; this was used as a species-specific character in Edmonds (2012). The trichomes on filaments are simple and uniseriate and usually weak-walled and tangled. The filament tube is generally very short to almost absent and free portion of the filaments distinct. Filament length in comparison to anther length is a useful character for distinguishing species. In most of the Old World species of Morelloids, the free portion of the filament is more or less equal to the anther length, but in some species pairs with otherwise similar anther length (e.g. *S. americanum*, *S. opacum*) differences in free filament length can be diagnostic (*S. opacum* has much longer filaments than *S. americanum*). The length of filaments can affect the biophysical properties of anther vibration and thus vibratile pollination (e.g. Timerman et al. 2014; Switzer and Combes 2017) and thus may be an important characteristic involved in speciation in this group.

Anthers of members of the Morelloid clade conform to the poricidal morphology of all other species of *Solanum* (see Knapp 2001). In common with other “non-spiny” solanums, the anther is ellipsoid and the terminal pore usually “unzips” during anthesis to become an elongate slit. The anthers are loosely connivent and not connected by either “glue” (as in *S. dulcamara*, see Glover et al. 2004) or elongate papillae (as in the tomatoes, see Peralta et al. 2008). Anther size is an important identification feature in the Morelloid clade and varies from less than 1 mm (*S. americanum*, *S. opacum*) to ca. 4 mm long (*S. alpinum*); in such small flowers, small differences can be very important.

### Gynoecium

The gynoecium in members of the Morelloid clade is bicarpellate; the carpels are fused in a superior ovary with axile placentation. The ovary is glabrous and usually conical to globose. The flowers lack nectaries, as do all species of *Solanum*. The style is straight or slightly curved and usually sparsely to densely pubescent in the lower half to third where it is enclosed in the anther cone. It is usually exserted from the anther cone, but in some species (e.g. most populations of *S. americanum*, *S. scabrum*) only barely exceeds the length of the stamens. This may be related to self-fertilisation and thus self-compatibility, as has been observed in the tomatoes (Rick et al. 1977, 1978, 1979; Rick and Tanksley 1981; Peralta et al. 2008), but all species of the Morelloid clade tested have been self-compatible (Edmonds 1979a; Schilling and Heiser 1979; Eijlander and Stiekema 1994; Olet 2004). None of the species of the Morelloid clade has heterostylous flowers, although the style length varies along the rhachis in some individuals of *S. tarderemotum* (see species description). The stigma is capitate and occasionally somewhat bilobed (e.g. *S. alpinum*, *S. pygmaeum*). The ovules are anatropous and non-arillate.

### Fruits

As with all species of *Solanum*, the fruit is a bicarpellate berry. Fruits of members of the Morelloid clade are usually brightly coloured and juicy. Most species have globose berries, but those of *S. villosum* are usually somewhat longer than wide. Berry colour can be green (*S. nitidibaccatum*, *S. opacum*, some populations of *S. tarderemotum*), greenish-yellow or yellow (some populations of *S. nigrum* and *S. villosum*, *S. palitans*), bright orange (*S. villosum*) or varying shades of purple (many species); immature berries are usually described as green on herbarium labels. Colour polymorphisms are common in species of this group; both *S. nigrum* and *S. tarderemotum*, for example, have individuals and populations with green or purple berries and *S. villosum* has either yellow or orange berries. Schilling and Andersen (1990) suggested there may be an environmental effect on berry colour in *S. villosum*; accessions labelled as having yellow or orange berries in the field in India all had orange berries in cultivation. Manoko (2007) showed that mature berry colour (green or black) did not differentiate groups within *S. nigrum*. Despite this variation, berry colour is an important identification aid in this group, but is often not recorded on herbarium labels, especially of older specimens.

The pericarp (epicarp) of the berries is thin and either matte (e.g. *S. chenopodioides*, *S. tarderemotum*) or shiny (e.g. *S. americanum*, *S. villosum*). Surface characteristics are useful for species identification, especially when combined with other characters (see discussion of *S. americanum*). The mesocarp is always juicy and very liquid; these fruits are eaten by both birds and mammals (including people). In general, the mesocarp of fresh fruits is green or greenish-yellow, but in species with purple berries, it is sometimes purplish. In the cultivated *S. scabrum*, this may be related to human-mediated selection in Africa and, in China, where on Hainan Island people are beginning to cultivate *S. americanum* (S. Knapp, pers. obs.), coloured fruit pulp is selected for by local people. This character is rarely mentioned on herbarium labels.

Like some other groups of non-spiny solanum such as the Pachyphylla clade (Bohs 1998) and the Archaesolanum clade (Symon 1994), berries of members contain small, hard inclusions commonly referred to as stone cells, sclerotic granules or brachysclereids (Bitter 1911, 1914). These concretions are composed of modified sclerenchyma cells with massively enlarged cell walls; the stone cells of pears and quinces (Rosaceae) are classic examples of this cell type. Neither their function nor their origin in Solanaceae is known. It has been suggested that stone cells may aid bird dispersal by protecting the seeds in the gizzard or by helping seeds to adhere to birds’ legs or plumage (Poczai et al. 2011). Bitter (1914) suggested that they existed in an evolutionary series in the family, with more “advanced” taxa lacking them altogether (e.g. the spiny solanums). Some members of the Archaesolanum clade (e.g. *S. aviculare* G.Forst. with an average of 12–55 seeds and 491–607 stone cells; Symon 1994) have more stone cells in each berry than seeds. In the Morelloid clade, these stone cells are usually quite small and are always round in shape, ca. 0.5 mm in diameter and brown to white in colour (Figure 3). Stone cells can usually be easily seen in dried specimens without dissecting the berry (see Figure 1 in Bitter 1914); they appear globose and are often distinctly larger or smaller than the seeds. Sometimes stone cells of different sizes are found in the same berry, but this character is not consistent within species. The number of these is usually relatively consistent within a species and varies from absent (e.g. *S. scabrum*, *S. villosum*) to 1–4 (e.g. *S. nitidibaccatum*, *S. memphiticum*) to >10 (e.g. *S. triflorum*). Bitter (1914) reported that, in crosses involving Morelloid species with and without stone cells, hybrids had stone cells present in the fruit, indicating to him that this was an inherited character. Cultivated species (e.g. *S. americanum*, *S. retroflexum*, *S. scabrum*, *S. villosum*) tend to lack stone cells; this may be related to human-mediated selection.

### Seeds

Members of the Morelloid clade have flattened seeds, like other solanums. Unlike other groups, however, they are usually tear-drop rather than kidney shaped, with the hilum and micropyle at one of the short ends of the seed. Seed size varies from 1–3 mm long, polyploid species usually have larger seeds than diploids (e.g. *S. americanum* seed size is 1–1.5 mm, while that of *S. scabrum* is 2–2.8 mm) and hence seed size is a good feature for distinguishing *S. nigrum* (hexaploid) from *S. americanum* (diploid). Seed number per berry in the Morelloid clade is generally quite high (Särkinen et al. 2015a), with usually 30–50 seeds in each berry. Some species, however, consistently have fewer seeds (e.g. *S. pseudospinosum*, *S. retroflexum*).

Seed coat morphology has been suggested as a useful character for species-level taxonomy in *Solanum* (Souèges 1907; Lester and Durrands 1984) and has been useful in delimiting groups in some clades (e.g. Geminata clade, Knapp 2002a). All of the Old World morelloid species have sinuate-walled (digitate) testal cells. The lateral walls of these cells of the outer epidermal layer develop lignified radial thickenings that form as hair-like structures (Souèges 1907; Lester and Durrands 1984; Axelius 1992; Peralta et al. 2008). When the outer wall of the epidermis is removed, either naturally (e.g. by passage through frugivore guts, see Barnea et al. 1990) or by enzymatic digestion (Lester and Durrands 1984; Knapp 2002a), seeds appear pubescent; seed measurements here include these projections. Edmonds (1983) examined seed coat patterns in some members of the Morelloid clade (species previously included in Solanum section Solanum) and found no useful variation for delimiting either species or species groups.

### Chromosomes

Chromosome numbers in the Morelloid clade are variations on the base number of 12 (Table 1). The chromosomes are very small with an average size of 1.57 – 1.81 µm (Melo et al. 2011); an unvouchered diploid accession grown in India (Bhiravamurty 1975) had median, submedian and subterminal centromeres. Chromosome staining showed that *S. americanum* and *S. nigrum* have a satellite chromosome pair (Rego et al. 2009; Melo et al. 2011). The Morelloid clade, along with the potatoes, is one of the few lineages in *Solanum* where polyploidy is common (see the section on Polyploidy and hybridisation below). DNA amounts in unreplicated gametic nuclei (C-values) vary between 1.03 pg (1,002 Mbp) in *S. americanum* (as *S. nodiflorum*) and 3.10 pg (3,032 Mbp) in *S. nigrum* (Bennett and Leitch 2012).

Many chromosome counts are reported for members of this group, often as unvouchered counts of “Solanum nigrum”. In the species treatments, we only record counts that are based on identifiable material or those that are vouchered and for which we have verified the specimen in question. Chromosome counts recorded in floras (e.g. Hawkes and Edmonds 1972) without vouchers are not listed.

## Biology and natural history

### Habitats and distribution

Members of the Morelloid clade are plants of disturbed habitats and occur in landslides, along roads and stream, and at the edges of cultivated fields. Many of the species have very broad elevational ranges (e.g. *S. americanum*) and extremely broad distributions, but a few of the Old World species are localised endemics (e.g. *S. alpinum*, *S. hirtulum*, *S. pseudospinosum*) primarily of montane areas.

These localised endemics are all from recent (ca. Miocene age), volcanic mountain ranges and are all probably of recent origin. *Solanum pseudospinosum* is a polyploid, while the ploidy levels of *S. alpinum* and *S. hirtulum* are not known. Molecular data suggests *S. hirtulum* to be a polyploid based on presence of multiple peaks in low copy nuclear gene sequences, similar to known polyploid species such as *S. nigrum* (Särkinen et al. 2015a). Geology and origins of the floras of these mountain ranges are discussed in the individual species treatments.

Several members of the group (e.g. *S. nigrum*, *S. nitidibaccatum*) are registered as noxious weeds of agriculture (see below) in both Europe and North America (Ogg et al. 1981; Defelice 2003). *Solanum triflorum* is listed as a declared weed in Tasmania (Weed Management Act 1999 2000). Many herbarium collections from Africa record morelloid species as “weeds on edge of agriculture”, but it is often unclear whether these were really weedy or were being cultivated using local traditional methods (see Uses below). Confusion over the identification of individual species (Ogg et al. 1981) and the common use of “S. nigrum agg.” in describing these species makes assessment of their status very difficult in the absence of vouchers.

We list the status and general distribution of the species in the group in Table 2 and, in Tables 3–6, document country distribution for Africa (Table 3), Asia (Table 4), Europe (Table 5) and the Pacific (Table 6) from herbarium specimens (see Materials and Methods).

**Table 3.** Country distribution of members of the Morelloid clade in Africa. Introduced species are shown in parentheses. Countries where these species are expected to occur, but from which we have seen no specimens are included, but no species listed.

Country	Species
Algeria	*S. nigrum*, *S. villosum*
Angola	*S. americanum*, *S. scabrum*, *S. tarderemotum*, *S. villosum*
Benin	*S. scabrum*
Botswana	*S. retroflexum*, *S. scabrum*, *S. tarderemotum*, *S. villosum*
Burkina Faso	*S. scabrum*
Burundi	*S. memphiticum*, *S. tarderemotum*, *S. villosum*
Cape Verde	*S. americanum*, (*S. nigrum*), *S. tarderemotum*
Cameroon	*S. americanum*, *S. pseudospinosum*, *S. scabrum*, *S. tarderemotum*
Central African Republic (CAR)	*S. scabrum*
Chad	*S. tarderemotum*, *S. villosum*
Comoros	*S. americanum*, *S. scabrum*, *S. tarderemotum*
Democratic Republic of the Congo	*S. americanum*, *S. scabrum*, *S. tarderemotum*
Republic of the Congo	*S. americanum*, *S. scabrum*
Cote d’Ivoire	*S. americanum*, *S. scabrum*
Djibouti	–
Egypt (incl. Hala’ib triangle)	*S. memphiticum*, *S. nigrum*, *S. scabrum*, *S. tarderemotum*, *S. villosum*
Equatorial Guinea	*S. americanum*, *S. pseudospinosum*, *S. scabrum*
Eritrea	*S. americanum*, *S. memphiticum*, *S. scabrum*, *S. tarderemotum*, *S. villosum*
Ethiopia	*S. americanum*, *S. hirtulum*, *S. memphiticum*, *S. scabrum*, *S. tarderemotum*
Gabon	*S. americanum*, *S. scabrum*
Gambia	*S. americanum*
Ghana	*S. americanum*, *S. scabrum*, *S. tarderemotum*
Guinea	*S. scabrum*, *S. tarderemotum*
Guinea-Bissau	*S. americanum*
Kenya	*S. americanum*, *S. memphiticum*, *S. scabrum*, *S. tarderemotum*, *S. villosum*
Lesotho	(*S. chenopodioides*), *S. retroflexum*, *S. scabrum*, *S. tarderemotum*
Liberia	*S. americanum*, *S. scabrum*, *S. villosum*
Libya	*S. nigrum*, *S. villosum*
Madagascar	*S. americanum*, *S. scabrum*, *S. tarderemotum*
Malawi	*S. americanum*, *S. retroflexum*, *S. scabrum*, *S. tarderemotum*, *S. villosum*
Mali	*S. tarderemotum*
Mauritania	(*S. scabrum*), *S. villosum*
Mauritius	*S. americanum*, (*S. chenopodioides*), (*S. tarderemotum*)
Morocco	*S. nigrum*, (*S. triflorum*), *S. villosum*
Mozambique	*S. americanum*, *S. scabrum*, *S. tarderemotum*, *S. villosum*
Namibia	*S. retroflexum*, *S. scabrum*, *S. tarderemotum*
Niger	*S. villosum*
Nigeria	*S. americanum*, *S. scabrum*, *S. tarderemotum*, *S. villosum*
Rwanda	*S. tarderemotum*
São Tome e Principe	*S. americanum*, *S. scabrum*
Senegal	*S. scabrum*, *S. tarderemotum*
Seychelles	*S. americanum*, *S. scabrum*
Sierra Leone	*S. americanum*, *S. scabrum*, *S. tarderemotum*
Somalia	*S. americanum*, *S. memphiticum*, *S. tarderemotum*, *S. villosum*

**Table 4.** Country distribution of members of the Morelloid clade in Asia. Introduced species are shown in parentheses. Countries where these species are expected to occur, but from which we have seen no specimens are included, but no species listed.

**Country**	**Species**
Afghanistan	*S. nigrum*, *S. villosum*
Armenia	*S. nigrum*, *S. villosum*
Azerbaijan	*S. nigrum*, *S. villosum*
Bahrain	*S. nigrum*, *S. villosum*
Bangladesh	*S. americanum*, *S. villosum*
Bhutan	*S. americanum*, *S. nigrum*, *S. villosum*
Brunei	–
Cambodia	*S. americanum*
China	*S. americanum*, *S. nigrum*, (*S. scabrum*), *S. villosum*
Cyprus	*S. nigrum*, *S. villosum*
Georgia	*S. nigrum*, *S. villosum*
India	*S. americanum*, *S. nigrum*, *S. villosum*
Indonesia	*S. alpinum*, *S. americanum*, *S. nigrum*, *S. opacum*, (*S. scabrum*)
Iran	*S. nigrum*, *S. villosum*
Iraq	*S. nigrum*, *S. villosum*
Israel	*S. nigrum*, *S. villosum*
Japan	*S. americanum*, (*S. chenopodioides*), *S. nigrum*, *S. opacum*
Jordan	*S. memphiticum*, *S. nigrum*, *S. villosum*
Kazakhstan	*S. nigrum*, *S. villosum*
Kuwait	*S. nigrum*, *S. villosum*
Kyrgyzstan	*S. nigrum*, *S. villosum*
Laos	*S. americanum*, *S. nigrum*
Lebanon	*S. nigrum*, *S. villosum*
Malaysia	*S. americanum*, *S. nigrum*, *S. opacum*
Maldives	–
Mongolia	*S. nigrum*
Myanmar (Burma)	*S. americanum*, *S. nigrum*
Nepal	*S. americanum*, *S. nigrum*, *S. villosum*
North Korea	*S. nigrum*
Oman	*S. nigrum*, *S. villosum*
Pakistan	*S. americanum*, *S. nigrum*, *S. villosum*
Palestine	*S. nigrum*, *S. villosum*
Philippines	*S. americanum*, *S. nigrum*, *S. opacum*
Qatar	*S. villosum*
Russian Federation	*S. nigrum*, *S. villosum*
Saudi Arabia	*S. memphiticum*, *S. nigrum*, *S. villosum*
Singapore	(*S. scabrum*)
South Korea	*S. nigrum*
Sri Lanka	*S. americanum*, *S. nigrum*
Syria	*S. nigrum*, *S. villosum*
Taiwan	*S. americanum*, *S. nigrum*, *S. opacum*
Tajikistan	–
Thailand	*S. americanum*, *S. nigrum*
Timor-Leste	–
Turkey	*S. nigrum*, *S. villosum*
Turkmenistan	*S. nigrum*, *S. villosum*
United Arab Emirates (UAE)	*S. nigrum*, *S. villosum*
Uzbekistan	*S. nigrum*
Vietnam	*S. americanum*, *S. nigrum*
Yemen	*S. memphiticum*, *S. nigrum*, *S. villosum*

**Table 5.** Country distribution of members of the Morelloid clade in Europe. Introduced species are shown in parentheses and species added from literature sources in boldface type (Pawłowskiego 1963; Natkevičaitė-Ivanauskienė 1976; Bertová and Goliašová 1993; Hämet-Ahti et al. 1998; Fischer et al. 2005; Jauzein and Nawrot 2011; Hartvig 2015; Rottensteiner 2015). Countries where these species are expected to occur, but from which we have seen no specimens are included, but no species listed.

Country	Species
Albania	*S. nigrum*, (***S. nitidibaccatum***), (***S. triflorum***), *S. villosum*
Andorra	–
Armenia	*S. nigrum*, *S. villosum*
Austria	*S. nigrum*, (***S. nitidibaccatum***), *S. villosum*
Azerbaijan	*S. villosum*
Belarus	*S. nigrum*
Belgium	*S. nigrum*, (*S. nitidibaccatum*), (*S. triflorum*), (*S. villosum*)
Bosnia and Herzegovina	*S. nigrum*, *S. villosum*
Bulgaria	*S. nigrum*, *S. villosum*
Croatia	(***S. chenopodioides***), *S. nigrum*, (***S. nitidibaccatum***), *S. villosum*
Cyprus	*S. nigrum*, *S. villosum*
Czech Republic	*S. nigrum*, *S. villosum*
Denmark	*S. nigrum*, (***S. nitidibaccatum***), *S. villosum*
Estonia	*S. nigrum*
Finland	(***S. americanum***), *S. nigrum*, (***S. nitidibaccatum***)
France	(*S. americanum*), (*S. chenopodioides*), *S. nigrum*, (***S. nitidibaccatum***), *S. villosum*
Georgia	*S. nigrum*, *S. villosum*
Germany	(*S. chenopodioides*), *S. nigrum*, (*S. sarrachoides*), (*S. triflorum*), *S. villosum*
Greece	(*S. chenopodioides*), *S. nigrum*, *S. villosum*
Hungary	(*S. americanum*), (*S. chenopodioides*), *S. nigrum*, (*S. scabrum*), *S. villosum*
Iceland	–
Ireland	*S. nigrum*, (*S. nitidibaccatum*)
Italy	(*S. chenopodioides*), *S. nigrum*, *S. villosum*
Kazakhstan	*S. nigrum*, *S. villosum*
Kosovo	–
Latvia	***S. nigrum***
Liechtenstein	–
Lithuania	***S. nigrum***, (***S. villosum***)
Luxembourg	–
Macedonia (FYROM)	*S. villosum*
Malta	*S. nigrum*, *S. villosum*
Moldova	–
Monaco	*S. villosum*
Montenegro	–
Netherlands	(*S. chenopodioides*), *S. nigrum*, (*S. nitidibaccatum*), (*S. triflorum*), (*S. villosum*)
Norway	*S. nigrum*
Poland	*S. nigrum*, *S. villosum*
Portugal	(*S. chenopodioides*), *S. nigrum*, *S. villosum*
Romania	*S. nigrum*, (*S. triflorum*), *S. villosum*
Russian Federation	*S. nigrum*, *S. villosum*
San Marino	*S. villosum*
Serbia	*S. villosum*
Slovakia	*S. nigrum*, *S. villosum*
Slovenia	*S. nigrum*, *S. villosum*
Spain	(*S. americanum*), (*S. chenopodioides*), *S. nigrum*, (*S. sarrachoides*), *S. villosum*
Sweden	(*S. americanum*), (*S. chenopodioides*), *S. nigrum*, (*S. nitidibaccatum*), (*S. sarrachoides*), (*S. triflorum*), (*S. villosum*)
Switzerland	(*S. chenopodioides*), *S. nigrum*, (*S. pygmaeum*), *S. villosum*
Turkey	*S. nigrum*, *S. villosum*
Ukraine	*S. nigrum*, *S. villosum*
United Kingdom (UK)	(*S. americanum*), (*S. chenopodioides*), *S. nigrum*, (*S. nitidibaccatum*), (*S. pygmaeum*), (*S. sarrachoides*), (*S. triflorum*), (*S. villosum*)

**Table 6.** Country distribution of members of the Morelloid clade in the Pacific, including Australasia. introduced species in shown in parentheses. Countries where these species are expected to occur, but from which we have seen no specimens are included, but no species listed.

**Country/Territory**	**Species**
American Samoa (USA)	*S. americanum*
Australia	*S. americanum*, (*S. chenopodioides*), (*S. furcatum*), *S. nigrum*, (*S. nitidibaccatum*), *S. opacum*, (*S. palitans*), (*S. retroflexum*), (*S. scabrum*), (*S. triflorum*), (*S. villosum*)
Cook Islands (New Zealand)	*S. americanum*, *S. opacum*
Fiji	*S. americanum*, *S. opacum*
French Polynesia (France)	*S. americanum*, *S. opacum*
Guam (USA)	*S. americanum*
Kiribati	–
Marshall Islands	*S. americanum*, *S. opacum*
Micronesia	*S. americanum*
Nauru	–
New Caledonia (France)	*S. americanum*, *S. opacum*
New Zealand	*S. americanum*, (*S. chenopodioides*), (*S. furcatum*), *S. nigrum*, (*S. nitidibaccatum*), *S. opacum*, (*S. villosum*)
Niue (New Zealand)	*S. americanum*
Norfolk Island (Australia)	*S. americanum*
Northern Mariana Islands (USA)	*S. americanum*
Palau	*S. americanum*
Papua New Guinea	*S. americanum*, *S. nigrum*, *S. opacum*
Pitcairn Islands (UK)	*S. opacum*
Rapa Nui (Chile, Easter Island)	*S. americanum*, *S. opacum*
Samoa	*S. americanum*, *S. opacum*
Solomon Islands	*S. opacum*
Tokelau (New Zealand)	–
Tonga	*S. americanum*, *S. opacum*
Tuvalu	–
Vanuatu	*S. americanum*, *S. opacum*
Wake Island (USA)	–
Wallis and Futuna (France)	–

### Pollination and dispersal

Like most solanums, flowers of members of the Morelloid clade are buzz-pollinated by bees (Buchmann et al. 1977; De Luca and Vallejo-Marín 2013). Females of solitary bees and bumblebees vibrate the anthers with their indirect flight muscles causing pollen to “squirt” out of the terminal pores; they curl their bodies over the anther cone and rotate around the flower (Buchmann et al. 1977). The pollen is then groomed from the body and packed into the corbiculae, but the area of the venter that contacts the stigma of the next flower cannot be reached. Smaller bees visit and buzz individual anthers (Symon 1979), but do not usually contact the stigma and thus solanums with large flowers are more properly seen as pollen thieves. Some bees also exhibit “milking” behaviour, where insects grasp the lower part of the anthers and try to force pollen out of the apical pores using upwards pressure (Buchmann et al. 1977. “Gleaning” of loose pollen grains is also done by various small bees and flies (Symon 1979; Knapp 1986). Buchmann et al. (1977) studied *S. douglasii* Dunal (a North American member of the Morelloid clade) where flowers were visited and buzzed by a wide range of bees in various families, but no recent pollination studies have been carried out specifically on any of the Old World morelloid species. Müller (1883: 426) cites syrphid flies milking the anthers from top to bottom in *S. nigrum* and quotes Sprengel’s (1793: 129) observations of “bees and humble-bees [*Bombus* spp.]” visiting the flowers.

Members of the Morelloid clade have juicy berries with thin pericarps (skins) that are typical for bird-dispersed fruits (Knapp 2002b). Studies of dispersal of morelloid species have mostly been done on the species occurring in the United States of America (e.g. Tamboia et al. 1996) with native bird and mammal frugivores (quail, American robins and deer mice). Bravo et al. (2014) record great bustards as frugivores and seed dispersers for *S. nigrum* in central Spain and Barnea et al. (1990) record blackbirds and bulbuls as frugivores of both *S. nigrum* and *S. villosum* (as *S. luteum*) in Israel. Green fruits are expected to be more attractive to mammals, but Tamboia et al. (1996) found that both birds and mammals preferred the purple berries of *S. americanum* to the green berries of *S. sarrachoides* (probably = *S. nitidibaccatum*, no vouchers cited). The suite of characters expected to be attractive to mammals such as green colour, odour and abscission shortly after ripening are all found in some of the Old World morelloids such as *S. tarderemotum* and suggests that mammals may be important fruit dispersers for some of these species as well. Only a few ecological studies have been done on the Old World species of morelloids in their native habitats (but see Barnea et al. 1990; Bravo et al. 2014), although there are many anecdotal references to their dispersal by birds. The ecological interactions are likely to be similar to those observed in North America.

Glycoalkaloid concentrations are very low in ripe berries of *S. americanum* and other members of the Morelloid clade that have been tested (Cipollini et al. 2002) and levels are similar across the clade. Higher concentrations in unripe fruit (Cipollini et al. 2002) of these species make them unattractive to frugivores (Cipollini and Levey 1997a). This loss of secondary metabolites in ripe berries is common across *Solanum* species with brightly coloured, fleshy fruits (e.g. Bradley et al. 1979) and is most likely related to fruit persistence (Cipollini and Levey 1997b), where risk of fungal infection is balanced by probability of animal ingestion and thus dispersal. Glycoalkaloids are known to have a constipative effect (see above, e.g. Gerard 1597) and to inhibit seed germination after ingestion (Cipollini and Levey 1997b), but Wahaj et al. (1998) found that ripe berries of *S. americanum* had a laxative effect on birds thus speeding seed passage through the gut. They suggested this was due to some other chemical compound (perhaps calystegines (?), see Dräger et al. 1994). *Solanum nigrum* germination was not affected by ingestion by bulbuls and blackbirds (Barnea et al. 1990), but germination was enhanced in *S. villosum* (as *S. luteum*). Barnea et al. (1990) suggest this is due to the more arid habitats in which *S. villosum* grows, where bird defecation around shrubs would enhance survival of seedlings. Bravo et al. (2014) showed that germination rates for *S. nigrum* were lower after ingestion by great bustards, but still concluded that the birds were likely to be efficient seed dispersal agents.

### Conservation status

Most Old World morelloid species are weedy and widely distributed; many are also cultivated (e.g. *S. tarderemotum*, *S. scabrum*, *S. villosum*) and are distributed widely via human migration. Many introductions of species to Europe have resulted from the use of wool shoddy as protection in market gardens (Blom 1935; McClintock 1977) but even casual visitors to far-flung places have been implicated in the introduction of alien species (Chown et al. 2012). It is likely that the early explorations of the southern Hemisphere inadvertently carried seeds of nightshades with them, accounting for the widespread nature of many of these taxa. The genetic structure of populations of extremely widespread species such as *S. americanum* will need to be investigated to determine if a structure exists in the distribution that can be related to natural or human-mediated causes.

The range-restricted endemic species of Old World morelloids suffer from taxonomic recognition issues that could seriously affect their conservation. In recent floras of many parts of the world, all taxa are treated as a single highly variable species (usually *S. nigrum*) and local endemic taxa are overlooked and equated with widespread invasive weeds. This means that these endemic taxa have possibly been placed at risk. Preliminary conservation assessments for the Old World members of the Morelloid clade (including introduced taxa) are presented in Table 7. Conservation status for introduced taxa includes worldwide data, from both native and introduced ranges.

**Table 7.** Preliminary conservation assessments for the Old World members of the Morelloid clade. For details, see Materials and Methods and individual species treatments. Preliminary assessments are based on EOO (Extent of Occurrence) only (see Materials and Methods) and have been calculated for worldwide ranges for each species. The conservation status of species known to be introduced in the Old World has been assessed considering native range only and is discussed under each species treatments.

**Species**	**Preliminary conservation assessment (IUCN 2016)**	**EOO (km^2^) [worldwide range**]
***Solanum alpinum***	VU (Vulnerable)	18,806
*Solanum americanum*.	LC (Least Concern)	51,956,485
*Solanum chenopodioides*	LC (Least Concern)	78,693,743
*Solanum furcatum*	LC (Least Concern)	105,324,815
***Solanum hirtulum***	NT (Near-Threatened)	26,035
*Solanum memphiticum*	LC (Least Concern)	4,489,278
*Solanum nigrum*	LC (Least Concern)	202,319,396
*Solanum nitidibaccatum*	LC (Least Concern)	83,736,975
*Solanum opacum*	LC (Least Concern)	78,180,634
*Solanum palitans*	LC(Least Concern)	24,975,848
***Solanum pseudospinosum***	EN (Endangered)	4,009
*Solanum pygmaeum*	LC (Least Concern)	16,699,600
*Solanum retroflexum*	LC (Least Concern)	19,067,920
*Solanum sarrachoides*	LC (Least Concern)	114,305,743
*Solanum scabrum*	LC (Least Concern)	49,116,048
*Solanum tarderemotum*	LC (Least Concern)	22,131,524
*Solanum triflorum*	LC(Least Concern)	91,711,479
***Solanum umalilaense***	EN [DD] (Endangered or Data Deficient)	2,559
*Solanum villosum*	LC (Least Concern)	364,611,726

## Polyploidy and hybridisation

The Morelloid clade is one of only a few cases of extensive ploidy level variation in *Solanum*. Potatoes and the Archaesolanum clade are the only other clades of *Solanum* in which ploidy level variation is extensive (Poczai et al. 2011; Spooner et al. 2014); due to the economic importance of the species in the Potato clade as contributors of genes for cultivated potato improvement, they have been much more extensively investigated than the morelloids (see Spooner et al. 2014). Potato species are also diploid, tetraploid or hexaploid, but the cultivated potato itself (*S. tuberosum* L.) comprises both diploid and tetraploid populations (see Spooner et al. 2007); this variation of ploidy level occurs in some New World morelloids, but we have not observed it in the species treated here. The morelloids have been the focus of only a few previous studies (e.g. Stebbins and Paddock 1949; Edmonds 1977, 1979a) and, of the ca. 75 species in the clade, only 44 species have verified and vouchered chromosome counts; the counts show that 30 species are diploid (*2n*=2x=24), nine are tetraploid (*2n*=4x=48) and five hexaploid (*2n*=6x=72). Octoploid individuals have only been recorded three times, all of these in South American plants and are thought to be the result of spontaneous chromosome doubling of tetraploid species (Heiser 1963; Edmonds 1977) and three species (*S. interandinum* Bitter, *S. macrotonum* Bitter, *S. pygmaeum*) appear to have multiple ploidy levels. Eight of the Old World native morelloids are polyploid (six tetraploids and two hexaploids) and sequence data indicate that *S. hirtulum* is also a polyploid based on the presence of multiple double peaks in low copy nuclear sequence reads similar to known polyploid species (e.g. *S. nigrum*), but this needs to be confirmed with a vouchered chromosome count (see Table 1; Särkinen et al. 2015a). The ploidy level of *S. alpinum* remains unknown. Natural hybridisation between sympatric species (Ganapathi and Rao 1985, 1986b) has been suggested as the reason for the large number of polyploids in the Morelloid clade, especially in the Old World, but evidence for this hypothesis is weak at best.

It is thought that most of the polyploids in the Morelloid clade are of alloploid, rather than autoploid origin, because bivalents show regular pairing at meiosis (Edmonds 1977, 1979a; Ganapathi and Rao 1987a; Ojiewo et al. 2007). The putative parental origin of these species has been investigated using traditional crossability and hybridisation techniques (Edmonds 1977; Ganapathi and Rao 1985, 1986b, 1986c, 1986d, 1987a, 1987b) and, more recently, using molecular markers (Manoko 2007; Poczai and Hyvönen 2011). Chromosome painting or GISH (genomic in situ hybridisation) techniques have not been used in determining progenitor genomes in the morelloids as they have with great success in *Nicotiana* (e.g. Chase et al. 2003; Clarkson et al. 2005) and in the potatoes (e.g. Pendinen et al. 2012).

Edmonds (1977) undertook an extensive crossing programme involving morelloid species from both the New and Old Worlds. She found that hybridisation was relatively common within ploidy level, while successful hybridisations between ploidy levels were rare. Successful crosses between tetraploids and hexaploids were postulated to have resulted due to possession of a common parental genome. She suggested that *S. sarrachoides* (as *S. sarachoides*) was a potential diploid parent of *S. nigrum*, because almost half of the crosses involving it and *S. nigrum* were successful; she also stated that *S. americanum* was the “undisputed [diploid] progenitor” of *S. nigrum*, probably based on the work of Nakamura (1935, 1937). Regular bivalent pairing in hybrids derived from *S. nigrum* × *S. scabrum* crosses indicate homology of parental genomes of the two hexaploids (Ganapathi and Rao 1987a). Evidence from other crossing studies suggests that the tetraploid *S. villosum*, *S. retroflexum* or one of their close relatives, has also been involved in the parentage of *S. nigrum* (Ganapathi and Rao 1986b, 1986c) and that *S. villosum* is one of the parents of *S. scabrum* (Ganapathi and Rao 1985, 1987a, 1987b). As only a limited set of the diploid and tetraploid species have been used in these crossing studies, results have to be interpreted with care in that the species tested, or any of its close relatives, could have been involved in the parentage. Detailed molecular phylogenetic studies could help to reveal closely related species to the ones used in these crossing studies to further understand potential parents that could then be tested.

Manoko (2007) used AFLP (Amplified Fragment Length Polymorphism) molecular markers to examine genetic variation within and between African diploids and polyploids. As AFLP analyses are based on fragment sizes, results from these analyses only allow us to understand genetic variation but do not directly link to phylogenetic relationships. The AFLP study found that tetraploid accessions split into two distinct groups; a set of tetraploids (group A; *S. retroflexum*, *S. tarderemotum* (as *S. florulentum*), *S. umalilaense* and *S. villosum*) clustered with hexaploid taxa (*S. nigrum*, *S. scabrum*) and another set (group B; *S. memphiticum*, *S. tarderemotum* and “*S. patens*”) clustered with diploid species used in the analysis. It was suggested that, because the tetraploids split into two clear groups in a Neighbour Joining (NJ) analysis, they were unlikely to share diploid progenitors (Manoko 2007), but as highlighted above, AFLP studies allow the inspection of genetic variation but not phylogenetic relationships. Poczai and Hyvönen (2011) used ISSR (Inter Simple Sequence Repeat) and SCoT (Start Codon Targeted) markers in a network analysis to investigate the origin of *S. nigrum*. They used a limited number of species that had previously been suggested as having common origins; *S. americanum*, *S. chenopodioides*, *S. nigrum*, *S. opacum*, *S. nitidibaccatum* (as *S. physalifolium* Rusby) *S. retroflexum*, *S. scabrum* and *S. villosum*. Their results supported the close relationship between *S. villosum* and the two hexaploids (*S. nigrum* and *S. scabrum*) and they suggested that these species share a diploid progenitor. They also showed that *S. villosum* is most likely an autotetraploid derived from *S. americanum*, confirming an earlier chromosome banding study (Sultana and Alam 2007) and hypothesised that the progenitors of *S. nigrum* are *S. villosum* and *S. americanum* and that the species evolved via amphiploidy of sterile triploid progeny (Poczai and Hyvönen 2011). *Solanum opacum* and *S. retroflexum* were associated with *S. chenopodioides* and *S. nitidibaccatum* (as *S. physalifolium*) and not with *S. americanum*, contrary to suggestions by Edmonds 1977, who thought *S. americanum* was involved in the origins of *S. retroflexum* based on crossability, suggesting their diploid progenitors are not shared with the other polyploids in the analysis. Results of previous work attempting to establish genome origins for morelloid polyploids is summarised in Table 8.

**Table 8.** Summary of putative parental origins of the polyploid species in the Morelloid clade (see text for references).

**Tetraploids 2n = 4× = 48**	**Type**	**Putative parental species**
*S. pseudospinosum*	Unknown	Unknown
*S. retroflexum*	autopolyploid	*S. chenopodioides* or *S. nitidibaccatum*
*S. umalilaense*	Unknown	Unknown
*S. memphiticum*	Unknown	Unknown
*S. tarderemotum*	Unknown	Unknown
*S. villosum*	autopolyploid	*S. americanum*
**Hexaploids 2n = 6× = 72**		
*S. furcatum*	Unknown	Unknown
*S. opacum*	autoallopolyploid	*S. retroflexum* × *S. chenopodioides*
*S. scabrum*	autoallopolyploid	*S. americanum* × *S. villosum*
*S. nigrum*	autoallopolyploid	*S. americanum* × *S. villosum*

The species of black nightshades are predominantly self-compatible (Edmonds 1979a; Schilling and Heiser 1979; Olet 2004). Interspecific crosses can be produced between most species within ploidy levels, although fertility is not always high and some breeding barriers exist (Edmonds 1977, 1979a; Olet 2004). Putative hybridisation in the wild has been reported between co-occurring diploid species in North America and Australia (Stebbins and Paddock 1949; D’Arcy 1974b; Henderson 1974). Schilling and Heiser (1979), however, suggested that the ability to cross was not a useful taxonomic character in the morelloids. Natural hybrids have also been reported between diploids and polyploids and between polyploids themselves (Henderson 1974; Leslie 1978; Venkateswarlu and Rao 1972), although Edmonds (1977) showed that within ploidy level crossability success was much higher than that between ploidy levels. Hybridisation, followed by backcrossing to parental species and/or polyploidisation, has been thought to explain some of the complex morphological variation found within the group (Stebbins and Paddock 1949; Edmonds 1979a). This assumption of widespread hybridisation being responsible for taxonomic complexity was also the case for the potatoes, but detailed studies with modern methods have shown that much of the variation is not due to hybridisation but instead to extensive within-population variability (Spooner et al. 2014). Schilling and Heiser (1979) pointed out that the hybrids created in the greenhouse were unlikely to occur in nature and expressed views that the use of the Biological Species Concept (BSC; e.g. Mayr 1982) in this group where most species are interfertile at some level is not useful (see Knapp 2008 for a discussion of the BSC and plant species recognition).

## Chemistry

There is considerable confusion regarding the toxicity of species of black nightshades (see Schilling et al. 1992; Olet et al. 2005). Many manuals or floras list black nightshade berries as toxic (e.g. Kingsbury 1964; Edmonds 2005). The European members of the group are often confused with the deadly nightshade *Atropa bella-donna* L., whose berries are similarly black and shiny, but contain highly toxic tropane alkaloids such as atropine and scopolamine (Parr et al. 1990; Ashtiana and Sefidkonb 2011). No species of *Solanum* has been shown to contain tropane alkaloids such as atropine or scopolamine (Tétényi 1987; Pigatto et al. 2015), but members of *Solanum*, including the black nightshades, do contain the nortropane alkaloids known as calystegines (Dräger et al. 1994; Pigatto et al. 2015). In Solanaceae, these compounds are synthesised along the same pathway as the tropanes, but are not as toxic when tested on laboratory rats (Stegelmeier et al. 2008); the finding that they occur in many commonly eaten foods suggests their activity differs from that of the tropanes such as atropine, scopolamine and nicotine (Asano et al. 1997; Welch et al. 2012).

Most species of *Solanum* contain a wide range of steroidal alkaloids (also known as glycoalkaloids; Bradley et al. 1979) that, while potentially toxic in very large quantities, are not dangerous to humans. Chemical surveys have shown that only unripe fruits contain these potentially somewhat toxic glycoalkaloids (Cipollini et al. 2002), while leaves and ripe fruits usually lack these compounds (Carle 1981; Voss et al. 1993). Many of the studies that have studied the chemical composition of morelloid species have mixed samples of immature and mature berries, leading to conclusions that berries are poisonous (Eldridge and Hockridge 1983; Mathé et al. 1980; Sharma et al. 1983; Arnold 1985). Such findings are important in agricultural trade where species of morelloids occur as weeds and their immature and mature berries are eaten by livestock and can contaminate harvests of a variety of grain and vegetable crops (Ogg et al. 1981; Rogers and Ogg 1981). Toxicity of unripe berries, however, appears to be reduced if forage is fermented as silage (Vogel and Gutzwiller 1993) and does not apply when ripe berries are harvested for human consumption.

## Uses

Species of black nightshades are used as leafy vegetables or fruits throughout the world, sometimes referred to as ‘supervegetables’ for their high protein and iron content (FAO 1988; Yang and Ojiewo 2013; Cernansky 2015; Ronoh et al. 2017; Fokon and Domugang 1989). Species of the group are wild-harvested or cultivated for their juicy berries in both the Old and the New Worlds, in both the tropics and temperate zones (e.g. Heiser 1969). Their use, however, has been particularly studied in Africa, where they are important components of indigenous cultivation systems (Edmonds and Chweya 1997; Schippers 2000; Keding et al. 2007; Pasquini et al. 2009) and are the focus of plant breeding strategies aimed at improving the nutrition and income generation of women and children in urban and peri-urban areas (Dinssa et al. 2016; Manoko and van der Weerden 2004a, 2004b).

In Africa, species are cultivated either for use as leaf vegetables (e.g. *S. americanum*, *S. memphiticum*, *S. scabrum*, *S. tarderemotum*, *S. umalilaense*, *S. villosum*) or for their fruits (e.g. *S. americanum*, *S. retroflexum*, *S. scabrum*, *S. villosum*). Leaves of African nightshades are high in flavonoids, vitamin C, folates, iron and antioxidants (Akubugwo et al. 2007; Keding et al. 2007; Yang and Ojiewo 2013; Ronoh et al. 2017) and the leaves are often cooked in milk to make them less bitter. Medicinal uses in Africa include antiseptic for eyes and skin, treatment of diarrhoea (Essou and Hermans 2006) and as a general tonic for health (Yang and Ojiewo 2013). Seeds have been found to be rich in lipids and a good source of essential fatty acids used in some areas in Africa (Nzikou et al. 2007).

In Asia, including the Indian subcontinent, *S. nigrum* is also used as a leaf vegetable (Arora 1981; Jain and Borthakur 1986), for its fruits (Abraham 1981; Vartak 1981) and in medicine (Ammaan and Subramanian 2017). Berries of several species are eaten (e.g. *S. americanum*, *S. retroflexum*, *S. scabrum*, *S. villosum*; see also individual species treatments). *Solanum nigrum* is mentioned in several different contexts in the 13^th^ century Chinese treatise *Jinhuang Bencao* which was the first of many Chinese works on uses of plants and introduced many plants into the diets of modern Chinese people (Zhu et al. 2015). In that work, it is mentioned both as a food (leaves and berries) and as a medicine. Today, leaves of morelloid species are commonly eaten, particularly in southern China; one of the authors (S. Knapp, pers. obs.) has also observed cultivation of *S. americanum* for its fruits there.

Disaggregating uses for these species is difficult because many authors treated all morelloids as a single, highly variable taxon; Burkhill (2000) treated all morelloids in western Africa as *S. americanum*, while they were most certainly several species. The cultivated status of many of these plants went unnoticed by many early plant collectors, who often noted them as “weeds of field margins”. Black nightshades are known by a great many indigenous names, especially across Africa and care must be taken with names applied to species in literature, given the complex taxonomy of the group and difficulties in transliteration of indigenous languages by early collectors. In Table 9, we list the more widely encountered common names by region and language and, in each individual species treatment, list those that we have verified for each species from either herbarium specimens or from literature where we are certain of the species identification.

**Table 9.** Widely used common names for black nightshades in the Old World. Where names can be confidently assigned to individual species, these are listed in the species accounts. We only include names here from which the language is available and unambiguous; many common names in the literature are taken from herbarium labels and both the original and transcription are often very dubious. Sources include Degener (1945), Edmonds and Chweya (1997), Schilling et al. (1992), D’Arcy and Rakotozafy (1994), Ghazanfar (1994), Warrier et al. (1996), Burkhill (2000), Hul and Dy Phon (2014), Ignacimuthu et al. (2006), Euro+Med (2006-), Almeida and Almeida (2008), Ved et al. (2016) and BSBI (2017).

Region		Name (language; alphabetically by language)
Africa	North Africa	‘anab el deeb (Arabic); morelle (French); yerba mora (Spanish)
	East Africa	ocogocuga, pot ocuga (Acholi); ociga/osiga (Alur [Jonam]); rinagu, amanagu (Gusii [Kisii]); ndolu (Kamba); encoratin (Karamojong); manago (Kikuyu); urusogo, isogo, ubusogosogo, ibwija, isiogwo (Kinyarwanda); isoyot, ol’momoit (Kipsigi); isogo, urusogo (Kirundi); manavu/mnavu (Kiswahili); kusochet (Kupsabiny); nswega (Kuria); lere (Kutuk na Kakwa); isusu (Loogoli); nsugga, nsugga enzirugavu (Luganda); asuaka (Lugbara); osuga, ocuga, ocokocok (Luo); mnavu tsaka, oromomoi/olomomoi (Maasai); isufa (Masaba); enswiga/enswiiga (Nkore/Kiga); cheporusion, kusuyo (Pökoot); okaka (Rutooro); esiga (Suvu); sochek (Tugen);
	West Africa	fué (Abure); bogolo, fassa kuna (Bambara); efo-odu, sapota (Creole); shien (Dan); feibii (Edo); ebibirba (Fante); djagato-fórô (Fula); gautan kâdii (Hausa); ugunakpe (Igbo); àljáng (Kanuri); bundu kaemba (Kono); wosangu, wosangu njika (Kwpe, Ngomba); usiga (Luba); itofo, itotofu (Lombo); sibito jambo, bassa béné, suludjatô (Mandingo); mulunda, muzirhu (Mashi); kemba/kimba (Mende); wosangu (Mokpwe); rushoo (Nandi); kumbo (Nso); lin-sah, pe-noh (Samba Daka); suga (Swahili); nsusua (Twi); kholekodelen-na (Yalunka); odu/o’du, ogunmo, ogumo (Yoruba)
	South Africa	nastergal, galbessie, nagskande (Afrikaans); umsobo, sheshoabohloko, umsobo-sobo (isiXhosa); umsobo, isihlalakuhe, udoye, umagqa, umgwaba, umsobo-sobo, umqunbabe (isiZulu); lethotho, seshoa-bohloko, momoli (Sotho); (u)msobo (siSwati); muxe, xaxadi (Venda); kophe (Xitsonga)
	Madagascar	anamamy (Malagache); brède noir, brède morelle (French)
Asia	Middle East	‘anab al deeb/’eneb el deeb (Arabic); mejaje, mesaeleha (Arabic); abriki (Arabic); ‘anamnam (Yemeni Arabic)
	Central Asia	taj rizi (Farsi)
	Southeast Asia	peng pâhs khmôch, pum pou nam, to:ngx tè:ng, tumx to:yz (Laotian); ranti (Malay/Javanese); leuntja (Sundanese); toem tok, ya-tomtok (Thai); bóp bép, (cay) tăm bóp, (cay) hôt nut, tom bóp (Vietnamese)
	Indian subcontinent	makŏg, gurkkamai (Hindi); kakarundi (Kannada); kakmachi (Marathi); manattakkâli, karintakâli, karimttakkâli (Malayam); kakamaci, kakamata (Sanskrit); manattakāli, milagutakkāli (Tamil); kâmanci, kacci, kaccipandu, gejjucettu (Telugu); ab makoh, inab-as-salab (Urdu) [also see http://envis.frlht.org/bot_search]
	China and Japan	kong lui (Chinese, Mandarin); baihua cai, jia dengleng cao (Chinese, Cantonese); lung-kwei (Chinese, Taiwan); inuhozuki (Japanese)
Europe		belarri malaka, blets (Basque); pebre d’ase (Catalan); sort natskygge (Danish); zwarte nachtschade (Dutch); black nightshade (English); must maavits (Estonian); mustakoiso (Finnish); morelle noire (French); schwarzer nachtschatten (German); skilosaphilo (Greek); fuath dubh (Gaelic/Irish); erba morella, erba mora, solano nero (Italian); juodoji kiauliauogė (Lithuanian); svartsøtvier (Norwegian); psianka czarna (Polish); erva moira (Portuguese); paslen ĉernyj (Russian); pomocnica (Serbian); lulock čierny (Slovenian); yerba mora, hierba mora (Spanish); nattskatta (Swedish); kopek uuzimii (Turkish); codwarth du (Welsh)
Pacific	Australia	nightshade (English)
	New Guinea	–
	Islands of the Pacific	popolo, huapopolo (Polynesian)
	New Caledonia	morelle

*Solanum scabrum* is by far the most commonly cultivated of the Old World species and is cultivated throughout Africa (Berinyuy et al. 2002; Defelice 2003; Bukenya and Carasco 1995; Olet et al. 2006), especially in western Africa. It is also known from the Caribbean where it was most likely brought by enslaved peoples from western Africa. In the United States, *S. scabrum* was marketed under the name of “Garden Huckleberry” (Soria and Heiser 1959), where it was introduced with great fanfare by the vegetable breeder Luther Burbank (Heiser 1969). Burbank also introduced what he called a new species, the “Sunberry”, later marketed as the “Wonderberry”, which turned out to be the southern African species *S. retroflexum* (Soria and Heiser 1959; Heiser 1969); Burbank claimed he had created it from a cross between *S. scabrum* and what he called *S. villosum* (probably = *S. sarrachoides*). The story of this and the accusations that Burbank was marketing a poisonous plant are well-documented in Heiser (1969).

*Solanum tarderemotum* and *S. villosum* are more commonly cultivated in eastern Africa and many specimen labels note that the fruits of *S. villosum* are particularly prized by children (also see Keding et al. 2007). *Solanum umalilaense* is cultivated locally as a leaf vegetable in the Umalila region of Tanzania (Schippers 2000; Manoko and van der Weerden 2004a, 2004b) and its highly branching morphology is perhaps due to human selection. In Asia, *S. villosum*, *S. americanum* and *S. nigrum* are all used for both their berries and leaves, as well as for medicine (Potawale et al. 2008; Jagatheeswari et al. 2013). Details of cultivars and more specific uses are given in the species treatments.

*Solanum* “*nigrum*” has a long history of use in Ayurvedic medicine in India, but it is clear that the concept medicinally does not distinguish between *S. nigrum* s.s. and *S. villosum* (see Warrier et al. 1996; Jagatheeswari et al. 2013; Ved et al. 2016). The plants are noted for their antiseptic and antidysenteric properties and, in common with other members of the family, are considered to be febrifuges and anti-inflammatories (see Dunal 1813). They have been used in the treatment of many different diseases and complaints in many areas of the Old World (e.g. Dunal 1813; Grieve 1931; Huang 1993; Ghazanfar 1994; Edmonds and Chweya 1997; Burkhill 2000). Early European uses are documented in the herbals (see above) and in the many pharmacopoeia of different countries (summarised in Grieve 1931); Dunal (1813) records *S. nigrum* as being the most used of European species of *Solanum*, and that with the most ancient use (dating back to Hippocrates, Dioscorides and Theophrastus). Common properties appear to be antiseptic, anti-inflammatory, expectorant and diuretic and these plants are often cited for the treatment of eye and stomach complaints (e.g. Ignacimuthu et al. 2006).

In Europe, seed remains of *S. nigrum* have been excavated from the Viking layers at York (Kenward and Hall 1995) and at ten other sites in Denmark, Germany, Ireland, Russia and Sweden (A. Poulsen and A. Kool, pers. comm., manuscript in preparation). *Solanum villosum* has been found in Israel in Acheulian site in Gesher Benot Ya‘aqov, amongst other food plants that were used by early humans (Melamed et al. 2016). The excavated seeds that represent the black nightshade species still in use today demonstrate that their use has been widespread across different cultures and areas dating back to early humans during their migration out of Africa in mid-Pleistocene (Melamed et al. 2016).

In the Pacific, uses for black nightshades (*S. americanum* and *S. opacum*, without distinction) are recorded from Hawaii (Degener 1945). As with other species of the group, both leaves and berries are eaten and used medicinally. Medicinal uses recorded are as an antiseptic, laxative (the juice of the plant) and as a tonic (Degener 1945); bruised leaves are used topically for stomach ache.

Glycoalkaloids from *S. americanum*, *S. nigrum* and *S. opacum* were often used as a steroid base for human contraceptives until the industry moved to production of synthetic compounds in the 1980s (Bradley et al. 1979; Carle 1981). Aqueous extract from *S. villosum* (as *S. alatum*) and *S. nigrum* have been shown to exhibit anti-inflammatory, hepato-protective, anti-cancer effects and potential to control and prevent gastric ulcers (Lin et al. 1995; Heo and Lim 2005). Leaf extracts of several morelloid species have been shown to have a molluscicidal effect against *Bulinus* (Ndamukong et al. 2006) and *Biomphalaria* (El-Sherbini et al. 2009) snails that act as hosts of *Schistosoma* parasites that cause the tropical disease bilharzia (schistosomiasis) and against the parasites themselves (Obare et al. 2016). On the Indian subcontinent (Singh et al. 2001), tests of leaf extracts of *S. nigrum* have shown promise as larvicides against mosquitoes that are important vectors of human disease such as the malaria vector *Anopheles culicifecies* Giles and the filariasis vectors *Culex quinquefasciatus* Say and *Aedes aegypti* (Linnaeus in Hasselquist). Extracts from the berries of *S. villosum* offer promise as biocontrol agents for the mosquito dengue fever vector *A. aegypti* (as *Stegomyia aegypti*) since resistance to commonly used insecticides is on the increase in many parts of the developing world (Chowdhury et al. 2008). The steroid alkaloid solanine from *S. americanum* has been used as an agricultural pesticide (Sumner 2000) and produces a proteinase inhibitor (PI II) that has shown to confer insect resistance in transgenic plants (Xu 2001).

Old World species of the Morelloid clade are also sources of resistance genes used in agriculture. Several species of black nightshades, including *S. nigrum*, *S. americanum* and *S. villosum*, are known to carry resistance genes against late blight (*Phytophthora infestans* (Mont.) de Bary), a major fungal pest in potato, tomato and eggplant (Kamoun et al. 1999; Campos et al. 2002; Flier et al. 2003; Lebecka 2008, 2009; Śliwka et al. 2012; Witek et al. 2016). Although resistance traits have been transferred to potato (*S. tuberosum* L.) in some experiments (Colon et al. 1993; Eijlander and Stiekema 1994; Horsman et al. 1997; Zimnoch-Guzowska et al. 2003), black nightshades have not been widely used in traditional plant breeding, but new cloning techniques coupled with the large number of resistance genes found in *S. americanum* suggest these plants hold promise for biotechnology (Witek et al. 2016). *Solanum nigrum* has also been used as a model system with which to study herbivore defence responses (Schmidt et al. 2004), especially since response chemistry seems to differ significantly from that seen in tomato (Schmidt and Baldwin 2006).

## Species concepts

Our goal for the treatment of the Old World species of the Morelloid clade has been to provide circumscriptions for the members of this morphologically variable group of species, while clearly highlighting areas, taxa and populations where further in-depth research is still needed to confirm current taxon concepts. Delimitation of species here basically follows what is known as the “morphological cluster” species concept (Mallet 1995): i.e. “assemblages of individuals with morphological features in common and separate from other such assemblages by correlated morphological discontinuities in a number of features” (Davis and Heywood 1963). Biological (Mayr 1982), phylogenetic (Cracraft 1989) and the host of other finely defined species concepts (see Mallet 1995) are almost impossible to apply in practice and are therefore of little utility in a practical sense (see Knapp 2008). It is important, however, to clearly state the criteria for the delimitation of species, rather than dogmatically follow particular ideological lines (see Luckow 1995; Davis 1997). Our decisions relied on clear morphological discontinuities to define easily distinguished species. Specific characters used for recognition are detailed with each species description and in the key. Some potential reasons for variability and intergradation are recent divergence, hybridisation and environmental influence on morphology. In this revision, we have tried to emphasise similarities between populations instead of differences, which so often reflect incomplete collecting or local variation. We have not recognised subspecies or varieties, but have described and documented variation where present, rather than formalise such variability with a name which then encumbers the literature. We have been conservative in our approach, recognising as distinct entities those population systems (sets of specimens) that differ in several morphological characteristics. Many of the species in the group (and of Morelloids in general) are extremely widespread and variable; variation exists in certain characters, but the pattern of variation is such that no reliable units can be consistently extracted, nor is geography a completely reliable predictor of character states. Here variability within and between populations seems more important than the variations of the extremes that other taxonomists have recognised as distinct. We describe this variation realising that others may wish to interpret it differently.

Although infraspecific taxa have been recognised by others within the group, we do not recognise any here due to the complex morphological variation observed within each species, where the inspection of a larger number of specimens quickly reveals no apparent natural breaks in variation but rather a mixing between highly morphologically variable populations of widespread species. Results from seed protein studies (Edmonds and Glidewell 1977), as well as from more recent population genetic studies (e.g. Manoko 2007; Manoko et al. 2008) support our morphological observations in finding no obvious patterns supporting infraspecific structure within particular variable species such as *S. americanum*, *S. villosum* and *S. nigrum*.

## Materials and methods

Our taxonomic treatment is based on results from recent molecular systematic studies considering the taxonomy of the section, including focused morphological and AFLP studies of the African species by Jacoby (2003: South Africa), Olet (2004) and Manoko (2007), network analysis of the polyploid species and their putative diploid ancestors by Poczai and Hyvönen (2011) and the molecular phylogenetic study of the entire Morelloid clade by Särkinen et al. (2015a). Descriptions are based on field work and examination of >10,000 herbarium specimens from 146 herbaria (A, AK, ALCB, ANG, ASE, AV, AZU, B, BA, BAH, BH, BHCB, BISH, BM, BP, BR, BRI, BSD, B-W, C, CAL, CAS, CEN, CEPEC, CESJ, CGE, CHR, CICY, COI, COL, CORD, CPUN, CTES, DD, DS, DSM, DUKE, DVPR, E, EA, EAC, ECON, EIU, ESA, F, FCQ, FHO, FT, FUEL, FURB, G, GB, G-BOIS, G-DC, GH, GOET, H, HAMAB, HAO, HAS, HBG, BSHC, HOH, HOXA, HST, HUCS, HUEFS, HUEM, H, IAC, ICN, INB, INPA, IPA, JOI, JPB, K, KUFS, L, LE, LG, LIL, LOJA, LP, LPB, LU, M, MA, MAC, MBM, MBML, MEL, MEXU, MHU, MO, MOL, MPU, MPUC, NHT, NIJ, NSW, NY, OTA, OUPR, OXF, P, PACA, PAL, PBL, PH, QAP, QCNE, S, SCA, SGO, SI, SJRP, SMDB, STU, TAN, TCD, TO, TUB, U, UC, UDBC, UEC, UFP, UFRN, UMO, UPCB, UPS, US, USF, USM, UT, VIES, W, WAG, WIS, WRSL, WU, YA, YU, Z). We have extensively compared introduced species across their entire ranges, not just in the Old World; this in part accounts for the large number of herbaria cited here.

Measurements were made from dried herbarium material supplemented by measurements from living material. Colours of corollas, fruits etc. are described from living material or from herbarium label data. Specimens with latitude and longitude data on the labels were mapped directly. Some species had few or no georeferenced collections; in these cases, we retrospectively georeferenced the collections using available locality data. Maps were constructed with the points in the centres of degree squares in a 1° square grid. Conservation threat status was assessed following the IUCN Red List Categories and Criteria (IUCN 2016) using the GIS-based method of Moat (2007) as implemented in the online assessment tools in GeoCat (http://geocat.kew.org). The Extent of Occurrence (EOO) measures the range of the species and the Area of Occupancy (AOO) represents the number of occupied points within that range based on the default grid size of 2 km^2^. We present only the EOO in the threat assessments for widespread species; AOO is very sensitive to georeferencing bias. For introduced taxa we also present their conservation status in the putatively native range in each of the species treatments.

Type specimens in this group have proved difficult to trace. Many taxa were described based on types housed in herbaria whose collections were lost during the Second World War, including the Berlin herbarium (B) (see Vorontsova and Knapp 2010). In our searches of many potential repositories for original material, we have been able to trace duplicates for at least some of these. For taxa recognised only as synonyms, we have cited the taxa in synonymy and indicated that duplicates have not been found rather than neotypifying these (often infraspecific) taxa. We have also made use of specimen images available via online databases: BP (for the herbarium of Pál Kitaibel, https://gallery.hungaricana.hu/hu/Herbarium/), G (http://www.ville-ge.ch/cjb/bd.php), P (https://science.mnhn.fr/institution/mnhn/collection/p/item/search/form), W (http://herbarium.univie.ac.at/database/search.php), Z (http://www.herbarien.uzh.ch/index.html), the African Plants Initiative and Global Plants (http://plants.jstor.org).

Many of the species and infraspecific names coined for European plants in the 19^th^ and early 20^th^ century floristic and other works have no specimens cited. In some cases, we have been able to trace potential original material, but in others, plants may have been described from living material. We have lectotypified these names where we have been able, but have not selected neotypes for those names that have rarely been used beyond their first description (mostly infraspecific taxa of *S. nigrum* and *S. villosum*). Where specific herbaria have not been cited in protologues, we have followed McNeill (2014) and designated lectotypes rather than assuming holotypes exist. We cite page numbers for all previous lectotypifications. In general, we have lectotypified names with the best preserved, or in some cases only, herbarium sheet we have seen; in these cases, we have not outlined our reasoning for the letotypifications. Where there has been difficulty or where the choice may not be obvious, we detail our reasoning at the end of the species discussions.

The amateur Czech botanist, Paul M. Opiz, collected prolifically and described many taxa based on his and other collections, most of these specimens being housed in PR or PRC (Kirschner et al. 2007). We have been unable to visit those collections during the course of this work, so have not lecto- or neotypified these names.

Georg Bitter (e.g., 1913a, 1917, 1921, 1923) described many taxa of *Solanum* in the course of his monumental work on African solanums and worked widely in Germany in the period between the two World Wars (Weber 1928), including, but not exclusively, at Berlin. His protologues frequently include specific herbarium citations, but not in all cases. We have cited specimens as holotypes only when a single specimen with a single herbarium citation is indicated in the protologue; we have not assumed his types are all in B.

Edmonds (1972, 1979b) cited as “holotype” specimens at single herbaria that are in fact more correctly cited as lectotypes; we have accepted these as effective lectotypifications (Art. 7.10, McNeill et al. 2012) but corrected her citation of “holotype” to lectotype. D’Arcy (1974a) cited “type”, “syntype” or “lectotype” for many of the names treated here in his treatment of *Solanum* for Flora of Panama. He explicitly cited some of these as “lectotype” and we treat these as validly published lectotypifications because his intention was clear. We also treat his citations as “type” coupled with the citation of a single herbarium as unintentional (“inadvertent”) lectotypifications (e.g. Prado et al. 2015), following the stipulations of Art. 7.10 of the *Code* (McNeill et al. 2012). We have tried to find previous lectotypifications to the best of our ability, but some done inadvertently in floras may have escaped our notice.

Type specimens are cited with their barcodes in square brackets after each herbarium code, written as they are spelled with barcode readers, either as a continuous string (i.e. [G00104280]) or with a dash (i.e. [MO–1781232]) depending on the style of barcode used in each herbarium. Sheet numbers are cited where barcodes are missing and these are indicated as distinct from barcodes (i.e. MO [acc. # 1037660]). For widespread species, we have not cited geographically representative specimens in the text under each species treatment, but instead listed the countries of occurrence by region (Africa, Asia, Europe, Pacific [incl. Australia]; also see Tables 1–3). Identities of all numbered collections seen for this study are in Supporting Material (Appendices 1–3, including Index to numbered collections and specimens cited in pdf, xls and csv formats). The full searchable specimen details are also available on the Solanaceae Source website (www.solanaceaesource.org) and in the dataset for this study deposited in the Natural History Museum Data Portal (https://doi.org/10.5519/0009648). For narrowly endemic taxa, we have cited all the specimens we have examined. For introduced taxa, we cite only Old World specimens in this treatment, but the data set on the NHM Data Portal (https://doi.org/10.5519/0009648) includes all material seen for these species, including New World collections examined for globally distributed species.

Citation of literature follows BPH-2 (Bridson 2004) with alterations implemented in IPNI (International Plant Names Index, http://www.ipni.org) and Harvard University Index of Botanical Publications (http://kiki.huh.harvard.edu/databases/publication_index.html). Following Knapp (2013), we have used the square bracket convention for publications in which a species is described by one author in a publication edited or compiled by another, the traditional “in” attributions such as Dunal in DC. for those taxa described by Dunal in Candolle’s *Prodromus Systematis Naturalis Regni Vegetabilis*. This work is cited here as Prodr. [A.P. de Candolle] and the names are thus attributed only to Dunal. For “ex” attributions, we cite only the publishing author, as suggested in the *Code* (McNeill et al. 2012). Standard forms of author names are according to IPNI (International Plant Names Index, http://www.ipni.org).

Common names and uses given under each species treatment are recorded for verified specimens only. The aim here is to provide an authoritative summary; further details on uses and names can be checked from the literature cited. Similarly, we refer to chromosome counts based on voucher specimens that we have been able to verify or based on studies that describe the voucher material in adequate enough morphological detail that allows us to draw firm conclusions on the identity of the original material. This means that some studies (e.g. Venkateswarlu and Bhiravamurty 1969) have been excluded.

## Taxonomic treatment

### The Morelloid clade sensu Bohs (2005) and Särkinen et al. (2013, 2015a)

*Solanum* grad. amig. *Maurella* Dunal, Hist. Solanum 119, 151. 1813. Lectotype species: *S. nigrum* L. (designated by D’Arcy 1972).

Solanum section Morella Dumort., Fl. Belg. 39. 1827. Lectotype species: *S. nigrum* L. (designated by D’Arcy 1972).

Solanum section Inermis G.Don, Gen. Syst. 4: 400. 1838. Lectotype species: *S. nigrum* L. (designated by D’Arcy 1972).

*Solanum* grad ambig. *Morella* G.Don, Gen. Syst. 4: 411. 1838. Lectotype species: *S. nigrum* L. (designated by D’Arcy 1972).

Solanum section Pachystemonum Dunal, Prodr. [A. P. de Candolle] 13(1): 28, 31. 1852. Lectotype species: *S. nigrum* L. (designated by D’Arcy 1972).

Solanum subsection Morella Dunal, Prodr. [A. P. de Candolle] 13(1): 28, 44. 1852. Lectotype species: *S. nigrum* L. (designated by D’Arcy 1972).

Solanum section Campanulisolanum Bitter, Repert. Spec. Nov. Regni Veg. 11: 234. 1912. Lectotype species: *S. fiebrigii* Bitter (designated by Seithe 1962).

Solanum section Episarcophyllum Bitter, Repert. Spec. Nov. Regni Veg. 11: 241. 1912. Lectotype species: *S. sinuatirecurvum* Bitter (designated by Seithe 1962).

Solanum section Morella (Dunal) Bitter, Bot. Jahrb. 54: 416, 493. 1917. Lectotype species: *S. nigrum* L. (designated by D’Arcy 1972).

Solanum section Chamaesarachidium Bitter, Repert. Spec. Nov. Regni Veg. 15: 93. 1919. Type species: *S. chamaesarachidium* Bitter (= *S. weddellii* Phil.).

*Solanum* series *Transcaucasica* Pojark., Bot. Mater. Gerb. Inst. Komarova Akad. Nauk S.S.S.R. 17: 332. 1955. Lectotype species: *S. transcaucasicum* Pojark. (= *S. villosum* Mill.) (designated by D’Arcy 1972 [as type]).

*Solanum* series *Alata* Pojark., Bot. Mater. Gerb. Inst. Komarova Akad. Nauk S.S.S.R. 17: 336. 1955. Type species: *S. alatum* Moench [nom. et typ. cons. prop.] (= *S. villosum* Mill.) (designated by D’Arcy 1972 [as type]).

*Solanum* series *Pseudoflava* Pojark., Bot. Mater. Gerb. Inst. Komarova Akad. Nauk S.S.S.R. 17: 338. 1955. Type species: *S. pseudoflavum* Pojark. (= *S. villosum* Mill.) (designated by D’Arcy 1972 [as type]).

Solanum section Parasolanum A.Child, Feddes Repert. 95: 142. 1984. Type species. *S. triflorum* Nutt.

Solanum section Dulcamara (Moench) Dumort. subsect. 2 “*herbaceous plants confined to the central Andes*” of Nee (1999: 295) [includes the species of Child’s section Parasolanum excluding the type].

Solanum section Solanum subsects. 1 “*Solanum*”, 2 “*Glandular pubescent group*”, 3 “*Campanulisolanum*”, 4 “*Chamaesarachidium*” and 6 “*Episarcophyllum*” of Nee (1999: 306–308), excluding his subsect. 5 “*Gonatotrichum*” [now recognised as being part of the Brevantherum clade, see Stern et al. 2013].

*Solanum* series *Lutea* Pojark. ex Ivanina, Bot. Zhurn. (Moscow & Leningrad) 85(6): 144. 2000. Type species. *S. villosum* Mill.

**Description**. Herbs, occasionally woody at the base; unarmed. Stems terete or angled, sometimes hollow, lacking true prickles but sometimes with prickle-like processes along the angles, glabrous or pubescent with simple or branched (only in the Americas) uniseriate trichomes, these eglandular or glandular. Sympodial units difoliate or trifoliate, the leaves usually not geminate. Leaves simple with entirely or variously dentate or lobed margins or occasionally deeply pinnatifid, concolorous, glabrous to densely pubescent with eglandular and/or glandular simple or branched (only in the Americas) uniseriate trichomes; petioles well developed or not, the leaves never sessile. Inflorescences opposite the leaves or arising internodally, unbranched or many times branched, not bracteate (except in *S. triflorum* where a single bracteole sometimes present), with few to many (up to 100) flowers; peduncle various, usually not longer than the inflorescence branches; pedicels articulated at the base. Flowers 5-merous, actinomorphic to very slightly zygomorphic, all perfect. Calyx with the lobes deltate to spathulate to long-triangular. Corolla stellate or rotate-stellate, white or purplish-tinged to lavender, usually with an “eye” at the base of the lobes of a contrasting colour (yellow, green or dark purple-black), the lobes spreading or reflexed at anthesis. Stamens equal or very slightly unequal, the filaments equal, glabrous or more usually densely pubescent with tangled uniseriate weak-walled simple uniseriate trichomes, the anthers ellipsoid (sometimes slightly tapering in *S. scabrum*) and connivent, with distal pores that elongate to slits with drying and/or age. Ovary conical, glabrous or occasionally very minutely puberulent; style straight or curved and bent, usually pubescent with simple uniseriate trichomes in the lower half, only very slightly exserted from the anther cone; stigma minutely capitate to capitate or clavate. Fruit a globose or somewhat elongate juicy berry with thin pericarp, green, black, yellow or red-orange at maturity; fruiting pedicels spreading or deflexed; fruiting calyx lobes reflexed, appressed or accrescent at fruit maturity. Seeds flattened and tear-drop shaped, yellow or tan-brown. Chromosome number: n=12, 24, 36 (see section on Chromosomes, and individual species treatments).

**Distribution.** A worldwide species group occurring on all continents except Antarctica, but with highest species diversity in South America and Africa.

**Discussion**. In the synonymy here, we have included all groups that are members of the clade as we define it, not only those containing Old World species; for more detailed discussion of morphology and group definition see Särkinen et al. (2015a). *Solanum nigrum* is the lectotype species of *Solanum* (Hitchcock and Green 1929) and thus, if this group were to be formally recognised at the infrageneric level at any given rank, it would necessarily be called [rank] *Solanum* (as recognised by Seithe 1962).

Members of the Morelloid clade are amongst the most widely collected of solanums, in part because are they are herbaceous, widespread and weedy. They are also amongst the most difficult to identify, due to their extreme vegetative plasticity (see Morphology above) and their lack of striking distinguishing characters. Combinations of characters are most useful for identification and we have included these in the species treatments as well as in the keys. Species are often most usefully identified based on geography (i.e. where they are found), but the large number of potentially invasive and introduced species means one must exercise caution if a species is not readily identifiable (taking into account variation, of course). The many specimens grown in the 19^th^ century in botanical gardens present special difficulties – these are not included here in species treatments or keys, except as type citations. Many morelloid species from the Americas were sent as seeds to European botanical gardens (e.g. *S. americanum*, *S. chenopodioides*, *S. emulans* Raf., *S. nigrescens* Martens & Galeotti) and, though cultivated, they did not escape and become part of the local floras of the regions. In order not to confuse users, we have limited our treatment here to include only native and a set of non-native introduced species that have become naturalised and persistent in the Old World.

The Morelloid clade suffers from two extreme sorts of taxonomic recognition issues. Firstly, in many parts of the world, especially in more recent floras, all taxa are treated as a single highly variable species (usually *S. nigrum*) and local endemic taxa are overlooked. Secondly and especially in Europe in the late 19^th^ and early 20^th^ century, many minor variants were described and then were transferred and recombined at different taxonomic levels, creating a confusing morass of names, many of which lack types. The latter is unfortunate because of the nomenclatural work entailed in sorting out the identities and types for these names, but the former is more serious, because endemic taxa have been overlooked and thus have possibly been placed at risk due to their being equated with widespread invasive weeds.

### Artificial key to species of the Morelloid clade occurring in the Old World (Africa, Asia, Europe and Australasia including the Pacific)

**Table:** 

1	Leaves 3-lobed nearly to the midrib; Australia (introduced and coastal)	***S. palitans***
1'	Leaves entire, sinuate-dentate or pinnatifid, but never regularly 3-lobed	**2**
2	Leaves shallowly to deeply pinnatifid	***S. triflorum***
2'	Leaves entire to sinuate-dentate	**3**
3	Glandular trichomes present (e.g. along stems, petioles and leaves), plants usually sticky to touch when fresh	**4**
3'	Glandular trichomes absent (e.g. along stems, petioles and leaves), plants not sticky to touch when fresh	**17**
4	Anthers < 1.8 mm long	**5**
4'	Anthers ≥ 1.8 mm long	**9**
5	Inflorescences with 10–40 flowers; pedicels spaced 1–2 mm apart, sharply bent at the base (near articulation point) in flower and fruit; Africa	***S. tarderemotum***
5'	Inflorescences with 2–5(-10) flowers; pedicels spaced 0–1 mm apart, nodding, erect or spreading in flower and fruit, reflexed and slightly curved in some species in fruit but never in flower; pantropical	**6**
6	Calyx lobes 1–1.5 mm long in flower; fruiting calyces not accrescent, the tube remaining 1–1.7 mm long and lobes 1–1.5 mm long; fruit black when ripe, not markedly shiny	**7**
6'	Calyx lobes 1.5–2.5 mm long in flower; fruiting calyces accrescent, the tube 3–4 mm long and lobes 2.5–8.0 mm long; fruit green when ripe, shiny	**8**
7	Leaves rhomboidal to lanceolate in shape; inflorescences unbranched, never furcate; buds globose; calyx lobes all equal in size, strongly reflexed in fruit; berries with a glaucous cast; southern Africa (also cultivated; introduced in Australia)	***S. retroflexum***
7'	Leaves elliptic to slightly ovate; inflorescences unbranched or rarely furcate; buds ellipsoid; calyx lobes unequal with two larger lobes, appressed to somewhat spreading in fruit; berries matte, but not markedly glaucous; Australasia and the Pacific	***S. opacum***
8	Leaf bases attenuate to cuneate; inflorescences mostly internodal, with 4–8(-10) flowers; pedicels spaced 0.3–1 mm apart; calyx lobes 1.7–2.5 mm long; corollas with yellow-green central eye with black-purple “V”-shaped margins; anthers 1.0–1.4 mm long; berries dark green to green-brown marbled with white lines, becoming usually translucent and shiny, lower half of berries covered with enlarged calyces but berry mostly visible; seeds brown; stone cells (1-)2–3, these 0.5 mm in diameter	***S. nitidibaccatum***
8'	Leaf bases truncate; inflorescences mostly leaf-opposed, with 2–5(-7) flowers; pedicels spaced 0(-1) mm apart; calyx lobes 1.5–2.0 mm long; corolla with yellow-green or translucent basal star without black-purple colouration; anthers 1.2–2.0 mm long; berries pale green, shiny becoming dull, opaque, usually completely enveloped by enlarged calyces; seeds pale yellow; stone cells 4–6, these (0.5-)0.8–1 mm in diameter	***S. sarrachoides***
9	Anthers ≥ 2.8 mm long	**10**
9'	Anthers < 2.8 mm long	**12**
10	Inflorescences with bracteoles present in most individuals; buds narrowly ellipsoid; corolla lobes narrowly lanceolate; berries with >30 stone cells	***S. triflorum***
10'	Inflorescences never with bracteoles; buds globose, ovoid or narrowly ellipsoid; corolla lobes variously triangular; berries with (0)2–4 stone cells	**11**
11	Flower buds globose to ovoid; calyx lobes 1–1.5(-2) mm long, long-triangular with rounded tips; corolla rotate-stellate; Arabian Peninsula to Egypt and south in eastern Africa	***S. memphiticum***
11'	Flower buds narrowly ellipsoid; calyx lobes 0.5–1.0 mm long, broadly deltate to triangular with acute tips; corolla stellate; Java to Lesser Sunda Islands	***S. alpinum***
12	Calyx lobes appressed to spreading in fruit, never strongly reflexed	**13**
12'	Calyx lobes strongly reflexed in fruit	**15**
13	Calyx accrescent in fruit, calyx tube 3–4 mm long and lobes 2.5–8 mm long	***S. sarrachoides***
13'	Calyx not accrescent in fruit, calyx tube 1–2 mm long and lobes 1–1.5 mm long	**14**
14	Buds ellipsoid; calyx tube 1.5–2.0 mm long, lobes 1–1.5 mm long, elongate-deltate with rounded tips; fruiting pedicels persisting when fruits mature and fall off; Cameroon line (Cameroon and Equatorial Guinea), usually above 2,000 m elevation	***S. pseudospinosum***
14'	Buds subglobose; calyx tube 0.8–1.0 mm long, lobes 0.5–0.8 mm long, triangular with rounded to acute tips; fruiting pedicels generally not persisting and falling off with mature fruits; common across Old World especially in temperate areas or below 1,000 m in the tropics and subtropics (Asia)	***S. nigrum***
15	Leaves rhomboidal to lanceolate; filaments 1.2–1.5 mm long, anthers 1.3–1.8(-2) mm long; seeds 1.6–1.8 mm long and 1.3–1.5 mm wide	***S. retroflexum***
15'	Leaves broadly to narrowly ovate to elliptic; filaments 0.5–1.3 mm long; anthers 1.8–2.5 mm long; seeds 1.8–2.2 mm long and 1.5–1.7 mm wide	**16**
16	Calyx with broad and relatively transparent sinuses, lobes elliptic to triangular, rounded at tip; free part of the filaments 1.0–1.3 mm long; mature berries slightly ellipsoid, shiny yellow, orange or red; stone cells always absent	***S. villosum***
16'	Calyx with narrow, sharp triangular sinuses, lobes deltate with acute or rounded tips; free part of the filaments 0.5–0.7 mm long; mature berries round, dull black or green; stone cells 0–4	***S. nigrum***
17	Anthers < 1.8 mm long	**18**
17'	Anthers ≥ 1.8 mm long	**21**
18	Pedicels spaced 1–2 mm apart, pedicels sharply bent at the base (near the articulation point) in flower and fruit; Africa	***S. tarderemotum***
18'	Pedicels spaced 0–0.5 mm apart, pedicels nodding, erect or spreading in flower and fruit; pantropical	**19**
19	Leaves with entire margins, occasionally sinuate-dentate; calyx lobes 0.3–0.5 mm long in flower, 1(-2) mm in fruit; mature fruits black, the surface very shiny	***S. americanum***
19'	Leaves shallowly toothed, occasionally entire; calyx lobes 1.0–1.5 mm long in flower, 1.5–2 mm in fruit; mature fruits purple-black or green, the surface matte	**20**
20	Leaves rhomboidal to lanceolate in shape; inflorescences unbranched, never furcate; buds globose; calyx lobes all equal in size, strongly reflexed in fruit; southern Africa (also cultivated; introduced in Australia)	***S. retroflexum***
20'	Leaves elliptic to slightly ovate; inflorescences unbranched or rarely furcate; buds ellipsoid; calyx lobes unequal with two larger lobes, appressed to somewhat spreading in fruit; Australasia and the Pacific	***S. opacum***
21	Anthers < 2.8 mm long	**22**
21'	Anthers ≥ 2.8 mm long	**31**
22	Berries without stone cells	**23**
22'	Berries with 2–22 stone cells	**28**
23	Pedicels persisting and not dropping with mature fruits; calyx lobes in fruit mostly strongly reflexed	**24**
23'	Pedicels dropping with mature fruits; calyx lobes in fruit appressed to slightly spreading, rarely strongly reflexed	**26**
24	Leaves rhomboidal to lanceolate; filaments 1.2–1.5 mm long, anthers 1.3–1.8(-2) mm long; seeds 1.6–1.8 mm long and 1.3–1.5 mm wide	***S. retroflexum***
24'	Leaves broadly to narrowly ovate to elliptic; filaments 0.5–1.3 mm long; anthers 1.8–2.5 mm long; seeds 1.8–2.2 mm long and 1.5–1.7 mm wide	**25**
25	Calyx with broad and relatively transparent sinuses, lobes elliptic to triangular, rounded at tip; filaments 1.0–1.3 mm long; mature berries slightly ellipsoid, shiny yellow, orange or red; stone cells always absent	***S. villosum***
25'	Calyx with narrow, sharp triangular sinuses, lobes deltate with acute tips; filaments 0.5–0.7 mm long; mature berries round, dull black or green; stone cells generally absent (2–4 stone cells common in Asian material)	***S. nigrum***
26	Buds elongate-oblong; fruiting peduncles strongly deflexed at the base (bent downwards at junction with the stem)	***S. chenopodioides***
26'	Buds ellipsoid to subglobose; fruiting peduncles straight or ascending	**27**
27	Pedicels spaced 1–2 mm apart, sharply bent at the base (near the articulation point) in flower and fruit; seeds 1.5–2 mm long and 1–1.5 mm wide; Africa	***S. tarderemotum***
27'	Pedicels spaced 0–0.7 mm apart, straight, spreading or reflexed in flower and fruit; pantropical; seeds 1.8–2 mm long and 1.5–1.6 mm wide; widespread	***S. nigrum***
28	Prostrate herb; leaves narrowly elliptic to lanceolate, base strongly attenuate; inflorescences with 1–5 flowers; pedicels stout and spreading; calyx lobes linear-oblong with rounded apices; mountains of Ethiopia	***S. hirtulum***
28'	Upright or spreading herb; leaves broadly ovate to elliptic, base acuminate, acute, obtuse, truncate to abruptly attenuate; inflorescences with 2–40 flowers; pedicels spreading to strongly reflexed; calyx lobes triangular, broadly deltoid or ovate with acute to rounded apices	**29**
29	Pedicels strongly bent at the base (near articulation point) in flower and fruit	***S. tarderemotum***
29'	Pedicels spreading, stout or pendent in flower, occasionally recurved in fruit but never strongly bent at the base	**30**
30	Inflorescences unbranched or more often branched, often with small leaves (bracteoles?); calyx lobes broadly deltate to mere enations of the rim; style exserted 1.0–1.5 mm beyond anther cone; mature berries 3–4(-5) mm in diameter, dull yellowish-brown	***S. umalilaense***
30'	Inflorescences unbranched, never with small leaves; calyx lobes triangular; style exserted 0–1 mm beyond anther cone; mature berries 6–10 mm in diameter, dull black	***S. nigrum***
31	Inflorescences with bracteoles present in most individuals; buds narrowly elliptic; berries with >30 stone cells	***S. triflorum***
31'	Inflorescences never with bracteoles; buds globose, ovoid or narrowly ellipsoid; berries with 0–14 stone cells	**32**
32	Berries without stone cells	**33**
32'	Berries with 2–14 stone cells	**35**
33	Buds narrowly ellipsoid; pedicels 1.0–1.3 cm long; anthers 2.8–3.8(-4) mm long	***S. alpinum***
33'	Buds elongate-oblong to globose; pedicels 0.5–1.0 cm long; anthers (2.0-) 2.3–3.0 mm long	**34**
34	Buds elongate-oblong; calyx lobes broadly deltate to triangular with acute tips; fruiting peduncles strongly bent at the base near junction with the stem; fruiting pedicels reflexed and slightly recurved; seeds 1.2–1.4 mm long and 1.0–1.2 mm wide	***S. chenopodioides***
34'	Buds globose-subglobose; calyx lobes broadly deltate with rounded tips; fruiting peduncles straight; fruiting pedicels stout, erect and spreading; seeds 2–2.8 mm long and 1.5–1.8 mm wide	***S. scabrum***
35	Leaves narrowly elliptic to lanceolate, bases strongly attenuate	**36**
35'	Leaves lanceolate, ovate or rhomboidal, bases cuneate-acute to occasionally attenuate	**37**
36	Calyx lobes 1.5–1.8 mm long, elliptic with long-acuminate to acute tips; anthers 3.0–3.8 mm long; Europe, occasional adventive associated with human activity, at or close to sea level	***S. pygmaeum***
36'	Calyx lobes 0.8–1.2 mm long, linear-oblong with rounded tips; anthers 2.3–2.8 mm long; Ethiopia, usually above 2,000 m elevation	***S. hirtulum***
37	Leaves ovate to rhomboidal; inflorescences furcate, occasionally simple; calyx lobes rectangular to narrowly obovate with obtuse to shortly acute tips; Australia and New Zealand (introduced at or close to sea level)	***S. furcatum***
37'	Leaves lanceolate to ovate, most commonly narrowly elliptic or broadly lanceolate; inflorescences always simple; calyx lobes broadly deltate to triangular with acute tips; Java and Lesser Sunda Islands, usually above 2,000 m elevation	***S. alpinum***

## Species descriptions

### 1. Solanum alpinum

Taxon classificationPlantaeSolanalesSolanaceae

Zoll. & Moritzi, Natuur- Geneesk. Arch. Ned.-Indië 2: 571. 1845.

Figure 5

Solanum viscidissimum Zoll. & Moritzi, Natuur- Geneesk. Arch. Ned.-Indië 2: 571. 1845.
Type. Indonesia. Java: Tengger [Tengger Range], above Gebok-Klacca [Klakah], 5500 ft, Oct 1844, *H. Zollinger 2514* (lectotype, designated here: G-DC [G00357866]; isolectotypes: BM [BM000778312], G [G00144593, G00343069], P [P00379791, P00379790], W [1889-0052800]).
Solanum dichrophyllum Dunal, Prodr. [A. P. de Candolle] 13(1): 48. 1852, nom. illeg. superfl.
Type. Based on (renaming of) Solanum alpinum Zoll. & Moritzi
Solanum anacamptocarpum Dunal, Prodr. [A. P. de Candolle] 13(1): 59. 1852.
Type. Indonesia. Java: Waliran [Gunung Welirang], 27 Aug 1844, *H. Zollinger 2177* (holotype: G-DC [G00144590]; isotypes: A [A00248838], BM [BM000886120], G [G00343304], MPU [MPU012652], P [P00369059, P00369060 [mixed sheet with right-hand stem belonging to S. nigrum], P00368907, P00368908]).
Solanum bromoense Kuntze, Revis. Gen. Pl. 453. 1891.
Type. Indonesia. Java: Mount Tengger, Bromo [Gunung Bromo], 2000 m, 15 Sep 1875, *O. Kuntze 5999* (lectotype, designated here: NY [0017228]; isolectotypes: GH [GH00077829], K [K000788117]).

#### Type.

Indonesia. Java: “in montibus Ardjune” [Gunung Arjuno], 2000–3000m, 14 Sep 1844, *H. Zollinger 2255* (lectotype, designated here: P [P00368905] (original label on this sheet with date and locality – “an 2177 var?”); isolectotypes: BM [BM000886119], G [G00144225, G00301669], MPU [MPU014201], P [P00368906]).

#### Description.

Annual or short-lived erect to somewhat spreading perennial herbs, height not known, likely subwoody and branching at base. Stems spreading to decumbent, terete, sometimes recorded as greenish-violet (*Afriastini 475*), older stems yellowish-brown, with no prickle-like projections, not markedly hollow; new growth densely pubescent with simple, spreading, uniseriate, glandular or eglandular trichomes, the trichomes 6-8(-10)-celled, ca. 0.5 mm long, if glandular then with a terminal gland, sometimes drying with a yellowish-brown tinge. Sympodial units trifoliate to plurifoliate, the leaves not geminate. Leaves simple, (2-)5–12 cm long, (0.5-)1–3 cm wide, lanceolate to ovate, most commonly narrowly elliptic or broadly lanceolate, membranous, concolorous, smell not known; adaxial surface pubescent with simple, uniseriate, glandular or eglandular trichomes evenly and moderately spread along veins and lamina; abaxial surface similar but the pubescence denser along the veins; major veins 7–8 pairs; base cuneate to attenuate; margins entire or shallowly toothed, the teeth, if present, narrow and acute; apex acute to acuminate; petioles ca. 1 cm long, pubescent like the stems and leaves. Inflorescences 2–4 cm long, internodal, unbranched or furcate, with 5–10 flowers mostly clustered at the distal end of the rhachis, pubescent with simple uniseriate trichomes like those of the stems and leaves; peduncle 1.0–3.5 cm long, straight; pedicels 1.0–1.3 cm long, ca. 0.5 mm in diameter at base and apex, filiform, spreading, pubescent with simple uniseriate trichomes like the rest of the inflorescence, articulated at the base; pedicel scars mostly clustered at the distal end of the rhachis and overlapping, sometimes up to 1.5 mm apart in the distal half of the rhachis. Buds narrowly ellipsoid, the corolla soon exserted from the calyx tube. Flowers 5-merous, all perfect. Calyx tube 1–2 mm long, broadly conical, the lobes 0.5–1.0 mm long, ca. 1 mm wide, broadly deltate to triangular, tips acute, densely to moderately pubescent with simple uniseriate 6–8-celled trichomes. Corolla 9–12 mm in diameter, purple or violet, stellate, lobed 2/3 to 3/4 of the way to the base, the lobes 3–4.5 mm long, 1.5–2.0 mm wide, spreading or reflexed, densely papillate along the margins and on the tips. Stamens equal; filament tube < 0.2 mm long; free portion of the filaments ca. 1 mm long, densely pubescent adaxially with tangled simple uniseriate trichomes; anthers 2.8–4 mm long, ca. 1 mm wide, ellipsoid, yellow, poricidal at the tips, the pores lengthening to slits with age and drying. Ovary rounded, glabrous; style 5–6 mm long, densely pubescent with tangled simple uniseriate trichomes in the basal half, exserted up to 2 mm from the anther cone; stigma minutely bilobed, the surfaces minutely papillate. Fruit a globose berry, 6–9 mm in diameter, black, the pericarp thin and matte; fruiting pedicels 1.2–1.3 cm long, ca. 0.5 mm in diameter at the base and apex, spreading or strongly deflexed, spaced (0-)0.5–1.5 mm apart, not falling with the berry, persistent on older inflorescences; fruiting calyx not accrescent, the tube less than 1 mm long, the lobes ca. 1 mm long, loosely appressed to the berry, not markedly reflexed. Seeds 20–50 per berry, 2.5–3.0 mm long, ca. 1 mm wide, flattened reniform, golden brown to brown, the surfaces minutely pitted, the testal cells small, rectangular to pentagonal in shape. Stone cells (0)2–4 per berry. Chromosome number not known.

**Figure 5.**
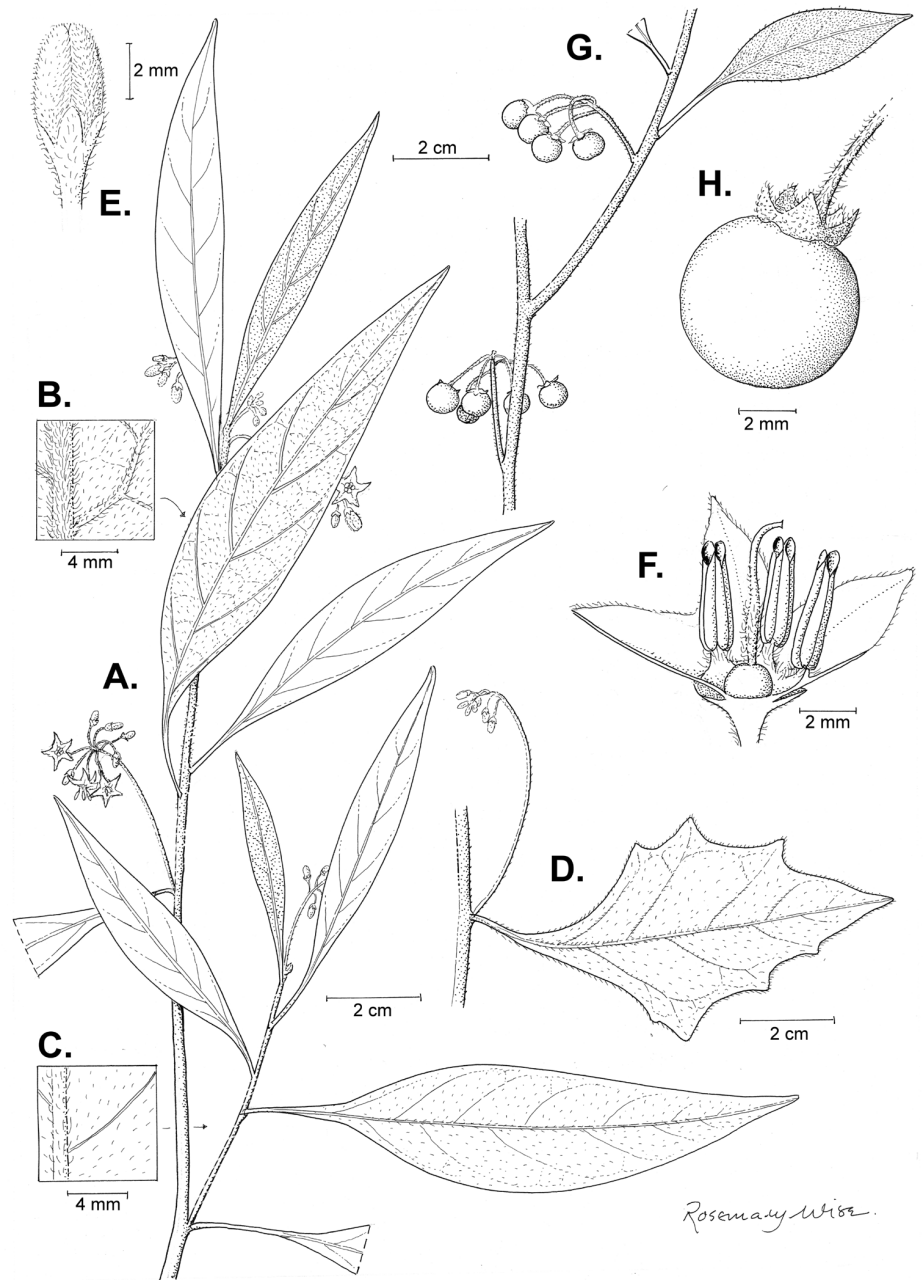
*Solanum alpinum* Zoll. & Moritzi **A** Flowering habit **B** Detail of abaxial leaf surface **C** Detail of adaxial leaf surface **D** Leaf with an inflorescence **E** Bud **F** Dissected flower **G** Fruiting habit **H** Fruit (**A–C, E–F** *Zollinger 2177*; **D** *Zollinger 2574*; **G–H** *Zollinger 2255*). Drawing by R. Wise.

#### Distribution

**Figure 6.**
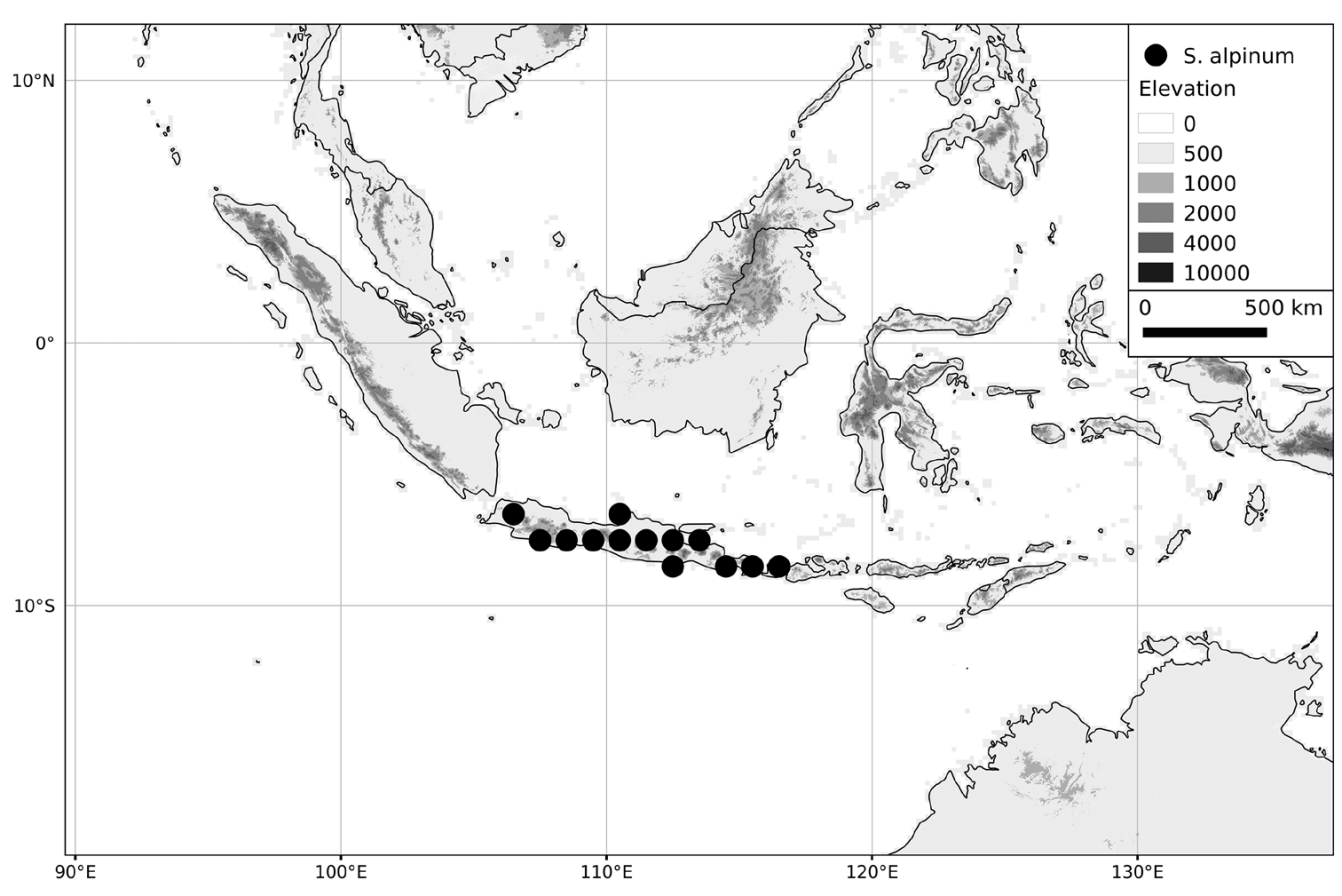
Distribution of *Solanum alpinum* Zoll. & Moritzi.

(Figure 6). Endemic to Indonesia and is found along the mountainous volcanic spine of Java, Bali and Lombok (Lesser Sunda Islands).

#### Ecology.

Grows in montane habitats, in grassy areas along forest margins or in clearings; between 1,700 and 3,100 m elevation.

#### Common names.

Indonesia: ranti hutan.

**Uses.** No specific uses recorded, but labels (*Afriastini 475*) indicate young berries are “edible but rather bitter”.

#### Preliminary conservation status

(IUCN 2016). *Solanum alpinum*, although found over a relatively large geographical area, is never common and is known from a small number of collections. With a relatively small AOO of 64 km^2^ (EN) and EOO of 18,806 km^2^ (VU) and, in view of the scattered populations at isolated high elevations, we assign the species a preliminary conservation status of VU (Vulnerable; Table 7). Forests on these mountains are under threat from land conversion and fire associated with human use (van Steenis 2006).

#### Discussion.

*Solanum alpinum* is a plant of high elevations with both glandular and eglandular morphs. Glandular forms were named as *S. viscidissimum*, while in the same publication eglandular forms were named *S. alpinum*. As the two names have not been synonymised before (neither name was included in Backer and Bakhuisen van der Brink 1965 or in van Steenis 2006), we have chosen to use *S. alpinum* over *S. viscidissimum* because the name is more generic and appropriate for the distribution of the species. It can be distinguished from *S. americanum* and *S. nigrum*, both of which also occur in Indonesia, by its larger usually violet flowers on long pedunculate inflorescences. Anthers of *S. alpinum* are 2.8–3.8(-4) mm long, while those of *S. americanum* are minute (ca. 1.5 mm) and those of *S. nigrum* ca. 2 mm long. The leaves are usually narrower and longer (more lanceolate) than either of those two species, but two Horsfield collections with smaller, rounder leaves are included here based on anther length and flower size, so variability could be greater than we have seen to date.

The plant illustrated as “Solanum nigrum” in van Steenis (2006: plate 51) is likely to be *S. alpinum*; the excellent illustration has the narrow leaves, long pedicels and large flowers characteristic of that species. The dismissal of it as a probable introduction has been the fate of all of the more narrowly distributed members of this group. The distribution of *S. alpinum* along the volcanic chain of Java above 2,000 m elevation is typical of other members of this rich flora that are found in areas created and influenced by fire resulting from volcanism and subsequent open habitat and grassland creation (as described in van Steenis 2006). *Solanum alpinum* is likely to also occur in the “tjemara forests” (*sensu* van Steenis 2006) dominated by *Casuarina junghuhniana* Miq. (Casuarinaceae). Further fieldwork and more accurately geolocated collections are needed to ascertain the distribution and habitat preferences of *S. alpinum*.

A single collection of Zollinger was cited for each of *S. alpinum* (“Herb. N. 2255”) and *S. viscidissimum* (“Herb. N. 2514”), but no herbarium was cited; we have lectotypified *S. alpinum* with the P (P00368905) duplicate of *Zollinger 2255*, because it has a label with the protologue locality. We have selected the G-DC (G00357866) sheet of *Zollinger 2514* as the lectotype for *S. viscidissimum* as the best preserved of the duplicates we have seen.

Dunal (1852) illegitimately coined the name *S. dichrophyllum* for *S. alpinum* because he felt the Zollinger’s and Moritzi’s name was inappropriate as the plant did not grow in the Alps (“Nomen alpinum mutavi quia in montibus certe alpinus non habitat” – I have changed the name because this certainly does not inhabit the Alps). He (Dunal 1852) used another sheet collected by Zollinger (*Zollinger 2177*) as the basis for his *S. anacamptocarpum*.

C.E.O. Kuntze named *S. bromoense* for Gunung Bromo in the Tenggar range, citing only “Java. Bromo 2000m” (Kuntze 1891). We have selected the sheet in NY (NY0017228) bearing the same locaity as the lectotype; Kuntze’s personal herbarium is held in NY.

#### Specimens examined.

**Indonesia**. **Bali**: bei der Quelle Jaritie auf Weg zum Gunung Ajaung, 2 Jun 1912, *Arens 19* (L); Kleine Soenda Eilanden, Bali, Z. helling Agoeng, 6 Apr 1936, *van Steenis 7839* (K);. **Java**: Central Java, Blumbang, Mt. Lawu, Central Java, 26 Nov 1982, *Afriastini 475* (A); West Java, Mt.Malabar, *Anderson 369* (CAL); East Java, Ardjoeno, tjemarabosch boven Lalidjiwo, 17 Oct 1915, *Arens s.n.* (L); East Java, 12 Oct 1915, *Arens 48* (L); East Java, Pasoeroean, G[unung] Tengge, boven Tosari, 4 Jun 1913, *Backer 8380* (L); East Java, Te Pasoeroean, Ngadisari, Jan 1925, *Backer 36563* (A); East Java, Pasoeroean, Tengge, boven Tosari, *Backer 36564* (L); Central Java, Soerkarta, Top van de Lawoe [Mount Lawu], 16 Jul 1936, *Brinkman 754* (NY); Sitiebondo, G[unung] Raneg [Raoeng] via Brembeinri, 15 May 1932, *Clason-Laarman EHH*-*157* (L); East Java, SE Java, 1880, *Forbes 1019* (BM); Central Java, Central Java, Slamet Mountain, between base camp Beraba-top of summit, 17 Mar 2004, *Hoover et al. Deden-113* (A); Central Java, Mt. Prahu, *Horsfield s.n.* (BM); Central Java, Surakarta, *Horsfield s.n.* (BM); Central Java, Mt. Prahu, *Horsfield s.n.* (BM); Central Java, Blambangan & Mt. Prahu, *Horsfield s.n.* (BM); East Java, Pasoeroean G[unung] Smeroe, *Jesweit s.n.* (L); Central Java, Tenggu, Penandjaan, 31 Jul 1932, *Kleinhoonte 371* (L); Central Java, Besoeki, G[unung] Merapi, 18 Jul 1916, *Koorders* & *Koorders-Schumacher 43218 B* (L); East Java, Res. Besoeki Kawah Idjen, 1 Nov 1916, *Koorders* & *Koorders-Schumacher 43214B* (L); East Java, Besoeki, Jang Plateau, 12 Aug 1916, *Koorders* & *Koorders-Schumacher 43463* (L); East Java, Tdjan plateau, Mt. Raung, Nov 1938, *Kostermans s.n.* (L); Central Java, G[unung] Muria, Tjollo, N of Kudus, 25 Nov 1951, *Kostermans 6278* (L); West Java, Malawar, Dec 1875, *Kuntze 5410* (DUKE, NY); West Java, Gunong Gede, 6 Mar 1979, *Murata et al. 2021* (L); East Java, Pasoeroean, 4 Jun 1935, *Neth Ind Forest Service 7038* (L); East Java, Pasoeroean, 4 Jun 1935, *Neth Ind Forest Service 7056* (A); East Java, Tosari 49, *Proefstation voor de Javasuikerindustrie s.n.* (L); East Java, Tosari 52, *Proefstation voor de Javasuikerindustrie s.n.* (L); Central Java, Central Java Merbaboe, Jul 1922, *Roorda van Eysinga s.n.* (MO); East Java, Tosari, Pasoeroean, *Teysmann s.n.* (CAL, K); East Java, Tengergebergte tusschen Ngadisari, Zandzee en Tosari, *Went s.n.* (L); Central Java, Meapi, Catu septr, *Without collector s.n.* (U); [iter Java secundum], *Zollinger 1790* (LE, P). **West Nusa Tenggara**: Klein Soenda Eilanden, Plawangan, Segara anak, 16 Jun 1936, *de Voogd 2654* (GH, NY); Lombok, Rindjani Vulkangebirge N Seite, 9 May 1909, *Elbert 1327* (L).

### 2. Solanum americanum

Taxon classificationPlantaeSolanalesSolanaceae

Mill., Gard. Dict. ed. 8, no. 5. 1768.

Figures 7 , 8

Solanum nigrum L. var. patulum L., Sp. Pl. 186. 1753.
Type. “Solanum procerius patulum, vulgaris fructu”, cultivated in England, at James Sherard’s garden in Eltham (Hortus Elthamensis) (lectotype, designated here: Dillenius, Hortus Elthamensis 2: 367, t. 275, f. 355. 1732). “Solanum procerius patulum, vulgaris fructu”, *Herb. Dillenius 441* (epitype, designated here [as lectotype by Edmonds 2012, pg. 136]: OXF [Dill-HE 275-355).
Solanum nodiflorum Jacq., Collectanea [Jacquin] 2: 288. 1789.
Type. Cultivated in Austria at Vienna, said to be from Mauritius (“crescit insula Mauritii”), *Herb. Jacquin s.n.* (lectotype, designated by Henderson 1974, pg. 28: BM [BM000617682]; isolectotype: W [W0022646]).
Solanum patulum (L.) Roth, Catal. Bot. 2: 23. 1800.
Type. Based on Solanum nigrum L. var. patulum L.
Solanum papilionaceum Dum.Cours., Bot. Cult. 2: 135. 1802.
Type. Cultivated from seeds received from the Jardin Nat. [Paris] (no specimens cited, likely to have been described from living material). Cultivated in Paris (“H.P.”), 1825, *Anon. [Herb. Maire] s.n.* (neotype, designated here: P [P00582223]).
Solanum strictum Zuccagni, Cent. Observ. Bot. [p. 20] No. 49. 1806.
Type. Cultivated in Italy; “Semina nobis communicavit Cl. Thouin sub nomine S. nigri sp. nova” (no specimens cited; Zuccagni’s herbarium originally at FI but destroyed). Cultivated in Italy in Bologna “j. de Bologna”, 6 Jul 1808, *Anon. s.n.* (neotype, designated here: G-DC [G00144215]).
Solanum rumphii Dunal, Hist. Nat. Solanum 157. 1813.
Type. Indonesia. Malaku: Amboina (no specimens cited; lectotype, designated here: Rumphius, Herbarium Amboinenese 6: t. 26, fig. 2, 1750).
Solanum oleraceum Dunal, Encycl. [J. Lamarck & al.] Suppl. 3: 750. 1814.
Type. “Antilles” *Herb, Richard s.n.* (lectotype, designated by D’Arcy 1974a, pg. 735: P [P00319557]; isolectotypes: G-DC [G00144258], MPU).
Solanum dillenii Schult., Öster. Fl., ed. 2, 1: 393. 1814, as “Dilleni”
Type. Hungary?. “In umbrosis Matra [Mátra]”, *P. Kitaibel s.n.* (lectotype, designated here: BP [Herb. Kit. fasc. IX, No. 102, small-flowered stem only]; isolectotype: B-W [B-W 04364-03]).
Solanum microspermum Dunal, Solan. Syn. 12. 1816.
Type. Origin unknown, 1815, *Anon. (Herb. Thibaud) s.n.* (lectotype, designated here: G-DC [G00144267]).
Solanum erythrocarpon G.Mey., Prim. Fl. Esseq. 109. 1818.
Type. Suriname. Saramacca: Hamburg (Essequibo), *E.K. Rodschied 31* (lectotype, designated here: GOET [GOET003505]).
Solanum desvauxii Ham., Prod. Pl. Ind. Occ. 26. 1825, nom. illeg. superfl.
Type. Based on S. nodiflorum Jacq. (cited in synonymy).
Solanum nigrum Vell., Fl. Flumin. 85. 1829 [1825], nom. illeg., not Solanum nigrum L. (1753)
Type. Brazil. [Rio de Janeiro]: “undequaeque nascitur” (lectotype, designated by Knapp et al. 2015, pg. 832: [illustration] Original parchment plate of Flora Fluminensis in the Manuscript Section of the Biblioteca Nacional, Rio de Janeiro [cat. no.: mss1198651_112] and later published in Vellozo, Fl. Flumin. Icon. 2: tab. 109. 1831).
Solanum tenuiflorum Steud., Nomencl. ed. 2, 2: 606. 1841.
Type. Based on (replacement name for) Solanum nigrum Vell.
Solanum indecorum A.Rich., Hist. Fls. Cuba, Fanerogamia 11: 121. 1841.
Type. Cuba. Sin loc., 1836, *R. de la Sagra s.n.* (lectotype, designated here: P [P00370899]).
Solanum nigrum L. subsp. nodiflorum (Jacq.) Sendtn., Fl. Bras. (Martius) 10: 16. 1846.
Type. Based on Solanum nodiflorum Jacq.
Solanum nigrum L. var. angulosum Sendtn., Fl. Bras. (Martius) 10: 16. 1846, as Solanum nigrum L. subsp. nodiflorum (Jacq.) Sendtn. var. angulosum Sendtn.
Type. Based on Solanum tenuiflorum Steud. (=Solanum nigrum Vell.)
Solanum nigrum L. subsp. aguaraquiya Sendtn., Fl. Bras. (Martius) 10: 17. 1846.
Type. Brazil. Rio Grande do Sul: “Pat. Joan a St. Barbara”, *C.F.P. Martius s.n.* (lectotype, designated here: M [M-0171809]; isolectotype: M [M-0171810]).
Solanum nigrum L. var. minus Hook.f., Trans. Linn. Soc. London 20(2): 201. 1847, as “*minor*”
Type. Ecuador. Galápagos Islands: James Island [Santiago], *C. Darwin s.n.* (lectotype, designated here: CGE [CGE00297]; isolectotype: K [K000922162]).
Solanum amarantoides Dunal, Prodr. [A. P. de Candolle] 13(1): 55. 1852.
Type. Brazil. Rio de Janeiro, *C. Gaudichaud 522* (lectotype, designated by D’Arcy 1974a, pg. 735 [as holotype]; second step designated here: P [P00319574]; isolectotypes: P [P00319575], MPU).
Solanum pterocaulum Dunal var. aguaraquiya (Sendtn.) Dunal, Prodr. [A. P. de Candolle] 13(1): 52. 1852, as ‘pterocaulon’.
Type. Based on Solanum nigrum L. subsp. aguaraquiya Sendtn.
Solanum ptychanthum Dunal, Prodr. [A. P. de Candolle] 13(1): 54. 1852.
Type. United States of America. Georgia: Chatham Co., Savannah, *Anon. s.n.* (holotype: G-DC [G00144485]).
Solanum nodiflorum Jacq. var. macrophyllum Dunal, Prodr. [A. P. de Candolle] 13(1): 46. 1852.
Type. Brazil. Rio de Janeiro: Rio de Janeiro, *C. Gaudichaud 521* (lectotype, designated by D’Arcy 1974a, pg. 735: P [P00319582]; isolectotypes: P [P00319583, P00319585], G-DC [G00144100], G [G00343373]).
Solanum nodiflorum Jacq. var. acuminatum Dunal, Prodr. [A. P. de Candolle] 13(1): 46. 1852.
Type. Brazil. Minas Gerais: Sin loc., *M. Vauthier 537* (lectotype, designated by D’Arcy 1974a, pg. 735 [as type ex Herb. Drake]: P [P00319615]; isolectotypes: P [P00319614], G-DC [G00343360]).
Solanum nodiflorum Jacq. var. petiolastrum Dunal, Prodr. [A. P. de Candolle] 13(1): 46. 1852.
Type. Brazil. Rio de Janeiro: Novo Friburgo, 1842, *P. Claussen 180* (holotype: P [P00319584]).
Solanum inops Dunal, Prodr. [A. P. de Candolle] 13(1): 55. 1852.
Type. Mexico. “sin. loc.” [Tamaulipas: Tampico, 4 Feb 1827], *J.L. Berlandier 46* (holotype: G-DC [G00144469]; isotypes: BM [BM000775579], F [V0073104F, acc. # 680275], LE, P [P00336046, P00336047, P00336048], W [1889-0291394, 1889-0144848]).
Solanum nigrum L. forma nodiflorum (Jacq.) Miq., Fl. Ned. Ind. 2: 637. 1856.
Type. Based on Solanum nodiflorum Jacq.
Solanum nigrum L. forma rumphii (Dunal) Miq., Fl. Ned. Ind. 2: 637. 1856.
Type. Based on Solanum rumphii Dunal
Solanum nigrum L. forma nodiflorum (Jacq.) Miq., Fl. Ned. Ind. 2: 637. 1856.
Type. Based on Solanum nodiflorum Jacq.
Solanum nigrum L. forma uniflorum Miq., Fl. Ned. Ind. 2: 638. 1856.
Type. Indonesia. Java: “op den Diëng, 6000-8000 ft”, *F.W. Junghuhn s.n.* (lectotype, designated here: U [U0113977]).
Solanum patulum Kit. ex Kanitz, Linnaea 32: 440. 1863, nom illeg., not Solanum patulum (L.) Roth (1800).
Type. Based on Solanum dillenii Schult. (cited in synonymy)
Solanum nigrum L. subsp. dillenii (Schult.) Schur, Enum. Pl. Transsilv. 478. 1866.
Type. Based on Solanum dillenii Schult.
Solanum nigrum L. var. dillenii (Schult.) A.Gray, Synopt. Fl. N. Amer. 2(1): 228. 1878.
Type. Based on Solanum dillenii Schult.
Solanum nigrum L. var. nodiflorum (Jacq.) A.Gray, Synopt. Fl. N. Amer 2(1): 228. 1878.
Type. Based on Solanum nodiflorum Jacq.
Solanum nigrum L. var. oleraceum (Dunal) Hitchc., Rep. Missouri Bot. Gard 4: 111. 1893.
Type. Based on Solanum oleraceum Dunal
Solanum nigrum L. var. nodiflorum (Jacq.) Hitchc., Rep. (Annual) Missouri Bot. Gard. 4: 111. 1893, nom. illeg., not Solanum nigrum L. var. nodiflorum (Jacq.) A.Gray (1878)
Type. Based on Solanum nodiflorum Jacq.
Solanum nigrum L. var. americanum (Mill.) O.E.Schulz, Symb. Antill. (Urban) 6: 160. 1909.
Type. Based on Solanum americanum Mill.
Solanum nigrum L. forma grandifolium O.E.Schulz, Symb. Antill. (Urban) 6: 160. 1909, as Solanum nigrum L. var. americanum (Mill.) O.E.Schulz forma grandifolia O.E.Schulz
Type. Puerto Rico. “prope Cayey in sylvis ad rivulum superiorem m. Sept. fl. et. fr.”, *P.E.E. Sintenis 2429* (probably type, no herbarium cited).
Solanum nigrum L. forma parvifolium O.E.Schulz, Symb. Antill. (Urban) 6: 160. 1909, as Solanum nigrum L, var. americanum (Mill.) O.E.Schulz forma parvifolia O.E.Schulz.
Type. Cuba. La Habana: Santiago de las Vegas, “*Baker Herb. Cub. 3377*” (probably type, no herbarium cited).
Solanum minutibaccatum Bitter, Repert. Spec. Nov. Regni Veg. 10: 549. 1912.
Type. Bolivia. La Paz: San Carlos, bei Mapiri, 750 m, Aug 1907, *O. Buchtien 1443* (lectotype, designated here: US [00027684, acc. # 1175843]; isotypes: GOET [GOET003478], NY [00172089]).
Solanum inconspicuum Bitter, Repert. Spec. Nov. Regni Veg. 11: 204. 1912.
Type. Peru. Lima: Lima, 12 Jul 1910, *C. Seler 222* (holotype: B, destroyed; no duplicates found).
Solanum tenellum Bitter, Repert. Spec. Nov. Regni Veg. 11: 219. 1912.
Type. Brasil. Minas Gerais: “Prope urbem Caldas florens fructibusque instructum”, 4 Oct 1869, *A.F. Regnell III 970* (holotype: UPS; isotypes: US [00027821, acc. # 201069, 01931849, acc. # 201352]).
Solanum minutibaccatum Bitter subsp. curtipedunculatum Bitter, Repert. Spec. Nov. Regni Veg. 11: 205. 1912.
Type. Bolivia. La Paz: Guanai-Tipuani, Apr-Jun 1892, *M. Bang 1462* (holotype: W; isotypes: BM [BM000617672], E [E00106087], M [M-0171808], MO [MO-503647], NDG [NDG42278], NY [00172090, 00172091, 00172092], PH [PH00030453], US [00027685, acc. # 1324656], WIS [0256198WIS]).
Solanum sciaphilum Bitter, Repert. Spec. Nov. Regni Veg. 11: 220. 1912.
Type. Brazil. Santa Catarina: Pedras Grandes, Aug 1890, *E. Ule 1678* (holotype: B, destroyed, F neg. 2851; lectotype, designated here: HBG [HBG-511539]; isolectotype: HBG [HBG-511540]).
Solanum curtipes Bitter, Repert. Spec. Nov. Regni Veg. 11: 228. 1912.
Type. Paraguay. Cordillera: San Bernardino, Aug 1898-1899, *É. Hassler 3104* (holotype: B, destroyed; lectotype, designated by Morton 1976, pg. 149; second step designated here: G [G00306710]; isolectotypes: G [G00306711, G00306712, G00306713, G00306714], K [K000532497], P [P00325762], NY [00139112], UC [UC950837]).
Solanum calvum Bitter, Repert. Spec. Nov. Regni Veg. 12: 81. 1913.
Type. Mexico. Baja California: Guadalupe Island, 1875, *E. Palmer 60* [pro parte] (holotype: UPS; isotypes: BM [BM001017192], MO [MO-159620, MO-568722], NY [00138967, 00759880], YU [YU065319]).
Solanum depilatum Bitter, Repert. Spec. Nov. Regni Veg. 12: 88. 1913.
Type. Madagascar. Toliara: Fort Dauphin [anchorage], 1897, *G. Paroisse 10* (holotype: P [P00338747]).
Solanum imerinense Bitter, Bot. Jahrb. Syst. 49: 566. 1913.
Type. Madagascar. Antananarivo: “Central Madagaskar, Imerina”, Dec 1880, *J.M. Hildebrandt 3796* (lectotype, designated by Edmonds 2012, pg. 136: M [M-0105626]; isolectotypes: CORD [CORD00006927], P [P00338727, P00338738], BM [BM000887188]).
Solanum sancti-thomae Bitter, Bot. Jahrb. Syst. 49: 560. 1913, as “*Sancti Thomae*”
Type. São Tome and Principe: São Tome, *F. Quintas & A. Moller 47* (syntypes: B, destroyed, COI, not located).
Solanum nodiflorum Jacq. var. sapucayense Chodat, Bull. Soc. Bot. Genève, sér. 2, 8: 150. 1916.
Type. Paraguay. Paraguarí: Sapucaí [“Sapucay”], 1914, *R. Chodat & W. Vischer 46* (holotype: G [G00306708]).
Solanum nigrum L. subsp. dillenii (Schult.) Probst, Mitteil. Naturfor. Gesellsch. Solothurn 9: 33. 1932.
Type. Based on Solanum dillenii Schult.
Solanum nigrum L. var. pauciflorum Liou, Contr. Inst. Bot. Natl. Acad. Peiping 3: 454. 1935.
Type. China. Hainan: Ngai District, Yeung Ling Shan, 5 Jun 1932, *S.K. Lau 209* (lectotype, designated here: BM [BM000942311]; isolectotypes: A, LU?, K [K001152446]).
Solanum merrillianum Liou, Contr. Inst. Bot. Nat. Acad. Peiping 3: 455. 1935.
Type. China. Hainan: Thai Hang, Shek Kuet Ts’o, Lin Fa Shan and vicinity, Lam Ko District, *W.T. Tsang 412* (holotype: LU [acc. no. 15911, not seen]; isotypes: A [A00077824, A00395157], E [E00718800], MO [acc. # 1037660], S [acc. # S-G-5703]).
Solanum photeinocarpum Nakam. & Odash., J. Soc. Trop. Agric., Taiwan 8: 54. 1936.
Type. Taiwan. “Taihoku” [Taipei?], 28 Feb 1936, *K. Odashima 17720* (lectotype, designated here: TAI).
Solanum pachystylum Polg., Trans.& Proc. Roy. Soc. N. Z. 69: 280. 1940.
Type. New Zealand. North Island: Auckland, Mt. Wellington, near Auckland, Plant Research Station, *H.H. Allan s.n.* (holotype: CHR [8954]; isotypes: CHR [8954 A], CHR [8954 B]).
Solanum americanum Mill. var. nodiflorum (Jacq.) Edmonds, J. Arnold Arb. 52: 634. 1971.
Type. Based on Solanum nodiflorum Jacq.
Solanum suffruticosum Schousb. ex Lange var. merrillianum (Liou) C.Y.Wu & S.C.Huang, Fl. Hainan. 3: 586. 1974.
Type. Based on Solanum merrillianum Liou
Solanum nodiflorum Jacq. subsp. nutans R.J.F.Hend., Contr. Queensland Herb. 16: 30. 1974.
Type. Australia. Queensland: Brisbane, Dept. Primary, Industrial grounds, 3 Jul 1969, *R.J.F. Henderson 518* (holotype: BRI [AQ0023172]; isotypes K [K001080528], NSW [NSW568939], MEL [MEL2289999A]).
Solanum americanum Mill. var. patulum (L.) Edmonds, Bot. J. Linn. Soc. 75: 171. 1977.
Type. Based on Solanum nigrum L. var. patulum L.
Solanum americanum Mill. subsp. nodiflorum (Jacq.) R.J.F.Hend., Austrobaileya 2: 555. 1988.
Type. Based on Solanum nodiflorum Jacq.
Solanum pauciflorum (Liou) H.Y.Zhang, Bull. Bot. Res., Harbin 19(2): 131. 1999.
Type. Based on Solanum nigrum L. var. pauciflorum Liou

#### Type.

Cultivated at the Chelsea Physic Garden [in protologue said to “grow naturally in Virginia”], *Herb. Miller s.n.* (lectotype, designated by Edmonds 1972, pg. 103 [as type]: BM [BM000617683]).

#### Description.

Annual to short-lived erect or weakly scrambling perennial herbs up to 1.5 m tall, subwoody and branching at base. Stems spreading, terete or somewhat angled with ridges, green to somewhat purple tinged, older stems often appearing spinescent, not markedly hollow; new growth pubescent with simple, spreading, uniseriate, translucent, eglandular trichomes, these 2-8-celled, 0.2–0.8 mm long, often clustered along the stem angles; older stems glabrescent, with only the trichome bases persisting as pseudo-spines. Sympodial units difoliate, the leaves not geminate. Leaves simple, 3.5–10.5 cm long, 1.0–4.5 cm wide, ovate to elliptic, membraneous, concolorous, without odour; adaxial surface sparsely pubescent with simple, uniseriate trichomes like those on stem, these evenly spread along the lamina and the veins; abaxial surface similar but more densely pubescent; major veins 3–6 pairs; base attenuate, decurrent on the petiole; margins entire or occasionally sinuate-dentate; apex acute; petioles (0.3-)2.0–3.8(-4.0) cm long, sparsely pubescent with simple uniseriate trichomes like those on stems. Inflorescences 0.6–2.5 cm long, internodal, simple or very rarely furcate, umbelliform to sub-umbelliform, with (3-)4–6(-8) flowers (very rarely with more flowers in branched inflorescences), sparsely pubescent with simple uniseriate trichomes like those on stems; peduncle (0.5-)1.0–1.8 cm long, straight and stout; pedicels 3–9 mm long, 0.2–0.3 mm in diameter at the base and 0.4–0.5 mm at the apex, stout, straight and spreading, articulated at the base; pedicel scars spaced 0–0.5 mm apart, clustered at the tip of the inflorescence. Buds broadly ellipsoid, the corolla 1 exserted/3 beyond the calyx lobe tips before anthesis. Flowers 5-merous, all perfect. Calyx tube 0.8–1.3 mm long, conical, the lobes 0.3–0.5 mm long, 0.5–0.6 mm wide, broadly triangular, the tips obtuse, sparsely pubescent with simple uniseriate trichomes like those of the stem. Corolla 3–6 mm in diameter, white with a yellow-green central portion near the base, stellate, lobed 1/2-2/3 of the way to the base, the lobes 2.0–3.2 mm long, 1.0–2.5 mm wide, strongly reflexed at anthesis, later spreading, densely papillate abaxially with 1-4-celled simple uniseriate trichomes, these denser on the tips and margins. Stamens equal; filament tube minute; free portion of the filaments 0.5–0.8 mm long, adaxially pubescent with tangled uniseriate trichomes; anthers 0.8–1.5 mm long, 0.5–0.6 mm wide, ellipsoid to almost globose, yellow, poricidal at the tips, the pores lengthening to slits with age and drying. Ovary globose, glabrous; style 2.2–2.6 mm long, densely pubescent with 2-3-celled simple uniseriate trichomes in the lower 2/3 where included in the anther cone, almost included to exserted 0.5(-1.0) mm beyond the anther cone; stigma minutely capitate, the surface minutely papillate, green in live plants. Fruit a globose berry, 4–9(-12) mm in diameter, purplish-black at maturity, the pericarp thin and markedly shiny; fruiting pedicels 13–18 mm long, 0.7–1.0 mm in diameter at the base and 0.8–1.0 mm at apex, stout, straight and spreading, spaced 0–0.5 mm apart, not falling with the fruit, remaining on the plant and persistent on older inflorescences; fruiting calyx not accrescent, the tube less than 1 mm long, the lobes 1(-2) mm long, strongly reflexed at fruit maturity. Seeds 30–50 per berry, 1.0–1.5 mm long, 0.8–1.3 mm wide, flattened and tear-drop shaped with a subapical hilum, pale yellow, the surfaces minutely pitted, the testal cells pentagonal in outline. Stone cells mostly absent (Australia, South Pacific and South America), but if present (North America, Mexico, Eurasia and Africa) 2–4(6) per berry, 2–4 larger ones >0.5 mm and two smaller ones <0.5 mm in diameter. Chromosome number: *2n*=2x=24 (Tokunaga 1933 [as *S. dillenii*]; Nakamura 1937 [as *S. photeinocarpum*]; Stebbins and Paddock 1949 [as *S. nodiflorum*]; Heiser 1955 under *S. nodiflorum*; Baylis 1958 as *S. nodiflorum*; Soria and Heiser 1961 [as *S. nodiflorum*]; Heiser et al. 1965 [as *S. nodiflorum*]; Edmonds 1972, 1977, 1982, 1983, 1984a; Venkateswarlu and Rao 1972 [vouchers labelled as *S. nigrum* S14, S30, S31]; D’Arcy 1974a; Henderson 1974; Tandon 1974; Bhiravamurty 1975 [as *S. nodiflorum*]; Randell and Symon 1976; Symon 1981; Ganapathi and Rao 1986a; Symon 1985; Schilling and Andersen 1990; Bukenya 1996; Jacoby and Labuschagne 2006; Moyetta et al. 2013; Olet et al. 2015).

**Figure 7.**
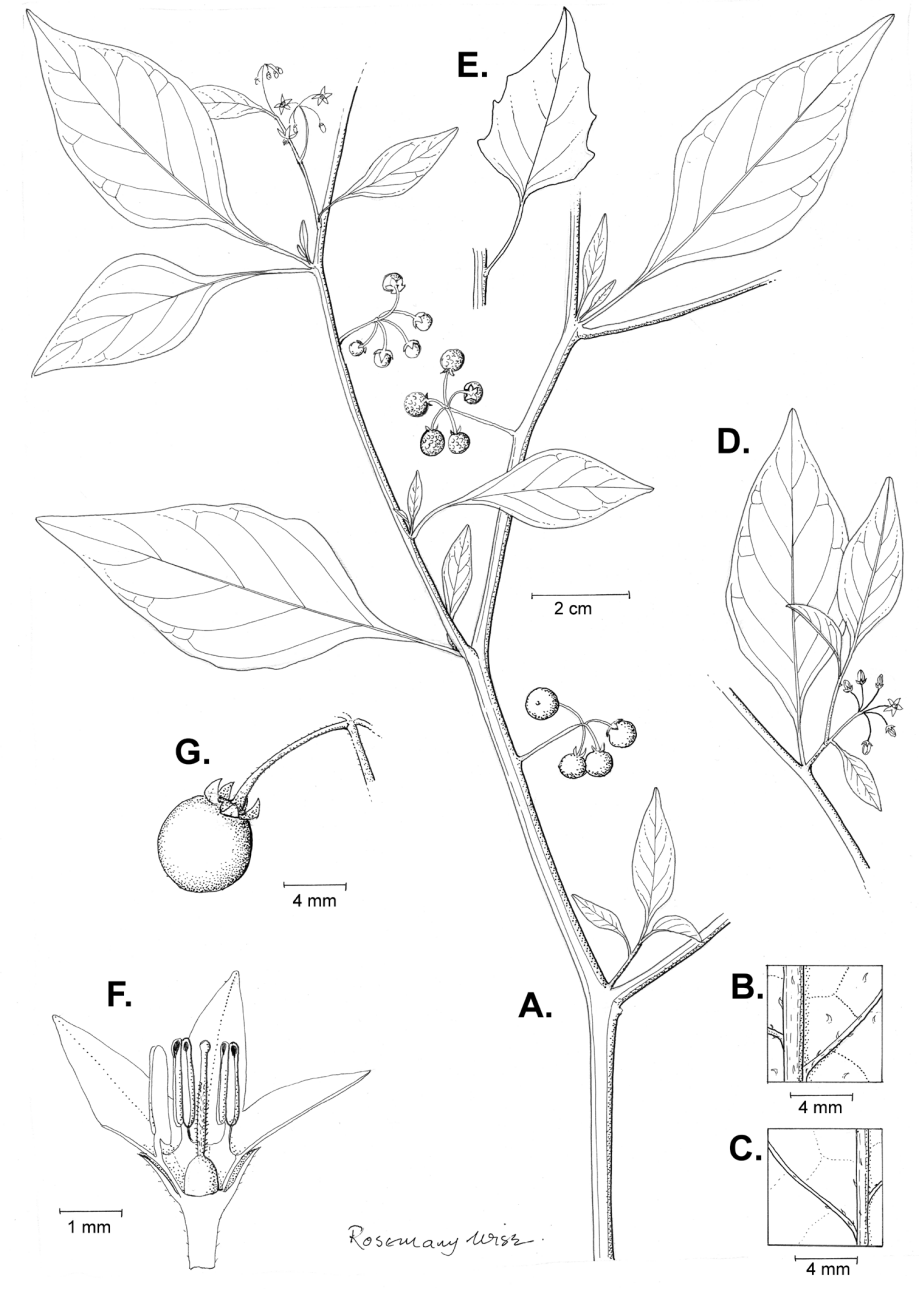
*Solanum americanum* Mill **A** Habit **B** Detail of abaxial leaf surface **C** Detail of adaxial leaf surface **D** Branch with inflorescence **E** Leaf **F** Dissected flower **G** Fruit (**A–D, F–G** *Cremers 8084*; **E** *Farrugia 2773*). Drawing by R. Wise.

**Figure 8.**
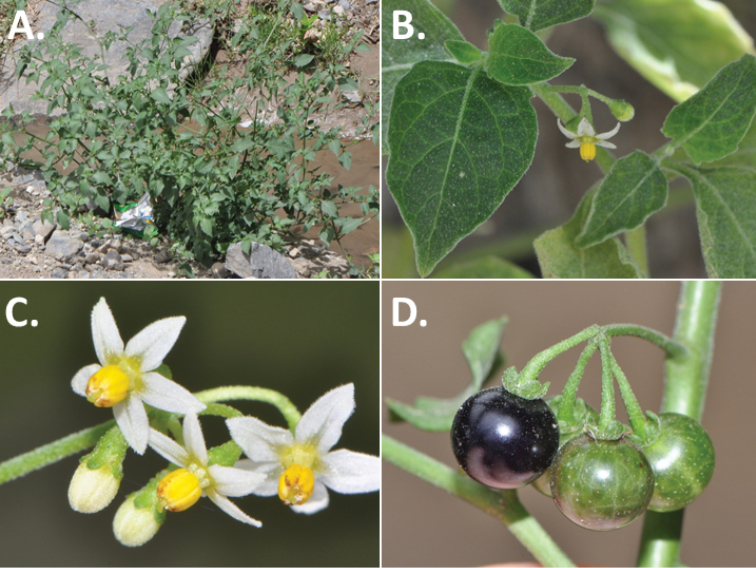
*Solanum americanum* Mill **A** Habit **B** Leaves and young inflorescence **C** Buds and flowers **D** Mature, shiny black fruits with reflexed calyx lobes (**A, D** *Knapp et al. 10210*; **B** *Knapp et al. 10205*; **C** *Knapp et al. 10360*). Photos by S. Knapp.

#### Distribution

**Figure 9.**
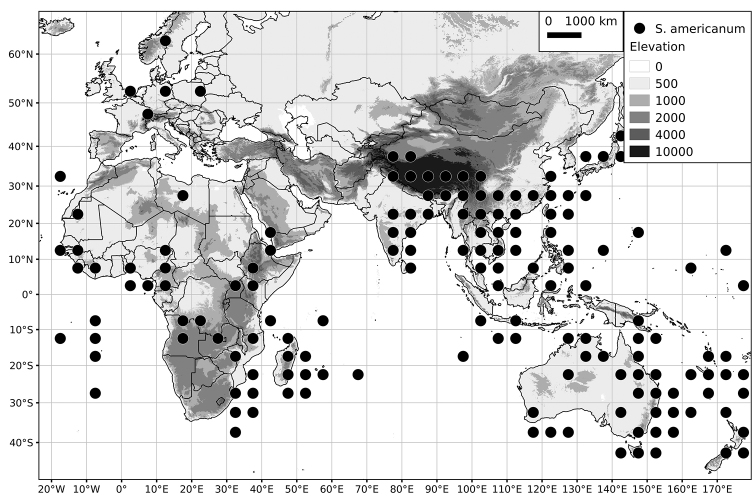
Distribution of *Solanum americanum* Mill in the Old World.

(Figure 9). Globally distributed weed found across tropical and subtropical areas; probably native to the Americas, but there is little evidence for its origin or introduction.

#### Ecology.

Grows in disturbed habitats and associated with human activities in tropical moist to dry areas, in dry areas often found growing in full shade close to water sources; between sea level and 2,000 (-2,500) m elevation.

#### Common names.

American Samoa: magalo; Australia: glossy nightshade (Symon 1981); Benin: odu, ogomo, feibii (Essou and Hermans 2006); China: shao hua long kui, guang zhi mu long kui (as *S. merrillianum*) (Zhang et al. 1994); Finland: amerikanmustakoiso (Hämet-Ahti et al. 1998); Ghana: ebibirba; Indonesia. booso, doehet ratti, lohoet/lohoetoe [lohoetoe-lohoetoe] ranti; Kenya: mnairi; Madagascar: anamama; Malaysia: beliwan, lutan, ranti, tutan, tutan-toposi; Mauritius: brede martin; New Zealand: small-flowered nightshade (Webb et al. 1988); Nigeria: odú; Niue: plo fua, polo kai; Norfolk Island: ang’adsindra, bwamunovi; Papua New Guinea: tuskombuk; Philippines: amti niitang [Ifugao people], onti; South Africa: black nightshade; Sri Lanka: kalu kamberiya; Sweden: Amerikansk nattskatta (Mossberg et al. 2003); Tanzania: imbenek, mavu, mnafu, mnaru, mwihakhi; Tonga: polo kai, polo tonga; Uganda: ocuga; United States of America[Hawaii]: popolo, popolohua; Vanuatu: ne poro, poro.

#### Uses.

In all parts of its range, the leaves of *S. americanum* are used as spinach and the ripe berries are eaten, either raw or cooked.

#### Preliminary conservation status

(IUCN 2016). *Solanum americanum* is an extremely widespread cosmopolitan weed and can be assessed as LC (Least Concern; Table 7).

#### Discussion.

*Solanum americanum* is a diploid species that can be easily recognised by its shiny black fruits on spreading pedicels with strongly reflexed calyx lobes (to parallel with the pedicel) that are somewhat papillate abaxially. In fruit, the pedicels remain on the plant after fruits fully mature and drop off, leaving behind a distinct group of tightly clustered spreading pedicels with reflexed calyx lobes; this character is easily visible in many herbarium specimens. In flower, *S. americanum* has tiny, almost globose anthers 0.8–1.5 mm long borne on short filaments. It can be distinguished from *S. opacum*, which also has tiny anthers of the same size, in its shorter filaments relative to anther size and in its deltate calyx lobes with rounded tips. *Solanum opacum* has longer filaments relative to anther size, long-triangular calyx lobes and, in fruit, the calyx lobes are appressed to the base of the berry. Three other morelloids with such small anthers can be difficult to distinguish from *S. americanum* and are often confused with it in herbaria. *Solanum emulans* Raf. does not occur in the Old World (except sometimes in old botanical garden collections, see discussion of the typification of *S. patulum* and *S. dillenii* below; it is a species of the north-eastern United States and Canada) has matte berries and longer calyx lobes. *Solanum nitidibaccatum* also has extremely small anthers, but can be easily distinguished by its glandular pubescence and accrescent calyx in fruit. *Solanum opacum* is the third morelloid with tiny anthers and the most difficult to distinguish from *S. americanum* in the Old World. *Solanum americanum* and *S. opacum* co-occur across the Pacific and distinguishing individual specimens can be difficult, but the shiny fruits (versus matte in *S. opacum*) and persistent pedicels with strongly reflexed calyx lobes are good characters by which to recognise *S. americanum*.

In southeast Asia and China, *S. americanum* is at least partially sympatric with *S. nigrum* (see discussion of *S. nigrum*). The species can be distinguished by anther size (0.8–1.5 mm versus ca. 2–2.5 mm) and by inflorescence morphology; *S. americanum* usually has few flowers that are tightly congested in the distal part of the inflorescence, while *S. nigrum* usually has more flowers that are more spaced out along the inflorescence rhachis, although young inflorescences of *S. nigrum* can appear sub-umbellate. In fruit, the strongly reflexed calyx lobes of *S. americanum* are distinctive and the seeds are smaller than those of the hexaploid *S. nigrum* (ca. 1 mm versus ca. 2 mm long).

*Solanum merrillianum* was recognised as a distinct species in the Flora of China (Zhang et al. 1994), but variation described fits within the observed variation of *S. americanum* after studies of the species across its global range. Specimens described as *S. merrillianum* show branching inflorescences in some individuals with generally larger number of flowers per inflorescence than seen in *S. americanum*, but the larger inflorescences may be due to pre-domestication and/or selection of the species in China and Taiwan, where fruits of *S. americanum* are commonly eaten.

*Solanum americanum* exhibits the highest infraspecific genetic diversity compared to polyploids (Dehmer and Hammer 2004). Based on its distribution, molecular and crossing experiments, it is believed to be the diploid parent of the two hexaploids *S. nigrum* and *S. scabrum* (Edmonds 1979a, Ganapathi and Rao 1987a, 1987b). There are a relatively few differences between the two species in SSR (Dehmer 2001), AFLP (Dehmer and Hammer 2004), RAPD (Poczai et al. 2010), ISSR and SCoT (Poczai and Hyvönen 2011) and intron-targeting markers (Poczai et al. 2014) and a number of additive bands could be counted between *S. americanum* and the two hexaploids indicating their parental relationships. *Solanum americanum* is also the putative parent of the tetraploid *S. villosum* (Poczai and Hyvönen 2011).

The taxonomic status and relationship of *S. americanum* to *S. nodiflorum* was studied by Manoko et al. (2007). *Solanum nodiflorum* has been considered as a distinct taxon by some (e.g. Henderson 1974, Heiser et al. 1979) while a synonym or infraspecific taxon of *S. americanum* by others (e.g. Edmonds 1971; D’Arcy 1974a, b; Symon 1981). Manoko et al. (2007) used AFLP data to study the relationship between the two taxa, but used different taxon concepts than we adopt here, in part because their examination of type specimens was limited. Based on detailed study of the voucher material used, it is clear that the taxon referred to as *S. nodiflorum* in Manoko et al. (2007) refers to what is considered *S. americanum* in this treatment, while material referred to as *S. americanum* represents *S. nigrescens* M.Martens & Galeotti, a species endemic to the New World. Such confusion is easy in this complex group when studying only a portion of species and their ranges even though type material was consulted in the original paper by Manoko et al. (2007). The taxon referred to as *Solanum* sp. from Brazil in Manoko et al. (2007) also refers to *S. americanum* as treated here, but represents a morphological variation mainly observed within the New World and was hence difficult to interpret with limited sampling in previous studies. The re-examination of the study results by Manoko et al. (2007) in the light of the new identifications, highlights the fact that clear population structure can be observed within *S. americanum* as circumscribed here, where populations from Brazil show high genetic divergence from the rest of the World, including northern South American material.

The results described above, based on AFLP markers, should be tested with modern population genetic tools such as functional markers (Poczai et al. 2013) or genotyping-by-sequencing (GBS) and phylogenetic analysis. Species-level phylogenetic studies with multiple accessions of all species would also be useful in confirming the monophyly of the highly variable and widespread *S. americanum* in the context of other species of the Morelloid clade.

Edmonds (2012) incorrectly designated as the lectotype of S. nigrum var. patulum L. a specimen in the Dillenian herbarium at OXF. This typification is in conflict with the protologue (see McNeill et al. 2012, Art. 9.19) because the specimens themselves are not original material for this name. Linnaeus never saw Dillenius’s herbarium (Jarvis 2007), but based his name entirely on the plates from *Hortus Elthamensis* (Dillenius 1732). We have therefore re-lectotypified this name based on original material and designated the specimen chosen by Edmonds as the epitype.

The identity of the species depicted in plate 355 (Dillenius 1732) has been the subject of much speculation. Thellung (1927) studied material in Dillenius’ herbarium at OXF in an attempt to come to grips with the identity of *S. dillenii* (see below) and made careful annotations on specimens associated with plate 355. Material stored under plate 355 is mixed; some specimens are of the North American endemic *S. emulans* Raf. with 9–11 stone cells, calyx lobes appressed to the berry and matte fruit texture and others are of *S. americanum* with no or up to 4 stone cells, strongly reflexed calyx lobes in fruit and shiny mature berries. Thellung (1927) associated the name *S. dillenii* with the Dillenian specimens of the North American native *S. emulans*, but did not realise that there were two taxa involved in the material stored under plate 355. Polgár (1939) re-described *S. dillenii*, confining it to his original circumscription (Polgár 1926) and equating it with *S. nodiflorum* and coined a new epithet, *S. dillenianum* Polg. for the North American material in the Dillenian herbarium that had been called S. nigrum var. dillenii by Gray (1878). We have epitypified the name S. nigrum var. patulum to conform with current usage because, in our view, the plate is unambiguously of a plant of *S. americanum* with small flowers, black fruit and sepals that are strongly reflexed in fruit.

*Solanum papilionaceum* was almost certainly described from living material only. Dumont de Courset (1802: 135) cited no specimens and gave no other provenance than “Cette morelle, qui m’a été envoye en graines du Jardin. nat”. Searches in Paris have revealed no authentic original material, so we have selected a neotype that is a specimen dated after the description that was cultivated in Paris (P00582223). The specimen matches the description, which is of a plant with small flowers in umbelliform inflorescences and fruits like “cassis” (blackcurrants).

The specimens in Zuccagni’s herbarium in Florence were consumed by fire, making the designation of a neotype for *S. strictum* Zucc. necessary. The specimen we have selected is dated later than the description, but is labelled as “*strictum* Zucc.” and is from Italy in cultivation (G00144215); it was used by Dunal in his *Prodromus* treatment as *S. strictum* (Dunal 1852).

The identity of Schultes’s (1814) name *S. dillenii* has a complex history. Schultes (1814) coined a replacement name because the epithet “patulum” had been used at the species rank by Persoon (1805: 223) for the Peruvian taxon now known as *S. ruizii* S.Knapp (see Knapp 1989, 2013). He did not realise that Roth (1800) had already recombined Linnaeus’ S. nigrum var. patulum (see above) at the specific rank, basing his description entirely on *Hortus Elthamensis* plate 355 (Dillenius 1732). In his protologue, Schultes refers to a collection by Kitaibel from Hungary (“das ich vor mir habe” [which I have before me]) and the illustration in *Hortus Elthamensis* (Dillenius 1732) and describes a plant with small flowers and fruits borne on erect pedicels. Polgár (1926) examined the specimen in Kitaibel’s herbarium (“A IX Fasc. 102”) labelled “*Solanum nigrum an patulum, Esse patulum affirmat Willdenow. In silvis Matrae*” and equated the specimen with the American species he called *S. nodiflorum* Jacq. and suggested it was not native to Hungary, but rather from a botanic garden. This sheet (Herb. Kit. Fasc. IX, No. 102) in BP is a tangled mixture of two elements (see Poczai et al. 2009). One stem has small flowers in sub-umbellate inflorescences and matches the protologue description (but has no fruit) and the other stem has larger flowers in elongate racemose inflorescences and was identified by Poczai et al (2009) as *S. scabrum.* It is quite possible that there was considerable mix-up with the labelling of plants in Kitaibel’s herbarium; a specimen at BP (Herb. Kit. Fasc. IX, No. 101), exactly matching the small-flowered stem of Herb. Kitaibel fasc. IX, No. 102, is labelled “*Solanum nigrum* β *patulum*” in Kitaibel’s hand and “ex horto” in another hand. It is possible that the large-flowered plants collected in Mátra (which we identify as *S. nigrum*) were mixed with small-flowered plants from cultivation and, in distributing duplicates, confusion ensued. As Schultes (1814) cited a specimen (see McNeill et al. 2012, Art. 9.12), we have lectotypified *S. dillenii* with the only sheet in the Kitaibel herbarium (BP) that bears the locality cited in Schultes’s (1814) protologue, but limit our typification to the stem with small flowers only. A sheet in B-W [B-W 04364-03] of Kitaibel’s with a label in his hand “161/*Solanum nigrum* an *patulum* ?/In sylvis Hungaria” is certainly a duplicate; we cannot be sure this is the sheet Schultes had in his possession, since the locality is not exactly the same as that in the protologue and it does not have fruit. The BP sheet (fasc. IX No. 101) is also possibly a duplicate; it does have berries borne on erect pedicels. Gray (1878) used Schultes’s epithet at the infraspecific rank (as S. nigrum L. var. dillenii (Schult.) A.Gray) for plants from north-eastern North America now known as *S. emulans* Raf.

In describing *S. microspermum*, Dunal (1816) used an unpublished name by L’Heritier and cited a specimen and his own unpublished illustration, now held in MPU. We have selected the specimen in G-DC that comes from “herb. Thibaud” and is annotated by Dunal as the lectotype. The online catalogue at G indicates the collector of this sheet as “L’Héritier de Brutelle”.

Although the protologue of *S. erythrocarpon* (Meyer 1818) indicates the fruits are red (“Baccae pendulae, pisi minoris magnitudine, lutescenti-rubrae, nitidae”), the specimen in GOET (GOET003505) that represents original material for this name (here selected as the lectotype) matches *S. americanum* in all other respects.

D’Arcy (1974a: 735) cited as “type” a specimen in P as “Type: Herb. Rich. (P)” with a footnote stating that the sheet has two labels, one with “Isle de France” in Dunal’s hand and the other indicating it is from Herb. Richard. Since the protologue does not mention Isle de France and Dunal had nothing to do with the description of this name, this unintentional lectotypification is in conflict with the protologue, in which the locality is “insulae Cubae” and Richard is mentioned. We therefore supersede it and designate a specimen in P (P00370899) that matches the protologue in being from Cuba and originally from Herb. Richard as the lectotype for *S. indecorum*.

Sendtner (1846) described his var. aguaraquiya referring to Piso’s (1648: 55) pre-Linnaean name “Aguara-quiya” and citing un-numbered collections of Sellow and one Martius collection with a number and locality we have here selected as the lectotype. The Sellow collections associated with this name (BR [BR0000005538058], K, W [W0004136]) have large anthers and represent *S. chenopodioides*; they have neither numbers nor localities. The specimen selected as lectotype here (*Martius 1225*, M-0171809) contains detail of collection locality cited in the protologue and is hence the best material.

D’Arcy (1974a) lectotypified both *S. amarantoides* (*Gaudichaud 552*) and S. nodiflorum var. acuminatum (*Vauthier 537*) by stating “type” and a single herbarium, “P”. In the case of *S. amarantoides*, two specimens are found in P; we have selected that which has a label with Dunal’s handwriting in a second step lectotypification (P00319574). For S. nodiflorum var. acuminatum, however, D’Arcy (1974a) cited “P, ex Herb. Drake”, indicating a single specimen. Unfortunately, this specimen (P00319615) is not the duplicate of *Vauthier 537* with Dunal’s label (that with the annotation from Dunal is P00315614), but must be accepted as the lectotype nevertheless.

In describing *S. minutibaccatum*, Bitter (1912a) cited a single Buchtien collection (*Buchtien 1443*), but no herbarium. We have selected the sheet in US (US00027684) as the lectotype because it is the best preserved of the duplicates we have seen.

*Solanum calvum* was described using “*Palmer 60* p. pte.” (Bitter 1913b) with a single herbarium cited (“herb. Upsal.”). Edward Palmer began his number series again on every collecting trip (McVaugh 1956), but the collection number in question here refers to plants collected on Guadalupe Island (Baja California) in 1875. Other duplicates of *Palmer 60* (another sheet at UPS, MO [MO-158569]) are part of type material of *S. profundeincisum* Bitter, a synonym of *Solanum douglasii* Dunal from Mexico and the south-western United States, while others are of material of *S. nitidibaccatum* (BM001017193 and MO-158570). We exclude these as types of *S. calvum* and urge caution when interpreting other duplicates of *Palmer 60*.

The protologue of S. nigrum var. pauciflorum (Liou 1935) cited three specimens from Hainan Island; *Chen 1*, *Wang 2* and *Lau 209*, all perhaps from LU, although no herbarium was cited. We select here the more widely distributed *Lau 209* with the BM sheet (BM000942311) as lectotype for this name.

#### Selected specimens examined.

A total of 1,074 specimens were examined from 73 countries during the study across Africa, Asia, Australia, Eurasia and the Pacific. Specimens from the New World were also studied in order to understand the full range of morphology within the species. All specimens examined from the Old World can be seen in Appendix 2 (csv format) and Appendix 3 (traditional Specimens Examined list in pdf format).

### 3. Solanum chenopodioides

Taxon classificationPlantaeSolanalesSolanaceae

Lam., Tabl. Encycl. 2: 18. 1794

Figures 10 , 11

Solanum sublobatum Willd. ex Roem. & Schult., Syst. Veg., ed. 15 bis [Roemer & Schultes] 4: 664. 1819.
Type. Argentina. Buenos Aires, *Anon. s.n.* [probably *P. Commerson*] (*Herb. Willdenow 4336*) (lectotype, designated by Edmonds 1972, pg. 105 [as type ex photo]: B [B-W04336-01-0]).
Solanum besseri Weinm., Syst. Veg., ed. 15 bis [Roemer & Schultes] 4: 593. 1819.
Type. “In America” [cultivated in Europe?], *Anon. s.n.* (no specimens cited; no original material located; neotype, designated here: G-DC [G00144198]).
Solanum subspatulatum Sendtn., Fl. Bras. (Martius) 10: 45, tab. 4, fig. 16–18. 1846.
Type. Brazil. Sin. loc., *F. Sellow s.n.* (holotype: B, destroyed, F neg. 3183; lectotype, designated by D’Arcy 1974a, pg. 735 [as type]: P [P00384051]; isolectotype: F [fragment]).
Witheringia chenopodioides (Lam.) J.Rémy, Fl. Chil. [Gay] 5: 69. 1849.
Type. Based on Solanum chenopodioides Lam.
Solanum isabellei Dunal, Prodr. [A. P. de Candolle] 13(1): 153. 1852.
Type. Uruguay. Montevideo, Lat. aust. 34°45’08”, 1838, A. Isabelle s.n. (lectotype, designated here: G-DC (G00145645); isolectotypes: F [V0073298F, acc. # 680251; V0073299F, acc. # 680253)], K [K000585686], P [P00384071], W [1889-115034]).
Solanum chenopodifolium Dunal, Prodr. [A. P. de Candolle] 13(1): 44. 1852.
Type. Argentina/Uruguay. “Buenos Aires et Montevideo”, *P. Commerson s.n.* (lectotype, designated Edmonds 1972, pg. 108 [as holotype], second step designated here: P [P00384081]).
Solanum crenatodentatum Dunal var. ramosissimum Dunal, Prodr. [A. P. de Candolle] 13(1): 54. 1852.
Type. United States of America. Louisiana: “Basse Louisiane”, 1839, *G.D. Barbe 82* (holotype: P [P00362535]).
Solanum gracile Dunal, Prodr. [A.P. de Candolle] 13(1): 54. 1852, nom. illeg., not Solanum gracile Sendtn. (1846).
Type. Brazil. Rio de Janeiro: “Rio de Janeiro”, 1831-1833, *C. Gaudichaud 520* (lectotype, designated by Henderson 1974, pg. 46: G-DC [G00144391]; isolectotypes: G [G00343457], P [P00384052, P00384053]).
Solanum gracile Dunal var. microphyllum Dunal, Prodr. [A. P. de Candolle] 13(1): 54. 1852.
Type. Argentina/Uruguay. “ Circa Buenos Ayres et Montevideo”, *P. Commerson s.n.* (lectotype, designated by Morton 1976, pg. 151: P [P00384061, Morton neg. 8207]; possible isolectotype: F [V0073283F, acc. # 976485, fragment only]).
Solanum nodiflorum Jacq. var. microphyllum Hassl., Repert. Spec. Nov. Regni Veg. 9: 118. 1911.
Type. Paraguay. Estrella: Mar, *É. Hassler 10271* (holotype: G?, Morton photo 8612).
Solanum vile Bitter, Repert. Spec. Nov. Regni Veg. 11: 221. 1912.
Type. Brazil. Rio de Janeiro: Restinga do Harpoador, *E. Ule 4310* (lectotype, designated here: CORD [CORD00004277]; isolectotype: HBG [HBG511507]).
Solanum gracilius Herter, Rev. Sudamer. Bot. 7: 266. 1943.
Type. Based on (replacement name for) S. gracile Dunal
Solanum ottonis Hyl., Uppsala Univ. Årsskr. 7: 279. 1945.
Type. Based on (replacement name for) Solanum gracile Dunal
Solanum americanum Mill. var. baylisii D’Arcy, Ann. Missouri Bot. Gard. 61: 837. 1974.
Type. New Zealand. Sin. loc., cultivated, 1953, *Momson s.n.* (holotype: OTA [OTA-00419]).

#### Type.

Mauritius. “Ex ins. Mauritiana”, *Herb. Lamarck s.n.* (lectotype, designated by Barboza et al. 2013, pg. 242: P [P00357629]).

#### Description.

Annual herbs to short-lived, erect to somewhat sprawling perennial herbs to 1.0 m tall, subwoody and branching at base. Stems spreading to decumbent, terete, green-grey to straw colour, older stems with no prickle-like projections, not markedly hollow; new growth pubescent with simple, appressed, uniseriate, eglandular trichomes, these 1-6-celled, 0.1–0.6 mm long; older stems more sparsely pubescent, glabrescent. Sympodial units difoliate, the leaves not geminate. Leaves simple, 1.5–5.5(-7.0) cm long, 0.5–3.0(-3.5) cm wide, lanceolate to narrowly ovate, rarely ovate, discolorous; adaxial surface green, sparsely pubescent with appressed 1-4-celled translucent, simple, uniseriate trichomes like those on stem, these denser along the veins; abaxial surface pale grey, more densely pubescent with trichomes like those of the upper surface evenly across lamina and veins; major veins 3–6 pairs, not clearly evident abaxially; base attenuate, decurrent on the petiole; margins entire or sinuate; apex acute to obtuse; petioles (0.5-)1.0–1.5(-3.5) cm long, sparsely pubescent with simple uniseriate trichomes like those of the stems and leaves. Inflorescences 1.0–2.5(-4.0) cm long, generally internodal but appearing leaf-opposed on young shoots, simple or rarely furcate, sub-umbelliform, with 3–7(-10) flowers, sparsely pubescent with appressed 1-2-celled simple uniseriate trichomes; peduncle 1.0–2.3(-4.0) cm long, straight but becoming strongly deflexed downwards in fruit; pedicels 0.5–1 cm long, ca. 0.5 mm in diameter at the base and 1 mm at apex, straight and spreading, articulated at the base; pedicel scars spaced ca. 0–1 mm apart. Buds elongate-oblong, the corolla only slightly exserted from the calyx tube before anthesis. Flowers 5-merous, all perfect. Calyx tube 0.5–1.0 mm long, conical, the lobes 0.6–1.2 mm long, less than 1 mm wide, broadly deltate to triangular with acute to obtuse apices, sparsely pubescent with 1-4-celled appressed hairs like those on stem but shorter. Corolla 6–12 mm in diameter, white with a black or yellow-green central portion near the base, the black colour usually distal to the yellow-green, deeply stellate, lobed 4/5 of the way to the base, the lobes 3.5–4.0 mm long, 1.5–1.9 mm wide, strongly reflexed at anthesis, later spreading, densely puberulent-papillate abaxially with 1-4-celled simple uniseriate trichomes, these denser on the tips and margins. Stamens equal; filament tube minute; free portion of the filaments 0.6–1.0 mm long, adaxially pubescent with tangled uniseriate 4-6-celled simple trichomes; anthers (2.0-)2.3–2.8 mm long, 0.5–0.8 mm wide, narrowly ellipsoid, yellow, poricidal at the tips, the pores lengthening to slits with age and drying, the connective becoming darker brown with age in dry plants. Ovary globose, glabrous; style 3.7–4.5 mm long, densely pubescent with 2-3-celled simple uniseriate trichomes in the lower half where included in the anther cone, exserted up to 1.5 mm beyond the anther cone; stigma capitate, minutely papillate, green in live plants. Fruit a globose berry, 4–9 mm in diameter, dull purplish-black at maturity, the pericarp thin and opaque, matte and somewhat glaucous; fruiting pedicels 6–13 mm long, 1.2–1.4 mm in diameter at the base and the apex, reflexed and slightly curving, dropping with mature fruits, not persistent; fruiting calyx not accrescent, the tube less than 1 mm long, the lobes 1–1.5 mm long, appressed against the berry. Seeds (13-)20–35(-50) per berry, 1.2–1.4 mm long, 1.0–1.2 mm wide, flattened and tear-drop shaped with a subapical hilum, pale yellow, the surfaces minutely pitted, the testal cells pentagonal in outline. Stone cells absent or occasionally 1–2 diminutive apical stone cells present. Chromosome number: *2n*=2x=24 (Baylis 1958; Soria and Heiser 1961 [as *S. gracile*]; Heiser et al. 1965 [as *S. gracile*]; Venkateswarlu and Rao 1972; Henderson 1974 [as *S. gracilius*]; Randell and Symon 1976; Edmonds 1972, 1977, 1982, 1983; 1984a [as *S. sublobatum*]; Moscone 1992; Jacoby and Labuschagne 2006; Moyetta et al. 2013).

**Figure 10.**
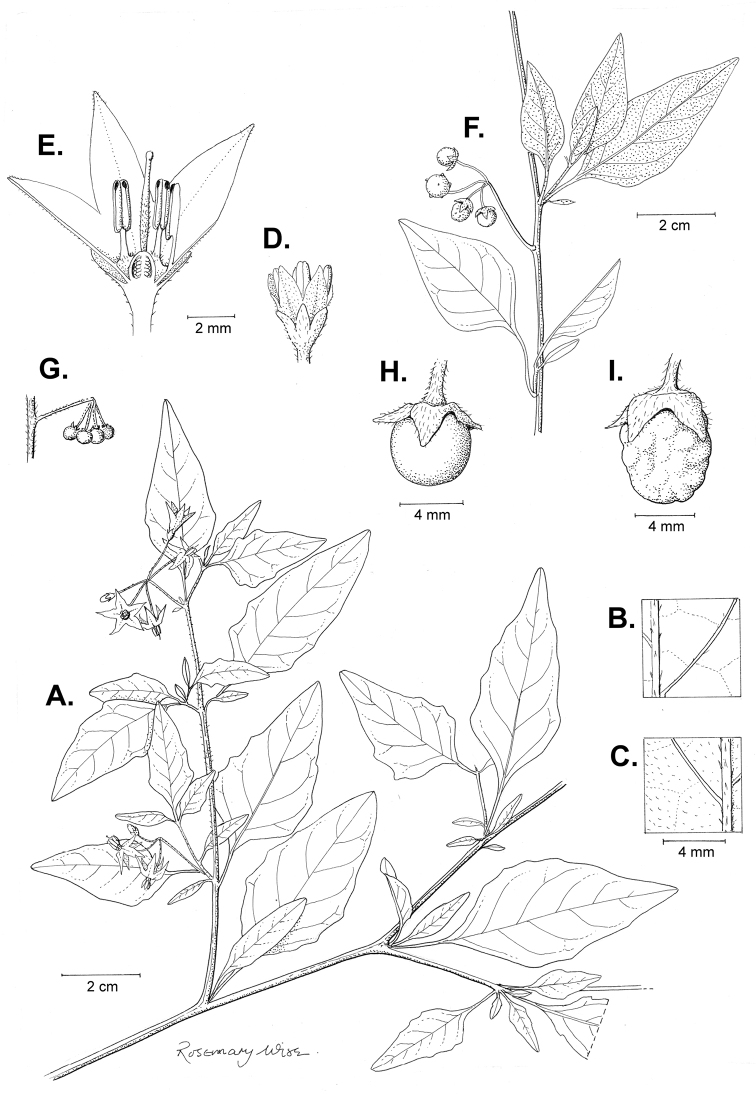
*Solanum chenopodioides* Lam. **A** Habit **B** Detail of adaxial leaf surface **C** Detail of abaxial leaf surface **D** Opening bud **E** Dissected flower **F** Fruiting branch **G** Detail of infructescence **H** Maturing fruit **I** Fully mature fruit (**A–E** *Fox s.n.*; **F–I** *Hieronymus s.n.*). Drawing by R. Wise.

**Figure 11.**
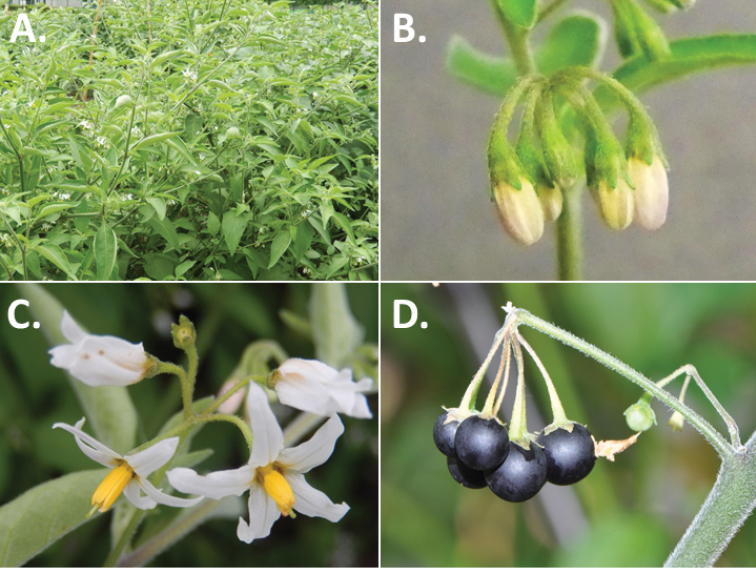
*Solanum chenopodioides* Lam **A** Habit **B** Buds **C** Flowers at full anthesis **D** Fully mature matte black fruits with appressed calyx lobes (**A–D** Nijmegen acc. A14750051). Photos by S. Knapp and G. van der Weerden.

#### Distribution

**Figure 12.**
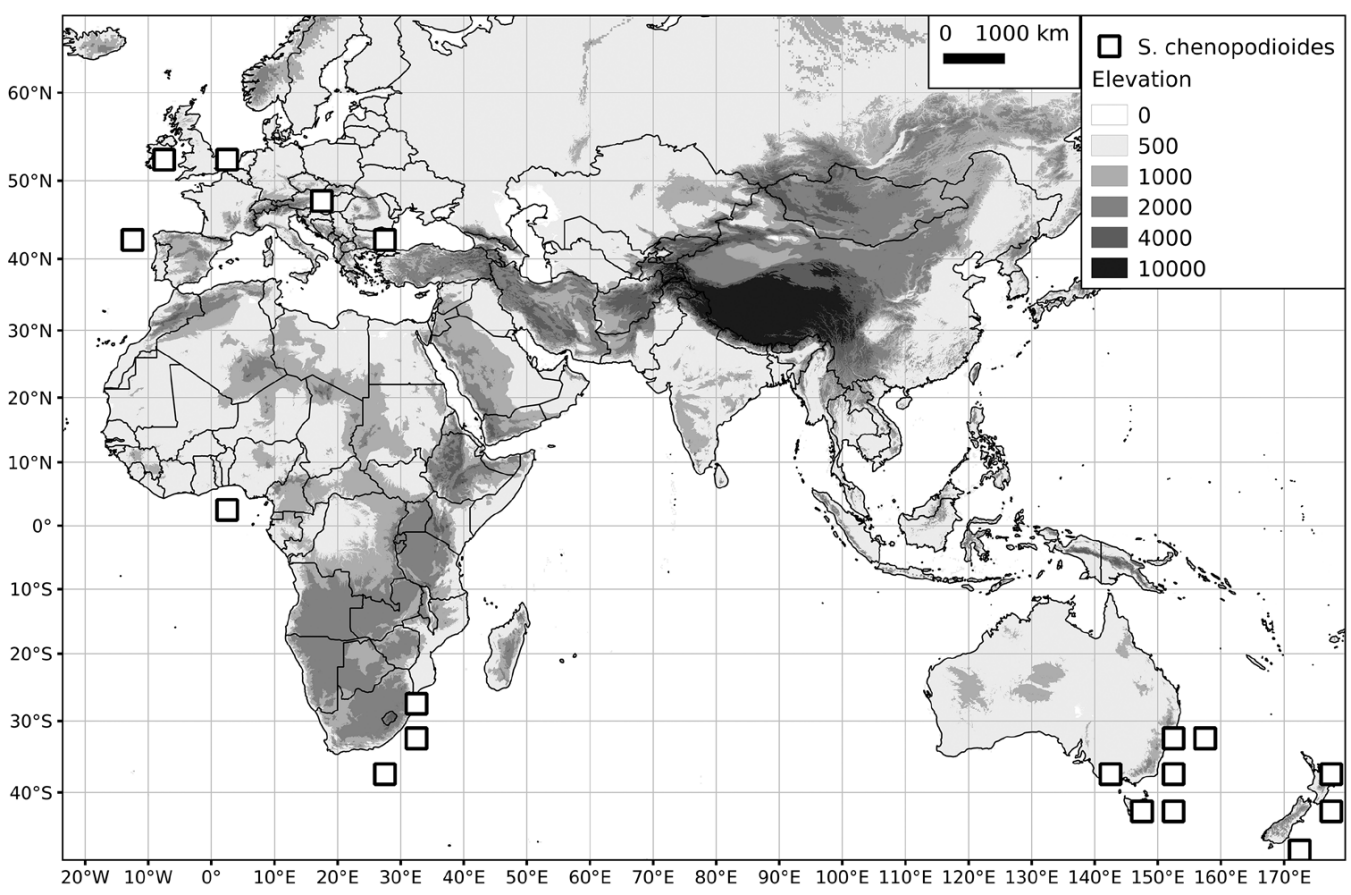
Distribution of *Solanum chenopodioides* Lam in its non-native range in the Old World.

(Figure 12). Thought to be native to southern South America (see Barboza et al. 2013), but introduced globally in temperate and subtropical areas.

#### Ecology.

Grows in humid, disturbed areas between rocks, along water sources and roads and in cultivated lands, common in areas with human disturbance; between sea level and 1,900 (-2,500) m elevation.

#### Common names.

Australia: whitetip nightshade (Symon 1981; Edmonds and Chweya 1997); New Zealand: velvety nightshade (Webb et al. 1988; Edmonds and Chweya 1997); South Africa: nastergal; United Kingdom: tall nightshade (Stace 2010).

#### Uses.

South Africa. leaves used as spinach (Edmonds and Chweya 1997); berries locally used for making jam (Viljoen 2011).

#### Preliminary conservation status

(IUCN 2016). *Solanum chenopodioides* is an extremely widespread cosmopolitan weed and can be assessed as LC (Least Concern; Table 7). When only the putatively native South American distribution is considered, *S. chenopodioides* still has a very large EOO of 2,279,138 km^2^ and remains LC.

#### Discussion.

*Solanum chenopodioides* can be distinguished from most of the other morelloids occurring in the Old World based on its narrowly lanceolate leaves with grey indumentum, inflorescences with ca. 3–7 flowers tightly congested near the tip of the peduncle, the stellate corollas that are deeply lobed to the base and usually with a dark purple or black central star and anthers that are usually more than 2 mm and up to 2.8 mm long. In fruit, the pedicels and proximal portion of the peduncle are strongly reflexed and the berries are not at all shiny. *Solanum retroflexum* has similar matte black berries, but has rhomboid leaves, less deeply divided corollas, shorter anthers and the calyx lobes are strongly reflexed in fruit. *Solanum chenopodioides* could also potentially be confused with the more common *S. nigrum*, especially in Europe, but differs from that species in its terete stems, matte black fruits on short strongly reflexed pedicels and its smaller seeds (1.2 mm long versus 2 mm long).

*Solanum chenopodioides* has a scattered distribution in the Old World, but it seems to be spreading, perhaps related to climate change and/or increased habitat alteration (Martínez Labarga et al. 2017).

This diploid species possibly contributed its genome to the tetraploid *S. retroflexum* and hexaploid *S. opacum*. Jacoby and Labuschagne (2006) reported that crosses made between *S. chenopodioides* and *S. retroflexum* were much more successful than between *S. americanum* and *S. retroflexum*. This relationship was also confirmed by arbitrary-amplified dominant markers (Jacoby et al. 2003; Poczai and Hyvönen 2011) and by whole genome DArT analysis by van der Walt et al. (2008).

Morton (1976) thought the locality on the type specimen of *S. chenopodioides* Lam. as “Mauritius” was in error, because the species was a New World taxon in his concept. Morton suggested that the Commerson material on which the name was based was actually from Argentina, but that the localities had been mixed up. This may be true, because there is much material collected by Commerson from Argentina and Uruguay, but *S. chenopodioides* also occurs on Mauritius as an introduced weed and we are hence reluctant to suggest the locality is an error.

In the protologue (Roemer and Schultes 1819: 593) of *S. besseri*, both the name and description are attributed to Johann Anton Weinmann (”Weinm. in litt.”) but no specimens or collections are cited. The description is of a plant with subumbellate inflorescences, black fruits and that comes from America, meaning that, in the absence of a type specimen, it is difficult to determine the identity of this species. Later Weinmann (1824) published a list of plants from St. Petersburg in which he lists ”*S. besserianum*” (a nomen nudum with no description) that is probably the same plant, stating it is from “America” and equating it with “*S. cestrifolium* Jacq.?” (see Doubtful names). Dunal (1852) made a detailed description of *S. besseri* and put *S. americanum* in synonymy with it. His description was based on a specimen he saw in “herb. DC” and living plants. In the absence of any original material for this name, we neotypify it here with the specimen in G-DC (G00144198) used by Dunal (1852) and labelled “*Solanum besseri*” in his hand. The specimen matches his detailed description exactly and it is not in conflict with the original description (Roemer and Schultes 1819). We do this in order to stabilise the identity of this name so it does not further disrupt names in this group (e.g. see discussions of *S. memphiticum* and *S. villosum*).

In the protologue of *S. isabellei*, Dunal (1852) cited specimens in G-DC (*Isabelle s.n.*) and P (*Gay s.n.*, 1828); we have selected the collection *Isabelle s.n.* (G00145645) as the lectotype because it is well represented by duplicates in other herbaria. He cited various sheets of plants collected by Philibert Commerson in Uruguay and Argentina as material for his new names *S. chenopodiifolium*, *S. gracile* and S. gracile var. microphyllum. There are many Commerson collections corresponding to *S. chenopodioides* in P, none of which we are treating as strict duplicates; although they have similar morphologies, they all have slightly different collecting localities and labels. Conrad V. Morton (1976) lectotypified S. gracile var. microphyllum by citing his photograph (Morton neg. 8207) of specimens in P and we have matched this to the individual sheet and add the barcode here as a second step lectotypification. We have selected another of the Commerson sheets in P that is annotated by Dunal as “*S. chenopodiifolium*” as the lectotype of that name.

*Solanum gracile* was incorrectly typified by D’Arcy (1974a) on “Hort. Monsp. 1831 (MPU)”, but Dunal did not cite herbarium material from Montpellier, he only cited living material from there (“v.v.”) along with herbarium material from G-DC and P (“v.s. in h. DC h. Mus Paris. et v.v.”). Any herbarium material prepared from such living specimens would be a neotype, but that is inappropriate while syntypes still exist. Henderson (1974) cited as lectotype for *S. gracile* a sheet in G-DC without citing a collector, but he did cite a microfiche number (IDC 800-61.2063:III.7) that corresponds to *Gaudichaud 520*, the material cited in the protologue. His lectotypifcation is effective, because he cited a single collection in a single herbarium (G-DC, G003144391); duplicates in G and P are isolectotypes. Morton (1976) later superfluously lectotypified *S. gracile* with Commerson material at P, citing his photograph (Morton neg. 8206); this specimen (P00384083) is not a type, although it was cited as the lectotype by Barboza et al. (2013).

#### Selected specimens examined.

**Australia**. **New South Wales**: N shore Lake Illawarra, Lake Hts, 5 Sep 2003, *Andrew s.n.* (WOLL); Nepean River, Douglas Park, 6 mi E of Picton, 11 Oct 1965, *Constable 6161* (AD, K, NSW); Bega Valley, Twofold Bay, Nullica Bay Beach 5 km SSW of Eden, 8 Feb 1979, *Haegi 1711* (AD, MEL, MO, NSW); Swamp 2 km N of Bodalla on Princes H[igh]w[a]y, just S of Tuross River crossing, 10 Feb 1979, *Haegi 1756* (AD, NSW); Bega Valley, Tathra, 20 Mar 1995, *Heyligers 95004* (PERTH); Bega Valley, Tathra, 20 Mar 1995, *Heyligers 95006* (PERTH); Tamworth Regional, Hanging Rock, 13 Apr 2008, *Hosking 3091* (CANB, MEL, NE, NSW); Murrumbidgee River at Cotter Bridge, Cotter Reserve, 1 Mar 1992, *Lepschi 740* (AD, CANB, NSW); Hillview (3 mi SW of Liverpool), 11 Dec 1968, *Rodd s.n.* (NSW); **Queensland**: Brisbane, 20 Jan 1966, *Henderson 128* (BRI, NSW); Brisbane, Clapham Junction, 20 Oct 1967, *Henderson 301* (AD, BRI); **South Australia**: Tea Tree Gully, Gorge Rd, 15 Jan 2010, *Brodie 1171* (AD); sin. loc, 1885, *Lea s.n.* (BM); Region 11, Southern Lofty, Lower Gorge Rd, 13 Feb 1997, *Symon 15462* (AD, K, MEL); **Victoria**: East Gippsland, N of Mallacoota township, 4 Apr 1999, *Clarke 2890* (AD, MEL); Melbourne, 70 m SW of Queens Bridge, 18 Feb 1983, *Clarke 1544* (AD, CANB, MEL); Wellington, c. 13 km from Licola, 28 Jan 1989, *Thompson 159* (AD).

**France**. **Nouvelle Aquitaine**: Gironde, Bordeaux, 28 Aug 1931, *Bouchon 6703* (BM); **Provence-Alpes-Côte d’Azur**: Bouches-du-Rhône, La Valentine, banlieue E de Marseille, route vers Saint Menet, 19 Nov 1973, *Martin 6848* (BM, H).

**Germany**. **Baden-Württemberg**: Karlsruhe, Carlsruhe, Oct 1834, *Braun s.n.* (K); Karlsruhe [?], Dec 1834, *Braun s.n.* (K).

**Greece**. **Crete**: sin. loc, *de Tournefort s.n.* (BM); **East Macedonia and Thrace**: Island of Thasos, SE of Potamia, 23 Nov 2016, *Biel IM-16054* (N/A).

**Italy**. **Friuli Venezia Giulia**: Monfalcone, Friulia, 25 Sep 1953, *Neumann s.n.* (W).

**Japan**. **Honshu**: Tochigi, Utsunomiya Agricultural College, Japan, 1935, *Kagawa s.n.* (K)Kyoto, Koyama, Chitose, Chitose-cho, Kameoka-shi, 12 Sep 2010, *Tsugaru et al. 7604* (MO).

**Lesotho**. Maseru airfield escarpment, Lesotho, 10 Dec 1969, *Without collector 348* (K); Maseru Ekp Stn, 3 Mar 1970, *Without collector 650* (K).

**New Zealand**. **North Island**: Auckland, Tawharanui Regional Park, 14 Jan 2012, *Salter & Duff s.n.* (AK); Northland, Te Arai Sanctuary, 27 Jan 1995, *Wright 12532* (AK); **South Island**: Tasman, Collingwood, 1 km W on Cape Farewell rd, 13 Mar 2006, *Brummitt 21567* (K).

**Portugal**. **Azores**: Faial, Horta, 27 Sep 1970, *Brooke 11376* (BM); Faial, Capelo, 1 Dec 1971, *Goncalves 374* (BM); Faial between Pedro Miguel and Espalhafatos, 15 Sep 2001, *Henderson et al. 98* (AZU, BM); **Centro**: Vila Nova de Barquinha, Ribatejo, 16 Jul 1963, *Rainha 6177* (W); **Madeira**: Between Monte and Funchal, 11 Sep 1984, *Davis 70402* (BM); **Norte**: Amarante, Marcos de Canavezes, Douro Litoral, 6 Jul 1960, *Pinto da Silva et al. 6754* (W).

**South Africa. Eastern Cape**: Old Town Quarry, Grahamstown, 20 Nov 1972, *Bayliss 5292* (A, BH, MO); Belmont Valley, Albany Dist, 1 Sep 1974, *Bayliss 6785* (K, MO); Humansdorp, Distr. Humansdorp, Tzitzikama Park, 2 Feb 1966, *Liebenberg 7909* (K); **Gauteng**: Germiston District, Dowerglen, 22 Oct 1992, *Balkwill 7163* (MO); Pretoria, Johannesburgh, Taamlik volop, 2627BB grid ref, Oct 1976, *Liebenberg 8568* (K, MO); **KwaZulu-Natal**: Pretoria, Greytown, Natal, 11 Feb 1939, *Galpin 14832* (K); Underberg, 2929CB Sani Pass, Undeberg Distr., 17 Feb 1982, *Hilliard* & *Burtt 15537* (K); Nottingham rd, Natal, Mar 1939, *McClean 869* (K, MO); **Mpumalanga**: Middelburgh District, just outside Middelburgh, 26 Jan 1995, *Balkwill 9130* (MO); **Western Cape**: 3323 Willowmore DC, 28 Dec 1982, *Goldblatt 6780* (MO).

**Spain**. **Cantabria**: Santander, rd to Pechon, 31 Jul 1972, *Brenan 12271* (BM, K).

**Sweden**. **Götaland**: Västra Götaland, Göteborg, Backa prope Brunnsbo, Aug 1938, *Blom s.n.* (BM); Västra Götaland, Agnesbergs kvarn, 30 Sep 1938, *Blom s.n.* (K, W).

**Switzerland**. **Ticino**: Locarno, bank of Maggia river, 17 Sep 2000, *Brummit 20476* (K).

**United Kingdom. Channel Isles**: Guernsey, nr entrance to Mont Cuet, Nov 2001, *Dupree s.n.* (BM); Guernsey, 6 Sep 1994, *McClintock* & *Ryan s.n.* (BM); Guernsey, St. Sampsons, 23 Jul 1968, *Simpson 68010* (BM); **England**: Greater London, Victoria Park, 4 Sep 2008, *Atchison 2* (BM); Worcestershire, Charlton, 25 Aug 1959, *Pannister 983* (BM); Cornwall, Bude, Aug 1925, *Thurston s.n.* (K); **Wales**: Vale of Glamorgan, Barry Docks, 12 Sep 1935, *Brenan* & *Sandwith 1449* (BM); Cardiff, Barry Docks, 12 Sep 1935, *Sandwith* & *Brenan s.n.* (K).

### 4. Solanum furcatum

Taxon classificationPlantaeSolanalesSolanaceae

Dunal, Encycl. [J. Lamarck & al.] Suppl. 3: 750. 1814

Figures 13 , 14

Solanum deltoideum Colla, Herb. Pedem. 4: 273. 1835.
Type. Cultivated in Italy at “h. Ripul:” [Hortus Ripulensis], the seeds originally sent by C. Bertero from Chile [“Chili Quillota”] (no specimens cited; lectotype, designated here: TO [herb. Colla]).
Solanum furcatum Dunal var. glabrum G.Don, Gen. Hist. 4: 412. 1837.
Type. “In Peruvia” (no specimens cited; no original material located).
Solanum furcatum Dunal var. pilosum G.Don, Gen. Hist. 4: 412. 1837.
Type. “In Peruvia” (no specimens cited; no original material located).
Solanum furcatum Dunal var. acutidentatum Nees, Nov. Act. Acad. Caes. Leop. 19, Suppl. 1: 386. 1843, as “*acutedentatum*”.
Type. “Chile ad Valparaiso, Februario; Peruvia in planitie circa Tacoram, alt. 14000-17000’, Aprili” both syntypes collected by *F.J.F. Meyen s.n.* (no specimens cited; no original material located).
Solanum furcatum Dunal var. obtusidentatum Nees, Nov. Act. Acad. Caes. Leop. 19, Suppl. 1: 386. 1843, as “*obtusedentatum*”.
Type. “Chile. Prov. de San Fernando in Llano del Rio Tinguiririca, 3000’ alf, martio”; Peruvia ad Arequipam, Aprili” both syntypes collected by *F.J.F. Meyen s.n.* (no specimens cited; no original material located).
Solanum furcatum Dunal var. subintegerrimum Nees, Nov. Act. Acad. Caes. Leop. 19, Suppl. 1: 386. 1843.
Type. “Chile: Copiapó, Aprili; Peruvia: circa Tacoram, Aprili” both syntypes collected by *F.J.F. Meyen s.n.* (no specimens cited; no original material located).
Witheringia furcata (Dunal) J.Rémy, Fl. Chil. [Gay] 5: 67. 1849.
Type. Based on Solanum furcatum Dunal
Solanum pterocaulum Dunal var. dichotimiflorum Dunal, Prodr. [A. P. de Candolle] 13(1): 52. 1852, as ‘pterocaulon’.
Type. Cultivated in France at Montpellier “Solanum speciosum hort. botan” (no specimens cited, described from living plants “v.v. hort. Monsp.”; neotype, designated here: MPU [MPU310703]).
Solanum crenatodentatum Dunal, Prodr. [A. P. de Candolle] 13(1): 54. 1852.
Type. Chile. Région VI (O’Higgins): Colchagua, San Fernando, “in selibus chilensibus San Fernando”, Mar 1831, *C. Gay 2* (lectotype, designated by D’Arcy 1974a, pg. 738: P [P00337274]).
Solanum rancaguense Dunal, Prodr. [A. P. de Candolle] 13(1): 150. 1852.
Type. Chile. Région VI (O’Higgins): Rancagua, May-Oct 1828, *C. Bertero 633* (lectotype, designated by Edmonds 1972, pg. 107 [as holotype], second step designated here: P [P00384088]; isolectotypes: BM [BM000617677], G [G00144259], M [M-0171928], MO [MO-503700], NY [00743695], P [P00384089], P [P00384090], P [P00384091], P [P00384092], P [P00482266], W [1889-0283789]).
Solanum bridgesii Phil., Linnaea 33: 203. 1864.
Type. Chile. Región V (Valparaíso): Panquegue, *R.A. Philippi s.n.* (lectotype, designated here: SGO [SGO000004549]).
Solanum coxii Phil., Linnaea 33: 200. 1864.
Type. Chile. Región X (Los Lagos): Todos los Santos, 1862, *G. Cox 38* (lectotype, designated here: SGO [SGO000004555]; isolectotype: W [W1903-0010246]).
Solanum rancaguinum Phil., Anales Univ. Chile 43: 523. 1873.
Type. Chile. Región VI (O’Higgins): Rancagua, Mar 1828, *C. Bertero s.n.* (lectotype, designated here: SGO [SGO000004594]).
Solanum caudiculatum Phil., Anales Univ. Chile 91: 12. 1895.
Type. Chile. Región VIII (Bío-Bío): prov. Ñuble, Coigüeco, *F. Puga s.n.* (no original material located, not at SGO).
Solanum subandinum Phil., Anales Univ. Chile 91: 13. 1895, nom. illeg., not Solanum subandinum F.Meigen (1893).
Type. Chile. Región XIII (Metropolitana): Santiago, Las Condes, *R.A. Philippi s.n.* (lectotype, designated here: SGO [SGO000004600, F neg. 2745]).
Solanum ocellatum Phil., Anales Univ. Chile 91: 14. 1895.
Type. Chile. Región XIII (Metropolitana): Prope Colina, *F. Philippi s.n.* (lectotype, designated here: SGO [SGO000004582]; isotypes: SGO [SGO000004581], W [1903-0010230]).
Solanum nigrum L. var. crentatodentatum (Dunal) O.E.Schulz, Symb. Antill. (Urban) 6: 160. 1909.
Type. Based on Solanum crenatodentatum Dunal
Solanum bridgesii Phil. var. ocellatum (Phil.) Witasek ex Reiche, Anales Univ. Chile 124: 460. 1909.
Type. Based on Solanum ocellatum Phil.
Solanum andinum Reiche, Fl. Chile 5: 346. 1910.
Type. Based on (replacement name for) Solanum subandinum Phil.
Solanum tredecimgranum Bitter, Repert. Spec. Nov. Regni Veg. 11: 6. 1912.
Type. Chile. Región V (Valparaíso): Valparaíso, 17 Aug 1895, *O. Buchtien s.n.* (lectotype, designated by Barboza et al. 2013, pg. 246: US [00432692, acc. # 1399293]; isolectotypes: HBG [HBG511497], US [00681745, acc. # 1399294]).
Solanum robinsonianum Bitter, Repert. Spec. Nov. Regni Veg. 11: 7. 1912.
Type. Chile. Región V (Valparaíso): Juan Fernández Island, *R.A. Philippi 742* (holotype: B, destroyed, F neg. 2743; lectotype, designated here: W [0001347]).
Solanum masafueranum Bitter & Skottsb., Nat. Hist. Juan Fernandez & Easter Island 2: 167, pl. 14. 1922.
Type. Chile. Región V (Valparaíso): Juan Fernández Islands, Masafuera [Isla Alejandro Selkirk], Las Chozas, 715 m, 3 Mar 1917 [20 Feb 1917 on label], *C. Skottsberg & I. Skottsberg 363* (lectotype, designated here: S [acc. # 04-2947]; isolectotypes: BM [BM000617676], LD [1643307], K [K000585692], NY [00172084], GOET [GOET003548], GB [GB0048742], P [P00337092], UPS [acc. # 104031]).
Solanum spretum C.V.Morton & L.B.Sm., Revis. Argentine Sp. Solanum 132. 1976.
Type. Argentina. Río Negro: Bariloche, 19 Mar 1939, *A.L. Cabrera 5024* (holotype: GH [GH00077764]; isotypes F [V0073411F, acc. # 1007493], LP [LP006791]).

#### Type.

Peru ?. “Cette plante croît au Perou”, *J. Dombey [343*] (lectotype, first step designated by Edmonds 1972, pg. 107 [as holotype], second step designated by Barboza et al. 2013, pg. 246: P [P00335357]; isolectotypes: CORD [CORD00006928], F [V0043232F, acc. # 976846], G [G00359946], G-DC [G00144483], P [P00335358]).

#### Description.

Annual or perennial erect to sprawling herbs or small shrubs to 1.0 m tall, subwoody and branching at base. Stems spreading to decumbent, terete or ridged, green to purple tinged, older stems pale yellowish-brown, not markedly hollow; new growth sparsely pubescent with simple, spreading, uniseriate, eglandular trichomes, these 1-5-celled, 0.1–0.5 mm long; older stems sparsely pubescent to glabrescent. Sympodial units difoliate, the leaves not geminate. Leaves simple, (1.5-)4.0–8.0(-12.0) cm long, (0.6-)2.2–4.6(-6.5) cm wide, ovate to rhomboidal, membranous, slightly paler beneath, without smell; adaxial surface sparsely pubescent with simple, uniseriate trichomes like those on stem, these evenly spread along lamina and veins; abaxial surface more densely pubescent; major veins 4–6 pairs; base cuneate to acute, slightly oblique, decurrent on the petiole; margins sinuate-dentate or entire; apex acute; petioles 1.0–3.5 cm long, sparsely pubescent with simple uniseriate trichomes like those on stem. Inflorescences (1.0-)1.5–3.0(-4.0) cm long, internodal, furcate or more rarely unbranched, sub-umbelliform or the flowers evenly spaced along the rhachis, with 6–14 flowers, sparsely pubescent with simple uniseriate trichomes like those on stems; peduncle (1.0-)1.5–2.0 cm long, straight; pedicels 4.0–7.5 mm long, 0.2–0.3 mm in diameter at the base and 0.3–0.4 mm at apex, straight and spreading, articulated at the base; pedicel scars spaced ca. 0.2–2.5 mm apart. Buds subglobose, the corolla exserted 1/3-1/2 from the calyx tube before anthesis. Flowers 5-merous, all perfect. Calyx tube 0.5–1.5 mm long, conical, the lobes 0.8–1.5 mm long, 0.6–1.0 mm wide, rectangular to narrowly obovate, tips obtuse to shortly acute, pubescent with simple uniseriate trichomes like those on stem but shorter. Corolla 12–20 mm in diameter, white to lilac with a green or yellow-green central portion near the base, this sometimes purplish near the lobe midvein, stellate, lobed 1/3–1/2 of the way to the base, the lobes 5.5–7.0 mm long, 2.8–5.5 mm wide, strongly reflexed at anthesis, later spreading, densely pubescent abaxially with 1-4-celled simple uniseriate trichomes, especially along the margins and apex, these shorter than the trichomes of the stems and leaves. Stamens equal; filament tube minute; free portion of the filaments 0.9–1.6(-2) mm long, adaxially pubescent with tangled uniseriate 4-6-celled simple trichomes; anthers 2.3–3.3(-3.6) mm long, 0.8–1.0 mm wide, ellipsoid, yellow, poricidal at the tips, the pores lengthening to slits with age. Ovary globose, glabrous; style 6.0–6.5 mm long, densely pubescent with 2-3-celled simple uniseriate trichomes in the lower 1/2–2/3 where included in the anther cone, exserted 2–3 mm beyond the anther cone and somewhat curved; stigma capitate, minutely papillate, yellow or green in live plants. Fruit a globose berry, 6–9 mm in diameter, dull green to purple at maturity, the pericarp thin and opaque; fruiting pedicels 7–12 mm long, 0.2–0.4 mm in diameter at the base and 0.5–1.0 mm at apex, strongly reflexed, dropping with mature fruits, not persistent; fruiting calyx not accrescent, the tube ca. 1 mm long, the lobes 1.5–2.5 mm long, appressed against the berry. Seeds 30–40 per berry, 1.8–2.0 mm long, 1.4–1.5 mm wide, flattened and tear-drop shaped with a subapical hilum, yellow-brown, the surface minutely pitted, the testal cells pentagonal in outline. Stone cells 2–13 per berry, largest 0.8–1.0 mm in diameter but varying in size. Chromosome number: *2n*=6x=72 (Stebbins and Paddock 1949; Edmonds 1982, 1983), but see *2n*=2x=24 in Randell and Symon (1976; possible voucher identification issue).

**Figure 13.**
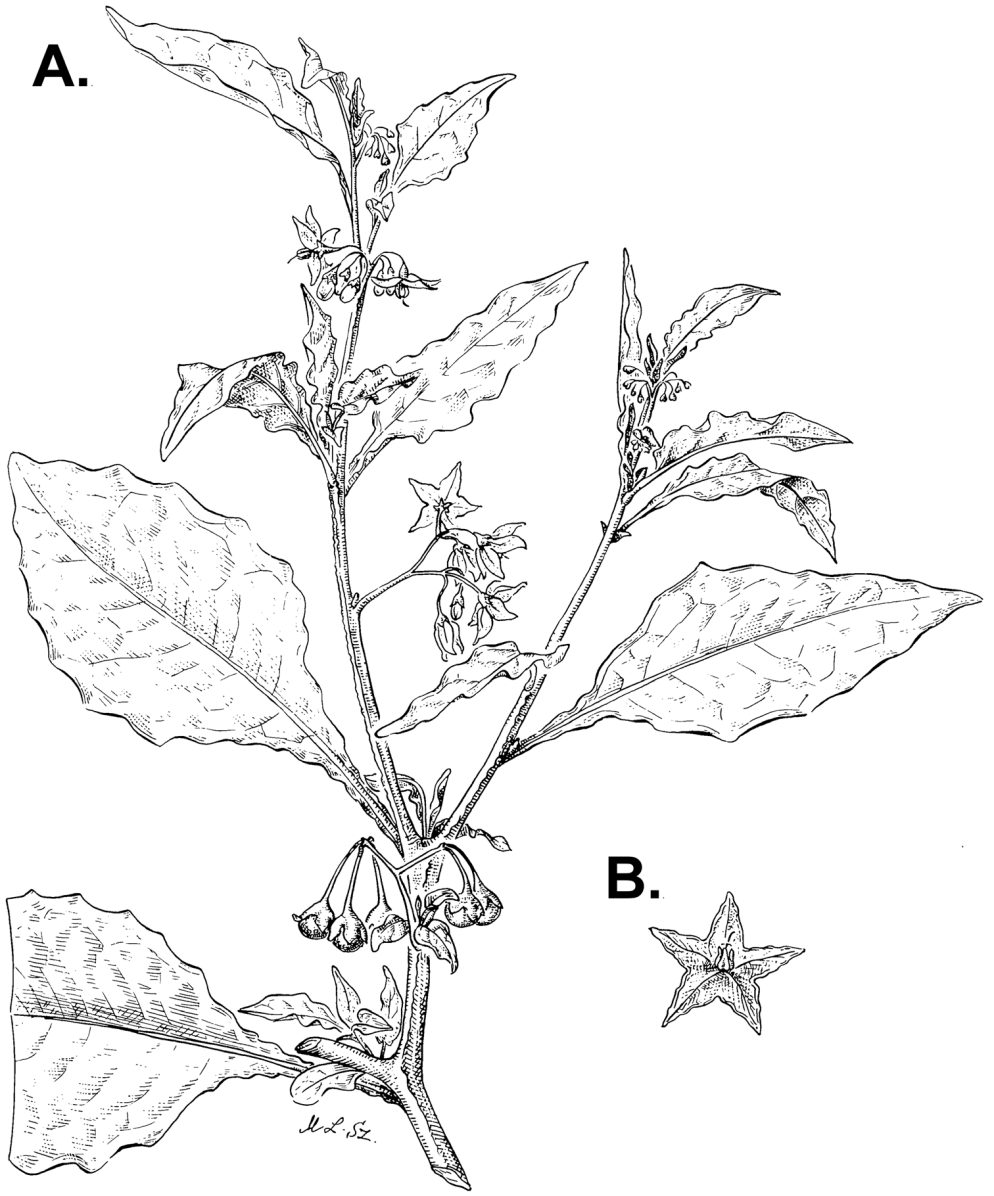
*Solanum furcatum* Dunal **A** Habit **B** Detail flower (**A–B** *Anonymous s.n.*, grown from seed sent by J. Edmonds, originally from California [ADW 42421]). Drawing by M.L. Szent-Ivany, first published in Symon (1981), courtesy of the Board of the Botanic Gardens and State Herbarium (Adelaide, South Australia), reproduced with permission.

**Figure 14.**
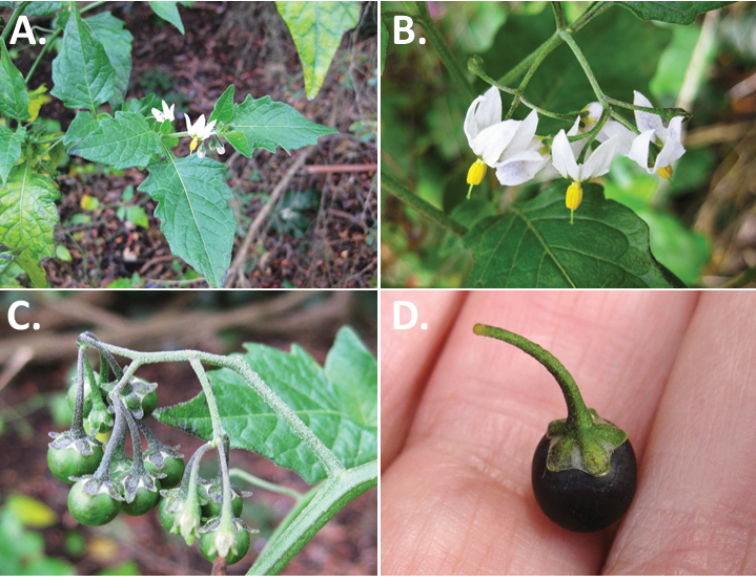
*Solanum furcatum* Dunal **A** Habit **B** Flowering branch **C** Infrutescence with developing fruits. **D** Fully mature fruit dropping off with pedicel (**A–D** *Knapp s.n.*, United States of America, California, San Francisco, Golden Gate Park, in front of California Academy of Sciences, 11 June 2013). Photos by S. Knapp.

#### Distribution

**Figure 15.**
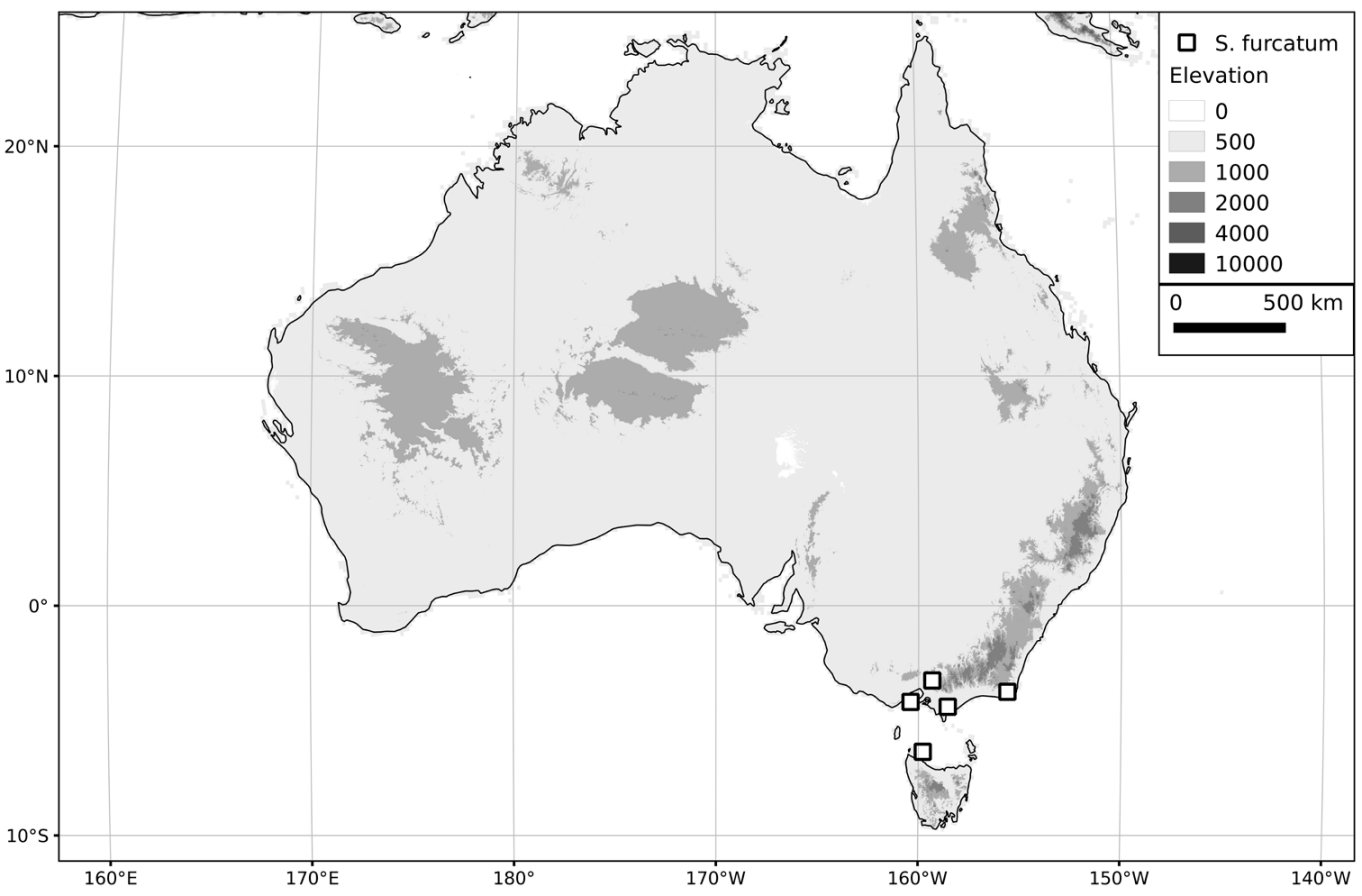
Distribution of *Solanum furcatum* Dunal in its non-native range in the Old World.

(Figure 15). Native to Pacific coastal South America and locally introduced and naturalised in the states of Victoria and Tasmania in Australia and in the western United States of America (California and Oregon, see Bohs in press); Although the disjunct distribution of the species appears peculiar, other taxa native to the Pacific coast of South America have become established in similar areas with Mediterranean climates (e.g. *Nicotiana acuminata* (Graham) Hook.).

#### Ecology.

Open places and disturbed areas, along roadsides and field margins; between sea level and 3,000 m elevation in its native range, in Australia collected in coastal habitats along the foreshore or along creeks near sea level.

#### Common names.

Australia: broad nightshade (Walsh and Entwisle 1999).

#### Uses.

None recorded for Old World collections.

#### Preliminary conservation status

(IUCN 2016). *Solanum furcatum*, while only rarely encountered in the Old World, is common in its native range in Chile (Barboza et al. 2013; Särkinen et al. 2015b) and can be assessed as LC (Least Concern; Table 7) on a worldwide basis. In its native range in South America, it has an EOO of 1,809,315 km^2^ and is not of conservation concern.

#### Discussion.

*Solanum furcatum* can be distinguished from all other Old World species based on its branched inflorescences, anthers 2.3–3.3 mm long and flowers where styles are exserted up to 3.0 mm beyond anthers. It has been sparingly introduced into areas of Mediterranean habitat (e.g. Australia and California) but appears not to spread. It is potentially confusable with *S. nigrum*, but is distinguished from it by the characters mentioned above and by its 2–13 stone cells per berry. Its subglobose buds are also distinct from the more ellipsoid buds of *S. nigrum* and *S. villosum*, but this character can be hard to see in herbarium specimens.

The name *S. douglasii* Dunal has been misapplied to some specimens of *S. furcatum* in Australia (e.g. Symon 1981; Walsh and Entwisle 1999). Careful assessment of the specimens cited in these publications and material labelled as *S. douglasii* confirms that the specimens from Victoria represent *S. furcatum*. *Solanum douglasii* is native to Mexico and the south-western United States of America and is known only from cultivation in the Old World.

Colla (1835) described *S. deltoideum* from material grown in Italy from seeds sent by C. Bertero from Chile as “Solanum scabrum”. Of the two specimens from Colla’s herbarium in TO, only one is labelled as originally identified as “S. scabrum” and this is the sheet we select as the lectotype of *S. deltoideum*.

*Solanum furcatum* was lectotypifed by Edmonds’ (1972) citation of “Type: Peru, *Dombey* s.n. (P, holotype!)”; Barboza et al. (2013) selected one of the two sheets in P (P00335357) as the second step lectotype.

The infraspecific taxa of *S. furcatum* described by Nees van Esenbeck (1843) were all based on specimens collected by F. Meyen (fide protologue) during his 1830–1832 voyage on the Princess Louise to Chile and Peru; we have found no duplicates of these and so have chosen not to lectotypify these names until further in-depth searches have been conducted. These may belong to *S. arequipense* Bitter described from Arequipa which was previously considered as synonym of *S. furcatum* but is distinct based on morphology, ecology and molecular evidence.

*Solanum rancaguense* was lectotypified by Edmonds (1972) by citation of a P specimen of Bertero 633 as “holotype”, but because there are several sheets of this collection number preserved in P, this is correctable to lectotype and we select a particular sheet (P00384088) of the five there as a second step lectotype.

We have selected lectotypes in SGO for the taxa described by Philippi here considered synonyms of *S. furcatum* following the advice of Smith and Figueiredo (2011). None of the protologues specifies herbaria and, although Philippi worked in Chile, we have followed McNeill (2014) in lectotypifying these names.

#### Selected specimens examined.

**Australia**. **Tasmania**: Circular Head, B22 between Irishtown and Edith Creek, 2 Nov 2004, *Baker 981* (HO); Circular Head, Grooms Cross rd (B22), between Irishtown and Edith Creek, 2 Nov 2004, *Baker 1010* (HO); Circular Head, Edith Creek, 2 Nov 2004, *Baker 1019* (HO); Circular Head, Copper Creek nr Smithton, May 1947, *Curtis s.n.* (HO); Circular Head, Copper Creek, May 1948, *Without collector s.n.* (HO); **Victoria**: Greater Geelong, Edwards Point State Faunal Reserve, 19 May 1984, *Albrecht 483* (MEL); Along Billy Creek in proposed extension to Morwell National Park, 15 Jul 1988, *Harris 4* (K); South Gippsland, Boolarra to Mirboo North rd, 22 Nov 1987, *Harris s.n.* (MEL); East Gippsland, Gauging Station on the Genoa River nr the Wangarabell rd, 10 Mar 2011, *Jeanes 2662* (MEL); Yarra Ranges, Tremont, 30 Jul 2006, *Stajsic 4292* (HO); Yarra Ranges, Tremont, 30 Jul 2006, *Stajsic 4292* (MEL)Greater Geelong, St Leonards, Bellarine Peninsula, 9 Nov 1947, *Willis s.n.* (MEL); Greater Geelong, St Leonards, Bellarine Peninsula, 9 Nov 1947, *Willis s.n.* (MEL); Mornington Peninsula, Main Creek, 24 Mar 1984, *Willis s.n.* (MEL); McCrae, between Dromana & Rosebud, 24 Feb 1963, *Willis s.n.* (MEL); McCrae, between Dromana & Rosebud, 24 Feb 1963, *Willis s.n.* (MEL).

**New Zealand. North Island**: Manawatu-Wanganui, Vinegar Hill, Rangitikei River near Rowa, 18 Nov 1964, *Healy 64/425* (AK).

### 5. Solanum hirtulum

Taxon classificationPlantaeSolanalesSolanaceae

A.Rich., Tent. Fl. Abyss. 2: 101. 1850

Figures 16 , 17

Solanum monactinanthum Dammer, Bot. Jahrb. Syst. 48: 236. 1912.
Type. Ethiopia. “Galla-Hochland: Arussi-Galla, Jidah”, 2600 m, Jul 1900, *H. Ellenbeck 1452* (holotype: B, destroyed; no duplicates found).
Solanum hirtulum Steud. ex Dunal, Prodr. [A. P. de Candolle] 13(1): 44. 1852, nom. illeg. (isonym), not S. hirtulum A.Rich. (1850).
Type. Based on same type as Solanum hirtulum A.Rich.

#### Type.

Ethiopia. Amhara: “Enschadcap” [Inch’et Kab Bota in Simien Mountains], 29 Jan 1838, *G.H.W. Schimper 977* (lectotype, designated by Lester 1997, pg. 286: P [P00343990]; isolectotypes: BM [BM000907529, BM000907530], BR [BR0000008799999, BR0000008421876], E [E00193283] G [G00343567, G00301657, G00343597], G-DC [G00137892], GH [GH00219333], GOET [GOET003536], HOH [HOH009832], K [K000922317], L [L0403968], LE, M [M0105600, M0105601, MO [MO-2289035, acc. # 2918258], MPU [MPU011268, MPU011269, MPU011270, P [P00343991, P00343992], PAL, REG [REG000397], S [acc. # S-G-5693], STU [STU000030, STU000031], TCD [TCD0000844], TUB [TUB003992, TUB003993], U [U0113936], W [1889-0288845, 0000606], WU [WU0033427], ZT [ZT- 00010272, ZT- 00010273]).

#### Description.

Annual to short-lived, mostly prostrate perennial herbs to 5–20(-150) cm high, branches ascending from woody tap-root. Stems decumbent to ascending, terete or very slightly winged from decurrent leaf bases, green or straw coloured, older stems not appearing spinescent, yellowish-brown, not markedly hollow; new growth moderately pubescent with simple, antrorse, uniseriate, eglandular trichomes, these 5-7-celled, 0.5–0.8 mm long, white. Sympodial units difoliate, the leaves not geminate. Leaves simple, 1.5–5.0(-6.0) cm long, 0.7–1.8 cm wide, narrowly elliptic to lanceolate, narrowing gradually to the base, concolorous, without smell; adaxial surface sparsely and evenly pubescent with simple, uniseriate trichomes like those on stem; abaxial surface with a few evenly scattered trichomes like those of the adaxial surface; major veins 5–7 pairs; base long-attenuate, decurrent on the petiole; margins entire to sinuate; apex acute to acuminate, the tip slightly rounded; petioles absent, the laminar tissue extending to the junction of leaf and stem. Inflorescences 0.5–2.2 cm long, opposite the leaves, simple, sub-umbelliform to shortly racemose, with 1–5 flowers clustered in the distal portion, sparsely pubescent with antrorse simple uniseriate trichomes like those of the stems; peduncle 0.4–1.8 cm long, straight; pedicels 0.7–1.6 cm long, ca. 0.5 mm in diameter at the base and apex, stout and spreading, articulated at the base; pedicel scars clustered at the tip of the inflorescence rhachis and overlapping, occasionally the basal scar 1–2.5 mm distant. Buds ellipsoid, the corolla halfway exserted from the calyx tube before anthesis. Flowers 5-merous, all perfect. Calyx tube 2.0–3.0 mm long, conical, the lobes 0.8–1.2 mm long, 0.8–1.2 mm wide, linear-oblong, tips rounded, densely pubescent with trichomes like those of the stems and pedicels. Corolla 12–18(-20) mm in diameter, deep purple to pale violet, stellate, lobed 3/4 of the way to the base, the lobes 4.5–8 mm long, 2–3 mm wide, spreading to reflexed, densely papillate abaxially, the papillae denser along margins and tips. Stamens equal; filament tube ca. 0.5 mm long; free portion of the filaments ca. 0.5–1.0 mm long, adaxially densely pubescent with tangled simple uniseriate trichomes; anthers 2.3–2.8 mm long, 0.6–0.9 mm wide, ellipsoid, yellow, poricidal at the tips, the pores lengthening to slits with age and drying. Ovary rounded, glabrous; style 4.5–5.5 mm long, densely pubescent with simple trichomes in the basal 1/3, exserted ca. 1 mm beyond anther cone; stigma large-capitate, the surfaces minutely papillose. Fruit a globose berry, 5–6 mm in diameter (immature?), mature berry colour not known, the pericarp thin and matte or somewhat shiny; fruiting pedicels 0.7–1.6 cm long, 0.5 cm in diameter at the base and 1.0–1.2 mm at the apex, stout with slight curving at the base, reflexed, dropping with mature fruits, not persistent; fruiting calyx not accrescent, the tube ca. 1 mm long, the lobes 1–1.5 mm long, appressed to the berry. Seeds (10-)20–35 per berry, ca. 1.8–2.0 mm long, ca. 1.5 mm wide, not markedly flattened, tear-drop shaped with a subapical hilum, pale yellow, the surfaces minutely pitted, the testal cells pentagonal to rectangular in outline. Stone cells 2 per berry, ca. 0.6 mm in diameter. Chromosome number: not known.

**Figure 16.**
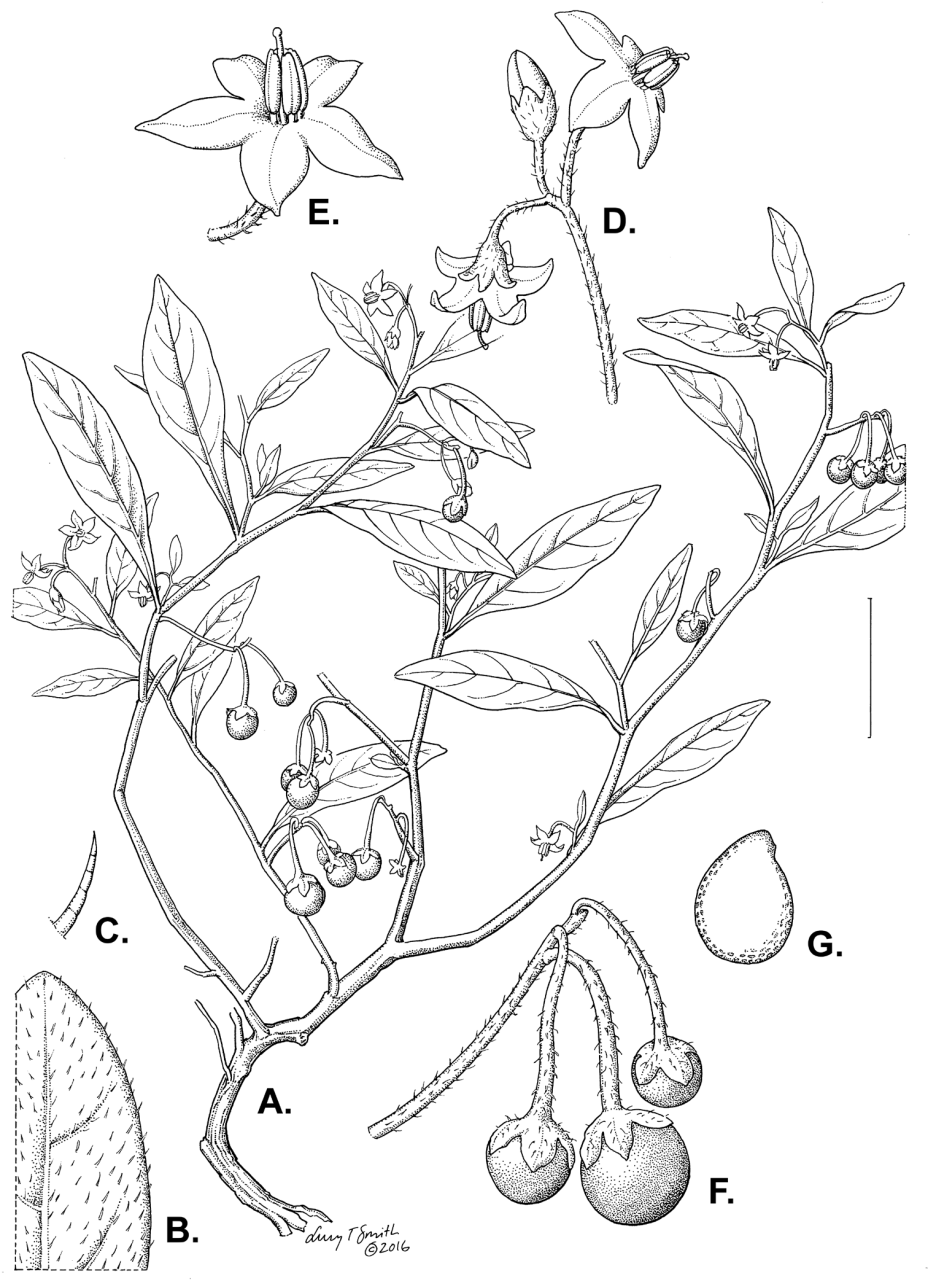
*Solanum hirtulum* A.Rich **A** Habit **B** Detail of adaxial leaf surface **C** Eglandular hair **D** Inflorescence **E** Flower **F** Infructescence **G** Seed (**A–G** *Friis et al. 11912*). Scale bar: 3 cm (**A**), 4 mm (**B**), 0.6 mm (**C**), 7 mm (**D**), 5 mm (**E**), 1 cm (**F**) and 2 mm (**G**). Drawing by L. Smith.

**Figure 17.**
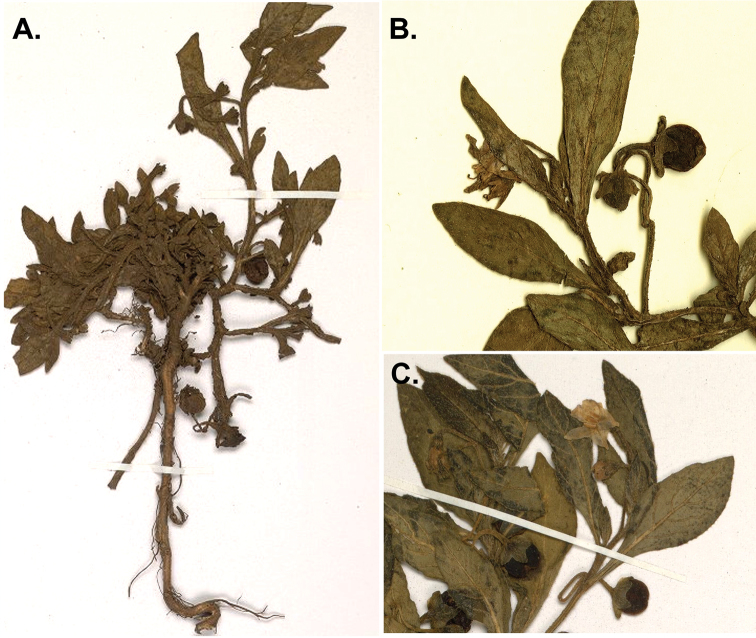
*Solanum hirtulum* A.Rich **A** Habit **B** Detail of flower and fruits **C** Detail of flower and fruits (*Schimper 977*, **A, C** isolectotype STU 000030, **B** isolectotype MPU 011270).

#### Distribution

**Figure 18.**
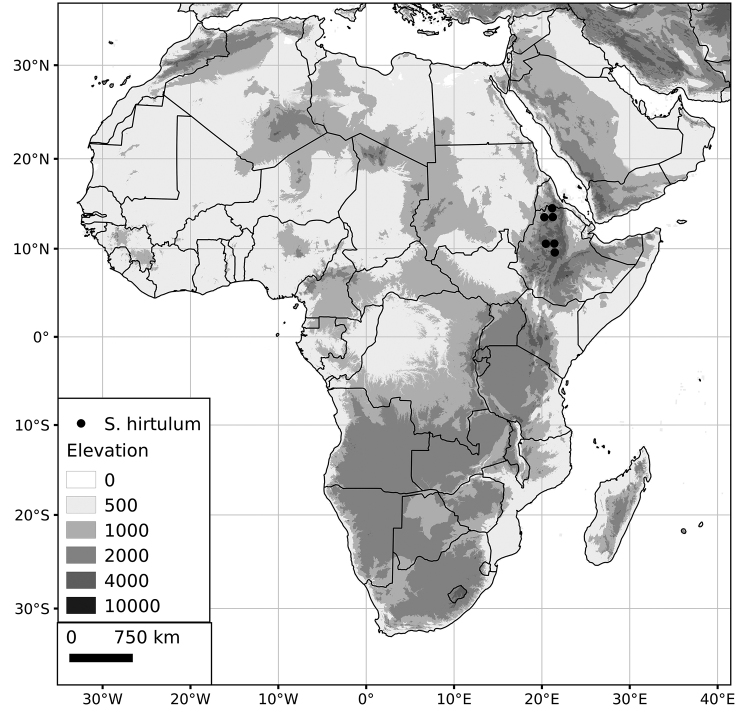
Distribution of *Solanum hirtulum* A.Rich.

(Figure 18). Endemic to the high mountains of Ethiopia on the western side of the Rift Valley.

#### Ecology.

Grows in open grassy areas, arable land, along river banks, in forest and along roadsides; between 2,200 and 3,500 m elevation.

#### Common names.

None recorded.

**Uses.** None recorded.

#### Preliminary conservation status

(IUCN 2016). *Solanum hirtulum* is a country-restricted endemic with AOO of 48 km^2^ (EN) and EOO of 49,487 km^2^ (LC). The species is known from a small number of records, and it could be that the lack of collections reflects small population sizes and/or local rarity. From the EOO measure, it would be assessed as LC (Least Concern), but since it is a single-country endemic, it merits some attention and we assign the species as NT (Near Threatened; Table 7). The high mountains where it grows are at threat from grazing and human disturbance, but *S. hirtulum*, like other members of this group, is a species of open, disturbed areas. The Simien Mountains have protected status as both a World Heritage Site and as a National Park in Ethiopia.

#### Discussion.

*Solanum hirtulum* is a distinctive prostrate, creeping herb with narrow lanceolate attenuate leaves with strigose, somewhat antrorse pubescence. The flowers are larger than others in the group in Africa and appear to always be purple or “blue”; this, however, must be taken with caution given the high degree of flower colour polymorphism in other species. The inflorescences are usually few-flowered with tightly spaced flowers at the very tip. *Solanum hirtulum* is similar to *S. memphiticum* in having black berries with a somewhat accrescent calyx, but the flowers of *S. memphiticum* are always smaller, usually white and more delicate (see description of *S. memphiticum*). The leaf bases in *S. hirtulum* are strongly attenuate and decurrent on to the stem.

*Solanum hirtulum* is a plant of Afromontane vegetation (sensu White 1981, 1983, 1993). Linder (Linder et al. 2005; Linder 2014) showed that this vegetation type (as “Tropic-montane”) was not necessarily a distinct unit across Africa, but instead was locally distinct based on high turnover in particular mountain blocks. Most clades from these forest types were Miocene in age (Linder 2014). For Ethiopia, Friis et al. (2010) further characterised this vegetation as “moist evergreen Afromontane forest”, with additional complexity added by the grasslands that are characteristic of the seral stages of forest regrowth in these areas. This forest type is only intact in limited areas (Friis et al. 2010); these are the forests where wild coffee grows and are areas of high endemism in the Ethiopian flora (Vivero et al. 2006). It is likely that *S. hirtulum* also occurs at the lower levels of Afroalpine vegetation (*sensu* Friis et al. 2010), in grasslands at forest edges. The type of *S. hirtulum* was collected from the Simien Mountains, the most important hotspot of endemism for Afroalpine and Afromontane species (Vivero et al. 2006).

The type number of *S. hirtulum* (*Schimper 977*) is also that for a plant collected in 1837 in Saudi Arabia (the type of *Seddera intermedia* Hochst., Convolvulaceae, see http://apps.kew.org/herbcat/getImage.do?imageBarcode=K000852498). He clearly reused numbers for his Ethiopian plants.

*Solanum monactinanthum* is here placed in synonymy based on the description and following Edmonds (2006a), no duplicates of the type (*Ellenbeck 1452*) that was destroyed in Berlin have been found.

#### Specimens examined.

**Ethiopia**. sin. loc., 26 Oct 1862, *Schimper 631* (BM, E, K, P, W); **Addis Ababa**: Addis Ababa, 30 Sep 1937, *Piovano 511* (FT); Addis Ababa, 19 Oct 1937, *Senni 1837* (FT); **Amhara**: Semien, Debarek Semien Gonder Region, 11 Jul 1909, *Chiovenda 889* (FT); 10 km SE of Debre Markos along rd to Addis Ababa, between Debre Markos and Addis Ababa, 25 Oct 2004, *Friis et al. 11912* (K); Bichena Awraja, c. 36 km NW of Debre Work (Gojjam region), 30 Oct 1981, *Mesfin Tadese* & *Kagnew 1659* (K); in campis Debra Eski, 19 Oct 1850, *Schimper 74* (P); Semien, Derasghie, 25 Dec 1952, *Scott 292* (K); Dejen to Debra Marcos, 48 km NW of Dejen, 24 May 1980, *Thulin 3914* (K, MO); mule track between Debarak and Geech, 16 Sep 1969, *de Wilde* & *Gilbert 5* (EA, MO); **Oromia**: Semien Shewa region, Holetta, 4 May 1953, *Mooney 4752* (K); Addis Alem, 20 Sep 1926, *Omer Cooper s.n.* (K); **Tigray**: Adua, Prope Adoam, Abyssinia, 1852, *Schimper s.n.* (P).

### 6. Solanum memphiticum

Taxon classificationPlantaeSolanalesSolanaceae

J.F.Gmel., Syst. Nat. ed. 13[bis] 2(1): 385. 1791

Figures 19 , 20

Solanum nigrum L. var. hirsutum Vahl, Symb. 2: 40. 1791.
Type. Based on Forsskål’s “S. aegyptiacum b) Fructu nigro; foliis integris villosissimus” (=*Solanum memphiticum* J.F.Gmel.)
Solanum hirsutum (Vahl) Dunal, Hist. Nat. Solanum 158. 1813.
Type. Based on Solanum nigrum L. var. hirsutum Vahl
Solanum grossidentatum A.Rich., Tent. Fl. Abyss. 2: 101. 1850, as “grossedentatum”.
Type. Ethiopia. “Tchélikote” [Chelicote], *R. Quartin-Dillon & A. Petit s.n.* (lectotype, designated by Lester 1997, pg. 285: P [P00344046]; isolectotypes: GOET, P [P00344047], Z; possible isolectotype: K [K001156454]).
Solanum nigrum L. var. rigidum Dunal, Prodr. [A. P. de Candolle] 13(1): 50. 1852.
Type. Yemen. Sin. loc., 1837, *P.E. Botta s.n.* (lectotype, designated by D’Arcy 1974a, pg. 738: P [P00055305]).
Solanum hirsutum (Vahl) Dunal var. abyssinicum Dunal, Prodr. [A. P. de Candolle] 13(1): 58. 1852.
Type. Ethiopia. Tigray: Adigrat, prope Adoam [Adoa] “nomen Abyssinicum: Alam tch’aguar”, *G.H.W. Schimper 46* (lectotype, designated by Edmonds 2012, pg. 143: G [G00015053]; isolectotypes: BM [BM000942983], BR [BR0000008422200], GH [GH00139634], HBG [HBG511468], K [K000922192], L [L0700161], LE [LE00016944, LE00016943], LG [LG0000090028793], M [M-0105602], P [P00344000, P00343998, P00343999, P00344001, P00344002], TUB [TUB003999], W [1889-0283797]).
Solanum pruinosum Dunal var. pilosulum Dunal, Prodr. [A. P. de Candolle] 13(1): 59. 1852.
Type. Sudan. Blue Nile: Sennaar, 1831, *G. Acerbi s.n.* (holotype: G-DC [G00144592]).
Solanum subuniflorum Bitter, Repert. Spec. Nov. Regni Veg. 10: 546. 1912.
Type. Tanzania. Marangu near Mount Kilimanjaro, *G. Volkens 2108* (neotype, designated by Edmonds 2007, pg. 665: BR [BR0000008800091]).
Solanum plebeium A.Rich. var. grossidentatum (A.Rich.) Chiov., N. Giourn. Bot. Ital. 26: 159. 1919, as “grossedentatum”.
Type. Based on Solanum grossidentatum A.Rich.
Solanum nigrum L. var. grossidentatum (A.Rich.) De Wild., Pl. Bequaert. 1: 431. 1922, as “grossedentatum”.
Type. Based on Solanum grossidentatum A.Rich.
Solanum memphiticum J.F.Gmel. var. abyssinicum (Dunal) Cufod., Bull. Jard. Bot. État Bruxelles 33(3): 872. 1963.
Type. Based on Solanum hirsutum (Vahl) Dunal var. abyssinicum Dunal

#### Type.

Locality unknown [Yemen or Saudi Arabia, but most likely Yemen], *Herb. Forsskål 421* (lectotype, designated by Edmonds 2007, pg. 665: C [C10013456]).

#### Description.

Annual or short-lived sprawling perennial herbs to 1.5 m tall, woody and branching at base. Stems spreading to decumbent, terete or occasionally very slightly angled, green, older stems green or straw colour, not markedly hollow; new growth densely viscid-pubescent with simple, spreading, uniseriate, mixed glandular and eglandular trichomes, these 3-10-celled, 0.5–2 mm long, with a terminal single-celled gland if glandular; older stems glabrescent. Sympodial units difoliate, the leaves not geminate. Leaves simple, (1.5-)2–9 cm long, (0.8-)1.2–5.5 cm wide, elliptic to ovate and widest in the basal third, membranous, concolorous, foul-smelling when crushed; adaxial surfaces moderately viscid-pubescent with a mixture of glandular and eglandular simple uniseriate trichomes 0.5–2 mm long like those of the stems, these denser along the veins; abaxial surfaces densely viscid-pubescent with similar glandular and eglandular simple uniseriate trichomes, these evenly distributed on veins and lamina; base acute, then attenuate and decurrent on to the petiole; margins entire or more often irregularly toothed, the teeth 2–4 mm long, acute; apex acute to acuminate, the tip often blunt and usually somewhat rounded; petioles 0.5–1.5 cm long, winged from the decurrent leaf base. Inflorescences 1–2.5(-3) cm long, internodal, simple, umbelliform to sub-umbelliform, with (2-)3–5(-8) flowers clustered at the tip, densely viscid-pubescent with mixed glandular and eglandular simple uniseriate trichomes like those of the stems; peduncle 0.9–2(-2.3) cm long, straight; pedicels 7–9 mm long, ca. 0.5 mm in diameter at the base, 0.5–0.8 mm in diameter at the apex, spreading, densely to moderately viscid-pubescent like the inflorescence axis, articulated at the base; pedicel scars clustered at the tip of the inflorescence, the scar from the basal flower spaced 1–2 mm from the rest. Buds globose to ovoid, the corolla exserted more than halfway from the calyx tube before anthesis. Flowers 5-merous, all perfect. Calyx tube 1–2.5 mm long, deeply conical, the lobes 1–1.5(-2) mm long, 0.5–0.8 mm wide, long-triangular, tips rounded, densely viscid-pubescent with mixed glandular and eglandular simple uniseriate trichomes to ca. 0.5 mm long. Corolla (8-)10–12 mm in diameter, white, rotate-stellate to stellate, lobed ca. 2/3 of the way to the base, the lobes 4–5 mm long, 3–4 mm wide, spreading or reflexed at anthesis, minutely pubescent-papillate abaxially with simple eglandular trichomes ca. 0.2 mm long, these white when dry. Stamens equal; filament tube minute; free portion of the filaments 0.5–1 mm long, glabrous or occasionally with a few tangled simple uniseriate trichomes adaxially; anthers (2-)2.5–3 mm long, 0.75–1 mm wide, ellipsoid, yellow, poricidal at the tips, the pores lengthening to slits with age and drying. Ovary globose, glabrous; style 4–6 mm long, minutely puberulent in the lower 1/4, merely papillate in plants with glabrous filaments, exserted ca. half the length of the anthers; stigma capitate-globose, bright green in live plants, the surface minutely papillate. Fruit a globose berry, 7–10 mm in diameter, black when ripe, the pericarp thin, matte and somewhat translucent; fruiting pedicels 0.8–0.9 cm long, ca. 0.75 mm in diameter at the base and to ca. 2 mm at the apex, somewhat woody, deflexed to spreading (often appearing like spokes of a wheel), dropping off with mature fruits, not persistent; fruiting calyx somewhat accrescent, the tube 2.5–3 mm long, the lobes 2.5–4 mm long, ca. 1.5 mm wide, somewhat spathulate, appressed to spreading, covering ca. 1/2 of the berry. Seeds 10–30 per berry, 1.5–2.5 mm long, 1.5–2 mm wide, flattened and tear-drop shaped with a subapical hilum, pale brown, the surfaces minutely pitted, the testal cells more or less pentagonal with some sinuate margins. Stone cells 2 per berry, 0.3–0.7 mm in diameter, usually borne near the base of the berry. Chromosome number: *2n*=6x=72 (Bhiravamurty and Rethy 1983; Olet et al. 2015).

**Figure 19.**
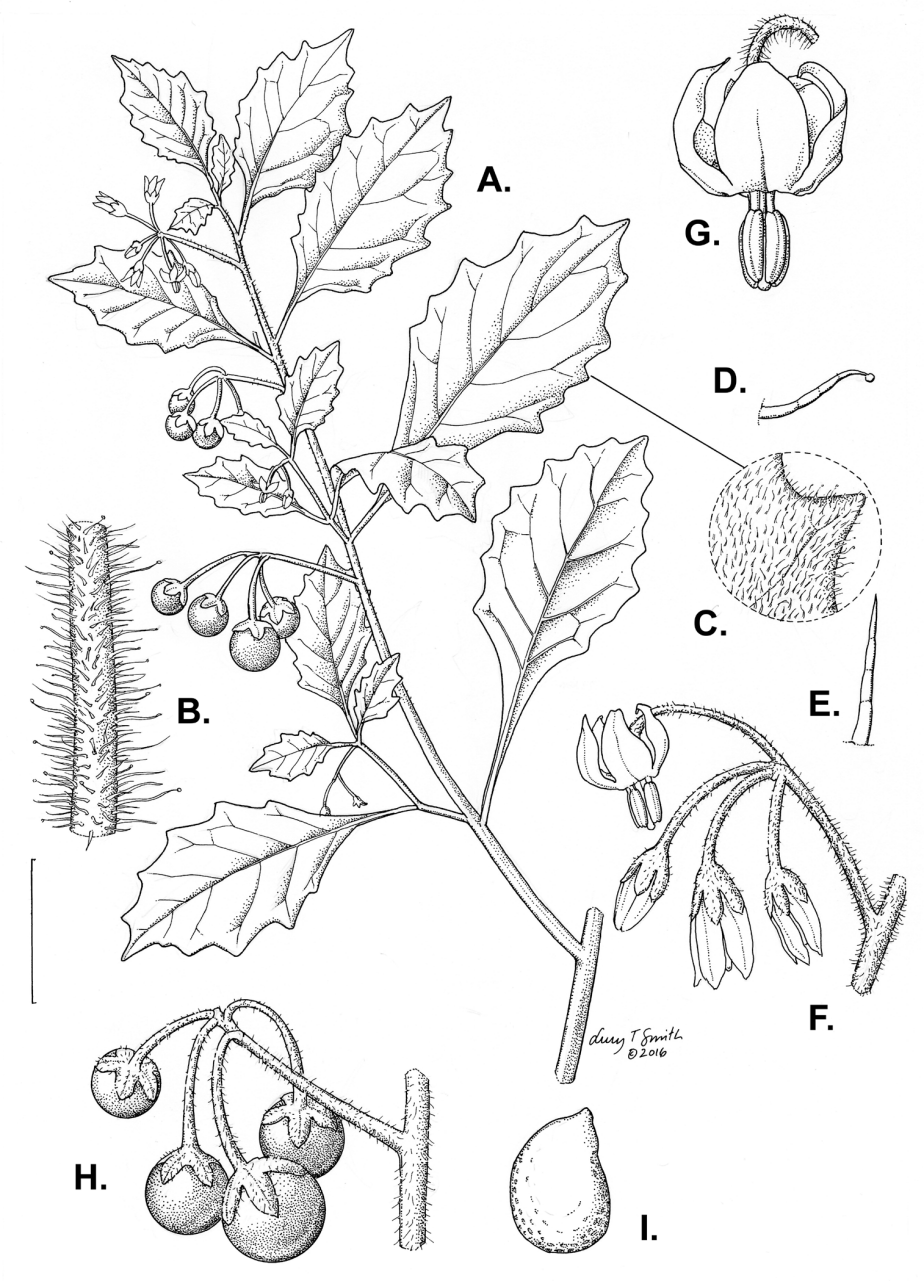
*Solanum memphiticum* J.F.Gmel **A** Habit **B** Detail of stem with glandular indumentum **C** Detail of abaxial leaf surface **D** Glandular trichome **E** Eglandular trichome **F** Inflorescence **G** Flower at anthesis **H** Infructescence **I** Seed (**A–E, H** *de Wilde 8017*; **F–I** *Gilbert 1387*; **G** Nijmegen acc. A34570474). Scale bar: 3 cm (**A**), 3 mm (**B**), 4 mm (**C, G**), 0.8 mm (**D–E**), 1.5 cm (**F, H**) and 2 mm (**I**). Drawing by L. Smith.

**Figure 20.**
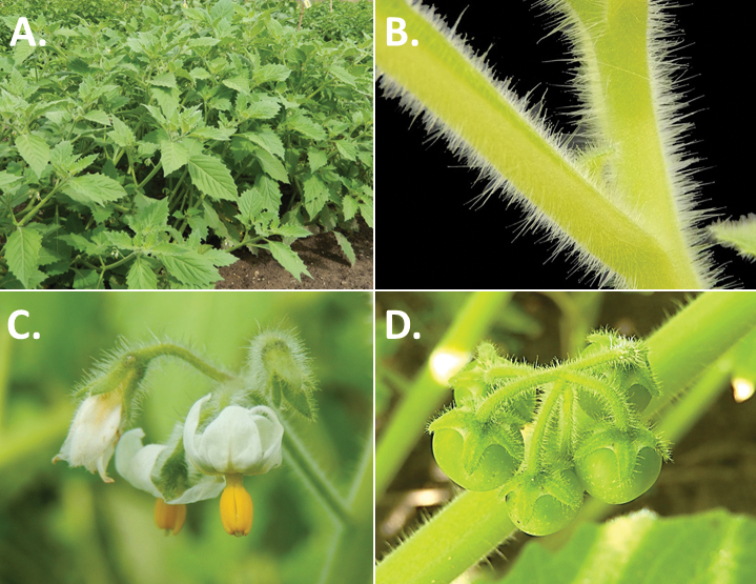
*Solanum memphiticum* J.F.Gmel **A** Habit **B** Glandular indumentum **C** Flowers at full anthesis **D** Maturing fruits with long, appressed calyx lobes (**A–B, D** Nijmegen acc. A14750032; **C** Nijmegen acc. A34750474). Photos by S. Knapp and G. van der Weerden.

#### Distribution

**Figure 21.**
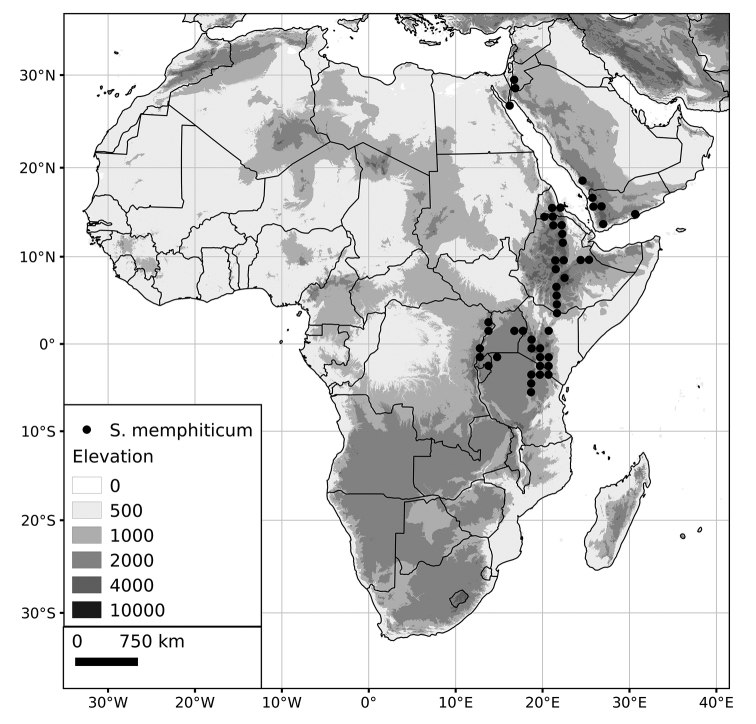
Distribution of *Solanum memphiticum* J.F.Gmel.

(Figure 21). Distributed from the Arabian Peninsula (Saudia Arabia and Yemen) to Egypt and south in eastern Africa to Kenya and Tanzania, we have only seen a single collection from eastern Democratic Republic of the Congo.

#### Ecology.

Grows in open areas and along streams in forests and dry areas, also cultivated in eastern Africa; from 800 to 3,000 (-3,500) m elevation.

#### Common names.

Ethiopia: alam tch’aguar; Kenya: ap-poinet, isusa, manage, nafu, ol’ momoit/olmomoi, soiyot, sonja sucha, sujet; Saudi Arabia: gebel soda; Uganda: ekyala ky’ente, kyalakyente, orushwiga, osiga.

#### Uses.

In eastern Africa, leaves are eaten as spinach and berries are said to be edible.

#### Preliminary conservation status

(IUCN 2016). *Solanum memphiticum* is a widespread species with AOO of 320 km^2^ (EN) and EOO of 4,489,278 km^2^ (LC); considering the weedy nature of the species and the large EOO, we suggest a preliminary status of LC (Least Concern; Table 7) for the species. Like other members of the group, it is a species of open, disturbed areas, but is less common than *S. tarderemotum* or *S. villosum* in eastern Africa.

#### Discussion.

*Solanum memphiticum* was long confused with *S. villosum*; both are similarly villous plants with toothed leaves that are sometimes pubescent with glandular trichomes. Edmonds (2007) clarified not only the differences between the two species, but also the identity and correct name for *S. memphiticum*. The species can be easily distinguished in fruit; *S. villosum* has characteristic red-orange (or yellow) berries with strongly reflexed calyx lobes, while *S. memphiticum* has green or black berries with the calyx lobes somewhat accrescent and covering at least a third of the berry. Fruiting pedicels of *S. memphiticum* are typically spreading and, in live plants, form a small bicycle spoke-shaped structure, while those of *S. villosum* are deflexed. Live plants of the two species are easy to distinguish: the leaves (glandular trichomes) of *S. memphiticum* have a strong foetid odour of rotting meat that is markedly different from the slightly pleasant odour of *S. villosum*.

In flower, *S. memphiticum* can be distinguished by its rotate-stellate corolla with small lobes that are strongly reflexed in flower and calyx lobes that extend beyond the corolla sinuses in open flowers. The flowers of *S. memphiticum* are delicate and only last a short time as compared to other species (the corolla bruises easily). Leaves of *S. memphiticum* are generally longer and thinner than those of *S. villosum* and the base is more decurrent on to the petiole so the free part of the petiole in *S. memphiticum* is much shorter than in *S. villosum*.

The parental origin of the tetraploid species has not been investigated in detail. Jaeger (1985) suggested that *S. memphiticum* might be part of a more broadly circumscribed *S. villosum*, which included a wide range of fruit colours. Manoko (2007) showed that *S. memphiticum* (as *S. grossedentatum*) does not group closely with *S. villosum* based on AFLP data but forms a distinct group related to accessions identified as *S. tarderemotum*, *S. chenopodioides*, *S. nitidibaccatum* (as *S. physalifolium*) and *S. tweedianum*. Further molecular work, based on sequence data, will be needed to understand the relationships and parental origins of *S. memphiticum*, but possible diploid parents could be the morphologically closely related *S. sarrachoides* or *S. nitidibaccatum* that both occur in the Old World. Both of these species are glandular and have an enlarged calyx in the fruit, characters shared with *S. memphiticum*.

*Solanum memphiticum* was previously commonly known as *S. grossidentatum* or *S. hirsutum* (see Manoko 2007), but Edmonds (2007), in her analysis of the morelloid solanums in Pehr Forsskål’s herbarium, showed that the correct name for this black-fruited, pubescent plant is *S. memphiticum* Gmel. Cufodontis (1963) had used the name earlier, but some confusion occurred due to the later homonym *S. memphiticum* Mart., that Edmonds (2007) suggested was synonymous with *S. scabrum*, but we place in the synonymy of *S. nigrum*. Homonymy is rampant in the morelloid solanums, making name changes like this inevitable; the name *S. memphiticum* is now well-established for this species in Africa (e.g. Edmonds 2012).

**Selected specimens examined. Burundi**. **Ruyigi**: Musongati, FTEA region: BUR, 10 May 1974, *Reekmans 3419* (EA, MO).

**Democratic Republic of the Congo**. **Nord-Kivu**: Ruindi, Nov 1937, *Lebrun 8380* (K).

**Egypt**. **South Sinai**: Wadi El Sheikh, Sinai, 15 Apr 1937, *Shabetai s.n.* (K).

**Eritrea**. **Anseba**: Geleb, Gheleb-Caropebir, 16 Jan 1893, *Terracciano* & *Pappi 2027* (FT); **Debub**: Adi Ugri-Mai Tacala, 4 Sep 1909, *Bellini 335* (FT); Saganeiti, gorge Gona pres Addingofon, 29 Mar 1892, *Schweinfurth* & *Riva 1319* (FT, K); **Gash Barka**: Badum, lungo fiume Mareb, 10 Jan 1906, *Pappi 6893* (FT); Get Arba, Gret-Arba, 7 Jan 1893, *Terracciano* & *Pappi 1706* (FT); **Maekel**: Asmara, 12 Sep 1912, *Baldrati 3536* (FT); Asmara, 2 Aug 1902, *Pappi 2099* (EA, MO, P, SI); **Semienawi Keyih Bahri**: Nefasit-Maha-bar, 2 Feb 1909, *Fiori 1593* (FT); Illalia-Scilliki; Assaorta, 28 Mar 1893, *Pappi 3601* (FT).

**Ethiopia. Addis Ababa**: Addis Ababa, 18 Jul 1962, *Mooney 9136* (FT, K); **Amhara**: c. 15 km S of Debre Sina on the main rd towards Debre Berhan, 22 Nov 2000, *Friis et al. 10115* (K); Dessie, Wollo Prov., 18 Aug 1946, *Hall 22* (BM); Wello Prov., Azewagedel mountain, 2 km E of Desse, 11 Apr 1969, *Sutherland 167* (MO); Dessie, 26 May 1938, *Vatova 2417* (FT); **Oromia**: Borena, Mega, presso fortino nuovo, 4 May 1937, *Cufodontis 627* (FT, W); Agheremariam-Dilla rd, 3 Dec 1952, *Gillett 14586* (EA, FT, K); lower slopes of Mt. Cilalo, nr Asella, 10 Sep 1965, *de Wilde* & *de Wilde-Duyfjes 8017* (K, MO, P); **Southern Nations (SNNP)**: Gurage Mountains, above Butajira towards the village of Ageta on the track towards Endibis, 24 Feb 2000, *Friis et al. 10066* (K); **Tigray**: Amba Alaga pass, 9 Oct 1995, *Friis et al. 6640* (K); Bellaka, 8 Nov 1854, *Schimper 506* (E, FT, W); Arba Tensesa, 7 Oct 1862, *Schimper 523* (BM).

**Jordan**. East Jordan, Pelit, 30 Apr 1963, *Gillett 15973* (K); **Aqaba**: Wadi Rum, 13 Apr 1945, *Davis 8987* (E); **Ma’an**: Petra, 28 Dec 1935, *Dinsmore 12151* (E).

**Kenya**. **Central**: Nyeri, Aberdare National Park, The Ark, 7 Apr 1975, *Hepper* & *Field 4912* (K); Kiambu, Makuyu, Fort Hall Dist, 26 Jun 1960, *van Someren 11980* (EA, K); **Eastern**: Makueni, Chyulu North, 21 May 1938, *Bally B-7788* (K); Makueni, Chyulu North, 28 Apr 1938, *Bally B-8306* (K); Makueni, Chyulu Hills North, Apr 1938, *van Someren 7644* (K); **Nairobi**: Mathare Valley, between Mathare Police Station and Eastleigh Section One, 11 Sep 1971, *Mwangangi* & *Kasyoki 1797* (EA, K); Nairobi, FTEA region K4, 1942, *Nattrass 341 b* (EA, MO); **Rift Valley**: Kericho, Londiani to Elburgon, Dec 1905, *Baker K-346* (EA, K); Nakuru, Lake Naivasha, West shore Mennel’s Farm, 14 Feb 1971, *Gillett 19300* (EA, K); *Glover et al. 181* (EA, K); Kajiado, Olekairtoror Escarpment, 20 mi from Narok on Nairobi rd, 14 Jul 1962, *Glover* & *Samuel, 3110* (EA, K); K6 Rift Valley, Suswa volcano, 1 Jun 1997, *Phillipson* & *Bytebier 4785* (MO); **Western**: Nandi, Kapsoret Forest, 15 Jun 1951, *Williams 240* (EA, K).

**Saudi Arabia**. Al Mahmoud, 35 km N of Abha, 21 May 1980, *Boulos* & *Ads 14150* (E, K); Abha Pass (nr. W. Abha), 25 Oct 1971, *Popov 71 257* (BM); **Asir**: Jebel Sudah, c. 18 km N of Abba, 5 Apr 1979, *Collenette 1269* (K).

**Somalia**. Upper Sheikh Kitchen Garden, 1 Dec 1919, *Godman 67* (BM, MO); **Saaxil**: Ally Ully nr Sheikh, 11 May 1973, *Wood S/73-65* (K).

**Sudan**. **Darfur**: Jebel Marra, Golel, c. 120 km E of Zalingei, 22 Jan 1965, *de Wilde et al. 5504* (K, MO).

**Tanzania**. **Arusha**: Lake Manyara National Park, Mto ya Ukindu, 29 Nov 1963, *Greenway* & *Kirrika 11096* (EA, K); Monduli Forest Reserve, T2. Nr Olchoropus Village, 25 Jan 2001, *Simon et al. 720* (MO); **Central**: Kondoa, Great North rd, 11 Jan 1962, *Polhill* & *Paulo 1130* (K); **Kagera**: Ngara, Murugwanza, Ibivyaza Bakobwa, Bugufi, 20 Jan 1961, *Tanner 5617* (K); **Kilimanjaro**: Osirwa Farm, TBL Estates, 26 Jan 1994, *Grimshaw 94-148* (K); Ulei, Kwa Sadala, 2 May 1994, *Grimshaw 94-470* (K); Moshi, Sanya River, Mar 1928, *Haarer 1209* (EA, K); **Lake**: Ngara, Kirushya, Bugufi, 23 Nov 1959, *Tanner 4530* (K); **Mbulu**: Pienaars Heights, Great North rd, 120 mi S of Arusha, 5 May 1962, *Polhill* & *Paulo 2342* (EA, K); **Northern**: Mbulu, Mbulumbul, Block D1, 24 Jun 1944, *Greenway 6952* (EA, K).

**Uganda**. **Central**: Masaka, Kasambya, Kigezi, Feb 1948, *Purseglove P-2595* (K); **Eastern**: Sironko, Budadiri, Bugishi, Jan 1932, *Chandler 405* (K); Serere, at Tira, Jul 1926, *Maitland 1290* (K); **Northern**: Zombo, Paidha, 28 Aug 1953, *Chancellor 185* (K); **Western**: Kigezi D.F.I, 28 Aug 1972, *Goode G-5 -72* (K); Kasese, Muhokya, Ruwenzori, 25 Dec 1925, *Maitland 1790* (K); Kigezi, Kachwekano Farm, Sep 1949, *Purseglove 3121* (EA, K); Kisoro, Virunga-Kette, Muhavura, Nkanda, 25 Nov 1954, *Stauffer 957* (EA, K, P).

**Yemen**. **Hadramaut**: Al Mukalla, Arabia, West rd, 6 Sep 1949, *Guichard KG/HAD 35* (BM); Kor Seiban, 14 Sep 2002, *Killian et al. YP-3578* (B); **Sa’dah**: Sadah, 1 Jul 1984, *Gordon 609 B* (E); **Sana’a**: Sana’a, 12 Mar 1981, *Miller 3004* (E); 14 Feb 1934, *Rathjens s.n.* (BM); Menacha, 7 Mar 1889, *Schweinfurth 1476* (BM, GH, K, P); Ar Rowdah nr San’a, 23 Feb 1972, *Wood 72-7* (BM); 10 Oct 1974, *Wood Y/74-20* (BM); **Ta’izz**: Jabal Sabir, c. 15 km S of Taizz, 11 Jun 1982, *Gordon 11* (E); Jabal Sabir, nr Taiz, 23 Sep 1977, *Lavranos 15947* (E).

### 7. Solanum nigrum

Taxon classificationPlantaeSolanalesSolanaceae

L., Sp. Pl. 1: 186. 1753

Figures 22 , 23

Solanum nigrum L. var. vulgare L., Sp. Pl. 186. 1753.
Type. “Solanum 3 α”; cultivated in George Clifford’s garden in Hartekamp, The Netherlands, *Anon. s.n.* (lectotype, designated here: BM [BM000558026]).
Solanum nigrum L. var. judaicum L., Sp. Pl. 186. 1753.
Type. Unknown (no specimens or illustrations cited).
Solanum vulgatum Baumg., Fl. Lips. 120. 1790, nom. illeg. superfl.
Type. Based on Solanum nigrum L. [S. nigrum L. from Syst. Veg. ed. 14: 224. 1784 cited in synonymy]
Solanum humile Salisb., Prodr. Stirp. Chap. Allerton 134. 1796, nom. illeg. superfl.
Type. Based on Solanum nigrum L. (cited in synonymy)
Solanum judaicum Besser, Prim. Fl. Galiciae Austriac. 1: 183. 1809.
Type. No localities of specimens cited; probably from what is now Ukraine (no specimens cited; no original material located).
Solanum morella Desv., Pl. d’Angers 113. 1818, nom. illegit. superfl.
Type. Based on Solanum nigrum L. (cited in synonymy)
Solanum parviflorum Moretti ex Badarò, Giorn. Fis. Chim. Storia Nat. Med. Arti Dec. 2, 7: 364. 1824, nom. illeg, not Solanum parviflorum Nocca (1793)
Type. Italy. [Liguria]“ in olivetis Liguriae occid[ose]”, 1824, *G.B. Badarò s.n.* (no specimens cited; lectotype, designated by D’Arcy 1974a, pg. 735 [as type]: G-DC [G00144311]).
Solanum cestrifolium Jacq. ex Spreng., Syst. Veg., ed. 16 [Sprengel] 1: 680. 1825.
Type. “Patria?” [origins unknown, although probably from cultivation?] (no specimens cited; no original material found).
Solanum rhinozerothis Blume, Bijdr. Fl. Ned. Ind. 13: 695. 1826.
Type. Indonesia [no locality cited in protologue], *C.L. Blume s.n.* (no specimens cited; neotype, designated here: L [L2883159]).
Solanum vulgatum (L.) Spenn., Fl. Friburg. 2: 427. 1826.
Type. Based on Solanum nigrum L. var. vulgare L.
Solanum vulgatum (L.) Spenn. var. nigrum (L.) Spenn., Fl. Friburg. 2: 427. 1826.
Type. Based on Solanum nigrum L.
Solanum vulgatum (L.) Spenn. var. chlorocarpum Spenn., Fl. Friburg. 3: 1074. 1829.
Type. Switzerland. Fribourg: Sin. loc. (no specimens cited; no original material found).
Solanum moschatum C.Presl, Delic. Prag. 77. 1832.
Type. Italy. Sicily: Palermo (“in cultis ruderatis Panormi Siciliae”, *Anon. s.n.* (no specimens cited; original material at PR?, PRC?, not found).
Solanum nigrum L. var. perennans Bertol., Fl. Ital. [Bertoloni] 2: 634. 1836.
Type. Based on Solanum moschatum C.Presl.; Solanum parviflorum Moretti ex Badarò (no specimens cited; no original material found).
Solanum nigrum L. var. atriplicifolium G.Mey., Chloris Han. 265. 1836.
Type. France. Pays de la Loire: “St. Germain de Calberte, basse Lozère”/”Mans [Le Mans]”[annotation “Solanum atriplicifolium” in Desportes hand], 1806, [*illegible] s.n.* (lectotype, designated here: G-DC [G00144334]).
Solanum nigrum L. var. atriplicifolium Desp. ex G.Don, Gen. Hist. 4: 412. 1838, nom. illeg. (isonym), not Solanum nigrum L. var. atriplicifolium G.Mey. (1836)
Type. Based on same original material as Solanum nigrum L. var. atriplicifolium G.Mey.
Solanum vulgare Hegetschw., Fl. Schweiz 219. Dec 1838-Jan 1839, nom. illeg. superfl.
Type. Based on “Solanum nigrum Willd.” (= S. nigrum L.)
Solanum chenopodium Raf., Autik. Bot. 107. 1840.
Type. “Europa” (no specimens cited; original material probably lost).
Solanum exaratum Raf., Autik. Bot. 107. 1840.
Type. “Europa” (no specimens cited; original material probably lost).
Solanum bidentatum Raf., Autik. Bot. 108. 1840.
Type. “Italia, Sicilia” (no specimens cited; original material probably lost).
Solanum tauschii Opiz, Oekon.-techn. Fl. Böhm. [Berchtold & al.] 3: XX. 1843.
Type. Czech Republic. “Prag”, *I.F. Tausch s.n. [Herb. Flor. Boehm. 1076*] (no herbaria cited; no original material found, perhaps at PR?); “Luzic”, *Sadel s.n.* (no herbaria cited; no original material found, perhaps at PR?); “Folimanta bei Prag”, *Tanbler s.n.* (no herbaria cited; no original material found, perhaps at PR?); Sin loc., *P.M. Opiz 10/8 40* (no herbaria cited; no original material found, perhaps at PR?).
Solanum reineggeri Opiz, Oekon.-techn. Fl. Böhm. [Berchtold & al.] 3(2): XIX. 1843.
Type. Austria. Niederösterreich; Sin. loc., *Reinegger s.n.* (no herbaria cited; no original material found, perhaps at PR?).
Solanum decipiens Opiz, Oekon.-techn. Fl. Böhm. [Berchtold & al.] 3(2): XXIV. 1843.
Type. Czech Republic. “Troja”, *P.M. Opiz 10/838*; “Ruchelbad”, *P.M. Opiz 23/10 835*; “Radlic”, *P.M. Opiz 4/8 40*; “Szaslau”, *Janoti 804* (no herbaria cited; no original material found, perhaps at PR?).
Solanum schultesii Opiz, Oekon.-techn. Fl. Böhm. [Berchtold & al.] 3(2): XXIV. 1843.
Type. Czech Republic. “ Im Baumgarten” *P.M. Opiz 10/10 35*; “Prag”, *P.M. Opiz s.n.* (no herbaria cited; no original material found, perhaps at PR?).
Solanum nigrum L. forma stenopetalum A.Braun ex Döll, Rhein. Flor. 412. 1843.
Type. Germany. “Bei Ettlingen”, *A. Braun s.n.* (no herbaria cited; no original material found, not found at KR); Germany. “Bei Carlsruhe in der Nähe des Linkenheimer Thores”, *A. Braun & J.C. Döll s.n.* (no herbaria cited; no original material found, not found at KR).
Solanum nigrum L. forma chlorocarpum A.Braun ex Döll, Rhein. Flor. 413. 1843.
Type. Germany. “Bei Gerolsau und Lichtenthal unweit Baden”, *A. Braun & J.C. Döll s.n.* (no herbaria cited; no original material found, not found at KR); Germany. “Bei Carlsruhe, Knielingen”, *A. Braun s.n.* (no herbaria cited; no original material found, not found at KR); Germany. “Mannheim, Dossenheim, Oppenheim”, *J.C. Döll s.n.* (no herbaria cited; no original material found, not found at KR).
Solanum guineense (L.) Lam. var. nepalense Dunal, Prodr. [A. P. de Candolle] 13(1): 49. 1852.
Type. Cultivated in Avignon Botanic Garden, from eastern Nepal, *Herb. Requien s.n.* (lectotype, designated here: AV [Herb. E. Requien]).
Solanum pterocaulum Dunal var. deppei Dunal, Prodr. [A. P. de Candolle] 13(1): 52. 1852, as ‘pterocaulon’.
Type. Cultivated in France at Montpellier “Solanum deppei. In hortis bot. cultum” (no specimens cited, described from living plants “v.v. Hort. Monsp.”; neotype, designated here: MPU [MPU310704]).
Solanum paludosum Dunal, Prodr. [A. P. de Candolle] 13(1): 57. 1852.
Type. Myanmar “cat. itir. Burm.”, 1827, *N. Wallich 402* (holotype: G-DC [G00144525]).
Solanum roxburghii Dunal, Prodr. [A. P. de Candolle] 13(1): 57. 1852.
Type. “In India orientali” (no specimens cited; lectotype, designated here: Wight, Icones plantarum Indiae Orientalis 2: t. 344. 1843) “Peninsula Ind. orientalis”, *R. Wight s.n. [herb. Wight 2326*] (epitype, designated here: E [E00718973]).
Solanum memphiticum Mart. var. repandum Dunal, Prodr. [A. P. de Candolle] 13(1): 47. 1852.
Type. Germany. Saxony: cultivated in Leipzig [fide JE where original set is stored] “colui”, 1824, *W. Gerhard s.n.* (holotype: G [G00144263]; isotypes: JE [JE00009838], W [1889-0232903, 1889-0283857]).
Solanum nigrum L. var. atriplicifolium Desp. ex Dunal, Prodr. [A. P. de Candolle] 13(1): 50. 1852, nom. illeg., Solanum nigrum L. var. atriplicifolium G.Mey. (1836).
Type. Based on same original material (specimen) as Solanum nigrum L. var. atriplicifolium G.Mey.
Solanum cechicum Opiz, Lotos 4: 94. 1854.
Type. Czech Republic. “Um Prag”, *P.M. Opiz s.n.* (no herbaria cited; no original material found, perhaps at PR?).
Solanum nigrum L. forma judaicum Miq., Fl. Ned. Ind. 2: 637. 1856.
Type. Indonesia. Java: [West Java] Buitenzorg [Bogor], *C.L. Blume s.n.* (lectotype, designated here: L [L2880952]).
Solanum nigrum L. forma paludosum (Dunal) Miq., Fl. Ned. Ind. 2: 637. 1856.
Type. Based on Solanum paludosum Dunal
Solanum hirsutum Kit. ex Kanitz, Linnaea 32: 440. 1863, nom. illeg., not Solanum hirsutum (Vahl) Dunal (1813).
Type. Slovakia. “ex itinere Arvensi”[Árva], *P. Kitaibel s.n.* (lectotype, designated here: BP [Herb. Kit. fasc. IX No. 103]).
Solanum acutifolium Kit. ex Kanitz, Linnaea 32: 440. 1863, nom. illeg., not Solanum acutifolium Ruiz & Pav. (1799).
Type. Slovakia. “ex itinere Arvensi”[Árva], *P. Kitaibel s.n.* (lectotype, designated here: BP [Herb. Kit. fasc. IX No. 103]).
Solanum nigrum L. var. macrocarpum Schur, Enum. Pl. Transsilv. 478. 1866.
Type. Romania. Sibiu: Sibiu “In ruderalis prope Cibinium Transilv.” [protologue “Schur sert. n. 1994, auf unbebauten Orten bei Hermannstadt, Aug.”], Oct, *F. Schur s.n.* (neotype, designated here: LW [LW00210121]; no duplicates found at BRNU).
Solanum nigrum L. var. chlorocarpum (Spenn.) Schur, Enum. Pl. Transsilv. 478. 1866.
Type. Based on Solanum vulgatum Spenn. var. chlorocarpum Spenn.
Solanum nigrum L. var. glabrum Lowe, Man. Fl. Madeira 2: 73. 1872.
Type. Portugal. Madeira: Mr. Gordon’s kitchen garden, the Mt., 8 Dec 1932, *R.T. Lowe 16* [a] (lectotype, designated by Edmonds 2012, pg. 127: BM [BM000943025]).
Solanum nigrum L. var. hebecaulon Lowe, Man. Fl. Madeira 2: 73. 1872.
Type. Portugal. Madeira: [“Levada de Sta. Luzia, above Funchal, Feb”, protologue], *R.T. Lowe [?] s.n.* (no specimens corresponding to this locality and date located).
Solanum morella Desv. subsp. nigrum (L.) Rouy, Fl. France 10: 364. 1908.
Type. Based on Solanum nigrum L.
Solanum ganchouenense H.Lév., Repert. Spec. Nov. Regni Veg. 11: 295. 1912.
Type. China. Guizhou: Gan Chouen, Aug 1910, *J. Cavalérie 3815* (holotype: E [E00284474]; isotypes: E [E00284475], K [K001080605], P [P00055234]).
Solanum chenopodiifolium H.Lév., Repert. Spec. Nov. Regni Veg. 12: 531. 1913.
Type. China. Yunnan: Tong-Tchouan plaine, Sep 1912, *E.E. Maire s.n.* (holotype: E [E00284477]).
Solanum nigrum L. subvar. atriplicifolium (Desp. ex Dunal) Schinz & Thell., Fl. Schweiz, ed. 3, 2: 295. 1914.
Type. Based on Solanum nigrum L. var. atriplicifolium Desp. ex Dunal (=Solanum nigrum L. var. atriplicifolium G.Mey.)
Solanum peregrinum E.P.Bicknell, Bull. Torrey Bot. Club 42: 332. 1915.
Type. United States of America. Massachusetts: Nantucket County, Nantucket street, *E.P. Bricknell 7719* (holotype: NY [00138955]; isotype: NY [00073847]).
Solanum probstianum Polg., Mitteil. Naturfor. Gesellsch. Solothurn 12: 30. 1938.
Type. Cultivated in Hungary at Györ, from seeds sent by R. Probst from Switzerland (Solothurn: Derendingen [Kammgarnfabrik Derendingen] in 1932), 23 Aug 1933, *S. Polgár s.n. [Herb. Polg. 4051*] (no specimens cited; lectotype, designated here: BP [BP-272406]).
Solanum nigrum L. var. schultesii (Opiz) Rouy, Acta Horti Gothob. 10: 201. 1935.
Type. Based on Solanum schultesii Opiz
Solanum pseudoflavum Pojark., Bot. Mater. Gerb. Inst. Komarova Akad. Nauk S.S.S.R. 17: 338. 1955.
Type. Kazakhstan. Between Vernoy Alma-Ata, Vernenskiy area, between town of Vtrnym and the station Karasukskaya, the village of Dimitrivka, *V.S. Titov 2220* (holotype: LE).
Solanum nigrum L. forma pallidum Wessely, Repert. Spec. Nov. Regni Veg. 63: 311. 1960, as “Solanum nigrum subsp. nigrum var. atriplicifolium forma pallidum”.
Type. Germany. Rhineland-Palatinate: Neuwied, *Wirtgen s.n.* (holotype: W [not seen]).
Solanum nigrum L. forma luridum Wessely, Repert. Spec. Nov. Regni Veg. 63: 311. 1960, as “Solanum nigrum subsp. schultesii forma luridum”.
Type. Germany. Saxony: Dresden, Trümmerstellen am Postplatz, 22 Sep 1957, *I. Wessely 0.23* (holotype: GFW).
Solanum nigrum L. subsp. schultesii (Opiz) Wessely, Feddes Repert. Spec. Nov. Regni Veg. 63: 311. 1960.
Type. Based on Solanum schultesii Opiz
Solanum nigrum L. var. incisum Täckh. & Boulos, Publ. Cairo Univ. Cairo Herb. 5: 101. 1974 [“1972”].
Type. Egypt. Faiyum, Sinnuris, *L. Boulos s.n.* (holotype: CAI [CAI000175]).

#### Type.

“Habitat in Orbis totius cultis” [sheet marked with Θ, meaning central part of Asia = Middle East], *Without collector* (lectotype, designated by Henderson 1974, pg. 19: LINN [LINN 248-18]).

#### Description.

Annual or short-lived erect to sprawling perennial herbs to 1.0 m tall, subwoody and branching at base. Stems spreading to decumbent, terete to sharply angled and ridged, green, the ridges often spinescent, older stems not appearing spinescent, not markedly hollow; new growth pubescent with simple, spreading, uniseriate, eglandular or glandular trichomes, these 1-6-celled, 0.5–0.6 mm long; older stems glabrescent, the trichome bases persisting as pseudospines. Sympodial units difoliate, the leaves not geminate. Leaves simple, 3.8–7.2(-14.5) cm long, 2.5–5.0(-9.5) cm wide, broadly ovate, membranous, green, concolorous, without smell or smell somewhat foetid; adaxial surface sparsely pubescent with spreading, simple, uniseriate trichomes like those on stem evenly scattered along veins and lamina; abaxial surface more densely pubescent along veins and sparsely along lamina with eglandular and/or glandular trichomes like those of the stems; major veins 5–7 pairs; base obtuse to truncate, somewhat attenuate; margins sinuate-dentate, especially in the lower 2/3, to occasionally entire or deeply toothed; apex acute; petioles 0.5–3.0 cm long, pubescent with simple uniseriate glandular and eglandular trichomes like those of the stems. Inflorescences 0.8–2.0 cm long, internodal, simple to occasionally furcate, the flowers spaced along the rhachis, with (3-)4–10 flowers, pubescent with spreading simple uniseriate trichomes like those on stem; peduncle 0.5–1.5 cm long, straight; pedicels 3–5 mm long, 0.2–0.3 mm in diameter at the base and 0.2–0.3 mm at the apex, spreading, articulated at the base; pedicel scars spaced 0.3–0.7 mm apart. Buds subglobose, the corolla approximately halfway exserted from the calyx before anthesis. Flowers 5-merous, all perfect. Calyx tube 0.8–1.0 mm long, conical, the lobes 0.5–0.8 mm long, 0.6–0.8 mm wide, triangular with acute or somewhat rounded apices, pubescent with spreading simple uniseriate eglandular and glandular trichomes like those of the pedicels. Corolla 10–12 mm in diameter, white with a yellow-green central portion near the base, stellate, lobed 1/2 to 2/3 of the way to the base, the lobes 4.0–5.0 mm long, 2.0–2.5 mm wide, strongly reflexed at anthesis, later spreading, densely papillate-pubescent abaxially with simple uniseriate eglandular trichomes. Stamens equal; filament tube very short to minute; free portion of the filaments 0.5–0.7 mm long, adaxially pubescent with spreading uniseriate simple trichomes; anthers 1.8–2.5 mm long, 0.8–1.0 mm wide, ellipsoid, very slightly wider at base, yellow, poricidal at the tips, the pores lengthening to slits with age and drying. Ovary globose, glabrous; style 2.5–3.5 mm long, densely pubescent with tangled 2-3-celled simple uniseriate trichomes in the lower half where included in the anther cone, exserted 0–1 mm beyond anther cone; stigma capitate, minutely papillate, green in live plants. Fruit a globose berry, 6–10 mm in diameter, purple-black or green to yellowish-green at maturity, the pericarp dull or slightly shiny; fruiting pedicels 10–12 mm long, 0.4–0.5 mm in diameter at the base and 1.0–1.1 mm at apex, generally spreading to occasionally recurved, spaced 1.0–2.0 mm apart, dropping with mature fruits, not persistent but occasionally remaining on the inflorescence; fruiting calyx not accrescent, the tube ca. 1 mm long, the lobes 1.0–2.0 mm long, spreading to reflexed in fruit. Seeds (15-)20–40 per berry, 1.8–2.0 mm long, 1.5–1.6 mm wide, flattened and tear-drop shaped with a subapical hilum, yellow, the surfaces minutely pitted, the testal cells pentagonal in outline. Stone cells absent (North America and Europe) but usually 2(-8) per berry in other areas (Asia), ca. 0.5 mm in diameter, brown. Chromosome number: *2n*=6x=72 (Jørgensen 1928; Tokunaga 1933; Ellison 1936; Nakamura 1937; Stebbins and Paddock 1949; Baylis 1958; Heiser et al. 1965; Saarisalo-Taubert 1967; Venkateswarlu and Rao 1972 [as *S. nigrum* S8, S11, S19]; Henderson 1974; Tandon 1974; Randell and Symon 1976; Edmonds 1977, 1981, 1982, 1983, 1984a; Ganapathi and Rao 1986a; Symon 1981; Bukenya 1996).

**Figure 22.**
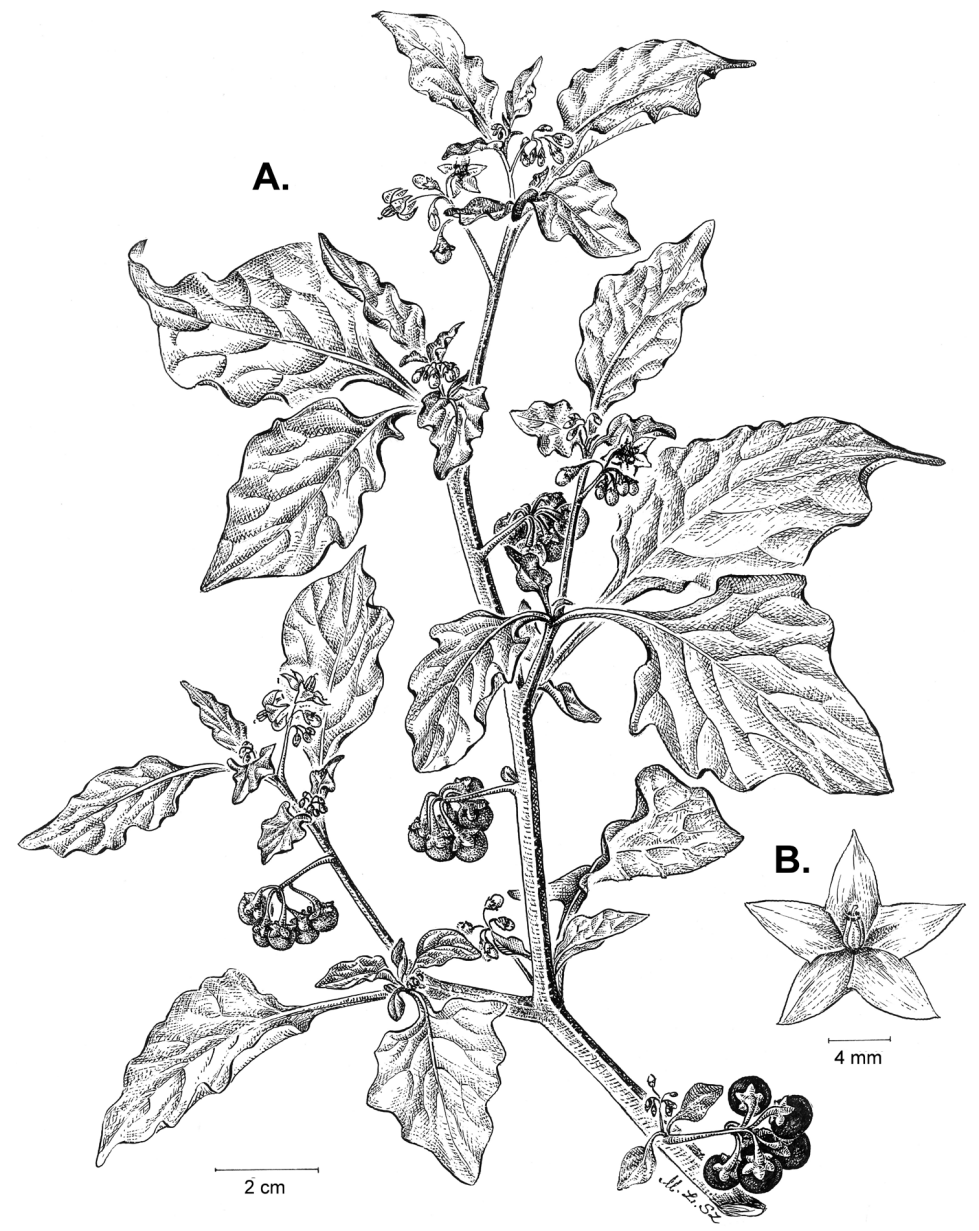
*Solanum nigrum* L. **A** Habit **B** Flower (**A, B** *Symon 5449* [ADW 35964]). Drawing by M.L. Szent-Ivany, first published in Symon (1981), courtesy of the Board of the Botanic Gardens and State Herbarium (Adelaide, South Australia), reproduced with permission.

**Figure 23.**
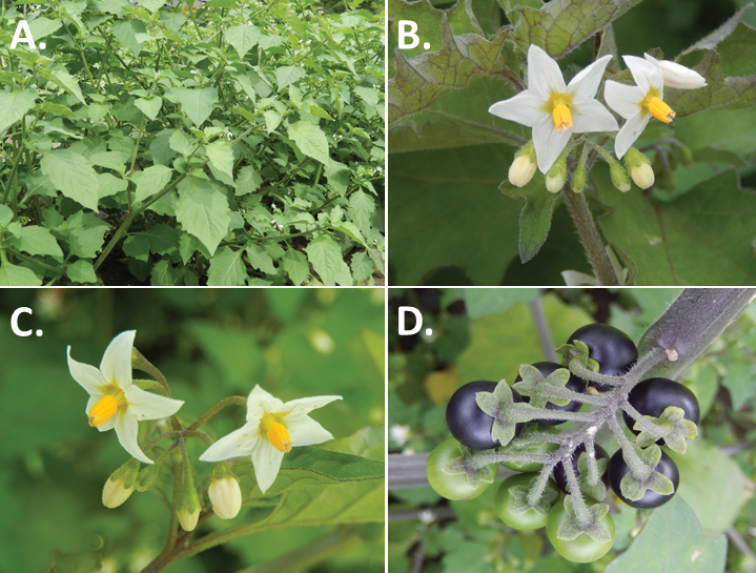
*Solanum nigrum* L. **A** Habit **B** Inflorescence (dense indumentum) **C** Inflorescence (sparse indumentum) **D** Fully mature full-black berries, calyx lobes remaining appressed or slightly spreading (**A** Nijmegen acc. 824750016; **B** Nijmegen acc. A34750479; **C** Nijmegen acc. 824750029A, **D** Nijmegen acc. A44750150). Photos by S. Knapp.

#### Distribution

**Figure 24.**
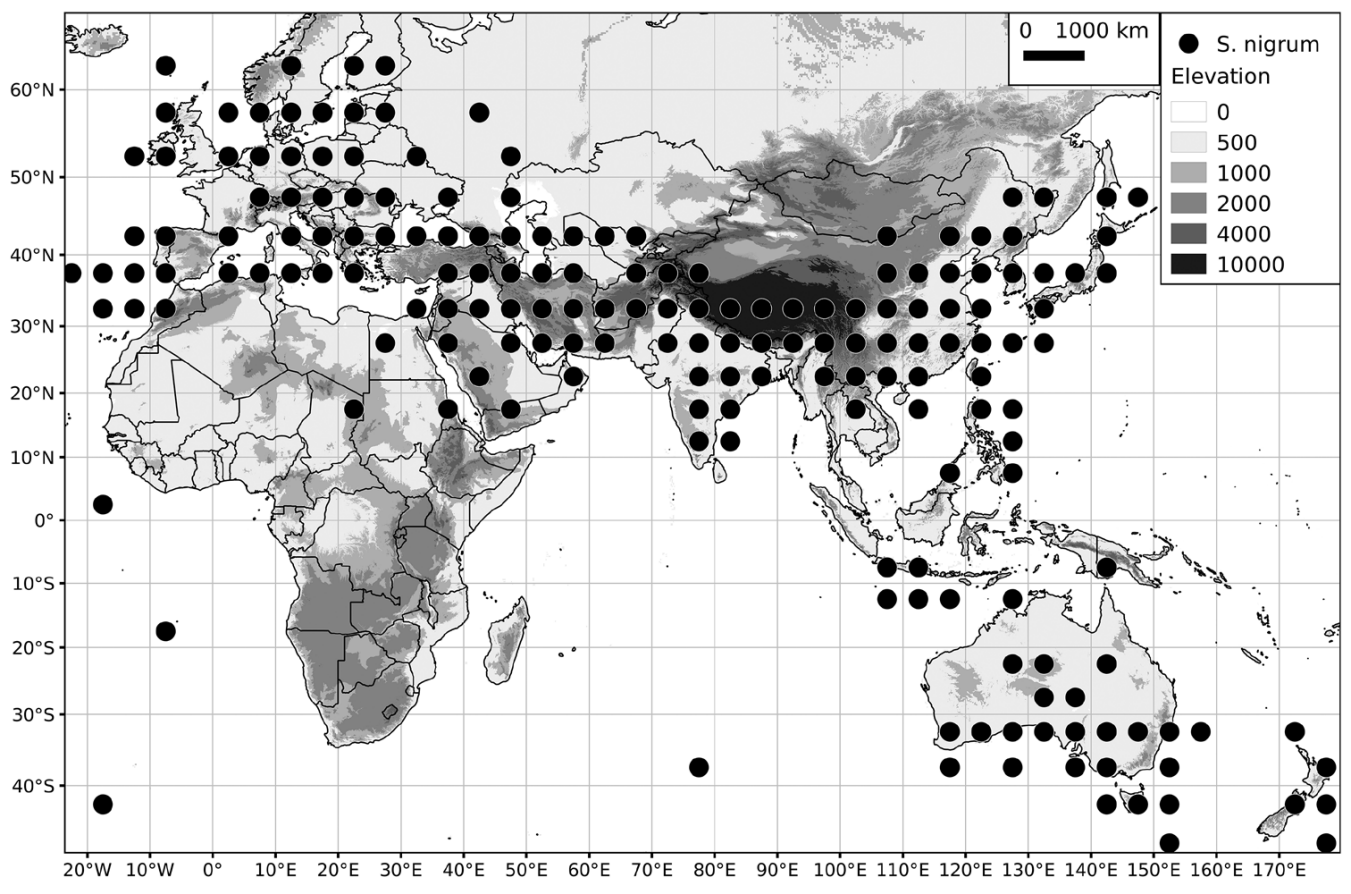
Distribution of *Solanum nigrum* L in the Old World.

(Figure 24). Widespread species native to Eurasia (western Europe to Japan), northern Africa and Australia, sporadically introduced in South Africa and naturalised locally in temperate North America.

#### Ecology.

Weed of cultivated land, open spaces in forests and roadsides; found in disturbed areas between 0–2,200 (3,500) m elevation.

#### Common names.

Australia: blackberry, black berry nightshade (Symon 1981); China: paak fa tsai ts’o [Hainan], long kui (Zhang et al. 1994); Denmark: kirtel-natskygge, sort natskygge (Hartvig 2015), sort natskygge (Frederiksen et al. 2006); Egypt: anabadib [Berber], aneb el dib, enab athib; Estonia: must maavits (Kukk and Kull 2005; Finland: mustakoiso, karvamustakoiso (Hämet-Ahti et al. 1998); France: morelle noire (Jauzein and Nawrot 2011); Germany: schwarzer Nachtschatten; India: makoi [Hindi], kakamachi [Bengali, Sanskrit], kakahva [Sanskrit]; Iraq: dubbais, habbat seda; Italy: morella commune, erba morella, pomidorella, ballerina (Pignatti 1982), solano nero; Japan: inuhozuki, inn-hodzuki, kanzashi-inuhoozuki; Libya: aneb el dib/aneb el dhib; Lithuania: juodoji kiauliauogė (Natkevičaitė-Ivanauskienė 1976); Malaysia: beliwan; Nepal: kamai; New Zealand: black nightshade (Webb et al. 1988); Norway: svartsøtvier (Lid et al. 2005); Pakistan: tolangur; Philippines: amti, amtih (Ifugao language), moti; Poland: psianka czarna (Pawłowskiego 1963); Portugal: herva moira morelle commune, pera de Santa Maria; Saint Helena: Tristan blackberry; Slovenia: lulok čierny (Bertová and Goliašová 1993); Spain: yerba mora, hierba mora; Sweden: nattskatta, hårig nattskatta (Mossberg et al. 2003); United Kingdom: black nightshade

#### Uses.

Leaves used as vegetable in India, China, southeast Asia and in Europe (in local areas); thought to be poisonous by association with the deadly nightshade, *Atropa belladonna* (see Uses in introductory section). Berries sometimes used for jam (Viljoen 2011).

#### Preliminary conservation status

(IUCN 2016). *Solanum nigrum* is a widely distributed amphitropical species across temperate and subtropical areas in the Old World; it can be assigned a status of LC (Least Concern; Table 7).

#### Discussion.

*Solanum nigrum*, the type species of the genus *Solanum*, is a widespread weed with much morphological variation recognised at various infraspecific levels by many different authors (e.g. Linnaeus 1753; Opiz 1843). There are almost 100 names associated with the taxon and the status of some of these remains uncertain due to difficulty in finding types but we include them here based on their descriptions (but see Doubtful species). Here, we adopt an inclusive and broad concept of the species and recognise all infraspecific taxa under a single, more widespread and morphologically variable species. Two of the most commonly recognised infraspecific units include S. nigrum subsp. schultesii (Opiz) Wessely (villous, with glandular hairs) and S. nigrum subsp. nigrum (eglandular). Previous studies have found no support for the recognition of these or any other infraspecific taxa within *S. nigrum* based on morphological (Henderson 1974), seed protein (Edmonds and Glidewell 1977) or molecular marker data (Dehmer 2001; Dehmer and Hammer 2004; Olet 2004; Manoko 2007).

We do not include material identified as *S. nigrum* in recent African regional floras (Edmonds 2006a, 2006b, 2012) in our circumscription of this species. We follow Olet (2004) and Olet et al. (2011) and consider this material to represent the wild form of *S. scabrum.* In Africa, we consider only material from northern Africa around the Mediterranean and a few specimens (probably introduced from Europe) from South Africa to be true *S. nigrum*. Both *S. nigrum* and *S. scabrum* are hexaploids and morphologically similar, but can be distinguished on the following suite of morphological characteristics; *Solanum nigrum* has leaves with rather indistinct petioles, inflorescences with the flowers spaced along the rhachis, acute calyx lobes that are more or less appressed to the berry in fruit, and berries that not markedly shiny and often have stone cells (particularly in Asia). *Solanum scabrum* has distinctly petiolate leaves, the flowers are usually tightly congested at the tips of inflorescences or inflorescence branches, the calyx lobes are rounded, irregular and strongly reflexed in fruit and the berries are shiny with thick pericarp and lack stone cells. In addition, the fruiting pedicels of *S. nigrum* are spreading to somewhat recurved, while those of *S. scabrum* are erect and strongly spreading.

Throughout its range in Europe and into Eurasia as far as western China, *S. nigrum* is sympatric with *S. villosum*. The simplest distinguishing character is mature berry colour; *S. villosum* has red, orange or yellow berries, while those of *S. nigrum* are black or green. Many old collections, however, do not state berry colour on the label, so identification can be difficult. Calyx lobes are useful for distinguishing these taxa; *S. nigrum* calyx lobes are usually deltate and acute, with sharp triangular sinuses, while those of *S. villosum* are longer, usually rounded at the tip and the sinuses are broad and quite transparent (see description of *S. villosum*), leaving a paler “window” just below the sinus in flower buds and early flowers. The shiny, translucent berries of *S. villosum* (mostly slightly ellipsoid) usually dry blackish-brown, but are distinct from the matte, more opaque berries of S. *nigrum*. Neither species has stone cells in Europe, but in Asia, *S. nigrum* usually has 2 (or occasionally more) stone cells in the berries. Both species retain pedicels after fruits drop, but *S. nigrum* is not as extreme in this regard and often plants are found with old inflorescences with no remaining pedicels.

*Solanum nigrum* has been considered native only to Europe, but our study of populations of this widespread weed across its range has shown that the largest morphological variation can be observed in Asia. Populations of *S. nigrum* from Asia have more stone cells in the fruit and the plants have a more delicate look overall with longer peduncles and often fewer flowers per inflorescence. Material from Asia has been described as different taxa (e.g. *S. guanchounense*, *S. chenopodiifolium*) but the variation is continuous across the range, with European populations being more invariant (except in leaf shape and indumentum). Populations in Europe represent a relatively monomorphic set of populations compared to material from China and eastern Asia. We therefore consider that the species is native to the entire Eurasian area, with limited introductions to North America. North American material we have seen appears most similar to European plants based on morphology, suggesting the introduction to North America came from European populations, but this has not been tested genetically.

Australian populations of *S. nigrum* could have originated either from Europe or from Asia. In general, *S. nigrum* in Australia does not seem to have stone cells in general and plants fall into two continuously variable groups: one is similar to classic European *S. nigrum* and the other is more similar to plants from eastern and south-eastern Asia.

*Solanum nigrum* is an autoalloploid species, now thought to have originated from a tetraploid *S. villosum* and a diploid *S. americanum* by spontaneous amphiploidy (Edmonds 1979a; Ganapathi and Rao 1986b, 1986c; Poczai and Hyvönen 2011). Cytogenetic and crossing studies show high fertility and regular pairing of bivalents at meiosis in crosses of *S. villosum*, *S. retroflexum* and *S. americanum* with *S. nigrum* (Edmonds 1979a; Ganapathi and Rao 1986b, 1986c, 1986d). Of the two tetraploid species, *S. villosum* and *S. nigrum* are sympatric at least across parts of their native ranges which would make it more likely that *S. nigrum* is an autoalloploid derived from the autopolyploid *S. villosum* and the diploid *S. americanum*. Molecular analyses have now shown that the two hexaploids *S. nigrum* and *S. scabrum* share a parental species with the tetraploid *S. villosum* (Poczai and Hyvönen 2011). Artificially produced hexaploids from tetraploid crosses of *S. villosum* and *S. americanum* have been shown to be fertile and represent the glandular variant of *S. nigrum* (sometimes recognised as subsp. schultesii (Opiz) Wessely; Edmonds 1979a). It is hence becoming more clear that the tetraploid parent of *S. nigrum* is *S. villosum* and not *S. retroflexum*. Artificial hexaploids have been produced using *S. villosum* and *S. americanum* by several authors and the artificial hexaploids resemble *S. nigrum* morphologically (Tandon and Rao 1964; Venkateswarlu and Rao 1969, 1972; Soria and Heiser 1959; Edmonds 1979a). Reciprocal backcrossing of the artificial hexaploids with *S. nigrum* results in a high fruit set (Venkateswarlu and Rao 1969, 1972; Edmonds 1979a), supporting the hypothesis that *S. villosum* and *S. americanum* are the parent species of *S. nigrum*. The autoallopolyploid origin of *S. nigrum* has been suggested by previous authors (Magoon et al. 1962; Rao 1971). In the light of the current molecular evidence combined with the accumulating evidence from cytological and crossing studies, the alternative hypotheses on the origin of *S. nigrum* as an autohexaploid (Jørgensen 1928; Nakamura 1935, 1937) and an allopolyploid derived from three distinct genomes (e.g. Tandon and Rao 1964, 1966a; Henderson 1974; Edmonds 1979a) are becoming less likely.

In parts of eastern England, S. × procurrens A.C.Leslie (Leslie 1978), a sterile tetraploid hybrid between *S. nitidibaccatum* and *S. nigrum*, is locally established where the two species grown together (Stace 2010: 531; Stace et al. 2015). The hybrid has also been recorded in New Zealand (Webb et al. 1988). These plants are intermediate between the two species, with glandular pubescence and black berries with somewhat accrescent calyx lobes. A good description and distribution map for this local hybrid can be found in Stace et al. (2015). We have not seen convincing material from elsewhere in Europe of this hybrid, but it potentially occurs wherever *S. nitidibaccatum* and *S. nigrum* co-occur. As it is sterile, however, it does not spread or persist.

Linnaeus (1753) recognised six varieties of his *S. nigrum*, of which four are now considered distinct species (var. patulum = S. americanum; var. villosum = S. villosum; var. guineense = S. scabrum; var. virginicum = *S. emulans* Raf.). Henderson (1974) lectotypified *S. nigrum* with the single sheet in the Linnean herbarium (LINN 248.28) corresponding to this species. In the protologue, Linnaeus (1753) cited several other elements and it is from these that we select the lectotype for var. vulgare while, for var. judiacum, he cites no elements. It is possible that a specimen from his herbarium was the basis for this infraspecific epithet and that the sheet in LINN is that referred to by this name.

In describing *S. judaicum*, von Besser (1809) did not specifically cite Linnaeus’s varietal name, so we have assumed he was coining a new name, rather than making a combination.

The name *S. cestrifolium* has a complex history; we can find no place of publication for “Solanum cestrifolium Jacq.” as cited by Sprengel (1825) and Weinmann (1824:100; in synonymy with his nomen nudum *S. besserianum*, see Doubtful names under *S. besseri* Weinm. ex Roem. & Schult.). The name *S. cestrifolium* has also been attributed to Willdenow by Gussone (1825, as a *nomen nudum*) and subsequently validated by Colla (1835); this epithet refers to plants now considered part of the species *S. bahamense* L. (Strickland-Constable et al. 2008). We have not lectotypified *S. cestrifolium* Jacq. ex Spreng. in the hope that material amongst the cultivated holdings at W will reveal potential original material.

Blume (1826) cited no specimens for any of the names in his *Bijdragen tot de flora van Nederlandsch Indië* nor do many of the descriptions have specific localities. We have found a specimen labelled *S. rhinozerothis* in Blume’s hand in L (L.2883159) that we here select as the neotype for that species, but have not found any material directly attributable to Blume that we could associate with *S. uliginosum* (see under Doubtful names).

The varietal epithet “*atriplicifolium*” was used by many authors to refer to plants of *S. nigrum* with dentate leaf margins. It appears to have been used in reference to a specimen that was annotated “Solanum atriplicifolium” by Narcisse Desportes, probably seen in Paris or Geneva. Georg Meyer of Hannover (1818) was the first botanist to effectively and validly publish this epithet and did so at the varietal rank. Dunal (1852) cited the same material and said that the collector was “Prost”, though the G herbarium catalogue states the collector as Desportes. We select this G-DC (G00144334) sheet as the lectotype for S. nigrum var. atriplicifolium because it appears to be original material by way of locality and annotation. The combinations made by Don (1837) and Dunal (1852) are isonyms and therefore have no standing, but we include them here because they have been widely used and cited and other combinations have been made based upon them.

The various names coined by the amateur Czech botanist Philipp Maximilian Opiz and here recognised as synonyms of *S. nigrum* were all included by him in the “superspecies” *S. nigrum* and, from descriptions and key, represent leaf shape, pubescence and inflorescence size variations of that species. Opiz’s enormous herbarium is housed mostly in PR (Kirschner et al. 2007) with duplicates in PRC and other major European herbaria. We have been unable to visit Prague to examine the no doubt extensive gatherings of *S. nigrum* and have found few unambiguous duplicates of the collections cited in the protologues. We therefore leave the typification of these infraspecific names until further study of Opiz’s herbarium can be undertaken. The same is true for the infraspecific names coined by Johann Döll (1843), whose protologues cited some presumed collections, but no herbaria. Searches at KR reveal no original material and the A. Braun collections cited were probably at B and were destroyed. Again, these infraspecies refer to the highly variable populations of *S. nigrum* in central Europe. Döll (1843) attributed the infraspecific epithets var. chlorocarpum and *stenopetalum* to Braun; he was thus coining a new name “chlorocarpum” and not citing Spenner’s earlier use of this epithet.

*Solanum chenopodium*, *S. exarmatum* and *S. bidentatum* were all coined by Rafinesque (1840) for European plants. He cited “S. nigrum var. undatum”, a name never published, in the protologue of *S. chenopodium* and suggested that *S. bidentatum* was the same as “*S. patulum* of India”. We here place these three in the synonymy of *S. nigrum* based on their rather meagre descriptions; Rafinesque’s herbarium was destroyed, but occasionally fragments he sent to European herbaria survive (e.g. material relating to *S. emulans* Raf.)

Dunal (1852) described several varieties of his complex taxon *S. pterocaulum* (in 1852 written as *pterocaulon*); most of these correspond to S. scabrum. His var. deppei was described from living material “v.v. Hort. Monsp.”; a sheet in MPU (MPU310704) labelled “Solanum deppei. In hortis bot. cultum” is here selected as neotype for this name.

A single collection of a plant from Nepal cultivated in Avignon was cited in the protologue of S. guineense var. nepalense (Dunal 1852), housed in “h. Requien”. A sheet at AV clearly labelled as “S. guineense β nepalense Prodr.” is most likely original material for the name and we designate this as the lectotype.

*Solanum roxburghii* was coined by Dunal (1852) as a replacement for “S. rubrum” as used by Roxburgh (1824) in his *Flora Indica*. Dunal cited the publications of Roxburgh (1824, but with the incorrect page number of 216 rather than 246) and Nees van Esenbeck (1837) and an illustration by Wight, but cited no herbarium material. We consider the illustration in Wight (1843) as the only unambiguous original material for this name and designate it as the lectotype for *S. roxburghii*. Both the citations of “Solanum rubrum” are based on the epithet of Miller and/or Linnaeus and are not at all clear. Differentiating *S. nigrum* from *S. villosum* (both which occur in India) can be difficult without ripe berries and, since berry colour in the original hand-coloured illustration at E is green, we are certain the Wight illustration depicts a plant of *S. nigrum*, with flowers spaced along the inflorescence and sepals that are not reflexed in fruit. We therefore designate an epitype from amongst the sheets collected by Wight (E00718973) to fix the application of this name. Early authors, such as Roxburgh and Nees van Esenbeck, included both red/orange and black-fruited plants in their circumscriptions of both *S. nigrum* and *S. rubrum*. The sheets in the East India Company herbarium at K (see de Candolle and Radcliffe-Smith 1981; Noltie 2005) are a complex and confusing mixture of *S. americanum* and *S. villosum*; none of these specimens matches the illustration as well as does the E sheet we select here as the epitype. This again illustrates the difficulty that early authors had with these very similar plants and the dangers of assuming that collections are duplicates.

Miquel (1857) recognised several forms of *S. nigrum* in his broad species circumscription. His forma judiacum was specifically coined to encompass *S. judaicum* Besser in the sense of Blume (1826), but excluding Besser’s material. We therefore consider this the coining of a new name and not a new combination based on Besser’s *S. judaicum*; the lectotype selected is a sheet in L (L2880952) with the locality “in uliginosis circa Buitenzorg” as cited by Blume and in Blume’s hand.

The names coined by the Hungarian botanist Pál Kitaibel were published posthumously by Kanitz (1863) and are largely illegitimate (some were used in earlier publications by Schultes, see discussion under *S. villosum*). *Solanum hirsutum* and *S. acutifolium* were published as alternative names “Solanum hirsutum vel acutifolium” and therefore have the same type; we have selected the sheet in the Kitaibel herbarium at BP with the locality matching that in the protologue (“ex itinere Arvensi”, BP [Herb. Kit. fasc. IX: 103]).

We have selected a specimen collected by Schur (LW00210121), but with a different date of collection and exact locality, as the neotype for S. nigrum var. macrocarpum; searches in the other relevant herbaria revealed no original material.

Edmonds (2012) only lectotypified one of the varieties of *S. nigrum* from Lowe’s *Manual Flora of Madeira* (1872). Lowe’s var. hebecaulon cited a single locality and date “Levada de Sta. Luzia, above Funchal, Feb”; no specimens corresponding exactly to that are found in BM or at K.

*Solanum probstianum* was described from material cultivated in Hungary from seeds sent by Rudolf Probst to Sandor Polgár in 1932. Although no specific specimens were cited in Polgár’s protologue (in Probst 1938), there are many specimens annotated “Solanum probstianum” in Polgar’s hand at BP that are clearly cultivated and appear to be from this original seed collection. We have selected one of these with both flowers and fruits as the lectotype of *S. probstianum* [BP-272406], it bears a collecting number (4051) and was collected in July 1933. We do not consider the other sheets with this number as necessarily duplicates; the sheets do not have consistent collection numbers and may represent individual plants from the cultivated population collected on different dates in the same or different years.

#### Selected specimens examined.

A total of 1,537 specimens were examined from 82 countries during the study across Africa, Asia, Australia, Eurasia and the Pacific. Adventive specimens from the New World were also studied in order to understand the full range of morphology within the species. All specimens examined can be seen in Appendix 2 (csv format) and Appendix 3 (traditional Specimens Examined list in pdf format).

### 8. Solanum nitidibaccatum

Taxon classificationPlantaeSolanalesSolanaceae

Bitter, Repert. Spec. Nov. Regni Veg. 11: 208. 1912

Figures 25 , 26

Solanum styleanum Dunal, Prodr. [A. P. de Candolle] 13(1): 44. 1852.
Type. Chile. Sin. loc., *J. Styles s.n.* (holotype: G-DC [G00144016]).
Bosleria nevadensis A.Nelson, Proc. Biol. Soc. Washington 18(30): 175. 1905.
Type. United States of America. Nevada: Washoe County, Pyramid Lake, 9 Jun 1903, *G.H. True s.n.* (holotype: RM [RM0004387]).
Solanum nitidibaccatum Bitter forma integrifolium Blom, Acta Horti Gothob. 24: 118. 1961.
Type. Sweden. Göteborg, som ogräs i Göteborgs Botaniska Trädgård, 10 Sep 1948, *C. Blom s.n.* (lectotype, designated here: GB [GB-0146965]; isolectotype: E [E00593447]).
Solanum patagonicum C.V.Morton, Revis. Argentine Sp. Solanum 146. 1976.
Type. Chile. Región XII (Magallanes): Río Paine, 100m, 15 Jan 1931, *A. Donat 415* (holotype: BM [BM000617673]; isotypes: BA, BAF, GH [GH00077732], K, SI [SI003331, SI003332], US [00027733, acc. # 2639758]).
Solanum physalifolium Rusby var. nitidibaccatum (Bitter) Edmonds, Bot. J. Linn. Soc. 92: 27. 1986.
Type. Based on Solanum nitidibaccatum Bitter

#### Type.

Chile. Sin. loc., 1829, *E.F. Poeppig s.n.* (lectotype, designated by Edmonds 1986, pg. 27: W [W0004151]; isolectotype: F [V0073346F, acc. # 875221]).

#### Description.

Annual prostrate or spreading herbs to 20 cm tall, branching at base. Stems decumbent or ascending, terete, green, not markedly hollow; new growth densely viscid-pubescent with simple, spreading, uniseriate, translucent, glandular trichomes, these 2-8(10)-celled, 1.5–2.0 mm long, with a glandular apical cell; older stems glabrescent. Sympodial units difoliate, the leaves not geminate. Leaves simple, 2.0–5.5(-9.5) cm long, 1.5–5.0(-6.5) cm wide, ovate to broadly ovate, rarely elliptic, membranous, green, concolorous, without smell; adaxial surface sparsely pubescent with spreading 2–4-celled translucent, simple, uniseriate gland-tipped trichomes like those of the stem, these denser along the veins; abaxial surface more evenly densely pubescent on the lamina and veins; major veins 3–6 pairs, not clearly evident abaxially; base attenuate to cuneate, at times asymmetric, decurrent on the petiole; margins entire or sinuate-dentate; apex acute to obtuse; petioles 0.5–2.7(-4.5) cm long, sparsely pubescent with simple uniseriate glandular trichomes like those of the stems and leaves. Inflorescences 1.0–2.0 cm long, generally internodal but occasionally leaf-opposed, simple, the flowers spaced along the rhachis, with 4–8(-10) flowers, sparsely pubescent with spreading trichomes like those on stems and leaves; peduncle 0.6–1.3 cm long, straight; pedicels 4–12 mm long, 0.1–0.2 mm in diameter at the base and 0.2–0.4 mm at apex, straight and spreading, articulated at the base; pedicel scars spaced 0.3–1 mm apart. Buds subglobose, the corolla only slightly exserted from the calyx tube before anthesis. Flowers 5-merous, all perfect. Calyx tube 1–2 mm long, conical, the lobes 1.7–2.5 mm long, less than 1 mm wide, triangular with acute to obtuse apices, sparsely pubescent with 1–4-celled glandular trichomes like those of the pedicels. Corolla 4–6 mm in diameter, white with a yellow-green central eye with black “V” or “U” shaped margins in the lobe sinuses, rotate-stellate, lobed 1/3 of the way to the base, the lobes 2.3–3.2 mm long, 2.5–3.7 mm wide, spreading at anthesis, sparsely papillate-pubescent abaxially with 1–4-celled simple uniseriate trichomes, especially along tips and midvein. Stamens equal; filament tube minute; free portion of the filaments 1.5–2.0 mm long, adaxially sparsely pubescent with tangled uniseriate 4–6-celled simple trichomes; anthers 1.0–1.4 mm long, 0.5–0.8 mm wide, ellipsoid, yellow, poricidal at the tips, the pores lengthening to slits with age and drying. Ovary globose, glabrous; style 2.5–3.0 mm long, densely pubescent with 2–3-celled simple uniseriate trichomes in the lower half where included in the anther cone, exserted 0.2–1.0 mm beyond the anther cone; stigma capitate, minutely papillate, green in live plants. Fruit a globose berry, 4–13 mm in diameter, brownish-green and marbled with white (this not easily visible in herbarium specimens) at maturity, the surface of the pericarp usually shiny; fruiting pedicels 4–13 mm long, ca. 0.2 mm in diameter at the base, spaced 1–3 mm apart, reflexed and slightly curving, dropping with mature fruits, not persistent; fruiting calyx accrescent, becoming papery in mature fruit, the tube ca. 3 mm long, the lobes 2.5–3.5(-4) mm long, 3–4 mm wide, appressed against the berry, but the berry clearly visible. Seeds 13–24 per berry, 2.0–2.2 mm long, 1.2–1.4 mm wide, flattened and tear-drop shaped with a subapical hilum, brown, the surfaces minutely pitted, the testal cells pentagonal in outline. Stone cells usually (1-)2–3 per berry, occasionally absent, ca. 0.5 mm in diameter. Chromosome number: 2*n*=2*x*=24 (Ellison 1936; Henderson 1974; Saarisalo-Taubert 1967; Edmonds 1977, 1981, 1982, 1983, 1984a, 1986; Randell and Symon 1976; Symon 1981; Olet et al. 2015).

**Figure 25.**
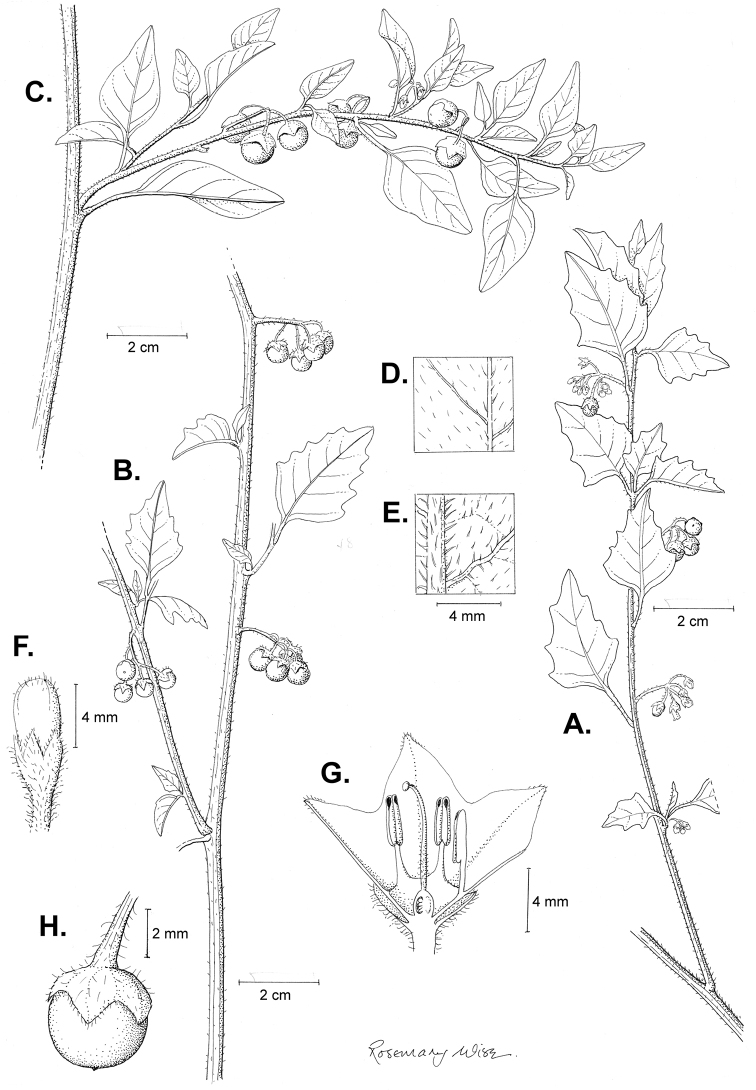
*Solanum nitidibaccatum* **A** Habit **B** Fruiting habit **C** Fruiting habit showing leaf variation **D** Detail of adaxial leaf surface **E** Detail of abaxial leaf surface **F** Bud **G** Dissected flower **H** Fruit (**A, C, F** *Henning 14*; **B, D-E, H** *Blake 186*; C *Arnow 740*). Drawing by R. Wise.

**Figure 26.**
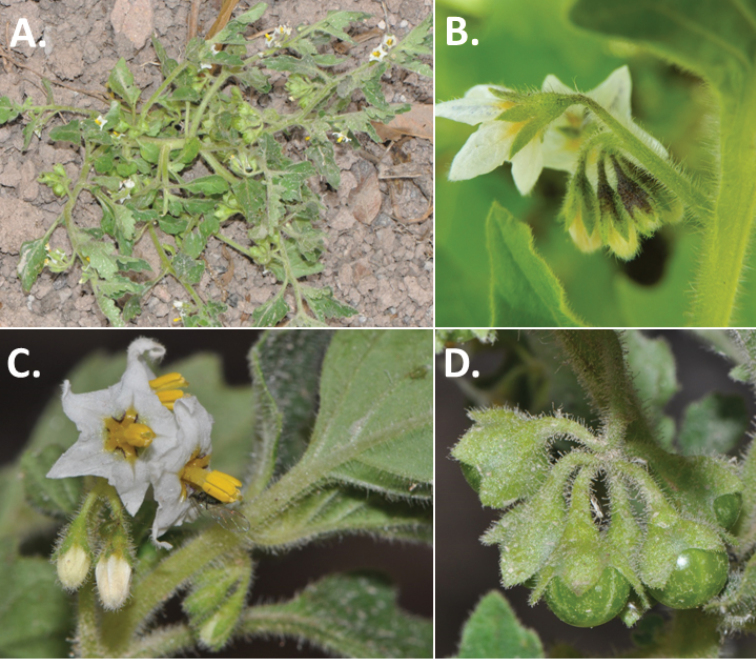
*Solanum nitidibaccatum* **A** Habit **B** Young inflorescence with flower buds **C** Flowers at anthesis **D** Maturing fruits (**A–D** *Särkinen et al. 4076*). Photos by T. Särkinen.

#### Distribution

**Figure 27.**
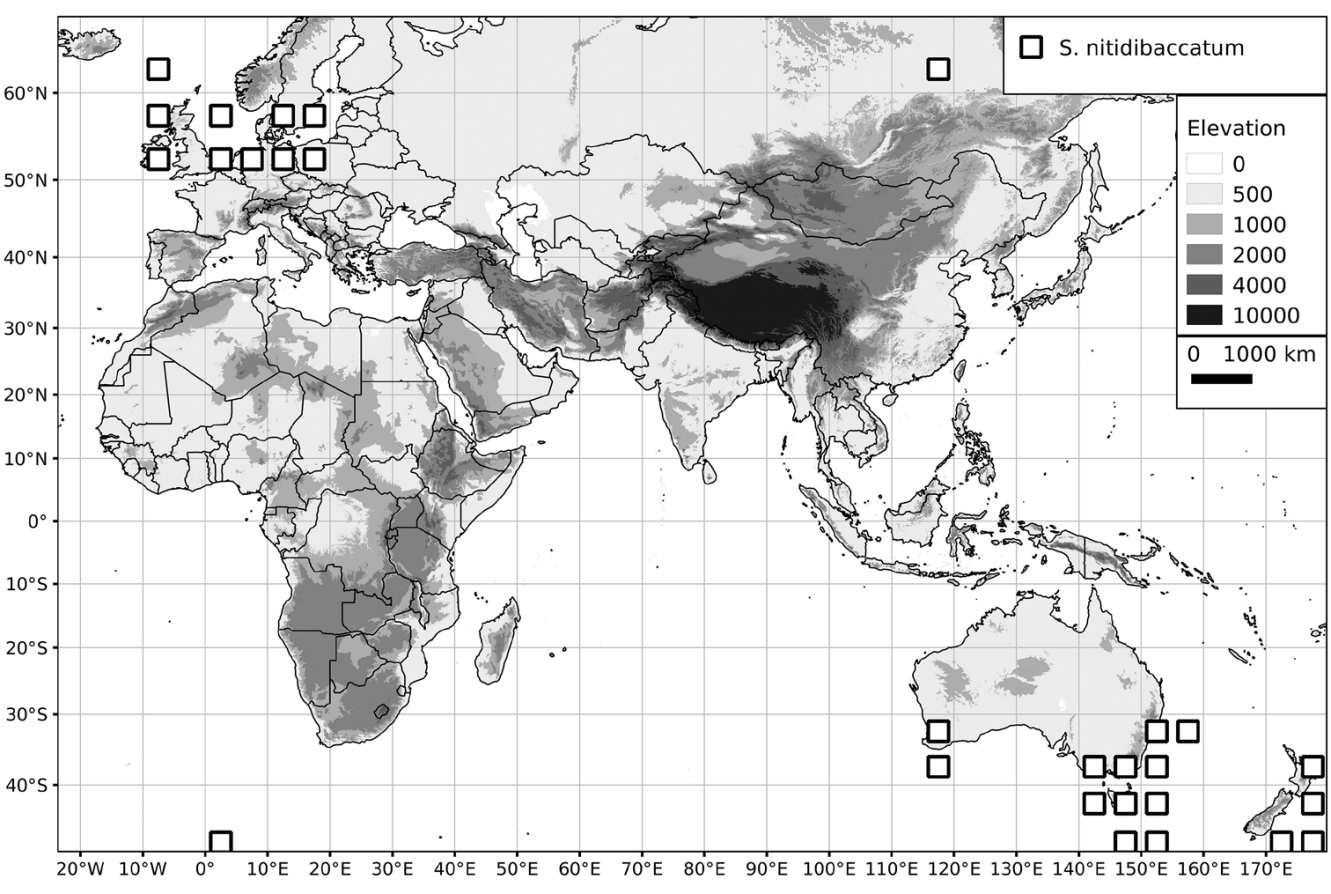
Distribution of *Solanum nitidibaccatum* in its non-native range in the Old World.

(Figure 27). Native to southern South America but sporadically to widely adventive in Europe, North America, Australia and New Zealand.

#### Ecology.

Grows along roadsides, in disturbed and cultivated areas in the shade of trees and shrubs, in rocky and sandy areas; between sea level and 2,000 (-2,700) m elevation in its native range, between sea level and 2,400 m in the Old World primarily as a weed in cultivations.

#### Common names.

Australia: cherry nightshade (Walsh and Entwisle 1999); Austria: Glanzbeeren-Nachtschatten (Fischer et al. 2005); Denmark: storbægret natskygge (Hartvig 2015); Finland: kehtokoiso (Hämet-Ahti et al. 1998); France: morelle à feuilles de coqueret (Jauzein and Nawrot 2011); New Zealand: hairy nightshade (Webb et al. 1988); Sweden: bägarnattskatta (Mossberg et al. 2003); United Kingdom: Argentinian nightshade, green nightshade (Stace 2010).

#### Uses.

None recorded; a weed of agriculture and actively controlled.

#### Preliminary conservation status

(IUCN 2016). *Solanum nitidibaccatum* is a relatively weedy species that is invasive where introduced; in its native range it is widespread and can be assigned a preliminary status of LC (Least Concern; Table 7). The EOO is relatively large even if considering only the native American range (1,591,444 km^2^) and the assessment status does not change.

#### Discussion.

*Solanum nitidibaccatum* is morphologically similar to *S. sarrachoides* and has been treated under that taxon in many previous treatments (e.g. Schilling 1981, Schilling and Heiser 1979). Edmonds (1986) clarified the distinction between the two taxa, together with a discussion of the problems surrounding its correct identification and its complex synonymy and lectotypification. *Solanum nitidibaccatum* was treated as a subspecies of *S. physalifolium* (Edmonds 1986). *Solanum physalifolium* is not known from the Old World and is endemic to South-Central Andes of Argentina, Bolivia and Peru to elevations between 2,000 and 2,900 m; distinguishing features of that species can be found in the key in Barboza et al. (2013).

*Solanum nitidibaccatum* is a diploid species native to the south-eastern parts of South America and within the Old World is morphologically most similar to *S. sarrachoides*, with which it has been confused. The two species are best distinguished by the complete inclusion of buds in calyx before anthesis, the larger stone cells in berries and the more erect habit of *S. sarrachoides* (see key in Barboza et al. 2013). *Solanum nitidibaccatum* has shorter and more ovoid anthers compared to *S. sarrachoides*, where anthers are more elongate-ellipsoid and the fruiting calyx of *S. nitidibaccatum* does not cover the berry as much as does that of *S. sarrachoides.*

*Solanum nitidibaccatum* has been introduced extensively to other parts of the world where it has become a prolific and successful weed of disturbed sites usually associated with agriculture of the wool trade. Trade with South America — particularly the importation of grain, seeds and the spreading of wool ‘shoddy’ — has been largely responsible for its introduction into Europe, with one of its common European names being the Argentinian nightshade. The taxon is now a widespread adventive in Europe where it is rapidly becoming naturalised and often forms extensive populations. It has been introduced into Australia on a number of occasions, where it persists as a weed of cultivation and is sparingly established in all States (Henderson 1974; Symon 1981). The species is locally abundant throughout North America (Ogg et al. 1981) where it is particularly widespread in the Pacific States and the West (Bohs in press). It was introduced into the South Island of New Zealand around 1968 where it rapidly became established and is now also found in northern parts of North Island (Healy 1974). It also has been sparingly introduced into equatorial regions of Africa (see Appendix 2).

In parts of eastern England, S. × procurrens A.C.Leslie (Leslie 1978), a sterile tetraploid hybrid between *S. nitidibaccatum* and *S. nigrum*, is locally established where the two species grow together (Stace 2010: 531; Stace et al. 2015). The hybrid has also been recorded in New Zealand (Webb et al. 1988). These plants are intermediate between the two species, with glandular pubescence and black berries with somewhat accrescent calyx lobes. A good description and distribution map for this local hybrid can be found in Stace et al. (2015). We have not seen convincing material from elsewhere in Europe of this hybrid, but it potentially occurs wherever *S. nitidibaccatum* and *S. nigrum* co-occur. As it is sterile, however, it does not spread or persist.

The amateur Swedish botanist Carl Blom rarely cited herbaria for his names; we have selected the sheet in GB, where he worked, as the lectotype of his var. integrifolium.

#### Selected specimens examined.

**Australia. Australian Capital Territory**: CSIRO Black Mountain site, Acton, Canberra, 9 Jan 1995, *Lepschi 1729* (AD, CANB, HO,NSW); **New South Wales**: Canyonleigh via Marulan, May 1976, *Cooper s.n.* (NSW); White Rock, 8 km upstream Bathurst, 9 May 1982, *Dellon s.n.* (NSW); **Queensland**: The Summit c. 5 miles NNE of Stanthorpe, 8 Feb 1972, *Henderson & Parham 1241* (AD, BRI, MEL, NSW); **South Australia**: Adelaide Hills, 20 Mar 1986, *Jackson 5976* (AD, CANB); Tea Tree Gully, 12 Mar 1974, *Spooner 3341* (AD); Mitcham, 18 Jan 1980, *Winn s.n.* (AD); **Tasmania**: Murphy’s Flat Reserve, 2010 (Bush Blitz), 25 Mar 2010, *Baker 2203* (HO); 12 Jan 2001, *Buchanan 15827* (HO); **Victoria**: Australian Alps, 19 Jan 1981, *Beauglehole 68690* (MEL); Melbourne; Ripponlea Estate, Hotham Street, Elsternwick, 6 Mar 1984, *Clarke 1682* (NSW); Milne’s property, 27 Jan 1966, *Shepherd 215* (CANB); Riverina, Jan 1975, *Without Collector s.n.* (MEL); **Western Australia**: Badgerup Road opposite Jambaris Road, Wanneroo, 15 May 1997, *Burt s.n.* (PERTH); Pinjarra, 17 Nov 1987, *Sheldow s.n.* (PERTH).

**Austria**. **Burgenland**: Nordburgenland, WSW von Eisenstadt, S von Müllendorf, 2 Aug 2007, *Barta s.n.* (BM); Seewinkel, zw. Apetion u Frauernkirchen, knapp W v E-Teil d. Fuchslochlacke, W v Feldweg, 12 Oct 2008, *Walter 7085* (W); **Nieder-Österreich**: Marchfeld, nahe Gänserndorf, Ackerrand neben der Strasse 1.5–1.7 km W der Kirche von Weikendorf, 28 Sep 2010, *Barta s.n.* (BM); Wiener Becken, Steinfeld, 7.5 km NNE Wiener Neustadt, 1.7 km NW Eggendorf, knapp WNW, 19 Sep 1998, *Walter 4189* (K, W); **Wien**: 10 Bezirk, in Kurpark Oberlau, 22 Oct 2012, *Barta 1219* (W); 22 Bezirk, knapp S der Ostbahngleitstrasse nahe der U2-Haltestelle Aspern-Nord, 6 Aug 2014, *Barta 3270* (W).

**Belgium. Wallonia**: Vesdre, Sep 1958, *Lousley s.n.* (K).

**France**. **Grand Est**: Bas-Rhin, Strasbourg, Graffenstaden, Oct 1961, *Patzak s.n.* (W); **Nouvelle Aquitaine**: Gironde, Bordeaux, Bassens, 24 Jul 1927, *Duffour 5538* (BM, MA); Gironde, Bassens, 9 Sep 1931, *Jallu 1038* (H, P).

**Germany**. **Hessen**: Frankfurt am Main, Frankfurt-am-Main, Hessen-Nassau, Sep 1911, *Peipers s.n.* (BM); **Niedersachsen**: Leer, Ostfriesl, Blamgelände, 10 Oct 1955, *Klimmek s.n.* (W).

**Ireland**. **Leinster**: Kilkenny, Rosbercon Port, 5 Oct 1995, *Reynolds s.n.* (BM),

**Netherlands**. **Gelderland**: Nijmegen, prov. Gelderland, 23 Sep 1928, *Kern* & *Reichgelt 14998* (BM, H).

**New Zealand**. **North Island**: Waikato, Pukekawa, 18 Dec 1986, *Dawes s.n.* (AK); Auckland, Arikikapakapa Golf Course, Rotorua Ecological District, Northern Volcanic Plateau Ecological Region, 15 Mar 2013, *Hobbs 13297* (AK). **South Island**: Canterbury, Christchurch, waste land behind Fendalton Mall buildings, Fendalton, 2 Feb 1999, *Healy 99/ 34* (AK, CHR).

**Sweden**. Prov. Halland, 26 Sep 1923, *Jungner s.n.* (BM); Haisenborg, vid fabrikenb Kärnan, 6 Oct 1938, *Lange s.n.* (BM); Skåne, Saxtorp, Flygeltofta, 28 Aug 1958, *Nilsson s.n.* (BM); **Götaland**: Halland, Halmstad, Gustavsfält, 22 Sep 1955, *Blom s.n.* (BM); Halland, pr. urben Halmiam, 26 Apr 1923, *Jungner s.n.* (BM); Halland, prope usbem Halmiam, 26 Sep 1923, *Jungner s.n.* (K); Halland, Halmstad, 26 Sep 1923, *Jungner 1375* (K); Västra Götaland, Göteborg, Ringon, 29 Sep 1951, *Blom s.n.* (K).

**United Kingdom. Channel Isles**: Jersey, N of St. Ouen’s Bay, Sep 1996, *Dupree s.n.* (BM); **England**: Essex, vice county 18, Dagenham dumps, 1938, *Airy Shaw s.n.* (K); Bedfordshire, Flitton, 14 Oct 1950, *Doug 1401* (K); Bedfordshire, nr Flitwick, 16 Sep 1988, *Hanson 499* (BM); Hampshire, Blackmoor, 24 Oct 1959, *Lousley s.n.* (BM); Berkshire, Reading, 25 Nov 1999, *Rutherford s.n.* (BM); Gloucestershire, Wapping Wharf, Bristol Harbour, 14 Sep 1942, *Sandwith s.n.* (BM); **Scotland**: Highlands, Auldearn, Broomhill Farm, 27 Oct 1967, *McCallum-Webster s.n.* (BM); Midlothian, Railway Tip, Borthwick, 20 Jul 1963, *McCallum-Webster s.n.* (BM); Midlothian, Borthwicts, 20 Jul 1963, *McCallum-Webster 8768* (K); **Wales**: Vale of Glamorgan, Barry Dock, 29 Sep 1923, *Melville s.n.* (BM).

### 9. Solanum opacum

Taxon classificationPlantaeSolanalesSolanaceae

A.Braun & C.D.Bouché, Ind. Sem. Hort. Berol.: 18. 1853

Figures 28 , 29

Solanum forsteri Seem., J. Bot. 1: 207. 1863.
Type. Chile. Región V (Valparaíso): Easter Island, *J.R. Forster & G. Forster s.n.* (lectotype, designated here: BM [BM000900372]).
Solanum fauriei H.Lév., Repert. Spec. Nov. Regni Veg. 10: 152. 1911.
Type. United States of America. Hawaii: Oahu, Kaela, Nov 1909, *U. Faurie 861* (lectotype, designated here: BM [BM000846671]; isolectotypes: P [P00315100, P03961997]).
Solanum apopsilomenum Bitter, Repert. Spec. Nov. Regni Veg. 12: 89. 1913.
Type. New Zealand. Mount [illegible, perhaps Zwart], 28 Sep 1838, *E. Schwarz s.n. [Exped. Novara*] (holotype: W [0022367]).
Solanum microtatanthum Bitter, Bot. Jahrb. Syst. 55: 63. 1919.
Type. Papua New Guinea. Kelil, 180 m, *F.R.R. Schlechter 16407* (holotype: B, destroyed; lectotype, designated here: E [E00273859]; isolectotypes: G [G00343298], L [L0003621], GH [GH00077834], K [K001080534, K001080535]).
Solanum brachypetalum Bitter, Bot. Jahrb. Syst. 55: 64. 1919.
Type. Papua New Guinea. “Ssigaun, in Dorfern”, 600 m, Jun 1896, *C. Lauterbach 2360* (holotype: B, destroyed; lectotype, designated here: WRSA).
Solanum insulae-paschalis Bitter, Nat. Hist. Juan Fernandez & Easter Island 2: 78. 1922.
Type. Chile. Región V (Valparaíso): Easter Island, Hanga ho ono (La Perouse), au linem actem Brunnen, *C. Skottsberg & I. Skottsberg 663* (lectotype, designated here: GOET [GOET003528]; isolectotype: UPS [UPS104030]).
Solanum nigrum L. var. nihoense F.Br., Bernice P. Bishop Mus. Bull. 81: 36, pl. 16B. 1931.
Type. United States of America. Hawaii: Leeward Island, Nihoa, *E.L. Caum 62* (holotype: BISH; isotypes: K [K000922201], NY [00172289]).
Solanum nigrum L. var. pitcairnense F.Br., Bernice P. Bishop Mus. Bull. 130: 255. 1935.
Type. Pitcairn Islands: Pitcairn island, 13 Mar 1922, *E.H. Quayle 375a* (holotype: BISH [BISH1005084]; isotype: BISH [BISH1005083]).
Solanum allanii Polgár, Trans.& Proc. Roy. Soc. N. Z. 69: 278. 1939.
Type. Cultivated in Györ Hungary from seeds of Miss L.B. Moore #8 (New Zealand, Auckland, Mt. Wellington), Jan 1938, *S. Polgár s.n.* (lectotype, designated here: BP [acc. # 146410]; isolectotype: BP [acc. # 261051]).

#### Type.

Cultivated in Germany at the Berlin Botanical Garden from seeds from Australia “New Holland (Listeman) hort. bot. Berlin”, *Anon. s.n.* (lectotype, designated here: HBG [HBG511471]).

#### Description.

Annual or short-lived sprawling to erect perennial herbs to 1 m tall, subwoody and branching at base. Stems spreading to decumbent, terete or sometimes slightly ridged, green to yellow-green, older stems greenish-grey, not or occasionally somewhat hollow; new growth densely to sparsely pubescent with simple, antrorse, uniseriate, translucent, eglandular or sometimes glandular trichomes, these 4–6-celled, 0.5–1 mm long; older stems glabrescent. Sympodial units difoliate, the leaves not geminate. Leaves simple, 1.5–8.0(-17) cm long, 1–4 -(9) cm wide, elliptic to slightly ovate, very variable in size, membranous, green, concolorous, without smell; adaxial surface glabrous or sparsely and evenly pubescent with simple uniseriate ca. 4-celled trichomes to 0.5 mm long; abaxial surfaces glabrous or sparsely pubescent with simple uniseriate trichomes along the veins; major veins 3–8 pairs, not prominent; base cuneate, decurrent on the petiole; margins entire or shallowly toothed, if present the teeth acute; apex acute to acuminate; petioles 0.5–3 cm long, sparsely pubescent with antrorse simple uniseriate trichomes like those of the stems. Inflorescences 1–2 cm long, internodal, unbranched but very rarely furcate, umbelliform to sub-umbelliform, with 3–7 flowers clustered near the tip of the rhachis, sparsely pubescent with antrorse simple uniseriate 3–4-celled trichomes like those of the stems; peduncle 1–3 cm long, straight and stout; pedicels 0.4–0.8 cm long, < 0.3 mm in diameter at the base, ca. 0.3 mm in diameter at the apex, filiform, nodding, pubescent like the peduncle, articulated at the base; pedicel scars clustered near the tip of the rhachis, often the lowest flower ca. 0.5 mm spaced from the rest. Buds ellipsoid, the corolla strongly exserted from the calyx tube long before anthesis. Flowers 5-merous, all perfect. Calyx tube 1–2 mm long, conical, the lobes often unequal, the lateral two largest 1–1.2 mm long, 0.5–0.6 mm wide, the top and lowermost 0.4–1.0 mm long, 0.3–0.5 mm wide, long triangular often with a rounded tip, glabrous or sparsely pubescent with antrorse simple uniseriate trichomes ca. 0.5 mm long. Corolla 6–10 mm in diameter, white or white with a purplish tinge, stellate, lobed ca. 1/2 way to the base, the lobes 3.0–4.2 mm long, 1.0–1.2 mm wide, spreading to reflexed, densely papillate on tips and margins. Stamens equal; filament tube < 0.1 mm long; free portion of the filaments 0.5–1.0 (-1.5) mm long, glabrous or adaxially pubescent with tangled simple uniseriate trichomes; anthers 1.2–1.6 mm long, 0.7–1.0 mm wide, ellipsoid, yellow, somewhat sagittate at the base, poricidal at the tips, the pores lengthening to slits with age and drying. Ovary rounded, glabrous; style 3–4 mm long, strongly curved in the distal 1/4, densely pubescent with simple uniseriate trichomes 0.2–0.5 mm long, these tangled in the basal 1/2 to 2/3 of the length, not exserted beyond anthers and only the stigma visible outside the anther cone; stigma capitate, the surfaces minutely papillate. Fruit a globose berry, 4–10 mm in diameter, green or bluish-black at maturity, the pericarp thin, matte; fruiting pedicels 0.7–1.5 cm long, ca. 0.5 mm in diameter at the base and the apex, erect or spreading, becoming yellow, falling with the mature fruits, not persistent; fruiting calyx lobes not accrescent, the tube less than 1 mm long, the lobes 1.2–1.7 mm long, appressed to the basal quarter of the berry, occasionally somewhat spreading, never strongly reflexed. Seeds 50–100 per berry, 1.2–2.2 mm long, 0.7–1.8 mm wide, flattened reniform, pale yellowish-tan, the surfaces minutely pitted, thin and the embryo clearly visible, the testal cells rectangular to pentagonal in outline. Stone cells (0-)2(-4) per berry, >0.5 mm in diameter, brown. Chromosome number: *2n*=6x=72 (Henderson 1974; Symon 1985).

**Figure 28.**
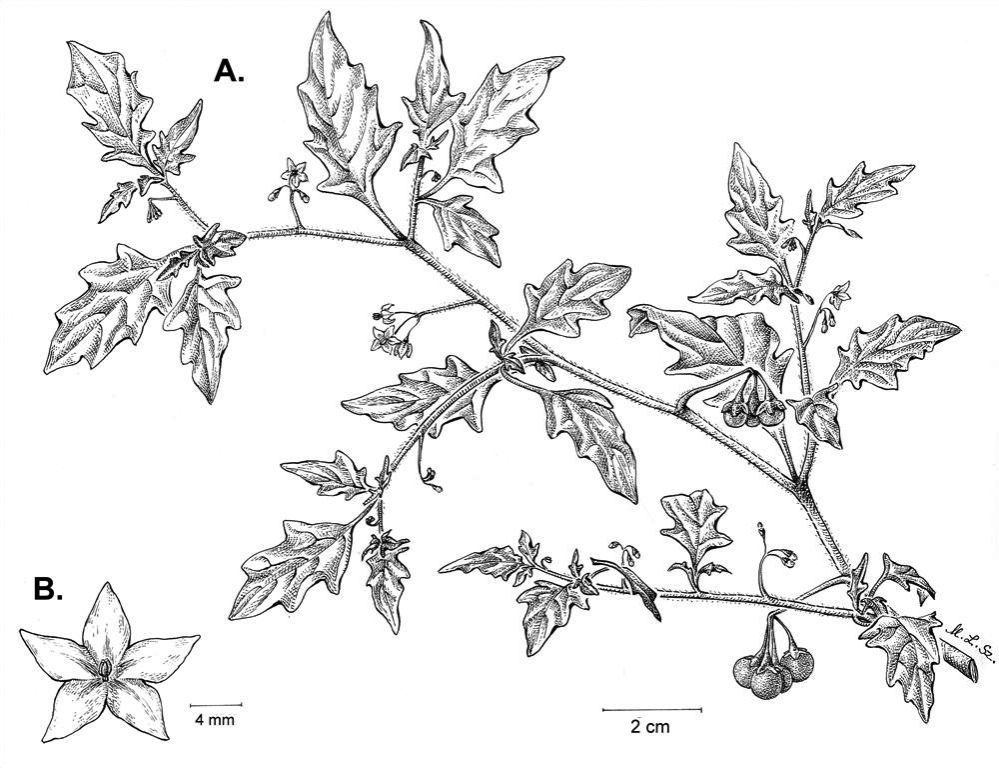
*Solanum opacum*. **A** Habit. **B** Detail flower (**A–B** *Symon s.n.* [ADW 40795]). Drawing by M.L. Szent-Ivany, first published in Symon (1981), courtesy of the Board of the Botanic Gardens and State Herbarium (Adelaide, South Australia), reproduced with permission.

**Figure 29.**
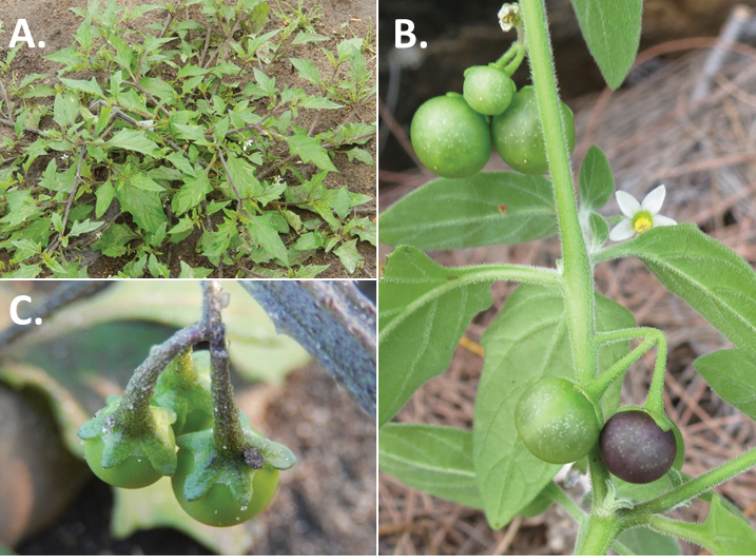
*Solanum opacum* **A** Habit **B** Shoot with flowers and maturing fruits **C** Detail of maturing fruits with appressed calyx lobes (**A** Nijmegen acc. 904570211, **B** *Butaud 3075*, **C** Nijmegen acc. 884570223). Photos by J.-F. Butaud and G. van der Weerden.

#### Distribution

**Figure 30.**
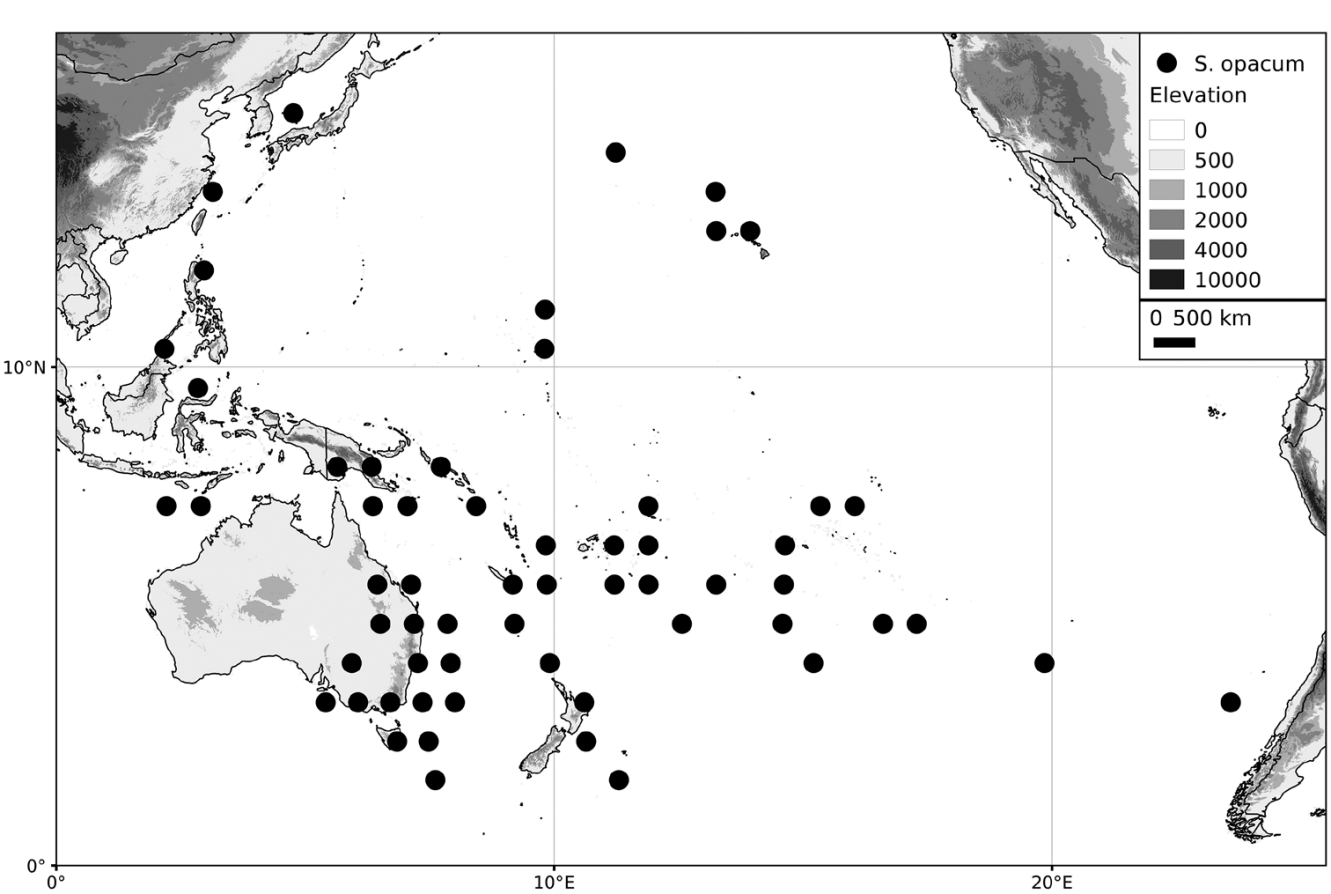
Distribution of *Solanum opacum*.

(Figure 30). Native in the Pacific islands, from Australia, Papua New Guinea and New Zealand, to Taiwan and Hawaii, west to the Pitcairn Islands and Rapa Nui (Easter Island).

#### Ecology.

Grows in disturbed areas, along roadsides and field edges; between sea level and 2,600 m elevation in a wide variety of habitats.

#### Common names.

Australia: currant, green-berry nightshade (Symon 1981; Harden 1993), dark nightshade (Walsh and Entwisle 1999), tamatha, tamada; Fiji: mau, mboro laukana, murimuri; Indonesia: kenkene; Malaysia: tutan; Papua New Guinea: bek, kepa, kepilam, ongkara, tagur/tagura, tuskombuk, wietat; Pitcairn Islands: o brew.

#### Uses.

Leaves eaten as greens throughout the species range.

#### Preliminary conservation status

(IUCN 2016). *Solanum opacum* is a relatively widespread species across the Pacific; it can be assigned a preliminary status of LC (Least Concern; Table 7) on a global scale, but local country assessments may differ. The species has a very scattered distribution in the Pacific and, although its overall range is very large, the land area it occupies is small.

#### Discussion.

*Solanum opacum* is extremely similar to *S. americanum*; both species have minute flowers with anthers usually less than 1.5 mm long. They co-occur across the Pacific, but can be distinguished easily, particularly in fruit. *Solanum opacum* has matte berries that are green or greyish-purple at maturity, with appressed or slightly spreading calyx lobes, while *S. americanum* has very shiny berries that are black or dark purple at maturity and calyx lobes that are very strongly reflexed. The pedicels of *S. americanum* remain on the plant as fruits fall, while those of *S. opacum* drop with the mature berries. In flower, the relative length of filament to anther is a useful distinguishing character; *S. opacum* has filaments that are as long as or slightly longer than the anthers, while the filaments of *S. americanum* are always shorter than the anthers.

Our circumscription of *S. opacum* encompasses a much wider distribution than that of Henderson (1974), who treated only the Australian populations. The treatments of “Solanum nigrum” or “Solanum americanum” in the Pacific could apply to either *S. opacum* or *S. americanum*. Degener (1945) records the use of morelloid species in Hawaii for both food and medicine; both species occur there.

*Solanum opacum* is hexaploid and, based on network analysis of arbitrary amplified DNA markers, Poczai and Hyvönen (2011) suggested that Australian populations of *S. retroflexum* were a possible tetraploid parent of *S. opacum*. The putative diploid parent is *S. chenopodioides*, *S. nitidibaccatum* or some other closely related species (Poczai and Hyvönen 2011). Morphologically, *S. retroflexum* somewhat resembles *S. opacum*, but the shared molecular markers could also come from a common progenitor of the two polyploid species.

Henderson (1974) neotypifed *S. opacum* with material collected by Robert Brown on the voyage of the Discovery (Bird Sound, 19 Sep 1802, *R. Brown s.n.* (neotype, designated by Henderson 1974, pg. 39: NSW [NSW125341]; isoneotypes: BM [BM000900380], K, MEL)) because he could find no original material corresponding to the collector or locality cited in the protologue (“E Nova Hollandia. Semina communicavit Listemann”) and presumed it to have been in Berlin and subsequently destroyed. However, recent digitisation of historical material in European herbaria has uncovered original material not seen by Henderson (http://plants.jstor.org/stable/viewer/10.5555/al.ap.specimen.hbg511471) that was annotated as “lectotype” in 1974 by J.M. Edmonds, but never published as such. The fact that original material has been found means that Henderson’s (1974) neotypification must be set aside (McNeill et al. 2012, Art. 9.19) and we designate the sheet in HBG (HBG511471) as the lectotype of *S. opacum*. It matches the wild-collected plants selected by Henderson exactly.

*Solanum forsteri* was named in honour of the Forster’s (father Johann and son Georg, who accompanied Captain Cook on the HMS *Resolution* from 1772 to 1775) and we have selected the sheet collected by them on Easter Island (BM000900372) as the lectotype. This specimen is mounted together with a collection of *S. americanum* that is on different paper (white rather than the blue associated with Forster’s material), showing the difficulty that early botanists (and this holds true still today) had in distinguishing these two species with minute anthers that co-occur across the Pacific.

In describing *S. microtatanthum*, Bitter (1919) cited a specimen in the Berlin herbarium, now destroyed; we have selected the most complete of the duplicates we have seen as the lectotype (E00273859). The protologue of *S. fauriei* cited no herbaria and we have selected the best preserved of the duplicates we have seen as the lectotype (BM000846671). We have selected the sheet with better material for both fruits and flowers (BP No. 146410) as the lectotype of *S. allanii*.

#### Selected specimens examined.

**Australia**. **New South Wales**: Botany Bay, 28 Apr 1770, *Banks* & *Solander s.n.* (BM); Sans Souci, nr Georges River, 27 Sep 1961, *Goode 346* (K, NSW); Kyogle, Toonumbar State Forest 26 km NW of Kyogle, 22 Feb 1972, *Henderson 1261* (AD, AD, NSW); Upper Hunter Shire, Ben Halls Gap State Forest, 20 Feb 1991, *Hosking 307* (NE); Wollongong, Bulli, 1875, *Johnson s.n.* (MEL); Wollongong, Mt Keira, 27 Jul 1983, *Mills s.n.* (WOLL); **Norfolk Island**: sin. loc, Oct 1805, *Caley s.n.* (BM); Kingston, 24 Aug 1964, *Uhe 1126* (K); **Northern Territory**: Adelaide River, Northern Territory, grown in glasshouse at Indooroopilly, Brisbane, 18 Dec 1972, *Henderson 1356* (CANB, K); **Queensland**: Nr border of Queensland/New South Wales, Main Range National Park, 10 Feb 2006, *Bohs et al. 3561* (BM, UT); Birnam Range 7.5 km NE of Beaudesert, Tremayne rd, Jan 2001, *Halford Q-3882* (BRI, K, MEL, NSW); c. 13 km WNW of Goondiwindi on rd to St. George, 30 Sep 1975, *Henderson 2357* (AD, BRI, K); **South Australia**: Upper River Murray nr 375 mile peg 4 mi downstream from Little Hunchee Island, on North bank, 16 Sep 1979, *Symon 11586* (AD, CANB, K); Kangaroo Island, Kangaroo Island, *Waterhouse s.n.* (MEL); **Tasmania**: Tasman, Port Arthur, 1893, *Bufton 11* (MEL); sin. loc, 1842, *Gunn 51[a*] (BM); Latrobe, Harford on Solomans Hill, 6 Nov 1932, *Hamilton 163* (CANB, HO); Van Diemen’s Land, Gunn, 1835, *Without collector s.n.* (K); King Island, Nr Sea Elephant River, 23 Mar 2009, *Wapstra et al. 691* (AD); **Victoria**: Mt Buffalo access rd 12 km WNW of Bright, 1 Jan 2003, *Lepschi 4916* (CANB, MEL); Latrobe, 18 Jul 1988, *Thompson 157* (AD, AD, MEL); Cedar Creek, Bentleigh district, 12 Feb 1920, *White s.n.* (NSW); **Western Australia**: c. 200 mi N of Chesapeake rd 1.5 km E of Mt Chuladup, 7 Dec 2001, *Barker 8355* (AD); Eyre, S of Cocklebeddy, 5 Dec 1962, *MacDonald 29* (K); Bayswater, Lower Swan River, 20 Mar 1909, *Morrison 19014* (K).

**Chile**. **Región V (Valparaíso)**: Easter Island, *von Chamisso s.n.* (F, NY); Juan Fernández Islands, Masafuera, Quebrada de Veradero, 12 Mar 1917, *Skottsberg* & *Skottsberg 568* (GOET).

**Cook Islands**. **Mitiaro**: just before entering swamp on rd to Atai Foodland, 22 Jul 1991, *Luttrell 187* (FHO); rd from the village of Takaue to the marsh, 24 Apr 1985, *Whistler 5576* (US).

**Fiji**. Yuen Yick’s farm, Nasinu, Naitasiri, 3 Dec 1957, *Ledua s.n.* (K); sin. loc., 1860, *Seemann 344* (BM, K); **Fulanga**: Limestone Formation, 22 Feb 1934, *Smith 1174* (K); **Kandavu**: Mount Mbuke Levu, 23 Oct 1933, *Smith 206* (K); **Viti Levu**: Mba, summit of Mt. Nanggaranambuluta E of Nandarivatu, 19 Jun 1947, *Smith 4849* (K, US); Hills E of Wainikoroiluva River, nr Namuamua, Namosi, 15 Oct 1953, *Smith 9070* (K, US).

**French Polynesia**. **Austral Islands**: Raivavae, Vaiuru, 10 Aug 1934, *Fosberg 11756* (US); Rurutu, du N de l’ile, 17 Apr 1981, *Hallé 7032* (US); Rurutu, N Moerai, 25 Apr 1981, *Hallé 7321* (US); Raivavae, Ahuoivi, 9 Aug 1934, *St. John 16070* (US); **Gambier Islands**: Mangareva, 1833, *Le Guillou s.n.* (US); Aukena, Koiovao, 29 May 1934, *St. John 14659* (US); **Society Islands**: Tahiti, 6 Apr 1840, *Barclay 3309* (BM)Tahiti, plateau de Taravao, sentier du captage de l’Hamoa, 16 Sep 1982, *Florence 3853* (US); Raiatea, first valley S of Uturoa, 14 Oct 1926, *Moore 212* (US); Me’et’ia, Fatia-po to Fareura, 12 May 1934, *St. John 14187* (US).

**Indonesia**. **Bali**: Kintamani, 14 Nov 1966, *Schwabe s.n.* (B); **East Nusa Tenggara**: Flores, nr Mataloko, *Verheijen 24* (L); Flores, nr Mataloko, *Verheijen 191* (L); **Papua**: Mimika Regency, PT-Freeport Indonesia Concession Area, 9 Aug 1998, *Johns et al. 9565* (A, K); Mimika Regency, W and above Tembagapura, 23 Aug 1998, *Sands 7296* (K); **Sulawesi**: Central Sulawesi, Sopu valley, 30 May 1975, *Balgooy 3555* (A, K); **West Papua**: West-Irian, Eipomek-Tal, bei Malingdam, 24 Feb 1976, *Hiepko* & *Schutze-Motel 1195* (B).

**Japan**. Nagasaki Pref., Katamatsuura-gun, Emukae-cho, Okugawachi-men, 17 Aug 1994, *Yonekura 3230* (MO).

**Malaysia**. **Sabah**: Kampung Tiung, side of hill Tiung Valley, Tuaran Distr., 8 Aug 1985, *Maikin Lantoh SAN-108976* (K)

**Marquesas Islands**. **Hatutaa Island**: vallon de la pointe SW, 10 Jul 1988, *Florence* & *Teikiteetini 9399* (US)

**Marshall Islands**. **Arno Atoll**: Ine village, 12 May 1950, *Anderson 3672* (L, US); **Jaluit Atoll**: Jabor, 28 Apr 1958, *Fosberg 39475* (US); **Kwajalein Atoll**: Kwajalein Islet, 19 Jan 1950, *Fosberg 31180* (US)

**New Caledonia**. sin. loc, *Caldwell s.n.* (K); NO de la Nouvelle Caledonie, 1867, *Krieger s.n.* (W); **Nord**: Ilôt Poudiou, observatory Island, Isle of Pines, Oct 1853, *MacGillivray 805* (K); **Sud**: Nouméa, Îlot Freycinet, Ilot de Freycinet, Aug 1884, *Grunow s.n.* (W); Noumea, Baie Tina, 24 Jul 1973, *MacKee 26964* (K).

**New Zealand**. **Chatham Islands**: Rekohu, (Chatham Island), Waitangi, Ellice Point, Chatham Islands Ecological Region and District, 1 Dec 2008, *de Lange &Horne CH-2032* (AK). **Kermadec Islands**: Macauley Island, Southern Kermadec Islands Group, 29 Jun 2006, *Barkla M- 26* (AK); Curtis Island, Nov 1900, *Shakespear s.n.* (AK). **North Island**: Northland, dunes near Te Arai Forest Sanctuary, Aupouri State Forest, N of Waimahru Stream, Mangonui County, 25 Sep 1985, *Bellingham 0144* (AK); Taranaki, Sugarloaf Island, off Mangonui, Moturoa Group, off Karikari Peninsula, 14 May 1976, *Wright 1252* (AK). **Outlying Islands**: Three Kings Islands, Great Island, above cliff S.E. Bay, 19 Apr 1946, *Turbott & Bell s.n.* (AK). **South Island**: Motuara, *Banks* & *Solander s.n.* (BM). **Tokelau Territory**: Fakaofu, Union Islands, Mar 1891, *Lister s.n.* (K).

**Niue**. Niue Island, “71 Polokai”, 1901, *Smith s.n.* (AK).

**Norfolk Island**. Rocky Point Reserve, 6 Oct 1989, *Gardner 5866* (AK).

**Papua New Guinea**. Boridi, 6 Sep 1935, *Carr 12985* (BM, K); Wiligimaan, Baliem, Div. Hollandia, 28 Jun 1961, *Versteegh BW-12516* (A, L); **Bougainville Island**: Namatea, NW Bougainville, 7 Mar 1932, *Waterhouse 691 B* (K, L); Crown Prince Mountain, Oct 1960, *Womersley NGF-13346* (A); **Central**: E side lake Myola No. 2, Distr. Central, Subdistr. Port Moresby, 13 Sep 1973, *Croft NGF-34532* (E, K, L); Kokoda trail, eastern side lake Myola, 22 Jul 1974, *Croft LAE-61910* (A, K, QRS); **Madang**: Saidor, Moro, Naho-Rawa Division, Finisterre mountains, 19 Nov 1964, *Perumal 21473* (BM); **Morobe**: Mount Kaindi, Wau, 4 Oct 1977, *Conn* & *Kairo 491* (CANB, K); Bulldog rd, nr Edie Creek, Wau, 13 Aug 1963, *Millar* & *Holttum NGF-15825[b*] (A, US); **Southern Highlands**: Hagen-Mendi rd, Mendi Subdistrict, Southern Highlands District, 21 Sep 1968, *Vandenberg et al. NGF-40062* (K); **Western Highlands**: nr Ampyak Highlands, Ecological Site 1, 15 Dec 1964, *Flenley ANU-2192* (K); nr Tomba village, S slope of Mount Hagen Range, 27 Aug 1956, *Hoogland* & *Pullen 6014* (BM, US); **Western Province**: Western, 19 Jun 1979, *Sohmer LAE-75542* (K); Kubor Range, Uinba, Nona-Minj Divide, 20 Aug 1963, *Vink 16311* (K).

**Philippines**. **Luzon**: Cordillera (CAR), Mount Pulag, 26 Jan 1968, *Jacobs 7173* (K).

**Pitcairn Islands**. **Oeno Island**: Close to hut, 23 Jun 1934, *St. John* & *Fosberg 15195* (US); **Pitcairn Island**: Bounty Bay, baie de la Bounty, Cap Est, 22 Apr 1991, *Florence 10774* (US); Bounty Bay, 15 Jun 1934, *Fosberg* & *Clark 11337* (US).

**Samoa**. **Apia**: Apia, 6 May 1907, *Vaupel Sol-3* (K); **Savai’i**: E of Olo, 8 Aug 1931, *Christerpherson* & *Hume 2311* (K).

**Solomon Islands**. **Guadalcanal**: north central Guadalcanal, Tina River, 14 Sep 1967, *Maurisi BSIP8109* (K).

**Taiwan**. **Pingtung County**: Wutain Hsiang, Tawu village, 13 Mar 1999, *Shu-hui Wu 1161* (MO); **Taoyuan County**: Kuanyin Hsiang, Hsinpo, 28 Dec 1999, *Ching-I Peng*, *17879* (MO).

**Tonga**. **Tofua**: sin. loc, Jan 1967, *Scarth-Johnson s.n.* (K); **Tongatapu**: Kologa, Jun 1926, *Setchell* & *Parks 15374* (US).

**United States of America**. **Hawaii**: Leeward Island, Nihoa, 17 Jun 1923, *Caum 62* (US); Oahu, Kaela, Nov 1909, *Faurie 861* (BM, P); Kauai, Kaholuamanoa, above Waimea, 1 Oct 1895, *Heller 2867* (AK, BM, E, US); Pearl and Hermes Atoll, Southeast Island, midway between pond and tower, 18 Aug 1964, *Young 112* (US).

### 10. Solanum palitans

Taxon classificationPlantaeSolanalesSolanaceae

C.V.Morton, Revis. Argentine Sp. Solanum 92. 1976

Figures 31 , 32

#### Type.

Argentina. Tucumán: Dept. Tafí del Valle, Yerba Buena, 19 Jan 1919, *S. Venturi 159* (holotype: US [00027724, acc. # 1548805]; isotypes: BA [BA2463], LIL [LIL001454], LP [LP010926], MA, SI [SI003329]).

#### Description.

Annual, decumbent or prostrate herbs, the young plants sometimes erect, up to 15 cm tall often rooting at the lower nodes, forming dense patches, the branches to ca. 1 m long. Stems decumbent or ascending, terete or somewhat angled with ridges, green, older stems yellowish-brown, not markedly hollow; new growth pubescent with simple, spreading, uniseriate, translucent, eglandular trichomes, these 0.5–1 mm long, to or nearly glabrous; older stems glabrous. Sympodial units difoliate, the leaves not geminate. Leaves simple, 2.5–9 cm long, 2.5–7.5 cm wide, broadly ovate, thinly membranous, green, concolorous, without smell; adaxial surfaces glabrous to sparsely pubescent with simple hairs to 0.5 mm on the major veins; abaxial surfaces glabrous; major veins 3–4 pairs; base long attenuate, decurrent on the petiole; margins 3-lobed nearly to the midrib, rarely the lateral lobes themselves lobed, the terminal lobe ovate, the lateral lobes asymmetrically ovate or lanceolate-ovate, acute at the tips, the sinuses sometimes sparsely ciliate; apex acute; petioles 0.5–2 cm, winged to the base, glabrous or sometimes sparsely ciliate near the base. Inflorescences 1.2–2.5 cm, internodal or often just below a node, unbranched or rarely with a few branches, the flowers spaced along the rhachis, with 4–9 flowers, glabrous to sparsely pubescent; peduncle 0.7–1.4 cm long, delicate; pedicels 3–5 mm long, 0.2–0.3 mm in diameter at the base and at the apex, filiform, spreading, articulated at the base; pedicel scars spaced 1–5 mm apart. Buds ellipsoid, the corolla completely covered by the calyx tube before anthesis. Flowers 5-merous, all perfect. Calyx tube 1.5–2 mm long, cup-shaped, the lobes ca. 0.75–1.5 mm long, less than 1 mm wide, lanceolate-oblong, the tips acute, glabrous. Corolla ca. 7 mm in diameter, white or rarely light violet, rotate-stellate, lobed ca. 1/2 way to the base, the lobes 1.5–2.5 mm long, 1–2 mm wide at the base, reflexed or spreading at anthesis, abaxially minutely white-puberulent on the tips of the lobes, glabrous adaxially. Stamens equal; filament tube minute; free portion of the filaments 0.5–1 mm long, adaxially pubescent with tangled uniseriate trichomes; anthers 1.6–2 mm long, 0.7–0.8 mm wide, oblong or ellipsoid, yellow, poricidal at the tips, the pores lengthening to slits with age and drying. Ovary glabrous; style 2.3–3.3 mm long, glabrous or sparsely pubescent in the lower part where included in the anther cone, exserted 0.5–0.8 mm beyond anthers; stigma capitate, the surface minutely papillate, green in live plants. Fruit a depressed-globose and bilobed (especially when young) berry, 6–8 mm in diameter, pale yellow, the pericarp thin and somewhat shiny; fruiting pedicels 4–7 mm long, 0.5–0.7 mm in diameter at the base and at the apex, spreading, recurved at the base to hold the fruit downwards, nearly in contact with the soil, dropping with the mature fruit, not persistent; fruiting calyx not markedly accrescent but the lobes somewhat elongating in fruit, the tube 2–3 mm long, the lobes 2–3(-4) mm long, covering the basal 1/3 of the berry, the tips somewhat recurved. Seeds 20???30 per berry, 1.5–1.6 mm long, 1.2–1.6 mm wide, flattened reniform, light yellow, the surfaces pitted, the testal cells sinuate in outline. Stone cells 2(4) per berry, 2 larger and apical (1–1.5 mm in diameter), the other 2 equatorial, smaller, 0.5–0.6 mm in diameter. Chromosome number: *2n*=2x=24 (Moscone 1992; Moyetta et al. 2013).

**Figure 31.**
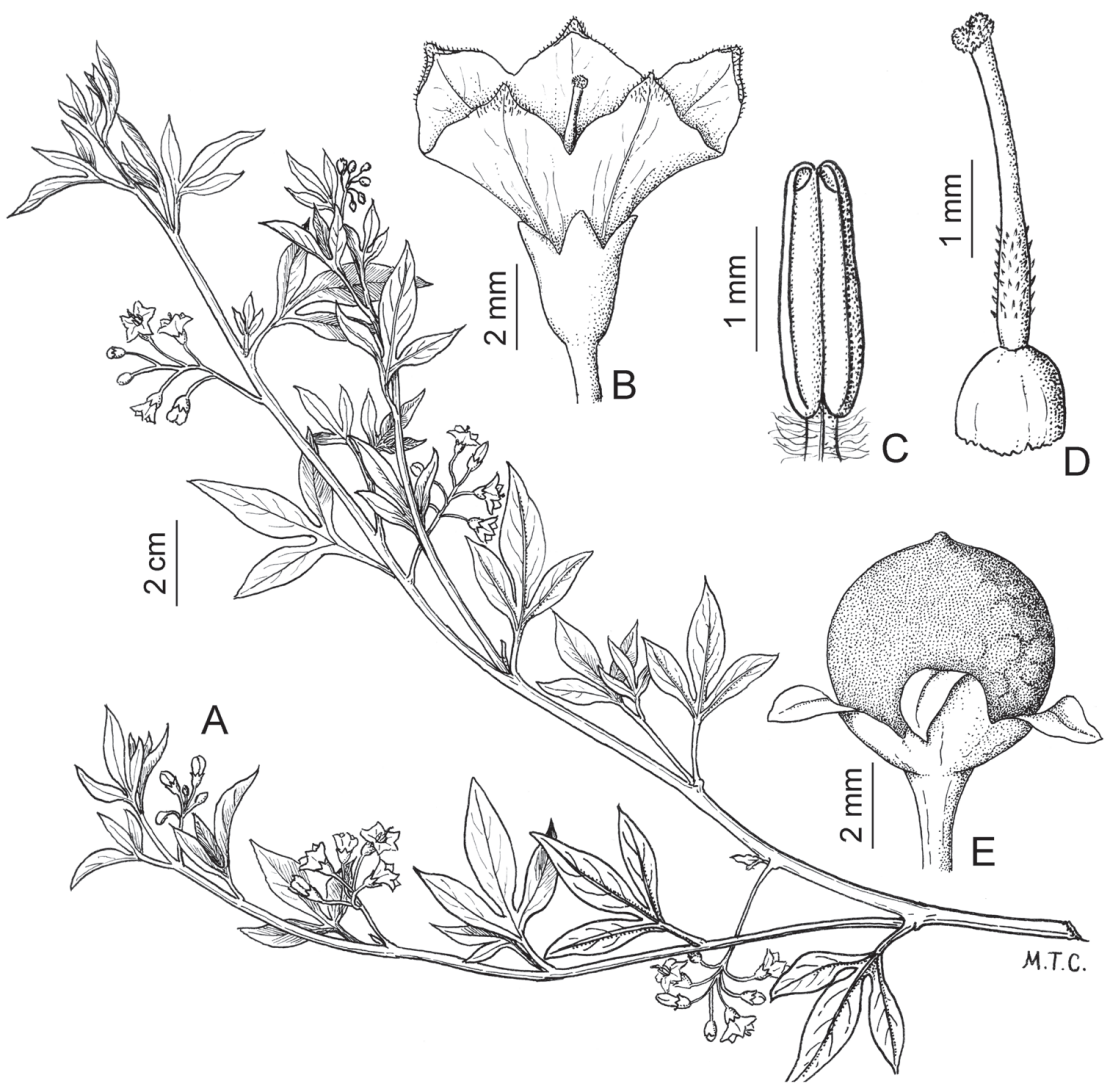
*Solanum palitans* **A** Habit **B** Flower **C** Anther **D** Gynoecium **E** Fruit (**A–F** Cabrera 13875). Drawing by Drawing by M.T. Cabrera, first published in Flora de Jujuy (1983), courtesy of the Board of the Instituto Darwinion (San Isidro, Buenos Aires, Argentina), reproduced with permission.

**Figure 32.**
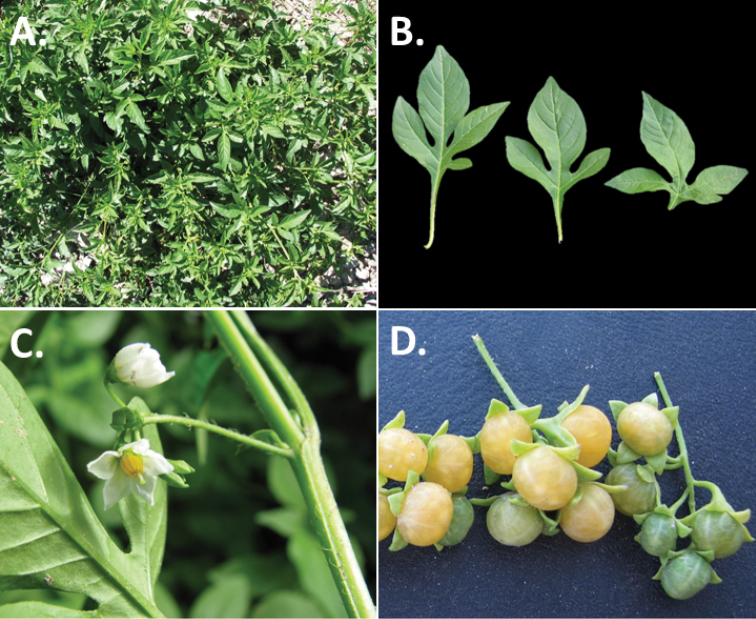
*Solanum palitans* **A** Habit **B** Leaves **C** Flowers and inflorescence **D** Fruits at different stages of maturity (**A–D** *Barboza et al. 3471*). Photos by Tiina Särkinen.

#### Distribution

**Figure 33.**
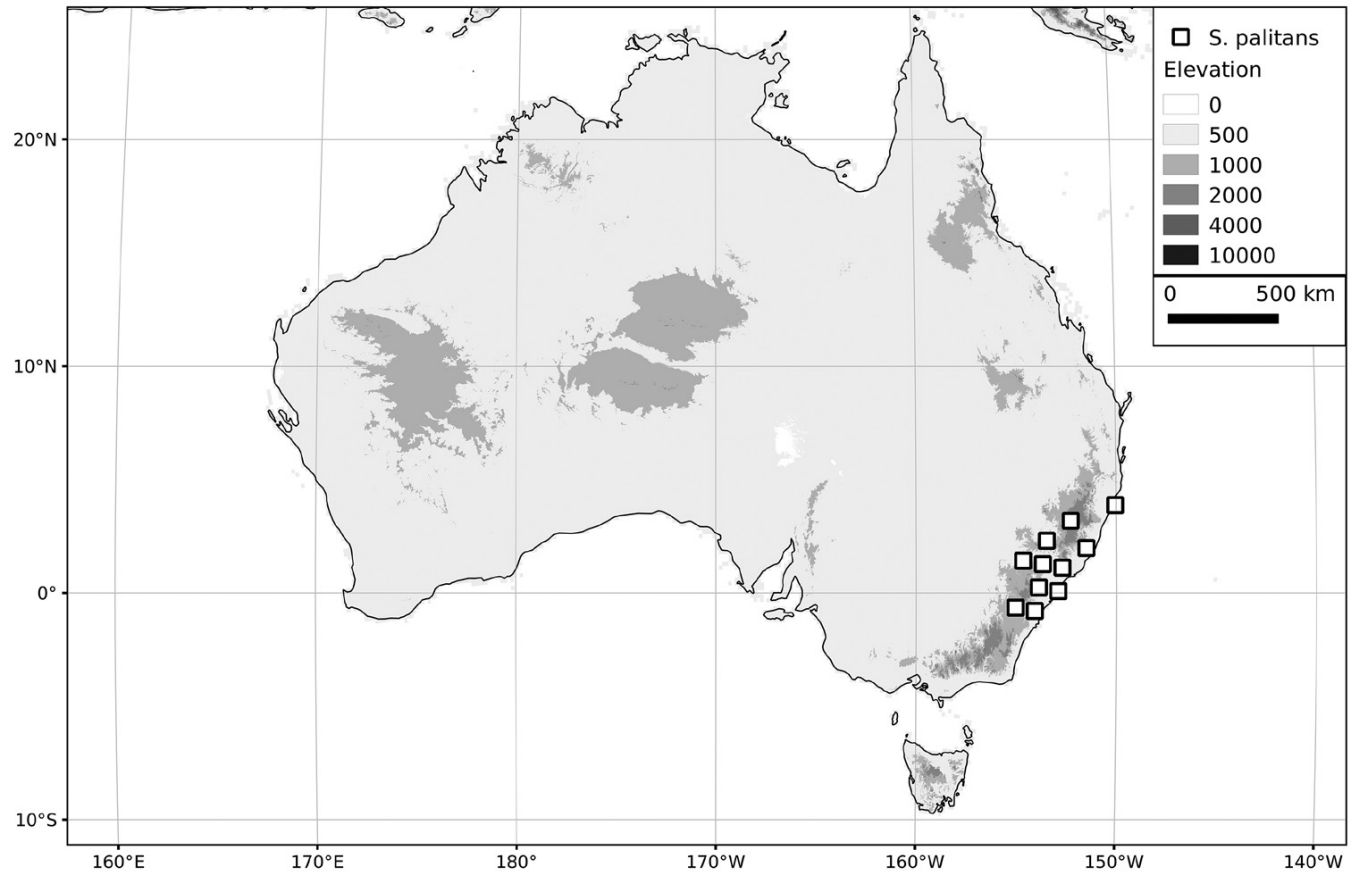
Distribution of *Solanum palitans* in its non-native range in the Old World.

(Figure 33). Native to north-western and central Argentina, northern Chile and Bolivia and apparently very locally naturalised in New South Wales (Australia).

#### Ecology.

Grows in disturbed sites, along roadsides and field margins, on rocky, sandy or clay soils; between (50-) 1,400 and 3,000 (-3,700) m in its native range, near sea level in Australia.

#### Common names.

None recorded for the Old World.

#### Uses.

None recorded for the Old World.

#### Preliminary conservation status

(IUCN 2016). *Solanum palitans* is a widespread species in its native range, has a relatively large EOO across its entire distribution and can hence be assigned a preliminary status of LC (Least Concern; Table 7). It is only locally established in coastal New South Wales. The preliminary assessment based on South American distribution is still LC based on EOO (898,907 km^2^).

#### Discussion.

*Solanum palitans* is distinct amongst the species of morelloids occurring in the Old World in its combination of uniformly 3-lobed, thin membranous leaves and pale yellow mature fruits. The species has a creeping habit, with stems growing close to the ground extending up to 3 m and often rooting at nodes. Outside of its native range in South America, it is only found in Australia, but may appear in other areas of suitable habitat given time. Details of its variability and habitats in Argentina can be found in Morton (1976) and Barboza et al. (2013).

Like other adventive members of this group, it is likely to have been introduced to Australia with wool waste from Argentina. The first collection cited in Symon (1981) is from 1911 and the most recent we have seen is from 2013. It appears not to be spreading from New South Wales, but could also occur in New Zealand due to habitat similarities, although we have seen no specimens of *S. palitans* from there. Symon (1981) records that it is not eaten by stock, perhaps accounting for its limited distribution.

#### Selected specimens examined.

**Australia. New South Wales**: Barallier via Moss Vale, May 1950, *Carlon s.n.* (NSW); Burragorang Lookout, 14 mi S of Yerranderie, 17 mi WNW of Bowral, 23 Mar 1966, *Constable 6738* (CANB, K); Wingecarribee, 39 km WNW of Mittagong, 16 Mar 1975, *Coveny et al. 6089* (AD, NSW); Tamworth Regional, Moorsville, The Forest, S of Moore Creek, 4 Sep 1996, *Hosking 1282* (CANB, MEL, NS, NSW); Campbelltown, Showground, 26 Apr 1964, *McBarron*, *9021* (AD, NSW); Armidale Dumaresq, Hillgrove mine, S of Hillgrove, 15 Oct 2014, *Without collector 16* (NE).

### 11. Solanum pseudospinosum

Taxon classificationPlantaeSolanalesSolanaceae

C.H.Wright, Fl. Trop. Afr. [Oliver et al.] 4, 2: 220. 1906

Figures 34 , 35

Solanum pachyarthrotrichum Bitter, Repert. Spec. Nov. Regni Veg. 10: 542. 1912.
Type. Cameroon. Sin loc., *H. Deistel 631* (holotype: B, destroyed; no duplicates found).
Solanum hypopsilum Bitter, Repert. Spec. Nov. Regni Veg. 10: 543. 1912.
Type. Cameroon. Sud-Ouest: Buea, 1000 m, 8 Mar 1898, *H. Lehmbach 175* (holotype: B, destroyed; no duplicates found).
Solanum molliusculum Bitter, Repert. Spec. Nov. Regni Veg. 10: 546. 1912.
Type. Cameroon. Sud-Ouest: Buea, canyon east of Mannsquelle, *P. Preuss 740a* (holotype: B, destroyed; no duplicates found).

#### Type.

Cameroon. Sud-Ouest: Mt Cameroon, Dec 1862, *G. Mann 1938* (holotype: K [K000028649]; isotype: GH [00395064]).

#### Description.

Annual or short-lived prostrate or sprawling perennial herbs to 0.5–1.5 m tall, subwoody and branching at base. Stems spreading, usually strongly ridged with pseudospinose dentate processes, green, older stems brown or grey, usually hollow; new growth densely pubescent with simple, spreading, uniseriate, translucent, mixed glandular and eglandular trichomes, these 7–10-celled, 1.0–1.5 mm long; older stems glabrescent. Sympodial units difoliate, the leaves not geminate. Leaves simple, 2.5–7 cm long, 1.3–4 cm wide, smaller at distal ends of branches, ovate, widest in the lower third, membranous, green, concolorous, smell not known; both surfaces evenly pubescent with simple uniseriate mostly eglandular trichomes like those on stem; major veins 4–5 pairs; base cuneate; margins entire or shallowly toothed in the basal half; apex acute to acuminate; petioles 0.5–2 cm long, pubescent with trichomes like the leaves. Inflorescences 1–2.5 cm long, opposite the leaves, unbranched, sub-umbelliform to racemose, with 2–5(-6) flowers clustered at the tip, pubescent with uniseriate trichomes like those of the stems; peduncle 0.3–1.5 cm long, straight; pedicels 0.7–0.8(-1) cm long, ca. 0.5 mm in diameter at the base, ca. 1 mm in diameter at the apex, slender, spreading, pubescent with simple uniseriate trichomes like the rest of the inflorescence, articulated at the base; pedicel scars clustered in the apical portion of the inflorescence rhachis, lowermost scars ca. 2 mm apart, the rest overlapping at the distal end. Buds ellipsoid, densely pubescent, the corolla at least 1/2 way exserted from the calyx tube until just before anthesis. Flowers 5-merous, all perfect. Calyx tube 1.5–2 mm long, conical, the lobes 1–1.5 mm long, 0.9–1.4 mm wide, elongate-deltate, rounded at the tips, densely pubescent with simple uniseriate trichomes like the rest of the inflorescence. Corolla 8–14 mm in diameter, white to pale violet (“blue”) or white with purple venation, stellate, lobed nearly to the base, the lobes 4–6 mm long, 2–2.5 mm wide, spreading at anthesis, abaxially moderately pubescent with simple uniseriate 2–3-celled trichomes, these denser near the tips and margins, also papillate along the margins and tips, adaxially glabrous. Stamens equal; filament tube minute; free portion of the filaments 0.5–1 mm long, densely pubescent adaxially with tangled simple trichomes; anthers 2–2.5 mm long, ca. 1 mm wide, ellipsoid, yellow, poricidal at the tips, the pores lengthening to slits with age and drying. Ovary conical, glabrous; style 3–5 mm long, densely pubescent with tangled simple trichomes in the basal portion inside the anther tube, exserted ca. 2 mm beyond the anther cone, often elongating early and protruding from unopened buds; stigma capitate, the surfaces minutely papillose, colour in live plants not known. Fruit a globose berry, 4–6 mm in diameter, purple or black at maturity, the pericarp thin and matte; fruiting pedicels 0.9–1.2 cm long, ca. 0.75 mm in diameter at the base and at the apex, spreading, not falling with the fruit, remaining on the plant and persistent on older inflorescences; fruiting calyx not accrescent, the tube ca. 1 mm long, the lobes 1.5–2.5 mm long, appressed to the berry, spreading and hyaline, the tips slightly reflexed. Seeds ca. 20 per berry, ca. 1.5 mm long, ca. 1 mm wide, not markedly flattened, tear-drop shaped with a subapical hilum, pale beige, the surface minutely pitted and the lateral testal cell walls elongate and the seed appearing hairy, the testal cells pentagonal in outline. Stone cells 4–6(-10), often found near the pedicel junction at the base of the berry. Chromosome number: *2n*=4x=48 (Morton 1993).

**Figure 34.**
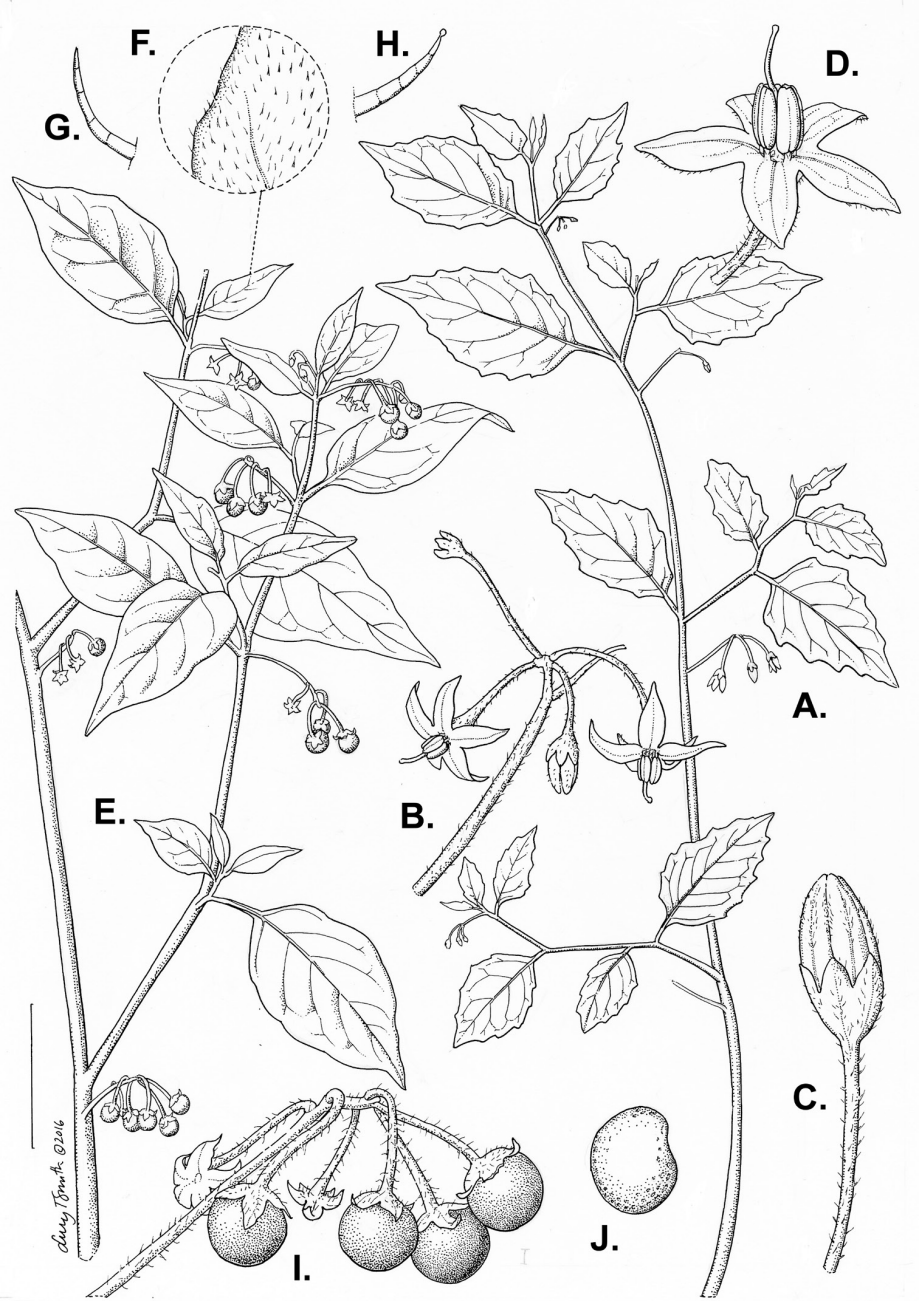
*Solanum pseudospinosum* **A** Flowering habit **B** Inflorescence **C** Bud **D** Flower at anthesis **E** Fruiting habit **F** Detail of adaxial leaf surface **G** Eglandular trichome **H** Glandular trichome **I** Infructescence **J** Seed (**A, C, D** *Casas 10313*; **B** *Casas 10157*; **E, J** *Breteler et al. 39*). Scale bar: 4 cm (**A, E**), 1 cm (**B, I**), 5 mm (**C**), 7 mm (**D**), 4 mm (F), 0.8 mm (**G–H**) and 2.5 mm (**J**). Drawing by L. Smith.

**Figure 35.**
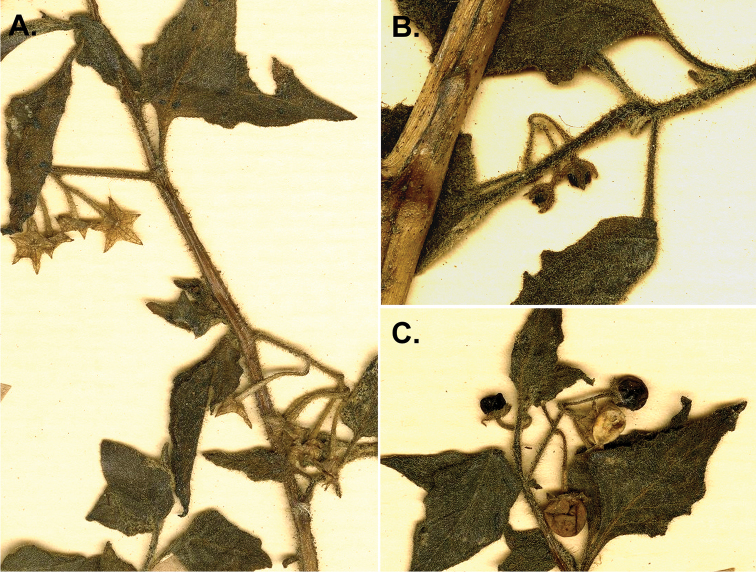
*Solanum pseudospinosum* **A** Infructescence **B** Fruiting pedicels remaining after fruits have dropped **C** Calyx in fruit remaining appressed (**A–B** *W. Meijer 15435*).

#### Distribution

**Figure 36.**
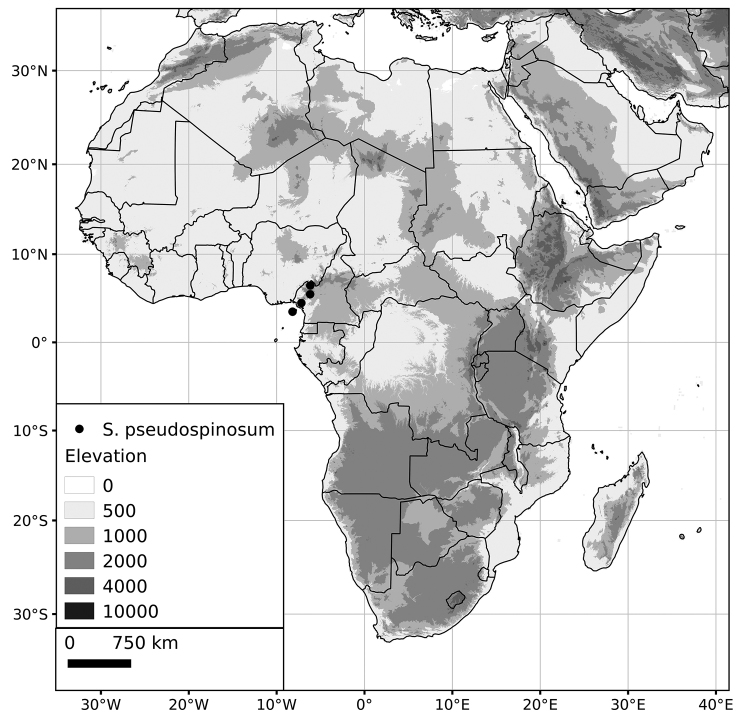
Distribution of *Solanum pseudospinosum*.

(Figure 36). Endemic to the escarpment running from the island of Bioko (Fernando Po) in Equatorial Guinea to Lake Oku in Cameroon; most collections we have seen are from the slopes of Mount Cameroon.

#### Ecology.

Grows at forest margins, in clearings and along roadsides; between 2,100 and 4,000 m elevation.

#### Common names.

Cameroon: afom.

#### Uses.

None recorded.

#### Preliminary conservation status

(IUCN 2016). *Solanum pseudospinosum* is a relatively narrow endemic with a relatively small AOO (48 km^2^, EN) and EOO (4,009 km^2^, EN; Table 7). Based on this narrow distribution and the fragmented high elevation habitat, it is given a preliminary conservation status of EN (Endangered). Most collections are from the protected area of Mount Cameroon and populations outside this national park will be vulnerable.

#### Discussion.

*Solanum pseudospinosum* is a high elevation species that, in our circumscription, only occurs along the so-called Cameroon line that is along the junction of the Congo and West African cratons (Fitton 1987). We recognise the East African plants identified as *S. pseudospinosum* by Edmonds (2012) as high elevation, particularly pubescent populations of the more widespread *S. tarderemotum*. The key characters distinguishing the two species are the pedicel that remains on the plant after fruit drop in *S. pseudospinosum*, while those of *S. tarderemotum* drop with the berries and the prominent articulations at the bases of the pedicels in *S. tarderemotum*. Other differences of *S. pseudospinosum*, but less easy to see, are the elongate, spathulate calyx lobes in fruit (those of *S. tarderemotum* are less hyaline and usually pointed) and the few-flowered congested inflorescences (as opposed to elongate, rather evenly spaced flowers along the rhachis in *S. tarderemotum*). *Solanum pseudospinosum* is also sympatric with wild populations of *S. scabrum* from which it can be distinguished by the dense spreading pubescence, the presence of stone cells in the fruit and the somewhat accrescent calyx lobes appressed to the berry.

The Cameroon line along which *Solanum pseudospinosum* occurs runs from the island of Bioko to the Tchabal Mbabo plateau and is of volcanic origin with activity beginning the Cretaceous period, but peaking in the Pliocene and continuing to the present (Wright et al. 1985). The island of Bioko is Pliocene in age (Wright et al. 1985) and has been connected and separated from the mainland several times throughout the Pleistocene. Smith et al. (2000) found only weak relationships between populations of two bird species along the Cameroon line, suggesting they evolved by allopatry. Plant diversity in this area is amongst the highest in tropical Africa (Sosef et al. 2017), but despite having within it some well-sampled units (e.g. Mount Cameroon), the northern extent of the escarpment is very poorly sampled (Sosef et al. 2017: 12, fig. 7). Better field knowledge of the distribution of *S. pseudospinosum*, coupled with better knowledge of its habitat preferences and genetic structure, will help unravel the evolutionary history of this narrowly endemic species.

*Solanum pseudospinosum* is a tetraploid species with unknown parentage (Olet et al. 2015). The species has not been included in any previous crossing and molecular studies that have focused on understanding the parental origin of the hexaploid species *S. nigrum* and *S. scabrum* (e.g. Edmonds 1977, 1979a; Ganapathi and Rao 1985, 1986b, 1986c, 1987a, 1987b; Jacoby et al. 2003; Jacoby and Labuschagne 2006; van der Walt et al. 2008; Poczai and Hyvönen 2011). It could represent one of the tetraploid parents of the hexaploids and should be included in further studies focused on understanding the origin of the polyploids.

All of the species described by Bitter (1912a) and here recognised as synonyms of *S. pseudospinosum* were described from collections housed at B that were destroyed in World War II (Vorontsova and Knapp 2010) and we have placed these in synonymy based on their very detailed descriptions. It is to be hoped that duplicates of these collections will eventually be found in herbaria not yet investigated or digitised. The differences between this species and that of C.H. Wright were very small, based on leaf size and on numbers of stone cells; we consider these minor differences insignificant.

**Specimens examined. Cameroon**. N slope of Cameroun Mountain, 7 Apr 1985, *Thomas 4642* (MO); **Nord-Ouest**: Piste Acha-Abaw au Lac Oku, 40 km NE Bamenda, 5 Dec 1974, *Letouzey 13444* (K); **Sud-Ouest**: Mt. Cameroon, above Buea, nr Hut 2, 1 Apr 1952, *Boughey GC6933* (K); Mt. Cameroon, Johann-Albrechtshöhe, 30 Jan 1962, *Breteler et al. MC39* (K); Mt. Cameroon, 9 Oct 1992, *Cheek* & *Sidwell 3664* (K, SCA, WAG, YA); Mt. Cameroon, 4 Nov 1993, *Cheek et al. 5352* (K); Mt. Cameroon, 17 Feb 1927, *Dalziel 8335* (E, K); Mt. Cameroon, nr Hut 2, Jan 1967, *Guile*, *1560* (MO); Bamenda, Southern Cameroons, Bamenda Division, Bafut-Ngemba forest reserve, 23 Feb 1958, *Hepper 2146* (K); Mt. Cameroon, beside Hut 2, 6 Apr 1937, *Hutchinson* & *Metcalfe 54* (K); Mt. Cameroon, To no 3 Hut, Jan 1931, *Maitland 1301* (K); Mt. Cameroon, Feb 1931, *Maitland 1333* (K); Mt. Cameroon, Jan 1862, *Mann 1321* (K); Mt. Cameroon, NW de Buea, 31 Mar 1981, *Meijer 15435* (MO); Mt. Cameroon, Kamerun-Berg, oberhalb Buea, 22 Dec 1928, *Mildbraed 10888 a* (K); Mt. Cameroon, Hut 2, 21 Dec 1958, *Morton 758* (K); Mt. Cameroon 2 mi W of Mann’s Spring, 29 Dec 1958, *Morton K874* (K); Mt. Cameroon, around Mann’s Spring, 3 Dec 1996, *Nning et al. 52* (K, MO, SCA, YA); Mt. Cameroon, Bokwango, 5 Oct 1992, *Thomas 9348* (K).

**Equatorial Guinea**. **Bioko**: cumbre del pico Basilé, 3 Jul 1986, *Fernández-Casas 10156B* (K, MO); Carretera del pico Basilé, 10 Jul 1986, *Fernández-Casas 10313* (BM, K); **Bioko Norte**: Pico Basilé, Distr. Barney, cruce de la Virgen de Bisila, 12 Dec 2007, *Cabezas et al. 907* (MA); Cumbre del pico Basilé, 8 Oct 1986, *do Carvalho 2544* (BM, H, K, MA, MO); Cumbre del pico Basilé, 3 Jul 1986, *Fernández-Casas 10157* (BM, K, MO); Pico Basilé, cumbre del pico, 5 Feb 1989, *Fernández-Casas 11184* (K, MA).

### 12. Solanum pygmaeum

Taxon classificationPlantaeSolanalesSolanaceae

Cav., Icon. 5: 23, tab. 439. 1799

Figures 37 , 38

Solanum pygmaeum Cav. var. hastatum Bonte ex Aellen, Ber. Schweiz. Bot. Ges. 50: 236. 1940.
Type. Argentina. Buenos Aires: Pergamino, J.A. de la Peña, 14 Jan 1925, *L.R. Parodi 6107* (lectotype, designated here: BAA [BAA00004675]).
Solanum pygmaeum Cav. var. integrifolium Bonte ex Aellen, Ber. Schweiz. Bot. Ges. 50: 236. 1940.
Type. Switzerland. Basel-Stadt: Basel, Hof der Aktienmühle, Feb 1939, *P. Aellen 3201* (lectotype, designated here: P [P03933622]; isolectotype: P [P03933624]).
Solanum pygmaeum Cav. var. sinuatodentatum Bonte ex Aellen, Ber. Schweiz. Bot. Ges. 50: 237. 1940, as “sinuato-dentatum”.
Type. Switzerland. Basel-Stadt: Basel, Hof der Aktienmühle, Feb 1939, *P. Aellen 3202* (lectotype, designated here: P [P04477345]; isolectotypes: BM [BM001035970], P [P03933623]).
Solanum pygmaeum Cav. var. latifolium Bonte ex Aellen, Ber. Schweiz. Bot. Ges. 50: 237. 1940.
Type. Hungary. Györ: Oelfabrik, 1915 and 1918, *S. Polgár s.n.* (syntypes; no duplicates located, at BP?); Germany. Neuss: Oelfabrik, 1915, *L. Bonte s.n.* (syntype; no duplicates located); Germany. Emmerich: mit Oelfrucht, 1929, *R. Scheuermann s.n.* (syntype; no duplicates located).
Solanum pygmaeum Cav. var. suspensum C.V.Morton, Revis. Argentine Sp. Solanum 138. 1976.
Type. Argentina. Córdoba: Alta Córdoba, barrio de la ciudad de Córdoba, *T. Stuckert 4713* (holotype: G [n.v.]; isotypes: CORD [CORD00004273, CORD00004274]).
Solanum deterrimum C.V.Morton, Revis. Argentine Sp. Solanum 138. 1976.
Type. Argentina. Buenos Aires: Sierra de la Ventana, 23 Feb 1944, *H. Ruíz de Huidrobo 1332* (holotype: A [00077613]; isotypes: NY [00139129], S [acc. # 12-27773], SI [SI003308, SI003307]).

#### Type.

Argentina. Buenos Aires: “in Pampas de Buenos Ayres esquina de Ballesteros”, Sep, *L. Née, s.n.* (lectotype, designated by Knapp 2007, pg. 200: MA [MA-476361!]; isolectotype: G [G00357891]).

#### Description.

Perennial small upright herbs up to 30 cm tall, subwoody at base, perennating via underground rhizomes. Stems decumbent or ascending, delicate, terete or somewhat angled with ridges, not markedly hollow; new growth pubescent with simple, appressed, uniseriate, translucent, eglandular trichomes, these 1–6-celled, 0.2–0.5 mm long or nearly glabrous; older stems glabrous or glabrescent. Sympodial units difoliate, the leaves not geminate. Leaves simple, 1.0–5.0 cm long, 0.5–3 cm wide, ovate to narrowly elliptic, pale green, concolorous, without smell; adaxial surface glabrous or sparsely pubescent along leaf lamina and margins with simple, uniseriate trichomes like those on stem; abaxial surface sparsely pubescent with similar trichomes but the pubescence denser along the midrib; major veins 3–4 pairs; base attenuate, decurrent on the petiole; margins sinuate to entire; apex acute to obtuse; petioles 0.5–1.7 cm long, with scattered simple, appressed, uniseriate eglandular trichomes like those on stem. Inflorescences 1–3 cm long, generally internodal, simple or rarely furcate, umbelliform to sub-umbelliform, with (2-)4–6 flowers clustered at the tip, glabrous or with scattered simple, appressed, uniseriate eglandular trichomes like those on stem; peduncle (1.3-)1.5–2.6 cm long, delicate; pedicels 6–13 mm long, 0.5–1 mm in diameter at the base, ca. 1 mm in diameter at the apex, straight and spreading, articulated at the base; pedicel scars spaced ca. 0–2.5 mm apart. Buds globose to broadly ovoid, the corolla strongly exserted from the calyx tube but only halfway exserted beyond the elongate and reflexed calyx lobes before anthesis. Flowers 5-merous, all perfect. Calyx tube (0.5-)1.7–2.0(-2.2) mm long, conical, the lobes 1.5–1.8 mm long, 0.7–0.9 mm wide, narrowly elliptic with long-acuminate to acute apices, glabrous to sparsely pubescent with simple uniseriate eglandular trichomes like those on stem. Corolla 9–16 mm in diameter, white to pale lilac with a yellow-green central portion near the base, stellate, lobed 1/2 way to the base, the lobes 5.0–6.7 mm long, ca. 3.0–3.5 mm wide, strongly reflexed at anthesis, later spreading, glabrous to sparsely pubescent abaxially with simple uniseriate trichomes like those of the stem but shorter. Stamens equal; filament tube minute; free portion of the filaments 1.0–1.2 mm long, adaxially pubescent with tangled uniseriate 4–9-celled eglandular trichomes to 0.5 mm long; anthers (3.0-)3.5–3.8 mm long, 0.7–1.0 mm wide, oblong-ellipsoid, yellow, poricidal at the tips, the pores lengthening to slits with age and drying. Ovary globose, glabrous; style ca. 6.3 mm long, densely pubescent with (1-)2–3-celled simple uniseriate trichomes in the lower 4/5, exserted 1–2 mm beyond the anther cone; stigma capitate to clavate, bilobed, minutely papillate, green in live plants. Fruit a subglobose berry, 8–10 mm in diameter, greyish-green at maturity, the pericarp opaque and glaucous; fruiting pedicels 12–15 mm long, ca. 1 mm in diameter at the base and at the apex, deflexed and often somewhat curved, dropping with mature fruits, not persistent; fruiting calyx not accrescent, the tube ca. 1 mm long, the lobes 1.5–2 mm long, appressed against the berry. Seeds >50 per berry, 1.8–2.0 mm long, 1.2–1.4 mm wide, flattened and tear-drop shaped with a subapical hilum, pale yellow, the surfaces minutely pitted, the testal cells irregularly quadrate in outline. Stone cells 6–8, the 2 apical ones 1.5–2 mm in diameter, usually very closely paired, the rest equatorial and 1–1.2 mm in diameter, pale whitish-brown. Chromosome number: *2n*=2x=24 (one individual with n=18 and chromosomal anomalies with supernumerary bivalents or univalents not segregating; Moscone 1992).

**Figure 37.**
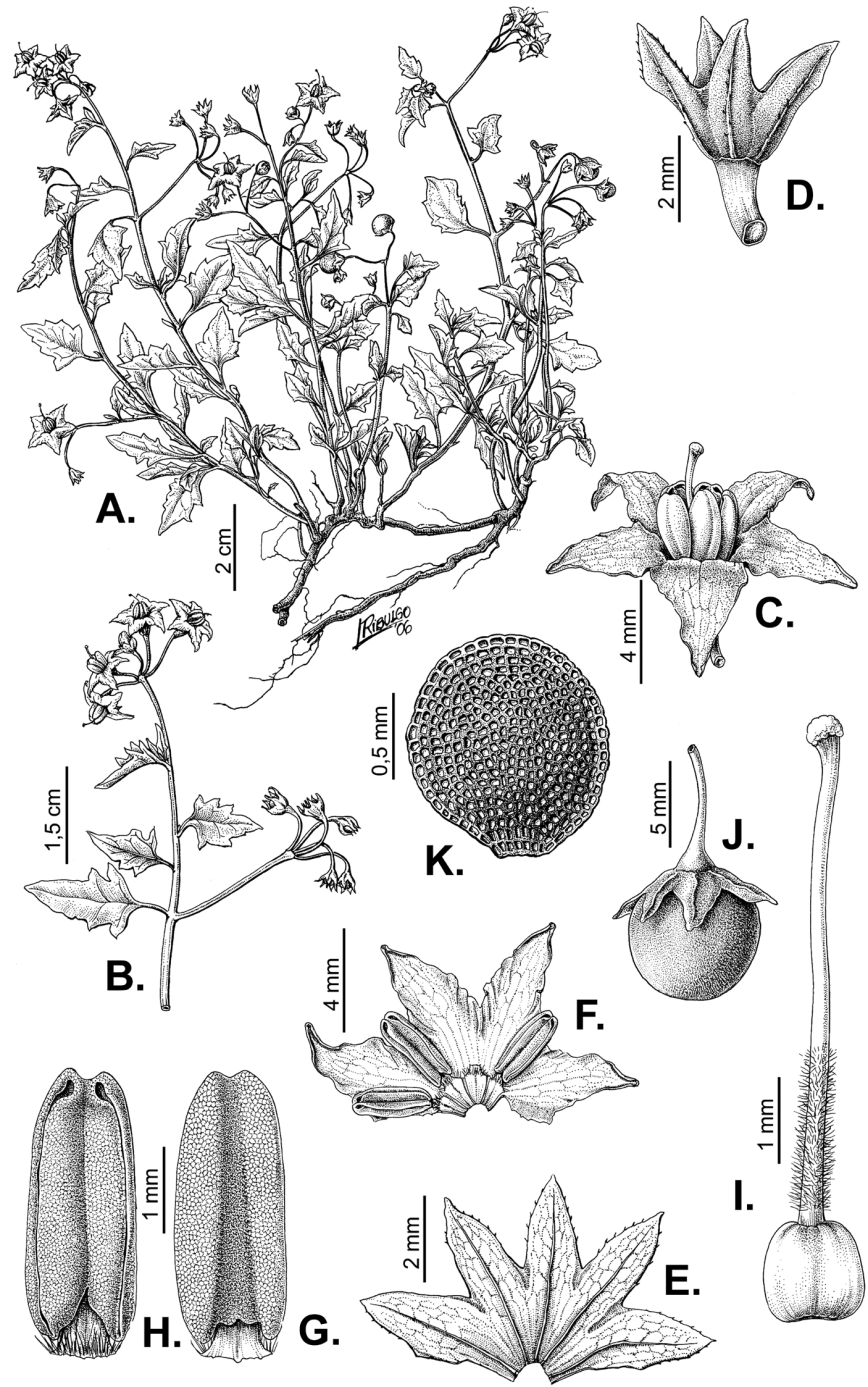
*Solanum pygmaeum*. **A** Habit **B** Flowering branch **C** Calyx **D** Dissected calyx, adaxial surface **E** Flower **F** Dissected flower **G** Stamen, dorsal view **H** Stamen, ventral view **I** Gynoecium **J** Fruit **K** Seed (**A–K** *Bernardello 476*). Drawing by L. Ribulgo.

**Figure 38.**
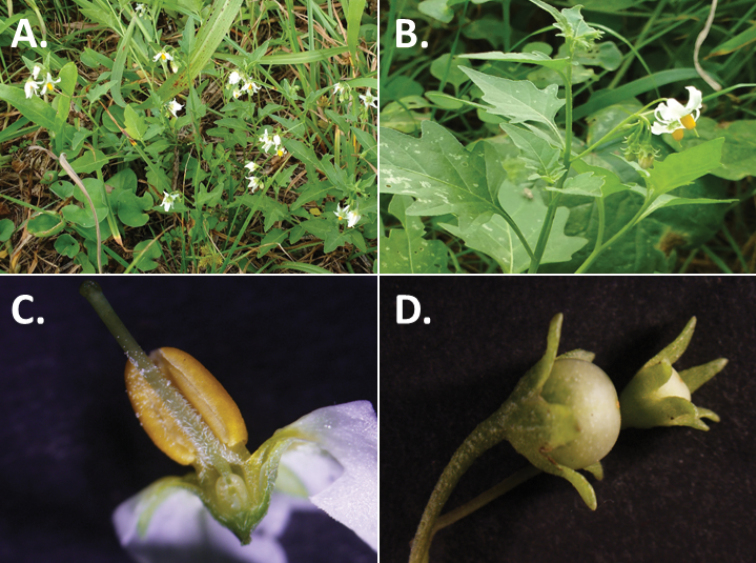
*Solanum pygmaeum* **A** Habit **B** Flowering branch **C** Dissected flower **D** Developing fruits (**A–D** *Chiarini 1341*). Photos by F. Chiarini.

#### Distribution

**Figure 39.**
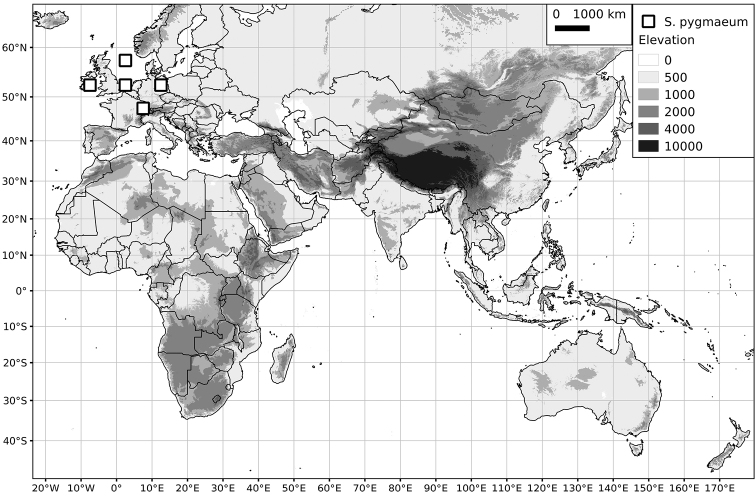
Distribution of *Solanum pygmaeum* in its non-native range in the Old World.

(Figure 39). Native to central and coastal Argentina; a few specimens known from Europe arriving as seeds through wool shipments but these are not usually established as permanent populations.

#### Ecology.

Grows in sandy and clay soils, along rail road tracks and road sides; between 100 and 1,000 m elevation in its native range, but brought only sporadically to Europe and North America via wool shipments and trade to ports at sea level and has not naturalised widely.

#### Common names.

None recorded in Old World.

#### Uses.

None recorded.

#### Preliminary conservation status

(IUCN 2016). *Solanum pygmaeum* is widespread in its native range and can be assigned a preliminary status of LC (Least Concern; Table 7). Populations from the Old World are relatively ephemeral. Within the native range in South America, *S. pygmaeum* still has a relatively large EOO of 700,962 km^2^.

#### Discussion.

*Solanum pygmaeum* is only occasionally collected in Europe, usually associated with wool waste. It is a plant that spreads by underground stems, so it is surprising that it has not become established; we include the species here so that old herbarium specimens can be identified and so that the species can be spotted if it does become established as climate changes.

The species is easy to distinguish from all other morelloids occurring in Europe by its large flowers (anthers > 3mm long), narrowly elliptic calyx lobes (1.5–1.8 mm long) and rhizomatous habit. Leaves are quite variable in size, but are usually narrowly elliptic, less often wider in the lower half.

We have selected the P duplicates that were part of the “Société cénomane d’exsicccata” as the lectotypes of var. integrifolium and var. sinuatodentatum because they are well-preserved and have both flowers and young fruits. The lectotype we have selected for var. hastatum is in an Argentinian herbarium (see best practice as outlined in Smith and Figueiredo 2011) and we have been unable as yet to trace original material of var. latifolium.

#### Selected specimens examined.

**France**. **Provence-Alpes-Côte d’Azur**: Hyères, *Fleming 306* (BM).

**United Kingdom. England**: Cornwall, Phillack Towans, Hayle, 12 Sep 1929, *Foggitt s.n.* (BM); Greater London, Winchmore Hill, 9 Sep 1920, *Herb. Hall s.n.* (BM); Greater London, Edgware, 22 Aug 1950, *Hill s.n.* (BM); Greater London, Southall, 14 Aug 1945, *Kent s.n.* (BM); Greater London, Hanwell, 3 Sep 1947, *Kent* & *Sandwith 3423* (K); Middlesex, 18 Aug 1945, *Lousley 4710* (BM); Hayle Towans, West Cornwall, 21 Sep 1929, *Melville* & *Smith 2922* (BM, K); Norfolk, Blickling, 3 Sep 1921, *Robinson s.n.* (BM).

### 13. Solanum retroflexum

Taxon classificationPlantaeSolanalesSolanaceae

Dunal, Prodr. [A. P. de Candolle] 13(1): 50. 1852

Figures 40 , 41

Solanum retroflexum Dunal var. angustifolium Dunal, Prodr. [A. P. de Candolle] 13(1): 50. 1852.
Type. South Africa. Eastern Cape: Graaff Reinet (“Graafreynet”), 3000-4000 ft, 1838, *J.F. Drège 7864b* (holotype: G-DC [G00144331]; isotypes: K [K000414172], S [acc. # S-G-5707]).
Solanum retroflexum Dunal var. latifolium Dunal, Prodr. [A. P. de Candolle] 13(1): 50. 1852.
Type. South Africa. Western Cape: Paarlberg, 1000-2000 ft, 1838, *J.F. Drège 7864a* (lectotype, designated here: G-DC [G00144331]; isolectotypes: BM [BM000943868], K [K000414175], P [P00341909]).
Solanum burbankii Bitter, Repert. Spec. Nov. Regni Veg. 12: 83. 1913, as ‘Burbanki’.
Type. Cultivated in Germany at Bremen, the seeds originally from Hamburg Botanical Garden thought to have been sent by Luther Burbank from United States of America. California, Santa Rosa (no specimens cited; no original material found); Cultivated in St. Louis, Missouri (USA) in the garden of Mr. Waldstein, seeds from Childs, 1 Sep 1909, *Anon. [Waldstein] s.n.* (neotype, designated here: MO [MO-3002957, acc. # 6651350]).
Solanum nigrum L. var. probstii Polg., Mitteil. Naturfor. Gesellsch. Solothurn 8: 71. 1928.
Type. Switzerland. Solothurn: Degend. K.K. [= Wollkompost der Kammgarnfabrik Derendingen], [in protologue “22, 24, 26, 27”], 24 Aug 1926, *R. Probst s.n.* (syntypes; no herbarium cited; lectotype, designated here: BP [acc. # 485285]).
Solanum burbankii Bitter var. glabrescens Polg., Mitteil. Naturfor. Gesellsch. Solothurn 8: 72. 1928.
Type. Switzerland. Solothurn: Degend. K.K. [= Wollkompost der Kammgarnfabrik Derendingen], [in protologue “22, 27”], 20 Oct 1922, *R. Probst s.n.* (syntypes; no herbarium cited; lectotype, designated here: BP [acc. # 485327]).

#### Type.

South Africa. Eastern Cape: Graaff Reinet (“Graafeynet”), 3000–4000 ft, 1838, *J.F. Drège 7864b* (lectotype designated here: G-DC [G00144331]; isolectotypes: K [K000414172], S [acc. # S-G-5707]).

#### Description.

Annual to perennial prostrate to erect herbs to 0.6 m tall, subwoody and branching at base. Stems sprawling, terete or ridged, 0.3–0.6 cm in diameter, if stems ridged the ridges sometimes spinescent, green to yellowish-brown, older stems straw coloured, not markedly hollow; new growth sparsely to densely pubescent with simple, spreading, uniseriate, translucent, glandular and/or eglandular trichomes, these 1–5(-8)-celled, 0.1–0.8 mm long; older stems glabrescent. Sympodial units difoliate, the leaves not geminate. Leaves simple, (0.5-) 1.5–7.5 cm long, 1.5–5.5 cm wide, rhomboidal to lanceolate, membranous, green, slightly discolorous, without smell; adaxial surface green, sparsely to densely pubescent with simple uniseriate trichomes like those on stem evenly spread along lamina and veins; abaxial surface slightly paler, more densely pubescent along veins and lamina; major veins 3–7 pairs; base truncate then abruptly attenuate along the petiole; margins shallowly toothed, the teeth rounded; apex acute, the tip sometimes rounded; petioles (0.5-) 1.5–3.5 cm long, sparsely to densely pubescent with simple uniseriate trichomes like those of the stems. Inflorescences 1.8–3.0 cm long, internodal, simple, sub-umbelliform, with 3–7 flowers clustered towards the tip of the rhachis, sparsely to densely pubescent with glandular and/or eglandular simple uniseriate trichomes like those on stems; peduncle 1.5–3.5 cm long, erect; pedicels 1.0–1.5 cm long, 0.3–0.6 mm in diameter at the base, 0.4–0.6 mm in diameter at the apex, recurving but not fully reflexed, pubescent like the peduncle, becoming woody, green or yellow-brown, articulated at the base; pedicel scars spaced 0–0.5 mm apart. Buds globose, the corolla 1/3 exserted from the calyx before anthesis. Flowers 5-merous, all perfect. Calyx tube 1.0–1.7 mm long, campanulate, the lobes equal, 1.0–1.5 mm long, less than 1 mm wide, oblong with rounded tips, green, sparsely pubescent with simple uniseriate trichomes like of the inflorescence. Corolla 11–16 mm in diameter, white, with a yellow basal star, stellate, lobed to 1/2–2/3 towards the base, the lobes 5.0–6.0 mm long, 2.5–2.7 mm wide, spreading to reflexed, densely papillate-pubescent abaxially with simple uniseriate trichomes, these denser on tips and margins. Stamens equal; filament tube minute; free portion of the filaments 1.2–1.5 mm long, glabrous or adaxially pubescent with tangled 6–8-celled simple uniseriate trichomes; anthers 1.3–1.8(-2.0) mm long, 1.0–1.5 mm wide, ellipsoid, yellow, poricidal at the tips, the pores lengthening to slits with age and drying, the connective becoming brownish in dry material. Ovary rounded, glabrous; style 1.9–2.2 mm long, slightly curved, pubescent with simple uniseriate trichomes 0.2–0.5 mm long in the basal 1/3 where included in the anther cone, exserted 0.5–1.5 mm beyond anther cone; stigma capitate, the surface minutely papillate. Fruit a globose berry, 6–10 mm in diameter, purple-black at maturity, the pericarp thin, matte with a glaucous cast; fruiting pedicels 1.0–1.5 cm long, 0.4–0.6 mm in diameter at the base, 1.0–1.2 mm at apex, becoming woody, recurving to deflexed, pale green to yellow-brown, spaced 0–0.5 mm apart, not falling with the fruit, remaining on the plant and persistent on older inflorescences; fruiting calyx not accrescent, the tube 1.0–1.5 mm long, the lobes 1.5–2.0 mm long, strongly reflexed. Seeds (5-)12–35 per berry, 1.6–1.8 mm long, 1.3–1.5 mm wide, flattened and tear-drop shaped with a subapical hilum, yellow to brown, the surfaces minutely pitted, the testal cells rectangular to pentagonal in outline. Stone cells absent. Chromosome number: *2n*=4x=48 (Henderson 1974; Randell and Symon 1976; Edmonds 1977, 1983; Symon 1981; Jacoby and Labuschagne 2006).

**Figure 40.**
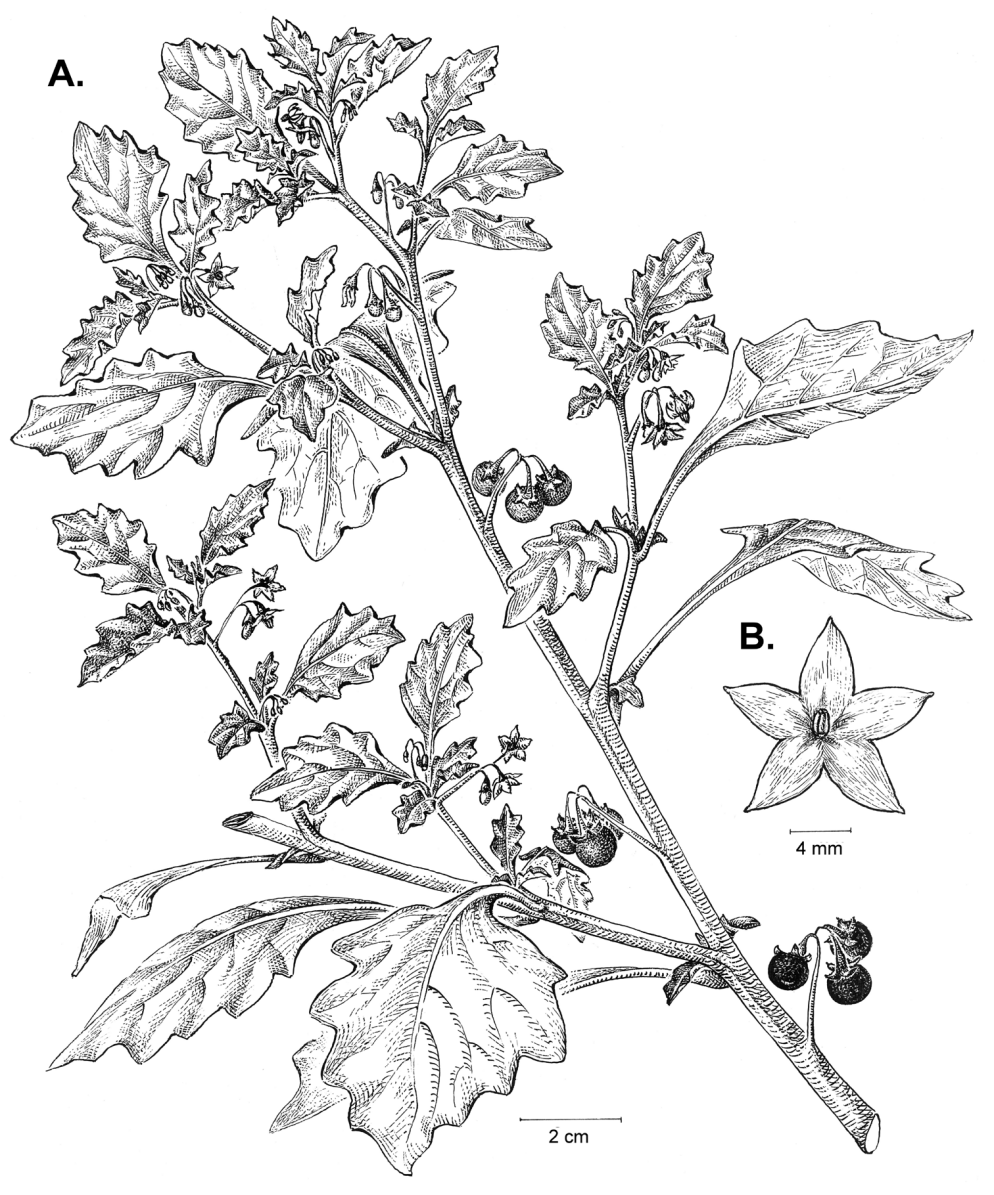
*Solanum retroflexum* **A** Habit **B** Flower. Drawing by M.L. Szent-Ivany, first published in Symon (1981), courtesy of the Board of the Botanic Gardens and State Herbarium (Adelaide, South Australia), reproduced with permission.

**Figure 41.**
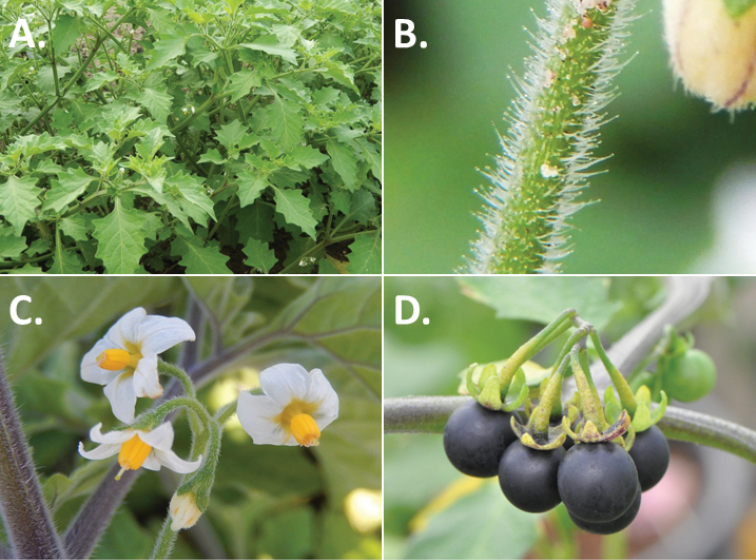
*Solanum retroflexum* **A** Habit **B** Glandular indumentum present in some individuals **C** Flowers **D** Mature, slightly ovoid dull black-purple fruits with reflexed calyx lobes (**A, C** Nijmegen acc. A1450022; **B** Nijmegen acc. 944750163; **C** Nijmegen acc. A14750023; **D** Nijmegen acc. A14750025). Photos by S. Knapp and G. van der Weerden.

#### Distribution

**Figure 42.**
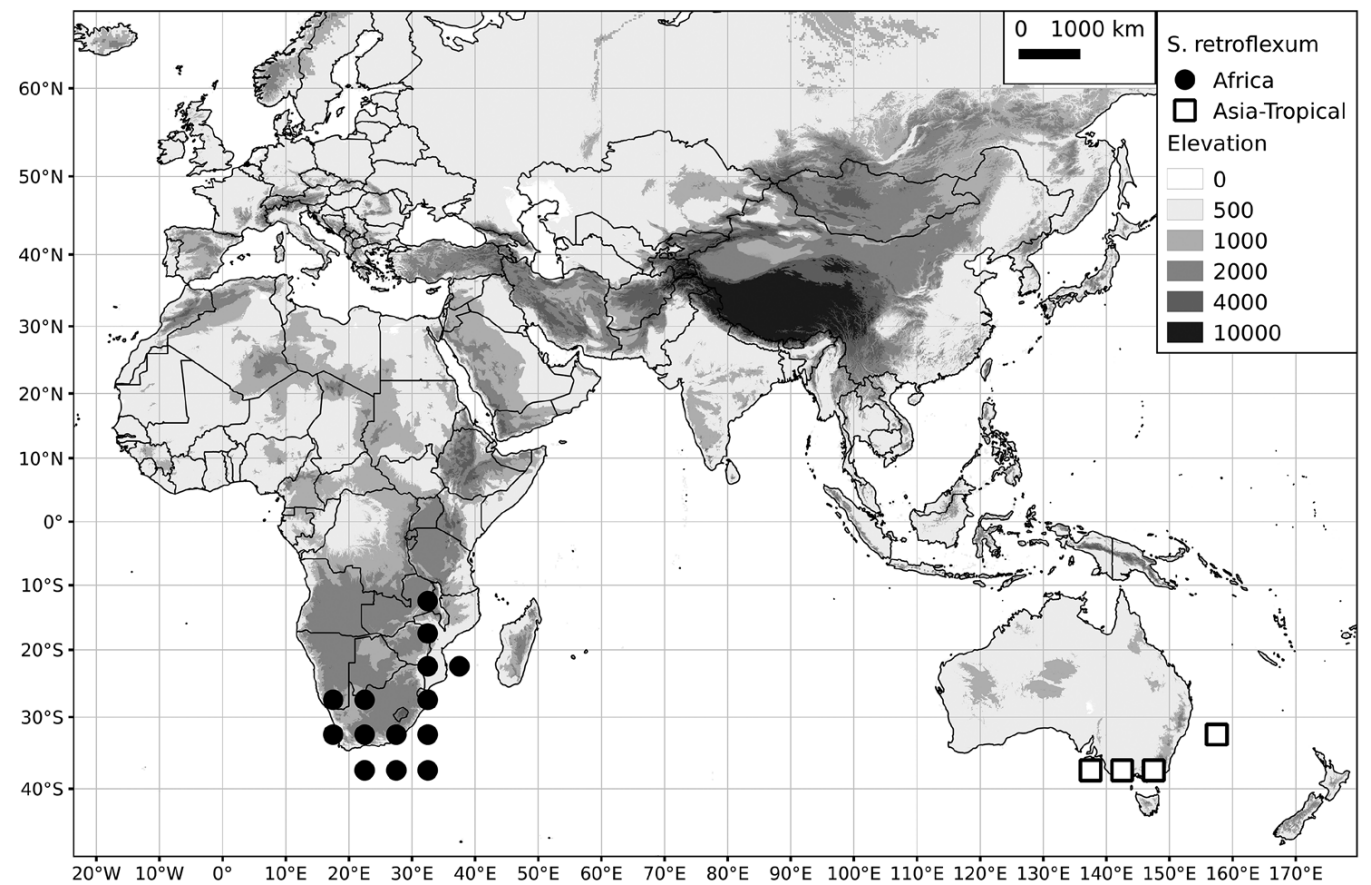
Distribution of *Solanum retroflexum* in the native range in Southern Africa (black circles) and in its non-native range (white squares).

(Figure 42). Endemic to southern Africa but has been introduced to Australia and was introduced as a garden plant to North America in the early 20^th^ century and now globally available through commercial seeds from online sources under the name “Garden Huckleberry”.

#### Ecology.

Grows in disturbed soil at watering holes, along dry watercourses, in shady places, in long grass, in *Euphorbia candelabrum*-*Buxus* zone, in dry hillsides and in disturbed areas along roads; between sea level and 1,800 (-2,300) m elevation.

#### Common names.

South Africa: mofhswe, nastegaal/nastergal, umsobo, gsoba, msoba (Heiser 1969); Zimbabwe: m’Sungula, muSungu sungu.

#### Uses.

Berries used raw and for jam (Viljoen 2011); leaves eaten as a pot-herb vegetable.

#### Preliminary conservation status

(IUCN 2016). *Solanum retroflexum* is widespread and can be assigned a preliminary status of LC (Least Concern; Table 7). Taking into account only collections from within the native range in Africa, the EOO is still very large (2,929,097 km^2^) and the assessment does not change.

#### Discussion.

*Solanum retroflexum* is a species that shows great variation in its indumentum, varying from nearly glabrous to densely pubescent with either eglandular or glandular trichomes. AFLP studies have shown that specimens of *S. retroflexum* with different indumentum types are genetically highly similar (Manoko 2007, as *S. hirsutum* (Vahl) Dunal, here recognised as a synonym of *S. memphiticum*). Developmental studies have shown that papillate glandular hairs are present on all young parts of *Solanum* plants (Seithe 1962, 1979; see Morphology above), suggesting that, in the absence of other distinguishing features, glandular pubescence might not be taxonomically significant. We circumscribe *S. retroflexum* here to include both eglandular and glandular populations; this character is polymorphic in most species of morelloids in the Old World, perhaps due to genetic lability due to polyploidy.

The species can be distinguished from other Old World morelloids based on the combination of characters of few-flowered inflorescences with 3–7 flowers, relatively long filaments (1.2–1.5 mm long) compared to anther length (1.3–1.8(-2.0) mm), strongly reflexed calyx lobes in fruit and matte purple-black berries that lack stone cells and drop without the pedicels. Leaf shape is generally rhomboidal, which is characteristic of the species and helpful in distinguishing it from closely related *S. nigrum* and *S. villosum*. Calyx lobes are generally longer than in *S. villosum*, *S. scabrum* or *S. nigrum*. It can be distinguished from the morphologically similar *S. villosum* based on its opaque purple-black fruit, its pedicels that remain behind after fruits drop, deeply stellate corolla with long corolla lobes and the long filaments as compared to anther length.

*Solanum retroflexum* is cultivated commercially in South Africa in KwaZulu-Natal and Free State for jam production (Viljoen 2011) and farmers select individual plants with large berries to propagate for the next season. In one farm in KwaZulu-Natal, 10 tonnes of berries harvested were used to make 20,000 jars of “Umsobo” jam, most marketed in the Gauteng area. On other farms, *S. retroflexum* is cultivated along with *S. chenopodioides* and *S. americanum* and putative hybrid plants were found (Viljoen 2011). Leaves of *S. retroflexum* are also produced under irrigation in Limpopo province, for the commercial market in the local area (van Averbeke et al. 2007). The markets for both leaves and fruits are controlled by small scale rural traders in South Africa.

*Solanum retroflexum* is a tetraploid species with unclear parentage. Previous studies have suggested that one of the contributing parents of the tetraploid *S. retroflexum* is the diploid *S. americanum* (Edmonds 1977), supported by crossing studies showing vigorous hybrids between the autotetraploid *S. villosum* derived from *S. americanum* and *S. retroflexum* (Ganapathi 1987). Jacoby and Labuschagne (2006), however, reported that crosses of *S. chenopodioides* and *S. retroflexum* are much more successful than crosses between *S. retroflexum* and *S. americanum*. Ganapathi & Rao (1980) also reported weak chromosome pairing at low frequency in hybrids between *S. retroflexum* and *S. americanum*. Molecular studies suggest that either *S. chenopodioides* or *S. nitidibaccatum* are the likely parental species of *S. retroflexum* (Jacoby et al. 2003; van der Walt et al. 2008; Poczai and Hyvönen 2011). Further molecular studies are needed to fully establish the parental species of *S. retroflexum*, but the current data from molecular and crossing studies suggests that *S. chenopodioides* or *S. nitidibaccatum* are the likely best candidates.

*Solanum retroflexum* or one of its close relatives has been suggested to have contributed to the origin of the hexaploid *S. nigrum* (Ganapathi and Rao 1986c). Most current evidence suggests, however, that *S. villosum* and *S. americanum* were the parents of *S. nigrum* (Edmonds 1979a; Ganapathi and Rao 1986b; Poczai and Hyvönen 2011).

In describing *S. retroflexum*, Dunal (1852) cited two elements; one from Saudi Arabia (“in Arabia circa Taifa”) and the other from South Africa (“caput Bona Spei, Drege”). He then used two different collections held at G-DC to describe the varieties. The specimen from Saudi Arabia corresponds to *S. villosum* (P00055172), so we have used the flowering collection (*Drège 7864b*) at G-DC (G00144331) that is also the holotype of var. angustifolium as the lectotype of *S. retroflexum*, making the two names homotypic.

*Solanum burbankii* was described from living material sent to Bitter (“colui ipso in horto Bremeno complures per annos e seminibus ab horto Hamburgensi acceptis” [grew in the Bremen garden for several years from seeds originally accepted from Hamburg]) and thought to have been from California (Bitter 1913b) and thus, perhaps, from Luther Burbank himself. Bitter (1913b) cites no herbarium specimens in the protologue; if they were in either Bremen or Berlin, they were destroyed during the Second World War and we have found no specimens in Göttingen where some small fragments of collections are found (Vorontsova and Knapp 2010). From the description of a somewhat pubescent plant (“utrinque sparsim breviter pilosa”) with pruinose berries with no stone cells, in which the calyx lobes are strongly reflexed at fruit maturity, we are certain Bitter is describing a sparsely pubescent form of *S. retroflexum*. We have selected a neotype for *S. burbankii* from specimens grown in St. Louis, Missouri that were obtained from the original distributor of Burbank’s “wonderberry” seeds, J.L. Childs of New York. These specimens are almost certainly amongst those seen by William Trelease during his part in the newspaper war that was waged over the identity of the “wonderberry” in the early part of the 19^th^ century (see Heiser 1969) and match the description well. Several collections grown in Mr. Waldstein’s garden are held at MO and we have chosen the sheet with the best fruiting material as the neotype.

The Swiss botanist Rudolf Probst worked extensively on the adventive flora found around woollen mills in Solothurn (Switzerland) and sent much of his *Solanum* material to the Hungarian botanist Sandor Polgár, apparently both as specimens and as seeds. In his various publications (Probst 1928, 1938), he quoted verbatim from Polgár’s letters, attributing both names and descriptions to Polgár for S. nigrum var. probstii and S. burbankii var. glabrescens. The numbers he cites in the protologues apparently refer to years of collection (e.g. “22, 24, 26, 27”), but we are not certain; because of the labels on specimens in BP seen and annotated by Polgár, we are assuming Probst sent herbarium specimens rather than seeds. We are therefore considering this material as lectotypes for these two names, both of which correspond to *S. retroflexum*. The letter quoted verbatim in the protologue of S. nigrum var. probstii is mounted on a sheet with a collection date (29 Jul 1929) later than the description, so we select another Probst collection from 24 Aug 1926 and annotated by Polgár (BP acc. # 485285) as the lectotype for this name. The specimen collected by Probst on 20 Oct 1922 (BP acc. # 485327) has elements of the text repeated in the protologue attached to it and we designate this as the lectotype for S. burbankii var. glabrescens.

#### Selected specimens examined.

**Australia**. **New South Wales**: Bouddi, 1970, *Edmonds C74* (K); **Queensland**: Somerset, Kentville, 28 Oct 1965, *Henderson 123* (BRI, K); **South Australia**: Lower Eyre Peninsula, Sec[tion] 10, Hundred of Flinders, 13 Nov 1966, *Alcock 1268* (AD); Pilli Waterhole, sect. 10, Hundred of Flinders, 27 Apr 1968, *Alcock 2099* (AD, CANB, K); **Victoria**: Mildura, Hattah/Kulkyne National Park, 11 Feb 1989, *Browne s.n.* (AD).

**Botswana**. Livingstone’s Cave, Melepelele, 35 mi NW of Gaberone, 21 Apr 1974, *Mott 236 C* (K).

**Lesotho**. **Leribe**: LHDA Phase 1A, 13 Jan 1996, *Phillipson 4757* (MO); Basutoland, *Dieterlen 157* (BM, K); Kolonyama plateau W border, 4 Oct 1969, *Williamson 48* (K).

**Malawi**. **Southern**: Blantyre, Ndirande Mountain, 2 May 1970, *Brummitt 10323* (K);

**Mozambique**. **Zambezia**: Mocuba, ao km 40, estrada de Milange, 18 Mar 1943, *Torre 4957* (MO);

**Namibia**. Erongo Mountains, 28 Jun 1916, *Pearson 9834* (K); **Khomas**: Windhuk Bergland, regio Finkenstein, 13 Dec 1963, *Seydel 3783* (A, K, MO).

**South Africa. Eastern Cape**: turnoff to Carlisle Bridge from Grt Riebeek East Rd, Grahamstown grid, 31 Mar 1974, *Bayliss BRI-B-761* (A, K); Annes Villa, Zuurberg, 2 Oct 1975, *Bayliss 7182* (A, MO); Graaff Reniet, *Bolus 50* (F, K); SA Nr camp site 1 km E of Steilkop, New Agatha Forest Reserve, 21 Apr 1971, *Scheepers 54* (EA); **Free State**: Fauresmith, Apr 1937, *Henrici 3080* (K); Mequatling’s Nek, Apr 1972, *Jacobs 8533* (K); **Gauteng**: Pretoria, Carlisle Bridge, Albany, 2 Apr 1978, *Bayliss 8679* (MO); Burttholm, Verreniging, 25 Apr 1918, *Burtt Davy 17658* (K); Magaliesberge, Pretoria District, 8 Jun 1955, *Schlieben 7009* (F, K, MO); **KwaZulu-Natal**: 2829 (Harrismith) Drakensberg, Royal Natal National Park, 19 Jan 1987, *Goldblatt* & *Manning 8416* (MO); Drakensberg Range, Tugela Valley & Mont-aux-Source, 3 Apr 1934, *Humbert 14849* (MA); Distr. Alexandra, Station Dumisa, Campbellson, 17 Dec 1912, *Rudatis 1805* (W); Ngome, 15 Dec 1969, *Strey 9409* (E, EA, K); **Limpopo**: Duiwelskop, grid no. 3219CA, Clamvillham District, 15 Oct 1983, *Bean 1366* (MO); New Agatha Forest Reserve, nr campsite 1 km E of Steilkop, 21 Apr 1971, *Múller* & *Schespere 54* (K); **Mpumalanga**: Ermelo, Nooitgedacht Research Station, 19 Jan 1976, *Balsinhas 2901* (K, MO); Namaqualand, Klipfontein, 23 Dec 1949, *MacDonald 117* (BM); Bei der Stadt Lydenburg, Mar 1884, *Wilms 1022 a* (BM, E); **North West**: head of Helskloof, Hottentosparadyskloof, 28 Aug 1977, *Thompson & Le Roux 134* (K); Magaliesberg, Jackson’s kiln, 28 Feb 1957, *Vueeden 97* (K); **Northern Cape**: Westeljike bellings van Rebunie, Calvinia, 27 Dec 1977, *Hanekom 2499* (K); Namaqualand, Ai-Ansi, 1 Jan 1950, *MacDonald 137* (BM); **Western Cape**: Pro Spei (Cape of Good Hope), 1771, *Banks* & *Solander s.n.* (BM); Nr drift of Cayman’s River, George, *Barkly s.n.* (BM); Table Mountain, *Burchell 856* (K); Simon’s Bay, Cape of Good Hope, 1853, *Wright s.n.* (GH).

**Swaziland**. Ukulula, Mbabane Dist, 13 Mar 1955, *Compton 25011* (K).

**Zambia**. **Central**: **Copperbelt**: Kitwe, 15 Jan 1960, *Fanshawe*, *F-5353* (K); **Northern**: Kaloswe, Koloswe, 16 Jul 1930, *Hutchinson* & *Gillett 3767* (K); **Zimbabwe**. Owelo Teachers College, 11 Nov 1966, *Biegel 1413* (K); Vumba, Clouds Downs, 29 Feb 1960, *Head 170* (BM); sin. loc, *Hislop Z-155* (K); Bulawayo, Jan 1898, *Rand 171* (BM); **Bulawayo**: Bulawayo, garden, 9 Oct 1946, *Best 499* (K); **Harare**: Harare, Salisbury Expt. Station, 26 May 1943, *Arnold 10206* (K); Harare, Salisbury Botanic Garden, 30 Jan 1969, *Muller 743* (K); **Manicaland**: Umtali, Nuza plateau, Mutasa Distr., Oct 1934, *Gilliland 974* (BM, K); Nyanga, Inyanga Distr., 11 Jan 1931, *Norlindh* & *Weimarck 4199* (BM, MO); **Mashonaland Central**: Mazowe, southern Rhodesia, Mazoe, Umvukwes, Ruorka Ranche, 17 Dec 1952, *Wild 3961* (MO); **Mashonaland East**: Pasture Research Station, Marandellas, 28 Mar 1934, *Brain 10574* (MO); **Masvingo**: Victoria, Rhodesia, 1909, *Monro 1024* (BM); **Matabeleland North**: Makoholi Experiment Station, Victoria District, 13 Mar 1978, *Senderayi 226* (K); **Matabeleland South**: Matobo, Farm Beana, Kobila, Apr 1957, *Miller 4341* (K); Gwanda, Tuli Experimental Station Reservior in pasture block, 16 Jan 1965, *Norris-Rogers 599* (K); **Midlands**: Sable Park, QueQue District, 13 Mar 1978, *Chipunga 168* (MO).

### 14. Solanum sarrachoides

Taxon classificationPlantaeSolanalesSolanaceae

Sendtn., Fl. Bras. (Martius) 10: 18, tab. 1, fig. 1–8. 1846

Figures 43 , 44

Solanum justischmidtii E.H.L.Krause, Deutschl. Fl. (Sturm), ed. 2, 10: 72. 1903.
Type. Germany. Hamburg: Hamburg, *J. Schmidt s.n.* (lectotype, designated by Edmonds 1986, pg. 17: HBG [n.v.]).
Solanum sarachidium Bitter, Repert. Spec. Nov. Regni Veg. 11: 211. 1912.
Type. Paraguay. "Gran Chaco: Loma Clavel", Nov 1903, *T. Rojas 2493* (lectotype, designated by Edmonds 1986, pg. 17: BM [BM000087577]) ; isolectotype: G [G00306752].
Solanum sarrachoides Sendtn. var. sarachidium (Bitter) C.V.Morton, Revis. Argentine Sp. Solanum 122. 1976.
Type. Based on Solanum sarachidium Bitter

#### Type.

Brazil. “Brasilia australis”, *F. Sellow s.n.* (lectotype, designated by Edmonds 1986, pg. 16: P [P00371162]).

#### Description.

Annual erect to decumbent herbs 30–70 cm tall, rarely to 1 m, somewhat branching at base. Stems sprawling, terete, green, not markedly hollow; new growth densely viscid-pubescent with simple, spreading, uniseriate, translucent, glandular trichomes, the trichomes of two lengths, the shorter 1–4-celled, 0.2–0.5 mm long and the longer 5–14-celled, 1–2 mm long, both with glandular apical cells; older stems glabrescent. Sympodial units difoliate, the leaves not geminate. Leaves simple, 3.0–7.5 cm long, 3.0–6.0 cm wide, broadly ovate, thin and membranous, concolorous, without smell; adaxial and abaxial surfaces sparsely to densely pubescent with spreading, simple, uniseriate glandular trichomes like those of the stem, evenly distributed on lamina and veins; major veins 3–4 pairs; base truncate to cordate, sometimes asymmetric; margins entire or regularly sinuate-dentate; apex acute; petioles 0.5–3.2 cm long, sparsely pubescent with trichomes like those of the stem and leaves. Inflorescences 0.7–1.7 cm long, usually leaf-opposed but occasionally internodal, simple, sub-umbelliform, with 2–5(-7) flowers clustered at the tip, sparsely pubescent with spreading trichomes like those of the stems; peduncle 0.7–1.0 cm long, straight; pedicels 5–7 mm long, 0.1–0.2 mm in diameter at the base, 0.3–0.4 mm in diameter at the apex, straight and spreading, articulated at the base; pedicel scars spaced ca. 0(-1) mm apart. Buds globose, the corolla included within the calyx lobes and only the tip of the bud showing. Flowers 5-merous, all perfect. Calyx tube 0.5–1.0 mm long, conical, the lobes 1.5–2.0 mm long, 0.5–0.7 mm wide, lanceolate to narrowly ovate with acute apices, sparsely pubescent with 1–4-celled spreading glandular trichomes like those on the pedicels but shorter. Corolla 5–8 mm in diameter, white with a yellow-green central eye, pentagonal-stellate, lobed 1/2–1/3 of the way to the base, the lobes 3.0–4.5 mm long, 5.0–7.0 mm wide, spreading at anthesis, sparsely papillate-pubescent abaxially with glandular 1–4-celled simple uniseriate trichomes and eglandular papillae, the denser along margins, tips and midvein. Stamens equal; filament tube minute; free portion of the filaments 1.0–1.5 mm long, adaxially sparsely pubescent with tangled uniseriate 4–6-celled simple trichomes; anthers 1.2–2.0 mm long, 0.4–0.8 mm wide, ellipsoid, yellow, poricidal at the tips, the pores lengthening to slits with age and drying. Ovary globose, glabrous; style 3.0–3.5 mm long, densely pubescent with 2–3-celled simple uniseriate trichomes in the lower 1/2–2/3 where included in the anther cone, not usually exserted beyond the anther cone; stigma capitate, minutely papillate, green in live plants. Fruit a globose berry, 6–9 mm in diameter, green-brownish grey at maturity, the pericarp thin and dull to somewhat shiny; fruiting pedicels 5–9 mm long, 0.2–0.3 mm in diameter at the base and at the apex, spaced 0–1 mm apart, reflexed, dropping with mature fruits, not persistent; fruiting calyx accrescent, becoming papery in mature fruit, the tube 3–4 mm long, the lobes 5.5–8.0 mm long and 3.5–4.0 mm wide, the tips slightly reflexed or spreading. Seeds (23-)59–69(-93) per berry, 1.3–1.7 mm long, 1.0–1.5 mm wide, flattened and tear-drop shaped with a subapical hilum, pale yellow, the surfaces minutely pitted, the testal cells pentagonal in outline. Stone cells 4–6 per berry, (0.5) 0.8–1 mm in diameter. Chromosome number: 2*n*=2*x*=24 (Edmonds 1972, 1977; Bukenya 1996; Moyetta et al. 2013; Olet et al. 2015).

**Figure 43.**
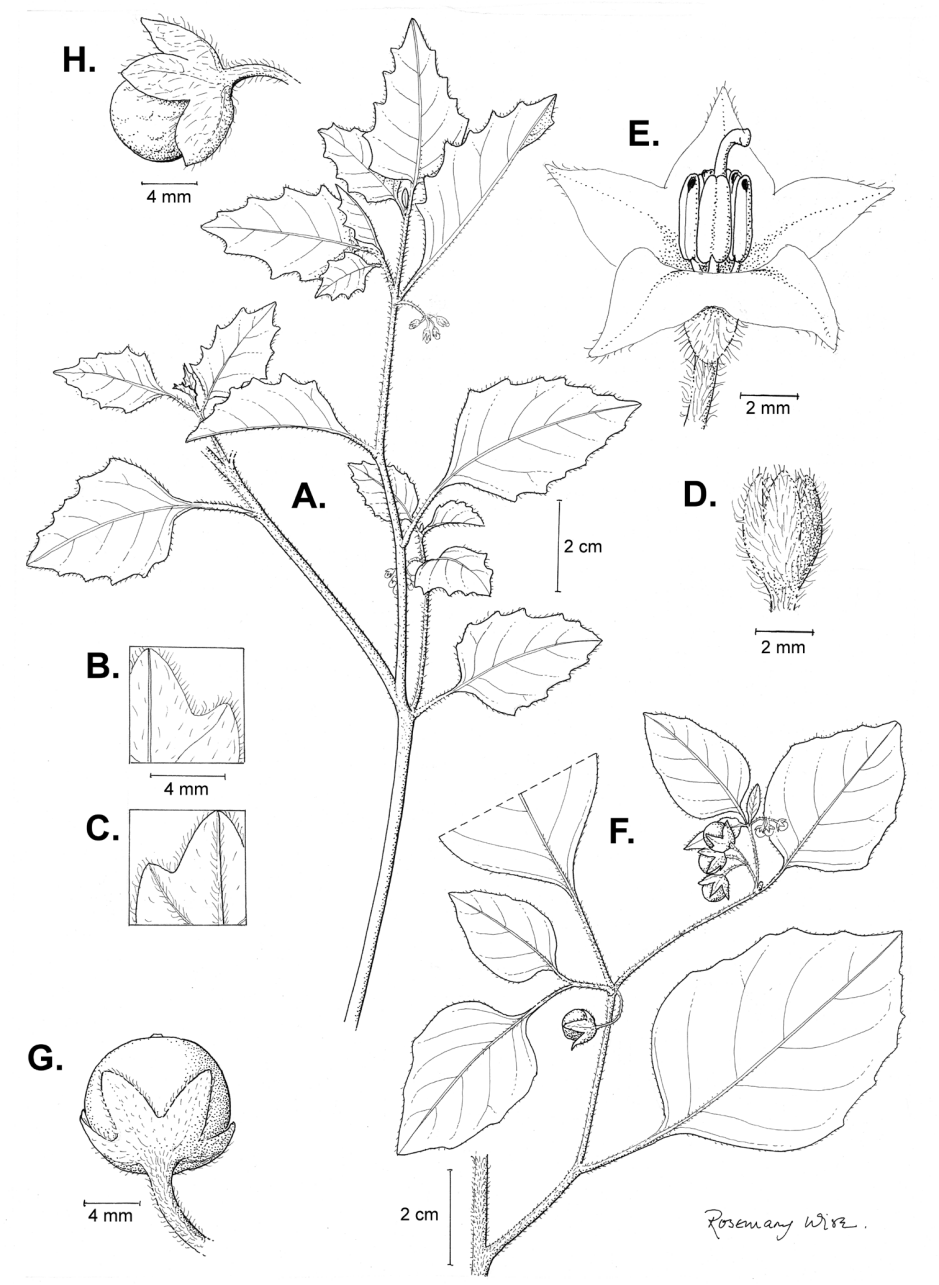
*Solanum sarrachoides* **A** Habit **B** Detail of adaxial leaf surface **C** Detail of abaxial leaf surface **D** Bud **E** Flower **F** Fruiting habit **G** Maturing fruit (**A–E** *Macoun s.n.*; **F–G** *Ahles 55038*). Drawing by R. Wise.

**Figure 44.**
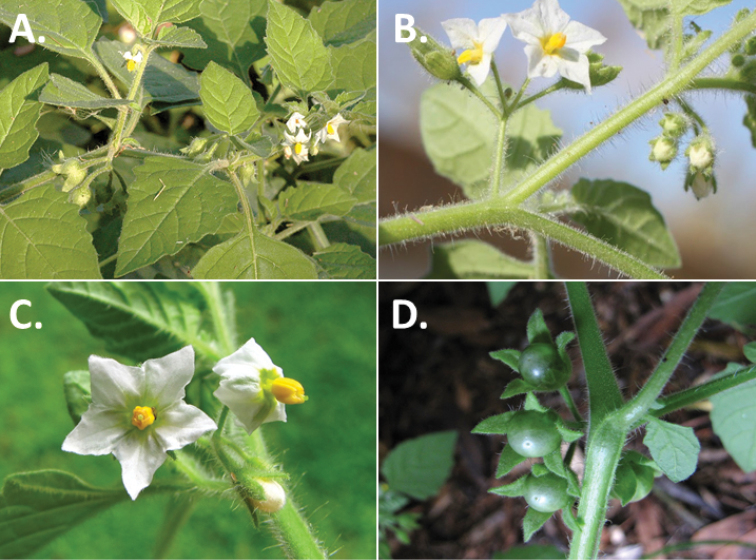
*Solanum sarrachoides* **A** Habit **B** Inflorescence **C** Flowers at full anthesis **D** Developing fruits (unvouchered photos). Photos by D.G.Smith, S. Martín de la Vega, and B.W.Wells Association.

#### Distribution

**Figure 45.**
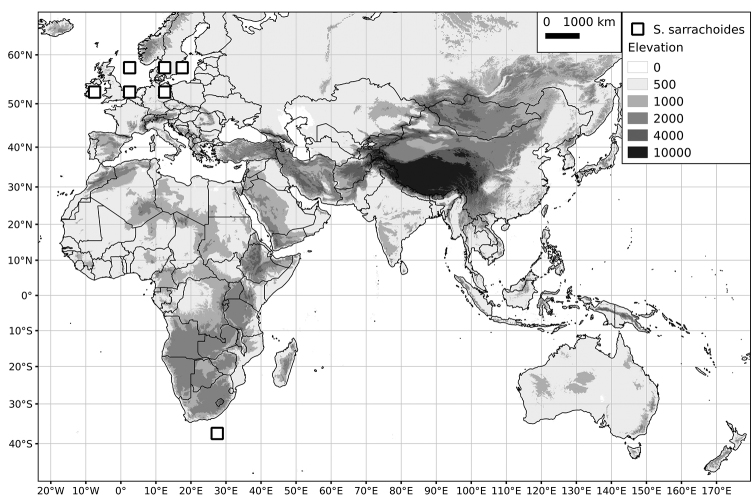
Distribution of *Solanum sarrachoides* in its non-native range in the Old World.

(Figure 45). Native to southern South America, but has been introduced globally as an agricultural weed.

#### Ecology.

Grows in urban areas, along riversides and other disturbed areas; between sea level and 2,300 m elevation in its native range, between sea level and 800 (1,400) m in the introduced range.

#### Common names.

South Africa: umrobe wezinja; Sweden: Klibbnattskatta (Mossberg et al. 2003); United Kingdom: leafy-fruited nightshade (Stace 2010).

#### Uses.

None recorded.

#### Preliminary conservation status

(IUCN 2016). *Solanum sarrachoides* is not a weedy species and has not spread far despite various introductions across the world; in its native range, it is widespread (EOO 2,089,288 km^2^) and can be assigned a preliminary status of LC (Least Concern; Table 7).

#### Discussion.

*Solanum sarrachoides* is morphologically similar to *S. nitidibaccatum*. The two taxa can be distinguished based on generally truncate leaf bases, leaf-opposed inflorescences that are umbellate to sub-umbellate with fewer flowers (2–5, rarely 6–7), shorter calyx lobes 1.5–2.0 mm long and a corolla with yellow-green central eye in *S. sarrachoides*, compared to *S. nitidibaccatum* which has attenuate to cuneate leaf bases, internodal inflorescences that are racemose with more flowers (4–8, occasionally up to 9–10), longer calyx lobes 1.7–2.5 mm long and a corolla with yellow-green central eye with black-purple “V”- or “U”-shaped margins. The buds of *S. sarrachoides* are included in the calyx until just before anthesis and the berries are usually matte instead of shiny as they are in *S. nitidibaccatum*.

*Solanum sarrachoides* is a diploid species native to north-eastern and central Argentina, Paraguay and southernmost Brazil. The introduction of the species to Europe and North America is largely due to trade with South America and the importation of seeds and grain together with the practice of spreading wool waste ('shoddy') as manure and, based on herbarium records, seems to have been introduced some time at the beginning of the 20^th^ century. Despite its introduction in the early 1900s, the species remains relatively uncommon in both Europe and North America, with sporadic records from elsewhere, including South Africa (e.g. Eastern Cape, *Phillipson & Hobson 5256*). *Solanum sarrachoides* has not spread to Australasia; all literature records (e.g. Healy 1974; Henderson 1974; Ogg et al. 1981; Schilling 1981; Symon 1981) suggest that material from Australasia refers to material we recognise as *S. nitidibaccatum*.

Within the Old World, *S. sarrachoides* has often been confused with *S. nitidibaccatum* and records of *S. sarrachoides* or *S. nitidibaccatum* in literature should be taken with caution due to common misidentification of voucher material. Many regional treatments do not separate between these morphologically very similar species (e.g. Stebbins and Paddock 1949).

Edmonds (1986) provides a discussion of the complex synonymy and lectotypification of *S. sarrachoides.* The species epithet is often seen spelled as “*sarachoides*”, because Sendtner was thought to have named this species after the genus *Saracha* Ruiz & Pav. (after the taxa now part of the genus *Jaltomata* Schltdl.), but in *Flora Brasiliensis* (Sendtner 1846), he used both “*Saracha*” and “*Sarracha*” and Edmonds (1986) determined that the name is correctly spelled *S. sarrachoides* following the original publication.

#### Selected specimens examined.

**Austria**. **Steiermark**: Fussee, Mistablagerumgsplatz, 26 Aug 1950, *Rechinger s.n.* (MA).

**France**. **Grand Est**: Bas-Rhin, Strasbourg, Petrolhafen in Strassburg, 18 Oct 1953, *Aellen s.n.* (W); **Nouvelle Aquitaine**: Gironde, Gironde, Bassem, 19 Sep 1924, *D’Alleizette s.n.* (W); Gironde, Bordeaux, 6 Sep 1926, *Herb. Guiol s.n.* (BM).

**Germany**. Neuft, [Speten], 20 Sep 1917, *Boutz s.n.* (B); **Hamburg**: Hamburg, bei Wandobek, Sep 1900, *Schmidt s.n.* (W); **Hessen**: Herdingen, Rheinwerfl, 26 Sep 1920, *Boutz s.n.* (B).

**South Africa. Eastern Cape**: Amatole Mountains, Elandsberg, Coolin farm, 20 Mar 1986, *Phillipson 1353* (K, MO); Rooiberge, nr Graaf Reinet Rooiskuur dam on Roodeberg Farm, 17 Dec 2000, *Phillipson* & *Hobson 5256* (K, MO).

**Spain**. **Andalucia**: Granada, Cacín, 9 Nov 1970, *Pérez Raya s.n.* (MA); **Castilla-La Mancha**: Toledo, Montalbán, embalse de Castrejón, 18 Apr 1983, *López s.n.* (MA).

**Sweden**. **Götaland**: Skåne, Malmö, Sep 1906, *Hylmö s.n.* (BM); Västra Götaland, Mölndal, Svenska Oljeslageriet, 30 Aug 1936, *Blom s.n.* (BM); Västra Götaland, Angered, Agnesbergskvarn, 31 Aug 1938, *Blom s.n.* (W); Västra Götaland, Molndal, Sveska Oljeslageriet, 30 Aug 1936, *Blom 1376* (CORD, K, W); Västergötland, Asfroued Au, Sjoliageu, 10 Sep 1966, *Westfelt s.n.* (BM).

**United Kingdom. England**: Bedfordshire, Cabbage field at Flitton, 8 Oct 1974, *Hanson 107a* (BM); Essex, Wasteground Dagenham, 2 Oct 1927, *Melville s.n.* (K); Essex, Dagenham, 6 Oct 1945, *Sandwith* & *Milne-Redhead s.n.* (BM, K); Essex, Barking Tip, 12 Sep 1953, *Welch 5298* (BM); Gloucestershire, Avonmouth Docks, 29 Aug 1922, *Polgár s.n.* (BM); Hertfordshire, Wheathampstead, 14 Oct 1962, *Dony 4165* (BM).

### 15. Solanum scabrum

Taxon classificationPlantaeSolanalesSolanaceae

Mill., Gard. Dict. ed. 8, no. 6. 1768

Figures 46 , 47

Solanum nigrum L. var. guineense L., Sp. Pl. 186. 1753.
Type. “Solanum guineense fructu magno instar cerasi nigerrimo umbellato” cultivated in England, at James Sherard’s garden in Eltham (Hortus Elthamensis) (lectotype, designated by Edmonds 1979b, pg. 224: Dillenius, Hort. Eltham. 2: 366, t. 274, f. 354. 1732).
Solanum guineense (L.) Mill., Gard. Dict., ed. 8, no. 7. 1768, nom. illeg., not Solanum guineense L. (1753)
Type. Based on Solanum nigrum L. var. guineense L.
Solanum melanocerasum All., Auct. Syn. Meth. Stirp. Hort. Regii Taur. 664. 1774.
Type. “Solanum guineense, fructo magno, instar cerasi nigerrimo, umbellato” cultivated in England at John Sherard’s garden in Eltham (Hortus Elthamensis), *Herb. Dillenius 336* (neotype, designated by Edmonds 2012, pg. 129 [as lectotype]: OXF [Dill. HE-274-234]).
Solanum guineense (L.) Lam. Tabl. Encycl. 2: 18. 1794, nom. illeg., not Solanum guineense L. (1753), Solanum guineense (L.) Mill. (1768)
Type. Based on Solanum nigrum L. var. guineense L.
Solanum triangulare Lam., Tabl. Encycl. 2: 18. 1794.
Type. “Ex Ind. Orient”, *Herb. Lamarck s.n.* (lectotype, designated by D’Arcy 1974a, pg 735 [as type]: P-LAM [P00357626]).
Solanum quadrangulare Lam. var. triangulare (Lam) Pers., Sys. 225. 1805.
Type. Based on Solanum triangulare Lam.
Solanum melanocerasum Willd., Enum. Pl. (Willdenow) 1: 237. 1809, nom. illeg., not Solanum melanocerasum All. (1773)
Type. Cultivated in Berlin Botanical Garden, from “Europa australi” [southern Europe] (no original material labelled as “melanocerasum” found in B-W; neotype, designated here: B-W [BW04368010]).
Solanum pterocaulum Dunal, Hist. Nat. Solanum 153. 1813, nom. illeg. superfl.
Type. Based on Solanum scabrum Mill. (cited in synonymy).
Solanum fistulosum Dunal, Encycl. [J. Lamarck & al.] Suppl. 3: 749. 1814.
Type. “Originaire de l’Isle de France [Mauritius], est cultivée en Amerique [Brazil]”, *Herb. Richard s.n.* (lectotype, designated by D’Arcy 1974a, pg. 735: P [P00335259]).
Solanum memphiticum Mart., Pl. Hort. Erlang. 63. 1814, nom. illeg., not Solanum memphiticum J.F.Gmel. (1791)
Type. Cultivated in Erlangen, Bavaria, Germany, origins not known (no specimens cited; no original material located, possibly at ER?).
Solanum nigrum L. var. melanocerasum (Willd.) Dunal, Solan. Syn. 12. 1816.
Type. Based on Solanum melanocerasum Willd.
Solanum nitens Opiz, Oekon.-techn. Fl. Böhm. [Berchtold & al.] 3(2): XXI. 1843.
Type. Czech Republic. Prague, “in Semubackern por dem Reuthore Prag”, *P.M. Opiz 10/840* (no herbaria cited; no original material found, perhaps at PR?).
Solanum oleraceum Dunal var. macrocarpum Dunal, Prodr. [A. P. de Candolle] 13(1): 50. 1852.
Type. Brazil. Bahia: Ilheus, 1841, *C.F.P. Martius 1255* (lectotype, designated by Edmonds 1972, pg. 108 [as holotype]: G-DC [G00144295]; isolectotype: P [P00366815]).
Solanum tinctorium Welw., Apont. 590. 1859 [1858].
Type. Angola. Golungo Alto, 1856, *F.M.J. Welwitsch 6103* (lectotype, designated here: BM [BM000942995]; isolectotypes: BM [BM000942996], K [K001029777]).
Solanum nigrum L. var. pterocaulum (Dunal) Schur, Enum. Pl. Transsilv. 478. 1866, as ‘pterocaulon’.
Type. Based on S. pterocaulum Dunal [“excl. German floras”]
Solanum boerhaavii Thell., Rep. Bot. Soc.& Exchange Club Brit. Isles 8: 187. 1927, as “Boerhaavii”.
Type. Based on Solanum nigrum L. var. guineense L. [as replacement name for Solanum guineense (L.) Mill.]
Solanum nigrum L. var. pterocaulum (Dunal) Domin, Biblioth. Bot. 89: 1127. 1928.
Type. Based on S. pterocaulum Dunal
Solanum intrusum Soria, Baileya 7: 33. 1959.
Type. Based on (replacement name for) Solanum nigrum L. var. guineense L., and Solanum guineense (L.) Lam.
Solanum scabrum Mill. subsp. laevis Olet, Novon 16(4): 510. 2006.
Type. Uganda. Buganda: Kampala district, Kawempe div., Kawempe North, Kalerwe, Tula road, 1220 m, 14 Feb 2001, *E.A. Olet 88* (holotype: MHU; isotypes: H, K, MO [ex descr.]).

#### Type.

Cultivated in Chelsea Physic Garden, said in protologue to “grow naturally in North America”, *Herb. Miller s.n.* (lectotype, designated by Henderson 1974, pg. 61 [as type]: BM [BM000847083]).

#### Description.

Annual or short-lived erect or ascending perennial herbs to 1.5 m tall, often woody at the base. Stems spreading, terete, ridged or winged, green to purple, if ridged or winged the stems later spinescent, older stems with or without prominent pseudospines, usually somewhat hollow; new growth puberulent with simple, spreading, uniseriate, translucent, eglandular trichomes, these 2–8-celled, 0.3–0.8 mm long; older stems glabrescent. Sympodial units difoliate, the leaves usually not geminate, but if leaves paired, then one is usually smaller. Leaves simple, 4–15(-20) cm long, 3–10(-16) cm wide, broadly ovate to elliptic, very variable in size depending on cultivars and growth conditions, membranous to somewhat fleshy, green to dark green to somewhat purple coloured above, slightly paler below, without smell; adaxial and abaxial surfaces glabrous or sparsely pubescent with simple uniseriate trichomes like those on the stem mainly along veins and scattered along lamina; major veins 3–6(-8) pairs, paler green or often purple tinged; base abruptly acute or truncate, narrowly winged on to the petiole; margins entire or rarely shallowly sinuate; apex rounded to acute; petioles 1–5(-8) cm long, glabrous or sparsely pubescent with simple uniseriate trichomes like those of the stem. Inflorescences 1–2(-4) cm long, internodal, simple, furcate or many times branched (in cultivars), sub-umbelliform, with 4–10(-30+) flowers clustered towards the tip(s) of the rhachis, glabrous or sparsely pubescent with simple uniseriate trichomes like those on the stem; peduncle 1–5(-8) cm long, erect and thick, much thickened at apex, subwoody; pedicels 0.4–1 cm long, 0.3–0.5 mm in diameter at the base, 0.75–0.9 mm in diameter at the apex, abruptly expanding to the calyx tube, stout, erect and/or spreading, articulated at the base; pedicel scars tightly clustered near the tip of the rhachis, spaced 0–2 mm apart, sometimes with short stumps ca. 0.5–1.0 mm long. Buds globose to subglobose, the corolla exserted 1/2–1/3 from the calyx tube before anthesis. Flowers 5-merous or occasionally fasciated and 6–7-merous in cultivars, all perfect. Calyx tube 0.9–1.1 mm long, abruptly cup-shaped with a broad base, the lobes slightly unequal, 0.9–1.5 mm long, 0.8–1.4 mm wide, broadly deltate with a rounded tip, green or purple-tinged, glabrous or sparsely pubescent with simple uniseriate trichomes like those of the pedicels, the margins often drying scarious and white. Corolla 7–12 mm in diameter, white, purple-tinged or occasionally lilac to dark purple, with a yellow basal star, stellate, lobed ca. 1/2 way to the base, the lobes 2.5–4 mm long, 1.5–3 mm wide, spreading or reflexed, densely papillate on tips and margins. Stamens equal; filament tube very short, to 0.1 mm long; free portion of the filaments 0.5–0.8 mm long, glabrous or pubescent with tangled uniseriate simple trichomes; anthers 2–3 mm long, ellipsoid or slightly tapering towards the tips, yellow, orange or brown, poricidal at the tips, the pores lengthening to slits with age and drying, the connective often becoming brownish-black in dry specimens. Ovary rounded, glabrous; style 2.5–5 mm long, densely pubescent with simple uniseriate trichomes 0.2–0.5 mm long in the basal 1/2 where included in the anther cone, exserted beyond anthers 0–1.5 mm; stigma capitate, the surface minutely papillate. Fruit a globose to depressed-globose berry, 10–20 mm in diameter, purplish-black at maturity, the pericarp thick, shiny or somewhat matte, not transparent; fruiting pedicels 0.7–1.5(-2.0) cm long, 0.5–1 mm in diameter at the base, 1.1–1.5 mm in diameter at the apex, stout, erect and spreading, purple or brown, usually not falling with the fruit, remaining on the plant and often persistent on older inflorescences; fruiting calyx not accrescent, the tube 1.5–2 mm long, usually tearing unevenly, the lobes 2–3 mm long, usually with thicker white margins in dry material, appressed or spreading to slightly reflexed. Seeds (20-)100–150 per berry, 2–2.8 mm long, 1.5–1.8 mm wide, flattened and tear-drop shaped with a subapical hilum, yellow-brown or purple, the surfaces minutely pitted, thin and the embryo clearly visible, the testal cells rectangular to pentagonal in outline. Stone cells absent. Chromosome number: *2n*=6x=72 (Soria and Heiser 1961 [as *S. melanocerasum*]; Heiser et al. 1965 [as *S. melanocerasum*]; Henderson 1974; Edmonds 1977, 1983; Symon 1981; Jacoby and Labuschagne 2006; Bukenya 1996; Olet et al. 2015).

**Figure 46.**
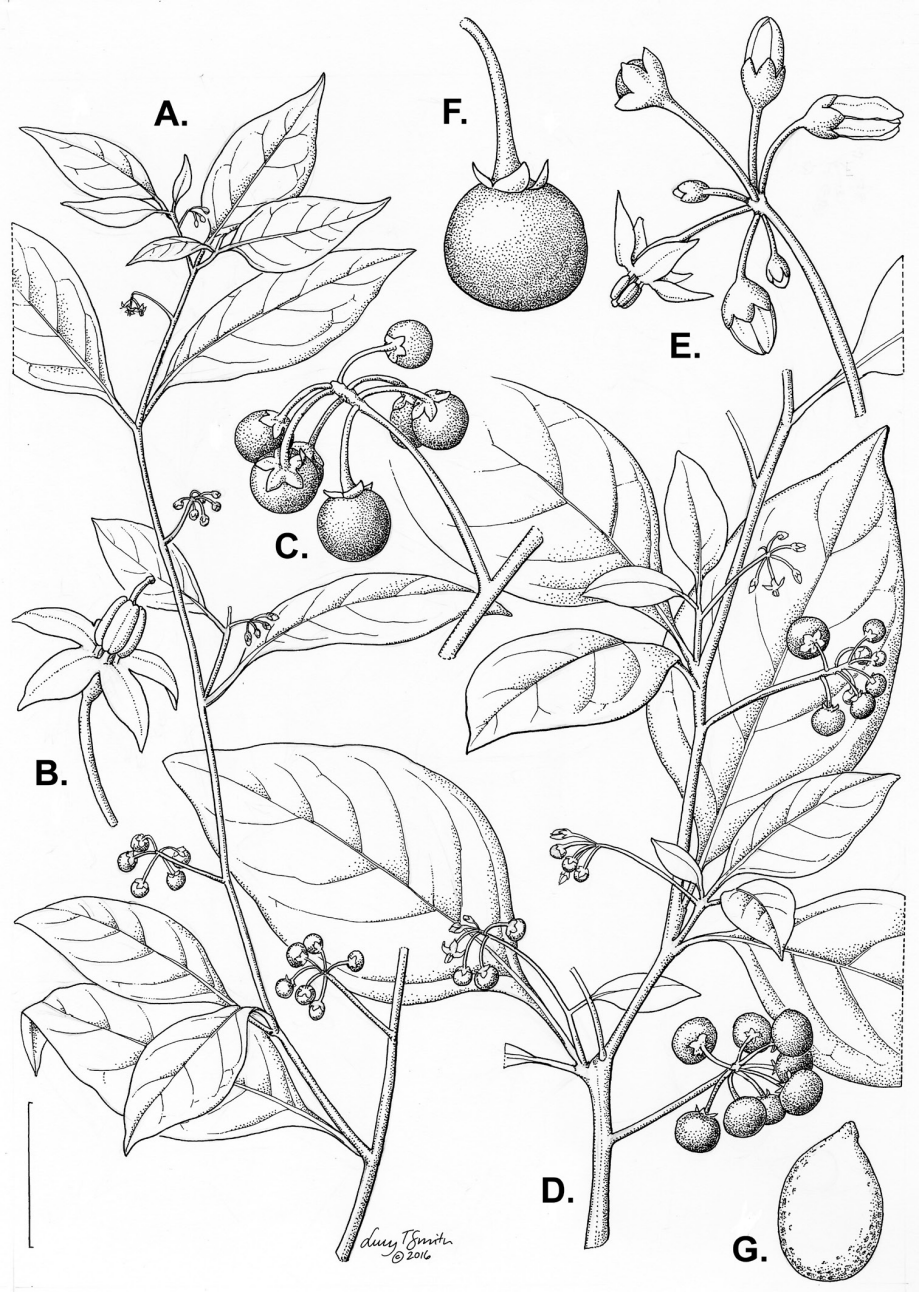
*Solanum scabrum* **A** Habit of wild form **B** Flower of wild form **C** Infructescence of wild form **D** Habit of cultivated form **E** Inflorescence of cultivated form **F** Fruit of cultivated form **G** Seed (**A–C** *Pilz 2108*; **D–G** *Nee 16088*). Scale bar: 4 cm (**A, D**), 3.3 mm (**B**), 1.5 cm (**C, F**), 7 mm (**E**) and 2 mm (**G**). Drawing by L. Smith.

**Figure 47.**
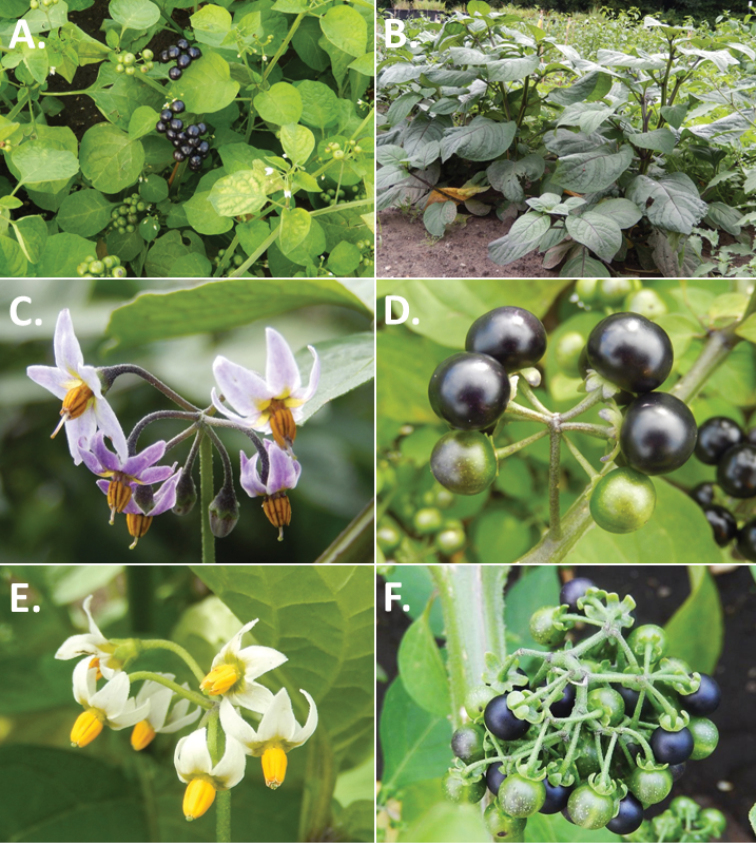
*Solanum scabrum* **A** Common habit **B** Habit in taller varieties **C** Flowers of the larger berried variety at full anthesis **D** Fruits of a larger berried variety **E** Flowers of the smaller berried variety at full anthesis **F** Fruits of a smaller berried variety (**A** Nijmegen acc. BG13; **B** Nijmegen acc. A34750072; **C** Nijmegen acc. GB22; **D** Nijmegen acc. H065; **E** Nijmegen acc. A34750067; **F** Nijmegen acc. 2010/3). Photos by S. Knapp.

#### Distribution

**Figure 48.**
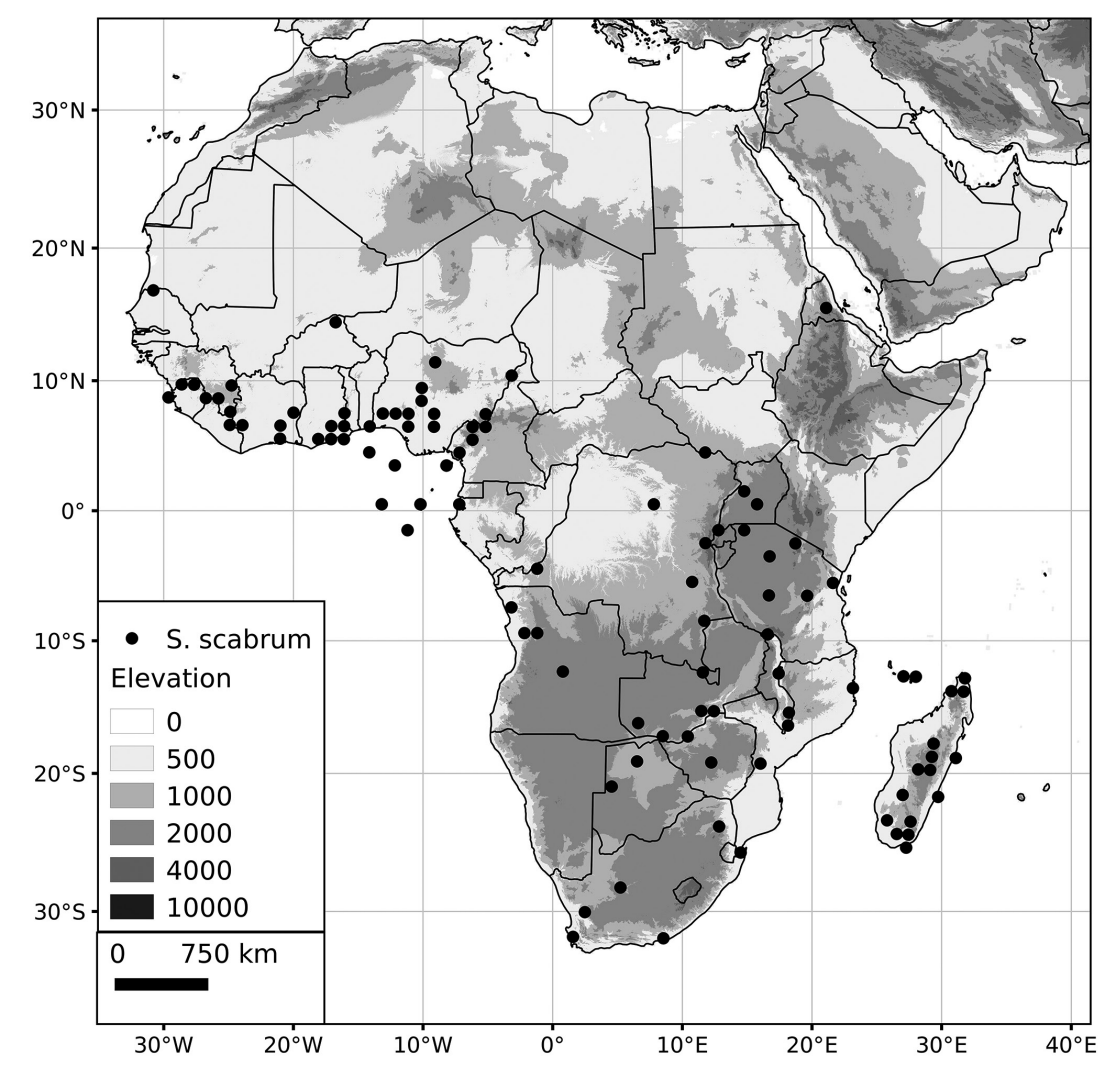
Distribution of *Solanum scabrum* in its native range.

(Figure 48). Native to tropical Africa, but introduced worldwide as a cultivated plant.

#### Ecology.

Grows in open areas, in a wide variety of habitats, in wet forests or drier areas, along roads and at field edges; often cultivated; between sea level and 2,300 m elevation.

#### Common names.

Benin: odu, ogomo, feibii (Essou and Hermans 2006); Cameroon: houlohada, legume vert, njebanyoon, ossan, tindar noon, wikitiniho, zom/zoum; China: mu long kui (Zhang et al. 1994); Côte d’Ivoire: foue; Equatorial Guinea: hierba mora, nahú, seba [Bubi people], sisa; Ghana: nsusua; Madagascar: anamamy, brede; Nigeria: awa ibo, gautan kaji, kumbi, odu, ogunmo, ugumakbe; São Tome e Principe: losua; Sierra Leone: a-kempa,borisuguli, filami, kemba, kembei, kholekoleden-na, magboli, reinpa, timbainyi; Slovenia: čučoried kovitý (Bertová and Goliašová 1993); South Africa: nastagal; South Sudan. nzuobire; Tanzania: isonga (Kinyakyusa language); Uganda: eshwiga, eshwiga enzungu, kujikuji, nswiga ya kizungu; United Kingdom: garden huckleberry (Stace 2010).

#### Uses.

Leaves used as greens (spinach) and cooked, sold in markets; berries used as ink; in Benin, the powdered leaves are used to cure dysentery (Essou and Hermans 2006).

#### Preliminary conservation status

(IUCN 2016). *Solanum scabrum* is widespread across Africa and an important leafy vegetable in cultivation; it can be assigned a preliminary status of LC (Least Concern; Table 7), but it may become important to conserve local populations in order to preserve genetic variation for plant breeding.

#### Discussion.

*Solanum scabrum* is the most commonly cultivated and widespread of the African black nightshades. It is the most important indigenous leafy vegetable in the black nightshade group and is commonly known as the African nightshade (Edmonds and Chweya 1997; Dinssa et al. 2016). *Solanum scabrum* shows great variation in growth form, leaf shape and size and berry number, in part due to the significant local variation introduced by human selection in cultivated populations.

The species has also been introduced to Europe, North America, Australia and New Zealand. In the United States of America, *S. scabrum* is known as “Garden Huckleberry” and its identity, origin and suitability to human consumption were the subject of great interest in the 1960s (as *S. guineense*, e.g. Soria and Heiser 1959, 1961; Heiser 1969).

Different cultivars are recognised, the late flowering plants cultivated for their larger abundance of leaves with smaller number of fruits and earlier flowering plants that have larger inflorescences which are cultivated for their fruits (Manoko 2007). Cultivated forms of *S. scabrum* stand out as quite distinctive; all forms have larger flowers and anthers that are often brownish in colour, while the forms cultivated for their leaves have larger, longer petiolate leaves and those cultivated for their fruits have larger, more numerous and shinier berries (the size of cherries) on erect or spreading pedicels. The pericarp in *S. scabrum* berries in all varieties is quite thick compared to other black nightshades, but mature berries of cultivars show variation in anthocyanin content where some individuals have dark purple mesocarp and others pale green. One of the major limitations to cultivation of *S. scabrum* as a leaf vegetable is the relatively low leaf yield due to early flowering and excessive fruiting (Ojiewo et al. 2013; Ojiewo et al. 2006). In response to this, enhanced varieties are being developed and new registered varieties are being released (Ojiewo et al. 2006; Ojiewo et al. 2013) such as “Medium leaf long-lasting” released in Kenya in 2006. Breeding is also focussing on local variation and drought resistance (Dinssa et al. 2016).

The presumed wild forms were described at the infraspecific rank by Olet et al. (2006) as subsp. laevis. This differs from the cultivated forms in having narrower leaves and smaller fruits that are globose rather than subglobose. Dehmer (2001) and Olet et al. (2011) have shown, however, that these differences are not reflected in relationships based on AFLP molecular markers. These wild forms of *S. scabrum* have often been called *S. nigrum* (e.g. Edmonds 2006a, 2006b, 2012), but they differ from true *S. nigrum* which, in Africa only occurs in the north along the Mediterranean, in their congested inflorescences, spreading pedicels, calyx lobes that tear unevenly and long-petiolate leaves. Olet et al. (2011) clearly demonstrated that the wild forms of *S. scabrum* are genetically closely related with cultivated *S. scabrum* accessions and form a distinct cluster away from European *S. nigrum*.

*Solanum scabrum* can be distinguished from the somewhat similar *S. americanum* by its larger anthers (2–3 mm long versus 0.8–1.5 mm long). In both these species, as well as *S. retroflexum*, the berries usually lack stone cells (some populations of *S. americanum* can have up to 4 stone cells) and drop without the pedicels at maturity, leaving the pedicels behind on old inflorescences. In both *S. scabrum* and *S. americanum*, berries are purple-black and shiny, while *S. retroflexum* has dull purple-black berries with a distinct grey bloom. The pedicels of *S. scabrum* are usually erect or spreading.

Plants described as *S. fistulosum* and S. oleraceum var. macrocarpon by Dunal (1814, 1852 respectively, see synonymy) from Brazil were almost certainly taken there from western Africa by enslaved people and were either collected from home gardens or became established in the New world.

*Solanum scabrum* is closely related to *S. nigrum* and *S. villosum* based on molecular data (Manoko 2007; Poczai and Hyvönen 2011). The close relationship amongst these polyploids indicates that they might share the same diploid parent. The idea that *S. villosum* is the likely tetraploid parent of the hexaploids *S. nigrum* and *S. scabrum* has also been supported by evidence from crossing experiments and cytological studies (Soria and Heiser 1961; Heiser et al. 1965; Rao et al. 1971; Jardine and Edmonds 1974; Edmonds 1977, 1978; Edmonds and Glidewell 1977; Edmonds 1978). Two possibilities still remain: one of the hexaploid species (*S. nigrum* and *S. scabrum*) evolved first and gave rise to the other or that the two hexaploids originated from the same parents independently (Poczai and Hyvönen 2011). These two scenarios will be difficult to distinguish based on molecular data due to the complexity caused by high ploidy level.

The nomenclature and typification of the various synonyms of *S. scabrum* and the earliest name applied to the taxon, have been extensively discussed by others (e.g. Gras 1863; Thellung 1927; Polgár 1939; Stebbins and Paddock 1949; Soria and Heiser 1959; Heine 1960; Edmonds 1979b). We merely add additional notes for our lectotypifications designated here or for those that have been designated inadvertently (e.g. Prado et al. 2015).

Edmonds (2012) designated a specimen in the Dillenian herbarium at OXF as the lectotype for *S. melanocerasum* All.; Allioni did not cite and probably never saw these specimens; this is correctable to neotype.

D’Arcy (1974a: 737) inadvertently lectotypifed *S. guineense* Lam. by citing a specimen in “Herb. *Lam. s.n.* (P)” as “Type”; this corresponds to a specimen in the Lamarck herbarium (P00357674) that is labelled “Solanum nigrum guineense L. planta glabra.. fructu magno nigro. Sol. guineense Lam. Ill”, suggesting that Lamarck was basing his name on Linnaeus’ S. nigrum var. guineense. In the protologue, Lamarck (1794) indirectly referred to Linnaeus, through citing the single element in the protologue of S. nigrum var. guineense, the illustration in Dillenius (1732). We are therefore treating these two names (S. nigrum var. guineense L. and *S. guineense* Lam.) as homotypic, rendering D’Arcy’s (1974a) lectotypification superfluous.

Lamarck’s (1794) *S. triangulare* has traditionally been considered a synonym of *S. africanum* Mill. (a member of the Dulcamaroid clade, Knapp 2013); the protologue cites two elements, only one of which “?” represents *S. africanum* and was questioned as only possibly being the same by Lamarck. The other cited element of *S. triangulare* is an illustration from *Herbarium Amboinense* (Rumphius 1750) that represents *S. americanum*. D’Arcy (1974a), however, cited the specimen in Lamarck’s herbarium (“Lamarck s.n. (P)”; P00357626) as “Type” and we must accept this as a valid lectotypification. This specimen is of a narrow-leaved plant of *S. scabrum.*

Willdenow (1809) did not specifically reference Allioni’s epithet in his protologue, so we are treating it as a new name. There is no material in the Willdenow herbarium labelled as *S. melanocerasum*, but a specimen (B-W04368010) of *S. scabrum* (labelled as “S. guineense” and “ex horto proprio W”) was clearly grown in Berlin and seen by Willdenow. We designate this as the neotype for *S. melanocerasum* Willd.

*Solanum pterocaulum* is illegitimate because Dunal (1813) cited *S. scabrum* in synonymy. In the *Prodromus* (Dunal 1852), his use of ‘*pterocaulon*’ was simply an orthographic variant and is correctable (see McNeill et al.. 2012, Art. 61.5 and associated provisions in Art. 61); it does not alter the legitimacy of the name.

No original material has been traced for *S. memphiticum* Mart., but the emphasis placed on the dark purplish colour of the stems and leaves by Martius (1814) in the protologue, coupled with the petiolate nature of the leaves and the erect peduncles, suggests this plant was *S. scabrum.*

Of the several species in this group described by P.M. Opiz (see discussion under *S. nigrum*), we are convinced from the description that *S. nitens* is a synonym of *S. scabrum*, rather than of *S. nigrum*, although Opiz (1843) places it in his “superspecies” *S. nigrum*. The mention of shiny berries on erect pedicels is distinctive; we have yet to confirm this with specimens from Opiz’s herbarium at PR.

The lectotype (G00144295) chosen for S. oleraceum var. macrocarpum is the better preserved of the two duplicates of *Martius 1225* cited by Dunal (1852).

The lectotype chosen for *S. tinctorium* (*Welwitsch 6103*) is the more clearly duplicated of the collections cited in the protologue; we have selected the best preserved sheet (BM000942995) as the lectotype. The name apparently comes from the staining berries that were used as ink.

#### Selected specimens examined.

**Angola**. **Bengo**: Ambriz, Abriz, Dec 1872, *Monteiro s.n.* (K); **Benguela**: Hochland zwischen Ganda und Caconda, Dec 1933, *Hundt 797* (BM); **Cuanza Norte**: Varzea do Isidoro ad rivum Cuango, Dto. Golungo Alto, Jul 1855, *Welwitsch 6100* (BM); **Malanje**: Distr. Pungo Andongo, prope Lusillo, Jan 1857, *Welwitsch 6108* (BM); **Namibe**: Mofsamedes, Herb. Moira, Habit Cavalheiros, Jul 1859, *Welwitsch 6033* (BM).

**Benin**. **Atlantique**: Wida, Dahomey, 26 Aug 1903, *Estève 111* (BM).

**Botswana**. **Ghanzi**: Ghanzi camp, 4 May 1969, *Brown 6019* (K); Ghanzi camp, 4 May 1969, *Cole 6019* (K); **North West**: Mutsoi NE of Nokaneng, 21 Mar 1967, *Lambrecht 90* (K).

**Burkina Faso**. **Seno**: Dori Dam, 16 Oct 2007, *Sanou & Leonard BUR-596* (K).

**Cameroon**. **Adamaoua**: Mayo-Banyo, Mambilla Plateau Cameroon, around Somie village, 21 Jun 2009, *Komaromi 48* (K); **Centre**: Mimboman area, Yaounde, 16 Sep 1986, *Manning 235* (MO); **Extreme-Nord**: Monts Mandara, Hossere Oupay, 15 km NNO de Mokolo, 15 Sep 1964, *Letouzey 6880* (K, P); **Littoral**: Donala, 16 Aug 1986, *Johns 86-481* (K); **Nord-Ouest**: Mezam, Above Bamenda, 20 Jan 1928, *Migeod 358* (BM, K); **Sud-Ouest**: Mt Kupe, Kupe Village, 24 May 1996, *Ryan 289* (K, MO, P).

**Central African Republic**. Mboukou Griko, Territoire du Haut-Obangi, 24 Sep 1902, *Chevalier 5518* (K).

**Comoros**. **Anjouan**: Anjouan (Comoro-Insel Johanna), Jun 1875, *Hildebrandt 1626[b*] (BM, W); **Moheli Island**: Moheli, NE center of island, 14 Aug 1987, *D’Arcy 17617* (MO, P); 1 Nov 1990, *D’Arcy 17765* (MO); **Njazídja**: Grande Comore, 9 Aug 1981, *Doutrelepont 1203* (MO, P).

**Côte d’Ivoire**. **Abidjan**: Adjame, 31 May 1973, *De Koning 1747* (MO); **Dimbokro**: c. 5 km W of Dimbokro, 13 Oct 1975, *van der Burg 1177* (MO); **M’Bahiakro**: Koffi-Akakro (s. Prikro), 25 Jul 1973, *Smittenberg-Visser 61* (MO); **Montagnes**: Man, 6 km N nr Yebegouin, 27 Jan 1984, *Hepper* & *Maley 7849* (K).

**Democratic Republic of the Congo**. **Katanga**: Lukonzolwa, Moero, 1933, *Quarré 3297* (K); Kongolo, 5 Feb 1920, *Schantz 646* (K); **Kinshasa**: Maluku, Route Menkao-Kingankati, 5 Nov 1971, *Breyne 2227* (MO); **Nord-Kivu**: Lubarika, 1953, *Gilon 315[a*] (MO); **Orientale**: Ituri, w.v. Albert-See, Oct 1934, *Gusinde s.n.* (W); Yangambi, 26 Jul 1938, *Louis 10507* (K, P); **Sud Kivu**: Kizozi, Jul 1933, *Lejeune 55* (K).

**Egypt**. **Giza**: Faculty of Agriculture, Giza, 13 Jun 1971, *Sisi s.n.* (MO).

**Equatorial Guinea**. **Annobon**: Pico de Fogo, SW side, 25 Jul 1959, *Melville 188* (BM, K, MA, P); **Bioko**: entre Moca y Riaba por el camino viejo, 20 Feb 1989, *Fernández-Casas 11820* (K, MA, MO, P); **Bioko Norte**: Pico Basilé, Carretera del pico Basile, 28 Mar 1990, *Do Carvalho 4305* (K, MA, MO, P); **Bioko Sur**: Moca, camino de Ureca, 18 Feb 1989, *Fernández-Casas 11725* (K, MA, MO).

**Eritrea**. **Maekel**: Asmara, 1 May 1892, *Terracciano* & *Pappi 187 [2206*] (FT); **Semienawi Keyih Bahri**: Asmara, Beless, Hamasen, 4 May 1892, *Terracciano* & *Pappi 2536* (FT).

**Gabon**. **Estuaire**: Jardin Cenarest, Libreville, Poubelle, 16 Jan 1986, *Louis 1990* (MO).

**Ghana. Ashanti**: nr Mampong, Ashanti, 7 Aug 1963, *Darko 5115* (K); **Central**: Aswansi, W.P, 12 Oct 1954, *Darko 1032* (K); **Eastern**: Akosombo, 25 May 1970, *Enti 1723* (MO); Akwapim, Mampong Scarp, 14 Jun 1953, *Morton s.n.* (K); **Greater Accra**: Accra, May 1961, *Irvine 5095* (K); **Volta**: Adzido, Keta, 16 Sep 1960, *Akpabla 2117* (K).

**Guinea**. **Nzérékoré**: Lola, Mt. Nimba, 1 Nov 2012, *Diabate* & *Mas 1419* (MO).

**Indonesia**. **Java**: Central Java, Mt. Slamet, 15 Mar 2004, *Hoover et al. 89* (A); **Seram**: Manusela National Park, 12 Sep 1987, *Argent C87-179* (A, E); **Sumatra**: E of Berastagi, Karo Highlands, 4 Jun 1928, *Si Toroes 407* (A, GH).

**Lesotho**. Sehlabathebe, 4 Jan 1973, *Bayliss 5476* (MO).

**Liberia**. **Central**: 3 mi NE of Suacoco, Gbarnga, 7 Feb 1951, *Daniel 117* (B, BM, MO); **Grand Gedeh**: Tchien, Mim Timber Co (Fijnhout), 16 May 1970, *de Koning 519* (MO); **Lofa**: mi along rd to Wologesi, 15 Jul 1970, *Jansen 2015* (K, MO, P); **Nimba**: Nimba Mountains, 27 Jul 1962, *Leeuwenberg* & *Voorhoeve 4668* (B, K, MO, P).

**Madagascar**. **Antananarivo**: Grande Terre-Ouangani, Apendzo-Jivany. Barakani, 7 May 2002, *Barthelat et al. 885* (K, MO, P); **Antsiranana**: Mt. d’Ambre, partie centrale, Prov. Diego-Suarez, 11 Nov 2007, *Gautier 5198* (K, MO); **Fianarantsoa**: Ankafana, 1880, *Cowan s.n.* (BM); 10 km W of Ivato on Route 35, 25 Jan 1975, *Croat 29606* (MO); **Toamasina**: au km 26 de la route de Tamatave, 18 Oct 1951, *Benoist 147* (P); **Toliara**: Beroroha, Vallée du Mangoky et de l’Isahaina aux environs de Beroroha, Oct 1933, *Humbert 11309* (K, MO, P); Andohahela RNI, Mt. Trafonaomby, Taolagnaro, 7 Apr 1994, *Randriamampionona 692* (MO, P).

**Malawi**. **Central**: Salima Distr., Liganga village A. Mpemba, 14 Jun 1985, *Kwatha et al. 210* (MO); Salima Distr., Luwadzi stream, 14 Jun 1985, *Salubeni et al. 4242* (MO); **Northern**: Nkhata Bay, Musalowa village, Chizumulu Island, 25 Mar 1989, *Balaka* & *Patel 2025* (K, MO); **Southern**: Malawe Hill, W of Port Herald, 23 Mar 1960, *Phipps 2653* (K, MO).

**Mauritania**. **Trarza**: Rosso Administrative area, Village Rosso, 9 Oct 1962, *Adam 18728* (MO).

**Mozambique**. **Baroma**: N’kanya, N of Zambesi River, 25 Jul 1950, *Chase 2857* (BM); **Cabo Delgado**: Pemba, Jun 1909, *Rogers 8256* (K); **Manica**: Lower slopes of Chimanimani Mountains, Apr 1967, *Westwater 192954* (K).

**Namibia**. Onjossariviers, im Ufergestrupp, 21 Jun 1957, *Seydel 1162* (A, K).

**New Zealand**. **North Island**: Bay of Plenty, Ohope, Whakatane Ecological Region, Taneatua Ecological District, 13 Mar 1979, *Grant s.n.* (AK).

**Nigeria**. **Delta**: [Ogwashi-Ukwu], 25 Nov 1912, *Thomas 2042* (K); **Edo**: Nikrowa Forest Reserve, Mid-west State, Iyekovia Distr., 8 Oct 1973, *Daramola FHI-72483* (K, MO); **Enugu**: 10 mi E of Erugu, Mar 1948, *Irvine 3607* (K); **Gongola**: Gemu Distr., Mambilla plateau, 18 Aug 1977, *Fagbemi 438* (MO); **Jos**: Naraguta, *Lely 31* (K); **Kaduna**: Zaria, 1975, *Magaji MG-725* (K); **Kano**: Kano, Wudil Distr, 50 km SE of the city of Kano, 16 Mar 1988, *Etkin 63[b*] (MO); **Kogi**: Odu, SW Nigeria, *Van Eyenhuisen 7* (K); **Lagos**: Lagos, *Dalziel 1188a* (K); Lagos, *Dalziel 1188b* (K); **Niger**: Nupe, *Barter 1054* (K, P); **Ondo**: between Ikare and Oke-Agbe, Ikare District, 2 May 1979, *Daramola* & *Osanyinlusi 154* (K); **Oyo**: Ibadan, 7 km W of Polytechnic, 11 Jun 1977, *Pilz 2108* (K, MO); **Taraba**: State of Gongola, Distr. Mambilla Plateau, Nguroje township, 23 May 1982, *Odewo 130* (MO).

**São Tome e Principe**. Bom Sucesso, 27 Jan 1949, *Espírito Santo 218* (BM); Vanhulst (Macambrará), 28 Oct 1932, *Exell 89* (BM).

**Senegal**. In paludilb[us] N’Boro nec non ins[ula] Bonavista, 1838, *Brunner 108* (BM, G, W); Sin. loc., 1828, *Perrottet 555* (BM, W); *Perrottet 556* (BM);

**Seychelles**. Morne Blanc, 2 Oct 1970, *Schlieben 11670* (B, K).

**Sierra Leone**. **Eastern**: Kailahun, Musaia, 19 Dec 1946, *Deighton 4571* (K); **Northern**: Port Loko, Rokupr, Magbema, 18 Apr 1959, *Jordan*, *1059* (K); Yonibana, 12 Nov 1914, *Thomas 4909* (W); **Western**: Freetown, 28 Apr 1965, *Morton s.n.* (K).

**South Africa. Eastern Cape**: Grahamstown, 7 Sep 1970, *Bayliss 4594* (A); **KwaZulu-Natal**: 2632 CD Bella Vista grid, between Ndumu Store and the Game Reserve, 30 Oct 1969, *Moll 4138* (A, K); **Limpopo**: Mopani, Shiluvane, Aug 1899, *Junod 575* (K); **North West**: Okawango Delta, Okavango Delta, Delta camp, 16 Jun 1994, *Cole 923* (K); **Northern Cape**: Augrabies Falls National Park, Orange river bank, 14 Mar 1978, *Balsinhas* & *Harding 3290* (K, MO); **Western Cape**: Dowweklip, Voelklip, Hermanus, 6 Jun 1980, *Williams 287* (K, MO); Skeleton Ravine, 3 Oct 1897, *Wolley-Dod 3180* (K).

**South Sudan**. **Bahr El Ghazal**: Anglo-Egyptian Sudan, Bahr El Ghazal Prov., Ibba, 10 Mar 1934, *Dandy 624* (BM, EA); **Equatoria**: R. Napere, 25 Nov 1937, *Wyld 347* (BM).

**Tanzania**. **Dodoma**: Mpwapwa, Mbuga Village, Kibakwe Division, Mbuga Ward, 31 May 2005, *Kindeketa et al. 2549* (EA, MO); **Iringa**: Ruaha National Park, FTEA region T7, top of Mpululu mountain, 21 May 1968, *Renvoize* & *Abdallah 2312* (EA, K); **Kagera**: Bukoba, 30 Oct 1992, *Breteler 11599* (MO); **Mbeya**: Rungwe, Ngumbulu Village, NE part of Rungwe Forest Reserve, 14 Mar 2008, *Abeid et al. 2848* (MO); **Morogoro**: Tanganyika, Ulugurus, Jan 1935, *Bruce 524* (BM, K); **Shinyanga**: Shinyanga, Nov 1938, *Koritschoner 2191* (EA, K).

**Togo**. Lomé, 22 Sep 1976, *Ern et al. 883* (B); slopes of Bauman Peak, 14 Aug 1962, *Morton A4277* (MO).

**Uganda**. **Central**: Kyadondo Mengo (U4); Nr Kanyanya, 16 Jun 1990, *Rwaburindore 2992* (MO); **Mengo**: Distr. W. Mengo, 4 mi Gayaza rd, 5 Jul 1980, *Rwaburindore 701* (MO); **Northern**: Yumbe, 26 Nov 1941, *Thomas 4070* (EA, K); **Western**: Kigezi DFI, 28 Aug 1972, *Goode G3-72* (K); Kigezi, Bugangari, Rhuzumbura, Kigezi, Feb 1949, *Purseglove 2712* (EA, K).

**United Kingdom. England**: Hertfordshire, Rye Meads Sewage Works, nr Rye House, 1 Oct 1996, *Hanson s.n.* (K).

**Zimbabwe**. **Mashonaland Central**: Imayanga, Nyamaropa TTk, 16 Jan 1967, *Biegel 1767* (MO); **Matabeleland North**: Binga Distr., Sanyam R. and Zambezi R. confluence, Sep 1955, *Davies 1514* (MO); **Matabeleland North**: Binga, Chizarira Game Reserve, Busi River, 10 Nov 1971, *Thomson 481* (K); **Wankie**: Wankie Distr., Victoria Falls, Elephant Hills Hotel, 19 Dec 1978, *Mshasha 144* (MO).

### 16. Solanum tarderemotum

Taxon classificationPlantaeSolanalesSolanaceae

Bitter, Repert. Spec. Nov. Regni Veg. 10: 547. 1912

Figures 49 , 50

Solanum dasytrichum Bitter, Bot. Jahrb. Syst. 49: 568. 1913.
Type. Tanzania. Tanga: Usambara, Kwai, *E. Eick 227* (holotype: B, destroyed; no duplicates found).
Solanum florulentum Bitter, Repert. Spec. Nov. Regni Veg. 10: 544. 1912.
Type. Tanzania. Tanga: Lushoto, Distr. Kwai, 1600 m, *E. Albers 189* (holotype: B, destroyed; no isotype at EA).
Solanum kifinikense Bitter, Repert. Spec. Nov. Regni Veg. 10: 545. 1912.
Type. Tanzania. Kilimanjaro: Kifinika volcano, Mar 1894, *G. Volkens 1909* (lectotype, designated by Edmonds 2012, pg. 127: HBG [HBG520843]; isolectotype: BR [BR0000008799418]).
Solanum pentagonocalyx Bitter, Repert. Spec. Nov. Regni Veg. 10: 544. 1912.
Type. Tanzania. Tanga: Usambara, Kwa Mstuza, Handei Kewegolot, Aug 1893, *C. Holst 9021* (lectotype, designated by Edmonds 2012, pg. 138: M [M-0105625]; isolectotypes: HBG [HBG520844], W [1894-0006577]).
Solanum tetrachondrum Bitter, Bot. Jahrb. Syst. 49: 565. 1913.
Type. Tanzania. Kilimanjaro: near Marangu, *G. Volkens 623* (holotype: B, destroyed; no duplicates found).
Solanum tetrachondrum Bitter var. subintegrum Bitter, Bot. Jahrb. Syst. 49: 566. 1913.
Type. Tanzania. Kilimanjaro: Marangu, *G. Volkens 622* (holotype: B, destroyed; no duplicates found).
Solanum viridimaculatum Gilli, Ann. Naturhist. Mus. Wien 77: 43. 1973.
Type. Tanzania. Njombe: Madunda, Livingstone-Gebirge am Nordostufer des Nyassasees, bei Madunda, Nebelwald, 2200 m, 29 Jul 1958, *A. Gilli 499* (holotype: W [1973-0001020]).

#### Type.

Tanzania. Kilimanjaro: Marangu, 1600 m, 10 Sep 1910, *H.J.P. Winkler 3856* (holotype: WRSL).

#### Description.

Annual to short lived erect to weakly scrambling perennials to 1.5 m tall, subwoody and somewhat branching at base. Stems spreading to decumbent, usually somewhat winged and with spinescent processes, fleshy, green to somewhat purple tinged, older stems drying pale yellowish-brown or whitish-grey, markedly hollow and even older stems collapsing in herbarium specimens; new growth glabrous or sparsely to moderately pubescent with simple, spreading, uniseriate, translucent, usually eglandular (sometimes tipped with a single-celled gland) trichomes, these 3–5-celled, 0.2–1.0 mm long; older stems glabrous. Sympodial units difoliate, the leaves usually not geminate. Leaves simple, 3–13 cm long, 1.5–2.5 cm wide, ovate to elliptic, very variable in size even on an individual plant, membranous, concolorous, without smell; adaxial surface glabrescent to sparsely pubescent with simple uniseriate trichomes like those on stem; abaxial surface usually less pubescent that the adaxial ones, glabrous to sparsely pubescent with simple uniseriate trichomes; major veins 4–8 pairs; base abruptly attenuate; margins entire or very rarely shallowly sinuate to lobed, if so the tips of the lobes acute or rounded; apex acute to attenuate; petioles (0-)4–8 cm long, pubescent with simple uniseriate trichomes like the leaves. Inflorescences 1–4(-6) cm long, internodal, unbranched or with up to 5 branches, but if branched usually only furcate, the flowers spaced along the rhachis, with 10–40 flowers, glabrous or sparsely pubescent like the stems; peduncle 1–3(14) cm long; pedicels 0.7–0.8 cm long, ca. 0.3 mm in diameter at the base, ca. 0.5 mm in diameter at the apex, slender, nodding at anthesis, sharply bent just above the insertion point so the base of the pedicel is an acute angle, this especially noticeable in fruit, articulated at the base or ca. 1 mm from the rhachis leaving a small stump, falling with the fruit at maturity; pedicel scars 1–2 mm apart in the distal part of the inflorescence, further apart towards the base. Buds ellipsoid, the corolla exserted ca. 1/2 way from the calyx until just before anthesis. Flowers 5-merous, perfect (although style length differs in flowers along the rhachis). Calyx tube ca. 1 mm long, conical with hyaline sinuses, the lobes 0.5–1 mm long, 0.4–0.9 mm wide, ovate to broadly triangular, glabrous to sparsely pubescent like the rest of the inflorescence. Corolla (6-)8–10 mm in diameter, white, stellate, lobed ca. 1/2 way to the base, the lobes 3–4 mm long, 1.5–2 mm wide, spreading or reflexed (apparently reflexed in older flowers), minutely papillate on the tips and margins. Stamens equal; filament tube ca. 0.25 mm long; free portion of the filaments 0.5–0.75 mmm long, adaxially pubescent with tangled simple uniseriate trichomes; anthers 1.5–2.5 mm long, 0.75–1 mm wide, ellipsoid, yellow, poricidal at the tips, the pores lengthening to slits with age and drying. Ovary rounded, glabrous; style 3.5–5 mm long, bent or straight, densely pubescent in the basal 1/2 to 1/3, exserted to ca. 1 mm or occasionally included in the anther cone and the stigma flush with the pores; stigma capitate, the surface minutely papillate. Fruit a globose berry, 4–6 mm in diameter, pale green or translucent blackish-grey when ripe, the pericarp thin, matte; fruiting pedicels 0.8–1.1 cm long, ca. 1 mm in diameter at the base, sharply bent at the base and strongly pendent, dropping with mature fruits, not persistent; fruiting calyx not accrescent, the tube ca. 1 mm long, the lobes 0.5–1 mm long, appressed to the berry, the calyx from above pentagonal or stellate. Seeds 20–40 per berry, 1.5–2 mm long, 1–1.5 mm wide, flattened and tear-drop shaped with a subapical hilum, tan or pale brown, the surfaces minutely pitted, the testal cells elongate-rectangular. Stone cells (0-)1–5(-10) per berry. Chromosome number: *2n*=4x=48 (Olet et al. 2015; Manoko 2007).

**Figure 49.**
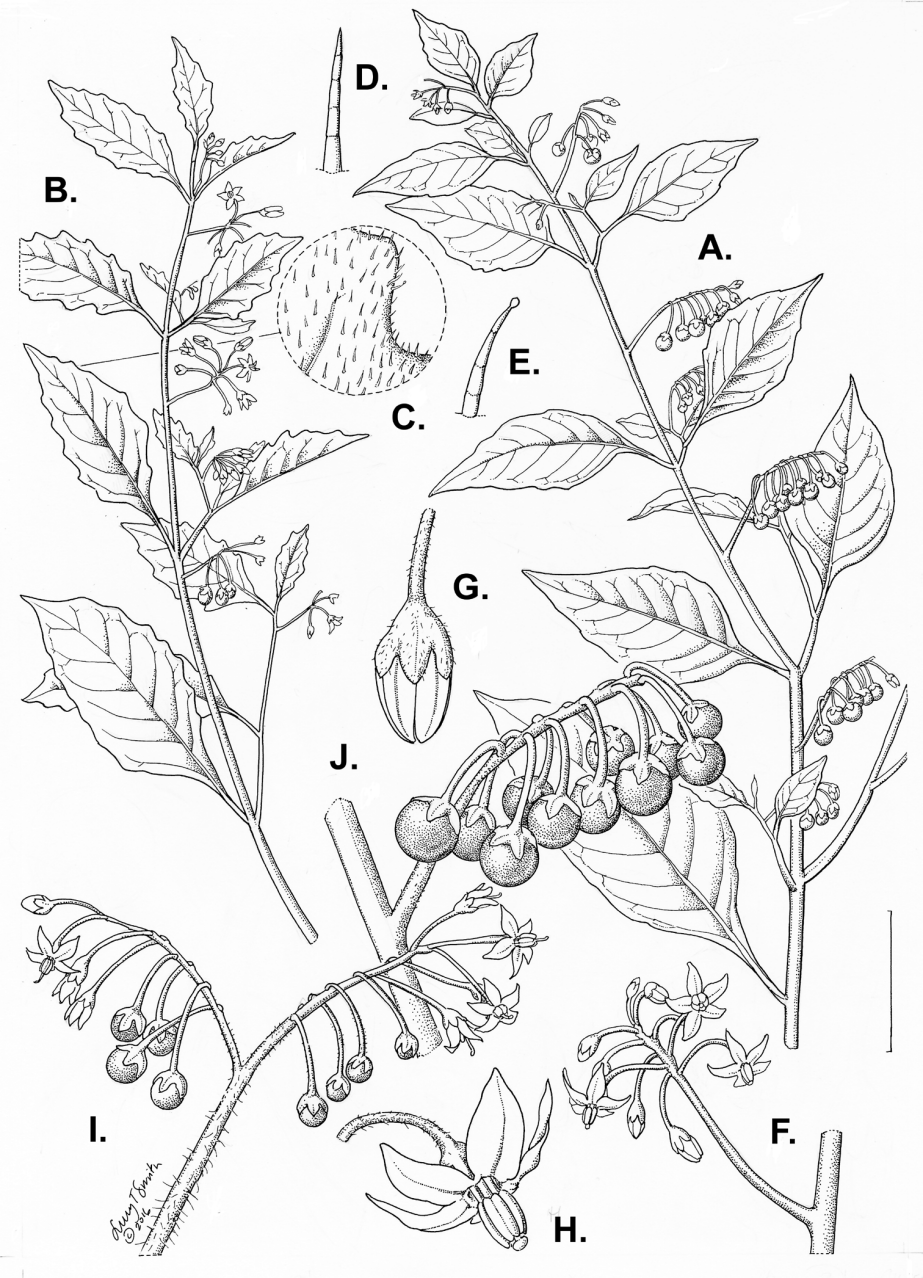
*Solanum tarderemotum* **A** Habit of glabrescent form **B** Habit of pubescent form **C** Detail of adaxial leaf surface **D** Eglandular trichome **E** Glandular trichome **F** Inflorescence **G** Bud **H** Flower at anthesis **I** Furcate inflorescence **J** Infructescence (**A, J** *Friis et al. 2047*; **B–E** *Burger 2006*; **F, G** *Gilbert & Thulin 651*; **H** Nijmegen acc. A34750039; **I** *Goode G/72*). Scale bar: 4 cm (**A–B**), 4 mm (**C**), 0.75 mm (**D–E**), 1 cm (**F, I**), 5 mm (**G–H**) and 1.5 cm (**J**). Drawing by L. Smith.

**Figure 50.**
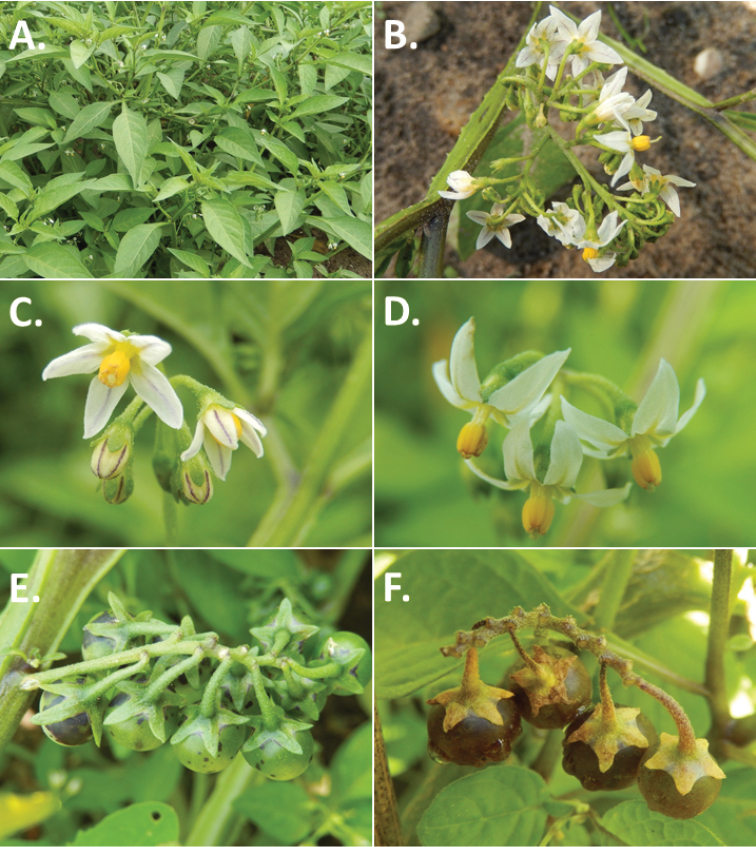
*Solanum tarderemotum* **A** Habit **B** Inflorescence **C** Buds **D** Flowers at full anthesis **E** Infructescence **F** Mature fruits (**A** Nijmegen acc. A54750214; **B** Nijmegen acc. A14750164; **C** Nijmegen acc. A34750035; **D** Nijmegen acc. A34750039; **E** Nijmegen acc. A54750214; **F** Nijmegen acc. HNSAR22). Photos by S. Knapp.

#### Distribution

**Figure 51.**
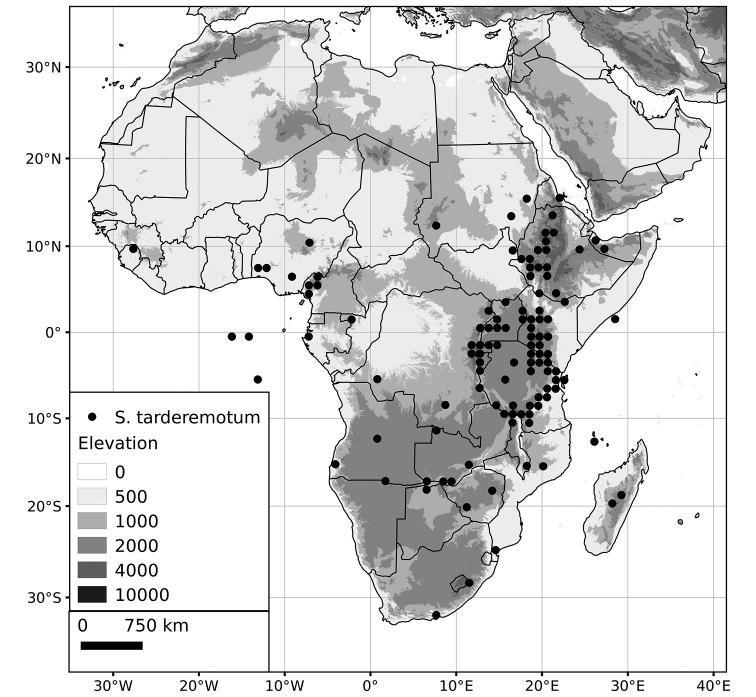
Distribution of *Solanum tarderemotum*.

(Figure 51). Common throughout sub-Saharan tropical Africa from Sudan to Mozambique and South Africa and west to Cameroon and Angola; we have also seen a few isolated collections from the Comoro Islands and Madagascar (perhaps introduced).

#### Ecology.

Grows in a wide variety of disturbed habitats, along forest margins and roadsides and in clearings, often cultivated; between (0-) 500 and 3,300 m elevation.

#### Common names.

Burundi: isogo [Kirundi language], urusongo; Cameroon: kifon, kre’ fom; Democratic Republic of the Congo: mulunda; Ethiopia: chau, murenchiwa/marenchiwa, nech tunaye; Kenya: abune, isoiik, isoyiot, isusa, litusta/liususa, lokitoemenyan, managu, mnavu, ol’ momoye, osuga, sujet, usuga; Rwanda: isogo, ururandayri [Kinyarwanda language]; Tanzania: enyafui [Kimaasai language], mhaki, mnavu, msogo, muhaka, ormomoi aves, soko, suga; Uganda: ensugga, enswiga/eshwiga/eswiga [Kigezi language], nouiga, nsuggaenzirugavu; Zimbabwe: m’sungu sungu-m’adzizambito.

#### Uses.

Leaves eaten as a vegetable throughout the range; also used as a medicinal plant (in Karamoja Distr., Uganda).

#### Preliminary conservation status

(IUCN 2016). *Solanum tarderemotum* is widespread across tropical Africa and can be assigned a preliminary status of LC (Least Concern; Table 7).

#### Discussion.

*Solanum tarderemotum* is a morphologically variable species. Edmonds (2012) recognised individuals with branched inflorescences as *S. florulentum* and applied *S. tarderemotum* only to material with simple inflorescences, but branched and simple inflorescences can occur on the same specimen, depending on age. Intermediate material was occasionally recognised in some accounts under the unpublished name “*Solanum eldorettii*” (Olet 2004; Manoko 2007). Crosses between the forms of *S. tarderemotum* (as *S. tarderemotum* A, B, and C, and *S. florulentum*) yielded the full range of phenotypes, including intermediate individuals and both “parental” phenotypes (Manoko 2007).

*Solanum tarderemotum* is distinguished from other sympatric species by its usually many-flowered inflorescences, long-exserted style that often is bent rather than perfectly straight (easier to see in live plants), spathulate calyx lobes that are rounded at apex, very strongly reflexed fruiting pedicels and berries that drop with the pedicel attached. Stems of *S. tarderemotum* are hollow and collapse upon drying, unlike the stems of the similar *S. villosum* that are more solid. *Solanum scabrum* also has hollow stems, but fewer-flowered inflorescences and larger fruits that drop without the pedicels. *Solanum nigrum* (North African populations), *S. scabrum* and *S. villosum* all lack stone cells, while the fruits of *S. tarderemotum* consistently have 2–6 stone cells in each berry. Most material of *S. tarderemotum* is either glabrous or has eglandular pubescence, but plants from higher elevations in the eastern African mountains are glandular pubescent; these were identified as *S. pseudospinosum* by Edmonds (2012).

Previous molecular studies identified *S. tarderemotum* form C (Clade IV) as a separate entity based on AFLP data that was analysed using Maximum Parsimony phylogenetic method (Manoko 2007). Individuals clustering apart from the main clade of *S. tarderemotum* and *S. florulentum* (Clade IV) showed simple inflorescences with fewer flowers, narrower corolla lobes and green fragrant fruits at maturity distinct from majority of accessions of *S. tarderemotum* and *S. florulentum* (Manoko 2007). The results from the AFLP phylogeny are not, however, supported by sequence based molecular phylogeny based on both rapidly evolving non-coding plastid and low-copy nuclear markers (Särkinen et al. unpublished). The accession, on which *S. tarderemotum* form C (Clade IV) was based (NIJ 964750060), does not have locality or origin data and hence, we prefer assuming that the collection represents morphological variation of *S. tarderemotum* as circumscribed here, supported by molecular sequence data. The differing relationships in the AFLP phylogeny in Manoko (2007) could be explained by the general difficulty in interpreting AFLP banding patterns objectively in closely related species and highlight the issues in using AFLP data in phylogenetic analyses.

*Solanum tarderemotum* is a tetraploid species with unknown parentage (Olet et al. 2015). The species has not been included in any previous crossing and molecular studies that have focused on understanding the parental origin of the hexaploid species *S. nigrum* and *S. scabrum* (e.g. Edmonds 1977, 1979a; Ganapathi and Rao 1985, 1986b, 1986c, 1987a, 1987b; Jacoby et al. 2003; Jacoby and Labuschagne 2006; van der Walt et al. 2008; Poczai and Hyvönen 2011). It could represent one of the tetraploid parents of the hexaploids and should be included in further studies focused on understanding the origin of the polyploids.

Both names *S. tarderemotum* and *S. florulentum* were published in the same publication. No publication has yet synonymised the two. Most of the types for all names associated with our circumscription of this species were destroyed in Berlin. We have chosen to use the name *S. tarderemotum* because the holotype (WRSL) is still extant, while no known duplicates of the type of *S. florulentum* have been located.

No duplicate material has been found for the type collection of *S. dasytrichum* that was described from a specimen in B that is no longer extant; Edmonds (2012) suggested this was a synonym of *S. florulentum* with the reservation that the description did not mention forked inflorescences, but otherwise matched. In the protologue, Bitter (1913a) says he originally identified this as *S. hildebrandtii* A.Braun & C.D.Bouché (= *S. villosum*), but that it differs in having 4 stone cells; we suspect this plant is a hybrid between *S. villosum* and *S. tarderemotum*, but we place it in synonymy here due to its possession of stone cells (lacking in *S. villosum*).

#### Selected specimens examined.

**Angola**. **Huila**: Ha, juxta ripas rivi de Sopollo, Dec 1859, *Welwitsch 6034* (BM, K); **Uíge**: Santa Cruz Mission, 12 Aug 1962, *Codd 7521* (K).

**Botswana**. **North-West**: Ngami, Central Management Unit, Selinda Reserve, 8 Apr 2005, *Heath* & *Heath 1023* (K); **Burundi**. Rwegura, Territoire Kayenza, 28 May 1969, *Lewalle 3620* (K); **Bugarama**: Teza, 29 Dec 1978, *Reekmans 7400* (EA, K, MO); **Bujumbura**: Katumba, 12 Dec 1979, *Reekmans 8444* (MO); **Muramvya**: Nyabigondo, 2 Feb 1967, *Lewalle 1536* (MO).

**Cameroon**. **Nord-Ouest**: Bui, Oku-Elak, 27 Oct 1996, *Cheek et al. 8455* (K, MO); Boyo, Ijim Mountain Forest, 21 Nov 1996, *Kamundi*, *et al. 673* (K, MO); **Sud-Ouest**: Mt Kupe, Nyasoso, 23 Oct 1995, *Cheek et al. 7470* (K, MO); Mt. Cameroon R.C., 24 Mar 1961, *Swarbrick SCA271* (E).

**Cape Verde**. **Santiago**: valley of Santo Domingo, 3 Nov 1839, *Hooker 117 bis* (K).

**Chad**. **Lac**: Koulfe, Chari Central, 28 Jun 1903, *Chevalier 8788* (K, P); **N’Djamena**: c. 65 km S of Fort Lamy, 1 Jan 1965, *de Wilde et al. 5118* (K).

**Comoros**. Iles Comores, *Boivin s.n.* (W); Mohilla Island, 5 Apr 1861, *Meller s.n.* (K); **Anjouan**: sin. loc, Jun 1875, *Hildebrandt 1626[a*] (BM); **Mwali**: Insul. Mohely, 1854, *Boivin s.n.* (BM).

**Democratic Republic of the Congo**. **Katanga**: a 3 km de Lukuni (Katanga), source de la Kasapa, 12 May 1961, *Poelman 7* (K); Katuba, ferme Droogmums, Kaletele, Jan 1927, *Quarré 18* (GH); **Kivu Nord**: Ngungu, 18 km SW Sake, 11 Aug 1954, *Stauffer 48* (K, P); Kibati, **Nord-Kivu**: Butembo, 21 Feb 1974, *Baudet 487* (K); NW slope of Mt. Vislke, 24 Feb 1975, *D’Arcy 8089* (MO); **Orientale**: Ituri, Kibali, Aru, Aug 1931, *Lebrun 3575* (GH, K); **Sud Kivu**: Lac Tsimuka, plaine de la Ruzizi, Jan 1950, *Germain 5593* (K); Kalongé, Kalonge, long riviere Nyamwamba endroit fran et ombrage, 12 Feb 1953, *de Witte 10477* (MO).

**Eritrea**. **Semienawi Keyih Bahri**: c. 10 km S of Nefasit, 2 Feb 1969, *de Wilde 4502* (K).

**Ethiopia**. **Amhara**: South Gondar, 4 km N of Debre Tabor, 13 Sep 2004, *Friis et al. 11552* (K); Semien Mountains, 1 Oct 2003, *Wieringa 4971* (K); **Dire Dawa**: Dire Dawa, 16 Oct 1969, *Parker 580* (K); **Harari**: between Harrar and Abbaba, Sep 1901, *Wellby s.n.* (K); **Oromia**: Gara Mullata, c. 50 km due W of Harar, 2 Aug 1962, *Burger 2006* (FT, K); Hana, E part of the Omo, 23 Mar 1976, *Fukui 15* (EA, K); c. 5 km N of Addis Ababa, 3 May 1965, *de Wilde* & *de Wilde-Duyfjes 6507* (K, MO); **Somali**: Harerge, on the rd from Alemaya to Asbe Tafari, 6 km from Kobbo, 16 Aug 1967, *Westphal* & *Westphal-Stevels 1227* (K); **Southern Nations (SNNP)**: Lower Omo River, 7 Oct 1970, *Carr 875* (EA, K); rd from Jimma to Serbo, 14 km from Jimma, 1 Aug 1968, *Westphal* & *Westphal-Stevels 5499* (K, MO).

**Ghana**. Ohamu, Agric. Res. Station, Jul 1961, *Irvine 4957* (K).

**Guinea**. **Nzérékoré**: Nzo, Mt Nimba, 5 Nov 1969, *Adam 24671* (MO).

**Kenya**. **Central**: vicinity of Lake Naivasha, 17 Jul 1909, *Mearns 842* (GH); Meru North, Mt. Kenya area, 18 Apr 2010, *Vorontsova et al. 198* (BM, BR, EA, K, MO, NY); **Coast**: Mwanda, Mgange Nyika, 3 Oct 1971, *Klungness 74* (K); **Eastern**: Makueni, Chyulu Hills, 15 May 1938, *Bally 7787* (EA, K); **Nairobi**: Nairobi, 1924, *McDonald 808* (K); nr Nairobi, *Whyte s.n.* (K); **Rift Valley**: Mt. Elgon, Kitale, 27 Dec 1960, *Löffler E-107* (W); Kericho, Southwestern Mau Forest Reserve, camp 7, river Dimbilil, 7 Aug 1949, *Maas Geesternanus 5603* (K, MO); Katilia forest, 12 mi NNE of Kangetet, Kerio River, 25 May 1970, *Mathew 6379* (EA, K); **Western**: Kakamega, Kaptiki Secondary School, 11 Nov 1984, *Hohl 157* (EA, W); pr. Forest Station ad mar, Mt Aberdare Expedition, 14 Jan 1922, *Rob* & *Fries 921* (MO).

**Lesotho**. Sehlabathebe, 4 Jan 1973, *Guillarmod et al. 138* (K).

**Madagascar**. **Antananarivo**: Ambohidratrimo, Ambohimanga, près de Tananarive, 19 Apr 1928, *Decary 6179* (P); Antananarivo-Nord, Tananarive, *Waterlot s.n.* (P); **Toliara**: valley half a mi W of Ampoza, 5 Sep 1929, *White s.n.* (BM).

**Malawi**. **Central**: Dedza Distr., Dedza mountain forest, 19 Jan 1987, *Balaka* & *Patel 1861* (MO); **Northern**: Rumphi Distr., Nykia Plateau, 2 mi E of Chelinda, 4 Mar 1977, *Pawek 12428* (K, MO); **Southern**: Mt. Mulanje, foot NE slopes of Namasile opposite Sombani hut, 3 Jan 1971, *Hilliard* & *Burtt 6134* (E).

**Mali**. Ackerrand kurz vor Koulikoro, 29 Sep 1992, *Ehrich 336* (B).

**Mozambique**. **Maputo**: Lourengo Marques, Namaacha, 1 Aug 1967, *Marques 2142* (MO); **Zambezia**: Namuli Mountain, Muretha Plateau, 26 May 2007, *Harris 186* (K).

**Namibia**. **Kavango West**: Tondoro Mission, Tondoro Camp 1 km E of Mission, 15 Dec 1955, *de Winter 3955* (K); **Zambezi**: E Caprivi, Zipfel, Lizazuli, 2 Jan 1959, *Killick* & *Leistner 3253* (K).

**Nigeria. Bauchi**: Toro, Panshanu Pass, 15 Aug 1962, *Lawlor* & *Hall 410* (K); **Cross River**: probably collected nr Obudu Cattle Range, 25 May 1971, *Meer van*, *1861* (MO); **Enugu**: Nsukka, University of Nigeria campus, 23 Jan 1962, *Okigbo*, *57* (K); **Osun**: Shasha Forest Reserve, 23 Apr 1968, *Gledhill*, *998* (K).

**Rwanda**. **Northern**: Mt. Visoke, 6 Apr 1970, *Fossey 16* (EA, K).

**Senegal**. Nr. Bono, nec non Ins. Bonavista, *Brunner s.n.* (K); Dec 1823, *Roger 17* (K).

**Sierra Leone**. **Northern**: Mt. Bintumani, Kabala (admin), Mt Loma, Mira, 30 Nov 1965, *Adam 22258* (GH, MO).

**Somalia**. **Banaadir**: 3 km from Muqdisho airport along rd to Jasiira, 4 May 1990, *Thulin* & *Hedrén 7172* (K); **Togdheer**: Wagga Mt., 1905, *Bury s.n.* (BM); **Woqooyi Galbeed**: Murak, Sep 1933, *Godding 162* (K); mountains above Qoton, 11 Feb 2002, *Thulin 10906* (K).

**South Africa. Eastern Cape**: Somerset, 1860, *Cooper 528* (K); Fort Beaufort, 1860, *Cooper 554* (K, W); Uitenhage, Enon, *Drège s.n.* (K); **Gauteng**: Pretoria, Region SWA, Kaokoveld, Otjomborombonga on Kunene River, 13 Jul 1976, *Leistner et al. 104* (K, MO); **KwaZulu-Natal**: Weza, Ingeli, 5 Mar 1972, *Strey 10899* (K, MO); **Mpumalanga**: Matebe Valley, May 1883, *Holub s.n.* (K); **North West**: nr Vryburg, 60 mi NW of Vryburg, Kalahari Desert, 5 Feb 1948, *Rodin 3500* (K, MO).

**South Sudan**. **Bahr El Ghazal**: Isablei [?], Lande der Bongo, 26 Nov 1869, *Schweinfurth 2649* (K); **Equatoria**: Imatong Mountains, Mt. Angargi, 14 Jun 1939, *Andrews 1947* (K); Distr. Torit, Lowiliwili, Imatongs, 14 Nov 1949, *Jackson 902* (BM); **Greater Upper Nile**: Upper Nile, Boing, 26 Oct 1951, *Sherif A2891* (K).

**Sudan**. **Blue Nile**: Sennar, *Würtemmburg s.n.* (W); **Darfur**: Jebel Marra, Nyertete, 21 Jan 1964, *Wickens 1044* (K); Jebel Marra, Zalingei, *Wickens 1776* (K); **Kassala**: Erkowit, Red Sea Hills, Mar 1929, *Lady Maffey 39* (K); Nr Kamobsana, Red Sea Prov., 25 Jan 1912, *MacDougal* & *Sykes 137* (BM); Kamobsana, Red Sea Prov., 25 Jan 1912, *MacDougal* & *Sykes 139a* (BM). **Kurdufan**: ad Cordofanum Milbes, 4 Dec 1839, *Kotschy 291* (BM, E, GH, K, P, W).

**Tanzania**. **Arusha**: Mbulmbul, Block DL, 49, 24 Jun 1944, *Greenway 6951* (EA, K); Ngorogoro Crater, 24 Jun 1938, *Pole Evans* & *Erens 936 A* (E, K, P); **Buha**: Kakombe Valley, E shore of Tanganyika Lake from Gombe stream to Missonge, 25 Dec 1963, *Pirozynski P86* (EA, K); **Eastern**: Morogoro, Lukwangulu Plateau, Uluguru Mts, 19 Sep 1970, *Thulin* & *Mhoro 1048* (K); **Iringa**: Kidatu, Iringa District, E dam site, 28 Mar 1971, *Mhoro 864* (EA, K); **Kagera**: Karagwe, 1 Mar 1862, *Speke* & *Grant 453* (K); **Kigoma**: Mpanda, Sisaga, Mahali Mountains, 27 Aug 1958, *Jefford et al. 1818* (K); Kakombe, 10 mi W of Kigoma, 7 Jul 1959, *Newbould* & *Harley 4285* (K, MO); **Kilimanjaro**:, Chome Forest Reserve, Namboja, 30 Mar 2001, *Mlangwa et al. 1506* (MO); **Mbeya**: Mbeya, 3 Mar 1932, *Davies 461* (EA, K); Rungwe, Nyassa Hochland-Station Kyimbila, 1911, *Stolz 384* (B, BM, K, MO, W); **Mbeya/Njombe**: Poroto Mountains, Mbeya Distr., 16 May 1957, *Richards 9735* (EA, K); **Mbulu**: Lake Manyara National Park, 2 Dec 1963, *Greenway* & *Kirrika 11113* (EA, K); Hanang Mt., 3 May 1962, *Polhill* & *Paulo 2302* (EA, K); **Morogoro**: Kiberege, Mar 1936, *Culwick 2* (K); Kanga Mountains, 11 May 2007, *Luke et al. 12026* (EA, K, MO); **Mwanza**: Mwanza, *Davis 201* (K); **Njombe**: Stromgebeit des obern Ruhudje, Landschaft Lupembe, nordlich des Flusses, Mar 1931, *Schlieben 413* (K); **Northern**: Moshi, Weru-Weru gorge, 22 Feb 1955, *Huxley 116* (EA, K); **Rukwa**: Nsanga Forest, Mpanda, Ufipa, 8 Aug 1960, *Richards 13004* (K); **Ruvuma**: Songea, Matagoro Hills, just S of Songea, 22 Feb 1956, *Milne-Redhead* & *Taylor 8869* (EA, K); **Shinyanga**: Shinyanga, Nov 1938, *Koritschoner 1903* (EA, K); **Tabora**: Unyamwezi, Mininga, 1860, *Speke* & *Grant 79* (K); **Tanga**: Usambaras, between Ngua and Magunga Estates, 17 Jul 1953, *Drummond* & *Hemsley 3347* (EA, K); **Ufipa**: Sumbawanga, 3 km SE of Moravian Mission at Nkutwe nr Tatanda, 31 Oct 1992, *Harder & Kayombo 1351* (EA, MO); **Ulanga**: Ifakara, 16 Jul 1959, *Haerdi 286o* (EA, K).

**Uganda**. **Central**: Mukono, Kipayo, Dec 1913, *Dümmer 563* (BM, K, P); Mengo, 12 mi to Kampala, Entebbe rd, May 1932, *Eggeling 697* (EA, K); **Eastern**: Mt. Elgon, 25 Jan 1993, *Katende* & *Sheil 1099* (K); **Northern**: Karamoja, Moroto Township, Sep 1958, *Wilson 609* (EA, K); **Western**: Kigezi D.F.I, 28 Aug 1972, *Goode G-2-72* (K); Mihunga, Ruwenzori, 12 Jan 1939, *Loveridge 350* (A, K, MO); Kigezi, Muhavura Hill, 11 Jan 1933, *Rogers 337* (BM, EA, K).

**Zambia**. **Lusaka**: Mt. Makulu, Kafue Basin, 10 Apr 1963, *van Rensberg 1886* (K); **North-Western**: Mwinilunga, 4 Oct 1937, *Milne-Redhead 2565* (K); **Northern**: below Kwimbi Mission, 10 Feb 1955, *Richards 4426* (K); **Southern**: Livingstone Distr., Victoria Falls, 21 Feb 1997, *Luwiika et al. 460* (MO).

**Zimbabwe**. **Harare**: Salisbury, 26 Feb 1927, *Eyles 4712* (K); **Manicaland**: Nr Chirinda, May 1906, *Swynnerton 481* (BM, K); **Mashonaland Central**: Mazowe, Umvukwes, 17 Dec 1952, *Wild 40771* (K); **Mashonaland East**: Distr. Salisbury, Mandara, 25 Sep 1974, *Bisgel 4632* (MO); Marandella, 8 Apr 1948, *Corby 1-20917* (K); **Matabeleland South**: Mazowe, Matobo National Park, Matopus NP, 24 Feb 1981, *Philcox* & *Leppard 8823* (K).

### 17. Solanum triflorum

Taxon classificationPlantaeSolanalesSolanaceae

Nutt., Gen. N. Amer. Pl. 1: 128. 1818

Figures 52 , 53

Solanum triflorum Nutt. var. majus Hook., Fl. Bor.-Amer. 2: 90. 1837, as “*major*”.
Type. Canada. Saskatchewan: “Carleton House Fort, Saskatchewan River”, *J. Richardson s.n.* (lectotype, designated here: BM [BM000934745]; isolectotype: K [K001159656, large plants]).
Solanum triflorum Nutt. var. minus Hook., Fl. Bor.-Amer. 2: 90. 1837, as “*minor*”.
Type. Canada. Saskatchewan: “In the Garden (a weed) of Carleton House Fort, entrance of Badger’s Hole, and Saskatchewan River to Edmonton House [protologue]”, *T. Drummond s.n.* (lectotype, designated here: E [E00526685]; isolectotypes: BM [BM000934744], K [K001159656]).
Solanum mendocinum Phil., Anales Univ. Chile 21(2): 403. 1862.
Type. Argentina. Mendoza: Mendoza, 1860–1861, *W. Díaz s.n.* (lectotype, designated by Barboza et al. 2013, pg. 260: SGO [SGO000004580]).
Solanum calophyllum Phil., Anales Univ. Chile 21(2): 403. 1862.
Type. Argentina. Mendoza: Mendoza, 1860–1861, *R. Philippi s.n.* (lectotype, designated here [cited as holotype in Barboza et al. 2013]: SGO [SGO000004552]; isolectotype: G [G00343450]).
Solanum pyrethrifolium Griseb., Abh. Königl. Ges. Wiss. Göttingen 24: 250. 1879.
Type. Argentina. Tucumán: Lules, Dec 1873, *P. G. Lorentz & G. Hieronymus* 1132 (lectotype, designated by Morton 1976, pg. 102: CORD [CORD00006111]; isolectotype: GOET [GOET003594]).
Solanum gaudichaudii Dunal var. pyrethrifolium (Griseb.) Kuntze, Revis. Gen. Pl. 3(3): 226. 1898.
Type. Based on Solanum pyrethrifolium Griseb.
Solanum triflorum Nutt. var. calophyllum (Phil.) Bitter, Abh. Naturwiss. Vereine Bremen 23: 144. 1914.
Type. Based on Solanum calophyllum Phil.
Solanum triflorum Nutt. var. pyrethrifolium (Griseb.) Bitter ex Probst, Mitteil. Naturfor. Gesellsch. Solothurn 9: 41. 1932.
Type. Based on Solanum pyrethrifolium Griseb.
Solanum ponticum Prodan, Bul. Fac. Agron. Cluj 7: 49. 1938.
Type. Romania. Satul noi, in arenosis, *I.E. Nyárádu s.n.* (no original material located; BUCA?, CLA?).
Solanum triflorum Nutt. var. ponticum (Prodan) Borza, Bul. Grad. Bot. Univ. Cluj 22: 20. 1942.
Type. Based on Solanum ponticum Prodan
Solanum triflorum Nutt. var. dentatum Ooststr., Gorteria 3: 90. 1966.
Type. Netherlands. Gelderland: Nijmegen, bij molen aan de Graafsweg, Jul 1938, *B. Reichgelt & T.J. Reichgelt s.n. [Herb. Kern & Reichgelt 4610*] (holotype: L [L3384989 [old barcode L0860668], acc. # 951.219 101]).
Solanum triflorum Nutt. forma malvinum De Langhe & D’hose, Belg. J. Bot. 119(2): 160. 1987 [“1986”].
Type. Belgium. Antwerpen, 14 Oct 1984, *J.-E. De Langhe & R. D’hose s.n. [219/84*] (holotype: “in herbario De Langhe sub numero 219/84”, not seen, BR?).

#### Type.

United States of America. North Dakota: Nr Fort Mandan, *Anon. [Lewis & Clark] s.n.* (lectotype, designated by Barboza et al. 2013, pg. 260: PH [PH00030496]).

#### Description.

Annual, decumbent and prostrate herbs to 40 cm tall, much branched at the base, to 70 cm in diameter. Stems decumbent to ascending, terete, green, forming adventitious roots at the nodes, not markedly hollow; new growth glabrous to sparsely pubescent with simple, spreading, uniseriate, translucent, eglandular trichomes, these (3-)4–10-celled, 0.5–2.0 mm long, occasionally with a few glandular trichomes with a 1-many-celled apical gland; older stems glabrescent. Sympodial units difoliate or trifoliate, the leaves not geminate. Leaves simple and shallowly lobed to deeply pinnatifid, (0.5-)2.0–4.0(-5.0) cm long, 0.2–2.9 cm wide, narrowly elliptic to oblong- to ovate-elliptic, fleshy in texture, concolorous, without smell; adaxial surface glabrous to sparsely pubescent with simple, uniseriate trichomes like those on stem, scattered along lamina and more densely along the veins; abaxial surface more densely pubescent on veins and lamina; major veins 3–6 pairs, not clearly evident abaxially; base cuneate, decurrent on the petiole; sinuate-lobate to deeply pinnatifid to near-pinnate, with 3–6 linear to triangular pairs of lobes; apex acute; petioles (0.5-)1.0–2.0(-2.4) cm long, pubescent with simple uniseriate trichomes like those of the stems. Inflorescences 1.0–2.0 cm long, internodal, simple, umbelliform to sub-umbelliform, with 1–5(-6) flowers clustered near the tip, glabrous to sparsely pubescent with spreading trichomes like those of the stems; peduncle 0.8–3.5 cm long, straight, often with apical leafy “bracteoles”; pedicels 3–12 mm long, 0.4–0.5 mm in diameter at the base and 0.4–0.5 mm at apex, straight and spreading, articulated at the base; pedicel scars spaced 0(-0.5) mm apart. Buds narrowly ellipsoid or occasionally narrowly ovoid, the corolla exserted 1/5–2/5 from the calyx tube before anthesis. Flowers 5-merous, all perfect. Calyx tube 1.0–1.5 mm long, conical, the lobes 2.5–3.5(-7) mm long, 0.8–1.0(-4) mm wide, triangular-oblong with acute apices, densely pubescent with simple, uniseriate eglandular trichomes like those of the stem. Corolla 10–14 mm in diameter, white to lilac with a yellow-green central eye with black-purple colouration at the base, stellate, lobed 1/2–1/3 to the base, the lobes 4.0–5.0 mm long, 1.8–2.2 mm wide, reflexed at anthesis, densely pubescent abaxially with short simple uniseriate eglandular trichomes like those on stems and leaves. Stamens equal; filament tube minute; free portion of the filaments 0.6–1.0 mm long, adaxially sparsely pubescent with tangled simple, uniseriate trichomes; anthers 2.8–3.1 mm long, 0.4–0.5 mm wide, narrowly ellipsoid, pale yellow, poricidal at the tips, the pores lengthening to slits with age and drying. Ovary globose, glabrous; style 2.5–3.5 mm long, densely pubescent with 2–3-celled simple uniseriate trichomes in the lower half, not exserted beyond the anther cone; stigma capitate, minutely papillate, green in live plants. Fruit a globose berry, 8–10(-20) mm in diameter, fleshy, dark green at maturity, the pericarp usually shiny; fruiting pedicels 12–17 mm long, 0.5–1.0 mm in diameter at the base, 1.0–1.5 mm at apex, spaced 0–0.5(-1.0) mm apart, reflexed and becoming woody, dropping with mature fruits, not persistent; fruiting calyx not markedly accrescent but the lobes slightly elongating in fruit, the tube less than 1 mm long, the lobes (4.0-)4.5–5.5(-8.0) mm long, strongly reflexed to spreading. Seeds 40–60 per berry, 2.0–2.5 mm long, 1.7–2.0 mm wide, subglobose, yellow, the surfaces minutely pitted, the testal cells pentagonal in outline. Stone cells 13–30, 1.0–1.5 mm in diameter. Chromosome number: 2*n*=2*x*=24 (Moscone 1992; Moyetta et al. 2013).

**Figure 52.**
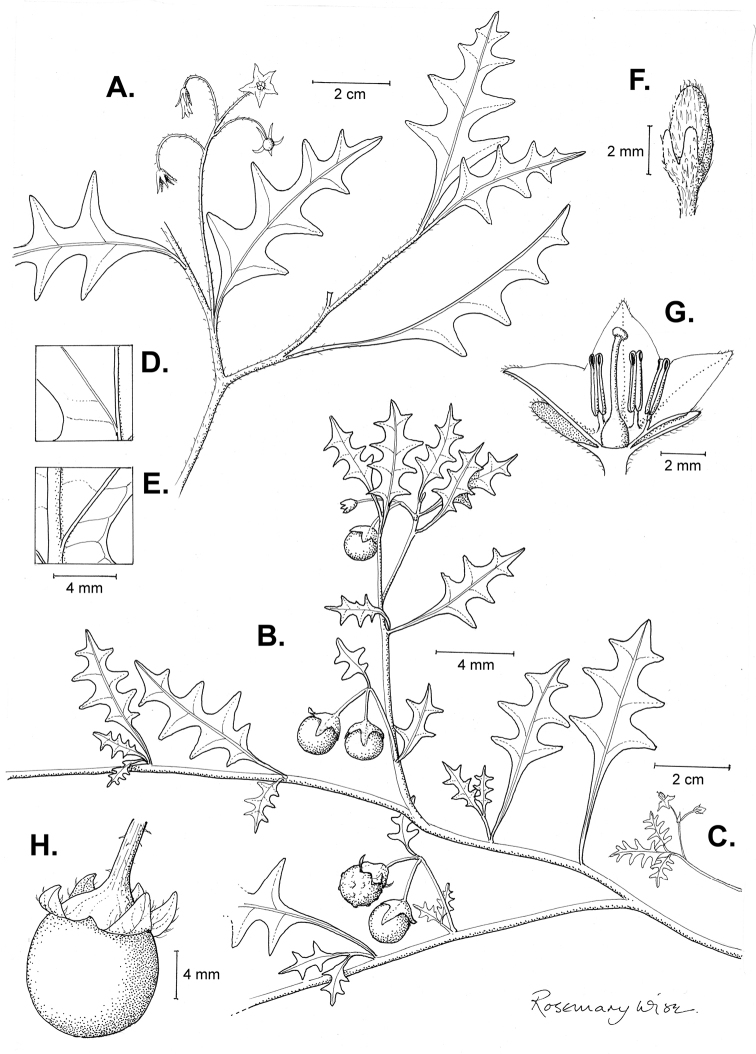
*Solanum triflorum* **A** Flowering habit **B** Fruiting habit **C** Flowering branch **D** Detail of adaxial leaf surface **E** Detail of adaxial leaf surface **F** Bud **G** Flower **H** Fruit (**A, C, F–G** *Donat 55*; **B, D–E, H** *Baker 577*). Drawing by R. Wise.

**Figure 53.**
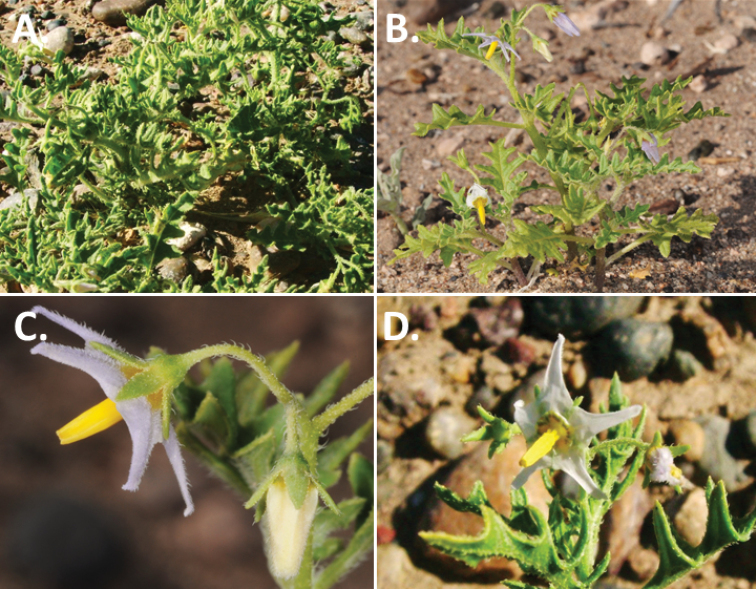
*Solanum triflorum* **A** Habit **B** Flowering habit **C** Flower and flower bud **D** Flower. (**A, D** *Barboza et al. 2345*; **B–C** *Sérsic 5040*). Photos by G. Barboza and A. Sérsic.

#### Distribution

**Figure 54.**
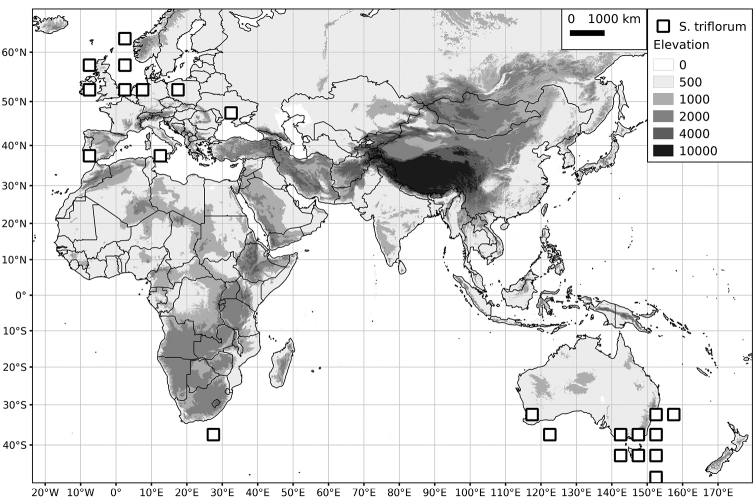
Distribution of *Solanum triflorum* in its non-native range in the Old World.

(Figure 54). Native to the Americas with an amphitropical distribution between temperate South and North America. Introduced outside its native range in Europe, South Africa and Australia, probably with agricultural seed or wool waste.

#### Ecology.

Grows across a wide range of habitats, along road sides, sandy soils, in cultivations and in salt plains; between sea level and 2,300 (-2,900) m elevation in its native range, between sea level and 1,800 m in the introduced range.

#### Common names.

Australia: cut-leaf nightshade (Walsh and Entwisle 1999), three-flowered nightshade (Harden 1993); United Kingdom: small nightshade (Stace 2010).

#### Uses.

None recorded; a weed of agriculture.

#### Preliminary conservation status

(IUCN 2016). *Solanum triflorum* is a weedy species that is invasive where introduced; it has a large EOO and can be assigned a preliminary status of LC (Least Concern; Table 7). The EOO, based on native range only, is also very large (19,993,876 km^2^), mainly due to the disjunct distribution between North America and southern South America.

#### Discussion.

*Solanum triflorum* is a weed of agricultural areas and appears not to spread aggressively based on collection numbers (see Knapp 2018). It is easily distinguished from other species in the Old World by its usually deeply lobed leaves that are often pinnatifid, but are sometimes only shallowly lobed (see van Ooststroom 1966; description of var. dentatum), the usual presence of a bracteole in the inflorescence, very narrowly elliptic buds and berries with > 30 stone cells per berry.

In its native range, *S. triflorum* has a very large range of berry sizes; most introduced material has larger berries, but it is possible we have not seen material that has the smaller berry size from the Old World. There is also large variation in the indumentum and leaf shape in *S. triflorum* in its native range (Subils 1989), some of which can be seen in the Old World material. This variation is likely caused by environmental factors and has no taxonomic relevance (Subils 1989).

The varieties of *S. triflorum* described by Hooker (1837) were described from plants collected on the second Franklin Expedition of 1825–1827, whose principal botanist was John Richardson and assistant botanist Thomas Drummond. William J. Hooker saw the material while he was in Glasgow and two specimens at E were originally from there; the material from the expedition eventually came to the Natural History Museum (BM), but some is also held at K. We have selected as the lectotype of var. minus the sheet labelled “S. trifidum β” (= var. minus) at E (E00526685) which has tiny plants and consider the small plants (also labelled “β” and on a sheet together with larger plants with a separate label) at BM (BM000934744) as isolectotype material. The sheet at K (K001159656) has both small and large plants mounted on it and is labelled “S. triflorum α & β”, but here the small plants are labelled” α”; we think these are mislabelled at K and are actually part of what Hooker was recognising as var. minus (his β). We select the large plants (BM000934745) as the lectotype of var. majus and assign the large plants on the K sheet (K001159656) as isolectotype material.

#### Selected specimens examined.

**Australia**. **New South Wales**: Queanbeyan, Queanbeyan Stockyards, 5 Feb 1931, *Calvert KEW-84* (CANB, PERTH); Murrumbidgee River, Dew’s Corner 4 km S of Fairvale Homestead, 8 May 1987, *Canning* & *Lloyd 6421* (AD, CANB, NSW); Gundaroo Creek, 4.7 km N of Gundaroo on rd to Gunning, 23 Mar 1991, *Lepschi 542* (AD, CANB, K); Fyshwick, Australian Capital Territory, 7 Apr 1991, *Lepschi 555* (CANB, HO); Mid-Western Regional, Bylong district, 11 Oct 1953, *McKee 654* (MEL); Armidale Dumaresq, Armidale, 10 Jan 1973, *Williams s.n.* (NE); **Queensland**: Southern Downs, Stanthorpe, 24 Feb 1954, *Alison s.n.* (BRI); Southern Downs, Ballandean, 23 Jan 1974, *Booth s.n.* (BRI); Southern Downs, 8 km of Ballandean, 20 Feb 1974, *Swann s.n.* (BRI); Southern Downs, Wallangarra, 22 Dec 1958, *Taylor s.n.* (BRI); **South Australia**: Region 9, Murray, 28 May 1972, *Alcock 3939* (AD, CORD); Culburra, c. 80 km NW of Bordertown, 12 Feb 1963, *Fry s.n.* (E); Keith, 5 Apr 1965, *Kain s.n.* (K); NSW Finley, 30 Jan 1975, *Symon 9807* (AD, MO, NSW); Murray Bridge, northern fenceline of Monarto Conservation Park, 6 Feb 1998, *Taylor 291a* (AD, MEL); **Tasmania**: Pitt Water, Seven Mile Beach Protected Area, 31 Mar 2006, *Baker 1707* (CANB, HO, MEL); Clarence, Seven Mile Beach, 3 Apr 2000, *Buchanan 15695* (AD, HO); **Victoria**: East Gippsland, 6 km NW of Mt Deddick, 17 Apr 1981, *Forbes* & *Rees 909* (AD, MEL); Banyule, nr Montpelier Billabong, 7 Mar 2011, *Lynch s.n.* (CANB, HO, MEL); **Western Australia**: Wickepin, 17 Jan 1949, *Eccles s.n.* (PERTH)Beverley, 25 km E of Brookton, *Hambly s.n.* (PERTH); Steerdale, John Forrest rd, Hopetoun, 3 Dec 1997, *Hill s.n.* (PERTH); Swan, Midland, 12 mi E of Perth, Jan 1968, *Moir s.n.* (AD); Brookton, *Williams* & *Williams s.n.* (PERTH).

**Austria**. **Wien**: Wien, Müllplatz Laaerberg, 1 Sep 1969, *Forstner* & *Melzer s.n.* (W).

**Belgium. Flanders**: De Panne, 15 Aug 1976, *Goetghebeur 2605* (H, MO); Gent, 7 Aug 1983, *Goetghebeur* & *Pauw De 5252* (H, MO, UT); Westhoek, 15 Aug 1976, *Goetghebeur 8626* (W); Antwerp, Linkeroever, Middenvijver, 22 Sep 2009, *Wieringa 6785* (BM, W).

**France**. **Hauts-de-France**: Pas-de-Calais, Camiers, au NW d’Etaples, chemin conduissant a la plage Saint Gabriel, 30 Oct 1973, *Lambinon et al. 1461* (BM, H).

**Germany**. **Nordrhein-Westfalen**: Niederrhein, Emmerich, 20 Oct 1929, *Krüger 700* (W).

**Morocco**. **Oriental**: Oued Charef, au nord de Ain-Benimathar, 21 Jun 2008, *Calvo et al. 2501* (MA).

**Netherlands**. **Zuid Holland**: Isle of Rosenburgh, De Beer, 26 Aug 1948, *van Hattum 4073* (MO); Isle of Rozenburg, De Beer, 26 Aug 1948, *van Hattum 4073* (K); Goeree, Havenhoofd, 13 Aug 1977, *Podlech 8624* (BM, H, MO).

**Romania**. Moldova, Distr. Covurlui, stationem viae ferreae Fulgeresti, 6 Sep 1922, *Petruscu* & *Borza 2465* (W).

**South Africa. Eastern Cape**: Morgendal, 10 Feb 1960, *Acocks 21049* (EA).

**Sweden**. **Götaland**: Skåne, Malmö, Sep 1906, *Hylmö s.n.* (BM).

**Tunisia**. **Gabes**: Gabes, Djebel Dirra, Apr 1909, *Pitard s.n.* (MA).

**United Kingdom. England**: nr Hartley, Wintney Hants, 17 Aug 1949, *Hodge 4288* (K); Essex, Barking, 17 Oct 1953, *Lousley s.n.* (BM); Northumberland, Holy Island, 5 Sep 1936, *Hutton s.n.* (K); Northumberland, Holy Island, 13 Sep 1955, *Lousley s.n.* (BM); Suffolk, Woodbridge, 14 Sep 1932, *Milne-Redhead* & *Airy Shaw 1834* (K); **Scotland**: Possil, Glasgow, 11 Sep 1918, *Grierson 955-18* (K); **Wales**: Gwynedd, Caernarfonshire, Dinas Dinlle, *Herrington s.n.* (BM); Cardiff, Cardiff, 22 Aug 1925, *Smith s.n.* (BM).

### 18. Solanum umalilaense

Taxon classificationPlantaeSolanalesSolanaceae

Manoko, PhytoKeys 16: 67. 2012

Figure 55

#### Type.

Tanzania. Mbeya: Mbeya District, Umalila Forest Reserve, ca. 7 km W of Ruanda II on road to Izumbwe, 2 km SSE of Mbogo Mtn. main peak, 9°11’S, 33°18’E, 2180 m, 14 Nov 1992. *R.E. Gereau, D.K. Harder, C.J. Kayombo & M.J. Kayombo 5084* (holotype: DSM; isotypes: EA, K [K001081869], MO [MO-2293667, acc. # 4281580], NHT).

#### Description.

Annual to short-lived erect perennial herbs to 0.5 m tall, erect, predominantly branching from the base. Stems spreading, ridged or winged, dark purple to green, erect, not markedly hollow; new growth puberulent with simple, spreading, uniseriate, translucent, eglandular trichomes, these 2–8-celled, 0.3–0.8 mm long; older stems glabrescent. Sympodial units difoliate, the leaves not geminate. Leaves simple, 1.8–2.8 cm long, 0.1–1.7 (-2.1) cm wide, ovate to elliptic, membranous, green to dark green above and below, without smell; adaxial and abaxial surfaces glabrous or sparsely pubescent with simple uniseriate trichomes like those on the stem mainly along veins; major veins 3–4 pairs, paler green or often purple tinged; base truncate, narrowly winged on to the petiole; margins entire; apex acuminate to acute; petioles 0.6–1.3 cm long, glabrous or sparsely pubescent with simple uniseriate trichomes like those of the stem. Inflorescences 2–6 cm long, internodal, simple or usually branched and often leafy, the flowers spaced along the rhachis, with 2–9(-20) flowers, glabrous or sparsely pubescent with simple uniseriate trichomes like those on the stem; peduncle (0.5-)1.1–4 cm long, erect, ca. 0.5 mm thick at apex, stout; pedicels 5–7(-9) mm long, ca. 0.2 mm in diameter at the base, ca. 0.5 mm in diameter at the apex, stout, pendent, articulated at the base; pedicel scars widely spaced (1-)3–8 mm apart. Buds subglobose, the corolla exserted 1/2 from the calyx tube before anthesis. Flowers 5-merous, perfect. Calyx tube 0.8–1.0 mm long, cup-shaped, the lobes equal, 0.1–0.5 mm long, 0.5–0.7 mm wide, broadly deltate with an acute tip, green or purple-tinged, glabrous or sparsely pubescent with simple uniseriate trichomes like those of the pedicels. Corolla 7–11 mm in diameter, white, margins occasionally tinged with purple, with a yellow basal star, stellate, lobed ca. 1/2 way to the base, the lobes 2.5–3.5 mm long, 2–3 mm wide, spreading or reflexed, densely papillate abaxially. Stamens equal; filament tube to 0.1 mm long; free portion of the filaments 0.5–1.0 mm long, pubescent adaxially with tangled uniseriate simple trichomes; anthers 1.8–2 mm long, 0.5–0.6 mm wide, ellipsoid, yellow, poricidal at the tips, the pores lengthening to slits with age and drying. Ovary rounded, glabrous; style 3.1–3.5(-4.2) mm long, densely pubescent with simple uniseriate trichomes 0.2–0.5 mm long in the basal 1/2–2/3, exserted 1.0–1.5 mm beyond anther cone; stigma clavate, the surface minutely papillate. Fruit a globose berry, 3–4(-5) mm in diameter, pale yellow-brown at maturity, aromatic, the pericarp soft, matte, not transparent; fruiting pedicels 0.8–1.3 cm long, 0.3–0.5 mm in diameter at the base, 0.5–1.0 mm in diameter at the apex, stout and becoming woody, erect or spreading, pale brown, falling with the mature fruit, not persistent; fruiting calyx not accrescent, the tube ca. 1 mm long, the lobes ca. 1 mm long, appressed to the berry. Seeds (5-)6–12 per berry, 1.6–2.1 mm long, 1.3–1.8 mm wide, flattened and tear-drop shaped with a subapical hilum, brownish, the surfaces minutely pitted, thin, the testal cells rectangular to pentagonal in outline. Stone cells 9–22 per berry, ca. 0.5 mm in diameter. Chromosome number: *2n*=4x=48 (Manoko et al. 2012).

**Figure 55.**
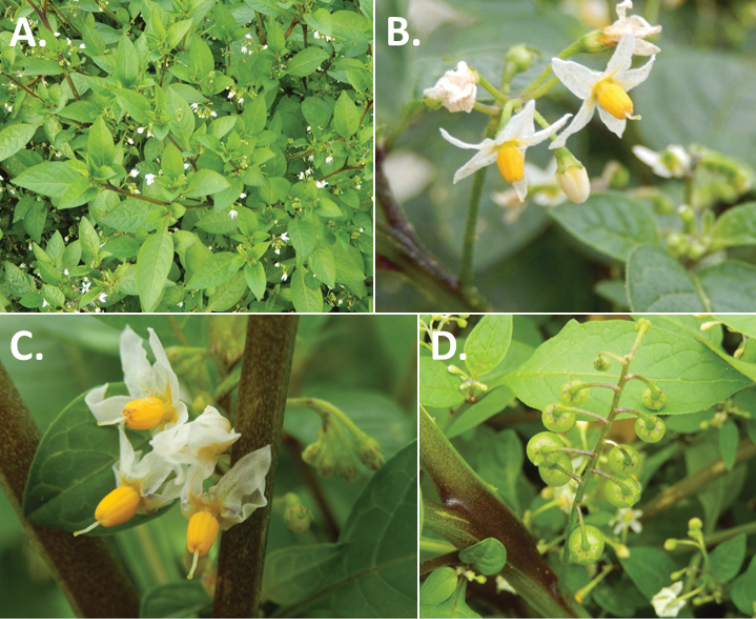
*Solanum umalilaense* **A** Habit **B** Flower and buds showing calyx shape **C** Inflorescence and leaves **D** Infructescence showing detail of winged stem. (**A–D** NIJ-A24750133). Photos by G. van der Weerden.

#### Distribution

**Figure 56.**
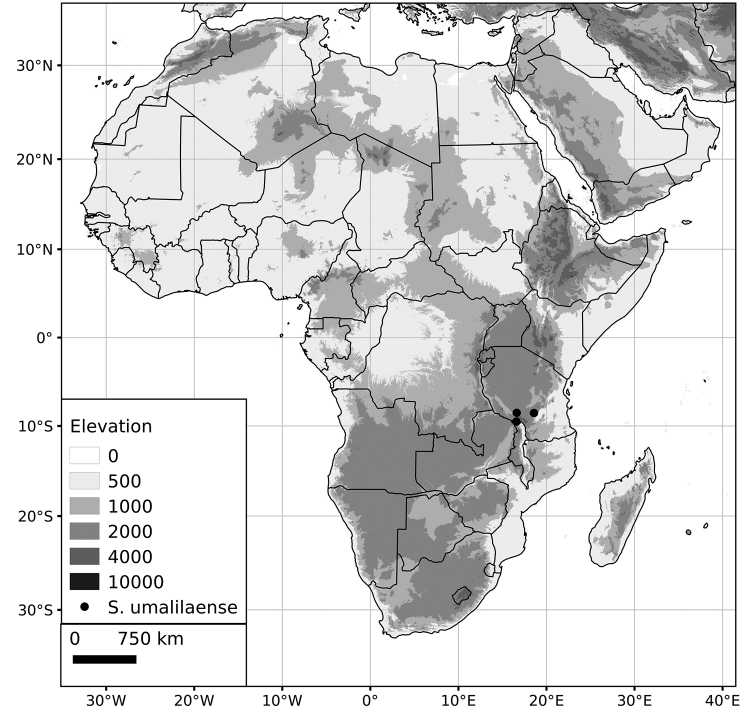
Distribution of *Solanum umalilaense*.

(Figure 56). Endemic to the southern highlands of Tanzania.

#### Ecology.

Grows on volcanic soils, frequent on the ash layer in charcoal-burning areas, also commonly cultivated; between 1,700 and 2,200 m elevation.

#### Common names.

Tanzania: insungwe [Malila people] (Schippers 2000).

#### Uses.

Leaves used as spinach; berries eaten raw.

#### Preliminary conservation status

(IUCN 2016). *Solanum umalilaense* is known mostly from cultivation in the southern part of Tanzania; based on its range (EOO = 2,559 km^2^; EN) and the number of populations (AOO = 32 km^2^), it would be assigned a preliminary conservation status of EN (Endangered), but might be better considered DD (Data Deficient). It is protected by local people and its range is poorly known.

#### Discussion.

*Solanum umalilaense* can be distinguished from other African species of morelloids by its simple to branched and often leafy inflorescences, flowers with very short rounded calyx lobes and persistent, small, light yellowish-brown fruits. *Solanum umalilaense* produces a large number of inflorescences such that, at full anthesis, the plant appears to be covered with white flowers, strikingly different from other species in the section (Manoko et al. 2012), except some populations of *S. tarderemotum* (the branched-inflorescence form previously recognised as *S. florulentum*). Like *S. tarderemotum*, the berries fall with the pedicels still attached.

Manoko (2007) used AFLP molecular markers to assess the relationships of the tetraploid African species and found that *S. umalilaense* (as “Sp. A”) was a member of a cluster including *S. retroflexum* (as *S. hirsutum* (Vahl) Dunal and *S. retroflexum*) that itself clustered with *S. tarderemotum*.

More field work is needed in southern Tanzania and adjacent Zambia to determine the true range and morphological variation of *S. umalilaense*; collections analysed to date come from what might be a single population.

**Specimens examined. Tanzania**. **Iringa**: Mufindi, Igowole, 10 Mar 1989, *Kayombo* & *Kayombo 215* (MO); **Mbeya**: Maganjo village, Lwindi ward, 8 Jul 2010, *Manoko 2010-1* (DSM, NIJ, WAG); Isangati village, Iyunga ward, 8 Jul 2010, *Manoko 2010-2* (DSM, NIJ, WAG); Igala village, Holondo ward, 8 Jul 2010, *Manoko 2010-8* (DSM, NIJ, WAG); Isangati village, Iyunga Mapinduzi ward, 9 Jul 2010, *Manoko 2010-11* (DSM, NIJ, WAG); Isangati village, Iyunga Mapinduzi ward, 9 Jul 2010, *Manoko 2010-12* (DSM, NIJ, WAG); nr Umalila Forest reserve, 9 Jul 2010, *Manoko 2010-14* (DSM, NIJ, WAG); Uyole, Mbeya, 22 May 1968, *Mwambunga 6* (DSM).

### 19. Solanum villosum

Taxon classificationPlantaeSolanalesSolanaceae

Mill., Gard. Dict. ed. 8, no. 2. 1768

Figures 57 , 58

Solanum nigrum L. var. villosum L., Sp. Pl. 186. 1753.
Type. “Solanum annuum hirsutus, baccis luteis”, cultivated in England, at James Sherard’s garden in Eltham (Hortus Elthamensis) (lectotype, designated by Henderson 1974, pg. 56: Dillenius, Hortus Elthamensis 2: 366, t. 274, f. 353. 1732).
Solanum luteum Mill., Gard. Dict. ed. 8, no. 3. 1768.
Type. “Solanumofficinarum lacinis luteis 1735, G.B. 166”, cultivated in England at the Chelsea Physic Garden, seeds said to come from America, *Herb. Miller s.n.* [691 on sheet] (lectotype, designated by Edmonds 1979b, pg. 214: BM [BM000942567]).
Solanum rubrum Mill., Gard. Dict. ed. 8, no. 4. 1768.
Type. Cultivated in England at the Chelsea Physic Garden, origin said to be the West Indies, *Herb. Miller s.n.* (lectotype, designated by Edmonds 1979b, pg. 215: BM [BM000942563]).
Solanum nigrum L. subsp. villosum (L.) Ehrh., Hannover. Mag. 1780, 14: 218. 1780.
Type. Based on Solanum nigrum L. var. villosum L.
Solanum villosum (L.) Willd., Prodr. Fl. Berol.: 87. 1787, nom. illeg., not Solanum villosum Mill. (1768)
Type. Based on Solanum nigrum L. var. villosum L.
Solanum nigrum L. var. rubrum (Mill.) Aiton, Hort. Kew. [W.Aiton] 1: 234. 1789.
Type. Based on Solanum rubrum Mill.
Solanum aegyptiacum J.F.Gmel., Syst. Nat., ed. 13 [bis] 2: 385. 1791.
Type. Egypt. Cairo: “circa Cairo”, 1762, *Herb. P. Forsskål 420* (lectotype, designated here: C [C10013600]).
Solanum villosum Lam., Tabl. Encycl. 2: 18. 1794, nom. illeg., not Solanum villosum Mill. (1768)
Type. “Ex Gallia australi, &c.”, *Anon. s.n.* (lectotype, designated by Edmonds 1979b, pag. 214 [as holotype]: P-LA [P00357624]).
Solanum villosum Moench, Methodus (Moench) 474. 1794, nom. illeg., not Solanum villosum Mill. (1768).
Type. Germany. Hessen [?], *C. Moench s.n.* (no original material located).
Solanum alatum Moench, Methodus (Moench) 474. 1794, nom. cons. prop
Type. Pakistan. Balochistan: Quetta, 30 km E Gumbaz, 1050 m, 30°02’N, 69°00’E, 17 May 1965, *K.H. Rechinger 29684* (type cons. prop. by Knapp et al. 2017, pg. 988: W acc. # 1972-0017910).
Solanum villosum (L.) Willd., Enum. Pl. (Willdenow) 236. 1809, nom. illeg., not Solanum villosum Mill. (1768).
Type. Based on Solanum nigrum L. var. villosum L.
Solanum humile Bernh. ex Willd., Enum. Pl. (Willdenow) 236. 1809.
Type. Cultivated in Berlin Botanic Garden, from “Europa australi” [southern Europe], *Anon. s.n.* (neotype, designated by Edmonds 2012, pg. 132 [as lectotype]: B [B-W04367 sheet 2]; isoneotype: B [B-W04367 sheet 1]).
Solanum suffruticosum Schousb. ex Willd., Enum. Pl. (Willdenow) 236. 1809.
Type. Cultivated in Berlin Botanic Garden, said to be originally from “Barbaria” [northern Africa west of Egypt], *Herb. Willdenow s.n.* (lectotype, designated here: B [B-W04363010]).
Solanum incertum Dunal, Hist. Nat. Solanum 155. 1813.
Type. “Nelen-Tsjunda” from Malabar [western India] (lectotype, designated here: Rheede von Draakestein, Hortus Malabaricus 10: t. 73. 1690).
Solanum erythraeum Dunal, Hist. Nat. Solanum 238. 1813, nom. illeg. superfl.
Type. Based on Solanum rubrum L. (as S. rubrum “Murr. Syst. Veg 183, Gmel. Syst. Nat 384”)
Solanum kitaibelii Schult., Öster. Fl., ed. 2, 1: 395. 1814.
Type. Hungary. Sin. loc. “Pannonia”, *Without collector [P. Kitaibel] s.n. [349*] (lectotype, designated by Edmonds 1979b, pg. 214 [as holotype]: M [M-0166013]).
Solanum flavum Kit. ex Schult., Öster. Fl., ed. 2, 1: 394. 1814.
Type. Hungary. Sin. loc. [“Pannonia”], *Without collector [P. Kitaibel] s.n.* (lectotype, designated by Edmonds 1979b, pg. 214 [as holotype]: B-W [B-W04421010]; probable isolectotypes: BP [herb. Kit. fasc. IX: 105, 3 sheets], M [M0164200]).
Solanum ochroleucum Bastard, J. Bot. (Desvaux) 3: 20. 1814.
Type. France. Pays de la Loire: Maine-et-Loire, Anjou [Angers], 1813, *Without collector [prob. T. Bastard] s.n.* (lectotype, designated here: G-DC [G00144474]; isolectotype: ANG [acc. # 2006.213]).
Solanum nigrum L. var. miniatum (Bernh. ex Willd.) Fr., Nov. Flor. Suec.: 111. 1823.
Type. Based on Solanum miniatum Bernh. ex Willd.
Solanum villosum Lam. var. miniatum (Bernh. ex Willd.) Kostel., Clav. Anal. Fl. Bohem. 34. 1824.
Type. Based on Solanum miniatum Bernh. ex Willd.
Solanum luteovirescens C.C.Gmel., Fl. Bad. 4: 177. 1826, nom. illegit. superfl.
Type. Based on S. humile Bernh. ex Willd. (cited in synonymy).
Solanum puniceum C.C.Gmel., Fl. Bad. 4: 176. 1826, nom. illegit. superfl.
Type. Based on S. miniatum Bernh. ex Willd. (cited in synonymy).
Solanum vulgatum (L.) Spenn. var. luteum Spenn., Fl. Friburg. 2: 427. 1829, nom. illeg. superfl.
Type. Based on Solanum nigrum L. var villosum L. (cited in synonymy)
Solanum nigrum L. subvar. ochroleucum (Bastard) Coss. & Germ., Intr. Fl. Anal. et Descr. Paris, 91. 1842.
Type. Based on Solanum ochroleucum Bastard
Solanum villosum Lam. subvar. miniatum (Bernh. ex Willd.) Germ. & Wedd., Introd. Fl. Anales Paris 91. 1842.
Type. Based on Solanum miniatum Bernh. ex Willd.
Solanum villosum Lam. forma miniatum (Bernh. ex Willd.) Döll, Rhein. Flor. 413. 1843.
Type. Based on Solanum miniatum Bernh. ex Willd.
Solanum nigrum L. forma humile (Bernh. ex Willd.) Döll, Rhein. Flor. 413. 1843.
Type. Based on Solanum humile Bernh. ex Willd.
Solanum nigrum L. forma villosum (Lam.) Döll, Rhein. Flor. 413. 1843.
Type. Based on Solanum villosum Lam.
Solanum nigrum L. forma angulosum Döll, Rhein. Flor. 413. 1843.
Type. Germany. “Bei Carlsruhe beim Linkenheimer Thore, *A. Braun*” (no specimens cited; no original material located, not found at KR); Germany. “Bei Philippsburg, *Stud. Bauer*” (no specimens cited; no original material located, not found at KR): Germany. “Bei Mannheim, Oppenheim, *J.C. Döll*” (no specimens cited; no original material located, not found at KR).
Solanum nigrum L. subsp. miniatum (Bernh. ex Willd.) Hartm., Sv. Norsk Exc.-Fl.: 35. 1846.
Type. Based on Solanum miniatum Bernh. ex Willd.
Solanum nigrum L. subsp. villosum (Lam.) Hartm., Sv. Norsk Esc.-Fl. 34. 1846.
Type. Based on Solanum villosum Lam.
Solanum nigrum L. subsp. humile (Bernh. ex Willd.) Hartm., Sv. Norsk Exc.-Fl. 34. 1846.
Type. Based on Solanum humile Bernh. ex Willd.
Solanum sinaicum Boiss., Diagn. Pl. Orient. ser. 1, 11: 135. 1849.
Type. Egypt. [Sinai] “Arabia petraea, Wadi Sheikh” [protologue “in fissuris rupium jugi Sianaitici in valle Wadi Scheick”], Mar 1846, *E. Boissier s.n.* (lectotype, designated here: G [G00343377] isolectotypes: DD, K [K000759386]).
Solanum plebeium A.Rich., Tent. Fl. Abyss. 2: 100. 1850.
Type. Ethiopia? [“Abyssinia”], Prov. Chiré [Shire?], *R.Q. Dillon & A. Petit s.n.* (lectotype, designated by Lester 1997, pg. 289: P [P00341983]; isolectotype: P [P00341984]).
Solanum nigrum L. subsp. luteovirescens Kirschl., Fl. Alsace 1: 532. 1852.
Type. Based on Solanum luteovirescens C.C.Gmel. (nom. illeg.)
Solanum nigrum L. subsp. luteum (Mill.) Kirschl., Fl. Alsace 1: 532. 1852.
Type. Based on Solanum luteum Mill.
Solanum nigrum L. subsp. puniceum Kirschl., Fl. Alsace 1: 532. 1852.
Type. Based on Solanum puniceum C.C.Gmel. (nom. illeg.)
Solanum ochroleucum Bastard var. flavum Dunal, Prodr. [A. P. de Candolle] 13(1): 56. 1852.
Type. Greece. Peloponnese: “in vineis agri Argolidis” [Argolis], *J. Sartori 86* (lectotype, designated by Edmonds 1979b, pg. 216: G-DC [G00144471]).
Solanum miniatum Bernh. ex Willd. var. stenopetalum Dunal, Prodr. [A. P. de Candolle] 13(1): 56. 1852.
Type. France. Auvergne-Rhône-Alpes: Isère, “circa Montaud prope Salon”, *J.L. Castagne s.n.* (lectotype, designated here: G-DC [G00144520]; isolectotype: MPU not seen).
Solanum miniatum Bernh. ex Willd. var. villosissimum Dunal, Prodr. [A. P. de Candolle] 13(1): 56. 1852.
Type. France. Provence-Alpes-Côte d’Azur: Var, Miraval, a l’entre de la grotte, 8 May 1807, *A.P. de Candolle s.n*. (lectotype, designated here: G-DC [G00144520]).
Solanum miniatum Bernh. ex Willd. var. subserratum Dunal, Prodr. [A. P. de Candolle] 13(1): 57. 1852.
Type. “Arabie”, *Without collector[No. 26*] (lectotype, designated here: MPU [MPU091575]).
Solanum minutiflorum Dunal, Prodr. [A. P. de Candolle] 13(1): 58. 1852.
Type. France. Hyères, *A. Richard s.n.* (lectotype, designated here: G-DC [G00144587]; isolectotype: P [P00582961]).
Solanum nilagiricum Schltdl., Linnaea 26: 484. 1855.
Type. Cultivated in Germany at Halle [from Nilgiri Hills, India?] (no specimens cited; no specimens located [HAL ? not seen]).
Solanum suffruticosum Schousb. ex Lange, Linnaea 28: 361. 1856, nom. illeg., not Solanum suffruticosum Schousb. ex Willd. (1809)
Type. Cultivated in Denmark at Copenhagen, seeds from “Pontevedra Galleciae Aug 1852 [Galicia, Spain], 1855, *Anon. s.n.* (lectotype, designated here: C [C10019275]).
Solanum nigrum L. var. croceum Neilr., Fl. Nied.-Oesterr.: 535. 1858, nom. illeg. superfl.
Type. Based on Solanum nigrum L. var. villosum L.
Solanum nigrum L. var. miniatum (Bernh. ex Willd.) Neilr., Fl. Nied.-Oesterr. 535. 1858, nom. illeg., not Solanum nigrum L. var. miniatum (Bernh. ex Willd.) Fr. (1823)
Type. Based on Solanum miniatum Bernh. ex Willd.
S. nigrum L. var. luteum Neilr., Fl. Nied.-Oesterr. 535. 1858.
Type. Based on Solanum flavum Kit. ex Schult.
Solanum nigrum L. var. suffruticosum (Schousb. ex Willd.) Moris, Fl. Sardoa 3: 148. 1858–1859.
Type. Based on Solanum suffruticosum Schousb. ex Willd. (cites Solanum suffruticosum Schousb. concept of Dunal)
Solanum nigrum L. var. luteum Döll, Fl. Baden [Döll] 767. 1859, nom. illeg., not Solanum nigrum L. var. luteum Neilr. (1858).
Type. Germany. “in der Carlsruher Gegend bei Dachslanden und Mühlburg, bei Schwetzingen, Mannheim und Dossenheim” (no specimens cited; no original material located at KR).
Solanum canescens Kit. ex Kanitz, Linnaea 32: 440. 1863, nom. illeg., not Solanum canescensBlume (1826).
Type. Hungary. “E Cottu Thurócz. v Barodiensi” [protologue “in Cottu Thurociensi et Borsodiensi”, *P. Kitaibel s.n.* (lectotype, designated here: BP [herb. Kit. fasc. IX: 105, numbered sheet]; isolectotypes: BP [unlabelled sheets in fasc. IX: 105]).
Solanum flavum Kit. ex Kanitz, Linnaea 32: 440. 1863, nom. illeg, not Solanum flavum Kit. ex Schult.(1814)
Type. Hungary. “E Cottu Thurócz. v Barodiensi” [protologue “in Cottu Thurociensi et Borsodiensi”, *P. Kitaibel s.n.* (lectotype, designated here: BP [BP [herb. Kit. fasc. IX: 105, numbered sheet]; isolectotypes: BP [unlabelled sheets in fasc. IX: 105]).
Solanum incanum Kit. ex Kanitz, Linnaea 32: 440. 1863, nom. illeg. superfl., also not S. incanum L. (1753)
Type. Based on Solanum kitaibelii Kit. ex Schult. (cited in synonymy).
Solanum olivaceum Kit. ex Kanitz, Linnaea 32: 440. 1863, nom. illeg. superfl.
Type. Based on Solanum humile Bernh. ex Willd. (cited in synonymy).
Solanum pseudovillosum Schur, Enum. Pl. Transsilv. 477. 1866.
Type. Transylvania [Romania], Hermannstadt [Sibiu] and Kronstadt [Brașov], Aug-Sep, *P.J.F. Schur s.n.* (no herbaria cited; no original material located; LW?, BRNU?).
Solanum nigrum L. subsp. alatum (Moench) Čelak., Prodr. Fl. Böhmen [2:?] 309. 1871.
Type. Based on Solanum alatum Moench
Solanum nigrum L. subsp. villosum (Lam.) Čelak., Prodr. Fl. Böhmen 309. 1871.
Type. Based on Solanum villosum Lam.
Solanum patens Lowe, Man. Fl. Madeira 2: 74. 1872.
Type. Portugal. Madeira: Ribeiria do Sta. Luzia, 1 Jul 1833, *R.T. Lowe 547* [a] (lectotype, designated by Edmonds 2012, pg. 133: BM [BM000083209]; other duplicates of *Lowe 547* with different collecting dates are not type material).
Solanum villosum Lam. var. velutinum Lowe, Man. Fl. Madeira 2: 76. 1872, as “*velutina*”
Type. Portugal. Madeira: Valle [garden], 24 Aug 1831, *R.T. Lowe [722*] (lectotype, designated here: BM [BM000056199]).
Solanum villosum Lam. var. laevigatum Lowe, Man. Fl. Madeira 2: 78. 1872, as “*laevigata*”
Type. Portugal. “Lisbon, court of the Museum [Jan 1865]”, *R.T. Lowe s.n.* (lectotype, designated by Edmonds 2012, pg. 133: BM [BM000847650]; isolectotype: K [K000414066]).
Solanum hildebrandtii A.Braun & C.D.Bouché, Ind. Sem. Hort. Berol. 18. 1874.
Type. Somalia. Saanag: Yafir, Ahlgebirge [Karin Yafir, Ahl Mountains, N Somalia], *J.M. Hildebrandt 865* (neotype, designated by Edmonds 2012, pg. 133 [as lectotype]: BM [BM000907531]; isoneotype: L [L.2879554]).
Solanum nigrum L. var. humile (Bernh. ex Willd.) Boiss., Fl. Orient. 4: 284. 1879.
Type. Based on Solanum humile Bernh. ex Willd.
Solanum nigrum L. var. induratum Boiss., Fl. Orient. 4: 284. 1879, nom. illeg. superfl.
Type. Based on Solanum nigrum L. var. suffruticosum (Schousb. ex Willd.) Moris (cited in synonymy)
Solanum villosum Lam. subsp. ochroleucum (Bastard) Nyman, Consp. Fl. Eur. 3: 526. 1881.
Type. Based on Solanum ochroleucum Bastard
Solanum nigrum L. subsp. miniatum (Bernh. ex Willd.) Arcang., Comp. Fl. Ital. (Arcangeli): 497. 1882.
Type. Based on Solanum miniatum Bernh. ex Willd.
Solanum morella Desv. subsp. luteum (Mill.) Rouy, Fl. France 10: 366. 1908.
Type. Based on Solanum luteum Mill.
Solanum plebeium A.Rich. var. brachysepalum Bitter, Bot. Jahrb. Syst. 49: 564. 1913.
Type. Ethiopia. Oromia: “Gallahochland, Ego”, 2000 m, *H. Ellenbeck 358* (holotype: B, destroyed; no duplicates located).
Solanum plebeium A.Rich. var. subtile Bitter, Bot. Jahrb. Syst. 49: 564. 1913.
Type. Ethiopia. Harari: “Harar, Hararmaja-See”, *H. Ellenbeck 462* (holotype: B, destroyed; no duplicates located).
Solanum nigrum L. forma glabrum Chiov., Res. Sci. Somalia Ital. 128. 1916.
Type. Somalia. Baay: Berdale [Berdaale], 12 Oct 1913, *G. Paoli 935* (holotype: FT [FT003070]).
Solanum nigrum L. forma humile (Bernh. ex Willd.) Lindm., Svensk Fan. Fl. 480. 1918.
Type. Based on Solanum humile Bernh. ex Willd.
Solanum nigrum L. var. sancti-marini Pamp., Boll. Mus. Republ. San Marino 4(3–4): 116. 1920.
Type. San Marino. Sin. loc., *Without collector s.n.* (no specimens cited; possibly at FI?; no original material located).
Solanum nigrum L. var. alatum (Moench) Fiori, Nuov. Fl. Italia 2: 311. 1926.
Type. Based on Solanum alatum Moench
Solanum nigrum L., var. aurantium Maire, Bull. Soc. Sci. Nat. Maroc 8(4–6): 139. 1928.
Type. Morocco. Souss-Massa: Anti-Atlas Mountains “Djebel Inter à Talaïnt” [Djebel Inter à Talguit (=Tafraoute?)], 600 m, 4 Apr 1922. *R. Maire s.n.* (lectotype, designated here: RAB [RAB-44657]; isolectotypes: MPU [MPU001957], P [P00489756]).
Solanum villosum Lam. var. miniatibaccatum Blom, Act. Hort. Gothoburg. 10: 207. 1935.
Type. Sweden. Göteborgstrakten: Göteborg, som ogräs i Botaniska Trädgården, 1935, *C. Blom s.n.* (lectotype, designated here: GB; isolectotypes: LD, S, UPS).
Solanum luteum Mill. subsp. alatum (Moench) Dostál, Květena Č.S.R. 1270. 1949.
Type. Based on Solanum alatum Moench
Solanum transcaucasicum Pojark., Bot. Mater. Gerb. Inst. Komorova Akad. Nauk S.S.S.R. 17: 332. 1955.
Type. Azerbaijan. Lankaran: Talysh, near Tatuni “Talysh bei Tatuni”, *R.F. Hohenacker s.n.* (holotype: LE).
Solanum olgae Pojark., Bot. Mater. Gerb. Inst. Komorova Akad. Nauk S.S.S.R. 17: 333. 1955.
Type. Uzbekistan. Samarkandaya Oblast’, [Tadzkikistan, Hissar, near Kschtut in protologue] Kschtut, on the road to Sherbut, *Fedotov & Gol’bek s.n.* (holotype: LE; isotype: LE).
Solanum zelenetzkii Pojark., Bot. Mater. Gerb. Inst. Komorova Akad. Nauk S.S.S.R. 17: 336. 1955.
Type. Ukraine: Crimea, “Tauria Australis”, Jalta [Yalta in protologue], Oreanda, 21 Aug 1906, *K. Golde s.n.* (holotype: LE).
Solanum woronowii Pojark., Bot. Mater. Gerb. Inst. Komorova Akad. Nauk S.S.S.R. 17: 337. 1955.
Type. Georgia. Abkhazia AR: “ Abchazia [Abkhazia], Gagry”, 30 Oct 1954, *A. Pojarkova 6* (holotype: LE).
Solanum luteum Mill. var. calvum Wessely, Feddes Repert. Spec. Nov. Regni. Veg. 63: 315. 1960.
Type. Cultivated in Germany at Greifswald, seeds from Hort. Bot. Marseille, *I. Wessely 214* (holotype: GFW).
Solanum luteum Mill. var. procerum Wessely, Feddes Repert. Spec. Nov. Regni. Veg. 63: 315. 1960.
Type. Cultivated in Germany at Greifswald, seeds from München Botanical Garden, 1957, *I. Wessely 320* (holotype: GFW).
Solanum luteum Mill. forma miniatibaccatum (Blom) Wessely, Feddes Repert. Spec. Nov. Regni. Veg. 63: 315. 1960.
Type. Based on Solanum villosum Lam. var. miniatibaccatum Blom
Solanum purpureilineatum Sabnis & Bhatt, Bull. Bot. Surv. India 12(1–4) 258. 1972 [“1970”].
Type. India. Gujarat: Baroda District, Baroda, L.V. Palace compound, 2 Oct 1960, *S.D. Sabnis 2762, 2763* (holotype: BARO [two sheet holotype, single gathering).
Solanum carmanicum Schönb.-Tem., Fl. Iranica 100: 8. 1972.
Type. Iran. Kerman: Bam, ca. 900 m, 7 May 1948, *K.H. Rechinger & F. Rechinger 3630* (holotype: W [1958-0003176]; isotype: E [E00251353], G [G00343074], US [00992058, acc. # 2316288]).
Solanum villosum Mill. subsp. alatum (Moench) Edmonds, Bot. J. Linn. Soc. 75: 171. 1977.
Type. Based on Solanum alatum Moench
Solanum nigrum L. var. humile (Bernh. ex Willd.) C.Y. Wu & S.C.Huang, Acta Phytotax. Sin. 16(2): 72. 1978, nom. illeg., not Solanum nigrum L. var. humile (Bernh. ex Willd.) Boiss. (1879).
Type. Based on Solanum humile Bernh. ex Willd.
Solanum villosum Mill. subsp. puniceum (Kirschl.) Edmonds, Bot. J. Linn. Soc. 78: 215. 1979.
Type. Based on Solanum nigrum L. subsp. puniceum Kirschl.
Solanum villosum Mill. subsp. miniatum (Bernh. ex Willd.) Edmonds, Bot. J. Linn. Soc. 89: 166. 1984.
Type. Based on Solanum miniatum Bernh. ex Willd.
Solanum villosum Mill. subsp. alatum (Moench) Dostál, Folia Mus. Rerum Nat. Bohemiae Occid., Bot. 21: 11. 1984, nom. illeg., not Solanum villosum Mill. subsp. alatum (Moench) Edmonds (1977)
Type. Based on Solanum alatum Moench
Solanum luteum Mill. var. miniatibaccatum (Blom) Hyl., Uppsala Univ. Årsskr. 7: 279. 1945.
Type. Based on Solanum villosum Lam. var. miniatibaccatum Blom

#### Type.

Cultivated in Chelsea Physic Garden from “Barbadoes where it is supposed to grow naturally”, *Herb. Miller s.n.* (lectotype, designated by Henderson 1974, pg. 54: BM [BM000942575]).

#### Description.

Annual to short lived erect or weakly scrambling perennial herbs up to 0.5 m tall, subwoody at base and much branched. Stems spreading to decumbent, terete to ridged, green to purple, not hollow; new growth densely pubescent with simple, spreading, uniseriate, translucent, eglandular and/or glandular trichomes, these 3–10-celled, 0.2–2.0 mm long; older stems glabrescent. Sympodial units difoliate, the leaves not geminate. Leaves simple, 1.5–5.0(-10.0) cm long, 0.7–2.5(-6.5) cm wide, broadly to narrowly ovate to elliptic, membranous, green on both sides, without smell or somewhat pleasant smelling; adaxial surfaces sparsely to densely pubescent with spreading, simple, uniseriate eglandular and/or glandular trichomes like those on stem evenly along veins and lamina; abaxial surfaces more densely pubescent on veins and lamina; major veins 4–6 pairs; base acute to truncate, short-attenuate, often asymmetric; margins sinuate-dentate to rarely entire; apex acute; petioles 0.5–3.0(-4.5) cm long, pubescent with simple uniseriate glandular and/or eglandular trichomes like those on stems. Inflorescences 0.4–2.0 cm long, internodal, simple, the flowers spaced along the rhachis, with (2-)3–5(-8) flowers clustered at the tip or more commonly spaced along the rhachis, pubescent with spreading simple glandular and/or eglandular uniseriate trichomes like those of the stems; peduncle 0.4–1.5 cm long, straight; pedicels 4–7 mm long, 0.2–0.3 mm in diameter at the base and 0.4–0.5 mm at apex, spreading, articulated at the base; pedicel scars spaced 0–1.0 mm apart. Buds globose, the corolla exserted ca. 1/5 from the calyx before anthesis. Flowers 5-merous, all perfect. Calyx tube 1.2–1.5 mm long, conical, the lobes 0.8–1.5 mm long, 0.5–0.8 mm wide, elliptic to triangular with obtuse thickened apices and paler (almost scarious) sinuses, pubescent with spreading simple uniseriate eglandular and/or glandular trichomes like those on stem. Corolla 8–15(-20) mm in diameter, white with a yellow-green central portion near the base and occasionally with purple stripes along lobe midveins abaxially, stellate, lobed 1/2 way to the base, the lobes 2.5–4.5 mm long, 2.0–3.5 mm wide, strongly reflexed at anthesis, later spreading, densely papillate-pubescent abaxially with simple uniseriate eglandular trichomes. Stamens equal; filament tube minute, pubescent with spreading uniseriate simple eglandular trichomes adaxially; free portion of the filaments 1.0–1.3 mm long, pubescent like the tube; anthers 1.8–2.2(-2.4) mm long, 0.5–0.7 mm wide, ellipsoid, yellow, poricidal at the tips, the pores lengthening to slits with age and drying. Ovary globose, glabrous; style 2.8–3.5(-4.0) mm long, densely pubescent with 2–3-celled simple uniseriate trichomes in the lower half, exserted 0–1 mm beyond anther cone; stigma capitate, the surface minutely papillate, green in live plants. Fruit an ellipsoid berry, usually somewhat longer than broad, 8.5–10 mm long, 8.0–9.5 mm wide, (red-)orange to yellow at maturity, the pericarp thin, shiny and translucent; fruiting pedicels 8–14 mm long, 0.4–0.5 mm in diameter at the base, 0.7–1.5 mm at apex, strongly reflexed, becoming woody, spaced 1.0–2.0 mm apart not falling with the fruit, remaining on the plant and always persistent on older inflorescences; fruiting calyx not accrescent, the tube 1.0–1.5 mm long, the lobes 2.0–3.0 mm long, strongly reflexed in fruit. Seeds 20–40 per berry, 1.8–2.2 mm long, 1.5–1.7 mm wide, flattened and tear-drop shaped with a subapical hilum, brown, the surfaces minutely pitted, the testal cells pentagonal in outline. Stone cells absent, but occasionally 1–2 found in North African and Arabian material, ca. 0.5 mm in diameter. Chromosome number: *2n*=4x=48 (Jørgensen 1928; Tokunaga 1933; Stebbins and Paddock 1949; Soria and Heiser 1961 [as *S. roxburghii* and *S. villosum*]; Heiser et al. 1965 [as *S. roxburghii*]; Gerasimenko and Reznikova 1968; Venkateswarlu and Rao 1972; Henderson 1974; Tandon 1974; Randell and Symon 1976; Edmonds 1977, 1982, 1983, 1984a; Ganapathi and Rao 1986a; Bukenya 1996; Ojiewo et al. 2006; Olet et al. 2015).

**Figure 57.**
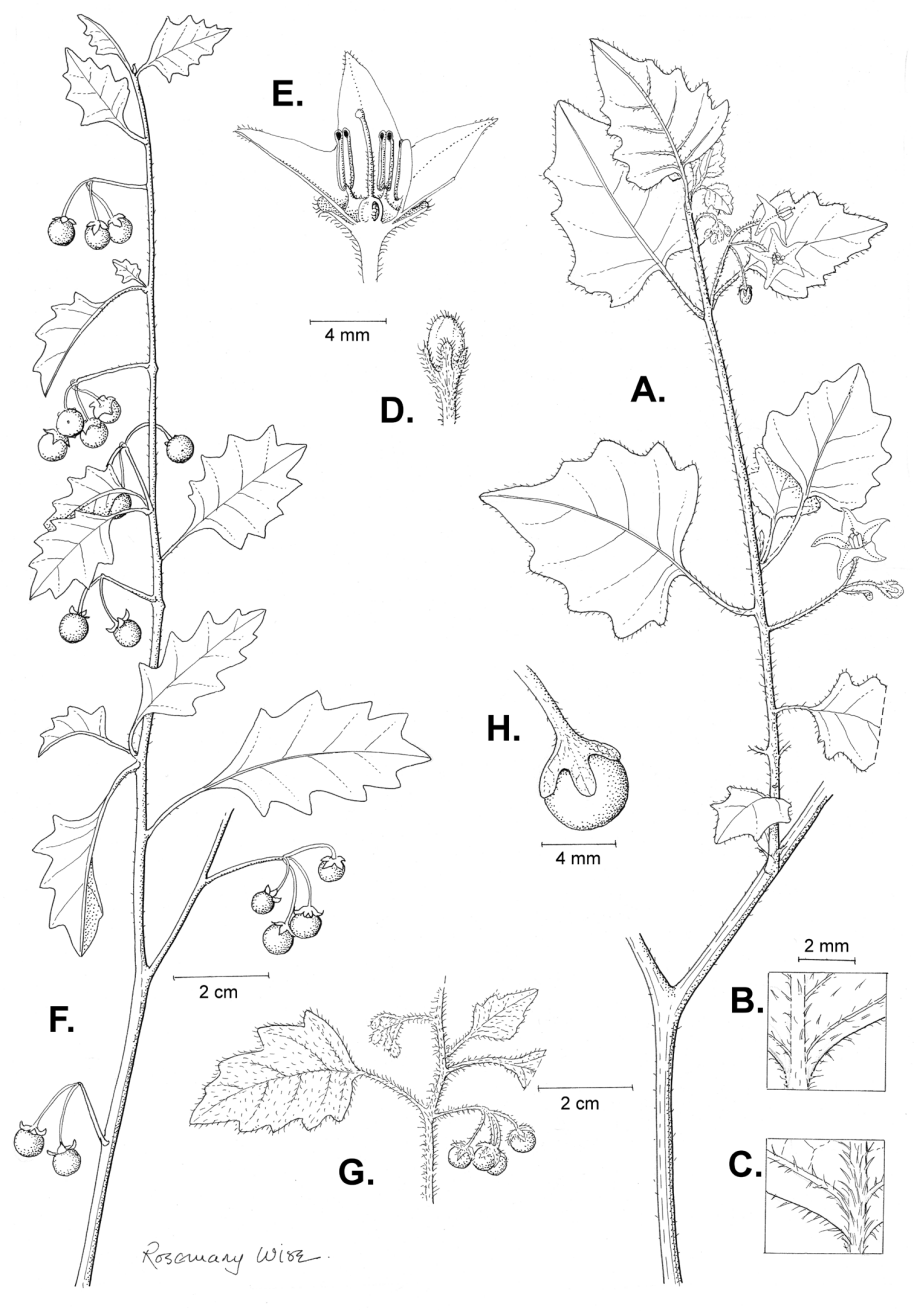
*Solanum villosum* **A** Flowering habit **B** Detail of adaxial leaf surface **C** Detail of abaxial leaf surface **D** Bud **E** Dissected flower **F** Fruiting habit **G** Fruiting habit with dense indumentum **H** Fruit (**A–E** *Wood 2193*; **F** *Popov GP/72/31*; **G** *Wood Y/74/265*). Drawing by R. Wise.

**Figure 58.**
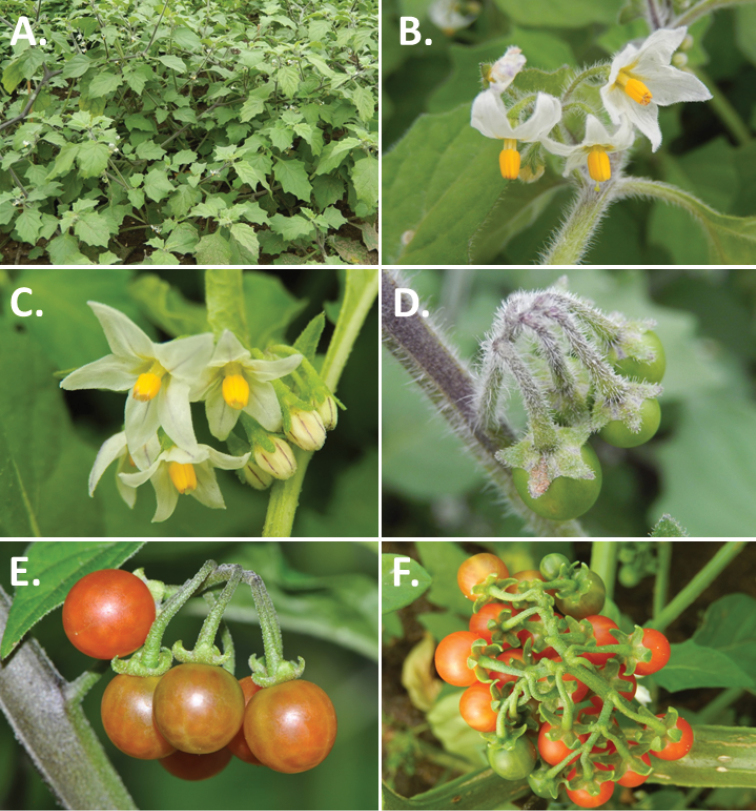
*Solanum villosum* **A** Habit **B** Inflorescence in hairy individual **C** Flowers and buds **D** Immature infructescence **E** Fully mature fruits **F** Fully mature fruits showing reflexed calyx lobes (**A** Nijmegen acc. 884750135; **B** Nijmegen acc. 914750047; **C** Nijmegen acc. A34750061, **D** Nijmegen acc. A34750043; **E** Nijmegen acc. A14750048; **F** Nijmegen acc. A34750038). Photos by T. Särkinen and G. van der Weerden.

#### Distribution

**Figure 59.**
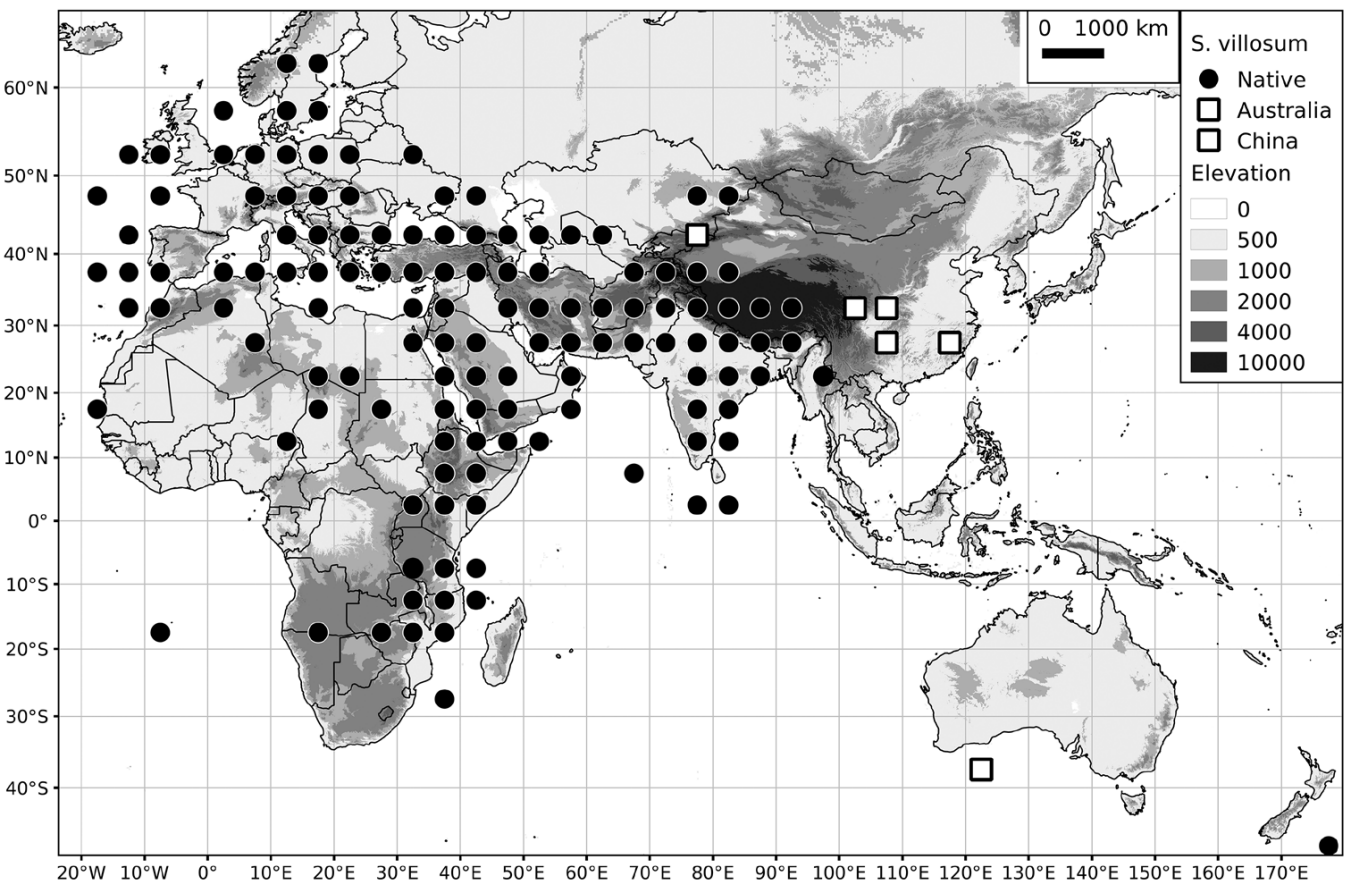
Distribution of *Solanum villosum* in the native range (black circles) and its non-native range (white squares) in the Old World.

(Figure 59). Native to the northern Hemisphere, from Europe, especially around the Mediterranean basin, Arabian Peninsula to dry sub-tropical Asia and eastern Africa (where it is cultivated for its fruit); introduced globally. The native distribution of *S. villosum* is restricted to the Old World, but cultivated and/or introduced populations that never seem to persist are occasionally found in North America and the Caribbean (Stebbins and Paddock 1949).

#### Ecology.

Grows in open and disturbed areas in a wide variety of habitat types, including in cities; between sea level and 3,000 (-3,600) m elevation.

#### Common names.

Austria: Rotbeeren-Nachtschatten, Gelbbeeren-Nachtschatten, Gelbrotbeeren-Nachtschatten (Fischer et al. 2005), Flügel-Nachtschatten; Chad: abriki, waatenni, waha tinni; China: hong guo long kui (Zhang et al. 1994); Denmark: højrød natskygge (Hartvig 2015); Egypt: ‘enneyb, aneb el dib/aneb el dik; ilshsmdhut, ribai hauwei; Ethiopia: auwt; Greece: styphnos; India: gammie, kambe, tutguna (Kalahandia language); Iraq: anab dheeb; Italy: morella rossa (Pignatti 1982); Kenya: isoiyot/isoyot, itulu, ksoya, kusuyo, manangwe, managu, munavu, ndolu, ol’ momoit/ol’ momoye, ormomoi; New Zealand: red-berried nightshade (Webb et al. 1988); Nigeria: gautan kaji; Oman: mjaj; Pakistan: tol angur; Palestine: inab ed dib; Poland: psianka niska, psianka kosmata, psianka skrzydlata (Pawłowskiego 1963); Portugal: erva de Santa Maria; Saudi Arabia: aah’al, khur ma; Slovenia: lulok žltý, lulok krídlatý (Bertová and Goliašová 1993); Somalia: biyeche El, karire; Spain: modalicou, yerba mora; Sweden: Gul nattskatta, röd nattskatta (Mossberg et al. 2003); Tanzania: emunauui, en’songwe, manakk, mnafu/mnavu, osuga, soku; Uganda: eswiga, ocuga; United Arab Emirates: black nightshade; United Kingdom: red nightshade (Stace 2010).

#### Uses.

Leaves used as spinach (usually boiled, often in milk) and as a pot-herb in Europe, Africa and the Middle East; berries eaten raw (especially by children, see Uses above) and cooked (Pojarkova 1955a, b). Medicinal uses include treatment of eye conditions and swellings (see Yang and Ojiewo 2013).

#### Preliminary conservation status

(IUCN 2016). *Solanum villosum* is a very widespread species and can be assigned a preliminary status of LC (Least Concern).

#### Discussion.

This common European species was long known as *S. luteum* (e.g. Hawkes and Edmonds 1972) and this name was in wide use in botanical gardens as the name for this red-fruited taxon. The name *S. villosum* Lam. was recognised as a synonym for *S. luteum.* The name *S. villosum* Mill. was not in use in Europe until resurrected by Edmonds (1979b), but had been used by Stebbins and Paddock (1949) in their treatment of the morelloids of California. Edmonds (1979b), following what is now Article 11.5 of the *Code* (McNeill et al. 2012), changed the name to *S. villosum* Mill. citing the explicit synonymisation of *S. luteum* with *S. villosum* Mill. by Stebbins and Paddock (1949). Dostál (1949), only a month later than Stebbins and Paddock (1949), synonymised *S. villosum* with *S. luteum*, but unfortunately used the name in the Lamarckian sense, although in a letter to Edmonds (quoted in Edmonds 1979b) equated *S. villosum* Mill. and *S. villosum* Lam. Many specimens in European herbaria are still identified as *S. luteum* or as *S. alatum* (see Knapp et al. 2017 for discussion of *S. alatum*).

Variation in pubescence and stem characteristics within *S. villosum* has been recognised at many ranks from subspecies, varieties and different species (Hawkes and Edmonds 1972; Edmonds 1979b, 1984b; Henderson 1974; Symon 1981; Edmonds and Chweya 1997; Olet 2004; Dehmer and Hammer 2004). The various synonyms and infraspecific ranks reflect the wide morphological variation observed within the species, where indumentum can range from nearly absent (the plants glabrous) to densely pubescent with eglandular and/or eglandular trichomes. The recognition of the glandular variants at infraspecific rank is not supported by AFLP data (Manoko 2007; Olet et al. 2011). In fact, studies have shown that pubescence varies in individuals according to age, where seedlings can exhibit glandular hairs which are lost during growth (Henderson 1974; Seithe 1979; Edmonds 1982). Here we have an inclusive, broad concept of *S. villosum* that includes this variability but does not recognise it at any rank.

*Solanum villosum* can be easily recognised from similar species by its orange-red berries (ranging from yellow to bright red) that are slightly elongate and ellipsoid. The only other species present in the Old World with pale yellow fruits is *S. palitans*, but this taxon differs in being a prostrate plant with thinly membranous 3-lobed leaves and is easy to tell apart. The rest of the morelloids in the Old World have rounded or subglobose black or green berries. *Solanum villosum* is much more common in southern Europe than is *S. nigrum*, while *S. nigrum* becomes more common towards northern and central Europe. These latter two species are difficult to tell apart based on herbarium material alone, especially when mature fruit colour is not available. Calyx lobes of *S. villosum* are generally longer than those of *S. nigrum* (relative to width), strongly reflexed at fruit maturity and the sinus is rounded (as opposed to acute in *S. nigrum*) and, in flower, the calyx tube has pale patches that look like small windows or white/translucent areas below the sinus itself. This is particularly easy to see in herbarium specimens. The leaves of *S. villosum* are similar to those of *S. retroflexum* in being usually more or less rhomboid with dentate margins, but leaves are more narrowly rhomboid than those of *S. retroflexum* (i.e. narrower relative to length). Specimens from the southern part of the range of *S. villosum* and the northern range of *S. retroflexum* (e.g. Malawi and Mozambique) can be difficult to distinguish in the absence of information on fruit colour (*S. retroflexum* has dull purple-black berries, also with strongly reflexed calyx lobes in fruit).

*Solanum villosum* has generally distinctly longer filaments relative to anther length than other species (except *S. opacum* and *S. retroflexum*, see descriptions of these species), although large variation can be observed within and between individuals especially in herbarium specimens. We have observed in the field that filaments elongate during flower maturation, where younger flowers in the same inflorescence can be seen with shorter filaments and older flowers with fully extended and distinctly longer filaments (SK photo 101532). *Solanum villosum* has a large capitate stigma that does not generally extend much beyond the anthers, in contrast to *S. tarderemotum* with which it often grows in sympatry in Africa, where the style is long-exserted and often bent and the stigma is smaller.

A set of herbarium and seed bank accessions of *S. villosum* from Kenya have narrower leaves with entire margins and larger, branched inflorescences with fruits in which calyx lobes do not recurve as strongly as in more typical populations, but that lack stone cells in the berries. These accessions are somewhat morphologically intermediate between *S. villosum* and *S. tarderemotum* and could present man-made or natural introgression between the two species (see discussion of *S. tarderemotum*).

The origin of the tetraploid *S. villosum* has been the focus of previous crossing and cytological studies (e.g. Edmonds 1979a). Molecular (Poczai and Hyvönen 2011), experimental breeding (Tandon and Rao 1966a, 1966b; Venkateswarlu and Rao 1972; Rao 1978) and chromosome binding data (Bhiravamurty and Rethy 1984; Sultana and Alam 2007) suggest that the species is an ancient autopolyploid with regular bivalent formation derived from *S. americanum*. Edmonds (1979a) suggested that *S. villosum* is derived from the diploid parents *S. americanum* and *S. sarrachoides*, because these two diploids are interfertile. The hypothesis in which a native of the New World, *S. sarrachoides*, would have given rise to an Old World endemic is not particularly convincing, but current distributions of progenitor taxa do not necessarily have to overlap (e.g. *Nicotiana tabacum* L., see Chase et al. 2003). The autopolyploid origin of *S. villosum* and its contribution to the autoalloploid *S. nigrum* derived from *S. villosum* and *S. americanum*, is more likely, considering their sympatry across the entire range of *S. villosum*. Furthermore, *S. sarrachoides* is reproductively isolated from all other diploids studied thus far (Edmonds 1977), making it less likely that the species has been involved in allopolyploidy. A possible diploid progenitor could be *S. chenopodioides*, however, as suggested by Edmonds (1979a), with which *S. nigrum* and *S. villosum* share some of their distributions. It is much more likely, however, based on the current molecular, morphological, artificial crossing and cytological studies that *S. nigrum* is an autoallopolyploid derived from a genome duplication of the tetraploid *S. villosum* that comprises genomes of two distinct populations of *S. americanum*. Morphological characters support this, in that *S. americanum* and *S. villosum* both drop their fruits without pedicels.

Edmonds (1979b) superfluously lectotypified S. nigrum var. villosum with a specimen in the Linnean herbarium (LINN 248.19), apparently not realising the name had already been lectotypified completely in accordance with the protologue by Henderson (1974), using the illustration from *Hortus Elthamensis* (Dillenius 1732; see Jarvis 2007).

Although Miller (1768) used Linnaean names extensively in his *Gardener’s dictionary*, it is unlikely that he had seen Linnaeus’ (1767) 12^th^ edition of *Systema naturae* where *S. rubrum* was first published (J.E. Dandy pers. comm. to J.M. Edmonds, quoted in Edmonds 1979b), so *S. rubrum* Mill. is considered a new name, rather than a citation of *S. rubrum* L. (nom. utique rej. prop. see Doubtful names).

Edmonds (2007) typified the names in morelloid solanums coined by J.F. Gmelin (1791) for plants collected by Pehr Forsskål, but for some reason did not designate a lectotype for *S. aegyptiacum*. We here select the specimen in the Forsskål herbarium (C10013600) that corresponds to Gmelin’s description and is labelled “aegyptiacum a) fructu rubro”. Edmonds (2007) considered “*S. aegyptiacum* Forssk.” to be not validly published because it lacked a description or diagnosis for the species, although she included one of its varieties in the synonymy of *S. memphiticum* (as “*S. aegypticum* b) *fructu nigro; foliis integris, villosissimus*”). We agree that Forsskål did not validly publish the name *S. aegyptiacum*, but it was validly published by J.F. Gmelin.

Lamarck (1794) coined his name *S. villosum* without reference to Miller and, because the more commonly used name for this species was *S. luteum* until Edmonds (1979b) changed the name (see above), many of the new combinations and infraspecific taxa attributed *S. villosum* to Lamarck rather than Miller.

The name *S. alatum* Moench has been commonly used for densely pubescent eglandular populations of *S. villosum* (Edmonds 1979b). There was considerable confusion over the identity of the type of *S. alatum*; Edmonds (1979b) typified it with an element she, in the same publication, excluded from her concept of *S. alatum*. The name has been proposed for conservation with a conserved type (Knapp et al. 2017) to overcome this disruptive typification; the conserved type proposed by Knapp et al. (2017) is cited here.

*Solanum suffruticosum* apparently was a “herbarium” name of Peter Schousboe, a Danish botanist who worked in Tangiers in the early part of the 19^th^ century. The epithet was used and attributed to Schousboe by Willdenow (1809) who referred to plants grown in Berlin that originated from northern Africa (seeds sent by Schousboe?); we have selected the sheet in the Willdenow herbarium with annotations of the name “Solanum suffruticosum” and “Hort. bot. Berol. W.” as the lectotype for this name. Almost 50 years later, Johan Lange, working in Copenhagen, also validated *S. suffruticosum*, but with a “?” as to the name (“d) Solanum suffruticosum Schousb.?” and citing a different collection from Spain (C10019275), that we here designate as the lectotype. Lange’s name is a later homonym and illegitimate.

Dunal (1813) cited a single element for his *S. incertum*, an illustration in Rheede von Draakestein’s *Hortus Malabaricus* (1689) with the common name “Nelen-Tsundja”; we here designate this plate as the lectotype for *S. incertum*. The accompanying description states the berries are orange.

*Solanum kitaibelii* and *S. flavum* are names coined by Schultes (1814) based on specimens of Pál Kitaibel; *S. kitaibelii* as a replacement for Kitaibel’s “Solanum incanum” that Schultes realised was pre-occupied (“Hr. Prof. Kitaibel nannte diese Art *S. incanum*; da aber Flora peruviana schon ein *S. incanum* hat, so nannte ich es nach meinem hochverdienten Freunde, der es zuerts in Ungern fand” – Professor Kitaibel named this species *S. incanum*, but there is already one in the Flora Peruviana, so I name it after my esteemed friend, who found it first in Hungary). Edmonds (1979b) cited specimens as holotypes for these names, effectively lectotypifying them. She cited a specimen in the Willdenow herbarium (B-W04421010) as the “holotype” of *S. flavum*; it is more likely however, that Schultes used specimens now held in M (Schultes’s herbarium at Landshuth, where he worked at the time of description of these two taxa and this is now part of M). The lectotype for *S. kitaibelii* has the name in Schultes’s hand (M-0166013), but the isolectotype for *S. flavum* does not (M-0164200).

We have designated a specimen with the exact locality as the lectotype of *S. ochroleucum*; the G-DC duplicate is better preserved (G00144474).

Döll’s various infraspecific taxa described in his *Rheinische Flora* (1843) as “Formen” (here taken as the rank of forma) are mostly new combinations of previously published epithets, but his forma angulosum is based on several cited localities in Germany. We have not been able to locate specimens for these collections and defer typification until more in-depth searches are done. It may be that these names are based on field observations and not herbarium material.

*Solanum sinaicum* was described by Boissier (1849) using a collection of his own from Arabia; he did not cite a herbarium, so we are lectotypifying this name with a specimen in G (G00343377). Although the protologue mentions “baccis nigricantibus”, the plants are unmistakably *S. villosum*; we suspect the fruit colour was cited from the dry specimen.

The spelling of Richard’s (1850) name *S. plebeium* has sometimes been corrected to “plebejum” (e.g. Edmonds 2012). This is not necessary and incorrect, “plebeium is the nominative neuter singular of “plebeius”, meaning “commoner” or “of the common people”.

Dunal (1852) described three varieties of S. miniatum; var. villosissimum and var. stenopetalum based on European specimens and var. subserratum based on a specimen from “Arabia”. Edmonds (1979b) discussed the identities of these varieties, but did not typify them. Dunal cited the same collection *Castagne s.n.* from “Montaud prope Salon” for both var. stenopetalum and var. villosissimum; we have lectotypifed var. villosissimum with the other specimen cited that does not overlap with that used for var. stenopetalum (G00144520). Material in Dunal’s herbarium at MPU (MPU091757), labelled as “Arabie” and cited by Edmonds (1979b), is labelled as var. villosissimum and numbered “No. 26” and is undoubtedly original material of var. subserratum. This sheet is here designated the lectotype for this name, despite its having an annotation label with the incorrect infraspecific name. In describing *S. minutiflorum*, he (Dunal 1852) cited two collections “Pannonia circa Bannat – (Terenbinki l.c.)” and a specimen from Richard. We have chosen the latter as the lectotype because it has two duplicates; neither Edmonds (1979b) nor we were able to locate the other syntype.

The names coined by the Hungarian botanist Pál Kitaibel were published posthumously by Kanitz (1863) and are largely illegitimate (some were used in earlier publications by Schultes, see above). *Solanum flavum* and *S. canescens* were published as alternative names “Solanum flavum vel canescens” and therefore have the same type; we have selected the sheet in the Kitaibel herbarium at BP with the locality on the label matching that in the protologue (“E Cottu Thurócz. v. Borsodiensi”, BP [Herb. Kit. fasc. IX: 105]).

In his *Manual flora of Madeira*, (Lowe 1872) coined several names we here consider synonyms of *S. villosum*, both at the specific (*S. patens*) and infraspecific level (S. villosum var. velutinum, S. villosum var. laevigatum). Edmonds (1979b: 215) lectotypified var. velutinum citing the type as the “same as the holotype of *Solanum villosum* Lam.”. Lamarck (1794) was cited along with many other works, including Linnaeus’ (1753) S. nigrum var. villosum, so arguably var. velutinum is an illegitimate superfluous name because S. nigrum var. villosum was cited in synonymy and the epithet “villosum” should have been taken up. We here re-lectotypify var. velutinum with a specimen cited by Lowe and represented in BM (BM000056199). Edmonds (1979b) lectotypified *S. patens* with “Lowe 547 BM!”, but we exclude all but the sheet labelled by J.M. Edmonds as “lectotype” (BM000083209) as type material; other specimens at BM labelled “Lowe 547” have different collecting dates and localities.

Edmonds (2012) designated wild-collected material as the lectotype of *S. hildebrandtii* stating that, in Bitter’s re-description of this species in *Solana africana* (1913a: 467–468), he had designated a holotype. However, Braun and Bouché clearly stated that the material they used was from cultivation and Bitter (1913a) did not re-typify *S. hildebrandtii* with wild-collected material, he merely cited it as something he had seen. This typification is corrected here to neotype from lectotype.

Two un-numbered collections were cited in the protologue of S. nigrum var. aurantium (Maire 1928); *Maire s.n.* (coll. 4 Apr 1922) and *Maire s.n.* (coll. 26 Mar 1923). We have selected the duplicate in Morocco (RAB-44657) of the former as the lectotype because it has numerous duplicates. We have only been able to find a single duplicate (MPU001957) of the collection made in March 1923.

Blom (1935) cited two collections (*H. Christoffersson s.n.*, coll. 1930; *C. Blom s.n.*, coll. 1935) and several herbaria in his description of S. villosum var. miniatibaccatum; we have selected the GB duplicate of his un-numbered collection from 1935 as the lectotype.

The names coined by Pojarkova (1955b) and here regarded as synonyms of *S. villosum* were first published in Russian only (Pojarkova 1955a), but later in the same year validated with Latin descriptions and type specimen designations.

*Solanum purpureilineatum* (Sabnis and Bhatt 1972) was described using two specimens with two different collecting numbers from a single gathering. Rather than lectotypifying this name, we are treating this as a two sheet holotype (see McNeill et al. 2012: 14–15, Art. 8.3 Ex. 4).

#### Selected specimens examined.

A total of 1,287 specimens were examined from 86 countries during the study across Africa, Asia, Australia, Eurasia and the Pacific. Specimens from the entire range (including adventives) were studied in order to understand the full range of morphology within the species. All specimens examined from the Old World can be seen in Appendix 2 (csv format) and Appendix 3 (traditional Specimens Examined list in pdf format).

##### Doubtful names

*Solanum cestrifolium* Jacq., mentioned by Weinmann (1824), as well as by Sprengel (1825), but no original publication of this epithet by Jacquin has been found (see discussion under *S. nigrum*).

*Solanum foetidum* Rottb., Acta Lit. Univ. Hafn. 1: 287. 1778. Type. “Surinam”, *C.F. Rottbøll s.n.* (no original material located); this name has sometimes been referred to the morelloids, but is likely not to even be a *Solanum*, it might refer to a plant of *Datura*.

*Solanum foliosum* Link, Phys. Beschr. Canar. Ins. [Buch] 144. 1825. Type. Spain. Islas Canarias: Tenerife, Puerto Orotava, *L.C. Buch s.n.* (no specimens cited; original material seen by Link in B, destroyed). Link’s description suggests this plant is like, not the same as, *S. nigrum*, but then goes on to describe a plant with pinnatifid leaves and flowers larger than those of *S. nigrum*. Possibly *S. nava* Webb & Berthel. (1845) or perhaps the cultivated *S. laxum* Spreng. (1824)

Solanum nigrum L. var. aspergilliflorum Sendtn., Fl. Bras. (Martius) 10: 16. 1846. Type. Sin. loc. [Brazil?] (no specimens or collectors cited). Might be *S. chenopodioides* Lam. based on Sendtner’s notes “*S. chenopodioides* Lam.?” beneath the description in the original publication, but the description mentions stellate hairs on the leaf undersides, a character that does not occur in the Morelloid clade.

Solanum nigrum L. var. humile Macloskie, Rep. Princeton Univ. Exped. Patagonia 8: 707. 1905. The “description” consists solely of a reference to “*S. humile* Salisb.” an illegitimate superfluous name citing *S. nigrum* L. in synonymy. *Solanum nigrum* does not occur in Patagonia, so this name cannot be considered homotypic; identity uncertain, but certainly a species native to the New World.

Solanum nigrum L. var. stylosum Witasek ex Reiche, Anales Univ. Chile 124: 456. 1909. Type. Chile. “Provincias Centrales” (no specimens cited; no original material found). The description is very short and no specimens annotated as this from Chile have been found at W or WU; it could be *S. alphonsei* Dunal of the Dulcamaroid clade; the description of the peduncle as twice as long as the branches is indicative of that species, rather than a morelloid.

*Solanum rubrum* L., Syst. Nat. ed. 12: 173. 1767, nom utique rej. prop. No original type material traced, Edmonds (1979b) treated this as a *nomen dubium* and it has been proposed for rejection (Knapp et al. 2017).

*Solanum uliginosum* Blume, Bijdr. Fl. Ned. Ind. 13: 695. 1826. No specimens or localities cited, not treated by Nees van Esenbeck (1837), description merely copied from Blume (1826) by Dunal (1852) could be *S. americanum*, *S. nigrum* or *S. scabrum.* We have found no specimens at L that could be unequivocally associated with this name; perhaps material is held in Bogor.

Solanum nigrum L. forma uliginosum (Blume) Miq., Fl. Ned. Ind. 2: 637. 1856.

Type. Based on *Solanum uliginosum* Blume

##### Names (designations) not validly published

Designations are listed in alphabetic order; Code Articles are numbered as in McNeill et al. (2012) - *Melbourne Code*.

*Solanum* series *Luteum* Pojark., Flora SSSR 22: 32. 1955, as “*Lutea*”, not validly published; in Russian, no diagnosis or description in Latin (Art. 39.1). [only included species *S. luteum* Mill. = *S. villosum* Mill.]

*Solanum aegyptiacum* Forssk., Fl. Aegypt.-Arab. 46. 1775, nomen nudum (see Edmonds 2007) = *S. memphiticum* J.F.Gmel. and *S. villosum* Mill.

*Solanum aegyptiacum* Forssk., var. b) *Fructu nigro; foliis integris villossissimus*, Fl. Aegypt.-Arab. 46. 1775, polynomial, not validly published (see Edmonds 2007). = *S. memphiticum* J.F.Gmel.

*Solanum amaranthifolium* Gillies ex Rusby, Bull. Torrey Bot. Club 26: 152. 1899, nomen nudum; based on a Gillies manuscript name at Kew; two specimens by Gillies (K001166701, K001166704) are annotated “S. amaranthifolium Gill.” in Gillies’ hand = *S. chenopodioides* Lam.

*Solanum arenarium* Schur, Enum. Pl. Transsilv. 477. 1866, pro syn. *Solanum humile* Bernh. ex Willd. = *S. villosum* Mill.

*Solanum asperum* Hornem. ex Walp., Repert. Bot. Syst. (Walpers) 3: 49. 1844, pro syn. *Solanum rumphii* Dunal = *S. americanum* Mill.

*Solanum atriplicifolium* Desp. ex Dunal, Solan. Syn. 12. 1816, herbarium name pro syn. *Solanum nigrum* L.= *S. nigrum L*.

*Solanum atriplicifolium* Desp. ex Dunal, Prodr. [A. P. de Candolle] 13(1): 50. 1852, pro. syn. Solanum nigrum var atriplicifolium Dunal = *S. nigrum* L.

*Solanum besserianum* Weinm., Elench. Hort. Pawlowsk.: 100. 1824, nomen nudum, no description or diagnosis; Cult. “hort. Pawl.”, said to be “S. cestrifolium Jacq.?” [no such published name found; validated by Sprengel (1825), see also Doubtful names] = *S. nigrum* L.

*Solanum cestrifolium* Gussone, Ind. Sem. Hort. Boccadifalco 11. 1825, nomen nudum = *S. nigrum* L.

*Solanum chlorocarpum* Schur, Enum. Pl. Transsilv. 478. 1866, pro. syn. Solanum nigrum L. var. chlorocarpum Koch. = *S. nigrum* L.

Solanum chousboe var. merrillianum (T.N.Liou) C.Y.Wu & S.C.Huang. This citation from *Flora of China* (Zhang et al. 1994) that also appears on Tropicos (accessed 12 August 2017) is a misprint and confounding of the attribution to Schousboe (Schousboe ex Willd.) with a specific epithet.

*Solanum cuneifolium* Hort. Avens ex Dunal, Prodr. [A. P. de Candolle] 13(1): 53. 1852, pro syn. *Solanum nigrum* L. = *S. nigrum* L.

*Solanum decurrens* Wall. ex Dunal, Prodr. [A. P. de Candolle] 13(1): 50. 1852, pro syn. *Solanum rhinozerothis* Blume = *S. nigrum* Mill.

*Solanum deppei* Dunal, Prodr. [A. P. de Candolle] 13(1): 52. 1852, pro syn. Solanum pterocaulon Dunal var. deppei Dunal = *Solanum scabrum* Mill.

*Solanum gracile* Otto ex Baxter, Hort. Brit. [Loudon] 2, Suppl.: 639 1850, nomen nudum; also later homonym of *S. gracile* Sendtn. (1846) = identity uncertain

*Solanum heterogonum* Dunal, Prodr. [A. P. de Candolle] 13(1): 52. 1852, pro syn. Solanum pterocaulon var. heterogonum Dunal = *S. scabrum* Mill.

*Solanum judaicum* Hack. ex Dunal, Prodr. [A. P. de Candolle] 13(1): 53. 1852, pro syn. *Solanum tauschii* Opiz. = *S. nigrum* L.

*Solanum kraussianum* Buchinger ex Krauss, Flora 27: 831. 1844, pro syn. of polynomial *Solanum nigrum* L. var. *fol. dentato-lobatis puberulis* and not intended as a new name, refers to *J.F. Drège 7864a* = *S. retroflexum* Dunal

*Solanum muricatum* Bertero ex Dunal, Prodr. [A. P. de Candolle] 13(1): 150. 1852, pro syn. *Solanum rancaguense* Dunal = *S. furcatum* Dunal

Solanum nigrum L. var. aegyptiacum Lam. ex C.C.Gmel., Hort. Carlsruh. 253 1811, nomen nudum, no description or diagnosis; the epithet “aegyptiacum” was not used by Lamarck in the genus *Solanum* and must be an error. = identity uncertain

*Solanum nigrum L. var. angustifolium* Filov, Kult. Fl. SSSR (Zhukovskii) 10: 382. 1958, not validly published; no diagnosis or description in Latin (Art. 39.1) = *S. villosum* Mill.

Solanum nigrum L. var. atriplicifolium Desp. ex Filov, Kult. Fl. SSSR (Zhukovskii) 10: 370–386. 1958, not validly published, no direct citation of basionym (Art. 39.1); also nom. illeg., later homonym of S. nigrum L. var. atriplicifolium G.Mey. = *S. nigrum* L.

Solanum nigrum L. subsp. australiense Filov, Kult. Fl. SSSR (Zhukovskii) 10: 380. 1958, not validly published; no diagnosis or description in Latin (Art 39.1) = *S. nigrum* L.

Solanum nigrum L. var. burbankii (Bitter) Filov, Kult. Fl. SSSR (Zhukovskii) 10: 383. 1958, as “*berbankii*”, not validly published, no direct citation of basionym (Art. 38.1) = *S. nigrum* L.

Solanum nigrum L. subsp. chinense Filov, Kult. Fl. SSSR (Zhukovskii) 10: 382. 1958, not validly published; no diagnosis or description in Latin (Art. 39.1) = *S. americanum* Mill.

Solanum nigrum L. var. chlorocarpum Koch, Syn. Fl. Germ. Helv., ed. 2: 600. 1846, not a new name, clear citation of Solanum nigrum var. chlorocarpum Spenn. (“S. nigrum chlorocarpum Spenn. fl. fri.”) = *S. nigrum* L.

Solanum nigrum L. subsp. cultum Filov, Kult. Fl. SSSR (Zhukovskii) 10: 383. 1958, not validly published; no diagnosis or description in Latin (Art. 39.1) = *S. scabrum* Mill.

Solanum nigrum L. var. frutescens Macloskie, Rep. Princeton Univ. Exped. Patagonia 8: 707. 1905, nomen nudum = *S. nitidibaccatum*, *S. triflorum*, *S. chenopodioides*, or *S. pygmaeum* that all occur in Northern Patagonia

Solanum nigrum L. forma genuinum Döll, Rhein. Flor. 412. 1843, not validly published (Art. 24.3) = *S. nigrum* L.

Solanum nigrum L. var. genuinum Godr., Fl. Lorraine 2: 133. 1843, not validly published (Art. 24.3) = *S. nigrum* L.

Solanum nigrum L. var. genuinum Hassl., Trab. Mus. Farm. Fac. Cienc. Med. Buenos Aires 21: 104. 1909, not validly published (Art. 24.3) = *S. nigrum* L.

Solanum nigrum L. var. griseocarpum Filov, Kult. Fl. SSSR (Zhukovskii) 10: 378. 1958, not validly published; no diagnosis or description in Latin (Art. 39.1) = *S. nigrum* L.

Solanum nigrum L. var. humile F.M.Bailey, Queensland Philos. Soc. 3: 1–4. 1881, not validly published; no diagnosis or description (Art. 38.1) = *S. nigrum* L.

Solanum nigrum L. var. inerme K.Koch, Linnaea 17: 283. 1843, not intended as a new name (also *nomen sub nudum*); protologue “Solanum nigrum L. β inerme. Rami non murieulati. In Transcaucasia abundat” is much less detailed than where in the same work Koch describes new taxa, neither does he attribute the name to himself as he does for other new taxa in this paper.

Solanum nigrum L. subsp. irano-algiricum Filov, Kult. Fl. SSSR (Zhukovskii) 10: 378. 1958, not validly published; no diagnosis or description in Latin (Art. 39.1) = *S. villosum* Mill.

Solanum nigrum L. var. legitimum Neilr., Fl. Nied.-Oesterr. 535. 1859, not validly published (Art. 24.3) = *S. nigrum* L.

Solanum nigrum L. subsp. memphiticum (Mart.) Marzell, Ill. Fl. Mitt.-Eur. [Hegi] 5(4): 2593. 1927, not intended as a new name; Marzell only says “Zur gesamtart S. nigrum gehört anscheinend auch S. memphiticum Mart.” [to the total *S. nigrum* also belongs *S. memphiticum* Mart.] = *S. nigrum* L.

Solanum nigrum L. var. merrillianum (Liou) Filov, Kult. Fl. SSSR (Zhukovskii) 10: 383. 1958, not validly published; no direct citation of basionym (Art. 38.1) = *S. americanum* Mill.

Solanum nigrum L. var. villosum (Mill.) Filov, Kult. Fl. SSSR (Zhukovskii) 10: 380. 1958, not validly published; no direct citation of basionym (Art. 38.1) = *S. villosum* Mill.

Solanum nigrum L. var. violaceum Chen ex Wessely, Feddes Repert. Spec. Nov. Regni Veg. 63(3): 293. 1960, nomen nudum; not intended as a new name, listed as one of the taxa accepted by Filov (1958, Kult. Fl. SSSR 20: 382). = *S. americanum* Mill.

Solanum nigrum L. var. vulgatum L., Sp. Pl. ed. 2: 266. 1762, evidently an orthographic variant of S*olanum nigrum* L. var. vulgare L. (1753). = *S. nigrum* L. (see Jarvis 2007).

Solanum nigrum L. var. vulgatum Dunal, Prodr. [A. P. de Candolle] 13(1): 50. 1852, not a new name, referring to Solanum nigrum var. vulgatum L. (and of others “auct.”) = *S. nigrum* L.

*Solanum nodiflorum* Desv. ex Dunal, Prodr. [A. P. de Candolle] 13(1): 46. 1852, pro syn. *Solanum desvauxii* Ham. = *S. americanum* Mill.

Solanum nodiflorum Jacq. var. acuminatum Chodat, Bull. Herb. Boissier, sér. 2, 2: 811. 1902, not intended as a new name, as “*acuminatum* (?)”, with no specimen cited. In the rest of this work the new taxa are clearly indicated with “nob.” and a specimen (or several) cited = *S. americanum* Mill.

Solanum nodiflorum var. fauriei (H.Lév.) O.Deg. & O.Deg., Plants Hawaii Natl. Parks 419. 1930, no evidence in any editions of Degener’s book (1930, 1945, 1974) that this name, occurring in several indices and based on *Solanum fauriei* H.Lév. was ever published; probably = *S. opacum* A.Braun & Bouché

Solanum nodiflorum Jacq. var. sativum A.Chev. Expl. Bot. Afrique Occ. Franç. 1: 464. 1920. nomen nudum; based on “Guinea. Kankan, dans les jardins cultives, 16 Mar 1899, *A.Chevalier 594*” = *S. scabrum* Mill.

*Solanum pigmaeum* Larrañaga, Escritos Damaso Antonio Larrañaga 2: 88. 1923, almost certainly not intended as a new name, an orthographic variant of *Solanum pygmaeum* Cav. (1806). In other parts of Larrañaga’s work (e.g. S. trilobum in vol. 1: 12), he indicates new taxa with “sp. n.” or gen. n.”; none of the names in vol. 2 is so designated, leading us to believe he was not coining new names, but instead using those already published. This work was compiled from his notes and diaries and these were heavily annotated (see intro in vol. 1 http://bdh-rd.bne.es/viewer.vm?id=0000016651&page=1) up to his death in 1848. = *S. pygmaeum* Cav.

Solanum photeinocarpum Nakam. & Odash. var. violaceum C.Y.Wu & S.C.Huang, Acta Phytotax. Sin. 16(2): 72. 1978, nomen nudum; incorrectly cited in this publication as *violaceum* Chen ex Wessely but Wessely (1960) did not formally publish this name, she cited Filov’s (1958) list of accepted taxa. Filov did not provide Latin descriptions or diagnoses (Art. 38.1) and so all names in that work are not validly published. The varietal epithet should be attributed only to Wu and Huang (1978), but they do not provide a Latin diagnosis either (Art. 39.1) = *S. americanum* Mill.

*Solanum pterocaulon* Rchb. ex Nyman, Consp. Fl. Eur. 3: 526. 1881. (1881), pro syn. *Solanum melanocerasum* Willd. = *Solanum scabrum* Mill.

*Solanum repens* Noronha, Verh. Batav. Genootsch. Kunsten 5: 84. 1791, nomen nudum = *S. nigrum* L.

*Solanum rubrum* Murray, Syst. Veg., ed. 14 (J. A. Murray) 224. 1784, not intended as a new name; Murray repeats Linnaeus’ (1767) polynomial exactly; Dunal’s (1813) citation of “Murr. Syst. veg. 183” in his description of *S. erythraeum* Dunal (1813: 238) is clearly an error = *S. rubrum* L. (nom. utique rej. prop.)

*Solanum villosum* Opiz ex Dunal, Prodr. [A. P. de Candolle] 13(1): 51. 1852, pro syn. *Solanum decipiens* Opiz. = *S. nigrum* L.

Solanum villosum Lam. var. alatum (Moench) Marzell, in Hegi, Ill. Fl. Mitt.-Eur. 5(4): 2594. 1927, not intended as a new name, citation of *Solanum alatum* Moench in text = *S. villosum* Mill.

Solanum villosum Lam. var. glabrescens Roem. & Schult., Syst. Veg., ed. 15 bis [Roemer & Schultes] 4: 592. 1819. This name was cited in Berchtold and Opiz (1843: 24), but it is not a published name; no such name was coined in *Systema Vegetabilium*; on page 592, however there is a “β glabrescens” in the synonymy of *S. villosum* Lam., but it is a reference in the synonymy “Solanum etc. B Hall. Helv. n 576 β glabrescens” – referring to Haller’s *Historia Stirpium Indigenarum Helvetiae Inchoata* (1768) a work in which only polynomials were used).

*Solanum viridescens* Hort. ex Rchb., Fl. Germ. Excurs. 391. 1831, pro syn. *Solanum humile* Bernh. ex Willd. = *S. villosum* Mill.

*Solanum virgatum* Endl. ex Sendtn., Fl. Bras. (Martius) 10: 13. 1846, pro syn. *Solanum gracile* Sendtn. = *S. chenopodioides* Lam.

## Supplementary Material

XML Treatment for Solanum alpinum

XML Treatment for Solanum americanum

XML Treatment for Solanum chenopodioides

XML Treatment for Solanum furcatum

XML Treatment for Solanum hirtulum

XML Treatment for Solanum memphiticum

XML Treatment for Solanum nigrum

XML Treatment for Solanum nitidibaccatum

XML Treatment for Solanum opacum

XML Treatment for Solanum palitans

XML Treatment for Solanum pseudospinosum

XML Treatment for Solanum pygmaeum

XML Treatment for Solanum retroflexum

XML Treatment for Solanum sarrachoides

XML Treatment for Solanum scabrum

XML Treatment for Solanum tarderemotum

XML Treatment for Solanum triflorum

XML Treatment for Solanum umalilaense

XML Treatment for Solanum villosum

## Supplementary materials

Supplementary material 1 Index to numbered collections Data type: (measurement/occurence/multimedia/etc.) Explanation note: species data This is an open access article distributed under the terms of the Creative Commons Attribution License (CC BY 4.0), which permits unrestricted use, distribution, and reproduction in any medium, provided the original author and source are credited.Tiina Särkinen, Peter Poczai, Gloria E. Barboza, Gerard M. van der Weerden, Maria Baden, Sandra Knapp

Supplementary material 2 Specimens examined (CSV format) Data type: species data This is an open access article distributed under the terms of the Creative Commons Attribution License (CC BY 4.0), which permits unrestricted use, distribution, and reproduction in any medium, provided the original author and source are credited.Tiina Särkinen, Peter Poczai, Gloria E. Barboza, Gerard M. van der Weerden, Maria Baden, Sandra Knapp

Supplementary material 3 Specimens examined (PDF format) Data type: species data This is an open access article distributed under the terms of the Creative Commons Attribution License (CC BY 4.0), which permits unrestricted use, distribution, and reproduction in any medium, provided the original author and source are credited.Tiina Särkinen, Peter Poczai, Gloria E. Barboza, Gerard M. van der Weerden, Maria Baden, Sandra Knapp

## References

[B1] Abraham Z (1981) Ethnobotany of the Todas, the Kotas and the Irulas of the Nilgiris. In: Jain SK (Ed.) Glimpses of Indian Ethnobotany. Oxford & IBH, New Delhi, 308–320.

[B2] Akubugwo IE Obasi AN Ginika SC (2007) Nutritional potential of the leaves and seeds of black nightshade Solanum nigrum L. var. virginicum from Afikpo-Nigeria. Pakistan Journal of Nutrition 6(4): 323–326. 10.3923/pjn.2007.323.326

[B3] Almeida SM Almeida MR (2008) Dictionary of scientific epithets with the meanings, local names, Sanskrit names and their botanical equivalents, in Maharashtra. TP Almeida, Mumbai.

[B4] Ammaan M Subramanian S (2017) Efficacy of organic manures and biofertilizers on soil microbial count and yield of Black Nightshade (*Solanum nigrum* L.). International Journal of Current Microbiology and Applied Sciences 6(7): 1780–1786. 10.20546/ijcmas.2017.607.215

[B5] Arnold SJ (1985) Eastern black nightshade: An increasing concern for soybean and forage producers. Crops Soils Magazine 37: 29–31.

[B6] Arora RK (1981) Native food plants of north eastern India. In: Jain SK (Ed.) Glimpses of Indian Ethnobotany. Oxford & IBH, New Delhi, 91–106.

[B7] Asano N Kato A Kizu H Matsui K Griffiths RC Jones MG Watson AA Nash RJ (1997) Enzymatic synthesis of the glycosides of calystegnines B_1_ and B_2_ and their glycosidase inhibitory activities. Carbohydrate Research 304(2): 173–178. 10.1016/S0008-6215(97)00227-9 PubMed 9449768

[B8] Ashtiana F Sefidkonb F (2011) Tropane alkaloids of *Atropa belladonna* L. and *Atropa acuminata* Royle ex Miers plants. Journal of Medicinal Plants Research 5(29): 6515–6522.

[B9] Axelius B (1992) Testa patterns in some species of *Physalis* L. and some other genera in the tribe Solaneae (Solanaceae). International Journal of Plant Sciences 153(3, Part 1): 488–502. 10.1086/297055

[B10] Backer CA Bakhuisen van der Brink RC (1965) Flora of Java. P. Noordhoff, Groningen.

[B11] Barboza GE (2003) Taxonomic revision of Solanum sect. Chamaesarachidium (Solaneae, Solanaceae). Nordic Journal of Botany 23(2): 155–168. 10.1111/j.1756-1051.2003.tb00377.x

[B12] Barboza GE Hunziker AT (2005) Revision of *Solanum fiebrigii* and *Solanum sinuatiexcisum*, and their inclusion in section Campanulisolanum. In: Keating RC Hollowell VC Croat TB (Eds) A festschrift for William G. D’Arcy: the legacy of a taxonomist, Monographs in systematic botany from the Missouri Botanical Garden, Vol. 104. Missouri Botanical Garden Press, St. Louis, 51–67.

[B13] Barboza GE Knapp S Särkinen T (2013) Grupo VII. Moreloide. In Anton AM, Zuloaga FO (Eds), Barboza GE (coord.) Flora Argentina vol. 13, Solanaceae. IOBDA-IMBIV, CONICET: Buenos Aires and Córdoba, Argentina, 231–264.

[B14] Barnea A Yom-Tov Y Friedman J (1990) Differential germination of two closely related species of *Solanum* in response to bird ingestion. Oikos 57(2): 222–228. 10.2307/3565943

[B15] Baylis GTS (1958) A cytogenetical study of New Zealand forms of *Solanum nigrum* L., *S. nodiflorum* Jacq. and *S. gracile* Otto. Transactions of the Royal Society of New Zealand 85: 379–285.

[B16] Bennett MD Leitch IJ (2012) Plant DNA C-values database (Release 6.0, Dec 2012). http://data.kew.org/cvalues [accessed 8 July 2017]

[B17] Berchtold F Opiz WB (1843) Oekonomisch-technische Flora Böhmens vol. 3. Thomas Thabor, Annakoster.

[B18] Bergau N Bennewitz S Syrowatka F Hause G Tissier A (2015) The development of type VI glandular trichomes in the cultivated tomato *Solanum lycopersicum* and a related wild species *S. habrochaites.* BMC Plant Biology 15(1): 289. 10.1186/s12870-015-0678-z PMC4676884 26654876

[B19] Berinyuy JE Fontem DA Focho DA Schippers RR (2002) Morphological diversity of *Solanum scabrum* accessions in Cameroon. Plant Genetic Resources Newsletter (Rome, Italy) 131: 42–48.

[B20] Bertová L GoliaŠová K (1993) Flóra Slovenska V/1. VEDA, vydavatel’stvo Slovenskej akadémie vied, Bratislava.

[B21] von Besser WSJG (1809) Primitiae florae Galiciae Austriacae utriusque pars 1. A. Doll, Vienna.

[B22] Bhiravamurty PV (1975) Pachytene chromosome morphology of *Solanum nodiflorum* Jacq. Caryologia 28(3): 287–294. 10.1080/00087114.1975.10796618

[B23] Bhiravamurty PV Rethy P (1983) Taximetric studies of the *Solanum nigrum* L. complex. Proceedings of the Indian National Science Academy B 49: 661–666.

[B24] Bhiravamurty PV Rethy P (1984) Origin and evolution of tetraploid forms within the *Solanum nigrum* L. complex. Proceedings of the Indiana Academy of Sciences 93: 553–560. [Plant Sciences]

[B25] Bitter G (1911) Steinzellkonkretionen im Fruchtfleisch beerentragender Solanaceen und deren systematische Bedeutung. Botanische Jahrbücher für Systematik, Pflanzengeschichte und Pflanzengeographie 45: 483–507.

[B26] Bitter G (1912a) Solana nova vel minus cognita I. Repertorium Specierum Novarum Regni Vegetabilis 10(33–36): 529–565. 10.1002/fedr.19120103305

[B27] Bitter G (1912b) Solana nova vel minus cognita II. Repertorium Specierum Novarum Regni Vegetabilis 11(9–15): 202–237. 10.1002/fedr.19120110917

[B28] Bitter G (1913a) Solana Africana I. Botanische Jahrbücher für Systematik, Pflanzengeschichte und Pflanzengeographie 49: 560–569.

[B29] Bitter G (1913b) Solana nova vel minus cognita X. XXVII. Morellae novae vel criticae. Repertorium Specierum Novarum Regni Vegetabilis 12: 75–89.

[B30] Bitter G (1914) Weitere Untersuchungen über das Vorkommen von Steinzellkonkretionen im Fruchtfleisch beerentragender Solanaceen. Abhandlungen des Naturwissenschaftlichen Vereins zu Bremen 23: 114–163.

[B31] Bitter G (1917) Solana Africana II. Botanische Jahrbücher für Systematik, Pflanzengeschichte und Pflanzengeographie 54: 416–506.

[B32] Bitter G (1919) Die papuasischen Arten von *Solanum*. Botanische Jahrbücher für Systematik, Pflanzengeschichte und Pflanzengeographie 55: 59–113.

[B33] Bitter G (1921) Solana Africana III. Botanische Jahrbücher für Systematik, Pflanzengeschichte und Pflanzengeographie 57: 248–286.

[B34] Bitter G (1923) Solana Africana IV. Repertorium Specierum Novarum Regni Vegetabilis 16: 1–320.

[B35] Blom C (1935) Adventiva *Solanum*-arter i Sveriges flora. Acta Horti Gothoburgensis 10: 195–208.

[B36] Blume C (1826) Solanaceae. Bijdragen tot de flora van Nederlandsch Indië pt. 13. Ter Lands Drukkerij, Batavia, 694–707.

[B37] Bohs L (1998) *Cyphomandra* (Solanaceae). Flora Neotropica 63: 1–176.

[B38] Bohs L (2005) Major clades in *Solanum* based on *ndh*F sequences. In: Keating RC Hollowell VC Croat TB (Eds) A festschrift for William G. D’Arcy: the legacy of a taxonomist, Monographs in Systematic Botany from the Missouri Botanical Garden, Vol. 104. Missouri Botanical Garden Press, St. Louis, 27–49.

[B39] Bohs L (in press) *Solanaceae* In: Flora of North America Editorial Committee (Eds) Flora of North America north of Mexico. Volume 14: Magnoliophyta: Gentianaceae to Hydroleaceae. Oxford University Press, New York.

[B40] Boissier P-E (1849) Diagnoses plantarum orientalium novarum. N^o^. 11. M.Ducloux & Cons., Paris.

[B41] Bradley V Collins DJ Eastwood FW Irvine MC Swan JM (1979) Distribution of steroidal alkaloids in Australian species of *Solanum*. In: Hawkes JG Lester RN Skelding AD (Eds) The biology and taxonomy of the Solanaceae. Academic Press, London, 203–209.

[B42] Bravo C Velilla S Bautista LM Peco B (2014) Effects of great bustard (*Otis tarda*) gut passage on black nightshade (*Solanum nigrum*) seed germination. Seed Science Research 24(03): 265–271. 10.1017/S0960258514000178

[B43] Bridson GDR (2004) BPH-2: Periodicals with Botanical Content. Hunt Institute for Botanical Documentation, Pittsburgh. Available and searchable online at http://fmhibd.library.cmu.edu/fmi/iwp/cgi?-db=BPH_Online&-loadframes

[B44] BSBI [Botanical Society of Britain and Ireland] (2017) Taxonomy “Leicester” database, http://rbg-web2.rbge.org.uk/BSBI/intro.php [accessed 23 August 2017]

[B45] Buchmann SL Jones CE Colin LJ (1977) Vibratile pollination of *Solanum douglasii* and *S. xanti* (Solanaceae) in southern California. Wasmann Journal of Biology 35: 1–25.

[B46] Bukenya ZR (1993) Studies in the taxonomy of the genus *Solanum* in Uganda. PhD thesis. Makerere University, Uganda.

[B47] Bukenya ZR (1996) Uses, chromosome number and distribution of *Solanum* species in Uganda. In: Van der Maesen LJG van Medenbach de Rooy JM (Eds) The Biodiversity of African Plants. Kluwer Academic Publishers, The Netherlands, 33–37.

[B48] Bukenya ZR Carasco JF (1999) Ethnobotanical aspects of *Solanum* L. (Solanaceae) in Uganda. In: Nee M Symon DE Lester RN Jessop JP (Eds) Solanaceae IV: Advances in Biology and Utilization. Royal Botanic Gardens, Kew, London, 345–360.

[B49] Bukenya ZR Carasco JF (1995) *Solanum* (Solanaceae) in Uganda. Bothalia 25: 43–59.

[B50] Bukenya ZR Hall JB (1988) *Solanum* (Solanaceae) in Ghana. Bothalia 18(1): 79–88. 10.4102/abc.v18i1.983

[B51] Burgert KL Burnside OC Fenster CR (1973) Black nightshade leaves its mark. Quarterly Serving Farm, Ranch, and Home, Lincoln College of Agriculture University of Nebraska 20: 8–10.

[B52] Burkhill HM (2000) The useful plants of West Tropical Africa, ed. 2. Vol. 5. Families S-Z. Royal Botanic Gardens, Kew.

[B53] Campos MA Ribeiro SG Rigden DJ Monte DC Grossi de Sa MF (2002) Putative pathogenesis-related genes within Solanum nigrum L. var. americanum genome: Isolation of two genes coding for PR5-like proteins, phylogenetic and sequence analysis. Physiological and Molecular Plant Pathology 61(4): 205–216. 10.1006/pmpp.2002.0430

[B54] Carle R (1981) Investigations on the content of steroidal alkaloids and sapogenins within Solanum sect. Solanum (=sect. Morella) (Solanaceae). Plant Systematics and Evolution 138(1–2): 61–71. 10.1007/BF00984609

[B55] Cernansky R (2015) The rise of Africa’s super vegetables. Nature 522(7555): 146–148. 10.1038/522146a 26062494

[B56] Chakrabarti SK Sharma A (1994) Karyosystematic studies in Solanum section Solanum: State-of-the-art report. The Nucleus 37: 142–197.

[B57] Chase MW Knapp S Cox AV Clarkson JJ Butsko Y Joseph J Savolainen V Parokonny AS (2003) Molecular systematics, GISH and the origin of hybrid taxa in *Nicotiana* (Solanaceae). Annals of Botany 92(1): 107–127. 10.1093/aob/mcg087 12824072 PMC4243627

[B58] Child A Lester RN (1991) Life form and branching with the Solanaceae In: Hawkes JG, Lester RN, Nee M, Estrada N (Eds) Solanaceae III: taxonomy, chemistry, evolution, Royal Botanic Gardens Kew, Richmond, 151–159.

[B59] Chowdhury N Ghosh A Chandra G (2008) Mosquito larvicidal activities of *Solanum villosum* berry extract against the dengue vector *Stegomyia aegypti* BMC Complementary and Alternative Medicine 8: 10, 10.1186/1472-6882-8-10 PMC2364612 18387176

[B60] Chown SL Huiskes ADL Gremmen NJM Lee JE Terauds A Crosbie K Frenot Y Hughes KA Imura S Kiefer K Lebouvrier M Raymond B Tsujimoto M Ware C van de Vijer B Bergstrom DM (2012) Continent-wide risk analysis for the establishment of nonindigenous species in Antarctica. Proceedings of the National Academy of Sciences of the United States of America 109(13): 4938–4943. 10.1073/pnas.1119787109 22393003 PMC3323995

[B61] Cipollini ML Bohs L Mink K Paulk E Böhning-Gaese K (2002) Secondary metabolites of ripe felshy fruits: ecology and phylogeny in the genus *Solanum*. In: Levey DJ Silva WR Galetti M (Eds) Seed dispersal and frugivory: ecology, evolution and conservation. CABI International, Wallingford and New York, 111–128.

[B62] Cipollini ML Levey DJ (1997a) Why are some fruits toxic? Glycoalkaloids in *Solanum* and fruit choice by vertebrates. Ecology 78: 782–798.

[B63] Cipollini ML Levey DJ (1997b) Antifungal activity of *Solanum* fruit glycoalkaloids: Implications for frugivory and seed dispersal. Ecology 78(3): 799–809. 10.1890/0012-9658(1997)078[0799:AAOSFG]2.0.CO;2

[B64] Clarkson JJ Lim KY Kovarik A Chase MW Knapp S Leitch AR (2005) Long-term genome diploidization in allopolyploid Nicotiana section Repandae (Solanaceae). The New Phytologist 168(1): 241–252. 10.1111/j.1469-8137.2005.01480.x 16159337

[B65] Colla A (1835) Herbarium Pedemontanum Vol. 4. Augustae Taurinorum, Torino.

[B66] Colon LT Eijlander R Budding DJ Pieters MMJ van Ijzendoorn MT Hoogendoorn J (1993) Resistance to potato late blight (*Phytophthora infestans* (Mont.) de Bary) in *Solanum nigrum, S. villosum* and their sexual hybrids with *S. tuberosum* and *S. demissum.* Euphytica 66(1–2): 55–64. 10.1007/BF00023508

[B67] Cracraft J (1989) Speciation and its ontogeny: the empirical consequences of alternative species concepts for understanding patterns of differentiation. In: Otte D Endler JA (Eds) Speciation and its consequences. Sinauer and Associates, New York, 28–59.

[B68] Cufodontis G (1963) Enumeratio Plantarum Aethiopiae Spermatophyta (Sequentia). Bulletin du Jardin Botanique de l’État à Bruxelles 33 (Suppl.): 830–876.

[B69] Culpeper N (1814) Culpeper’s complete herbal. [1814 edition; first published in 1653]. Richard Evans, London.

[B70] D’Arcy WG (1972) Solanaceae studies II: Typification of subdivisions of *Solanum*. Annals of the Missouri Botanical Garden 59(2): 262–278. 10.2307/2394758

[B71] D’Arcy WG (1974a) [“1973”]) Solanaceae, Flora of Panama. Part 9. Annals of the Missouri Botanical Garden 60: 573–780.

[B72] D’Arcy WG (1974b) *Solanum* and its close relatives in Florida. Annals of the Missouri Botanical Garden 61(3): 819–867. 10.2307/2395032

[B73] D’Arcy WG Rakotozafy A (1994) Solanaceae. In: Morat P (Ed.) Flore de Madagascar et des Comores, Famille 176. Muséum National D’Histoire Naturelle, Paris, 1–146.

[B74] Dammer U (1906) [“1907”]) Solanaceae africanae I. Botanische Jahrbücher für Systematik, Pflanzengeschichte und Pflanzengeographie 38: 176–196.

[B75] Danert S (1958) Die Verzweigung der Solanaceen im reproduktiven Bereich. Abhandlungen der Deutschen Akademie der Wissenschaften zu Berlin, Klasse für Chemie, Geologie und Biologie 6: 1–183.

[B76] Danert S (1967) Die Verzweigung als infragenerisches Gruppenmerkmal in der Gattung *Solanum* L. Die Kulturpflanze 15(1): 275–292. 10.1007/BF02095719

[B77] Danert S (1970) Infragenerische Taxa der Gattung *Solanum* L. Die Kulturpflanze 18(1): 253–297. 10.1007/BF02095597

[B78] Davis JI (1997) Evolution, evidence, and the role of species concepts in phylogenetics. Systematic Botany 22(2): 373–403. 10.2307/2419463

[B79] Davis PH Heywood VH (1963) Principles of angiosperm taxonomy. Van Nostrand, New York.

[B80] de Candolle R Radcliffe-Smith A (1981) Nathaniel Wallich, MD, PhD, FRS, FLS, FRGS (178601854) and the herbarium of the Honourable East India Company, and their relation to the de Candolles of Geneva and the *Great Prodromus.* Botanical Journal of the Linnean Society 83(4): 325–348. 10.1111/j.1095-8339.1981.tb00355.x

[B81] De Luca PA Vallejo-Marín M (2013) Whats the ‘buzz’ about? The ecology and evolution of buzz-pollination. Current Opinion in Plant Biology 16(4): 429–435. 10.1016/j.pbi.2013.05.002 23751734

[B82] Defelice MS (2003) The Black Nightshades, Solanum nigrum L. et al.—Poison, Poultice, and Pie. Weed Technology 17(2): 421–427. 10.1614/0890-037X(2003)017[0421:TBNSNL]2.0.CO;2

[B83] Degener O (1945) Plants of Hawaii National Park. Illustrative of plants and customs of the South Seas. Edwards Brothers, Ann Arbor, Michigan.

[B84] Dehmer KJ (2001) Conclusions on the taxonomy of the *Solanum nigrum* complex by molecular analyes of IPK germplasm accessions. In: van den Berg RG Barendse GWM van der Weerden GM Mariani C (Eds) Solanaceae V: Advances in taxonomy and utilization. University Press, Nijmegen, 85–96.

[B85] Dehmer KJ Hammer K (2004) Taxonomic status and geographic provenance of germplasm accessions in the *Solanum nigrum* L. complex: AFLP data. Genetic Resources and Crop Evolution 51(5): 551–558. 10.1023/B:GRES.0000024163.86762.fc

[B86] del Vitto LA Petenatti FM (1999) Notas en *Solanum* (Solanaceae) de Argentina II. Aportes al conocimiento de la Sect. Episarcophyllum. Kurtziana 27: 319–326.

[B87] Dillenius JJ (1732) Hortus Elthamensis seu plantarum rariorum. Published by the author, London.

[B88] Dinssa FF Hanson P Dubois T Tenkouano A Stoilova T Hughes JA Keatinge JDH (2016) AVDRC – the World Vegetable Center’s women-oriented improvement and development strategy for traditional African vegetables in sub-Saharan Africa. European Journal of Horticultural Science 81(2): 91–105. 10.17660/eJHS.2016/81.2.3

[B89] Dodoens R (1554) Cruijdeboeck. Jan van der Loe, Antwerp, available online (with colored plates, at http://leesmaar.nl/cruijdeboeck/index.htm

[B90] Dodoens R (1563) Crŭijde Boeck. Antwerp.

[B91] Dodoens R (1583) Stirpium historiae pemptades sex, sive libri XXX. C. Plantini, Antwerp.

[B92] Döll JC (1843) Rheinische flora: Beschreibung der wildwachsenden und cultivirten Pflanzen des Rheingebietes. H.L. Brönner, Frankfurt am Main.

[B93] Don G (1837) A general history of the dichlamydeous plants. Vol. 4. J.G. and F. Rivington, London.

[B94] Dostál J (1949) Květena ČSR. Československá Botanická Společnost, Prague.

[B95] Dräger B Funck C Höhler A Mrachatz G Nahrstedt A Portsteffen A Schaal A Schmidt R (1994) Calystegines as a new group of tropane alkaloids in Solanaceae. Plant Cell, Tissue and Organ Culture 38: 235–240. 10.1007/BF00033882

[B96] Dumont de Courset GLM (1802) Le botaniste cultivateur. Vol. 2. J.J. Fuchs, Paris.

[B97] Dumortier BCJ (1827) Florula Belgica. J. Casterman, Tournay.

[B98] Dunal MF (1813) Histoire naturelle, médicale et économique des *Solanum* et des genres qui ont été confundus avec eux. Renaud, Montpellier.

[B99] Dunal MF (1814) *Solanum*. In: Lamarck J, Poiret JLM (Eds) Encyclopedie methodique. Botanique. Suppl. 3(2): 369–780. Agasse, Paris.

[B100] Dunal MF (1816) Solanorum generumque affinium synopsis. Renaud, Montpellier.

[B101] Dunal MF (1852) Solanaceae In: Candolle AP de (Ed.) Prodromus systematis naturalis regni vegetabilis 13(1): 1–690. V. Masson, Paris.

[B102] Edmonds JM (1971) Solanaceae. In: Stearn WT Taxonomic and nomenclatural notes on Jamaican gamopetalous plants. Journal of the Arnold Arboretum 52: 634–635.

[B103] Edmonds JM (1972) A synopsis of the taxonomy of Solanum sect. Solanum (Maurella) in South America. Kew Bulletin 27(1): 95–114. 10.2307/4117874

[B104] Edmonds JM (1977) Taxonomic studies on Solanum L. section Solanum (Maurella). Botanical Journal of the Linnean Society 75(2): 141–178. 10.1111/j.1095-8339.1977.tb01482.x

[B105] Edmonds JM (1978) Numerical taxonomic studies on Solanum L. section Solanum (Maurella). Botanical Journal of the Linnean Society 76(1): 27–51. 10.1111/j.1095-8339.1978.tb01497.x

[B106] Edmonds JM (1979a) Biosystematics of Solanum L., section Solanum (Maurella). In: Hawkes JG Lester RN Skelding AD (Eds) Biology and Taxonomy of the Solanaceae. Linnean Society of London, Academic Press, London, 529–548.

[B107] Edmonds JM (1979b) Nomenclatural notes on some species of *Solanum* L. found in Europe. Botanical Journal of the Linnean Society 78(3): 213–233. 10.1111/j.1095-8339.1979.tb02194.x

[B108] Edmonds JM (1981) The artificial synthesis of Solanum × procurrens Leslie (*S. nigrum* L. × *S. sarrachoides* Sendtn.). Watsonia 13: 203–207.

[B109] Edmonds JM (1982) Epidermal hair morphology in Solanum L. section Solanum. Botanical Journal of the Linnean Society 85(3): 153–167. 10.1111/j.1095-8339.1982.tb02583.x

[B110] Edmonds JM (1983) Seed coat structure and development in Solanum L. section Solanum (Solanaceae). Botanical Journal of the Linnean Society 87(3): 229–246. 10.1111/j.1095-8339.1983.tb00992.x

[B111] Edmonds JM (1984a) Pollen morphology of Solanum L. section Solanum. Botanical Journal of the Linnean Society 88(3): 237–251. 10.1111/j.1095-8339.1984.tb01573.x

[B112] Edmonds JM (1984b) Solanum L. section Solanum – A name change in *S. villosum* Miller. Botanical Journal of the Linnean Society 89(2): 165–170. 10.1111/j.1095-8339.1984.tb01007.x

[B113] Edmonds JM (1986) Biosystematics of *Solanum sarrachoides* Sendtn. and *S. physalifolium* Rusby (*S. nitidibaccatum* Bitter). Botanical Journal of the Linnean Society 92(1): 1–38. 10.1111/j.1095-8339.1986.tb01425.x

[B114] Edmonds JM (2005) Solanum L. section Solanum. In: Pope GV, Martin ES (Eds) Flora Zambesiaca 8, Royal Botanic Gardens, Kew, London, 81–86.

[B115] Edmonds JM (2006a) Solanum L. section Solanum. In: Thulin M (Ed.) Flora of Somalia 3. Royal Botanic Gardens, Kew, London, 207–208.

[B116] Edmonds JM (2006b) Solanum L. section Solanum. In: Hedberg I Edwards S (Eds) Flora of Ethiopia and Eritrea, Vol 5. Uppsala and Addis Ababa, 115–122.

[B117] Edmonds JM (2007) Forsskål's Solanum sect. Solanum (Solanceae) specimens. Kew Bulletin 62: 657–670.

[B118] Edmonds JM (2012) *Solanum* spp. In: Beentje H (Ed.) Flora of Tropical East Africa Solanaceae. Royal Botanic Gardens, Kew, 105–125.

[B119] Edmonds JM Chweya JA (1997) Black nightshades. *Solanum nigrum* L. and related species. Institute of Plant Genetics and Crop Plant Research Gatersleben/ International Plant Genetic Resources Institute, Rome.

[B120] Edmonds JM Glidewell SM (1977) Acrylamide gel electrophoresis of seed proteins from some Solanum (section Solanum) species. Plant Systematics and Evolution 127(4): 277–291. 10.1007/BF00985991

[B121] Eijlander R Stiekema WJ (1994) Biological containment of potato (*Solanum tuberosum*): Outcrossing to the related wild species black nightshade (*Solanum nigrum*) and bittersweet (*Solanum dulcamara*). Sexual Plant Reproduction 7(1): 29–40. 10.1007/BF00241885

[B122] Eldridge AC Hockridge ME (1983) High-performance liquid chromatographic separation of eastern black nightshade (*Solanum ptychanthum*) glycoalkaloids. Journal of Agricultural and Food Chemistry 31(6): 1218–1220. 10.1021/jf00120a019

[B123] Ellison W (1936) Synapsis and sterility in a *Solanum* hybrid. Journal of Genetics 32(3): 473–478. 10.1007/BF02982527

[B124] El-Sherbini GT Zayed RA El-Sherbini ET (2009) Molluscidal activity of some *Solanum* species extracts against the snail *Biomphalaria alexandrina*. Journal of Parasitology Research 2009, Article ID 474360, 5 pp. 10.1155/2009/474360 PMC2915658 20721329

[B125] Essou J-P Hermans MJW (2006) Solanaceae. In: Akoegninou A van der Burg WJ van der Maesen LJG (Eds) Flore analytique du Bénin. Universitéd’Abomey-Calavi, Cotonou, 947–958.

[B126] Euro+Med (2006) Euro+Med PlantBase – the information resource for Euro-Mediterranean plant diversity. http://ww2.bgbm.org/EuroPlusMed/ [accessed 23 August 2017].

[B127] FAO (1988) Traditional Food Plants. A resource book for promoting the exploitation and consumption of food plants in arid, semi-arid and sub-humid lands of eastern Africa, 458–466. FAO Food and Nutrition Paper 42. 3255608

[B128] Filov AI (1958) *Solanum nigrum* L. In: Zhukovshy PM (Ed.) Kulturnaya Flora SSSR Vol. 20 (Brezhnev DD, Ed.), Vegetable plants fam. Solanaceae. State Agricultural Publishing Office, Moscow & Leningrad, 370–386.

[B129] Fischer MA Adler W Oswald K (2005) Exkursionsflora für Österreich, Liechtenstein und Südtirol. Land Oberösterreich, Biologiezentrum der OÖ Landesmuseen, Linz.

[B130] Fitton JG (1987) The Cameroon line, West Africa: a comparison between oceanic and continental alkaline volcanism. Geological Society Special Publication 30: 273–291.

[B131] Flier WG van den Bosch GBM Turkensteen LJ (2003) Epidemiological importance of *Solanum sisymbriifolium, S. nigrum* and *S. dulcamara* as alternative hosts for *Phytophthora infestans*. Plant Pathology 52(5): 595–603. 10.1046/j.1365-3059.2003.00922.x

[B132] Fokon E Domugang F (1989) In vivo assessment of nutritive values of proteins “in situ” in 99 leaves of *Solanum nigrum* L., *Xanthostoma* sp. and *Gnetum africanum* L. The Indian Journal of Nutrition and Dietetics 26: 366–373.

[B133] Frederiksen S Rasmussen FN Seberg O (2006) Dansk Flora. Gyldendal, Københavns.

[B134] Friis I Demissew S van Breugel P (2010) Atlas of the potential vegetation of Ethiopia. Biologiske Skrifter 58: 1–307

[B135] Frodin DG (2004) History and concepts of big plant genera. Taxon 53(3): 753–776. 10.2307/4135449

[B136] Fuchs L (1542) De Historia Stirpium. M Isingrin, Basel.

[B137] Fuchs L (1543) Neu Kreuterbuch. M Isingrin, Basel. [fascimile edition printed in 2001 and published by Taschen, Cologne].

[B138] Ganapathi A (1987) Phyletic relationship of some tetraploid taxa of the Solanum L. section Solanum (Maurella). Israel Journal of Botany 36: 31–39.

[B139] Ganapathi A Rao GR (1980) Genetic system and interrelationship between *Solanum retroflexum* and *S. nodiflorum* of *S. nigrum* complex. Current Science 49: 598–599.

[B140] Ganapathi A Rao GR (1985) Spontaneous triploidy in *Solanum nigrum* L. complex. Current Science 54: 1242.

[B141] Ganapathi A Rao GR (1986a) Taxonomic status of the *Solanum nigrum* complex found in India. Proceedings of the Indiana Academy of Sciences 96: 241–246. [Plant Science]

[B142] Ganapathi A Rao GR (1986b) A note on origin and cytology of a heptaploid plant of Solanum section Solanum. Current Science 55: 867–868.

[B143] Ganapathi A Rao GR (1986c) The crossability and genetic relationship between *Solanum retroflexum* Dun. and *S. nigrum* L. Cytologia 51(4): 757–762. 10.1508/cytologia.51.757

[B144] Ganapathi A Rao GR (1986d) Nature of sterility and mechanism of the evolution of higher ploidy in Solanum section Solanum (Maurella). Canadian Journal of Genetics and Cytology 28(6): 1044–1048. 10.1139/g86-146

[B145] Ganapathi A Rao GR (1987a) Phylogenetic relationships in the evolution of *Solanum scabrum.* Genome 29(4): 639–642. 10.1139/g87-107

[B146] Ganapathi A Rao GR (1987b) Cytogenetics of a pentaploid hybrid of *Solanum scabrum* Mill. and *S. villosum* Mill. Current Science 56: 1024.

[B147] Ganapathi A Rao GR (1988) Cytogeography and nomenclatural notes on Solanum L. section Solanum. Current Science 57: 739.

[B148] Gerard J (1597) The Herball or Generall Historie of Plantes. John Norton, London.

[B149] Gerasimenko II Reznikova SA (1968) A cytological investigation of the genus *Solanum* L. Botanicheskii Zhurnal 53: 505–513. [in Russian]

[B150] Ghazanfar SA (1994) Handbook of Arabian medicinal plants. CRC Press, Boca Raton.

[B151] Glover BJ Bunnewell S Martin C (2004) Convergent evolution within the genus *Solanum*: The specialized anther cone develops through alternative pathways. Gene 331: 1–7. 10.1016/j.gene.2004.01.027 15094186

[B152] Gmelin JF (1791) Systema naturae, ed. 13 bis. Vol. 2. G.E. Beer, Leipzig.

[B153] Gras A (1863) Nouvelle note sur quelques rectifications de synonymie. Bulletin de la Société botanique de France 10: 592–595.

[B154] Gray A (1878) Order XCV. Solanaceae. Synoptical Flora of North America vol. 2(1). Ivison, Blakeman, Taylor & Company, New York, 224–244.

[B155] Grieve M (1931) A modern herbal. Jonathan Cape, London.

[B156] von Haller A (1768) Historia stirpium indigenarum Helvetiae inchoate. Bernae, Sumptibus Societatis typographicae.

[B157] Hämet-Ahti L Suominen J Ulvinen T Uotila P (1998) Retkeilykasvio. Luonnontieteellinen keskusmuseo, Kasvimuseo, Helsinki.

[B158] Harden GJ (1993) Flora of New South Wales Vol. 3. New South Wales University Press, Kensington, Australia.

[B159] Hartvig P (2015) Atlas Flora Danica Vol. 3. Gyldendal, Københavns.

[B160] Hawkes JG Edmonds JM (1972) *Solanum* L. In: Tutin TG Heywood VH Burges NA Moore DM Valentine DH Walters SM Webb DA (Eds) Flora Europaea, Vol. 3. Cambridge University Press, Cambridge, 197–199.

[B161] Healy AJ (1974) Identification of nightshades (*Atropa* and *Solanum*) in New Zealand. Proceedings of the 27^th^ New Zealand Weed and Pest Control Conference: 41–58.

[B162] Heine H (1960) Notes on *Solanum*. Kew Bulletin 14(2): 245–249. 10.2307/4114799

[B163] Heiser CB (1955) The *Solanum nigrum* complex in Costa Rica. Ceiba 4: 293–299.

[B164] Heiser CB (1963) Estudio biosistematico de *Solanum* (Morella), en el Ecuador. Ciencia y Naturaleza 6: 50–58.

[B165] Heiser CB (1969) Nightshades, the paradoxical plants. WH Freeman and Co, San Francisco.

[B166] Heiser CB Burton DL Schilling EE (1979) Biosystematic and taxometric studies of the *Solanum nigrum* complex in eastern North America. In: Hawkes JG Lester RN Skelding AD (Eds) The biology and taxonomy of the Solanaceae. Academic Press, London, 513–527.

[B167] Heiser CB Jr Soria J Burton DL (1965) A numerical taxonomic study of *Solanum* species and hybrids. American Naturalist 99(909): 471–488. 10.1086/282392

[B168] Henderson RJF (1974) *Solanum nigrum* L. (Solanaceae) and related species in Australia. Contributions from the Queensland Herbarium 16: 1–78.

[B169] Heo KS Lim KT (2005) Glycoprotein isolated from *Solanum nigrum* L. modulates the apoptotic-related signals in 12-o-Tetradecanoylphorbol 13-Acetate-stimulated MCF-7 cells. Journal of Medicinal Food 8(1): 69–77. 10.1089/jmf.2005.8.69 15857213

[B170] Hitchcock AS Green ML (1929) Standard-species of Linnean genera of Phanerogamae (1753–54). In: International Botanical Congress, Cambridge, England, 1930. Nomenclatural proposals by British Botanists: 111–199.

[B171] Hooker WJ (1837) Ord. LXV. Solanaeae. In: Flora boreali-Americana; or the botany of the northern parts of British America. Vol. 2. Henry G. Bohn, London, 90–91.

[B172] Horsman K Bergervoet JEM Jacobsen E (1997) Somatic hybridization between *Solanum tuberosum* and species of the *S. nigrum* complex: Selection of vigorously growing and flowering plants. Euphytica 96(3): 345–352. 10.1023/A:1003038419126

[B173] Huang KC (1993) The pharmacology of Chinese herbs. CRC Press, Boca Raton.

[B174] Hul S Dy Phon P (2014) Solanaceae. In: Aubréville A, Leroy JF, Morat P (Eds) Flore du Cambodge, du Laos et du Viêtnam. Vol. 35. Muséum Nationale d’Historie Naturelle and Royal Botanic Garden, Edinburgh, Paris and Edinburgh.

[B175] Ignacimuthu S Ayyanar M Sankarasivaraman K (2006) Ethnobotanical investigations among tribes in Madurai district of Tamil Nadu, India. Journal of Ethnobiology and Ethnomedicine 2006: 25. 10.1186/1746-4269-2-25 PMC1475842 16689985

[B176] IUCN (2016) Guidelines for Using the IUCN Red List Categories and Criteria. Version 12. Prepared by the Standards and Petitions Subcommittee. http://www.iucnredlist.org/documents/RedListGuidelines.pdf

[B177] Jacoby A Labuschagne MT (2006) Hybridization studies of five species of the *Solanum nigrum* complex found in South Africa and two cocktail tomato cultivars. Euphytica 149(3): 303–307. 10.1007/s10681-005-9078-z

[B178] Jacoby A Labuschagne MT Viljoen CD (2003) Genetic relationships between Southern African *Solanum retroflexum* Dun and other related species measured by morphological and DNA markers. Euphytica 132(1): 109–113. 10.1023/A:1024657827796

[B179] Jaeger P-ML (1985) Systematic studies in the genus *Solanum* L. in Africa. PhD thesis, University of Birmingham, Birmingham, UK, 1–272.

[B180] Jagatheeswari D Bharathi T Sheik Jahabar Ali H (2013) Black night shade (*Solanum nigrum* L.) – and updated review. International Journal of Pharmaceutical and Biological Archives 4(2): 288–295.

[B181] Jain SK Borthakur SK (1986) Solanaceae in Indian tradition, folklore and medicine. In: D’Arcy WG (Ed.) Solanaceae Biology and Systematics. Columbia University Press, New York, 577–583.

[B182] Jardine N Edmonds JM (1974) The use of numerical methods to describe population differentiation. The New Phytologist 73(6): 1259–1277. 10.1111/j.1469-8137.1974.tb02155.x

[B183] Jarvis CE (2007) Order Out of Chaos: Linnaean Plant Names and Their Types. Linnean Society of London, London.

[B184] Jauzein P Nawrot O (2011) Flore d’Île-de-France. Éditions Quæ, Toulouse.

[B185] Jørgensen CA (1928) The experimental production of heteroploid plants in the genus *Solanum*. Journal of Genetics 19(2): 133–211. 10.1007/BF02984237

[B186] Kamoun S Huitema E Vleeshouwers VGAA (1999) Resistance to oomycetes: A general role of the hypersensitive response? Trends in Plant Science 4(5): 196–200. 10.1016/S1360-1385(99)01404-1 10322560

[B187] Kanitz A (1863) Pauli Kitaibelii Additamenta ad Floram Hungaricam. Linnaea 32: 305–641.

[B188] Keding G Weinberger K Swai I Mndiga H (2007) Diversity, traits and use of traditional vegetables in Tanzania. Technical Bulletin 40. AVRDC-The World Vegetable Center, Shanhua, Taiwan.

[B189] Kenward HK Hall AR (1995) Biological evidence from Anglo-Scandinavian deposits at 16–22 Coppergate. The Archaeology of York 14: 435–797.

[B190] Kingsbury JM (1964) Poisonous plants of the United States and Canada. Prentice-Hall, New Jersey.

[B191] Kirschner J Kirschnerová L Štĕpánek J (2007) Generally accepted plant names base on material from the Czech Republic and published in 1753–1820. Preslia 79: 323–365.

[B192] Knapp S (1986) Reproductive biology of Solanum section Geminata in a Costa Rican cloud forest. In: D’Arcy WG (Ed.) Solanaceae: biology and systematics. Columbia University Press, New York, 253–263.

[B193] Knapp S (1989) A revision of the *Solanum nitidum* group (section Holophylla pro parte): Solanaceae. Bulletin of the British Museum (Natural History). Botany 19: 63–102.

[B194] Knapp S (2001) Is morphology dead in *Solanum* taxonomy? In: van den Berg RG Barendse GWM van der Weerden GM Mariani C (Eds) Solanaceae V: advances in taxonomy and utilization. Nijmegen University Press, Nijmegen, 23–38.

[B195] Knapp S (2002a) Solanum section Geminata (G. Don) Walpers (Solanaceae). Flora Neotropica 84: 1–405.

[B196] Knapp S (2002b) Tobacco to tomatoes: A phylogenetic perspective on fruit diversity in the Solanaceae. Journal of Experimental Botany 53(377): 2001–2022. 10.1093/jxb/erf068 12324525

[B197] Knapp S (2007) Lectotypification of Cavanilles’ names in *Solanum* (Solanaceae). Anales del Jardin Botanico de Madrid 64(2): 195–203. 10.3989/ajbm.2007.v64.i2.175

[B198] Knapp S (2008) Species concepts and floras: What are species for? Biological Journal of the Linnean Society. Linnean Society of London 95(1): 17–25. 10.1111/j.1095-8312.2008.01090.x

[B199] Knapp S (2013) A revision of the Dulcamaroid clade of *Solanum* L. (Solanaceae). PhytoKeys 22(0): 1–432. 10.3897/phytokeys.22.4041 PMC3689140 23794937

[B200] Knapp S (2018) *Solanum pimpinellifolium* ‒new for the alien flora of Austria, with comments on Austrian records of *S. triflorum* and *S. nitidibaccatum.* Neilreichia 9: 49–53.

[B201] Knapp S Barboza GE Romero MV Vignoli-Silva M Giacomin LL Stehmann JR (2015) Identification and lectotypification of the Solanaceae from Vellozo’s *Flora Fluminensis.* Taxon 64(4): 822–836. 10.12705/644.14

[B202] Knapp S Barboza GE Särkinen T (2017) (2546–2547) Proposals to reject the name *Solanum rubrum* and to conserve the name *S. alatum* with a conserved type (*Solanaceae*). Taxon 66(4): 988–989. 10.12705/664.21

[B203] Knapp S Lamas G Nic Lughadha E Novarino G (2004) Stability or stasis in the names of organisms: The evolving Codes of nomenclature. Philosophical Transactions of the Royal Society, series B. Biological Sciences 359(1444): 611–622. 10.1098/rstb.2003.1445 15253348 PMC1693349

[B204] Knapp S Vorontsova MS (2016) A revision of the “African Non-Spiny” Clade of *Solanum* L. (*Solanum* sections *Afrosolanum* Bitter, *Benderianum* Bitter, *Lemurisolanum* Bitter, *Lyciosolanum* Bitter, *Macronesiotes* Bitter, and *Quadrangulare* Bitter: Solanaceae). PhytoKeys 66: 1–142. 10.3897/phytokeys.66.8457 PMC4957028 27489494

[B205] Kukk T Kull T (2005) Eesti taimede levikuatlas. Eesti Maaülikool Póllumajandus-ja Keskkonnainstituut, Tartu.

[B206] Kuntze CEO (1891) Revisio Generum Plantarum, Vol. 2. A. Felix, Leipzig.

[B207] Lamarck J-B (1794) Tableau encyclopédique et méthodique des trois règnes de la nature. Botanique. Vol. 2. Panckoucke, Paris.

[B208] Lebecka R (2008) Host-pathogen interaction between *Phytophthora infestans* and *Solanum nigrum, S. villosum* and *S. scabrum.* European Journal of Plant Pathology 120(3): 233–240. 10.1007/s10658-007-9211-z

[B209] Lebecka R (2009) Inheritance of resistance in *Solanum nigrum* to *Phytophthora infestans*. European Journal of Plant Pathology 124(2): 345–348. 10.1007/s10658-008-9414-y

[B210] Leslie AC (1978) The occurrence of *Solanum nigrum* L.× *S. sarrachoides* Sendtn. in Britain. Watsonia 12: 29–32.

[B211] Lester RN (1997) Typification of nineteen names of African *Solanum* species described by A. Richard and others, including *S. campylacanthum* and *S. panduriforme*. Botanical Journal of the Linnean Society 125: 273–293.

[B212] Lester RN Durrands P (1984) Enzyme treatment as an aid in the study of seed surface structures of *Solanum* species. Annals of Botany 53(1): 129–131. 10.1093/oxfordjournals.aob.a086662

[B213] Lid J Lid DT Elvn R (2005) Norsk flora. Det Norske Samlaget, Oslo.

[B214] Lin CC Lin WC Yang SR Shieh DE (1995) Anti-inflammatory and hepatoprotective effects of *Solanum alatum.* The American Journal of Chinese Medicine 23(01): 65–69. 10.1142/S0192415X95000092 7598093

[B215] Linder HP (2014) The evolution of African plant diversity. Frontiers in Ecology and Evolution 2: 38, 10.3389/evo.2014.00038

[B216] Linder HP Lovett J Mutke JM Borthlott W Jürgens N Rebelo T Küper W (2005) A numerical re-evaluation of the sub-Saharan phytochoria of mainland Africa. Biologiske Skrifter 55: 229–252.

[B217] Linnaeus C (1737) Hortus cliffortianus. Amsterdam.

[B218] Linnaeus C (1753) Species plantarum. L. Salvius, Stockholm.

[B219] Linnaeus C (1767) Systema naturae, 12^th^ edition. Vol. 2 – Regnum vegetabilium. L. Salvius, Stockholm.

[B220] Liou TN (1935) Sur le groupe de *Solanum nigrum*. Contributions from the Institute of Botany. National Academy of Peiping 3: 453–458.

[B221] Lippman ZB Cohen O Alvarez IP Abu-Abied M Pekker I Prana I Eshed Y Zamir D (2008) The making of a compound inflorescence in tomato and related nightshades. PLoS Biology 6(11): e288. 10.1371/journal.pbio.0060288 PMC2586368 19018664

[B222] Lowe RT (1872) Order LXIV. Solanaceae. The Nightshade Family. In: A manual flora of Madeira and the adjacent islands of Porto Santo and the Desertas. Vol. 2, part 1. John Van Voorst, London, 68–113.

[B223] Luckow M (1995) Species concepts: Assumptions, methods and applications. Systematic Botany 20(4): 589–605. 10.2307/2419812

[B224] Magoon ML Ramanujam S Cooper DC (1962) Cytological studies in relation to the origin and differentiation of species in the genus *Solanum* L. Caryologia 15(1): 151–252. 10.1080/00087114.1962.10796057

[B225] Maire R (1928) Contributions à l'étude de la flore de l'Afrique du Nord. Bulletin de la Société des Sciences Naturelles du Maroc 8(4–6): 128–142.

[B226] Mallet J (1995) A species definition for the modern synthesis. Trends in Ecology & Evolution 10(7): 294–299. 10.1016/0169-5347(95)90031-4 21237047

[B227] Manoko MLK (2007) A systematic study of African Solanum L. section Solanum (Solanaceae). PhD Thesis, Radboud University, Nijmegen, The Netherlands, http://dare.ubn.kun.nl/bitstream/2066/30032/1/30032.pdf

[B228] Manoko MLK van den Berg RG Feron RMC van der Weerden GM Mariani C (2007) AFLP markers support separation of *Solanum nodiflorum* from *Solanum americanum* sensu stricto (Solanaceae). Plant Systematics and Evolution 267(1–4): 1–11. 10.1007/s00606-007-0531-4

[B229] Manoko MLK van der Berg RG Feron RMC van der Weerden GM Mariana C (2008) genetic diversity of the African hexaploid species *Solanum scabrum* Mill. and *Solanum nigrum* L. (Solanaceae). Genetic Resources and Crop Evolution 55(3): 409–418. 10.1007/s10722-007-9248-z

[B230] Manoko MLK van der Weerden GM (2004a) *Solanum americanum*. In: Grubben GJH Denton OA (Eds) Plant Resources of Tropical Africa 2. PROTA Foundation Wageningen/CTA Wageningen, Backhuys Publishers, Leiden, the Netherlands, 477–480.

[B231] Manoko MLK van der Weerden GM (2004b) *Solanum villosum*. In: Grubben GJH Denton OA (Eds) Plant Resources of Tropical Africa 2. PROTA Foundation Wageningen/CTA Wageningen, Backhuys Publishers, Leiden, the Netherlands, 503–507.

[B232] Manoko MLK van der Weerden GM van den Berg RG Mariani C (2012) A new tetraploid species of Solanum L. sect. Solanum (Solanaceae) from Tanzania. PhytoKeys 16(0): 65–74. 10.3897/phytokeys.16.2884 PMC3492932 23233812

[B233] Martínez Labarga JM García Guillén E Meliá Vaca D Knapp S (2017) *Solanum chenopodioides* Lam. (Solanaceae) en la ciudad de Madrid. Biodiversidad Virtual News Publicaciones Cientificas (BVnPC) 6(74): 40–47, http://www.biodiversidadvirtual.org/taxofoto/sites/default/files/solanum_chenopodioides_lam._solanaceae_en_la_ciudad_de_madrid.pdf

[B234] Martius CFP (1814) Plantarum horti academici Erlangensis enumeratio. Hilpertianis, Erlangen.

[B235] Mathé I Jr Mathé I Sr Mai H (1980) Studies on the Morella section of Solanum genus. VI Variation in the solasodine production in stands of various stages of development of *Solanum nigrum* L. during the vegetative period. Herba Hungarica 18: 143–150.

[B236] Matthioli PA (1544) Di Pedacio Dioscoride Anazarbeo libri cinque della histori, et materia medicinale trodotti in lingua uolgare Italiana. N. de Bascarinin, Venice.

[B237] Mayr E (1982) The growth of biological thought. Harvard University Press, Cambridge.

[B238] McClintock D (1977) J.E. Lousley and plants alien in the British Isles. Watsonia 11: 287–290.

[B239] McNeill J (2014) Holotype specimens and type citations: General issues. Taxon 63(5): 1112–1113. 10.12705/635.7

[B240] McNeill J Barrie FR Buck R Demoulin V Greuter W Hawksworth DL Herendeen PS Knapp S Marhold K Prado J Prud’homme van Reine WF Smith GF Weirsema JH Turland NJ (2012) International Code of Nomenclature for algae, fungi, and plants (Melbourne Code) – Regnum Vegetabile 154. Koelz Scientific Books, Königstein.

[B241] McVaugh R (1956) Edward Palmer: plant explorer of the American West. Norman, University of Oklahoma Press.

[B242] Melamed Y Kislev ME Geffen E Lev-Yadun S Goren-Inbar N (2016) The plant component of an Acheulian diet at Gesher Benot Ya’aqov, Israel. Proceedings of the National Academy of Sciences of the United States of America 113(51): 14674–14679. 10.1073/pnas.1607872113 27930293 PMC5187744

[B243] Melo CAF Martins MIG Oliveira MBM Benko-Iseppon AM Carvalho R (2011) Karyotype analysis for diploid and polyploid species of the *Solanum* L. Plant Systematics and Evolution 293(1–4): 227–235. 10.1007/s00606-011-0434-2

[B244] Meyer GFW (1818) Primitiae florae essequeboensis. H. Dietrich, Göttingen.

[B245] Miller P (1768) The gardeners’ dictionary, 8^th^ edition. Printed for the author, London.

[B246] Miquel FAW (1857) Solaneae. In: Flora van Nederlandsch Indie vol. 2. C.G. van der Post, Amsterdam, 633–672.

[B247] Moat J (2007) Conservation assessment tools extension for ArcView 3.x, version 1.2. GIS Unit, Royal Botanic Gardens, Kew. http://www.rbgkew.org.uk/cats

[B248] Morton CV (1976) A revision of the Argentine species of *Solanum*. Academia Nacional de Ciencias, Córdoba.

[B249] Morton JK (1993) Chromosome numbers and polyploidy in the flora of Cameroon Mountain. Opera Botanica 121: 159–172.

[B250] Moscone EA (1992) Estudios sobre cromosomas meióticos en Solanaceae de Argentina. Darwiniana 31: 261–297.

[B251] Mossberg B Stenberg L Karlsson T (2003) Den nya Nordiska floran. Wahlstrom & Widstrand, Stockholm.

[B252] Moyetta NR Stiefkens LB Bernardello G (2013) Karyotypes of South American species of the Morelloid and Dulcamaroid clades (*Solanum*, Solanaceae). Caryologia 66(4): 333–345. 10.1080/00087114.2013.855389

[B253] Müller H (1883) The fertilization of flowers. MacMillan, London.

[B254] Nakamura M (1935) Preliminary note on the polyploidy in *Solanum nigrum* Linn. Journal of the Society for Tropical Agriculture 7: 255–257.

[B255] Nakamura M (1937) Cyto-genetical studies on the genus *Solanum*. I. Autopolyploidy of *Solanum nigrum* Linn. Cytologia Fujii Jubilae 1(1): 57–68. 10.1508/cytologia.FujiiJubilaei.57

[B256] Nandhini T Paramaguru P (2014) Evaluating the taxonomic status of *Solanum nigrum* L. using flow cytometry and DNA barcoding technique. Journal of Spices and Aromatic Crops 23: 233–242.

[B257] Natkevičaitė-Ivanauskienė M (1976) Lietuvos T.S.R. Flora V. [Valstybine Politines ir Mokslines Literaturos Leidykla, Vilnius.]

[B258] Ndamukong KJ Ntomifor NN Mbuh J Atemnkeng AF Akam MT (2006) Molluscidal activity of some Cameroonian plants of *Bulinus* species. East African Medical Journal 8393: 102–109. 10.4314/eamj.v83i3.9405 16771107

[B259] Nee M (1999) Synopsis of *Solanum* in the New World. In: Nee M Symon DE Lester RN Jessop JP (Eds) Solanaceae IV: advances in biology and utilization. Royal Botanic Gardens Kew, Richmond, 285–333.

[B260] Nees van Esenbeck CG (1837) Monograph of East Indian Solaneae. Transactions of the Linnean Society of London 17(1): 37–82. 10.1111/j.1095-8339.1834.tb00017.x

[B261] Nees van Esenbeck CG (1843) Solanaceae F.J.F. Meyen’s Beitrage zur Botanik gesammelt auf einer Reise um die Erde. Novorum Actorum Academiae Caesareae Leopoldino-Carolinae Naturea Curiosum 19(Suppl. 1): 383–393.

[B262] Noltie HJ (2005) The Botany of Robert Wight – Regnum Vegetabile 145. A.R.G. Gantner, 579 pp.

[B263] Nzikou JM Mvoula-Tsieri M Matos L Batouba E Ngakegni-Limbili AC Linder M Desobry S (2007) *Solanum nigrum* L. seeds as an alternative source of edible lipids and nutriment in Congo Brazzaville. Journal of Applied Sciences (Faisalabad) 7: 1107–1115. 10.3923/jas.2007.1107.1115

[B264] Obare BA Yole D Nonoh J Lwande W (2016) Evaluation of cercaricidal and miracicidal activity of selected plant extracts agains larval stages of *Schistosoma mansoni*. Journal of Natural Sciences Research 6: 24–31.

[B265] Ogg Jr AG Rogers BS Schilling EE (1981) Characterization of black nightshade (*Solanum nigrum*) and related species in the United States. Weed Science 29: 27–32.

[B266] Ojiewo CO Agong SG Murakami K Masuda M (2006) Chromosome duplication and ploidy level determination in African nightshade *Solanum villosum* Miller. The Journal of Horticultural Science & Biotechnology 81(2): 183–188. 10.1080/14620316.2006.11512048

[B267] Ojiewo CO Mbwambo O Swai I Samali S Tilya MS Mnzava RN Mrosso L Minja R Oluoch M (2013) Selection, evaluation and release of varieties from genetically diverse African nightshade germplasm. International Journal of Plant Breeding 7: 76–89.

[B268] Ojiewo CO Murakami K Masinde PW Agong SG (2007) Polyploidy breeding of African nightshade (Solanum section Solanum). International Journal of Plant Breeding 1: 10–21. http://www.globalsciencebooks.info/Online/GSBOnline/images/0706/IJPB_1(1)/IJPB_1(1)10-21o.pdf

[B269] Olet EA (2004) Taxonomy of Solanum L. section Solanum in Uganda. PhD thesis, Agricultural University of Norway, Ås.

[B270] Olet EA Arnstein K Heun M (2011) Amplified fragment length polymorphisms (AFLPs) analysis of species of Solanum section Solanum (Solanaceae) from Uganda. African Journal of Biotechnology 10: 6387–6395.

[B271] Olet EA Heun M Lye KA (2005) African crop or poisonous nightshade: The enigma of poisonous or edible black nightshade solved. African Journal of Ecology 43(2): 158–161. 10.1111/j.1365-2028.2005.00556.x

[B272] Olet EA Heun M Lye KA (2006) A new subspecies of *Solanum scabrum* Miller found in Uganda. Novon 16(4): 508–511. 10.3417/1055-3177(2006)16[508:ANSOSS]2.0.CO;2

[B273] Olet EA Lye KA Stedje B (2015) Crossing relationships and chromosome numbers of Solanum section Solanum in Uganda. Nordic Journal of Botany 33(4): 472–483. 10.1111/njb.00592

[B274] Opiz PM (1843) Deutschlands Nachtschattenarten. In: Berchtold F, Opiz PM (Eds) Oekonomisch-technische Flora Böhmens vol 3. Thomas Thabor, Annakloster.

[B275] Parr AJ Payne J Eagles J Chapman BT Robins RJ Rhodes MJC (1990) Variation in tropane alkaloid accumulation within the Solanaceae and strategies for its exploitation. Phytochemistry 29(8): 2545–2550. 10.1016/0031-9422(90)85185-I

[B276] Pasquini MW Assogba-Komlan F Vorster I Shackleton CM Abukutsa-Onyango MO (2009) The production of indigenous vegetables in urban and peri-urban agriculture: a comparative analysis of case studies from Benin, Kenya and South Africa. In: Shackleton CM Pasquini MW Drescher AW (Eds) African indigenous vegetables in urban and peri-urban agriculture. Earthscan, London, 177–224.

[B277] Pawłowskiego B (1963) Flora Polska. Rośliny naczyniowe Polski i ziem ościennych, Vol. 10. Państwowe Wydawnictwo Naukowe, Warszawa, Kraków.

[B278] Pendinen G Spooner DM Jiang J Gavrilenko T (2012) Genomic in situ hybridization reveals both auto- and allopolyploid origins of different North and Central American hexaploid potato (Solanum section Petota) species. Genome 55(6): 407–415. 10.1139/g2012-027 22594521

[B279] Peralta IE Spooner DM Knapp S (2008) Taxonomy of wild tomatoes and their relatives (*Solanum* sections *Lycopersicoides, Juglandifolia, Lycopersicon*; Solanaceae). Systematic Botany Monographs 84: 1–186.

[B280] Persoon CH (1805) Synopsis plantarum. vol. 1. C.F. Cramer, Paris and J.G. Cottam, Tubigen.

[B281] Pigatto AGS Blanco CC Mentz LA Soares GLG (2015) Tropane alkaloids and calystegines as chemotaxonomic markers in Solanaceae. Anais da Academia Brasileira de Ciências 87(4): 2139–2149. 10.1590/0001-3765201520140231 26536852

[B282] Pignatti S (1982) Flora d’Italia Vol. 2. Edagricole, Bologna.

[B283] Piso W (1648) Historiae rerum naturalium Brasiliae. Libro VIII: De ipsa regione et illius incolis. Heller, Leiden.

[B284] Poczai P Cernák I Varga I Hyvönen J (2014) Nuclear intron-targeting markers in genetic diversity analysis of black nightshade (Solanum sect. Solanum, Solanaceae) accessions. Genetic Resources and Crop Evolution 61(1): 247–266. 10.1007/s10722-013-0031-z

[B285] Poczai P Hyvönen J (2011) On the origin of *Solanum nigrum*: Can networks help? Molecular Biology Reports 38(2): 1171–1185. 10.1007/s11033-010-0215-y 20574711

[B286] Poczai P Hyvönen J Symon DE (2011) Phylogeny of kangaroo apples (Solanum subg. Archaesolanum, Solanaceae). Molecular Biology Reports 38(8): 5243–5259. 10.1007/s11033-011-0675-8 21258867

[B287] Poczai P Mátyás K Taller J Szabó I (2010) Study of the origin of the rarely cultivated edible *Solanum* species: Morphological and molecular data. Biologia Plantarum 54(3): 543–546. 10.1007/s10535-010-0096-x

[B288] Poczai P Taller J Szabó I (2009) Molecular genetic study on a historical *Solanum* (Solanaceae) herbarium specimen collected by Paulus Kitaibel in the 18^th^ century. Acta Botanica Hungarica 51(3–4): 337–346. 10.1556/ABot.51.2009.3-4.10

[B289] Poczai P Varga I Laos M Cseh A Bell N Valkonen JPT Hyvönen J (2013) Advances in plant gene-targeted and functional markers: A review. Plant Methods 9(1): 6. 10.1186/1746-4811-9-6 PMC3583794 23406322

[B290] Pojarkova AI (1955a) Solanaceae. In: Shishkin BK Bobrov EG (Eds) Flora SSSR. Vol. 22. Academy of Science USSR, Leningrad [St. Petersburg], 1–105.

[B291] Pojarkova AI (1955b) Species novae generis *Solanum* L. ex URSS [in Russian]. Botanicheskie Materialy Gerbariya Botanicheskogo Instituta Imeni V. L. Komarova Akademii Nauk S S S R. Leningrad 17: 328–340.

[B292] Polgár S (1926) *Solanum*-tanulmányok I. Mi a *Solanum Dillenii* Schult? Botanikai Közlemények 23: 30–36.

[B293] Polgár S (1939) Solanum dillenianum. Acta Horti Gotoburgensis 13: 281–288.

[B294] Potawale SE Sinha SD Shroff KK Dhalawat HJ Boraste SS Gandhi SP Tondare AD (2008) *Solanum nigrum* Linn.: A phytopharmacological review. Pharacologyonline 3: 140–163.

[B295] Prado J Hirai RY Moran RC (2015) (046–048) Proposals concerning inadvertent lectotypifications (and neotypifications). Taxon 64: 651.

[B296] Probst R (1928) Dritter Beitrag zur Adventivflora von Solothurn und Umgebung. Mitteilungen der Naturforschenden Gesellschaft Solothurn 8: 41–82.

[B297] Probst R (1938) Sechster Beitrag zur Adventivflora von Solothurn und Umgebung mit Berücksichtigung der Adventiflora von Olten bis Aarau. Mitteilungen der Naturforschenden Gesellschaft Solothurn 12: 3–39.

[B298] Rafinesque CS (1840) Autikon botanikon. Philadephia.

[B299] Randell BR Symon DE (1976) Chromosome numbers in Australian *Solanum* species. Australian Journal of Botany 24(3): 369–379. 10.1071/BT9760369

[B300] Rao GR (1971) Cytology of a pentaploid hybrid and genome analysis in *Solanum nigrum* L. Genetica 42(1): 157–164. 10.1007/BF00154846

[B301] Rao GR (1978) Role of fruit pigments in understanding the inter-relationships and mechanism of evolution of higher chromosomal forms of species of the *Solanum nigrum* L. complex. Acta Botanica Indica 6: 41–47.

[B302] Rao GR Khan R Khan AH (1971) Structural hybridity and genetic system of *Solanum nigrum* complex. Botanical Magazine Tokyo 84(995): 335–338. 10.15281/jplantres1887.84.335

[B303] Rego LNAA da Silva CRM Torezan JMD Gaeta ML Vanzela ALL (2009) Cytotaxonomical study in Brazilian species of *Solanum, Lycianthes* and *Vassobia* (Solanaceae). Plant Systematics and Evolution 279(1–4): 93–102. 10.1007/s00606-009-0149-9

[B304] Rheede von Draakestein H (1689) Hortus Indicus Malabaricus. J. van Someren & J. van Dyck, Amsterdam.

[B305] Richard A (1850) Tentamen florae abyssinicae. A. Bertrand, Paris.

[B306] Rick CM Fobes JF Holle M (1977) Genetic variation in *Lycopersicon pimpinellifolium*: Evidence of evolutionary change in mating systems. Plant Systematics and Evolution 127(2–3): 139–170. 10.1007/BF00984147

[B307] Rick CM Fobes JF Tanksley SD (1979) Evolution of mating systems in *Lycopersicon hirsutum* as deduced from genetic variation in electrophoretic and morphological characters. Plant Systematics and Evolution 132(4): 279–298. 10.1007/BF00982390

[B308] Rick CM Holle M Thorp RW (1978) Rates of cross-pollination in *Lycopersicon pimpinellifolium*: Impact of genetic variation in floral characters. Plant Systematics and Evolution 129(1–2): 31–44. 10.1007/BF00988982

[B309] Rick CM Tanksley SD (1981) Genetic variation in *Solanum pennellii*: Comparison with two other sympatric tomato species. Plant Systematics and Evolution 139(1–2): 11–45. 10.1007/BF00983920

[B310] Riley HT (1855) English translation of Pliny the Elder’s *Naturalis Historia* Book XXIV. HG Bohn, London. http://www.perseus.tufts.edu/hopper/text?doc=urn:cts:latinLit:phi0978.phi001.perseus-eng1:27

[B311] Roe KE (1971) Terminology of hairs in the genus *Solanum*. Taxon 20(4): 501–508. 10.2307/1218251

[B312] Roemer JJ Schultes JA (1819) Systema vegetabilium, 15^th^ edition [bis]. Vol. 4. J.G. Cotta, Stuttgart.

[B313] Rogers BS Ogg AG (1981) Biology of weeds in the *Solanum nigrum* complex (Solanum section Solanum) in North America. US Department of Agriculture Science and Education Administration, Publ. ARM-W 23: 1–30.

[B314] Ronoh R Ekhuya NA Linde M Winkelmann T Abukutsa-Onyango M Dinssa FF Debener T (2017) African nightshades: Genetic, biochemical and metabolite diversity of an underutilised indigenous leafy vegetable and its potential for plant breeding. The Journal of Horticultural Science & Biotechnology. 10.1080/14620316.2017.1358112

[B315] Roth AW (1800) Catalecta Botanica. fasc. 2. J.F. Gleditsch, Leipzig.

[B316] Rottensteiner WK (2015) Exkursionsflora für Istrien. Verlag des Naturwissenschaftlichen Vereins für Kärnten, Klagenfurt.

[B317] Roxburgh W (1824) Flora indica, or descriptions of Indian plants. Vol. 2. Mission Press, Serampore.

[B318] Rumphius [Rumpf] GE (1750) Herbarium amboinense. Vol. 6. F. Changuion & H. Uytwerf, Amsterdam.

[B319] Rusby HH (1907) An enumeration of the plants collected in Bolivia by Miguel Bang With descriptions of new genera and species. Part 4. Bulletin of the New York Botanical Garden 4: 309–470.

[B320] Saarisalo-Taubert A (1967) Adventive *Solanum* species of the group Morella in Eastern Fennoscandia. Annales Botanici Fennici 4: 87–94.

[B321] Sabnis SD Bhatt RP (1972) A new species of *Solanum* Linn. from Gujarat, India. Bulletin of the Botanical Survey of India 12: 258–260.

[B322] Särkinen T Barboza GE Knapp S (2015a) True Black nightshades: Phylogeny and delimitation of the Morelloid clade of *Solanum*. Taxon 64(5): 945–958. 10.12705/645.5

[B323] Särkinen T Baden M Gonzáles P Cueva M Giacomin LL Spooner DM Simon R Juárez H Nina P Molina J Knapp S (2015b) Listado anotado de *Solanum* L. (Solanaceae) en el Perú. Revista Peruana de Biología 22(1): 3–62. 10.15381/rpb.v22i1.11121 [Annotated checklist of Solanum L. (Solanaceae) for Peru]

[B324] Särkinen T Knapp S (2016) Two new non-spiny *Solanum* (Solanaceae) from the Gran Chaco Americano and a key for the herbaceous glandular-pubescent solanums from the region. PhytoKeys 74: 19–33. 10.3897/phytokeys.74.10159 PMC5234547 28127235

[B325] Särkinen T Olmstead RG Bohs L Knapp S (2013) A phylogenetic framework for evolutionary study of the nightshades (Solanaceae): A dated 1000-tip tree. BMC Evolutionary Biology 13(1): 214. 10.1186/1471-2148-13-214 PMC3850475 24283922

[B326] Schilling EE (1981) Systematics of Solanum sect. Solanum (Solanaceae) in North America. Systematic Botany 6(2): 172–185. 10.2307/2418547

[B327] Schilling EE Andersen RN (1990) The black nightshades (Solanum section Solanum) of the Indian subcontinent. Botanical Journal of the Linnean Society 102(3): 253–259. 10.1111/j.1095-8339.1990.tb01879.x

[B328] Schilling EE Jr Heiser CB Jr (1979) Crossing relationships among diploid species of the *Solanum nigrum* complex in North America. American Journal of Botany 66(6): 709–716. 10.1002/j.1537-2197.1979.tb06275.x

[B329] Schilling EE Ma Q-S Andersen RN (1992) Common names and species identification in black nightshades, Solanum sect. Solanum (Solanaceae). Economic Botany 46: 223–225. 10.1007/BF02930641

[B330] Schippers RR (2000) African indigenous vegetables. An overview of the cultivated species. Natural Resources Institute/ACP-EU Technical Centre of Agricultural and Rural Cooperation, Chatham, UK.

[B331] Schmidt DD Kessler A Kessler D Schmidt S Lim M Gase K Baldwin IT (2004) *Solanum nigrum*: A model ecological expression system and its tools. Molecular Ecology 13(5): 981–995. 10.1111/j.1365-294X.2004.02111.x 15078438

[B332] Schmidt S Baldwin IT (2006) Systemin in *Solanum nigrum*. The tomato-homologus polypeptide does not mediate direct defense responses. Plant Physiology 142(4): 1751–1758. 10.1104/pp.106.089755 17071641 PMC1676034

[B333] Schultes JA (1814) CLXIV. Nachtschatten (*Solanum*) Morelle. In: Österreich Flora: ein Handbuch auf Botanischen Excursionen vol. 2. C. Schaumberg & Compagnie, Vienna, 392–395.

[B334] Seithe A (1962) Die Haararten der Gattung *Solanum* L. und ihre taxonomische Verwertung. Botanische Jahrbücher für Systematik, Pflanzengeschichte und Pflanzengeographie 81: 261–336.

[B335] Seithe A (1979) Hair types as taxonomic characters in *Solanum*. In: Hawkes JG Lester RN Skelding AD (Eds) The biology and taxonomy of the Solanaceae. Academic Press, London, 307–319.

[B336] Sendtner (1846) Solanaceae et Cestrineae. Flora Brasiliensis 10: 1–231.

[B337] Sharma SC Chand R Sati OP Sharma AK (1983) Oligofurostanosides from *Solanum nigrum.* Phytochemistry 22(5): 1241–1244. 10.1016/0031-9422(83)80231-3

[B338] Singh SP Raghavendra K Singh RK Subbarao SK (2001) Studies on larvicidal properties of leaf extract of *Solanum nigrum* Linn. (family Solanaceae). Current Science 81: 1529–1530.

[B339] Śliwka J Tomczynska I Chmielarz M Stefanczyk E Lebecka R Zimnoch-Guzowska E (2012) Resistance to *Phytophthora infestans* in three *Solanum nigrum* F_3_ families. Plant Breeding and Seed Science 66: 63–73.

[B340] Smith GF Figueiredo E (2011) Responsible species description: A change of attitude is needed to facilitate and improve access to biological material. Taxon 60: 1549–1551.

[B341] Smith TB Holder K Girman D O’Keefe K Larison B Chan Y (2000) Comparative avian phylogeography of Cameroon and Equatorial Guinea mountains: Implications for conservation. Molecular Ecology 9(10): 1505–1516. 10.1046/j.1365-294x.2000.01032.x 11050546

[B342] Soria J Heiser CB (1959) The Garden Huckleberry and the Sunberry. Baileya 7: 33–35.

[B343] Soria J Heiser CB (1961) A statistical study of relationships of certain species of the *Solanum nigrum* complex. Economic Botany 15(3): 245–255. 10.1007/BF02862165

[B344] Sosef MSM Dauby G Blach-Overgaard A van der Burgt X Catarino L Damen T Deblauwe V Dessein S Dransfield J Droissart V Duarte MC Engledow H Fadeur G Figueira R Gereau RE Hardy OJ Harris DJ de Heij J Janssens S Klomberg Y Ley AC Mackinder BA Meerts P van de Poel JL Sonké B Stévart T Stoffelen P Svenning J-C Sepulchre P Zaiss R Wieringa JJ Couvreur TLP (2017) Exploring the floristic diversity of tropical Africa. BMC Biology 15(1): 15. 10.1186/s12915-017-0356-8 PMC5339970 28264718

[B345] Souèges R (1907) Développement et structure de tégument seminal chez les Solanacées. Annales des Sciences Naturelles, Botanique, séries 9(6): 1–124.

[B346] Spooner DM Ghislain M Simon R Jansky SH Gavrilenko T (2014) Systematics, diversity, genetics, and evolution of wild and cultivated potatoes. Botanical Review 80(4): 283–383. 10.1007/s12229-014-9146-y

[B347] Spooner DM Knapp S (2013) *Solanum stipuloideum* Rusby, the correct name for *Solanum circaeifolium* Bitter. American Journal of Potato Research 90(4): 301–305. 10.1007/s12230-013-9304-5

[B348] Spooner DM Núñez J Trujillo G Herrera M de R Guzmán F Ghislain M (2007) Extensive simple sequence repeat genotyping of potato landraces supports a major reevaluation of their gene poll structure and classification. Proceedings of the National Academy of Sciences, USA 104: 19398–19403. 10.1073/pnas.0709796104 PMC2148301 18042704

[B349] Sprengel C (1793) Das entdeckte Geheimniss der Natur im bau und in der Befruchtung der Blumen. Bei Friedrich Vieweg dem aeltern, Berlin.

[B350] Sprengel C (1825) Systema vegetabilium (16^th^ edn). Vol. 1. Dietrich, Göttingen.

[B351] Stace C (2010) New flora of the British Isles (3^rd^ edn). Cambridge University Press, Cambridge.

[B352] Stace C Preston CD Pearman DA (2015) Hybrid flora of the British Isles. Botanical Society of Britain and Ireland, Bristol.

[B353] Stearn WC (1974) Miller’s *Gardener’s dictionary* and its abridgement. Journal for the Society for the Bibliography of Natural History 7(1): 125–141. 10.3366/jsbnh.1974.7.PART_1.125

[B354] Stebbins GL Paddock EF (1949) The *Solanum nigrum* complex in Pacific North America. Madrono 10: 70–80.

[B355] Stegelmeier BL Molyneux RJ Asano N Watson AA Nash RJ (2008) The comparative pathology of the glycosidase inhibitors swainsonine, castanospermine, and calystegines A3, B2, and C1 in mice. Toxicologic Pathology 36(5): 651–659. 10.1177/0192623308317420 18497426

[B356] Stern S Bohs L Giacomin LL Stehmann JR Knapp S (2013) A revision of Solanum section Gonatotrichum Bitter (Solanaceae). Systematic Botany 38(2): 471–496. 10.1600/036364413X666624

[B357] Stern SR Agra M de F Bohs L (2011) Molecular delimitation of clades within New World species of the “spiny solanums” (Solanum subgenus Leptostemonum). Taxon 60: 1429–1441. 10.1002/tax.605018 PMC7169808 32327851

[B358] Strickland-Constable R Schneider H Ansell SW Russell SJ Knapp S (2008) Species identity in the *Solanum bahamense* species group (Solanaceae, Solanum subgenus Leptostemonum). Taxon 59: 209–226.

[B359] Subils R (1989) Sobre el indumento de *Solanum triflorum* (Solanaceae) y su importancia taxonómica. Kurtziana 21: 111–152.

[B360] Sultana SS Alam SS (2007) Differential fluorescent chromosome banding of *Solanum nigrum* L and *Solanum villosum* L. from Bangladesh. Cytologia 72(2): 213–219. 10.1508/cytologia.72.213

[B361] Sumner J (2000) The natural history of medicinal plants. Timber Press, Portland, Oregon.

[B362] Switzer CM Combes SA (2017) Bumblebee sonication behaviour changes with plant species and environmental conditions. Apidologie 48(2): 223–233. 10.1007/s13592-016-0467-1

[B363] Symon DE (1979) Sex forms in *Solanum* (Solanaceae) and the role of pollen collecting insects. In: Hawkes JD Lester RN Skelding AD (Eds) The biology and taxonomy of the Solanaceae. Academic Press, London, 385–397.

[B364] Symon DE (1981) A revision of the genus *Solanum* in Australia. Journal of the Adelaide Botanic Gardens 4: 1–367.

[B365] Symon DE (1985) The Solanaceae of New Guinea. Journal of the Adelaide Botanic Gardens 8: 1–171.

[B366] Symon DE (1994) Kangaroo apples; Solanum sect. Archaeosolanum. Published by the author, Adelaide.

[B367] Tamboia T Cipollini ML Levey DJ (1996) An evaluation of vertebrate seed dispersal syndromes in four species of black nightshade (Solanum sect. Solanum). Oecologia 107(4): 522–532. 10.1007/BF00333944 28307396

[B368] Tandon SL (1974) *Solanum nigrum* L. In: Hutchinson J (Ed.) Evolutionary studies in world crops: Diversity and change in the Indian subcontinent. Cambridge University Press, London, 109–117.

[B369] Tandon SL Rao GR (1964) Cytogenetical investigations in relation to the mechanism of evolution in hexaploid *Solanum nigrum* L. Nature 201(4926): 1348–1349. 10.1038/2011348b0

[B370] Tandon SL Rao GR (1966a) Interrelationship within the *Solanum nigrum* complex. Indian journal of Genetics and Plant Breedings 26(2): 130–141.

[B371] Tandon SL Rao GR (1966b) Biosystematics and origin of the Indian tetraploid *Solanum nigrum* L. Current Science 35: 524–525.

[B372] Tétényi P (1987) A chemotaxonomic classification of the Solanaceae. Annals of the Missouri Botanical Garden 74(3): 600–608. 10.2307/2399328

[B373] Thellung A (1927) Qu’est-ce que le *Solanum dillenii* Schultes? Reports of the Botanical Society and Exchange Club of the British Isles 8: 186–191.

[B374] Timerman D Green DF Urzay J Ackerman JD (2014) Turbulence-induced resonance vibrations cause pollen release in wind-pollinated *Plantago lanceolata* L. (Plantaginaceae). Journal of the Royal Society, Interface 11(101): 20140866. 10.1098/rsif.2014.0866 PMC4223907 25297315

[B375] Tokunaga K (1933) Studies on the chromosome numbers of some species in Solanaceae. Japanese Journal of Genetics 9(4): 231–238. 10.1266/jjg.9.231

[B376] van Averbeke W Tshikalange TE Juma KA (2007) The commodity systems of Brassica rapa L. subsp. chinensis and *Solanum retroflexum* Dun. in Vhembe, Limpopo province, South Africa. Water S.A. 33: 349–354.

[B377] van der Walt E van Schalkwyk A Berger DK (2008) Genetic relationships between South African *Solanum retroflexum* and other related species using partial 18S sequencing. South African Journal of Botany 74(2): 391. 10.1016/j.sajb.2008.01.162

[B378] van Ooststroom SJ (1966) Een nieuwe variëteit van *Solanum triflorum* Nutt. Gorteria (Leiden) 3: 90–91.

[B379] van Steenis CGGJ (2006) The mountain flora of Java. 2^nd^ edition. Koninklijke Brill, Leiden.

[B380] Vartak VD (1981) Observations on wild edible plants from the hilly regions of Maharastra and Goa: resume and future prospects. In: Jain SK (Ed.) Glimpses of Indian Ethnobotany. Oxford & IBH, New Delhi, 261–271.

[B381] Ved DK Sureshchandra ST Barve V Srinivas V Sangeetha S Ravikumar K Kartikeyan R Kulkarni V Kumar AS Venugopal SN Somashekhar BS Sumanth MV Noorunissa Begum Sugandhi Rani Surekha KV Desale N (2016) FRLHT’s ENVIS Centre on Medicinal Plants, Bangalore. http://envis.frlht.org [accessed 21 August 2017]

[B382] Venkateswarlu J Bhiravamurty PV (1969) Morphology of the pachytene chromosomes of the Indian diploid *Solanum nigrum* L. Genetica 40(1): 407–412. 10.1007/BF01787366

[B383] Venkateswarlu J Rao MK (1969) Chromosome numerical mosaicism in some hybrids of the *Solanum nigrum* complex. Genetica 40(1): 400–406. 10.1007/BF01787365

[B384] Venkateswarlu J Rao MK (1972) Breeding system, crossability relationships and isolating mechanisms in the *Solanum nigrum* complex. Cytologia 37(2): 317–326. 10.1508/cytologia.37.317

[B385] Viljoen E (2011) Morphology and genetic relationships in members of the *Solanum nigrum* L. complex used for jam production in the Highveld of South Africa. MSc thesis, Biotechnology, University of Pretoria, Pretoria, 1–242.

[B386] Vivero JL Kelbessa E Demissew S (2006) Progress on the Red List of plants of Ethiopia and Eritrea: conservation and biogeography of endemic flowering taxa. In: Ghazanfar SA Beentje HJ (Eds) Taxonomy and ecology of African plants, their conservation and sustainable use. Royal Botanic Gardens, Kew, Richmond, 761–778.

[B387] Vogel R Gutzwiller A (1993) De la morelle noire dans l'ensilage de maïs: Prudence! Revue Suisee d’Agriculture 25: 315–323.

[B388] Vorontsova MS Knapp S (2010) Lost Berlin (B) types of *Solanum* (Solanaceae) – found in Göttingen (GOET). Taxon 59: 1585–1601.

[B389] Voss KA Chamberlain WJ Brennecke LH (1993) Subchronic toxicity study of eastern black nightshade (*Solanum ptychanthum*) berries in Sprague-Dawley rats. Journal of Food Safety 13(2): 91–97. 10.1111/j.1745-4565.1993.tb00097.x

[B390] Wahaj SA Levey DJ Sanders AK Cipollini ML (1998) Control of gut retention time by secondary metabolites in ripe *Solanum* fruits. Ecology 79(7): 2309–2319. 10.1890/0012-9658(1998)079[2309:COGRTB]2.0.CO;2

[B391] Walsh NG Entwisle TJ (1999) Flora of Victoria, Vol. 4 Dicotyledons: Cornaceae to Asteraceae. Inkata Press, Melbourne.

[B392] Warrier PK Nambiar VPK Ramankutty C Arya Vaidya Saya (1996) Indian medicinal plants. Vol. 5. Orient Longman Ltd., Hyderabad.

[B393] Webb CJ Sykes WR Garnock-Jones PJ (1988) Flora of New Zealand, Vol. 4: Naturalised pteridophytes, gymnosperms, dicotyledons. Botany Division, DSIR, Christchruch.

[B394] Weber CA (1928) Georg Bitter. Berichte der Deutschen Botanischen Gesellschaft 46: 148–156. [obituary]

[B395] Weed Management Act 1999 (2000) Weeds index – Declared weeds. Government of Tasmania, http://dpipwe.tas.gov.au/invasive-species/weeds/weeds-index/declared-weeds-index [accessed 10 July 2017]

[B396] Weese T Bohs L (2007) A three-gene phylogeny of the genus *Solanum* (Solanaceae). Systematic Botany 33(2): 445–463. 10.1600/036364407781179671

[B397] Weinmann JA (1824) Elenchus plantarum horti imperialis Pawlowskiensis et agri Petropolitani. Ex Typographia Orphanotrophii, St. Petersburg.

[B398] Welch KD Panter KE Gardner DR Stegelmeier BL (2012) The good and the bad of poisonous plants: An introduction to the USDA-ARS Poisonous Plants Research Laboratory. Journal of Medical Toxicology; Official Journal of the American College of Medical Toxicology 8(2): 153–159. 10.1007/s13181-012-0215-5 22367563 PMC3550245

[B399] Wessely I (1960) Die mitteleuropaischen Sippen der Gattung *Solanum* Sektion *Morella*. Feddes Repertorium 63: 290–321.

[B400] von Wettstein R (1895) Solanaceae. In: Engler A, Prantl K (Eds) Die Natürlichen Pflanzenfamilien 4(3b): 4–38.

[B401] White F (1981) The history of the Afromontane archipelago and the scientific need for its conservation. African Journal of Ecology 19(1–2): 33–54. 10.1111/j.1365-2028.1981.tb00651.x

[B402] White F (1983) The vegetation of Africa. UNESCO, Paris.

[B403] White F (1993) The AETFAT chrological classification of Africa: History, methods and applications. Bulletin du Jardin Botanique National de Belgique 62(1/4): 225–281. 10.2307/3668279

[B404] Wight R (1843) Icones Plantarum Indiae Orientalis Vol. 2. Madras, JB Pharoah.

[B405] Willdenow CL (1809) Enumeratio plantarum hortii regii botanici Berolinensis. Taberna Libraria Scholae Realis, Berlin.

[B406] Witek K Jupe F Witek AI Baker D Clark MD Jones JD (2016) Accelerated cloning of a potato late-blight-resistance gene using RenSeq and SMRT sequencing. Nature Biotechnology 34(6): 656–660. 10.1038/nbt.3540 27111721

[B407] Wright JB Hastings DA Jones WB Williams HR (1985) Geologyand Mineral Resources of West Africa. George Allen & Unwin Publishers, London.

[B408] Wu CY Huang SC (1978) Materiae ad Floram Solanorum et Lycianthium Sinensium. Zhiwu Fenlei Xuebao 16: 72–80.

[B409] Xu ZF (2001) Proteinase inhibitor II from *Solanum americanum*: molecular characterization and potential use in generating insect-resistance transgenic vegetables. PhD thesis, University of Hong Kong, Hong Kong.

[B410] Yang R-Y Ojiewo C (2013) African nightshades and African eggplants: Taxonomy, crop management, utilization and phytonutrients. ACS Symposium Series 1127: 137–165. 10.1021/bk-2013-1127.ch011

[B411] Zhang ZY Lu AM D’Arcy WG (1994) Solanaceae. In: Wu ZY Raven PH (Eds) Flora of China, Vol. 17. Science Press Beijing and Missouri Botanical Garden, St. Louis, 300–332.

[B412] Zhu SZ Wang JX Tang YC (2015) Jinhuang bencao, or treatise on wild food plants used for saving famime (translation and annotation by Wang JX, Tang YC; in Chinese). Shanghai gu ji chu ban she, Shanghai.

[B413] Zimnoch-Guzowska E Lebecka R Kryszczuk A Maciejewska U Szczerbakowa A Wielgat B (2003) Resistance to *Phytophthora infestans* in somatic hybrids of *Solanum nigrum* L. and diploid potato. Theoretical and Applied Genetics 107(1): 43–48. 10.1007/s00122-003-1221-4 12835931

