# Supplementary material for: A revision of the Old World Black Nightshades (Morelloid clade of Solanum L., Solanaceae)
Source: PhytoKeys. 2018 Jul 25;(106):1–223. doi: 10.3897/phytokeys.106.21991 (PMC6070582; doi:10.3897/phytokeys.106.21991)
Supplement: Supplementary material 1 — Index to numbered collections [file phytokeys-106-001-s001.docx]

# **Appendix 1. Index to numbered collections**

Only first collectors in collections made by two or more collectors are listed here; pairs of collectors are listed in the Specimens examined sections of species treatments, while groups of three or more are listed as *et al*. Collections by anonymous collectors without date or other identifying features are not listed here. Full collector strings can be found on Solanaceae Source (<http://www.solanaceaesource.org>) or on the NHM Data Portal (doi provided on acceptance).

Abbott, J.R. 16181 (americanum).

Abd El Ghani, M. 3184, 6139 (villosum).

Abdallah, C.S. 1630 (villosum).

Abeid, Y.S. 150, 533 (villosum); 2848 (scabrum).

Aberdeen University Amanus Expedition C1170, D1234 (villosum).

Abrams, L. 2520 (americanum).

Abu-Laila, K. 70-4 (nigrum); 70-2 (villosum).

Acevedo Rodríguez, P. 3140, 4254 (americanum); 4487 (palitans).

Acocks, J.P.H. 21049 (triflorum).

Acosta, C. 126 (palitans).

Acosta-Solís, M. 5351, 6055 (americanum).

Adair, R.J. 3341 (opacum).

Adam, J.G. 18728 (scabrum); 20529 (americanum); 22258 (tarderemotum); 23788 (scabrum); 24671 (tarderemotum).

Adames, P. 577 (scabrum).

Adams, C.D. 6145, 11774 (americanum).

Adams, L.G. 537 (triflorum).

Adamson, R.S. 3225 (retroflexum); 3317 (scabrum).

Addor, E.E. 172 (triflorum).

Adelowokan, M.A. 5 (nigrum).

Aedo, C. 10790 (americanum); 10806 (furcatum); 13543 (nigrum).

Aellen, P. 3 (villosum); 8, 16, 18 (nigrum); 3201, 3202 (pygmaeum); 63975 (nigrum).

Afriastini, J.J. 475 (alpinum).

Agacino, M. 164934 (villosum).

Agha, A.S. NHI-5370 (villosum).

Agnew, A.D.Q. 1227, 1228, 5598 (villosum).

Agra, M.F. 786, 790, 792, 1108, 1110, 1114, 1116, (americanum).

Aguilar M, G. 8184 (americanum).

Aguilar, R. 1744 (americanum).

Aguon, C. 134 (americanum).

Ahart, L. 4830, 5440 (americanum).

Ahles, H.E. 52986, 55038 (nitidibaccatum).

Ahmad, H. NHI-9409 (nigrum).

Ahumada, O. 350 (chenopodioides); 1085 (americanum).

Aitchison, J.E.T. 314, 727 (nigrum); 611 (villosum); 727 (nigrum).

Akeroyd, J.R. 239, 3583 (villosum).

Akhani, H. 11571 (nigrum); 12246 (villosum); 12247, 12252 (nigrum).

Akpabla, G. 2117 (scabrum).

Al Dabbagh NHI-45862 (villosum).

Al Khaisi NHI-42715 (villosum); NHI-45296 (nigrum); NHI-46088, NHI-50505 (villosum); NHI-50616 (nigrum); NHI-51173 (villosum).

Al Khayat NHI-46318 (villosum).

al Rawi, A. NHI-5951, NHI-32404 (nigrum).

al Zebar, A.M. 9272 (villosum).

Albán, J. 10240 (furcatum).

Albee, B. 3177 (triflorum).

Albers, C.C. 61013 (memphiticum); 61067 (tarderemotum); 62349 (villosum).

Albert, R. 286, 721, 973 (villosum).

Albert, V. 96/10, 96/12 (triflorum).

Albrecht, D.E. 384, 483 (furcatum); 514, 517 (opacum); 738 (americanum); 2499 (nitidibaccatum).

Alcock, C.R. 1268, 2099 (retroflexum); 3939 (triflorum); 4454 (americanum); 6808 (triflorum); 8528, 8583 (opacum); 8588, 8594, 8665, 8741 (americanum); 11053 (nitidibaccatum);11266, 11267, 11273 (americanum); 11425 (nigrum).

Alcorn, J.B. 2347 (americanum).

Aldridge, A.E. 962 (nigrum).

Alexande, E. 41, 43, 52 (americanum).

Alexander, J.C.M. 126 (villosum).

Alexiades, M. 850052 (americanum).

Allard, H.A. 22042, 22459 (americanum).

Allen, B.M. 6505 (villosum).

Allen, C.E.F. 724 (tarderemotum).

Allen, G.G. 101 (triflorum).

Allen, P.H. 876 (americanum).

Allorge, L. 2632 (americanum).

Almeida, J. 451, 1495, 1969 (americanum).

Almeida, S.S. de 665 (americanum).

Alonso G, P. 19 (americanum).

Alonso, J.M. 250, 324, 350 (pygmaeum).

Alston, A.H.G. 11212 (villosum); 1289, 11214, 11218, 11268, 17405 (nigrum).

Althofer, G.W. 8582 (opacum).

Álvarez M, D. 303 (americanum).

Álvarez, D. 4038, 8726, 11053 (americanum).

Álvarez, R. 343 (americanum).

Alverson, W.S. 3856 (americanum).

Alves, E.M. 493 (americanum).

Alves, M. EU-26 (villosum).

Am Sharif NHI-47305 (nigrum).

Amaral, A. 418 (americanum).

Ambrish, K. NC-120913 (americanum).

Ambrosetti, J.A. 1474 (triflorum); 1478 (nitidibaccatum).

American Colony Jerusalem 102 (villosum).

Amorim, A. 21 (scabrum);. 8020 (americanum).

Ancuash Atsut, E. 447 (americanum).

Anders 1587 (nigrum).

Anders, O. 3705, 4826, 5138, 5351, 10364, 10426, 10586, 10801 (villosum).

Andersen, J.S. 247 (nigrum).

Anderson, ? 157 (villosum).

Anderson, B. 1920, 2832 (americanum).

Anderson, D. 128b (americanum); 3672 (opacum).

Anderson, D.L. 1336, 1748 (pygmaeum); 2464 (nitidibaccatum); 2867, 3126 (pygmaeum); 3265 (nitidibaccatum); 3271 (triflorum); 3300, 3427 (nitidibaccatum); 3428 (triflorum); 3475 (pygmaeum); 3609 (triflorum).

Anderson, E.A. 3773 (nigrum).

Anderson, E.F. 3836 (nigrum).

Anderson, J. 10, 140 (furcatum).

Anderson, J.R. 254 (nitidibaccatum).

Anderson, J.P. 605 (nigrum).

Anderson, L.C. 17746 (americanum).

Anderson, T. 367, 369, 432 (alpinum); 1018, 1027 (nigrum).

Anderson, W.R. 9935 (americanum).

Andreasen, M.L. 623 (americanum).

Andreata, R. 180 (americanum).

Andrews, C. 658 (nigrum).

Andrews, F.W. A1947 (tarderemotum); 3590 (villosum).

Angus, A. 1528 (scabrum).

Annadale, N. EB-323 (nigrum).

Annels, A.R. 4680 (americanum).

Antío, J.A. 128 (americanum).

Antúnez De Mayolo, K. 81 (furcatum).

Aparecida, M. 2753 (americanum).

Aponte, H. 256 (americanum).

Arakaki, M. 34 (furcatum); 85 (americanum).

Araque M, J. 882 (sarrachoides).

Araquistain, M. 174, 2716 (americanum).

Araújo-M, A. 1977, 3107 (americanum).

Arbo, M.M. 1019, 7920 (americanum).

Archbold, M.E. 937 (americanum); 2545 (villosum).

Archer, W.A. 44, 4197 (americanum); 6836 (triflorum); 7134, 7289 (nitidibaccatum).

Archibald, E. 5905 (chenopodioides).

Archibald, J. 2661, 2778 (villosum).

Archley, J.C. 161 (villosum).

Arenas P, G. 59 (furcatum).

Argent, G. [C.G.] C87-179 (scabrum); 1305 (americanum); PPI-9752 (nigrum); 92248 (americanum).

Argüelles, E. 667, 996, 2876, 3441 (americanum).

Argüelles, J. 122 (americanum).

Ariza E, L. 3621 (chenopodioides).

Armit, W.E.M. 682 (opacum).

Armstrong, J.A. 98 (nigrum).

Arnason, J.T. 17626 (americanum).

Arnay, E. d' 272 (triflorum).

Arnold, H. 10206 (retroflexum).

Arnoldo, M. 1719 (americanum).

Arnow, L. 544a (triflorum); 740 (nitidibaccatum); 3189 (triflorum); 3740 (nitidibaccatum); 4692 (triflorum); 5552 (nitidibaccatum); 6779, 7175 (triflorum).

Aroha, C.M. NC-38461, NC-53202 (villosum).

Arroyo R, F. 018 (furcatum).

Arsène, G. 3334 (americanum); 17348 (triflorum).

Arsène, L. 517/3 (villosum).

Articó, L. 240, 249 (triflorum).

Arvidsson, I. 191 (villosum).

Asplund, E. 1372 (nigrum); 5283, 8941, 15390, 16600, 18053, 19851 (americanum).

Assadi, M. 1690, 1976 (villosum).

Aston, H.I. 2013 (americanum).

Astridge, S. AEE-4106 (americanum).

Aswal, B.S. NC-55377 (villosum).

Atchison, G. 1 (nitidibaccatum); 2 (chenopodioides); 3, 4, 5, 6, 7, IM-8, IM-9 (nigrum); 25 (palitans).

Atha, D.E. 5196 (villosum); 5835, 6869, 7932, 14060 (nigrum).

Atherton, G.E. 301, 387, 669, 861 (villosum).

Atkinson, C.L.W. 35 (americanum).

Atwood, N.D. 13495 (triflorum); 26560, 29082 (americanum).

Aubert, G. d' 205 (chenopodioides).

Aucher-Eloy, P.M.R. 2482 (villosum); 5030 (nigrum); 5031 (villosum).

Auld, B. 120043 (chenopodioides).

Auquier, P. 2721 (tarderemotum).

Autran, E.J.B. 4 (pygmaeum).

Avendaño Reyes, S. 126 (americanum).

Averett, J.E. 1253 (americanum).

Ayala, M.G. 449 (americanum).

Ayanzi, F.B. Edin.5887 (nigrum).

Aye, T.T. 20623 (americanum).

Aylmer, G. 171, 517 (villosum).

Babu, C.R. NC-34679a (nigrum); NC-34679 (americanum).

Backer, C.A. 36563 (alpinum).

Bacon, E. 93 (nigrum).

Bagshawe, A.G. 1121 (tarderemotum).

Bajhang 2009 Expedition 20913010 (nigrum).

Bakari, D.N.G. 144 (americanum).

Baker, C.F. 231 (americanum); 577, 622 (triflorum); 677 (americanum); 1426 (nitidibaccatum).

Baker, G.S. K-346 (memphiticum).

Baker, J.G. 50 (americanum).

Baker, M. 981, 1010, 1019 (furcatum).

Baker, M.L. 1450 (opacum); 1707 (triflorum); 2203 (nitidibaccatum).

Bakhuizen van den Brink, R.C. 6552 (nigrum).

Bakia, A. 603 (americanum).

Balaka, J.L. 1861 (tarderemotum); 2025 (scabrum).

Balakrishnan, N. 996 (americanum); 1177 (villosum); 1177[b] (nigrum).

Balapure, K.M. 537 (nigrum).

Baldrati, I. 3536 (memphiticum).

Balech, E. 49 (pygmaeum).

Balgooy, M.M.J. 3555 (opacum).

Balkwill, K. 6291 (retroflexum); 7163, 9130 (chenopodioides).

Ballantyne, G.H. 418 (nigrum).

Bally, P.R.O. B872 (memphiticum); B1022 (villosum); B-1023, B5736 (memphiticum); 7787 (tarderemotum); B-7788, B8033 (memphiticum); B8235 (tarderemotum); B-8306 (memphiticum); B11056 (villosum); B11292 (tarderemotum); 12482 (nigrum).

Balodi, P. NC-75653 (americanum).

Balsinhas, A. 647 (americanum); 2901 (retroflexum); 3290 (scabrum).

Balvanera L, P. 204, 259 (americanum).

Banerjee, D. 30, 33 (villosum).

Banerjee, R.N. 16180 (americanum).

Banerju, R.N. CNH-16284, CNH-17468 (nigrum).

Bang, M. 539, 1462 (americanum).

Bangerter, E.B. 5024 (nigrum); 5165 (americanum); 5508 (nigrum).

Banlugan, G. 72719 (nigrum).

Banze, A.C. 277 (americanum).

Barbon, E.B. PPI1565, PPI-1832 (americanum); PPI-9142 (nigrum).

Barboza, G.E. 69, 73 (triflorum); 95 (palitans); 190, 197 (nitidibaccatum); 566 (chenopodioides); 605 (palitans); 1579, 2091 (americanum); 2128 (chenopodioides); 2178, 2228 (palitans); 2292 (chenopodioides); 2309 (pygmaeum); 2316, 2317 (chenopodioides); 2345 (triflorum); 2399 (furcatum); 2429 (triflorum); 3058 (americanum); 3462, 3471, 3544, 3570 (palitans); 3668 (nitidibaccatum); 3682, 3687 (triflorum).

Barclay, C. 2342 (nigrum).

Barclay, G.W. 1605, 1607 (americanum); 3309, 3362 (opacum).

Bark, P. 109 (americanum).

Barker, W.R. 51 (nigrum); 8355 (opacum).

Barkla, J.W. M-26 (opacum).

Barkley, F.A. 3709 (villosum); 3716 (nigrum); 33Ir4015, 9042 (villosum).

Barkly, F.O. 13A162 (nigrum).

Barnard, C. 12 (chenopodioides).

Barnes, F.T. 955 (nigrum).

Baron, R. 860, 1744, 1744 (scabrum); 1916 (tarderemotum).

Barros, A.A.M. de 2267, 3504, 5160 (americanum).

Barroso, R.M. 131, 135 (americanum).

Barry, R. 1 (chenopodioides); 9 (americanum).

Barta, T. 2006-20 (nigrum); 2005-34, 53, 121, 2004-138, 175 (villosum); 2003-372 (nigrum); 1151, 1153 (villosum); 1219 (nitidibaccatum); 2181, 2205 (villosum); 2223, 2606, 3064, 3139 (nitidibaccatum); 3155 (villosum); 3198, 3270, 4122 (nitidibaccatum).

Barter, C. 1054 (scabrum).

Barthelat, F. 210, 885 (scabrum).

Bartholomew, B. 6200 (nigrum).

Bartlett, H.H. 479 (americanum); 8569, 14825 (nigrum).

Barunjee, R.N. 18760 (americanum).

Basak, R.K. 101, 109 (villosum).

Bastián, E. 204 (palitans).

Bastos, B.C. 134 (americanum).

Basu, P. ANC-6618 (torvum); ANC-6866 (viarum); ANC-6869 (lasiocarpum); BSHC-8481 (nigrum).

Batanouy, K.H. 2281 (villosum).

Bates, R.J. 689 (opacum); 953 (triflorum); 16832 (americanum); 17501 (nitidibaccatum); 18455, 30341, 30742 (opacum); 41122 (chenopodioides); 51012, 64001 (triflorum).

Bateson, G. 121 (nigrum).

Batianoff, G.N. 205108, 210495, 210654, 210686, 210686, 410011, 630272, 92-0727, 98-1061, 2010485, 2112477 (americanum); 20011234 (opacum); 2001-11110, 2001-11125, 2001-11141 (chenopodioides); 2001-11222 (opacum); 2001-11342, 2001-11356, 2001-12169 (chenopodioides).

Battiscombe, E. K453 (villosum).

Batty, M. 233 (tarderemotum); 274 (villosum); 1100 (americanum).

Baudet, J.C. 487 (tarderemotum).

Bauer, K. 1108 (nigrum).

Baur, R. 102 (retroflexum).

Baxter, P. 4142 (villosum).

Baxter, P.R. 24 (furcatum).

Baylis, G.T.S. 10134 (nigrum).

Bayliss, R.D.A. BRI-B-761, 2994, 3171 (retroflexum); 4594 (scabrum); 5292 (chenopodioides); 5476 (villosum); 6785 (chenopodioides); 7182, 8679 (retroflexum).

Baytop, A. 13628 (villosum).

Beach, K.H. 46 (nigrum); 969 (triflorum); 5410 (villosum).

Beaman, J.H. 6908 (americanum); 8216 (nigrum); 9048, 9079, 10798 (americanum).

Bean, A.R. 4736, 14804 (opacum); 18613 (chenopodioides); 20107, 20521 (opacum); 20752 (americanum); 24318, 24483, 24670, 27321, 28602 (opacum); 29340 (chenopodioides); 30336, 30722 (opacum); 31405 (chenopodioides); 32481 (opacum); 32579, 32684 (chenopodioides).

Bean, P.A. 1366 (retroflexum).

Beauglehole, A.C. 4332, 12438, 21847 (americanum); 31122, 32006, 33110 (opacum); 33117, 33186, 33238 (triflorum); 35137 (opacum); 35771, 36566 (triflorum); 37084, 41769 (*nitidibaccatum*); 50427 (opacum); 50427, 58411, 58411, 62800 (americanum); 62813 (opacum); 67535 (triflorum); 67603 (opacum); 67651A, 67673, 68030 (triflorum); 70231, 70633, 71312, 71472, 71844 (americanum); 76965, 76966, 77426, 77854, 78536 (opacum); 79123 (americanum); 79124 (*nitidibaccatum*); 79189, 79190, 79646, 83205 (americanum).

Beck, S.G. 1390 (palitans).

Beckley, V.A. 670 (villosum).

Beckner, J. 2256 (americanum).

Bedi, R. 667 (villosum).

Beever, R.E. 80-109 (opacum); 80-095 (nigrum).

Béguin, D. 108 (scabrum).

Béguinot, A. 2515, 2516, 2518 (nigrum); 2520, 2521, 23618 (villosum); 23622, 23623 (nigrum); 23624, 23625, 23626 (villosum).

Belibasis, L. de 58 (americanum).

Bell, A.S. 36 (villosum).

Bellingham, P.J. 0142 (nigrum); 0144 (opacum); 637 (chenopodioides); 1840 (opacum).

Bellini, A. 335 (memphiticum); 464 (villosum).

Bello, E. 4945 (americanum).

Beltrán, G.D. 21 (americanum).

Beltrán, H. 205 (furcatum).

Ben, D. van der 185, 1430 (tarderemotum).

Benedetto, P. 49 (villosum).

Benedictis, A. de 389 (villosum).

Bennet, S.S.R. 75, 267 (villosum).

Bennett, C. 8 (nigrum).

Bennett, H.R. 7749 (triflorum).

Bennett, M. 1088 (americanum).

Benoist, R. 62 (americanum); 147 (scabrum).

Benol, D.M. 3 (americanum).

Bensman, R. 177 (americanum).

Benson, J.S. 2791 (chenopodioides).

Bentley, P.S. 55, 169, 306 (americanum).

Bento, M.S. 54 (americanum).

Benusan, J. 51 (villosum).

Berg, C. [F.G.C.] 137 (pygmaeum).

Berg, C.C. P-19836 (americanum).

Bergman, H.F. 2057, 2568 (triflorum).

Berlandier, J.L. 46, 504, 586 (americanum).

Berlin, B. 1682, 1965, 3671 (americanum).

Bernal, R. 2670 (americanum).

Bernardello, G. 476 (pygmaeum).

Bernardello, L.M. 476 (pygmaeum).

Bernoulli, K.G. 2332, 2346, 2379, 2386, 2406 (americanum).

Berro, M.B. 1458, 7206 (sarrachoides).

Bertero, C.L.G. 633 (furcatum); 636, 637 (nitidibaccatum); 1324 (furcatum).

Bertero, D. 638 (furcatum).

Best, E.B. 499 (retroflexum).

Betancourt A, C. 28 (americanum).

Betancur, J.C. 1405 (americanum).

Betche, E. 59 (americanum).

Beusekom, C.F. van 1030 (americanum).

Bhargava, N. ANC-4371 (americanum).

Bhattacharyya, U.C. NC-12853, NC-12986 (villosum); NC-13026 (villosum); NC-13891 (americanum); NC-17976 (villosum); NC-18435 (americanum); NC-21043 (nigrum); NC-37783 (americanum); 37783 (villosum); NC-40707 (americanum); NC-44895 (villosum); NC-45161 (nigrum); NC-45932 (villosum).

Bianor Hermano 957, 958 (villosum).

Bicknell, E.P. 7719 (nigrum).

Biddulph, M.M. 14, 186 (opacum).

Biegel, H.M. 1413 (retroflexum); 1767 (scabrum).

Biel, B. IM-16054 (chenopodioides).

Biganzoli, F. 130 (americanum).

Billiet, F. 5526 (furcatum).

Billot, C. 22 (nigrum); 25 (villosum).

Bingham, M.G. 1544 (scabrum).

Binot, A. 34 (americanum).

Bird, W. 85, 2395 (nigrum).

Bis Ram 347 (nigrum).

Bisby, R. 4036 (chenopodioides).

Bisgel, H.M. 4632 (tarderemotum).

Bishop, W. 178 (chenopodioides).

Bisset, J. 272, 733, 734, 3559 (nigrum).

Biswas, K. 3049 (villosum); 6754 (americanum).

Biswas, M.C. 244 (americanum).

Biurrun, F. 1711, 3211 (nitidibaccatum); 4261 (triflorum); 6086, 6155, 6156 (nitidibaccatum).

Biye, E. 55 (tarderemotum).

Blackmore, S. 210A, 534 (tarderemotum).

Blake, A.L. 175 (triflorum); 186 (nitidibaccatum).

Blake, M.E. 116 (nitidibaccatum).

Blake, S.T. 19542, 21507 (americanum).

Blanchard, M. 46 (furcatum).

Blanchet, J.S. 183, 869 (americanum).

Blankinship, J.W. 408 (triflorum).

Blatter, E. 383 (americanum).

Blaylock, B.J. 2357 (chenopodioides).

Bloembergen, S. 3995 (americanum).

Blom, C.M. 36 (villosum); 1373 (nigrum); 1376 (sarrachoides).

Blom, M. van 176 (villosum).

Blum, K.E. 477 (americanum).

Blumer, J.C. 1768 (nitidibaccatum).

Böcher, T.W. 200 (americanum).

Bocquet, G. 15520, 16910 (nigrum).

Bodner, C.C. 150 (nigrum).

Boelcke, O. 56a (chenopodioides); 3009 (pygmaeum); 4201 (triflorum); 6464 (furcatum); 11974 (pygmaeum); 16088, 16250 (triflorum).

Bogdan, A. 1774 (tarderemotum).

Bohs, L. 2099 (sarrachoides); 2400 (americanum); 2449 (palitans); 2534, 2698 (nigrum); 2796 (palitans); 3062 (triflorum); 3095, 3189a, 3309 (americanum); 3531, 3531 (nigrum); 3561 (opacum); 3652 (americanum).

Boiteau, P.L. 371 (americanum).

Bolus, F. 55 (retroflexum).

Bolus, H. 50 (retroflexum).

Bonilha, C. 313 (americanum).

Booth, E.S. 77 (nigrum).

Booth, R. 2100, 2162 (americanum).

Borden, T.E. 2070 (americanum).

Bornmüller, A. 565 (americanum).

Bornmüller, J. 496, 636 (nigrum); 1798 (villosum).

Boron, B. NC-75048 (nigrum).

Boron, D. NC-76339 (villosum).

Bos, J.J. 3803 (americanum); 7496 (memphiticum).

Bosser, J.M. 926, 8658, 14707 (americanum).

Botany staff National Herb. Iraq NHI-43788, NHI-43803 (villosum).

Botany students 1458 (villosum).

Bouchon, A. 6703 (chenopodioides).

Boufford, D.E. 24134, 29001 (nigrum); 31001 (villosum); 37533 (nigrum).

Boughey, A.S. 109 (scabrum); GC-6933 (pseudospinosum); GC-12516 (tarderemotum).

Boulos, L. 7563, 11195, 14102 (villosum); 14150 (memphiticum); 14223, 14225 (villosum); 15722 (nigrum); 15839, 16400 (villosum); 16730 (nigrum); 17155, 17234, 17419 (villosum); 19046 (nigrum); 19200, 19384, 19457 (villosum); 20191 (nigrum).

Bourgeau, E. 557, 934 (villosum); 2525 (americanum).

Bourne, E.T. 359 (americanum); 405, 472, 3673 (villosum).

Bovey, M. 61 (nigrum).

Bovini, M.G. 1056, 1827, 2207, 2884, 3646 (americanum).

Bowden, L.F. 250, 367, 685, 871 (nigrum).

Bowen, H. 294 (nigrum).

Bowman, E.M. 104 (americanum).

Box, H.E. 649, 1107 (americanum).

Boyd, S. 2434, 2534 (triflorum); 7135 (americanum).

Brace, L.J.K. 1580 (americanum).

Braga, J.M.A. 11, 293, 636, 3056 (americanum);

Bragg, K. 133 (nigrum).

Brain, C.K. 10574 (retroflexum).

Brandbyge, J.S. 42414 (nitidibaccatum).

Brant, A. 1044 (americanum).

Brant, A.E. 4590 (sarrachoides).

Brasil, I. 105 (americanum).

Brass, L.J. 11277 (opacum).

Breckwoldt, A. 1 (chenopodioides).

Breedlove, D.E. 39222, 47646 (americanum).

Breidy, J. LEB-31 (nigrum); LEB-34 (villosum).

Breitung, A.J. 5135 (triflorum).

Brenan, J.P.M. 1449, 12271 (chenopodioides).

Breteler, F.J. MC-39 (pseudospinosum); 11599 (scabrum).

Bretting, P.K. 151 (americanum).

Breyne, H. 2227 (scabrum).

Bridges, T. 400 (nitidibaccatum).

Brierley, E.M. 127, 142 (retroflexum).

Brigada Vásquez 392 (americanum).

Briggs, B.G. 4046 (triflorum); 6964 (chenopodioides).

Brinkman, R. 754 (alpinum).

Brinton-Lee, D. 1002 (villosum).

Bristow, S.C. 17 (nigrum).

Britez, R.M. 2046 (americanum).

Brizuela, A. 1042 (sarrachoides); 1621 (chenopodioides).

Broadway, W.E. 2817, 3126, 3620 (americanum).

Brodhurst-Hill, E. 197 (memphiticum); 328 (villosum).

Brodie, C.J. 192 (nigrum); 1171, 1183 (chenopodioides); 1247, 1346, 1653, 2448, 2520, 2546, 2601, 2669, 2859, 2888, 3040, 3111 (nigrum); 5435, 5435 (chenopodioides).

Brooke, W.M.A. 122 (villosum); 5011 (palitans); 11376 (chenopodioides).

Brotherus, A.H. 767 (nigrum).

Brown, ? 330 (retroflexum).

Brown, E.A. 302, 38020 (palitans).

Brown, H.E. 39 (nitidibaccatum); 932 (americanum).

Brown, K.L. 158 (americanum).

Brown, R. [Bennett #] 2667 (opacum).

Brown, R.C. 6019 (scabrum).

Brown, S. 198 (americanum).

Browne, J.H. 574, 838, 838A, 853, 912 (opacum).

Bruce, E.M. 524 (scabrum).

Bruegmann, M.M. 143 (americanum).

Brumbach, W.C. 8720 (americanum).

Brummit, R.K. 20476 (chenopodioides).

Brummitt, R.K. 298[a] (nigrum); 10323 (retroflexum); 16107, 16264 (tarderemotum); 21567 (chenopodioides).

Brunner, D.R. 859, 1272, 1298 (americanum); 1639 (sarrachoides).

Brunner, S. 108 (scabrum).

Brunt, M. 2056 (americanum); 2490 (villosum).

Bryant, E.G. 1002 (scabrum).

Bryson, C.T. 16966 (americanum).

Buchanan, A.M. 11179 (opacum); 13453 (americanum); 15695 (triflorum); 15827 (*nitidibaccatum*).

Buchanan, R.E. 470 (americanum).

Buchner, P. 83-14-8 (villosum).

Buchtien, O. 1443, 3247, 3890, 4027[b], 4384, 5548, 5550 (americanum).

Buendía S, M.C. 23 (americanum).

Bufton, J. 11 (opacum).

Buller, R.H. REP-19191 (villosum);

Bullock, J.R. 323 (nigrum).

Bunting, G.S. 5572 (americanum).

Burch, D. 677, 1033, 2336, 2379, 2401, 2535, 4307 (americanum).

Burch, D.G. 1033 (americanum).

Burchell, W.J. 78 (villosum); 856 (retroflexum).

Burg, W.J. van der 1177 (scabrum).

Burger, W.C. 374, 378 (villosum); 1483 (memphiticum); 2006 (tarderemotum).

Burgers, ? 1147 (triflorum).

Burkart, A. 5015 (pygmaeum); 6303 (nitidibaccatum); 7693 (chenopodioides); 8276 (nitidibaccatum); 8891, 9062 (pygmaeum); 11176 (palitans); 15574, 17868, 18056, 18214 (pygmaeum); 19874 (nitidibaccatum); 22721, 24253, 24931, 26043, 26360, 28197 (pygmaeum).

Burkhill, H.M. 852, 2876, SF-13939 (americanum).

Burkill, I.H. 119 (americanum); 190 (nigrum); 31067 (villosum).

Burman, A.E. 33 (nigrum).

Burne, E. 86 (villosum).

Burns, T.E. 433, 611, 736 (nigrum).

Búrquez, A. 92-287 (americanum).

Burton, R.M. AE88.133 (nigrum).

Burtt Davy, J. 17658 (retroflexum).

Bush, B.F. 3007, 3882, 3962, 6007, 7096A, 12167 (triflorum).

Bye, R.A. 6067, 7330, 7334, 7346 (americanum); 16922 (chenopodioides); 18175, 18188, 18421, 18436, 19414 (americanum); 26929 (nitidibaccatum).

Cabezas, F. 907 (pseudospinosum); 1095 (scabrum); 1191 (americanum).

Cable, S. 498 (tarderemotum).

Cabrera R, I. 3694 (americanum).

Cabrera, A.L. 5267 (*chenopodioides*); 6065, 11089 (pygmaeum); 12021, 23393, 23993 (palitans); 33163, 33217 (triflorum); 34181 (nitidibaccatum).

Cabrera, E. 11424, 15867, 16124 (americanum).

Cáceres, F. 597, 602, 760, 766a, 858, 2912, 5280 (furcatum).

Calatayud, G. 4350 (americanum).

Calder, C.C. 153 (americanum).

Calvert, J. KEW-84 (triflorum).

Calvo, J. 2457 (nigrum); 2501 (triflorum); 3846 (villosum).

Calzada, J.I. 4914, 7765, 9745 (americanum).

Camargo, C. de 122 (americanum).

Cambridge Congo Expedition 1959 19, 207 (tarderemotum).

Cambridge University Expedition H.91 (nigrum).

Cameron, D.G. 3136 (opacum); 6123, 8123 (americanum).

Cameron, E.K. A-49a, A-118 (americanum); 532 (opacum); 2110 (americanum); 2555 (opacum); 3398, 6217, 6767 (americanum); 7914, 9894, 9895 (opacum); 9896 (americanum); 10948a, 11642 (nigrum); 12506, 12543, 12995, 13237 (americanum); 13822 (nigrum); 14001, 14394 (americanum); 14430 (nigrum); 15205 (americanum); 15216 (opacum); 15371, 15560, 15642 (nigrum); 15643 (opacum); 15792 (nigrum); 16127, 16879, 26836 (americanum).

Cameron, K.J. 13 (tarderemotum).

Campbell, D.G. P22040 (americanum).

Campos, A.L. 20 (americanum).

Campos, J. 2097, 2755, 6474, 6925 (americanum).

Cañigueral C, J. 80 (palitans).

Canning, E.M. 4232, 4284, 5736, 6209 (americanum); 6421, 6549 (triflorum).

Cannon, J.F.M. 3154, 3349, 3526, 4428, 5038 (nigrum).

Cano, A. 2190, 2265, 4735, 5776 (americanum); 7915, 7988 (furcatum); 8688, 12631 (americanum).

Cantero, J.J. 7046 (palitans).

Cantino, P. 729 (nitidibaccatum).

Canton Christian College 373 (americanum); 373 (nigrum); 3383 (americanum); 51926 (nigrum).

Cao Zi-yu 16 (nigrum).

Capparelli, P.N. 257 (americanum).

Carauta, J.P.P. 3227 (americanum).

Carauta, P. 310, 1236 (americanum).

Cárdenas, M. 2053, 4356 (americanum).

Cardoso, J. 134, 194 (americanum).

Cardoso, L.J.T. 79 (americanum).

Carenzo, V. 3636 (chenopodioides).

Carne, W.M. 53 (americanum).

Carolina, M. 17 (americanum).

Carpio, C. del 131 (furcatum).

Carr, C.E. 12985 (opacum).

Carr, C.J. 534 (villosum); 875 (tarderemotum).

Carr, G.W. 10138 (opacum).

Carr, W.R. 30854 (americanum).

Carranza Batista, H. (1)3 (americanum).

Carrasquilla, L. 3024 (americanum).

Carrick, J. 2169 (nigrum); 3279 (opacum).

Carter, H.E. 569 (villosum).

Carter, J.E. 717 (villosum).

Carter, S. 381, 1815 (villosum).

Carter, S.H. 14 (opacum).

Carvalho, B. 16, 26 (americanum).

Carvalho, D.M.G. 5 (americanum).

Carvalho, M.F. do 2544 (pseudospinosum); 3708, 4305 (scabrum); 5522 (americanum).

Carvalho-Silva, M. 1487, 1562 (americanum).

Casey, E.C. 24, 1283 (villosum).

Casey, G.B. 442 (americanum).

Castaño A, N. 1660 (americanum).

Castellanos, A. 820 (pygmaeum); 17860 (chenopodioides).

Castillo, J.J. 1653 (americanum).

Castro, R. 17690 (americanum).

Castroviejo, S. 18239 (villosum).

Catat, L.D.M. 1208 (scabrum).

Caum, E.L. 62 (opacum).

Cavalcanti, A.C.S. 136, 163 (americanum).

Cavalerie, P.J. 3815 (nigrum); 7581 (americanum).

Cazzaniga, M.G. 3400 (chenopodioides).

Cemé, J. 75 (americanum).

Cerón M, C.E. 18909 (americanum).

Cerón, C.E. 15513, 20545, 29283 (americanum).

Cerrate V, E. 1218 (furcatum).

Cerrate, E. 264, 4255 (furcatum); 5064 (americanum).

Chakraborty, P. BSHC-925 (americanum); BSHC-1176 (americanum); BSHC-1934 (americanum); BSHC-2159 (nigrum); ANC-4650 (americanum);

Chakravady, H.L. NHI-30765 (nigrum).

Chan, C. 177, 352, 4924 (americanum).

Chancellor, R.J. 185 (memphiticum).

Chandler, P. 298 (tarderemotum); 405 (memphiticum); 458 (tarderemotum).

Chandra, S. 1304 (americanum).

Chandrabose, M. 28410 (americanum).

Chapman, G.D. 1245 (tarderemotum).

Chapman, J.D. 1219 (tarderemotum); 2914 (scabrum).

Charanpreet 9456 (americanum).

Chardou, C.Z. 87 (americanum).

Charette, L.A. 1509 (nigrum); 1866 (americanum).

Charpin, A. 158 (nigrum); 21516 (sarrachoides).

Charrabanty, P. 3338 (villosum).

Chase, N.C. 2857 (scabrum).

Chávez A, E. 46 (americanum).

Chávez, A. 15 (americanum).

Chávez, E. 46 (americanum).

Chawdhury, H.I. NC-78102 (villosum).

Cheek, M. 3664, 5352 (pseudospinosum); 7117, 7470, 8455 (tarderemotum).

Cheeseman, T.F. 47 (nigrum).

Chemin-Bassler, H. 90 (americanum).

Chen Chih-Hsiung 04625 (americanum).

Chen, L. 110 (nigrum).

Cheo, H.C. 60 (nigrum).

Cheo, T.Y. 60 (nigrum).

Chesterfield, E.A. 101 (americanum); 852, 1869 (opacum).

Chevalier, A. 5518 (scabrum); 8788 (tarderemotum).

Chevallier, L. 73bis (villosum).

Chiang, F. 408 (americanum).

Chiao, C.Y. 18517 (nigrum).

Chiapella, J. 1806 (nitidibaccatum); 1809 (triflorum); 1840 (nitidibaccatum); 1888 (triflorum).

Chiarini, F. 650, 795, 805, 820 (nitidibaccatum); 1088, 1341 (pygmaeum).

Chien, S.S. 198, 395 (nigrum).

Chilton, L. 41, 122 (nigrum).

Chin, S.C. 3713 (americanum).

Ching-I Peng 4506 (americanum); 17879 (opacum).

Chiovenda, E. 889 (hirtulum); 1204 (villosum).

Chipunga, L. 168 (retroflexum).

Chorley, M. 21 (nigrum).

Chow, K.S. 80-300 (nigrum).

Christenhusz, M.J.M. 894, 895 (nigrum); 5827 (villosum).

Christerpherson, E. 976, 2254 (americanum); 2311 (opacum).

Christian, R.W. 1031 (triflorum).

Christian, T. 6, 31 (furcatum).

Christophersen, E. 2324 (nigrum); 2759 (americanum).

Christopherson, E. 2302 (nigrum).

Christy, R.M. 100 (triflorum).

Chu, K.L. 3819 (nigrum).

Chuang, T.I. 3896, 4324 (americanum).

Chun, N.K. 40185 (americanum).

Chung, H.H. 782 (americanum); 1647 (nigrum); 1729 (americanum); 4892 (nigrum); 5621 (villosum); 5974 (nigrum).

Churchill, J.R. 713 (nigrum).

Clarke, C.B. 1827 (villosum); 6653 (americanum); 9011 (nigrum); 22378 (villosum); 26311, 26835 (americanum); 27399, 36740 (nigrum).

Clarke, I.C. 1479, 1544 (chenopodioides); 1682, 1772 (nitidibaccatum); 2714, 2890 (chenopodioides); 2891 (americanum); 4181 (chenopodioides).

Clarkson, J.R. 5498, 9357 (americanum).

Claussen, P. 180, 16846 (americanum).

Clemens, J. 3020 (nigrum); 29744 (americanum); 29774 (nigrum).

Clement, E.J. TB93/723 (americanum).

Clements, F.E. 87 (americanum); 148 (triflorum).

Clevidence, J.P. 7526 (americanum).

Clokey, I.W. 8485 (triflorum).

Clos, E.C. 3840 (chenopodioides).

Close, B. 1094-22 (americanum).

Clover, E.U. 1516 (americanum).

Coburn, F.S. 1263 (americanum).

Cochrane, A.C. 563 (chenopodioides).

Cock, M.J.W. 30 (opacum).

Cocucci, A.A. 399 (triflorum); 890, 993, 2073 (*palitans*); 2589 (nitidibaccatum)3293, 3318, 3348 (palitans).

Cocucci, A.E. 327, 331 (pygmaeum).

Codd, L.E. 7243 (scabrum); 7443 (villosum); 7521 (tarderemotum); 10152 (retroflexum).

Codrington, K. 41 (nigrum).

Coeli, R. 116 (americanum).

Cogollo P, Á. 601 (americanum).

Cogollo, A. 451 (americanum).

Cole, D.T. 923 (scabrum).

Cole, M.M. 6019 (scabrum).

Colenso, W. 57 (opacum).

Coleridge, A. 55 (villosum).

Collenette, C.L. 239 (villosum).

Collenette, I.S. 72-146 (villosum); 1269 (memphiticum); 1542, 1592, 1730, 2675, 2696, 3133, 3309, 3313, 3332, 4349 (villosum); 6212 (nigrum).

Collett, H. 111, 416 (villosum).

Collins, D.J. 1579 (nigrum).

Collins, F.S. 281 (americanum).

Comber, H.F. 736 (pygmaeum).

Commis, R.B. 283 (americanum).

Compton, R.H. 659, 781 (americanum); 25011 (retroflexum).

Comte, F. 4551 (triflorum).

Conklin, H.C. 79580 (americanum).

Conn, B.J. 88, 323, 490 (americanum); 491 (opacum).

Connock, J. 262 (americanum).

Conrad, J. 1998, 2120, 2551 (americanum); 2653 (chenopodioides); 2852 (americanum).

Constable, E.F. 5630 (chenopodioides); 5633 (americanum); 5866 (opacum); 5966, 6161, 6164 (chenopodioides); 6730, 6738 (palitans); 6897 (chenopodioides); 11096 (opacum); 11099 (triflorum).

Constable, H.L.W. 1192 (americanum).

Coode, M.J.E. 1141 (villosum); 4582 (scabrum).

Cook, A.C. 809 (americanum).

Cook, O.F. 460, 533 (americanum).

Cooke, L.A. 46 (villosum).

Cooley, G.R. 2545 (americanum).

Coombs, R.E. 2456 (triflorum).

Cooper, R. 6019 (nigrum).

Cooper, R.E. 3355 (americanum).

Cooper, T. 187 (retroflexum); 528, 554 (tarderemotum).

Cooray, R.G. 68-100301R (americanum).

Copley, B. 1838 (retroflexum).

Corby, W.D.G. 120917 (tarderemotum).

Córdoba, W.A. 422 (americanum).

Corley, R.H.V. 68 (nigrum).

Cornejo Tenorio, G. 3628 (americanum).

Cornes, M. 263 (villosum).

Correa, A. 2991 (americanum).

Correia, M.F. 1136, 1741 (villosum).

Corrick, M.G. 7567 (americanum).

Cortés R, C. 55, 137 (americanum).

Cortés, M.E. 13 (americanum).

Costa, D. 242 (americanum).

Costa-Lima, J.L. 1605, 1837, 1837 (americanum).

Cottam, W.P. 3877 (triflorum).

Cotton Experimental Station 10 (americanum).

Coulter, T. 1231 (americanum).

Coveny, R. 6087 (nigrum); 6088 (chenopodioides); 6089 (palitans); 6397 (nigrum); 6398, 6486 (americanum); 6512 (palitans); 7466 (chenopodioides); 11825 (palitans); 11827 (chenopodioides).

Coveny, R.G. 742 (chenopodioides); 825 (triflorum); 4036 (chenopodioides); 5425 (triflorum); 6088 (chenopodioides); 6089 (palitans); 6159 (chenopodioides); 6398, 6424, 6486, 6487 (americanum); 6512 (palitans); 6535 (chenopodioides); 6536, 6537, 7465 (americanum); 7466 (chenopodioides); 9020, 9998 (americanum); 11825 (palitans); 11827 (chenopodioides); 13589 (nigrum).

Coville, F.V. 185 (nigrum); 256, 1256 (americanum).

Cowgill, W.H. 2076 (nigrum).

Cowie, I. 4476 (americanum).

Cowles, H.C. 232 (triflorum).

Craib, W.G. 579 (nigrum).

Craven, L. 9442 (americanum).

Craven, L.A. 2580 (chenopodioides).

Crawford, A.R. 372 (opacum).

Crawford, I. 418 (americanum); 1557 (opacum); 4024, 5827 (chenopodioides); 6196 (triflorum); 6312 (chenopodioides).

Crawford, I.R. 4024 (chenopodioides).

Creed, K.E. 280 (americanum).

Cremers, G. 8084 (americanum).

Creswell, A.M. 31 (villosum).

Cribb, P. 10246, 10345 (americanum).

Crisp, M.D. 1984, 2141 (americanum); 2494 (chenopodioides); 2757 (opacum); 4470 (americanum).

Crisp, P. 123 (nigrum).

Croat, T.B. 19668, 20836, 21777, 24287, 25067, 28440, 28489, 28522, 28684 (americanum); 29606, 29698 (scabrum); 34391, 34504, 38415, 51125, 51537, 57786, 58383, 62947, 64402, 65478, 66041, 70552, 74460, 74531 (americanum); 77396 (nigrum); 81916, 92283, 97618, 100194 (americanum).

Croft, J.R. NGF-34532, LAE-61910 (opacum).

Crofts, F. 7 (americanum).

Cronk, Q.C.B. 335 (nigrum); 346 (villosum); 409, 410, 447, 460 (nigrum); 488 (villosum).

Cronquist, A. 7827 (triflorum).

Crookshank, H. 98 (villosum).

Crosby, C.S. 117 (americanum).

Crosby, M.R. 1717, 2905 (americanum).

Crosby, M.T. 7 (chenopodioides).

Crouch, V.E. 1029 (americanum).

Cruden, R.W. 1100 (americanum).

Crutchfield, J.R. 3199 (americanum).

Cruz Rivas, A. 244ac, 268ac (americanum).

Cruz, J.S. de la 1664, 4237, 4442, 4602 (americanum).

Cuatrecasas, J. 22978 (americanum).

Cueva M, M. 26 (furcatum); 612 (americanum).

Cufodontis, G. 627 (memphiticum).

Culhati, S.C. 13 (nigrum).

Culwick, G.M. 2 (tarderemotum).

Cuming, H. 266, 266 (furcatum); 649 (americanum); 783[b] (nitidibaccatum); 999 (americanum); 1033 (nigrum); 21263 (nitidibaccatum).

Cumming, R.J. 22821 (americanum).

Cunha, L.S. 72 (americanum).

Cunnell, G.J. 167 (villosum).

Cuong, N.M. 551 (americanum).

Curle, C. 27 (villosum).

Curran, H.M. 188 (chenopodioides).

Curry, P. 651 (opacum).

Curtis, A.G. 310 (villosum); 613, 622, 780 (tarderemotum).

Curtiss, A.H. 6493 (villosum); 6863 (nigrum).

D'Arcy, W.G. 543, 2244, 2278, 2291, 2366, 2392 (americanum); 2467 (chenopodioides); 2483, 2493, 2500, 2506, 2570, 2597, 2619A, 2624, 2625, 2638, 2852, 3002 (americanum); 3644 (nigrum); 3692 (americanum); 3717, 3717 (triflorum); 3899, 3900, 3966, 5122, 5533 (americanum); 5599, 5675, 5710, 5711 (triflorum); 6035, 6052, 6171 (americanum); 7616, 7655, 7716a, 7793, 8089, 8525, 8604 (tarderemotum); 10474, 11676B, 11892, 11986, 12125, 13386, 14041, 15201, 15212 (americanum); 15215 (scabrum); 15282, 15304, 15305, 15307, 15348, 17521, 17535 (americanum); 17571 (scabrum); 17602 (americanum); 17617 (scabrum); 17681 (americanum); 17682 (scabrum); 17684, 17697 (americanum); 17698 (scabrum); 17698 (americanum); 17765 (scabrum); 17766 (nigrum); 19269 (americanum).

Dahl, A. 905 (nigrum).

Dahl, H.S. D.39b., D.45a. (nigrum).

Dahlstrand, K.A. 2919 (retroflexum).

Dainelli, G. 89 (memphiticum); 90 (villosum); 91 (memphiticum); 94, 98 (villosum); 279 (memphiticum).

Dalby, J.M. 88/05 (nigrum).

Dalton, M.G. 8 (villosum).

Dalziel, J.M. 1188 (americanum); 1188a, 1188b (scabrum); 8335 (pseudospinosum).

Dandy, J.E. 135 (villosum); 624 (scabrum).

Dangol, D.R. 12336 (nigrum).

Daniel, P. NC-67369 (americanum).

Daniel, P.M. 117 (scabrum).

Dann, M.P. 60 (nigrum).

Daramola, B.D. 154 (scabrum).

Daramola, B.O. FHI-72483 (scabrum).

Darko, K.O. 1032, 5115 (scabrum).

Darwin Chilean Initiative 1815 (furcatum).

Das, C.R. 112 (nigrum).

Das, D. 105 (villosum); 209 (americanum).

Das, R.K. 33921 (nigrum).

Dash, S.S. BSHC-21171 (americanum).

Daumlira, E.K. D-35, 36 (tarderemotum).

Daveau, J. 2446 (villosum).

Davidse, G. 5472 (americanum); 6704, 6812 (retroflexum); 7371, 10549, 11202, 11819, 36560 (americanum); 38975 (sarrachoides).

Davies, J.N. 222 (americanum).

Davies, R.M. 461 (tarderemotum); 1514 (scabrum).

Davis, D. 62812 (villosum).

Davis, P.H. 26067, 1514K, 2080, 6070B (villosum); 8987 (memphiticum); 26067, D38512, 40176, D47039, D48539, 50060, 53890, 55296, 67986, D68139 (villosum); 70402 (chenopodioides).

Davis, R.L. 201 (tarderemotum).

Davis, R.M. 1190 (retroflexum).

Davis, T. 661 (nitidibaccatum); 662 (triflorum); 714 (americanum).

Dawkins, H.C. D-752 (tarderemotum).

Dawson, G. 434 (americanum); 2457, 2716 (nitidibaccatum).

DBL 373 (villosum).

DCI (Darwin Chilean Initiative 2002-2005) 1815 (furcatum).

de Mera, G. 1510 (nitidibaccatum).

De Nevers, G. 10240 (nigrum).

Dean, C.H. 8987 (villosum).

Deb, D.B. BG-3534, 3534 (villosum); 27496 (americanum).

Decary, R. 755a, 755b, 2600 (americanum); 6179 (tarderemotum); 6179, 6656, 19370 (americanum).

Decker, B.G. 1280, 2185 (americanum).

Degen, R. 831, 2203 (americanum); 3132 (sarrachoides).

Degener, O. 16190 (triflorum); 33618, 33660, 34134, 35908, 36120 (americanum).

Deginani, N. 1281, 1321 (americanum).

Deguchi, K. 5408 (nigrum).

Deighton, F.C. 474, 1033 (americanum); 4571 (scabrum); 5660 (americanum).

de Lange, P.J. CH-625, K-790 (americanum); K-814, K-847 (nigrum); K-863, K-1269 (americanum); K-1270, K-1294, K-1317 (nigrum); K-1318, 1361, CH-2032 (opacum); 2913 (nigrum); 2914 (opacum); 3632 (nigrum); 6292 (nitidibaccatum).

Del Aguila, M. 162 (americanum).

del Carpio, C. 2537 (furcatum).

Del Vitto, L.A. 851 (triflorum); 3411, 4071 (nitidibaccatum).

Delgado B, A.A. 11 (americanum).

Delimitation Commission under Colonel Wahab 60 (villosum).

Delnatte, C. 2622 (nigrum); 2930, 3039, 3484 (americanum).

Demaio, P. 294 (chenopodioides).

Denham, S.S. 352 (chenopodioides).

Descoings, B. 775 (americanum).

Déséglise, A. 457 (villosum); 460 (nigrum).

Desert Locust Survey P.T.49 (villosum).

Despréaux, J.M. Webb-44 (villosum).

Devred, R. 137 (tarderemotum).

Dhoromick, D.C. 5291 (americanum).

Dhwoj, L. 51 (nigrum).

Di Fulvio, T.E. 386, 479, 483, 849 (pygmaeum).

Diabate, M. 1419 (scabrum).

Diamini, M.D. 612 (retroflexum).

Dias, R.S. 02 (americanum).

Díaz Gonzáles, J. 791 (americanum).

Díaz M, D. 1057 (americanum).

Díaz S, C. 2354 (americanum).

Díaz Vilchis, I. 381 (americanum).

Díaz Z, A.L. 193 (americanum).

Díaz, C. 3539, 9478, 9503 (americanum).

Dickson, H.V. 387 (villosum).

Dickson, J.D. 103 (nigrum).

Dieckman, L. 177 (americanum).

Diers, L. 90 (palitans).

Diesel, H. 631 (pseudospinosum).

Dieterlen, A. 157 (retroflexum).

Dietrich, A. 466, 827, 1049 (opacum); 1523 (americanum).

Dik, A. 1310 (americanum).

Dillewaard, H.A. 210 (americanum).

Dillon, L.A. 915 (triflorum).

Dillon, M.O. 3641 (americanum); 4539, 6503 (furcatum).

Dinklage, M. 2116 (americanum).

Dinsmore, J.E. 907, 1151, 2810, 5171, 8810, 9151, 10810, 11151, 12102 (villosum); 12151 (memphiticum); 12810 (villosum).

Dixon, S. 1/99 (nigrum); 148/99 (villosum).

DNEP1 303 (nigrum).

Döbbeler, P. 1072 (americanum).

Dobereiner 756 (americanum).

Dobson, A.T. 283, 300, 337 (nigrum).

Dodson, C.H. 11523 (americanum).

Dolan, J. 19 (americanum).

Dolman, J. 331 (nigrum).

Dombey, J. 343 (furcatum).

Domingo, M.A. 42 (furcatum); 57 (nitidibaccatum).

Domínguez Cadena, R. 2936 (americanum).

Donaldson Smith, A. 218, 221 (villosum).

Donat, A. 55 (triflorum); 415 (nitidibaccatum).

Dönmez, A.A. 13562, 16221 (nigrum).

Donner, N.N. 10365 (nigrum).

Dony, J.G. 4165 (sarrachoides).

Dorantes, J. 4009 (americanum).

Dorr, L.J. 2729 (americanum).

Doug, J.G. 1401 (nitidibaccatum).

Douglas, D. 23 (americanum).

Doutrelepont, H. 1203 (scabrum).

Dowson, W.J. 298 (memphiticum); 299 (villosum); 482 (tarderemotum).

Drake (Mr) 44 (villosum).

Drège, J.F. 7864a, 7864b (retroflexum).

Drewe, P.B. 174 (retroflexum).

Drummond, J.R. 1872, 2293, 20551, 25857 (nigrum); 25867 (villosum); 25868 (nigrum).

Drummond, R.B. 3347 (tarderemotum).

Du Parquet, R. 253 (villosum).

Duan, L.D. 3648, 20020641 (nigrum).

Duaneh, J. 319, 320, 444 (americanum).

Dubugnon, N. 187 (chenopodioides).

Duffey, E.A.G. 200 (americanum).

Duffour, C. 5538 (nitidibaccatum).

Dugand G, A. 1103, 4537, 5024 (americanum).

Dugand, A. 5024 (americanum).

Dujhlón, F.C. FCD1811 (scabrum).

Duke, J.A. 4044, 5199, 7336, 15437 (americanum).

Dulong Jiang Investigation Team 46 (americanum); 241, 287, 1022 (nigrum).

Dulta, A.K. 32, 235 (villosum).

Dümmer, R.A. 563 (tarderemotum); 1937 (memphiticum).

Dunaski, A. 89 (americanum).

Duncan, W.H. 3835 (americanum).

Dunlap, D. 2 (tarderemotum).

Dunn, D.B. 16384 (nigrum).

Duque Jaramillo, J.M. 2476, 3665, 4047A (americanum).

Durán, R. 1425 (americanum).

Duré, R. 208 (americanum).

Dusén, P. 386 (furcatum); 5465 (triflorum).

Duthie, J.F. 7535 (villosum).

Dutt, A.K. 647 (villosum); 651 (nigrum).

Dutta, R.M. 50 (nigrum); 261, 507 (americanum).

Duval, D.J. 2308, 2330 (opacum).

Duvdevani, S. 284 (villosum).

Dwyer, J. 13193 (villosum).

Dwyer, J.D. 1855, 6787, 7325, 11145 (americanum); 13147, 13811 (villosum).

Dybowska, A. 853 (nigrum).

Dziekanowski, C.T. 2554 (triflorum).

Earle, F.S. 152, 192 (triflorum).

Earthy, E.D. 8 (scabrum).

East India Company [Wallich cat.] 2618 (villosum).

Economic Botanist 25214 (nigrum).

Economides, S. ARI-980 (villosum).

Edaño, G.E. 24897 (americanum).

Edgecombe, M. 94 (americanum).

Edgeworth, ? 7009, 7045 (villosum).

Edmonds, J.M. C73, C74 (retroflexum).

Edmondson, J.R. 3311, E3419 (villosum).

Edwards, D.C. 3104 (villosum).

Edwards, S. 3674 (villosum).

Egea, J. de 807 (americanum).

Eggeling, W.J. 697 (tarderemotum).

Eggers, H.F.A. 286 (americanum).

Ehrich, R. 336 (tarderemotum).

Eichler, H. 21649 (chenopodioides).

Eichler, J. 43 (triflorum).

Einarsson, S. 234 (nigrum).

Eiten, G. 1596, 1895, 6239, 7876 (americanum).

El Assi, A. 523 (villosum).

El Ghani, M.A. 1195, 1453, 1653, 3633, 4339, 4517 (nigrum); 4552 (villosum); 5151, 6982, 7106 (nigrum).

Elbert, J. 4547 (americanum).

Elias 219 (americanum).

Elías, H. 2954 (villosum).

Elias, S.I. 178 (americanum).

Elias, T.S. 10628 (americanum).

Ellemann, L. 613 (tarderemotum).

Ellis, C.C. 251 (triflorum).

Ellis, R. 557 (villosum).

Ellman, E. 68, 338 (nigrum).

Elmer, A.D.E. 8105 (americanum); 8788, 15697 (nigrum); 17677 (americanum).

Elorsa C, M. 101, 1444 (americanum).

Emson, H.E. 296 (americanum).

Emwiogbon, J.A. FHI-43533 (scabrum).

Encarnación B, R. 71 (americanum).

Enti, A.A. 1723 (scabrum).

Erlanson, E.W. 5070 (nigrum).

Ern, H. 883 (scabrum).

Ertter, B. G-124 (americanum).

Escobar A, N. 10 (palitans).

Espínola, M.C. JPB-666 (americanum).

Espinosa Garcia, F.J. 197, 243 (americanum).

Espinosa H, A. 47 (americanum).

Espírito Santo, J. 218 (scabrum).

Esplen, J.C. 103 (villosum).

Essi, L. 303 (americanum).

Estelrich 7 (triflorum).

Estève, L. 111 (scabrum).

Etkin, N.L. 63A (villosum); 63[b] (scabrum).

Etuge, M. 1556 (scabrum); 2305, 3416 (tarderemotum).

Eugenio, J. 1089 (americanum).

Eupunino, E. 489 (americanum).

Evans, I.M. 57 (villosum); 412 (tarderemotum).

Evans, O.D. 3 (chenopodioides).

Everist, S.L. 2642, 5606 (americanum); 6160 (opacum); 7291 (nigrum); 10054 (americanum).

Evrard, C. 10603 (furcatum).

Ewan, J.A. 15691 (americanum).

Exell, A.W. 89, 396 (scabrum).

Expedition Purdy 109 (villosum).

Eyenhuisen, G. van 7, 25 (scabrum).

Eyerdam, W.J. 22152 (furcatum); 23162 (chenopodioides); 23555 (triflorum); 23682 (chenopodioides); 23905, 23954, 24449 (triflorum).

Eyles, F. 4712 (tarderemotum).

Faber, E. 63, 618 (nigrum).

Fabris, H.A. 3175 (nitidibaccatum); 6477 (palitans).

Faden, R.B. 67/198, 74/676 (villosum).

Fagbemi, A. 438 (scabrum).

Fagerlind, F. 517, 607, 696 (americanum).

Fagerström, K. 62 (villosum).

Fan Hsioh Niao 9120 (nigrum).

Fan, C.S. 179 (nigrum).

Fang, W.P. 12359, 12462, 19724 (nigrum).

Fang, W.Y. 8001 (nigrum).

Fanshawe, D.B. F1085 (americanum); F-5353, JMM-8310 (retroflexum).

Farfán, J. 1196, 1721 (americanum).

Farruggia, F. 2637, 2646, 2713, 2737, 2781 (americanum).

Faulkner, H.G. 1780, 3120 (americanum); 3746 (villosum); 4036, 4065 (americanum); 4858 (villosum).

Faurie, U. 512 (nigrum); 636, 637 (americanum); 690, 777 (nigrum); 861 (opacum); 863 (americanum); 864 (opacum); 880 (americanum); 1172, 1915, 3109, 5979, 6720 (nigrum).

Fawcett, H.C. 112 (opacum).

Fay, J.M. 1012 (villosum).

Fayed, A.A. 1295 (villosum).

Fealy, C. 31 (chenopodioides).

Felger, R.S. 90-73, 89-170, 88-614, 85-667 (americanum).

Félix, A.L. 55 (americanum).

Fendler, A. 249, 606 (americanum); 671 (triflorum).

Feng, H.T. 31 (nigrum).

Feng, K.M. 2350, 3391 (nigrum).

Fensham, R. 1290 (americanum).

Ferguson, L.F. 2731, 2884, 2927 (villosum).

Fernandes, A. 9373 (nigrum).

Fernandes, D. 291 (americanum).

Fernández Alonso, J.L. 5845A, 6498, 6746, 6772, 6911, 15973, 16864, 20790, 21674, 24816 (americanum).

Fernández Casas, J. 7290, 7389 (americanum); 10156B, 10157, 10313, 11184 (pseudospinosum); 11725, 11820 (scabrum).

Fernández N, R. 1352 (americanum).

Fernández, P. 49 (chenopodioides).

Ferreira, V.F. 3045 (americanum).

Ferreyra, R. 1486 (furcatum); 4402 (americanum); 6074, 6081b, 8255, 8952, 9718 (furcatum); 11625 (americanum); 12038, 12065 (furcatum); 12836, 17231, 17683 (americanum); 18319, 18361, 18361 (furcatum); 18866, 19959 (americanum).

Festo, L. 905, 1064, 1339 (tarderemotum).

Feuerer, T. 5492 (palitans).

Fidalgo de Carvalho, M. 3150 (americanum).

Field, H. 24 (nigrum).

Figueiredo, E. 95 (scabrum).

Figueroa Rosas, J. 34 (americanum).

Filipps, R. De 164 (americanum).

Filson, R.B. 3743 (americanum).

Fiori, A. 1593, 1594 (memphiticum); 1597, 1598, 1599, 1958 (villosum).

Fischer, S. 26 (nigrum).

Fischer, W. 100, 215 (triflorum).

Fisher, J.C. 1325 (americanum).

Fitzpatrick, W.M. 9 (villosum).

Flamigni, A. 352 (scabrum).

Fleming, G.W.T.H. 306 (pygmaeum).

Flenley, J.R. ANU-2192 (opacum).

Florence, J. 2724 (americanum); 3853 (opacum); 3946, 4154, 4365, 5031 (americanum); 9399, 10774 (opacum).

Flores C, A. 718 (americanum).

Flores Tolentino, M. 251 (americanum).

Flores, G. 27 (americanum).

Flores, S. 8, 61, 133 (americanum).

Flowers, S. 94, 146, 853 (triflorum).

Floyer (Mrs) 8 (pygmaeum).

FLSP 480 (furcatum); 759 (americanum); 913 (furcatum); 949, 1116 (americanum); 1149, 1240, 1291 (furcatum); 1325, 1415 (americanum); 1460 (furcatum).

Flynn, D. 17 (chenopodioides).

Fogg, J.M. 1462 (nigrum).

Fogaça, J. 02 (americanum).

Foggie, A. 21 (villosum).

Fohlen S-40a (villosum).

Folsom, J.P. 5161 (americanum).

Fonnegra G, R. 2722 (americanum).

Fonseca Vaz, A. 303 (paucidens).

Font Quer, P. 560, 561 (villosum).

Fontana, A.P. 5764 (americanum).

Forbes, F.B. 307 (nigrum).

Forbes, H.O. 1019 (alpinum); 2544 (nigrum); 3785 (americanum); 3881 (nigrum).

Forbes, S.J. 341A, 909 (triflorum); 937 (americanum).

Forero P, L.E. 428, 466, 1620 (americanum).

Forero, E. 1974 (americanum).

Forest Office 42514 (villosum).

Forrest, G. 4479, 7289, 8373, 12277 (nigrum).

Forsskål, P. 421 (memphiticum).

Forster, P.I. 17931 (opacum); 26512, 26512, 29317 (americanum); 37983 (chenopodioides); 40548, 42049 (opacum); 42109 (chenopodioides); 42140, 43098 (opacum).

Forstner, S. 2464 (nigrum).

Fortunato, R.H. 6041 (americanum).

Fosberg, F.R. 11337, 11756 (opacum); 20503, 25149, 25348, 27930, 27961[b], 28236, 29282, 31177 (americanum); 31180 (opacum); 32631, 35310 (americanum); 39475 (opacum); 44774, 44785, 44788, 44823, 48351, 48896, 57125, 57408, 59773, 61328 (americanum).

Fossey, D. B16 (tarderemotum).

Foster, R.B. 9756, 10334 (americanum).

Fox, M.D. 8204069 (americanum).

Frame, G.W. 24 (tarderemotum).

Franceschi, F. 16 (americanum).

Francia, P. 4 (americanum).

Franck, C.W. 180 (nigrum).

Franklin, ? 16383 (triflorum).

Freeman, J.D. 15 (americanum).

Freire de Carvalho, L d'A 148, 153, 570 (americanum).

Freitag, H. 2075, 5203 (villosum).

Freitas, E. 285 (americanum).

Freitas, L. 4, 5 (americanum).

Friedrichsthal, E. von 558 (americanum).

Friis, I. 203, 1621, 1740, 1855, 2047 (tarderemotum); 6612 (villosum); 6640 (memphiticum); 7133, 8263 (tarderemotum); 8350 (villosum); 8660, 10066, 10115 (memphiticum); 10411, 10524, 10525, 10536 (villosum); 10689 (memphiticum); 10699 (villosum); 11022, 11552 (tarderemotum); 11912 (hirtulum); 14694 (tarderemotum).

Frisch, R.O. 133 (americanum).

Fritchey, J.Q.A. 120 (triflorum).

Fuchs Q, F.M. 211 (americanum).

Fuentes, R.U. PPI-38985 (nigrum).

Fujikawa, K. 50167, 89358, 94443 (americanum).

Fukui, K.M. 15 (tarderemotum).

Funez, L.A. 129 (americanum).

Furlaud, J.M. 79 (nigrum).

Furse, P. 2821, 7778 (nigrum); 8230 (villosum); 8932 (nigrum).

Furtado, C.X. 25897 (scabrum).

Furuse, M. 1962 (americanum); 4837 (nigrum); 7793 (americanum); 9548, 26892 (nigrum); 40504 (americanum); 40522, 40677, 40678, 42704, 42834, 44637, 45139, 46072, 46168 (nigrum).

Gabrielith, R. 24, 121 (villosum).

Gachathi, F.N. 366 (villosum).

Gadelha Neto, P.C. 736 (americanum).

Gaerlan, F. PPI-5373 (nigrum).

Galán, P. 4753 (scabrum).

Gallagher, M.D. 7487/10 (villosum).

Gallinal PE3710 (chenopodioides).

Galpin, E.E. M-747 (retroflexum); 14832 (chenopodioides).

Gamble, J.S. 3398 (nigrum); 4478A (villosum); 15153, 17435 (americanum).

Game, J.C. 69/306 (americanum).

Gammie, G.A. 18462 (nigrum).

Gandoger, M. 17, 27, 215, 282 (nigrum).

Gaoligong Shan Biodiversity Survey 15695, 17210, 18169, 19296 (nigrum); 19656 (americanum); 21066, 21646, 22185, 22260, 22490, 22849, 23095, 23409 (nigrum); 23485 (americanum); 25609, 25832, 27451 (nigrum); 27683, 28955 (americanum); 32463, 33280, 34191 (nigrum).

Gaoligong Shan Biotic Survey Expedition Autumn 19656 (nigrum).

Gaoligong Shan Expedition 7345 (americanum).

García E, J.D. 16 (americanum).

Garcia, I. 70 (palitans).

García, M.A. 1417 (villosum).

García, P. 210 (americanum); 932 (pygmaeum).

García, R.C.A. 541 (palitans).

García-Barriga, H. 6308, 18415 (americanum).

Gardiner, J.S. 108 (americanum).

Gardner, G. 838, 1788, 2266 (americanum).

Gardner, M.F. 82, 94, 174 (furcatum); 1252 (villosum); 4034, 6738, 6963, 8322, 8356, 8462, 8478, 8649 (furcatum).

Gardner, R.O. 5866 (opacum*);* 10165, 11230 (americanum).

Garnett, C.S. 1017 (villosum).

Garrett, A.O. 2087, 2184, 5219 (triflorum); 6219, 6606 (nitidibaccatum); 7480, 8671 (triflorum); 8736 (nitidibaccatum).

Gasperetti, P.R. PG-422 (nigrum).

Gathorne-Hardy, R.E. 823 (villosum).

Gaudichaud, C. 112 (furcatum); 521, 522 (americanum); 1847 (furcatum).

Gaum, E.L. 15 (opacum).

Gaumer, G.F. 1533, 23546 (americanum).

Gautier, L. 5198 (scabrum).

Gay, C. 2 (furcatum).

Gbile, E.C. FHI-20567 (scabrum).

Ge, X. 242 (americanum).

Geay, F. 8096, 8978 (americanum).

Geerinck-Coutrez, ? 4368 (nigrum).

Geerling, C. 417 (scabrum); 1498 (americanum).

Geilinger, G. 2482 (tarderemotum).

Gentle, P.H. 7799 (americanum).

Gentry, A.H. 6621, 6758, 9375, 16416, 16464, 17944, 25381, 27256, 27384 (americanum); 35737, 36111 (furcatum); 63561, 63701 (americanum).

Gentry, H.S. 1269, 4232 (americanum).

Geoffray, C. 180 (americanum).

George, A.S. 3172 (americanum); 16913 (triflorum).

Gereau, R.E. 1548 (americanum); 4426 (tarderemotum); 5084 (umalilaense).

Germain, R. 5593 (tarderemotum).

Germishuizen, G. 7215 (retroflexum).

Gerrans, M.B. 1560, 1619 (nigrum).

Gerrard, W.T. 412 (tarderemotum).

Ghafoor, A. 4432 (villosum); 5204 (nigrum).

Ghosh, A.K. CNH-30437, 30497 (villosum); 37675 (americanum).

Ghosh, M.K. 57, 75 (americanum); CU-11036 (villosum).

Giacomin, L.L. 1646, 1971, 1974, 2009, 2013, 2021 (americanum).

Giardelli, M.L. 52, 326, 1056 (pygmaeum); 1165 (sarrachoides).

Gibbons, R.B. 294 (villosum).

Gibbs Russell, G.E. 3704 (retroflexum).

Gibbs, P.E. 1236.69 (villosum).

Gibert, E.J. 147 (sarrachoides).

Gibson, N. 3953 (americanum).

Gil, M. 21 (americanum).

Gilbert, E.F. 192 (memphiticum).

Gilbert, M.G. 651, 877 (tarderemotum); 1387 (memphiticum); 6305 (villosum); 7043 (tarderemotum).

Gilbert, V.C. E37, 2655 (villosum).

Gill, N. 645 (villosum).

Gillespie, J.W. 1110 (americanum).

Gillespie, L. 25 (villosum).

Gillett, J.B. 4126, 4923 (villosum); 5285 (memphiticum); 12957, 14057 (tarderemotum); 14586 (memphiticum); 15249 (scabrum); 15973 (memphiticum); 16081 (villosum); 19300 (memphiticum); 19889 (villosum); 19099 (tarderemotum).

Gillett, J.M. 16487 (americanum).

Gilli, A. 30 (nigrum); 222 (americanum); 499 (tarderemotum); 3240, 3241, 3242 (villosum).

Gilliat-Smith, B. 2123a (villosum).

Gillies, J. 3 (sarrachoides); 18 (triflorum); 33 (pygmaeum); 35 (triflorum); 38 (chenopodioides); 43, 44 (triflorum); 151 (chenopodioides); 1431x (pygmaeum); 1434 (triflorum); 1436 (palitans).

Gilliland, H.B. 476B, 974 (retroflexum); 1402 (tarderemotum).

Gillis, W.T. 7677 (americanum).

Gilmartin, A.J. 581 (americanum).

Gilon, A. 315[a] (scabrum); 315[b] (tarderemotum).

Gines, H. 2072 (americanum).

Giordano, G. 3, 22 (nitidibaccatum).

Giordano, L.C. 748 (americanum).

Giordano, O. 8 (triflorum).

Giraldo, G. 979 (americanum).

Glanville, R.R. 412 (scabrum).

Glaumann, F. 105 (americanum).

Gledhill, D. 998 (tarderemotum).

Glen, H.F. 1943 (retroflexum).

Glenfield Vet Research Station SN45-13 (triflorum); SN48/344 (palitans); SN48/2638 (chenopodioides).

Glocker, E.F. von 85 (americanum).

Glover, E.C. 3608 (tarderemotum).

Glover, P.E. 180 (villosum); 181 (memphiticum); 494, 615, 1238, 2252 (villosum); 2744, 3110 (memphiticum).

Gobbo, G. 666 (tarderemotum).

Godding, H.V. 162 (tarderemotum).

Godfrey, R.K. 52830 (americanum).

Godman (Dame Alice) 93 (villosum).

Godman, E.M. 67 (memphiticum).

Godoy, S.A.P. de 2024, 2033, 2047, 2063 (americanum).

Goel, A.K. NC-53907 (americanum); NC-64200, NC-64620 (villosum).

Goës, C.C. 596 (americanum).

Góes, O.C. 97 (americanum).

Goetghebeur, P. 2605, 5252, 8626 (triflorum).

Goldblatt, P. 5553 (retroflexum); 6780 (chenopodioides); 7915, 8416 (retroflexum).

Goldsmith, B.C. 74 (triflorum).

Gomes, L.A. 495 (americanum).

Gómez Chagala, B. 306 (americanum).

Gómez Marín, E. 157 (scabrum).

Goncalves, A.C. PSACF_EX4342 (americanum).

Goncalves, B. 240 (nigrum); 374 (chenopodioides); 1277, 2262, 2607, 3513, 3762, 4064 (nigrum); 4698 (chenopodioides).

Gonde, H.P. 371 (scabrum).

Gonzales, A. 583 (americanum).

Gonzáles, P. 17, 494, 531, 663B (furcatum); 1738 (americanum); 1853, 1992, 2853, 2859, 2870, 2874, 2875, 2928 (furcatum); 2964, 2972 (americanum).

González, S. 3112 (americanum).

González Elizondo, M.S. 822 (americanum).

González-Espinosa, M. 1185 (americanum).

González L, L.A. 4152 (americanum).

González Medrano, F. 16964 (americanum).

González R, E. 42 (americanum).

Goode, P.M. G-1-72, G-2-72 (tarderemotum); G3-72 (scabrum); G-5-72 (memphiticum).

Goode, R.H. 346 (opacum).

Goodier, R. 287 (villosum).

Goodman, C.M. 613 (triflorum).

Goodrich, S. 19277 (nitidibaccatum).

Goodwing, H.B. 197 (americanum).

Gordon, K.J. 11, 609B (memphiticum).

Goring, V. 2 (nigrum).

Gosper, D. 12 (americanum).

Gossweiler, J. 464 (americanum); 530b (scabrum).

Gould, B.J. 628 (nigrum).

Goulding, J.H. 563, 718 (nigrum).

Graan, J.F. van 406 (retroflexum).

Grace 6 (villosum).

Graham, J.G. 231, 4146 (americanum).

Graham, R.A. 1409 (nitidibaccatum).

Graham, R.D. 315 (villosum).

Grainger, J. 67 (villosum).

Granados-Tochoy, J.C. 892 (americanum).

Grant, J.M. 367 (nitidibaccatum).

Grant, M.L. 3623, 7433, 7439 (americanum); 15861 (nigrum).

Grassely, P.G. AR426 (triflorum).

Gray, M. 3712, 5901 (triflorum); 6796 (nitidibaccatum).

Graywood Smyth, E. 19 (americanum).

Green, P.S. RSNH1265, 1571 (americanum).

Greene, E.L. 231 (americanum).

Greenman, J.M. 50 (americanum).

Greenway, P.J. 6882 (villosum); 6951 (tarderemotum); 6952 (memphiticum); 6997 (villosum); 7459 (memphiticum); 10635 (villosum); 11096 (memphiticum); 11113 (tarderemotum); 11332 (memphiticum).

Greenwood, W. 105 (opacum).

Gremmen, N.J.M. T070024 (nigrum).

Greuter, W. 26375 (americanum).

Grierson, A.J.C. 1324 (americanum).

Grierson, M. 253 (villosum).

Grierson, R. H.955-18 (triflorum).

Griffith [663] (nigrum).

Griffith, W. 5901/1 (nigrum).

Griffiths, M.E. 47 (tarderemotum).

Grijalva, A. 1506, 2976, 4098 (americanum).

Grimshaw, J.M. 94-145 (tarderemotum); 94-148 (memphiticum); 93-455 (americanum); 94-470 (memphiticum); 93-728 (villosum).

Grondona, E.M. 6373 (pygmaeum).

Grossheim, A. I27 (villosum).

Grove, A.T. 5, 31A (villosum).

Guam Experiment Station 438 (americanum).

Guánchez M, F.J. 2122 (americanum).

Güemes, J. 1517 (villosum).

Guerin, J.R. 18 (opacum).

Guerrero, W. 336 (americanum).

Guest, E.R. 181, 304 (nigrum).

Guichard, K.M. KG/HAD35 (memphiticum); KG/HAD/234 (nigrum); KG/Lib/447 (villosum).

Guile, D.P.M. 1560 (pseudospinosum).

Guillarmod, J. 138 (tarderemotum).

Guillarmond, A.J. 5158 (sarrachoides); 5160 (retroflexum).

Guimarães, J.G. 14 (americanum).

Guinea, E. 1111, 1348, 1637 (scabrum).

Guiol, F.G. 1564 (nigrum); 1601 (villosum); 1602 (nigrum).

Gunn, R.C. 51[a], 51[b] (opacum).

Gust, G. 518 (nigrum).

Gutiérrez V, G. 17C669 (americanum).

Gutiérrez, J. 14 (chenopodioides); 155 (palitans).

Gutte, P. 16 (palitans); 8149, 8619 (americanum); 9536a (furcatum).

Guzmán, M. 1397 (americanum).

Guzmán-Teare, M. 756, 979, 1805 (americanum).

Haarer, A.E. 566 (villosum); 1209 (memphiticum).

Haast, J.F. von 265 (nigrum).

Haber, W. 10149 (americanum).

Haber, W.A. 11198, 11714 (americanum).

Hackforth-Jones, J. 396 (americanum).

Haegi, L.[A.R.] 1602 (chenopodioides); 1607 (americanum); 1711, 1714 (chenopodioides); 1715 (americanum); 1724 (chenopodioides); 1725 (americanum); 1730 (chenopodioides); 1737, 1744 (americanum); 1744 (opacum); 1756 (chenopodioides).

Haerdi, F. 286o (tarderemotum).

Hahn, L. 1, 325 (americanum).

Hahn, W. 766, 4818 (americanum).

Hahn, W.J. 531, 2056, 2470 (americanum).

Haines, H.H. 1979 (villosum).

Hajra, P.K. NC-82320 (americanum).

Hale, M. 44 (villosum).

Haley, B.R. 228 (villosum).

Halford, D. Q-3882 (opacum).

Hall, E. 467 (triflorum).

Hall, G.P. 22 (memphiticum); 29, 84 (villosum).

Hall, H.H. 21, 564 (triflorum).

Hall, H.N. 41 (nigrum).

Hall, J.B. 2411, 2416 (americanum); 46137 (scabrum); 47120 (americanum); 47139 (scabrum).

Hallé, N. RSNH6400 (americanum); 7032, 7321 (opacum).

Halliday, T.A. 354 (americanum).

Halse, R.R. 2725 (nigrum); 4702 (triflorum); 6115 (americanum); 8122 (nitidibaccatum); 8733 (americanum).

Hamad, K. NHI-46355 (villosum).

Hamel, C. 535 (scabrum).

Hamid, H. NHI-42695 (villosum).

Hamilton, H.J. 163 (opacum).

Hamilton, Surgeon Major 17900 (villosum).

Hammel, B.E. 3827, 5404 (americanum); 5895 (furcatum).

Hammond, H.D. 11511, 11928 (nitidibaccatum).

Hammond, M.M. 172 (villosum).

Hampshire, R.J. 80 (nigrum); 417 (villosum); 439 (nigrum); 730 (americanum).

Hams, E.H.M. 10 (villosum).

Hance, H.F. 861 (nigrum).

Handel-Mazzetti, H.F. 859 (villosum); 920 (nigrum); 2561, 2923 (villosum); 3133 (nigrum).

Hanekom, W.J. 564 (chenopodioides); 2499 (retroflexum).

Hansen, B. 1721 (americanum).

Hansen, G. 1175, 1388 (americanum).

Hanson, C.G. SP-07-20 (villosum); BS9625, BS9730 (nigrum); Yugo86-12 (villosum); CG96*N3 (furcatum); 107a (sarrachoides); 107b (nitidibaccatum); 255 (americanum); 329 (villosum); 499 (nitidibaccatum); 2000*C (villosum).

Hanson, H.C. 132 (triflorum).

Hara, H. 1544, 15412, 6303541 (nigrum).

Haradjian, M. 330 (villosum).

Harder, D.K.; Kayombo, M.J. 1351 (tarderemotum).

Hardial 528 (americanum).

Harding, D.P. 22 (villosum).

Harker, M. 134 (americanum).

Harley, R.M. 22191, 22926 (americanum); 27292 (chenopodioides).

Harley, W.J. 621, 1029 (scabrum).

Harling, G. 209, 310, 372, 9142, 26240 (americanum).

Harper, K.T. 578 (triflorum).

Harper, R.M. 1519 (nigrum).

Harris, B.J. 1078 (americanum).

Harris, J.A. 27756 (triflorum).

Harris, K. 4 (furcatum).

Harris, T. 186 (tarderemotum).

Harris, T.M. 90 (americanum).

Harris, W. 8538, 11898 (americanum).

Harrison, B.F. 1221, 6248 (americanum); 10707 (nitidibaccatum).

Hart, J.A. 1283 (americanum).

Hart, R. 4013 (americanum).

Hartweg, K.T. 202 (americanum).

Hashemi 12929E (villosum).

Hassler, É. 474, 622, 3104, 6400 (americanum); 10271 (chenopodioides); 12197 (americanum).

Hatschbach, G. 63010 (americanum); 71691 (sarrachoides).

Hattum, H.J. van 4073 (triflorum).

Haugen, T. 1740 (tarderemotum).

Haught, O. 138, 6590 (americanum).

Hausmann, F. von 126 (nigrum); 44, 183, 274 (villosum).

Hay, W.R. 286, 463 (villosum).

Hazel, C. 219 (tarderemotum); 486 (scabrum).

Hazra, P. 24 (villosum).

Head, D. 170 (retroflexum).

Healy, A.J. 59/283 (nigrum); 64/425 (furcatum); 68/159 (villosum); 99/34 (nitidibaccatum).

Heap, J. SOLTR1&2 (triflorum).

Heath, A. 1023 (tarderemotum).

Heath, I.B. 340A, 394 (villosum).

Hedberg, O. 4965 (villosum).

Hedge, I.C. 3931, W4073, W4290 (villosum).

Heiser, C.B. S128, 3627, 6098 (americanum).

Heldreich, T. von 3206 (nigrum).

Heller, A.A. 632 (nigrum); 2389, 2509, 2867 (opacum); 6572 (furcatum); 9458 (triflorum); 12208 (nitidibaccatum).

Heller, T.M. 289, 340 (villosum).

Helmkamp, G.K. 14256 (nigrum); 18451 (americanum).

Hemming, C.F. 2226 (villosum).

Hemsley, J.H. L-79 (nigrum).

Henderson, G. 477 (villosum).

Henderson, L.F. 5510 (triflorum).

Henderson, N.C. 92-336 (sarrachoides); 96-739 (americanum).

Henderson, R.J.F. 123 (retroflexum); 128 (chenopodioides); 246, 250 (opacum); 281 (americanum); 285 (opacum); 288 (americanum); 290, 291, 292 (opacum); 298, 299 (americanum); 301 (chenopodioides); 302, 359, 394, 396, 402, 403 (opacum); 404, 405 (nigrum); 518, 523, 544, 563, 564 (americanum); 1241 (nitidibaccatum); 1242 (nigrum); 1261 (opacum); 1271, 1273, 1274 (nigrum); 1290 (opacum); 1303, 1343 (americanum); 1356, 1357 (opacum); 1358, 1361, 1362, 1405, 1414, 1572, 2278, 2284 (americanum); 2314 (nigrum); 2315, 2357 (opacum); 2607, 2791 (americanum); 2922 (opacum); 3131 (nigrum).

Henderson, S. 98 (chenopodioides).

Hendrickx, F.L. 1488 (tarderemotum).

Heng, L. 10342 (nigrum).

Henning, W.H. 14 (nitidibaccatum).

Henrich, J.E. 450 (americanum).

Henrici, M. 3080 (retroflexum).

Henriques, C. 53 (villosum).

Henry, A. 625, 1024, 3210, 8511, 9870 (nigrum).

Henry, A.N. 52 (americanum).

Henry, R.D. 5764 (sarrachoides).

Hepper, F.N. 1297 (americanum); 2146 (pseudospinosum); 3109 (villosum); 4412, 4455, 4478, 4600, 4784 (americanum); 4823, 4837 (villosum); 4912 (memphiticum); 5500, 5614, 5628, 5649A, 5930 (villosum); 5984 (americanum); 6132, 6285, 6311, 6600, 6694, 7052 (villosum); 7453 (americanum); 7662, 7696 (nigrum); 7849 (scabrum); 8696, 8742 (villosum); 9760 (nigrum).

Herb. Amani 3580 (tarderemotum).

Herb. Gadeceau 1006 (villosum); 5106 (scabrum).

Herb. Lacaita 11416, 11417 (villosum).

Herb. Willdenow 4336 (chenopodioides).

Herbert, E.J. 49 (memphiticum).

Herbst, D. 6010 (americanum); 6210 (opacum); 6350, 6499, 6824, 6866 (americanum).

Heringer, E.P. 723, 4928, 15204 (americanum).

Hermann, F.J. 4912 (triflorum).

Hermann, H.A. van 527, 633 (americanum).

Hermann, M. 417 (chenopodioides).

Hernández M, M. 5473 (americanum).

Hernández Magaña, R. 6717, 9218 (americanum).

Hernández Ortega, R. 62 (americanum).

Hernández R, J. 5334 (americanum).

Hernani A, L. 1207 (furcatum).

Herrera, A. 438 (americanum).

Herrera, G. 6063 (americanum).

Herrgott, J.A.F.D. 116 (opacum).

Herter, W.G. 186 (chenopodioides); 17039 (sarrachoides); 17060, 70323 (chenopodioides).

Hespenheide, H.A. 932 (americanum).

Heyligers, P.C. 88025 (chenopodioides); 88050 (americanum); 88051 (chenopodioides); 89016, 92010, 92010, 94009, 95003 (americanum); 95004, 95004 (chenopodioides); 95005, 95005 (americanum); 95006, 95006, 95006 (chenopodioides); 99003, 99003 (americanum); 99004 (chenopodioides); 99005, 99005, 99005, 99005, 99005, 99006, 99006 (americanum).

Heywood, V.H. 955 (nigrum).

HFC (Herbario de Flora de Cuba) 43449 (americanum).

HGT 754 (villosum).

HHV FLSP759, FLSP1723a, 03570 (americanum).

Hicken, C.M. 372, 448, 509, 943 (pygmaeum).

Hicken, R. 38 (pygmaeum).

Hiepko, P. 1195 (opacum).

Hieronymus, G. 710 (triflorum).

Higgins, D.M. 1016 (nigrum).

Higgins, L.C. 757 (americanum); 15979 (nitidibaccatum); 19750 (nigrum).

Hikmat Abbas 616 (nigrum).

Hildebrandt, J.M. 475 (tarderemotum); 696 (memphiticum); 865 (villosum); 1626[B] (scabrum); 1626[A] (tarderemotum); 3110, 3401a (scabrum); 3796 (americanum); 34014a (scabrum).

Hilgert, N. 2165 (palitans).

Hill, S.R. 11711 (triflorum).

Hillebrand, W. 117, 118 (opacum).

Hilliard, O.M. 6134 (tarderemotum); 15180 (retroflexum); 15537 (chenopodioides).

Hinchinsbrooke, ? 69 (villosum).

Hingley, M.R. 168 (villosum).

Hipe, N.T. 1129 (nigrum).

Hislop, A. Z-155 (retroflexum).

Hislop, M. 2026 (americanum).

Hitchcock, A.S. 239 (americanum); 361 (triflorum); 13679 (americanum); 14294 (opacum); 19974, 20357 (americanum).

Hjerting, J.P. 645 (americanum).

Hmama, H. 894 (nigrum).

Hoare, A. 16 (americanum); 125 (nigrum).

Hobbs, J.F.F. 13297 (nitidibaccatum).

Hodge, J.B.S. D.4288 (triflorum).

Hodge, W.H. 799 (americanum).

Hodges, M. 96, 97 (americanum).

Hodgkin, H.T. 23, 82 (scabrum).

Hoehne, W. 12502, JPB-17122, JPB-17127 (americanum).

Hoff, M. 6245 (americanum).

Hoffmann, W.A. 245 (americanum).

Hofmeister, W. [42] (villosum).

Hohenacker, R.F. 768 (nigrum); 1077 (americanum).

Hohl, A. 157 (tarderemotum).

Hojra, P.K. BSHC-37 (americanum).

Holdridge, L.R. 1139 (americanum).

Hollermayer, A. 42 (furcatum).

Holliday, L.G. 9 (americanum).

Holm-Nielsen, L.B. 2027, 2048, 2735 (americanum).

Holmberg, N.J. 2085 (americanum); 2309 (sarrachoides); 2730 (triflorum).

Holmgren, I. 33 (americanum).

Holst, B.K. 6027 (americanum).

Holst, C. 2840, 9021 (tarderemotum).

Hoogland, R.D. 6014, 7444 (opacum); 8720, 11308 (americanum).

Hooker, J.D. 62? (villosum); 117bis (tarderemotum); 639 (villosum).

Hooper, D. REP-25810 (torvum); 39413 (americanum).

Hooper, S.S. 2014 (villosum).

Hoover, W.S. Deden89 (scabrum); Deden113 (alpinum); 30037 (nigrum); 30064 (scabrum).

Hopa, T.H. NC-87129 (americanum).

Hörandl, E. 26365 (nigrum).

Horiuchi, K. FOK-057483 (nigrum).

Hornby, H.H. 350 (villosum).

Horsfield, T. 5[a], 5[b], 7, 12 (alpinum).

Horton, P. 56301 (nigrum).

Hosaka, E.Y. 3112 (americanum).

Hoshino, T. 9666069 (villosum).

Hosking, J.R. 307 (opacum); 888, 959 (chenopodioides); 1022 (opacum); 1282 (palitans); 2117, 3091, 3091, 3496 (chenopodioides).

Hosseus, C.C. 147 (nitidibaccatum); 249 (pygmaeum).

Hostmann, F. 992 (americanum).

Hostmann, F.W.R. 992 (americanum).

Howard, R.A. 6558, 8506, 9640 (americanum).

Howell, T. 232 (nitidibaccatum).

Howitt, A.W. 411 (opacum).

Hu, ? 972 (nigrum).

Hu, S.Y. 19992 (nigrum); 22241 (americanum).

Huang, T.C. 1953 (americanum); 10632 (opacum).

Huashikat, V. 52, 206 (americanum).

Hubbard, C.E. 2508 (americanum); 3535 (opacum); 13261, 13262, 13263 (nitidibaccatum).

Hucks, M. 627 (villosum).

Hudson, H.S. 148 (sarrachoides).

Hudson, J. 964 (americanum).

Hügel, A. 1723 (americanum).

Hugh 72/28 (villosum).

Huidobro, A.M. 1410 (triflorum).

Huk, A. 57 (americanum).

Hukui, K. 26 (villosum).

Hull, H. 68 (chenopodioides).

Humbert, H. 1244, 1375, 6671 (scabrum); 11308 (americanum); 11309, 12191 (scabrum); 13124 (americanum); 14849 (retroflexum); 18679 (scabrum); 19091, 19707, 19707, 20182bis (americanum).

Humbles, J. 5014 (villosum).

Humphries, C.J. 206 (villosum); 334 (nigrum).

Hundt, O. 797, 798 (scabrum).

Hunn, E. OAX-205, OAX-793 (americanum).

Hunter, J.T. 2593, 2921, 3926 (opacum).

Hunter, R. 20 (americanum).

Hunziker, A.T. 588 (pygmaeum); 614 (sarrachoides); 1624 (chenopodioides); 1875 (palitans); 2141, 2242 (pygmaeum); 3301 (chenopodioides); 4420 (pygmaeum); 5548 (americanum); 6237, 7183, 7503, 7541, 7549, 7926, 8692 (pygmaeum); 8829 (nitidibaccatum); 9781 (triflorum); 10359, 10445 (pygmaeum); 11629 (triflorum); 11868 (nitidibaccatum); 12544, 13037 (triflorum); 13250 (pygmaeum); 14078, 14114 (nitidibaccatum); 14398, 15028 (triflorum); 15702, 18361, 18543 (palitans); 19552 (americanum); 20141 (triflorum); 21928 (palitans); 21967 (pygmaeum); 23521 (americanum); 24228, 24785 (nitidibaccatum); 24805, 24807 (triflorum); 24952 (pygmaeum); 25322 (nitidibaccatum); 25330, 25331 (pygmaeum).

Hunziker, J.H. 84 (pygmaeum); 1624 (americanum); 7509 (pygmaeum).

Hunziker, J.J. 1624 (chenopodioides).

Huq, A.M. 10557 (americanum).

Hurrell, J.A. 6037, 6439 (chenopodioides).

Hurworth, W.E. 35 (nigrum).

Hutchinson, J. 54 (pseudospinosum); 3767 (retroflexum).

Hutchison, P.C. 3458, 3853 (americanum).

Hutton, D. N13413 (nitidibaccatum).

Huxley, P. 116 (tarderemotum).

Hynniewta, T.M. BSHC-31971 (americanum).

Ibáñez, M. 33 (furcatum).

Ibarra Manríquez, G. 3189 (americanum).

Ichikawa, K. 33 (nigrum).

Idjan 63 (americanum).

IECAMA G58 (memphiticum).

Ignacio, D. 4 (americanum).

Ihue U, Y. 200425, 200475 (furcatum).

Ikabanga, D. 110 (americanum).

Ikegami, Y. 15792 (nigrum).

Illsey G, C. 1297 (americanum).

Iltis, H.H. 388, 744, 1554, 1593 (americanum); 30226 (nitidibaccatum).

Ináyat Khan 19969 (villosum); 22443 (nigrum).

Ingall, R.G. 005 (nigrum).

Ingoldby, C.M. 553 (villosum).

Inostroza, ? CONC-35154 (furcatum).

Irvine, F.R. 27 (americanum); 93, 3607 (scabrum); 4957 (tarderemotum); 5095, 5096, 5191 (scabrum).

Irwin, H.S. 5865 (americanum); 9151 (chenopodioides); 10990, 18095, 19170, 19812a, 20328, 20958, 23766, 28500, 29614, 30413a, 34909, 55947 (americanum).

Isern, J. 2535 (furcatum); 6302 (americanum).

Islip, S. 44 (nigrum).

Islomov, B. UPL-00304 (nigrum).

Ivens, G.W. 2503 (villosum).

Iwatsuki, K. t-10367 (americanum).

Izabella 200 (americanum).

Izuzquiza, A. 1082AI (villosum).

Jacintho, S.F. 2 (americanum).

Jackson, E.N.S. 5976 (nitidibaccatum).

Jackson, G. 1582 (opacum).

Jackson, J.K. 902 (tarderemotum); 2887, 4187 (villosum).

Jacobs, M. 7173 (opacum); 8533 (retroflexum).

Jacobs, S.W.L. 420, 767 (americanum); 2834 (chenopodioides).

Jacquemont, M.M. 783, 937 (villosum).

Jacques-Georges, A. 26547 (scabrum).

Jaeger, P. 1050, 4279 (tarderemotum).

Jaeschke, H. 304 (villosum).

Jafei, S.M.H. 1173 (nigrum).

Jaimes, M.S. 10 (americanum).

Jallu, J. 1038 (nitidibaccatum).

James, T. 29 (americanum).

Jameson, W. 96, 153 (americanum).

Janardhanan, K.P. NC-46338, NC-46372, NC-52836, NC-77525 (villosum).

Janaki Ammal, E.K. 1523 (villosum).

Jangoux, J. 166 (americanum).

Jansen, J.W.A. 1193 (americanum); 2015 (scabrum).

Jansen, P.C.M. 1644 (villosum); 4290 (tarderemotum); 8065 (americanum).

Jansen, R.K. 499 (americanum).

Jansen-Jacobs, M.J. 5503 (americanum).

Japp, R.N. 362 (americanum).

Jara, A. 483 (americanum).

Jara-Muñoz, A. 287 (americanum).

Jarquin L, E. 22 (americanum).

Jarrett, T. 454 (villosum).

Jarvis, C.E. 46 (nigrum).

Játiva, C.D. 153 (americanum).

Jayakar, A.S.G. 41 (villosum).

Jayasuriya, A.H.M. 2228 (americanum).

Jayaweera, D.M.A. 1043 (nigrum).

Jeanes, J.A. 2662 (furcatum).

Jeffery, G.W. K480 (villosum).

Jefford, T.G. 1818 (tarderemotum).

Jeffrey, C. 522, 2326 (americanum).

Jelski, C. de 49 (americanum).

Jenke, D. 17 (americanum).

Jennings, E.A. 20 (nigrum).

Jermy, A.C. 9070, 9239 (nigrum); 9805 (villosum).

Jermyn, S.T. 42, 338 (nigrum).

Jerónimo, H. 6, 279 (nigrum).

Jiménez E, N.D. 315 (americanum).

Jiménez, B. 107BJ (chenopodioides); 1339 (americanum).

Jiménez, Q. 1188 (americanum).

Jirbedi, G.N. Pharm-505 (villosum).

Jobson, P.C. 1037 (chenopodioides).

Johns, R.J. 9565 (opacum).

Johns, T.J. 86-481 (scabrum).

Johnson, L. 1272 (americanum).

Johnson, L.A.S. 49, 78, 1272 (americanum); 23530 (triflorum).

Johnson, R.W. 70, 462 (opacum).

Johnsone, S. 1 (opacum).

Johnston, E.L. 144a (triflorum).

Johnston, H.H. 76 (tarderemotum).

Johnstone, R.L. 2311 (americanum).

Jones, M.E. 1485, 4009, 5475 (triflorum); 24119, 27342 (americanum).

Jones, R.F. 48 (villosum).

Jones, W.T. 238(h) (americanum); 238 (opacum); 3317 (americanum).

Jordan, H.D. 1059 (scabrum).

Jörgensen, P. 3340 (*sarrachoides*).

Jorgensen, P.M. 56275 (americanum).

Joseph, J. 15280, 39848, 48402 (americanum).

Jouvin, P.P. 179, 533 (americanum).

Joyal, E. 1588 (americanum).

Junge, C. 1271, 2862 (furcatum); 3178 (chenopodioides).

Junghuhn, F.W. 405 (alpinum).

Jungmann, M.K. 22 (americanum).

Jungner, J.R. 1375 (nitidibaccatum).

Junod, H.A. 575 (scabrum).

Jury, S.L. 12606, 12717 (villosum); 13040 (nigrum); 13276, 15047 (villosum); 16503, 17766 (nigrum); 17823, 19031, 20761 (villosum).

Jørgensen, P.M. 56275 (americanum).

Kahatt Soto, N. 156 (americanum).

Kahn, F. 2980 (americanum).

Kairo, A. 60 (americanum); 513, 10639 (opacum); 10641 (americanum).

Kamau, W.M. 734 (villosum).

Kamundi, D. 673 (tarderemotum).

Kao, H.S. 4696 (nigrum).

Kao, M.T. 5702 (opacum).

Kassner, T. [ L.C.T.] 434 (americanum); 3110 (tarderemotum).

Katende, A.B. K1239, K1722, 1723 (tarderemotum).

Katende, T. 1099 (tarderemotum).

Kaunda, K. 44, 219 (tarderemotum).

Kaune, S.M. 616 (americanum).

Kaur, U. 16 (americanum).

Kayambo, E.J. 54 (americanum).

Kayap, R. 1182, 1436 (americanum).

Kayombo, C.J. 215 (umalilaense); 638 (tarderemotum); 3225 (villosum); 5641 (tarderemotum).

Keighery, B.J. 754, 755 (americanum).

Keighery, G.J. 1282 (nigrum); 1722, 5018, 8887, 9645, 11071, 15045, 16223, 16460 (americanum).

Keith, H.G. 352, 384, 419 (nigrum); 1032 (villosum); 1033 (nigrum); 1036 (villosum).

Kelaole, E.T. A81 (villosum).

Kellogg, A. 716b (americanum).

Kemp, J. TH7330 (opacum).

Kendrick, L. 64 (villosum).

Kenneally, K.F. 6095 (nigrum).

Kennedy, P.B. 1720 (triflorum).

Kent, D.H. 3423 (pygmaeum).

Kercher, P. AL.137 (villosum).

Kerfoot, O. 637, 638, 1103, 1419, 2440 (tarderemotum); 3547 (villosum); 3581 (memphiticum).

Kern, J.H. 4610 (triflorum); 14998 (nitidibaccatum).

Kerr, A.F.G. 403, 1231 (nigrum); 3500 (americanum); 4567 (nigrum).

Kerr, F.H.W. 2436A (villosum).

Kerstan, G. 2016 (villosum).

Kesharanandi 720 (villosum).

Kessler, M. 18 (americanum).

Kessler, P.J.A. 3103 (americanum).

Keytel, P.C. 1807 (nigrum).

Khan, R. 23, 1000 (americanum).

Khanna, K.K. GC-39916 (villosum).

Kharkevich, S. 686 (nigrum).

Khutsishvili, M. 7, 172 (nigrum).

Kiesling, R. 1619 (palitans); 6500, 6562 (triflorum).

Killian, N.T. YP-3578 (memphiticum).

Killick, D.J.B. 3253 (tarderemotum).

Killip, E.P. 14060, 22970, 23424, 24058, 25218, 28827, 41162, 41582 (americanum).

Kindeketa, W. 2549 (scabrum).

Kindeketa, W.J. 254 (tarderemotum).

King, D.O. 301 (pygmaeum); 401B (furcatum); 401A (nitidibaccatum); 535 (chenopodioides); 537 (triflorum); 538, 630, 720 (pygmaeum).

King, R.A. 308 (villosum).

Kingdon-Ward, F. 17085, 20022 (nigrum); 20240 (americanum).

King's collector 550 (nigrum); 566 (americanum).

Kings, W. C.B.65 (americanum).

Kirika, P. 75 (tarderemotum); 241 (memphiticum).

Kirizawa, M. 1048 (americanum).

Kirkbride Jr, J.H. 1597, 2143, 2684 (americanum).

Kisalye, N. 515 (tarderemotum).

Kisena, C. 529 (tarderemotum).

Kissane, J.C. 709 (chenopodioides).

Kitaibel, P. 349 (villosum).

Klein, V.L.G. 700, 1192 (americanum).

Klitgaard, B.B. 1405 (americanum).

Klungness, L.M. 74 (tarderemotum).

Knapp, S. 1181, 1808, 7626a, 7627, 7738, 7802, 9054 (americanum); 9838 (nigrum); IM-10079 (chenopodioides); IM-10095 (nigrum); 10112 (americanum); 10143 (nigrum); IM-10161 (americanum); IM-10162, IM-10163, IM-10164, IM-10165, IM-10166, IM-10167 (nigrum); 10205, 10206, 10210 (americanum); 10217 (furcatum); 10294, 10302, 10360 (americanum); 10488, 10504, 10513 (triflorum); 10579, 10608, 10648 (americanum); 10677 (villosum); 10690, 10754 (americanum); 10780 (villosum); IM-10782 (triflorum); 10785 (villosum); IM-10786 (nigrum); IM-10787 (americanum).

Knight, D.H. 756 (americanum).

Kobakhidze, L. 1009 (nigrum).

Kodela, P.G. 577 (americanum); 578 (chenopodioides).

Koelz, W. 11622 (villosum); 12172 (nigrum); 13217 (villosum); 13219, 13911 (nigrum); 14269, 14583 (villosum).

Koga, T. 146 (americanum).

Köie, M. 2897 (villosum); 3127 (nigrum).

Kolenati, F.A.R. 2057 (nigrum).

Komaromi, R. 48, 61 (scabrum).

Komarov, V. 1376 (nigrum).

Koning, J. de 519 (scabrum); 715, 1743 (americanum); 1747 (scabrum); 7088 (americanum).

Konta, F. 3309 (americanum).

Koorders, S.H. 37852B (alpinum).

Korhonen, M. 1108 (villosum).

Koritschoner, H. 1903 (tarderemotum); 2191 (scabrum).

Kotschy, K[C].G.T. 166[c] (villosum); 184[a] (tarderemotum); 184[b], 184[c], 313 (villosum); 291 (tarderemotum); 313, 355, 446, 622 (villosum).

Koyama, H. 7669 (nigrum).

Koziol, E. 1635 (nigrum).

Kral, R. 38204, 44479, 51349, 53519, 68065 (americanum).

Krapovickas, A. 2729 (triflorum); 2824, 3275 (chenopodioides); 4348 (triflorum); 20006 (americanum); 22308 (triflorum); 25161 (americanum); 26156 (chenopodioides); 27060, 27444 (pygmaeum); 27529, 27720 (chenopodioides); 47749 (sarrachoides).

Krieger, L. 3311, 19452 (americanum).

Kress, W.J. 94-4666, 94-4995 (americanum).

Krishnan, E.K. 30 (nigrum).

Krug, B. 7450 (nigrum).

Krüger, H. 700 (triflorum).

Kuhlmann, J.G. 2602 (americanum).

Kruif, A.P.M. de 2186, 5550 (tarderemotum).

Kumar, K.R. 3350 (americanum).

Kumar, S. ANC-26937 (americanum); ANC-26968 (americanum); ANC-27623 (melongena); NC-81210, NC-92384 (americanum); NC-93367, NC-101615 (nigrum).

Kumar, V. 731 (nigrum).

Kurwari, G.R. 12959 (americanum).

Kruse, H. 1028 (americanum).

Kubo, R.R. 101 (americanum).

Kucera, D. 171 (nigrum).

Kujikat, A. 457 (americanum).

Kukkonen, I. 8166 (villosum).

Kummer, F. 5811 (villosum).

Kunstler, H. 566 (americanum).

Kuntze, C.E.O. 234 (americanum); 3018 (triflorum); 5410, 5999 (alpinum).

Kurbanov, D. 1606 (nigrum).

Kurosawa, T. 3149 (nigrum).

Kurtz, F. 912, 923 (pygmaeum); 4612 (triflorum); 4685 (pygmaeum); 4707 (triflorum); 5265, 5269 (pygmaeum); 5534b (triflorum); 6994a (nitidibaccatum); 9245 (pygmaeum); 14205 (triflorum); 15727, 16057, 16149 (pygmaeum).

Kurz, S. 201 (nigrum); 682 (alpinum).

Kwatha, R.B. 210 (scabrum).

La Croix, I.F. 2909 (tarderemotum).

La Torre, M.I. 1791, 1808, 1890 (furcatum).

La Touche, J. de 512 (nigrum).

Lab Staff (Uganda Agric Dept) 2153 (tarderemotum).

Lace, J.H. 580 (nigrum); 3394, 4034 (villosum).

Lachashvili, N. 1050 (nigrum).

Ladd, D. 22294 (sarrachoides).

Ladell, W.R.S. 263 (nigrum).

Lahitte, R. 293 (chenopodioides).

Lai, P. NC-63043 (americanum).

Lakela, O. 30601 (americanum).

Lakshnakara, M.C. 368 (nigrum).

Lambdon, P.W. A036 (americanum).

Lambinon, J. 86/354, 906 (nigrum); 907 (villosum); 1461 (triflorum).

Lambrecht, F.L. 90 (scabrum).

Lammers, T.G. 8400 (sarrachoides); 8490 (americanum).

Lamond, J. 1611, 2283 (villosum).

Lamoureux, C. 2225, 2298 (americanum).

Lamoureux, C.H. 4075 (americanum).

Landrum, L.R. 4416, 7429 (furcatum); 7807 (americanum).

Langenheim, J.H. 3336 (americanum).

Lanjouw, J. 3122 (americanum).

Lankester, T.E. 336 (villosum); 1419 (nigrum).

LaRivers, I. 767 (triflorum).

Larsen, E.L. 176, 5830, 7208 (triflorum).

Larsen, K. 67 (nigrum); 34265 (americanum).

Lasser, T. 739 (americanum).

Latilo, M.G. FHI-77429 (scabrum).

Latz, P.K. 13385 (nigrum).

Lau, S.K. 209 (americanum); 1005 (nigrum); 3752 (americanum); 4236[a], 4238, 5025 (nigrum); 5963, 25582 (americanum).

Lavranos, J. 15827, 15875 (villosum); 15947 (memphiticum).

Lavranos, J.J. 15827, 15875 (villosum).

Lawairee, A. 22320 (villosum).

Lawalrée, A. 15503, 21201, 23602 (nigrum).

Lawlor, D.W. 410 (tarderemotum).

Lawrance, A.E. 168 (americanum).

Lawrence, D.W. 169 (chenopodioides).

Lawrence, E. 138 (americanum).

Lawrence, W.E. 4368 (triflorum).

Lazar, Y. 92 (nigrum).

Lazarides, M. 332 (nigrum).

Lazeo, F.S.M. 5 (americanum).

Lazor, R.L. 2136, 2795 (americanum).

Le Clezio 259 (americanum).

Le Cussan, J.M. 939 (americanum).

Le Tram Chan C-96 (americanum).

Léandri, J. 3092 (americanum).

Lebrun, J.P. 3575, 7466, 7719 (tarderemotum); 8015 (americanum); 8318 (tarderemotum); 8380 (memphiticum); 9163 (tarderemotum).

Leeuwenberg, A.J.M. 2130 (americanum); 4668 (scabrum).

Legrand, C.D. 419 (chenopodioides).

Lehmbach, H. 175 (pseudospinosum).

Lehto, E. 3496 (nitidibaccatum).

Lei, C.I. 159 (americanum).

Leiberg, J.B. 877 (americanum); 929 (nigrum).

Leigh, J.H. S600 (triflorum).

Leishman, A.J. 115 (americanum).

Leistner, O.A. 104 (tarderemotum); 2706 (retroflexum).

Leitão, F. 111, 133 (americanum).

Lejeune, A.L.S. 55 (scabrum).

Leland, B. 78 (americanum).

Lely, H.V. 31 (scabrum); P.392 (villosum).

Lemos Fróes, R. de 11663 (americanum).

Lent, R.W. 582 (americanum).

León G, J. 48 (americanum).

León, R.J. 4071 (pygmaeum).

Leonard, J. 3627 (villosum); 3708 (nigrum); 4408 (villosum); 4738 (nigrum).

Leonard, S.W. 3470 (chenopodioides).

Leoni, L.S. 108 (americanum); 6554 (americanum).

Leonis, C. 137 (villosum).

Lepschi, B.J. 137 (chenopodioides); 381, 391 (americanum); 481, 489, 493, 539 (chenopodioides); 542, 555 (triflorum); 683, 740, 786, 897 (chenopodioides); 903, 904 (americanum); 930, 930 (chenopodioides); 974 (opacum); 1153 (americanum); 1729 (*nitidibaccatum*); 1752, 2580, 4113 (americanum); 4916 (opacum); 5088 (chenopodioides).

Lescure, J.P. 630 (americanum).

Lester, R.N. 43, 45 (scabrum).

Leteinturier, B. 145 (villosum).

Letouzey, R. 6880 (scabrum); 13444 (pseudospinosum).

Leveque, R. 36, 200A (americanum).

Levine, C.O. 178 (americanum).

Lewalle, J. 1536 (tarderemotum); 2978 (villosum); 3620 (tarderemotum); 5327 (villosum); 9857 (nigrum); 10085, 10673 (villosum); 11182 (nigrum); 11247 (villosum).

Lewer, S. 5 (palitans).

Lewis, C.M. 674 (triflorum).

Lewis, M. 88105 (americanum).

Lewis, W.H. 388, 554, 3299, 3398, 5183 (americanum); 7336 (nitidibaccatum); 8003, 10197 (americanum).

Leybourn, W.A. 75 (triflorum).

Li Sheng-tang 81-1327 (americanum).

Li, H. 8809 (americanum); 10342, 12853, 13913, 15251 (nigrum).

Liane 3661 (americanum).

Libbey, R.P. H536/44 (nitidibaccatum).

Liben, L. 1124 (tarderemotum).

Licata, A. 383 (americanum).

Liebenberg, L.C.C. 6877, 7410 (retroflexum); 7909 (chenopodioides); 8240 (retroflexum); 8568 (chenopodioides); 8842 (retroflexum).

Liesner, R.L. 5388 (americanum).

Lim, B.K. 1440 (nigrum).

Lima, J.R. 84 (americanum).

Limbach, C.F. 145 (americanum).

Lin, C.H. 147, 953 (americanum).

Lind, E.M. 82 (villosum).

Lindberg, K. 756 (villosum).

Lindeman, H.A. 352 (tarderemotum).

Linden, J.J. 250 (americanum).

Linder, D.H. 2214 (tarderemotum).

Lindsay, N. 1023 (nigrum).

Liney, J. 2765 (chenopodioides).

Liogier, A.H. 9024-14 (americanum).

Liou, S.Z. 305 (nigrum).

Lippert, W. 16053 (villosum).

Lithgow, G.M. 246 (americanum).

Liu Pi 6042, 6512 (nigrum).

Liu, S.L. 890109 (nigrum).

Liu, Y.C. 302 (nigrum).

Llatas Q, S. 733 (americanum).

Lleras P, E. 1943 (americanum).

Lobão, A.Q. 13, 45 (americanum).

Lobo Miranda, F.C. 2444 (villosum).

Löffler, H. E-107 (tarderemotum).

Loher, A. 4380 (opacum).

Loibl, J. 1954 (villosum).

Lojacono, M. 215 (nigrum).

Long, C. 124 (americanum).

Long, C.R. 1720, 2238, 2318, 2383 (americanum).

Long, R.W. 2828 (americanum).

Longbottom, W.D. 11015 (americanum).

López C, R. 7828 (americanum).

López Ch, L. 234 (americanum).

López L, L.M. 18 (americanum).

López T, E. 035 (furcatum).

López, A. 91 (palitans).

Lopez, I. 32 (americanum).

López, N. de 924 (americanum).

Lorence, D.H. M179, 5751 (americanum).

Lorentz, P.G. 26 (chenopodioides); 83, 106[b] (pygmaeum); 194 (palitans); 550 (pygmaeum); 1132 (triflorum).

Lotan, R. Soni-161008, Soni-200150 (nigrum).

Lott, E.J. 3397 (americanum).

Louis, A.M. 1990 (scabrum); 2292 (americanum).

Louis, J.L.P. 5419 (tarderemotum); 10507 (scabrum).

Lourteig, A. 1135, 1912 (americanum).

Lousley, J.E. W/493 (triflorum); 605 (americanum); 4710 (pygmaeum).

Loveridge, M.V. 350 (tarderemotum).

Lovett, J. 327 (tarderemotum).

Lovett, J.C. 1489 (tarderemotum); 2122 (villosum); 2192 (tarderemotum); 3696 (americanum); 3731 (tarderemotum).

Low, A. 382 (villosum).

Lowe, J. 2951 (scabrum).

Lowe, R.T. 16[A], 16[B], 37[A], 37[B], 119 (nigrum); 547, 722, 722[a], 722[b] (villosum).

Lox, B. Q94-01 (chenopodioides).

Ludlow, F. 6010 (nigrum).

Lugagne, R. 7256 (nigrum).

Lugard, E.J. 209 (tarderemotum).

Lugas, L. 2760 (americanum).

Lugo S, H. 6082, 6094 (americanum).

Luke, W.R.Q. [Q.] 12026, 14111 (tarderemotum); 12339 (scabrum); 14210 (villosum); 14736 (tarderemotum).

Lundell, C.L. 1185 (americanum).

Lurvey, E. 480 (americanum).

Luttrell, C. 187 (opacum).

Luwiika, B. 460 (tarderemotum).

Lyne, A.M. 1936 (chenopodioides).

Lynes, H. 37c, 132, 134 (villosum).

Lyons, M.N. 4693 (americanum).

Maas Geesternanus, R.A. 4314 (nigrum); 5603 (tarderemotum).

Mabatha, F.W. 2542 (americanum).

Mabberley, D.J. 1169 (tarderemotum).

Macbride, J.F. 734, 738 (nigrum).

MacBryde, B. 1061 (americanum).

MacDaniels, L.H. 174 (americanum).

MacDonald, E.C. 29 (opacum); 117, 137 (retroflexum); 164, 174 (chenopodioides); 233 (opacum).

MacDougal, D.T. 137, 139a (tarderemotum); 213 (triflorum).

MacDougal, J.M. 3754 (americanum).

MacGillivray, J. 805 (opacum).

Machado, B. 1826 (americanum).

Machado, D.N.S. 337 (americanum).

MacInnes, D. 154 (tarderemotum).

MacIntosh, D. 62 (villosum).

MacKee, H.S. 20631, RSNH-24143, RSNH-24217 (americanum); 26964 (opacum).

MacKeever, F.C. 434 (nitidibaccatum).

Macmillan, H.F. 219, 220 (nigrum).

Macmillan, H.G. 22 (furcatum).

Maconochie, J.R. 2990 (villosum).

Madrigal, B. 540 (americanum).

Madsen, J.E. 63358, 63457 (americanum).

Madulid, D.A. 910, 1565, 8801 (americanum).

Maesen, L.J.G. van der 962 (americanum); 3190, 7766 (villosum).

Maffey (Lady) 5 (villosum); 39 (tarderemotum).

Magaji, S.O. MG-725 (scabrum).

Magaña, M.A. 876 (americanum).

Maguire, B. 2066 (triflorum).

Maheshwari, J.K. 4027 (villosum); 4635, 5614 (nigrum).

Maikin Lantoh SAN-108976 (opacum).

Maioli, V. 168 (americanum).

Maire, E.E. 78, 409 (nigrum); 531 (villosum); 813 (americanum).

Maitland, D. 103 (nigrum).

Maitland, T.D. 147 (tarderemotum); 414, 522 (villosum); 916 (tarderemotum); 1290 (memphiticum); 1301, 1333 (pseudospinosum); 1790 (memphiticum).

Maity, D. BSHC-21633, BSHC-23245 (americanum); BSHC-25627, BSHC-25657 (nigrum).

Majumdar, S.C. NC-86359 (villosum).

Majunder, N.C. 8, 10, 203 (americanum).

Makin, J. 168 (nigrum).

Makings, E. 4677 (americanum).

Makinson, R.O. 209, 842, 842 (americanum).

Malcomber, S.T. 1716 (americanum).

Malhotra, B.K. NC-19716 (americanum).

Malhotra, C.L. NC-12018, NC-12054, NC-12464 (americanum); NC-13359 (nigrum); NC-13480 (villosum); NC-22832 (nigrum); NC-23596 (americanum); NC-23691 (nigrum); NC-26752, NC-26817 (americanum); NC-27338, NC-31432 (villosum); NC-31595, NC-50575 (nigrum).

Malhotra, S.K. NC-13156 (nigrum); NC-15364, NC-15874, NC-19165 (americanum); NC-19716, NC-22715 (villosum); NC-28784 (americanum); NC-31226 (villosum).

Malick, K.C. 45 (villosum).

Malik, K.I. 115 (nigrum).

Maliwanag, E. 132 (americanum).

Malthastra, M.C. NC-19580 (villosum).

Mamadou, E.M. 194 (americanum).

Mamaslu 2008 Expedition 20816047 (nigrum).

Mambo, P. 282 (tarderemotum).

Man, H. 85132 (americanum).

Man, L.S. 54019, 55105, 92854 (americanum); 93206 (nigrum); 96061 (americanum).

Manaslu 2008 Expedition 20815026 (nigrum).

Mandal, N.R. BSHC-10021 (americanum); BSHC-10183, BSHC-10441 (nigrum);

Mandaville, J.P. 308, 2499, 3414 (nigrum); 3538, 3986, 6286, 6358, 6406, 6673, 6980, 7414 (villosum).

Manikandan, R. NC-112491 (americanum).

Manissadjian, A. 349b, 981b (villosum).

Mann, G. 1321, 1938 (pseudospinosum).

Manning, S.D. 235 (scabrum).

Manoharan, C. 19575 (americanum).

Manoko, M.L.K. 2010-1, 2010-2, 2010-8, 2010-11, 2010-12, 2010-14 (umalilaense).

Mantuano, M. 64 (americanum).

Manua, M.K. 998, 1092 (americanum).

Marcan, A. 2225 (nigrum).

Marcelo, J. 62, 63 (americanum).

Marcolino, F. 163 (americanum).

Margery, C. 4071 (americanum).

Maries, C. 191, 249 (nigrum).

Marques, A. 2142 (tarderemotum).

Marquete, R. 4312, 4555 (americanum).

Marten, A.M. van 100 (americanum).

Martensz, P.N. 76 (triflorum); 188 (americanum).

Marticorena, C. 1077 (furcatum).

Martin, G.J. M-242 (americanum).

Martin, J.H. 3243 (villosum); 3592 (americanum).

Martin, J.S. 17 (americanum).

Martin, P. 6848 (chenopodioides).

Martin, R.F. 1321 (americanum).

Martin, W.H.R. 230 (tarderemotum).

Martinelli, G. 3579, 3969 (americanum).

Martínez Calderón, G. 1342, 1850 (americanum).

Martínez Crovetto, R. 6261 (palitans).

Martínez L, A. 124 (americanum).

Martínez S, E. 8044, 29419, 29430, 39832 (americanum).

Martínez, F.R. 95 (villosum).

Martins, J. 310 (nigrum).

Martius, C.F.P. 1255 (scabrum).

Mashaly, I. 20189 (villosum).

Masheti, S. H314 (tarderemotum).

Mason, C.T. 3875 (americanum).

Mason, R. 1363, 1609, 1724, 1726, 2170, 2200, 2298, 2476 (nigrum).

Massawe, G. 257 (tarderemotum).

Maté, C. 14 (scabrum).

Matesevach, M. 10 (nitidibaccatum); 38 (triflorum).

Mathenge, S.G. 64 (villosum).

Mathew, B. 6379 (tarderemotum).

Mathews, A. 270 (furcatum); 730 (americanum).

Mathey, A. 164 (americanum).

Matthei, O. 73 (furcatum); 138 (nitidibaccatum); 510 (americanum).

Matthew, K.M. 17002, 19984 (americanum); 24905 (nigrum); 46324 (americanum).

Mattos, J.R. 50, 74 (americanum).

Matzenbacher, N.I. 3064 (americanum).

Mauad, L.P. 29, 443 (americanum).

Maurisi, R. BSIP-8109 (opacum).

Mautone, L. D23, 554 (americanum).

Maxon, W.R. 3230 (americanum).

Maxwell, J.F. 92-100 (nigrum); 91-474 (americanum); 94-536, 94-1244 (nigrum).

May, F. 668 (americanum).

Mbuthia, K.W. 406, 734 (villosum).

McBarron, E.J. 9 (chenopodioides); 3094, 3118 (triflorum); 4253 (americanum); 6803, 7960 (chenopodioides); 9021 (palitans); 9998, 11054, 11545, 11725, 12357, 14881 (chenopodioides); 15534, 15583 (palitans); 16154 (triflorum); 20125 (chenopodioides).

McCallum-Webster, M. 1431, 2142, 7015 (nitidibaccatum); 7041 (pygmaeum); 8768 (nitidibaccatum).

McClean, A.P.D. 378 (retroflexum); 869 (chenopodioides); 28270 (retroflexum).

McClelland, D. 365 (nigrum).

McCornish, J.D. 46 (americanum).

McDaniel, S. 8866, 13861, 23804, 25378, 30219, 32880 (americanum).

McDonald, ? 1528 (americanum).

McDonald, J. 808, 1548 (tarderemotum).

McDonald, T.J. 00548 (americanum).

McDowell, T. 2604, 3207 (americanum).

McFadden, I. 9 (nigrum).

McGill, L. 20785 (americanum).

McGill, L.A. 6254 (triflorum).

McGillivray, D.J. 3627 (americanum).

McGregor, R.L. 31057 (sarrachoides).

McKee, H.S. 654 (triflorum); 3794 (americanum).

McKenna, S.G. 217, 468 (americanum).

McLeish, I.M. 425, 2006, 2140, 2257, 2510, 3317, 3636 (villosum).

McMahon, A.H. 47/118 (villosum).

McNamara, S. 12 (nigrum).

McNeill, J. 225, 496 (villosum).

McPherson, G. 4594 (americanum).

Mearns, E.A. 831, 842 (tarderemotum).

Medeiros 395 (americanum).

Medina C, M.A. 228 (americanum).

Medler, B. 105 (americanum).

Medley Wood, J. 83 (americanum); 811 (retroflexum); 7077 (tarderemotum).

Meenakshi, B. 65 (americanum).

Meer, P.P.C. van 1861 (tarderemotum).

Meier, W. 16386 (americanum).

Meijer, W. 15435 (pseudospinosum).

Meikle, R.D. 2735, 5026 (villosum); 5048 (nigrum).

Meinertzhagen, R. 152 (villosum).

Melo, P.H.A. 875 (americanum).

Melville, F.A. 4 (americanum); 188 (scabrum).

Melville, R. 2922 (pygmaeum).

Memeryan 5159 (villosum).

Mendonça, F.A. 1646 (villosum).

Menendez, F. 216 (americanum).

Menkins, I.L. ILM-584 (opacum).

Merrill, E.D. 361 (americanum); 464 (nigrum).

Merton, L. ARI-8 (villosum).

Mesfin Tadese 1659 (hirtulum).

Messer, E. 212a (americanum).

Metcalfe, O.B. 138, 1005 (triflorum).

Metlen, R. 3103 (nitidibaccatum).

Mettam, R.V. 228 (memphiticum).

Mexia, Y. 4336, 5930 (chenopodioides); 6298 (americanum).

Meyer, F.G. 797 (nitidibaccatum); 7497 (memphiticum); 8635, 8759 (tarderemotum).

Meyer, R.S. 285 (nigrum).

Meyerhoff, E. 22 (villosum).

Meyers, F. B-4102 (nigrum); 4810 (villosum).

Meyers, F.S. 102, 102c, 102a (villosum); 401 (nigrum); 809, 2809, 3151 (villosum); 4102 (nigrum); 5151, 6102 (villosum).

Meza T, E.I. 856 (triflorum).

Mhoro, B. 864 (tarderemotum).

Michael, N. 694A, 3026 (opacum).

Michel, R. de 2769 (sarrachoides).

Middleton, D.J. 2476 (americanum).

Miers, J. 276 (furcatum); 1235, 1412, 1427, 1428 (chenopodioides); 1815, 4540[b] (americanum).

Miéville, R. 37297 (americanum).

Migeod, F.W.H. 88, 358 (scabrum).

Mikoláš, V. 2375 (nigrum); 2541, 3928 (villosum); 5956 (nigrum); 6304, 6359, 6369 (villosum); 6723 (nigrum); 7887, 8082, 8530, 8588, 8619 (villosum).

Mildbraed, G.W.J. 10888a (pseudospinosum).

Millan, B. 01 (americanum).

Millar, A.N. NGF-15825[b] (opacum); NGF-15825[a] (americanum).

Mille, L. 977 (americanum).

Miller, A. 114, 555, 592, 873 (villosum).

Miller, A.C. 3004 (memphiticum).

Miller, A.G. 42 (villosum); 114 (nigrum); 147, 374 (villosum); 592 (nigrum); 2395 (villosum); 3424 (nigrum); 5292, 6632, 7196 (villosum); 7715 (nigrum); M.8248 (villosum).

Miller, E.M. M12/226 (americanum).

Miller, J.S. 6349 (americanum); 6772 (triflorum); 6882 (nitidibaccatum); 8833 (americanum); 9208 (nigrum).

Miller, O.B. 4341 (retroflexum).

Milliken, W. 1362 (nigrum); 1581 (opacum).

Millspaugh, C.F. 1082 (americanum); 4432 (chenopodioides); 4436 (americanum).

Milne, W.G. 2 (opacum).

Milne-Redhead, E.W. 1834 (triflorum); 2565, 8869 (tarderemotum); 10186 (villosum); 10387, 10430, 10440 (tarderemotum).

Milthorpe, P.L. 5072 (triflorum).

Mimoro, K. 2645 (nigrum).

Mintzer, M. 1 (palitans).

Miranda, A.G. 562 (americanum).

Misra, O.P. 9771 (villosum); NC-41511 (americanum); NC-41840, NC-44354 (villosum); NC-44450 (americanum); NC-46912 (villosum).

Mitchell, A.A. 5513, 5513, 6682, 6682, 6682, 7413, 7413, 8071 (americanum).

Mitchell, R.S. 7838 (nitidibaccatum).

Miyamoto, F. 10017, 94-10017 (nigrum).

Miyazaki, T. 99-1120II/5 (villosum); 0310334 (americanum).

Mlangwa, J.A. 482 (villosum); 1506 (tarderemotum).

Mocquerys, A. 163 (americanum).

Modenke, H.N. 442 (americanum).

Mohan, A. 13135 (americanum).

Mokim, S. 1280 (villosum).

Molas, L. 1391 (sarrachoides).

Moldenke, H.N. 442, 867a (americanum).

Molepo, L. 18 (retroflexum).

Molina R, A. 35125 (americanum).

Molina, A. 209 (americanum).

Moll, E.J. 4138 (scabrum); 5119 (americanum).

Moller, A.F. 2444 (nigrum).

Monachino, J.V. 176 (nitidibaccatum).

Monod, T. 11733, 11884 (scabrum).

Monro, A.K. 2916, 3953, 6415 (americanum).

Monro, C.F.H. 1024 (retroflexum).

Monteiro, T. dos PSACF_EX3733 (americanum).

Montero O, G. 212, 5618 (furcatum).

Montes, J.E. 2143, 2324, 16065 (americanum).

Moodie, M.E. 1208 (triflorum).

Mooney, H.F. 2236, 2473 (americanum); 3160 (villosum); 4752 (hirtulum); 5743, 5858 (villosum); 6232 (tarderemotum); 6680 (villosum); 8719 (tarderemotum); 9136 (memphiticum); 9164 (tarderemotum); 22361 (americanum).

Moore, C.W.E. 3055 (triflorum); 9158, 9391, 9450 (americanum).

Moore, G.C. 327 (americanum).

Moore, J.W. 212 (opacum).

Moorthy, S. AC-341 (nigrum).

Moran, R. 4978 (nigrum); 6448 (americanum); 20118 (americanum).

Moran, R.C. 7781[b] (americanum).

Moran, R.V. 4978, 6914, 29534 (americanum).

Morel, I. 2740, 2857, 2930, 3466, 3749, 5782 (americanum).

Moreno, P.P. 347, 820, 2628, 2752, 2990, 6691, 7070, 8142, 9975, 10181, 10561, 10998 (americanum).

Morero, R.E. 110 (chenopodioides).

Moretti, C. 1168 (americanum).

Mori, S.A. 11624 (americanum).

Moriarty, V.K. 1513, 1538 (americanum).

Morley, M. 680 (americanum).

Morong, T. 9 (pygmaeum); 262 (americanum); 1104 (furcatum).

Morris, D.I. 86418 (nitidibaccatum).

Morrison, A. 14228 (nigrum); 19014 (opacum).

Morrone, O. 646, 1286 (americanum).

Morton, C.V. 2658, 4923 (americanum).

Morton, J.K. EA-18 (tarderemotum); 48, K243 (scabrum); 346 (tarderemotum); A-435 (americanum); SL-445, SL-446 (tarderemotum); K.758, K874 (pseudospinosum); SL-892 (americanum); K1398 (scabrum); SL-1575 (tarderemotum); A-1796 (americanum); A-2383 (scabrum); SL-2644, SL-3143 (tarderemotum); A-3675, A-4028, A4277 (scabrum); 8101 (americanum); GC-24359 (scabrum).

Moscone, A. 84, 105 (chenopodioides).

Mosén, C.W.H. 4483 (americanum).

Moss, C.E. 13307 (chenopodioides).

Mott, P.J. 236C (retroflexum).

Moulton, L.A. 7 (americanum).

Moura-Júnior, E.G. 8 (americanum).

Mrkvicka, A.C. 1964, 4638 (villosum).

Mshasha, E. 144 (scabrum).

Muasya, J. GBK003/008/, 628, 729 (villosum).

Muehlenbach, V. 1436 (sarrachoides).

Muir, T.B. 2789 (triflorum); 3244 (opacum).

Mukerjee, S.K. 5016 (americanum).

Mukherjee, A. MN-2462 (nigrum).

Mulford, I. 460 (triflorum).

Muller, G. 9544 (furcatum).

Müller, G.K. 3639 (nitidibaccatum).

Múller, P.J. 54 (retroflexum).

Muller, T. 743 (retroflexum).

Müller-Hohenstein, K. 544 (villosum).

Mungai, G.M. 13884 (tarderemotum).

Munn-Estrada, X. 841 (americanum).

Munton, P.N. 17 (villosum).

Munyenyembe, P. 790 (tarderemotum).

Murata, G. T-14870 (americanum); 20136 (nigrum); 39046, 6303595 (americanum).

Murata, J. 25627 (nigrum).

Murfet, D.E. 3676 (triflorum).

Murphy, H. 314, 475 (americanum).

Murti, S.K. NC-62161 (americanum).

Murty, V.K. 56 (americanum).

Musk, H. 157 (scabrum).

Mutimushi, J.M. 2820 (scabrum).

Mwambunga, A.A.M. 6 (umalilaense).

Mwangangi, O.M. 1397 (tarderemotum); 1797 (memphiticum).

Mwangulango, N.A. 45 (tarderemotum).

Nafday, K.U.R. 30 (americanum).

Naganuma, R. 01 (americanum).

Naguib, M.I. 9 (nigrum).

Nair, N.C. NC-1099 (americanum); NC-2034 (villosum); ANC-6250 (americanum); NC-16121, NC-18954 (nigrum); NC-20005, NC-21756 (villosum); NC-22000 (americanum); NC-22166, NC-22215, NC-22480 (villosum); NC-23125 (nigrum); NC-24678 (americanum); NC-24764 (nigrum); NC-25266 (villosum); NC-26044 (nigrum); NC-26179 (americanum); NC-27506 (nigrum); NC-29874 (americanum); NC-32949 (villosum); NC-35793 (americanum); NC-36383 (nigrum); 61452 (americanum).

Nair, V.J. NC-14732 (nigrum); 14778 (villosum); NC-19915 (americanum); NC-19972, NC-21618 (villosum); NC-23226, 57128 (americanum).

Naithani, B.D. NC-43928, NC-53810, NC-68002 (villosum).

Naithani, H.B. 213 (villosum); 6018 (americanum).

Nalhoiba, C.L. NC-51540 (villosum).

Nantinjal, K.C. 25421 (nigrum).

Napier, E.R. 135 (villosum); 337 (memphiticum); 1847, 2455 (tarderemotum).

Narayanaswami, V. 3339 (nigrum).

Nash, E. 68 (villosum).

Nasher, A.K. 26/85, IH126 (villosum).

Nath, B.K. 13362 (americanum).

Nation, O. 49 (retroflexum).

Nattrass, R.M. 341b (memphiticum).

Nava Rodríguez, V. 80 (americanum).

Ndegwa, J. 177 (villosum).

Neck, J. 72B (americanum).

Necker, W.L. 16, 326 (americanum).

Nee, M. 3397, 3435, 3494, 3621, 3966, 4043, 4176, 4929, 7651 9081, 9182, 9544, 14121 (americanum); 14623b, 14623, 14629b (scabrum); 14678 (chenopodioides); 16063 (scabrum); 16069, 16073 (nigrum); 16081 (scabrum); 16082 (opacum); 16088 (scabrum); 16093 (chenopodioides); 16099 (scabrum); 16672, 16674 (americanum); 18112 (chenopodioides); 18248 (scabrum); 18259 (retroflexum); 19522 (americanum); 27524 (scabrum); 30110 (nigrum); 30237, 30257, 30275 (americanum); 30349 (palitans); 30488, 31258, 31331, 31350, 31415, 31508, 31813, 31899, 32011 (americanum); 32059 (palitans); 32519, 33342, 33520 (americanum); 33936 (palitans); 36780 (nigrum); 37163, 38763, 42350, 42564 (americanum); 43042 (nitidibaccatum); 43058, 43068 (palitans); 45551 (nigrum); 45983, 50082 (americanum); 50529 (palitans); 50960 (nigrum); 51080 (nitidibaccatum); 52967 (americanum); 53365 (nigrum); 53590, 54632 (americanum); 54714 (nigrum); 54867 (americanum); 54919 (palitans); 55229 (americanum); 55538 (nigrum); 57020 (villosum); 57198, 57207, 60196 (nigrum); 60201, 60203, 60214, 60220, 60223, 60226, 60228, 60235, 60237, 60242, 60246, 60247, 60248, 60249, 60259, 60270, 60276 (americanum).

Negri, G. 136 (villosum); 205, 244 (memphiticum); 307, 563 (villosum); 564 (memphiticum).

Negritto, M.A. 290 (palitans).

Negrón Bonilla, L.A. 2 (americanum).

Neldner, V.J. 3987 (americanum).

Nelson, A. 2804 (nitidibaccatum); 8076 (triflorum).

Nelson, B.W. 782 (americanum).

Nelson, D.J. 523 (nigrum).

Nelson, E.W. 191, 3868, 6640, 7176 (americanum).

Nesbit Evans, E.M. 53 (tarderemotum).

Neth Ind Forest Service 7056 (alpinum).

Neto, L. 14 (americanum).

Neubauer, H.F. 849, 3153 (villosum).

Neves, R. 222, 226 (americanum).

Newberry, P.E. 202 (villosum).

Newbould, J.G.B. 83 (villosum); 3289 (memphiticum); 3542 (tarderemotum).; 4285 (tarderemotum).

Newby, J. ZP-51 (villosum).

Newman, E.I. 170 (villosum).

Ngomba, J.N. 2 (tarderemotum); 3 (scabrum).

Ngoni, J.F. 409 (scabrum).

Nicholas, A. 2369 (retroflexum).

Niederlein, G. 149, 286 (nigrum).

Nielsen, I. 230 (nigrum).

Njoroge, S.J. S4 (villosum).

NMK 18 (villosum).

Nning, J. 52 (pseudospinosum).

Noblick, L.R. 4260 (americanum).

Nohan, A. 312, 13505 (americanum).

Nolan, M.E. 19 (americanum).

Norlindh, T. 4199 (retroflexum).

Norman, C. 119 (americanum).

Norman, J.K. UNK-9 (americanum).

Norris-Rogers, A.J. 599 (retroflexum).

Novara, L. 7904 (palitans).

Novara, L.J. 595, 653, 1588, 2642, 7375, 7904, 8125, 8276, 8700 (palitans).

Novelo R, A. 752 (americanum).

Núñez V, P. 6017, 6490, 8601, 8764, 9514, 12319, 20824 (americanum).

Núñez, E.M. 6 (furcatum).

Núñez, V. 524 (palitans).

Nusbaumer, L. LN-1674 (americanum).

Nuvunga, A. 256 (americanum).

Nyakundi, D. 728 (villosum).

O'Donell, C.A. 4490 (chenopodioides).

O'Keefe, J. 37 (americanum).

O'Ryan, K. 39, 39 (chenopodioides); 39 (opacum).

O'Shanesy, P.A. 71 (opacum).

Oberneder, R. 6650 (villosum).

Oberti, J.C. CORD297 (triflorum).

Obregón B, R. 17C911 (americanum).

Occhioni, E.M. 526 (americanum).

Occhioni, P. 459 (americanum).

Ochoa, C.M. 2688, 14627, 14662, 14901 (furcatum); 15043 (americanum).

Ochoterena, H. 931, 933 (americanum).

Octjabreva, N. 5887 (nigrum).

Odewo, T.K. 130, 151, 157, 159 (scabrum); 592 (americanum); 727 (scabrum).

Ogden, F.H. 11 (villosum).

Ogle, C.C. 7, 37 (opacum).

Ohashi, H. 774285 (nigrum).

Ohba, H. 8310439, 85-30831 (nigrum); 8350528, 8350558 (villosum).

Ohlinger, L.B. 1396 (chenopodioides).

Okigbo, B.N. 57 (tarderemotum).

Oldeman, R.A.A. 22 (scabrum).

Oldham, R. 236, 336, 575, 852 (nigrum).

Olet, E.A. 1 (americanum); 40 (villosum); 44 (memphiticum); 46, 47 (tarderemotum); 48, 49 (scabrum); 66 (americanum); 88 (scabrum); 106 (tarderemotum).

Olivas Matey, C.M. 138 (americanum).

Oliveira de Britto, Y.L. 242 (americanum).

Oliver, R.L. 2677 (americanum).

Olorunfemi, J. FHI-57034 (scabrum).

Olson, T. 11 (triflorum).

Omar, S. NHI-50389 (villosum); NHI-50421 (nigrum).

Ommanney, H.T. 140 (retroflexum).

Oosterzee, P. van 15 (americanum).

Orchard, A.E. 3351 (americanum); 3364 (nigrum); 3412 (americanum); 3413 (nigrum); 3435, 3454 (americanum); 3499 (nigrum); 3516, 3594 (opacum); 3639 (nigrum); 3680 (americanum); 3778 (opacum); 3917, 3948 (nigrum); 4008 (opacum); 5024 (nigrum).

Orcutt, C.R. 594 (americanum).

Ordóñez, M.J. 68 (americanum).

Ordóñez, M.T. 14 (americanum).

Ordoñez, O. 14 (americanum).

Orlandini, P. 182 (americanum).

Orozco, C.I. 3907 (americanum).

Ortega, J.G. 4224 (americanum).

Ortega, L.C. de 123, 1164, 1528, 1828, 2096 (americanum).

Ortiz V, E. 140, 1215, 1239 (americanum).

Ortiz, E.M. 1215, 1239 (americanum).

Ortíz, F. 1798 (americanum).

Ortiz, M. 1121 (americanum).

Ortíz, R.T. 1168 (americanum).

Osborne-Day, C. 55 (americanum).

Osmaston, H.A. 2328 (tarderemotum).

Osten, C. 4506, 22526 (sarrachoides).

Ostenfeld, C.H. 5298 (chenopodioides).

Oswald, P.H. 22 (villosum).

Oteke, J. 87 (tarderemotum).

Otero, J.I. 165 (americanum).

Otobed, D.O. PW-10137 (americanum).

Ottley, A.M. 7 (americanum).

Pabst, G. 4251 (americanum).

Pachano, A. 169 (nitidibaccatum).

Páez V, J.A. 13 (americanum).

Paget, A. 2919 (chenopodioides).

Palacios, M.A. 749 (chenopodioides).

Palmer, E. 11, 60[b] (americanum); 60[c] (nitidibaccatum); 362, 363, 860 (americanum).

Palmer, E.J. 196[a], 554 (americanum); 5884 (triflorum); 12023 (nitidibaccatum); 13882, 38000 (triflorum); 52976 (sarrachoides); 60872 (nitidibaccatum).

Palmer, P.R. D-7 (americanum).

Pammel, L.H. 2, 3897 (triflorum).

Panatkool, M. 221, 322 (nigrum).

Pancho, J.V. 14990 (americanum).

Pandey, R.P. 4186 (villosum).

Pani, P.C. NC-80670 (americanum).

Panigrahi, G. CC-2132 (nigrum); CC-2839 (villosum); 6157 (americanum); 11246, 11606 (villosum); CC-12610 (nigrum); 15543 (americanum).

Panigrahi, S. 5778 (nigrum).

Pannister, C.W. 983 (chenopodioides).

Pant, P.C. NC-43450, NC-43694 (americanum); NC-43796 (villosum).

Paoli, G. 935 (villosum).

Pappi, A. 2099 (memphiticum); 2901, 3188 (villosum); 3601 (memphiticum); 3772 (villosum); 4318 (scabrum); 4359 (memphiticum); 4387 (villosum); 6893 (memphiticum).

Parada, G.A. 1004, 1635 (americanum).

Parada-Gutierrez, G.A. 1635 (americanum).

Parangbye, N.P. Rustam-38 (nigrum).

Paris, E.G. 279 (villosum).

Park, B.C. 3, 519 (villosum).

Parker, C. O88, E558 (villosum); E580 (tarderemotum).

Parmar, P.J. AC-8623 (nigrum); 9573 (villosum).

Paroisse, G. 10 (americanum).

Parris, B.S. 12071 (nigrum).

Pastore, A.L. 1195 (pygmaeum).

Pastore, G.J. 1185 (chenopodioides).

Pataskan, R.D. 104144 (villosum).

Patel, I.H. 204 (americanum); 3607 (tarderemotum).

Patiño, D. 4 (americanum).

Patoni, C. 527 (americanum).

Patrick, S.J. 5063 (americanum); 5066 (chenopodioides).

Patzlaff, R. 55 (americanum).

Paul, T.K. CNH-43603 (villosum); 43603, 43752 (americanum).

Paula, C.H.R. de 277, 621 (americanum).

Paula-Souza, J. 7178 (americanum).

Pauwels, L. 5099 (americanum).

Pawek, J. 8205 (scabrum); 12428, 14222 (tarderemotum).

PBG 184 (nigrum).

Pearce, R.D. 154, 155 (chenopodioides); 156 (americanum); 160 (opacum).

Pearson, H.H.W. 9834 (retroflexum).

Peck, M.E. 9059 (furcatum); 9750, 10712 (nitidibaccatum).

Peden, A. 3 (triflorum).

Pedersen, T.M. 385 (americanum); 1054, 2767 (pygmaeum); 3079 (americanum); 6065 (pygmaeum); 7253 (sarrachoides); 8252 (chenopodioides); 10288 (pygmaeum); 10890 (americanum); 12513 (pygmaeum); 12514 (chenopodioides); 12893 (sarrachoides); 13868 (americanum); 15379 (sarrachoides).

Pedley, L. 2774 (americanum); 3012 (opacum); 5024, 5024 (americanum); 5403, 5403 (chenopodioides); 5816, 5816, 5816, 5816, 5816 (americanum).

Peláez, C. 239 (palitans).

Pelosi, E. 44, 60 (pygmaeum).

Peña-Chocarro, M.C. 1489, 1529 (chenopodioides).

Pennell, F.W. 12663, 13048 (furcatum); 14460 (americanum); 14538 (furcatum); 16799 (americanum).

Pensiero, J. 2741 (americanum).

Penther, A. 1846 (retroflexum).

Perdomo, R. 22 (americanum).

Perdue, R.E. 6363, 8280, 9065 (tarderemotum).

Perea, J. 3413 (americanum).

Pereira, A. 533 (americanum).

Pereira, E. 1810, 4098 (americanum).

Pereira, S.C. 1511A (chenopodioides).

Pérez Arbeláez, E. 6336 (americanum).

Pérez, B. 19, 86 (americanum); 207 (sarrachoides); 263, 675 (americanum).

Perret, H. 1765 (villosum).

Perrotet, G.S. 555 (scabrum); 903[a], 904, 904 (americanum).

Perrottet, G.S. 555, 556 (scabrum).

Perry, G. 255, 255 (americanum).

Perry, W. 2 (americanum).

Perumal, P. 16700, 16724 (americanum); 21473 (opacum).

Pessôa, S.V.A. 585 (lacteum).

Petrak, F. 676 (nigrum).

Petruscu, C. 2465 (triflorum).

Phengklai, C. 3085 (americanum).

Philcox, D. 3986, 4647 (americanum); 8823 (tarderemotum).

Philippi, F. 742 (furcatum).

Philippi, R.A. 278 (furcatum).

Philipson, W.R. 10168 (americanum).

Phillips, A.M. 9372 (americanum).

Phillips, E. 843, 1519 (tarderemotum).

Phillips, M.E. 88, 933, 1047, 1064 (americanum).

Phillips, S.P. 2524 (opacum).

Phillipson, P.B. 1077 (retroflexum); 1353 (sarrachoides); 1354 (retroflexum); 3272 (americanum); 4757 (retroflexum); 4783 (tarderemotum); 4784 (villosum); 4785 (memphiticum); 5256 (sarrachoides).

Phipps, J.B. 1368, 2653 (scabrum).

Pickard, J. 1216 (nigrum); 1470, 2749, 2763, 2832, 2896, 2904, 2930, 3002 (americanum).

Pietrellini, F. 285, 286 (furcatum).

Piffard, F. 22 (villosum); 24, 88 (tarderemotum).

Pilz, G.E. 2108 (scabrum).

Pinkard, A.B. 41 (nigrum).

Pinto, G.C.P. 196/84 (americanum).

Pinto da Silva, A.R. 6754 (chenopodioides).

Pinzl, A. 2467 (nitidibaccatum).

Piovano, G. 511 (hirtulum); 512 (villosum).

Pipoly, J.J. 7319 (americanum).

Pires, J.M. 9223 (americanum).

Pirozynski, K.A. P86 (tarderemotum).

Pistarino, A. 744 (nigrum).

Pitard, C.J. 1755 (villosum).

Pittier, H.F. 2543, 4378, 6087 (americanum).

Pizziolo, W. 47 (americanum).

Platt, J.W.G. 397, 512 (nigrum).

Platt, J.W.O. 397, 512 (nigrum).

Plowman, T.C. 2403 (americanum); 2621, 2622 (furcatum); 5513 (americanum); 8395, 8930 (americanum).

Po, U.S. 12110 (nigrum).

Podlech, D. 8624 (triflorum); 11394, 11517, 12710 (villosum); 16842, 18676 (nigrum); 19449 (villosum); 19792, 19950, 32146 (nigrum); 32273 (villosum).

Podzorski, A.C. 1198 (villosum).

Poelman, L. 7 (tarderemotum).

Poeppig, E.F. 156 (furcatum); 538 (nitidibaccatum); 1366 (americanum).

Pohl, J.B.E. 600, 2393, 2489, 5408 (americanum).

Poilane, E. 11791 (americanum); 20429 (nigrum).

Pojarkova, A. 6, 210 (villosum).

Pole Evans, I.B. 936A (tarderemotum).

Polgár, S. 2698 (americanum).

Polhill, E. 139 (villosum); 145, 148 (tarderemotum).

Polhill, R.M. 828, 974 (villosum); 1130, 1130 (memphiticum); 1207 (villosum); 1656 (tarderemotum); 1686 (villosum); 2302 (tarderemotum); 2342, 2342 (memphiticum); 12188 (villosum).

Polunin, O. 353, 1858 (nigrum); 4281, 13930 (villosum).

Ponsonby, L. 26 (scabrum).

Popov, G.B. PB17, 69131 (villosum); 71257 (memphiticum); GP7231, 18734, 18719 (villosum).

Porter, C.L. 10792 (americanum).

Porter, D.M. 1577 (furcatum); 4067 (americanum).

Post, G.E. 281 (villosum).

Pote, L. 114 (nigrum).

Pottier, R. 36c, 171 (americanum).

Powell, D.A. 410 (nigrum).

Powell, J.M. 804 (americanum).

Pradhan, P. 126, 201 (villosum).

Prado, D. 629, 640 (pygmaeum).

Prado, M. 34 (americanum).

Prain's collector 745 (nigrum).

Prance, G.T. 6261, 10131, 58736 (americanum).

Prasad, M. 39377 (villosum).

Press, J.R. 26 (nigrum); 169 (villosum); 515, 607, 623 (nigrum); 1166 (villosum); 1209 (nigrum); 1285 (villosum).

Preston, N.C. 37 (villosum).

Preuss, P.R. 740a (pseudospinosum).

Prévost, M.F. 3911 (americanum).

Proctor, G.R. 11443, 15277, 16962, 17630, 17826, 18369, 19020, 19115, 21179, 23681, 24759 (americanum).

Prokes, F. 5 (villosum).

Prudhomme, E. 28 (scabrum).

Pujupet, J. 1047 (americanum).

Puleston de 7631 (americanum).

Pullen, R. 43, 2600 (triflorum); 3964 (chenopodioides); 8005 (americanum).

Pulley, J. 1234 (americanum).

Purdie, R.W. 2001 (triflorum); 6559, 6559 (americanum); 6711 (triflorum); 7081, 7081, 8316 (chenopodioides); 8386, 9350 (triflorum).

Purseglove, J.W. P308 (tarderemotum); P-2595 (memphiticum); P2712 (scabrum); P3035 (tarderemotum); P3121 (memphiticum).

Pusalkar, P.K. NC-104639 (americanum).

Put, N. 3548, 4188 (nigrum).

Qaiser 154 (villosum).

Qisheng Ma 88-0804 (nigrum).

Quarin, C. 357 (americanum); 1427 (pygmaeum); 2369 (americanum).

Quarré, P. 18, 2898 (tarderemotum); 3297, 3509 (scabrum).

Quayle, E.H. 375 (opacum).

Queiroz, E.P. 2161 (americanum).

Quevedo G, E. 33 (americanum).

Quevedo, F.L. 1794 (americanum).

Quibell, C.F. 1729 (americanum).

Quinlan, M.B. 51, 182 (americanum).

Quintas, F.J.D. 47 (americanum).

Quipuscoa S, V. 1583 (furcatum).

Quirico, A.-.L. 7 (americanum).

Radcliffe-Smith, A. 3884 (villosum).

Raes, N. 59 (americanum).

Ragazzi, V. 53, 158 (americanum).

Rai, S.K. BSHC-24575 (americanum).

Raimundo, A.F. 838 (americanum).

Rainha, B.V. 6177 (chenopodioides).

Raizada, M.B. 7426 (villosum).

Rajeurlar 31057 (americanum).

Raju, D.C.S. BSHC-4088 (americanum).

Rakotozafy, A. 59 (americanum).

Ralfs, P.H. 718 (chenopodioides).

Ram, B. 2184 (nigrum).

Ramamoorthy, T.P. 3593 (americanum).

Ramamurthy, K. 16047, 50680 (americanum).

Rambo, B. 29020, 42637, 43100 (americanum); 45745 (chenopodioides).

Ramírez B, A. 10 (americanum).

Ramírez H, A. 2 (americanum).

Ramírez R, R. 836 (americanum).

Ramírez, N. 2670 (americanum).

Ramírez-Delgadillo, R. 2668 (americanum).

Ramos, J.E. 442 (americanum).

Ramos, M. 27573, 40353 (nigrum).

Ramsbottom, J. x7c, x19 (villosum).

Rand, R.F. 171 (retroflexum).

Randriamampionona, B. 692 (scabrum).

Ranga, D. 245 (villosum).

Ranjan, ? 45320 (villosum).

Rankin, M. 2749 (americanum).

Rao, A.S. NC-63640 (americanum); NC-63680 (villosum).

Rao, A.V.N. 18242 (americanum).

Rao, R.S. 7454, 7857 (nigrum); 10291, 10316, 10857, 10879, 17489 (americanum).

Rao, T.A. NC-444, NC-626 (americanum); 626, 848 (villosum); NC-1061 (americanum); NC-4975, NC-4975, NC-6584 (villosum); NC-6584[b] (americanum); NC-7346 (villosum); NC-11009 (nigrum).

Rasingam, L. ANC-25856 (americanum).

Rathjens, C. 37/189, 37/205 (memphiticum).

Ratkowsky, D.A. 359 (nigrum).

Rattray, J.M. 560 (villosum).

Rau, M.A. NC-1318 (americanum); NC-3543, NC-8126 (villosum); NC-12625 (americanum); NC-14592 (villosum).

Raute-Horscher, W. P-72 (furcatum).

Raveill, J.A. 1682 (sarrachoides).

Raven, P.H. 1773 (furcatum); 2178A (nigrum); 19912 (americanum).

Rawi, A. NHI-10215 (villosum); 11679 (nigrum); NHI-19683, NHI-19706, NHI-19809, NHI-26567, NHI-29033 (villosum).

Ray, G.P. 2516 (villosum).

Raynal, A. 18654 (americanum).

Reading MSc Expedition 227 (villosum).

Reading University/BM Expedition 160 (villosum).

Rechinger, K. 278 (americanum).

Rechinger, K.H. 87, 3630 (villosum); 5640 (nigrum); 5710, 8060 (villosum); 8117, 8418 (nigrum); 13017, 16293, 16549, 16957 (villosum); 19350 (nigrum); 19498 (villosum); 24868 (nigrum); 27945, 28191, 28669, 29683, 29684, 29885, 30305, 30866, 31004, 34624 (villosum); 37815, 38210, 39047 (nigrum); 45775 (villosum); 61377, 61404 (nigrum).

Redfields, J.H. 5883 (triflorum).

Redgen, B. 48 (americanum).

Reed, C.F. 32667, 32796, 32880, 32891 (villosum); 39318 (sarrachoides); 104036 (villosum); 105844 (nigrum).

Reekmans, M. 3419 (memphiticum); 7400, 8444 (tarderemotum); 9831, 9922 (villosum).

Rees, M. 302 (nitidibaccatum).

Regnell, A.F. 970 (americanum).

Rego, L.N.A.A. 6 (americanum).

Reiche, C.F. 69 (furcatum).

Reid, J.C. 2717 (nitidibaccatum).

Reijenga, T.W. 995 (americanum).

Reina G, A.L. 97-446 (americanum).

Reitz, P.R. c72, 464, C693, 871 (americanum).

Rekas, K. 290 (nigrum).

Rendle, A.B. 71, 292, 396 (americanum).

Rensberg, H.J. van 1886 (tarderemotum).

Rentería A, E. 1624 (americanum).

Renvoize, S.A. 885 (americanum); 2312 (scabrum).

Resgate, E. 196 (americanum).

Reverchon, J. 3919 (triflorum).

Revilla, J. 11 (americanum).

Rex Smith, G. 9 (villosum).

Reyes García, A. 4947 (americanum).

Reyes S, J. 192 (americanum).

Reyes, P. 14270, 14356 (americanum).

Reyes-García, A. 2064 (americanum).

Reynoso D, J.J. 635 (americanum).

Reynoso, E. PPI-21644 (americanum).

Ribeiro, B.G.S. 1391 (americanum).

Rich, H.H. 26 (villosum).

Richards, A.F. 149 (americanum).

Richards, H.M. Mrs 1183, 4216, 4426, 6571, 9735 (tarderemotum); 9775B, 12233 (villosum); 13004, 13858 (tarderemotum); 16686, 18304 (villosum); 18770, 20349, 21308, 24130 (tarderemotum); 24245 (memphiticum).

Riches, C.R. 25 (americanum).

Richter, L. von 436 (chenopodioides).

Ricketson, J. 1861 (triflorum).

Ricksecker, Mrs Rev JJ 251 (americanum).

Ridley, H.N. 77, 14983 (americanum).

Riedel, L. 402[b] (americanum).

Rilke, S. 561 (palitans).

Rimachi Y, M. 4134, 7077, 8340, 8897, 10277, 10760, 11116, 11804 (americanum).

Rincón G, A. 1998 (americanum).

Ripart, J. 249 (nigrum).

Risler, J. 970 (americanum).

Ritchie, ? 508/2 (nigrum).

Ritter, N. 1124 (americanum).

Rivera H, J. 2164, 3708 (americanum).

Rivera Reyes, J. 415, 2545 (americanum).

Rob, E. 921 (tarderemotum).

Robbins, F. ACB-17123 (opacum); ACB-50381 (chenopodioides).

Roberts, E.C. 11388 (nitidibaccatum).

Robertson, S.A. 643 (villosum); 3454 (americanum).

Robinson, H.C. 86, 175 (americanum).

Robles, S.T. 584, 1860, 1898, 2096, 2139, 2277 (chenopodioides).

Robleto, W. 1039, 1412 (americanum).

Robson, N.K.B. 1427 (tarderemotum).

Rockley, C.T. 4 (chenopodioides).

Rocrlach, K.D. 290 (nigrum).

Rodarte, A. 2E (americanum).

Rodd, A.N. 442 (palitans); 455 (triflorum); 688 (americanum); 689 (opacum); 690, 691 (americanum); 1640 (chenopodioides); 1751 (americanum); 121632 (chenopodioides).

Rodin, R.J. 3500 (tarderemotum).

Rodrigues, J.E. 189 (americanum).

Rodríguez B, D. 101 (americanum).

Rodríguez C, A. 2133 (americanum).

Rodríguez, D. 372, 1345 (palitans).

Rodríguez, R. 4, 185 (americanum).

Rodschied, E.C. 31 (americanum).

Rodway, E. 121 (opacum).

Rodway, F.A. 121 (nigrum); 645 (opacum); 735, 1092, 1334, 1377 (americanum); 1661 (chenopodioides); 2140 (opacum); 2258, 2694 (nigrum); 4766, 6483, 6486 (americanum); 6487 (opacum); 6488, 6489, 6490, 6496, 11333 (americanum); 14904, 14940 (chenopodioides).

Roe, K.E. 2320 (americanum).

Roe, R. 695 (triflorum).

Roger, ? 17 (tarderemotum).

Rogers, C.G. 337 (tarderemotum).

Rogers, F.A. 08, 103 (villosum); 7402 (scabrum); 8225 (tarderemotum); 8256 (scabrum).

Rogers, ? (Mr). 14 (nigrum).

Roivainen, H. 2646, 2773 (triflorum).

Rojas, R. 882 (americanum).

Rojas, T. 2493 (sarrachoides); 5975 (americanum).

Roldan, F.J. 1293 (americanum).

Rolfs, P.H. 718 (chenopodioides).

Rolla, S.R. 88606 (villosum).

Román, M.L. 978, 1267 (americanum).

Romanos, A.E. 53 (furcatum).

Rombouts, H.E. 718 (americanum).

Romero, E. 70 (americanum).

Romero-Castañeda, R. 743 (americanum); 6386 (americanum).

Romo Díza, G. 1263 (americanum).

Rondeau, R. 134 (americanum).

Roque, J. 22, 87, 208, 396, 693 (americanum); 978, 1937 (furcatum).

Rosa, N.A. 434, 2189, 3548 (americanum).

Rosário, C.S. 1878 (americanum).

Rosas R, M. 1167 (americanum).

Rosas, A. 310 (americanum).

Rose, J.N. 12422 (americanum); 13811, 14134, 14363 (americanum); 14697, 14877 (americanum); 22532 (americanum).

Rose, L.S. 66088 (americanum).

Rosen, D.J. 5304 (americanum).

Rosengurtt, B. 5783 (chenopodioides).

Ross, R. SAN-128 (americanum); 489, 576 (tarderemotum); SAN-585 (americanum); 809, 840, 936 (tarderemotum).

Rossato, M. 3339, 3480 (americanum).

Rotman, A.D. 803 (palitans).

Roux, J.P. 2080 (nigrum).

Roy, G.P. AC-3863 (nigrum); 5060 (villosum); CC-34072 (nigrum).

Royce, R.D. 5846, 8408 (americanum).

Royl, ? 6826 (villosum).

Rubtzoff, P. 10202 (furcatum).

Rudatis, H. 1805 (retroflexum).

Rugel, F. 44 (americanum).

Ruiru Pest Control Staff 354 (villosum).

Ruiz, E. 1007 (furcatum).

Ruiz Huidobro, A.M. 1332 (pygmaeum); 1410 (triflorum); 1449 (chenopodioides); 1614 (pygmaeum).

Rusby, H.H. 753 (triflorum); 808 (palitans); 5981 (triflorum).

Russell-Smith, J. 5421 (americanum).

Rwaburindore, P.K. 701 (scabrum); 1060 (tarderemotum); 1705 (americanum); 2992 (scabrum); 3936, 4199 (americanum).

Ryan, P. 289 (scabrum).

Rydberg, P.A. 4905 (triflorum).

Ryding, P.O. 1128, 1536 (villosum).

Rzedowski, J. 39828, 40130, 46293, 49107, 51543 (americanum).

Saab, S.A.G.P. 184 (americanum).

Sabaya, W.D. 34 (villosum).

Sabeti 1018 (villosum).

Sabnis, S.D. 568 (villosum).

Saccardo, D. 35 (villosum).

Sachet, M.H. 2254 (americanum).

Sachse, B. 5 (retroflexum).

Safford, W.E. 1396 (americanum).

Safrui, B. 20 (americanum).

Sagástegui A, A. 11416, 14169 (furcatum); 16128 (americanum).

Sagot, P.A. 453, 493 (americanum).

Sahebi 2047 (villosum).

Sahmi, K.C. 25025 (americanum).

Sainty, G.R. 329 (triflorum).

Salasoo, H. 937, 1815 (chenopodioides); 3061, 3061 (palitans); 3333 (americanum).

Salavakan 3035 (villosum).

Saldanha, C.J. 13567 (villosum); 16110 (americanum).

Saldías, M. 172 (palitans).

Salm, H. 1 (americanum).

Salter, T.M. 9711 (retroflexum).

Salubeni, A.J. 4242 (tarderemotum); 4242 (scabrum); 4444, 4600, 4818 (tarderemotum); 4841 (villosum).

Salzmann, P. 391, 3855 (americanum).

Sampaio, A.F. 8955 (americanum).

Samuelsson, G. 1086 (villosum).

Sandberg, J.H. 985 (triflorum).

Sanders, A.C. 12462 (nitidibaccatum); 23119 (americanum); 37173 (retroflexum).

Sandino, J.C. 677, 777, 2731 (americanum).

Sands, M.J.S. 333 (americanum); 7296 (opacum).

Sandwith, C.I. 3169a (villosum).

Sandwith, N.Y. 1703 (americanum); 2395, 3413 (villosum).

Sankey, H.J. 200 (retroflexum).

Sanou, L.F. BUR-596 (scabrum).

Santapau, H. 204 (americanum).

Santo, E. 218 (scabrum).

Santos Martínez, J. 1510[a] (americanum).

Santos, J.K. 32073 (nigrum).

Sanyal, M.N. 649 (villosum).

Saran, R. 2429 (villosum).

Saravia T, C. 2398, 3428 (americanum).

Sarin, Y.K. NC-1501, NC-1509, NC-5159 (americanum); NC-8573 (villosum).

Särkinen, T. 4015, 4017, 4018, 4023, 4027, 4033, 4041, 4072 (americanum); 4076 (nitidibaccatum); 4080 (americanum); 4081, 4082, 4083, 4084 (furcatum); 4085 (nitidibaccatum); 4090, 4095, 4099 (furcatum); 4113, 4116 (americanum); 4117 (chenopodioides); 4505, 4511, 4514, 4520, 4528, 4534, 4543, 4555, 4574, 4579, 4619, 4622, 4632, 4649, 4818, 4823, 4862, 4867 (americanum); 4873 (furcatum).

Sartori, J. 86 (villosum).

Sasaki, S. 275 (nigrum).

Sastre, C. 2708 (americanum).

Saueressig, D. 1909 (americanum).

Saulleres, A. 63 (americanum).

Saunders, S.G.E. 335 (furcatum).

Savatier, L. 875 (nigrum).

Saxena, H.O. 525, 582, 2159 (americanum).

Sayago, M. 103 (pygmaeum); 554, 588 (palitans); 796 (nitidibaccatum); 2128 (pygmaeum).

Saynes V, A. 2245 (americanum).

Scarlett, N.H. 87-38 (americanum); 87-39 (nigrum).

Scatigna, A.V. 863 (americanum).

Schäfer, H. 5569, 9370 (nigrum).

Schafer, J.A. 3 (americanum).

Schäfer, P.A. 5306, 7053 (americanum).

Schäffler, Y. 55 (americanum).

Schaffner, J.G. 692[a] (americanum).

Schallert, P.O. 3765 (chenopodioides).

Schantz, H.L. 646 (scabrum).

Schatz, G.E. 1444 (americanum).

Scheepers, J.C. 54 (retroflexum); 281 (americanum).

Schiavone, M.M. 11681C (sarrachoides).

Schickendantz, F. 113 (sarrachoides).

Schiede, C.J.W. 46 (americanum).

Schimper, G.H.W. 46 (memphiticum); 74 (hirtulum); 129 (tarderemotum); 255 (villosum); 506 (memphiticum); 509 (tarderemotum); 523 (memphiticum); 631 (hirtulum); 826 (memphiticum); 977 (hirtulum); 2043 (villosum).

Schimpff, H.J.F. 218 (americanum).

Schindler, A.K. 1409 (nigrum).

Schinini, A. 4746 (chenopodioides); 5174, 5427 (americanum); 5380, 6420 (pygmaeum); 6806, 6845, 11102, 11276 (americanum); 16377 (sarrachoides); 18656, 19013 (pygmaeum).

Schinz, H. 806 (retroflexum); 907 (tarderemotum).

Schlagintweit, H.A.R. von 4368 (nigrum).

Schlechter, F.R.R. 2165 (scabrum); 16407 (opacum).

Schlieben, H.J. 413 (tarderemotum); 3550 (villosum); 4527 (tarderemotum); 7009 (retroflexum); 11670 (scabrum).

Schlim, L.J. 194 (americanum).

Schmidt, A.A. 592 (scabrum).

Schmitt, G. 163 (americanum).

Schmitz, A. 61 (americanum).

Schoenwetter, J. JSOX-58 (americanum).

Schneider, C.K. 705 (nigrum).

Schoch, O. 67 (nigrum).

Schodde, R. 5015 (opacum).

Scholten, G. 38 (nigrum).

Schott, A.C.V. 5410 (americanum).

Schreiter, R. 7410 (chenopodioides).

Schulte, M. 14 (palitans).

Schultes, R.E. 5139 (americanum).

Schultz, F.G. 705 (villosum).

Schultz, N. 166 (chenopodioides).

Schunke V, J. 1448, 4958, 6077, 6992 (americanum).

Schwarz, G.J. 1138, 1212, 4632, 4697, 8145 (americanum).

Schweinfurth, G.A. 59 (tarderemotum); 1259 (villosum); 1319 (memphiticum); 1402 (villosum); 1403 (tarderemotum); 1404 (nigrum); 1406, 1410 (villosum); 1411 (nigrum); 1476 (memphiticum); 2649 (tarderemotum).

Scoggan, H.J. 10224 (triflorum).

Scott, H. 240 (nigrum); 269 (villosum); 292 (hirtulum); 438 (nigrum); 491 (villosum).

Scott-Elliot, G.F. 173, 416 (furcatum); 3354 (nigrum); 6760 (memphiticum); 7248, 7659, 7821 (tarderemotum).

Scouler, J. 121 (americanum).

Seaman, C. 41 (sarrachoides).

Sears, R.R. 141 (americanum).

Sebastine, K.M. 1311, 2173, 2790, 4008, 6782, 8488 (americanum); 8954, 13928 (villosum); 15356, 16691, 24589 (americanum).

Seegeler, C.J.P. 2038 (villosum); 2399 (tarderemotum); 2472 (villosum); 3227 (tarderemotum).

Seemann, B.C. 81 (americanum); 344 (opacum).

Seibert, R.J. 1297 (nigrum).

Seidel, R. 2717 (americanum).

Seidenschwarz, F.G. 111 (americanum).

Seijo, G. 1869 (chenopodioides).

Seillant, ? 27 (palitans).

Seler, C. 204 (furcatum).

Sell, P.D. 671814 (nigrum).

Sellow, F. 225, 280 (chenopodioides).

Semajunde NC-73194 (americanum).

Senderayi, E. 226 (retroflexum).

Sengupta, G. 122 (americanum); 1221 (americanum); 1348 (americanum); CC-14548 (nigrum).

Sennen, E.C. 125 (nigrum).

Senni, L. 63 (memphiticum); 310 (americanum); 819 (memphiticum); 877 (villosum); 1107 (memphiticum); 1837 (hirtulum); 2182 (memphiticum).

Sequeira, M. 2790 (villosum).

Sérsic, A. 5040 (triflorum).

Setchell, W.A. 137 (americanum); 15374 (opacum).

Seto, K. 31840 (nigrum).

Seydel, R. 1162 (scabrum); 3783 (retroflexum).

Seymont 1060 (nitidibaccatum).

Seyrig, A. 163 (americanum); 217 (scabrum).

Shabani, S. 335, 513 (tarderemotum).

Shabetai, J.R. 190 (nigrum).

Shah, M. 170 (americanum).

Shankarmani 164 (nigrum).

Sharifi, M.R. 531 (nigrum); 540 (villosum).

Sharma, J.B. NC-67127 (americanum).

Sharma, J.P. NC-70364 (americanum); NC-77942 (villosum).

Sharma, S. S.S.359 (villosum).

Sharma, V.S. 583 (villosum).

Sharsmith, C.W. 3982 (nitidibaccatum).

Shaw, I. 112 (chenopodioides).

Shaw, R.J. 2994 (nitidibaccatum).

Sheik, A.H. 13176, 13329 (villosum).

Shepherd, C.J. 215 (nitidibaccatum); 718 (chenopodioides).

Sheppard, W.B. 76 (triflorum).

Sherif, I.M. A2891 (tarderemotum).

Shetty, B.V. 42 (villosum); 384, AC-566, AC-2214 (nigrum); 4107, 8134 (villosum).

Shevock, J.R. 11009, 11030 (americanum).

Shiki, D. 143 (americanum).

Shillingford, C.A. 209 (americanum).

Shillito, E.M. 58 (tarderemotum).

Shiota, K. 107, 3815, 3999, 4663, 9095 (nigrum).

Shipchinskii, N. 286 (villosum).

Shiu Ying Hu 5432, 6781, 7009 (americanum).

Shivas, R. 811 (americanum).

Short, M.J. 46 (nigrum).

Shu-hui Wu 1161 (opacum).

Shukla, A.N. NC-123920 (americanum).

Shukla, B.K. BSHC-22169 (nigrum).

Shukla, R.K. BSHC-18673 (americanum).

Si Boeea, R. 5991, 7308, 8026, 8301, 8611, 9227, 10755 (americanum); 10775 (scabrum).

Si Toroes, R. 407 (scabrum).

Sibil, J. 48, 171, 252, 317 (americanum).

Sidey, J.L. 1624 (retroflexum).

Siegenthaler, I.E. X28 (tarderemotum).

Siegert, R. 79 (opacum).

Siehe, W. 298 (villosum).

Sih-Chiu, M. 442 (nigrum).

Sikes, K.G. 36 (sarrachoides).

Sikdar, J.K. 126, 167 (americanum); CNH-236, CNH-4183 (villosum); 4303, 4338 (americanum).

Silva Filho, P.J.S. 716 (americanum).

Silva Neto, S.J. 1021 (americanum).

Silva, A.S.L. da 433 (altissimum).

Silva, C.A.S. 18 (americanum).

Silva, D.A. 4611 (torvum).

Silva, G.P. da 2259 (americanum).

Silva, J.M. 147 (americanum).

Silva, J.R. 4000 (paucidens).

Silva, L.A.M. 3509 (americanum).

Silva, N.C.B. 47 (americanum).

Silva, S.J. 981 (americanum).

Silveira, G.H. 493, 4176 (americanum).

Silverstone Sopkin, F.A. 2041, 3239 (americanum).

Sim, J. JS10 (americanum).

Simaga, J.M. 713 (americanum).

Simon, G. 698 (tarderemotum); 720 (memphiticum).

Simon, P.M. 165 (triflorum).

Simonds 32 (villosum).

Simonis, J.E. 125 (americanum).

Simpson, B.B. 8566 (furcatum).

Simpson, D.R. 439 (americanum).

Simpson, N.D. 16, 112, 1390, 1429, 2995, 3021, 3062, 3787i, 3787ii, 3852 (villosum); 4460 (nigrum); 4494, 4795 (villosum); 5354 (nigrum); 5456 (villosum); 5663, 5781 (nigrum); 6077 (villosum); 6334 (villosum); 8691, 8865, 9394, 9728, 28096, 28118, 34461, 34479 (nigrum); 53043 (villosum); 68010 (chenopodioides).

Sinclair, J. SF-40388 (americanum).

Singer, A. Soni-40708, Soni-80507 (nigrum).

Singh, A.N. 3264, 3282, 6232 (villosum).

Singh, D.K. NC-88573 (nigrum).

Singh, G. ANU-18340 (chenopodioides).

Singh, H. 528 (americanum).

Singh, K.N. 109 (nigrum).

Singh, M.P. NC-19653 (americanum).

Singh, N.P. NC-19452 (villosum); NC-23085, NC-31620, NC-31632, NC-31930 (americanum).

Singh, N.T. NC-25543 (villosum).

Singh, P. BSHC-15537 (americanum); BSHC-16449 (americanum).

Singh, R. 421 (villosum).

Singh, S. 34010, NC-89850 (villosum); NC-90276, NC-90499 (americanum).

Singh, U. 12, 42 (americanum).

Singh, V. 2988 (nigrum); 5397, 5618 (villosum).

Sinha, A.K. 43249 (americanum).

Sinha, G.P. BSHC-15201, BSHC-15236 (americanum); BSHC-16704 (nigrum).

Sino-American Expedition 429 (nigrum).

Sintenis, P. 636 (nigrum); 675, 1126 (villosum); 3573 (americanum); 7469 (nigrum).

Skottsberg, C. 260 (furcatum); 363, 526, 568, 663, 695 (opacum).

Skutch, A.F. 5331 (americanum).

Slanis, A.C. 139 (palitans).

Slater, C. 45 (americanum).

Slee, A.V. 4662 (triflorum).

Sleumer, H. 2091 (americanum).

Small, J.K. 607, 952, 3029, 3564, 3724, 4696, 4703, 5488, 5588, 5592, 7465, 8254, 8712, 10335, 10786 (americanum).

Smith, ? 69 (americanum).

Smith, A.C. 206, 1174, 4310, 4849, 9070 (opacum); 9450 (americanum).

Smith, C. 33 (americanum).

Smith, D.N. 6691 (americanum).

Smith, E.E. S&S85 (furcatum).

Smith, [K.A.]H. 4552, 6586, 6842 (nigrum).

Smith, H.H. 450, 639 (americanum).

Smith, J.F. 182 (americanum).

Smith, J.M.B. 15825 (triflorum).

Smith, L.B. 12573 (americanum); 13848 (chenopodioides).

Smith, L.S. 3071 (nigrum); 13884 (opacum).

Smith, N. 4382 (americanum).

Smith, P.A. 1080 (scabrum); 1494 (retroflexum).

Smith, R.V. 64/118, 64/118 (triflorum).

Smith, S.F. 743 (americanum).

Smith, S.G. 1141 (americanum).

Smith, W.W. 721 (americanum).

Smittenberg-Visser, L. 61 (scabrum).

Sneddon, B. 43 (nigrum).

Sneidern, K. von 4833 (americanum).

Sniderman, K. 8 (chenopodioides); 10 (nitidibaccatum).

Snowden, J.D. 564 (tarderemotum).

Soakai, E. 941 (americanum).

Sobrinho, F.A. 62 (americanum).

Socalski, N.D. 493 (villosum).

Sodombekov, I. KPL_00292 (villosum).

Sohmer, S.H. LAE-75542 (opacum).

Soibeh, D. 51 (americanum).

Solano, C. 506 (americanum).

Solling, K.L. 117 (americanum).

Solomon, J.C. 2697 (americanum); 4099 (nitidibaccatum); 4120 (chenopodioides); 4665 (triflorum).

Sorger, F. 80-41-4, 82-23-50, 84-72-100, 85-41-59 (villosum).

Soria, N. 1514, 2671 (americanum).

Soriano, A. 2606 (triflorum).

Sota, A. de la 4577 (americanum).

Soto N, J.C. 5275, 5802, 7252, 7253, 7319, 7346, 7998, 12894 (americanum).

Soukup, J. 2550, 4616 (americanum); 4622 (furcatum).

Souza, G.R. 1686 (americanum).

Souza, L.O.F. de 102 (americanum).

Souza, V.C. 4305, 12295 (americanum).

Sowerby, B. 204 (nigrum).

Sparre, B. 13011, 13014, 13212, 13218, 15262, 15423, 15561, 15562, 17945, 17994, 18107, 18112, 19055, 19454, 19816, 19833 (americanum).

Sparre, B.B. 5302 (pygmaeum).

Spegazzini, R.A. 56646 (nitidibaccatum).

Speke, J.H. 79, 453 (tarderemotum).

Spellenberg, R.W. 7174 (nigrum); 7258, 7328 (villosum).

Spellman, D.L. 1861 (americanum).

Spence, D.H.N. S233 (villosum).

Sperling, C.R. 5653 (americanum).

Spire, C.J. 1060 (americanum).

Spitzenberger, F. 180 (villosum).

Spooner, A.G. 3341 (nitidibaccatum); 5777 (*triflorum*); 6898 (nitidibaccatum); 14125 (triflorum).

Spruce, R. 3983, 3984 (americanum).

Srinivasan, S.R. 63653 (americanum).

Srivastava, N. NC-105544 (americanum).

Srivastava, R.C. BSHC-10371 (americanum); BSHC-10371[b[, BSHC-13167 (nigrum).

Srivastava, S.K. NC-73736 (nigrum); NC-96030 (villosum); NC-96090, NC-96751 (americanum).

St Clair-Thompson, G.W. 204 (scabrum); 854 (tarderemotum).

St. John, H. 1314 (nigrum); 4938 (triflorum); 14187, 14659, 15044, 15195, 15383, 15682, 16070, 16310 (opacum); 19374, 22763 (americanum).

Staff NC-857 (americanum).

Stainton, J.D.A. 53, 150 (nigrum); 3142 (villosum); 3384, 4959 (nigrum).

Stajsic, V. 4292, 4292, 4292, 4292 (furcatum).

Standley, J.P. 72 (americanum).

Standley, P.C. 4928 (triflorum); 25290, 32374, 32644, 39022, 46920 (americanum).

Stanes, E. 28148 (villosum).

Stanley, T. 602 (opacum).

Stapf, O. 2731 (villosum).

Staple, A.J. 17 (nigrum).

Starmuehlner, F. 270 (nigrum).

Stauffer, H.U. 48, 567 (tarderemotum); 957 (memphiticum).

Stearn, W.T. 113, 181, 271, 330, 365, 432, 483, 542, 584, 1000 (americanum); 1145, 1174 (villosum).

Steenis, C.G.G.J. van 7839 (alpinum); 24079 (retroflexum).

Stehmann, J.R. 6162, 6340 (americanum).

Steibel, P. 8662, 8676 (pygmaeum).

Steinbach, J. 5972, 8614, 8661, 8790 (palitans).

Steiner, E. 22 (nigrum).

Steinmann, V.W. 4052 (americanum).

Stern, S. 88, 301 (americanum).

Steudner, H. 734, 734 (villosum).

Stevens, G.W. 852 (triflorum).

Stevens, W.D. 2931, 7310, 9524, 10088, 10163, 14522, 15537, 15590, 15848, 16638, 20895, 21299, 21300, 21894 (americanum).

Stevenson, P. 1940 (americanum).

Steward, A.N. 3022 (americanum); 9691 (nigrum).

Stewart, A. 3406, 3407, 3409 (americanum).

Stewart, G. 497 (chenopodioides).

Stewart, K. sol1 (triflorum).

Stewart, R.B. E10 (tarderemotum).

Stewart, R.R. 19404A (nigrum).

Stewart, V. 3 (opacum).

Steyermark, J.A. 88968, 130943 (americanum).

Stoddart, D.R. 2198, 2249, 7126, 8103 (americanum).

Stolz, A. 384 (tarderemotum).

Stone, B.C. 4701[b], 14055 (americanum).

Stone, J. 4167 (americanum).

Story, R. 5799 (tarderemotum).

Strachey, R. 1 (nigrum); 1[b] (villosum).

Straka, G. 9 (americanum).

Street, J.K. 222, 249, 258, 306 (villosum).

Streimann, H. 8078 (americanum); NGF-44518 (opacum).

Strey, R.G. 4311, 4573 (americanum); 9409 (retroflexum); 10899 (tarderemotum); 11303 (americanum).

Strid, A. 2882 (scabrum).

Stuart, C. 77, 784 (opacum).

Stuckert, T. [J.V.] 40, 404, 578, 870, 4713, 4743, 4798 (pygmaeum); 5022 (triflorum); 9039, 12272 13438, 15161, 15584, 23341, 24046 (pygmaeum).

Sturgess, H.F. 6968 (palitans).

Subba Rao, G.V. 66 (americanum); 19565 (nigrum).

Subils, R. 35 (triflorum); 568, 994, 1615 (pygmaeum); 2333 (nitidibaccatum); 2615 (palitans); 3382, 3398 (pygmaeum); 3673, 3695 (palitans); 3825 (pygmaeum); 3941 (nitidibaccatum); 4059, 4076, 4108, 4113 (triflorum); 4168, 4269 (americanum); 4668 (chenopodioides).

Subramanyam, K. 142, 6473 (americanum); 7199, 64511 (villosum).

Suclli, E. 1140 (americanum).

Sucre, D. 1976, 4952, 6464, 7842, 9644, 9680 (americanum).

Sugden, A. 170 (americanum).

Suksdorf, W. 1480 (nitidibaccatum); 2318 (americanum); 12045 (triflorum).

Sulit, M.D. 16987 (americanum).

Sullivan, J.M. 102992 (sarrachoides).

Sumithraarachchi, D.B. 512 (americanum).

Summers, J.W. 249, 3510, 4481, 9448, 9897, 10626 (sarrachoides).

Sun, S.C. 634 (nigrum).

Sutherland, J. 167 (memphiticum); 281 (villosum).

Suzuki, M. 88-11167 (villosum); 88-20090, 88-20210, 92-40045 (nigrum).

Svendson, J. 400 (nigrum).

Swan, J.M. 128 (americanum).

Swann, E.L. A, C, 1931, K3258, K3259 (nitidibaccatum).

Swarbrick, J.T. SCA271 (tarderemotum); 2572 (scabrum); 2875 (tarderemotum); 7451 (nitidibaccatum); 10855 (chenopodioides); 13131 (americanum).

Swynnerton, C.F.M. 342, 481 (tarderemotum); 1794 (retroflexum).

Sykes, W.R. 87-T, 36-08 (americanum); 136/91 (campanulatum); 220/91 (diploconos); 221/91 (cajanumense); 276/86 (opacum); 514/K (americanum); 521/93 (*nigrum*); 897/K (americanum); 902/K, 937/K, 1055/K, 1057/K (nigrum); 1059/K, 1102/K, 1379/K, 1466/K (americanum); 175961 (americanum).

Symes, Y.E. 392 (tarderemotum).

Symoens, J.J. 8680 (tarderemotum).

Symon, D.E. SS-83, SS-85 (nigrum); SS-86 (nitidibaccatum); SS-89 (nigrum); SS-91 (*nitidibaccatum*); SS-92 (nigrum); SS-94, SS-95 (americanum); SS-99, SS-108, SS-109, 110, 111, 112, 113, SS-115, 117, 118, 119, 120, SS-125 (nigrum); SS-127 (americanum); SS-137, SS-138, SS-141 (nigrum); SS-142, SS-143 (americanum); 222, SS-265 (nigrum); SS-267, 268, SS-268, SS-270 (americanum); SS-276, SS-293, SS-294, SS-313, SS-314 (nigrum); SS-315 (americanum); SS-316, SS-318, 879, S-1142, 1165, 1181, NPYE-1604, 1944, 2089, 2109, 2134, 2918, 2921, 3430, 4065, 4531 (nigrum); 4717 (palitans); 4742, 4742 (triflorum); 4779, 4879, 4880 (americanum); 4954, 5449, 5462, 5486, 5599, 5945 (nigrum); 6090 (americanum); 6608, 6635, 6690 (nigrum); 6715 (palitans); 7263, 7618 (nigrum); 7626 (nitidibaccatum); 7627, 7631, 7633, 8012, 8164, 8845 (nigrum); 9807 (triflorum); 9922, 9929, 9931, 9952, 10310 (nigrum); 10628 (opacum); 10665 (americanum); 11275 (nigrum); 11515 (chenopodioides); 11521, 11525 (americanum); 11578, 11586 (opacum); 11587, 11777 (nigrum); 12144, 12151, 12829 (opacum); 13742, 14144, 14261 (nigrum); 14342 (opacum); 14613, 14618, 14855 (nigrum); 14882 (americanum); 15064, 15165, 15263 (nigrum); 15462 (chenopodioides); 15941, 16610, 16858, 16910, 17075, 18149 (nigrum); 35881, 35883 (scabrum).

Synge, P.M. 856 (tarderemotum).

Syngrassides, A. 244 (villosum).

Synnott, T.J. 737 (scabrum); 1404 (villosum).

Sørensen, T. 1756 (nigrum).

Taam Ying-Wah 1144 (americanum).

Taborda, I. 4 (americanum).

Tadesse Ebba 597 (tarderemotum); 627 (villosum).

Tadesse, E. 627 (villosum).

Tai, H.H. 81 (nigrum).

Tai, L.Y. 11835 (nigrum).

Takamatsu, M. 772 (americanum).

Takayama, H. 92-39004 (nigrum).

Tallantire, A.C. T-633 (tarderemotum).

Tamashiro, J.Y. 143, 1009 (americanum).

Tamura, M. 26714 (americanum).

Tanaka, T. 1760 (nigrum).

Tang Siu Ging 6978, 7322, 13156, 13358, 13526, 13981 (nigrum).

Tanner, R.E.S. 829, 924 (villosum); 1975 (tarderemotum); 3020 (villosum); 3622 (tarderemotum); 4530 (memphiticum); 4532 (villosum); 5617 (memphiticum).

Tap, N. HLF3387 (americanum).

Taquet, E.J. 298, 1145 (nigrum).

Tate, R. 258(219) (americanum).

Tawakali, E.J. 1566 (tarderemotum).

Taws, N.M. 592, 592 (americanum).

Taylor, C.M. 2045, 6684, 6706, 7161 (americanum); 10187 (furcatum); 11096, 11804, 12967 (americanum).

Taylor, G. 2014, 2158, 2503 (tarderemotum).

Taylor, H.C. 3205 (retroflexum).

Taylor, J. 15637 (americanum).

Taylor, M.J. 267 (triflorum).

Taylor, M.S. 2195 (americanum).

Taylor, R.J. 15637 (americanum).

Taylor, R.S. BS87-182, 291a, 291a, 1530, 1530 (triflorum).

Taylor, W.E. 100 (americanum).

Teague, A.J. 74 (retroflexum).

Telford, I.R. 7215 (americanum); 7352 (triflorum).

Téllez V, O. 1414 (americanum).

Ten, S. 12 (nigrum).

Teng, S.W. 259, 91053 (nigrum).

Tengwall, T.A. 40 (villosum).

Tenore, M. 152 (villosum).

Tepe, E.J. 2272 (americanum).

Terracciano, A. 187 [2206] (scabrum); 807, 984 [2207] (villosum); 987 [2201] (memphiticum); 1600 (villosum); 1706 (memphiticum); 1716 (villosum); 2027 (memphiticum); 2105, 2109, 2116, 2188 (villosum); 2536 (scabrum).

Terry, P.J. 3027 (americanum).

Thackery, F.A. 524 (americanum).

Thamer NHI-46781 (nigrum); NHI-47404 (villosum).

Thode, V. 91 (americanum).

Thomas, A.S. Th4070 (scabrum); Th4266 (tarderemotum).

Thomas, D. 1005 (villosum); 69805 (americanum).

Thomas, D.W. 1239, 2997 (scabrum); 4642, 9348 (pseudospinosum).

Thomas, J.H. 10878 (nitidibaccatum).

Thomas, M.B. MT-551 (americanum).

Thomas, N.W. 2042, 3703 (scabrum); 4396 (americanum); 4909 (scabrum); 5435 (tarderemotum); 6408, 9638 (scabrum).

Thomas, R. 661 (americanum).

Thomas, R.D. 95065, 126797, 151462 (americanum).

Thompson, E.J. 168 (opacum).

Thompson, J. 1138 (opacum).

Thompson, J.W. 485 (triflorum); 3663 (nigrum); 3729 (americanum); 3766b (nitidibaccatum); 3802 (americanum); 11907, 11984 (triflorum).

Thompson, M.F. 134 (retroflexum).

Thompson, O. 157 (opacum); 159 (chenopodioides).

Thompson, S.A. 1753 (scabrum).

Thomson, P.J. 481 (scabrum).

Thomson, T. 62 (nigrum); 62[b] (americanum); 62[c] (villosum); 62[d] (nigrum); 62[e] (villosum).

Thorpe, M.J. 377, 384 (opacum).

Thothathri, K. 9960 (nigrum); 10461 (americanum)*.*

Threlfall, W. 10 (villosum).

Thulin, M. 1048, 1048 (tarderemotum); 3914 (hirtulum); 7172, 10906 (tarderemotum).

Thuret, G.A. 12 (villosum); 334 (nigrum).

Thurston, H.R. 3195 (americanum).

Tiehm, A. 6218 (triflorum); 6219 (nitidibaccatum); 6255, 7412 (triflorum); 10921 (nitidibaccatum); 15075 (triflorum); 16510 (nigrum).

Tilden, J.E. 78 (americanum); 832 (nigrum).

Till, W. 71 (nigrum).

Timaná, M. 2008, 2203, 2214, 2226, 2678, 3251 (americanum).

Timberlake, J.R. 1635 (villosum); 4521 (retroflexum).

Tink, E. 266 (americanum).

Tirado, N. 18, 76 (americanum).

Titov, V.S. 2220 (nigrum).

Tiwari, G.C. 35A (americanum).

Tiwari, G.L. AC-836 (nigrum).

Tiyoy, L. 1209 (tarderemotum).

Tlapa A, M. 438 (americanum).

To Hara 79/41, 115/35 (nigrum).

Tobey, C. 817 (nigrum); 2244 (villosum).

Todaro, A. 874 (villosum); 875 (nigrum).

Tonduz, A. 148b, 1537, 4237, 8711 (americanum).

Topa, E. 2684 (villosum).

Toppin, S.M. 82 (nigrum).

Toriz A, G. 716 (americanum).

Torre, A.R. 2214 (villosum); 4957 (retroflexum).

Torrecillas, E. 200 (americanum).

Torres C, R. 2336, 15288 (americanum).

Toscani, H.L. 50 (chenopodioides).

Toth, J. 3 (triflorum); 8 (opacum); 9 (americanum).

Tothill, B.H. 2393 (tarderemotum).

Tothill, J.D. 152 (memphiticum).

Tovar, J.D. 4 (americanum).

Tovar, O. 254, 268, 635, 646 (furcatum).

Townsend, C.C. 85/4 (villosum).

Tracy, S.M. 32a (americanum).

Train, P. 1838 (triflorum).

Trapnell, C.G. 1453 (scabrum).

Travers, W.A. 116 (nigrum).

Tregrubov, V. 168 (nigrum).

Tressens, S.G. 6401 (americanum).

Trethewy, A.W. 83, 183, 193, 262 (villosum).

Triana M, L.A. 266 (americanum).

Triana, J.J. 3855 (americanum).

Tribedi, G.N. 1, 201 (americanum).

Troiani 3431, 3648A (triflorum).

Troncoso, N. 1312 (pygmaeum).

Troncoso, N.S. 2288 (americanum).

Trott, A.C. 90 (villosum); 982A (nigrum); 1283 (villosum).

Trujillo Vasquez, R. 43 (americanum).

Tsai, H.T. 52294, 52833, 53616, 54308 (nigrum); 54780, 54844 (americanum); 55155 (villosum); 56071, 56549, 57961, 58490, 58839, 59074, 60501, 62021, 63005 (nigrum).

Tsang, W.T. 231, 331, 412 (americanum); 16639, 20890 (nigrum).

Tsi Zhuanhuo 91-390, 92-162 (americanum).

Tsiang Ying 133 (nigrum); 1968 (americanum); 10817 (nigrum).

Tsiang, Y. 16371 (americanum).

Tsugaru, S. B-2383, 3378 (americanum); 7604 (chenopodioides); 7717 (nigrum).

Tullgren, A. 95 (villosum).

Tun Ortíz, R. 34, 1168, 1490 (americanum).

Turner, D. DT113/77(2) (villosum).

Turner, S.R. 15-100 (nigrum).

Tweedie, J. 34 (pygmaeum); 38, 39 (chenopodioides); 50 (palitans); 432 (sarrachoides); 441 (palitans).

Tweedie, M. [Mrs] 725 (tarderemotum); 1068 (memphiticum); 1480, 1481 (tarderemotum); 1586 (memphiticum).

Tweedy, F. 831 (villosum).

Twisselmann, E.C. 11634 (nigrum); 17685 (nitidibaccatum).

Tyrer, P.J. 110 (villosum).

Tyrrel, A. 24 (americanum).

Tyson, E.L. 1732, 2136, 5103, 6303 (americanum).

Ucán Ek, E. 2270, 3247, 3523, 4390 (americanum).

Udulutsch, R.G. 196 (americanum).

Ugent, D. 687, 2717 (americanum).

Uhe, G. 1126 (opacum).

Ule, E. 4310 (chenopodioides).

Ulibarri, E.A. 457 (sarrachoides).

United Fruit Company 228 (americanum).

Universidade dos Açores-NHM 37, 46 (nigrum).

Uniyal, B.P. NC-61013 (americanum); NC-80282 (villosum); NC-91885, NC-92625 (americanum).

Uong Sing Po 12110 (nigrum).

Uotila, P.J. 19873, 19926, 20709b, 20780 (villosum).

Urdampilleta, J.D. 717 (palitans).

USA Typhus Commission 71 (americanum).

Utitiay, A. 0 (americanum).

Vainio-Mattila, K. 95-9 (americanum); 95-53 (tarderemotum).

Valencia, N. 051, 052 (furcatum).

Valenzuela, L. 468, 5148, 6650, 6785, 12648 (americanum).

Valeur, E.J. 138 (americanum).

van der Werff, H. 15742 (americanum).

Van Devender, T.R. 93-300, 93-378, 93-1012 (americanum).

Van Someren, G.R.C. 11980 (memphiticum).

Van Someren, V.G.L. 7644 (memphiticum).

Vandenberg, J. NGF-40062 (opacum).

Vanni, R. 2304 (sarrachoides).

Vanni, R.O. 679, 804 (americanum).

Vanoverbergh, M. 3542 (nigrum).

Varela, F. 687 (chenopodioides).

Vargas C, I.G. 2444 (palitans); 2663 (americanum); 4559, 4559 (palitans).

Vargas R, C. 214 (americanum).

Vargas, C. USM14721 (furcatum).

Vargas, I.G. 6185 (americanum).

Vargas, P. 304 (villosum).

Vajravelu, E. 22435 (americanum); 60641 (villosum).

Valdemarin, K.S. 686 (americanum).

Vale, G.D. 101 (americanum).

Válka, R.J. 1477 (americanum).

Vasey, G.R. 352 (triflorum).

Vásquez, E. 192 (americanum).

Vásquez, R. 13379, 20795, 25177 (americanum).

Vasudeva Rao, M.K. ANC-7429, ANC-13066 (americanum).

Vatova, A. 101, 1854 (villosum); 2417 (memphiticum).

Vaughan, J.H. 1205 (americanum).

Vaupel, F. Sol-3 (opacum).

Vauthier, M. 537 (americanum).

Vázquez Hernández, J.P. 351 (americanum).

Vega A, R. 4278 (americanum).

Vega, C. 870 (pygmaeum).

Vegetti, ? 746 (americanum).

Velayos, M. 9450 (nigrum); 9695 (villosum); 10893 (nigrum); 10916 (villosum); 11623 (americanum).

Vélez N, M.C. 743 (americanum).

Vélez, C. 6333 (americanum).

Véliz, I. 1357 (americanum).

Véliz, M. 92-2183 (americanum).

Vendruscolo, G.S. 721 (sarrachoides).

Ventania, P. 870 (chenopodioides).

Ventura, E. 2273, 3468 (americanum).

Venturi, S. 159 (palitans); 350 (pygmaeum); 2239 (palitans); 5005 (chenopodioides); 40368 (palitans).

Verdcourt, B. 564 (villosum).

Verdon, D. 121 (opacum).

Verma, A.K. BSHC-3568 (nigrum); BSHC-4226, BSHC-6728 (americanum); CC-30761 (nigrum).

Verma, D.M. CC-1816, 6728 (villosum).

Versteegh, C. BW-12516 (opacum).

Vervoorst, F. 3479 (sarrachoides); 5723 (triflorum).

Vesey-Fitzgerald, L.D.E.F. 7540, 17068/3 (villosum).

Vianna, E.C. 146 (americanum).

Vibrans, H. 4449, 4544, 4968, 6260, 6933, 7315 (americanum).

Vicherek, J. 1554 (nigrum).

Vickery, R. 1472 (nitidibaccatum).

Vidal López, M. 67 (villosum).

Vidyamayi, ? 1134 (villosum).

Vieira, R.F. 1676 (americanum).

Vietnam Highland VH-5351 (americanum).

Vignoli-Silva, M. 239 (americanum).

Vilcapoma, G. 42, 283, 5981 (furcatum).

Villamil, C.B. 11706 (pygmaeum).

Vincent, M.A. 5004 (sarrachoides).

Vink, W. 16311 (opacum).

Virgo, K.J. 130 (nigrum).

Visher, S.S. 3326 (triflorum).

Viswanathan, M.V. 55656 (villosum); 55656[b] (villosum);.

Viswe, A.K. BSHC-4765 (americanum).

Vitek, E. 02-18 (nigrum); 97-25 (villosum); 96-136 (nigrum); 99-269 (villosum); 96-381 (nigrum); 98-1622 (villosum); 03-1710 (nigrum).

Viveros, J.L. 215 (americanum).

Vogel, J.R.T. 8 (americanum).

Vohra, J.N. NC-6035A, NC-9900, NC-11244, NC-58005 (americanum); NC-78290 (villosum).

Volkens, G. 1909 (tarderemotum); 2108 (memphiticum).

Von Platen, L. 149 (triflorum).

Voogd, C.N.A. de 2654 (alpinum).

Vorontsova, M.S. 162 (americanum); 184, 185 (scabrum); 194, 198 (tarderemotum); 200 (villosum); 1647 (tarderemotum).

Votava, F. 36 (americanum).

Vreeland, F.K. 638 (triflorum).

Vueeden, D.V. 97 (retroflexum).

Wadwha, B.M. 216, 786 (villosum); CC-5211, CC-7495 (nigrum); NC-52902, NC-52914, (americanum); NC-57375, NC-60184 (villosum); NC-60187, NC-62972 (americanum). NC-63483, NC-66840, (americanum). NC-83715 (nigrum); NC-85432 (americanum).

Wagenknecht, R. 18532 (furcatum).

Wagner, W.L. 5351 (opacum); 5708 (americanum).

Walker, E. .P. 333 (triflorum).

Walker, E.H. 5442 (nigrum).

Walker, E.P. 333 (triflorum).

Walker, H.A. 178 (americanum).

Wallace, K.L. 346 (americanum).

Wallich, N. [cat. #s] 197, 2615E (americanum); 2615 (nigrum); 2615[Y] (americanum).

Wallnöfer, B. 4213 (villosum); 4343 (nigrum).

Walsh, N.G. 999, 999, 2254 (opacum); 2255 (americanum); 3644 (chenopodioides); 4629 (opacum); 7011 (americanum).

Walter, B.M.T. 4143 (americanum).

Walter, D. 9008 (nitidibaccatum); 9264, 11528 (triflorum).

Walter, J. 01/0245A (villosum); 4135, 4136 (nigrum); 4189 (nitidibaccatum); 4674 (nigrum); 5583, 5595 (villosum); 7085, 7086 (nitidibaccatum); 7509b, 7509a (nigrum); 8946 (villosum); 9163a (nigrum).

Walton, D. 186 (villosum).

Wang Zhong-tao 87-0016 (nigrum).

Wang, ? 399 (americanum).

Wang, B.M. 050 (nigrum).

Wang, C. 33561, 35331 (americanum); 43427 (nigrum); 44166 (villosum).

Wang, C.M. 2550 (nigrum).

Wang, C.W. 62905, 66363, 67054, 69255, 73077, 74462 (nigrum).

Wang, C.Y. 7567 (nigrum).

Wapstra, M. 691, 691 (opacum); 1115 (triflorum).

Ward, D.B. 6404 (americanum).

Warren, R.C. 18 (villosum).

Warrick, R.B. 431 (americanum).

Wasum, R. 1084 (chenopodioides); 1941 (americanum); 4079 (chenopodioides).

Wasum, R.A. 3993 (americanum); 4298 (sarrachoides).

Waterfall, U.T. 15748 (americanum).

Waterhouse, B.M. 1273, 5401, 5401, 5466, 5519, 5519, 5956 (americanum).

Waterhouse, J.H.L. 691B (opacum).

Waterlot, E.G. 407 (scabrum).

Watermeyer, A.M. 123 (villosum).

Watson, H.C. 146, 146b (nigrum).

Watson, J. 12 (chenopodioides).

Watson, J.M. 744 (villosum).

Watt, G. 7053 (scabrum).

Wawra, H. 719, 2599 (americanum).

Webb, L.J. 10256 (opacum).

Webb, P.B. 44 (villosum).

Webster, G.L. 15941 (americanum).

Weigend, M. 2000579 (furcatum).

Weight, K.E. Kew907Z, 935Z (triflorum).

Welch, B. 5298 (sarrachoides).

Wellman, F.C. 1776 (tarderemotum).

Welman, M. 369 (retroflexum).

Welwitsch, F.M.J. 226 (nigrum); 6033 (scabrum); 6034, 6035, 6036b, 6036 (tarderemotum); 6053, 6099, 6100, 6101, 6102, 6103, 6103, 6108 (scabrum); 6109 (tarderemotum).

Wendelbo, P. 1475, 14504 (nigrum).

Wenzel, C.A. 242 (nigrum).

Werdermann, E. 359 (furcatum).

Werger, M.J.A. 236 (retroflexum).

Wesche, K. 500 (tarderemotum).

Westaway, J.O. 3656, 4018 (americanum).

Western, R. 135, 197 (villosum).

Weston, S.C. 67 (triflorum).

Westphal, E. 1227 (tarderemotum); 1483 (villosum); 1589, 1643 (memphiticum); 1674 (villosum); 1729 (memphiticum); 1735 (tarderemotum); 1749, 1924 (villosum); 2453 (tarderemotum); 2548, 2616 (villosum); 2617 (memphiticum); 2740 (villosum); 2805 (memphiticum); 2908, 3496, 3963 (villosum); 3989, 4042 (memphiticum); 4043 (tarderemotum); 4044 (memphiticum); 5499 (tarderemotum); 8733, 8739, 8758, 8765, 8766, 8803, 9429, 9660, 9675, 9700, 10014 (scabrum).

Westphal-Stevels, J.M.C. 1989 (scabrum).

Westra, L.Y.T. 179 (villosum).

Westwater, M. 192954 (scabrum).

Weyland, M.C. 447, 2040 (americanum).

Whalen, M.D. 853, 895 (americanum).

Wheeler Haines, R. 12, W-1359 (villosum).

Wheeler, J.M. ANU-5730 (opacum).

Wheeler, L.C. 3994 (nitidibaccatum); 3995 (triflorum); 5554 (nitidibaccatum); 8165 (americanum).

Whinray, J.S. 998, 1085 (opacum).

Whissen, P.S. 4742 (triflorum).

Whistler, A. 1929, 2589, 4675 (americanum); 5576 (opacum); 6746, 8436, 8858, 10810, 12899 (americanum).

Whitcombe, R. 183, 464, 815, 873 (villosum).

White, C.T. 13009 (opacum).

White, J.C. 100 (americanum).

White, O.E. 1091 (americanum).

White, S. 731 (americanum).

Whitefoord, C. 3678 (americanum).

Whitehead, B. 7 (americanum).

Whiting, M.M. 35-60 (villosum); 100 (americanum).

Whitmee, S.J. 22 (americanum).

Whittaker, J. 87, 601 (nigrum).

Wiadrowski, R. 1 (triflorum).

Wickens, G.E. 1044 (tarderemotum); 1191, 1711 (villosum); 1776 (tarderemotum); 2391 (villosum).

Wieringa, J.J. 4971 (tarderemotum); 6785 (triflorum).

Wierzbicki, P. 393, 2375 (nigrum).

Wiesbaur, J. 2239 (villosum).

Wiggins, I.L. 126, 547, 4731, 5678, 18265 (americanum).

Wight, R. 2010 (nigrum); 2326 (americanum).

Wilbur, R.L. 484 (opacum); 36451, 36689 (americanum).

Wild, H. 539, 3961 (retroflexum); 40771 (tarderemotum).

Wilde, J.J.F.E. de 5 (hirtulum); 12, 24 (scabrum); 204 (memphiticum); 1839 (villosum); 4502 (tarderemotum).

Wilde, W.J.J.O. de 5118 (tarderemotum); 5377 (villosum); 5504, 6029 (memphiticum); 6507 (tarderemotum); 7356, 8017 (memphiticum).

Wilford, C. 68 (americanum).

Williams, D.E. 959 (americanum).

Williams, G.R. 240 (memphiticum); 241 (tarderemotum).

Williams, I. 2744 (retroflexum).

Williams, L. 5831 (nitidibaccatum); 12825 (americanum).

Williams, L.D. 379, 4112 (triflorum).

Williams, L.H.J. 8336 (nigrum).

Williams, R.P. 1139, 1351 (triflorum).

Williams, R.S. 1079 (americanum).

Williams, S.L. 287 (scabrum); 1079 (retroflexum).

Williamson, B. 502, 533 (villosum).

Williamson, C. 48 (retroflexum).

Williamson, J. 53 (villosum).

Willian, J.G. 258[a] (americanum).

Willkomm, H.M. 475 (nigrum); 637 (villosum).

Wilman, M. 3140, 3192 (retroflexum).

Wilmott, A.J. D207 (nigrum).

Wilms, F. 1022a, b1022, 3458 (retroflexum).

Wilson, C.T. 77 (scabrum).

Wilson, E.H. 2660 (nigrum).

Wilson, J. 19 (americanum).

Wilson, J.G. 370 (villosum); 609 (tarderemotum); 633 (villosum).

Wilson, K.L. 1254 (americanum); 1276, 2410, 2410 (chenopodioides); 3842 (americanum); 4417, 4417 (palitans); 5881 (opacum).

Wilson, P.G. 18 (chenopodioides); 19 (opacum); 3597 (nigrum); 6069, 6249 (americanum).

Windham, M.D. 92-271, 91-271 (nitidibaccatum).

Windler, D.R. 979, 2731b (americanum).

Winkler, H.J.P. 3856 (tarderemotum).

Winter, B. de 3955 (tarderemotum); 5550 (scabrum).

Wissman, H. von 3230 (nigrum).

Wit, ? de A-2921 (scabrum).

Witsell, T. 07-605 (sarrachoides).

Witte, G.F. de 2757 (americanum); 10287, 10477 (tarderemotum).

Wolf, C.E. 3837 (americanum).

Wolley-Dod, A.H. 56A (nigrum); 56 (villosum); 336 (americanum); 856 (retroflexum); 1542, 1595 (villosum); 1606 (nigrum); 3180 (scabrum); 3318 (retroflexum).

Wolston, A. 856 (americanum).

Womersley, J.S. 564, NGF-13346, NGF-24678 (opacum).

Wood, D. 815 (tarderemotum); 1445 (americanum).

Wood, J.R.I. 72-7, Y/74-20 (memphiticum); 71-27 (villosum); S/73-65 (memphiticum); Y/74-73, S/72-84, Y/74-265, Y/75-742, Y/75-757, 2157, 2193, 2206, 2293 (villosum); 13577, 17194, 21698 (americanum); 21970 (palitans).

Wood, K.R. 11179 (opacum).

Woodruff, M. H-1990 (americanum).

Woodson, R.E. 942 (americanum).

Woolls, W. 49 (opacum).

Wooton, E.O. 214 (triflorum).

Worth, C.R. 15704, 16487 (furcatum).

Woytkowski, F. 8152 (americanum).

Wright, A.E. 629 (americanum); 853 (nigrum); 1204 (americanum); 1252 (opacum); 1405 (americanum); 1698 (opacum); 1896 (nigrum); 2189 (opacum); 2484, 2532, 2920 (nigrum); 3438 (americanum); 4237 (opacum); 4848 (nigrum); 4953 (americanum); 5034 (opacum); 5237 (nigrum); 5311 (americanum); 5465 (nigrum); 5828, 6233 (americanum); 6271 (opacum); 6343, 6518 (americanum); 7054 (nigrum); 7285, 7755, 8842 (americanum); 8876 (nigrum); 11422 (americanum); 12532 (chenopodioides); 12984, 12991 (nigrum).

Wright, C. 47 (villosum); 196, 347, 383 (americanum); 1077 (nigrum).

Wright, W.G. 1256 (americanum).

Wrigley, T.C. 188, 426, 493 (scabrum).

Wuang, S.T. 399 (americanum).

Wullschlägel, H.R. 374 (americanum).

Wunderlin, R.P. 5386, 8418 (americanum).

Wyld, J.W.G. 347, 842 (scabrum).

Wylie, J. 3420 (chenopodioides).

Xiao Bai-Zhong 4195, 4230 (nigrum).

Xu Ren-xin 40 (nigrum).

Yao, K. 8384, 8509, 8611 (nigrum).

Yarupaitán, G. 806 (furcatum).

Yates, C.C. 56 (scabrum).

Yates, H.S. 2622 (nigrum).

Yatskievych, G.A. 96-88 (sarrachoides).

Yepes A, S. 1118 (americanum).

Yonekura, K. 3230 (opacum); 3620, 97453 (americanum).

Yorimitsu, T. FOS-5704 (nigrum).

Young, A.L. 112 (opacum); 152 (americanum).

Young, T.P. 1032 (villosum).

Youthed, G. 790 (chenopodioides).

Yu, T.T. 564, 8530, 13333, 17345 (nigrum).

Yugovic, J.Z. 218 (furcatum).

Yuncker, T.G. 5670, 9632, 15272, 18015 (americanum).

Záchia, R.A. 3097 (americanum).

Zak, V. 1980 (americanum).

Zanoni, T.A. 11466, 30178 (americanum); 47320 (nigrum).

Zardini, E.M. 2712, 3385, 3426, 4974, 5335, 5701, 5753, 5756, 5792, 6642, 6650, 6983, 11390, 13465, 13605, 14630, 15600, 16042, 20517, 21222, 22107, 22282, 22465, 22745, 28234, 28350, 32112, 32116, 32856 (americanum); 40428 (sarrachoides); 54796, 55288 (americanum).

Zárgani 14236-E (villosum).

Zarucchi, J.L. 2623, 4006 (americanum).

Zavala-Gallo, L. 180 (triflorum); 273 (pygmaeum).

Zeller, S.M. 1143 (nitidibaccatum).

Zeyher, C.L.P. 3473 (retroflexum).

Zhu, G.H. 2228, 2897 (nigrum).

Zohrab, J. 57 (nigrum).

Zoller, C. 1991.184 (americanum).

Zollinger, H. 678 (nigrum); 1279 (americanum); 1790, 2177 (alpinum); 2177[bis] (nigrum); 2255, 2514 (alpinum).

Zöllner, O. 8950, 14543 (furcatum).

Zuccarini, J.G. 186 (villosum).

Zuloaga, F.O. 5577, 6653 (americanum); 9388, 10832 (palitans).

Zunguze, D. 113, 597 (americanum).

Zwickey, A.L. 148 (nigrum).
