# Supplementary material for: A revision of the Old World Black Nightshades (Morelloid clade of Solanum L., Solanaceae)
Source: PhytoKeys. 2018 Jul 25;(106):1–223. doi: 10.3897/phytokeys.106.21991 (PMC6070582; doi:10.3897/phytokeys.106.21991)
Supplement: Supplementary material 3 — Specimens examined (PDF format) [file phytokeys-106-001-s003.pdf]

Appendix 3. Specimens examined

Note: Specimens are not listed alphabetic within major areas by collector.

Solanum alpinum

INDONESIA. Sin. loc, *Without Collector s.n.* (L); **Bali**: bei der Quelle Jaritie auf Weg zum Gunung Ajaung, 2 Jun 1912, *Arens 19* (L); Kleine Soenda Eilanden, Bali, Z. helling G. Agoeng, 6 Apr 1936, *van Steenis 7839* (K); **Java**: Central Java, Blumbang, Mt. Lawu, Central Java, 26 Nov 1982, *Afriastini 475* (A); West Java, MtMalabar, Oct 1861, *Anderson 367* (CAL); West Java, MtMalabar, Oct 1861, *Anderson 369* (CAL); West Java, G[unung] Guntar., 1861, *Anderson 432* (CAL); East Java, Ardjoeno, tjemarabosch boven Lalidjiwo, 17 Oct 1915, *Arens s.n.* (L); East Java, 12 Oct 1915, *Arens 48* (L); East Java, Pasoeroean, G[unung] Tengge, boven Tosari, 4 Jun 1913, *Backer 8380* (L); East Java, Te Pasoeroean, Ngadisari, Jan 1925, *Backer 36563* (A); East Java, Pasoeroean, S. Tengge, boven Tosari, *Backer 36564* (L); Central Java, Soerkarta, Top van de Lawoe, 16 Jul 1936, *Brinkman 754* (NY); Sitiebondo, G[unung] Raneg [Raoeng] via Brembeinri, 15 May 1932, *Clason-Laarman 157* (L); East Java, south east Java (CAL sheet has locality Malawar, Praesingar, 6000ft[?] but very hard to read), 18 Mar 1880, *Forbes 1019* (BM, CAL); Central Java, Central Java, Slamet Mountain, 17 Mar 2004, *Hoover et al. 113* (A); Central Java, MtPrahu, *Horsfield s.n.* (BM); Central Java, Surakarta, *Horsfield s.n.* (BM); Central Java, MtPrahu, *Horsfield s.n.* (BM); Central Java, Blambangan & Mt. Prahu, *Horsfield s.n.* (BM); sin. loc, *Horsfield s.n.* (K); sin. loc, *Horsfield 5* (K); Sello, purchased 1859, *Horsfield 5* (K); Sin. loc, 1802, *Horsfield 7* (CAL, K); Sin. loc, 1802, *Horsfield 12* (K); East Java, Pasoeroean G[unung] Smeroe, *Jesweit, J s.n.* (L); sin. loc, *Junghuhn s.n.* (L); Sin. loc, *Junghuhn 405* (K, L); Central Java, Tenggu, Penandjaan, 31 Jul 1932, *Kleinhoonte 371* (L); Sin. loc, 20 Oct 1899, *Koorders 37852B* (K); East Java, Res. Besoeki Kawah Idjen, 1 Nov 1916, *Koorders & Koorders-Schumacher 43214B* (L); Central Java, Besoeki, G[unung] Merapi, 18 Jul 1916, *Koorders & Koorders-Schumacher 43218B* (L); East Java, Besoeki, Jang Plateau, 12 Aug 1916, *Koorders & Koorders-Schumacher 43463* (L); East Java, Tdjan plateau, Mt. Raung, Nov 1938, *Kostermans s.n.* (L); Central Java, G[unung] Muria, Tjollo, N of Kudus, 25 Nov 1951, *Kostermans 6278* (L); West Java, Malawar, Dec 1875, *Kuntze 5410* (DUKE, NY); East Java, Bromo, 15 Sep 1875, *Kuntze 5999* (GH, K, NY); West Java, Gunung Salak, 6 Sep 1863, *Kurz 682* (CAL); West Java, Gunong Gede, 6 Mar 1979, *Murata et al. J-2021* (L); East Java, Pasoeroean, 4 Jun 1935, *Neth Ind Forest Service 7038* (L); East Java, Pasoeroean, 4 Jun 1935, *Neth Ind Forest Service 7056*(A); East Java, Tosari 49, *Proefstation voor de Javasuikerindustrie s.n.* (L); East Java, Tosari 52, *Proefstation voor de Javasuikerindustrie s.n.* (L); Central Java, Central Java, G. Merbaboe, Jul 1922, *Roorda van Eysinga s.n.* (MO); East Java, Tosari, Pasoeroean, *Teysmann s.n.* (CAL, K); East Java, Tengergebergte tusschen Ngadisari, Zandzee en Tosari, *Went s.n.* (L); Central Java, Meapi, Catu septr, *Without Collector s.n.* (U); Iter Java secundum, *Zollinger 1790* (LE, P); East Java, Waliran, 27 Aug 1844, *Zollinger 2177* (A, BM, BM, G, G-DC, MPU, P); East Java, in montibus Ardjune, 14 Sep 1844, *Zollinger 2255* (BM, G, G-DC, L, MPU, P, P); East Java, Tengger, above Gebok-Klacca, Oct 1844, *Zollinger 2514* (BM, G, G, G-DC, P, W); **West Nusa Tenggara**: Lombok, Rindjani Vulkangebirge N Seite, 9 May 1909, *Elbert 1327* (L); Klein Soenda Eilanden, Plawangan, Segara anak, 16 Jun 1936, *de Voogd 2654* (GH, NY).

Solanum americanum

AMERICAN SAMOA. **Ofu-Olosega**: Feaiia Point, 25 May 1925, *Christerpherson* & *Garber 976* (K). **Tutuila**: Faga'itua Cove, 11 Apr 1992, *Whistler 8436* (K).

ANGOLA. **Luanda**: Loanda, 1903, *Gossweiler 464* (BM, K, P).

AUSTRALIA. 12 m N of Busselton, 6 Sep 1965, *Beauglehole 12438* (AD); Ashfield in garden, Jan 1908, *Cheel s.n.* (NSW); Ball Bay, on beach below Reserve, 27 Dec 1967, *Hoogland 11308* (CANB); Dawson, 17 Jul 1847, *Leichhardt s.n.* (NSW); Ugar (Stephen's) Island, Torres Strait, 6 Mar 2009, *McKenna 468* (CANB); Sin. loc., *Rowan s.n.* (MEL); Victoria River, *Without Collector s.n.* (MEL); Sin. loc., *Woolls s.n.* (MEL). **Christmas Island**: Grants Well, Christmas Island, 6 Aug 1986,

Shivas 811 (PERTH); Track ML 116 to Field 23A, Christmas Island, Indian Ocean, 4 Sep 1996, *Swarbrick 13131* (PERTH). **New South Wales**: Saltwater Creek camping ground, S of Eden, 17 Sep 1984, *Albrecht 738* (AD, MEL); Wollongong, Bulli, coast, S of Sydney, 14 Sep 1981, *Alcock 8741* (AD, NSW); *Atkinson 35* (MEL); Barrenjoey, 19 Jun 1975, *Barry 9* (AD, K, NSW); Moree Plains, Apex Park, on bank of Macintyre River, Goondiwindi, 12 Dec 2001, *Batianoff & Halford 2112477* (NSW); Willoughby, Balls Head, 28 Apr 1928, *Black s.n.* (MEL); Hornsby, Cowan Creek, Cowan, 10 Mar 1918, *Blakely s.n.* (NSW); Dungog, Allyn River, Aug 1906, *Boorman s.n.* (NSW); Gosford, Wisemans Ferry, 55 km NNW of Sydney, 19 Sep 1971, *Briggs s.n.* (NSW); Sin. loc, 1799, *Caley s.n.* (BM); Reclaimed ground, Governor's Domain, Jul 1902, *Camfield s.n.* (NSW); Shoalhaven, Kioloa, Edith & Joy London Foundation of A.N.U., 4 Apr 1977, *Canning 4232* (CANB); Shoalhaven, Kioloa, track to beach near Edith and Joy London Foundation of A.N.U., roadside of foredune, 20 Oct 1977, *Canning 4284* (CANB); Shoalhaven, Yadboro State Forest, c. 21 km along Western Distributor Forest Road from S end at Bimberamala River crossing, 23 Aug 1983, *Canning 5736* (CANB); Hawkesbury, Farm of the Hawkesbury Agricultural College, Richmond, 5 Dec 1921, *Carne s.n.* (NSW); Hunters Hill, 5 Aug 1912, *Carne s.n.* (NSW); Hornsby, Beecroft, 3 May 1914, *Carne & Hudson 53* (NSW); Lismore, Tregeagle, 19 Mar 1946, *Chadwick s.n.* (NSW); Canada Bay, Ashfield, Nov 1932, *Cheel s.n.* (NSW); The Hills Shire, Parramatta, 10 Sep 1916, *Cheel s.n.* (NSW); Gosford, Woy Woy, Oct 1916, *Cheel s.n.* (NSW); Wingecarribee, Hill Top, Jan 1915, *Cheel s.n.* (NSW); Canada Bay, Ashfield, Dec 1916, *Cheel s.n.* (NSW); Sutherland Shire, Cronulla, May 1918, *Cheel s.n.* (NSW); The Hills Shire, Castle Hill, Mar 1918, *Cheel s.n.* (NSW); Wollongong, Woonoona near Bulli, 16 Feb 1918, *Cheel s.n.* (NSW); Canada Bay, Ashfield, 10 Sep 1916, *Cheel s.n.* (NSW); Randwick, Kensington, Apr 1917, *Cheel s.n.* (NSW); Wollongong, Gooseberry Island, 6 Apr 1912, *Cheel s.n.* (NSW); Sutherland Shire, Miranda via Sutherland, Nov 1917, *Cheel s.n.* (NSW); Richmond Valley, Broadwater, Sep 1916, *Cheel s.n.* (NSW); North Sydney, Neutral Bay, Mar 1916, *Cleland s.n.* (NSW); Hornsby, Hawk[esbury] R[iver] Railway St[atio]n, Mar 1916, *Cleland 28* (AD); Kogarah, The Lake, Scarborough Park, c. 7 mls SSW of Sydney, 14 Jan 1965, *Constable 5633* (AD, NSW); Manning River, 22.5 km (14 miles) by road NNW of Gloucester on Walcha Road, 1 Jun 1975, *Coveny et al. 6398* (K); Gloucester, Manning River, 22.5 km (14 miles) by road NNW of Gloucester on Walcha Road, 1 Jun 1975, *Coveny & Phillips 6398* (AD, NSW); Randwick, La Perouse, 3 Jun 1975, *Coveny 6424* (NSW); Cockle Creek, N of Booragul, 10 Nov 1975, *Coveny & Powell 6486* (K); Cockle Creek, N of Booragul, 10 Jun 1975, *Coveny & Powell 6486* (AD, NSW); Cockle Creek, N of Booragul, 10 Jun 1975, *Coveny 6487* (NSW); Singleton, Bulga Service Station on the Windsor - Singleton Road, 11 Jun 1975, *Coveny 6536* (NSW); Singleton, Bulga Service Station on the Windsor - Singleton Road, 11 Jun 1975, *Coveny & Powell 6537* (AD); Wollondilly, Rotherwood Road off Mount Hercules Road, Razorback Range, 10 km SSW of Camden, 17 Mar 1976, *Coveny 7465* (NSW); Moree Plains, Waa Gorge, 68 km NE of NArrabri by road, 23 Nov 1976, *Coveny 9020* (NSW); Ryde, Brush Farm Park, Eastwood, 24 Jan 1978, *Coveny 9998* (AD, NSW); Tollgate Island (inner), Batemans Bay, 20 Mar 1976, *Crisp 1984* (CANB, NSW); Eurobodalla, Mystery Bay, 9 km S of Narooma, 28 Aug 1976, *Crisp 2141* (AD, CANB); The Clear Place, 20 Oct 1978, *Crisp 4470* (CANB); Ballina, Wollongbar, 1 Aug 1952, *Crofts 7* (NSW); Willoughby, North Sydney district, Jun 1904, *Dixon s.n.* (NSW); Ryde, Eastwood to Denistone, 6 Nov 1952, *Evans s.n.* (AD); Eastwood to Denistone, 6 Nov 1952, *Evans s.n.* (NSW); Shellharbour, Macquarie Pass, between Robertson and Albion Park, 24 Aug 1979, *Fallding & Benson s.n.* (NSW); Goulburn Mulwaree, Bank of the Shoalhaven River, Bungonia, 28 Jan 1961, *Filson 3743* (MEL); Randwick, Maroubra, Nov 1954, *Ford s.n.* (NSW); Ballina, Fishery Creek, Barlows Road Industrial Area, 19 Apr 2003, *Forster 29317* (MEL, NSW); Big Gibber ridge, 11 km ENE of Bombah Point, 23 Apr 1982, *Fox 8204069* (NSW); Lord Howe Island, in the W Base of Mt Gower, 2 Sep 1969, *Game 69/306* (K); Wollongong, West Dapto, Reed Park area, 9 Oct 1990, *Gay & Ooi s.n.* (WOLL); Clarence Valley, Grafton district, 20 Jul 1939, *Glenfield Vet Research Station s.n.* (NSW); Port Stephens, Ash Island, Newcastle, Jun 1971, *Gosper 12* (NSW); Lord Howe Island, SE lower slope of Malabar, 11 Nov 1963, *Green 1571* (K); Nepean River, on eastern bank c. 5 km SW of Penrith, 21 Jan 1979, *Haegi 1607* (K); Penrith, Nepean River, on E bank. c. 5 km SW of Penrith, 21 Jan 1979, *Haegi 1607* (AD, NSW); Bega Valley, Merimbula Creek crossing on Tathra road, NE outskirts of Merimbula, 9 Feb 1979, *Haegi 1715* (NSW); Bega Valley, c. 15 km N of Merimbula on Tathra road, near turn off to Bondi Lake, 9 Feb 1979, *Haegi 1725* (AD, NSW); Bega Valley, Brockelos Creek crossing on Bega to Bermagui road, c.

15 km S of Bermagui, 9 Feb 1979, *Haegi 1737* (AD, NSW); Eurobodalla, Stony Creek crossing on Princes Highway c. 4.5 km S of Bodalla, 9 Feb 1979, *Haegi 1744* (AD, NSW); Ballina, Wollongbar, Dec 1916, *Haywood s.n.* (NSW); Kempsey, Yard enclosure. Smoky Cape Lighthouse, ENE of Kempsey, 23 Jan 1973, *Henderson 1405* (AD, NSW); Norah Head 70 km NE of Sydney, 13 Jan 1975, *Henderson 2278* (NSW); Hornsby, Epping, 6 Apr 1921, *Henry s.n.* (NSW); Montagu Island, 25 Mar 1988, *Heyligers 88050* (CANB); Montagu Island, 31 Mar 1989, *Heyligers 89016* (CANB); Montagu Island, 29 Mar 1992, *Heyligers 92010* (CANB, NSW); Montagu Island, 29 Mar 1994, *Heyligers 94009* (CANB); Bega Valley, Tathra, 20 Mar 1995, *Heyligers 95003* (CANB); Bega Valley, Tathra, 20 Mar 1995, *Heyligers 95005* (CANB, PERTH); Montagu Island, 28 Mar 1999, *Heyligers 99003* (CANB, NSW); Montagu Island; in the shearwater study area of the North Island, 28 Mar 1999, *Heyligers 99005* (CANB, NSW); Montagu Island, 28 Mar 1999, *Heyligers 99006* (CANB); Near Lovers Bay, 31 Oct 1963, *Hoogland 8720* (CANB, NSW); Sydney, *Hooker s.n.* (K); Hay, Murrumbidgil Creek, 10 mls N of Booligal on Ivanhoe Road, 15 Oct 1972, *Jacobs 420* (NSW); Sydney, Centennial Park, Sydney, 30 Aug 1974, *Jacobs 767* (NSW); Currarong Beach, 29 Sep 1981, *James 29* (NSW); Wollongong, Bulli, 1875, *Johnson s.n.* (MEL); Wollondilly, Broughton Pass, lower Cataract River, 9 May 1951, *Johnson s.n.* (NSW); Hornsby, Cheltenham, 14 Feb 1954, *Johnson s.n.* (NSW); The Hills Shire, New Line Road, Pye's Creek, West Pennant Hills, 23 May 1945, *Johnson 78* (NSW); Lord Howe Island, NW side of Transit Hill, Lowrd Howe Island, 11 Sep 1970, *Johnson & Rodd 1272* (K); NW side of Transit Hill, 11 Sep 1970, *Johnson 1272* (NSW); c. 670 m along border track from where it joins the Mount Lindesay road, c. 150 m east of border gate, North Coast, 14 Dec 2007, *Johnstone & Orme 2311* (CANB, K, NSW); Richmond Valley, 12 km ENE of Woodburn, Bora Ridge, 2-3 km down Benauds Road, 22 Apr 2009, *Jungmann 22* (NE); Wingecarribee, Wingecarribee Swamp, southwestern arm, 4 Apr 2001, *Kodela & Sainty 577* (NSW); Clear Place, 19 Oct 2000, *Le Cussan 939* (NSW); Mulsons Ferry. [?Milson's Point, Sydney], 6 Aug 1842, *Leichhardt s.n.* (NSW); 9 km east of Nelson Bay, Port Stephens, 13 Oct 2002, *Leishman 115* (NSW); Muswellbrook, 11.5km W of Jerrys Plains, 15 Sep 1990, *Lepschi 381* (CANB); Mid-Western Regional, 6.2km NNE of Bulga, 15 Sep 1990, *Lepschi 391* (CANB); Shoalhaven, 2 km N of Huskisson - Tomerong road junction on Jervis Bay - Nowra rd, 8 Dec 1992, *Lepschi & Craven 903* (AD, CANB); Shoalhaven, 2 km N of Huskisson - Tomerong rd junction on Jervis Bay - Nowra road, 8 Dec 1992, *Lepschi & Craven 904* (AD, CANB); Walgett, 0.5 km W of Pilliga on road to Come-By-Chance, 12 Dec 1998, *Lepschi 4113* (CANB); Port Stephens, Nelson Bay, 10 Nov 1965, *Lithgow 246* (NSW); Sutherland Shire, Cronulla, Sep 1952, *Loaney s.n.* (NSW); Pittwater, Barrenjoey headland, SE face, 4 Apr 1986, *Makinson 209* (NSW); Jervis Bay area, c. 8 km (direct) S of Vincentia, Australian National Botanic Gardens Annex, western side of Lake Mackenzie, on track, 28 Aug 1991, *Makinson 842* (CANB); Wollongong, Mount Kiera, Wollongong. In vicinity of scout's camp, 29 Nov 1969, *Martensz 188* (CANB); Shellharbour, 25 Nov 1949, *McBarron 4253* (NSW); Lord Howe Island, Nov 1936, *McCornish 46* (K); Auburn, Flemington Saleyards, Sydney, 23 Apr 1969, *Michael s.n.* (CANB); Eurobodalla, Malua Bay south coast, 16 May 1976, *Mills s.n.* (WOLL); Inverell, Lisgar, 24km S of Yetman on road to Warialda. (1km E of house), 4 Oct 1990, *Moore 9158* (CANB); Kyogle, Wiangarie State Forest N of Kyogle, NE, 6 Mar 1974, *Moriarty 1513* (CANB); Byron, Byron Bay, Belongil Beach, on edge of track behind sand dunes in front of 'The Wreck', 13 May 2010, *Moulton 7* (NE); Clarence Valley, Clarence River, 1894, *Murray s.n.* (MEL); Clarence Valley, Grafton, 5 May 1953, *O'Grady s.n.* (NSW); Nambucca, Missabotti Creek, 8 km NW of Bowraville along road to Bellingen, 21 May 2005, *O'Keefe 37* (NE); Shoalhaven, Durras Lake, 10 km N of Batemans Bay Site 1, 22 May 1983, *van Oosterzee 15* (CANB); Singleton, Hunter Valley, Darrowby, c. 3.5 km W of Broke on the road to Milbrodale, 6 Aug 1998, *Palmer D-7* (CANB); Shoalhaven, Sussex Inlet, Suncrest Avenue, 1 Sep 2007, *Patrick 5063* (CANB); Bega Valley, Bermagui South, Dickinson Caravan Park, 30 Oct 1979, *Pearce 156* (AD, NSW); Penrith, Orchard Hills Defence Area - site 10, 26 Aug 2002, *Pellow & Clarke s.n.* (WOLL); Tenterfield, Glengray [Glengarry?], Leeville, Casino, Jul 1923, *Perry 2* (NSW); Greater Taree, Red Head, 7 Mar 1961, *Phillips s.n.* (CANB); Shellharbour, Minnamurra, 3 May 1961, *Phillips 933* (CANB); Summit ridge of Mount Lidgbird, 17 May 1971, *Pickard 1470* (NSW); N end Little Slope, 9 Dec 1975, *Pickard 2749* (NSW); Potato Hills, S end of Little Slope, 10 Dec 1975, *Pickard 2763* (NSW); Rocky Run Valley, 13 Dec 1975, *Pickard 2832* (NSW); Tenth of June, 14 Dec 1975, *Pickard 2896* (NSW); Tenth of June, 14 Dec 1975, *Pickard 2904* (NSW); Mutton Bird Island, 15 Dec 1975, *Pickard 2930* (NSW); Central Darling, Marlow Gypsum Mine 22 km N of Conoble

Railway Station, 23 Oct 1976, *Pickard 3002* (NSW); St South Pebbles, 27 Feb 1973, *Pulley 1234* (CANB); Camden, Summit of Mt Annan, Campbelltown, 27 May 1987, *Quirico & Richards 7* (NSW); Ballina, 16 m [25.5 km] from Lismore toward Ballina, 17 Jul 1966, *Richards 149* (CANB); Hornsby, McKell Park, Brooklyn, 11 Oct 1968, *Rodd & Tindale s.n.* (NSW); Wollongong, Hillview, 3 mls SW of Liverpool, 11 Dec 1968, *Rodd s.n.* (NSW); Great Lakes, Seal Rocks, 5 Aug 1968, *Rodd 688* (NSW); Great Lakes, Seal Rocks, 5 Aug 1968, *Rodd 690* (AD, NSW); Great Lakes, Seal Rocks, 5 Aug 1968, *Rodd 691* (AD, K, NSW); Lord Howe Island, valley between North Beach and New Gulch, 23 Mar 1971, *Rodd 1751* (K); Valley between North Beach and New Gulch, 23 Mar 1971, *Rodd 1751* (NSW); Nielsen Park, Port Jackson, 25 Apr 1933, *Rodway s.n.* (NSW); Wollongong, Austinmer, 16 Jul 1933, *Rodway s.n.* (NSW); Shoalhaven, Huskisson, Jervis Bay, 16 May 1934, *Rodway s.n.* (NSW); Montaque Island, 125 mls S of Norova, 4 Apr 1932, *Rodway 735* (K); on side of road over Cambewarta range between Norova and Kanyam Valley, 6 Jul 1933, *Rodway 1092* (K); Austinmer, 22 Apr 1934, *Rodway 1334* (K); Bowen Island, Jervis Bay, 3 Jun 1934, *Rodway 1377* (K); South Crookhaven Head, 1 Jan 1941, *Rodway 4766* (NSW); Brush Island, 16 Aug 1936, *Rodway 6483* (NSW); Shoalhaven, Cambewarra Range, near Bellawangra, 29 Oct 1939, *Rodway 6486* (NSW); Bowen Island, Jervis Bay, 3 Jun 1934, *Rodway 6488* (NSW); Brush Island, 6 Sep 1936, *Rodway 6489* (NSW); Montague Island, 4 Apr 1932, *Rodway 6490* (NSW); Shoalhaven, Tabourie Island, 1 Jan 1940, *Rodway 11333* (NSW); Great Lakes, SW shore of Wallis Lake, SW of Forster, 7 Jan 1967, *Salasoo 3333* (NSW); Marrickville, Beside Manning House, Sydney University, 18 Mar 1972, *Solling 117* (NSW); Hornsby, Pennant Hills, Jan 1919, *Steel s.n.* (NSW); Port Macquarie-Hastings, Gannons Creek, 11 km SW of Wauchope, 28 Aug 1978, *Streimann 8078* (CANB); Rockdale, Kogarah, 3 Apr 1968, *Symon s.n.* (NSW); Wyong, 3 Apr 1968, *Symon s.n.* (NSW); Rockdale, Kogarah, Jan 1966, *Symon s.n.* (NSW); Rockdale, Kogarah, 2 Apr 1968, *Symon SS-143* (NSW); Bellingen, Dorrigo Plateau, Moses Rock road Dorrigo State Forest, 14 Jan 1979, *Symon 11521* (AD); Mylestom, 15 Jan 1979, *Symon 11525* (AD, NSW); Jervis Bay National Park, Bowen Island near houses, 17 Sep 1996, *Taws 592* (CANB, NSW); Cowra, 7 Apr 1976, *Toth 9* (NSW); Eurobodalla, 14 km N of Yowrie P.O. on N side of Tuross river, 15 km NW of Cobargo, 19 Feb 1978, *Tyrrel 24* (CANB); Wyong, 19 May 1966, *Whitehead s.n.* (AD); Gosford, Bouddi Range, 27 Dec 1965, *Whitehead 7* (AD); Bega Valley, Wolumba road 2.5 mls SE of Candelo, 7 Nov 1973, *Willis s.n.* (AD); Eurobodalla, Ohlson's Creek near Narooma, 23 May 1964, *Willis s.n.* (MEL); Lord Howe Island, Mount Gower ridge, 31 Aug 1981, *Willis s.n.* (MEL); Bega Valley, Wolumla road c. 2.5 mls SE of Candelo, 7 Nov 1973, *Willis s.n.* (MEL); Cape Banks, La Perouse, 28 Feb 1975, *Wilson 1254* (NSW); Lismore, SW outskirts of Lismore on Casino rd, 12 May 1981, *Wilson 3842* (NSW); Coffs Harbour, Ocean View Beach, Arrawarra, Mar 1993, *Wissmann s.n.* (NE); Ocean View Beach, 10 Jan 2000, *Wissmann s.n.* (NE); Kyogle, Moore Park Nature Reserve, NW of Grevillia, upper Richmond River, 17 Aug 1990, *Without Collector s.n.* (NE); Lismore, Lismore distr., Sep 1949, *Without Collector s.n.* (NSW); Coogee, May 1885, *Without Collector s.n.* (AK); Forbes, Back Yamma, Apr 1963, *Without Collector 170* (AD); Budgewoi, 5 Jan 1964, *Without Collector 1229* (AD); Byron, Byron Bay, Jun 1965, *Yabsley s.n.* (NSW). **Norfolk Island**: Commune de Coconi, a la sortie de ville en direction de Dembéni, 26 Nov 2012, *Delnatte & Gallay 2930* (BM); Steels Point, 31 Oct 1978, *Telford 7215* (CANB); Collins Head Road, Norfolk Island. Pineapple plantation owned by Terry Jope, 14 Oct 1999, *Waterhouse 5519* (AD, CANB). **Northern Territory**: Roper Gulf, Katherine River, W of Katherine, Stuart H[i]ghw[a]y, 8 Aug 1978, *Beauglehole & Errey 58411* (AD, DNA); East Arnhem, Gove, 5 Feb 1997, *Booth 2100* (DNA); Gove Golf Club, 6 Feb 1997, *Booth 2162* (DNA); Nhulunbuy, 9 Oct 1993, *Cowie 4476* (CANB, DNA); Tiwi Islands, Melville Isle; SE.Coast, 6 Apr 1992, *Fensham 1290* (DNA); Adelaide River, Northern Territory, 2 Jan 1973, *Henderson 1361* (CANB, K); Coomalie, Adelaide River farms, 28 Sep 1972, *Holmes s.n.* (AD); Coomalie, Jettner's Farm, Adelaide River, 22 Nov 1972, *Holmes s.n.* (AD); Coomalie, adelaide river farms, 28 Sep 1972, *Holmes s.n.* (DNA); Coomalie, Jettners Farm, 22 Nov 1972, *Holmes s.n.* (DNA); Sin. loc., 23 Jan 2003, *Mitchell & Cameron 8071* (AD); West Arnhem, Kakadu National Park, Gungarre Walk at South Alligator, 2 Oct 2007, *Purdie 6559* (CANB, DNA); Alligator Head, Marrakai Station, 2 Aug 1983, *Rankin 2749* (AD); Barkly, Lake Nash Stn, SE end of Waterhole behind Hmstd, 4 Aug 2001, *Risler 970* (DNA); Woolner Station, source of Whitestone Creek, 2 Jun 1988, *Russell-Smith 5421* (DNA); Nhulunbuy, Gyngaru walk, 18 Sep 1998, *Smith 4382* (DNA); Coomalie, Adelaide River Farms, 1 Feb 1973, *Symon s.n.* (AD); Victoria-Daly, Palumpa (Nganmarriyanga) Tip, ca 50 km E of

Port Keats, 6 Jun 2012, *Westaway 4018* (CANB, DNA). **Queensland**: Brisbane, Wilsdon, N suburb of Brisbane, 5 Nov 1973, *Alcock 4454* (AD); Goondiwindi, Yelarbon, roadside, 60 km E of Goondiwindie, 15 Jul 1981, *Alcock 8588* (AD); Bundaberg, Gin Gin, on Bruce Highway, 30 Jul 1981, *Alcock 8594* (AD); Atherton Tablelands E of Cairns, Tinaroo Reserve, 18 Aug 1981, *Alcock 8665* (AD); Brookvale Park, 8km N of Oakley, 30 Jun 1993, *Alcock 11266* (AD, MO); Toowoomba, Brookvale Park, some 8 km N of Oakley, 30 Jun 1993, *Alcock 11267* (AD); Toowoomba, Creek bank some 20 km SW of Millerran, 30 Jun 1993, *Alcock 11273* (AD, CANB); Lockyer Valley, S.A.C. Lawes, 7 Sep 1937, *Allen s.n.* (CANB); Charters Towers, Charters Towers, Jun 1892, *Bailey s.n.* (NSW); Goondiwindi, Toowoomba area, 30 Dec 1988, *Bates 16832* (AD); McEwens Beach, Mackay, 15 Jul 1992, *Batianoff & Dillewaard 92-0727* (AD); Wilson Island, 12 km N of Heron Island, 19 Oct 1998, *Batianoff & Hacker 98-1061* (AD); 27 May 2000, *Batianoff 205108* (NSW); Southern Downs, Ranges station, Sundown National Park, 47 km SW Stanthorpe, 11 Apr 2001, *Batianoff & Collyer 210495* (DNA, NSW); Southern Downs, Roadside, 17 km SW of Leyburn, 33 km SE of Millmerran, 26 Apr 2001, *Batianoff 210654* (CANB); Southern Downs, Roadside quarry, 20 km SE of Warwick, 9 Apr 2001, *Batianoff 210686* (CANB, NSW); Spinnaker Park, Gladstone, Gladstone Shire, 11 Oct 2004, *Batianoff 410011* (MEL); Tablelands, Tully Gorge National Park, 9 Feb 2003, *Batianoff 630272* (DNA); Goondiwindi, Goondiwindi, 5 Oct 2000, *Batianoff & Appelman 2010485* (NSW); Moreton, Indooroopilly, Brisbane, Jun 1968, *Baxter s.n.* (K); Central Highlands, Rolleston-Injune road, 9 km N of Carnarvon turnoff, 4 Sep 2003, *Bean 20752* (CANB); Green Hills WNW of Forsayth, banks of Gilbert River, 14 Jul 1954, *Blake 19542* (AD); Southern Downs, Mt Roberts, 26 May 1951, *Blake 21507* (AD); Rockhampton, Neerkool Creek, *Bowman 104* (MEL); Quilpie, 10.9 km from Adavale toward Blackall at tank, 25 Oct 1983, *Canning 6209* (CANB); Cook, Honey Dam, 2.2km South West of the Peninsula Development Road on Lakeland Downs Station, 4 Sep 1984, *Clarkson 5498* (AD, K); Cook District, Heathlands Ranger Base, 7 Mar 1992, *Clarkson 9357* (AD, K); Pinkenba, 1 Sep 1918, *Cleland s.n.* (AD); Gympie, Creek at Glastonbury, 17 May 1973, *Conn s.n.* (MEL); Cook, Mount Glennie, Macpherson Ranges, 8 Jan 1953, *Constable s.n.* (NSW); Mackay, on Eungella Range Rd, 75 km W of Mackay, 2 May 2004, *Cumming 22821* (CNS); Cook, Mareeba, Oct 1967, *Cunningham s.n.* (K); Rockhampton, Rockhampton, Jul 1865, *Dietrich 1523* (MEL); Brisbane, c. 70 km W of Bollon, 5 Nov 1980, *Dillewaard & Olsen 210* (AD); Noosa Bay, 1872, *Eaves s.n.* (MEL); Noosa Bay, *Eaves s.n.* (MEL); Boatman Stn, overflow from No.8 Bore, 9 Aug 1946, *Everist 2642* (AD); Belmont, near Brisbane, 11 Sep 1956, *Everist 5606* (CANB, K); Moreton, Stradbroke Island, Point Lookout, 12 Sep 1971, *Everist & Simon 10054* (K); Cassowary Coast, Innisfail, 20 Aug 1945, *Flynn s.n.* (NSW); Cairns, Wooroonooran National Park, Mt Bellenden Ker Summit area, 5 Dec 2000, *Forster et al. 26512* (AD); Rosedale-Bundaberg rd at Rosedale, 4 Apr 1975, *Halliday 354* (AK, HO); South Burnett, Blackbutt, 8 Aug 1967, *Henderson 281* (AD, NSW); Benarkin State Forest, Blackbutt, 8 Aug 1967, *Henderson 288* (AD); Moreton District, Salisbury, Brisbane, 6 Aug 1967, *Henderson 298* (AD, K); Moreton District, Salisbury, Brisbane, 6 Aug 1967, *Henderson 299* (AD, K); Moreton Bay, Toowong, Brisbane, 15 Aug 1969, *Henderson 523* (AD); Moreton District, Indooroopilly, Brisbane, May 1970, *Henderson 544* (AD, BM, CANB, K, MEL, NSW); Atherton, 19 Mar 1971, *Henderson 563* (K, NSW); Cook District, Atherton, 19 Mar 1971, *Henderson 564* (K); Moreton District, Levers Plateau, on Qld/NSW border ca 90 km SSW of Brisbane, 6 Apr 1972, *Henderson 1303* (AD, K, NSW); Southern Downs, The Heads, 40 km SSW of Boonah on road to Killarney, 29 Aug 1972, *Henderson & Sharpe 1343* (AD); Moreton Distr., Indooroopilly, Brisbane, near Plant Pathology Branch building, 19 Dec 1972, *Henderson 1358* (CANB, K); Darling Downs Distr., Cunningham's Gap, ca. 45 km NE of Warwick, 17 Mar 1973, *Henderson 1414* (K, NSW); Cook, Ca. 3.5 km S of Mareeba, 2 Apr 1973, *Henderson 1572* (K); South Burnett, Benarkin State Forest, Blackbutt Range, just E of Benarkin township, 3 Apr 1975, *Henderson 2284* (AD); Brisbane, Indooroopilly, 18 Nov 1977, *Henderson 2607* (CANB); Isaac, nr the homestead on Moonoomoo Station, 14 Oct 1983, *Henderson et al. 2791* (AD); Tamborine Mtn., c. 45 mls S of Brisbane, 5 May 1930, *Hubbard 2508* (K); Tarragindi, Brisbane, 15 Aug 1969, *Ising s.n.* (AD); Suburban Mackay, 6 Aug 1968, *Jackes s.n.* (AD); Mount Roberts, Macpherson Ranges, 19 May 1951, *Johnson 49* (NSW); Brisbane, Brisbane, Oct 1956, *Jones 238 (h)* (CANB); Brisbane, University grounds Brisbane, 27 Nov 1966, *Jones 3317* (CANB); Cairns, Bellenden Ker Range, 1881, *Karsten. ? s.n.* (MEL); Western Downs, Jimba, 30 Sep 1844, *Leichhardt s.n.* (NSW); Barcaldine, Mooney Creek, 52 km N of Blackall on road to Barcaldine, 15 Oct 1993, *Lepschi & Slee 1153* (AD, CANB); Masthead Island, Sep 1912,

Longman s.n. (K); Leichhardt, 70 km by road N of Dingo on Dingo-Mt. Flora Road, 7 Jul 1972, *McDonald 00548* (K); Tablelands, S of Longlands Gap, Herberton Range, 3 Aug 2003, *McDonald 1528* (DNA); Torres, Horn Island, Wasaga, house on Jena St, 15 Aug 2008, *McKenna 217* (CANB); Gold Coast, Pimpama, 9 mls SE of Beenleigh, 18 Jun 1968, *Middleton s.n.* (NSW); Mackay, Cattle Creek at crossing on Mackay - Eungella rd, ca 20 km from Eungella, 8 Aug 1994, *Moore 9391* (CANB); Mackay, Cattle Creek at crossing of Mackay-Eungella Rd., ca 20 km from Eungella, 17 Sep 1996, *Moore 9450* (CANB); Brisbane, Graceville, Brisbane, 21 Jun 1974, *Moriarty 1538* (CANB); Cook, Crest of Altanmoui Range, adjacent to grassy plateau, Cape Melville National Park (Altanmoui Range section), 24 May 1993, *Neldner 3987* (AD); Rockhampton, *O'Shanesy s.n.* (MEL); Gold Coast, Southport, Paradise Point, 14 Sep 1968, *Pedley 2774* (AD); Toowoomba, About 40 km ENE of Inglewood nr Gore, 10 Jan 1984, *Pedley 5024* (AD); Toowoomba, Near Gore c. 40 km ENE of Inglewood, 10 Jan 1984, *Pedley 5024* (NSW); Brisbane, Indooroopilly, 31 Oct 1999, *Pedley 5816* (AD); Brisbane, Indooroopilly, 31 Oct 1999, *Pedley 5816* (CANB); Brisbane, Indooroopilly, 31 Oct 1999, *Pedley 5816* (DNA); Brisbane, Indooroopilly, 31 Oct 1999, *Pedley 5816* (MEL); Brisbane, Indooroopilly, 31 Oct 1999, *Pedley 5816* (NSW); Cook, Endeavour River, *Persieh s.n.* (MEL); Moreton Bay, Mt. Glorious, South fall of Joyner's Ridge, 10 Aug 1963, *Phillips 88* (CANB); Moreton Bay, Mt Glorious, 28 May 1961, *Phillips 1047* (CANB); Moreton Bay, Mt Glorious, 28 May 1961, *Phillips 1064* (CANB); Charters Towers, *Plant s.n.* (NSW); Cairns, Summit area of Bellenden Ker, S of Cairns, 16 Sep 1977, *Powell et al. 804* (AD, NSW); 10 m N of Chinchilla, 2 May 1966, *Redgen 48* (AD); Fairfax Island, Nov 1927, *Rodway 6496* (NSW); Heron Island, Capricorn Group, 20 Dec 1960, *Slater 45* (CANB); Kuranda Railway Station, Aug 1974, *Swan 128* (AD); Salisbury, Brisbane, 3 Apr 1968, *Symon 268* (AD); Just N of Laura, turnoff from Cooktown road, 20 May 1967, *Symon 4779* (AD, CANB, K, L); Ravenshoe caravan park, 25 May 1967, *Symon 4879* (AD, CANB, K); Nr Millstream Falls just W of Ravenshoe, Atherton Tableland, Q, 26 May 1967, *Symon 4880* (AD, CANB, K); Moreton Bay, Forest access track, Maiala Reserve (near Mt Glorious), 28 Jun 1989, *Symon 14882* (AD); Tablelands, Ravenshoe, 8 Apr 1937, *Thurston 3195* (AD); Johannsons Caves - within a mile of Mt Etna nr The Caves N of Rockhampton, 11 Oct 1968, *Tracey s.n.* (AD); Mt Bellenden Ker - peak, 5 Jan 1978, *Valentine s.n.* (AD); Tablelands, Mt Molloy Campground, 30 Aug 1999, *Waterhouse 5401* (AD); Tablelands, Mt Molloy Campground, 30 Aug 1999, *Waterhouse 5401* (CANB); Cook, Coen Township, 8 Sep 1999, *Waterhouse 5466* (CANB); Hope Vale, Hopevale Township, 6 Jun 2000, *Waterhouse 5956* (CANB); Gladstone, Heron Island, 9 Nov 1985, *White s.n.* (NE); Heron Island, 9 Nov 1985, *White s.n.* (NSW); Botanic Gardens, Brisbane, 5 Nov 1907, *White s.n.* (NSW); Brisbane, Brisbane River, Mar 1915, *White s.n.* (NSW); Islands of Bunker & Capricorn Groups, *Without Collector s.n.* (AD); Rockhampton, *Without Collector s.n.* (MEL); Rockhampton, *Without Collector s.n.* (MEL). **South Australia**: Creekline-Paralana Springs, Northern Flinders, 24 Aug 1968, *Symon 6090* (AD, CANB, K). **Tasmania**: Clarence Point, West Tamar, 28 Sep 1993, *Buchanan 13453* (HO); Bass Strait, Kent Group, Erith Island, 1969, *Murray-Smith s.n.* (MEL). **Victoria**: Wellington, C. 18 km N of Maffra, Valencia Creek, 10 May 1979, *Aston 2013* (MEL); Latrobe, Rosedale Shire, 3 mls NW of Toongabbie, 13 mls NNE of Traralgon, 12 Feb 1972, *Beauglehole 4332* (MEL); Ararat, Ararat, McDonald Park, 15 Nov 1966, *Beauglehole 21847* (AD, MEL); Yarra Ranges, Moori Yalock Picnic Ground area, c. 0.5 km NW of Yellingbo, 22 Mar 1976, *Beauglehole 50427* (MEL); Wellington, The Lakes National Park. Near Lake Reeve Lookout, 19 Dec 1978, *Beauglehole 62800* (MEL); Surf Coast, Angahook - Lorne State Park, 17 Mar 1982, *Beauglehole 70231* (MEL); Queenscliffe, Swan Island, 31 Jul 1982, *Beauglehole 70633* (MEL); Mornington Peninsula, Nepean State Park, 9 Nov 1982, *Beauglehole 71312* (MEL); French Island State Park, 14 Nov 1982, *Beauglehole 71472* (MEL); Cardinia, Parker Flora Reserve, 26 Nov 1982, *Beauglehole 71844* (MEL); Glenelg, Dartmoor Picnic area, Glenelg River, 1 Jan 1985, *Beauglehole 79123* (MEL); Wellington, Thomson River Natural Feature Zone. 6 km SE of Walhalla, 22 Apr 1985, *Beauglehole 79189* (MEL); Wellington, Thomson River Natural Feature Zone, 6 km SE of Walhalla, 22 Apr 1985, *Beauglehole 79190* (MEL); Wellington, Perry River bridge area, 18 km ESE of Stratford Post Office, 6 May 1985, *Beauglehole 79646* (MEL); Gannawarra, Murray River Reserve, 29 Dec 1985, *Beauglehole 83205* (MEL); East Gippsland, Wood Point on Snowy River, 21 Mar 1976, *Cameron 6123* (MEL); East Gippsland, W bank of Snowy River, Lochend, 10 Feb 1977, *Cameron 8123* (MEL); East Gippsland, Scorpion Forest Management Block, 10 Feb 1984, *Chesterfield 101* (MEL); East Gippsland, N of Mallacoota township, by dirt rd SW off intersection of Narbethong

Avenue and Lakeside Drive, 4 Apr 1999, *Clarke 2891* (AD, MEL); Baw Baw, Walhalla, 28 Apr 1991, *Connock 262* (MEL); Boroondara, Balwyn, 7 Glenluss St, Feb 1982, *Corrick 7567* (MEL); Wellington, Gippsland Lakes National Park, Romatah Island, 30 Dec 1985, *Crawford 418* (MEL); East Gippsland, W bank of Snowy River at Wood Point, 14 km NW of Orbost, 19 Jun 1982, *Forbes 937* (AD, MEL); Near Ararat, *Green s.n.* (MEL); Milgrove, along the rd to tip in grove in front of Pallotti College, 13 Jul 1990, *LeBreton s.n.* (CANB); Colac-Otway, Cape Otway, 14 May 1983, *LeBreton s.n.* (MEL); South Gippsland, Road from Sealer's Cove to Windy Saddle, 18 Apr 1991, *LeBreton s.n.* (MEL); Yarra Ranges, Milgrove, 13 Jul 1990, *LeBreton 237* (AD); Yarra Ranges, Melbourne, outer suburb of Montrose. Bungalook Creek, in Leversha Reserve, 13 Sep 1997, *Lorimer s.n.* (MEL); Baw Baw, Beynon's Creek flats at Morgan's Mill (W Tyers) 7 km (direct) NW of Erica, 20 Apr 1987, *Scarlett 87-38* (AD); Nillumbik, Eltham, Lenister Farm, Yarra River, 15 Jun 1994, *Thomas 661* (MEL); Yarra Ranges, Dandenong Ranges, Sherbrooke Forest, c. 1 km SSW of Kiosk at Grants Picnic Area, Kallista, 4 Feb 1989, *Walsh 2255* (MEL); Cardinia, Bunyip State Park, Labertouche North, 250 m due S of junction of Labertouche Nth Rd and Bunyip River Rd, 15 Jun 2010, *Walsh 7011* (MEL); East Gippsland, Brodribb River below Cabbage Tree Creek, 10 Mar 1965, *Webb s.n.* (CANB); South Gippsland, Tallwood on upper Bass River nr Nyora, 21 Jul 1990, *Willis s.n.* (CANB); Baw Baw, Banks of Latrobe River at Noojee, 10 Dec 1963, *Willis s.n.* (MEL); Yarra Ranges, Healesville, Sir Colin Mackenzie Fauna Park, 15 May 1979, *Willis s.n.* (MEL); Cape Conran, E of Marlo, 7 Feb 1972, *Willis s.n.* (MEL); East Gippsland, Mallacoota, above Devlin's Inlet, 12 Apr 1990, *Willis s.n.* (MEL); Pyrenees, Trawalla Forest, near 96-mile post on Highway 8, just E of Beaufort, 9 Nov 1974, *Willis s.n.* (MEL). **Western Australia**: Manjimup, Ritter Rd, 8.6 km S of Old Vasse Road junction, 20 Oct 1994, *Annels & Hearn 4680* (PERTH); Ravensthorpe, ca 50 m E of access track to Dunns Swamp near Hopetoun, 3 Apr 2007, *Bennett 1088* (PERTH); Melville, Blue Gum Lake, 25 May 1999, *Brown 158* (PERTH); Northampton, Grey's Gorge, c. 4 km SE of Kalbarri township, 25 Oct 1994, *Craven 9442* (CANB); Lake Mealup, 25 km W of Pinjarra, *Creed 280* (PERTH); Swan, Saunders St (W end), Aboriginal Community, Henley Brook, 10 Dec 1996, *Edgecombe 94* (PERTH); South Perth, Near Collier School, Como, 25 Sep 1969, *George s.n.* (PERTH); Cockburn, Bibra Lake, 23 Nov 1961, *George & Marchant 3172* (PERTH); Northampton, On the northern boundary of the reserve, 700 m W of NE corner, Chilimony Nature Reserve, c. 27 km NNW of Northampton, 30 Sep 1998, *Gibson 3953* (PERTH); Canning, opposite intersection of Fern Road and Fern Place, Wilson, 5 May 2004, *Hart 4013* (PERTH); South Perth, Canning foreshore, Waterford, 18 Jun 2000, *Hislop 2026* (PERTH); Serpentine-Jarrahdale, 9 km WNW of Serpentine, 13 Aug 1992, *Keighery & Gibson 754* (PERTH); Serpentine-Jarrahdale, Lowlands property (M103) 8 km WNW of Serpentine, 13 Aug 1992, *Keighery & Gibson 755* (PERTH); Wanneroo, Yonderup Lake, Yanchep, 50 km N Perth, 19 Apr 1988, *Keighery & Alford 1722* (PERTH); Foggerthorpe Cres, Maylands, suburb of Perth, 19 Jul 1982, *Keighery 5018* (CANB, K, PERTH); Albany, Misery Beach; Torndirrup, 20 km SSE Albany, 27 Oct 1986, *Keighery 8887* (PERTH); Albany, Misery Beach, Torndirrup, 18 km S of Albany, 4 Nov 1987, *Keighery 9645* (PERTH); Plantagenet, Porongurup National Park, 3.5 km along Angwin Peak Rd from Bolganup Road, 10 Dec 1988, *Keighery 11071* (PERTH); Northampton, Yerina Spring, Northampton to Kalbarri, 13 Jun 1997, *Keighery 15045* (PERTH); Busselton, Tuart Forest, Abba River, E of Busselton, 16 Dec 2001, *Keighery 16223* (PERTH); Wanneroo, Lake Nowergup Nature Reserve, E of Neerabup National Park, 1 Aug 2003, *Keighery 16460* (PERTH); Bayswater, NW bank of Swan River at Garratt Rd Bridge, Bayswater, Perth, 6 Mar 1995, *Lepschi & Lally 1752* (CANB, PERTH); Nr junction of Princess Royal Drive and York Road, Albany, 2 May 1996, *Lepschi & Lally 2580* (AD, CANB, PERTH); Irwin, 24km NW of Mingenew, on S side of road 3.5km E of intersection with Burma Road on Allanooka Springs Road, 30 Sep 2000, *Lyons & Lyons 4693* (PERTH); Albany, Mount Manypeak River, *Maxwell s.n.* (MEL); Albany, Bald Island, *Maxwell s.n.* (MEL); Esperance, S end of Mount Ragged of W side, 1 Jul 1976, *McGillivray 3627* (NSW); Perth, *McKaige s.n.* (PERTH); SW Australia, Jun 1861, *Mills s.n.* (MEL); Wyndham-East Kimberley, P. Lamereux small block, River Farm Road, Ord River Irrigation Area, c. 5 km N of Kununurra, 26 Jun 1998, *Mitchell 5513* (AD); Wyndham-East Kimberley, River Farm Road, Ord River Irrigation area, ca 5 km N of Kununurra, 26 Jun 1998, *Mitchell 5513* (PERTH); Broome, Kirby's Block, 12 Mile Area, 12.98 km from Broome P[ost] O[ffice], 30 Mar 2001, *Mitchell 6682* (AD, DNA, PERTH); Broome, Greys banana plantation, Skewthorpe area, about 20 km E of Broome, 11 Mar 2003, *Mitchell 7413* (AD); Broome, Greys banana plantation, Skewthorpe area,

about 20 km E of Broome, 11 Mar 2003, *Mitchell 7413* (PERTH); Harvey, Crampton Road Wetland, Lot 1093, Crampton Road, W of Harvey SWCC.WH. 03a Plot CRTN02, 4 Oct 2007, *Morley 680* (PERTH); Bayswater, Lower Swan River, 20 Mar 1909, *Morrison s.n.* (BM); Albany townsite, 9 Aug 1974, *Perry 255* (PERTH); Albany townsite, 9 Aug 1974, *Perry 255* (PERTH); South Perth, 16 Jun 1959, *Royce 5846* (PERTH); South Perth, 6 Jan 1968, *Royce 8408* (AD, PERTH); Wanneroo, Boomerang Gorge, Yanchep, 9 Aug 1971, *Smith s.n.* (PERTH); Stirling, side of walk trail nr carpark off Jon Sanders Drive on edge of Herdsman Lake, 16 Aug 2002, *Smith 182* (PERTH); Ravensthorpe, Around house, 2 km E of Ravensthorpe up road on left hand side, 22 Aug 1998, *Tink 266* (PERTH); Broome, Kirbys Block, 12 Mile Area, 12.98 km from Broome Post Office, 15 Sep 2011, *Westaway 3656* (CANB); Belmont, Belmont, c. 8 km E of Perth, 26 Aug 1967, *Wilson 6069* (AD); Belmont, Belmont, ca 8 km E of Perth, 26 Aug 1967, *Wilson 6249* (AD, PERTH).

BANGLADESH. Dhaka, 22 Mar 1868, *Clarke 6653* (CAL); **Chittagong**: Cox's Bazar region, Cox's Bazar area, 17 May 1999, *Huq 10557* (F, GH, MO, NY).

BHUTAN. Timpu, 11 Aug 1914, *Cooper 3355* (BM); Dung Dung, lower Mangde Chu valley, 25 May 1979, *Grierson* & *Long 1324* (K).

CAMBODIA. **Kampot**: Kampot, 9 Nov 1903, *Geoffray 180* (P); Kampot, Trach Kol, Peing-Pa, Khomch, Jul, *Hahn 1* (P); Kampot, Hotel Parc Boker, 27 Mar 2006, *Long et al. 124* (P).

CAMEROON. 5.5 mi S of Kribi, 30 Jan 1969, *Bos 3803* (K, MO, P).

CAPE VERDE. sin. loc, 1895, *Cardoso 134* (K); sin. loc, 1895, *Cardoso 194* (K). **Santiago**: sin. loc, Nov 1873, *Moseley s.n.* (K). **Santo Antão**: Jun 1841, *Vogel 8* (K).

CHINA. Fujian: Xiamen Island, Amoy Island, 27 Nov 1922, *Chung 782* (W); S. Fukien, 1923, *Chung 1729* (K); University of Amoy, Amoy Island, 10 Jul 1933, *Steward 3022* (A). **Guangdong**: Lin Dist[rict], 3 Oct 1918, *Canton Christian College 3383* (GH); Gan-chowen, *Cavalerie 7581* (E, K); Wutongshan, 26 Apr 2014, *Knapp 10690* (BM); Shenzhen, along the Greenway between Yantian and Dameisha, 15 Jun 2016, *Knapp IM-10787* (BM); Honan Island, 13 Oct 1916, *Levine 178* (A); Ying Tak Distr., 31 Mar 1929, *Tsiang Ying 1968* (E, F, W); vicinity of Yuen Vei City, 17 Jan 1928, *Wang 399* (A); Yeung Kong, rd outside American Presbyt. Hospital, 24 Jan 1935, *Whiting* & *Stewart 100* (K); 3 Apr 1923, *Without Collector 11048* (BM); nr city, Yun Fou Distr. [Yunfu], 17 Jan 1928, *Wuang 399* (E). **Guangxi**: Longan, 28 Feb 2009, *Monro* & *Wei Yi-Gang 6415* (BM). **Hainan**: Kowng tung Prov., Honam Island, 20 Jan 1917, *Canton Christian College 373* (MO); Chim Fung Ling nr Sam Mo Wat village (Kan-en Distr.), 11 Apr 1934, *Lau 3752* (GH); Nam Shan Leng, Yai-hsein Distr., 7 Apr 1935, *Lau 5963* (A); Bak Sa, 5 Mar 1936, *Lau 25582* (A); Mei Man and vicinity, Ching Mai Distr., 22 Oct 1932, *Lei 159* (K); Ka Tang Lung Ta'o, Lin Fa Shan, Taam Chau-Lam Ko Distr., 8 May 1928, *Tsang 231* (A, K); sin. loc, 5 Aug 1933, *Wang 33561* (K); Hainan Island, 12 Dec 1933, *Wang 35331* (MO, NY, P). **Hong Kong**: Hong Kong, 10 Jan 1930, *Chun 40185* (K); sin. loc, *Forbes s.n.* (BM); Castle Peak, power station, 2 Jun 1993, *Hu* & *But 22241* (A); Chung Chi College, New Territories, 1 Jun 1968, *Shiu Ying Hu*, *5432* (K); Lamma Island, 30 Mar 1969, *Shiu Ying Hu*, *6781* (K); Hong Kong, Shatin, Kowloon, Chuck-Pok-Hang, 15 Mar 1940, *Taam Ying-Wah*, *1144* (F); Hong Kong [Hong Kong Island], Nov 1857, *Wilford 68* (K); Hong Kong [Hong Kong Island], *Without Collector 330* (K); Hong Kong [Hong Kong Island], 1853, *Wright s.n.* (K); 1853, *Wright 347* (GH). **Jiangsu**: Wu County, Wu County, Mt. Dong, Dongshan, lake side of Lake Taihu, 1 Nov 1993, *Konta 3309* (GH). **Macao Special Admin Region**: Macau, Macao, weed of the Old Fort, 11 Jun 1975, *Lewis 8003* (MO); Macao, below Bishop residence, 19 Apr 1969, *Shiu Ying Hu 7009* (K). **Yunnan**: sin. loc, 1875, *Anderson s.n.* (K); Gaoligong Shan Region. Lushui Xian, Luzhang Zhen In the vicinity of Liuku on the W side of the Nu Jiang, 31 Oct 1990, *Dulong Jiang Investigation Team 46* (CAS); Fugong Xian, Maji Xiang, Mujiajia village, on the W side of the Nujiang, c. 6.5 direct km S of the border with Gongshan Xian, 28 Apr 2004, *Gaoligong Shan Biodiversity Survey 19656* (BM); Longling Xian, Zhen'an Zhen Vicinity of Songshan on the new rd from Baoshan to Tengchong via Nankang Yakou, 23 May 2005, *Gaoligong Shan Biodiversity Survey 23485* (BM, E, MO);

Fugong Xian, Maji Xiang, Majimi Cun, nr the Nujiang in the vicinity of hte Maji hydroelectric power plant, E side of Gaoligong Shan, 16 Aug 2005, *Gaoligong Shan Biodiversity Survey 27683* (BM, GH); Gaoligong Shan Region. Fugong Xian, Zilijia Xian Tuoping Cun, c. 40 km S of Fugong on the W side of the Nujiang, E side of Gaoligong Shan, 23 Aug 2005, *Gaoligong Shan Biodiversity Survey 28955* (CAS, GH); Nujiang Lisu Aut. Pref., 35 km N of Likui, Yi Di Ba, Lushui Co, 10 Oct 1996, *Gaoligong Shan Expedition 7345* (E); Xishuangbanna, Mengyuan, village along rd from

Menglun to Mengla, 19 Sep 2007, *Knapp et al. 10112* (BM); Kunming Botanical Institute, Yuanbo Shan, 6 Apr 1981, *Li Sheng-tang 81-1327* (A); Nujiang Lisu, Gongshan, track from Bingzhonglou to Shi Meng Guan (Stone Gates), 10 Sep 1997, *Li* & *Kunming Institute of Botany 1997 8809* (MO); Qiaojia, plaine de Kiao-Kia, May 1913, *Maire 813* (E); Shang-pa Hsien, 5 Oct 1933, *Tsai 54780* (GH); Shang-pa Hsien, 9 Oct 1933, *Tsai 54844* (GH); Xishuangbanna, Mengla, 20 Apr 1992, *Tsi Zhanhuo 92-162* (MO); Mengla Xian, 30 Aug 1991, *Tsi Zhanhuo 91-390* (A, MO); nr Feng-tai-szu, Cheng-kiang, Mar 1939, *Tsiang* & *Wang 16371* (A).

COCOS (KEELING) ISLANDS. Direction Is, 18 Jun 1987, *Williams s.n.* (CANB).

COMOROS. **Anjouan**: Anjouan: Between Domoni and Moya and on South Coast, 13 Aug 1987, *D'Arcy 17602* (MO). **Grande Comore**: Grande Comore: Hotel Ylang Ylang, S side of Moroni, 6 Aug 1987, *D'Arcy 17521* (MO); Grande Comore, edge of Moroni, 7 Aug 1987, *D'Arcy 17535* (MO).

COOK ISLANDS. **Aitutaki**: Ootu, 29 Aug 1969, *Stoddart 2198* (US); Vaipae, on lagoon side, main island, 31 Aug 1969, *Stoddart 2249* (US). **Rarotonga**: Motutoa, nr Motu Toa, 30 Aug 1969, *Philipson 10168* (L, US).

CÔTE D'IVOIRE. Nr Grand Bassam, NW along rd to Abalasp 3.43W, 5.13N, 12 Nov 1968, *Hepper 5984* (MO). **Abidjan**: Abidjan, Market at Adjame, 6 Jun 1970, *de Koning 715* (MO); Abidjan, local market at Adjame, 31 May 1973, *de Koning 1743* (MO). **Comoé**: Ayamé, 15 Nov 1967, *Geerling* & *Bokdam 1498* (MO). **Lagunes**: nr Adiopodoumé, 17 km W of Abidijan, 4 Aug 1958, *Leeuwenberg 2130* (B, K, MO, P).

DEMOCRATIC REPUBLIC OF THE CONGO. **Bas-Congo**: Kimuenza, 14 Feb 1968, *Pauwels 5099* (MO). **Katanga**: Parc National de l'Upemba, Lusinga, 6 Aug 1947, *de Witte 2757* (K); **Nord-Kivu**: Ruindi, Oct 1937, *Lebrun 8015* (K).

EQUATORIAL GUINEA. Bata-Bome, 12 May 1994, *Do Carvalho 5522* (MA, MO); Bata, 6 Aug 1957, *Davies 222* (K). **Annobon**: Palé-Lago A. Pot, margens esquerda, 28 Sep 1987, *Fidalgo de Carvalho 3150* (MA); subiendo al pico Lago desde el lago A. Pot, 23 Nov 2010, *Velayos et al. 11623* (MA).

ERITREA. **Maekel**: Asmara, Aug 1892, *Ragazzi 53* (FT). **Semienawi Keyih Bahri**: Ghinda, Sep 1892, *Ragazzi 158* (FT).

ETHIOPIA. **Shewa region (SU)**: Addis Ababa, Giardino Castagna, 30 Mar 1937, *Senni 310* (FT).

FIJI. **Rotuma Islands**: Haua Meamea Island, 1 Aug 1938, *St. John 19374* (US). **Viti Levu**: Serua, flat coastal strip in vicinity of Ngaloa, 26 Nov 1953, *Smith 9450* (K, L, US).

FRANCE. **Grand Est**: Haut Rhin, Ensisheim, 2 Sep 1961, *Rechinger s.n.* (W). **Occitanie**: Hérault, Montpellier, Hort. Delile, *Without Collector s.n.* (K).

FRENCH POLYNESIA. **Society Islands**: Moorea, Oponohu, nord du domaine, 21 Aug 1983, *Florence 5031* (US); Tahiti, FAAA, route du Mt. Marou, sous le plateau Mamanu, 24 Mar 1982, *Florence 2724* (US); Tahiti, Mont Marau, 10 Oct 1982, *Florence 3946* (US); Tahiti, ridge E of Vallee de Tuauru above Mahina, 29 Jul 1981, *Fosberg* & *Stone 61328* (BM, US); Tahiti, Distr. Pare: Diadem, 14 May 1930, *Grant 3623* (US); Tahiti, Papenoo, Oct 1909, *Leland et al. 78* (BM, E); Tahiti, sin. loc, 1850, *Ribourt s.n.* (US); Tahiti, Mont Marau, 27 Jun 1976, *Sachet* & *Jay 2254* (US); Tahiti, sin. loc, 25 May 1922, *Setchell* & *Parks 137* (US); Tahiti, Distr. of Papenoo, Oct 1909, *Tilden et al. 78* (US).

GABON. **Estuaire**: Libreville Nord, Santa-Clara, 20 Feb 2011, *Ikabanga 110* (MO); CENAREST, Libreville, Pelouse, 14 Jan 1988, *Louis 2292* (MO). **Ngounie**: Village Divinde, 21 Jan 2008, *Mamadou 194* (MO).

GAMBIA. Yundum, 2 Jul 1979, *Terry 3027* (K).

GHANA. sin. loc, 29 Dec 1923, *Eady s.n.* (K). **Central**: Otsiabo Hill, nr Aseba, 6 Nov 1962, *Hall 2411* (K); Cape Coast [castle], 20 Nov 1962, *Hall 2416* (K); Cape Coast (Castle), 10 Jun 1979, *Hall* & *Bukenya 47120* (K, MO). **Volta**: Ancobra Bridge, Axim, 11 Mar 1952, *Morton 8101* (K); Aguapim, *Vogel s.n.* (K). **Western**: Ancobra River Ferry, W Region, 10 Nov 1982, *Hepper 7453* (K); Busua Bay, on coast, 30 Mar 1954, *Morton A-435* (K); Busua Bay, 22 Feb 1956, *Morton A-1796* (K, MO).

GUAM. Commarianas, 2 Sep 1949, *Anderson, 128 b* (US); sin. loc, *Constable 1192* (US); jsut south of Ylig Bay, E coast of island, 3 Jul 1946, *Fosberg 25348* (US); just S of North Field, 15 Apr

1950, *Fosberg & Anderson 32631* (US); Dededo, 27 Dec 1953, *Fosberg 35310* (US); sin. loc, *Guam Experiment Station 438* (US); Ritidian Point, 1 Jul 1946, *Hosaka 3112* (US); Mt. Tenjo, 7 Mar 1946, *Moore 327* (US); Haputo Point, 21 Aug 1945, *Necker 16* (US); Pati Point, 19 Sep 1945, *Necker 326* (US); Ritidian Point, 27 Feb 1963, *Stone 4701[b]* (DUKE); Mangilao, College campus, 28 Apr 1962, *Yshmull 60* (L).

GUINEA-BISSAU. **Tombali**: Bissau, no terreno anexo a reparticao dos S.A.V, 10 Jan 1961, *Raimundo & Guerra 838* (MA).

INDIA. Herb. Wight Wallich 1830 H.I 2615, 1830, *Herb. Wight s.n.* (K); in montibus Nilagiri, *Hohenacker 1077* (K, W); Chooron nr Rurai [illegible], 7 Jul 1850, *Jameson 96* (K); Haueylhangh, 19 Jun 1849, *Jameson 153* (K);Peninsula India Orientalis, *Rottler s.n.* (K); *Roxburgh s.n.* (E); East India [ex herb. Forsyth], 27 Oct 1953, *Roxburgh s.n.* (K); regio Maisor & Carnatic, *Thomson s.n.* (W); sin. loc, *Wallich 2615 E* (BM); Peninsula Ind. Orientalis, *Wight 2326* (K); sin. loc, *Without Collector s.n.* (BM); planes Doust [d'Ouest], *Without Collector 303 /138* (K); **Andaman and Nicobar Islands**: Little Andaman, Dugong Creek, 2 Sep 1976, *Bhargava NANC-4371* (PBL); North Nicobars, Katchal Island, Mildera, 9 Nov 1976, *Chakraborty PANC-4650* (PBL); Lehagas achipelago, 13 Mar 1975, *Harris 90* (BM); South Andamans, Port Blair, Dairy Farm, 5 Apr 2008, *Kumar SANC-26937* (PBL); South Andamans, Port Blair, Corbyn's Cove Rd, 17 May 2008, *Kumar SANC-26968* (PBL); South Andamans, Dhanikhari, 13 Oct 1977, *Nair GANC-6250* (PBL); Little Andaman, South Bay, 29 Nov 2005, *Rasingam LANC-25856* (PBL); North Nicobars, Katchal Island, Mildera Forest, nr spices introduction area, 28 Mar 1979, *Vasudeva Rao KANC-7429* (PBL); South Nicobars, Chapin Island, 27 Apr 1988, *Vasudeva Rao KANC-13066* (PBL); South Andamans, Cadellganj, 8 Dec 1893, *Without Collector s.n.* (BM); **Andhra Pradesh**: Cuddapah, Houleylunda, Jul 1884, *Gamble 15153* (K); West Godavari, Devarapalli, on the rd, 29 Sep 1920, *Nohan 312* (CAL); **Arunachal Pradesh**: Lohit valley rd, Mar 1916, *Alexande 41* (CAL); Lohit valley rd, Mar 1916, *Alexande 43* (CAL); Lohit valley rd, Mar 1916, *Alexande 52* (CAL); West Kameng, Sessa, 12 Sep 1964, *Joseph, J39848* (CAL); Lohit, 8 km from Kherem towrads Wakroo, Lohit Distr., 30 Nov 1969, *Joseph, J48402* (CAL); Anjaw, Assam, Walong, 7 Oct 1950, *Kingdon-Ward 20240* (BM); West Kameng, Sissini, 27 Mar 1957, *Panigrahi 6157* (CAL); West Kameng, Kalaktang, 14 May 1958, *Panigrahi 15543* (CAL); Lohit, Foothills of Badam to Sonegodam, 10 Nov 1957, *Rao 10291* (CAL); Lohit, Foothills of Badam to Sonegodam, 10 Nov 1957, *Rao 10316* (CAL); Lohit, Paya to Shoeliang, 28 Nov 1957, *Rao 10857* (CAL); Lohit, Shoeliang to Dreign, 30 Nov 1957, *Rao 10879* (CAL); East Siang, Geling-Kepangla, 10 Nov 1958, *Rao 17489* (CAL); **Assam**: Goalpara, nr Goalpara Rest House, Goalpara Distr., Assam, 26 Apr 1964, *Henry 52* (CAL); Hailakandi, Nunai Beat House to Bhutan boarder, 23 Apr 1958, *Nath 13362* (CAL); Namchik River valley, 21 mile mark on Ledo rd, 3 Aug 1945, *USA Typhus Commission et al. 71* (K); **Bihar**: Nalanda, Rajgir, 17 Mar 2005, *Ghosh 37675* (CAL); Betiah, Gonauli forest margin area, Valmiki Tiger Reserve, Aug 2002, *Rajeurlar & Singh 31057* (CAL); **Chhattisgarh**: Surguja, Kamaleswarpur [Kamleshwarpur], Sambalpur [=Surajpur?], Surguja [distrisct] State, 5 Jun 1943, *Mooney 2236* (K); Surguja, nr Kumalesurarpur, Mainpat, 5 Jun 1943, *Mooney 22361* (DD); **Haryana**: on way Kamal to Assandh, nr Kamal, 28 Mar 1998, *Kumar NC-92384* (BSD); Panipat, 16 Oct 1962, *Nair NC-24678* (BSD); Hisar, 25 Apr 1962, *Nair NC-19915* (BSD); Rohtak, Bhalot, 14 Aug 1962, *Nair NC-23226* (BSD); Yamunanagar, Kalesar National Park, nr Kalesar temple, 18 Nov 2014, *Shukla & Srivastava NC-123920* (BSD); Panipat, Punjab, 27 Aug 1959, *Vohra NC-9900* (BSD); **Himachal Pradesh**: Nadaun, L bank of Beas River, 7 Aug 1977, *Without Collector NC-61548* (BSD); Kyelang, Lahul Distr., 10 Aug 1970, *Bhattacharyya NC-40707* (BSD, CAL); Bilaspur, Bilaspur to Barighat [Badhiat], nr Nathi, 27 Apr 1977, *Lai NC-63043* (BSD); Dharamshala, 21 Jul 1963, *Malhotra NC-28784* (BSD); Hoshiarpur, Pandoga, 11 Jul 1971, *Misra NC-44450* (BSD, CAL); Mandi, Panarsa, 28 Aug 1971, *Murti & Prasad NC-62161* (BSD); Nichar, 28 May 1962, *Nair NC-22000* (BSD); Rampur, 24 Aug 1963, *Nair NC-29874* (BSD); Sunah, Mahasur Distr., 20 Jul 1965, *Nair NC-35793* (BSD); Solan, Kundra, 22 May 1986, *Pani NC-80670* (BSD); Kangra, Pong Dam, reservoir, 31 Oct 2000, *Srivastava NC-96090* (BSD); Renuka Lake, 13 Feb 2002, *Srivastava NC-96751* (BSD); Una, on way to Jogi Panga Khad, 16 Jul 1977, *Uniyal NC-61013* (BSD); Sahu, Kalan Village, 14 Jun 1974, *Wadhwa NC-52902* (BSD); Sahu nala valley, 15 Jun 1974, *Wadhwa NC-52914* (BSD); Rakhi along Ran Aira, 2 Jul 1974, *Wadhwa NC-63483* (BSD); **Jammu & Kashmir**: Kathua, Billawar, 10 Aug 1986, *Hajra NC-82320* (BSD); Udhampur, Jammu to Srinigar highway, 26 May 1986, *Kumar NC-81210* (BSD); Shalimar, outside garden, 2 Nov 1977, *Rao NC-63640* (BSD);

Chandanwari, 4 Jul 1956, *Rao NC-444* (BSD, CAL); Chandanwari, 16 Jul 1956, *Rao NC-626* (BSD); on way to Buchamandi, 14 Sep 1996, *Uniyal NC-92625* (BSD); Ladakh, Menchigaon, on Kasgil [=Kargil]-Suru rd, 5 Sep 1975, *Visanatham NC-55656* (BSD); Dachigam Game Sanctuary [=Dachigam National Park], 6 Sep 1977, *Wadhwa NC-66840* (BSD); Samblar, 25 Aug 1987, *Wadhwa & Murti NC-85432* (BSD); Kargil, 5 Sep 1976, *Wadwha NC-60187* (BSD); **Jharkhand**: Kathara Dumping place, Chotanagon Coalfield region, Bhar, East Bokaro, 6 Oct 1996, *Chandra 1304* (CAL); Palamau, Neleshat, 27 Sep 1981, *Manua 998* (CAL); Palamau, Netarhat, 2 Oct 1981, *Manua 1092* (CAL); Chatra, Nazaribagh [Chota Nagpur], 10 Jan 1878, *Wood s.n.* (K); **Karnataka**: Hassan, Halebid-Hassan Rd, 22 Jan 1970, *Saldanha 16110* (K, MO); **Kerala**: Tinnevelly, *Beddome s.n.* (BM); Keezhariyur, Naduvathunastra, Aug 1913, *Calder & Ramaswami 153* (CAL); **Maharashtra**: Khandala, Kune Station, 27 Apr 1944, *HS Blatter Herb. 4087* (K); Thane, *Hügel 1723* (W); Nr Baggi village 5-8 km from Claragard to Iruni route, 30 May 2010, *Meenakshi 65* (DD); Nagpur, Ramdaspeth, 5 Dec 1962, *Nafday 30* (CAL, K); **Odisha**: Pustiguda, Sambalpur, Orissa, 21 May 1944, *Mooney 2473* (DD, K); Bhubaneswar, Orissa, 17 Jan 1971, *Zimprich s.n.* (W); **Punjab**: Gurdaspur, Bahala, 24 Aug 1969, *Bhattacharyya NC-37783* (BSD); Madhopur, 7 Aug 1983, *Charanpreet 9456* (BSD); Hoshiarpur, Urmar Tanda, 13 Sep 1979, *Daniel NC-67369* (BSD); Hoshiarpur, 25 Aug 1970, *Misra NC-41511* (BSD); Lambi, Freozpore Distr., 21 Feb 1963, *Nair NC-26179* (BSD, CAL); Hoshiarpur, 23 Aug 1977, *Vohra & Daniel NC-6035A* (BSD); **Rajasthan**: Lohargal, Mar 1960, *Nair NC-1099* (BSD); **Sikkim**: North Sikkim, Dikchu, 13 May 1945, *Biswas 6754* (CAL); East Sikkim, surrounding Enchey monastery, 22 Feb 1980, *Chakraborty BSHC-925* (BSHC); East Sikkim, Ranipul, 4 Apr 1981, *Chakraborty BSHC-1176* (BSHC); East Sikkim, Pandam, 16 Dec 1981, *Chakraborty BSHC-1934* (BSHC); East Sikkim, Rangpo, dam site, 11 Nov 1998, *Dash BSHC-21171* (BSHC); North Sikkim, Tumloong, 13 Jul 1892, *Gammie A276* (CAL); East Sikkim, Gangtok Development Area, 1980, *Hojra BSHC-37* (BSHC); South Sikkim, Maenam Wildlife Sanctuary, 12 Aug 2005, *Hynniewta BSHC-31971* (BSHC); Sikkim, 21 Apr 1913, *Lacaita s.n.* (BM); North Sikkim, Nampuk, 20 Jun 1999, *Maity BSHC-21633* (BSHC); North Sikkim, Bey, 17 Sep 2000, *Maity BSHC-23245* (BSHC); East Sikkim, Gangtok, 6 May 1967, *Majunder 8* (CAL); East Sikkim, Gangtok, 6 May 1967, *Majunder 10* (CAL); East Sikkim, Gangtok, 6 Sep 1968, *Majunder & Banerji 203* (CAL); North Sikkim, Chungthang, 24 Jul 1989, *Mandal BSHC-10021* (BSHC); North Sikkim, Dikchu, 29 May 1959, *Mukerjee 5016* (CAL); West Sikkim, Tauredaura, 15 Jul 2010, *Rai BSHC-24575* (BSHC); East Sikkim, Rengli Schola, 27 Jul 1985, *Raju BSHC-4088* (BSHC); West Sikkim, Alaya Bayer to Legislup Rd, 12 Feb 1996, *Shukla BSHC-18673* (BHSC, BSHC); West Sikkim, Genzing to Lingchiam, 20 Feb 1994, *Singh BSHC-15537* (BSHC); West Sikkim, Dentam, 1 Sep 1994, *Singh BSHC-16449* (BSHC); West Sikkim, Nayahoza-Soreng rd, nr Zorem village, 6 Jan 1994, *Sinha BSHC-15201* (BSHC); South Sikkim, Namchi-Damthiong rd, 5 km area, 8 Jan 1994, *Sinha BSHC-15236* (BSHC); Rungbee, 26 Oct 1908, *Smith 721* (CAL); North Sikkim, on way to Bay, 14 Aug 1989, *Srivastava BSHC-10371* (BSHC); South Sikkim, Kewzing, 18 Aug 1985, *Verma BSHC-4226* (BSHC); South Sikkim, Melli Reserve Forest, 25 Jul 1986, *Verma BSHC-6728* (BSHC); South Sikkim, Borong Hot Spring, 18 Dec 1985, *Viswe BSHC-4765* (BSHC); **Tamil Nadu**: Theni, High Wavy Mountain, May 1917, *Blatter & Hallberg 383* (CAL); Nilgiris, Coonoor, 26 May 1896, *Bourne s.n.* (K); Dindigul, Kodaikanal - Pulneys [Palni Hills], 20 Jun 1901, *Bourne s.n.* (K); Periya Shola, Pulneys [Palani Hills], 11 Jun 1897, *Bourne 359* (K); Coimbatore, Distillary rd, Lainbatore, 19 Apr 1964, *Chandrabose 28410* (CAL); Nilgiri, Nilghiris, Jun 1886, *Gamble 17435* (K); sin. loc. [Madras], *Gray s.n.* (K); Tirunelveli, Kannikatti, 21 Feb 1913, *Hooper & Ramaswami 39413* (CAL); Chennai, Vellore (Madras), 30 Nov 1825, *Hunter s.n.* (K); Tirunelveli, Kalakkadu R.F, 13 Nov 1962, *Joseph 15280* (CAL); Dindigul, Thoppukkadu, Karanthamalai, 7 Jun 1986, *Kumar 3350* (CAL); Theni, Kumbukarai, 17 Sep 1961, *Kurwari 12959* (CAL); Thuraiyur, Tiruchirappalli, Kolli Hills, Puliancholai W, 30 Nov 1978, *Manoharan 19575* (CAL, K); Krishnagiri, Dharmapuri, Anchetty village lands, 6 Sep 1978, *Matthew & Venugopal 17002* (CAL); Namakkal, Salem, Velur, Cauvery banks, 1 km below bridge, 10 Dec 1978, *Matthew 19984* (CAL); Dindigul, Kodaikanal, Palamalai-Varagarappari path, Pannikkarai aaru, Palni Hills, 17 Aug 1986, *Matthew & Charles 46324* (K); Salem, Namakkal, Kollihills, Koottaru, 19 Apr 1978, *Mohan 13135* (CAL); Ramanathapuram, Sethur R.F, 25 Feb 1979, *Nair 61452* (CAL); Nagapattinam, Nayapatranam, Thanjavur Distr., 27 May 1978, *Nair 57128* (CAL); Salem, Yercaud Distr., Shevaroys, Ghat rd, 10 May 1978, *Nohan 13505* (CAL); Nilgiris, Nilgenia, *Perrotet 903[a]* (W); Nilgiris, *Perrotet 904* (W); Nilgiris, *Perrotet 904* (W);

Salem, Shevaroys Distr., Vellayappankeil, 25 Aug 1978, *Perumal & Manoharan 16700* (CAL); Dharmapuri, Harur Distr., Shevaroys, Bommidi to K.N. Puthur Mallapuram Ghat rd, 26 Aug 1978, *Perumal & Manoharan 16724* (CAL); Coimbatore, Nellimalai R.F, 22 Mar 1963, *Ramamurthy 16047* (CAL); Gomuki river bank, South Ancot Distr., 7 Sep 1977, *Ramamurthy 50680* (CAL); Salem, Orchidarium area, 25 Jun 1964, *Rao 18242* (CAL); Kjaria, Koddaikanal, 21 Feb 1913, *Saulleres 63* (CAL); Coimbatore, Maruthamalai, Combatwe Distr., 14 Nov 1956, *Sebastine 1311* (CAL); Nilgiri, Kundha, 22 Jan 1957, *Sebastine 2173* (CAL); Tirunelveli, Courtallam falls, 21 Apr 1957, *Sebastine 2790* (CAL); Nilgiri, Upper Tiger Shola, Coonoor, 25 Jul 1957, *Sebastine 4008* (CAL); Tirunelveli, Way to Kadlamalar estate, Kanni, 11 Jul 1959, *Sebastine 8488* (CAL); Coimbatore, Aligar submergible area, 24 Nov 1962, *Sebastine 15356* (CAL); Coimbatore, Sholaigor submergible area, 27 Jul 1963, *Sebastine 16691* (CAL); Madurai, Nr Pomparai-Kodaikanal, 27 Jul 1965, *Sebastine 24589* (CAL); Ramanathapuram, Krishnathevarthoppu, Mamsapuram, 10 Mar 1980, *Srinivasan 63653* (CAL); Coimbatore, On the way to Varapalayam, Combratore Distr., 6 Jul 1956, *Subramanyam 142* (CAL); Jamunamarathur, Nr Komattiyur, 7 Sep 1958, *Subramanyam 6473* (CAL); Salem, roadside Pennagaram, 4 Dec 1964, *Vajravelu 22435* (CAL); Nagapattinam, from Wight, Negapotam, *Wight s.n.* (A); **Telangana**: Medak, Narsapur town, 27 Sep 1958, *Sebastine 6782* (CAL); **Tripura**: North Tripura, Dasda, 5 Feb 1962, *Deb 27496* (CAL); **Uttar Pradesh**: Bijnor, 21 Jan 1983, *Husain s.n.* (K); Lakhimpur, 31 Aug 1962, *Malhotra NC-23596* (BSD, CAL); Saharanpur, Bhagwanpur, Jun 1983, *Murty & Goel 56* (BSD); Barhpura, 17 Mar 1953, *Sarin NC-5159* (BSD); Bijnor, E of Najibabad toward Katdawar, 2 Nov 1979, *Sharma NC-67127* (BSD); Bulandshahr, Pahasu, R. Shaba, 20 Aug 1964, *Singh NC-31930* (BSD); Unchapul, Haldwani, 16 Jun 1962, *Singh 42* (BSD); Moradabad, Moradabad, Mar 1845, *Thomson 62[b]* (K); Noida, 15 May 2002, *Uniyal NC-91885* (BSD); Pathari, 17 Sep 1980, *Wadwha NC-62972* (BSD); **Uttarakhand**: Almora, Kalyani Ashram, 30 Nov 2017, *Ambrish NC-120913* (BSD); Dehradun, Rispana, 12 Dec 1964, *Babu NC-34679* (BSD); Pithoragath Distr., Basbagar, 20 Apr 1984, *Balodi NC-75653* (BSD); Dehradun, Rajpur Rd, 15 Apr 1961, *Bhattacharyya NC-13891* (BSD); Dehradun, Nathuwala, 13 Nov 1961, *Bhattacharyya NC-18435* (BSD); Tenri Garhwal, Kirtinagar, 9 Apr 1978, *Goel NC-53907* (BSD); Chamoli, on way to Lala, Aug 1988, *Hopa NC-87129* (BSD); Karanpur, 4 Oct 1989, *Kaur 16* (BSD); Dehradun, Dehradun Jungle, India, 27 Mar 1970, *van der Maesen 962* (WAG); Dehradun, Sahastradhara, 17 Jan 1962, *Malhotra NC-19716* (BSD); Dehradun, Sahastradhara, 15 Jul 1960, *Malhotra & Malhotra NC-12018* (BSD); Dehradun, Swala, 18 Jul 1960, *Malhotra & Malhotra NC-12054* (BSD); Dehradun, Mussoorie, Lal Tibba, 6 Aug 1960, *Malhotra NC-12464* (BSD); Rudraprayag, 19 Apr 1963, *Malhotra NC-26752* (BSD); Garhwal, Agastyamuni, 21 Apr 1963, *Malhotra NC-26817* (BSD); Lacchiwala, 17 May 1961, *Malhotra NC-15364* (BSD); Dehradun, Rajpur, Canal Rd, 19 May 1961, *Malhotra NC-15874* (BSD); Dehradun, Nathuwala, 3 Jan 1962, *Malhotra NC-19165* (BSD); Dehradun, Govind Pashu Vihar National Park, Alaxa on way to Taluka, 10 Sep 2010, *Manikandan, RNC-112491* (BSD); Dehradun, Travor rd, New Forest, 18 Sep 1968, *Naithani 6018* (DD); Nainital, Corbett National Park, Babalmunda, 29 Nov 1970, *Pant NC-43450* (BSD, CAL); Nainital, Domunda Bridge, 25 Apr 1971, *Pant NC-43694* (BSD); Uttarkashi, Gangotri National Park, between Gunshi and Bhaironghati, 21 Aug 2003, *Pusalkar NC-104639* (BSD); Dehradun, Song River bank, 26 Oct 1956, *Rao NC-1061* (BSD); Samadhura-Tejam, Kumaon Distr., 2 Jun 1958, *Rao NC-6584[b]* (BSD); Dehradun, Mussoorie, The Mall, 19 Dec 1956, *Rau NC-1318* (BSD); Dehradun, 11 Sep 1968, *Rau NC-12625* (BSD); Balaw Gangnani, Uttar-Kash forest division Sehme Garhwal, 12 Jun 1956, *Sahmi 25025* (DD); Dehradun, Mussoorie, Barlowganj, 27 Dec 1956, *Sarin NC-1501* (BSD); Dehradun, Mussoorie, Barlowganj, 27 Dec 1956, *Sarin NC-1509* (BSD); Dehradun, Rajpur, Dehradun, 17 Apr 1960, *Saxena 525* (DD); Mussoorie, Nr Bhatta fall, Mussoorie, 22 May 1960, *Saxena 582* (DD); Mussoorie, Barloganj, Mussoorie, 19 Aug 1961, *Saxena 2159* (DD); Uttarkashi Distr., Janki Chatti, 8 Oct 1993, *Semajunde & Singh NC-73194* (BSD); L side of Yumuna [=Yamuna] River, Da(R) Pathar [= Dakpatthar], 15 Jul 1985, *Sharma NC-70364* (BSD); Dehradun, Harbanswala Tea Estate, 24 May 1962, *Singh NC-19653* (BSD); Dehradun, Harbanswala Tea Estate, 9 Nov 1962, *Singh NC-23085* (BSD); Dehradun, Niranjanpur, 7 Nov 1963, *Singh NC-31620* (BSD); Dehradun, Niranjanpur, 7 Nov 1963, *Singh NC-31632* (BSD); Jarmola, Myvarkashi Distr., 5 Oct 1995, *Singh NC-90276* (BSD); Dehradun, 192 Kanlagonh Rd, Oct 1994, *Singh NC-90499* (BSD); Dehradun, 1928, *Singh 12* (W); Garhwal, Hardiwar, *Srivastava NNC-105544* (BSD); Dehradun, Hathibarkala, 19 Sep 1956, *Staff NC-857* (BSD); Pauri Garhwal, Oct 1975, *Tiwari 35A* (DD); Garhwal, Marola [Mahraula?]-Landsdowne,

26 Feb 1960, *Vohra NC-11244* (BSD); Almora, Binsar, on rdside below F.R.H, 20 Oct 1975, *Vohra NC-58005* (BSD); **West Bengal**: Murshidabad, Berlampur, 13 Oct 1965, *Bakari 144* (CAL); Uttar Dinajpur, Sibdangi, Islampur, West Dinajpur, 5 Nov 1983, *Banerjee 16180* (CAL); Kolkatta[Calcutta], Birati, 24 Parganas, 19 Apr 1967, *Bark 109* (CAL); 24 Parganas, Garia, 22 Aug 1986, *Barunjee 18760* (CAL); Kolkatta[Calcutta], Calcutta maiden nr Esplanade, 28 May 1980, *Biswas & Debnath 244* (CAL); Maldah, Bengal, Maldah, 30 Apr 1875, *Clarke 26311* (BM); Bogra, 30 Dec 1875, *Clarke 26835* (CAL); Darjeeling, Kurseong, 25 May 1966, *Das 209* (CAL); Howrah, Baksara, 4 Jan 1961, *Dhoromick, D.C5291* (CAL); Malda, Saluka hill area, 28 Apr 1966, *Dutta 261* (CAL); Malda, Bhaluka Rd, 26 Aug 1966, *Dutta 507* (CAL); Kolkatta[Calcutta], Village Bolekhali (salt lake), suburban Calcutta, 20 Mar 1962, *Ghosh 57* (CAL); Kolkatta[Calcutta], Vill. Nalbon, Salt Lakes, Suburgan Calcutta, 24 Mar 1967, *Ghosh 75* (CAL); Kalimpong, Samsing and surrounding areas, 29 Apr 1981, *Krishna, BBSHC-1526* (BSHC); Darjeeling, Sukna, 10 Mar 2008, *Paul & Kumar 43603* (CAL); Darjeeling, Sevoke to Kalighora hill slopes, 14 Mar 2008, *Paul & Kumar 43752* (CAL); Kolkatta[Calcutta], Garfa, 24 Parganas, 9 Feb 1961, *Safrui 20* (CAL); Darjeeling, Munsong, 28 Apr 1965, *Santapau & Mukerjee 204* (CAL); Jalpaiguri, Changmari, 24 Feb 1975, *Sikdar 126* (CAL); Jalpaiguri, Apalchand, 26 Feb 1975, *Sikdar 167* (CAL); Jalpaiguri, Chilapata, 25 Feb 1976, *Sikdar 4303* (CAL); Jalpaiguri, Bania, 26 Feb 1976, *Sikdar 4338* (CAL); Birbhum, Ballarpur Mouza nr Bolpur, 5 Mar 2008, *Sinha 43249* (CAL); 24 Parganas, Baranagore, 12 Dec 1971, *Tribedi 1* (CAL); 24 Parganas, Parmadan Forest, 21 Mar 1972, *Tribedi 201* (CAL); Kolkatta[Calcutta], Calcutta [Kolkata], Mar 1945, *Without Collector s.n.* (K); Kolkatta[Calcutta], Hort. Bot. Cal., 1856, *Without Collector s.n.* (K).

INDONESIA. **North Maluku**: Ternate, 3 Sep 1951, *Idjan* & *Mochtur 63* (L). **East Nusa Tenggara**: Timor, Timor Tengah Selatan, Mollo Selatan, Hutan Naususu, 23 Feb 1980, *Widjaja 1360* (L). **Java**: West Java, Tjibodes (Gede), lage torgangspad van berghuis, 30 Apr 1950, *van Orststroom 13791* (L); Sin. loc, 1855, *Waitz s.n.* (GOET); sin. loc, *Zollinger 1279* (A). **Maluku**: Wetar, Wexar umbegung des Tihuscas, 4 Mar 1911, *Elbert 4547* (K); Ternate, 3 Sep 1951, *Idjan* & *Mochter 63* (K); **South Sulawesi**: Enrekang, Enrekang Distr., Latimojong Mts., valley c. 1.5 km to the N of Bunte Tjejeng, 1.5 km from Base Camp, 1 Nov 1969, *Sands 333* (GH, K). **Sulawesi**: Menado Paloe, ten Osten van het Lindoe-mee, naar den top der Goenoeng Ngilalaki, 9 Jul 1939, *Bloembergen 3995* (A); Taweli Karumba, Sulawesi Tengah, Kecamatan Paul Taweli, 31 Mar 2000, *Kessler 3103* (K, L). **Sumatra**: Asahan, east coast, 1918, *Bartlett* & *La Rue 479* (GH); Korinchi, Sandaran Agong, 25 Apr 1914, *Robinson* & *Kloss 86* (BM); Korinchi, Sandaran Agong, 29 May 1914, *Robinson* & *Kloss 175* (BM); Tapianoeli, subdivision Toba, Distr. Toetoepan, 4 Nov 1933, *Si Boeea 5991* (A, US); east coast, vicinity of Loebam Ria, Asahan, 5 Feb 1935, *Si Boeea 7308* (GH); Asahan, vicinityof Loemban Ria, *Si Boeea 8026* (A, US); Asahan, Aer Djoman, Asahan, E of Serbangan, Jul 1935, *Si Boeea 8301* (US); Adian Rindang, Asahan, vicinity of Hoeta Tomosan Dolok (Toemoean Dolok), 17 Nov 1935, *Si Boeea 8611* (US); Asahan, vicinity of Aek Moente (Aer Moette), Asahan, northeast of Tomonean Dolok and W of Salabat, 15 Jun 1936, *Si Boeea 9227* (L, US); Asahan, headwaters of Aek Liang, Asahan (region between Dolok Si Manoek-manoek and Tor Matoetoeng), 15 Oct 1936, *Si Boeea 10755* (US); Ketambe Research Station and vicinity, Atlas River valley, 20 Jun 1979, *de Wilde* & *de Wilde-Duyfjes 18171* (L). **Timor**: Sin. loc., *Forbes 3785* (US).

JAPAN. Ryokkai-mura, Sambu-gun, Prov, Kadzusa, Prefeceture China, Hondo, 5 Sep 1962, *Furuse s.n.* (A). **Bonin Islands**: Oho-mura Is. Chichi-jima, Ogasawara Islands (Bonin Islands), 12 Jan 1975, *Furuse 7793* (K); Bonin Island, Nov 1907, *Without Collector s.n.* (E); Sin. loc., 1853, *Wright 196* (GH, K). **Honshu**: Honshu Island, on Iwakuni air base, Iwakuni City, on the N bank of the Imazu river, 25.6 mi W of Hiroshima city, 8 Aug 1954, *Charette 1866* (MO); Ryokki-mura Sambu-gun Prov. Kadzusa, PreChiba, Hondo, *Furuse 40504* (K); Miyagi, Mt. Inokurayama, Iwanuma-shi, 16 Oct 1990, *Koga* & *Kurosawa 146* (MO); Kameoka, Kyoto, Japan, Honshu, Kyoto-fu, Mikkaichi, Umaji-cho, Kaeoka-shi, 10 Sep 2006, *Tsugaru et al. 3378* (GH); Miyagi, Sendai-shi, Taihaku-ku, Tomizawa-2-chome, around Tomizawa site museum, 6 Oct 1997, *Yonekura* & *Nagamatsu 97453* (MO). **Kyushu**: Nagasaki, Sasebo-shi, Yokoo-cho, 31 Dec 1994, *Yonekura 3620* (A, MO). **Okinawa**: Okinawa, N of Kadena, 12 Mar 1955, *Moran 4978* (E). **Ryuku Islands**: Yoshiwara, Is. Ishigakim PreOkinawa, 8 Jan 1972, *Furuse 1962* (K); Izena Island, Chinjinyama, 24 Mar 1979, *Tamura et al. 26714* (B). **Shikoku**: Kochi, Between Zuchuu and Amaji, Ohtuki Town, 14 Oct 2003, *Miyazaki 0310334* (MO).

KENYA. Rabai Hills, Mombaz. Place Kisulutini, Sep 1885, *Taylor s.n.* (BM). **Central**: Machakos, Kilima Kiu, 1926, *Prescott-Decie s.n.* (BM). **Coast**: Kwale, Inepanga, Chumpe Swano, Daruma Distr., 22 Mar 1902, *Kässner 434* (BM, K); Magarini, 1 Nov 1982, *Robertson 3454* (K); Rabai Hills, Aug, *Taylor s.n.* (BM); Kilifi, Rabai Hills, Mombaz, Jul 1885, *Taylor s.n.* (BM).

LAOS. sin. loc., *Spire 1060* (P); sin. loc, 8 Apr 1955, *Tixier s.n.* (P). **Luang Prabang**: prov. Louang Prabang, Ban Long O, 2 Mar 1932, *Pottier 36c* (P); prov. Louang Prabang, Ban Xiengmène, rive du Mékong, 22 Jun 1922, *Pottier 171* (P). **Savannakhet**: km 20 route de Savannakhet à Quang Tri, 24 Jan 1925, *Poilane 11791* (P). **Xiangkhouang**: Prov. Tranminh, environs de Xieng-Khouang, 1917, *Miéville 37297* (P).

LIBERIA. **Grand Bassa**: Grand Bassa, 15 Oct 1899, *Dinklage 2116* (B, GH). **Montserrado**: Sinkor, nr Monrovia, 18 Sep 1963, *van Marten 100* (K). **Nimba**: Nimba Expedition, 9 Jan 1965, *Adam 20529* (K, MO). **Sinoe**: nr airport, 18 Jan 1969, *Jansen 1193* (MO).

MADAGASCAR. Sin. loc., *Balfour s.n.* (K); Angavokely, 24 Apr 2010, *Cabezas et al. 1191* (MA); Ambatobe, 7 Jul 1917, *Decary s.n.* (P); Sin. loc., 1 Apr 1928, *Decary 6179* (K, P); Vallée de l'Anilahy, vallon d'Amdranalahy, 5 Feb 1947, *Humbert 20182 bis* (P); Central Madagascar, *Without Collector 4269* (K). **Antananarivo**: Antananarivo-Nord, environ de Tananarive, 5 Aug 1950, *Benoist 62* (P); Tsimbazaza, 11 May 1935, *Boiteau 37 1* (P, TAN); Ankaratra, Nov 1955, *Bosser 8658* (P); Ambanitsena, vallée de Soavina; K. 23, route de Tamatave, Nov 1960, *Bosser 14707* (MO); Antananarivo-Nord, vicinity of city center, 15 Jan 1975, *Croat 28440* (K, MO, P); Antananarivo-Nord, Distr. Imerina, 15 Jan 1975, *Croat 28489* (K, MO, P);Along route number 2 to Tamstavive from Tananarive between Ahimangakely and Carion, in valley between Ampasimbe River, 16 Jan 1975, *Croat 28522* (EA, MO); Analamanga, rd from airport to Tananarive, 22 Apr 1983, *D'Arcy* & *Randrianasolo V.15201* (MO); Analamanga, between village of Ambatofotsy and irrigated lands flanking River Sisaony, 23 Apr 1983, *D'Arcy* & *Rakotozafy 15212* (MO); Analamanga, weeds around Ambotofotsy, village 21 km S of center of Tananarive, Sep 1987, *D'Arcy 17681* (MO); Analamanga, weeds around Tananarive, Sep 1987, *D'Arcy 17684* (MO); Antananarivo-Nord, 2 Apr 1943, *Decary 19370* (P); Fenoarivo-Centre, Beanana, centre Ouest, vestige forestier, sur le Tampokatsa, Jun 1951, *Bosser 926* (P); Vakinankaratra, Behenjy, 6 May 1983, *D'Arcy* & *Rakotozafy 15304* (MO); Vakinankaratra, Behenjy, 6 May 1983, *D'Arcy* & *Rakotozafy 15305* (MO); Vakinankaratra, Edge of Ambatolampy, 7 May 1983, *D'Arcy* & *Rakotozafy 15307* (MO); Environs de Tananarive, 29 Jul 1928, *Decary 6656* (K, P); Parc Tsimbazaza, Antananarivo. Parc de Tsimbazaza, 6 Feb 1984, *Dorr 2729* (MO); environs d'Andromasina (30 km SSE de Tananarive), 7 Feb 1960, *Léandri et al. 3092* (P); bord de route Tananarive, Aug 1961, *Rakotozafy 59* (MO). **Antsiranana**: DIANA, Mont Ambohitra, c. 20 km S of Antsiranana, 14 Sep 1987, *D'Arcy 17697* (MO); DIANA, Mont Ambohitra, c. 20 km S of Antsiranana, 14 Sep 1987, *D'Arcy 17698* (MO); Antsiranana Rural, a la base Nord des collines et plateaux calcaires de l'Analamera (Prov. Diego-Suarez), Jan 1938, *Humbert 19091* (K, P); Antsiranana Rural, Joffreville, SW of Antsiranana, Parc National de Montagne d'Ambre, 16 Nov 1992, *Malcomber et al. 1716* (K, MO, MO, P); Vohemar, Daraina, 24 Nov 2005, *Nusbaumer* & *Ranirison 1674* (K, MO); Sava, Makirovana Massif, N of Sambava, 6 May 2010, *Taylor* & *Madaka 12967* (MO); Diego Suarez, River Cave, canyon forestiere, Ankarana, 3 Sep 1986, *Wilson 19* (K). **Fianarantsoa**: Fianarantsoa Rural, 9 Feb 2001, *Allorge* & *Rakotozafy 2632* (P); Mananjary, Prov. Mananjary, 1909, *Geay 8096* (P); Ranomafana PN, Eastern Domain, 7-10 km W of Ranomafana, 18 Aug 1987, *Schatz* & *Lowry 1444* (MO). **Toamasina**: Vatomandry, vicinity of Ivato, NW of Tananarive, 18 Jan 1975, *Croat 28684* (K, MO); Atsinanana, Toamasina (Tamatave), 2 May 1983, *D'Arcy 15282* (MO); Ambatondrazaka, Imerimandroso, 27 Jun 1921, *Decary 755[a]* (P); Ambatondrazaka, Lac Alaotra, Ilot d'Ambato, 27 Jun 1921, *Decary 755[b]* (K); Toamasina Urban, Tamatave, 1909, *Geay 8978* (P); Forêt d'Analalava, 8 km W of Foulpointe, 26 Oct 1996, *Miller et al. 8833* (MO). **Toliara**:); Anosy, Fort Dauphin, 10 May 1983, *D'Arcy* & *Rakotozafy 15348* (MO); Ambovombe-Androy, 6 Apr 1924, *Decary 2600* (P); Sud-Ouest, bas Mangoky, 5 Sep 1955, *Descoings 775* (MO); Beroroha, Oct 1933, *Humbert 11308* (P); Vallée de la Manambolo (bassin du Mangrare), au confluant de la Sakamalio, mont Morahariva, Dec 1933, *Humbert 13124* (P); Forêt entre Lambomakandro et Sakaraha, 10 Dec 1946, *Humbert 19707* (K, MO, PBekily, Ampandrandava, entre Bekily et Tsivory, Oct 1942, *Seyrig 163* (P).

MALAWI. **Southern**: Chikwana, Kamuzu Bridge, 28 Jul 1978, *Patel 204* (MO).

MALAYSIA. nr Kota Bahsee, Aug 1880, *Kunstler 566* (A); Alor Bukit, Johore, 22 Nov 1966, *Singh 528* (A). **Johor**: Alor Bukit, Johore, Malay Peninsula, 22 Nov 1966, *Hardial 528* (K). **Kuala Lumpur**: Ampang, 25 Jun 1924, *Burkhill SF-13939* (K). **Pahang**: S Palas Estate, Pahang, Cameron Highlands, 8 Sep 1956, *Burkhill 852* (K); Tringkap, Cameron Highlands, Pahang, 11 Oct 1961, *Burkhill 2876* (K); Sabai Estate, Bentong, Kemanshul Div., Pahang, 27 Jan 1958, *Shah 170* (K); along path to Lari Tembakau, Genting Highlands Hotel area, nr. Ulu Kali, Pahang, 18 Mar 1979, *Stone 14055* (L, MO); Pahang, Distr. of Bentung, Genting Highlands, vicinity of Genting Hotel, 15 Feb 1987, *Worthington 12432* (L). **Perlis**: Chupeng, Mar 1910, *Ridley 14983* (BM); Chuping, Perlis (Flora of Perlis), Mar 1910, *Without Collector 14983* (K). **Sabah**: Sensuron raod nr Gunong Alab, Crocker range, 17 Feb 1980, *Argent 1305* (E); Kota Marudu Distr., Kampung Sorinsim, 16 Jul 1998, *Bakia 603* (K); Tambunan Distr., Crocker Range, km 55 on Kota Kinabalu-Tambunan rd, 4 Sep 1983, *Beaman et al. 6908* (GH, K, MO, US); Ranau Distr., above W bank of East Mesilau River at Mt. Kinabalu golf course site, 22 Mar 1984, *Beaman et al. 9048* (GH, K, MO, US); Ranau Distr., East Mesilau River, between Mt. Kinabalu golf course and Mesilau Cave, 26 Mar 1984, *Beaman et al. 9079* (GH, K, MO, US); Ranau Distr., Pinosuk Plateau, East Mesilau River where rd to asparagus farm crosses it, 28 Jul 1984, *Beaman et al. 10798* (K); Mt. Kinabalu, Tenompok, 25 May 1932, *Clemens* & *Clemens 29744* (A); Nangke, Beluh Distr., Kampung Sisu Nuluh, 19 Mar 1993, *Duaneh 319* (K); Nangke, Beluh Distr., Kampung Sisu Nuluh, 19 Mar 1993, *Duaneh 320* (K); Kota Belud Sistrict, Kampung Kiau Toburi, 2 Jan 1994, *Duaneh 444* (K); Long Pasia, Ulu Padas, southwest Sabah, 2 Nov 1999, *Hoare* & *Barok 16* (K); Kota Belud Distr., Kampung Melangkap Tomis, 13 Jul 1998, *Lugas 2760* (K); Kampung Sorinsim, 3 Oct 1992, *Sibil 48* (K); Gurion, Kota Maradu Distr., Kampung Sorinsum, Kinabalu, 5 Apr 1993, *Sibil 171* (K); Kilou, Kota Maradu Distr., Kampung Sorsinsim, 14 Jul 1993, *Sibil 252* (K); Bundu, Kota Marudu Distr., Kampung Sorinsim, 16 Nov 1993, *Sibil 317* (K); Sebereng-bereng, Ranau Distr., Kampung Bundu Tuhan, Kampung Gondohon, 29 Sep 1992, *Soibeh 51* (K). **Selangor Darul Ehsan**: Selangor, Banting, 21 May 1987, *Chin 3713* (A, K). **Terengganu**: Trenganu [Terengganu], *Japp 362* (K); Kuala Terengganu, Jalan Masjid opposite Grand Hotel, Kuala Trengganu, 7 Nov 1954, *Sinclair SF-40388* (K).

MARQUESAS ISLANDS. **Hiva Oa**: partially dissected upland between Hanamenu and cave Anatuakina, 8 Jan 1964, *Decker 1280* (US); Hanaiapa, route S du village, 14 Mar 1977, *Schäfer 5306* (US). **Nuku Hiva**: Taiohae-Hatiheu trail, 5 May 1964, *Decker 2185* (US); Taiohae, route de Toovii, terrain des T.P., 29 Nov 1982, *Florence 4154* (L, US); route Toovii-Terre Deserte, km 6.5 après el col, 9 Dec 1982, *Florence 4365* (L, US).

MARSHALL ISLANDS. **Guam**: between Pugua and Hapatu, 7 Jul 1980, *Fosberg 59773* (K, US). **Kwajalein Atoll**: Kwajalein Islet, 19 Jan 1950, *Fosberg 31177* (L, US).

MAURITIUS. sin. loc, 1846, *Boivin s.n.* (W). **Rodrigues Islands**: Rodrigues, Aug 1874, *Balfour s.n.* (K); Rodrigues (Transit of Venus Expedition), 1874, *Balfour s.n.* (BM). **Vacoas**: at Vacoas, Mauritius, Mascarene island, 8 Sep 1973, *Lorence 179* (MO, P).

MICRONESIA. **Pohnpei**: Anapong-pa, 'Caroline Islands, Ponape', 5 Feb 1936, *Takamatsu 772* (K).

MOZAMBIQUE. estrada para Chinbenguanine, 31 Jun 1978, *Zunguze* & *Boane 113* (K, P). **Cabo Delgado**: Munava, 1887, *Taylor 100* (BM). **Lourenco Marques**: Costa de Sol, 3 Oct 1963, *Balsinhas 647* (B, K); Lourengo Marques, entre Macia e Ozamento para Zinavane, 28 May 1965, *Pereira et al. 533* (MO); Lourengo Marques, entre Manhica e Palmeira, 15 Jan 1965, *Rodrigues et al. 189* (MO). **Manica**: Chimanimani, Zomba, 4 Jul 2015, *Banze 277* (K). **Maputo**: nr Marracuene, valley of the Incomati river, 14 May 1982, *Jansen* & *Manhiça 8065* (K, MO); Namaacha, 13 Jun 1978, *de Koning 7088* (MO); Maputo, Manhica, Nacandzene, proximo duma Lagoa, 14 Aug 1980, *Nuvunga* & *Mafumo 256* (BM, K, MO); Marracuene, Bobole. Ilha de Chifecunze, 14 Nov 1979, *Schäfer 7053* (K, MO); Matutuíne, Catuane, Empresa Agro-Pecuaria de Catuane, Bloco de Manjene, 30 Sep 1983, *Zunguze et al. 597* (K, MO).

MYANMAR (BURMA). Sinlok, 13 Mar 1903, *Aubert & Gage s.n.* (CAL); Wall. Cat. Burm. 197, 8 Sep 1826, *Wallich 197* (G-DC); **Chin State**: Mindat Distr., Kanthar Yan village, nr Kanpetlet, Natma Taung National Park, 28 Feb 2007, *Fujikawa 50167* (BM); Mindat Distr., in the vicinity of Kanpetlet, Natma Taung National Park, 16 Jun 2007, *Man S55105* (BM); Mindat Distr., along the rdside between Maswati rd and Kanpetlet, Natma Taung National Park, 21 Jun 2013, *Man 92854* (BM); Mindat Distr., along the rdside between Makyauk Ar village and Kanpetlet, Natma Taung

National Park, 31 Dec 2012, *Man 96061* (BM); Mindat Distr., between Kanpetlet and Makyauk Ar Village, 5 Sep 2011, *Man et al. 85132* (BM); Mindat Distr., Oak Pho village, Kanpetlet Township, Natma Taung National Park, 11 Apr 2007, *Man et al. 54019* (BM). **Magway Region**: Gangaw Distr., Chaw Shit Chaung (river) between Kangyi and Saw, 27 May 2012, *Fujikawa 89358* (BM); Gangaw Distr., Saw town, along Saw river, 25 Aug 2013, *Fujikawa et al. 94443* (BM). **Mandalay Region**: Myingyan Distr., Popa village to Sandal Wood forest, along the jungle trail of Mt. Popa, 13 Aug 2000, *Aye & Htwe 20623* (BM). **Sagaing Region**: Upper Burma, Schwebo, Jun 1897, *Huk 57* (CAL).

NEPAL. sin. loc, 29 Aug 1975, *Dawson 434* (BM); Kathmandu, 13 Oct 1963, *Murata et al. 6303595* (K); sin. loc, *Wallich 2615[Y]* (K).

NEW CALEDONIA. Mont Dore, 2 Apr 1914, *Compton 659* (BM); Ouen Toro, 20 Apr 1914, *Compton 781* (BM); Canala, sentier Ciu-Coinde, 28 Aug 1969, *MacKee 20631* (K); Ile Hunter, 7 Dec 1977, *Veillon 3402* (L); Loyalty Islands, Lifu, *Whitmee s.n.* (BM). **Sud**: Nouméa, Noumea, 15 Jan 1956, *McKee 3794* (K); Ouen Toro, at southern end of Noumea, 12 Jun 1982, *McPherson 4594* (K, MO, P).

NEW ZEALAND. **Chatham Islands**: Rangatira Island, South East (Rangatira) Island Nature Reserve, 14 Feb 2006, *de Lange & Heenan CH-625* (AK). **Kermadec Islands**: Raoul Island, 1 Jun 1956, *Cooper s.n.* (AK); Macauley Island, 21 May 2011, *de Lange K-790* (AK); L'Esperance Rock, Southern Kermadec Islands Group, 26 May 2011, *de Lange K-863* (AK); Dayrell Island, Northern Kermadec Islands group, 18 May 2011, *de Lange K-1269* (AK); Raoul Island, 3 Jan 1967, *Sykes 514/K* (AK); South Chanter Islet, 26 Nov 1976, *Sykes 1102/K* (AK); North Meyer Island, 8 Feb 1978, *Sykes 1379/K* (AK); Raoul Island, 18 Feb 1966, *Sykes 175961* (K); Curtis Island, Kermadec Group, Nov 1989, *Taylor s.n.* (AK); Macauley Island, 22 Mar 2000, *Wilson s.n.* (AK). **North Island**: Auckland, Mt. Wellington, Jan 1938, *Allan s.n.* (CHR); Auckland, Awhitu, 23 Aug 2003, *Aspin & Wilcox s.n.* (AK); Northland, rd between Mokau Bay and Oakura Bay, 29 Nov 1972, *Astridge AEE-4106* (AK); Auckland, Mairangi Bay, 27 May 1974, *Bangerter 5165* (AK); Bay of Plenty, Kawerau, 5 Jul 1981, *Brown s.n.* (AK); Auckland, Muriwai, 11 Feb 1978, *Brownsey s.n.* (AK); Auckland, Arid Island, 31 Dec 1980, *Cameron A-49 a* (AK); Auckland, Rakitu (Arid) Island, Hauraki Gulf, 3 Jan 1981, *Cameron A-118* (AK); Auckland City, 25 Mar 1985, *Cameron 3398* (AK); Auckland, small island between Ponui and Rotoroa Islands, 10 Nov 1990, *Cameron 6217* (AK); Auckland, Great Barrier Island, 2 Apr 1992, *Cameron 6767* (AK); Northland, Oakura, 29 Sep 2006, *Cameron 12506* (AK); Waikato, N side of Waikawau Estuary, 8 Oct 2004, *Cameron 12543* (AK); Waikato, Motueka Island, 1 Mar 2005, *Cameron 12995* (AK); Northland, Woodhill Forest, 12 Dec 2005, *Cameron 13237* (AK); Auckland, Tirititi Matangi Island, 3 Dec 2006, *Cameron 14001* (AK); Northland, Whangaruru Harbour, Motutara (Henry Island), 26 Jan 2007, *Cameron 14394* (AK); Auckland, islet off Church and Fossil Bays, 20 Aug 2009, *Cameron 15205* (AK); Northland, northern Woodhill, 17 Aug 2013, *Cameron 16127* (AK); Auckland, Motuhoropapa, Inner Gulf Islands, Noises Islands, 22 Nov 2016, *Cameron 16879* (AK); Auckland, Great Barrier Island, 6 Jan 1983, *Cameron & Gardner 2110* (AK); Auckland, Laingholm, 10 Oct 1999, *Cameron & Hatch 9896* (AK); Auckland, Rangitoto Island, 2 Sep 2016, *Cameron & Ranatunga 26836* (AK); Northland, Woodhill State Forest, 1 Aug 1973, *Carter s.n.* (AK); Waikato, Whiritoa, Ohinemura Co., 15 Sep 1965, *Cooper s.n.* (AK); Waikato, Manaia, Coromandel Co., 18 Feb 1966, *Cooper s.n.* (AK); Waikato, Papa Aroha, 31 Jan 1965, *Cooper s.n.* (AK); Northland, Kelly Bay, 8 Sep 1968, *Cooper s.n.* (K); Northland, Waipu Gorge, Whangarei Co., 8 Jun 1966, *Cooper s.n.* (AK); Northland, Ngaiotonga saddle, Bay of Islands Co., 18 Apr 1965, *Cooper s.n.* (AK); Northland, Pouto, Hobson Co., 8 Sep 1968, *Cooper s.n.* (AK); Northland, Waikare, Bay of Islands Co., 17 Apr 1965, *Cooper s.n.* (AK); Northland, Kelly Bay, Hobson Co., 8 Sep 1968, *Cooper s.n.* (AK); Auckland, Great Barrier Island, 30 Nov 1964, *Cooper s.n.* (AK); Auckland, Mt. Auckland, Rodney Co., 6 Oct 1974, *Court s.n.* (AK); Waikato, Half Island, Aldermen Islands, 19 May 1972, *Court s.n.* (AK); Northland, Poor Knights Island, 13 Feb 1937, *Cranwell s.n.* (AK); Northland, Woodhill State Forest, 8 Aug 1973, *Dickson & Cameron s.n.* (AK); Wellington, Porirua Harbour, 6 Aug 2000, *Enright s.n.* (AK); Wellington, ca. 1 km NE of Judgeford, 28 May 2000, *Enright s.n.* (AK); Manaw, Keebles Bush, 21 Mar 1971, *Esler s.n.* (AK); Auckland, Mangere Basin, 17 Mar 2000, *Gardner 10165* (AK); Auckland, Waitakere Range, 16 Jul 2014, *Gardner 11230* (AK); Auckland, Great Barrier Island, 22 Aug 1972, *Hynes s.n.* (AK); Waikato, Red Mercury Island, 24 Aug 1971, *Hynes s.n.* (AK); Waikato, Aotea Harbour, 16 May 1963, *Hynes s.n.* (AK); Auckland, Herne Bay, 4 Jul 1983, *Kirkbride s.n.* (AK); Auckland, Waitakere

Ranges, Waitemata Co., 11 Jun 1978, *Mackinder s.n.* (AK); Auckland, Waitakere Ranges, 11 Jun 1978, *Mackinder s.n.* (AK); Auckland, Waihau Bay, 2 Oct 2012, *Miller M12/226* (AK); Auckland, Highway 1, 4 Jun 1972, *Orchard 3351* (AK, K); Auckland, hills above Mercer Bay, Waitemata Co., 2 Aug 1972, *Orchard 3412* (AK); Glen Innes Domain, Auckland metropolitan area, 21 Aug 1972, *Orchard 3435* (AK); Auckland, Dingle Dell Reserve, St. Heliers, Auckland metropolitan area, 1 Oct 1972, *Orchard 3454* (AK); Northland, ca. 1.5 km SW of Oakura Bay settlement, Whangarei Co., 29 Nov 1972, *Orchard 3680* (AK); Whatapuke Island, Hen and Chickens Islands, 7 Feb 1968, *Parris s.n.* (AK); Northland, Motuopao Island, 11 May 1994, *Parrish s.n.* (AK); Waikato, Castle Island, Coromandel Co., 8 Nov 1983, *Potter s.n.* (AK); Waikato, Kakepuku Historic Reserve, 16 Mar 1985, *Rattenbury s.n.* (AK); Auckland, Waiwera, Rodney Co., 26 Feb 1980, *Straka 9* (AK); Parnell, Cathedral Place, Auckland metropolitan area, 6 May 1975, *Turbott s.n.* (AK); Auckland, Mangere Sludge Ponds, 17 Mar 2000, *Wilcox s.n.* (AK); Northland, Aorangi (Tunnel) Island, Poor Knights, 19 Nov 1933, *Without collector s.n.* (AK); Waikato, Whitianga, 21 Aug 1974, *Wright 629* (AK); Bay of Plenty, Whale Island, 10 May 1976, *Wright 1204* (AK);Northland, Waipoua State Forest, Hokianga Co., 2 Sep 1976, *Wright 1405* (AK); Whakiripiha Island, Bay of Islands Co., 12 Jan 1980, *Wright 3438* (AK); Northland, Stephenson Island, Whangaroa Co., 23 Aug 1982, *Wright 4953* (AK); Auckland, Great Barrier Island, 1 Jan 1983, *Wright 5311* (AK); Waikato, Motuwi, 1 Sep 1983, *Wright 5828* (AK); Lizard Island, Mokohinau Islands, 6 Jan 1984, *Wright 6233* (AK); Northland, Tawhiti Rahi Island, 22 Mar 1984, *Wright 6343* (AK); Northland, Aorangi Island, 30 Aug 1984, *Wright 6518* (AK); Auckland, Mt. Wellington, 2 Jun 1985, *Wright 7285* (AK); Northland, Tawhiti Rahi Island, 23 Apr 1991, *Wright 11422* (AK). **Outlying Islands**: Three Kings Islands, Northeast Island, 8 Jul 1996, *Brook & McGlynn s.n.* (AK); Three Kings Islands, Great Island, 5 Mar 1989, *Wright 8842* (AK). **South Island**: Nelson, Buller, Gentle Annie, 17 Jan 2008, *Sykes 36-08* (AK).

NIGERIA. **Adamawa**: Gurumpawo, at foot of pass to R. Kirimi, 11 Nov 1957, *Hepper 1297* (B, K). **Gongola**: Gembu Distr., Mambilla plateau, 16 Aug 1977, *Odewo 592* (MO). **Lagos**: Lagos, 23 Oct 1917, *Dalziel 1188* (K). **Niue**. nr Hakapu, 6 Nov 1997, *Whistler 10810* (K); 6 mi W of Alofi, 13 Jan 1940, *Yuncker 9632* (K). **Norfolk Island**. Commune de Coconi, a la sortie de ville en direction de Dembéni, 26 Nov 2012, *Delnatte* & *Gallay 2930* (BM); Steels Point, 31 Oct 1978, *Telford 7215* (CANB); Collins Head rd, Norfolk Island. Pineapple plantation owned by Terry Jope, 14 Oct 1999, *Waterhouse 5519* (AD, CANB).

NORTHERN MARIANA ISLANDS. **Rota Island**: 3/4 mi E of Sabana, second terrace from top of island, 21 Jun 1946, *Fosberg 25149* (DUKE, US). **Saipan**: quarry site, W side of Naftan Point, 5 May 1982, *Herbst* & *Falanruw 6866* (US). **Tinian**: 500 m SE of Peipeinimaru, 25 Apr 1982, *Aguon 134* (US); Hagoi area, 30 Apr 1982, *Herbst* & *Falanruw 6824* (US).

PAKISTAN. **Punjab**: Punjab, Sadhai, 15 Feb 1885, *Drummond s.n.* (K).

PALAU. Koror, (Caroline Islands, Micronesia), 7 Aug 1978, *Otobed PW-10137* (US).

PAPUA NEW GUINEA. Musom village, upper Sankwet logging area, not far from Lae; this is a pot grown Waite in open from seed S-991 ex Symon 10665 originally collected in Papua New Guinea, 22 Dec 1977, *Symon s.n.* (K); Daru; around settlement, 29 Nov 1990, *Waterhouse 1273* (CANB). **East Papua**: Vitamen forest camp, between Bonenau and Mt Dayman, Milne Bay Distr., East Papua, 15 Aug 1969, *Pullen 8005* (GH, L). **Morobe**: S of Boana, Lae, Morobe Prov, 20 Feb 1977, *Conn et al. 88* (K); above Edie Creek, 15 Jul 1977, *Conn et al. 323* (A, K); Mt. Kaindi, 4 Oct 1977, *Conn* & *Kairo 490* (K); Lae, New Guinea, collected by Womersley, plant grown in glasshouse at Indooroopilly, Brisbane, 2 Jan 1973, *Henderson 1362* (CANB, K); Mt. Kaindi, Wau, 6 Oct 1978, *Kairo 60* (A, K); Mt. Kaindi, summit, SubDistr. Wau, 30 May 1977, *Kairo* & *Symon 10641* (K); Bulldog rd, Edie Creek, nr Wau, Morobe Distr. N.G, 13 Aug 1963, *Millar* & *Holttum NGF-15825[a]* (K); Lae market on sale; this is a pot grown Waite in open from seed S-987 ex Symon 10624 originally collected in Papua New Guinea, 22 Dec 1977, *Symon s.n.* (K); nr Musom village, upper Sankwet logging area, 16 Jun 1977, *Symon 10665* (MO); S summit, Mt. Kaindi, 27 Jan 1896, *van Valkenburg 362* (L). **Western**: Daru, Kunini Village, nr village, 8 May 1986, *Simaga 713* (K, L).

PHILIPPINES. Cabuting River, Samar, Feb 1916, *Edaño 24897* (GH). **Luzon**: Cagayan Valley, Mt. Iraya, Batan Island, Batanes Prov., 19 Mar 1991, *Barbon et al. PPI 1565* (K, L); Central Luzon, Mt. Mariveles, Lamao River, Bataan Prov., Sep 1904, *Borden 2070* (E, K); Cordillera (CAR), Bayninan, Banaue, Ifugao, Mountain Prov., 7 Mar 1963, *Conklin 79580* (A, K); Calabarzon, Los Baños, Laguna Prov., Apr 1905, *Elmer 8105* (E, K); Calabarzon, Mt. Maquiling, Los Baños, Laguna

Prov., Jun 1917, *Elmer 17677* (BM, F, GH, K, P, W); Cagayan Valley, Batanes, Mt. Iraya, Found along the trail to Mt. Iraya, Batan Island, 19 Mar 1991, *Madulid 1565* (MO); Mimaropa, Bongabob and Pinamalayan, Mindoro Island, 5 Feb 1941, *Maliwanag 132* (A); NCR, Manila, 29 Jul 1902, *Merrill 361* (GH, K)Mt. Makling, Tadlac, Los Banos, Laguna Prov., 20 Feb 1969, *Pancho* & *Orlido 14990* (MO); Calabarzon, Mt. Banahaw, Brgy. Kinabuhayan, Dolores, along the trail going to Kristalino and Suplina Falls, Quezon Prov., 27 Feb 1996, *Reynoso* & *Majaducon PPI-21644* (K); Mimaropa, Mt. Yagaw, E slope, Mindoro Island, Nov 1952, *Sulit* & *Conklin 16987* (K); Cordillera (CAR), Baguio, N Luzon, Benguet Prov., 4 Jun 1904, *Williams 1079* (GH, K). **Mindanao**: Caraga, Tungao, NALCO, Agusan Norte Prov., 14 May 1991, *Barbon*, *et al. PPI-1832* (K); SOCCSKSARGEN, So. Cotabato Prov., Mt. Matutum, 15 Apr 1992, *Gaerlan*, *et al. PPI-5373* (L); Davao, Gumate Distr., on the E slopes of the Mt. Apo complex, Davao Prov., Jan 1965, *Kellman ANU-1745* (L). **Visayas**: Eastern Visayas, Northern Leyte, Tongonan, Ormoc, found along the PNOC forest reservation, 22 Aug 1992, *Madulid* & *Fuentes 8801* (MO); Negros Island, Negros Oriental, Sibulan, Kabalinan Balinsasayao, Negros Island, 17 May 1991, *Madulid 910* (MO).

SAINT HELENA. Sin. loc, *Andersson s.n.* (S); Sin. loc, *Andersson s.n.* (S); St. Helena, *Burchell s.n.* (K); Millennium Forest, nr Longwood, 4 Apr 2015, *Cronk* & *Driver s.n.* (BM); Sin. loc., 12 Jul 1929, *Rendle 71* (BM).

SAMOA. Apia, 13 Feb 1880, *Betche 59* (MEL); *Whitmee 22* (MEL). **Fanuatapu**: top of the island by the signal light, 3 Jan 1981, *Whistler 4675* (K). **Nu'ulua**: islet of E end of Upolu, 13 Apr 1974, *Whistler 1929* (K). **Savai'i**: Above Salailua, 23 Sep 1931, *Christophersen 2759* (US); Olo, 5 Aug 1931, *Christerpherson* & *Hume 2254* (K); Ash plain on E side of the Mata-ole-Afi crater, 1 Jun 1975, *Whistler 2589* (K, US). **Savaii**: On the crater just southeast of Mt. Silisili, 17 Jun 1992, *Whistler 8858* (K). **Upolu**: beim Wasserfall, Papaloa, Jul 1905, *Rechinger* & *Rechinger 278* (W).

SEYCHELLES. Silhouette, Biede Martin, 1908, *Gardiner 108* (K); Mahe Is., Souvenir, 24 Nov 1961, *Jeffrey* & *Zelia 522* (EA, K, P); Sin. loc., 1867, *Neville s.n.* (K); Ause Royal Gool: Yussery Mahi, 15 Jul 1936, *Osborne-Day 55* (BM); Denis Island, 1 Dec 1977, *Stoddart 8103* (K); Marie Louise Island, 21 Nov 1970, *Wood 1445* (K); sin. loc, 1867, *Wright s.n.* (BM); **Aldabra**: Aldabra Atol, Aldabara, South Island, Aldabra island, Cinq Cases dunes, 18 Jan 1968, *Fosberg 48896* (MO); South Island, Cinq Cases, 31 Jan 1968, *Renvoize 885* (B, EA).

SIERRA LEONE. Katuna, 9 Jan 1928, *Deighton 1033* (K). **Southern**: Mano, 17 Nov 1951, *Deighton 5660* (B, K); Bo, 19 Jan 1914, *Thomas 4396* (K). **Western**: Freetown, 22 Dec 1926, *Deighton 474* (K); Freetown, Fourah Bay College, 12 Aug 1958, *Melville 4* (K); Freetown, Fourah Bay College, on Havelock Farm, 28 Feb 1964, *Morton SL-892* (K).

SOUTH AFRICA. **Eastern Cape**: Tunzini, Mar 1976, *Riches 25* (K). **Gauteng**: Pretoria, Manitoba Drive, Valley bottom S of junction of Morelettaspruit tributaries, 27 Nov 1971, *Saab 1-84* (EA). **KwaZulu-Natal**: Oribi Gorge on rd twoards Eland lake, 2 Apr 2009, *Mabatha* & *Nkuna 2542* (K); Menda, 1873, *Medley Wood 83* (K); Chief Mzimba Kraal, Muzi, 20 Oct 1970, *Moll 5119* (EA, K); Natal, nr Lake St. Lucia, 2832 AB, 25 Jun 1989, *Phillipson 3272* (MO); Durban Botanical Garden, 20 Sep 1962, *Strey 4311* (K); Berea, Durban Prov, Dec 1962, *Strey 4573* (K); Palm Beach, 3130AA Port Edward, 22 Feb 1974, *Strey 11303* (K, MO). **Limpopo**: c. 10 mi N of Marble Hall Groblersdal Prov, 25 Jul 1905, *van Hoepen s.n.* (K); along rd to Darru, Letaba Prov, 21 Apr 1958, *Scheepers 281* (K). **Mpumalanga**: Barberton, 27 Nov 1936, *Cotton Experimental Station 10* (K).

SPAIN. **Canary Islands**: Gran Canaria, Guia, Feb 1897, *Cook 809* (MO).

SRI LANKA. Sin. loc, Jan 1976, *Schwabe s.n.* (B); Sin. loc., *Walker s.n.* (E); Sin. loc., *Walker s.n.* (K). **Central**: Kandy, 3 Oct 1968, *Cooray 68-100301 R* (K, MO); nr Pussellawa mi 20 from Kandy, Kandy Distr., 20 Jun 1972, *Hepper 4412* (K, L); Lindula, Nuwara Eliya Distr., 21 Jun 1972, *Hepper 4455* (K, L); Talawakele to Kotmale, Nuwara Eliya Distr., 23 Jun 1972, *Hepper et al. 4478* (K, L); Matale Distr., 9 Jul 1972, *Hepper 4600* (K); University of Ceylon Peradeniya campus, Kandy Distr., 5 Jul 1975, *Jayasuriya* & *Pemadasa 2228* (K); Matale, Wewala tank area, Matale Distr., Central Prov., 29 Oct 1974, *Sumithraarachchi 512* (K, MO); **North Central**: Polonnaruwa Distr., c. 1 mi NE of Elabera along the Amban Ganga, 10 Oct 1974, *Davidse 7371* (K); Mailewewa nr Adampane (km 14), Trincomalee Distr., 1 Dec 1977, *Fosberg* & *Jayasinghe 57125* (K); **Southern**: Boossa, mile marker 68/1, Galle Distr., 21 Nov 1971, *Balakrishnan 996* (K); **Uva**: Ella, Bedulla Distr., Uva Prov., 21 Jul 1972, *Hepper* & *de Silva 4784* (K).

SWEDEN. **Götaland**: Västra Götaland, Göteborg, Mölndal: Krokslätt, 8 Sep 1958, *Blom s.n.* (BM); Västra Götaland, Göteborg, Mölndal: Krokslätt, 20 Sep 1950, *Blom s.n.* (BM).

TAIWAN. Tainan Hsien, Chiangchun Hsiang, Tingtan, 7 Apr 2003, *Chen Chih-Hsiung, 04625* (MO); Little Quemoy, 7 Jun 1961, *Chuang 4324* (A); Taipei, Sue Oyuan-ti, 25 Apr 1960, *Chuang* & *Kao 3896* (A); Chiayi Hsien, Alishan Hsiang, Alishan Recreation Area, along Alishan railway, Mienyuen Line, landslide area caused by 921 Earthquake, 11 Apr 2000, *Lin 147* (MO); Keitou, Nan Tow county, 7 Aug 1979, *Murata* & *Hishimura 39046* (MO). **Kaohsiung**: Da-Kun-Shan, temple grounds, 9 May 1998, *D'Arcy et al. 19269* (MO). **Pingtung**: Wanluan, Bankinsing, Feb 1914, *Faurie 637* (BM, P); Pingtung Hsien, Kenting National Park, O'luanpi Park, 16 Sep 1991, *Lammers 8490* (F, MO). **Taipei**: Chuwei, Tanshuei, 2 Feb 1983, *Ching-I Peng, 4506* (A); Taipei, Taiheku, Dec 1913, *Faurie 636* (BM, P); Shih-lin, 7 Jan 1961, *Huang 1953* (A). **Yilan**: Ilan Hsien, Chiaohsi Hsiang, Huotankeng, nr Dachaiohsi, 15 Nov 2000, *Lin 953* (MO).

TANZANIA. Kange Forest, 29 Nov 1955, *Faulkner 1780* (B, K). **Dar-Es-Salaam**: Kinondoni, N shore of Dar es Salaam Harbor; nr edge of low sea cliffs along Kenyatta Dr, 21 Dec 1984, *Gereau 1548* (MO). **Iringa**: Kilosa, Mikumi Game Lodge, 24 Oct 1970, *Batty 1100* (K, MO); Ludewa, Mlangali, Aug 1997, *54* (W). **Kagera**: Karagwe, 16 Aug 1967, *Archbold 937* (K); Hale Estate, Distr. Kiroge, 3 Oct 1964, *Faulkner 4036* (K). **Kilimanjaro**: Leranjwa, village area, 30 Jul 1993, *Grimshaw 93-455* (K). **Mbeya**: Mbeya, Old cultivation at base of Pungaluma Hills, 12 Dec 1989, *Lovett et al. 3696* (MO). **Morogoro**: below Bondwa Ridge, Uluguru Mountains (T6), 22 Jan 1976, *Cribb* & *Grey-Wilson 10345* (K). **Njombe**: Msima Stock Farm, Iringa region, 1932, *Emson 296* (EA, K). **Tanga**: Lushoto, Mazumbai Forest Reserve, West Usambara Mountains (T3), 16 Jan 1976, *Cribb* & *Grey-Wilson 10246* (K); Paugeve-Maweni, Tonga Distr., 29 Nov 1967, *Faulkner 4065* (K, MO); Muheza, East Usambara, Amani, in vicinity of Bombole village, 10 Dec 1995, *Vainio-Mattila et al. 95-9* (H, MA); Korogwe, West Usambara Mountains, Tanzania, Tanga Prov., Korogwe Distr., Kunga Tea Estates, 22 Mar 2010, *Vorontsova et al. 162* (BM, BM, DSM, K, NY, UT). **Uzaramo**: Dar es Salaam, 14 Oct 1967, *Harris 1078* (EA, K, MO). **Zanzibar South and Central**: Zanzibar Central, Zanzibar, Massazine, 30 Oct 1962, *Faulkner 3120* (B, EA, K); Zanzibar Central, Zanzibar, Poole's shamba, 14 Feb 1930, *Vaughan 1205* (EA, K).

THAILAND. **Chiang Mai**: Muang, Doi Sutep, 2 Jan 1915, *Kerr 3500* (K); Fang, Chaibragan, Sidongyen subDistr., Ban Wieng Pa Pattana (Lahu village), 24 May 1991, *Maxwell 91-474* (E, GH, P); Doi Sutep, 2 Jul 1988, *Maxwell 88-845* (L); Wat Chiang Dao, 24 Sep 1971, *Murata et al. T-14870* (MO). **Kanchanaburi**: Kanchanaburi, 4 Mar 1973, *Phengklai 3085* (K). **Khao Luang**: Nakhon Si Thammarat prov., Khao Luang, along trail down to Khiri Wong, 26 May 1968, *van Beusekom* & *Phengkhlai 1030* (K). **Mae Hong Son**: along the rd between Mae Sariang and Mae Hue, 18 Sep 1967, *Iwatsuki* & *Fukuoka 10367* (K, L). **Prachuap Khiri Khan**: Kui Buri, Kui Buri National Park, 24 Jan 2004, *Middleton et al. 2476* (E). **South**: Nakhom Si Thammarat, Nakhom Si Thammarat prov., Khao Long, trail down to Khiri Long, 26 May 1968, *van Beusekom* & *Phengkhlai 1030* (L); **Trang**: Khao Pap Pa, 7 Sep 1974, *Larsen* & *Larsen 34265* (K).

TOGO. **Plateaux**: Mt. Agou, 17 Sep 1974, *Mathey* & *Scholz 164* (B).

TONGA. Vavau, Volo, Feb 1892, *Crosby 117* (MEL). **Eua**: Hango College, 18 Jun 1977, *Sykes 87 -T* (US). **Eueikei**: (Tonga group), 25 Jul 1959, *Soakai 941* (K). **Tongatapu**: Tuamotu, 2 Oct 1958, *Lawrence 138* (K); Nuku'alofa, in the area, 1 Jun 1989, *Whistler 6746* (K); Tongatapu, below the village of Haakame, on southwestern side of the island, 12 Mar 1953, *Yuncker 15272* (BM, US).

UGANDA. **Central**: Naminya N of Njeru town council, East Mengo, Mukono Distr., Kyagwe county, 22 Aug 2000, *Olet 1* (K); Kyadondo, Kyadondo county, W Mengo Distr., Luzura, 6 Mar 1984, *Rwaburindore 1705* (MO); E Mengo U4, Kyagwe County, 3 km E of Namugongo, 3 Dec 1995, *Rwaburindore 3936* (MO); Naguru Valley, Kyadondo County, Kampala U4 Distr., 9 Aug 1997, *Rwaburindore 4199* (MO). **Northern**: 3 km S of Lira town, Lango, Lira Distr., Erute county, Lira Central, Ereda, 15 Jan 2001, *Olet 66* (K).

UNITED KINGDOM. **Ascension Island**: Ascension Island, 1958, *Duffey 200* (K); Garden Cottage, 30 Sep 2008, *Lambdon A036* (K); Green Mountain, Elliots trail, 15 Jan 2015, *Sim JS10* (K). **England**: Hertfordshire, appeared in garden, Ware, Herts, Aug 1978, *Hanson 255* (BM); West Yorkshire, Kirkheaton, SW York, Wool adventive flora, 28 Sep 1958, *Lousley 605* (K).

UNITED STATES OF AMERICA. **Hawaii**: Oahu, Pohakea Pass, Waianae Mt, 3 Jun 1939, *Baxter s.n.* (MO); Oahu, Koko Head, Honolulu Distr., 18 Feb 1984, *Bruegmann 143* (US); NW Hawaiian

Islands, Kure Atoll, 3 Oct 1959, *Clay s.n.* (US); North Kona Distr., Hawaii Co., *Close & Popolizio 1094-22* (US); Oahu, Waineae Range, 9 Jul 1964, *Crosby & Anderson 1717* (DUKE); park along Hilo Bay, 29 Jan 1976, *Degener 33660* (B, W); Waha'ula Heiau, Hawaii Volcanos National Park, 23 Jan 1976, *Degener & Degener 33618* (AK); Punaluu, Kau, 7 Feb 1976, *Degener & Degener 34134* (B); Pahala, Kau, 27 Mar 1980, *Degener & Degener 35908* (US); Pahala, Kau, Jan 1984, *Degener & Degener 36120* (AK, US); Halawa, Jun 1909, *Faurie 863* (BM); Oahu, Kali he, Oct 1909, *Faurie 880* (BM); Maui, Haleakala, 25 Mar 1967, *Fosberg 48351* (MO, US); Oahu, Ridge N of Kuapa Pond, 7 Aug 1927, *Gillespie 1110* (MO); Oahu, Kalena, Waianae, 30 Sep 1934, *Grant 7433* (CANB); Oahu, Kalena, Waianae, 30 Sep 1934, *Grant 7439* (CANB); Oahu, Waipio, 27 Nov 1967, *Herbet 745* (L); Oahu, Popoi'a Island, Kailua Bay, 7 Feb 1978, *Herbst 6010* (US); Oahu, Honolulu, 15 Jun 1916, *Hitchcock 13679* (US); NW Hawaiian Islands, Midway-Sand Island, 29 Jun 1980, *Herbst & Takeuchi 6350* (US); NW Hawaiian Islands, Lisianski Island, 11 Jul 1980, *Herbst & Takeuchi 6499* (US); NW Hawaiian Islands, Cable Co. compound, 15 Dec 1962, *Lamoureux, C., 2225* (US); NW Hawaiian Islands, north of Barrier Hanger, 17 Dec 1962, *Lamoureux 2298* (US); Oahu, Kaohikaipu Islet, Makapuu Point, 10 Aug 1967, *Lamoureux 4075* (CANB); Oahu, Mokuauia, 16 May 1964, *Long 1720* (US); NW Hawaiian Islands, Kure Atoll, 14 Sep 1964, *Long 2238* (US); NW Hawaiian Islands, Lisianski Island, 18 Sep 1964, *Long 2318* (US); NW Hawaiian Islands, Laysan Island, 19 Sep 1964, *Long 2383* (US); Kauai, Kokee State Park, 28 Jan 1988, *Lorence et al. 5751* (MO, US); Oahu, 1889, *Lyons s.n.* (DUKE); Oahu, Palolo Valley, Honolulu, Jan 1927, *MacDaniels 174* (BM); Oahu, 42 Coehlo Way, Honolulu, Jul 1941, *Neal s.n.* (BM); Oahu, Moku Manu, 18 Jun 1937, *Neal s.n.* (MO); Molokai, Kamiloloa, 13 Mar 1992, *Norman UNK-9* (US); Oahu, Honolulu, *Pietschmann s.n.* (W); Nihoa, Nonopapa, 13 Aug 1947, *St. John 22763* (US); Kauai, 15 Mar 1983, *Symon s.n.* (MO); Kauai, Kaewanui Bay, 30 Jul 1987, *Wagner & Lorence 5708* (L, US); Oahu, Maricopa, 10 Mar 1983, *Woodruff H-1990* (DNA); NW Hawaiian Islands, Laysan Island, 17 Sep 1964, *Young 152* (US); Oahu, Waianae, 3 May 1991, *Zoller 1991.184* (US).

VANUATU. New Hebrides & Banks Islands, *Commis 283* (K). **Erromango**: in vicinity of Nouankao camp, 3 Aug 1971, *Green 1265* (K); Cook's Bay, 22 Jul 1896, *Morrison s.n.* (K). **Espiritu Santo**: Hog Harbour, New Hebrides, 20 Nov 1933, *Baker 50* (BM); Valée de l'Apouna, 1 Sep 1971, *MacKee 24143* (K); Nokowula, 5 Sep 1971, *MacKee 24217* (K). **Malakula**: Matanoui, bord de la riv. Matanoui, 10 Oct 1971, *Hallé 6400* (K).

VIETNAM. Hà Son Binh, Ha Son Binh, Luong Sin, Lam Sin, 31 May 1983, *Le Tram Chan, C-96* (K). **Kon Tum**: Mang La forest enterprise, Kon Plong Distr., Hieu Municipality, 18 Apr 2000, *Vietnam Highland et al. VH-5351* (MO). **Ninh Binh**: Cuc Phuong National park, site CP100, 21 Sep 1999, *Cuong 551* (MO). **Ninh Thuan**: Nui Chua National Park. Ninh Hai Distr., Vinh Hai Municipality, Vill. Da Hang, 28 Mar 2004, *Tap et al. HLF3387* (MO).

ZAMBIA. **Western**: Ndola, 12 Apr 1954, *Fanshawe 1085* (K).

Solanum chenopodioides

AUSTRALIA. Swamp, c. 2 km N of Bodalla on Princes H[igh]w[a]y, just S of Tuross River crossing, 10 Feb 1979, *Haegi 1756* (AD, NSW); Hillview, 11 Dec 1968, *Rodd s.n.* (NSW). **New South Wales**: N shore Lake Illawarra, Lake Hts, 5 Sep 2003, *Andrew s.n.* (WOLL); Blue Mountains, Around Wentworth Falls, 26 Apr 1995, *Bates 41122* (AD); Walcha, Macdonald River, Woolbrook, ca. 25 km W of Walcha, 29 Dec 2009, *Bean 29340* (NSW); Warrumbungle Shire, Wallumburrawang Creek, SE of Warrumbungle National Park, c. 25 km due WSW of Coonabarabran, 23 Mar 2009, *Benson & Waller 2791* (NSW); Audley, Royal National Park, 3 mls (4.8 km) S of Sutherland, 21 Mar 1972, *Bisby & Coveny 4036* (AD, K); Batehaven, c. 5 km SE Batemans Bay, 25 Jan 1977, *Blaylock 2357* (AD); Lithgow, c. 6 km E of Glen Davis at junction of Running Stream C[ree]k and Capertee R[iver], 2 Dec 1978, *Briggs 6964* (AD, CANB, NSW); Eurobodalla, Slopes of Mount Dromedary, Central Tilba (c. SW of Narooma), 12 Sep 1953, *Constable s.n.* (AD); Campbelltown, Minto, 23 Mar 1961, *Constable s.n.* (AD); Canterbury, Cook's River, Mascot Bridge, c. 5 mls S of Sydney, 8 Jun 1965, *Constable 5966* (AD, NSW); Nepean River, Douglas Park, 6 mls E of Picton, 11 Oct 1965, *Constable 6161* (AD, K, NSW); Wollondilly, Nepean River, Douglas Park, 6 mls E of Picton, 11 Oct 1965, *Constable 6164* (AD, NSW); Eurobodalla, Head of Moruya River, Dampier State Forest, 15

mls W of Bodalla, 11 May 1966, *Constable 6897* (AD, NSW); Wingecarribee, Netherby, Mossvale, 2 Jan 1982, *Cosh s.n.* (WOLL); Wingecarribee, Moss Vale, 2 Jan 1982, *Cosh s.n.* (WOLL); junction of Jock's Creek and Wollondilly River nr Goodman's Ford, 39 km WNW of Mittagong, 16 Mar 1975, *Coveny et al. 6088* (K); Wingecarribee, Junction of Jock's Creek and Wollondilly River nr Goodman's Ford, WNW of Mittagong, 16 Mar 1975, *Coveny et al. 6088* (AD, NSW); Lithgow, Farmers Creek, Bowenfels, 24 Mar 1975, *Coveny et al. 6159* (AD, NSW); Singleton, Bulga Service Station on the Windsor - Singleton Road, 11 Jun 1975, *Coveny & Powell 6535* (AD, NSW); Rotherwood Rd off Mt Hercules Road, Razorback Range, 10 km SSW of Camden, 17 Mar 1976, *Coveny et al. 7466* (K); Bellbird Hill Reserve Rotary Lookout, Kurrajong Heights, 28 Mar 1984, *Coveny & Miller 11827* (K, MO); Hawkesbury, Bellbird Hill Reserve Rotary Lookout, Kurrajong Heights, 28 Mar 1984, *Coveny & Miller 11827* (AD, NSW); Hornsby, Pennant Hills, 1934, *Fraser s.n.* (NSW); Colo River crossing on Putty Road, c. 20 km N of Windsor, 14 Jan 1979, *Haegi 1602* (AD, MO, NSW); Bega Valley, Twofold Bay, Nullica Bay Beach, c. 5km SSW of Eden, 8 Feb 1979, *Haegi 1711* (AD, MEL, MO, NSW); Bega Valley, Overflow area of Bega River, under bridge on Princes H[igh]w[a]y, at Bega North, 9 Feb 1979, *Haegi 1730* (AD, NSW); Bega Valley, Tathra, 20 Mar 1995, *Heyligers 95004* (PERTH); Bega Valley, Tathra, 20 Mar 1995, *Heyligers 95006* (PERTH); Gunnedah, nr E bank of Mooki River, S of Breeza to Currabubulah rd, 11 Nov 2001, *Hosking 2117* (AD, CANB, MEL, NE, NSW); Tamworth Regional, lower Sheba Dam, Hanging Rock, 13 Apr 2008, *Hosking 3091* (CANB, MEL, NE, NSW); Tamworth Regional, Gully on ridge S of Teamsters Rest Campsite, ca 13.5 km SW of Nundle, 20 Mar 2011, *Hosking 3496* (CANB, NE); Blue Mountains, Mt. Tomah, 30 Jan 1966, *Ingram s.n.* (AD); Murrumbidgee River, c. 2.5 km SE Tharwa, 18 Jun 1989, *Lepschi 137* (AD, CANB); Murrumbidgee River at Cotter Bridge, Cotter Reserve, 1 Mar 1992, *Lepschi 740* (AD, CANB, NSW); Palerang, Warri Bridge Reserve, Shoalhaven River, c. 12.5km NNW of Braidwood, 28 Feb 1993, *Lepschi 930* (CANB, HO); Eurobodalla, Alongside lookout platform, Durras Beach, South Durras, 16 Jan 2011, *Liney 2765* (NSW); Southgate, 9 mls, NE of Grafton, 2 Apr 1963, *MacDonald 164* (K); Southgate, 9 mls, NE of Grafton, 2 Apr 1963, *MacDonald 174* (K); Sutherland Shire, Railway land, Ingleburn, 10 Feb 1968, *McBarron 14881* (AD, NSW); Snowy River, Kosciuszko National Park, 790 m N of the NSW/Vic border, 6 Dec 2013, mls *s.n.* (CANB); Wollongong, Mount Ousley, Wollongong, 25 Jan 1983, *Mills s.n.* (WOLL); Eurobodalla, Rosedale. Beachfront area, 28 Oct 1979, *Pearce 154* (AD); Bega Valley, Bermagui South, Dickinson Caravan Park, 30 Oct 1979, *Pearce 155* (AD); Bellingen, Dorrego, 20 Feb 1989, *Pedley 5403* (AD); Penrith, Orchard Hills Defence Area - site 5, 19 Aug 2002, *Pellow & Clarke s.n.* (WOLL); Liverpool, Holsworthy Army Base, 15 Apr 2002, *Pellow s.n.* (WOLL); Armidale Dumaresq, Paddock fence-line at Lagoon Creek, 67km W of Kempsey, along Armidale Kemspey Road, 6 Sep 2010, *Perkins & Renner s.n.* (NSW); Shoalhaven, Shallow Crossing, S Coast, 8 Mar 1961, *Phillips s.n.* (AD); Currowan State Forest nr Nelligen Coast, 6 Dec 1963, *Pullen 3964* (CANB, MO, NSW); Palerang, Euradux Road, off Nerriga Road, N of Braidwood, 25 Jan 2009, *Purdie 7081* (CANB); Cooma-Monaro, Scottsdale Bush Heritage property, c. 4 km N of Bredbo, 28 Nov 2011, *Purdie 8316* (CANB); Camden, Woodland Conservation Area, Annan Creek side, 25 Nov 2010, *von Richter 436* (NSW); Lake Macquarie, Wyee, 2 km (direct) NW from Wyee Railway Station, N boundary of Bethshan Ministries Conference Centre, 25 Jan 2007, *Rockley 4* (NSW); Hillview, 3 mls SW of Liverpool, 11 Dec 1968, *Rodd 121632* (K); Howra (Shoalhaven), 10 Jan 1935, *Rodway 1661* (K, NSW); Broulee Head between Bateman Bay & Boruya, S coast, 19 Dec 1948, *Rodway 14904* (K, NSW); Port Macquarie-Hastings, Rocky Creek, SE of Dorrigo, 2 Jan 1960, *Salasoo 1815* (NSW); Wollongong, Lake Illawarra, 5 Sep 2003, *Schofield s.n.* (WOLL); South Coast, Raifhaven, Old Tilba Road, 0.2km S of Wallage Lake Road, on beach side of rd, 17 Mar 1995, *Schultz 166* (CANB, MO); NSW Dorrigo Plateau, just below the Dorrigo National Park, on the roadside to the coast, 10 Jan 1979, *Symon 11515* (AD, MO); Bellingen, nr Nash's Road, c. 15 mls west of Dorrigo, 5 Apr 1970, *Williams s.n.* (NE); c. 4 km NE of Williamtown on Port Stephens Road. Beside rd c. 0.5 km SW of Raymond Terrace turn-off, *Wilson 2410* (AD); Blue Mountains, Mt. Tomah, 28 Apr 1963, *Without Collector 303* (AD). **Queensland**: Southern Downs, 27 Nov 2001, *Batianoff 11110* (BRI); Southern Downs, 27 Nov 2001, *Batianoff 2001-11125* (BRI); Southern Downs, 27 Nov 2001, *Batianoff 2001-11141* (BRI); Southern Downs, 29 Nov 2001, *Batianoff 2001-11342* (BRI); Southern Downs, 29 Nov 2001, *Batianoff 2001-11356* (BRI); Southern Downs, 7 Apr 2002, *Bean 18613* (BRI); Toowoomba, 16 Dec 2011, *Bean 31405* (BRI); Southern Downs, 31 Dec 2015, *Bean 32579* (BRI); Southern

Downs, 3 Jan 2016, *Bean 32684* (BRI); Toowoomba, 11 Apr 2011, *Forster 37983* (BRI); Southern Downs, 18 Mar 2015, *Forster 42109* (BRI); Brisbane, 20 Jan 1966, *Henderson 128* (BRI, NSW); Brisbane, Brisbane, Clapham Junction, 20 Oct 1967, *Henderson 301* (AD, BRI); Toowoomba, 5 Dec 1977, *McKenzie s.n.* (BRI); Toowoomba, 21 Nov 1994, *Swarbrick 10855* (BRI). **South Australia**: Tea Tree Gully, Gorge Rd, 15 Jan 2010, *Brodie 1171* (AD); Adelaide Hills, Off Gorge Rd. On 6th Creek bank, 15 Jan 2010, *Brodie & Symon 1183* (AD); Adelaide Hills, Adelaide Hills, Corkscrew Rd Sixth Creek, c. 2 km from Gorge Rd past first property, 11 Apr 2014, *Brodie 5435* (AD); Adelaide Hills, Adelaide Hills. Corkscrew Rd. Sixth Creek, c. 2 km from Gorge Rd past first property, *Brodie 5435* (MEL); sin. loc, 1885, *Lea s.n.* (BM); Region 11: Southern Lofty, Lower Gorge Rd, 13 Feb 1997, *Symon 15462* (AD, K, MEL). **Victoria**: Port Phillip, Carpark, E side of Kavanagh Street, just N of Power Street, South Melbourne, 23 Nov 1982, *Clarke 1479* (AD, MEL); Melbourne, Wasteland beside Yarra River NW side of Yarra Bank Road, c. 70 m SW of Queens Bridge, South Melbourne, 18 Feb 1983, *Clarke 1544* (AD, CANB, MEL); East Gippsland, N of Mallacoota township, by dirt rd SW off intersection of Narbethong Avenue and Lakeside Drive, 4 Apr 1999, *Clarke 2890* (AD, MEL); Kingston, Melbourne south-east suburb of Heatherton, E side of Warrigal Road, N of Fairchild Street, Karkarook Park, 25 Mar 2013, *Clarke 4181* (MEL); Wellington, beside Wellington River about 13 km from Licola, 28 Jan 1989, *Thompson 159* (AD).

FRANCE. **Nouvelle Aquitaine**: Gironde, Bordeaux, 28 Aug 1931, *Bouchon 6703* (BM). **Provence-Alpes-Côte d'Azur**: Bouches-du-Rhône, La Valentine, banlieue E de Marseille, route vers Saint Menet, 19 Nov 1973, *Martin 6848* (BM, H).

GERMANY. **Baden-Württemberg**: Karlsruhe, Carlsruhe, Oct 1834, *Braun s.n.* (K); Karlsruhe [?], Dec 1834, *Braun s.n.* (K). **Greece**. **Crete**: sin. loc, *de Tournefort s.n.* (BM). **East Macedonia and Thrace**: Island of Thasos, SE of Potamia, 23 Nov 2016, *Biel IM-16054* (BM);

ITALY. **Friuli Venezia Giulia**: Monfalcone, Friulia, 25 Sep 1953, *Neumann s.n.* (W).

JAPAN. **Honshu**: Tochigi, Utsunomiya Agricultural College, Japan, 1935, *Kagawa s.n.* (K); Kyoto, Koyama, Chitose, Chitose-cho, Kameoka-shi, 12 Sep 2010, *Tsugaru et al. 7604* (MO).

LESOTHO. Maseru airfield escarpment, Lesotho, 10 Dec 1969, *Without Collector 348* (K); Maseru Ekp Stn, 3 Mar 1970, *Without Collector 650* (K).

NEW ZEALAND. University of Otago, 17 Oct 1967, *D'Arcy s.n.* (MO); **North Island**: Northland, inland from Te Arai, Aupouri Ecological Region and Distr., 24 Jul 1993, *Bellingham 637* (AK); Auckland, Waima, 1 Rimatuka Place, Auckland Ecological Region, Waitakere Ecological Distr., 25 Feb 1999, *McKain s.n.* (AK); Auckland, Tawharanui Regional Park, Auckland Region, Rodney Distr., 14 Jan 2012, *Salter & Duff s.n.* (AK); Northland, Te Arai Sanctuary, Aupouri Ecological Region and Distr., 27 Jan 1995, *Wright 12532* (AK). **South Island**: Tasman, Collingwood, 1 km W on Cape Farewell Road, 13 Mar 2006, *Brummitt 21567* (K); Canterbury, Canterbury, Upper Rakaia Gorge, 23 Jan 1964, *Hynes s.n.* (AK).

PORTUGAL. **Azores**: Faial, Horta, 27 Sep 1970, *Brooke 11376* (BM); Faial, Capelo, 1 Dec 1971, *Goncalves 374* (BM); Pico, São Roque, Cais, 25 Aug 1972, *Goncalves 4698* (BM); Faial between Pedro Miguel and Espalhafatos, 15 Sep 2001, *Henderson et al. 98* (AZU, BM). **Centro**: Vila Nova de Barquinha, Ribatejo, 16 Jul 1963, *Rainha 6177* (W). **Madeira**: Between Monte and Funchal, 11 Sep 1984, *Davis 70402* (BM). **Norte**: Amarante, Marcos de Canavezes, Douro Litoral, 6 Jul 1960, *Pinto da Silva et al. 6754* (W).

SOUTH AFRICA. Eastern Cape: Alexandria, Kaba rd, Kaba Rd, Distr. Alexandria, 29 Jun 1953, *Archibald 5905* (K); Old Town Quarry, Grahamstown, 20 Nov 1972, *Bayliss 5292* (A, BH, MO); Belmont Valley, Albany Dist, 1 Sep 1974, *Bayliss 6785* (K, MO); Wes van Springbok Park, 23 Feb 1966, *Hanekom 564* (K); Humansdorp, Distr. Humansdorp, Tzitzikama Park, 2 Feb 1966, *Liebenberg 7909* (K); Grahamstown, 17 Mar 1971, *Shaw 112* (MO); Humandorp Distr., 17 Apr 1999, *Youthed 790* (MO). **Gauteng**: Germiston Distr., Dowerglen, 22 Oct 1992, *Balkwill 7163* (MO); Beryl Street, Johannesburgh Distr., Cyrildene, 20 Nov 1995, *Hull 68* (MO); Pretoria, Johannesburgh, Taamlik volop, 2627BB grid ref, Oct 1976, *Liebenberg 8568* (K, MO); Witswatersrand, Alberton, Witwaterssand Distr., Transvaal, *Moss 13307* (K). **KwaZulu-Natal**: Pretoria, Greytown, Natal, 11 Feb 1939, *Galpin 14832* (K); Underberg, 2929CB Sani Pass, Undeberg Distr., 17 Feb 1982, *Hilliard & Burtt 15537* (K)Nottingham rd, Natal, Mar 1939, *McClean 869* (K, MO); Greytown, Natal, Oct 1931, *Wylie 3420* (K). **Mpumalanga**: Middelburgh Distr., just outside Middelburgh, 26 Jan 1995, *Balkwill 9130* (MO). **Western Cape**: 3323 Willowmore DC, 28 Dec 1982, *Goldblatt 6780* (MO).

Spain. **Cantabria**: Santander, rd to Pechon, 31 Jul 1972, *Brenan 12271* (BM, K).
Sweden. **Götaland**: Västra Götaland, Göteborg, Backa prope Brunnsbo, Aug 1938, *Blom s.n.* (BM); Västra Götaland, Agnesbergs kvarn, 30 Sep 1938, *Blom s.n.* (K, W).
Switzerland. **Ticino**: Locarno, bank of Maggia river, 17 Sep 2000, *Brummit 20476* (K).
United Kingdom. Channel Isles: Guernsey, nr entrance to Mont Cuet, Nov 2001, *Dupree s.n.* (BM); Guernsey, 6 Sep 1994, *McClintock* & *Ryan s.n.* (BM); Guernsey, St. Sampsons, 23 Jul 1968, *Simpson 68010* (BM). **England**: Greater London, Victoria Park, 4 Sep 2008, *Atchison 2* (BM); Kent Platt, Garden at Bracken Hill, 1 Aug 1986, *McClintock s.n.* (BM); Greater London, Southwark, 8 Oct 1989, *Palmer s.n.* (BM); Worcestershire, Charlton, 25 Aug 1959, *Pannister 983* (BM); Cornwall, Bude, Aug 1925, *Thurston s.n.* (K); Greater London, Southwark, London, 2 Oct 1990, *Wurzell s.n.* (K); Greater London, Bermondsey, 1 Jan 1982, *Wurzell s.n.* (K). **Wales**: Vale of Glamorgan, Barry Docks, 12 Sep 1935, *Brenan* & *Sandwith 1449* (BM); Cardiff, Barry Docks, 12 Sep 1935, *Sandwith* & *Brenan s.n.* (K).

Solanum furcatum

Australia. **Tasmania**: Circular Head, B22 between Irishtown and Edith Creek, 2 Nov 2004, *Baker 981* (HO); Circular Head, Grooms Cross rd (B22), between Irishtown and Edith Creek, 2 Nov 2004, *Baker 1010* (HO); Circular Head, Edith Creek, 2 Nov 2004, *Baker 1019* (HO); Circular Head, Copper Creek nr Smithton, May 1947, *Curtis s.n.* (HO); Circular Head, Copper Creek, May 1948, *Without Collector s.n.* (HO). **Victoria**: Greater Geelong, Edwards Point State Faunal Reserve, 19 May 1984, *Albrecht 483* (MEL); Along Billy Creek in proposed extension to Morwell National Park, 15 Jul 1988, *Harris 4* (K); South Gippsland, Boolarra to Mirboo North rd, 22 Nov 1987, *Harris s.n.* (MEL); East Gippsland, Gauging Station on the Genoa River nr the Wangarabell rd, 10 Mar 2011, *Jeanes 2662* (MEL); McCrae, between Dromana & Rosebud, 24 Feb 1963, *Willis s.n.* (MEL); Yarra Ranges, Tremont, 30 Jul 2006, *Stajsic 4292* (HO, MEL); Greater Geelong, St Leonards, Bellarine Peninsula, 9 Nov 1947, *Willis s.n.* (MEL); Greater Geelong, St Leonards, Bellarine Peninsula, 9 Nov 1947, *Willis s.n.* (MEL); Mornington Peninsula, Main Creek, 24 Mar 1984, *Willis s.n.* (MEL); McCrae, between Dromana & Rosebud, 24 Feb 1963, *Willis s.n.* (MEL).
New Zealand. **North Island**: Manawatu-Wanganui, Vinegar Hill, Rangitikei R[iver], near Rowa, 18 Nov 1964, *Healy 64/425* (AK).

Solanum hirtulum

Ethiopia. Abyssinia, *Schimper s.n.* (P); sin. loc, 26 Oct 1862, *Schimper 631* (BM, E, K, W). **Addis Ababa**: Addis Ababa, 30 Sep 1937, *Piovano 511* (FT); Addis Ababa, 19 Oct 1937, *Senni 1837* (FT). **Amhara**: Semien, Debarek Semien Gonder Region, 11 Jul 1909, *Chiovenda 889* (FT); 10 km SE of Debre Markos along rd to Addis Ababa, between Debre Markos and Addis Ababa, 25 Oct 2004, *Friis et al. 11912* (K); Bichena Awraja, c. 36 km NW of Debre Work (Gojjam region), 30 Oct 1981, *Mesfin Tadese* & *Kagnew 1659* (K); in campis Debra Eski, 19 Oct 1850, *Schimper 74* (P); Semien, Derasghie, 25 Dec 1952, *Scott 292* (K); Dejen to Debra Marcos, 48 km NW of Dejen, 24 May 1980, *Thulin 3914* (K, MO); mule track between Debarak and Geech, 16 Sep 1969, *de Wilde* & *Gilbert 5* (EA, MO). **Oromia**: Addis Alem, 20 Sep 1926, *Omer Cooper s.n.* (K); Semien Shewa region, Holetta, 4 May 1953, *Mooney 4752* (K). **Tigray**: Adua, Prope Adoam, Abyssinia, 1852, *Schimper s.n.* (P).

Solanum memphiticum

Burundi. **Ruyigi**: Musongati, 10 May 1974, *Reekmans 3419* (EA, MO).
Democratic Republic of the Congo. **Nord-Kivu**: Ruindi, Nov 1937, *Lebrun 8380* (K).
Egypt. **South Sinai**: Wadi El Sheikh, Sinai, 15 Apr 1937, *Shabetai s.n.* (K).
Eritrea. Golo, 5 Dec 1905, *Dainelli* & *Marinelli 91* (FT); Golo, 6 Dec 1905, *Dainelli* & *Marinelli 279* (FT). **Anseba**: Geleb, Gheleb - Caropebir, 16 Jan 1893, *Terracciano* & *Pappi 2027*

(FT). **Debub**: Adi Ugri - Mai Tacala, 4 Sep 1909, *Bellini 335* (FT); Embataca, 6 Dec 1905, *Dainelli* & *Marinelli 89* (FT); Saganeiti, Ocule Cusai, 7 Mar 1909, *Fiori 1594* (FT); Saganeiti, gorge Gona pres Addingofon, 29 Mar 1892, *Schweinfurth* & *Riva 1319* (FT, K). **Gash Barka**: Badum, lungo fiume Mareb, 10 Jan 1906, *Pappi 6893* (FT); Get Arba, Gret - Arba, 7 Jan 1893, *Terracciano* & *Pappi 1706* (FT). **Maekel**: Asmara, 12 Sep 1912, *Baldrati 3536* (FT); Asmara, 2 Aug 1902, *Pappi 2099* (EA, MO, P, SI). **Semienawi Keyih Bahri**: Nefasit - Maha-bar, 2 Feb 1909, *Fiori 1593* (FT); Illalia - Scilliki; Assaorta, 28 Mar 1893, *Pappi 3601* (FT); Torrente Baratanti, 18 Apr 1893, *Pappi 4359* (FT); Nacfa - Maio, 19 May 1892, *Terracciano* & *Pappi 987 [2201]* (FT).

ETHIOPIA. Sep 1872, *Hildebrandt 696* (BM, L, W); Wouramboulchi, Sep 1926, *Omer Cooper* & *Cooper s.n.* (K); sin. loc, *Quartin-Dillon* & *Petit s.n.* (K); Semen, *Schimper 826* (W); Adama - Moggio, 9 Jun 1937, *Senni 819* (FT). **Addis Ababa**: Addis Ababa, 27 Sep 1961, *Albers 61013* (K); Addis Ababa, Good Shepherd School, 20 Dec 1966, *Gilbert 192* (K); 17 km SE of Addis Ababa, 22 Nov 1961, *Meyer 7497* (K); Addis Ababa, in compound of University College, 18 Jul 1962, *Mooney 9136* (FT, K); Addis Ababa, 22 Apr 1909, *Negri 205* (FT); Addis Ababa, 15 Jan 1937, *Senni 63* (FT); Addis Ababa, 9 Jul 1937, *Senni 1107* (FT); Addis Ababa, 20 Nov 1937, *Senni 2182* (FT); Addis Ababa, 30 Mar 1965, *de Wilde 6029* (K). **Amhara**: c. 15 km S of Debre Sina on the main rd towards Debre Berhan, 22 Nov 2000, *Friis et al. 10115* (K); Dessie, Wollo Prov., 18 Aug 1946, *Hall 22* (BM); Wello Prov., Azewagedel mountain, 2 km E of Desse, 11 Apr 1969, *Sutherland 167* (MO); Dessie, Dessie, 26 May 1938, *Vatova 2417* (FT); Semien Gondar, Semian Mountains, 24 Sep 1969, *de Wilde* & *Gilbert 204* (K). **Oromia**: Gara Mullata, 14 Feb 1962, *Burger 1483* (K); On the Mega Escarpment at the trail from Mega to the border with Kenya, 16 Dec 1997, *Friis et al. 8660* (K); Arengvadi Hayk, 5 km W of Bishoftu, 10 Jul 1969, *Gilbert* & *Gilbert s.n.* (K); Green Lake, 5 km W of Bishoftu, 10 Jul 1969, *Gilbert* & *Gilbert s.n.* (K); 5 km W of Debre Zeit, 10 Jul 1969, *Gilbert* & *Gilbert 1387* (EA, K); Gara Mullata, 3 Mar 1933, *Gillett 5285* (FT, K); Agheremariam-Dilla rd, 3 Dec 1952, *Gillett 14586* (EA, FT, K); Goba 3.4 km S of centre, Bale Prov., Togona and Micha Basin, 7 Aug 1962, *Herbert*, *49* (K); Kulubi, Misraq Hararghe Zone, Dec 1959, *IECAMA G58* (FT, K); Entotto, 28 Apr 1909, *Negri 244* (FT); 82 km from Nazareth, rd from Ethaya to Asella, 2 Sep 1967, *Westphal* & *Westphal-Stevels 1589* (MO); 7 km from Asella, rd to Bekoji, 4 Sep 1967, *Westphal* & *Westphal-Stevels 1643* (MO); 33 km from Shashemene, rd Koffale to Dodolla, 8 Sep 1967, *Westphal* & *Westphal-Stevels 1729* (MO); rd into Wondo valley, 4 km from Shashamane, 15 Nov 1967, *Westphal* & *Westphal-Stevels 2617* (MO); Bale Prov, 27 km W of Dinshu, along rd to Adaba, 10 Jan 1971, *de Wilde 7356* (MO); lower slopes of Mt. Cilalo, nr Asella, 10 Sep 1965, *de Wilde* & *de Wilde-Duyfjes8017* (K, MO, P); Alemaya College, Mar 1958, *Without Collector A-42* (K); Borena, Mega, presso fortino nuovo, 4 May 1937, *Cufodontis 627* (FT, W); Guli, Neghelli, 24 Nov 1967, *Westphal* & *Westphal-Stevels 2805* (EA, MO); Misraq Hararghe, Alemaya, campus of college of Agriculture, 11 Mar 1974, *Bos 7496* (MO); Misraq Hararghe, Alemaya, 9 Apr 1968, *Westphal* & *Westphal-Stevels 3989* (K); Semien Shewa region, Holetta, 26 May 1909, *Negri 564* (FT); West Arsi, Shashamene to Soddu, 97 km SW of Shashamene, 10 May 1968, *Westphal* & *Westphal-Stevels 4042* (K); West Arsi, Shashamane, Shashamene to Soddu. c. 97 km SW of Shashamene, 10 May 1968, *Westphal* & *Westphal-Stevels 4044* (K, MO). **Southern Nations (SNNP)**: Gurage Mountains, above Butajira towards the village of Ageta on the track towards Endibis, 24 Feb 2000, *Friis et al. 10066* (K). **Tigray**: Amba Alaga pass, Pass above Amba Alaga, 9 Oct 1995, *Friis et al. 6640* (K); just above the top of the escarpment where the new rd from Angula to Berhale and the Afar Depression begins descending to the plains, 15 Oct 2001, *Friis et al. 10689* (K); Adigrat, 15 Nov 1837, *Schimper I 46* (K); Bellaka, 8 Nov 1854, *Schimper 506* (E, FT, W); Arba Tensesa, 7 Oct 1862, *Schimper 523* (BM).

JORDAN. East Jordan, Pelit, 30 Apr 1963, *Gillett 15973* (K). **Aqaba**: Wadi Rum, 13 Apr 1945, *Davis 8987* (E). **Ma'an**: Petra, 28 Dec 1935, *Dinsmore 12151* (E).

KENYA. **Central**: Kiambu, Kabete grasslands, 1930, *Mettam 228* (K); Kiambu, Makuyu, Fort Hall Dist, 26 Jun 1960, *van Someren 11980* (EA, K); Nyeri, Aberdare National Park, The Ark, 7 Apr 1975, *Hepper* & *Field 4912* (K). **Eastern**: Makueni, Chyulu North, 21 May 1938, *Bally B-7788* (K); Makueni, Chyulu North, 28 Apr 1938, *Bally B-8306* (K); Makueni, Chyulu Hills North, Apr 1938, *van Someren 7644* (K). **Nairobi**: Kirichwa Ndogo, 3 Jun 1940, *Bally B 872* (EA, K); By Nairobi River, 28 Aug 1951, *Bally B 8033* (EA, K); Nairobi, Feb 1915, *Dümmer 1937* (BM, K, MO); Nairobi, 27 Sep 1915, *Dowson 298* (K, MO); Nairobi, Coryndon Museum area, 8 Jun 1956, *Kirika 241* (K); Mathare Valley, between Mathare Police Station and Eastleigh Section One, 11 Sep 1971,

Mwangangi & *Kasyoki 1797* (EA, K); Thompson Estate, 20 Jul 1930, *Napier 337* (EA, K); Nairobi, 1942, *Nattrass 341 b* (EA, MO). **Rift Valley**: Kericho, Londiani to Elburgon, Dec 1905, *Baker K-346* (EA, K); Ewaso Kedong, Mt. Margaret Estate, Jun 1940, *Bally B-1023* (K); Kajiado, Ngong escarpment, 21 Dec 1947, *Bally B 5736* (EA, K); Uasin Gishu, Soy, Oct 1931, *Brodhurst-Hill 197* (EA, K); Nakuru, Lake Naivasha, West shore Mennel's Farm, 14 Feb 1971, *Gillett 19300* (EA, K); Narok, Mara River, Ngerendei, 26 Mar 1961, *Glover et al. 181* (EA, K); Narok, Suswa, Lower NW slopes of mt, 20 Apr 1962, *Glover* & *Samuel, 2744* (EA, K); Kajiado, Olekairtoror Escarpment, 20 mi from Narok on Nairobi rd, 14 Jul 1962, *Glover* & *Samuel, 3110* (EA, K); Nakuru, Ol Longonot Estate, Naivasha Dist, 14 Jan 1962, *Kerfoot 3581* (EA, K); Samburu, Mathews Range, Ol Doinyo Kengio, Mt. Nyiru, 20 Dec 1958, *Newbould 3289* (EA, K); K6 Rift Valley, Suswa volcano, N rim of caldera inner S-facing slope, 1 Jun 1997, *Phillipson* & *Bytebier 4785* (MO); Nakuru, Elemteita, Dec 1893, *Scott-Elliot 6760* (K); Trans Nzoia, NE Elgon, Nov 1951, *Tweedie 1068* (K); Trans Nzoia, Mt. Elgon, to the NE, Jun 1958, *Tweedie 1586* (EA, K). **Western**: Nandi, Kapsoret Forest, 15 Jun 1951, *Williams 240* (EA, K).

SAUDI ARABIA. Al Mahmoud, 35 km N of Abha, 21 May 1980, *Boulos* & *Ads 14150* (E, K); Abha Pass (nr. W. Abha), 25 Oct 1971, *Popov 71 257* (BM); Arabia felicis, 1872, *Schimper s.n.* (GOET); SW Saudi Arabia, 10 Aug 1952, *Tothill 152* (BM). **Asir**: Jebel Sudah, c. 18 km N of Abba, 5 Apr 1979, *Collenette 1269* (K).

SOMALIA. Upper Sheikh Kitchen Garden, 1 Dec 1919, *Godman 67* (BM, MO). **Saaxil**: Ally Ully nr Sheikh, 11 May 1973, *Wood S/73-65* (K).

SUDAN. **Darfur**: Jebel Marra, Golel, c. 120 km E of Zalingei, 22 Jan 1965, *de Wilde et al. 5504* (K, MO).

TANZANIA. **Arusha**: Lake Manyara National Park, Mto ya Ukindu, 29 Nov 1963, *Greenway* & *Kirrika 11096* (EA, K); Lake Manyara National Park, Lake Manyara NP, Marera River, 9 Mar 1964, *Greenway* & *Kanuri 11332* (EA, K); Monduli, Kitumbeine Mt, 2 Mar 1969, *Richards 24245* (K); Monduli Forest Reserve, T2. Nr Olchoropus Village, 25 Jan 2001, *Simon et al. 720* (MO). **Central**: Kondoa, Great North rd, 11 Jan 1962, *Polhill* & *Paulo 1130* (K). **Kagera**: Ngara, Murugwanza, Ibivyaza Bakobwa, Bugufi, 20 Jan 1961, *Tanner 5617* (K). **Kilimanjaro**: Osirwa Farm , TBL Estates, 26 Jan 1994, *Grimshaw 94-148* (K); Ulei, Kwa Sadala, 2 May 1994, *Grimshaw 94-470* (K); Moshi, Sanya River, Mar 1928, *Haarer 1209* (EA, K). **Lake**: Ngara, Kirushya, Bugufi, 23 Nov 1959, *Tanner 4530* (K). **Mbulu**: Pienaars Heights, Great North rd, 120 mi S of Arusha, 5 May 1962, *Polhill* & *Paulo 2342* (EA, K). **Northern**: Mbulu, Mbulumbul, Block D1, 24 Jun 1944, *Greenway 6952* (EA, K); Mbulu, Mbulumbul, 26 Jun 1945, *Greenway 7459* (EA, K); Mbulu, Pienaars Heights, Great North rd, 120 mi S of Arusha, 5 May 1962, *Polhill* & *Paulo 2342* (K).

UGANDA. **Central**: Masaka, Kasambya, Kigezi, Feb 1948, *Purseglove P-2595* (K). **Eastern**: Sironko, Budadiri, Bugishi, Jan 1932, *Chandler 405* (K); Serere, at Tira, Jul 1926, *Maitland 1290* (K). **Northern**: Zombo, Paidha, 28 Aug 1953, *Chancellor 185* (K). **Western**: Kigezi D.F.I, 28 Aug 1972, *Goode G-5-72* (K); Kabale, Kigezi, Kabale Distr., Ndorwa County, Kyanamira subcounty, Kyonyo parish, 7 Jan 2000, *Olet 44* (K); Kasese, Muhokya, Ruwenzori, 25 Dec 1925, *Maitland 1790* (K); Kigezi, Kachwekano Farm, Sep 1949, *Purseglove 3121* (EA, K); Kisoro, Virunga-Kette, Muhavura, Nkanda, 25 Nov 1954, *Stauffer 957* (EA, K, P).

YEMEN. Taifa, 1838, *Botta s.n.* (LE). **Hadramaut**: Al Mukalla, Arabia, West rd, 6 Sep 1949, *Guichard KG/HAD 35* (BM); Kor Seiban, 14 Sep 2002, *Killian et al. YP-3578* (B). **Sa'dah**: Sadah, 1 Jul 1984, *Gordon 609 B* (E). **Sana'a**: Sana'a, 12 Mar 1981, *Miller 3004* (E); 14 Feb 1934, *Rathjens s.n.* (BM); 28 Feb 1934, *Rathjens s.n.* (BM); 29 Sep 1937, *Rathjens 37/189* (BM); north Yemen, 15 Sep 1937, *Rathjens 37/ 205* (BM); Menacha, 7 Mar 1889, *Schweinfurth 1476* (BM, GH, K, P); Ar Rowdah nr San'a, 23 Feb 1972, *Wood 72-7* (BM); 10 Oct 1974, *Wood Y/74-20* (BM). **Ta'izz**: Jabal Sabir, c. 15 km S of Taizz, 11 Jun 1982, *Gordon 11* (E); Jabal Sabir, nr Taiz, 23 Sep 1977, *Lavranos 15947* (E).

Solanum nigrum

AFGHANISTAN. Sari Casma, 31 Jul 1939, *Bacon 93* (GH); Kanard, 12 Aug 1940, *Cowgill 2076* (GH); Kohri-Elburz 10 mi SW Mazar-i-Sharif, 10 Jun 1966, *Furse 7778* (K); sin. loc, *Griffith [663]*

(K). **Badakhshan**: Shuturjangal, 30 Jun 1937, *Koelz 12172* (W); Kalat, 14 Oct 1937, *Koelz 13219* (W). **Balkh**: 10 km W Balkh an der Straße nach Aqcha, 7 Oct 1970, *Podlech 19792* (K, KUFS). **Bamian**: Kala Sarkari, 8 Sep 1939, *Koelz 13911* (W). **Kabul**: Paghman W Kabul, 23 Sep 1969, *Podlech 16842* (KUFS). **Khandahar**: Pirzada, nr Kandahar, 4 Jun 1948, *Köie 3127* (W). **Kunar**: Bashgal Tal bei Ormol, 29 Jul 1978, *Podlech 32146* (KUFS). **Nangarhar**: ghaziabad, an der Strasse Jalalabad-torkham Farmgelaene, 24 Oct 1970, *Podlech 19950* (KUFS); Jalalabad pohantuns Garten, 24 Oct 1969, *Sharifi 531* (KUFS). **Wardak**: unteres Maidan Tal bei Kita-i-Ashro, 26 Jul 1970, *Podlech 18676* (KUFS).

ALBANIA. **Central Albania**: Tirana, 28 Aug 1918, *Schneider s.n.* (W). **North Albania**: Shkodër, Skutari, 11 Aug 1944, *Hopflinger s.n.* (W). **South Albania**: Vlorë, Sarandë, Santi Quaranta, 1 Jun 1933, *Alston* & *Sandwith 1289* (BM, K).

ALGERIA. Kouba, Oct 1878, *Gandoger 27* (BM); Cap Lindles pres Oran, Jul 1849, *Romain s.n.* (BM); **Biskra**: Col des Chiens, 27 Jun 1902, *Chevallier s.n.* (K).

ARMENIA. **Tavush**: Distr. Dilishan, montes Gugarac, inter oppidium Dilishan et vicum Shamakhyan, 2 Oct 1974, *Vašák s.n.* (W).

AUSTRALIA. Nov. Hollad. meridional., *von Mueller s.n.* (W); Waite Institute, 2 Apr 1968, *Symon SS-141* (NSW); Celle, Hanover, Sep 1876, *Unknown s.n.* (E). **Christmas Island**: 3 Oct 1981, *Powell 410* (K). **New South Wales**: junction of Jock's Creek & Wollondilly River nr Goodman's Ford, 39km WNW of Mittagong, 16 Mar 1975, *Coveny et al. 6087* (K, MO); Manning River, 22.5 km (14 miles) by rd NNW of Gloucester on Walcha Rd, 1 Jun 1975, *Coveny et al. 6397* (K); Thomsons Creek nr Mt Wood homestead, NE of Tibooburra in Sturt National Park, 6 Sep 1989, *Coveny et al. 13589* (MO); Central West, Gillies Bridge over Trigalong Ck, c. 4km W of Temora, on the rd to Griffith, 15 Apr 1988, *Dalby et al. 88/05* (MO); Western Plains, ca. 10km NE of Narrabri, 23 Sep 1975, *Henderson 2314* (AD, MO); Turrell Creek, Kars Springs, c. 38.5 km (24 miles) WNW of Scone, 10 Jun 1969, *Pickard & Coveny 1216* (K); Haura, Jan 1926, *Rodway s.n.* (K); Brush Island 1/4 mile S off Murramarang Point 15 mls S of Ulladulla, 16 Aug 1936, *Rodway 2258* (K); Windang Island, entrance to Lake Illawara S of Wallongong, 17 Apr 1938, *Rodway 2694* (K); Hornsby, Epping, 2 Apr 1968, *Symon SS-108* (NSW); Canberra, 24 Feb 1972, *Symon 7627* (AD); **Norfolk Island**: Sin. loc., *Without Collector s.n.* (K); **Northern Territory**: CS Dist., Junction Bore, Numery Stn, 16 Sep 1993, *Latz 13385* (MO); Uluru Ayers Rock-Mt. Olga National Park, Ulura Ayers Rock, Mutitjulu Maggie Springs walk, 1.7km NE of the Ranger Stn, 11 Aug 1988, *Lazarides & Palmer 332* (K, MO); **Queensland**: NW of Oakey, corner of Warrego Hwy and Cockburn's Road, 29 Jan 2006, *Bohs et al. 3531* (BM, UT); Burke District, Hughenden, 23 Jul 1963, *Everist 7291* (K); Moreton Bay, 27 Jun 1968, *Henderson 404* (BRI); Moreton Bay, 27 Jun 1968, *Henderson 405* (BRI); Southern Downs, The Summit, c. 5 mls (8 km) NNE of Stanthorpe, 8 Feb 1972, *Henderson & Parham 1242* (AD, BRI, MEL, PERTH); Burnett Distr., Kingaroy, 16 Apr 1947, *Smith 3071* (AD, K); Carpentaria, At the Flinders River crossing, 40 mile SW of Normanton, 29 May 1967, *Symon 4954* (AD, BRI, CANB); **South Australia**: Adelaide Plains, Beneath bridge 8km north of Gepp's Cross on Pt. Wakefield rd, first creek met; Gepp's Cross is a northern suburb of Adelaide, 20 Jun 1967, *Barker 51* (MO); Stirling, Mt. Lofty Range, angle between Pine Street and rd connecting it with Milan terrace, 19 Apr 1957, *Booth 77* (E); Watson railway lines, Sep 1956, *Bowen 294* (K); Adelaide Hills, Slapes Gully quarry site, 25 Mar 2009, *Brodie & Symon 192* (AD); Mount Gambier, Mt Gambier Lakes, Valley Lake, 28 Jan 2010, *Brodie & Symon 1247* (AD); Wattle Range, by Glencoe on Kalangadoo Flat Rd by Glencoe Drain in native patch by the Everglades, 29 Jan 2010, *Brodie & Symon 1346* (AD); Coober Pedy, Lot 754, Coober [Pedy] Flat's Drive, 22 Apr 2010, *Brodie et al. 1653* (AD); Franklin Harbour, on Beach Rd between Cowell and Pt Gibbon, 7 Feb 2011, *Brodie & Symon 2448* (AD); Tumby Bay, On Highway c. 10 km N of Port Neill, 8 Feb 2011, *Brodie & Symon 2520* (AD); Tumby Bay, just off Lincoln H[igh]w[a]y c. 25-27 km N of Tumby Bay in rest area, 8 Feb 2011, *Brodie & Symon 2546* (AD); Lower Eyre Peninsula, Licoln H[igh]w[a]y c. 19 km N of P[or]t Lincoln, 9 Feb 2011, *Brodie & Symon 2601* (AD); Elliston, 2 km S of Sheringa on Flinders H[igh]w[a]y, 10 Feb 2011, *Brodie & Symon 2669* (AD); Adelaide Hills, Uraidla, 16 Mar 2011, *Brodie et al. 2859* (AD); Port Adelaide Enfield, Adelaide met[ropolitan] beach, 6 Apr 2011, *Brodie & Symon 2888* (AD); Alexandrina, SE of Langhorne Creek by Lake Alexandrina at Tolderol Game Reserve, 8 Jun 2011,

Brodie & Symon 3111 (AD); South Eastern, Glencoe West Rd, nr Millicent, 25 Jan 1969, *Carrick 2169* (MO); Kyeema Conservation Park, Region II, Southern Lofty, 13 Dec 1984, *Donner 10365* (MO); Left bank of Murray, 3km upstream of Berri, 17 Aug 1975, *Horton 56301* (MO); Adelaide Botanical Garden, 15 Sep 1994, *Nee 45551* (MO); 13 m SE Alice Springs, 23 Aug 1962, *Nelson 523* (K); Buxton County, Eyre Peninsula, at crossroads between section 65 Buckleboo and Pinkawillinie, ca. 40 km WNW of Kimba, 12 Apr 1959, *Rocrlach 290* (K); 78 Stunt Ave., Clapham, South Australia, 29 Jan 1963, *Symon s.n.* (K); Wilpena, 2 Apr 1968, *Symon s.n.* (AD); Cummins Road, Sect[ion] 15, H[undre]d of Mortlock, 2 Apr 1968, *Symon s.n.* (AD); Sin. loc., 9 Apr 1968, *Symon s.n.* (AD); Mitcham, Rose Garden, WARI, Glen Osmond, 18 Jan 1960, *Symon s.n.* (AD); Adelaide Hills, Shores of Millbrook Reservoir, 13 Mar 1958, *Symon s.n.* (AD); Burnside, Upper part of Hartley Grove, Glen Osmond, 12 Jun 1969, *Symon s.n.* (AD); Mount Remarkable, Lower Mambray Creek, 11 Jun 1960, *Symon s.n.* (AD); Creek bed, Arkaba, 9 Sep 1961, *Symon s.n.* (AD); Gov[ernment] Works Dept and Petrol Yard at Leigh Creek, 14 Aug 1968, *Symon s.n.* (AD); Oraparinna National Park, 13 Sep 1971, *Symon s.n.* (AD); Mount Gambier, Leg of Mutton Lake, Mt. Gambier, 20 Jan 1965, *Symon s.n.* (AD); Mount Gambier, Shores of Brown Lake, Mt. Gambier, *Symon s.n.* (AD); Provenance unknown, 13 May 1969, *Symon s.n.* (AD); Sin. loc., 13 May 1969, *Symon s.n.* (AD); Robe, Little Dip Conservation Park, 26 Jun 1984, *Symon s.n.* (AD); Mitcham, Directors Garden, Waite Institute, 20 Jan 1965, *Symon s.n.* (AD); Mitcham, 78 Sturt Ave, Clapham, 29 Jan 1963, *Symon s.n.* (AD); Wilpena, 16 Feb 1966, *Symon s.n.* (AD); Fowlers Bay, 29 Sep 1959, *Symon s.n.* (AD); Inila Rock [Waters]. 14 m N [of] Koonibba Siding, 30 Sep 1959, *Symon s.n.* (AD); Adelaide, Below Insectary, WARI Waite Institute, 17 Jan 1962, *Symon s.n.* (NE); Pt Clinton, 2 Apr 1968, *Symon 110* (AD, CANB); Wilpena, 12 Mar 1968, *Symon 113* (AD); W end of clearing nr West Point, Flora and Fauna Reserve, H[undre]d of Flinders, 2 Apr 1968, *Symon 117* (AD, CANB); Lower Eyre Peninsula, Cummins Road, Sect[ion] 15, H[undre]d of Mortlock, 2 Apr 1968, *Symon 119* (AD); Yalunda Flat, H[undre]d of Kappio, 2 Apr 1968, *Symon 120* (AD, CANB); Tumby Bay, 5 m from turnoff to Koppio, on the Tumby Bay - Lincoln rd, on the rd to Koppio, 16 m from Tumby Bay, 12 Nov 1960, *Symon 879* (AD); Adelaide Hills, 28 Feb 1961, *Symon 1165* (AD); Brown Lake, Mt Gambier, 6 Mar 1961, *Symon 1181* (PERTH); Mt Christie Siding, 20 Oct 1987, *Symon NPYE-1604* (AD); Mitcham, WARI, 17 Jan 1962, *Symon 1944* (AD); Mt Remarkable, N end of Horrocks Pass, 14 Mar 1962, *Symon 2089* (AD); Onkaparinga, Waite plots, Happy Valley, 11 Apr 1962, *Symon 2109* (AD); Along the creek at Wilpena, 28 May 1962, *Symon 2134* (AD); Clements Gap, 13 Aug 1964, *Symon 2918* (AD, K); Yorke Peninsula, Monesty just N of Maitland, 14 Aug 1964, *Symon 2921* (AD); About the dam site at Wynbring Rocks, 21 Feb 1965, *Symon 3430* (AD); Hesso Station, boundary of Yudnapinna and Hesso St[atio]n nr the Grid at Ives Dam, 28 Sep 1966, *Symon 4065* (AD); Down at the sink hole at Koonalda, 17 Feb 1967, *Symon 4531* (AD, CANB, K, NSW); Elliston, Far S end of the Hundred of Blesing in Cutting Grass Flat nr windmill and tank, 10 Oct 1967, *Symon 5486* (AD, CANB); 15 m NE of Mundy Crk on rd to Murnpeowie Stn, 15 Aug 1968, *Symon 5599* (AD); In the bed of the creekline at Moolawatana Stn, 22 Aug 1968, *Symon 5945* (AD, CANB); Base of small creekline into North Bay, Pearson Island at base of Hill 781, 6 Jan 1969, *Symon 6608* (AD); Pearson Island, S side of main island, 9 Jan 1969, *Symon 6635* (AD); Tea Tree Gully, L.H. Wich's Nursery, Highbury, 13 Feb 1969, *Symon 6690* (AD); Oraparinna National Park. oming down ABC Range into Aroona Valley, almost opposite Mt. Hayward, 13 Sep 1971, *Symon 7263* (CANB); Franklin Harbour, Mt Olinthus, Cowell Hills, 25 Jan 1972, *Symon 7618* (AD); Mitcham, in angle of The Devils Elbow, Mt Barker Rd, 3 Mar 1972, *Symon & Henderson 7631* (AD, CANB); Adelaide Hills, Protected site, Raywood Nursery, Crafers, 3 Mar 1972, *Symon & Henderson 7633* (AD); Mitcham, in angle of The Devils Elbow, Mt Barker Road, 13 Sep 1972, *Symon 8012* (AD); Gawler Ranges, Chimney Well due S of Lake Acraman, Yardea grid ref. 338019, 5 Oct 1972, *Symon 8164* (AD, CANB, MO); Cleve, Carappee Hill, about and above camp site, 14 Sep 1974, *Symon 8845* (AD); [Cultivated] Pot grown at Waite Agricultural Research Institute, 16 Oct 1975, *Symon 9931* (AD, PERTH, PERTH); Beresford Tank, 3 Oct 1978, *Symon 11275* (AD); Murray Bridge, Upper R[iver] Murray nr 375 mile peg on N bank, 14 Sep 1979, *Symon 11587* (AD); Lower Eyre Peninsula, Along creekline at Coulta Village, 2 Oct 1979, *Symon 11777* (AD); Alexandrina, Kuitpo Forest at junct[ion] of Adams Gully Rd and main

Meadows - Willunga R[oa]d, 25 Jan 1984, *Symon 13742* (AD); Moralana St[atio]n. Red Well P[a]d[oc]k, 11 Oct 1984, *Symon 14144* (AD); Adelaide Hills, Just S of Lenswood, off main rd and leading to creekline, 22 May 1986, *Symon 14261* (AD, NSW); Region 5, Flilnders Ranges, Moralana Station, rd and rail-crossing of Bunyeroo Creek, 10 Jul 1987, *Symon 14613* (MO); Moralana Station, Rd and rail [cross]ing of Bunyeroo Creek, 10 Jul 1987, *Symon 14618* (AD); Adelaide, Torrens Linear Park between Gilberton Swing Bridge and Stephen Terrace, 8 Apr 1989, *Symon 14855* (AD); Manunda Creek at rd crossing. 15km NE of Faraway Hill, 23 Jul 1991, *Symon 15064* (AD); Goyder, 27 km due N of Burra & 8 km SE of Hallett, 20 Jan 1994, *Symon 15165* (AD); Kennebery Creek, adjacent to Kennebery Waterhole, 27 Sep 1995, *Symon 15263* (AD); Yorke Peninsula, N shore of Marion Lake, 6 Nov 2001, *Symon & Symon 16610* (AD); Light, Roseworthy Agric[ultural] College, 1946, *Symon 18149* (AD); S Adelaide, *Whittaker s.n.* (K); Klemzig, a suburb 7 km NE of Adelaide, Adelaide Plains, 23 May 1964, *Wilson 3597* (AK); **Tasmania**: Blythe Heads, 17 Jan 1961, *Burns 433* (K); Pipe Clay Bay, George Town, 25 Dec 1965, *Burns 611* (K); Pipeclay Bay, George Town, 9 Jun 1969, *Burns 736* (K); mouth of Derwent River, South Arm Road, at Gellibrand Rd, ca. 4.5 km E of South Arm, 9 Oct 1978, *Orchard 5024* (AK); Knocklofty, 29 Mar 1973, *Ratkowsky & Ratkowsky 359* (MO); North Bruny, Jan 1931, *Rodway 121* (K); Waratah/Wynyard, Sisters Beach, 16 Jan 1999, *Symon & Symon 15941* (AD, HO); sin. loc, *Without Collector s.n.* (W); lawrance V.D.Ld [Van Danems Land], *Without Collector 55* (W); **Victoria**: Melbourne, Shedly Park, 1 Mar 1871, *Morrison s.n.* (E); Frankston, 7 Oct 1887, *Morrison s.n.* (E); Brighton, 24 Apr 1891, *Morrison s.n.* (E); Lilydale, 6 Mar 1871, *Morrison s.n.* (E); Kew, 23 Apr 1885, *Morrison s.n.* (E); Sin. loc, *Mueller s.n.* (MA); Sin. loc, *von Mueller s.n.* (W); Beynan's Creek flats at Morgan's Mill, Western Tyers, 7km direct NW of Erica, 20 Apr 1987, *Scarlett 87-39* (MO); Northern Grampians, View Point Rock, Grampians, 11 Nov 1959, *Symon 222* (AD); Grampian Mountains, Hall's Gap, Dec 1912, *Tilden 832* (BM, K, MO). **Western Australia**: Murcheson River, Old School House E & S of the bridge at Galena camp area, NW coast Highway, National Route1, c. 75km E of Kalbarri, 27 Sep 1993, *Alcock 11425* (MO); Claremont, Aug, *Andrews 658* (BM, K); Northampton, Champion Bay, Apr 1909, *Campbell s.n.* (K); Gingin, Ca 6 km SE of Lancelim, on rd to Gin Gin, 8 Sep 1988, *Henderson 3131* (MEL, PERTH); Booragoon Lake, 10 km S of Perth off Leach Highway, 19 Mar 1987, *Keighery & Alford 1282* (CANB, K, PERTH); 2.5km S of Point Coulomb, Dampierland, N of Broome, 21 Apr 1977, *Kenneally 6095* (K); Cannington, lower Canning river, 10 Jun 1905, *Morrison s.n.* (BM); Subiaca, 413, Rokeby Road, 11 Oct 1908, *Morrison s.n.* (BM, E); Perth, Garden at 413 Rokeby Road, 11 Oct 1908, *Morrison s.n.* (K); Swan view, Midland Junction, 20 Apr 1904, *Morrison 14228* (K); Northampton, 41 m N of Geraldton, 4 Jul 1967, *Symon 5449* (AD, CANB, PERTH); Sandstone, 5 Jul 1967, *Symon 5462* (AD, PERTH); 25 km S of Leonora, 12 May 1975, *Symon 9922* (AD); 58km N of Leonora, 12 May 1975, *Symon 9929* (AD, MO); Leonora, 95 km S of Wiluna, 13 May 1975, *Symon 9952* (AD); Halls Creek, 2 Jun 1975, *Symon 10310* (AD, CANB, PERTH); Dundas, Roe Plain, 29 km W of Eucla, 29 Sep 2002, *Symon & Symon 16858* (AD); Dundas, Fraser Range Stn, 3 Oct 2002, *Symon & Symon 16910* (AD); Dundas, Fraser Range Stn, 30 Dec 2002, *Symon 17075* (AD); York Road, Jun 1877, *Unknown s.n.* (E); Coolgardie distr., *Webster s.n.* (BM).

AUSTRIA. **Nieder-Österreich**: Marchtal, uberschwemmt gewesenes Brachland, c. 0.65-0.9 km E-ESE der Kirche von Dürnkrut, 10 Aug 2006, *Barta s.n.* (W); Stadtischer Müllableerplatze an der Mannswörther Strasse, 12 Oct 1969, *Forstner s.n.* (W); Illmitz, Burgenland, Nieder Osterreich, rive N du Neusiedler-See, 25 Oct 1975, *Lambinon 906* (BM); Leitha-Gebiet, Bruck-Zurndorf, 24 Oct 1964, *Neumann s.n.* (W); Austria inferior. Prope Wien, Oct 1918, *Rechinger s.n.* (BM); Klause bei, 9 Oct 1927, *Ronniger s.n.* (W); Austria inferior, ad sepes et muros in vico Pillichsdorf, 10 Aug 1917, *Vettinger s.n.* (K); Marchfeld, zwischen Markgrafneuseidl und Parbasdorf, c. 1.3 km WNW, 4 Sep 1996, *Walter 4135* (W); marchfeld, zwischen Markgrafneuseidl und Parbasdorf, c. 1.3 km WNW, 4 Sep 1996, *Walter 4136* (W); Donautal, unterhalb Wien, Fischamend-Markt, E d Bahnhofs, 5 Sep 1995, *Walter 4674* (W). **Ober-Österreich**: Linz, Ortsgebeit, (ehemaliger) Frachten-bahnhof, zwischen Lastenstrasse und Autobahnbrücke, 28 Sep 2006, *Karl s.n.* (W). **South Tyrol**: S.E. Tyrol, Botzen, 21 Aug 1861, *Hort s.n.* (BM). **Steiermark**: Steiermark, Aussee, nach Grundelsee, 21 Sep 1947, *Rechinger s.n.* (W). **Tirol**: Nordtirol, Wipptal, zw. Europabrücke und Stephansbrücke linke Talseite, 24 Jul 1971, *Polatschek s.n.* (W); Nordtirol, Zillertal, Linkes Ziller-Ufer zwischen Zell/Ziller

und Hippach, 4 Aug 1986, *Polatschek s.n.* (W). **Vorarlberg**: Walgau, Thüringen, 3 Aug 1972, *Titz s.n.* (W). **Wien**: Vienna, Umberfafen, 2 Sep 1951, *Alston 11214* (BM); Vienna, between Reichsbrücke & Ostbahnbrücke, 2 Sep 1951, *Alston 11218* (BM); Vienna, Reichsbrücke, 2 Sep 1951, *Alston 11268* (BM); 11 Bezirk, auf Odland in einem aufgelassenen Garten nahe der Ecke Simmeringer Hauptstrasse/Otto Mraz-Weg, 5 Dec 2006, *Barta 2006-20* (W); 3 Bezirk, ruderaler Strassenrand and der Grasbergergasse zwishen Leberstrasse und Rennweg, 23 Aug 2003, *Barta 2003-372* (W); Vienna, Jul 1919, *Hübl s.n.* (BM); Floridsdorf, 12 Sep 1979, *Pull s.n.* (W); Vienna, Nieder-Österreic prope Wien, Oct 1918, *Rechinger s.n.* (BM); Ober St. Veit, 10 Oct 1927, *Seiller s.n.* (W); Acker bei Ober St. Veit, 30 Aug 1925, *Seiller s.n.* (W); Wienerwald, öberhalb des Stfiftes Heiligenkreuz in Gumpoldskirchen, 25 Oct 1981, *Till 71* (W); Vienna, Erster Bezirk, Ruderalflur vor dem Natur-historischem Museum (Seite zum Ring), c. 200 m, 20 Nov 1992, *Wallnöfer 4343* (BM); 16 Bezirk, Hasnerstrasse/Haberlgasse, 29 Sep 2011, *Walter 9163 a* (W).

BAHRAIN. **Bahrain**: Isa Town, 1985, *Naguib 9* (K).

BELARUS. **Grodno Region**: Navahrudak, Lithuania, Niankow, distr. Nowogrodek, 5 Sep 1898, *Dybowska 853* (E, W); Navahrudak, Niankava, Nianków, 1898, *Dybowska* & *Woloszczak s.n.* (BM).

BELGIUM. Brussels: Brussels, quartier de la gare du Nord, 11 Oct 1978, *Lawalrée 21201* (BM); Wohne St. Lambert chemin des u Deux Maisons, 4 Sep 1925, *LeDoux s.n.* (W). **Flanders**: Flemish Brabant, Brabant, Meise, nouveau parking di Jardin botanique, Aug 1987, *Geerinck-Coutrez, 4368* (BM). **Wallonia**: Faulx-les-Tombes, 24 Aug 1981, *Lawalrée 23602* (BM).

BHUTAN. Trongsa & Tsanka, 28 Jun 1938, *Gould 628* (K).

BOSNIA AND HERZEGOVINA. In valle Miljac ad Sarajevo c. 560 m, 24 Sep 1900, *Maly s.n.* (K); Vranja, 30 Sep 1919, *Tindall Lucas s.n.* (BM).

BULGARIA. prope Sliven, 25 Jul 1907, *Schneider 705* (MO); Sadovo, 10 Sep 1899, *Stribrny s.n.* (E).

CAPE VERDE. **Santo Antão**: sin. loc, 30 Dec 1974, *Gilli 30* (W).

CHINA. Shantung Prov., Meng Shan, Mei Hsien, 13 Jul 1936, *Cheo* & *Yen 60* (GH); sin. loc, *Forrest 7289* (K); Patung Distr., 1887, *Henry s.n.* (GH); *Herb. Pallas s.n.* (BM); *Herb. Pallas s.n.* (BM); N Central China. Mt Miao-Wang-san, 1899, *Hugh s.n.* (BM); Mt Miao-Wang-san, 1899, *Hugh s.n.* (BM); N Central China. Mt Ngo-san, Jul 1899, *Hugh s.n.* (BM); *von Jacquin s.n.* (BM); Journey from Mukden to Kirin, between Hui Fa River and Kirin, Dec 1886, *James s.n.* (K); Manshuria Chinensis, provincia Kininensiy, via inter Ninguta et Orioso, Vally Tehitu-densa, 14 Jul 1896, *Komarov 1376* (K); Liaoning Sheng, Lueda Shi, the campus of Dalian Institute of Foreign Languages, Dalian-Shi, 25 Jul 1985, *Koyama 7669* (A); China bor. Tschili, Haitou-schan, pr. Yati, 18 Aug 1930, *Licent s.n.* (W); sin. loc, *Millett s.n.* (K); sin. loc, *de la Touche 512* (K); E coast, *Su s.n.* (BM); Si-Kang Prov., Dzer-nar, Tsa-wa rung, Sep 1935, *Wang 66363* (A); S. Wushan, *Wilson, 2660* (K, W); Between the way from han po kou to wu lao feng, 23 Sep 1983, *Yao 8611* (A, K, MO, P). **Heilongjiang**: Manschurei: Gegend von Harbin, 1926, *Jettmar s.n.* (W). **Anhui**: Shek Kuet Ts'o, Chiu Hwa Shan, 1934, *Fan, C.S.* & *Li, Y.Y. 179* (ECON, K); Fengyangshan, Nov 1873, *Forbes 307* (BM); Anhui Sheng, Yuexi Xian, Yaoluoping, 15 Jul 2002, *Xu Ren-xin, 40* (K); Ma-an shan, 2 Nov 1982, *Yao 8509* (A, MO). **Beijing**: Beijing, 3 Jul 1925, *Chien 198* (W); Beijing, nr the Peking wall, 15 Nov 1898, *Niederlein 149* (A); Beijing, nr the Imperial Ming tombs, Nov 1898, *Niederlein 286* (A); nr the Summer Palace, 23 Jul 1986, *Wang 050* (EA, H). **Chongqing**: Yangtze-Kiang, Chungking, *Faber 63* (K). **Fujian**: Foochow and vicinity, 4 May 1936, *Chen 110* (A); Amoy Island, grounds of University of Amoy, 25 May 1923, *Chung 1647* (K); Nanputo, Amoy, 6 May 1926, *Chung 4892* (A); Amoy, 2 May 1927, *Chung 5974* (A); Shaowu and vicinity, 1926, *Fan Hsioh Niao 9120* (A); Amoy Island, around Canton and Amoy; Company's garden, 5 Mar 1834, *Lay s.n.* (BM); Amoy Re, Kulangen Island, 29 Apr 1923, *Lim 1440* (K); Hingwha and vicinity, Mong-Go-Lau, 14 Jul 1926, *Liu Pi, 6042* (A); Hingwha and vicinity, 7 Sep 1926, *Liu Pi, 6512* (W); Gushan, kushan Monastery, 3 Aug 1926, *Po 12110* (BM); Asai Island, 17 Aug 1926, *Tai 11835* (BM); Ngie su sang, university and vicinity, Foochow, Fukien Prov., 31 Dec 1926, *Tang Siu Ging 6978* (MO); Fukien Prov., Gang Cia Hiong, Along Min River, N of Foochow, 24 Jan 1927, *Tang Siu Ging 7322* (MO); Siong Siu village, University and vicinity, Foochow, 9 Mar 1927, *Tang Siu Ging 13156* (A); IngHok, Gak-liang, 27 Mar 1927, *Tang Siu Ging 13358* (A); Foochow and vicinity, Siu San, 12 Apr 1927, *Tang Siu Ging 13526* (A); Hok Chiang, Ling-Soik temple and vicinity, 17 May 1927, *Tang Siu Ging 13981* (A); Kushan monastery, 3 Aug 1926, *Uong Sing Po 12110* (A). **Gansu**: Wen Xian, Motianling Shan, Baishui Jiang

Nature Reserve, ENE of city of Bikou, Ping Li, along Xiaotuanyu He, 8 May 2007, *Boufford et al. 37533* (A). **Guangdong**: Honan Island, 20 Jan 1927, *Canton Christian College 373* (GH); Guangzhou, Dec 1884, *Herb. Sampson, 442 A* (BM); Guangzhou, Canton vicinity, *Levine s.n.* (US); sin. loc, 6 Jul 1886, *Murray s.n.* (BM); Guangzhou, 24 Sep 1883, *Sih-Chiu 442* (BM); Lantau Island, 28 Dec 1927, *Tsang 16639* (A); Kwangtung, Tai Hang Pah, Chong Uen Shan nr Kau Fung, Pak Fa Tsoi, Loh Ch'ang Distr., 2 Nov 1932, *Tsang 20890* (K, MO, W); to Taipo Station, 5 Apr 1928, *Tsiang Ying 133* (W). **Guangxi**: Tian'e Xian, Guangxi, 21 May 1957, *Wang 43427* (MO). **Guizhou**: sin. loc, 1936, *Teng 91053* (GH). **Hainan**: sin. loc, Nov 1889, *Henry 8511* (K); Nodoa and vicinity, 3 Feb 1933, *Lau 1005* (A, BM, P); Chim Fung Mtn., nr Sha Mo Kwai village, Kan-en Distr., 13 Dec 1934, *Lau 5025* (A). **Hebei**: Sulu Hsien, Hsin Chi (Central Hopei plants), 15 Aug 1948, *Beach 46* (K). **Henan**: Lo Yang, 11 Jul 1928, *Chiao 18517* (F); Teng-feng, im Kreise Teng fong, Aug 1907, *Schindler 1409* (BM); Kikungshan (Honan Prov.), 25 Jul 1925, *Steward 9691* (K). **Hong Kong**: sin. loc., *Champion s.n.* (K); sin. loc., 20 Apr 1989, *Goring 2* (K); Oct 1855, *Hance 861* (BM); Champon, 1841, *Hinds s.n.* (K); CHUK campus, Hong Kong, 17 Jan 1992, *Hu* & *But 19992* (A, MO). **Hubei**: Yichang, *Henry 625* (K); Yichang, IChang, *Henry 1024* (K); Nan-to and mountains northward, *Henry 3210* (K); along rd nr Wuhan Botanic Garden, 30 Sep 2007, *Knapp et al. 10143* (BM); Shennongjia Forest Distr., vicinity of Mucheng along the Jiuching River, 31 Aug 1980, *Sino-American Expedition 429* (A). **Hunan**: Anhua Co, 27 Oct 2004, *Duan 3648* (MO); Shaoyang Co, Daxiangqu, 25 Aug 2003, *Duan 20020641* (MO); Yizhang Xian, Mt. Mangshan, 30 May 2005, *Xiao Bai-Zhong 4195* (K); Yizhang Xian, Mt. Mangshan, 4 Jun 2005, *Xiao Bai-Zhong 4230* (K). **Hupeh**: Howng-shan, Wuchang, 9 Nov 1932, *Teng 259* (W). **Jiangsu**: Nanjing, 16 Oct 1928, *Chien 395* (K); Nanjing, 1980, *Chow 80-300* (BM, K); I-hing, 1 Oct 1979, *Fang 8001* (MO); Taizhou, Duzihe, c. 5 km N of Taixing City, 1 Oct 1997, *Miller & Zhu 9208* (E, GH, MO); Paohuashan, Kuyung, 29 Oct 1932, *Tsiang Ying 10817* (W); Nanjing, 19 Oct 1920, *Without Collector 1571* (K); wild in garden of Jiangsu Botanical Institute, 15 Sep 1932, *Yao 8384* (A). **Jiangxi**: Kiangsi, *Canton Christian College 51926* (A); Soochow University, 3 Nov 1924, *Feng 31* (GH); sin. loc, 1921, *Hu 972* (K); Sai Hang Cheung, nr Tung Lei village, Kiennan Distr., 1 Aug 1934, *Lau 4236[a]* (GH); Kiennan, Tung-Liu, nr Tung Lei Village, Sai Hang Cheung, 1 Aug 1934, *Lau 4238* (BM); Xiu-shiu county, Jiangsi, 17 Sep 1989, *Liu 890109* (MO). **Jilin**: between Hui Fa River and Kirin [Jilin], May 1886, *James s.n.* (A). **Kongbo**: Tsango Valley, South Eastern Tibet, Tsela Dzong, 8 Aug 1938, *Ludlow et al. 6010* (BM). **Liaoning**: Newchwang, 28 Jun 1893, *Bullock 323* (K). **Macau Special Admin Region**: Macao, 1862, *Tate s.n.* (K). **Ningxia**: Yin Chuan, 21 Sep 1979, *Virgo 130* (K). **Shaanxi**: China interior, provincia Shen-si septentr, 1897, *Giraldi s.n.* (K); Shaanxi, Zhouzhi Xian, Xhouxhi, Taibaishan, Houzhenzi, 23 Jul 1999, *Zhu et al. 2228* (MO); Yang Xian, Huayang, 8 Jul 1999, *Zhu et al. 2897* (MO). **Shandong**: Meng Shan, Meng Shan. Fei Hsien, 13 Jul 1936, *Cheo* & *Yen 60* (BM, P, W). **Shanxi**: Yuanqu, Shunwangping, Yuan-Chu Distr., Shui-wang-ping, 24 Jul 1921, *Smith 6586* (MO); Taiyuan, Jinci, ad templum Chin-ssu, 10 Aug 1924, *Smith 6842* (MO). **Sichuan**: Dujiangyuan Municipality (formerly Guan Xian) Qingchenhou Mountain in Wulonggou, 24 Aug 1988, *Boufford et al. 24134* (A); Xiangcheng Xian, Xiarewu, on rd between Xiangcheng and Daxue Shan (road to Zhongdian from Xiangcheng), 22 Jul 1998, *Boufford et al. 29001* (A); Guan Xian, Jushouhemantan, 18 Jul 1987, *Cao Zi-yu, 16* (K); Pao-hsing Hsien, Pao-hsi-hsien, 8 Sep 1936, *Chu 3819* (BM, W); Mt. Omei, *Faber 618* (K); Chengdu, 13 May 1938, *Fang 12359* (A, BM); Chengdu, 18 Jul 1938, *Fang 12462* (BM, W); Chengtu, 20 May 1943, *Fang 19724* (A); Drogochi, vid byn, 22 Sep 1915, *Smith 4552* (MO); Omei Hsien, 7 Jul 1939, *Sun, S.C.* & *Chang 634* (A); Kiating Sichuan, 26 Jul 1938, *Tai 81* (A); sin. loc. [label in Chinese], 14 Dec 1943, *Wang 7567* (E); Mt. Omei, 28 Apr 1932, *Yu 564* (GH); Guan Xian, Jushouhemantan, 18 Jul 1987, *Wang Zhong-tao 87-0016* (A, MO). **Yunnan**: Dali, vicinity of 3-Pagoda Temple area above pagodas, 3 Jul 1995, *Croat 77396* (MO); Gaoligong Shan Region, Gongshan Xian, Binzhongluo Xian along Shuangla He, N of Gongshan on the W side of the Nujiang, 7 Nov 1990, *Dulong Jiang Investigation Team, 241* (CAS); Gaoligong Shan Region. Gongshan Xian, Dulongjiang Xian In the vicinity of Bapo, on the E side of the Dulong Jiang, 14 Nov 1990, *Dulong Jiang Investigation Team, 287* (CAS); Gaoligong Shan Region. Gongshan Xian, Dulongjiang Xian In the vicinity of Maku, southern region of the Dulong Jiang valley on the W side of the Dulong Jiang, 14 Dec 1990, *Dulong Jiang Investigation Team, 1022* (CAS); N flank of Haba Snow range, 14 Sep 1939, *Feng 2350* (A);Chungtien, Zer-i on banks of Yangtze, 18 Nov 1939, *Feng 3391* (A); Tali valley, 25 04 N, May 1906, *Forrest 4479* (K); Tengyueh, Jun 1912, *Forrest 8373* (K); Gaoligong

Shan Region. Gongshan Xian, Binzhongluo Xian Vicinity of Shimenguan (Stone Gate), N of Bingzhongluo along the W side of the Nu Jian E side of Gaoligong Shan. E facing 30-60 degree slope, 2 Oct 2002, *Gaoligong Shan Biodiversity Survey 15695* (CAS); Gaoligong Shan Region. Longyang Qu, Lujiang Xian Along the W side of the Nu Jiang on the E side of the Gaoligong Shan. E facing 0-10º slope, 20 Aug 2003, *Gaoligong Shan Biodiversity Survey 17210* (CAS); Gaoligong Shan Region. Longyang Qu, Lujiang Xian Nr Dongfeng bridge across the Nu Jiang on the E side of the river on the old rd from Baoshan to Tengchong via Dahaopin e side of Gaoligong Shan. E facing 0-10º slope, 30 Aug 2003, *Gaoligong Shan Biodiversity Survey 18169* (CAS); Fugong Xian, Laoqiao Xiang, Qiaotou Cun, directly opposite the S end of Fugong city, on the W side of the Nujiang, 22 Apr 2004, *Gaoligong Shan Biodiversity Survey 19296* (BM, E); Chen kang, Ai-fang, 16 Aug 1938, *Yu 17345* (A); Fugong, Mujiajia village, on the W side of the Nujiang, c. 6.5 direct km S of the border with Gongshan Xian, 28 Apr 2004, *Gaoligong Shan Biotic Survey Expedition Autumn 19656* (E); Fugong Xian, Shangpa Xiang, vicinity of Shili village, W side of the Nujiang, c. 5.7 direct km N of Fugong city, 9 May 2004, *Gaoligong Shan Biodiversity Survey 21066* (BM, E, GH); Gaoligong Shan Region. Gongshan Zizhixian, Dulongjiang Xian E side of the Dulong Jiang in mountain behind Longyuan village, Bailai Cun c. 7.5 direct km S of Dizhengdang (Lengdang) and c. 15 direct km N of Kongdan SW facing 10-30º slope, 1 Nov 2004, *Gaoligong Shan Biodiversity Survey 21646* (CAS, E); Gaoligong Shan region, Gongshan Zizhixian, Binzhongluo Xian W side of the Nujiang between Gongshan and Binzhongluo c. 1 km from Suangla village, 9 Nov 2004, *Gaoligong Shan Biodiversity Survey 22185* (CAS); Gaoligong Shan Region, Gongshan Zizhixian, Binzhongluo Xian E side of Gaoligong Shan NE of Bingzhongluo, 9 Nov 2004, *Gaoligong Shan Biodiversity Survey 22260* (CAS); Gaoligong Shan Region, Gongshan Zizhixian, Binzhongluo Xian S of the W end of the bridge across the Nujiang c. 5 km N of the Stone Gate between Sijitong and Qiunatong villages, 11 Nov 2004, *Gaoligong Shan Biodiversity Survey 22490* (CAS); Lushui Xian, Pianma Xiang, vicinity of Ganheluo, c. 8.5 km N of Pianma on the rd to Gangfang, W side of Gaoligong Shan, 13 May 2005, *Gaoligong Shan Biodiversity Survey 22849* (BM, E, GH, MO); Gongshan Zizhixian, Cikai Zheng, E side of Gaoligong Shan on the rd fro mGongshan to Kongdang, 12 Nov 2004, *Gaoligong Shan Biodiversity Survey 23095* (BM); Lushui Xian, Pianma Xiang, Xiao Pinama, Wanping village, NW of Pianma on the rd to Gangfang nr the China-Myanmar border, W side of Gaoligong Shan, 19 May 2005, *Gaoligong Shan Biodiversity Survey 23409* (BM, E, MO); Gaoligong Shan Region, Fugong Xian, Maji Xian Mujiajia Cun, in the vicinity of Waluodong bridge, just above the Nujiang, E side of Gaoligong Shan, 5 Aug 2005, *Gaoligong Shan Biodiversity Survey 25609* (CAS); Gaoligong Shan Region, Lushui Xian, Luobenzhuo Xian E'ga Cun, on forest rd at km 30, E side of Gaoligong Shan, 9 Aug 2005, *Gaoligong Shan Biodiversity Survey 25832* (CAS); Gaoligong Shan Region. Fugong Xian, Lishadi Xian Ziguduo Cun, just S of Ziguduo village nr the Nujiang, E side of Gaoligong Shan, 13 Aug 2005, *Gaoligong Shan Biodiversity Survey 27451* (CAS); Gaoligong Shan Region, Gongshan, Dulongjian along trail toward Qinglan Dang, W side of the Dulong Jiang valley, c. 0.3 direct km SW of Maku and c. 4.2 direct km NE of the Myanmar border, 18 Aug 2006, *Gaoligong Shan Biodiversity Survey 32463* (CAS, GH); Gaoligong Shan Region, Gongshan, Cikai, vicinity of Yimaluo Cun, on the W side of the Danzhu He, c. 1.4 direct km SW of Danzhu Cun and c. 10.8 direct km SSW of Gongshan, E side of Gaoligong Shan, 14 Aug 2006, *Gaoligong Shan Biodiversity Survey 33280* (CAS); Gaoligong Shan Region, Gongshan, Cikai, Heiwadi Cun in the vicinity of Dimupo on the rd from Gongshan to Kongdang, c. 6.4 direct km WNW of Gongshan, E side of Gaoligong Shan, 27 Aug 2006, *Gaoligong Shan Biodiversity Survey 34191* (CAS); Lushui Xian, Xingangfang, along the Gulang Jiang which froms the border between China and Myanmar, between Pianma and Gangfang, N of Pianma on W side of Gaoligong Shan, 13 Oct 1998, *Heng et al. 10342* (A); Mi Lo Distr., *Henry 9870* (A, K, MO); Kunming, Western Hills, 18 Oct 2005, *Knapp,* & *Monro 9838* (BM, GH, MO); Lushui Xian, Xinigangfang, along the Gulang Jiang which forms the border between China and Myanmar, g between Pianma and Ganfang, N of Pianma on the W side of Gaoligonshan, 13 Oct 1998, *Li et al. 10342* (E); Gaoligong Shan region, Gongshan Xian, Pengdang Xian Along the W side of the Nu Jiang, N of Gongshan at Jimudeng, 5 Jul 2000, *Li et al. 12853* (CAS, E, MO); Gaoligong Shan Region. Gongshan Drungzu Nuzu Zizhixian, Cikai Zhen Heiwadi, E side of Gaoligong Shan, along the Pula He on the new rd to Dulong Jiang valley, 17 May 2001, *Li et al. 13913* (CAS); Gaoligong Shan region. Gongshan Drungzu Nuzu Zizhixian, Dulongjiang Xian Kongdang, W side of Gaoligong Shan, along the new rd from Kongdang to Gongshan County, at about the 80 km place to Gongshan,

24 Jul 2002, *Li et al. 15251* (CAS); Dongchuan, vers Tong-tchouan, *Maire s.n.* (BM); Dongchuan, plaines de Tong-tchouan, Jul 1912, *Maire s.n.* (E); Dongchuan, plaines de Tong-tchouan, Sep 1912, *Maire s.n.* (E); Dongchuan, plaines de Tong-tchouan, Aug 1912, *Maire s.n.* (E); Dongchuan, plaines de Tong-tchouan, Jul 1912, *Maire s.n.* (E); plaine de Tong-tchouau, Aug 1912, *Maire s.n.* (W); plaine de Tong-tchouau, Jul 1912, *Maire s.n.* (W); plaine du Tong-tschouan, Aug 1912, *Maire s.n.* (W); Dongchuan, *Maire s.n.* (K); plaines de Tong-tchouan, Jul 1913, *Maire 78* (BM, E); plaines de Tong-Tchuan, Aug 1914, *Maire 409* (BM, E); Kunming, c. 200 m below the Tomb of Nie Er, West Mountain, 28 Aug 1988, *Qisheng Ma 88-0804* (MO); Yunnan Fu, 4 May 1916, *Schoch 67* (K); Beyendjing, 5 Nov 1915, *Ten 12* (W); Cheng-hsiung Hsien, 21 Jun 1932, *Tsai 52294* (GH); Yung-jen Hsien, 13 May 1933, *Tsai 52833* (GH); Lu-feng Hsien, 28 Jun 1933, *Tsai 53616* (GH); Shang-pa Hsien, 18 Sep 1933, *Tsai 54308* (GH); Lan-ping Hsien, 9 Sep 1933, *Tsai 56071* (GH); Shang-pa Hsien, 20 Sep 1933, *Tsai 56549* (GH); Wei-se Hsien, 19 Sep 1934, *Tsai 57961* (GH); Chee-tee-lo, 11 Sep 1934, *Tsai 58490* (GH); Shang-pa, 20 Oct 1934, *Tsai 58839* (GH); Shang-pa, 29 Oct 1934, *Tsai 59074* (GH); Ping-pien Hsien, 29 Jun 1934, *Tsai 60501* (A); Ping-pien Hsien, 31 May 1934, *Tsai 62021* (GH); Wei-se Hsien, 4 Nov 1934, *Tsai 63005* (GH); Kunming, Kun-Ming, Apr 1936, *Wang 62905* (A); Lung pan la Champu tung, Oct 1935, *Wang 67054* (A); Huann-fu-ping, A tun-tze, Sep 1935, *Wang 69255* (A); Shung-Kiang Hsein, Apr 1936, *Wang 73077* (A); Fo-hai, Jun 1936, *Wang 74462* (A); sin. loc, 1937, *Yu 8530* (A); sin. loc, 1937, *Yu 13333* (A).

CROATIA. **Istria**: Pula, Zeitplatz, 27 Jul 1970, *Schwabe s.n.* (B).

CYPRUS. **Páfos**: Paphos, in a flower bed nr the harbour, 30 Oct 1981, *Meikle 5048* (K); c. 6 km Paphos an der Küste, 22 Sep 2003, *Vitek 03-1710* (W).

CZECH REPUBLIC. Flora Bohemiae et Moraviae, Mähr., Aug 1911, *Petrak 676* (BM, E). **South Moravia**: Moravia centr. -merid., distr. Brno, 23 Aug 1966, *Vicherek 1554* (H, K).

DENMARK. **Hovedstaden**: Københavns, Gentofte, 10 Jul 1941, *Dahl 45a.* (BM). **Midtjylland**: Skæring, N of Aarhus, 1 Oct 1964, *Larsen* & *Pedersen 67* (BM, W). **Sjaelland**: Sjælland: Vemmetofte Strand, 14 Aug 1843, *Dahl 39* (BM); Moseby, 29 Sep 1968, *Ernstsen s.n.* (BM); Jiderup field DY2, Jul 1967, *Hjorih-Olsen s.n.* (BM); Jylderup station. DY2, Sep 1960, *Hjorih-Olsen s.n.* (BM); Jyderu D. Y2, Jul 1969, *Hjorth* & *Olsen s.n.* (BM); Seeland, zwischen Kopenhagen und Roskilde, Torslunde 5 km SW Tåstrup, Toftegård, 23 Jul 1966, *Karl s.n.* (W); Zealand, Amager Faelled, Copenhagen, 9 Sep 1970, *Svendson* & *Vollesen 400* (W).

EGYPT. Nile Valley, Feb 1877, *Alcott s.n.* (GH); between Mafaloch and Sinot Upper Egypt, 7 Dec 1856, *Bromfield s.n.* (K); sin. loc, 1851, *Bromfield s.n.* (K); Vallis Fatme, *Fischer 26* (BM, K, W); Upper Egypt, *Fox* & *Fox s.n.* (K); Assinto, Jan 1898, *Marchesetti s.n.* (W); Ferayg, 27 Feb 1851, *Marsh s.n.* (MO); Nilufer zwischen Cairo und Keneh Farnkuut, Dec 1864, *Schweinfurth 1404* (K); Wady Halfa, Feb 1910, *Scott-Elliot 3354* (BM); Misqa Farm, Ekiad, 5 Mar 1928, *Simpson 5781* (K). **Alexandria**: Bahig, 4 May 1976, *Ahmed* & *Samaan s.n.* (UT); Ikingi-Mariut, 7 Sep 1975, *Amin s.n.* (MO). **Aswan**: Aswan, on a Nile bank, 26 Jan 1959, *Boulos s.n.* (K); Assouan, a environ 1 km en aval du barrage, 21 Dec 2011, *Delnatte* & *Gallay 2622* (BM); Kom Ombo U.E., Dec, *Fox s.n.* (K); Aswan, *De Montbret s.n.* (W); Sardar Island, 15 Feb 1927, *Simpson 4460* (K). **Asyut**: zwischen Cairo und Keneh, 15 May 1864, *Schweinfurth 1411* (W); Beni Hasan, Expedition to the Souce of the Nile, 1860, *Speke* & *Grant s.n.* (K). **Beheira**: Rosetta, Nile Delta, El-Geddia village, 20 May 2003, *Boulos 20191* (K). **Cairo**: Helwan, 23 Dec 1927, *Simpson 5663* (K). **Faiyum**: Abouska, Ibshwai Distr., 10 Apr 1982, *El Ghani 3633* (K); Tersa, Sinnuris Distr., 6 Nov 1982, *El Ghani 4339* (K); Kom Aushim, Tamiya Distr., 12 Nov 1982, *El Ghani 4517* (K); Manshat Sinnuris, Sinnuris Distr., 5 Jan 1983, *El Ghani 5151* (K); Kom Aushim, Tamiya Distr., 17 Jan 1984, *El Ghani 6982* (K); nr Kom Aushim, 17 Jan 1984, *Hepper 7662* (K); nr Hawara village, 17 Jan 1984, *Hepper 7696* (K). **Giza**: Al Gedida village farms, Bahariya Oasis, Western Desert, 27 Nov 1978, *El Ghani 1195* (K); Bahariya Oasis, Western Desert, Airport well, 22 Jan 1979, *El Ghani 1453* (K); Al Zabw, Bahariya Oasis, Western Desert, 23 Feb 1979, *El Ghani 1653* (K); Giza W, Cairo environs, 16 Nov 1930, *Shabetai 190* (K); Giza, Faculty of Agriculture, 13 Jun 1971, *Sisi s.n.* (A); Ad Doqi, Agric. Museum's garden; Dokki, 25 Nov 1956, *Without Collector s.n.* (W). **New Valley**: Ain Sheminada, Forfra Oasis, Western Desert, 4 Apr 1984, *El Ghani 7106* (K). **Qalyubia**: Tukh, Nile Delta, c. 30 km N of Cairo, 8 Oct 1999, *Boulos 19046* (K).

ESTONIA. Dorpat, Sep 1860, *Gruner s.n.* (BM).

FINLAND. **Southwest Finland**: Regio Aboensis, par. Nagu, Ernholm, in horto, 28 Jul 1913, *Dahl 905* (K). **Uusimaa**: Helsinki, Helsingfors, hortus botanicus, 5 Sep 1961, *Nordström s.n.* (E).

FRANCE. St. Denis, *Fraser s.n.* (E); Montagne, au Sobrieux, Vendú, 21 Sep 1818, *Genevier s.n.* (BM); Royan (Charente-Infer.), sables du Parc, 21 Aug 1890, *Guillon s.n.* (BM); Brue, Valle' Oise, 21 Jun 1885, *Mouillefarine s.n.* (BM). **Alsace**: Frankreic Dept, Ht. Rhin, in Issenheim, 25 Oct 1965, *Aellen* & *Baumgartner 18* (K). **Auvergne-Rhône-Alpes**: Haute-Savoie, Bonneville, *Depierre s.n.* (BM); Ardèche, Ardance: le long de la N 82, 19 Sep 1968, *Lawalrée 15503* (BM); Grenoble, Domène, Aug 1869, *Verlot s.n.* (BM). **Bourgogne-Franche-Comté**: Dole, Jura, 25 Sep 1865, *Billot 22* (BM). **Bretagne**: Redon, 11 Sep 1902, *Drouilhet s.n.* (BM). **Centre-Val de Loire**: Bourges, place Séraucourt, terres fraîchement renneés, 25 Oct 1874, *Ripart 249* (BM). **Corse**: La Chiappa, östl. von Porto Vecchio Garten, 25 Jul 1973, *Bocquet 15520* (BM); 2 km au sud de Folelli, 27 Jul 1976, *Bocquet 16910* (BM); Haute-Corse, Aléria, cote orientale, plage du Padulone, entre le Tavifnano et l'etang de Diane, 1 Dec 1986, *Lambinon* & *Rouselle 86/354* (B). **Grand Est**: Alsace, Issenheim, Haut-Rhin, 1960, *Aellen 8* (BM); Haut-Rhin, Issenheim, 25 Oct 1965, *Aellen 16* (E); Alsace, Elsass, Gebwiler, auf einem mit Baumwolle gedungten Acker, 9 Nov 1959, *Aellen 63975* (K); Haut-Rhin, Issenheim, Baumwollkompost, 11 Oct 1969, *Aellen* & *Baumgartner s.n.* (BM, W); Elsass, Wissemburg, Schutt am Aau Unger, 16 Oct 1964, *Neumann s.n.* (W); Haut Rhin, Ensisheim, 2 Sep 1961, *Rechinger s.n.* (W). **Hauts-de-France**: Pas-de-Calais, Calais, 31 Jul 1893, *Without Collector s.n.* (E). **Île-de-France**: Seine-Saint-Denis, Montreuil, Belfroi, 11 Oct 1963, *Boreau s.n.* (BM); Île de France, *Duret s.n.* (BM); Paris, Paris, Jardin de l'Ecole, Jul 1906, *Thuret 334* (BM);. **Normandie**: Calvados, E edge of Luc-sur-Mer on the Route de Lyon, 8 Aug 2010, *Atchison IM-9* (BM). **Nouvelle Aquitaine**: Creuse, Saint Avit de Tardes, 10 Sep 1975, *Lugagne 7256* (BM, H); Dordogne, St Hilaire des Dordogne, a du Roche Choton, 1 Sep 1854, *Genevier s.n.* (BM). **Occitanie**: Aude, La Nouvelle, 6 Aug 1909, *Gillot s.n.* (BM); Gard, Champs, au Vigan (Gard), Aug 1866, *Billot s.n.* (BM); Gard, Le Vigan, 6 Sep 1874, *Herb. Heribaud s.n.* (BM); Pyrénées-Orientales, Sorède, 20 May 1894, *Castanier s.n.* (BM). **Pays de la Loire**: Loire-Atlantique, Trentemoult, 29 Aug 1878, *Genevier s.n.* (BM); Maine-et-Loire, Augen, 1 Sep 1851, *Genevier s.n.* (BM); Loire-Atlantique, Trentemoult, 19 Sep 1879, *Herb. Gadeceau s.n.* (BM); Chantenay, 6 Jul 1874, *Herb. Gadeceau s.n.* (BM); Loire-Atlantique, Nantes, Indret, Aug 1893, *Herb. Gadeceau s.n.* (BM). **Provence-Alpes-Côte d'Azur**: Hautes-Alpes, Serres, du Vigan (Yard), 19 Aug 1876, *Anthouard s.n.* (BM); Alpes-Maritimes, Cipieres commune, cultivated field, formerly oats, sainfoin, wheat E55 to S of village, 7 Sep 1993, *Hepper 9760* (K).

GEORGIA. Lechkhkhumi Distr., vill. Alpana area, Ladjanuri Gorge, 16 Sep 2006, *Khutsishvili* & *Chiboshvili 172* (W); Lasistan, Caucasus, 1885, *Massalsky s.n.* (K); Caucasus, Bayern, *Massalsky s.n.* (K). **Adjara AR**: Shuakhevi Distr., along Georgia Highway A306 (main rd between Batumi and Akhaltsikhe), c. 40.3 km (by air) E of Batumi city center, c. 15 km (by air) N of Turkish border, 15 Sep 2007, *Atha et al. 5835* (W); Adjara, Ajara Autonomous Repulbic, Batumi, Msvane Kontshi (Green Cape), 7 Aug 2012, *Kobakhidze 1009* (K); Adzharia, Distr. Chelvascauri [Kelvachuari], p[ope] Machindzhauri, 9 Sep 1990, *Mazurenko s.n.* (W). **Kakheti**: Kakheti, between vil. Kardenakhi and Anaga, 18 Sep 2003, *Khutsishvili* & *Lachashvili 7* (MO); Akhmota Distr., Ilto gorge, environs of village of Sabue, 10 Apr 2007, *Lachashvili* & *Khutsishvili 1050* (W).

GERMANY. **Baden-Württemberg**: Carlsruhe (grand-duché de Bade), 4 Oct 1856, *Billot s.n.* (BM); Heidenheim afssreuz, Wurttemberg, Aug 1908, *Meebold s.n.* (K); Freiburg-Baden, Aug 1920, *Meebold s.n.* (K). **Bayern**: Wällen u, Wegen in der Nähe der Dörfen, *Without Collector s.n.* (BM). **Berlin**: St. Kelitz, *Ball s.n.* (E); Berlin, edge of Grünewald in W of city, along River Havel (Wannsee) N of Grüunewaldturm (Kaiser-Wilhelm-Turm), 8 Nov 2008, *Knapp IM-10164* (BM); Berlin, edge of Grünewald in W of city, along River Havel (Wannsee) N of Grüunewaldturm (Kaiser-Wilhelm-Turm), 8 Nov 2008, *Knapp IM-10165* (BM); Berlin, Alexanderplatz, 9 Nov 2008, *Knapp IM-10166* (BM); along Walltostrasse, W Berlin, 10 Nov 2008, *Knapp IM-10167* (BM). **Hamburg**: Hamburg, Aug 1891, *Schinz s.n.* (W). **Hessen**: Starkenburg, 4 Sep 1924, *Herb. Heribaud s.n.* (BM). **Nordrhein-Westfalen**: Rhede, *Harting s.n.* (BM); Cologne, Max-Planck-Institut fur Züchtungsforschung (MPIZ), in experimental garden, 15 Oct 2008, *Knapp IM-10162* (BM); Cologne, Max-Planck-Institut fur Züchtungsforschung (MPIZ), in experimental garden, 15 Oct 2008, *Knapp IM-10163* (BM). **Sachsen**: Marienau, Flora Silesiae centralis, 4 Oct 1882, *von Uechtritz s.n.* (BM); Leipzig, Aug 1846, *Herb. Auerswald s.n.* (BM). **Schleswig-Holstein**: Eckernförde, Karlsminde, 28 Aug 1974, *Nielsen et*

al. 230 (BM, H, W). **Thuringia**: Acker am Wartenberg bei Eisenach, 25 Aug 1950, *Launert s.n.* (BM);

GREECE. **Attica**: pr. Marathon, 12 Jun 1930, *Guiol 1564* (BM); Menidi, Sep 1930, *Guiol 1602* (BM). **Central Greece**: Insula Euboea septentrionalis, prope Asimion (Potoki), 29 Sep 1958, *Rechinger 19350* (W). **Crete**: Zaros, 14 Aug 1971, *Jermy* & *Brownsey 9070* (BM); Chania, Gerani, 22 Aug 1971, *Jermy* & *Brownsey 9239* (BM). **East Macedonia and Thrace**: Thrakien, Berg Kallidhea, im Boukate Dagh, 10 km N Esimi, Nomos Evrou, 19 Sep 1966, *Bauer* & *Spitzenberger 1108* (W). **Epirus**: Pindus M5, Kerasoron village rubbish dump, 31 Jul 1961, *Cambridge University Expedition 91* (K). **Ionian Islands**: Pirgi, Corfu, Aug 1962, *Bally 12482* (K); Kerkira, Insel Kérkira, an der Strasse von Kondokáli nach Temblóni, c. 1.4 km W-WSW Kondokáli, 2 Nov 1991, *Hörandl* & *Gutermann 26365* (W); insula Kerkira/Korfu in oppido Kerkira, 1 Jul 1995, *Vašák s.n.* (W); Corfu, Paleokastrítsa und nähere Umbegung, 1 Apr 1985, *Vitek s.n.* (W). **North Aegean**: Chios, The Kampos, 15 Feb 1940, *Platt 397* (K); Chios, 31 May 1940, *Platt 512* (K); Insula Samos, inter Hagios Konstantinos et Nenedes, 23 Sep 1978, *Rechinger 61377* (W); Insula Samos, Hagios Konstantinos, 23 Sep 1978, *Rechinger 61404* (W). **Peleponnese**: Messenia, Methoni, in ruinis casteli, 11 May 1964, *Rechinger 24868* (W). **South Aegean**: East Aegean Islands Leros, Agia Marina, 2 May 1988, *Burton 88.133* (BM); Prov. Cambia Deka, 1914, *Gandoger 282* (MO). **Thessaly**: The Kampos, 15 Feb 1940, *Platt 397* (K); The Kampos, 31 May 1940, *Platt 512* (K); Thessalia, Plaka 19 km a Katerini meridiem versus, 9 Sep 1969, *Rechinger 37815* (W); Thessalia, Plaka 19 km a Katerini meridiem versus, 9 Sep 1969, *Rechinger 38210* (W). **West Greece**: Achaea, Mt. Kyllene, 1930, *Guiol s.n.* (BM). **Western Macedonia**: Macedonia Ocidentalis, Stena Portas prope Servia, 20 Aug 1970, *Rechinger 39047* (W).

HUNGARY. Bur, 18 Jul 1954, *Krzisch s.n.* (W). **Bács-Kiskun**: Kalocsa, Donau-Ufer bei Taks Schutt, Aug 1876, *Menyhárth s.n.* (W).

INDIA. sin. loc, *Beddome s.n.* (BM);sin. loc, *Buchanan-Hamilton s.n.* (BM);, *Carey, Ws.n.* (K); sin. loc., *East India Compan, s.n.* (K);Bengal, *Hooker & Thomson s.n.* (A, BM);Kashmir, Oct 1848, *Hooker & Thomson s.n.* (K); plains of India, *Royle s.n.* (K); Maisor & Carnatic, *Thomson s.n.* (BM, E); sin. loc, Mar 1888, *Wallich s.n.* (BM); sin. loc, *Wallich 2615* (BM); sin.. loc., *Wallich 2615* (K); Peninsular Indorientalis, *Wight s.n.* (BM);Peninsula Indiae Orientalis, *Wight 2010* (GH); Kashmir, Oct 1848, *Without Collector s.n.* (K); **Andhra Pradesh**: Visakhapatnam Distr., East of I bungalow-Annantagiri, 13 May 1964, *Subbarao 19565* (CAL); **Arunachal Pradesh**: Manipur, Ukhrul, 13 Mar 1948, *Kingdon-Ward 17085* (A, BM, MO); Kameng F.D. (NEFA), Dirang Dzong nr APOs quarters, 16 May 1957, *Rao 7454* (CAL); Kameng F.D. (NEFA), Zang Camp to Tawang, 16 May 1957, *Rao 7857* (CAL); **Assam**: Sanheti, Mar 1902, *Chatterjee s.n.* (GH); Assam, Di Chu, 27 Jun 1950, *Kingdon-Ward 20022* (BM); on way back to Chardwar [tea estate?], 20 Feb 1957, *Panigrahi 5778* (CAL); Jalabosty, nr Joeckgat, Feb 1999, *Prain's collector 745* (A); Subansiri P.D. (NEFA), Palin to Nyapin, ca. 0.5 miles from Palin, 15 Nov 1964, *Sastry 40638* (L); **Bihar**: , Bhagalpur, 11 Feb 1967, *Paul s.n.* (CAL); Gobardhana, Champaran, 17 Nov 1963, *Shetty 384* (CAL); Udaipur, Udaiput forest in plantation, Champaran, 8 Apr 1963, *Thothathri 9960* (CAL); nr the Kabar Lake, 16 Dec 1983, *Without Collector CNH-14266* (CAL); **Chandigarh**: NW India, Punjab. nr Chandighar, 20 Mar 1885, *Nanak s.n.* (K); **Chhattisgarh**: Bastar, nr Chhotedongar [Chhota Dongar], Abujh-Marh, 26 May 1983, *Roy CC-34072* (CAL); **Delhi**:Delhi, University Ridge, 10 Feb 1960, *Culhati, S.C13* (UT); New Delhi Ridge, 2 Feb 1956, *Singh s.n.* (F); **Gujarat**: Chitral, Kaghosi, 13 May 1958, *Without Collector 2416* (BM); **Haryana**: Hisar, in dictione Lirsa (Hissar), 3 Feb 1886, *Drummond 2293* (K); Karnal, Baldhi, 16 May 1886, *Drummond 25857* (K); Khadzal, Hissar (Punjab) [Hisar], 16 May 1886, *Drummond 25868* (K); Sonti River forest, 24 Mar 1998, *Kumar NC-93367* (BSD); nr Kalaser [Kalesar] temple side, 11 Dec 1997, *Kumar NC-101615* (BSD); Jind, 29 Jul 1961, *Nair NC-16121* (BSD, CAL); Sirsa, Oto bridge, 2 Dec 1961, *Nair NC-18954* (BSD, CAL); Balman wan, Rohtok Rd, 12 Aug 1962, *Nair NC-23125* (BSD); Jind, 16 Oct 1962, *Nair NC-24764* (BSD); Hisar, Mojukhera, 18 Feb 1963, *Nair NC-26044* (BSD, CAL); Gurgaon, 21 Dec 1959, *Rao NC-11009* (BSD, CAL); **Himachal Pradesh**: Lahul, Tandi, 6 Aug 1971, *Bhattacharyya NC-45161* (BSD, CAL); Shimla, infra Simla, 1884, *Drummond 1872* (K); Chamba, Kulal, 3 Sep 1896, *Gammie 18462* (DD, K); Kinnaur, nr. Rarang, 27 Aug 1890, *Lace 580* (CAL); Chamba, nr Dhár, Mar 1885, *Nanak s.n.* (K); Sutlej River area, 18 Feb 1985, *Srivastava NC-73736* (BSD); Shimla, Nov 1885, *Without Collector s.n.* (CAL); **Jammu & Kashmir**: Jul 1856, *von Schlagintweit s.n.* (GH); environs of Srinagar, Srinagger, within a circle of 8 miles radius, 10 Aug 1856, *von Schlagintweit s.n.* (BM); Srinagar, environs of Srinagger,

within a circle of 8 miles radius, Sep 1856, *Von Schlagintweit 4368* (GH); Jammu, R.O.L. Campus, 13 Aug 1994, *Singh NC-88573* (BSD); Ganderbal, Manas Bal [=Manasbal Lake], 16 Sep 1986, *Wadhura & Murti NC-83715* (BSD); **Jharkhand**: Hazaribagh, Chota Nagpur, Gonda river bed, 2 Mar 1952, *Kerr s.n.* (BM); **Karnataka**: prope Bettigherry, terr. Canara, *Hohenacker 768* (BM); **Kerala**: Travancore, Stable dunes, 4 miles south of Trivandrum, 7 Dec 1933, *Erlanson 5070* (A); **Madhya Pradesh**: Rampur, Chalgali, Pratappur, 25 Nov 1999, *Kumar 731* (DD); Bheda Ghat, Jabbalpur Distr., 17 Jan 1961, *Maheshwari 4635* (CAL); Gwalior, 1890, *Maries 191* (BM, CAL); Gwalior, 1890, *Maries 249* (BM, CAL); Dhupgarh, Harshangabad, 20 Aug 1949, *Narayanaswami 3339* (CAL); Sidhi, Maphali, 17 Jan 1964, *Panigrahi CC-2132* (CAL); Sidhi, Chitrangi, 23 Feb 1971, *Sengupta CC-14548* (CAL); Tikamgarh, Orcha Kila, 15 Sep 1980, *Verma CC-30761* (CAL); **Maharashtra**: Mumbai[Bombay], Mumbai, *Dalzell s.n.* (K); Ram Ghat [Ujjian], Jun, *Ritchie 508 /2* (K); Mumbai[Bombay], Bombay Presidency, Southern Maratha country and north Canara, 3 Jan 1882, *Young s.n.* (BM); **Meghalaya**: , Cherrapunji, [=Sohra], Khasia Hills, 1 Jun 1911, *Burkill & Banerjee 190* (CAL); Khasi hills, Khasia, Dorpu, 1930, *Dhwoj 51* (BM); **Nagaland**: Khonoma, 13 May 1886, *Prain s.n.* (CAL); **Odisha**: Sundargarh, Koira, 12 Oct 1987, *Mukherjee & Namhata MN-2462* (CAL); **Punjab**: Punjab, nr Chandighar, 20 Mar 1885, *Drummond 20551* (BM); Jammu Agar, 6 May 1963, *Nair NC-27506* (BSD); Firozpur, Firozpur, 14 Mar 1961, *Nair NC-36383* (BSD); Rawalpindi, 25 Apr 1939, *Stewart 19404A* (GH);Punjab, *Thomson s.n.* (BM); **Rajasthan**: Pali, by the side of Kharda Dam, 29 Nov 1972, *Moorthy, SAC-341* (CAL); Research station nursery, Jodhpur, 4 Feb 1957, *Nantinjal 25421* (DD); Bharatput, Ghana Bird Sanctuary [=Keoladeo National Park], 22 Mar 1982, *Parmar AC-8623* (CAL); Ganganagar, Raisinghnagar, 12 Nov 1976, *Roy AC-3863* (CAL); Ghati village, Ronk Dt, 17 Feb 1973, *Shetty AC-566* (CAL); Balotra-Barmer rdside, 17 Oct 1975, *Shetty AC-2214* (CAL); Jaisalmer, Amar Sagar, 16 Nov 1973, *Tiwari AC-836* (CAL); Jhalawar, 16 Sep 1964, *Wadwha & Verma CC-7495* (CAL); Jaisalmer, 4 Sep 1964, *Walhwa CC-5211* (CAL); **Rajasthan**: Banswara, Danpur Forest Rest House, 22 Aug 1976, *Singh 2988* (K); **Sikkim**: Sinchul, Jul 1862, *Anderson 1018* (CAL); Kalimpong, 7 Jul 1862, *Anderson 1027* (CAL); West Sikkim, on way to Melli, 14 Jun 1988, *Basu BSHC-8481* (BSHC); East Sikkim, hills above Chandwari nr 2 miles, 29 May 1982, *Chakraborty BSHC-2159* (BSHC); Sikkim, Selim, 12 Oct 1884, *Clarke 36740* (BM); Yoksam [Yuksom]-Bakkim, 18 May 1960, *Hara et al. 15412* (K); sin. loc, 13 Feb 1876, *King s.n.* (CAL);sin. loc, 1878, *King s.n.* (CAL); East Sikkim, lower Burtuk Barty, 28 Feb 1980, *Krishna BSHC-851* (BSHC); North Sikkim, Bonsoi, 7 Dec 2002, *Maity & Pradhan BSHC-25627* (BSHC); North Sikkim, Bonsoi, 7 Dec 2002, *Maity & Mandal BSHC-25657* (BSHC); North Sikkim, on the way from Bey to Tingbong, 14 Aug 1989, *Mandal BSHC-10183* (BSHC); North Sikkim, on way from Chungthang-Lachen, 16 Aug 1989, *Mandal BSHC-10441* (BSHC); West Sikkim, Karchi R.F, 29 Oct 1999, *Shukla BSHC-22169* (BSHC); West Sikkim, Karchi Reserve Forest, 15 Dec 1994, *Sinha & Pradhan BSHC-16704* (BSHC); North Sikkim, on way to Bay, 14 Aug 1989, *Srivastava BSHC-10371[b]* (BSHC); South Sikkim, above Phamtamp, 10 May 1991, *Srivastava BSHC-13167* (BSHC); South Sikkim, on way to Yangyang, 22 May 1985, *Verma BSHC-3568* (BSHC); **Tamil Nadu**: Coimbatore, Palni Hills, Chettipalayam, 8km from Podanur, 15 Jan 1994, *Chorley & Bharatan 21* (BM); Chennai, Madras, 1825, *Hooker s.n.* (K); Tiruchi, Pachaimalais, Sobanapuram, 8 Dec 1979, *Matthew 24905* (A); Nilgiri, in montibus Nilagiri, *Wright 1077* (BM); **Uttar Pradesh**: Mahoba, Hanurpur, 3 Apr 1962, *Bhattacharyya NC-21043* (BSD, CAL); Lucknow Bazaar, 25 Nov 1909, *Das 33921* (CAL); Saharanpur, 12 Dec 1906, *Economic Botanist 25214* (CAL); Lakhimpur Kheri, Kathna nadi, 27 Mar 1898, *Ináyat Khan 22443* (DD); Lucknow National Botanic Garden, 1 Apr 1967, *Maheshwari 5614* (A); Lakhimpur Kheri, Mailani, 17 Dec 1960, *Malhotra NC-13359* (BSD, CAL); Mahoba, 22 Jul 1962, *Malhotra NC-22832* (BSD, CAL); Bahraich, Payagpur, 14 Feb 1963, *Malhotra NC-23691* (BSD, CAL); Pilibhit, 13 May 1976, *Malhotra NC-31595* (CAL); Subhdaspur, Pilibhit Distr., 30 Aug 1972, *Malhotra NC-50575* (CAL); Jamna [=Yamuna?] Bridge, 15 Dec 1960, *Malhotra NC-13156* (BSD, CAL); Meerut, Annkerai, 5 Jan 1962, *Nair NC-14732* (BSD, CAL); Mirzapur, Banmohari, outskirts of Khajuri village, 13 Mar 1970, *Panigrahi CC-12610* (CAL); Moradabad, Feb 1843, *Thomson 62* (BM, K); **Uttarakhand**: Dehradun, Rispana, 10 Mar 1962, *Babu NC-34679 a* (BSD); Dehradun, New Forest, 16 Jul 1955, *Balapure 537* (A);Dhami Kholla (Munsiyar [=Munsiari]), 12 Sep 1993, *Boron NC-75048* (BSD); Dehradun, 2 Jul 1930, *Krishnan 30* (GH); Champawat, Lohaghat, *Lindsay s.n.* (E); Garhwal, Fadhakhal, 16 Jun 2014, *Malik 115* (BSD); Dehradun, 22 Oct 1929, *Radzada s.n.* (GH); Kumaon, Gori river, East Alruna, div. Kumaon, 16 May

1933, *Ram 2184* (DD);Horai sal forest, Chakhata range, 2 May 1914, *Shankarmani 164* (DD); Dehradun, 1 Dec 1921, *Singh 109* (A); Kumaon, Sirmosia, *Strachey & Winterbottom 1* (CAL, GH); Bhimtal, Apr 1844, *Thomson 62[d]* (K); **West Bengal**: Balurghat, 9 Apr 1984, *Banerju CNH-16284* (CAL); Uttar Dinajpur, Raigunj, 13 Apr 1984, *Banerju CNH-17468* (CAL); Darjeeling, Sukna [to] Tarai Eastern Himalaya, 20 Mar 1913, *Cave s.n.* (E); Darjeeling, Rungbee, 4 Sep 1869, *Clarke 9011* (BM); Darjeeling, 6 Sep 1875, *Clarke 27399* (K); Kersoeng, Sitong, 11 Dec 1908, *Craib 579* (CAL); Madarihat, Khoyarbari forest, 25 Apr 1959, *Das, C.R112* (CAL); Bhedia, 15 Mar 1965, *Dutt 651* (CAL); Maldah, Old Malda, 19 Nov 1965, *Dutta 50* (CAL); Darjeeling, Rangbil, Jul 1874, *Gamble 3398* (K); Kolkatta[Calcutta], Maidan, 29 Mar 1960, *Hara et al. 1544* (A, BM); Darjeeling, 21 Sep 1977, *Ohashi et al. 77 4285* (A, BM); Kolkatta[Calcutta], Ballingunge College compound [Calcutta University], Mar 1945, *Pal s.n.* (K).

INDONESIA. **Java**: Idjen Plateau, 11 Feb 1930, *Franck 180* (US); Central Java, Surakarta, *Horsfield s.n.* (BM); Central Java, Surakarta, *Horsfield s.n.* (BM); East Java, Waliran, *Zollinger 2177 [bis]* (P); West Java, omgessing Tjibodes (Gede), van de Gegerbentong, 1 May 1950, *van Orststroom 13867* (L). **Lampung**: Gunung Sekkau, nr Liwa, 18 Jan 1998, *Hoover* & *Cognets 30037* (GH). **Papua**: Iliamik Village, vicinity of Ilamik village, Snow Mountains region, E of the Baliem valley, Ka Jayawijaya, Kec. Kurima, Oct 1992, *Milliken 1362* (K). **Sumatra**: Deleng Singkoet, N of Berastagi, Karo Plateau, 24 Jun 1927, *Bartlett 8569* (US); Sin. loc., 1880, *Forbes 2544* (BM, GH, P); Brastagi, East Coast, *Yates 2622* (W). **Timor**: sin. loc, *Forbes 3881* (BM).

IRAN. **Ardabil**: Khusavar, Ardabil re, Caspian coast, 16 Aug 1966, *Crisp 123* (K). **Bushehr**: Bouchyr, *Aucher-Eloy 5030* (BM, P). **Fars**: Hoseinabad, 40 km W of Shiraz, 28 Jun 1964, *Grant 15861* (MO, W). **Ghilan**: Lahidjan, 12 Nov 1949, *Starmuehlner 270* (W); Gilan, im Gebirge südlich von Hastpar bei Asalom (Navrud), 16 Jul 1968, *Steiner* & *Steiner 22* (W). **Gilan**: Sin. loc., 1936, *Lindsay, 1023* (BM, K). **Golestan**: Abr Pass, 18 Aug 1968, *Dobson 283* (K); Abr Pass, 18 Aug 1968, *Dobson 300* (K); Abr Pass, 18 Aug 1968, *Dobson 337* (K). **Hormozgan**: Kish Island, 22 Feb 1893, *Bornmüller 496* (W). **Isfahan**: Tadschrisi, aus samen von den Bazaren in Ispahan gezogen in Wiener Bot. Gast., *Stapf s.n.* (K); **Kerman**: Mahan, Jupar, Kerman, Aug 1950, *Armstrong 98* (K). **Kermanshah**: nr Kangavar, 24 Aug 1963, *Corley 68* (K). **Khuzestan**: Abadan, W Persia, Apr 1927, *Macmillan 219* (K); Abadan, Apr 1927, *Macmillan 220* (K). **Mazandaran**: Golestan National Park, NW Khorasan, N Semnan, c. 4 km SE of Tangereh, Lateh-Khodaqoli, 3 Jul 1995, *Akhani 11571* (W); Golestan National Park, NW Khorasan, N Semnan, Tangereh (off Park), 13 Nov 1996, *Akhani 12247* (W); Golestan National Park, NW Khorasan, N Semnan, SE of Tangerah, Khan-dushan, 13 Nov 1996, *Akhani 12252* (W); 34 km S of Amol, 6 Jul 1969, *Andersen,* & *Pedersen 247* (E, K, W); Chalus Gorge, 25 Jun 1962, *Furse 2821* (E, K, W); Babol, 19 Jun 1948, *Rechinger* & *Rechinger 5640* (W); entre Zirab et Sherga, 6 Dec 1965, *Tregrubov 168* (W); Haraz valley, between Aliabad and Siah Bisheh, 24 Jul 1959, *Wendelbo 1475* (W); by Lavij River c. 5 km S of Nosrat Abad, 29 Aug 1974, *Wendelbo* & *Assadi 14504* (W). **North Khorasan**: Bojnourd, Attrek Valley E of Bojnurd, 16 Aug 1966, *Furse 8932* (K). **Sistan and Baluchestan**: Sistan, 1965, *Ayanzi Edin 5887* (E). **Tehran**: nr Tehran, Apr 1943, *Trott 982 A* (K).

IRAQ. Eskilelek, 8 Oct 1961, *Chakravady* & *Nuri NHI-30765* (K); Chelala nr Amarah, 28 Apr 1934, *Field* & *Lazar 24* (F); Baghdad, Harthiyah, 8 Nov 1932, *Lazar 92* (F). **Al-Anbar**: Al Qaim, 4 Jul 1979, *Omar* & *Hamad NHI-50421* (K). **Al-Basrah**: Sin. loc., Oct 1929, *Guest 304* (K); Basra, 7 Oct 1910, *Handel-Mazzetti 3133* (W); Maqil nr Basra, Basra Liwa, on island of Schatt al Arab, 16 Mar 1957, *Rechinger 8418* (W). **Al-Qadisiyyah**: 15 km S of Diwaniya, Diwaniya Liwa, 25 Oct 1962, *Barkley et al. 3716* (K). **Baghdad**: Baghdad, 1 Feb 1947, *Ahmad NHI-9409* (K); 3 km W of Baghdad, 26 Oct 1977, *Am Sharif et al. NHI-47305* (K); Sin. loc., Mar 1929, *Guest 181* (K); in insula Tigridis infra urbem Baghdad, 23 Apr 1910, *Handel-Mazzetti 920* (W); Abu Ghraib Farm, Baghdad Liwa, 9 Oct 1963, *Hikmat Abbas 616* (K, W); Karadah, Karredeh, Jan 1920, *Parangbye Rustam-38* (K); Daurah on Baghdad-Hilla rd, 27 Oct 1956, *Rechinger 8117* (W). **Maysan**: in nr of Chibba, 10 km SE of Musharrah, 29 Oct 1962, *Al Rawi* & *Khatib NHI-32404* (K); Hor al Hawiza, 15 km E of Calat Salih, 17 May 1977, *Thamer* & *Wedad Hana NHI-46781* (K). **Nineveh**: N of Mosul, 2 Jul 1967, *Anders 1587* (W). **Wasit**: Badra, 19 May 1976, *Al Khaisi* & *Yahya NHI-45296* (K); Badra, 23 Apr 1979, *Al Khaisi* & *Al Khayat NHI-50616* (K); Badra, 11 Feb 1947, *Al Rawi* & *Gillett NHI-5951* (K);

IRELAND. **Munster**: Cork, Ballycotton, Sep 1855, *Herb. Carroll s.n.* (BM).

ISRAEL. Rehovot, Center Distr., Hebrew University greenhouses, 15 Apr 2016, *Knapp IM-10786* (BM); E of Givat Ram, Jerusalem, Judean Mountains, 18 Nov 1981, *Liston* & *Monias s.n.* (E); Lower Galilee, 16 Oct 2008, *Lotan Soni-161008* (K); Upper Jordan Valley, 20 May 2010, *Lotan Soni-200150* (K); Ein Gedi, 1863, *Lowne s.n.* (W); Jerusalem, American Colony, 25 Feb 1903, *Meyers 401* (F); Sharon Plain, 14 Jul 2008, *Singer Soni-40708* (K); Sharon Plain, 8 May 2007, *Singer Soni-80507* (K). **Haifa**: River Kishon, 10 Nov 1910, *Meyers* & *Dinsmore 4102* (E).

ITALY. **Apulia**: Taranto, Leucaspide (Massafra/Statte), 2 Oct 2005, *Mariani s.n.* (BM); Taranto, Leucaspide (Massafra/Statte), 2 Oct 2005, *Mariani s.n.* (BM). **Campania**: ad radices Montis Vesuvii, prope Neapolim, May 1842, *Ball s.n.* (E); circa Poestum, Lucania, May 1842, *Ball s.n.* (E). **Liguria**: St. Margherita Ligure, Riviera di Levante, May 1907, *Birger s.n.* (S). **Lombardy**: Como, Menagio, 9 Jul 1886, *Murray s.n.* (BM). **Puglia**: Taranto, Leucaspide, Massafra/Statte, 2 Oct 2005, *Mariani s.n.* (BM); Taranto, Leucaspide, Massafra/Statte, 2 Oct 2005, *Mariani s.n.* (BM); Taranto, Leucaspide, Massafra/Statte, 2 Oct 2005, *Mariani s.n.* (BM). **Sardinia**: Maddalena: nr Abbatogia NE of Sardinia, 16 Apr 1973, *Humphries* & *Richardson 334* (BM); Laconia, Prope Laconi Sardninæ, Aug 1827, *Müller s.n.* (E). **Sicily**: prov. Trapani, Scopello 7.5 km NW Castellammare del Golfo, bei den Faraglioni di Scopello, 10 Apr 1969, *Karl s.n.* (W); Palermo, Oct 1880, *Lacono s.n.* (E); Palermo, Oct 1880, *Lojacono 215* (BM); Etna Mt., In regionae inferiore montis Aetnae, 24 Jul 1874, *Strobl s.n.* (BM); Palermo, Sep, *Todaro s.n.* (W); Palermo, Aug, *Todaro 875* (BM). **Toscana**: Lucca, 'Agro Lucchese' luogo detto, 1837, *Puccinelli s.n.* (BM). **Trentino Alto-Adige**: Meran, Tyrol, 19 Jul 1888, *Murray s.n.* (BM); Tyrol, ad vias prope Bozen, Sep, *von Hausmann 12 6* (BM). **Valle d'Aosta**: Aosta, 27 Jul 1987, *Pistarino 744* (BM). **Veneto**: loco Lido dicto, 27 Jul 1920, *Béguinot s.n.* (BM); in loco Lido dicto, 27 Jul 1920, *Béguinot s.n.* (BM); Venetia, in agris Zeae Maydis cultis et incultis, loco Lido dicto, solo arenoso, 27 Jul 1920, *Béguinot 2515* (K); Venetia, in cultis et incultis, loco Lido dicto solo arenoso, 27 Jul 1920, *Béguinot 2516* (K); Venetia, Padova, sponte et copiosum in R. Horto Botanico, Jul 1920, *Béguinot 2518* (K); Venezia, in Lido dicto, 27 Jul 1920, *Béguinot 23622* (BM); loco Lido dicto, 27 Jul 1920, *Béguinot 23623* (BM).

JAPAN. Insula Nippon, Aomori, Oct 1904, *Faurie 5979* (BM); Botanical Gardens, Toyko, 18 Jun 1912, *Fox s.n.* (BM); Prov. Kii, 4 Oct 1962, *Furuse 40678* (K); Kyushu Island, Hakozaki Kasuyagun, 25 Oct 1928, *Ichikawa 33* (BM, P); Toyano, Niigata city, 5 Nov 1950, *Ikegami 15792* (MO); Taihoku, 5 Aug 1920, *Ito s.n.* (BM); Hakodate, 1861, *Maximowicz s.n.* (BM, K); plains above Yokahama, 1878, *Milne s.n.* (BM); sin. loc, *Morrow* & *Williams s.n.* (GH); nr Kawabara, 26 Oct 1941, *To Hara 79/41* (K); Todoroki gorge, 15 Sep 1935, *To Hara*, *115/35* (K); sin. loc, 30 Jul 1863, *Without Collector s.n.* (K). **Hokkaido**: Otaru, 8 Aug 1886, *Faurie 1172* (K); Otaru, 6 Sep 1888, *Faurie 3109* (K); Wakkanai-shi, Prov. Kitami, Hokkaido (Yezo), 24 Aug 1975, *Furuse 9548* (K); Hokkaido, Sapporo, Prov. Ishikari, 18 Sep 1889, *Tokukuchi s.n.* (GH). **Honshu**: Kanagama, Yokohama, 21 Jul 1876, *Bisset 272* (E); Kanagama, Yokohama, 5 Sep 1876, *Bisset 733* (E); Kanagama, Yolo, 21 Sep 1876, *Bisset 734* (E); Kanagama, Yokohama, Nov 1885, *Bisset 3559* (E); Yamaguchi, Iwakuni Air Base, Iwakuni City, 26 Oct 1953, *Charette 1509* (MO); Chiba, Hebara, Katsuura-shi, along the R 128, 17 Oct 1980, *Deguchi 5408* (A); Tokyo, 26 Aug 1890, *Faurie 6720* (K); Shibaura Tokyo, Prov. Musashi, Hondo, 23 Sep 1962, *Furuse s.n.* (A); Hondo (Prov. Idzu), 27 Oct 1953, *Furuse 26892* (K); Prov. Kazusa, Hondo, 12 Sep 1962, *Furuse 40522* (K); Prov. Kii, Hondo, 4 Oct 1962, *Furuse 40677* (K); Hondo (Prov. Kadsuza), 18 Oct 1964, *Furuse 42704* (K); Hondo (Prov. Sagami), 27 Sep 1966, *Furuse 44637* (K); Hondo (Prov. Awa), 31 Oct 1967, *Furuse 46168* (K); Chiba, Inuboo zaki, Chyooshi-shi, Prov. Shimofusa, 21 Oct 1959, *Furuse s.n.* (A); Northern Honshu, Miyagi, Sendai-shi, Oimawashi, 24 Sep 1989, *Kurosawa 3149* (MO); Kanagama, Yokohama, 1862, *Maximowicz s.n.* (GH, W); Hyogo, Shiroyama, Hamasaka-cho, Mikata-gun (Prov. Tazima), 7 Nov 1967, *Murata 20136* (E, K, MO, P, US); N Honshu, Kawauchi, Ninomaru, Inohozuki, 22 Sep 1970, *Okazaki* & *Kanno s.n.* (MO); Yokuska, 1866, *Savatier 875* (K); Tokyo, 17 May 1910, *Sakurai s.n.* (E); Nara, nr Ogura Temple, E foot of Mt. Ikoma, 23 Oct 1986, *Seto 31840* (A); Kyoto, Yamasiro, 21 Jul 1925, *Shiota 107* (GH); Mino Prov, 10 Sep 1930, *Shiota 3815* (GH); Mino Prov, 15 Sep 1919, *Shiota 3999* (GH); Aichi, Owari, 5 Nov 1930, *Shiota 4663* (GH); Mino Prov, 27 Aug 1935, *Shiota 9095* (GH); Tokyo, Oct 1906, *Terasaki s.n.* (K); Tokyo, Prov. Musashi, 5 Sep 1888, *Without Collector s.n.* (K); Tokyo, Jun 1909, *Yokahama Nursery s.n.* (E); Tokyo, Sep 1909, *Yokahama Nursery s.n.* (E);

Kyushu: Kyushu, 4 Jun 1967, *Furuse 45139* (K); Kikaicho, Ooshimagun, Kikaizima, 2 Apr 1981, *Mimoro et al. 2645* (MO); Nagasaki, *Oldham 575* (GH, P, W); Nagasaki, 1862, *Oldham 852* (K); Nagasaki, Iki Island, Takenotsuji Gonoura-cho, Iki-gun, 1 Dec 1966, *Tsugaru 7717* (A). **Okinawa**: Okinawa, Ryukyu islands, N of Kadena, 12 Mar 1955, *Moran 4978* (GH, MO). **Ryuku Islands**: E side Mt. Nohara Ueno-son Is. Miyako, Miyako Islands, 19 Mar 1974, *Furuse 4837* (K). **Shikoku**: Shikoku (Prov. Tosa), 3 Nov 1964, *Furuse 42834* (K); Nagayama, Aki City, 6 Jul 2003, *Horiuchi et al. FOK-057483* (MO); Kochi, Yogaku, Tosayamada-cho, Kami City, 3 Dec 2013, *Yorimitsu FOS-5704* (BM); Tokushima, 1919, *Krug 7450* (B).

JORDAN. **Amman**: the desert rd to Thibham-Umm Al Rasas, 1 Sep 2002, *Abu-Laila et al. 70-4* (K); Wadi Shu'eib, 11.5 km NNW Tell Iktanu, 7 km N Kafrein, 17 Mar 1992, *Walter 7509b* (W); Wadi Shu'eib, 11.5 km NNW Tell Iktanu, 7 km N Kafrein, 17 Mar 1992, *Walter 7509a* (W).

KOREA. Sin loc., Jul 1901, *Faurie 512* (BM, P); Gualpaut, Aug 1907, *Faurie 1915* (W); Sep 1907, *Taquet 298* (E, W); 13 Aug 1908, *Taquet 1145* (K). **Jeju-do**: Quelpaert, Oct 1906, *Faurie 777* (BM, P). **Seoul**: Seoul Railway Yard, 24 Aug 1952, *Dann 60* (BM); prope montis Nansan, 20 Nov 1893, *Sontag s.n.* (BM, K).

LAOS. **Luang Prabang**: Prov. Luang Prabang, bas cours de la N'Hou, 18 Mar 1932, *Poilane 20429* (P).

LEBANON. Qasmich, South, 5 Feb 2002, *Breidy* & *Khairallah LEB-31* (K); Kesbah, 18 Feb 1945, *Trench s.n.* (BM).

LIBYA. Cyrenaica - Cymene, 3 Aug 1908, *Gregory s.n.* (BM); Oasis de Koufra hameau de El Giof, 4 Jan 1965, *Leonard 3708* (BM, K, MO). **Al Kufrah**: Koufra, 12 Oct 1968, *Leonard 4738* (BM, K). **Cirenaica**: Kouf [El-Kouf] National Park, nr park HQ, 29 Jun 1980, *Hemsley L-79* (K); Jebel el Akhdar, W end of Wadi El Kuf, 9 Jan 1959, *Keith 352* (K); Attag, Fueihat (Cyrenaica Distr.), 17 Apr 1959, *Keith 384* (K); Attag, Fueihat, Cyrenaica Distr., 12 May 1959, *Keith 419* (K). **Tripolitania**: Misurata, Tripolitania Distr., 21 Aug 1962, *Keith 1033* (K); Bir Meki, 7 mi W of Tarhuna, 15 Apr 1970, *Maitland 103* (K).

MALAYSIA. **Sabah**: Ranau Distr., Tenompok Ridge along Tamparuli - Ranau rd 6 km W of Kinabalu Park headquarters, 3 Jan 1984, *Beaman et al. 8216* (GH, K, MO); Mt. Kinabalu, nr Kundusan, by trail, 25 May 1932, *Clemens* & *Clemens 29774* (BM, K); Long Bayur, 5 mi upriver (on Abpa Matang) from Long Pasia, 10 Apr 2000, *Hoare* & *Barok 125* (K). **Malta.** Mar 1920, *Reade s.n.* (BM).

MOROCCO. Mogadose, 25 Apr 1859, *Lowe s.n.* (K); AA: 12 km E of Tiznit. Assaka, 3 Apr 1974, *Miller et al. 592* (BM, MO); Ulad Sebbut, lit[oral] du Muluya, 1 Jul 1933, *Sennen* & *Mauricio s.n.* (BM). **Béni Mellal-Khénifra**: Cascade d'Ouzoud, Azilal Prov., 21 Sep 1999, *Hmama et al. 894* (K). **Drâa-Tafilalet**: 2 km below Msemrir, 58 km from Boumalne along rd to Msemrir above the Gorges du Dadès, 10 Jul 1997, *Jury et al. 17766* (BM, MA). **Fès-Meknès**: Meserah, Larache, 19 Mar 1923, *Cabelleros s.n.* (MA). **Oriental**: Ennador, Nador, Apr 1912, *Cabelleros s.n.* (MA); près de Aïn-Benimathar, à l'est, 21 Jun 2008, *Calvo et al. 2457* (MA); 19: Beni Snassen, 10 km SSW Berkane, on rd from Taforalt to Grotte du Chameau, 29 Oct 1993, *Jury* & *Upson 13040* (BM). **Rabat-Salé-Kénitra**: Temara, 21 May 1981, *Lewalle 9857* (BM, MO); Temara, 20 Apr 1985, *Lewalle* & *Lambinon 11182* (BM, H, MA, MO); 16 km S of Rabat, Temara-Plage, 28 Mar 1974, *Miller et al. 114* (BM); c. 50 km SSSE of Rabat, above the Korifla gorde, Khemisset, 18 Jun 1996, *Vitek* & *Achhal el Kadmiri 96-381* (W). **Tanger-Tetouan-Al Hoceima**: SW of Chefchaouèn, 2.3 km up rd to Mokrissèt from Pont du Loukos, 21 Apr 1995, *Jury et al. 16503* (BM); Tangier, Apr 1921, *Pau s.n.* (MA).

MYANMAR (BURMA). Ngah Kyum, 16 Jan 1864, *Anderson s.n.* (CAL); Tonyaghat, S. Pagodas, *Kurz 201* (CAL); Upper Chindushi, Nansaka drainage, 7 Mar 1927, *Without Collector 5721* (CAL). **Chin State**: Mindat Distr., along the rdside between Kanpetlet and Yelong Pan Village, Natma Taung National Park, 21 Jul 2013, *Man 93206* (BM); Mindat Distr., Natma Taung National Park, Western Myanmar, 7 Dec 2002, *Murata 25627* (BM); **Kayah State**: Laikow, southern Shan States, 1903, *Khalil s.n.* (CAL). **Mandalay Region**: Bagan, Pagan, Mar 1864, *Anderson s.n.* (CAL). **Shan State**: Inle Lake, southern Shan States, 16 Feb 1917, *Annadale EB-323* (CAL); Fort Stedman, 1892, *Huk s.n.* (CAL); Fort Stedman, 2 Jul 1893, *King's collector 550* (CAL).

NEPAL. Far Western Development Region, Seti Zone, Doti Distr., trail from Sigadi to Khaptad National Park, 30 Jun 2009, *Bajhang 2009 Expedition 20913010* (E); Katuligaon, W Nepal, 2 May 1925, *Bis Ram 347* (BM); Sukia Tarai, 20 Mar 1913, *Cave s.n.* (A); Rongbe, 19 May 1913, *Cave s.n.*

(A); Badamtam, 21 Apr 1913, *Cave s.n.* (A); Sanku, 10 mi NE of Kathmandu, 8 Jul 1956, *Codrington 41* (BM); Eastern Development Region, Sagarmatha, Solu Khumbu, Dudh Khosh Valley, Toktok to Phakding, trail from Namche Bazar to Lukla, nr Toktok, 24 May 2004, *DNEP1 303* (E); Tarrakof, 29 May 1973, *Einarsson et al. 234* (BM); Illam, 7 Dec 1963, *Hara et al. 6303541* (BM); Kathmandu, Rupandehi, Khasyauli, May 1971, *Makin 168* (BM); Western Development Region, Gandaki Zone, Gorkha Distr., Phillim to Dyang, 29 Jul 2008, *Mamaslu 2008 Expedition 20816047* (E); Western Development Region, Gandaki Zone, Gorkha Distr., Machha Khola to Dobhan, before Tatopani, 26 Jul 2008, *Manaslu 2008 Expedition 20815026* (E); Bagmati zone, Rasuwa Distr., Syabru Bensi - Parbati Kund, 24 Jul 1994, *Miyamoto et al. 94-10017* (BM, E); Kali Gandaki, Tatopani - Dana - Titre - Ghasa, 16 Jul 1983, *Ohba et al. 83-10439* (BM); Janakpur zone, Ramechhap Distr., Shivalaya - Khasrubus - Deolari - Bhandar, 17 Aug 1985, *Ohba et al. 85-30831* (E); bridge across upper Thaple Kholu, 8 Apr 1953, *PBG-184* (BM); Poyora, 28 Jul 1952, *Polunin et al. 353* (BM); Syarpagoan, 23 Aug 1949, *Polunin 1858* (BM, E); Baglung, 19 Apr 1954, *Stainton et al. 53* (BM); Arun Valley of Chainpur, 29 Apr 1956, *Stainton 150* (BM); nr Jagat, 5 Jul 1954, *Stainton et al. 3384* (BM); N of Bokhara, 21 Apr 1954, *Stainton et al. 4959* (BM); Koshi zone, Sankhuwa Sabha Distr., by path nr stream Khandbari - Manebanjang - Danda Pangma - Sekaha - Chipe Gaun - Bhotebas, 7 Jul 1988, *Suzuki et al. 88-20210* (BM); Mechi Zone, Taplejung Distr., Khokling - Libang - Thunglung, 12 May 1992, *Suzuki et al. 92-40045* (E); Bagmati Zone, Rasuwa Distr., Dunche - Bhargu - Barbal - Gonpagon - Syabru, 10 Jul 1992, *Takayama et al. 92-39004* (E); N of Dharan, Sanguri Lekh, 3 Sep 1967, *Williams* & *Stainton 8336* (BM). **Central Development Region**: Kathmandu, Kirtipur, Nayabazar, 25 Mar 1997, *Dangol 12336* (W). **Dhankuta**: Koshi zone, Hile, 2 Jul 1988, *Suzuki et al. 88-20090* (A). **Western**: below Gandrung [Ghandrung] on trail to Chomro, 25 May 1971, *Barclay* & *Synge 2342* (K).

NETHERLANDS. **Gelderland**: Culemborg, 16 Aug 1997, *Christenhusz 894* (BM). **Overijssel**: Losser, Beuningen village, Jul 1997, *Christenhusz 895* (BM). **Zuid Holland**: den Haag, Bezuidenhout on ruderal ground, 29 Jul 1948, *Bakhuizen van den Brink 6552* (K); Woubrugge, 15 Aug 1948, *Maas Geesternanus 4314* (K); Leiden, 4 Sep 1906, *Wise s.n.* (E).

NEW ZEALAND. **Chatham Islands**: Chatham Island, 7 Dec 1993, *Sykes 521/93* (AK). **Kermadec Islands**: Sandy Island, Aug 1887, *Cheeseman 47* (K); Macauley Island, Shearwater Valley mouth, 22 May 2011, *de Lange K-814* (AK); Cheeseman Island, Southern Kermadec Islands Group, 24 May 2011, *de Lange K-847* (AK); Dayrell Island, Herald Islets, 18 May 2011, *de Lange K-1270* (AK); Chanter Island, Herald Islets, 16 May 2011, *de Lange K-1294* (AK); Dayrell Island, Herald Islets, 18 May 2011, *de Lange K-1317* (AK); Cheeseman Islet, 3 Mar 1971, *Sykes 937/K* (AK). **North Island**: Auckland, University of Auckland, Mar 1964, *Arminger s.n.* (AK); Auckland, Auckland metropolitan area, Pakuranga, 5 May 1976, *Back s.n.* (AK); Auckland, Mairangi Bay, 27 Apr 1973, *Bangerter 5024* (AK, K, MO); Auckland, Milford, Waitemata Co., 28 Mar 1981, *Bangerter 5508* (AK); Auckland, Campbell's Bay, 26 Oct 1951, *Baylis 10134* (MO); Northland, Moturohia Island, Bay of Islands Co., 7 Jan 1980, *Beever 80-095* (AK); Northland, Aupouri State Forest, 25 Sep 1985, *Bellingham 0142* (AK); Waikato, Middle Island, Mercury Islands, 13 Sep 1984, *Bellingham s.n.* (AK); Auckland City, Apr 1958, *Brady s.n.* (AK); Auckland, Lake Tahuna, 8 Jul 1981, *Brown s.n.* (AK); Bay of Plenty, Tarawera, Rotorua Distr., Jul 1981, *Brown s.n.* (AK); Auckland, Ponui (Chamberlin's) Island, 31 Aug 1978, *Brown s.n.* (AK); Auckland, Albany Hotel, 13 Feb 1978, *Brownsey s.n.* (AK); Wellington, Kaitoke Waterworks, 16 Apr 1978, *Brownsey s.n.* (AK); Wellington, Kaitoke Waterworks, 16 Apr 1978, *Brownsey s.n.* (AK); Northland, Motuti Island, 16 Jan 2002, *Cameron 10948 a* (AK); Northland, Whatikau Rock, 23 Apr 2003, *Cameron 11642* (AK); Auckland, Karamuramu Island, 12 Apr 2006, *Cameron 13822* (AK); Auckland, Tiritiri Matangi Island, 25 Mar 2007, *Cameron 14430* (AK); Auckland, Motukaha Island, 17 Jan 2010, *Cameron 15371* (AK); Auckland, Motuora, 16 Oct 2010, *Cameron 15560* (AK); Auckland, Challenger Island, 21 Feb 2011, *Cameron 15792* (AK); Waikato, Korapukai Island, Coromandel Region, Mercury Islands, 30 Nov 2010, *Cameron & Bellingham 15642* (AK); Auckland, Inner Gulf Islands, Wooded Island, 1 Sep 1998, *Cashmore s.n.* (AK); Mt. Eden, Nov 1848, *Cheeseman s.n.* (E); Mt. Eden, near Auckland, *Cheeseman s.n.* (US); Tapu Hill, 19 Feb 1966, *Cooper s.n.* (AK); Bay of Plenty, Katikati, Tauranga Co., 10 May 1965, *Cooper s.n.* (AK); Waitakere Range, Sep 1979, *Cooper & Nickerson 6019* (US); Auckland, Duck Creek, Roskill South, Jul 1954, *Cooper s.n.* (AK); Auckland, Huia, 1 Jun 1965, *Cooper s.n.* (AK); Manawatu-Wanganui, Mangaohae, Waitomo Distr., 13 May 1966, *Cooper*

s.n. (AK); Waikato, Maramaratotara, 18 Dec 1961, *Cooper s.n.* (AK); Waikato, Shoe Island, 24 Aug 1973, *Court s.n.* (AK); Waikato, Rabbit Island, 21 Aug 1973, *Court s.n.* (AK); Northland, Motukawanui Island, Waiiti Bay, Cavalli Island group, 1 Jan 1979, *Court s.n.* (AK); Mt. Albert Research Centre, Auckland City, 12 Nov 1974, *Court s.n.* (AK); Auckland, Otata Island, Apr 1951, *Edwards s.n.* (AK); Wellington, Wairarapa Plains Ecological Region and Distr., 11 May 2003, *Enright & John s.n.* (AK); Manawatu-Wanganui, Moutoa, Foxton, 1 Apr 1969, *Esler s.n.* (AK); Manawatu-Wanganui, Palmerston North, 20 May 1960, *Esler s.n.* (AK); Auckland, Great Barrier Island, Tataweka, 10 Feb 1965, *Frater et al. s.n.* (AK); Auckland, Grafton Gully, 21 May 1971, *Goulding & Hynes s.n.* (AK); Auckland, 4 Jul 1974, *Goulding & Bangerter 563* (AK, K); Auckland metropolitan area, Newmarket, 2 Dec 1976, *Goulding 718* (AK); Auckland, Middle Junction Island, 3 Jan 1985, *Grace s.n.* (AK); Waikato, Ruamahuanui Island, Aldermen Islands, 19 May 1972, *Hardacre s.n.* (AK); Auckland, Riverhead State Forest, 9 Jun 1985, *Hedley s.n.* (AK); Auckland, University of Auckland, 6 Feb 1959, *Hurt s.n.* (AK); Auckland, Waikowhai Park, 20 Mar 1951, *Hynes s.n.* (AK); Bay of Plenty, Whale Island, 30 Aug 1970, *Hynes s.n.* (AK); Auckland, Matheson Bay, Hobson Co., 8 Nov 1970, *Hynes s.n.* (AK); Auckland, Mount Roskill, 4 Dec 1972, *Hynes s.n.* (AK); Northland, Aorangi Island, 12 Dec 1998, *de Lange 3632* (AK); Auckland, Motuora, 19 Oct 1994, *de Lange & McFadden 2913* (AK); Auckland, Waitakere Ranges, Waitemata Co., 17 Jun 1978, *Mackinder s.n.* (AK); Wellington, 6 Mar 1952, *Mason 1363* (AK); Manawatu-Wanganui, Manakau, Wellington Prov, 7 Mar 1953, *Mason 2476* (AK); Northland, Motukino (Fanal) Island, 21 Mar 1995, *McFadden 9* (AK); Waikato, Channel Island, 6 Feb 1986, *McFadden s.n.* (AK); Northland, S side of Whangaripo Valley, 26 May 1985, *Moss s.n.* (AK); Auckland, Auckland Museum, 15 Jun 1972, *Orchard 3364* (AK); Auckland, Waitakere Range, Waitemata Co., 2 Aug 1972, *Orchard 3413* (AK); Northland, headland at E end of Opito Bay, 9 Oct 1972, *Orchard 3499* (AK); Northland, Mangonui Co., 15 Oct 1972, *Orchard 3639* (AK); Auckland, Auckland metropolitan area, 16 Jan 1973, *Orchard 3917* (AK); Waikato, ca. 5 km E of Coromandel, 10 Mar 1973, *Orchard 3948* (AK, K); Auckland, Otata Island, 20 Mar 1993, *Parris 12071* (AK); Waikato, Cuvier Island, 25 Aug 1968, *Parris s.n.* (AK); Northland, Whangarei Heads, Feb 1900, *Shakespear s.n.* (AK); Auckland, Mt. Eden, 20 Sep 1981, *Smith s.n.* (AK); Auckland, Parnell, Feb 1976, *Turbott s.n.* (AK); Auckland, Grey Lynn, 20 Jul 1946, *Wadham s.n.* (AK); Auckland, Piha, 13 Apr 1949, *Walker 5442* (MO, US); Auckland, Huia, 17 Dec 1949, *Wood s.n.* (AK); Auckland, Huia, Woods section, Waitemata Co., 17 Sep 1949, *Wood s.n.* (AK); Northland, North Cape Scenic Reserve, North Cape Co., 17 Nov 1975, *Wright 853* (AK); Auckland, Bethells Beach, Waitemata Co., 6 Feb 1977, *Wright 1896* (AK); Bay of Plenty, Waipoua State Forest, Hokianga Co., 9 Dec 1977, *Wright 2532* (AK); Northland, Kahangaroiti Island, 30 Dec 1978, *Wright 2920* (AK); Northland, Stephenson Island, 22 Aug 1982, *Wright 4848* (AK); Auckland, Unknown Island, Great Barrier Co., 6 Jan 1983, *Wright 5465* (AK); Northland, Puketi State Forest, Bay of Islands Co., 26 Jan 1985, *Wright 7054* (AK); Auckland, Auckland Museum, 6 Apr 1989, *Wright 8876* (AK); Waikato, Coromandel Co., 30 Aug 1983, *Wright s.n.* (AK); Northland, Taranga (Hen) Island, Taranga Distr., 9 Dec 2011, *Young & Aspin s.n.* (AK). **Outlying Islands**: Three Kings Islands, Northeast Island, 31 Dec 1947, *Buddle s.n.* (AK); Three Kings Islands, Great Island, 15 Dec 1982, *Wright 5237* (AK); Three Kings Islands, West Island, 17 Mar 2003, *Wright 12984* (AK); Three Kings Islands, Great Island, 17 Mar 2003, *Wright 12991* (AK). **South Island**: Canterbury, Middleton, Christchurch, 24 Mar 1959, *Healy 59/283* (AK); Bank's Pass, Jan 1866, *von Haast 265* (K); West Coast, Buller Co., 23 Jan 1953, *Mason & Moar 1609* (AK); West Coast, Buller Co., 25 Jan 1953, *Mason & Moar 1724* (AK); West Coast, Buller Co., 25 Jan 1953, *Mason & Moar 1726* (AK); West Coast, Westport, 1 Jan 1953, *Mason & Moar 2170* (AK); West Coast, Birchfield, Buller Co., 2 Feb 1953, *Mason & Moar 2200* (AK); West Coast, Tauranga Bay, Buller Co., 5 Feb 1953, *Mason & Moar 2298* (AK); Nelson, Pohara Beach, 46.03 Golden Bay, 5 Feb 2009, *Sneddon 43* (AK); Sumner, Jun 1869, *Travers 116* (K); Tasman, Farewell Spit, 25 May 1977, *Wright 2484* (AK).

NORTH KOREA. **Pyongyang**: Pyongyang, Pyengyang, 1 Oct 1938, *Smith s.n.* (F).

NORWAY. Forhingen ad Christianie, 16 Sep 1967, *Collett s.n.* (K); Nydalen, 27 Aug 1927, *Holmboe s.n.* (BM); Nydalen, 27 Aug 1927, *Holmboe s.n.* (BM).

OMAN. Sultanate of Oman Al Hajar, 14 Mar 1972, *Mandaville 3414* (BM). **Dhofar**: Jabal al Qara' Qara, N of Tawi Atair at beginning of desert zone, 19 Sep 1985, *Miller 7715* (E, K).

PAKISTAN. **Azad Kashmir**: Paryai, Saran range, Gozára Duayal, 3 Sep 1898, *Duthie s.n.* (K); **Balochistan**: Scinde, 1877, *Duke s.n.* (CAL); ca. 3 km from Johan on way to Kalat, Sarawan Valley,

13 May 1990, *Ghafoor & Goodman 5204* (E, F); **Gilgit–Baltistan**: Gilgit, Rana Valley, 7 Aug 1967, *Lankester & Pearson 1419* (BM); on the right side of the Shayok, via Kiris to Neru, on the right side of the Indus, 2 Aug 1856, *von Schlagintweit s.n.* (BM); **Khyber Pakhtunkhwa**: Northwest Frontier Province, Chitral Distr., CADP yard, Chitral town, 26 Jul 1999, *Dixon & Expedition to the Hindu Kush 1/99* (K); Chitral, Drosh, Apr 1908, *Toppin 82* (K); **Punjab**: Lahore, Apr 1938, *Chandhuri s.n.* (GH); **Sindh**: Khaipur DivPiryeloi, 26 Oct 1955, *Jafei 1173* (K); Sinde, *Stocks s.n.* (K).

PAPUA NEW GUINEA. Komaniamambuno, Mount Wilhelm, 4 May 1972, *Smith ANU-15352* (L). **East New Britain**: Lulka, *Bateson 121* (K);

PHILIPPINES. Sin loc., 1841, *Cuming 1033* (BM, K, P, W). **Luzon**: Central Luzon, San Luis, Brgy. Diteki Watershed area, Aurora Prov., 10 Mar 1993, *Barbon, et al. PPI-9142* (K, L); Cordillera, Benguet Prov., May 1904, *Barnes 955* (K); Ilocos, Currimao, Ilocos Norte Prov., 28 Oct 1935, *Bartlett 14825* (F); Cordillera, Chenglis, Bontoc Municipality, Mountain Prov., 18 Nov 1982, *Bodner 150* (MO); Bicol, Patang, Irosin, Sorsogon Prov., May 1957, *Edaño* & *Gutierrez 37715* (L); Cordillera, Baguio, Benguet Prov., Mar 1907, *Elmer 8788* (E, K); Bicol, Irosin, Sorsogon Prov., Apr 1916, *Elmer 15697* (BM, F, GH, K, MO, W); Cagayan Valley, Brgy. Cabudadan, Cagayan Prov., 13 Feb 1997, *Fuentes* & *de la Rosa PPI-38985* (GH, K, L, MO); Cordillera, Benguet Prov., May 1914, *Merrill 464* (BM, F, K, MO, P, W); Ilocos, Bangui, Ilocos Norte Prov., Feb 1917, *Ramos 27573* (BM)Cordillera, Mount Simacoco, Benguet Prov., Oct 1921, *Ramos* & *Edaño 40353* (BM); Cordillera, Pauai, Benguet Prov., Apr 1919, *Santos 32073* (BM); Cordillera (CAR), Bontoc Subprov., Mar 1913, *Vanoverbergh 3542* (F). **Mindanao**: Davao, Mount. Apo, North Cotabano, Ilomavis, Lake Venado, 17 Mar 1992, *Argent* & *Gaerlan PPI-9752* (K, MO); Mount Matutum, South Cotabato Prov., 15 Apr 1992, *Gaerlan et al. PPI-5373* (MO); ARMM (Muslim Mindanao), vicinity of Malabang, Lanao Prov., 10 Sep 1938, *Zwickey 148* (F). **Visayas**: Eastern Visayas, Leyte Island, 26 Jun 1913, *Wenzel 242* (E, F, GH, MO).

POLAND. **Kuyavia-Pomerania**: Borussica occid. Thorn Ziegeleowaldhohen, 21 Sep 1891, *Froelich s.n.* (PAL); Borussica occid. Thorn Uforbussen, 24 Sep 1889, *Froelich s.n.* (PAL); Borussica occid., 21 Sep 1890, *Froelich s.n.* (PAL); **Lower Silesia**: Slask Dolny, Wzgórza Dalkowskie, Szczyglice kolo Glogowa, Wzgórza Dalkowskie, Szczyglice nr Glogów, 18 Sep 2006, *Koziol 1635* (H, W); Slask Dolny, Wroclaw, na miejscach ruderalnych na Wzgórzu Partyzantów, Wroclaw, 16 Sep 1952, *Rekas 290* (H, W).

PORTUGAL. Caldas da Perez, 25 Jun 1887, *Murray s.n.* (BM); sin. loc, *Valorado s.n.* (BM). **Alentejo**: Serra do Ficalho, Apr 1910, *Chodat s.n.* (G). **Algarve**: Bai prov. Santa Catarina, 7 Dec 2006, *Aedo 13543* (MA). **Azores**: São Miguel, Seara nr Sete Cidades Lagoa Azul - West Bank, 26 Jul 1970, *Dolman 331* (BM);St. Miguel, 1857, *Drouet s.n.* (BM); Faial, Santo Amaro, 20 Mar 1962, *Goncalves 240* (BM); Flores, Fazenda das Lajes, 6 Aug 1963, *Goncalves 1277* (BM); Pico, São Roque, 6 Jul 1968, *Goncalves 2262* (BM); CORVO: Caldeirão, 8 Jun 1971, *Goncalves 2607* (BM); Sao Jorge, viveiro dos Rosais, 5 Sep 1971, *Goncalves 3513* (BM); Santa Maria, Vila/Aeroporto, 22 Apr 1972, *Goncalves 3762* (BM); São Miguel, Agua d'Alte, 5 May 1972, *Goncalves 4064* (BM); Faial, Largo Jaime Meio, Abzweguy nach Flamengos, 2 Jan 1999, *Schäfer 5569* (BM); Santa Maria, Cruz dos Picos, 21 Apr 2001, *Schäfer 9370* (BM); Flores. Ponta Delgada, Fajã do Gato: Path leading from village to fields below, 7 Jun 2008, *Universidade dos Açores-NHM 37* (BM); Flores, Ponta Delgada, footpath leading to Ponta do Ilheu, 7 Jun 2008, *Universidade dos Açores-NHM 46* (BM); Flores, Jul 1842, *Watson 146* (E); Pico, *Without Collector s.n.* (BM). **Centro**: Coimbra, bairro deJose', Dec 1886, *Moller s.n.* (BM); Chapel de Coimbra, Apr 1877, *Moller 2444* (BM). **Lisboa**: Sintra, Estremadura, prope Cintra, Oct 1850, *Ball s.n.* (E); Terrenos de Paia, arredoresde Lisboa, Estremadura, 3 Jun 1947, *de Lemos s.n.* (K); Parque Eduardo VII, 14 Oct 1971, *Martins 310* (MO); ad basim Serrae de Cintra, May 1840, *Welwitsch 226* (E, W). **Madeira**: Baia de Abra, 6 Mar 1986, *Cannon & Cannon 5038* (BM); Cabo Girão, to E of Fajã dos Asnos, 25 Nov 1989, *Chilton* & *Turland 41* (BM); Levada de Central da Ribeira da Janela, by edge of levada, 27 Nov 1989, *Chilton* & *Turland 122* (BM); Footpath from Ribeiro Frio to Balcoes, 30 Jul 1981, *Hampshire 80* (BM); Track from Camacha to Relogio, 23 Aug 1981, *Hampshire 439* (BM); sin. loc, 10 May 1859, *Lowe s.n.* (BM); Levada da Fajãa dos Vinhaticos (Sena do Fayal), Oct 1858, *Lowe s.n.* (BM); Mrs Gordon's kitchen garden at the Mt, 8 Dec 1832, *Lowe 16[a]* (BM); everywhere in roads, in walls etc, 31 Jun 1828, *Lowe 16[b]* (BM); Barro de Martianez Orotava, 10 Dec 1857, *Lowe 37[a]* (BM); Orotava, La Dehesa, 13 Feb 1858, *Lowe 37[b]* (BM); Pico de Conselho, 4 May 1855, *Lowe 119* (BM); Funchal, nr the

Lido, 29 Mar 1985, *Press 607* (BM); Funchal, nr the Lido, 29 Mar 1985, *Press 623* (BM); Cavao, top of rd from village, 13 Mar 1984, *Press* & *Short 26* (BM); Quebradas to Funchal. Along Levada dos Piornais, 1 Apr 1984, *Press* & *Short 515* (BM); Porto Santo, Pico do Facho, Barranco Branco, 12 Apr 1986, *Press* & *Short 1209* (BM); Levada da Serra do Faial, between Aguas Mensas and Santo da Serra, 21 Oct 1984, *Short 46* (BM); Isla Deserta Grande, alrededores de la casa de los forestales, 22 Mar 2007, *Velayos et al. 10893* (MA); Levada dos Tornos NE von Funchal, N von Sao Joao do Latrao, 27 Mar 2002, *Vitek 02-18* (W); ENE Funchal, Levada Dos Tornos zwishen der Strasse Aguas Mansas-Gaula und Camacha, 29 Mar 1996, *Vitek 96-136* (W). **Norte**: alquerrubim, entreJoao de Loure e Albergaria a Velha, 23 Apr 1965, *Fernandes et al. 9373* (UT); Pocinho (Douro Valley), 11 Jun 1889, *Murray s.n.* (BM); 4 mi N of Bragança, 8 Aug 1957, *Without Collector 304* (BM).

ROMANIA. Crisana, distr. Satu-Mare, circa pagum Satulung, 25 Aug 1939, *Forstner 2464* (W); Distr. Donstentje, ad Mongolia, in agris, *Jacobescu s.n.* (K). **Vest**: Caras-Severin, Oravita, In Wäldern, an Zäunen und an Hecken bei Orawicza, Csiklova im Bannat, Jul 1843, *Wierzbicki s.n.* (BM); Caras-Severin, Oravita, Auf Schutt, un Zaunen, in Obstgarten und in Waldern bei Orawicza, Flor. Banat, 1845, *Wierzbicki 393* (K); Caras-Severin, Oravita, in waldern, an zaunen und an Hecken bei Orawicza Csiklova im Bannat, Apr 1844, *Wierzbicki 2375* (K); Timis, Ciacova, Banat, Cziklowa, *Wierzbicki s.n.* (K).

RUSSIAN FEDERATION. Vladikavkas, Aug 1881, *Brotherus* & *Brotherus 767* (BM, H); In lavi callis et ad rias, Jul 1867, *Golde s.n.* (K); ex herbario horti bot. Petropolitani, *Maximowicz s.n.* (K); Prov. Rjasan, distr. Spassk, pa Brykin Bor., 6 Aug 1975, *Octjabreva* & *Czerevan 5887* (A, BM, E, H, K, W); 1861, *Without Collector s.n.* (BM); 25 Jul 1908, *Without Collector s.n.* (K); Soviet Central Asia, 26 Jun 1935, *Without Collector 93* (BM). **Far Eastern**: Sakhalin Oblast, Insula Sagalien, in littorae Korsakof, Aug 1908, *Faurie 690* (BM, E, P, W); Primorskay Oblast, Vladivistock, Primorskiy territory, suburb of Vladivostok, vicinity of Academic town, at the rd, by a heating pipeline, 23 Oct 1992, *Kharkevich,* & *Buch s.n.* (MO); Primorskay Oblast, Vladivistock, suburbs, vicinity of Academic Town, 23 Oct 1992, *Kharkevich et al. 686* (K). **Northwestern**: in ruderatis cultis, Florae Ingricae, Jul 1861, *Without Collector 449* (K). **Volga**: Volgogradskaya Oblast, Sarepta, 3 Jul 1879, *Becker s.n.* (BM).

SAINT HELENA. May 1771, *Banks* & *Solander s.n.* (BM); Kent House, Half-tree Hollow, 25 Feb 1983, *Cronk 335* (E); Below the ridge nr High Peak on weather side, 8 Aug 1986, *Cronk 409* (E); beneath the ridge nr High Peak on weather side, 8 Aug 1986, *Cronk 410* (E); sin. loc, *Cronk 447* (E); Ebony Plain, 20 Aug 2003, *Cronk 460* (E); Millennium Forest, nr Longwood, 4 Apr 2015, *Cronk* & *Driver s.n.* (BM); sin. loc, *Lind s.n.* (BM); Dover Spith St. Helena, 28 Aug 1839, *Link s.n.* (K); sin. loc, *Siebold s.n.* (W); Sin. loc., *Without Collector s.n.* (BM). **Tristan da Cunha**: Inaccessible Island, Blenden Hall 18 Feb 1938, *Christophersen 2324* (BM); field by Big Watron, The Settlement, 21 Feb 1962, *Dickson 103* (BM, E); Edinburgh, settlement plain, beach N of settlement, 13 Nov 2007, *Gremmen T07 0024* (E, K); Inaccessible Island, Blenden Hall 31 Oct 1982, *Hall 41* (BM); Sin. loc., 1908, *Keytel 1807* (BM); sin. loc, *Rogers 14* (BM).

SAUDI ARABIA. Makkah bypass, km 101, 3 Dec 1982, *Gasperetti PG-422* (MO); Saum, 14 Sep 1949, *Guichard KG/HAD/234* (BM, EA); Badanah, 6 Sep 1964, *Mandaville 308* (BM, W). **Eastern**: Hofuf, 30 km N, 1 Apr 1987, *Collenette 6212* (E). **Makkah**: Jeddah, Aug 1881, *Zohrab 57* (K). **Riyadh**: 16 kms WNW Riyadh in Wadi Wubayr, 14 Aug 1969, *Mandaville 2499* (BM);

SLOVAKIA. Malé Karpaty Mountains, Plavecky Peter village, in direction SE in little valley, 20 Oct 2010, *Mikoláš 2375* (W); Zemplinske hills, Cernochov village, Medovy vrch M[ountain], 0.7 km W at rd to village Cejkov, 10 Aug 2007, *Mikoláš 6723* (W). **Košice**: Košice, NW, hill Hdradová, 0.1 km E, 2 Aug 2008, *Mikoláš 5956* (W). **Nitra Region**: Tribec Mts, Nitra town, nr the medical institution, 23 Oct 2010, *Kucera* & *Slovák 171* (K). **Slovenia**. Carniolia, prope Labacum, Aug, *Paulin s.n.* (BM).

SPAIN. Prov. Jaen. Sierra de Cazorla, Casa Forestal de Nava de San Pedro to Agujeros de San Pedro, Montes de Cazorla, 11 Aug 1948, *Heywood* & *Davis 955* (BM); Algesiras, Jul 1924, *Hubbard s.n.* (BM). **Andalucia**: Malaga, flats nr shore W of Toron, 8 May 1927, *Ellman* & *Helmes 68* (K); Jaen, Aldeaquemada, la Venta, 9 Oct 1953, *Galiano s.n.* (BM); Jerez, 1893, *Higgins s.n.* (BM); Costa de la Luz, between El Palmar and Coñil de la Frontera on A5209, farm to E of rd, 12 Apr 2007, *Knapp IM-10095* (BM); Realejo, *Sinclair s.n.* (K); Cádiz, Isthmus Gaditani inter Betanas, 7 Feb 1845, *Willkomm 475* (K). **Baleares**: Mallorca, beside rd nr Military base on Puig Major, 2 Jun 1966,

Bowden & Sims 250 (BM); Mallorca, Cala Ratjada, 4 Jun 1966, *Bowden & Sims 367* (BM); Menorca Mahon, c. 3 km from Mahon round end of Cala Serja towards La Mola, N side of Mahon harbour, 31 Mar 1967, *Bowden & Sims 685* (BM); Ibiza, Mahon, c. 4 km from Mahon on rd to Ciundadela, 4 Apr 1967, *Bowden & Sims 871* (BM); Ibiza, Es Cana nr Santa Eulalia, 23 May 1966, *Cannon & Cannon 3154* (BM); Menorca, cala Alcaufar, 9 Aug 1970, *Cannon & Cannon 3526* (BM); Mallorca, Alcudia, rd to Barcarets, 6 Aug 1967, *Cannon & Cannon 3349* (BM); Mallorca, bei S'Arraco, 13 Jan 1973, *Gilli s.n.* (W). **Canary Islands**: Fuerteventura, South of Cotillo, 19 Feb 1973, *Aldridge 962* (BM); Tenerife, rd from Valle de Guerra to Cruz Chiquita, 1 Apr 1975, *Cannon et al. 4428* (BM); Tenerife, Sierra Anaga. Barranco de San Andres, 3 km N of San Andres, 4 Apr 1977, *Jarvis* & *Murphy 46* (BM)Island of Lanzarote, Arrieta, 20 Aug 1969, *Islip 44* (BM); Lanzarote Island, 28 May 1875, *Lowe s.n.* (BM); Gran Canaria, Teror, 13 May 1892, *Murray s.n.* (BM); Bario Fatima, San Miguel de La Palma, El Paso, 3 Sep 1969, *Staple 17* (BM). **Catalunya**: Tarragona, 20 km southwest of Tarragona, bewteen Cambrils and Hospitalet, 29 May 1962, *Brummitt 298[a]* (E); Barcelona, Massif on Zibidabi Pas del Rey, 9 Aug 1929, *Sennen s.n.* (BM); Barcelona, 18 Nov 1914, *Sennen s.n.* (BM); Barcelona, Penitents, Nov 1918, *Sennen s.n.* (BM); Barcelona, 26 Oct 1917, *Sennen s.n.* (BM). **Ciudad Autónoma de Melilla**: Melilla, *Pau s.n.* (MA). **Extremadura**: Extremadura, Galisteo, Oct 1850, *Ball s.n.* (E). **Madrid**: sin. loc., 24 Aug 1917, *Jerónimo 6* (BM); 7 Sep 1918, *Jerónimo 279* (BM); **Murcia**: Cartagena, E of the town, above La Union, 24 Apr 1926, *Ellman* & *Sandwith 338* (K).**Valencia**: Canet lo Roiga, 11 Jun 1999, *Velayos 9450* (W).

SRI LANKA. sin. loc, *Macrae 635* (BM); Brooks, 18 Sep 1931, *Simpson 8691* (BM); Negombo, 26 Nov 1931, *Simpson 8865* (BM); Rambaveva, 28 Mar 1932, *Simpson 9394* (BM); Haragama, nr Kandy, *Simpson 9728* (BM); sin. loc, *Without Collector s.n.* (BM); Bombay Presidency, Southern Maratha country and N Canara, *Young s.n.* (BM).

SUDAN. **Khartoum**: neighbourhood of Khartoum, 1861, *Petherick s.n.* (K).

SWEDEN. **Götaland**: Ystad, Oct 1908, *Ahlin* & *Cristoffersson s.n.* (BM); Västra Götaland, Göteborg, Marieholm, 6 Sep 1931, *Blom s.n.* (BM); Västra Götaland, Nödinge sn, Bohus station, 5 Aug 1937, *Blom s.n.* (BM, W); Västra Götaland, Mölndal, Svenska Oljeslageriet, 18 Oct 1950, *Blom s.n.* (BM); Västra Götaland, Bohns station, 5 Aug 1937, *Blom s.n.* (BM); Västra Götaland, Göteborg, Delsjöupplaget, 3 Sep 1956, *Blom s.n.* (BM); Västra Götaland, Göteborg, Delsjöupplaget, 6 Oct 1958, *Blom s.n.* (BM); Västra Götaland, Nodinge sn, Bohus station, ruderatplats, 5 Aug 1937, *Blom s.n.* (K); Västra Götaland, Göteborg, Molndal, Korkslatt, inkommen med bomull, 31 Jul 1943, *Blom s.n.* (K); Västra Götaland, Göteborg, Marieholm, 6 Sep 1931, *Blom 1373* (K); Västra Götaland, Göteborg, 31 Jul 1943, *Blom s.n.* (MO, W); Västra Götaland, Göteborg, Sep 1937, *Blom s.n.* (MO); Skåne, Malmö, May 1905, *Granvik s.n.* (BM); Maliceš, 1913, *Granvik s.n.* (BM); Skåne, Höganäs, 20 Sep 1909, *Hasslow s.n.* (BM); Skåne, Höganäs, 3 Oct 1910, *Hasslow s.n.* (BM); Scania, Bauhofha, Sep 1912, *Johansson s.n.* (BM); Skåne, Malmö, Sep 1886, *Johanson s.n.* (BM); Skåne, Malmö, 18 Sep 1887, *Johanson s.n.* (BM); Kalmar, Oskarshamn, Smaland, 20 Sep 1911, *Köhler s.n.* (BM); S [=Smaland], Oskarshamn, 20 Oct 1908, *Köhler s.n.* (BM); Smaland Oteaushamn, 20 Oct 1908, *Köhler s.n.* (BM); Skåne, Simrishamn, 7 Jul 1945, *Melderis s.n.* (BM); Skåne, Hörby, Sep 1927, *Påhlman s.n.* (K); Västra Götaland, Göteborg, Oct 1906, *Palmér s.n.* (BM); W Skallsjö, Oskarshöjd, Aug 1896, *Thedenius s.n.* (BM); Mölnlycke, Oct 1894, *Uhlemann s.n.* (BM); Mölnlycke, Oct 1894, *Uhlemann s.n.* (BM). **Svealand**: Uppsala, Aug 1870, *Ahlberg s.n.* (BM); Södermanland, Paroecia vagnhärad, Lökholmen, 24 Aug 1926, *Asplund s.n.* (BM); Södermanland, Paroecia Vagnharad, Lökholmen, in hortulo, 24 Aug 1926, *Asplund 1372* (K); Stockholm, Aug, *Afzelius s.n.* (MO); Stockholm, Aug, *Afzelius s.n.* (K); Stockholm, Sep 1863, *Nyman s.n.* (BM); Stockholm, Sep 1863, *Walmsted s.n.* (BM); Stockholm, Aug, *Without Collector s.n.* (BM).

SWITZERLAND. sin. loc, Aug 1859, *Balfour s.n.* (E). **Geneva**: Jussy, nr Geneva, 6 Oct 1972, *Charpin 158* (BM, E, H, UT); Lancy, champs, 11 Oct 1855, *Ducommun s.n.* (BM); 16 Sep 1885, *Herb. Déséglise, 169* (BM). **Ticino**: Lugano, ad montemSalvatoris, Aug 1842, *Ball s.n.* (K). **Valais**: Les cultures, Martigny, 5 Sep 1876, *Déséglise 460* (BM); Mouthey, 13 Aug 1863, *Hort s.n.* (BM). **Vaud**: Lausanne, 25 Sep 1884, *Hamilton s.n.* (E).

SYRIAN ARAB REPUBLIC. Haleb (Aleppo), Jul 1910, *Haklis s.n.* (W).

TAIWAN. Formosa, Ko-long-su, 1874, *Campbell s.n.* (BM); Formosa, 1864, *Oldham 236* (BM); Formosa, 1864, *Oldham 336* (GH, K, P, W); Taihoku, 24 Oct 1926, *Sasaki 275* (MO). **Kinmen**: Fuchien, Kinmen Hsien, Kinhu Town, Hulung, 5 Mar 1997, *Wang* & *Lin 2550* (MO). **Nantou**:

Nantou Hsien, Feng Huan-ku Bird Park, SE of Nantou, 17 Mar 1992, *Bartholomew* & *Boufford 6200* (A, F, MO). **Taichung**: Ta-hsüeh Shan, 6 Nov 1971, *Liu* & *Ou 302* (BM). **Taipei**: Urai, 11 Dec 1961, *Kao* & *Chuang 4696* (A); Taipei, vicinity on Taihoku, 18 Dec 1929, *Tanaka 1760* (BM, GH).

THAILAND. Nong Kae, W coast of Gulf of Siam, 28 Sep 1927, *Collins 1579* (BM); Nantien valley, Mar 1914, *Forrest 12277* (GH); Bangkok, from chinese medical shop, 18 Feb 1932, *Kerr s.n.* (BM); Ranheng, Nr Wang Chao (Raheng), 12 Nov 1920, *Kerr 4567* (BM); Puk Ta Wan, nr Puk Tah Wan, 18 Apr 1926, *Ladell 263* (BM, GH); Pra Jasm, Tepah, 23 Mar 1928, *Lakshnakara 368* (BM); Hua Hin, 5 Nov 1927, *Marcan 2225* (BM); Chiengrai forest station, 1 Mar 1958, *Sørensen et al. 1756* (A, E); Korat, Kao Lem, 27 Dec 1930, *Put 3548* (BM); Surat, Kantuli, 10 Sep 1931, *Put 4188* (BM). **Chiang Mai**: nr Chiang Mai Coeducational Center, Ping River drainage, 10 Aug 1976, *Anderson 3773* (MO); Chiang Mai Prov., 35.7 km NE of Chiang Mai on the Doi Saket rd to Chiang Rai, 4 Nov 1976, *Anderson 3836* (MO); Doi Sutep, 30 Sep 1921, *Kerr 403* (BM); Chiang Mai, 20 Jun 1910, *Kerr 1231* (BM); Jawm Tong, Mae Soi Ridge, Mae Soi subDistr., nr ban Bah Gluay (Meo) village, 28 Mar 1992, *Maxwell 92-100* (A). **Chiang Rai**: Muang, Huay Mae Liam, SubDistr. Huay Chompu, 18 Mar 1989, *Bragg 133* (GH). **Lampang**: Jaehom, Jaehomwittaya school, 13 Nov 1999, *Panatkool 221* (A); Jaehom, Jaehomwittaya school, Southern area in the forest, 18 Jun 2000, *Panatkool 322* (A). **Lampoon**: Mae Tah, Doi Kuhn Dahn National Park, Tah Goo Station area, Hong Hang Stream valley, 1 Dec 1994, *Maxwell 94-1244* (A, L). **Mae Hong Song**: Pai, Ban Tah By, Toong Yow sunDistr., nr the By River, 12 Sep 1992, *Maxwell 94-536* (GH).

TRISTAN DA CUNHA. Inaccessible Island, Blenden Hall 18 Feb 1938, *Christopherson 2302* (K); Inaccessible Island, alogn path leading to the summit from Denstone Hut, 8 Oct 1989, *Roux 2080* (K); Tristan da Cunha, 1953, *Stapleford s.n.* (K).

TUNISIA. **Nabuel**: Nabuel, Sep 1907, *Gandoger 17* (K); Nabuel, Oct 1907, *Gandoger 215* (K);

TURKEY. Antalya, Alanya, Demirtas, Camlica Koyu, Beslengi Mahallesi, 14 Jun 2006, *Dönmez et al. 13562* (MO); A5 Sinop, Akgol Inalti Magarasi yol ayrimindan Ayancik'a 6 km, 15 Sep 2009, *Dönmez 16221* (W); Szandschak Gümüschkhane, Taltaban, 30 Aug 1894, *Sintenis 7469* (W); Vilayet, Samsun, E of town, 24 Aug 1964, *Tobey 817* (E); Sep 1863, *Without Collector s.n.* (BM).

TURKMENISTAN. Ashgabat City, Regio transcaspica, Aschabad, in cultis Iter transcaspico-persicum, Sep 1900, *Bornmüller 636* (K); Western Kopet Dag, in the canyon Aiydere, 12 Jul 2001, *Kurbanov 1606* (MO); Iter Turkestanicum, Kulosoka, 1877, *von Regel s.n.* (K); Jul 1877, *von Regel s.n.* (K); Regio transcaspica, Aschabad, Sep 1900, *Sintenis 636* (BM, E, P, W); *Without Collector s.n.* (K).

UKRAINE. Kiev, Prope op Umanj, 2 Sep 1904, *Czernous s.n.* (MO). **Kiev**: prope op Umanj, 2 Sep 1904, *Czernous s.n.* (BM); Uman, 2 Sep 1902, *Czernous s.n.* (W); prope op Umanj, in ruderatis, 2 Sep 1904, *Czernous s.n.* (K); prov. Kiev, op Umanj, 15 Jun 1907, *Lochanko* & *Boguschevsky s.n.* (E, W); op Umanj, in ruderato, 15 Jun 1907, *Lochanko s.n.* (K); op Umary, 15 Jun 1907, *Lochonki* & *Boguschevsky s.n.* (BM). **Krym**: Saky, Tavricheskaya Gu, Saki, 25 Jul 1908, *Shiraevskii s.n.* (E). **Kyiv**: Kiev, Kyivska oblast, Obolon, 11 Jul 1987, *Zaverukha s.n.* (MO).

UNITED ARAB EMIRATES. **Abu Dhabi Emirate**: Abu Dhabi Emirate, Al-Ain Hilton Hotel, 26 Feb 1986, *Boulos* & *Al-Hasan 15722* (BM).

UNITED KINGDOM. Upper Burtley, 1831, *[E.] s.n.* (BM); Sin. loc., 27 Jul 1921, *Beal s.n.* (BM); Sin. loc., 1862, *Benbow s.n.* (BM); Sin. loc., *Herb. Davies s.n.* (BM); Sin. loc., 16 Sep 1867, *Dyer s.n.* (BM); Sin. loc., 1846, *Forbes s.n.* (BM); Our own garden, 1868, *Taylor s.n.* (BM); Sin. loc., 1845, *Without Collector s.n.* (BM); Sin. loc., 15 Aug 1885, *Wright s.n.* (BM). **Channel Isles**: Alderney, Braye Harbour, Cultivated Ground, 18 Aug 1932, *Jackson s.n.* (BM); Guernsey, at Les Nouettes, 6 May 1985, *McClintock, et al. s.n.* (BM); Jersey, Sea Sand, St Ouens Bay, Aug 1880, *Shates s.n.* (BM); Sark, 13 Jul 1953, *Sowerby 204* (BM); Jersey, Jersey, 29 Aug 1930, *Williams s.n.* (BM). **England**: Devon, Lundy Island, 5 Sep 1955, *Alston 17405* (BM); Greater London, Mile End Park, 4 Sep 2008, *Atchison 3* (BM); East Sussex, Madeira Drive, 3 Sep 2008, *Atchison 4* (BM); Greater London, Victoria Park, 4 Sep 2008, *Atchison 5* (BM); Greater London, Mile End Park, 4 Sep 2008, *Atchison 6* (BM); Staffordshire, Kidsgrove, Below Lock 46 on the eastern bank of the Trent and Mersey canal, 15 Sep 2008, *Atchison 7* (BM); Herefordshire, Breinton, Jun 1865, *Augustín Ley s.n.* (BM); Suffolk, Ampton, 20 Sep 1877, *Babington s.n.* (BM); Kent, Sandown Castle, 30 Aug 1878, *Bailey s.n.* (BM); Norfolk, Cromer, 3 Sep 1874, *Bailey s.n.* (BM); Greater London, Royal Botanic

Gardens, Kew, Aug 1877, *Baker s.n.* (BM); Greater London, Chiswick, House grounds, 13 Sep 1932, *Bangerter s.n.* (BM); Greater London, Chiswick, 8 Sep 1936, *Bangester s.n.* (BM); Gloucestershire, nr. Tewkesbury, 26 Oct 1954, *Bannister s.n.* (BM); Worcestershire, Charlton, 4 Nov 1957, *Bannister s.n.* (BM); Kent, Pegwell Bay, v.c. 15, 19 Jul 1905, *Barton s.n.* (BM); Greater London, Uxbridge Common, 1894, *Benbow s.n.* (BM); Surrey, Croydon, 1 Sep 1863, *Bennett s.n.* (BM); Surrey, Mitcham Common, 23 Sep 1899, *Bennett s.n.* (BM); Surrey, Croydon, Jul 1911, *Bennett s.n.* (BM); Greater London, Royal Botanic Gardens, Kew, Aug 1979, *Bennett 8* (AK); Greater London, *Blow s.n.* (BM); Kent, Faversham, 1875, *Blow s.n.* (BM); Greater London, Hampstead Heath, 1863, *Boswell-Syme s.n.* (BM); Kent, Woolwich, 1852, *Boswell-Syme s.n.* (BM); Greater London, Isle of Dogs, 1852, *Boswell-Syme s.n.* (BM); Greater London, Haverstock Hill, Sep 1867, *Boswell-Syme s.n.* (BM); Greater London, Haverstock Hill, Sep 1866, *Boswell-Syme s.n.* (BM); Greater London, Hampstead Heath, Jul 1866, *Boswell-Syme s.n.* (BM); Greater London, Twickenham, Sep 1867, *Boswell-Syme s.n.* (BM); Norfolk, Kings' Lynn, 20 Aug 1849, *Boswell-Syme s.n.* (BM); Norfolk, Kings' Lynn, 20 Aug 1849, *Boswell-Syme s.n.* (BM); Surrey, Battersea, 1852, *Boswell-Syme s.n.* (BM); Greater London, Hampstead Heath, Aug 1850, *Boswell-Syme s.n.* (BM); East Sussex, South Lancing, 7 mls W of Brighton, 5 Aug 1976, *Bovey 61* (AK); Greater London, Richmond Park, garden of lodge at Richmond Gate, 20 Sep 1979, *Bristow 17* (AK); Surrey, Hook, 5 Oct 1930, *Britton s.n.* (BM); Surrey, Hook, 5 Oct 1930, *Britton s.n.* (BM); Surrey, Hook, 5 Oct 1930, *Britton s.n.* (BM); Surrey, Hook, 5 Oct 1930, *Britton s.n.* (BM); Surrey, Hook, 5 Oct 1930, *Britton s.n.* (BM); Warwickshire, Milverton, Aug 1894, *Bromwich s.n.* (BM); Warwickshire, Milverton, Aug 1898, *Bromwich s.n.* (BM); Warwickshire, Milverton, Sep 1897, *Bromwich s.n.* (BM); Lancashire, Linacre, Aug 1873, *Brown s.n.* (BM); Greater London, Chiswick, Barrowgate Road, client's back garden, 1 Aug 1976, *Burman 33* (AK); Lincolnshire, Gibraltar Point, 15 Aug 1891, *Burtt Davy s.n.* (BM); Greater London, Tottenham, 1888, *Burtt Davy s.n.* (BM); Surrey, Combe Wood, 26 Sep 1863, *Bywater s.n.* (BM); Suffolk, Burgh Castle, 5 Nov 1938, *Campbell s.n.* (BM); Suffolk, Burgh Castle, 5 Sep 1938, *Campbell s.n.* (BM); Surrey, Sheen Common, 2 Aug 1894, *Cave s.n.* (CAL); Buckinghamshire, Marlow, 23 Jul 1864, *Chandler s.n.* (BM); Lincolnshire, Sutton St Edmunds, 5 Nov 1891, *Chandler s.n.* (BM); Cambridgeshire, Parsons Drove, 6 Nov 1891, *Chandler & Burtt Davy s.n.* (BM); Greater London, Clapton, 16 Aug 1909, *Cooke s.n.* (BM); Cornwall, Coast - Mount's Bay, Penzance, Jun 1877, *Coumoce s.n.* (BM); Surrey, Wandsworth Common, , *Crabbe s.n.* (BM); Greater London, London, besdie Grand Union Canal, nr Mile End, Sep 2004, *Dupree s.n.* (BM); Hampshire, Portsea, nr Hayling Ferry, Aug 1951, *Dupree s.n.* (BM); Surrey, Bank at Reigate Heath (vc17), 9 Aug 1838, *Elgar s.n.* (BM); Isle of Wight, St Helens, Jul 1884, *Fawcett s.n.* (BM); Berkshire, Buckland, nr Faringdon, 14 Aug 1956, *Fletcher s.n.* (BM); Berkshire, Buckland, nr Faringdon, 14 Aug 1956, *Fletcher s.n.* (BM); Norfolk, Larling, 9 Sep 1926, *Foggitt s.n.* (BM); Cambridgeshire, Chippenham gravel pit, 26 Aug 1909, *Foggitt s.n.* (BM); Lincolnshire, North Lincolnshire, 1876, *Fowler s.n.* (BM); Surrey, Putney, Aug 1915, *Fox s.n.* (BM); Surrey, Putney, Jul 1921, *Fox s.n.* (BM); Surrey, Surrey, Jul 1913, *Fox s.n.* (BM); Northamptonshire, Northampton, 1 Sep 1867, *French s.n.* (BM); Devon, Torquay, 17 Oct 1921, *Geff s.n.* (BM); West Sussex, banks of River Arun, Pulborough, 30 Sep 1969, *Gerrans 1560* (BM); Berkshire, Aston/Remenham, water meadows beside River Thames between Aston and Remenham, 7 Sep 1970, *Gerrans 1619* (BM); Surrey, Surrey, Unreadable exact location - ???tlalie, 2 Oct 1875, *Groves & Groves s.n.* (BM); Kent, Waste ground between Hayes and Bromley, W.Kent, 28 Sep 1901, *Groves s.n.* (BM); Somerset, Clapton, 9 Aug 1884, *Hanbury s.n.* (BM); Hertfordshire, Ware, Jul 1996, *Hanson BS96 25* (BM); Hertfordshire, Ware, 27 Sep 1997, *Hanson BS97 30* (BM); Hampshire, Boscombe, 20 Sep 1893, *Hauts s.n.* (BM); Greater London, Bombed-site Shadwell, the Highway, 25 Aug 1952, *Henson s.n.* (BM); Greater London, Bombed-site The Highway, Shadwell, 25 Aug 1952, *Henson s.n.* (BM); Kent, W- New Romney, Kent, Aug 1875, *Herb. Hanbury s.n.* (BM); Kent, Ospringe, 13 Sep 1875, *Herb. Hanbury s.n.* (BM); Cambridgeshire, Histon, 2 Nov 1908, *Herb. Adamson s.n.* (BM); Suffolk, Tuddenham, 16 Jul 1908, *Herb. Adamson s.n.* (BM); Greater London, Islington, Aug 1837, *Herb. Ballard s.n.* (BM); Bedfordshire, Maulden, 27 Sep 1960, *Herb. Donald Peter Young s.n.* (BM); Bedfordshire, Flitwick, 25 Oct 1958, *Herb. Donald Peter Young s.n.* (BM); Cheshire, New Brighton, Sep 1901, *Herb. Drabble s.n.* (BM); Essex, Hale End, , *Herb. Forster s.n.* (BM); Essex, Barhams, *Herb. Forster s.n.* (BM); Surrey, Croydon, Sep 1866, *Herb. French s.n.* (BM); Greater London, Tufnell Park, 25 Aug 1875, *Herb. French s.n.* (BM);

Greater London, Upper Holloway, 19 Sep 1875, *Herb. French s.n.* (BM); Wiltshire, between Melksham & Lacock, 20 Oct 1882, *Herb. Gregory s.n.* (BM); Greater London, Palmers Green, 3 Sep 1912, *Herb. Hall s.n.* (BM); Isle of Wight, Freshwater, 17 Aug 1871, *Herb. Hanbury s.n.* (BM); East Sussex, Eastbourne, 26 Jul 1871, *Herb. Hanbury s.n.* (BM); Greater London, Uxbridge, 12 Aug 1904, *Herb. J. Roffey s.n.* (BM); Hampshire, Oakhanger, 8 Aug 1836, *Herb. J.W. Curtis s.n.* (BM); West Yorkshire, Calverley, 28 Aug 1909, *Herb. Lees s.n.* (BM); Greater London, Hornsey, 9 Aug 1901, *Herb. London Natural History Society s.n.* (BM); Surrey, Parkstall Rise, Croydon, *Herb. Mennell s.n.* (BM); Surrey, Croydon, , *Herb. Mennell s.n.* (BM); Somerset, Minehead, 10 Sep 1892, *Herb. Murray s.n.* (BM); Surrey, Camberwell, Glebe Road, *Herb. Payne s.n.* (BM); Devon, nr Canal Exeter, Sep 1922, *Herb. Pegler s.n.* (BM); Greater London, Tottenham, Jul 1838, *Herb. Pryor s.n.* (BM); West Sussex, Felpham, Aug 1914, *Herb. Redgrove s.n.* (BM); East Sussex, Pett Level, Aug 1914, *Herb. Redgrove s.n.* (BM); Surrey, Battersea Fields, , *Herb. Sowerby' s.n.* (BM); Kent, Faversham, Sep 1875, *Herb. T. B. Blow s.n.* (BM); East Sussex, Hailsham, Carter's Corner, 2 Nov 1947, *Herb. Young s.n.* (BM); Essex, Dagenham, 13 Aug 1950, *Herb. Young s.n.* (BM); Surrey, Croydon, 1880, *Herb.Bennett s.n.* (BM); Cornwall, Fields, nr Penzance, Aug 1878, *Herb.Bennett s.n.* (BM); Bedfordshire, Ampthill, 27 Aug 1901, *Higgins s.n.* (BM); Chepstow, Aug 1897, *Higgins s.n.* (BM); Devon, Braunton, 8 Aug 1889, *Higgins 1016* (BM); West Sussex, Wiston, , *Hilton s.n.* (BM); West Sussex, Heenfield, Aug 1886, *Hilton s.n.* (BM); West Yorkshire, Esholt, 10 Aug 1957, *Houseman s.n.* (BM); West Yorkshire, Esholt sewage works tip, 9 Oct 1960, *Houseman s.n.* (BM); Surrey, Winkworth Arboretum, 10 Aug 1977, *Ingall 005* (AK); Essex, West Hanningfield, 10 Oct 1953, *Jermyn 42* (BM); Essex, Leigh, 29 Oct 1954, *Jermyn 338* (BM); Greater London, Ealing, Jul 1913, *Jerrel, a, s.n.* (BM); Essex, Norton Heath, 3 Oct 1923, *Keath s.n.* (BM); Greater London, Kew Bridge, 7 Oct 1945, *Kent s.n.* (BM); West Yorkshire, Meanwood, Low side (west) at Back Bentley Lane, 5 Aug 1917, *Lees s.n.* (BM); West Yorkshire, Woodhouse, 17 Sep 1917, *Lees s.n.* (BM); West Yorkshire, Back of Wood lane, Headingley, where clearing of hedge to enlarge Gilted Crowther's garden had been made, 21 Jul 1917, *Lees s.n.* (BM); West Yorkshire, Meanwood, 7 Sep 1915, *Lees s.n.* (BM); Dorset, Poole, 9 Sep 1910, *Linton s.n.* (BM); Dorset, Poole, 22 Aug 1893, *Linton s.n.* (BM); Dorset, Poole, 9 Sep 1910, *Linton s.n.* (BM); Dorset, Poole, 22 Aug 1893, *Linton s.n.* (BM); Dorset, Poole, 9 Sep 1910, *Linton s.n.* (BM); Dorset, Poole, 9 Sep 1910, *Linton s.n.* (BM); Dorset, Poole, 22 Aug 1893, *Linton s.n.* (BM); Hampshire, Boscombe, 20 Sep 1893, *Linton s.n.* (BM); Dorset, Poole, 9 Sep 1910, *Linton s.n.* (BM); Hampshire, Kingston Common, 25 Aug 1893, *Linton s.n.* (BM); Hampshire, Pokesdown, Sep 1893, *Linton s.n.* (BM); Hampshire, Pokesdown, Sep 1893, *Linton s.n.* (BM); Hampshire, Pokesdown, 20 Sep 1893, *Linton s.n.* (BM); Hampshire, Boscombe, 20 Sep 1893, *Linton s.n.* (BM); Oxfordshire, Walton Manor, 1 Aug 1870, *Linton s.n.* (BM); Gloucestershire, Bristol, 25 Aug 1836, *Long s.n.* (BM); Bedfordshire, Ampthill, 15 Oct 1953, *Lousley s.n.* (BM); Hampshire, Southampton Docks, 12 Oct 1958, *Lousley s.n.* (BM); Devon, Newton Abbot, 12 Aug 1959, *Lousley s.n.* (BM); Worcestershire, Charlton, 1 Sep 1953, *Lousley s.n.* (BM); Bedfordshire, Flitwick, 25 Oct 1958, *Lousley s.n.* (BM); Worcestershire, Charlton, 2 Oct 1960, *Lousley s.n.* (BM); Worcestershire, Severn Stoke, 15 Oct 1961, *Lousley s.n.* (BM); Hampshire, Bournemouth, Oct 1884, *Lower s.n.* (BM); Surrey, Mortlake, Oct 1875, *Marshall s.n.* (BM); Devon, Dawlish Warren, 15 Oct 1888, *Marshall s.n.* (BM); Derbyshire, Kedleston, Aug 1958, *Mason s.n.* (BM); Devon, Braunton Burrows, Aug 1850, *Maw s.n.* (BM); Devon, Braunton Burrows, Aug 1850, *Maw s.n.* (BM); Greater London, Buckingham Palace Garden, 4 Sep 1969, *McClintock s.n.* (BM); Surrey, Mortlake, Oct 1878, *Mcholsen s.n.* (BM); Greater London, Royal Botanic Gardens, Kew, Sep 1979, *McNamara 12* (AK); Greater London, Hackney Marshes, 29 Aug 1927, *Melville s.n.* (BM); Shropshire, Sheinton, *Herb. Miss Moseley s.n.* (BM); Surrey, Wandsworth, Aug 1886, *Monington s.n.* (BM); Surrey, Mortlak, Sep 1888, *Monington s.n.* (BM); West Sussex, East Preston, Ferring, 15 Sep 1918, *Herb. Mrs. Atkins s.n.* (BM); Dorset, Wareham, 26 Aug 1885, *Murray s.n.* (BM); Isle of Wight, St Helens, 24 Sep 1885, *Murray s.n.* (BM); Dorset, Wareham, 11 Sep 1899, *Murray s.n.* (BM); Hampshire, Ringwood, 25 Aug 1893, *Murray s.n.* (BM); Somerset, Berrow, 14 Aug 1883, *Murray s.n.* (BM); Somerset, Butleigh, 2 Sep 1881, *Murray s.n.* (BM); Greater London, Acton, 14 Aug 1891, *Nuth s.n.* (BM); Kent, Romney Marsh, 1 Sep 1881, *Parker s.n.* (BM); West Yorkshire, Rawcliffe, 30 Sep 1875, *Parsons s.n.* (BM); Surrey, Streatham, Jul 1907, *Paton s.n.* (BM); Essex, Walthamstow, Aug 1881, *Paulson s.n.* (BM); Devon, Braunton, 10 Aug 1918, *Riddelsdell s.n.* (BM);

Bedfordshire, Oakley, 10 Aug 1924, *Riddelsdell s.n.* (BM); Bedfordshire, Oakley, 10 Aug 1924, *Riddelsdell s.n.* (BM); Greater London, Hale End, 31 Jul 1904, *Robbins s.n.* (BM); Norfolk, Walton, Jul 1911, *Robinson s.n.* (BM); South Yorkshire, 5 Sep 1952, *Rolpom s.n.* (BM); Gloucestershire, Bristol, St Philips marsh, 13 Oct 1921, *Roper s.n.* (BM); East Sussex, Eastbourne, 5 Oct 1882, *Roper s.n.* (BM); Gloucestershire, Bristol, St Philips marsh, 13 Oct 1922, *Roper s.n.* (BM); Gloucestershire, Bristol, St. Philips Marsh, 13 Oct 1922, *Roper s.n.* (BM); Gloucestershire, Bristol, St Philips Marsh, 13 Oct 1922, *Roper s.n.* (BM); Cambridgeshire, inter Westwick et Cottenham G.R. 52/425657, 17 Oct 1967, *Sell 67-1814* (BM, H, W); Surrey, Wandsworth, 19 Sep 1891, *Showpson s.n.* (BM); Gloucestershire, Clifton, 1905, *Simpson 5354* (BM); Dorset, Poole, Liliput Village, 13 Sep 1928, *Simpson 28096* (BM); Hampshire, Mudeford, Christchurch, 17 Sep 1928, *Simpson 28118* (BM); Dorset, Wareham, 9 Sep 1934, *Simpson 34461* (BM); Dorset, Parkestone Dump, 11 Sep 1934, *Simpson 34479* (BM); Essex, South Ockendon, 31 Jul 1949, *Small s.n.* (BM); Cheshire, West Kirby, 22 Jul 1883, *Smith s.n.* (BM); Essex, Thorpe, 14 Aug 1894, *Standen s.n.* (BM); Suffolk, Lowestoft, 28 Jul 1859, *Trimen s.n.* (BM); Essex, North Essex, 22 Sep 1883, *Vaughan s.n.* (BM); Cheshire, Dicksons Nurseries, Chester, Oct 1916, *Waterfall s.n.* (BM); Cheshire, Kilmorey Park, Hoole, 25 Sep 1937, *Waterfall s.n.* (BM); Cheshire, Chester, Oct 1916, *Waterfall s.n.* (BM); Surrey, Thames Ditton, 1852, *Watson s.n.* (BM); Surrey, North Surrey, 1865, *Watson s.n.* (BM); Kent, 15 Sep 1875, *Webb s.n.* (BM); Greater London, Greenford (Canal), 28 Jul 1918, *Wernham s.n.* (BM); Greater London, Holborn, Bartholomew's Close, 17 Sep 1950, *Whittaker 87* (BM); Greater London, Holborn, Millman Street, 10 Aug 1950, *Whittaker 601* (BM); Surrey, Balham Park Rd, Wandsworth Common, Aug 1885, *Whitwell s.n.* (BM); Surrey, Surrey, 19 Aug 1943, *Williams s.n.* (BM); Dorset, Poole, 9 Sep 1910, *Wilmott D20 7* (BM); Surrey, Oct 1875, *Without Collector s.n.* (BM); Suffolk, Raydon, Aug 1835, *Without Collector s.n.* (BM); Surrey, nr Esher, *Without Collector s.n.* (BM); Dorset, *Without Collector s.n.* (BM); Greater London, Royal Botanic Gardens, Kew, 9 Sep 1884, *Wright s.n.* (BM); Surrey, Esher, Wadside, 15 Aug 1892, *Wright s.n.* (BM); Surrey, Esher, 16 Aug 1892, *Wright s.n.* (BM); East Sussex, Tidebrook Manor Farm, 4 Aug 1986, *Zeylstra s.n.* (BM). **Gibraltar**: Almandral, Flora Calpensis, 23 Mar 1913, *Wolley-Dod 1606* (K). **Scotland**: Fife, Waste ground at Whyte-Melville rd, Kirkcaldy, 8 Nov 1970, *Ballantyne 418* (E); Wigtownshire, between Glenluce and P[ort] William (vc74), *Boswell-Syme s.n.* (BM)Moray, 14 Aug 1976, *Webster s.n.* (BM); Orkney Islands, Papa Westray, Holland House, 17 Sep 1933, *Traill s.n.* (BM); South Ayrshire, Ayr, Oct 1839, *Veronge s.n.* (BM). **Wales**: Barmouth, Aug 1880, *Hagger s.n.* (BM); Aberafan, 18 Sep 1909, *Riddelsdell s.n.* (BM); Cardiff, Civic Centre, 10 Sep 1975, *Pinkard 41* (UT); Cardiff, Canton, 31 Jul 1913, *Riddelsdell s.n.* (BM); Gwynedd, Barmouth Estuary, 10 Aug 1930, *Herb. Meinertzhagen s.n.* (BM); Monmouthshire, Rumney, nr. Cardiff, 23 Aug 1974, *Hurworth 35* (UT); Neath Port Talbot, Jul 1904, *Riddelsdell s.n.* (BM); Rhondda Cynon Taf, Hirwaun, Sep 1902, *Riddelsdell s.n.* (BM); Swansea, Whiteford Burrows, Oct 1905, *Riddelsdell s.n.* (BM); Vale of Glamorgan, Estuary of River Ogmore, Jul 1904, *Riddelsdell s.n.* (BM).

UZBEKISTAN. Samarkand, Pasdargom, Kichik-Chiboy, 14 Aug 2004, *Islomov UPL00304* (MO).

VIETNAM. Annam: Mt. Bani is in the main coast range c. 25 km from Tourane, May 1927, *Clemens* & *Clemens 3020* (BM, P). **Thua Thien-Hue**: Phu Loc Distr., Bach Ma National Park, Bach Ma mountain, Hai Vong Dai peak, 20 Apr 2003, *Hipe et al. 1129* (MO).

YEMEN. **Adan**: El Kod, 1979, *Jennings 20* (K); Western Aden Protectorate, Foot of Jebel Harir, Dhala Highlands, 31 Oct 1937, *Scott* & *Britton 240* (BM). **Ibb**: c. 1.5 mi W of Ibb, on ISAI grounds, 9 Jul 1983, *Spellenberg 7174* (K, MO). **Lahij**: Dhala town, 10 Jun 1987, *Boulos et al. 16730* (BM, E); Hinterland von Aden, 5 Apr 1939, *von Wissman 3230* (BM). **Ma'rib**: Ma'rib, 4 Apr 1981, *Miller* & *Long 3424* (E, K). **Sana'a**: Wadi Dhahr, c. 6.5 mi NW of San'a, 21 Jan 1938, *Scott* & *Britton 438* (BM).

Solanum nitidibaccatum

AUSTRALIA. Sin. loc., 16 Oct 1896, *Anderson 254* (NSW); **Australian Capital Territory**: Sin. loc., 8 Apr 1976, *Gray 6796* (AD, CANB); CSIRO Black Mountain site, Acton, Canberra, 9 Jan 1995, *Lepschi 1729* (AD, CANB, HO, NSW); **New South Wales**: Kogarah, Feb 1898, *Camfield s.n.*

(NSW); Canyonleigh via Marulan, May 1976, *Cooper s.n.* (NSW); White Rock, 8 km upstream Bathurst, 9 May 1982, *Dellon s.n.* (NSW); Cowra, Jan 1960, *Green s.n.* (NSW); Blayney Distr., 15 Feb 1951, *Madsen s.n.* (NSW); **Queensland**: The Summit c. 5 miles NNE of Stanthorpe, 8 Feb 1972, *Henderson & Parham 1241* (AD, BRI, MEL, NSW); Darling Downs, Ballandean, S of Stanthorpe, 16 Jan 2001, *Hutton 13413* (BRI); Moreton, Wolffdene, via Beenleigh, 9 Jul 1959, *Marshall s.n.* (BRI); Darling Downs, Stanthorpe, Mar 2003, *Parry s.n.* (BRI); Darling Downs, Glen Niven, Stanthorpe, on property of B. Goulter, 26 Jan 1972, *Swann s.n.* (BRI); Darling Downs, 10km W of Stanthorpe, 13 Mar 1984, *Swarbrick 7451* (BRI); 28 Sep 2005, *Tillack s.n.* (AD, BRI, MEL); **South Australia**: Sin. loc., 6 Feb 1989, *Bates 17501* (AD); Sin. loc., 25 Mar 1952, *Dunstone s.n.* (AD); Sin. loc., *Dunstone s.n.* (AD); Sin. loc., 15 Feb 1989, *Hannay s.n.* (AD); Sin. loc., 4 Mar 1993, *Hincks s.n.* (AD); Sin. loc., 20 Mar 1986, *Jackson 5976* (AD, CANB); Sin. loc., 18 Jan 1980, *McAlister s.n.* (AD); Sin. loc., 12 Mar 1974, *Spooner 3341* (AD); Sin. loc., 16 Feb 1980, *Spooner 6898* (AD); Sin. loc., 4 Apr 1968, *Symon SS-86* (CANB); Sin. loc., 4 Apr 1968, *Symon, D.E SS-91* (CANB); Sin. loc., 1 Mar 1972, *Symon 7626* (CANB); Sin. loc., 18 Jan 1980, *Winn s.n.* (AD); **Tasmania**: Sin. loc., 25 Jan 1989, *Alcock 11053* (AD); Murphys Flat Reserve, 2010 (Bush Blitz), Sin. loc., 25 Mar 2010, *Baker 2203* (HO); Sin. loc., 12 Jan 2001, *Buchanan 15827* (HO); Sin. loc., Apr 1952, *Cock s.n.* (HO); Sin. loc., 25 Jan 2001, *Couson s.n.* (HO); Sin. loc., Apr 1952, *Curtis s.n.* (AD); Sin. loc., Apr 1952, *Curtis s.n.* (AD); Sin. loc., Apr 1952, *Curtis s.n.* (HO); Sin. loc., Jan 2003, *Frost s.n.* (HO); Sin. loc., 15 Feb 2000, *Lane s.n.* (HO); Sin. loc., 13 Dec 1983, *Lane s.n.* (HO); Sin. loc., 18 Feb 1982, *Lehman s.n.* (AD, HO, MEL); Sin. loc., 31 Jan 1990, *Morris 86418* (HO, MEL); Sin. loc., 5 Mar 1999, *Roberts s.n.* (HO); Sin. loc., 29 Feb 1968, *Townrow s.n.* (HO); **Victoria**: South Eastern Highlands, 21 Feb 1986, *Albrecht 2499* (MEL); South East Corner, Jan 1981, *Barton s.n.* (MEL); Sin. loc., 27 Feb 1971, *Beauglehole 37084* (AD); Australian Alps, 27 Feb 1971, *Beauglehole 37084* (MEL); South East Coastal Plain, 5 Apr 1971, *Beauglehole 37804* (MEL); Sin. loc., 23 Apr 1973, *Beauglehole 41744* (AD); South Eastern Highlands, 23 Apr 1973, *Beauglehole 41744* (MEL); Sin. loc., 26 Apr 1973, *Beauglehole 41769* (AD); South Eastern Highlands, 26 Apr 1973, *Beauglehole 41769* (MEL); Australian Alps, 19 Jan 1981, *Beauglehole 68690* (MEL); Victorian Volcanic Plain, South West Study Area, Sector c (Private Property), 5 Jan 1985, *Beauglehole 79124* (MEL); Sin. loc., 14 Mar 1986, *Clarke 1772* (AD); Sin. loc., 6 Mar 1984, *Clarke 1682* (AD); South East Coastal Plain, 6 Mar 1984, *Clarke 1682* (MEL); Melbourne; Ripponlea Estate, Hotham Street, Elsternwick, 6 Mar 1984, *Clarke 1682* (NSW); South East Coastal Plain, 14 Mar 1986, *Clarke 1772* (MEL); Victorian Midlands, 1887, *Dickinson s.n.* (MEL); Victorian Volcanic Plain, 6 Mar 1924, *Hallebone s.n.* (MEL); Victorian Volcanic Plain, 8 Jun 1985, *LeBreton s.n.* (MEL); South East Coastal Plain, 8 Jan 2008, *Reid 2717* (MEL); Milne's property, 27 Jan 1966, *Shepherd 215* (CANB); Victorian Midlands, 12 Feb 1994, *Sniderman 10* (MEL); Victorian Midlands, 30 Mar 1964, *Willis s.n.* (MEL); Sin. loc., 16 Mar 1965, *Willis s.n.* (MEL); South East Coastal Plain, 10 Nov 1914, *Wilson s.n.* (MEL); Riverina, Jan 1975, *Without Collector s.n.* (MEL); **Western Australia**: Badgerup Road opposite Jambaris Road, Wanneroo, 15 May 1997, *Burt s.n.* (PERTH); Sin. loc., 16 May 2000, *Dempster s.n.* (CANB); Pinjarra, 17 Nov 1987, *Sheldow s.n.* (PERTH).

AUSTRIA. **Burgenland**: Nordburgenland, WSW von Eisenstadt, S von Müllendorf, ruderale Stellen nahe enem Weingartenrand c. 0.5-0.6 km N-NNE der Fölligkapelle, 2 Aug 2007, *Barta s.n.* (BM); Nordburgenland, Parndorfer Platte, Brachfeld c. 5 km E-ESE der Strassenbrücke über die Autobahn zwischen Parndorf und Neusiedl/See, 13 Sep 2009, *Barta s.n.* (BM); Seewinkel, zw. Apetion u Frauernkirchen, knapp W v E-Teil d. Fuchslochlacke, W v Feldweg, 12 Oct 2008, *Walter 7085* (W); Seewinkel, zw. Apetion u Frauernkirchen, knapp W v E-Teil d. Fuchslochlacke, W v Feldweg, 12 Oct 2008, *Walter 7086* (W). **Nieder-Österreich**: Marchfeld, nahe Gänserndorf, Ackerrand neben der Strasse 1.5-1.7 km W der Kirche von Weikendorf, 28 Sep 2010, *Barta s.n.* (BM); Wiener Becken, zwischen Baden und Bad Vöslau, überschwemmt gewesener Acker auf den Teichwiesen c. 1.5 km ESE der Kirche von Sooss, 7 Oct 2008, *Barta s.n.* (BM); Wiener Becken, E-ESE von Gramatneusiedl, überschwemmt gewesener Ackerrand c. 0.8-1 km NW-NNW der Kirche von Pischelsdorf, 9 Oct 2010, *Barta s.n.* (BM); Marchfeld, nahe Strasshof, c. 0.6 km SSW der Schnell-bahn-Haltestelle Helmahof, 10 Sep 2014, *Barta s.n.* (BM); SE-Rand des Weinvertels, unteres Kamptal, nahe Langenlois, Ackerrand am Kogelbery c. 0.85 km NW-NNW der Eisenbahn-Haltestelle Zöbing, 11 Oct 2008, *Barta s.n.* (BM); Wiener Becken, Grünbrachfläche c. 1.1-1.2 km NE der Kirche von Himburg bei Kote 172, 23 Aug 2009, *Barta s.n.* (BM); Wiener Becken, Lücken in einem

Sonnenblumenfeld c. 1.1-1.2 km NE der Bahnübergangs am N-Ende des Bahnhofs Himberg, 26 Aug 2009, *Barta s.n.* (BM); Marchfeld, W.von Deutsch-Wagram, c. 0.6-0.9 km W-WNW der Schnellbahn-Haltestelle Kapellerfeld, 27 Aug 2013, *Barta 2223* (W); Marchfeld, W von Deutsch-Wagram, c. 2.1 km WNW der Schnellbahn-Haltestelle Kapellerfeld, 2 Jul 2013, *Barta 2606* (W); Marchfeld, nahe Strasshof, c. 0.6 km SSW der Schnellbahn-Haltestelle Helmahof, 19 Sep 2014, *Barta 3064* (W); Marchfeld, con Strasshof, c. 2.1-2.25 km N-NNW der Kirche von Markgrafneusiedl, 4 Oct 2014, *Barta 3139* (W); Marchfeld, neben dem Bahnhof Deutsch-Wagram, 6 Sep 2014, *Barta 3198* (W); Marchfeld, c. 300 m W Marcheff-Bahnhof, 8 Sep 1998, *Karl s.n.* (W); Wiener Becken, Steinfeld, 7.5 km NNE Wiener Neustadt, 1.7 km NW Eggendorf, knapp WNW, 19 Sep 1998, *Walter 4189* (K, W). **Wien**: 10 Bezirk, in Kurpark Oberlau, 22 Oct 2012, *Barta 1219* (W); 22 Bezirk, knapp S der Ostbahngleitstrasse nahe der U2-Haltestelle Aspern-Nord, 6 Aug 2014, *Barta 3270* (W); 23 Bezirk, ungefahr W vom S-Ende der Jochen Rindt-Strasse, 26 Sep 2015, *Barta 4122* (W).

BELGIUM. **Wallonia**: Vesdre, Sep 1958, *Lousley s.n.* (K).

FRANCE. **Grand Est**: Bas-Rhin, Strasbourg, Graffenstaden, Oct 1961, *Patzak s.n.* (W). **Nouvelle Aquitaine**: Gironde, Bordeaux, Bassens, 24 Jul 1927, *Duffour 5538* (BM, MA); Gironde, Bassens, 9 Sep 1931, *Jallu 1038* (H, P).

GERMANY. **Hessen**: Frankfurt am Main, Frankfurt-am-Main, Hessen-Nassau, Sep 1911, *Peipers s.n.* (BM); **Niedersachsen**: Leer, Ostfriesl, Blamgelände, 10 Oct 1955, *Klimmek s.n.* (W).

IRELAND. **Leinster**: Kilkenny, Rosbercon Port, 5 Oct 1995, *Reynolds s.n.* (BM).

NETHERLANDS. **Gelderland**: Nijmegen, prov. Gelderland, 23 Sep 1928, *Kern* & *Reichgelt 14998* (BM, H).

NEW ZEALAND. Heretaunga Ecological Distr./Hawkes Bay Ecological Region/North Island Ecological Region/NZ Eco Region, *Esler s.n.* (AK); Tamaki Ecological Distr./Auckland Ecological Region/North Island Ecological Region/NZ Eco Region, *de Lange & de Lange 6292* (AK); Old Man, South Island, Otago SW of Alexandra, Conroys Dam, 8 Apr 1986, *Ogle s.n.* (WELT); Rangitikei, Marton, Tutaenui Stream, 22 Apr 2011, *Ogle 5928* (CHR); End of Harewood Road, Christchurch, 22 Mar 1958, *Without Collector s.n.* (CHR); Islington, nr. Christchurch, 14 Jan 1958, *Without Collector s.n.* (CHR); Sockburn, nr. Christchurch, 9 Feb 1958, *Without Collector s.n.* (CHR); Springston, 22 Apr 1966, *Without Collector s.n.* (CHR); Templeton, Canterbury, 26 Jan 1966, *Without Collector s.n.* (CHR); Akaroa, 15 Jan 1968, *Without Collector s.n.* (CHR); Nr Christchurch, 6 Feb 1966, *Without Collector s.n.* (CHR); Lake Bryndwr, Christchurch, 2 Apr 1968, *Without Collector s.n.* (CHR); Botany Division, D.S.I.R Lincoln, 7 Jan 1971, *Without Collector s.n.* (CHR); C.R.D D.S.I.R Lincoln, 23 Mar 1970, *Without Collector s.n.* (CHR); Canterbury, Burwood, Burwood Hospital, [Christchurch], 6 Jan 1985, *Without Collector s.n.* (CHR); Otago, Kawarau Gorge, Gentle Annie Stream, 17 Mar 1992, *Without Collector s.n.* (CHR); Akaroa, Banks Peninsula, Woodills Road, 13 Mar 1987, *Without Collector s.n.* (CHR); Canterbury, Christchurch, 19 Gleneagles Terrace, *Without Collector s.n.* (CHR); Banks Peninsula, Akaroa, 24 Feb 1996, *Without Collector s.n.* (CHR); waste land behind Fendalton Mall buildings, Fendalton, Christchurch, 2 Feb 1999, *Without Collector s.n.* (CHR); Banks Peninsula, Brocheries Road, 21 Feb 2001, *Without Collector s.n.* (CHR); Christchurch, Travis Swamp, 2 Dec 2002, *Without Collector s.n.* (CHR); Near Christchurch, 9 Dec 1940, *Without Collector s.n.* (CHR); Rattray St. Riccarton, 14 Nov 1956, *Without Collector s.n.* (CHR); Sin. loc., *Without Collector 1974 1* (CANU); Sin. loc., *Without Collector 1971 4 2* (CANU). **North Island**: Waikato, Pukekawa, 18 Dec 1986, *Dawes s.n.* (AK); Hawkes Bay, near Hastings, Heretaunga Ecological Distr., 25 Feb 1961, *Esler s.n.* (AK); Auckland, Arikikapakapa Golf Course, Rotorua Ecological Distr., Northern Volcanic Plateau Ecological Region, 15 Mar 2013, *Hobbs 13297* (AK); Auckland, Symond Street, University of Auckland Campus, vicinity of Thomas Building, Tamaki Ecological Distr., 27 Nov 2004, *de Lange & de Lange 6292* (AK). **South Island**: Canterbury, Christchurch, waste land behind Fendalton Mall buildings, Fendalton, 2 Feb 1999, *Healy 99/34* (AK, CHR).

RUSSIAN FEDERATION. Sin. loc., Sep 1949, *Blom s.n.* (CNS).

SWEDEN. **Götaland**: Västra Götaland, Göteborg, Delsjönplaget, 25 Aug 1950, *Blom s.n.* (BM); Västra Götaland, Göteborg, Ringon, 29 Sep 1951, *Blom s.n.* (K); Halland, Halmstad, Gustavsfält, 22 Sep 1955, *Blom s.n.* (BM); Halland, pr. urben Halmiam, 26 Apr 1923, *Jungner s.n.* (BM); Halland, prope usbem Halmiam, 26 Sep 1923, *Jungner s.n.* (K); Halland, Halmstad, 26 Sep 1923, *Jungner 1375* (K); Halland, 26 Sep 1923, *Jungner s.n.* (BM); Haisenborg, vid fabrikenb Kärnan, 6 Oct 1938,

Lange s.n. (BM); Skåne, Saxtorp, Flygeltofta, 28 Aug 1958, *Nilsson s.n.* (BM). Halland, Halmstad, 11 Aug 1928, *Olson s.n.* (BM).

UNITED KINGDOM. Channel Isles: Jersey, N of St. Ouen's Bay, Sep 1996, *Dupree s.n.* (BM); Jersey, 29 Aug 1930, *Williams s.n.* (BM); Jersey, 29 Aug 1930, *Williams s.n.* (BM); Jersey, 29 Aug 1930, *Williams s.n.* (BM). **England**: Essex, vice county 18, Dagenham dumps, 1938, *Airy Shaw s.n.* (K); Greater London, Wennington rd, 4 Sep 2008, *Atchison 1* (BM); Staffordshire, The Farm Institute, 24 Oct 1944, *Bates s.n.* (K); Staffordshire, The Farm Institute, 24 Oct 1944, *Bates s.n.* (BM); Greater London, Highgate, Queens Wood, Coppice Path (vc21), 11 Aug 2009, *Bevan s.n.* (BM); Warwickshire, Milverton, Sep 1893, *Bromwich s.n.* (BM); Warwickshire, Milverton, Sep 1893, *Bromwich s.n.* (BM); Warwickshire, Milverton, Sep 1893, *Bromwich s.n.* (BM); Warwickshire, Milverton, Oct 1901, *Bromwich s.n.* (BM); Suffolk, Felixstowe Dock, 4 Aug 1936, *Campbell s.n.* (BM); Bedfordshire, Flitton, 14 Oct 1950, *Doug 1401* (K); Bedfordshire, vice county 30, Beds, 22 Oct 1950, *Graham* & *Doug 1409* (K); Bedfordshire, nr Flitwick, 15 Sep 2000, *Hanson s.n.* (BM); Bedfordshire, Flitton, 8 Oct 1974, *Hanson 107 b* (BM); Bedfordshire, nr Flitwick, 16 Sep 1988, *Hanson 499* (BM); Bedfordshire, Old Warden, 19 Sep 1953, *Herb. Young s.n.* (BM); West Yorkshire, Calton Hall 1 Oct 1962, *Houseman s.n.* (BM); Nottinghamshire, Spalford, 1960, *Howitt s.n.* (K); Norfolk, Appleton, 30 Aug 1949, *Hubbard 13261* (K); Norfolk, Appleton, 30 Aug 1949, *Hubbard 13262* (K); Norfolk, Appleton, 30 Aug 1949, *Hubbard 13263* (K); Hertfordshire, Barnet, 6 Oct 1968, *Jukes s.n.* (BM); Greater London, Canal bank by soya foods Ltd., Springwell, 6 Oct 1945, *Kent s.n.* (BM); Greater London, Forecourt of Soya Foods Ltd., Springwell, 2 Oct 1948, *Kent s.n.* (BM); Greater London, Flour mill nr Springwell Lock, 17 Aug 1945, *Kent s.n.* (BM); Greater London, Forecourt of flour mill by the canal nr Springwell Lock, 1 Sep 1945, *Kent s.n.* (BM); Greater London, nr Harefield, 19 Oct 1946, *Kent* & *Sandwith s.n.* (K); Norfolk, 23 Oct 1944, *Libbey H536/44* (K); Norfolk, at Barnsey, 20 Oct 1945, *Lousley s.n.* (K); Greater London, Harefield, 1 Sep 1945, *Lousley s.n.* (K); Greater London, Harefield, 21 Sep 1948, *Lousley s.n.* (K); Bedfordshire, Flitton, 8 Sep 1957, *Lousley s.n.* (BM); Bedfordshire, Sandy, 1 Sep 1951, *Lousley s.n.* (BM); Surrey, Abinger Hammer, 2 Sep 1950, *Lousley s.n.* (BM); West Yorkshire, Ripponden, 26 Sep 1961, *Lousley s.n.* (BM); Hampshire, Blackmoor, 24 Oct 1959, *Lousley s.n.* (BM); West Yorkshire, v/c 63, 2 Oct 1957, *McCallum-Webster 1431* (K); Hampshire, Culton, Hanks, Blackmoor Fruit Farm, 8 Nov 1959, *McCallum-Webster 2142* (K); Bedfordshire, Maulden, vice county 30 Bedford, 30 Sep 1961, *McCallum-Webster 7015* (K); Surrey, Guildford tip, 5 Oct 1969, *McLean s.n.* (BM); Essex, N Dagenham, 2 Jun 1927, *Melville s.n.* (K); Greater London, Crayford, Bexley, Oct 1973, *Palmer s.n.* (BM); Kent Dartford Marshes, 9 Sep 1992, *Palmer s.n.* (BM); Hampshire, Christchurch, 22 Aug 1963, *Robinson s.n.* (BM); Berkshire, Reading, 25 Nov 1999, *Rutherford s.n.* (BM); Gloucestershire, Wapping Wharf, Bristol Harbour, 14 Sep 1942, *Sandwith s.n.* (BM); Gloucestershire, Wapping Wharf, Bristol Harbour, Oct 1942, *Sandwith s.n.* (K); Somerset, Bristol, Ashton Gate, 26 Sep 1933, *Sandwith s.n.* (K); Norfolk, Appleton, 15 Sep 1949, *Swann A* (K); Norfolk, Appleton, 15 Sep 1949, *Swann C* (K); Norfolk, Appleton, 8 Sep 1949, *Swann 1931* (BM); Norfolk, Appleton, 1 Oct 1949, *Swann K 3258* (K); Norfolk, Appleton, 1 Oct 1949, *Swann K3259* (K); Surrey, Frensham Great Pon, Aug 1945, *Warren s.n.* (BM); Surrey, Frensham Great Pon, 19 Aug 1943, *Williams s.n.* (BM); Greater London, *Wilmott s.n.* (BM). **Scotland**: Highlands, Auldearn, Broomhill Farm, 27 Oct 1967, *McCallum-Webster s.n.* (BM); Midlothian, Railway Tip, Borthwick, 20 Jul 1963, *McCallum-Webster s.n.* (BM); Midlothian, Borthwicts, 20 Jul 1963, *McCallum-Webster 8768* (K). **Wales**: Vale of Glamorgan, Barry Dock, 29 Sep 1923, *Melville s.n.* (BM).

Solanum opacum

AUSTRALIA. Sin. loc, 1770, *Banks & Solander s.n.* (BM); 25 Nov 1802, *Brown 2667* (K); Murray River, *Herrgott 116* (MEL); Dawson, *Leichhardt s.n.* (NSW); *Without Collector s.n.* (MEL); **New South Wales**: Balranald, 11 Jul 1981, *Alcock 8583* (AD); Wellington, Mt Arthur, Brennans Way, 18 Oct 1978, *Althofer 8582* (NSW); Wollongong, Bul[l]i, 1 Oct 1915, *Ashby s.n.* (AD); Botany Bay, 28 Apr 1770, *Banks & Solander s.n.* (BM); Clarence Valley, Summit of Munningyundo Mountain, between Grafton and Glen Innes, 22 Mar 2003, *Bean 20107* (NSW); Tenterfield, 19 km from Legume, towards Tenterfield, 10 Feb 2006, *Bean 24670* (NSW); Port Macquarie-Hastings,

Hastings River, *Beckler s.n.* (MEL); Oberon, Jenolan Caves, Feb 1900, *Blakely s.n.* (NSW); Purgatory Creek, 7 mls NW of Ramornie, Jul 1922, *Blakely s.n.* (NSW); Guyra, Chandlers Peak, Mar 1917, *Boorman s.n.* (NSW); Port Jackson, Sydney, 1802, *Brown s.n.* (BM); Port Jackson, Sydney, 1802, *Brown s.n.* (BM); Wentworth, Red Cliffs, Kings Billabong State Game Reserve, 7 Feb 1993, *Browne 838* (MEL); Wentworth, Red Cliffs, Kings Billabong State Game Reserve, 7 Feb 1993, *Browne 838 A* (MEL); Carrathool, Hillston, *Brueckner s.n.* (MEL); Blue Mountains, Mount Irvine, 10 Apr 1970, *Burgess s.n.* (CANB); Hawkesbury, Farm of the Hawkesbury Agricultural College, Richmond, 5 Dec 1921, *Carne s.n.* (NSW); Gloucester, Pigeon Top and track, ca. 20 km S of Nowendoc, 7 Nov 1972, *Carrick 3279* (AD); Upper Hunter Shire, Moonan Brook nr Scone, 1885, *Carter 14* (MEL); Bathurst Regional, Wambool rd, (SE of Bathurst), 7 Mar 1950, *Constable s.n.* (AD); Tenterfield, Girard State Forest, 3 mls W of Drake (E of Tenterfield), 28 Apr 1956, *Constable s.n.* (AD); Oberon, Jenolan Caves, 10 Mar 1950, *Constable s.n.* (NSW); Bathurst Regional, Wambool Rd, 7 Mar 1950, *Constable s.n.* (NSW); Goulburn Mulwaree, Heap's Gully, 3 mls SE of Bungonia Caves, 24 Jan 1956, *Constable s.n.* (NSW); Tenterfield, 3 mls [4.8 km] W of Drake, 28 Apr 1956, *Constable s.n.* (NSW); Kanangra Deep, Kanangra, 10 mls SE of Jenolan Caves, 23 May 1965, *Constable 5866* (NSW); Jenolan Caves Road, 10 Mar 1950, *Constable 11096* (K); Walcha, Moona Plains, Jan 1885, *Crawford 372* (MEL); Singleton Army Area 6, 1.8 km N of Rothbury Creek, 30 Nov 1991, *Crawford 1557* (CANB); Richmond Valley, Richmond River, *Fawcett 112* (MEL); Griffith, Apr 1943, *Fraser s.n.* (NSW); Wooloondool, 7 mls from Hay, Feb 1916, *Froggatt s.n.* (NSW); Sans Souci, nr Goerges River, 27 Sep 1961, *Goode 346* (K, NSW); Kyogle, Toonumbar State Forest, c. 26 km NW of Kyogle, 22 Feb 1972, *Henderson 1261* (AD, AD, NSW); Ca 10 km NE of Narrabri, 23 Sep 1975, *Henderson 2315* (AD, K); Wentworth, junction of Darling & Murray R, May 1887, *Holding s.n.* (MEL); Wentworth, junction of Darling & Murray R, *Holding s.n.* (MEL); Upper Hunter Shire, Ben Halls Gap State Forest, 20 Feb 1991, *Hosking 307* (NE); Australasia Montes Coerulei, *Hügel s.n.* (W); Guyra, Howell, 30 Nov 1994, *Hunter 2593* (NE); Armidale Dumaresq, Parlour Mt, NW of Armidale, c.3 km N Longford CSIRO, 28 Mar 1995, *Hunter 2921* (NE); Glen Innes Severn, Butterleaf State Forest, c. 20 km NE of Glen Innes, Mt Scott, 18 Jun 1996, *Hunter 3926* (NE); Mid-Western Regional, Mudgee, Mar 1908, *Inspector of Stock s.n.* (NSW); Wollongong, Bulli, 1875, *Johnson s.n.* (MEL); Cooma-Monaro, Tinderry Range, Michelago to Captains Flat rd, c.7 km ESE ofMichelago, 7 Apr 1993, *Lepschi & Puttock 974* (CANB); Southalping, 15 mls E of Tenterfield, 10 Apr 1963, *MacDonald 233* (K); Wakool, Lower Edwards River, *Mein s.n.* (MEL); Tamworth Regional, Murrurundi, Jan 1948, *Middleton s.n.* (CNS); Wollongong, Mt Keira, 27 Jul 1983, *Mills s.n.* (WOLL); Bogan [River], 1888, *Morton s.n.* (MEL); Balranald, China Walls, N of Tooma River, 17mls SE of Tumbarumba, 12 Feb 1964, *Muir 3244* (MEL); Shoalhaven, ca. 78 km from Braidwood on Braidwood-Nowra rd, 13 Aug 1984, *O'Ryan 39* (NSW); Great Lakes, Seal Rocks, 5 Aug 1968, *Rodd 689* (NSW); Bowen Island, Jerris Bay, 25 Dec 1931, *Rodway 645* (K); Firie Islands, small rocky islands off Port Kembla, 10 Mar 1936, *Rodway 2140* (K); Bowen Island, Jervis Bay, 27 Dec 1931, *Rodway 6487* (NSW); Blue Mountains, Port Jackson, Mt Wilson, 3 Jun 1884, *Siegert 79* (MEL); East Australia, 1865, *Statter s.n.* (BM); Tenterfield, Demon Creek, *Stuart s.n.* (MEL); Tenterfield, Clear Creek, *Stuart 77* (MEL); Tenterfield, Timbarra, *Stuart 784* (MEL); Tenterfield, 29 Nov 2007, *Thompson 168* (BRI); 9.6 km E of Brewarrina on Nyngan Rd, 31 Aug 1971, *Thompson 1138* (NSW); Cowra, 7 Apr 1976, *Toth 8* (NSW); Dungog, Williams River Rd, Allyn River State Forest, 15 Aug 1969, *Verdon 121* (CANB); Gosford, Bouddi Range, 27 Dec 1965, *Whitehead s.n.* (AD); The Hills Shire, Dundas, nr Vineyard Creek, F. Robertson Park off Kissing Point Road, 20 Jul 1972, *Williams s.n.* (NSW); Blue Mountains, Royal Botanic Gardens, Mount Tomah Annexe, 4 Jun 1985, *Wilson 19* (NSW); Cobar, Sandy Crowl Creek, 21 km N of Nymagee junction on Mount Hope-Cobar rd, 23 Mar 1984, *Wilson 5881* (NSW); Windsor District, Oct 1924, *Without Collector s.n.* (K); Tamworth Regional, Bowling Alley Point, 4 Apr 1990, *Without Collector s.n.* (NE); Hawkesbury, Farm of the Hawkesbury Agricultural College, Richmond, 29 May 1923, *Without Collector s.n.* (NSW); Armidale Dumaresq, Cooney, c. 20 km E of Armidale along Waterfall Way, 10 Apr 2004, *Without Collector 13* (NE); Inverell, nr Kings Plains Creek, Kings Plains National Park, 27 Apr 1994, *Without Collector 1842* (NE); Bega Valley, Tanja (Bega) distr., 25 Oct 1940, *Without Collector 40/1979* (NSW); Glen Innes Severn, Warra SF, E Llangothlin, 21 Feb 1995, *Without Collector 2694*

(NE); Glen Innes Severn, Warra SF, E of Llangothlin, Crown Mtn FLR entrance, 21 Feb 1995, *Without Collector 2703* (NE); The Hills Shire, Parramatta, *Woolls s.n.* (MEL); Gilgandra, Barrangan beyond Mudgee, *Woolls 49* (MEL). **Norfolk Island**: sin. loc, Oct 1805, *Caley s.n.* (BM); Rocky Point Reserve, 6 Oct 1989, *Gardner 5866* (AK); in residence in village of Burnt Pine, Mar 1971, *Harley s.n.* (K); (HMS Herald - No. 2 Norfolk), 3 Jan 1865, *Milne 2* (K); Kingston, 24 Aug 1964, *Uhe 1126* (K). **Northern Territory**: Adelaide River, 18 Dec 1972, *Henderson 1356* (CANB, K). **Queensland**: Etheridge, Einasleigh and Gilbert River, *Armit 682* (MEL); Toowoomba, *Bailey s.n.* (BRI); Southern Downs, 5 Dec 2001, *Batianoff 2001- 1234* (BRI); Southern Downs, 28 Nov 2001, *Batianoff 2001-11222* (BRI); Whitsunday, 10 Jul 1992, *Bean 4736* (BRI); Southern Downs, 11 Apr 1999, *Bean 14804* (BRI); Gold Coast, 13 Jun 2003, *Bean 20521* (BRI); Balonne, 9 Sep 2005, *Bean 24318* (BRI); Toowoomba, 16 Feb 2008, *Bean 27321* (BRI); Gympie, 9 Feb 2009, *Bean 28602* (BRI); Lockyer Valley, 18 Sep 2010, *Bean 30336* (BRI); Scenic Rim, 31 Dec 2010, *Bean 30722* (BRI); Southern Downs, 10 Dec 2015, *Bean 32481* (BRI); Blackall Tambo, Mt Playfair, 1890, *Biddulph 14* (MEL); Blackall Tambo, Mar 1961, *Biddulph 186* (BRI); nr border of Queensland/New South Wales, Main Range National Park, Spring Creek Road, E of Killarney, 10 Feb 2006, *Bohs et al. 3561* (BM, UT); Toowoomba, 18 Jun 2003, *Butler s.n.* (BRI); Southern Downs, Wallangarra, Apr 1924, *Cheel s.n.* (NSW); Gladstone, Many Peaks Range, ca 40 km SE of Gladstone, 0.5 km WSW of Mt Castletower, 2 Jun 1977, *Crisp 2757* (CANB); Charters Towers, Sea View Range, tops of the range, Rockingham Bay, 4 Jun 1865, *Dallachy s.n.* (MEL); Rockhampton, Aug 1865, *Dietrich 466* (MEL); Rockhampton, Feb 1866, *Dietrich 827* (MEL); Gladstone, *Dietrich 1049* (MEL); Southern Downs, 8 Feb 1960, *Everist 6160* (BRI); North Burnett, 1.5 km NNE of Didcot, 31 Oct 1995, *Forster 17931* (MEL); Southern Downs, 23 Oct 2013, *Forster 40548* (BRI); Somerset, 11 Feb 2015, *Forster 42049* (BRI); Southern Downs, 18 Mar 2015, *Forster 42140* (BRI); Gold Coast, 21 Oct 2015, *Forster 43098* (BRI); Birnam Range 7.5 km NE of Beaudesert, Tremayne Rd, Jan 2001, *Halford Q- 3882* (BRI, K, MEL, NSW); Burnet, *Haly s.n.* (MEL); Moreton Bay, 21 Apr 1967, *Henderson 246* (BRI); South Burnett, 2 May 1967, *Henderson 250* (BRI); South Burnett, Benarkin State Forest, Blackbutt, 8 Aug 1967, *Henderson 285* (AD, BRI); South Burnett, 8 Aug 1967, *Henderson 290* (BRI); South Burnett, 8 Aug 1967, *Henderson 291* (BRI); South Burnett, 8 Aug 1967, *Henderson 292* (BRI); Toowoomba, Bunya National Park, c. 48 km NE of Dalby, 28 Nov 1967, *Henderson 302* (AD, BRI, MEL, NSW); Scenic Rim, The Head, c. 45 km ESE of Warwick, 22 Feb 1968, *Henderson 359* (AD, BRI, NSW); Southern Downs, 7 Mar 1968, *Henderson 394* (BRI); Banana, 12 Mar 1968, *Henderson 396* (BRI); Moreton Bay, 27 Jun 1968, *Henderson 402* (BRI); Moreton Bay, Mt. Mee, c. 56 km NNW of Brisbane, 27 Jun 1968, *Henderson 403* (AD, BRI); Scenic Rim, Levers Plateau, on Qld/NSW border, c. 90 km SSW of Brisbane, 5 Apr 1972, *Henderson 1290* (AD, BRI); ca 13 km WNW of Goondiwindi on rd to St. George, 30 Sep 1975, *Henderson 2357* (AD, BRI, K); Isaac, 17 Oct 1983, *Henderson 2922* (BRI); Scenic Rim, 4 May 2003, *Hines s.n.* (BRI); Western Downs, Jimba, plains of the Condamine, 30 Sep 1844, *Hodgson s.n.* (NSW); Cairns, Alongside Wright Creek, S of Edmonton, 13 Nov 1994, *Hosking 1022* (NSW); Main Range, between Spring Bluff and Murphy's Creek, 2 Aug 1930, *Hubbard 3535* (K, L); Townsville, Cleveland Bay, 1876, *Johnson s.n.* (MEL); Townsville, Cleveland Bay, 1876, *Johnson s.n.* (MEL); Western Downs, 7 Jul 1955, *Johnson 70* (BRI); Darling Downs Distr., Bell-Bunya Mountains rd, about 3 mls S of Mt Mowbulland Quest House, 3 May 1958, *Johnson 462* (BRI, CANB, K); Brisbane, Mt Gravatt, Oct 1956, *Jones 238* (CANB); Mackay, 25 May 2003, *Kemp TH7330* (BRI); Sunshine Coast, May 1910, *Keys s.n.* (BRI); Blackall Ranges, May 1910, *Keys s.n.* (NSW); Moreton District, Kenmore, Brisbane, 24 Jan 1965, *Kleinschmidt s.n.* (K); Moreton Distr., Kenmore, 17 Jul 1960, *Kleinschmidt s.n.* (K); Moreton Bay, Kenmore, Brisbane, 24 Jan 1965, *Kleinschmidt s.n.* (AD); Brisbane, 24 Jan 1965, *Kleinschmidt s.n.* (BRI); Brisbane, KENMORE, 17 Jul 1960, *Kleinschmidt s.n.* (BRI); Brisbane, Kenmore, 17 Jul 1960, *Kleinschmidt s.n.* (CANB); Brisbane, Kenmore, 24 Jan 1965, *Kleinschmidt s.n.* (MEL, NSW); Western Downs, Jimba, 30 Sep 1844, *Leichhardt s.n.* (NSW); Toowoomba, 16 Dec 2011, *Menkins ILM- 584* (BRI); Whitsunday, 10 Dec 1919, *Michael 694 A* (BRI); South Burnett, 23 May 1947, *Michael 3026* (BRI); Rockhampton, 22 Apr 1867, *O'Shanesy 71* (MEL); Darling Downs Distr., Rockwood, ca. 20 mls SW of Chinchilla, 20 Nov 1969, *Pedley 3012* (K); Moreton Bay, 5 Oct 2014, *Phillips 2524* (BRI); Longreach, Barcoo, B. Downs, 1870, *Schneider s.n.* (MEL); Brisbane, 8 Dec 1888, *Simmonds s.n.* (BRI); South Burnett, 13

Apr 1968, *Smith 13884* (BRI); Rockhampton, 18 Feb 1980, *Stanley 602* (BRI); Western Downs, 11 Jun 1958, *Stewart 3* (BRI); Brisbane, 4 Aug 1998, *Thomas s.n.* (BRI); Barcaldine, Jericho, 1890, *Walker s.n.* (MEL); Tablelands, 18 May 1970, *Webb 10256* (BRI); Brisbane, ENOGGERA, Mar 1916, *White s.n.* (BRI); Brisbane, ENOGGERA, Jun 1919, *White s.n.* (BRI); Brisbane, Enoggera, Jun 1919, *White s.n.* (NSW); Brisbane, Enoggera nr Brisbane, Mar 1916, *White s.n.* (NSW); Moreton Distr., Wilson's Peak, 19 Apr 1949, *White 13009* (BRI, CANB, K); Sunshine Coast, Dec 1971, *Wilson s.n.* (BRI); Ipswich, Pine Mountain, Feb 1982, *Without Collector s.n.* (NE). **South Australia**: Mid Murray, Murray River, Purnong Landing, Apr 1980, *Bates 689* (AD); Renmark Paringa, 15 May 1989, *Bates 18455* (AD); Kangaroo Island, Breakneck R[iver], 21 Dec 1992, *Bates 30341* (AD); Kangaroo Island, 22 Dec 1992, *Bates 30742* (AD); Renmark Paringa, Murtho Native Forest Reserve, 9 Nov 2011, *Duval 2308* (AD); Calperum Station, S shores of Lake Merreti, 12 Jan 2012, *Duval 2330* (AD); Mid Murray, Murray River W bank, c. 1.5 km S of Moorundie, 24 Feb 2012, *Guerin 18* (AD); Kangaroo Island, Ravine des Casoars, 15 Jan 1983, *Jackson 1582* (AD); Adelaide Hills, Ad ripas Torrens prope montum Lofty, 25 Dec 1847, *Mueller s.n.* (MEL); Norwood Payneham St Peters, Third Creek, 10 Jan 1848, *Mueller s.n.* (MEL); Adelaide Hills, Ad. rivul. Mont Lofty-ranges, Nov 1850, *Mueller s.n.* (MEL); Mid Murray, Murray [River], 1850, *Mueller s.n.* (MEL); Bet[ween] Flinders Range and Lake Torrens, *Richards s.n.* (MEL); Bet[ween] Flinders Ranges and Lake Torrens, *Richards s.n.* (MEL); Purnong Landing, 23 Nov 1980, *Symon s.n.* (AD); Purnong Landing, 23 Nov 1980, *Symon s.n.* (AD); Renmark Paringa, Upper River Murray. Due W of Queens Bend, 13 Sep 1979, *Symon 11578* (AD); Upper River Murray nr 375 mile peg, c. 4 mls downstream from Little Hunchee Island, 16 Sep 1979, *Symon 11586* (AD, CANB, K); Renmark Paringa, Upper River Murray, 26 May 1980, *Symon 12144* (AD, AD, CANB); Renmark Paringa, Upper River Murray, 28 May 1980, *Symon 12151* (AD); Purnong Landing, River Murray, 6 Nov 1980, *Symon 12829* (AD); River Murray, Wiela Station opposite Chowilla Station, 9 May 1987, *Symon 14342* (AD); Mid Murray, N of Moorundie, 13 Jan 2012, *Thorpe 377* (AD); Mid Murray, S of Page Drive in Blanchetown, 13 Jan 2012, *Thorpe 384* (AD); Kangaroo Island, *Waterhouse s.n.* (MEL); Berri and Barmera, Murray River, 25 Dec 1913, *Without Collector s.n.* (AD); River Murray, c. 2.5 km WSW of Murtho Park Homestead, c. 1.5 km N of Warwilla Homestead, 15 Sep 1979, *Womersley & Symon 564* (AD). **Tasmania**: Break O'Day, St Mary's Pass, 16 Dec 1980, *Alcock 8528* (AD); George Town, George Town, 12 Jan 2005, *Baker 1450* (HO); Burnie, *Black s.n.* (MEL); Tasman, Port Arthur, 1893, *Bufton 11* (MEL); Tasmania, *Burbury s.n.* (HO); sin. loc, 1842, *Gunn 51 [a]* (BM); George Town, Point Effingham, nr Bell Bay, 5 Jul 1843, *Gunn 51 [b]* (HO); Latrobe, Harford on Solomans Hill, 6 Nov 1932, *Hamilton 163* (CANB, HO); King Island, *Neate s.n.* (MEL); Kentish, 9.6 km from Gowrie Park, on rd to Cradle Mountain, 3 Nov 1980, *Pearce 160* (AD); Kingborough, North Bruny, Jan 1931, *Rodway 121* (CANB); Flinders, Inner Sister Island, 6 Dec 2010, *Visoiu s.n.* (HO); King Island, The Nook swamp, 13 Dec 2011, *Visoiu s.n.* (HO); King Island, nr Sea Elephant River, 23 Mar 2009, *Wapstra et al. 691* (AD); King Island, nr Sea Elephant River, 23 Mar 2009, *Wapstra 691* (HO); Flinders, Freestone Bay, Deal Island, Kents Group, 8 Dec 1971, *Whinray 1085* (AD); Van Diemen's Land, Gunn, 1835, *Without Collector s.n.* (K). **Victoria**: Colac-Otway, Carlisle Heath, Tuckers Orchard Rd, 500 m W of Gellibrand - Lavers Hill Road, 2 Jul 2005, *Adair 3341* (MEL); Colac-Otway, Between Glen Aire and Johanna Bay, Southern Otways, 13 Apr 1963, *Allender s.n.* (MEL); East Gippsland, Wingan Inlet National Park, W of Wingan Track and Inlet, 23 Nov 1969, *Beauglehole 32006* (MEL); East Gippsland, Bonang - Wulgulmerang Road. 3.7 mls W of Tubbut, 5 Jan 1970, *Beauglehole 33110* (MEL); East Gippsland, Snowy River, nr junction with Stony Creek, 3 Dec 1970, *Beauglehole 35137* (AD); Yarra Ranges, Dandenong Block. Woori Yalock Picnic Ground Area. c. 0.5 Km NW of Yellingbo, 22 Mar 1976, *Beauglehole 50427* (MEL); Wellington, The Lakes National Park, nr Lake Reeve Lookout, 19 Dec 1978, *Beauglehole 62813* (AD, MEL); East Gippsland, S of Monument Ridge, NE of Amboyne settlement. 13km NW of Tubbut Post Office, 22 Jan 1980, *Beauglehole 67603* (MEL); East Gippsland, Buchan - Murrindal Roadside Reserve, 13 Sep 1984, *Beauglehole 76965* (MEL); East Gippsland, Buchan - Murrindal Roadside Reserve, 13 Sep 1984, *Beauglehole 76966* (MEL); Wellington, Freestone Creek Natural Feature Zone, 28 Sep 1984, *Beauglehole 77426* (MEL); Wellington, Avon - Mount Hedrick Natural Feature - Scenic Reserve, 5 Oct 1984, *Beauglehole 77854* (MEL); Wellington, Glenmaggie Regional

Park, 19 Oct 1984, *Beauglehole 78536* (MEL); Mildura, Hattah/Kulkyne National Park, 11 Feb 1989, *Browne 574* (AD, MEL); Mildura, Hattah/Kulkyne National Park, 4 Mar 1993, *Browne 853* (MEL); Mildura, Woods Lagoon, 18 Km W of Merbein township, 10 Nov 1993, *Browne 912* (MEL); East Gippsland, Musk Gully nr Melwood, 9 Mar 1975, *Cameron 3136* (MEL); East Gippsland, Buldah Forest Block, 600 m NE of Mt Petterson. Buldah Road, 1 Km S of Buldah Gap Road, 28 Oct 1984, *Carr 10138* (AD, MEL); East Gippsland, Orbost Region, 16 Feb 1986, *Chesterfield 852* (MEL); East Gippsland, 18 Mar 1987, *Chesterfield 1869* (MEL); Greater Geelong, Portarlington, 1870, *Dickinson s.n.* (MEL); East Gippsland, Ellery Forest Block, Pumpkin Hill Track nr Brodribb River, 15 Jan 1987, *Earl 352* (MEL); East Gippsland, Murendel River, Gippsland, 1882, *Howitt 411* (MEL); Golden Plains, Meredith, 1883, *Johnsone 1* (MEL); Alpine, c. 50 m downslope from Eurobin Picnic Area, Mt Buffalo access road, c. 12 km WNW of Bright, 1 Jan 2003, *Lepschi 4916* (CANB, MEL); East Gippsland, Gabo Island, 1870, *Maplestone s.n.* (MEL); Towong, Snowy Creek, *Martin s.n.* (MEL); Buloke, Shores of Lake Buloke, N of Donald, 4 Jul 1990, *Robb s.n.* (MEL); East Gippsland, Marlo, S of Orbost, 1937, *Robbins ACB- 17123* (MEL); Latrobe, Pine forest off Downies Lane at Traralgon South, 18 Jul 1988, *Thompson 157* (AD, AD, MEL); East Gippsland, Jones Creek (jungle), 9 km ENE of Cooaggalah Hill, Oct 1983, *Walsh 999* (AD, MEL); Yarra Ranges, Dandenong Ranges. Sherbrooke Forest, 4 Feb 1989, *Walsh 2254* (MEL); East Gippsland, Snowy River National Park, cliffs c. 0.6 km NNE from Mt William, 4 Nov 1996, *Walsh 4629* (MEL); Cedar Creek (Bentleigh district), 12 Feb 1920, *White s.n.* (NSW); Austral. Felix, *Without Collector s.n.* (CANB). **Western Australia**: Granite slab c. 200 m N of Chesapeake Rd, c. 1.5 km E of Mt Chuladup, 7 Dec 2001, *Barker 8355* (AD); Eyre, S of Cocklebeddy, 5 Dec 1962, *MacDonald 29* (K); Bayswater, Lower Swan River, 20 Mar 1909, *Morrison 19014* (K).

CHILE. **Región V (Valparaíso)**: Easter Island, *von Chamisso s.n.* (F, NY); Juan Fernández Islands, Masafuera, Quebrada de Veradero, 12 Mar 1917, *Skottsberg* & *Skottsberg 568* (GOET).

COOK ISLANDS. **Mitiaro**: just before entering swamp on rd to Atai Foodland, 22 Jul 1991, *Luttrell 187* (FHO); rd from the village of Takaue to the marsh, 24 Apr 1985, *Whistler 5576* (US).

FIJI. Yuen Yick's farm, Nasinu, Naitasiri, 3 Dec 1957, *Ledua s.n.* (K); sin. loc., 1860, *Seemann 344* (BM, K). **Fulanga**: Limestone Formation, 22 Feb 1934, *Smith 1174* (K). **Kandavu**: Mount Mbuke Levu, 23 Oct 1933, *Smith 206* (K). **Viti Levu**: Falls, Mt. Evans, Lantoka, 3 Oct 1920, *Greenwood 105* (K); Mba, vicinity of Nalotawa, E base of St. Evans Range, 28 Apr 1947, *Smith 4310* (US); Hills E of Wainikoroiluva River, nr Namuamua, Namosi, 15 Oct 1953, *Smith 9070* (K, US); Mba, summit of Mt. Nanggaranambuluta E of Nandarivatu, 19 Jun 1947, *Smith 4849* (K, US).

FRENCH POLYNESIA. **Austral Islands**: Rurutu, du N de l'ile, 17 Apr 1981, *Hallé 7032* (US); Rurutu, N Moerai, 25 Apr 1981, *Hallé 7321* (US); Raivavae, Vaiuru, 10 Aug 1934, *Fosberg 11756* (US); Rapa Iti, Titikaveka, E side of Mt. Vaitau, 5 Jul 1934, *St. John 15383* (US); Marotiri, Southeast Islet, 22 Jul 1934, *St. John 15682* (US); Raivavae, Ahuoivi, 9 Aug 1934, *St. John 16070* (US); Tubuai, Mata'ura, 15 Aug 1934, *St. John* & *Fosberg 16310* (US). **Gambier Islands**: Mangareva, 1833, *Le Guillou s.n.* (US); Aukena, Koiovao, 29 May 1934, *St. John 14659* (US). **Society Islands**: Tahiti, 6 Apr 1840, *Barclay 3309* (BM); Tahiti, plateau de Taravao, sentier du captage de l'Hamoa, 16 Sep 1982, *Florence 3853* (US); Tahiti, Mar 1826, *Lay* & *Collie s.n.* (BM); Tahiti, Mar 1826, *Lay* & *Collie s.n.* (BM); Raiatea, first valley S of Uturoa, 14 Oct 1926, *Moore 212* (US); Tahiti, 1850, *Ribourt s.n.* (US); Me'et'ia, Fatia-po to Fareura, 12 May 1934, *St. John 14187* (US).

INDONESIA. **Bali**: Kintamani, 14 Nov 1966, *Schwabe s.n.* (B). **East Nusa Tenggara**: Flores, nr Mataloko, *Verheijen 24* (L); Flores, nr Mataloko, *Verheijen 191* (L). **Papua**: Mimika Regency, PT-Freeport Indonesia Concession Area, km 86, 9 Aug 1998, *Johns et al. 9565* (A, K); Iliamik Village, Snow Mountains region, Oct 1992, *Milliken 1581* (K); Mimika Regency, W and above Tembagapura, 23 Aug 1998, *Sands 7296* (K). **Sulawesi**: Central Sulawesi, Sopu valley, 30 May 1975, *Balgooy 3555* (A, K). **West Papua**: West-Irian, Eipomek-Tal, bei Malingdam, 24 Feb 1976, *Hiepko* & *Schutze-Motel 1195* (B).

JAPAN. Nagasaki Pref., Katamatsuura-gun, Emukae-cho, Okugawachi-men, 17 Aug 1994, *Yonekura 3230* (MO).

MALAYSIA. **Sabah**: Kampung Tiung, side of hill Tiung Valley, Tuaran Distr., 8 Aug 1985, *Maikin Lantoh SAN-108976* (K).

MARQUESAS ISLANDS. **Hatutaa Island**: vallon de la pointe SW, 10 Jul 1988, *Florence & Teikiteetini 9399* (US).

MARSHALL ISLANDS. **Arno Atoll**: Ine village, 12 May 1950, *Anderson 3672* (L, US). **Jaluit Atoll**: Jabor, 28 Apr 1958, *Fosberg 39475* (US). **Kwajalein Atoll**: Kwajalein Islet, 19 Jan 1950, *Fosberg 31180* (US).

NEW CALEDONIA. sin. loc, *Caldwell s.n.* (K); NO de la Nouvelle Caledonie, 1867, *Krieger s.n.* (W). **Nord**: Ilôt Poudiou, observatory Island, Isle of Pines, Oct 1853, *MacGillivray 805* (K); **Sud**: Nouméa, Îlot Freycinet, Ilot de Freycinet, Aug 1884, *Grunow s.n.* (W); Noumea, Baie Tina, 24 Jul 1973, *MacKee 26964* (K).

NEW ZEALAND. sin. loc, 1768, *Banks & Solander s.n.* (BM, E); sin. loc, 1768, *Banks & Solander s.n.* (BM); sin. loc, 1768, *Banks & Solander s.n.* (BM); sin. loc, 1768, *Banks & Solander s.n.* (BM); sin. loc, 1768, *Banks & Solander s.n.* (US); sin. loc, *Colenso 57* (K); sin. loc, *Hooker s.n.* (BM); *Hooker s.n.* (G-BOIS); *Hooker s.n.* (G-BOIS); sin. loc, *Hooker s.n.* (K); sin. loc, *Hooker s.n.* (W); Sin. loc, 1838, *Hooker s.n.* (K); sin. loc, *Hügel s.n.* (W); sin. loc., *Logan s.n.* (K); sin. loc, *Without Collector*, *s.n.* (W). **Chatham Islands**: Rekohu, (Chatham Island), Waitangi, 1 Dec 2008, *de Lange & Horne CH-2032* (AK). **Kermadec Islands**: Macauley Island, Southern Kermadec Islands Group, 29 Jun 2006, *Barkla M-26* (AK); Raoul Island, 24 Nov 1994, *Cameron 7914* (AK); Sunday Island, Aug 1887, *Cheeseman s.n.* (AK); Sunday Island, Aug 1887, *Cheeseman s.n.* (AK); Dayrell Island, Herald Island Group, Northern Kermadec Islands, 18 May 2011, *de Lange K-1318* (AK); Curtis Island, Nov 1900, *Shakespear s.n.* (AK); Raoul Island, 13 Nov 1966, *Uhe s.n.* (AK). **North Island**: Thames probably, *Adams s.n.* (AK); Northland, Motu Muka Island, Hen and Chickens Group, 25 Aug 1964, *Beever & Jane s.n.* (AK); Northland, Motuarohia Island, Bay of Islands County, 7 Jan 1980, *Beever 80-109* (AK); Northland, Mangonui County, 25 Sep 1985, *Bellingham 0144* (AK); Waikato, Ruamahuanui, 9 Nov 2007, *Bellingham 1840* (AK); Northland, Hokianga Harbour, 26 Oct 1973, *Braggins s.n.* (AK); Auckland, Tiritiri Matangi Island, 28 May 1981, *Cameron 532* (AK); Waikato, Middle Island, Mercury Group, 16 Dec 1983, *Cameron 2555* (AK); Auckland, Laingholm, 10 Oct 1999, *Cameron 9894* (AK); Auckland, Laingholm, E side of Victory Road, 10 Oct 1999, *Cameron & Hatch 9895* (AK); Auckland, Papakohatu, (Crusoe Island), 18 Sep 2009, *Cameron 15216* (AK); Waikato, Korapuki Island, Mercury Islands, Coromandel Region, 30 Nov 2010, *Cameron & Bellingham 15643* (AK); Auckland, Mount Eden, near Auckland, *Cheeseman s.n.* (AK); Waikato, Orere Point, 10 Mar 1965, *Cooper s.n.* (AK); Waikato, Slipper Island, 19 Aug 1973, *Court s.n.* (AK); Waikato, Motuoruhi, (Goat Island), Camp Bay, off Coromandel County, 14 Dec 1970, *Dickson s.n.* (AK); Wellington, 24 Mar 2002, *Enright s.n.* (AK); Auckland, Mount Albert, 21 Jan 1970, *Esler s.n.* (AK); Northland, Waitiki Stream, Mangonui County, 11 Jun 1993, *Forester s.n.* (AK, HO); Northland, Matapia Island, 21 May 1993, *Forester s.n.* (AK); Northland, Lake Omapere, 15 Mar 2007, *Forester s.n.* (AK); sin. loc, *Gourlie s.n.* (BM); Auckland, Mt. Eden, 29 Jan 1990, *Hampton s.n.* (AK); Northland, Motu Muka Island, Hen and Chickens Group, 25 Aug 1964, *Hynes s.n.* (AK); Auckland, Little Barrier Island, 5 Sep 1963, *Hynes s.n.* (AK); Auckland, Auckland, *Kirk s.n.* (AK); Taranaki, 12 May 1992, *de Lange 1361* (AK); Waikato, Lake Koraha, Otorohanga County, 2 Dec 1987, *de Lange s.n.* (AK); Hamilton City, Waikato County, 20 Mar 1989, *de Lange s.n.* (AK); Waikato, Waikato County, 12 Jan 1988, *de Lange s.n.* (AK); Waikato, Lake Whangape, Raglan County, 4 Mar 1988, *de Lange s.n.* (AK); Waikato, Lake Koraha, Otorohanga County, 2 Dec 1987, *de Lange s.n.* (AK); Waikato, Te Kauwhata area, 25 Jan 1988, *de Lange s.n.* (AK); Auckland, Tarahiki Island, 19 Oct 1994, *de Lange & McFadden 2914* (AK); Waikato, Ruamahua-iti, Aldermen Islands off Coromandel Coast, 19 May 1972, *Lynch & Court s.n.* (AK); Northland, Whangarei, 5 Dec 1949, *Mason s.n.* (AK); Waikato, Channel Island, Coromandel County, 6 Feb 1986, *McFadden s.n.* (AK); Bay of Plenty, Whale Island, 29 May 1984, *Ogle 7* (AK); Bay of Plenty, Whale Island, 29 May 1984, *Ogle 37* (AK); Northland, Forest Road, ca. 5 km E of Rangiahua, Bay of Islands County, 10 Oct 1972, *Orchard 3516* (AK); Northland, ca. 10.5 km due W of Kaeo, 14 Oct 1972, *Orchard 3594* (AK); Northland, Russell State Forest, Bay of Islands County, 30 Nov 1972, *Orchard 3778* (AK); Waikato, Franklin County, 26 Aug 1973, *Orchard 4008* (AK); Northland, Mt. Aubrey, Aug 2005, *Parr s.n.* (AK); Waikato, Mayor Island, Feb, *Phillips-Turner s.n.* (AK); Auckland, Little Barrier Island, 1900, *Shakespear s.n.* (AK); Auckland, Little Barrier Island, 1897, *Smith s.n.* (AK); Auckland, Little Barrier Island, 1897, *Smith s.n.* (AK); Auckland, Great Barrier Island, 25 Nov 1986, *Sykes 276/86* (AK); Northland, Herokino State Forest, 28 Jan 1984, *Taylor s.n.* (AK); Taranaki, Sugarloaf Island, 14 May

1976, *Wright 1252* (AK); Auckland, Waitemata County, 26 Jan 1977, *Wright 1698* (AK); Northland, Hen Island, Hen and Chickens Islands, 1 Sep 1977, *Wright 2189* (AK); Northland, Stephenson Island, Whangaroa County, 27 Aug 1982, *Wright 5034* (AK); Waikato, Mokukaramarama (Bush) Island, Coromandel County, 30 Aug 1983, *Wright s.n.* (AK); Waikato, Mayor Island, 18 Nov 1981, *Wright 4237* (AK); Waikato, Pirongia State Forest, 19 Feb 1984, *Wright 6271* (AK). **Outlying Islands**: Three Kings Islands, Great Island, 19 Apr 1946, *Turbott & Bell s.n.* (AK). **South Island**: Motuara, *Banks & Solander s.n.* (BM). **Tokelau Territory**: Fakaofu, Union Islands, Mar 1891, *Lister s.n.* (K).

PAPUA NEW GUINEA. 18 km NE of Lake Habbema camp, Bele River, Nov 1938, *Brass 11277* (A); Boridi, 6 Sep 1935, *Carr 12985* (BM, K); Utakwa River to Mt. Carstensz, camp vib, 12 Feb 1913, *Kloss s.n.* (BM); Wiligimaan, Baliem, Div. Hollandia, 28 Jun 1961, *Versteegh BW-12516* (A, L); Noreikora Swamp, nr Kainantu, Eastern Highlands Distr., Apr 1966, *Wheeler ANU-5730* (A, K). **Bougainville Island**: Namatea, NW Bougainville, 7 Mar 1932, *Waterhouse 691 B* (K, L); Crown Prince Mountain, Oct 1960, *Womersley NGF-13346* (A). **Central**: E side lake Myola No. 2, Distr. Central, Subdistr. Port Moresby, 13 Sep 1973, *Croft NGF-34532* (E, K, L); Kokoda trail, eastern side lake Myola, 22 Jul 1974, *Croft LAE-61910* (A, K, QRS). **Madang**: Saidor, Moro, Naho-Rawa Division, Finisterre mountains, 19 Nov 1964, *Perumal 21473* (BM). **Morobe**: Mount Kaindi, Wau, 4 Oct 1977, *Conn* & *Kairo 491* (CANB, K); Lao, collected by Womersley, this plant grown at Dept. of Priimary Industries glasshouse, Indooroopilly, Brisbane, 18 Dec 1972, *Henderson 1357* (CANB, K); Hekwangi village, 7 km N of Menyamya, 2 May 1982, *Kairo 513* (A); Mount Kaindi, summit, subdistr. Wau, 30 May 1977, *Kairo* & *Symon 10639* (K); Bulldog rd, nr Edie Creek, Wau, 13 Aug 1963, *Millar* & *Holttum NGF-15825[b]* (A, US); E slope of the Spreader Divide, c. 6 mi NW of Aseki, 20 Apr 1966, *Schodde* & *Craven 5015* (K); Otetei Village, Langimar River, Menyamya sub-distr., Morobe Distr. N.G, 25 Nov 1970, *Streimann* & *Kairo NGF-44518* (A, K); Aseki rd from Bulolo, subdistr. Wau, 29 May 1977, *Symon 10628* (K); Bulldog rd, 2 mi from Edie Creek, Morobe Distr. TNG, 2 Sep 1965, *Womersley NGF-24678* (A, K). **Southern Highlands**: Hagen-Mendi rd, Mendi SubDistr., Southern Highlands Distr., 21 Sep 1968, *Vandenberg et al. NGF-40062* (K). **Western Highlands**: nr Ampyak Highlands, Ecological Site 1, 15 Dec 1964, *Flenley ANU-2192* (K); nr Tomba village, S slope of Mount Hagen Range, 27 Aug 1956, *Hoogland* & *Pullen 6014* (BM, US); Laiagam, 16 Aug 1960, *Hoogland* & *Schodde 7444* (BM). **Western Province**: Western, 19 Jun 1979, *Sohmer LAE-75542* (K); Kubor Range, Uinba, Nona-Minj Divide, 20 Aug 1963, *Vink 16311* (K).

PHILIPPINES. **Luzon**: Cordillera (CAR), Mount Pulag, 26 Jan 1968, *Jacobs 7173* (K); NCR, Manila, 15 Dec 1890, *Loher 4380* (K).

PITCAIRN ISLANDS. **Oeno Island**: Close to hut, 23 Jun 1934, *St. John* & *Fosberg 15195* (US). **Pitcairn Island**: Bounty Bay, baie de la Bounty, Cap Est, 22 Apr 1991, *Florence 10774* (US); Bounty Bay, 15 Jun 1934, *Fosberg* & *Clark 11337* (US); Ted side, 15 Jun 1934, *St. John 15044* (US).

SAMOA. **Apia**: Apia, 6 May 1907, *Vaupel Sol-3* (K). **Savai'i**: E of Olo, 8 Aug 1931, *Christerpherson* & *Hume 2311* (K). **Solomon Islands**. **Guadalcanal**: north central Guadalcanal, Tina River, 14 Sep 1967, *Maurisi BSIP8109* (K).

TAIWAN. Taitung, Lanyu, Swasy-rock, 19 Feb 1986, *Huang et al. 10632* (MO); Tong-shiau, Miaoli Hsien, 2 Aug 1964, *Kao 5702* (MO). **Pingtung County**: Wutain Hsiang, Tawu village, 13 Mar 1999, *Shu-hui Wu 1161* (MO). **Taoyuan County**: Kuanyin Hsiang, Hsinpo, 28 Dec 1999, *Ching-I Peng, 17879* (MO).

TONGA. **Tofua**: Sin. loc, Jan 1967, *Scarth-Johnson s.n.* (K). **Tongatapu**: Kologa, Jun 1926, *Setchell* & *Parks 15374* (US).

UNITED STATES OF AMERICA. **Hawaii**: Leeward Island, Nihoa, 17 Jun 1923, *Caum 62* (BISH, K, NY); Oahu, Kaela, Nov 1909, *Faurie 861* (BM, P); Kauai, Waimea, Feb 1909, *Faurie 864* (BM); Kauai, Ka'ula Island, 18 Aug 1932, *Gaum 15* (US); Kauai, along the Hanapepe River, 2 Jul 1895, *Heller 2389* (US); Kauai, Hanapepe and Wahiawa watershed, 5 Jul 1895, *Heller 2509* (B, BM, E); Kauai, Kaholuamanoa, above Waimea, 1 Oct 1895, *Heller 2867* (AK, BM, E, US); Kauai, Ka'ula Island, 21 Aug 1978, *Herbst 6210* (US); Sin. loc, *Hillebrand s.n.* (BM); sin. loc, *Hillebrand 117* (K); sin. loc, *Hillebrand 118* (K); Hawaii, Mauna Kea, 22 Aug 1916, *Hitchcock 14294* (US); Oahu, Wahoo, sin. loc, *Nuttall s.n.* (BM); Oahu, Mokuleia Forest Reserve, 8 Apr 1984, *Wagner et al. 5351* (US); Oahu, Puu Kana, 29 Feb 1948, *Wilbur 484* (DUKE, W); Sandwich Ins., *Without Collector, s.n.* (W); Maui, Mokeehia Island, W Maui, 4 Apr 2005, *Wood et al. 11179* (US); Pearl and Hermes Atoll, Southeast Island, 18 Aug 1964, *Young 112* (US).

VANUATU. **Efaté**: Eraté, Port Vila, 20 Nov 1983, *Cock 30* (K). **Espiritu Santo**: Shark Bay, Industrial Forestry Plantation, 9 Sep 1992, *Curry 651* (K).

Solanum palitans

AUSTRALIA. Sin. loc., 2010, *Sewell s.n.* (NE). **New South Wales**: Hawkesbury, North Richmond area, May 1955, *Boyle s.n.* (NSW); Cessnock, Branxton distr., Jan 1949, *Brown s.n.* (NSW); Wollondilly, 116 Bronzewing Street, Tahmoor, 23 Mar 2003, *Brown 302* (CANB); Wollondilly, 116 Bronzewing Street, Tahmoor, 23 Mar 2003, *Brown 38020* (NE); Maitland, West Maitland, Jan 1911, *Burgess s.n.* (NSW); Barallier via Moss Vale, May 1950, *Carlon s.n.* (NSW); Upper Lachlan Shire, Bannaby, nr Taralga, Oct 1959, *Chalker s.n.* (NSW); Cessnock, 13 Jun 1926, *Cheel s.n.* (AD, NSW); Wingecarribee, Burragorang Lookout, 22 km S. of Yerranderie, 23 Feb 1966, *Constable s.n.* (NE); Wollondilly, Burragorang Lookout, 14 mls S of Yerranderie, 23 Feb 1966, *Constable 6730* (AD); Burragorang Lookout, 14 mls S of Yerranderie, 17 mls WNW of Bowral, 23 Mar 1966, *Constable 6738* (CANB, K); Wingecarribee, Junction of Jock's Creek and Wollondilly River nr Goodman's Ford, 39 km WNW of Mittagong, 16 Mar 1975, *Coveny et al. 6089* (AD, K, NSW); North Arm of Wollombi Brook bridge, Millfield on Cessnock-Wollombi Road, 11 Jun 1975, *Coveny & Powell 6512* (K); Cessnock, North Arm of Wollombi Brook bridge, Millfield on the Cassnock-Wollombi Rd, 11 Jun 1975, *Coveny & Powell 6512* (AD, NSW); Bellbird Hill Reserve Rotary Lookout, Kurrajong Heights, 28 Mar 1984, *Coveny & Miller 11825* (K); Hawkesbury, Bellbird Hill Reserve Rotary Lookout, Kurrajong Heights, 28 Mar 1984, *Coveny & Miller 11825* (AD, NSW); Singleton, 15 Jun 1977, *Fenwick s.n.* (NSW); Newcastle distr., Sep 1949, *Glenfield Vet Research Station s.n.* (NSW); Maitland, Maitland distr., Feb 1948, *Glenfield Vet Research Station SN48/344* (NSW); Sin. loc., 10 Dec 2003, *Hart s.n.* (NSW); Tamworth Regional, Moorsville, The Forest, south of Moore Creek, 4 Sep 1996, *Hosking 1282* (CANB, MEL, NS, NSW); Wellington, NE of Wellington, Burrendong dam attachment, 10 May 2013, *Knop s.n.* (NSW); Upper Hunter Shire, Akuna, 30 Jan 2009, *Lewer 5* (NSW); Campbelltown, Showground, 26 Apr 1964, *McBarron 9021* (AD, NSW); Strathfield, Saleyards, Flemington, 10 Aug 1968, *McBarron 15534* (NSW); Strathfield, Saleyards, Flemington, 24 Aug 1968, *McBarron 15583* (NSW); Muswellbrook, 12 Feb 1951, *McMullen s.n.* (NSW); Auburn, Old Saleyards, Flemington, 23 Apr 1969, *Michael s.n.* (CANB); Upper Hunter Shire, Scone, 5 Feb 1951, *Monteith s.n.* (NSW); Muswellbrook, Oct 1948, *Olsen s.n.* (NSW); Gloucester, Bundook via Wingham, 14 Jun 1968, *Relf s.n.* (NSW); Auburn, Flemington Saleyards, Sydney, 25 Aug 1969, *Reyenga s.n.* (CANB, NSW); The Sheepwash, about 0.5 mile N of Joorilands station, Burragorang Valley, 26 Feb 1967, *Rodd 442* (NSW); Wingecarribee, ca. 2 mls NW of Bullio, 28 Jun 1965, *Salasoo 3061* (AD, NSW); Swan Creek, ca. 6 mls NE of Grafton on Pacific Highway, 15 Nov 1955, *Shepherd s.n.* (NSW); Hawkesbury, Peel's dairy on Terrace Road, North Richmond, 28 Nov 2000, *Sherring s.n.* (NSW); Muswellbrook, 6 Dec 1948, *Sturgess s.n.* (NSW); Muswell Brook, 6 Dec 1948, *Sturgess 6968* (K); Dungog, Bird Hill, Vacy, 29 May 1967, *Wilson s.n.* (AD, NSW); Wingecarribee, 4 km E of Wombeyan Caves on Mittagong road, 28 Apr 1982, *Wilson 4417* (AD, NSW); Armidale Dumaresq, Hillgrove mine, S of Hillgrove, Metz Gully off Bakers Creek, 15 Oct 2014, *Without Collector 16* (NE); Tamworth Regional, Moorsville, The Forest, S of Moore Creek, 4 Sep 1996, *Without Collector 1282* (NE); Wollondilly, Belimba Park, 225 Binnalong St, 17 Jan 2001, *Without Collector 6930* (NE).

Solanum pseudospinosum

CAMEROON. N slope of Cameroun Mountain, 7 Apr 1985, *Thomas 4642* (MO). **Nord-Ouest**: Piste Acha - Abaw au Lac Oku, 40 km NE Bamenda, 5 Dec 1974, *Letouzey 13444* (K). **Sud-Ouest**: Mt. Cameroon, above Buea, nr Hut 2, 1 Apr 1952, *Boughey GC6933* (K); Mt. Cameroon, Johann - Albrechtshöhe, 30 Jan 1962, *Breteler et al. MC39* (K); Mt. Cameroon, 9 Oct 1992, *Cheek* & *Sidwell 3664* (K, SCA, WAG, YA); Mt. Cameroon, 4 Nov 1993, *Cheek et al. 5352* (K); Mt. Cameroon, 17 Feb 1927, *Dalziel 8335* (E, K); Mt. Cameroon, nr Hut 2, Jan 1967, *Guile, 1560* (MO); Bamenda, Southern Cameroons, Bamenda Division, Bafut-Ngemba forest reserve, 23 Feb 1958, *Hepper 2146*

(K); Mt. Cameroon, beside Hut 2, 6 Apr 1937, *Hutchinson* & *Metcalfe 54* (K); Mt. Cameroon, To no 3 Hut, Jan 1931, *Maitland 1301* (K); Mt. Cameroon, Feb 1931, *Maitland 1333* (K); Mt. Cameroon, Jan 1862, *Mann 1321* (K); Mt. Cameroon, NW de Buea, 31 Mar 1981, *Meijer 15435* (MO); Mt. Cameroon, Kamerun-Berg, oberhalb Buea, 22 Dec 1928, *Mildbraed 10888 a* (K); Mt. Cameroon, Hut 2, 21 Dec 1958, *Morton 758* (K); Mt. Cameroon 2 mi W of Mann's Spring, 29 Dec 1958, *Morton K874* (K); Mt. Cameroon, around Mann's Spring, 3 Dec 1996, *Nning et al. 52* (K, MO, SCA, YA); Mt. Cameroon, Bokwango, 5 Oct 1992, *Thomas 9348* (K).

EQUATORIAL GUINEA. **Bioko**: cumbre del pico Basilé, 3 Jul 1986, *Fernández-Casas 10156B* (K, MO); Carretera del pico Basilé, 10 Jul 1986, *Fernández-Casas 10313* (BM, K). **Bioko Norte**: Pico Basilé, Distr. Barney, cruce de la Virgen de Bisila, 12 Dec 2007, *Cabezas et al. 907* (MA); Cumbre del pico Basilé, 8 Oct 1986, *do Carvalho 2544* (BM, H, K, MA, MO); Cumbre del pico Basilé, 3 Jul 1986, *Fernández-Casas 10157* (BM, K, MO); Pico Basilé, cumbre del pico, 5 Feb 1989, *Fernández-Casas 11184* (K, MA).

Solanum pygmaeum

FRANCE. **Provence-Alpes-Côte d'Azur**: Var, Hyères, *Fleming 306* (BM).

UNITED KINGDOM. **England**: Cornwall, Hayle Towans, West Cornwall, 21 Sep 1929, *Britton s.n.* (K); Cornwall, Phillack towans, Hayle, Cornwall, 12 Sep 1929, *Foggitt s.n.* (BM); Cornwall, Phillack Towans, 12 Sep 1929, *Foggitt s.n.* (BM); Cornwall, Hayle Towans, 21 Sep 1929, *Melville s.n.* (BM); Cornwall, Hayle Towans, 21 Sep 1929, *Melville s.n.* (BM); Cornwall, Hayle Towans, 21 Sep 1929, *Melville s.n.* (K); Cornwall, Hayle Towans, 21 Sep 1929, *Melville* & *Smith s.n.* (BM); Cornwall, Hayle Towans, 21 Sep 1929, *Melville* & *Smith s.n.* (BM); Cornwall, Hayle Towans, 21 Sep 1929, *Melville* & *Smith s.n.* (K); Cornwall, Hayle Towans, West Cornwall, 21 Sep 1929, *Melville* & *Smith 2922* (K); Greater London, Winchmore Hill, 9 Sep 1920, *Herb. Hall s.n.* (BM); Greater London, Edgware, 22 Aug 1950, *Hill s.n.* (BM); Greater London, Southall 14 Aug 1945, *Kent s.n.* (BM); Greater London, Southall, 7 Oct 1945, *Kent s.n.* (BM); Greater London, Hanwell, 3 Sep 1947, *Kent* & *Sandwith 3423* (K); Greater London, Hanwell, 18 Aug 1945, *Lousley s.n.* (K); Middlesex, 18 Aug 1945, *Lousley 4710* (BM); Greater London, Hahwell, 3 Oct 1961, *McCallum-Webster 7041* (K); Norfolk, Blickling, 3 Sep 1921, *Robinson s.n.* (BM).

Solanum retroflexum

AUSTRALIA. **New South Wales**: Bouddi, 1970, *Edmonds C74* (K). **Queensland**: Somerset, Kentville, 28 Oct 1965, *Henderson 123* (BRI, K). **South Australia**: Lower Eyre Peninsula, Sec[tion] 10, Hundred of Flinders, 13 Nov 1966, *Alcock 1268* (AD); Pilli Waterhole, sect. 10, Hundred of Flinders, 27 Apr 1968, *Alcock 2099* (AD, CANB, K); Barunga West, Section 200, Hundred of Wiltunga, 25 Jan 1968, *Copley 1838* (AD); Eyre Peninsula, 1970, *Edmonds C 73* (K); Hundred of Flinders, 16 Apr 1968, *Symon s.n.* (AD); Pillie Waterhole, 2 Apr 1968, *Symon s.n.* (AD); Pillie Waterhole, 2 Apr 1968, *Symon s.n.* (AD). **Victoria**: Mildura, Hattah/Kulkyne National Park, 11 Feb 1989, *Browne s.n.* (AD).

BOTSWANA. Livingstone's Cave, Melepelele, 35 mi NW of Gaberone, 21 Apr 1974, *Mott 236 C* (K).

LESOTHO. Leribe, Basutoland, *Dieterlen 157* (BM, K); Kolonyama plateau W border, 4 Oct 1969, *Williamson 48* (K). **Leribe**: LHDA Phase 1A, 13 Jan 1996, *Phillipson 4757* (MO).

MALAWI. **Southern**: Blantyre, Ndirande Mountain, 2 May 1970, *Brummitt 10323* (K).

MOZAMBIQUE. **Zambezia**: Mocuba, ao km 40, estrada de Milange, 18 Mar 1943, *Torre 4957* (MO).

NAMIBIA. Erongo Mountains, 28 Jun 1916, *Pearson 9834* (K). **Khomas**: Windhuk Bergland, regio Finkenstein, 13 Dec 1963, *Seydel 3783* (A, K, MO).

SOUTH AFRICA. Cape Prov., *Adamson 3225* (BM); Cape Town, *Barkly s.n.* (BM); *Barrett-Hamilton s.n.* (BM); Griqualand West between Schmidts drift, Vaal river and Griquatown, 1902, *Barrett-Hamilton s.n.* (BM); Sepani F.S, Apr 1931, *Brierley*, *127* (BM); Sepani F.S, Mar 1932,

Brierley 142 (BM); British Kaffraria, 1864, *Cooper 187* (BM, E, K); sin. loc, *Drège s.n.* (BM); Uitenhage, *Harvey s.n.* (BM); Johannesburg Distr., 2 May 1903, *Ommanney 140* (BM); Orange Kloof, 15 Nov 1955, *Salter 9711* (BM); Habitat rempublic, Transvaal, Distr. Lydenburgh, Mar 1884, *Wilms b1022* (BM); Sin. loc., 25 Jan 1896, *Wolley-Dod 856* (BM, K). **Eastern Cape**: Basiya, T[h]embuland, *Baur 102* (K); turnoff to Carlisle Bridge from Grt Riebeek East Rd, Grahamstown grid, 31 Mar 1974, *Bayliss BRI-B-761* (A, K); 6 mi E of Fort Beaufort, Cape, 6 Nov 1965, *Bayliss 2994* (MO); Humansdorp, 10 Feb 1966, *Bayliss 3171* (A); Annes Villa, Zuurberg, 2 Oct 1975, *Bayliss 7182* (A, MO); Eland's Hoek nr Aliwal North, Apr 1903, *Bolus 55* (MO); Graaff Reniet, *Bolus 50* (F, K); Hogsbank Forest Reserve, 29 Mar 1972, *Dahlstrand 2919* (MO); Fort Hare Farm, Honeydale section, W.S.W. Trollope's Burn-Browse plots, Ft. Beaufort 3226 DD Alice, 31 Mar 1977, *Gibbs Russell 3704* (MO); Stutterheim, Happy Valley, nr Cathcart, 30 Jan 1973, *van Graan 406* (MO); Butternooth, Transkei region, 2 Feb 1966, *Guillarmond 5160* (MO); Kestell, Goden Gate Hoogland Nas. Park, Jan 1974, *Liebenberg 8240* (MO); Mkambati Nature Reserve, Transkei Coast, c. 2 km from Mkambati waterfall, 11 Dec 1986, *Nicholas 2369* (K, MO); Amatole Mountains, Elandsberg, 24 Mar 1985, *Phillipson 1077* (MO); Amatole Mountains, Elandsberg, 20 Mar 1986, *Phillipson 1354* (K, MO); SA Nr camp site 1 km E of Steilkop, New Agatha Forest Reserve, 21 Apr 1971, *Scheepers 54* (EA); Zwartkops River, Uitenhage Distr., *Zeyher 3473* (K, W). **Free State**: Fauresmith, Apr 1937, *Henrici 3080* (K); Mequatling's Nek, Apr 1972, *Jacobs 8533* (K); Golden Gate National Park, Jan 1963, *Liebenberg 6877* (K); Golden Gate National Park, *Liebenberg 7410* (K); Harrismith, Kestall, Golden Gate Hoogland National Park, Jan 1905, *Sankey 200* (K); Zaaihoek, 15 mi N van Bethelem, 16 Jan 1969, *Werger 236* (K). **Gauteng**: Pretoria, Carlisle Bridge, Albany, 2 Apr 1978, *Bayliss 8679* (MO); Burttholm, Verreniging, 25 Apr 1918, *Burtt Davy 17658* (K); Pretoria, Hhohho Distr., Malandzela Area, on Maphalaleni rd, 10 km from Nyokana turn off, 28 Jan 1994, *Germishuizen 7215* (MO); Krugersdorp Distr., Swartkop, Banks of Bloubank spruit, 8 Apr 1990, *Glen 1943* (MO); Noordevoet van Wapadskop net oos van Lynnwood, Pretoria, Mar 1977, *Liebenberg 8842* (K); Magaliesberge, Pretoria Distr., 8 Jun 1955, *Schlieben 7009* (F, K, MO). **KwaZulu-Natal**: Itala Nature Reserve, c. 2 mi from Bivane-Pongola junction, Louwberg dist, 17 Jan 1976, *Brown* & *Shapiro 330* (K); Natal, 11 km NW of Utrecht on rd to Wakkerstroom, 22 Feb 1974, *Davidse 6812* (MO); 2829 (Harrismith) Drakensberg, Royal Natal National Park, 19 Jan 1987, *Goldblatt* & *Manning 8416* (MO); 5-7 mi NNW of Castle View Farm, headwaters of Mlahlangubo River, Underberg Dist, 19 Jan 1982, *Hilliard* & *Burtt 15180* (K); Drakensberg Range, Tugela Valley & Mont-aux-Source, 3 Apr 1934, *Humbert 14849* (MA); Natal, in Otibi Gorge, Apr 1937, *McClean 378* (MO); Oribi Gorge, Paddock, Apr 1937, *McClean 28270* (K); sin. loc, May 1880, *Medley Wood 811* (K); Umgeni [Umgeni River], 19 Feb 1895, *Penther 1846* (W); Distr. Alexandra, Station Dumisa, Campbellson, 17 Dec 1912, *Rudatis 1805* (W); Fish River, Gros-Namaland, Fishfluss, 12 Dec 1884, *Schinz 806* (K); Mt/At Mokolane in the Tooge River bed, 1 Nov 1975, *Smith 1494* (MO); Magaliesberg, top gondola about Hartebeestpoortdam, 16 Nov 1975, *van Steenis 24079* (K); Ngome, 15 Dec 1969, *Strey 9409* (E, EA, K). **Limpopo**: Duiwelskop, grid no. 3219CA, Clamvillham Distr., 15 Oct 1983, *Bean 1366* (MO); Mosdene, Naboomspruit, Waterberg Dist, 25 Feb 1925, *Galpin M-747* (K); Berlin Mission Farm, 13 mi NW of Nylstroom, Waterberg Dist, 13 Mar 1939, *Molepo 18* (K); New Agatha Forest Reserve, nr campsite 1 km E of Steilkop, 21 Apr 1971, *Múller* & *Schespere 54* (K). **Mpumalanga**: Nelspruit Distr., Berlin State Forest, c. 6 km E of Kaapsehoop, 21 Mar 1991, *Balkwill et al. 6291* (MO); Ermelo, Nooitgedacht Research Station, 19 Jan 1976, *Balsinhas 2901* (K, MO); Middelburgh, Bangor Farm, Jan 1918, *Bolus s.n.* (MO); 17 mi SE of Ermelo, 5 Mar 1962, *Codd 10152* (K); 27 km SE of Bethal, 20 Feb 1974, *Davidse 6704* (MO); Ossewakop, Wakkerstrom area, 24 Feb 2003, *Diamini et al. 612* (K); Namaqualand, Klipfontein, 23 Dec 1949, *MacDonald 117* (BM); Lswepe Tol, 20 Mar 1949, *Sidey 1624* (MO); Knock Dhu farm nr Lothair 13 km SE of Lake Chrissie on Lothair rd, Grassveld, 9 Jan 1984, *Welman 369* (K, MO); Vogelkat Kloof, Caledon Division, 2 Apr 1979, *Williams 2744* (K, MO); Klipfontein, Hay, Eriqualand West, Mar 1934, *Wilman 3140* (K); Bei der Stadt Lydenburg, Mar 1884, *Wilms 1022 a* (BM, E). **North West**: Rustenburg, in vlei at Modderfontein, 9 Jan 1904, *Nation 49* (K); head of Helskloof, Hottentosparadyskloof, 28 Aug 1977, *Thompson & Le Roux 134* (K); Magaliesberg, Jackson's kiln, 28 Feb 1957, *Vueeden 97* (K). **Northern Cape**: Westeljike bellings van Rebunie, Calvinia, 27 Dec 1977, *Hanekom 2499* (K); Beeshoek, 5 mi WNW of Postmasburg, 26 Aug 1961, *Leistner* & *Joynt 2706* (K); Namaqualand, Ai-Ansi, 1 Jan 1950, *MacDonald 137* (BM); Kimberley, Magersfontein kop, Apr 1934, *Wilman 3192* (K). **Western Cape**:

Pro Spei (Cape of Good Hope), 1771, *Banks* & *Solander s.n.* (BM); Nr drift of Cayman's River, George, *Barkly s.n.* (BM); Table Mountain, *Burchell 856* (K); 3419 Caledon AD Fernkloof Nature Reserve, 10 Mar 1985, *Drewe 174* (K, MO); Stong Vale C.S., *Gill s.n.* (K); St.Muller's Point, Londagis River, C.S., *Gill s.n.* (K); 3419 Simonstown AB, SE slopes of Little Lions Head, above Hout Bay, 6 Jun 1980, *Goldblatt 5553* (MO); AD Vogelgat nr Hermanus, 15 Oct 1986, *Goldblatt 7915* (MO); C.S, *Harvey s.n.* (K); Cape of Good Hope, *Harvey s.n.* (BM, E); Ceres Distr., Tankwa Guest Farm31 Jul 2006, *Sachse 5* (MO); Jonkershoek, 15 Mar 1962, *Taylor 3205* (K); 3419 Caledon AD, Kettle Point, Hermanus, 17 Apr 1985, *Williams 1079* (MO); Devil's Peak, bei Capstadt, 13 Sep 1883, *Wilms 3458* (K); banks above path to Smitminkel, 24 Sep 1897, *Wolley-Dod 3318* (K); Simon's Bay, 1853, *Wright s.n.* (K); Simon's Bay, Cape of Good Hope, 1853, *Wright s.n.* (GH).

SWAZILAND. Ukulula, Mbabane Dist, 13 Mar 1955, *Compton 25011* (K).

ZAMBIA. **Central**: Sin. loc., 2 Feb 1964, *Fanshawe, JMM-8310* (K). **Copperbelt**: Kitwe, 15 Jan 1960, *Fanshawe, F-5353* (K). **Northern**: Kaloswe, Koloswe, 16 Jul 1930, *Hutchinson* & *Gillett 3767* (K).

ZIMBABWE. Owelo Teachers College, 11 Nov 1966, *Biegel 1413* (K); Vumba, Clouds Downs, 29 Feb 1960, *Head, 170* (BM); sin. loc, *Hislop Z-155* (K); Bulawayo, Jan 1898, *Rand 171* (BM). **Bulawayo**: Bulawayo, garden, 9 Oct 1946, *Best 499* (K). **Harare**: Harare, Salisbury Expt. Station, 26 May 1943, *Arnold 10206* (K); Harare, Salisbury Botanic Garden, 30 Jan 1969, *Muller 743* (K). **Manicaland**: Makone, Chiduku, Apr 1955, *Davis 1190* (K); Southern Rhodesia (E). Mt. Nuza, 23 Jun 1934, *Gilliland 476B* (BM, K); Umtali, Nuza plateau (Mutasa Distr.), Oct 1934, *Gilliland 974* (BM, K); Nyanga, Inyanga Distr., 11 Jan 1931, *Norlindh* & *Weimarck 4199* (BM, MO); Chimanimani, Melsetter, 29 Apr 1907, *Swynnerton 1794* (BM); Umtali, Odzani River valley, 1914, *Teague 74* (K); Penhalonga Distr., 9 Dec 1945, *Wild 539* (K). **Mashonaland Central**: Mazowe, southern Rhodesia, Mazoe, Umvukwes, Ruorka Ranche, 17 Dec 1952, *Wild 3961* (MO). **Mashonaland East**: Pasture Research Station, Marandellas, 28 Mar 1934, *Brain 10574* (MO). **Masvingo**: Victoria, Rhodesia, 1909, *Monro 1024* (BM). **Matabeleland North**: Makoholi Experiment Station, Victoria Distr., 13 Mar 1978, *Senderayi 226* (K). **Matabeleland South**: Matobo, Farm Beana, Kobila, Apr 1957, *Miller 4341* (K); Gwanda, Tuli Experimental Station Reservior in pasture block, 16 Jan 1965, *Norris-Rogers 599* (K); River ranch, off Maera rd, Beitbridge, Umzingwane River, 9 Feb 2000, *Timberlake* & *Cunliffe 4521* (K). **Midlands**: Sable Park, QueQue Distr., 13 Mar 1978, *Chipunga 168* (MO).

Solanum sarrachoides

AUSTRIA. **Steiermark**: Fussee, Mistablagerumgsplatz, 26 Aug 1950, *Rechinger s.n.* (MA).

FRANCE. **Grand Est**: Bas-Rhin, Strasbourg, Petrolhafen in Strassburg, 18 Oct 1953, *Aellen s.n.* (W). **Nouvelle Aquitaine**: Gironde, Gironde, Bassem, 19 Sep 1924, *D'Alleizette s.n.* (W); Gironde, Bordeaux, 6 Sep 1926, *Herb. Guiol s.n.* (BM).

GERMANY. Neuft, [Speten], 20 Sep 1917, *Boutz s.n.* (B). **Hamburg**: Hamburg, bei Wandobek, Sep 1900, *Schmidt s.n.* (W). **Hessen**: Herdingen, Rheinwerfl, 26 Sep 1920, *Boutz s.n.* (B).

SOUTH AFRICA. **Eastern Cape**: Butterworth, Transval Region, 2 Feb 1966, *Guillarmond 5158* (MO); Amatole Mountains, Elandsberg, Coolin farm, 20 Mar 1986, *Phillipson 1353* (K, MO); Rooiberge, nr Graaf Reinet Rooiskuur dam on Roodeberg Farm, 17 Dec 2000, *Phillipson* & *Hobson 5256* (K, MO).

SPAIN. **Andalucia**: Granado, Cacín, 9 Nov 1970, *Pérez Raya s.n.* (MA). **Castilla-La Mancha**: Toledo, Montalbán, embalse de Castrejón, 18 Apr 1983, *López s.n.* (MA).

SWEDEN. **Götaland**: Västra Götaland, Västergötland, Mölndal, Svenska Oljeslageriet, 30 Aug 1936, *Blom s.n.* (BM); Västra Götaland, Angered, Agnesbergskvarn, 31 Aug 1938, *Blom s.n.* (W); Västra Götaland, Molndal, Sveska Oljeslageriet, 30 Aug 1936, *Blom 1376* (CORD, K, W); Skåne, Malmö, Sep 1906, *Hylmö s.n.* (BM); Västergötland, Asfroued Au, Sjoliageu, 10 Sep 1966, *Westfelt s.n.* (BM).

UNITED KINGDOM. **England**: Hertfordshire, Wheathampstead, 14 Oct 1962, *Dony 4165* (BM); Hertfordshire, Nov 1955, *Doug s.n.* (K); Bedfordshire, Cabbage field at Flitton, 8 Oct 1974, *Hanson 107a* (BM); Essex, Dagenham, 28 Jun 1929, *Hubbard et al. s.n.* (K); Essex, Dagenham dump, 28 Aug 1954, *Lousley s.n.* (K); Cornwall, below Middle Town, St Martin's, 5 Sep 1952, *Lousley s.n.* (BM);

Hertfordshire, Wheathampstead, 6 Oct 1963, *Lousley s.n.* (BM); Hertfordshire, Wheathampstead, 6 Oct 1963, *Lousley s.n.* (K); Essex, Dagenham, Wasteground, 10 Sep 1926, *Melville s.n.* (BM); Essex, Waste ground, nr Dagenham, 2 Oct 1927, *Melville s.n.* (BM); Essex, Wasteground Dagenham, 2 Oct 1927, *Melville s.n.* (K); Gloucestershire, Avonmouth Docks, 29 Aug 1922, *Polgár s.n.* (BM); Essex, Dagenham, 6 Oct 1945, *Sandwith* & *Milne-Redhead s.n.* (BM, K); Essex, Barking Tip, 12 Sep 1953, *Welch 5298* (BM).

Solanum scabrum

ANGOLA. Cazengo, 6 Sep 1911, *Gossweiler 530 b* (BM); Hochland von Benguella zwischen Ganda und Caconda, Facenda Xangorolo, 1933, *Hundt 798* (BM); Ambriz, *Monteiro s.n.* (W); Dec 1853, *Welwitsch 6053* (BM); Varzea gr. do Coango, Golungo alto, Jun 1856, *Welwitsch 6099* (BM, K); Prope Camilungo, May 1855, *Welwitsch 6102* (BM, MO). **Bengo**: Ambriz, Abriz, Dec 1872, *Monteiro s.n.* (K). **Benguela**: Hochland zwischen Ganda und Caconda, Dec 1933, *Hundt 797* (BM). **Cuanza Norte**: Varzea do Isidoro ad rivum Cuango, Dto. Golungo Alto, Jul 1855, *Welwitsch 6100* (BM); Golungo Alto, Aug 1855, *Welwitsch 6101* (BM). **Malanje**: Distr. Pungo Andongo, prope Lusillo, Jan 1857, *Welwitsch 6108* (BM). **Namibe**: Mofsamedes, Herb. Moira, Habit Cavalheiros, Jul 1859, *Welwitsch 6033* (BM).

BENIN. **Atlantique**: Wida, Dahomey, 26 Aug 1903, *Estève 111* (BM).

BOTSWANA. Botletle River at Ioromoja, 22 Apr 1975, *Ngoni 409* (K, MO). **Ghanzi**: Ghanzi camp, 4 May 1969, *Brown 6019* (K); Ghanzi camp, 4 May 1969, *Cole 6019* (K). **North West**: Mutsoi NE of Nokaneng, 21 Mar 1967, *Lambrecht 90* (K); nr Maun, southern outlet of Maphaneng pan, 14 Sep 1974, *Smith 1080* (K).

BURKINA FASO. **Seno**: Dori Dam, 16 Oct 2007, *Sanou & Leonard BUR-596* (K).

CAMEROON. Nr. Santa, c. 15 km S of Bamenda, 3 Jan 1977, *Lowe 2951* (K); Buea, Cameroon Mt, 27 Mar 1983, *Morton* & *Venn 48* (MO); garden of De Of Agriculture, ENSA, 10 Jul 1976, *Westphal-Stevels 1989* (K). **Adamaoua**: Mayo-Banyo, Mambilla Plateau Cameroon, around Somie village, 21 Jun 2009, *Komaromi 48* (K); Mayo-Banyo, Mambilla Plateau Cameroon, around Somie village, 7 Jun 2009, *Komaromi* & *Ganfi 61* (K). **Centre**: Mimboman area, Yaounde, 16 Sep 1986, *Manning 235* (MO). **Extreme-Nord**: Monts Mandara, Hossere Oupay, 15 km NNO de Mokolo, 15 Sep 1964, *Letouzey 6880* (K, P). **Littoral**: Donala, 16 Aug 1986, *Johns 86-481* (K). **Nord-Ouest**: Mezam, Above Bamenda, 20 Jan 1928, *Migeod 358* (BM, K). **Sud-Ouest**: Buea, 15 Dec 1951, *Akpabla s.n.* (K); Mt. Cameroon, Buea, 8 Nov 1927, *Migeod 88* (BM, BM, K); Mt. Cameroon, Buea, 5 Apr 1981, *Morton* & *Venn K1398* (MO); Mt. Cameroon, above Batoke, 9 Jan 1984, *Thomas 2997* (MO); Mt. Cameroon, Farmland, Buea, 13 Nov 1960, *Ngomba 3* (K); Mt Kupe, Kupe Village, Ridge above Daniel Ajang's Saprophyte site, 24 May 1996, *Ryan 289* (K, MO, P).

CENTRAL AFRICAN REPUBLIC. Mboukou Griko, Territoire du Haut-Obangi, 24 Sep 1902, *Chevalier 5518* (K).

COMOROS. Grande Terre, Coconi, Jardin du SEF, 10 Jan 2001, *Barthelat* & *Sifary 210* (G, K, MO); Denis, 24 Mar 1974, *Coode* & *Bosser 4582* (K, P). **Anjouan**: Anjouan (Comoro-Insel Johanna), Jun 1875, *Hildebrandt 1626[b]* (BM, W). **Moheli Island**: Moheli, NE center of island, 14 Aug 1987, *D'Arcy 17617* (MO, P); Sin. loc., 1 Nov 1990, *D'Arcy 17765* (MO). **Njazídja**: Grande Comore, 9 Aug 1981, *Doutrelepont 1203* (MO, P).

CÔTE D'IVOIRE. Toumodi, 2 Jun 1986, *Béguin* & *Gautier 108* (MO); nr Buyo, 26 Apr 1963, *Oldeman 22* (K). **Abidjan**: Adjame, 31 May 1973, *De Koning 1747* (MO). **Dimbokro**: c. 5 km W of Dimbokro, 13 Oct 1975, *van der Burg 1177* (MO). **M'Bahiakro**: Koffi-Akakro (s. Prikro), 25 Jul 1973, *Smittenberg-Visser 61* (MO). **Montagnes**: Man, 6 km N nr Yebegouin, 27 Jan 1984, *Hepper* & *Maley 7849* (K).

DEMOCRATIC REPUBLIC OF THE CONGO. Nouvelle Anvers, 1908, *Flamigni 352* (K); vallée de la Karavia, Oct 1933, *Quarré 3509* (GH). **Katanga**: Lukonzolwa, Moero, 1933, *Quarré 3297* (K); Kongolo, 5 Feb 1920, *Schantz 646* (K). **Kinshasa**: Maluku, Route Menkao-Kingankati, 5 Nov 1971, *Breyne 2227* (MO). **Nord-Kivu**: Lubarika, 1953, *Gilon 315[a]* (MO). **Orientale**: Ituri, w.v. Albert-See, Oct 1934, *Gusinde s.n.* (W); Yangambi, 26 Jul 1938, *Louis 10507* (K, P). **Sud Kivu**: Kizozi, Jul 1933, *Lejeune 55* (K).

EGYPT. **Giza**: Faculty of Agriculture, Giza, 13 Jun 1971, *Sisi s.n.* (MO).

EQUATORIAL GUINEA. Pico de Fogo, SW side, Annobon island, 25 Jul 1959, *Wrigley* & *Melville 188* (MO). **Annobon**: Pico de Fogo, SW side, 25 Jul 1959, *Melville 188* (BM, K, MA, P). **Bioko**: entre Moca y Riaba por el camino viejo, 20 Feb 1989, *Fernández-Casas 11820* (K, MA, MO, P); Musola, Puente, Poo, 13 Jan 1947, *Guinea 1348* (K); Finca de D. Mandino, Puente, Fernando Poo, 20 Jan 1947, *Guinea 1637* (MA, MO); Fernando Po, 14 Sep 1959, *Wrigley* & *Melville 493* (K). **Bioko Norte**: Montes de Oca, Malabo-Baney km 15, 11 Sep 1988, *do Carvalho 3708* (K, MA); Pico Basilé, Carretera del pico Basile, 28 Mar 1990, *do Carvalho 4305* (K, MA, MO, P); Basilé, en el poblado, 9 Apr 1989, *Gómez Marín 157* (MA). **Bioko Sur**: Moka, Fernando Po, 9 Dec 1951, *Boughey 109* (K); camino de Moca Malabo hacia el lago Biaó, 18 Dec 2007, *Cabezas et al. 1095* (MA); Moca, camino de Ureca, 18 Feb 1989, *Fernández-Casas 11725* (K, MA, MO); Gran Caldera de Luba, Ureca-Hormiga, 18 Mar 2007, *Galán* & *Barberá 4753* (MA); Moca, 13 Nov 1942, *Guinea 1111* (MA); Moca, pt. 128, 15 Mar 2008, *Luke 12339* (K); Moca, Lago Biao, *Maté* & *Colell 14* (MA); Moka, 1 Sep 1959, *Wrigley* & *Melville 426* (K).

ERITREA. **Maekel**: Asmara, 1 May 1892, *Terracciano* & *Pappi 187 [2206]* (FT). **Semienawi Keyih Bahri**: Uagarti - Saharti, 17 Apr 1893, *Pappi 4318* (FT); Asmara, Beless, Hamasen, 4 May 1892, *Terracciano* & *Pappi 2536* (FT).

GABON. Cristal Mountains, 7 km along the rd from Tchimbele to Kinguele, 17 Jan 1983, *de Wilde et al. 24* (MO). **Estuaire**: Jardin Cenarest, Libreville, Poubelle, 16 Jan 1986, *Louis 1990* (MO).

GHANA. *Irvine 93* (K); 28 May 1960, *Morton s.n.* (MO); Danango, 8 Jun 1960, *Morton s.n.* (MO). **Ashanti**: nr Mampong, Ashanti, 7 Aug 1963, *Darko 5115* (K). **Central**: Aswansi, W.P, 12 Oct 1954, *Darko 1032* (K). **Eastern**: Akosombo, 25 May 1970, *Enti 1723* (MO); Akwapim, Mampong Scarp, 14 Jun 1953, *Morton s.n.* (K); Mompang Scarp, Akwapim, 14 Jun 1953, *Morton s.n.* (MO). **Greater Accra**: Accra, May 1961, *Irvine 5095* (K); Accra, May 1961, *Irvine 5096* (K); Acora Distr., Aug 1961, *Irvine 5191* (K); Shai Hills, 29 Nov 1956, *Morton A-2383* (K); Lagon Hill, 29 May 1959, *Morton A-3675* (K); Achimota, 9 Feb 1954, *Morton GC-24359* (K). **Volta**: Adzido, Keta, 16 Sep 1960, *Akpabla 2117* (K); Amedzofe, 14 Nov 1958, *Morton s.n.* (K); Volta Gap, Ajema to Jaketi rd, 11 Oct 1953, *Morton s.n.* (K); Akparafe Waterfall, N of Amedzofe T.V.T., 6 Nov 1960, *Morton A-4028* (K); Amedzofe, 8 Jan 1958, *de Wit* & *Morton A-2921* (K).

GUINEA. **Nzérékoré**: Lola, Mt. Nimba, 1 Nov 2012, *Diabate* & *Mas 1419* (MO).

INDONESIA. **Java**: Central Java, Mt. Slamet, 15 Mar 2004, *Hoover et al. 89* (A). **Seram**: Manusela National Park, 12 Sep 1987, *Argent C87-179* (A, E). **Sumatra**: Deleng Singkoet, Karo Highlands, 8 Jun 1928, *Hamel* & *Si Toroes 535* (GH); South Sumatera, Gunung Dempo, 20 Jan 1998, *Hoover* & *de Cognets 30064* (GH); headwaters of Aek Liang, Asahan, 15 Oct 1936, *Si Boeea 10775* (A); E of Berastagi, Karo Highlands, 4 Jun 1928, *Si Toroes 407* (A, GH).

LESOTHO. Sehlabathebe, 4 Jan 1973, *Bayliss 5476* (MO).

LIBERIA. sin. loc, 28 Jul 1967, *Geerling* & *Bokdam 417* (MO); sin. loc, 19 Jul 1935, *Harley 621* (K). **Central**: 3 mi NE of Suacoco, Gbarnga, 7 Feb 1951, *Daniel 117* (B, BM, MO). **Grand Gedeh**: Tchien, Mim Timber Co (Fijnhout), 16 May 1970, *de Koning 519* (MO). **Lofa**: Nr Bolahun, NW Liberian hinterland, 1933, *Earthy 8* (BM); 7 mi along rd to Wologesi, 15 Jul 1970, *Jansen 2015* (K, MO, P). **Nimba**: Yekepa, Granfield village, 22 Sep 1964, *Adam 23788* (MO); Nimba Expedition, 22 Sep 1964, *Adames 577* (K); Ganta, 24 Oct 1936, *Harley 1029* (K); Nimba Mountains, 27 Jul 1962, *Leeuwenberg* & *Voorhoeve 4668* (B, K, MO, P).

MADAGASCAR. sin. loc, 1897, *Baron s.n.* (P); sin. loc, *Baron 860* (K, P); sin. loc, Jun 1889, *Baron 1744* (P); Central Madagascar, Oct 1882, *Baron 1744* (K); Béravy, Béravi intérieur, Jul 1879, *Hildebrandt 3110* (BM, P, W); Central Plateau, 1914, *Hodgkin* & *Stansfield 23* (K); Central Plateau, 1914, *Hodgkin* & *Stansfield 82* (K); sin. loc, 1887, *Le Myre de Vilers s.n.* (P); sin. loc, 1887, *Le Myre de Vilers s.n.* (P). **Antananarivo**: Antananarivo-Sud, Ambohijoky, Sep 1905, *Académie Malgache s.n.* (P); Grande Terre - Ouangani, Apendzo-Jivany. Barakani, 7 May 2002, *Barthelat et al. 885* (K, MO, P); Tsimbazaza, 16 Jan 1917, *Decary s.n.* (P); Manjakandriana, Prov. Tananarive, Distr. Manjakandriana, 11 Nov 1912, *Humbert* & *Viguier 1244* (P); Prov. Vakinankaratra, Distr. Betafo, pente Nord du pic de Vohimalaza, 18 Nov 1912, *Humbert* & *Viguier 1375* (P); Antananarivo, 13 Feb 1979, *Ponsonby 26* (K); Antananarivo-Nord, Ambohipotsy, 10 Jan 1917, *Decary s.n.* (P); Antananarivo-Nord, Ilafy, 4 Feb 1917, *Decary s.n.* (P); Antananarivo-Nord, Tananarive et les environs, Feb 1897, *Prudhomme 28* (P). **Antsiranana**: Mt. Ambohitra, c. 20 km S of Antsiranana, 14

Sep 1987, *D'Arcy 17698* (MO); Mt. d'Ambre, partie centrale, Prov. Diego-Suarez, 11 Nov 2007, *Gautier 5198* (K, MO); Nossi-Bé, Mar 1880, *Hildebrandt 3401a* (BM, P, W); Ambanja, Bassi supérieur du Sambirano, 1937, *Humbert 18679* (P); Ambilobe, Ambilobe, *Waterlot 407* (P). **Fianarantsoa**: Mananjary, 19 May 1889, *Catat 1208* (P); Ankafana, 1880, *Cowan s.n.* (BM); 10 km W of Ivato on Route 35, 25 Jan 1975, *Croat 29606* (MO); 50-60 km E of Findandrahana, 25 Jan 1975, *Croat 29698* (MO); Befotaka, massif de l'Ivakoany, 1933, *Humbert 12191* (P). **Toamasina**: au km 26 de la route de Tamatave, 18 Oct 1951, *Benoist 147* (P). **Toliara**: Betioky-Sud, Hanga en Hova, Sakondry, Amelo en hova, 26 Jul 1898, *Grandidier s.n.* (P); Amboasary-Sud, haute vallée du Mandrare (Sud-Est), Nov 1928, *Humbert 6671* (P); Beroroha, Vallée du Mangoky et de l'Isahaina aux environs de Beroroha, Oct 1933, *Humbert 11309* (K, MO, P); Andohahela RNI, Mt. Trafonaomby, Taolagnaro, 7 Apr 1994, *Randriamampionona 692* (MO, P); Bekily, environs d'Ampandrandava (entre Bekily et Tsivory), Madagascar Sud, Oct 1942, *Seyrig 217* (P).

MALAWI. **Central**: Salima Distr., Liganga village A. Mpemba, 14 Jun 1985, *Kwatha et al. 210* (MO); Salima Distr., Luwadzi stream, 14 Jun 1985, *Salubeni et al. 4242* (MO). **Northern**: Nkhata Bay, Musalowa village, Chizumulu Island, 25 Mar 1989, *Balaka* & *Patel 2025* (K, MO); Mzimba Distr., Mzuzu, Marymount, 9 Mar 1974, *Pawek 8205* (MO). **Southern**: Chikwana, River Shine, Aug 1863, *Kirk s.n.* (K); Malawe Hill, W of Port Herald, 23 Mar 1960, *Phipps 2653* (K, MO); Zomba, University of Bristol Lake Chilwa Expedition, Jun 1989, *Without Collector 3* (K).

MAURITANIA. **Trarza**: Rosso Administrative area, Village Rosso, 9 Oct 1962, *Adam 18728* (MO).

MOZAMBIQUE. **Baroma**: N'kanya, N of Zambesi River, 25 Jul 1950, *Chase 2857* (BM). **Cabo Delgado**: Pemba, Jun 1909, *Rogers 8256* (K). **Manica**: Lower slopes of Chimanimani Mountains, Apr 1967, *Westwater 192954* (K); **NAMIBIA**. Onjossariviers, im Ufergestrupp, 21 Jun 1957, *Seydel 1162* (A, K); Kaokoveld, Kaoko Otavi Fountain, 20 Apr 1957, *de Winter* & *Leistner 5550* (K).

NIGERIA. sin. loc, *Yates 56* (K). **Delta**: [Ogwashi-Ukwu], 25 Nov 1912, *Thomas 2042* (K). **Edo**: Nikrowa Forest Reserve, Mid-west State, Iyekovia Distr., 8 Oct 1973, *Daramola FHI-72483* (K, MO). **Enugu**: 10 mi E of Erugu, Mar 1948, *Irvine 3607* (K). **Gongola**: Gemu Distr., Mambilla plateau, 18 Aug 1977, *Fagbemi 438* (MO); Gemu Distr., Mambilla plateau, 24 Aug 1977, *Odewo 727* (MO). **Jos**: Naraguta, *Lely 31* (K). **Kaduna**: Zaria, 1975, *Magaji MG-725* (K); Zaria-Samaru motor rd directly opposite the Chief Engineer's office, Zaria Prov., Zaria Distr., 24 May 1966, *Olorunfemi FHI-57034* (K). **Kano**: Kano, Wudil Distr, 50 km SE of the city of Kano, 16 Mar 1988, *Etkin 63[b]* (MO). **Kogi**: Odu, SW Nigeria, *Van Eyenhuisen 7* (K); Ogumo, 17 Jul 1971, *Van Eyenhuisen 25* (K). **Lagos**: Lagos, *Dalziel 1188a* (K); Lagos, *Dalziel 1188 b* (K). **Niger**: Nupe, *Barter 1054* (K, P). **Ondo**: between Ikare and Oke-Agbe, Ikare Distr., 2 May 1979, *Daramola* & *Osanyinlusi 154* (K); c. 1 mi to Alaro, Akure Distr., 6 Feb 1969, *Gbile et al. FHI-20567* (K). **Oyo**: Shasha Forest Reserve, Ife Distr., 10 Nov 1961, *Emwiogbon FHI-43533* (K); 8 km W of Olokemeiji at edge of forest reserve, 1 Aug 1962, *Gillett 15249* (K); Ibadan, Forestry Research Institute of Nigeria, 3 Mar 1980, *Lester 45* (K); Idaban, 15 km N of Ibadan, International Institute for Tropical Agriculture, 25 Feb 1980, *Lester 43* (K); Ibadan, 7 km W of Polytechnic, 11 Jun 1977, *Pilz 2108* (K, MO); Ibadan, Moor Plantation, 7 Nov 1961, *Swarbrick 2572* (K). **Taraba**: Ndarup, Mambilla Plateau, NE State, 26 Jun 1972, *Chapman 2914* (K); State of Gongola, Distr. Mambilla Plateau, Nguroje township, 23 May 1982, *Odewo 130* (MO); State of Gongola, Mambilla Plateau Distr., Nguroje, along Area Court rd, 25 May 1982, *Odewo 151* (MO); State Congola, Mambilla Plateau, Nguroje-Jungogia rd, 25 May 1982, *Odewo 157* (MO); State of Gongola, Distr. Mambilla, 25 May 1982, *Odewo 159* (MO).

SÃO TOME E PRINCIPE. Bom Sucesso, 27 Jan 1949, *Espírito Santo 218* (BM); Vanhulst (Macambrará), 28 Oct 1932, *Exell 89* (BM); Vanhulst (Macambrará), 19 Nov 1932, *Exell 396* (BM); Lagoa Amelia, 15 Aug 1956, *Monod 11733* (BM); 17 Aug 1956, *Monod 11884* (BM); Nova Moka-Bom Sucesso, 17 Jan 1949, *Santo 218* (K); 1912, *Watt 7053* (BM). **São Tomé**: between Zampalma and Trás-os-Montes, 26 Oct 1993, *Figueiredo* & *Arriegas 95* (K); Sao Nicolau, 9 Jan 1980, *de Wilde et al. 12* (MO).

SENEGAL. In paludilb[us] N'Boro nec non ins[ula] Bonavista, 1838, *Brunner 108* (BM, G, W); Sin. loc., 1828, *Perrottet 555* (BM, W); Sin. loc., *Perrottet 556* (BM).

SEYCHELLES. Morne Blanc, 2 Oct 1970, *Schlieben 11670* (B, K).

SIERRA LEONE. Sin. loc, 1821, *Don s.n.* (BM); Kambitz, 23 Sep 1930, *Glanville 412* (K); 29 Aug 1979, *Jacques-Georges 26547* (MO); Maloloka, 29 Jul 1914, *Thomas 1239* (BM); Maghile, 8

Dec 1915, *Thomas 6408* (W); Sin. loc, 1915, *Thomas 9638* (BM). **Eastern**: Kailahun, Musaia, 19 Dec 1946, *Deighton 4571* (K). **Northern**: Port Loko, Rokupr, Magbema, 18 Apr 1959, *Jordan*, *1059* (K); Yonibana, 12 Nov 1914, *Thomas 4909* (W); Tonkolili, Bumbuna, 20 Oct 1914, *Thomas 3703* (K). **Western**: Freetown, 28 Apr 1965, *Morton s.n.* (K).

SOUTH AFRICA. Zuurbraak, 20 Jan 1893, *Schlechter 2165* (BM, K, W). **Eastern Cape**: Grahamstown, 7 Sep 1970, *Bayliss 4594* (A). **KwaZulu-Natal**: 2632 CD Bella Vista grid, between Ndumu Store and the Game Reserve, 30 Oct 1969, *Moll 4138* (A, K). **Limpopo**: Mopani, Shiluvane, Aug 1899, *Junod 575* (K). **North West**: Okawango Delta, Okavango Delta, Delta camp, 16 Jun 1994, *Cole 923* (K). **Northern Cape**: Augrabies Falls National Park, Orange river bank, 14 Mar 1978, *Balsinhas* & *Harding 3290* (K, MO); Prieska, Mar 1934, *Bryant*, *1002* (K); Calvinia, 1936, *Schmidt 592* (K). **Western Cape**: Rondebosch, 8 Jan 1942, *Adamson 3317* (BM); Skeleton Ravine, 3 Oct 1897, *Wolley-Dod 3180* (K); Dowweklip, Voelklip, Hermanus, 6 Jun 1980, *Williams 287* (K, MO).

SOUTH SUDAN. **Bahr El Ghazal**: Anglo-Egyptian Sudan, Bahr El Ghazal Prov., Ibba, 10 Mar 1934, *Dandy 624* (BM, EA). **Equatoria**: R. Napere, 25 Nov 1937, *Wyld 347* (BM); Kpirabe, 19 Sep 1940, *Wyld 842* (BM).

TANZANIA. Sin. loc., 10 Dec 1930, *Musk 157* (EA, K). **Arusha**: Oldungo Lengai Rift-Wall, 9 Jul 1931, *St Clair-Thompson 204* (K). **Dodoma**: Mpwapwa, Mbuga Village, Kibakwe Division, Mbuga Ward, 31 May 2005, *Kindeketa et al. 2549* (EA, MO). **Iringa**: Ruaha National Park, top of Mpululu mountain, 21 May 1968, *Renvoize* & *Abdallah 2312* (EA, K). **Kagera**: Bukoba, 30 Oct 1992, *Breteler 11599* (MO). **Mbeya**: Rungwe, Ngumbulu Village, NE part of Rungwe Forest Reserve, 14 Mar 2008, *Abeid et al. 2848* (MO). **Morogoro**: Tanganyika, Ulugurus, Jan 1935, *Bruce 524* (BM, K). **Shinyanga**: Shinyanga, Nov 1938, *Koritschoner 2191* (EA, K). **Togo**. Lomé, 22 Sep 1976, *Ern et al. 883* (B); slopes of Bauman Peak, 14 Aug 1962, *Morton A4277* (MO).

UGANDA. sin. loc, May 1879, *Wilson 77* (K). **Central**: Kyadondo Mengo (U4); Nr Kanyanya, 16 Jun 1990, *Rwaburindore 2992* (MO). **Mengo**: Distr. W. Mengo, 4 mi Gayaza rd, 5 Jul 1980, *Rwaburindore 701* (MO). **Northern**: Terego, Upper Nile, Apr 1938, *Hazel 486* (K); Yumbe, 26 Nov 1941, *Thomas 4070* (EA, K). **Western**: Kigezi DFI, 28 Aug 1972, *Goode G3-72* (K); Kigezi, Kabale Distr., Rubanda County, Bubaale Subcountry, Kachwekano DFI, 25 Sep 2000, *Olet 48* (K); Kigezi, Kabale Distr., Rubanda County, Bubaale Subcountry, Kachwekano DFI, 25 Sep 2000, *Olet 49* (K); Kigezi, Bugangari, Rhuzumbura, Kigezi, Feb 1949, *Purseglove 2712* (EA, K); Budongo Nature Reserve, Bujenje County, Bunyoro Distr., 11 Nov 1971, *Synnott 737* (K).

UNITED KINGDOM. England: Hertfordshire, Rye Meads Sewage Works, nr Rye House, 1 Oct 1996, *Hanson s.n.* (K).

ZAMBIA. Copperbelt**,** Mufulira, 15 Nov 1968, *Mutimushi 2820* (K). **Lusaka**: Mt. Makulu Research Station, Mt. Makulu Res. Stn. 12 mi S of Lusaka, 9 Jun 1956, *Angus 1528* (K); 111 mi from Lusaka along Great East rd, 14 mi W turn-off to Feira 970 m, 30 Dec 1972, *Strid 2882* (K, MO). **North-Western**: Mwinilunga, Samukwakwa's, South Mwiwinilunga Distr., Sep 1934, *Trapnell 1453* (K). **Southern**: Livingstone. N. Bank of Zambesi, *Rogers 7402* (BM). **Western**: Nr Senanga, 30 Jul 1952, *Codd 7243* (K).

ZIMBABWE. **Mashonaland Central**: Imayanga, Nyamaropa TTk, 16 Jan 1967, *Biegel 1767* (MO); Mana Pools, Mana pool, Angwa River, 3 Jun 1965, *Bingham 1544* (K). **Matabeleland Nort**: Binga Distr., Sanyam R. and Zambezi R. confluence, Sep 1955, *Davies 1514* (MO); **Matabeleland North**: nr Binga, 6 Nov 1958, *Phipps 1368* (K); Binga, Chizarira Game Reserve, Busi River, 10 Nov 1971, *Thomson 481* (K). **Wankie**: Matetsi Safari Headquarters, 17 Dec 1981, *Gonde 371* (MO); Wankie Distr., Victoria Falls, Elephant Hills Hotel, 19 Dec 1978, *Mshasha 144* (MO).

Solanum tarderemotum

ANGOLA. Amboland, Feb 1884, *Schinz 907* (K); sin. loc, 17 Feb 1908, *Wellman 1776* (K); Pr. Lopolo (Embala), 8 Nov 1859, *Welwitsch 6035* (BM, K); Iter Benguellense, Distr. Mofsamedes, Bero, Jul 1859, *Welwitsch 6036b* (BM); in dumetosis ad cataractas pr. Lopolo, 11 Jan 1860, *Welwitsch 6036* (BM, K); Pungo Andongo, Apr 1857, *Welwitsch 6109* (BM, K). **Huila**: Ha in herbidis humidiusculis juxta ripas rivi de Sopollo, Dec 1859, *Welwitsch 6034* (BM, K). **Uíge**: Santa Cruz Mission, 12 Aug 1962, *Codd 7521* (K).

BOTSWANA. **North-West**: Ngami, Central Management Unit, Selinda Reserve, 8 Apr 2005, *Heath* & *Heath 1023* (K).

BURUNDI. Rwegura, Territoire Kayenza, 28 May 1969, *Lewalle 3620* (K). **Bugarama**: Teza, Prov. Muramvya, 29 Dec 1978, *Reekmans 7400* (EA, K, MO). **Bujumbura**: Katumba, 12 Dec 1979, *Reekmans 8444* (MO). **Muramvya**: Nyabigondo, 2 Feb 1967, *Lewalle 1536* (MO).

CAMEROON. Cameroon Mountain, 3 Jan 1926, *Dunlap 2* (K); Mt. Cameroon, 24 Dec 1958, *Morton 346* (MO). **Nord-Ouest**: Bui, Oku-Elak, 27 Oct 1996, *Cheek et al. 8455* (K, MO); Bui, Mt. Oku, KA, Oku, Kilum Reserve, 11 Jun 1996, *Etuge et al. 2305* (K); Boyo, Aboh, Gikwang-towards Nyasoso, 18 Nov 1996, *Etuge 3416* (K); Boyo, Ijim Mountain Forest, 21 Nov 1996, *Kamundi, et al. 673* (K, MO); Bui, Elak, Mt. Oku, 29 Oct 1996, *Munyenyembe et al. 790* (K). **Sud-Ouest**: Mt. Cameroon, 2 mi W of Mann's Springs, 12 Dec 1952, *Boughey GC-12516* (K); Mt. Cameroon, Upper Boando, 9 Dec 1993, *Cable* & *Mukete 498* (K); Mt Kupe, Kupe Village, 24 Nov 1999, *Biye et al. 55* (K); Mt Kupe, 24 Jan 1995, *Cheek et al. 7117* (K); Mt Kupe, Nyasoso, 23 Oct 1995, *Cheek et al. 7470* (K, MO); halfway between Madie village and Mbu-Bakundu, 3 Dec 1986, *Mambo et al. 282* (MO); Farmland, Bowango, Buea, 13 Nov 1960, *Ngomba 2* (K); Mt. Cameroon R.C., 24 Mar 1961, *Swarbrick SCA271* (E).

CAPE VERDE. **Santiago**: valley of Santo Domingo, 3 Nov 1839, *Hooker 117 bis* (K).

CHAD. **Lac**: Koulfe, Chari Central, 28 Jun 1903, *Chevalier 8788* (K, P). **N'Djamena**: c. 65 km S of Fort Lamy, 1 Jan 1965, *de Wilde et al. 5118* (K).

COMOROS. Iles Comores, *Boivin s.n.* (W); Mayotte, 1846, *Boivin s.n.* (W); Mohilla Island, 5 Apr 1861, *Meller s.n.* (K); Mohilla Island, Apr 1861, *Meller s.n.* (W). **Anjouan**: sin. loc, Jun 1875, *Hildebrandt 1626[a]* (BM). **Mwali**: Insul. Mohely, 1854, *Boivin s.n.* (BM).

DEMOCRATIC REPUBLIC OF THE CONGO. Foothills of Mountains West of Katana - Lake Kivu, 6 Jul 1959, *Cambridge Congo Expedition 1959 19* (BM); W of Katana, Lake Kivu, 29 Jul 1959, *Cambridge Congo Expedition 1959 207* (BM); Ruwenzori slopes, Oct 1908, *Kassner 3110* (BM, E, K); Lulongo, 4 Mar 1927, *Linder 2214* (GH); Ruwenzori, 1893, *Scott-Elliot 7821* (BM, K). **Katanga**: a 3 km de Lukuni (Katanga), source de la Kasapa, 12 May 1961, *Poelman 7* (K); Katuba, ferme Droogmums, Kaletele, Jan 1927, *Quarré 18* (GH); Haut-Lomani, Kamina, 1932, *Quarré 2898* (K); Haut-Lomani, Kamina, source de la Kasapa, 12 May 1961, *Symoens 8680* (K). **Nord-Kivu**: Butembo, 21 Feb 1974, *Baudet 487* (K); Lac Kivu, La Sebaya à Mgisa, 7 Mar 1953, *van der Ben 185* (K); NW slope of Mt. Vislke and saddle running North, 24 Feb 1975, *D'Arcy 8089* (MO); Kabara, SE base of Mt. Mikeno, 15 Mar 1975, *D'Arcy 8525* (MO); Lubarika, 1958, *Gilon 315[b]* (MO); Volcan Karisimbe, Aug 1937, *Lebrun 7466* (K); Katanda, Sep 1937, *Lebrun 7719* (K); Rutshuru, Sep 1937, *Lebrun 8318* (K); Rutshuru, Mosst Katale (Kivu), Dec 1957, *Lebrun 9163* (K); Volcan Karisimbe, Parc National Albert, 20 Aug 1937, *Louis 5419* (K); Upper Ruamoli Valley, 3 Aug 1952, *Ross 809* (BM); Ruamoli Valley, 5 Aug 1952, *Ross 840* (BM); Ngungu, 18 km SW Sake, 11 Aug 1954, *Stauffer 48* (K, P); Kibati, Virunga-Kette, 1 Oct 1954, *Stauffer 567* (K, P); Parc National Albert, 10 Jun 1954, *de Witte 10287* (K). **Orientale**: Lac Albert, village Makatji, un peu au S de Ndaro, 20 Apr 1954, *van der Ben 1430* (K); Ituri, Kibali, Aru, Aug 1931, *Lebrun 3575* (GH, K); Ituri, Lodjo Camp, 27 Oct 2010, *Luke* & *Bujo 14736* (K). **Sud Kivu**: Lac Tsimuka, plaine de la Ruzizi, Jan 1950, *Germain 5593* (K); Mushuva, 2 Dec 1940, *Hendrick 1488* (K); Kalongé, Kalonge, long riviere Nyamwamba endroit fran et ombrage, 12 Feb 1953, *de Witte 10477* (MO).

EGYPT/SUDAN. Nubia, *Kotschy 184[a]* (BM).

ERITREA. **Semienawi Keyih Bahri**: Massawa, Massana, Ras Madour, 4 Feb 1892, *Schweinfurth* & *Riva 59* (K); c. 10 km S of Nefasit, 2 Feb 1969, *de Wilde 4502* (K).

ETHIOPIA. Abyssinia, Sep 1872, *Hildebrandt 475* (BM); Gudaja, 28 Oct 1954, *Mooney 6232* (K); Wollega, Siré-Lekemti, 1957, *Rankin s.n.* (E); 2 Sep 1862, *Schimper 129* (BM); Bellaka, 8 Nov 1854, *Schimper 509* (FT, W). **Amhara**: upper Ghiedeb Valley, nr Wed Mkt., on Mota rd, 4 Aug 1957, *Evans 412* (K); South Gondar, 4 km N of Debre Tabor, 13 Sep 2004, *Friis et al. 11552* (K); Zegie village, Zeghie Peninsual, Lake Tana, nr Zeghie Village, 26 Oct 1964, *Meyer 8635* (K); Semien Mountains, 1 Oct 2003, *Wieringa 4971* (K). **Dire Dawa**: Dire Dawa, 16 Oct 1969, *Parker 580* (K). **Harari**: between Harrar and Abbaba, Sep 1901, *Wellby s.n.* (K). **Oromia**: Debre Zeit, crater Lake, c. 40 km S of Addis Ababa, 7 Oct 1961, *Albers 61067* (K); Gara Mullata, c. 50 km due W of Harar, 2 Aug 1962, *Burger 2006* (FT, K); Belleta Forest, Kaffa Prov., c. 40 km SW of Jimma on the Bonga rd, 9 Nov 1970, *Friis et al. 203* (K); 38 km S of Jimma at the Sheki-Goreb river rd, 7 Dec 1972, *Friis et*

al. 1621 (K); at bridge across Gabba River, 33 km N of Mettu, 15 Dec 1972, *Friis et al. 1740* (K); Gore to Moccha, 25 km S of Gore, 19 Dec 1972, *Friis et al. 1855* (K); Jimma Agricultural School, 1 Jan 1973, *Friis et al. 2047* (K); c. 3 km N of Yavello along main rd to Agere Maryam (Sidamo region), 25 Nov 1997, *Friis et al. 8263* (K); Arero State Forest, 17 Dec 2002, *Friis et al. 11022* (K); Kelem Welega, unfinished rd S of Gidami, 12 km S of Gidami towards Dembidolo, 17 Nov 2012, *Friis et al. 14694* (K); Hana, E part of the Omo, 23 Mar 1976, *Fukui 15* (EA, K); E slope of Didessa Valley, Wollega region, 12 Sep 1975, *Gilbert* & *Thulin 651* (K, MO); c. 4 km S of Thibe, Shoa region, 19 Sep 1975, *Gilbert* & *Thulin 877* (K, MO); Sidamo, Neghelle, 21 Oct 1990, *Haugen 1740* (K); C. 45 km from Alemaya-Aire Dgwa rd, Hargege Region, 23 Oct 1973, *Jansen 4290* (MO); Beletta Forest, 27 Jul 1962, *Mooney 9164* (FT, K); Wourambouldi, Sep 1926, *Omer Cooper s.n.* (K); 92 km W of Harar-Dire Dawa rd; 4 km W of Baroda (Hararge Prov.), 11 Dec 1964, *Perdue 6363* (K); Guder, 145 km W of Addis Ababa, 23 Feb 1958, *Piffard 24* (K); Ghimbi to Gore, 1 Apr 1958, *Piffard 88* (K); Jimma, campus of institute of Agriculture, 20 Aug 1972, *Seegeler 2399* (MO); Jimma, 12 Jun 1958, *Siegenthaler X28* (EA, K); Gerin Farm, 1 km E of Jimma, 15 Feb 1956, *Stewart E10* (EA, K, K); 37 km from Shashemene, rd Koffale to Dedolla, 8 Sep 1967, *Westphal* & *Westphal-Stevels 1735* (MO); rd Bedeno, to Anya, 1 km from Bedeno, along rd in Sorghum field, 27 Oct 1967, *Westphal* & *Westphal-Stevels 2453* (MO); Shsshamene to Soddu, 97 km SW of Shashmene, 10 May 1968, *Westphal* & *Westphal-Stevels 4043* (K, MO); c. 5 km N of Addis Ababa, 3 May 1965, *de Wilde* & *de Wilde-Duyfjes 6507* (K, MO). **Somalia**: Harerge, on the rd from Alemaya to Asbe Tafari, 6 km from Kobbo, 16 Aug 1967, *Westphal* & *Westphal-Stevels 1227* (K). **Southern Nations (SNNP)**: Lower Omo River, 7 Oct 1970, *Carr 875* (EA, K); 38 km S of Aman, towards Berhan village, 13 Nov 1995, *Friis et al. 7133* (K); Bonga, Keffa Region, 21 Jul 1975, *de Kruif 2186* (MO); along path to Bonga waterfall, Keffa Region, 24 Mar 1976, *de Kruif 5550* (MO); Shashamene to Soddu, 90 km W of Shashamene, 14 Nov 1964, *Meyer 8759* (K); Little Gesha, 25 Feb 1960, *Mooney 8719* (FT, K); Bonga, Roman Catholic Mission, Keffa region, 17 Mar 1973, *Seegeler 3227* (MO); Chichu, c. 8 km S of Dilla, 24 Aug 1967, *Tadesse Ebba 597* (K); rd from Jimma to Serbo, 14 km from Jimma, 1 Aug 1968, *Westphal* & *Westphal-Stevels 5499* (K, MO).

GHANA. Ohamu, Agric. Res. Station, Jul 1961, *Irvine 4957* (K).

GUINEA. **Nzérékoré**: Nzo, Mt Nimba, 5 Nov 1969, *Adam 24671* (MO).

KENYA. Loita Plains, 30 Jun 1923, *Curtis 613* (GH); Loita Plains, 30 Jun 1923, *Curtis 622* (GH); nr hot springs, Loita Plains, 21 Jul 1923, *Curtis 780* (GH); sin. loc., Sep 1916, *Dowson 482* (EA, K); Af Mt Elgon, 14 Jun 1920, *Lindblom s.n.* (S); Mt. Elgon, Oct 1930, *Lugard 209* (K); Lake Naivasha, British East Africa, 17 Jul 1909, *Mearns 831* (BM); Kichich, Mathews Ranch (K1), 23 Dec 1959, *Newbould 3542* (K); Mt. Kenya, Feb 1914, *Orde Browne s.n.* (BM); Naivasha, 1926, *Prescott-Decie s.n.* (BM). **Central**:

Kiambu, Limuru, Kiambu Dist, Jul 1980, *Gachathi s.n.* (MO); Kiambu, c. 9 km E of Kieni on rd to Thika, 14 Mar 1982, *Gilbert 7043* (EA, K); Kirinyaga, Castle Forest Station, 4 Apr 1970, *Gillett* & *Mathew 19099* (EA, K); Murang'a, Kimakia Forest Reserve, East Aberdares, 28 Jul 1958, *Kerfoot 637* (EA, K); Muranga, Kimakia Forest Station, East Aberdares, 28 Jul 1958, *Kerfoot 638* (EA, K); Nyandarua, Wamuhu, Kinangop Forest Reserve, 23 Nov 1959, *Kerfoot 1419* (EA, K); Nyeri, Naro Mora Route, Mt. Kenya, 10 Jun 2010, *Kirika et al. 75* (K); Kiambu, Muguga, Nr Scott Labs, 11 May 1932, *McDonald 1548* (EA, K, MO); Vicinity of Lake Naivasha, 17 Jul 1909, *Mearns 842* (GH); Nyeri, 18 Jan 1933, *Napier 2455* (EA, K); Embu, Mt. Kenya forest, Vicinity of Kamwete Forest Station Rest House, 19 Dec 1966, *Perdue* & *Kibuwa 8280* (EA, K); Kiambu, Limuru, 5 Jun 1918, *Snowden 564* (BM, K); Embu, 17 Apr 2010, *Vorontsova et al. 194* (BM, EA); Meru North, Mt. Kenya area, 18 Apr 2010, *Vorontsova et al. 198* (BM, BR, EA, K, MO, NY); Kiambu, Kikuya and on rd to Eldama Ravine, Sep 1898, *Whyte s.n.* (K). **Coast**: Mwanda, Mgange Nyika, 3 Oct 1971, *Klungness 74* (K). **Eastern**: Makueni, Chyulu Hills, 15 May 1938, *Bally 7787* (EA, K); Marsabit, Moyale, 28 Apr 1952, *Gillett 12957* (EA, K); Marsabit, Moyale, 1952, *Gillett 14057* (EA, K); Samburu, Mathews Range, *Luke 14111* (EA, K); Marsabit, Mt. Kulal, 13 Jan 1977, *Masheti,* & *Gagah H 314* (EA, K); Marsabit, Narangani, Mt. Kulal, 8 Jun 1960, *Oteke 87* (EA, K). **Nairobi**: Nairobi, 1924, *McDonald 808* (K); nr Nairobi, *Whyte s.n.* (K). **Rift Valley**: Nakuru, Lake Naivasha, 21 Jul 1952, *Bally B8235* (EA, K); Uasin Gishu, 25 mi NNE of Eldoret, 23 Jun 1948, *Bogdan 1774* (K); Narok, Suswa, volcanic cave area, 6 Apr 1963, *Glover 3608* (EA, K); Samburu, Mathews Range, Situi Forest, 10 Jun 1959, *Kerfoot 1103* (EA, K); Samburu, Mathews Range, Kichich River, 6 Dec 1960, *Kerfoot 2440* (EA, K);

Mt. Elgon, Kitale, 27 Dec 1960, *Löffler E-107* (W); Kericho, Southwestern Mau Forest Reserve, camp 7, river Dimbilil, 7 Aug 1949, *Maas Geesternanus 5603* (K, MO); Turkana, from Marsabit on Kulal Mtns., Jul 1934, *Martin 230* (K); Katilia forest, 12 mi NNE of Kangetet, Kerio River, 25 May 1970, *Mathew 6379* (EA, K); Trans Nzoia, 15 Sep 1984, *Mungai 138 84* (EA, K); South Turkana, Lokora, 28 Jul 1969, *Mwangangi 1397* (EA, K); Nakuru, Lake Naivasha, 1 May 1932, *Napier 1847* (K, P); Samburu, Maralal area, 1/4 mi E of Mwamur Dam, 25 Mar 1968, *Nesbit Evans 53* (K); Nakuru, Londiani, 15 Nov 1967, *Perdue* & *Kibuwa 9065* (K); K6 Rift Valley, Suswa volcano, 1 Jun 1997, *Phillipson* & *Bytebier 4783* (MO); Nakuru, Lake Naivasha, 31 Oct 1965, *Polhill 145* (K); Nakuru, Lake Naivasha, 12 May 1972, *Polhill 148* (EA, K); Trans Nzoia, SW of Mt. Elgon, 12 Aug 1958, *Symes 392* (EA, K); Trans Nzoia, NE Elgon, Aug 1948, *Tweedie 725* (K); Trans Nzoia, NE Elgon, Dec 1957, *Tweedie 1481* (K); Nandi, 1898, *Whyte s.n.* (K); Eldoret, Kaposoret Forest Reserve, 15 Jun 1951, *Williams 241* (EA, K).

Western: Kakamega, Kaptiki Secondary School, 11 Nov 1984, *Hohl 157* (EA, W); pr. Forest Station ad mar, Mt Aberdare Expedition, 14 Jan 1922, *Rob* & *Fries 921* (MO).

LESOTHO. Sehlabathebe, 4 Jan 1973, *Guillarmod et al. 138* (K).

MADAGASCAR. **Antananarivo**: Ambohidratrimo, Ambohimanga, près de Tananarive, 19 Apr 1928, *Decary 6179* (P); Antananarivo-Nord, Tananarive, *Waterlot s.n.* (P). **Toliara**: valley half a mi W of Ampoza, 5 Sep 1929, *White s.n.* (BM).

MALAWI. Distr. Kasungu, Mtunthama, Kamuzu Academy, 20 Feb 1979, *Blackmore et al. 534* (BM); Shiri Highlands, 1885, *Buchanan s.n.* (E); Nyasaland, 20 Apr 1961, *Chapman 1245* (MO); Blantyre, 1895, *Medley Wood 7077* (E). **Central**: Dedza Distr., Dedza mountain forest, 19 Jan 1987, *Balaka* & *Patel 1861* (MO); Lilongwe, Dzalanyama Forest Reserve, valley NW of Kazuzu Hill, 24 Feb 1982, *Brummitt et al. 16107* (K); Dedza, Damwe Hill, 1 Apr 1961, *Chapman 1219* (K); Salima Distr., Liganga village A. Mpemba, along Linthipe river, 17 Jun 1985, *Kaunda et al. 219* (MO); Dedza, in Dambo, 13 Jan 1987, *Patel* & *Balaka 3607* (MO); Dedza, 5 Apr 1976, *Pawek 14222* (MO); Salima Distr., Luwadzi stream, 14 Jun 1985, *Salubeni et al. 4242* (MO). **Northern**: Chitipa, Mafinga Mountains, 2 Mar 1982, *Brummitt et al. 16264* (K); Rumphi Distr., Nykia Plateau, 2 mi E of Chelinda, 4 Mar 1977, *Pawek 12428* (K, MO); Tazima, Nyika Plateau, 2 Jan 1976, *Phillips 843* (MO); Northern Prov., Malawi, Dam 11, 23 Mar 1976, *Phillips 1519* (K, MO). **Southern**: Zomba Plateau, N side of Chiradzulu, 27 Jan 1979, *Blackmore 210A* (BM); Namadzi, Namasi, 23 Mar 1899, *Cameron 13* (K); Mt. Mulanje, foot NE slopes of Namasile opposite Sombani hut, 3 Jan 1971, *Hilliard* & *Burtt 6134* (E); Zomba, Mpita Tobacco Estate, Thondwe, 17 Apr 1984, *Kaunda* & *Salubeni 44* (MO); Thylolo Distr., Bvumbwe, 22 May 1985, *La Croix 2909* (MO); Zomba Distr., Mpita Estate, Thondwe, 18 Apr 1986, *Salubeni* & *Kaunda 4444* (MO); Blantyre Distr., Michiru Hill, 8 Jul 1986, *Salubeni* & *Tawakali 4600* (MO); Zomba, Chuka Village, Chisi Island, Lake Chilwa, 7 Nov 1986, *Salubeni* & *Kwatha 4818* (K); Blantyre, Michiru Mountain forest, 28 Jul 1989, *Tawakali* & *Kaunda 1566* (MO).

MALI. Ackerrand kurz vor Koulikoro, 29 Sep 1992, *Ehrich 336* (B).

MOZAMBIQUE. **Maputo**: Lourengo Marques, Namaacha, 1 Aug 1967, *Marques 2142* (MO). **Zambezia**: Namuli Mountain, Muretha Plateau, 26 May 2007, *Harris 186* (K).

NAMIBIA. Kaokoveld, 15 Aug 1956, *Story 5799* (K). **Kavango West**: Tondoro Mission, Tondoro Camp 1 km E of Mission, 15 Dec 1955, *de Winter 3955* (K). **Zambezi**: E Caprivi, Zipfel, Lizazuli, 2 Jan 1959, *Killick* & *Leistner 3253* (K).

NIGERIA. 1912, *Dalziel s.n.* (BM); West Block, Gambari [Experimental Station], Idaban, Nov 1962, *Swarbrick 2875* (E). **Bauchi**: Toro, Panshanu Pass, 15 Aug 1962, *Lawlor* & *Hall 410* (K). **Cross River**: probably collected nr Obudu Cattle Range, 25 May 1971, *Meer van, 1861* (MO). **Enugu**: Nsukka, University of Nigeria campus, 23 Jan 1962, *Okigbo, 57* (K). **Osun**: Shasha Forest Reserve, 23 Apr 1968, *Gledhill, 998* (K).

RWANDA. Au km 47 de la route Kigeme-Gisovu, 2 Mar 1972, *Auquier 2721* (H, MO); 15 Feb 1975, *D'Arcy 7793* (MO). **Northern**: Parc des Volcans, Karisimbi/Visoke saddle, 12 Feb 1975, *D'Arcy 7616* (K, MO); Musanze, base of Mt. Visoke, Ruhengeri, 9 Feb 1975, *D'Arcy 7655* (MO); Parc des Volcans, Karisimbi/Visoke saddle, 12 Feb 1975, *D'Arcy 7716 a* (MO); Musanze, Ruhengeri, 21 Mar 1975, *D'Arcy 8604* (MO); Mt. Visoke, 6 Apr 1970, *Fossey 16* (EA, K).

SENEGAL. Nr. Bono, nec non Ins. Bonavista, *Brunner s.n.* (K); Dec 1823, *Roger 17* (K).

SIERRA LEONE. Tingi Mountains N Kono, 15 Dec 1965, *Morton* & *Gledhill SL-3143* (K); Roniesta, 17 Nov 1914, *Thomas 5435* (K). **Northern**: Mt. Bintumani, Kabala (admin), Mt Loma, Bintumane, Mira, 30 Nov 1965, *Adam 22258* (GH, MO); Bintumani, Mt. Loma, 16 Aug 1945, *Jaeger 1050* (K, P); Koinadugu, Loma Mountains, 5 Feb 1952, *Jaeger 4279* (K); Bintumani, Loma Mountains, 31 Dec 1963, *Morton SL-445* (K); Bintumani, 31 Dec 1963, *Morton SL-446* (K); Bintumani, 18 Nov 1965, *Morton SL-2644* (K).

SOMALIA. Mait Escarpment, 31 Oct 1956, *Bally B11292* (EA, K). **Banaadir**: 3 km fro Muqdisho airport along rd to Jasiira, 4 May 1990, *Thulin* & *Hedrén 7172* (K); **Togdheer**: Wagga Mt., 1905, *Bury s.n.* (BM). **Woqooyi Galbeed**: Murak, Sep 1933, *Godding 162* (K); mountains above Qoton, 11 Feb 2002, *Thulin 10906* (K).

SOUTH AFRICA. Standerton, Transvaal, 25 Jan 1905, *Burtt Davy s.n.* (BM); sin. loc., 1837, *Drège s.n.* (W); Zulu-Land, *Gerrard 412* (BM, K). **Eastern Cape**: Somerset, 1860, *Cooper 528* (K); Fort Beaufort, 1860, *Cooper 554* (K, W); Uitenhage, Enon, *Drège s.n.* (K). **Gauteng**: Pretoria, Region SWA, Kaokoveld, Otjomborombonga on Kunene River, 13 Jul 1976, *Leistner et al. 104* (K, MO); **KwaZulu-Natal**: Weza, Ingeli, 5 Mar 1972, *Strey 10899* (K, MO). **Mpumalanga**: Matebe Valley, May 1883, *Holub s.n.* (K). **North West**: nr Vryburg, 60 mi NW of Vryburg, Kalahari Desert, 5 Feb 1948, *Rodin 3500* (K, MO).

SOUTH SUDAN. **Bahr El Ghazal**: Isablei [?], Lande der Bongo, 26 Nov 1869, *Schweinfurth 2649* (K). **Equatoria**: Imatong Mountains, Mt. Angargi, 14 Jun 1939, *Andrews 1947* (K); Distr. Torit, Lowiliwili, Imatongs, 14 Nov 1949, *Jackson 902* (BM). **Greater Upper Nile**: Upper Nile, Boing, 26 Oct 1951, *Sherif A2891* (K).

SUDAN. ad Cordofanum Milbes, 4 Dec 1839, *Kotschy 291* (BM, E, GH, K, P, W). **Blue Nile**: Sennar, *Würtemmburg s.n.* (W). **Darfur**: Jebel Marra, Nyertete, 21 Jan 1964, *Wickens 1044* (K); Jebel Marra, Zalingei, *Wickens 1776* (K). **Kassala**: Erkowit, Red Sea Hills, Mar 1929, *Lady Maffey 39* (K); Nr Kamobsana, Red Sea Prov., 25 Jan 1912, *MacDougal* & *Sykes 137* (BM); Kamobsana, Red Sea Prov., 25 Jan 1912, *MacDougal* & *Sykes 139a* (BM); Al Qadarif, Gallabat, 1865, *Schweinfurth 1403* (BM).

TANZANIA. Sin. loc, 1884, *Johnston 76* (K); Tabera, Aug 1937, *Lindeman 352* (BM); Mt Mbiya, 16 Mar 1938, *MacInnes 154* (BM); Sin. loc., 11 Jan 1934, *Schlieben 4527* (BM). **Arusha**: Sin. loc., 2 Jun 1993, *Ellemann 613* (MO); Monduli, Ketumbeine Forest Reserve, SW of Losirwa Village, 14 Feb 2001, *Festo 905* (MO); Ngorongoro, Empakaai Crater, Ngorongoro conservation area, 10 Aug 1972, *Frame 24* (EA, K, MO); Monduli, Ketumbeine Forest Reserve, 1 Apr 2000, *Gobbo et al. 666* (MO); Mbulmbul, Block DL, 49, 24 Jun 1944, *Greenway 6951* (EA, K); Arumeru, Arusha National Park (ANAPA), Themi water pipe area, 22 Sep 2008, *Kayombo et al. 5641* (MO); Monduli, Ketumbeine Forest Reserve, W of Elang'atadapashi, S peak of Ketumbeine, 1 Apr 2000, *Kindeketa 254* (MO); Arumeru, Kivesi Hill, E of Arusha municipality, 26 Apr 1999, *Massawe et al. 257* (MO); Ngorogoro Crater, 24 Jun 1938, *Pole Evans* & *Erens 936 A* (E, K, P); Ngurdoto Crater National Park, Ngurdoto Crater Forest, 5 May 1965, *Richards 20349* (EA, K); Arumeru, Mt. Meru, Olmotonyi, 21 Feb 1969, *Richards 24130* (K); Monduli, Monduli Forest Reserve, Mwandeti Forest, 23 Jan 2001, *Simon 698* (MO); Hanang, Hanang Mountain, Jerdom valley, 15 Jun 2014, *Vorontsova et al. 1647* (EA, K, NHT). **Buha**: Kakombe Valley, E shore of Tanganyika Lake from Gombe stream to Missonge, 25 Dec 1963, *Pirozynski P86* (EA, K). **Eastern**: Morogoro, Lukwangulu Plateau, Uluguru Mts, 19 Sep 1970, *Thulin* & *Mhoro 1048* (K). **Iringa**: Ludewa, Livingstone Mountains, Broad saddle NW of summit of Mt. Msalaba, 21 Mar 1991, *Gereau* & *Kayombo 4426* (MO); Mufindi, Igowole, 30 May 1989, *Kayombo 638* (MO); Ulanga, Uzungwa Mountains, 22 Jul 1984, *Lovett 327* (K); Mufindi, Ngwazi, 10 Feb 1987, *Lovett 1489* (K, MO); Mufindi, Lake Kihanga, 24 May 1987, *Lovett* & *Lovett 2192* (K, MO); Kidatu, Iringa Distr., E dam site, 28 Mar 1971, *Mhoro 864* (EA, K); Mt. Image, 50 mi NE of Iringa, a litlle N of Morogoro rd, 3 Mar 1962, *Polhill* & *Paulo 1656* (EA, K); Ruaha National Park, Mbagi camp, 14 Feb 1960, *Richards 21308* (BM, EA, P). **Kagera**: Bukoba Rural, Minziro Forest Reserve, W of Minziro, 20 Mar 2001, *Festo* & *Francis 1064* (MO); Bukoba Rural, Minziro Forest Reserve, 21 Apr 2001, *Festo et al. 1339* (MO); Karagwe, 1 Mar 1862, *Speke* & *Grant 453* (K). **Kigoma**: Mpanda, Sisaga, Mahali Mountains, 27 Aug 1958, *Jefford et al. 1818* (K); Kakombe, 10 mi W of Kigoma, 7 Jul 1959, *Newbould* & *Harley 4285* (K, MO). **Kilimanjaro**: Moshi, Marangu, 13 Aug 1968, *Batty 233* (K); Osirwa Farm, TBL Estates, Kilimanjaro, 26 Jan 1994, *Grimshaw 94-145* (K); Moshi, Weru-Weru gorge, 22 Feb 1955, *Huxley 116* (EA, K); Same, Chome Forest Reserve,

Namboja, 30 Mar 2001, *Mlangwa et al. 1506* (MO). **Mbeya**: Mbeya, 3 Mar 1932, *Davies 461* (EA, K); Rungwe, 19 Sep 1932, *Geilinger 2482* (K); Mbeya, 16 Dec 1989, *Lovett et al. 3731* (MO); Rungwe, Ngozi, Poroto Mountains, 17 Oct 1956, *Richards 6571* (K); Mbosi Circle, Ndungu Estate, 11 Jan 1961, *Richards 13858* (K); Rungwe Mountain, W slopes, *St Clair-Thompson 854* (K); Rungwe, Nyassa Hochland - Station Kyimbila, 1911, *Stolz 384* (B, BM, K, MO, W). **Mbeya/Njombe**: Poroto Mountains, Mbeya Distr., 16 May 1957, *Richards 9735* (EA, K). **Mbulu**: Lake Manyara National Park, 2 Dec 1963, *Greenway* & *Kirrika 11113* (EA, K); Hanang Mt., 3 May 1962, *Polhill* & *Paulo 2302* (EA, K). **Morogoro**: Kiberege, Mar 1936, *Culwick 2* (K); Kanga Mountains, 11 May 2007, *Luke et al. 12026* (EA, K, MO); Uluguru North Forest Reserve, 17 Jul 1972, *Mabberley*, *1169* (K); Kilombero, Udzungwa Mountains National Park, T6, 16 Oct 1998, *Mwangulango* & *Massawe 45* (MO); Lukwangule Plateau, Uluguru Mountains, 19 Sep 1950, *Thulin* & *Mhoro 1048* (EA, K). **Mwanza**: Mwanza, *Davis 201* (K). **Njombe**: Tanganyika, Milo - Livingstone Mts, 1935, *Rae s.n.* (BM); Stromgebeit des obern Ruhudje, Landschaft Lupembe, nordlich des Flusses, Mar 1931, *Schlieben 413* (K). **Rukwa**: Nsanga Forest, Mpanda, Ufipa, 8 Aug 1960, *Richards 13004* (K). **Ruvuma**: Songea, Matagoro Hills, just S of Songea, 22 Feb 1956, *Milne-Redhead* & *Taylor 8869* (EA, K); Songea, Lupembe Hill, 20 May 1956, *Milne-Redhead* & *Taylor 10387* (EA, K); Songea, Luwiri Kitesa, 24 May 1956, *Milne-Redhead* & *Taylor 10430* (K); Songea, c. 1.5 km SW of Mpapa, Matapo Hills, Songea Distr., 25 May 1956, *Milne-Redhead* & *Taylor 10440* (EA, K). **Shinyanga**: Shinyanga, Nov 1938, *Koritschoner 1903* (EA, K). **Tabora**: Unyamwezi, Mininga, 1860, *Speke* & *Grant 79* (K). **Tanga**: Amani, *Herb. Amani*, *3580* (K); Usambaras, between Ngua and Magunga Estates, 17 Jul 1953, *Drummond* & *Hemsley 3347* (EA, K); Amboni, Jun 1893, *Holst 2840* (K); Lushoto, Mtae, 6 Feb 1987, *Kisena 529* (K); Lushoto, Camphor, nr Staff quarters, 10 Mar 1969, *Shabani 335* (EA, K); Lushoto, Campher, 26 Dec 1969, *Shabani 513* (EA, K); Kigombe, Madanga, Pangani, Tanga Prov., 20 Jul 1955, *Tanner 1975* (K); Kibubu, Mkuzikatani, Madanga, Pangani, Tanga Prov., 24 Jul 1952, *Tanner 3622* (K); Muheza, Amani, East Usambara, 14 Dec 1995, *Vainio-Mattila et al. 95-53* (H, MA). **Ufipa**: Sumbawanga, 3 km SE of Moravian Mission at Nkutwe nr Tatanda, 31 Oct 1992, *Harder & Kayombo 1351* (EA, MO). **Ulanga**: Ifakara, 16 Jul 1959, *Haerdi 286o* (EA, K).

UGANDA. Kitagweta Toro, 30 Jul 1906, *Bagshawe* & *Camb 1121* (BM); Serima Teas, Dec 1931, *Chandler 298* (K); Kampala, Aug 1931, *Lab Staff (Uganda Agric Dept)*, *2153* (K); 1893, *Scott-Elliot 7248* (BM). **Central**: Wakiso, Nakyesanja, nr Kawanda, 7 Mar 1972, *Daumlira D-35* (K); Bumbu-Kiteezi, 7 Mar 1972, *Daumlira 36* (K); Mukono, Kipayo, Dec 1913, *Dümmer 563* (BM, K, P); Mengo, 12 mi to Kampala, Entebbe rd, May 1932, *Eggeling 697* (EA, K); Mukono, Kituza, c. 35 mi SE of Kampala, Agric. Dept. Coffee Research Station, Jun 1957, *Griffiths 47* (K); Mengo, Budo, 9 Nov 1972, *Katende K1722* (EA, MO); Mengo, Budo, 9 Nov 1972, *Katende 1723* (EA, MO); Mubende, 0.5 km W of Kasanda trading centre, West singo county, 10 Aug 1974, *Katende K1239* (MO); Bulimezi, Luwelo, 1924, *Maitland s.n.* (K); Kampala, 24 Jun 1915, *Maitland 147* (K); Masaka, Katera, Oct 1925, *Maitland 916* (K); Mengo, County Kyadondo, Distr. W. Mengo, Kikaaya Hill, 6 Nov 1982, *Rwaburindore 1060* (MO); Wakiso, Namanve Forest, Manga Distr., 12 Feb 1963, *Tallantire* & *Lua T-633* (K). **Eastern**: Bugisu, Budadiri, Bugisha, nr River Sisoko, Jan 1932, *Chandler 458* (K); Mt. Elgon, 500 m E from the ladders, Sasa pat w-ern section of Mt Elgon, 25 Jan 1993, *Katende* & *Sheil 1099* (K); Mt. Elgon, ridge between Kajeri and Sisa rivers, 13 Mar 1993, *Kisalye* & *Van Heist 515* (K); Bugishu, Mt. Elgon, Bulambuli, Aug 1934, *Synge 856* (BM); 1.5 km ESE of Atari River gorge, N and NW section of Mt. Elgon, plot 45, 16 Jan 1993, *Tiyoy 1209* (K); nr Bulambuli, Mt. Elgon, 12 Nov 1933, *Tothill 2393* (K); Mt. Elgon, NE Elgon, Dec 1957, *Tweedie 1480* (K); Mt. Elgon, 28 Dec 1996, *Wesche 500* (K); Busoga villages, 13 Dec 1898, *Whyte s.n.* (K). **Northern**: Karamoja, Moroto Township, Sep 1958, *Wilson 609* (EA, K). **Western**: Masindi E Albert, South Nyakafunjo Block, 21 May 1951, *Dawkins D-752* (EA, K); Kigezi, 28 Aug 1972, *Goode G-1 -72* (K); Kigezi D.F.I, 28 Aug 1972, *Goode G-2-72* (K); Toro, Fort Portal, 4 Apr 1932, *Hazel 219* (K); Mihunga, Ruwenzori, 12 Jan 1939, *Loveridge 350* (A, K, MO); Kabale, Kigezi, Kabale Distr., Ndorwa County, Kyanamira subcounty, Kyonyo parish, 7 Jan 2000, *Olet 46* (K); Kigezi, Kabale Distr., Ndorwa County, Kyanamira subcounty, Kyonyo parish, 7 Jan 2000, *Olet 47* (K); Mbarara, Rivebikoona, Kamakuzi Division, May 2002, *Olet 106* (K); River Nyamugasani, W Ridge, 23 Aug 1952, *Osmaston 2328* (BM); Mt. Ruwenzori, Aug 1938, *Purseglove 308* (K); Kigezi, Kachwekano Farm, Jul 1949, *Purseglove 3035* (EA, K); Kigezi, Muhavura Hill, 11 Jan 1933, *Rogers 337* (BM, EA,

K); Mahoma Valley, nr Nyabitaba, 9 Jul 1952, *Ross 489* (BM); Above Kichuchu, Mituku Valley, 15 Jul 1952, *Ross 576* (BM); Lume Valley, 23 Aug 1952, *Ross 936* (BM); Ruwenzori Mountains, Mar 1893, *Scott-Elliot 7659* (K); Toro, Nyabesuro, 18 Feb 1932, *Shillito 58* (K); Toro, Virunga Mountains, Sabinio, 24 Nov 1934, *Taylor 2014* (BM, MO); Kigezi, Virunga Mountains, Lake Bunyoni, 27 Nov 1934, *Taylor 2158* (BM); Ruwenzori, Namwamba Valley, Kilembe, 19 Dec 1934, *Taylor 2503* (BM); Kigezi, Rubaya, 4 Jul 1945, *Thomas 4266* (EA, K).

ZAMBIA. NW Rhodesia, Oct 1908, *Allen 724* (K); 1895, *Buchanan 7* (BM, E); 4 mi S of Dedza, 2 Feb 1959, *Robson 1427* (BM). **Lusaka**: Mt. Makulu, Kafue Basin, 10 Apr 1963, *van Rensberg 1886* (K). **North-Western**: Mwinilunga, 4 Oct 1937, *Milne-Redhead 2565* (K). **Northern**: Mbala, Chilongowelo, 19 Mar 1952, *Richards 1183* (K); Mbala, Chilongowelo, Abercorn Distr., 22 Jan 1955, *Richards 4216* (K); below Kwimbi Mission, 10 Feb 1955, *Richards 4426* (K); Mbala, Itembwe Gorge, Abercorn Distr., Abercorn, 15 Jan 1964, *Richards 18770* (K). **Southern**: Livingstone Distr., Victoria Falls, 21 Feb 1997, *Luwiika et al. 460* (MO); Kalomo, Kalomo, May 1909, *Rogers 8225* (K).

ZIMBABWE. Mt. Nuza, Jan 1935, *Gilliland 1402* (BM). **Harare**: Salisbury, 26 Feb 1927, *Eyles 4712* (K). **Manicaland**: Nr Chirinda, May 1906, *Swynnerton 481* (BM, K); Chimanimani, Apr 1906, *Swynnerton 342* (BM). **Mashonaland Central**: Mazowe, Umvukwes, 17 Dec 1952, *Wild 40771* (K). **Mashonaland East**: Distr. Salisbury, Mandara, 25 Sep 1974, *Bisgel 4632* (MO); Marandella, 8 Apr 1948, *Corby 1-20917* (K). **Matabeleland South**: Mazowe, Matobo National Park, Matopus NP, 24 Feb 1981, *Philcox* & *Leppard 8823* (K).

Solanum triflorum

AUSTRALIA. Sin. loc, Mar 1932, *Without Collector s.n.* (NSW); Sin. loc, Mar 1920, *Without Collector s.n.* (NSW). **New South Wales**: nr Willowvale Homestead, Tennant Distr., 17 Feb 1963, *Adams 537* (CANB, NSW); Yass Valley, Taemus, 1 Apr 1962, *d'Arnay 272* (CANB); Snowy River, 3 mls S of Cooma, 13 Mar 1962, *Beeton s.n.* (CANB); Above Cotter River, 8 Feb 1962, *Beeton s.n.* (CANB); Snowy River, 3 km NW of Dry Plain at turn to Caddicat, c. 25 km NW of Cooma, 19 Feb 1971, *Briggs 4046* (NSW); Pine Island Reserve, Murrumbidgee River, 23 Jan 1947, *Burbidge s.n.* (CANB); Cotter Hills, 8 Feb 1962, *Burgess s.n.* (CANB); Cowra, 28 Jan 1976, *Butler s.n.* (NSW); Queanbeyan, Queanbeyan Stockyards, 5 Feb 1931, *Calvert KEW-84* (CANB, MEL, PERTH); Murrumbidgee River, Dew's Corner, ca. 4 km S of Fairvale Homestead, 8 May 1987, *Canning & Lloyd 6421* (AD, CANB, NSW); Curtin, corner of Groom and Carruthers Streets, 21 Dec 1988, *Canning 6549* (CANB, CANB, NSW); Canberra Botanic Garden, 10 Mar 1967, *Carroll s.n.* (CANB); Snowy River, Jindabyne, Mar 1919, *Carruthers s.n.* (NSW); Bathurst Regional, Wambool, 7 Mar 1950, *Constable s.n.* (NSW); Uralla, Winburndale, Glanmire, 16 Mar 1955, *Constable s.n.* (NSW); Wambool, 7 Mar 1950, *Constable 11099* (K); Penrith, c. 0.5 mile N of Penrith Railway Station, 5 Feb 1969, *Coveny 825* (AD, NSW); Snowy River, Snowy River, 43 km SSW of Jindabyne in Kosciusko National Park, 21 Mar 1974, *Coveny 5425* (AD, MEL, NSW); Palerang, Talpa c. 6.5 km S of Queanbeyan, 28 Mar 2001, *Crawford 6196* (CANB, NSW); Uralla, Chiswick, nr Armidale, 17 Feb 1949, *Crossley s.n.* (NE); Cobar, Tara, Nymagee, 5 Mar 1976, *Cunningham s.n.* (CANB); Coolamon, Matong, May 1950, *Driver s.n.* (NSW); Walteela, Wagga Wagga, Mar 1919, *Fletcher s.n.* (NSW); Yass Valley, Canberra, along Molonglo River, 9 Dec 1949, *Gauba s.n.* (AD, CANB, NSW); Capital Circle, E of new Parlament House, Canberra, 20 Feb 1988, *George 16913* (CANB); Mid-Western Regional, Goolma, 35 km NE of Wellington, 4 Feb 1972, *Gleeson s.n.* (NSW); Cabonne, Molong distr., 7 Jan 1945, *Glenfield Vet Research Station SN 45 -13* (NSW); Uralla, Chiswick, 17 Feb 1949, *Grassely 426* (CANB); 20 mls S of Tenterfield, 18 Jan 1956, *Gray s.n.* (NSW); Tenterfield, 20 mls S of Tenterfield, 18 Jan 1956, *Gray 3712* (CANB); Snowy River, nr Old Jindabyne, Monaro Region, 10 Feb 1966, *Gray 5901* (CANB); C.S.I.R.O. plots, Canberra, 11 Feb 1931, *Hartley s.n.* (CANB); Mid-Western Regional, Junction Honeysuckle and Murrumbo Creeks, 11 Apr 1953, *Johnson s.n.* (NSW); Junction of Honeysuckle and Murrumbo Creeks, ca. S of Merriwa, 11 Apr 1953, *Johnson 23530* (K); Junee, Wantabadgery, E Wagga Wagga, Jan 1923, *Le Souef s.n.* (NSW); Murray, nr Mathoura, 18 Apr 1969, *Leigh S600* (CANB); Gundaroo Creek, 4.7 km N of Gundaroo on rd to

Gunning, 23 Mar 1991, *Lepschi 542* (AD, CANB, K); Jerrabomberra wetlands, Fyshwick, 7 Apr 1991, *Lepschi 555* (CANB, HO); Dubbo, Ballimore, 2 Feb 1982, *Lockrey s.n.* (NSW); Goulburn Mulwaree, Lake Bathurst, nr Tarago, 5 Mar 1969, *Martensz 76* (AD, CANB, NSW); Uralla, 12 mls W of Armidale along Bundarra Rd, Mar 1972, *McAlister s.n.* (NE); Uralla, 19 km W of Armidale, on Bundarra rd, Mar 1972, *McAlister s.n.* (NE); Uralla, Antrim, Arding, 11 km WSW of Armidale, Feb 1976, *McAlister s.n.* (NE); 19 km W of Armidale, Mar 1972, *McAlister s.n.* (NSW); Greater Hume Shire, Culcairn, railway enclosure, 18 Feb 1949, *McBarron 3094* (NSW); Greater Hume Shire, Walbundrie, 25 Feb 1949, *McBarron 3118* (NSW); Strathfield, Saleyards, Flemington, 28 Dec 1968, *McBarron 16154* (NSW); Mid-Western Regional, Bylong district, 11 Oct 1953, *McKee 654* (MEL); C.S.I.R.O. grounds, Black Mountain, Canberra, Mar 1939, *Melvaine s.n.* (CANB); C.S.I.R.O. plots, Canberra, 20 Jan 1941, *Millett s.n.* (CANB); Lachlan, 6 mls N of Condobolin, 15 Mar 1975, *Milthorpe s.n.* (CANB); Lachlan, Barthurst Street, Condobolin, 14 Dec 1976, *Milthorpe s.n.* (CANB); Lachlan, Bathurst St, Condobolin 8331, 14 Dec 1976, *Milthorpe & Cunningham 5072* (NSW); nr Fairbairn, 9 Mar 1955, *Moore 3055* (CANB); Warrumbungle Shire, Coonabarabran, 21 Mar 1938, *Nicholson s.n.* (NSW); Blayney, Belubula River, 12 mls from Canowindra, Dec 1922, *O'Malley s.n.* (NSW); Cooma-Monaro, Colinton, nr Cooma, Feb 1917, *Peden 3* (NSW); Snowy River, W side of Myalla rd, 3 mls from Cooma, 1 Apr 1963, *Phillips s.n.* (CANB); Turner, Canberra, 25 Feb 1957, *Pullen 43* (CANB); C.S.I.R.O. grounds, east slope of Black Mountain, Canberra, 12 Apr 1961, *Pullen 2600* (CANB); National Botanic Gardens, Canberra, Jan 1981, *Purdie 2001* (CANB); Jerrabomberra wetlands, 26 Apr 2008, *Purdie 6711* (CANB); Cooma-Monaro, Scottsdale Bush Heritage property, N of Bredbo, 15 Jan 2012, *Purdie 8386* (CANB); Canberra Nature Park, Black Mountain, along Black Mountain Drive on south side, 10 Jan 2014, *Purdie 9350* (CANB); Cave Creek, ca. 2 mls above junction with Goodradigbee River, 20 mls NNE of Kiandra, 24 Mar 1967, *Rodd 455* (AD, K, NSW); Armidale Dumaresq, 13 Apr 1950, *Roe 695* (CANB, NE); Carrathool, Tabbita, 20 Mar 1967, *Sainty 329* (NSW); Temora, 5 Feb 1953, *Shelley s.n.* (NSW); Barton (suburban Canberra): Themeda Wall at the Churches Centre, 6 Feb 2005, *Slee 4662* (CANB); Snowy River, Jindabyne, 26 Nov 1972, *Smith 15825* (CANB); Narromine, Mar 1947, *Stening s.n.* (NSW); Cooma-Monaro, Cooma, Feb 1916, *Stock Insp Bloomfield s.n.* (NSW); Cowra, Dec 1920, *Stoul s.n.* (NSW); 20 km W of Glen Innes, 5 Mar 1984, *Strachan s.n.* (NSW); Bombala, ca. 24 km SE of Cooma, turn off Monaro Highway just S of Rock Flat, 16 Feb 1984, *Taylor & James 267* (NSW); Murrumbidgee River, 2 km below Kambah Pool, 3 Feb 1980, *Telford 7352* (CANB); Cowra and Richmond, Jan 1976, *Toth 3* (NSW); 30 km W of Wellington, Feb 1988, *Walker s.n.* (NSW); Berrigan, Boomanoomana, 17 Mar 1973, *Wallace s.n.* (NSW); Canberra, Jan 1922, *Weston 67* (NSW); Armidale Dumaresq, Botany Gardens, 10 Apr 1990, *White s.n.* (NE); Inverell, Willows, Bonshaw Rd, c. 43 km WNW Glen Innes, 12 Feb 1992, *White s.n.* (NE); Armidale Dumaresq, Armidale, 10 Jan 1973, *Williams s.n.* (NE); Cabonne, Cudal, Jan 1938, *Without Collector s.n.* (NSW); Forbes, Back Yamma S[tate] F[orest], 21 Apr 1963, *Without Collector s.n.* (AD); Junee, 15 Mar 1938, *Without Collector s.n.* (NSW); Mid-Western Regional, Gulgong, 18 Dec 1946, *Without Collector s.n.* (NSW); Wagga Wagga, Wagga distr., 24 Jan 1945, *Without Collector SN45/151* (NSW); Murrumbidgee River nr Yass, *Yass PP s.n.* (NSW).
Queensland: Southern Downs, 24 Feb 1954, *Alison s.n.* (BRI); Southern Downs, 23 Jan 1974, *Booth s.n.* (BRI); Southern Downs, 20 Feb 1974, *Swann s.n.* (BRI); Southern Downs, 22 Dec 1958, *Taylor s.n.* (BRI). **South Australia**: Region 9, Murray, Environs of Murray Bridge, 28 May 1972, *Alcock 3939* (AD, CORD); Port Adelaide Enfield, Pooraka, Gepps Cross, 22 Mar 1979, *Alcock 6808* (AD); Tea Tree Gully, Fairview Park School, 12 Apr 1981, *Bates 953* (AD); Adelaide Hills, Mylor Parklands, 5 Dec 1998, *Bates 51012* (AD); Southern Mallee, 5 km N of Parrakie, 20 Mar 2005, *Bates 64001* (AD); Tatiara, SW of Bordertown, 1968, *Catt s.n.* (AD); The Coorong, Tintinara, 10 Mar 1946, *Filsell s.n.* (AD); Mid Murray, Mannum (Ponde), 6 Jan 1986, *Frahn s.n.* (AD); Culburra, ca. 80 km NW of Bordertown, 12 Feb 1963, *Fry s.n.* (E); The Coorong, Culburra, 12 Feb 1963, *Fry s.n.* (AD); Murray Bridge, Tepko, 13 Dec 2010, *Heap SOLTR 1 &2* (AD); The Coorong, Johnsfield, Tintinara, 26 Mar 1959, *Johns & Johns s.n.* (AD); Keith, 5 Apr 1965, *Kain s.n.* (AD, K); Alexandrina, Harvey Rd, 27 Dec 1999, *Murfet & Taplin 3676* (AD); Tatiara, Mundulla West, 20 May 1981, *Ryan & Murrie s.n.* (AD); Murray Bridge, 11 Apr 1949, *Scott s.n.* (AD); Murray Bridge, Sect[ion] 332, H[undre]d of Monarto, 1 Mar 1978, *Spooner 5777* (AD); Murray Bridge, Riverglades, NE of Murray

Bridge, 23 Feb 1993, *Spooner 14125* (AD); Mount Barker, 1 ml E of Kanmantoo, 11 Mar 1954, *Symon s.n.* (AD); 5 mls on Coorong side of Tintinara, 20 Apr 1967, *Symon s.n.* (NSW); The Coorong, 5 m[iles] on Coorong side of Tintinara, 20 Apr 1967, *Symon 4742* (AD, CANB); NSW Finley, Railway stockyards, 30 Jan 1975, *Symon 9807* (AD, MO, NSW); Port Augusta, 1.4 km direct ENE of Miranda, Winninowie Conservation Park, 16 Oct 1996, *Taylor & Oppermann BS87-182* (AD); Murray Bridge, Northern fenceline of Monarto Conservation Park, 6 Feb 1998, *Taylor 291a* (AD, MEL); Victor Harbor, on Dennis Rd, 28 Jan 2013, *Taylor 1530* (AD); 5m on Coorong side of Tintinara, 20 Apr 1967, *Whissen 4742* (K); Kangaroo Island, Hog Bay Rd, Mitchell Drive intersection, 18 Feb 2010, *Wiadrowski 1* (AD); Mid Murray, Bowhill, 18 Dec 1951, *Williams 379* (AD); Meningie, 30 Mar 1972, *Williams 4112* (AD); The Coorong, Hundred of Richards, County of Cardwell, Apr 1949, *Without Collector s.n.* (AD); The Coorong, Tintinara Agric[ultural] Bureau, 9 Apr 1942, *Without Collector 1* (AD). **Tasmania**: Pitt Water, Seven Mile Beach Protected Area, 31 Mar 2006, *Baker 1707* (CANB, HO, MEL); Clarence, Seven Mile Beach, 3 Apr 2000, *Buchanan 15695* (AD, HO); Service Depot, Five Mile Beach, 10 Mar 2006, *Crane s.n.* (HO); Clarence, 533 Pass Rd, Mornington, R.S.P.C.A, 11 Apr 2011, *Moore s.n.* (HO); Clarence, Seven Mile Beach, sand quarry site adjacent to Centre Rd, 9 Dec 2008, *Stewart s.n.* (CANB, HO, MEL); Pitt Water, Pittwater Rd, Ichigo Australia, 8 Jan 2004, *Swan s.n.* (HO); Clarence, Tasman Highway, Tunnel Hill section, 9 Jun 2010, *Wapstra 1115* (HO). **Victoria**: Mildura, 20km directly W of Carwarp, 12 Jan 2005, *Allen 101* (MEL); East Gippsland, Bonang-Wulgulmerang Rd, 3.7mls W of Tubbut, ca. 15 mls direct NW of Bonang, 5 Jan 1970, *Beauglehole 33117* (MEL); East Gippsland, Suggan Buggan River, 6 Jan 1970, *Beauglehole 33186* (MEL); East Gippsland, nr the Snowy River Road, at the junction of Sandy Creek and Snowy River, 6 Jan 1970, *Beauglehole 33238* (MEL); East Gippsland, Tubbut-Bonang Rd, between Dellicknora Junction and Taylors Creek, 4 Jan 1971, *Beauglehole 35771* (MEL); East Gippsland, Cowombat Plain, between Cobberas and NSW border, 26 Jan 1971, *Beauglehole 36566* (MEL); East Gippsland, Snowy River National Park, N end of Deddick Trail, 15 km W of Tubbut Post Office, 21 Jan 1980, *Beauglehole 67535* (MEL); East Gippsland, S of Monument Ridge, NE of Amboyne Settlement, 13 km NW of Tubbut Post Office, 22 Jan 1980, *Beauglehole 67651 A* (MEL); East Gippsland, Amboyne Creek area, 11 km NW of Tubbut Post Office, 22 Jan 1980, *Beauglehole 67673* (MEL); East Gippsland, Tingaringy National Park, W of Monument Ridge, 15 km NW of Tubbut Post Office, 30 Jan 1980, *Beauglehole 68030* (MEL); East Gippsland, Snowy River, opposite junction with Gattamurh Creek, Tallangatta Sheet, 22 Jan 1981, *Cheal s.n.* (MEL); Swan Hill, Ca 500m W of Gowanford, 27 Apr 1996, *Eichler 43* (MEL); East Gippsland, Suggan Buggan River - Snowy River rd crossing, 14 km ESE of Mount Wombargo, 21 Apr 1980, *Forbes 341 A* (MEL); East Gippsland, E side of McKillop's Crossing of Snowy River, 6 k[m] NW of Mt Deddick, 17 Apr 1981, *Forbes & van Rees 909* (AD, MEL); Banyule, nr Montpelier Billabong, 7 Mar 2011, *Lynch s.n.* (CANB, HO, MEL); Greater Shepparton, Dookie Agricultural College, Feb 1955, *Madden s.n.* (HO); Mornington Peninsula, Red Hill, 1 Apr 2008, *Mitchell s.n.* (CANB, MEL); East Gippsland, Doctors Flat, 24 Feb 1963, *Muir 2789* (MEL); Black Mountains, 83 mls E of Bairnsdale, Feb 1922, *Rogers s.n.* (MEL); Southern Grampians, Branxholme, 8 May 1964, *Smith 64/118* (AD, MEL); Greater Bendigo, Bendigo, 4 Feb 1941, *Without Collector s.n.* (MEL). **Western Australia**: Katanning, *Annice s.n.* (PERTH); Quairading, South Quairading, 31 Mar 1989, *Broun s.n.* (PERTH); Wickepin, 17 Jan 1949, *Eccles s.n.* (PERTH); Pingelly, 30 Jan 1951, *Ford s.n.* (PERTH); Pingelly, 30 Jan 1951, *Ford s.n.* (PERTH); Beverley, 25 km E of Brookton, *Hambly s.n.* (PERTH); Steerdale, John Forrest Rd, Hopetoun, 3 Dec 1997, *Hill s.n.* (PERTH); Katanning, Badgebup, 5 Feb 1946, *Kemble s.n.* (PERTH); Wickepin area, 29 Mar 1978, *Leeson s.n.* (PERTH); Denmark, 6 km W along the old railway line, Kojonup, 5 Sep 2008, *Lewis 674* (PERTH); Dumbleyung, 3 Feb 2006, *Maloney s.n.* (P, PERTH); Wickepin, Property of J. Dare, nr Harrismith, *McInerny s.n.* (PERTH); Swan, Midland, 12 mls E of Perth, Jan 1968, *Moir s.n.* (AD); Northam area, *Mulder s.n.* (PERTH); Katanning, *Patterson s.n.* (PERTH); Brookton, *Williams & Williams s.n.* (PERTH); Wickepin, *Without Collector s.n.* (PERTH).

AUSTRIA. **Wien**: Wien, Müllplatz Laaerberg, 1 Sep 1969, *Forstner* & *Melzer s.n.* (W).

BELGIUM. **Flanders**: Antwerp, Ravels, Werf van Gorp, 16 Jul 1996, *Albert 96/ 10* (BM); Antwerp, Turnhout, 22 Jul 1996, *Albert 96/ 12* (BM); De Panne, 15 Aug 1976, *Goetghebeur 2605* (H, MO); Westhoek, 15 Aug 1976, *Goetghebeur 8626* (W); Gent, 7 Aug 1983, *Goetghebeur* & *de Pauw 5252* (H, MO, UT); Oostduinkerke, 29 Jul 1950, *Stockmans s.n.* (MO); Antwerp, Linkeroever, Middenvijver, 22 Sep 2009, *Wieringa 6785* (BM, W).

FRANCE. **Hauts-de-France**: Pas-de-Calais, Camiers, au NW d'Etaples, chemin conduissant a la plage Saint Gabriel, 30 Oct 1973, *Lambinon et al. 1461* (BM, H).

GERMANY. **Nordrhein-Westfalen**: Niederrhein, Emmerich, 20 Oct 1929, *Krüger 700* (W).

MOROCCO. **Oriental**: Oued Charef, au nord de Ain-Benimathar, 21 Jun 2008, *Calvo et al. 2501* (MA).

NETHERLANDS. **Zuid Holland**: Isle of Rosenburgh, De Beer, 26 Aug 1948, *van Hattum 4073* (MO); Isle of Rozenburg, De Beer, 26 Aug 1948, *van Hattum 4073* (K); Goeree, Havenhoofd, 13 Aug 1977, *Podlech 8624* (BM, H, MO).

ROMANIA. Moldova, Distr. Covurlui, stationem viae ferreae Fulgeresti, 6 Sep 1922, *Petruscu* & *Borza 2465* (W).

SOUTH AFRICA. Cape: Morgendal, 10 Feb 1960, *Acocks 21049* (EA).

SWEDEN. **Götaland**: Skåne, Malmö, Sep 1906, *Hylmö s.n.* (BM).

TUNISIA. **Gabes**: Gabes, Djebel Dirra, Apr 1909, *Pitard s.n.* (MA).

UNITED KINGDOM. **England**: Worcestershire, Pinvin, 13 Aug 1956, *Goodman 613* (BM); Gloucestershire, Allotment, Bristol Harbour, 27 Jul 1912, *Herb. Linton s.n.* (BM); nr Hartley, Wintney Hants, 17 Aug 1949, *Hodge 4288* (K); Northumberland, Holy Island, 5 Sep 1936, *Hutton s.n.* (K); Worcestershire, Pinvin, 23 Sep 1957, *Lousley W/493* (BM); Northumberland, Holy Island, 13 Sep 1955, *Lousley s.n.* (BM); Northumberland, Holy Island, 13 Sep 1955, *Lousley s.n.* (K); Essex, Barking, 17 Oct 1953, *Lousley s.n.* (BM); Suffolk, Woodbridge, 14 Sep 1932, *Milne-Redhead* & *Airy Shaw 1834* (K); Norfolk, nr Wolferton station, 1 Aug 1949, *Petch s.n.* (K); Gloucestershire, Bristol Docks, 21 Nov 1912, *Roper s.n.* (K); Gloucestershire, Avonmouth Dock, 6 Oct 1930, *Sandwith s.n.* (K); Kent Gravesend, 25 Sep 1919, *Smith s.n.* (K); Suffolk, Heath nr MeldenHall, 23 Sep 1948, *Southwell s.n.* (BM); Norfolk, Wolferton, V-C 28, 9 Aug 1958, *Townsend s.n.* (K); Northumberland, Holy Island, 4 Aug 1933, *Wallace s.n.* (BM); Gloucestershire, Bristol, Wapping Wharf, 22 Aug 1912, *White s.n.* (BM). **Scotland**: Possil, Glasgow, 11 Sep 1918, *Grierson 955-18* (K). **Wales**: Gwynedd, Caernarfonshire, Dinas Dinlle, *Herrington s.n.* (BM); Cardiff, Cardiff, 22 Aug 1925, *Smith s.n.* (BM).

Solanum umalilaense

TANZANIA. **Iringa**: Mufindi, Igowole, 10 Mar 1989, *Kayombo* & *Kayombo 215* (MO). **Mbeya**: Maganjo village, Lwindi ward, 8 Jul 2010, *Manoko 2010-1* (DSM, NIJ, WAG); Isangati village, Iyunga ward, 8 Jul 2010, *Manoko 2010-2* (DSM, NIJ, WAG); Igala village, Holondo ward, 8 Jul 2010, *Manoko 2010-8* (DSM, NIJ, WAG); Isangati village, Iyunga Mapinduzi ward, 9 Jul 2010, *Manoko 2010-11* (DSM, NIJ, WAG); Isangati village, Iyunga Mapinduzi ward, 9 Jul 2010, *Manoko 2010-12* (DSM, NIJ, WAG); nr Umalila Forest reserve, 9 Jul 2010, *Manoko 2010-14* (DSM, NIJ, WAG); Uyole, Mbeya, 22 May 1968, *Mwambunga 6* (DSM).

Solanum villosum

AFGHANISTAN. Cabul, 1978, *Collett 111* (K); middle part of Tang-i-Charu, prov. Kabul, 17 Jun 1962, *Hedge* & *Wendelbo W 4290* (E); Iudiki to Cabul, 1879, *Johnston s.n.* (E); Weilar Lake, 2 Sep 1962, *Neubauer s.n.* (W); Kamdesh, Kumul Village, 13 Oct 1965, *Street 222* (F); Nuristan, 14 Oct 1965, *Street 249* (F); Nuristan, 17 Oct 1965, *Street 258* (F); 3 mi E of Girishk, 11 Nov 1965, *Street 306* (F); Karizimir, 3 Apr 1963, *Without Collector s.n.* (KUFS). **Badakhshan**: Badakshan, Khumbak-e-Payaen, 17 Aug 1969, *Carter 569* (K); Kalat, 14 Oct 1937, *Koelz 13217* (W). **Baglan**: Massif du Salang, Sep 1968, *Fohlen*, *S-40 a* (W); 10 mi W of Doshi, Hindu Kush, 3 Jul 1966, *Furse 8230* (K). **Balkh**: Prov. Mazur-i-Sharif, Aq Kupruk, 7 Jun 1962, *Hedge* & *Wendelbo 3931* (E); prov. Mazar-i Sharif, in faucibus fluvii Balkh supra Aq Kupruk, 7 Jun 1962, *Rechinger 16293* (K, W).

Bamyan: Bamyan, 25 Aug 1948, *Köie 2897* (W). **Farah**: 4 km NW Farah Rud, 7 May 1969, *Freitag 5203* (KUFS). **Helmand**: island opposite Lashkarge, 4 Oct 1967, *Freitag 2075* (W). **Herat**: Khochk Aoua, 1962, *Lindberg s.n.* (W). **Kabul**: Mittlere Tang-e Gharu, 1 km unterhalb Mahipar, 22 May 1970, *Anders 3705* (KUFS, W); Surobi, nr Sarobe, 18 Mar 1970, *Carter 717* (K); 3 mi up Bamian rd from main Kabul-Salang rd, 14 May 1971, *Gibbons* & *Gibbons 294* (K); Kabul, 24 Aug 1949, *Gilli 3241* (W); Kabul, 8 Aug 1951, *Gilli 3242* (W); Kabul, 24 Jun 1935, *Hay 286* (K); Kabul, 3 Oct 1935, *Hay 463* (K); Tange Gharou, 6 Aug 1959, *Lindberg 756* (W); Shakardara, 31 Aug 1939, *Motte s.n.* (KUFS); Kabul, 16 Jun 1963, *Neubauer 3153* (KUFS, W); Kabul, Karte Chahar, 6 Aug 1978, *Podlech 32273* (KUFS); in faucibus Tang-e Gharru inter Kabul et Sarobi, 17 Jun 1962, *Rechinger 16957* (W); Kabul: University-campus, 16 Apr 1963, *Without Collector s.n.* (KUFS). **Kapisa**: NW Kayli, 23 Jul 1973, *Anders 10801* (KUFS). **Khandahar**: Gorgan, Kalaleh, 15 Jul 1967, *Hashemi 12929 E* (W); deserti Registan prope Bhagat, 19 May 1967, *Rechinger 34624* (W). **Kunar**: Chaghsarai, Konar, Chaghasaray, Acker, 20 Aug 1970, *Anders 4826* (KUFS, W); Darya-e-Katigal Tal, Oberhalb Barge Matal, 24 Aug 1970, *Anders 5138* (KUFS); Chapadarrah, zwischen Wersek und Kandar, 5 Jul 1973, *Anders 10586* (KUFS). **Laghman**: Laghman, 27 May 1937, *Koelz 11622* (W). **Nangarhar**: Universitats campus (Nangahar, Jalalabad), 20 Oct 1970, *Anders 5351* (KUFS, W); N Haenge des Safeed Koh S Oghz, 16 Jun 1973, *Anders 10364* (KUFS); Mamakhel, 17 Jun 1973, *Anders 10426* (KUFS); Nuristan, Jalalabad, an der Darunta, 17 Aug 1951, *Neubauer 849* (W); Tor Khama, ad confines Pakistaniae, 17 Jun 1965, *Rechinger 31004* (W); Jalalabad, Darunta Haenge, 25 Oct 1969, *Sharifi 540* (KUFS). **Nuristan**: Mühlbachufer bei Barikot, 25 Jun 1950, *Gilli 3240* (W). **Paktiya**: Gardez, 16 Aug 1970, *Podlech 19449* (KUFS). **Qataghan**: between Dushi and Doab, 11 Jun 1962, *Hedge* & *Wendelbo W4073* (E); Salang Pass, 1 Jul 1965, *Lamond 2283* (E); in valle fluvii Qunduz inter Doshi et Doab, 11 Jun 1962, *Rechinger 16549* (K, W). **Takhar**: Ak Masjed, sudwestlich von Taluqan, 18 Jun 1965, *Podlech 11394* (E); unteres Namakab-Tal, umbegung des Ortes Taqcha Khana, prov. Takhar, 24 Jun 1965, *Podlech 11517* (E); unteres Farkhar-Tal, Fir-i-Farkhar, 5 km N von Farkhar, 22 Sep 1965, *Podlech 12710* (E).

ALBANIA. Chiafa, Valeona, Aug 1894, *Baldacci s.n.* (BM); Sjnime, 1 Sep 1918, *Schneider s.n.* (W).

ALGERIA. Ex regione inferiori Atlantis minoris, in sinu Oued el Kebir juxta Blidah, 27 Jan 1856, *Ball s.n.* (E); environs d'Oran, 7 Nov 1937, *Faure s.n.* (E); Constantina, Mansourah, 29 Sep 1868, *Paris*, *279* (BM); 1849, *Romain s.n.* (BM); Algiers, 25 Nov 1876, *Without Collector s.n.* (E). **Algiers**: Algiers, Jan 1856, *Wolfe s.n.* (K). **Biskra**: Biskra, 2 Mar 1853, *Balansa s.n.* (W); Biskra, 3 Jul 1902, *Chevallier s.n.* (K); Biskra, 30 Sep 1902, *Chevallier 73 bis* (DS). **Blida**: Blidah, 17 Jun 1875, *Cosson s.n.* (K). **Oran**: Oran à Gambetta, 27 Nov 1912, *Faure s.n.* (BM). **Tamanrasset**: Hoggar Mountains, 6 km SE of Djanet turnoff, 30 Jul 1982, *Baxter et al. 4142* (E); Haggar Mtns., 15 Feb 1931, *Meinertzhagen 152* (K); Ahaggar Mountains, Assekrem region, 6 Aug 1966, *Ogden 11* (E).

ANGOLA. E of Cuanza River, 23 Sep 1923, *Curtis 310* (GH). **Huíla**: Sá da Bandeira, estrada para Mudimba, 13 May 1962, *Henriques 53* (BM, K).

ARMENIA. sin. loc, *Calvert* & *Zohrab s.n.* (E); nr Erevan, banks of River Razdan, nr Arabkir, 9 Oct 1961, *Pojarkova 210* (K).

AUSTRALIA. **Western Australia**: 29 km W of Broomehill, 6 Apr 2006, *O'Neill s.n.* (K).

AUSTRIA. Äcker, 1880, *Roemer s.n.* (W). **Burgenland**: Nordburgenland, Parndorfer Platte, Ackerrand am E-Fuss des Heidl, c. 0.8 km N-NNW der Kirche von Nickelsdorf bei Kote 144, 21 Sep 2007, *Barta s.n.* (W); Nordburgenland, Prandorfer Platte, Holzschlag am E-Fuss des Heidl bei Nickelsdorf, c. 0.4-0.45 km SSE des Bahnübergangs bei Kote 133, 16 Sep 2004, *Barta 2004-138* (W); Parndorf, in den Gräben, 11 Sep 1931, *Wendelberger s.n.* (W). **Nieder-Österreich**: Altenburg, Aug 1890, *Aust s.n.* (W); Altenburg, Sep 1890, *Aust s.n.* (W); Marchfeld, SE von Gänserndorf, Ackerrand in der Weikendorfer Remise c. 3.75 km WSW der Kirche von Overweiden, 15 Aug 2007, *Barta s.n.* (BM, W); Donautal, nahe Stockerau, ruderaler Wegrand nahe der Bahnlinie c. 0.1-0.3 km W-WNW des Bahnhofs Spillern, 21 Sep 2008, *Barta s.n.* (BM); Marchfeld, Ortsnand v. Strassh, 13 Oct 2003, *Barta s.n.* (W); Marchfeld, Brachfeld E von Ganserndorf, c. 0.15-0.2 km NNW der Eisenbahn-Haltestelle Stripfing, 5 Oct 2005, *Barta 2005-34* (W); Marchfeld, Nahe Strasshof, c. 0.7-0.75 km SW der Schnellbahn-Haltestelle Helmahof, 26 Oct 2013, *Barta 2181* (W); Marchfeld, nahe Strasshof, im Althofer Wlad 2.4 km NE der Schnell-bahn Haltestelle Helmahof, 18 Oct 2011, *Barta 53* (W); Wiener Becken, im Ortsgebiet von Himberg 0.3 km NE der Kirche, 11 Sep 2011, *Barta 175* (W);

Pitten, Sep 1990, *Buchner s.n.* (W); Hoheneich, 19 Aug 1905, *Hübl s.n.* (BM); am Zahudamem bei Schrechat, 16 Oct 1911, *Korb s.n.* (W); Marchfeld, Marchegg, bei vem Salmhof, 2 Oct 1908, *Korb s.n.* (W); Illmitz, Burgenland, Nieder Osterreich, rive N du Neusiedler-See, 25 Oct 1975, *Lambinon 907* (BM); Marterkreuz, zwischen Augern und Protes, *Matz s.n.* (W); Hainburg, unter der Konigswart a.d. Strobe von Wolfshal nach Berg, 26 Aug 1959, *Neumann s.n.* (W); Siebensprunn, 7 Sep 1920, *Schneider s.n.* (W); Augern, 15 Sep 1917, *Schneider s.n.* (W); Wüste Platze beim Salmhof nächst Marchegg, 2 Oct 1908, *Vetter s.n.* (W); Marchfeld, c. 2 km NW of Marchegg, on the rd to Baumgarten, 29 Jul 2011, *Walter* & *Hille 8946* (W); Leithagebirge. Sommerein, 11 Oct 1878, *Wiesbaur s.n.* (BM); Kalenderberg, Modling, *Without Collector s.n.* (W). **Salzburg**: Salzburg, Salzachtal, and der Autobahn-Raststatte Golling auf Odland, 28 Aug 2003, *Melzer s.n.* (W). **Steiermark**: Fussee, Mistablagerungsplatz, 26 Aug 1950, *Rechinger s.n.* (W). **Wien**: Vienna, Winterhafen, 2 Sep 1952, *Alston 11 212* (BM); Wien 1 und 3, Bezirk ruderale Boschung und Wegrand am Weinfluss im Stadtpark, 20 Oct 2009, *Barta s.n.* (BM, W); Wien, Bezirk, Wegrand un Sandhaufen zwischen Donaukanal und Winterhafen knapp W der Freudenauer Hafenbrücke, 1 Nov 2009, *Barta s.n.* (BM, W); Wien, Bezirk, im Schönbrunner Park am rand einer Hecke W von Schloss, 15 Nov 2010, *Barta s.n.* (BM, W); Wien, Wein 1. Bezirk, zwischen Ziersträuchern am S-Rand des Rathausparkes, 4 Dec 2009, *Barta s.n.* (BM, W); Wien, Wein 2. Bezirk, Wegrand beim Flaktrum im westlichen Teil des Augartens, 28 Oct 2009, *Barta s.n.* (BM, W); Wien, Bezirk, ruderale Stellen zwischen Ziersträuchern im Park am Friedrich Schmidt-Platz nahe dem Rathaus, 30 Nov 2009, *Barta s.n.* (BM, W); Wien, Wein 13 Bezirk, Im NE-Teil des Schönbrunner Parks, 9 Dec 2013, *Barta s.n.* (BM); Wien 10 Bezirk, ruderale Stelle am rand einer Baustelle im westlichsten Teil der Landgutgasse nahe der Eisenbahnbrücke, 14 Nov 2010, *Barta s.n.* (BM); Wien, ruderalen Wegrand, Böschung knapp W d. Freudenauer Kafenbrücke, 5 Dec 2009, *Barta s.n.* (W); Wien 2 Bexirk, am aufgelassenen Frachtenbahnhof Wien-Nord nördlich der Schnellbahn-Haltestelle Praterstern, 4 Oct 2011, *Barta 121* (W); 2 Bezirk, am aufgelassenen Frachtenbahnhof Wien-Nord, ungefahr knapp W der Kreuzung Leystrasse/Schweidlgasse, 25 Nov 2012, *Barta 1151* (W); 3 Bezirk, knapp N-NNW vom Wildgansplatz SSW-SW der Schnellbahn-Haltestelle St. Marx, 11 Nov 2012, *Barta 1153* (W); 13 Bezirk, Im NE-Teil des Schönbrunner Parks, 9 Dec 2013, *Barta 2205* (W); 13 Bezirk, Im NE-Teil des Schönbrunner Parks, 7 Oct 2014, *Barta 3155* (W); prope Leesdorf (Baden?), 26 Jul 1880, *Braun s.n.* (W); Stadlau Ableerplatze neben Boraxwerk, 11 Oct 1966, *Forstner s.n.* (W); 11 Bezirk, Simmering, Leberweg, 26 Aug 1986, *Loibl 1954* (W); Odenburgstrasse, Wien XIX, 8 Oct 1996, *Mrkvicka 1964* (W); Wien XX, Leystrasse 43 im Innenhof, 13 Sep 2007, *Mrkvicka* & *Drozdowski 4638* (W); Floridsdorf, 12 Sep 1979, *Pull s.n.* (W); Vienna, erster Bezirk. auf dem Baugelände swischen Bellariastraße und Naturhistorischem Museum, 2 Oct 1992, *Wallnöfer 4213* (BM, W); Schotterbank an der Alten Donau, 30 Sep 1934, *Wendelberger s.n.* (W).

AZERBAIJAN. sin. loc, 28 Mar 1865, *Gruner s.n.* (BM). **Aran**: Steppa Mil, inter Beljuk-Chenly et laoum Stary-Su, Gu Baku, Distr. Saljany, 15 Oct 1926, *Grossheim* & *Yaroshenko I 27* (LE); Elisabethpol, 1897, *Makowsky s.n.* (W). **Lankaran**: Nr village Tangovan, 26 Jun 1931, *Shipchinskii 286* (LE).

BAHRAIN. Janoosan Avenue, W side, Mar 1985, *Cornes 263* (E).

BANGLADESH. Barisal: nr Barisal, 22 Jan 1851, *Hooker* & *Thomson 639* (K).

BELGIUM. **Flanders**: Antwerp, Env. d'Anvers, Jul 1865, *van Heurck s.n.* (E); Antwerp, Malines, Jul 1958, *Lousley s.n.* (K). **Wallonia**: Liege, quai de la Ribuée entre le No. 4 de cette rue et le pont des Arches, 7 Oct 1986, *Rousselle s.n.* (BM).

BHUTAN. **Thimpu Distr.**: Motithang, 11 Aug 1977, *Bedi 667* (K).

BOSNIA AND HERZEGOVINA. **Herzegovina**: Mostar, Jasenica, 5 Nov 1911, *Schneider s.n.* (W); Mostar, 29 Oct 1911, *Schneider s.n.* (W); Mostar, 30 Oct 1911, *Schneider s.n.* (W); Mostar, 17 Dec 1911, *Schneider s.n.* (W).

BOTSWANA. **South-East**: Gaberone, Content Farm, Water storage tower, 28 Nov 1972, *Kelaole A81* (MO).

BULGARIA. Sophia, 8 Nov 1921, *Grigorieff s.n.* (K).

BURUNDI. **Bujumbura**: Bujumbura ville, jachère, 10 Mar 1968, *Lewalle 2978* (MO). **Bujumbura Rural**: Magara, prov. Bujumbura, 20 Mar 1981, *Reekmans 9831* (EA, K). **Buriri**: Rumonge, Prov. Buriri, 2 Apr 1981, *Reekmans 9922* (EA, K). **Gitega**: Bubanza, 17 Mar 1971, *Lewalle 5327* (EA, MO); **CHAD**. Tibesti-Tarso, Toussidé, 25 Aug 1957, *Grove* & *Johnson 5* (K);

Tibesti-Toussidé, 26 Aug 1957, *Grove & Johnson 31A* (K); Tibesti, mountains of E of Bardai, 30 Mar 1966, *Hinchinsbrooke 69* (K); Faya Largeau, pépinières Agriculture, 22 Dec 1964, *Leonard 3627* (BM, K); Tibesti [Mountains], trou au Natron, 8 Aug 1961, *McDowell s.n.* (E). **Lac**: entre Djimtile et le lac Tchad, branche nord-est du delta, 28 Jan 1968, *Leonard 4408* (K).

CHINA. 1834, *Wilkinson s.n.* (BM); Aug 1763, *Without Collector s.n.* (BM). **Fujian**: Amoy, 19 May 1927, *Chung 5621* (A). **Guangdong**: Ruyuan Xian, 20 Nov 1957, *Wang 44166* (MO). **Sichuan**: Derong Xian, upstream from city of Derong on rd to Xiangcheng, 21 Jul 2004, *Boufford et al. 31001* (A); West-Setschwan, Min-tal v. Maodschou bis unter Wöntschwan, Aug 1914, *Weigold s.n.* (W). **Xinjiang Uyghu**: Kashgar, 12 Jun 1928, *Deutsche Zentralasien Expedition s.n.* (W). **Yunnan**: Dongchuan, plaines de Tong-tchouan, Jul 1914, *Maire 531* (E); Ping-pien Hsien, 17 May 1934, *Tsai 55155* (GH).

CROATIA. Brijuni, Oct 1902, *Makowsky s.n.* (W); Tribunj, Orlovkrug, 10 Jul 2014, *Prokes 5* (W); rd crossing to Dubrovnik just N of the town, 12 Sep 1972, *Uotila 20709b* (E, H); 5 km S of Senj, 14 Sep 1972, *Uotila 20780* (E, H). **Istrien**: SW Teil v. Cres, c. 4 km N Nerezine, knapp SE Osor (Ossero), neben Küste, 10 Aug 1997, *Walter 5583* (W). **Lika-Senj**: Insel Pago, 6 Aug 1831, *Hübl s.n.* (BM); Velebit, Senj, Feb 1925, *Leathes s.n.* (BM). **Slavonski Brod-Posavina**: GV Istrien, Rijecki zaljev SW Opatjia W Icici, Poljane, 10 Aug 1995, *Walter 5595* (W). **Split-Dalmatia**: Lesina, 2 Apr 1910, *Marsevic s.n.* (W); Dalmacija, Hvar gegen das Wasswerwerk (Insel Hvar), 3 Aug 1939, *Oberneder & Oberneder 6650* (BM); CYPRUS. Pentadactylas, Jan 1953, *Casey 1283* (K); Kissonerga to Maa Beach, 24 Jul 1967, *Economides ARI-980* (K); Amathus, 1978, *Holub s.n.* (K); sin. loc, 1 Dec 1901, *Lascelles & Lascelles s.n.* (K); Sintenis et Rigo Iter cyprium Hagios Aniso nikos, 7 Jun 1880, *Sintenis & Rigo 675* (E); Kantara Castle, 25 May 1972, *Spitzenberger 180* (W); Lefka orange groves, 4 Apr 1932, *Syngrassides 244* (K).

CYPRUS: Silikou, Distr. Limassol, 27 Dec 1940, *Davis 2080* (E); Stavros, 27 Oct 1932, *Foggie 21* (E). **Kerýneia**: Kyrenia-Tehingen, 5 Aug 1955, *Atherton 301* (K); Kyrenia-Loukkos nr Thermia, 17 Aug 1955, *Atherton 387* (K); Bellapais, 31 Jan 1956, *Atherton 861* (K); Kyrenia, 3 Sep 1948, *Casey 24* (K); juxta Kyrenia, 9 Jun 1939, *Lindberg s.n.* (K). **Larnaca**: in vicinatate litoris prope pagum Voroklini, 2 Dec 1995, *Vašák s.n.* (W). **Lefkosía**: Mavres Sykies, 28 Apr 1962, *Meikle 2735* (K); Nicosia, English School, 23 Nov 1958, *Oswald 22* (K). **Limassol**: Prodromos, 10 Sep 1955, *Atherton 669* (K); Kato Plátres, Plabis on Mt. Troodos, 26 Oct 1948, *Cowper s.n.* (K); Foini, Phini, *Meikle 5026* (K). **Nicosia**: Kokkinotrimithia, 17 Sep 1966, *Merton ARI-8* (K).

CZECH REPUBLIC. Prope Mariaschein Bohemia, 2 Sep 1885, *Wiesbaur s.n.* (BM). **Central Bohemia**: Prague, Oct 1937, *Milos Deyl s.n.* (E). **South Moravia**: Zluty Kopec, ad pedem meridionalem collis Zluty kopec dicti in suburbio Staré Brno, 23 Aug 1966, *Vicherek s.n.* (E). **Ústí nad Labem**: Mariaschein, 20 Sep 1885, *Wiesbaur 2239* (H, K).

DENMARK. **Sjaelland**: Kalundborg42, Jul 1969, *Hjorih-Olsen s.n.* (BM).

EGYPT. Sinai, *Aucher-Eloy 2482* (K); Southern Sinai, Wadi El-Sheikh, S of St. Katherine Village, 5 May 2001, *Boulos & Ali 19384* (K); Entre Ramleh et le canal Mahmondieh, 15 Feb 1871, *Du Parquet 253* (BM); Sinai, St. Katherina, Wadi El-Arbaein, 25 Mar 2004, *Eberl & Kaiser s.n.* (W); Mt. Sinai, St. Catherine's Monastery, 31 Mar 1987, *Hepper 8742* (K, MO); below Derr, 19 Feb 1951, *Marsh s.n.* (MO); Nile Delta, Rosetta, El-Geddia village, 20 May 2003, *Mashaly & Boulos 20189* (K); Feiran Oasis, Sinai, Mar 1928, *Meinertzhagen s.n.* (BM); Feiran Oasis, Sinai, 1928, *Meinertzhagen s.n.* (BM); Sin. loc., *de Montbret s.n.* (W); Sin. loc., *Oudney s.n.* (BM); Catta, *Sieber s.n.* (E); nr Moheb wa Salaya, 30 Jul 1922, *Simpson 1429* (K); Gebel Shellah, 1925, *Simpson & Murray 3787 ii* (K); Tummuh, 7 Mar 1927, *Simpson 4494* (K); Talbiya to Giza, 29 Nov 1927, *Simpson 5456* (K); N of Thebes, 28 Dec 1864, *Without Collector s.n.* (BM); Hor Tamanib, Red Sea, *Without Collector s.n.* (K); **Al Qahirah**: Aus der Umgegend von Cairo, 25 Sep 1864, *Schweinfurth 1402* (BM). **Aswan**: Abu Si.bil, nv. mer, 14 Mar 1963, *Abdallah 1630* (MO); Saluga Island, Assuan, 10 Dec 1943, *Davis 6070 B* (E); Kom Ombo, 5 Mar 1925, *Simpson 3062* (K); **Cairo**: Cairo, 1836, *Kotschy 622* (W); Umgegend von Cairo, Apr 1866, *Schweinfurth 1410* (K); S of El Ma'adi, 5 Dec 1921, *Simpson 16* (K); Bashtil, 10 Mar 1927, *Simpson 4795* (K). **Faiyum**: Ibshwai Distr. Sainaru el-Qibliya, 25 Dec 1981, *El Ghani 3184* (E); Beni Salih, Al Faiyum Distr., 1 Jun 1983, *El Ghani 6139* (E); El-Silien, Sinuris Distr., 11 Nov 1982, *El Ghani 4552* (K). **Gharbia**: Ag Lur el Kubra nr Qahab, 23 Jun 1922, *Simpson 1390* (K). **Giza**: Saqqara, Nile nr Sakhara, 13 Jan 1887, *Kirk s.n.* (E); Al Barajil, nr El Baragil, 16 Dec 1921, *Simpson 112* (K). **Luxor**: Luxor, 28 Feb 1925, *Simpson 3021* (K). **New Valley**: Rashida, Dakhla

Oasis, 14 Apr 1928, *Simpson 6077* (K). **Red Sea**: Wadi Ehmit, Jebel Elba, 30 Jan 1933, *Shabetai s.n.* (K); Bir Sartut, Dec 1923, *Simpson* & *Murray 2995* (K); Kasam Elba, 1925, *Simpson* & *Murray 3787 i* (K); Wadi Shendib, 1925, *Simpson* & *Murray 3852* (K); foot of Jebel Elba, 19 Mar 1928, *Simpson* & *Khattab 6334* (K). **South Sinai**: Feiran Oasis, Bedouin farm, 29 Nov 1999, *Boulos* & *Ali 19200* (K); Er Mym, *Holland s.n.* (K); Mt. Sinai to the sea, Jun 1868, *Lord s.n.* (K); E'nib, Sinai, W Nusb, *Drake 44* (K); Annebe, ad montem Sinai, 8 Aug 1835, *Schimper 255* (E, W); top of Wadi Mey'ar, Sinai, 15 May 1937, *Shabetai s.n.* (K); Wadi Kyd, *Without Collector s.n.* (K).

EGYPT/SUDAN. **Hala'ib Triangle**: Red Sea, Ssoturba-Gebirges an der Nubischen Küste, Jebel Schellal, 6 Mar 1865, *Schweinfurth 1406* (BM, K, P).

ERITREA. Golo, 5 Dec 1909, *Dainelli* & *Marinelli 90* (FT); Oddora, 16 Dec 1909, *Dainelli* & *Marinelli 94* (FT); Mahio, 14 Dec 1905, *Dainelli* & *Marinelli 98* (FT); Sella Mogasa - Mt. Ira, 5 Feb 1893, *Terracciano* & *Pappi 807* (FT). **Anseba**: Torrente Metteb, 29 Mar 1909, *Fiori 1599* (FT); Valle Catalaben, 19 Jan 1893, *Terracciano* & *Pappi 1600* (FT); Rora Ualicaue, 8 Jan 1893, *Terracciano* & *Pappi 1716* (FT); Rora Ualicaue, 8 Aug 1893, *Terracciano* & *Pappi 2105* (FT); Aba Maitain - Dada, 8 Jan 1893, *Terracciano* & *Pappi 2188* (FT). **Debub**: Adi Ugri, 13 Aug 1909, *Bellini 464* (FT); Embatakalla, 18 Apr 1932, *de Benedictis 389* (FT); Embatakalla, betweeen Asmara and Mitswa (EE), 5 Feb 1985, *Edwards* & *Tewolde 3674* (K); Saganeiti, Vallée de Degerra, 26 Mar 1892, *Schweinfurth* & *Riva 1259* (FT, K). **Gash Barka**: Get Arba, 7 Jan 1893, *Terracciano* & *Pappi 2109* (FT); Get Arba, 7 Jan 1893, *Terracciano* & *Pappi 2116* (FT). **Maekel**: Asmara, Keren rd, 2 Sep 1949, *Desert Locust Survey 49* (EA); Arbaroba, 20 Jan 1909, *Fiori 1597* (FT); Medrizien, 19 Jan 1909, *Fiori 1598* (FT); Medrizien, 19 Jan 1909, *Fiori 1958* (FT); Eritrea, Dinternidi Asmara, Jan 1902, *Pappi 4387* (W); Asmara, 27 Apr 1988, *Ryding 1128* (K); Asmara, 30 Oct 1988, *Ryding 1536* (K). **Semienawi Keyih Bahri**: Haha, 27 Mar 1893, *Pappi 2901* (FT); Masciabo, sorgente; 20 Mar 1893, *Pappi 3188* (FT); Majo – Illalila, 28 Mar 1893, *Pappi 3772* (FT); Nacfa – Maio, 19 May 1892, *Terracciano* & *Pappi 984 [2207]* (FT).

ETHIOPIA. Dessie, Wollo Prov., 10 Aug 1946, *Hall 29* (BM); Haik, 18 Aug 1946, *Hall 84* (BM); in Cullis prope Berber 1837, *Kotschy 313* (W); sin. loc, 1837, *Kotschy 446* (K, W); Abyssinia, *Pearce s.n.* (BM); Abyssinia prope Dscheladscheranne, *Schimper 2043* (BM). **Addis Ababa**: Addis Ababa, 15 Apr 1951, *Curle 27* (BM); Addis Ababa, 26 Apr 1909, *Negri 136* (FT); Entoto, 14 May 1909, *Negri 307* (FT); Addis Ababa, 27 Oct 1937, *Piovano 512* (FT). **Amhara**: Woldia, American Lutheran Mission, 6 Sep 1962, *Albers 62349* (GH, MO); Semien Gondar, Gondar, 30 Jul 1909, *Chiovenda 1204* (FT); Semien Gondar, Gorgora, N Lake Tana, 4 Sep 1953, *Esplen 103* (K); Kombolcha, 6 Oct 1969, *Parker 558* (K); Semien Shewa, Let Marefia, 8 Sep 1886, *Ragazzi s.n.* (FT); Wello Prov., Azewagedel Mountain, 2 km E of Desse, 18 May 1969, *Sutherland 281* (MO); Semien Gondar, Semien, Abenna at Ataba, Jan 1862, *Steudner 734* (K). **Dire Dawa**: Rd to Dira Dawa, 37 km from gate of College of Agriculture at Alemaya, Harerge Region, 23 Jun 1975, *Jansen 1644* (MO); Melka Jabdu, c. 7 km S of Dire Dawa, 10 Sep 1967, *Tadesse Ebba 627* (K). **Oromia**: Sajo, Jun 1938, *Benedetto 49* (FT); Misraq Hararghe, Lake Alemaya, 15 km NW of Harrar, college area, 25 Jul 1961, *Burger 374* (K); Misraq Hararghe, College area, NE of Lake Alemaya (Haramaia), c. 15 km NW of Harar, 25 Jul 1961, *Burger 378* (FT, K); Mirab Arsi, Neghelle, 1939, *Corradi s.n.* (FT); Turn from Yavello-Mega to Arero 20 km from the main rd Yavello-Mega on the rd to Arero, 29 Nov 1997, *Friis et al. 8350* (K); West Arsi, Shashamane, Shashamene to Sole, nr Shashamene, 13 Feb 1954, *Mooney 5743* (K); Debub Mirab Shewa, nr Woliso, 5 Oct 1954, *Mooney 5858* (K); Oletta [Holet], 26 May 1909, *Negri 563* (FT); Guder, 145 km W of Addis Ababa, 23 Feb 1958, *Piffard 22* (K); Kakkisi - Addis Ababa, Jun 1957, *Rankin s.n.* (E); Alemaya College, main campus, 6 Aug 1971, *Seegeler 2038* (MA, MO); Akaki, piana di, 5 Jun 1937, *Senni 877* (FT); Mirab Arsi, Neghelle, 21 Oct 1937, *Vatova 101* (FT); 60 km from Awash, 30 Aug 1967, *Westphal* & *Westphal-Stevels 1483* (K, MO); 161 km from Mojo, rd to Shashemene, 6 Sep 1967, *Westphal* & *Westphal-Stevels 1674* (MO); 109 km SE of Shashamene, 8 Sep 1967, *Westphal* & *Westphal-Stevels 1749* (K, MO); Nazareth to Mojo, 15 km from Nazareth, 2 Oct 1967, *Westphal* & *Westphal-Stevels 1924* (K, MO); Gergertu, rd to Harawacha, 7 km from Harawacha along rd, 2 Nov 1967, *Westphal* & *Westphal-Stevels 2548* (EA); 4 km from Shashamane, 15 Nov 1967, *Westphal* & *Westphal-Stevels 2616* (MO); 2 km from Neghelli, 23 Nov 1967, *Westphal* & *Westphal-Stevels 2740* (MO); Misraq Hararghe, Alemaya, along rd to the College of Agriculture, 4 Apr 1968, *Westphal* & *Westphal-Stevels 3963* (K, MO). **Somali**: Ourso, 15 Mar 1968, *Westphal* & *Westphal-Stevels 3496* (K, MO). **Southern Nations (SNNP)**: Omo River, 10 mi N

of Kalaam, Aug 1968, *Carr 534* (EA); Gedeo, Ghiedeb, Choke Mts., tributary of Godab, 3 Aug 1957, *Evans 57* (K); Botor Kefa region, 17 Mar 1956, *Mooney 6680* (K); Keffa, Bonga, Nov 1937, *Saccardo 35* (FT); Jimma, Institute of Agriculture, Keffa Region, 24 Aug 1972, *Seegeler 2472* (MO); Torrente Ganta, 14 Feb 1938, *Vatova 1854* (FT); rd from Soddo to Arba Mintch, 80 km from Soddo, 2 Dec 1967, *Westphal* & *Westphal-Stevels 2908* (MO). **Tigray**: Negash, Mekelle to Adigrat, 8 Oct 2001, *Friis et al. 10524* (K); Negash, Mekelle to Adigrat, 8 Oct 2001, *Friis et al. 10525* (K); Mekelle to Adigrat, c. 2 km NE of the village of Salwa, c. 5 km N of Sinkara, 8 Oct 2001, *Friis et al. 10536* (K); Agula, E of town, 15 Oct 2001, *Friis et al. 10699* (K); Amba Alaga, 9 Oct 1995, *Friis et al. 6612* (K); Debubawi, Mek'ele, 23 Sep 2001, *Friis et al. 10411* (K); Abbeum am Ssaba, Jan 1862, *Steudner 734* (K).

FRANCE. Lenormand, 1833, *Without Collector s.n.* (E). **Auvergne-Rhône-Alpes**: Puy-de-Dôme, Clermont Ferrand, Cantal. Aubière, Beaumont, Fontgiève, Oct 1878, *Heribaud s.n.* (BM); Drôme, Romans, décombres au faubourg Clerieux, Oct 1873, *Hervier-Basson s.n.* (E); Pierre-Bénite (Rhone), le long des murs au Perron, 25 Sep 1877, *Perret 1765* (K); endroits incultes aux bords des chemins et des champs dans la vallee du Rhone, a la Pape, pres de Lyon, Apr 1845, *Schultz 705* (K). **Bretagne**: C. Champs de Batz, 2 Nov 1872, *Gadeceau s.n.* (BM); Le Croisic, 13 Jul 1891, *Gadeceau s.n.* (BM); Village de Kerlay, près Batz, 21 Aug 1892, *Gadeceau s.n.* (BM); bourg de Batz, 18 Aug 1877, *Genevier s.n.* (BM); bourg de Batz, 18 Aug 1877, *Genevier s.n.* (BM). **Centre-Val de Loire**: Indre, Loches, 30 Sep 1857, *Genevier s.n.* (BM); **Corse**: Ajaccio, 18 Apr 1933, *Godman Dame Alice et al. 93* (BM). **Grand Est**: Haut-Rhin, Dept. Ht.-Rhin, Baumwollkompost in Issenheim, 9 Oct 1962, *Aellen* & *Baumgartner s.n.* (BM, W). **Île-de-France**: Paris, Passy, Jul, *Depierre s.n.* (BM); Paris, Rue St. Hilaire, en lau libraire du Museum, 2 Oct 1952, *Gillet s.n.* (BM). **Normandie**: Normandie, Oct 1871, *Without Collector 1010* (BM). **Nouvelle Aquitaine**: Gironde, Bordeaux, Leognan. pres du château La Louviere, 9 Oct 1927, *Bouchon s.n.* (BM). **Occitanie**: Pyrénées-Orientales, Salses, 15 km N of Perpignan, vineyard at W end of village, 19 Oct 1982, *Akeroyd 239* (BM); Valergues, De du Heurault, Oct 1853, *Ball s.n.* (E); Gard, Vigan, 28 Aug 1865, *Billot s.n.* (BM); Gard, Vigan, 8 Sep 1861, *Billot 25* (BM); Hérault, Montpellier, *Bentham s.n.* (E); Aude, Narbonne, *Martrin s.n.* (BM); Narbonne a Librette, *Martrin s.n.* (BM); Hérault, gorge of Hérault, NE of Le Vigen, Gard, 11 Sep 1952, *Webb s.n.* (W); Hérault, Montpellier, an der Aiguelangue in NW der Stadt, 13 Nov 1938, *Wendelberger s.n.* (W). **Pays de la Loire**: Loire-Atlantique, Nantes, Indret, 29 Oct 1893, *Gadeceau s.n.* (BM); Loire-Atlantique, Nantes, St Sebastian, 8 Dec 1895, *Gadeceau s.n.* (BM); Loire-Atlantique, pres Nantes, 6 Sep 1904, *Gadeceau s.n.* (BM); Loire-Atlantique, Indret, 11 Aug 1878, *Gadeceau s.n.* (BM); Loire-Atlantique, Indret, 29 Aug 1893, *Gadeceau s.n.* (BM); Loire-Atlantique, Indret, Aug 1893, *Gadeceau s.n.* (BM); Loire-Atlantique, Bouaye. loire Inf, 13 Oct 1870, *Gadeceau 1006* (BM); Maine-et-Loire, Angers, 1 Nov 1891, *Genevier s.n.* (BM); Sables-d'Olonne, Vendée, 3 Sep 1863, *Genevier s.n.* (BM); Loire-Atlantique, Indret, 4 Sep 1860, *Genevier s.n.* (BM); Maine-et-Loire, Angers, Oct 1850, *Legé s.n.* (BM); Maine-et-Loire, Angers, port Ayrault, 7 Oct 1890, *Legé s.n.* (BM); Maine-et-Loire, Angers, Aug 1891, *Réchin s.n.* (BM); Maine-et-Loire, Angers, Aug 1891, *Réchin s.n.* (BM). **Provence-Alpes-Côte d'Azur**: Toulon, Talus de la voie du chemin de fer en costruction de Molseheim a Mutzig (Bas Rhin), Sep 1862, *Billot s.n.* (BM); Var, Hyères, Environs d'Hyeres, 29 Sep 1880, *Heribaud s.n.* (BM); Bouches-du-Rhône, Camargue, Bouches du Rhone, Tour du Valat, Le Sambuc, 1 Sep 1967, *Kendrick* & *Moyes 64* (BM); Alpes-Maritimes, Cap D'Ail, 4 Nov 1910, *Lowe s.n.* (BM).

GEORGIA. **Tbilisi**: Tbilisi, river gorge W of city center and W of the Botanical Garden, 12 Aug 2005, *Atha* & *Reveal 5196* (W);

GERMANY. **Baden-Württemberg**: Baumwollkompost in Atzenbach bei Zell, 9 Oct 1952, *Aellen s.n.* (BM); Baumwollkompost in Atzenbach bei Zell, 9 Oct 1952, *Aellen 3* (BM); Baden, *Kummer 581 1* (BM). **Bayern**: prope Rotenburgum, *Hoppe s.n.* (BM). **Berlin**: Berlin, *Hellwig s.n.* (E); **Brandenburg**: Prignitz, Lenzen, am Marienberge, Sep 1885, *Schütz s.n.* (W). **Hessen**: Frankfurt, (Main), zwischen Gleisanlagen im Westhafen, Aug 1943, *Behr* & *Behr s.n.* (W); Frankfurt, *Herb. Auerswald s.n.* (BM). **Niedersachsen**: Asse, Braunschweig, 12 Jul 1882, *Kretzer s.n.* (W); Nieder Elbe, Lenzen, Aug 1885, *Schütz s.n.* (BM). **Nordrhein-Westfalen**: Bonn, 30 Jul 1851, *Blackie s.n.* (E). **Sachsen**: Leipzig, Leipzig, Sep 1844, *Auerswald s.n.* (BM); Leipzig, Leipzig, Sep 1846, *Auerswald s.n.* (BM); Leipzig, Röglitz bei Leipzig, *Hofmeister s.n. [42]* (W); Sachsen, Schuttplätze bei Wittenberg, Aug 1912, *Matthies s.n.* (BM). **Sachsen-Anhalt**: Sachsen, Schuttplatze bei

Wittenberg, 8 Sep 1912, *Matthies s.n.* (MO); Wittemberg, Aug 1912, *Matthies s.n.* (W); Wittemberg, 9 Oct 1904, *Matties s.n.* (W); auf Schutt bei Pahl's Ziegelei an der Berliner Chausee bei Magdeburg, Oct 1907, *Quasig s.n.* (W). **Schleswig-Holstein**: Lübeck, Travemund, *Griewank s.n.* (BM); Schenefeld, Holstein, Aug 1850, *Lauder Lindsay s.n.* (E). **Thuringia**: Schwellenburg, bei Erfurt, 1840, *Eversmannvon s.n.* (BM); Flora thuring; Wüste Orte bei Grossfurna, Sep 1887, *Herzing s.n.* (E); bei Sondershausen, Aug 1884, *Theinmann s.n.* (BM); Crimderode, 8 Sep 1886, *Vocke s.n.* (BM);

GREECE. sin. loc, *von Friedrichsthal s.n.* (W); 5 km from Ermioni towards Galatas, 9 Jul 1979, *Thomas s.n.* (BM); sin. loc, *Zuccarini s.n.* (W); Nomphia, *Zuccarini 186* (K). **Attica**: nr Kephissia, Jun 1930, *Archley 161* (K); Cephissum, Sep 1930, *Guiol 1601* (BM); Kephissia, ad radices montis Pentelici, *von Heldreich s.n.* (BM). **Central Macedonia**: Salonica, 1917, *Ramsbottom s.n.* (BM). **Crete**: Prov. Sitia, zwischen Tourloti und Sfaka, 5 Jul 1987, *Burri* & *Krendl s.n.* (W); Nomos Lasithiou, Selinaris, bas de la route de Vrachasion, 8 Apr 1980, *Lawairee 22320* (BM); Distr. Hierapetra, prope Males, 10 May 1900, *Leonis 137* (W); Kydonia, inter Skines et Nea Rumata, 24 Jul 1973, *Rechinger 45775* (W); Sitia, inter Sphaka et Turloti, 17 May 1942, *Rechinger 13017* (BM, K, W). **East Macedonia and Thrace**: Thessaloniki, Salonica, 1917, *Ramsbottom x19* (BM). **Ionian Islands**: Ithaca island, 19 May 1986, *Hepper 8696* (K, MO); Ionishce Islen, Nomos Korfu, 12 Oct 2013, *Karl s.n.* (W). **North Aegean**: Below Salakos, Is. Rhodes, 22 Oct 1981, *Davis 67986* (E); Samos, at Despoti Vrysi of Prygos, 17 Jul 1964, *Gathorne-Hardy 823* (E); Lemnos, 1916, *Prior s.n.* (BM); Lemnos, 1916, *Prior s.n.* (BM). **South Aegean**: Siuri Village, 3 Jul 1918, *Day .n.* (BM); Naxos, Amorgos Island, Langadha, 15 Apr 1940, *Davis 1514 K* (E); Anafi, Cyclades, Aug 1962, *Gill s.n.* (K); Rhodes, Filerimos bei Trianta, 17 Dec 1975, *Gilli s.n.* (W); South Macedonia, Neighbourhood of Mikra Bay, c. 6 mls of Salonica, Aug 1918, *Preston 37* (BM).

HUNGARY. Pesth (Budapest), *Without Collector s.n.* (E). **Komárom-Esztergom**: Kalocsa, ad vias pagi Komlod, 17 Aug 1876, *Menyhárth s.n.* (K). **Southern Great Plain**: Bacs-Kiskun, Kalocsa in Hungaria, ad viaj prope Kömläd, 11 Jul 1876, *Menyhárth s.n.* (E).

INDIA. 25 Aug 1880, *Aitchison 611* (DD); Ichirsa, Pauri Distr., 1 Jun 1975, *Aswal NC-55377* (BSD); Mongosong, Bengaland Borders, 18 Feb 1935, *Biswas 3049* (CAL); sin. loc, 3 Jul 1891, *Gammie s.n.* (K); Garibul, Kashmir, 3 Jul 1891, *Gammie s.n.* (CAL); Panmlully, 15 May 1848, *Hooker & Thomson s.n.* (K); Herb. Ind., *Jacquemont 783* (K); sin. loc, *Jacquemont 937* (K); Balagu, *Janaki Ammal 1523* (K); NW Himalayas, Chakrah, 28 Sep 1936, *Raizada 7426* (DD); Kashmir, Jul 1956, *Rao 626* (CAL); Kashmir, Jul 1956, *Rao 848* (CAL); Jakko, Simla, 19 Sep 1915, *Rich 26* (K); Penins. Ind. Or., 1838, *Russell s.n.* (K); sin. loc, *Without Collector s.n.* (K); **Andhra Pradesh**: Chittoor, Tirumala Hills, 19 Feb 2006, *Ranga 245* (CAL); **Bihar**: Bengal, Champaran, Mar 1904, *Haines 1979* (K); **Chhattisgarh**: Bastar, Rd side, Kondagaon, 19 Nov 1958, *Subramanyam 7199* (CAL); **Delhi**: Delhi, 8 Jun 1953, *Bajaj s.n.* (RB); Delhi, University campus, 28 Jan 1968, *Vidyamayi 1134* (UT); **Goa**: Panjim, 13 Oct 1974, *Ghosh CU-11036* (CAL); **Gujarat**: M.S. University of Baroda campus, Gujarat, 10 Nov 1964, *Sabnis 568* (K); Palanpur, 24 Dec 1972, *Singh 5397* (CAL); Ahmedabad, Chanod, 3 Jan 1978, *Singh 5618* (CAL); **Haryana**: Gurgaon, 25 Oct 1962, *Nair NC-25266* (BSD); Hisar, 10 Jan 1962, *Nair 14778* (CAL); Hisar, 25 Apr 1962, *Nair NC-19972* (BSD); Yamunanagar, Yamuna Nagar, banks of Yamuna River, 21 Apr 1986, *Sharma NC-77942* (BSD); **Himachal Pradesh**: Allahabad, Kalhgorh, 17 Dec 1964, *Arora 6073* (CAL); Manali, Manaton V[alley], 31 Jul 1971, *Bhattacharyya NC-44895* (BSD, CAL); Trilakinath, U. Chenab, 21 Aug 1971, *Bhattacharyya NC-45932* (BSD);East Rd around Jako [Jakko Hill], 16 May 1902, *Bourne 3673* (K);Dalhousie, 14 Sep 1874, *Clarke 22378* (K); Shimla, Shimla, Nov 1885, *Collett 416* (K);in Parbaté valley, Kulu [Parvati Valley, Kullu], Sep 1855, *Edgeworth 7045* (K); Shimla, Annandale, 30 May 1877, *Gamble 4478 A* (K); Lahul, Punjab, *Jaeschke 304* (K); Baddari [Baddi] nr the village, Baspa valley, 29 Sep 1971, *Janardhanan NC-46338* (BSD, CAL); Kusharang, on way from Kalpa to Raghi, 2 Oct 1971, *Janardhanan NC-46372* (BSD, CAL); 6 km ahead on Karcham, Sangla rd, Baspa valley, 26 Aug 1973, *Janardhanan NC-52836* (BSD, CAL); Kinnaur Distr. [Kilba Khas], 31 May 1972, *Janardhanan NC-77525* (BSD); Dalhousie, (labelled as Punjab), 13 Jul 1963, *Malhotra & Nair NC-27338* (BSD); Jeori, [Mandi Region], 24 May 1962, *Nair NC-21756* (BSD, CAL); Urni Khas, Unri, Kimmor, 31 May 1962, *Nair NC-22166* (BSD); Urni Khas, Urni, 31 May 1962, *Nair NC-22215* (BSD); between Panji and Jangi, Kinnr Distr., 6 Jun 1962, *Nair NC-22480* (BSD); Tissa, Malah, Chamba, 31 Jul 1964, *Nair NC-32949* (BSD, CAL); Shimla, 29 May 1831, *Ramsay s.n.* (K); Shimla, May 1831, *Ramsay s.n.* (K); Renuka Lake, 1 Jun 2000, *Srivastava NC-96030* (BSD); Mandi-Loran,

16 Sep 1985, *Vohra & Pani NC-78290* (BSD); **Jammu & Kashmir**: Upper Topa (Murree hills), Aug 1920, *Barbour s.n.* (BM);"NW Himalaya, Upper Chenab, 1880, *Ellis 557* (K); Ladakh, *Hooker & Thomson s.n.* (K); Gulmarg, 4 Nov 1977, *Rao NC-63680* (BSD); Batote, Ghenab valley, 10 Sep 1958, *Rao NC-7346* (BSD); Kishtwar, 28 May 1986, *Uniyal NC-80282* (BSD); Doda, Bhadrawah, 30 Aug 1962, *Wadhura 786* (CAL); Anantnag, Pahalgam to Chrudarwari, 25 Sep 1961, *Wadhwa 216* (CAL); Lukung-Chushul Rd (up 10-15 km) along Pangang Tso base, 21 Aug 1976, *Wadwha NC-60184* (BSD); **Jharkhand**: Dalma wildlife sanctuary, Behind forest 1B Mango, 20 Jun 2000, *Charrabanty 3338* (CAL); Hazaribagh, Chota Nagpur, banks of the Gonda river, 10 Mar 1954, *Kerr 2436A* (BM); Sahebganj, 11 Dec 1957, *Panigrahi 11606* (CAL); Latehar, Betla National Park, Jharkhand, 8 Nov 2004, *Singh 34010* (CAL); **Karnataka**: Canara, prope Bettigherry, Feb, *Hohenacker s.n.* (K); Hassan, Hassan Town, 26 May 1969, *Saldanha 13567* (MO); **Kerala**: Palakkad, Way to Kathalakandi, 24 Feb 1979, *Vajravelu 60641* (CAL); **Madhya Pradesh**: Nimar, top of Asirgarh, between Burhanpur and Khandwa, 25 Sep 1908, *Burkill 31067* (CAL); Bhopal, Raj Bhavan, 23 Apr 1987, *Khanna GC-39916* (CAL); Khargone, Pepal Jhopa, Khargeon, 7 Feb 1987, *Prasad 39377* (CAL); Jabalpur, forest nr Sunderson factory, Katni, 26 Sep 1959, *Sebastine 8954* (CAL); Jabalpur, South of forest Bungalow, Kunduward, 12 Mar 1962, *Sebastine 13928* (CAL); **Maharashtra**: Forest areas nr Mandvi Khurd, 6 Mar 1965, *Pataskan 104144* (CAL); Poona, Purandkar, nr Canteen, 19 Jul 1963, *Rolla 88606* (MO); Pune, Parvathi Hill to Lakshmi Park, 5 Aug 1960, *Subramarian 64511* (CAL); **Odisha**: on bank of Tel River at Tonsil, nr Kesinga, 9 Dec 1948, *Mooney 3160* (K); **Punjab**: Gurdaspur, Batala, Gurdaspur, 24 Aug 1969, *Bhattacharyya 37783* (CAL); Mukerian, Distt. Hoshlarpur, 17 Sep 1970, *Misra NC-41840* (BSD); Rampur, Hoshiarpur Distr., 7 Jul 1971, *Misra NC-44354* (BSD, CAL); Bankhandi, Hoshiarpur Distr., 12 Apr 1972, *Misra NC-46912* (BSD, CAL); Sampla, 13 Mar 1962, *Nair NC-20005* (BSD); Sima, Othe bridge area, 30 Apr 1962, *Nair NC-21618* (BSD, CAL); Daryapur, Sheran, 12 Dec 1957, *Rau NC-3543* (BSD); **Rajasthan**: Lohargal, Mar 1960, *Nair NC-2034* (BSD); Pali, nr Paras Ram Mahader temple, 15 Apr 1977, *Pandey 4186* (CAL); Alwar, Keraska, Sariska Tiger Reserve, 25 Oct 1983, *Parmar 9573* (CAL); Churu, Tal Chhapar, 19 Mar 1976, *Ray 2516* (CAL); Ganganagar, Suratgarh, govt. farm, 14 Oct 1977, *Roy 5060* (CAL); Jaipur, Amer Rd, 25 Dec 1963, *Sharma 359* (CAL); Govt. college waste fields, Ajmer, 16 Sep 1968, *Sharma 583* (DD); Jaisalmer, Bara Bagh, 12 Mar 1977, *Shetty 4107* (CAL); Jaisalmer, By the side of Amar Sagar tank, 7 Mar 1978, *Shetty 8134* (CAL); Jodhpur, Teori, 14 Oct 1976, *Singh 3264* (CAL); Jodhpur, Bilara RH, 26 Feb 1977, *Singh 3282* (CAL); Bhilwara, Amarpura village, 19 Sep 1978, *Singh 6232* (CAL); Jodhpur, Kailana afforestation area, compt. V, 4 Jan 1973, *Singh 421* (CAL); Chandrafun, Rajasthan, 19 Dec 1963, *Verma CC-1816* (CAL); sin. loc., 15 Dec 1964, *Verma 6728* (CAL); **Sikkim**: Runqfro, 25 May 1909, *Without Collector 1473* (CAL); **Tamil Nadu**: Pulneys [Palni Hills], Bear Shola [Falls], 13 Jun 1897, *Bourne 405* (K); Kodaikanal, Koraikanal Shola, Pulneys [Palni Hills], 17 Jun 1897, *Bourne 472* (K); Chennai, from Wondalur [Vandalur], Apr 1811, *Without Collector s.n.* (K); **Telangana**: Manmool, ICRISAT site, 30 km NW of Hyderabad, 22 Jun 1978, *van der Maesen 3190* (CAL, K); **Uttar Pradesh**: Banda, 16 Feb 1901, *Bell 36* (CAL); Mihjapin, 7 Feb 1961, *Bhattacharyya NC-12853* (BSD); Banda, 30 Sep 1961, *Bhattacharyya NC-17976* (BSD, CAL); Tehri, way to Ghuttu, 31 Jul 1978, *Goel NC-64200* (BSD); Tehri, Tehri City, 17 Feb 1979, *Goel NC-64620* (BSD); Moradaban [Moradabad], Mar 1845, *Hooker & Thomson 62* (K); R.H. forest [Etawah=Ishtikapuri], 25 Jan 1961, *Malhotra NC-13480* (BSD, CAL); Gola Gokarannath, 16 Apr 1964, *Malhotra NC-31432* (BSD); Najibabad, 4 Mar 1962, *Malthastra NC-19580* (BSD); Allahabad, Kaira, 18 Jul 1965, *Misra 9771* (CAL); Kheri, Motipur, 11 Mar 1964, *Panigrahi CC-2839* (CAL); Allahabad, Kuraon [Koraon], 26 Apr 1967, *Panigrahi 11246* (CAL); Bahraih, 7 Feb 1959, *Rau NC-8126* (BSD); Faizabad, R[iver] Ghagra banks, 6 Aug 1959, *Sarin NC-8573* (BSD); Bulandshahr, Aurangabad, 19 Feb 1962, *Singh NC-19452* (BSD, CAL); Dadri, 19 Apr 1963, *Singh NC-25543* (BSD, CAL); Moradabad, Jan 1844, *Thomson 62[e]* (K); **Uttarakhand**: Champowat, 27 Feb 1971, *Aroha NC-38461* (BSD); Kumaon, Dafia Dhoora, 30 Aug 1973, *Aroha NC-53202* (BSD); Nainital, Bhawali rd, 10 Jun 1958, *Arora 1437* (CAL); Dehradun, Chakrata, 29 Jul 1961, *Battacharyya NC-13026* (BSD); Dehradun, Rajpur rd, 7 Jan 1961, *Bhattacharyya NC-12986* (BSD); Almora, towards market, 11 Jun 1996, *Boron NC-76339* (BSD); Dhanligonga Valley, Pithoragarh Distr., 11 Sep 1985, *Chawdhury & Singh NC-78102* (BSD); Nainital, Saria Tal, Kumaon, 3 Aug 1913, *Gill 645* (CAL); Dehradun, Sep 1930, *Krishnan s.n.* (K); Hanuman Chatti, 2 Oct 1993, *Majumdar & Singh NC-86359* (BSD); Dehradun, Sahastradhara, 17 Jan 1972, *Malhotra NC-19716* (CAL); Dehradun, Sahastradhara, 22 Jun 1962, *Malhotra NC-22715*

(BSD, CAL); Dehradun, Sahastradhara, 10 Mar 1964, *Malhotra NC-31226* (BSD, CAL); Mandal-Ana, Garhwal, 20 May 1971, *Naithani NC-43928* (BSD, CAL); Jumma, Garhwal, 13 Aug 1974, *Naithani NC-53810* (BSD); Gauchar, towards Srikot side, 19 Jun 1979, *Naithani NC-68002* (BSD); Dehradun, Nr general post office, 15 Jun 1974, *Naithani 213* (DD); Baram, G.G. valley, Pithoragarh Dist, 21 Jun 1982, *Nalhoiba NC-51540* (BSD); Corbett National Park, Kanda-Hathipani, 3 May 1971, *Pant NC-43796* (BSD, CAL); Mussoorie, Jabberkhet, Mussoorie, 6 Jun 1967, *Pradhan 126* (CAL); Dehradun, Sahasradhara [Sahastradhara], 15 Jun 1967, *Pradhan 201* (CAL); Nainital, Kumaon, Laria Kanta, 11 Oct 1957, *Rao NC-4975* (BSD); Nainital, Lamia Kantha, 11 Oct 1957, *Rao NC-4975* (BSD); Samadhura-Tejam, Kumaon Distr., 9 Jun 1954, *Rao NC-6584* (BSD); Dehradun, 2 Apr 1961, *Rau NC-14592* (BSD, CAL); Mori-Netwar, Uttarkashi Dist, 17 Sep 1995, *Singh NC-89850* (BSD); Nainital, Mynee Tal, Apr 1844, *Thomson 62[c]* (K); Dehradun, Manchigam on Icargil-Susu Rd, 5 Sep 1975, *Viswanathan 55656* (F); Dwarahat, nr guest house, 5 Oct 1975, *Wadwha NC-57375* (BSD); Dehradun, Robber's Cave, 17 Jun 1967, *Without Collector 259* (CAL); **West Bengal**: Howrah, Makardah, 16 Mar 1961, *Banerjee 30* (CAL); Howrah, Makardal, 20 Mar 1901, *Banerjee 33* (CAL); Birbhum, Nalhati, 25 Mar 1966, *Basak 101* (CAL); Birbhum, Nalhati, 25 Mar 1966, *Basak 109* (CAL); Howrah, Gestkeen, 11 Apr 1963, *Bennet 75* (CAL); Howrah, Chengail, 15 May 1963, *Bennet 267* (CAL); Darjeeling, Lloyd Botanic Garden, 20 May 1966, *Das 105* (CAL); Kolkatta[Calcutta], Keyatala, 20 Apr 1986, *Deb BG-3534* (CAL, CAL); Burdwan, Damodar bank nr Sadarghat, 7 Mar 1960, *Dulta 32* (CAL); Burdwan, Sakligarh and ajoining areas, 12 Feb 1963, *Dulta 235* (CAL); Burdwan, Bhedel, 15 Mar 1965, *Dutt 647* (CAL); Uttar Dinajpur, Raiganj WZS, 23 Feb 2002, *Ghosh CNH-30437* (CAL); Uttar Dinajpur, Raiganj WZS, Uttar Dinajpur, 28 Mar 2003, *Ghosh 30497* (CAL); Kolkatta[Calcutta], Tantishal, Nooghly, 11 Feb 1961, *Hazra 24* (CAL); North 24 Parganas, Harnabad, 18 Jan 1974, *Jirbedi Pharm-505* (CAL); Darjeeling, Birch Hill, 16 Jun 1960, *Maheshwari 4027* (CAL); Hooghly, Pargopalnagar, Jan 1967, *Malick 45* (CAL); Balligunghe College compound, Mar 1945, *Pal s.n.* (K); Darjeeling, Sukna, 10 Mar 2008, *Paul & Kumar CNH-43603* (CAL); Jalpaiguri, Medla Tower Range, 24 Feb 2009, *Ranjan & Kumar 45320* (CAL); Bankura, Kotulpur, Thana-Kotipur, 19 Apr 1965, *Sanyal 649* (CAL); Howrah, Ramarajathala, 6 Jul 1963, *Shetty 42* (CAL); Jalpaiguri, Chapramari, 21 May 1975, *Sikdar CNH-236* (CAL); Jalpaiguri, S. Bholka, 6 Dec 1975, *Sikdar CNH-4183* (CAL); Kalapathar, *Strachey & Winterbottom 1[b]* (K); Darjeeling, Meadow Bank, 3 Jul 1973, *Without Collector CBL-12261* (CAL); Kolkatta[Calcutta], Brace Bridge, 3 Dec 1983, *Without Collector ECO-14156* (CAL).

IRAN. sin. loc, Feb, *Threlfall 10* (E). **Bushehr**: sin. loc, *McDonald s.n.* (BM). **Chaharmahal and Bakhtiari**: Kouh-Cherri, Jul 1899, *Martínez de la Escalera s.n.* (MA); valle de Bazouft, May 1899, *Martínez de la Escalera s.n.* (MA). **East Azerbaijan**: Tabriz, 1927, *Gilliat-Smith 2123a* (K); Chavan Bala, c. 13 km N of Maragheh, of Kuh-e-Sahand, 10 Aug 1966, *Warren 18* (K). **Fars**: Schurab[le], Pers. med., Oct 1868, *Haussknecht s.n.* (BM); Schurah, Pers. med, Oct 1866, *Haussknecht s.n.* (K); Jahrum, 20 Mar 1940, *Koelz 14583* (W); Shiraz, Dashte-Arjan, 5 Sep 1971, *Zárgani 14236-E* (W). **Golestan**: Kharasan, Gulestan Forest, 23 Aug 1967, *Walton 186* (E). **Hamadan**: prov. Hamadan, *Sabeti 1018* (W). **Isfahan**: prov. Kashan, Ghamsar [Qamsar], Aug 1948, *Manucehri s.n.* (W); Isfahan-Kaboutar rd, S of Isfahan, 23 Sep 1960, *Sahebi 2047* (E). **Kerman**: Kuh-e Khabr, E side, 12 km S of Khabr, Khabr va Rouchun protected region, 50 km SSW of Baft, Kerman prov, 8 Jun 1977, *Assadi et al. 1690* (E); Esfandaqeh to Jiroft, 25 km W to Jiroft (Saabzvaran), 12 Jun 1977, *Assadi et al. 1976* (E); Ghabrfarsakh, Kerman, 14 Jan 1940, *Koelz 14269* (US, W). **Kurdistan**: Aug Felsen am Grab d. Scheich Hadschiaband, 13 Jul 1885, *Stapf 2731* (K). **Lorestan**: Luristan, leaft bank of Kashgan Rud, above Fol-i-Khallor, 60 km W of Khorramabad, 11 Jul 1966, *Archibald 2661* (E, K); Bisheh, 50 km a Khorramabad, 14 Jul 1948, *Rechinger* & *Rechinger 5710* (W). **Mazandaran**: Golestan National Park, NW Khorosan, N Semnan, Tangerah (off the park), 13 Nov 1996, *Akhani 12246* (W). **Sistan and Baluchestan**: Balochistan, Sarbaz, Rasak, 7 Apr 1949, *Salavakan 3035* (W). **Yazd**: Yezd, Kuh Rezd above Taft, 17 Jul 1966, *Archibald 2778* (E); Ardistan, Besuk, 8 Sep 1956, *Haley 228* (BM).

IRAQ. 10 km S of Diwaniya on Samaura rd, central alluvial plain Distr., 25 Oct 1962, *Agnew* & *Barkley 1227* (E); 10 km S of Riwaniya on Samaura rd, Central Alluvial Plain Distr., 25 Oct 1962, *Agnew* & *Barkley 1228* (E); 15 km to Chumorta, 3 Nov 1976, *Al Khayat et al. NHI-46318* (K); Abu Ghraib, 15 km S of Diwaniya, Diwaniya Liwa, 25 Oct 1962, *Barkley et al. 3709* (F); Country surrounding Mosul, Dec 1927, *Campbell Thompson s.n.* (BM); Amara, 13 Apr 1918, *Evans s.n.* (E);

Masharra, by Amara, 18 Aug 1918, *Evans s.n.* (E); Tigris Plain, 7 Jul 1936, *Low 382* (BM); between Muqdadia and Baquba, in Diyala Liwa, 24 Oct 1956, *Rechinger* & *Khudairi 87* (BM, E, K, W); Shaibah, Jan 1929, *Rogers 08* (BM, K); Qaradah, Apr 1929, *Rogers 103* (BM, E); Garwa, nr Faluja, 15 Sep 1959, *Wheeler Haines s.n.* (E); Baghdad, Alwiyah, Nov 1957, *Wheeler Haines s.n.* (E); Abu Ghraib, Baghdad, 24 Oct 1954, *Wheeler Haines 12* (E). **Al-Anbar**: 5 km E of Ana, 3 Jul 1979, *Omar* & *Hamad NHI-50389* (K). **Al-Basrah**: Siba, 19 May 1976, *Hamad et al. NHI-46355* (K); [Al Basrah], 17 Aug 1934, *Memeryan, 5159* (K); Abul Khasib, 15 Feb 1978, *Thamer NHI-47404* (K). **Al-Sulaymaniyah**: Darbandikhan, Sulaimaniya liwa, 27 Nov 1964, *al Zebar* & *Jamel 9272* (W); Mt. Hauraman, 22 Jun 1961, *Rawi NHI-19809* (K). **Baghdad**: 50 km E of Baghdad on Kut Highway, Baghdad Liwa, 5 Mar 1963, *Barkley 33Ir4015* (K); Kazimain, Mesopotamia, Jan 1919, *Graham 315* (BM); Zafaraniya, 19 May 1975, *Hamid NHI-42695* (K). **Diyala**: Saadiya, 24 Sep 1976, *Al Khaisi NHI-42715* (K); Diltawa, 18 Mar 1948, *Rawi* & *Gillett NHI-10215* (K); between Khalis and Karkuk, 27 Jun 1960, *Rawi NHI-19706* (K); between Muqdadia and Baquda, in Diyala Liwa, 24 Oct 1956, *Rechinger* & *Khudairi s.n.* (MO); inter Mungdadiye et Baquba, 24 Oct 1956, *Rechinger 8060* (W). **Dohuk**: Dairalok village 16 km E Amadiya, 12 Jul 1976, *Al Dabbagh NHI-45862* (K); Aradin, 15 km W by N Amadiya, 14 Jul 1976, *Al Khaisi* & *Hamad NHI-46088* (K); 20 km from Sheikhan to Atrush, 7 Jul 1979, *Al Khaisi NHI-50505* (K); Zakho, 1 Sep 1975, *Botany staff National Herb Iraq NHI-43788* (K); 25 km from ZaKho to Kani Mosi, 2 Sep 1975, *Botany staff National Herb Iraq NHI-43803* (K); nr Dohuk, *Guest s.n.* (K); Sharanish village, 25 km NE of ZaKho, 15 Sep 1959, *Rawi et al. NHI-29033* (K). **Erbil**: between Arbil and Shaqlawa, 21 Jun 1960, *Rawi NHI-19683* (K); Pushtashan, NE of Rania lower slope of Qandil range, 17 Aug 1958, *Rawi* & *Serhang NHI-26567* (K); Sersang, 24 Aug 1954, *Wheeler Haines W-1359* (E, K). **Ninawa**: N of Majmua Thakafia Mosul, 27 Jun 1974, *Mosharraf Hassan s.n.* (E); **Nineveh**: Barojet Lamana, Mosul liwa, 28 Oct 1964, *Barkley 9042* (W). **Sulaymaniyah**: Sulaymaniyah, 23 Sep 1934, *Agha NHI-5370* (K); Penjwin, 14 Nov 1979, *Al Khaisi* & *Al Khayat NHI-51173* (K).

ISRAEL. Lake Hula, Galilee, Sep 1952, *Beach 5410* (US); Dead Sea, nr Elisha's Fountain, Maris Mortui, 29 Aug 1907, *Dinsmore 1151* (E); Jerusalem, 5 Sep 1904, *Dinsmore 2810* (E); Latrun, 23 Sep 1911, *Dinsmore 5171* (E); Kiryath-Anavim, nr Jerusalem, 16 Oct 1933, *Duvdevani* & *Bumstein 284* (BM, E, K, MA, W); sin. loc, *Grierson 253* (K); Golan Heights (Occupied Territory), Yehovida, 14 Apr 2016, *Knapp 10785* (BM); Ain Zahala, Sep 1911, *Letchworth s.n.* (K); Askelon, 11 Nov 1963, *Maitland s.n.* (K); Jaffa, Jul 1902, *Meyers 102* (E, F); Mt. Carmel, 2 Mar 1912, *Meyers* & *Dinsmore 102c* (E); Jerusalem, 11 Jul 1903, *Meyers 809* (E, F); Jaffa, 5 Sep 1911, *Meyers* & *Dinsmore 2809* (E); Capernaum, 11 Nov 1910, *Meyers* & *Dinsmore 3151* (E, K); Jerusalem, 15 Aug 1911, *Meyers* & *Dinsmore 5151* (E); Jerusalem, 15 Aug 1911, *Meyers* & *Dinsmore 6102* (E, MA); by the River Jordan, 21 Mar 1985, *Townsend 85/4* (K).

ITALY. Ex rupibus calcareis Liguriæ juxta nizza maritima, 1 Oct 1853, *Ball s.n.* (E); An wüsten Plätzen Laurana, 9 Oct 1833, *Hübl s.n.* (BM); Lucca, *Puccinelli s.n.* (BM); sin. loc., *Tenore 152* (E). **Calabria**: Aspromonte, c. 30 km S of Reggio, Leonardo, 10 May 1979, *Davis* & *Sutton, 62812* (BM, E). **Campania**: Salerno, Pontone, 9 Oct 1909, *Herb. Lacaita 11416* (BM); Salerno, Pontone, 9 Oct 1909, *Herb. Lacaita 11417* (BM). **Emilia Romagna**: Parma, *Without Collector s.n.* (BM). **Friuli Venezia Giulia**: Trieste, Santa Croce, 18 Sep 1953, *Neumann s.n.* (W). **Lazio**: Mte Circeo, prope pagum San Felice, Nov 1917, *Béguinot s.n.* (BM); monte Circeo, prope pagum San Felice, Nov 1917, *Béguinot 23626* (BM); Latina, Monte Circeo, Nov 1917, *Béguinot 2521* (K); Rome, 18 Apr 1901, *Neilsen s.n.* (E). **Sardinia**: 1 km NE of Argentieras, 10 Apr 1973, *Humphries* & *Richardson 206* (BM); 5 km SW Siniscola, Steilflanken des Monte Albo, 19 Apr 1963, *Malicky s.n.* (W); prope Laconi, 1827, *Muller s.n.* (W); in agris humidis prope Laconi Sardiniae, Aug 1827, *Muller s.n.* (K). **Sicily**: Catania, 10 km NE Taormina, Cape San Alessio, by side of minor rd to Forza d'Agro, 17 Jul 1983, *Akeroyd et al. 3583* (BM, E, H); Trapani, Pantalleria, May 1997, *Albert* & *Watzka s.n.* (W); Isola di Levanzo, 23 Aug 1964, *Davis 40176* (E); Palermo, proximidades de Esnelo, 4 Jun 2000, *García 1417* (MO); Palermo, prope Ficarazzi, 8 Apr 1855, *Huet du Pavillon* & *Huet du Pavillon s.n.* (K); Palmero, 15 Sep 2013, *Knapp 10677* (BM); Ragusa, 1898, *Makowsky s.n.* (W); Mt. Etna, 24 Jun 1874, *Strobl s.n.* (W); Palermo, Aug, *Todaro 874* (BM). **Toscana**: Abetone, 21 Jul 1904, *Neilsen s.n.* (E); prope Pisa, 28 Nov 1862, *Without Collector s.n.* (E). **Trentino Alto-Adige**: Bozen und vorzuglien, um Salen, *von Hausmann 44* (W); Bolzano, Botzen, *von Hausmann 183* (E); Botzen, Sud Tyrol, Salurn, 1838, *von Hausmann 274* (BM); Nago, above Torbole, N end of Lago di Garda, 16

Aug 1977, *Lippert 16053* (W). **Veneto**: Vincenza, Bassano del Grappo, Ex calcareis montanis Venetis juxta Bassano, 15 Oct 1854, *Ball s.n.* (E); Venetia, Patavium, 25 Oct 1919, *Béguinot 2520* (K); Padova, 20 Oct 1919, *Béguinot s.n.* (BM); Padova, in R. Horto Botanico, Jul 1920, *Béguinot s.n.* (BM); Padova, 25 Oct 1919, *Béguinot s.n.* (BM); Padova, 20 Oct 1919, *Béguinot 23618* (BM); Padova, Jul 1920, *Béguinot 23624* (BM); Padova, in R. Horto Botanico, Jul 1920, *Béguinot 23625* (BM).

JORDAN. 30 km SW Rum Police Station, along hte cist to Aqaba, 23 Mar 1975, *Boulos et al. 7563* (E, K); Byn-Musa, 26 Oct 1936, *Dinsmore 12810* (E). **Amman**: Thibham-Umm Al Rasas, 24 Jun 2002, *Abu-Laila et al. 70-2* (K); Zerka River, Dead Sea, E side, 5 Oct 1931, *Gabrielith 24* (K); Wadi Zarqa, above Desi Alla, Gilead, 5 Mar 1953, *Simpson 53043* (K). **Aqaba**: Wadi Rum, Hang, Mar 1992, *Albert s.n.* (W); Wadi Malagan, c. 10 km ENE Aqaba, 30 Mar 2005, *Albert 721* (W); Wadi Rum, 13 Apr 1945, *Dean 8987* (K); Wadi Rum, 2 km N of fort, 6 May 1963, *Gillett 16081* (K). **Jerash**: Jerash, Apr 1963, *Maitland s.n.* (K). **Ma'an**: Petra, Mar 1990, *Albert s.n.* (W); Ma'an, Petra, Bayda, Wadi Mu-aqsra ash Sharqiyya, 23 Apr 1998, *Albert 286* (W); Petra, 10 Apr 1935, *Dinsmore 10810* (K); Petra, 28 Dec 1935, *Dinsmore 11151* (K); Petra, 11 Mar 1967, *Hepper 3109* (K); **Madaba**: Totes Meer Wadibett des Wadi Mujib, Apr 1995, *Albert s.n.* (W). **Tafilah**: Wadi el Hesa, Talsohle an Strasse zwischen Mazar und Tafila, 22 Mar 2005, *Albert 973* (W). **Zarqa**: Rusayfah, 17 Apr 1936, *Dinsmore 12102* (E, K).

KAZAKHSTAN. **Almaty**: Zailiiskii Altai, Semirech Oblast, Vernenskii Okrug, 7 Aug 1919, *Socalski 493* (BM, E).

KENYA. 30 Oct 1894, *Donaldson Smith 218* (BM); Tsavo East National Park, Thabangunji, nr pump house, 19 Jan 1966, *Hucks 627* (EA); Kikuyu, 1889, *Jackson s.n.* (BM). **Central**: Kiambu, Muguga, 7 Sep 1950, *Beckley 670* (EA); Kiambu, Kabete, 26 Nov 1934, *Edwards 3104* (EA, K); Kiambu, Thika, Shah Central High School Compound, 16 Apr 1967, *Faden 67/198* (EA); Nyeri, Zawadi, Estate c. 7 km on Nyeri-Kiganjo rd, Amboni River, 1 Jun 1974, *Faden et al. 74/676* (EA, K); Nyeri, Nyeri to Nanyuki, c. 40 km S of Nanyuki, 3 Apr 1975, *Hepper* & *Field 4837* (K); Machakos, Tulimani, Kanzui area, 21 Aug 1985, *Muasya 628* (EA); Machakos, Tulimani, Kanzui area, 3 Sep 1985, *Muasya 729* (EA); Kiambu, Muguga, 4 Jul 1968, *Njoroge S 4* (EA); North Nyeri, Kwanganga village, Timau, 23 Apr 2010, *Vorontsova et al. 200* (BM, EA). **Coast**: Samburu, Between Maralal and Baragoi, Maralal to Baragoi rd, 10 Nov 1978, *Hepper* & *Jaeger 6694* (EA, K, P); Taita Taveta, Ngangao Forest, 15 Aug 2000, *Kamau* & *Mwangangi 734* (EA); Taita Taveta, Mbololo Hill Forest, 8 Jun 2000, *Mbuthia et al. 406* (MO); Taita Taveta, Ngangao Forest, 15 Aug 2000, *Mbuthia* & *Mwangangi 734* (MO); Taita Taveta, 12 Dec 1961, *Polhill* & *Paulo 974* (EA, K, P). **Eastern**: Machakos, Beacon, 16 Feb 1964, *Agnew 5598* (EA); Marsabit forest, 15 Jul 1951, *Dalton 8* (K); Marsabit, Mt. Kulal, around Gatab, 24 Nov 1978, *Hepper* & *Jaeger 7052* (EA, K, P); Marsabit, 27 Jan 1961, *Polhill 12188* (EA); Kitui, Mwingi, 19 Feb 2001, *NMK 18* (K); Machakos, Katumani Experimental Farm, May 1959, *Thomas 1005* (EA). **Kilifi**: Kibarani, 1 Mar 1946, *Jeffery K480* (EA); Marafa, 25 mi NW of Malindi, 21 Nov 1961, *Polhill* & *Paulo 828* (EA, K, P). **Kwale**: Diani Forest, 11 Jul 1972, *Gillett* & *Kibuwa 19889* (EA). **Laikipia**: Rumuruti, 5 Nov 1978, *Hepper* & *Jaeger 6600* (EA, K). **Makueni**: Kiboko, 26 Oct 1981, *Ndegwa 177* (EA, MO). **Nairobi**: Nairobi, *Battiscombe 453* (EA, K); Nairobi, 27 Sep 1915, *Dowson 299* (K); Kangemi, 8 Aug 1977, *Gachathi 366* (EA); Nairobi, Kouen, 4 Jan 1950, *Hale 44* (K); Langata, 7 Feb 1970, *Ivens 2503* (EA); Nairobi, 4 May 1930, *Napier 135* (EA, K); Dandora, 24 Aug 1990, *Nyakundi 728* (EA); Thika rd House, nr termitaria, 21 Jul 1951, *Verdcourt 564* (EA, K, MO). **Narok**: Mara River, Ngerendei, 29 Mar 1961, *Glover et al. 180* (EA, K); Orengitok, 17 May 1961, *Glover et al. 1238* (EA); Morijo Loita, Entasekera, 14 Jul 1961, *Glover et al. 2252* (EA, K). **Rift Valley**: Nakuru, Mt. Margaret, Kedong, Jun 1940, *Bally 1022* (EA, K); Nandi, Kipkarren, Oct 1931, *Brodhurst-Hill 328* (EA, K); Laikipia, 75 km N of Rumuruti on Maralal rd, 14 Nov 1977, *Carter* & *Stannard 381* (K); Uasin Gishu, Eldoret, 16 May 1952, *Cooke 46* (K); Nakuru, Mt. Longonot, 26 Jul 1981, *Gilbert* & *Hedberg 6305* (EA, K); Nakuru, Gilgil, 19 Jul 1971, *Harding 22* (EA); Nakuru, Lake Nakuru National Park, 11 Dec 1972, *Hingley 168* (EA); Nakuru, Ol Longonot Estate, Naivasha Distr., 3 Jan 1962, *Kerfoot 3547* (EA, K); Samburu, Mathews Range, *Luke 14210* (K); Meru National Park, *Mathenge 64* (EA, K); West Pokot, Wei Wei, Katuw, 19 Jun 1978, *Meyerhoff 22* (K); Nakuru, Mai-Mahiu, Naivasha rd, 9 Jun 2001, *Muasya GBK003/008* (EA, K); Rift Valley, Suswa volcano, 1 Jun 1997, *Phillipson* & *Bytebier 4784* (MO); Nakuru, Lake Naivasha, 7 Oct 1965, *Polhill 139* (K); Nakuru, Mau Forest, Upper Molo, 17 Dec 1953, *Ruiru Pest Control Staff 354*

(EA); Baringo, Chemolingot, 31 Dec 1977, *Timberlake 1635* (EA); Kajiado, Ol Tukai, Amboseli National Park, 22 Jun 1987, *Young 1032* (EA). **Western**: 2nd and 3rd days out from Mumias, 8 Dec 1898, *Whyte s.n.* (K).

KUWAIT. **Al Asimah**: Kuwait City, University Campus at Khaldiya, 1 Apr 1987, *Boulos 16400* (BM); Kuwait City, KISR Experimental sites (A & B), nr Maidan Hawally, 29 Feb 1988, *Boulos* & *Armer 17155* (BM, E).

KYRGYZSTAN. Chui, River basin Alamedin, 23 Sep 2004, *Sodombekov* & *Rogova KPL00292* (MO).

LEBANON. Beka'a, 16 Oct 2001, *Breidy* & *Khairallah LEB-34* (K); Tyre, 4 Sep 1932, *Dinsmore 8810* (K); c. 4 S of Aim Ab, 26 Jun 1945, *Ingram s.n.* (BM); Mouth of the Nahr el Kelb, 5 Jun 1959, *Maitland 414* (K); Itsara, 24 Jul 1959, *Maitland 522* (K); Beirut, Syriae Littoralis, May 1875, *Post 281* (BM); Nahr el Kelb, 21 Apr 1932, *Samuelsson 1086* (K); Tripoli Blanche's garden, 20 Feb 1865, *Without Collector s.n.* (E); Beirut, 14 May 1871, *Without Collector s.n.* (E); Aabay, 23 Sep 1805, *Without Collector s.n.* (E).

LIBYA. Below Cyrene (Shahat) ruins, 26 Mar 1970, *Davis 50060* (E, K); Gargaresc garden, 1 Jun 1952, *Guichard KG/Lib/447* (BM); Libyan desert, Mut Dakleh Oasis, 1911, *Harding-King s.n.* (BM); Rashuk, 26 Apr 1909, *Harding-King s.n.* (BM); Libyan desert, 1911, *Harding-King s.n.* (BM); sin. loc, 1957, *Park 3* (K); Wadi Shira, 18 May 1958, *Park 519* (K); Tripolitania, Meshich of Tripoli, 12 Jan 1819, *Ritchie s.n.* (BM); Tripolitania, Meshich of Tripoli, 13 Jan 1819, *Ritchie s.n.* (BM); Tripolitania. Meshich of Tripoli, 12 Jan 1819, *Ritchie s.n.* (BM); sin. loc, *Sandwith 2395* (BM, K). **Tripolitania**: Bani Walid, Beni Ulid, Tripolitania Distr., 14 Aug 1962, *Keith 1032* (K); Bani Walid, Beni Ulid, Tripolitania Distr., 14 Aug 1962, *Keith 1036* (K).

MACEDONIA.Yugoslavia, 18 Aug 1986, *Hanson s.n.* (BM); South Macedonia, nr 28th Hos Harurankeui, Oct 1917, *Ramsbottom x7c* (BM). **Gevgelija**: c. 15 km NNW of Gevgelija on E 5, 8 Sep 1972, *Korhonen 1108* (H, MO).

MALAWI. Hara River bank, 15 Aug 1969, *Fitzpatrick 9* (BM). **Northern**: Karonga, 31 Jul 1952, *Williamson 53* (BM). **Southern**: Mulanje, Mlanje Distr., Mlanje Mt., 27 May 1957, *Goodier 287* (K); Nyasaland, Mt. Mlanje, Tuchila Plateau, 24 Jul 1956, *Newman* & *Whitmore 170* (BM); Mulanje, Mlanje Distr., Chembe Basin ridge, Lichulesi side, 14 Jun 1962, *Richards 16686* (K); Zomba Distr., Chuka village, Chisi island, Lake Chilwa, 10 Nov 1986, *Salubeni* & *Kwatha 4841* (MO).

MALTA. sin. loc, 1926, *Bankart s.n.* (BM); Marsa Scala, 29 Nov 1871, *Duthie s.n.* (BM). Gneyna, 25 Mar 1874, *Duthie s.n.* (BM); Gozo I[sland], 14 Apr 1973, *Hepper 4823* (K); Encita, Apr, *Reade s.n.* (BM); Balluta to Pieta, Apr, *Reade s.n.* (BM); above Gnien Ingraw, nr Mellieña, 23 Oct 1971, *Westra* & *van Rooden 179* (E); sin. loc, *Without Collector s.n.* (W).

MAURITANIA. Rosso, 31 Dec 1962, *Arvidsson 191* (K).

MONACO. Brasserie, 5 Mar 1912, *Herb. Corstorphine s.n.* (BM).

MOROCCO. Prov. Haha, prope Mogador, 29 Apr 1871, *Ball s.n.* (K); GA: Oukaimeden to Ourika valley, 18 Jul 1973, *Davis 55296* (BM, E); Alto Atlas, entre Aït-Youl y Bou Thrarar, 2 Jul 1997, *Güemes et al. 1517* (MA); Al Hocciema, 28 Aug 1965, *Heath 394* (BM, K); Sheshoua, May 1871, *Hooker s.n.* (K); montagnes au sud-ouest de la ville de Maroc, Djebel Afougueur, 12 Jul 1876, *Ibrahim s.n.* (K); Region 9 (Zerhoun); c. 25 km due NNE of Meknès rd from Moulay Idriss to Nzaia-des-Beni-Ammar, 5 Jun 1994, *Jury et al. 15047* (BM); Tmeoghgamma, Sud Ouest du Maroc, 1875, *Mardochée s.n.* (GH); Dar Rhamdour, 11 May 1913, *Pitard 1755* (K); circa Tirgidum, Aug 1825, *Salzmann s.n.* (K); Muluya, alluvions. Ulad, 14 Jun 1932, *Sennen* & *Mauricio s.n.* (BM); Bon Gamez, en route to Ait Boulli, 10 Aug 1951, *Spence S233* (E); Marrakech - Gueliz, Mar 1930, *Trethewy s.n.* (BM); Marrakech - Gueliz, Mar 1930, *Trethewy s.n.* (BM); c. 12 mi E of Tedhala, Mar 1931, *Trethewy 193* (K); Mekues, Mar 1931, *Trethewy 262* (K); Xanen, 10 Jun 1921, *Vidal López s.n.* (MA); sin. loc, 19 Mar 1926, *Vidal López 67* (MA). **Béni Mellal-Khénifra**: Atlas Mountains, Prov. of Beni Mellal, 6 Sep 1964, *Lankester 336* (BM). **Drâa-Tafilalet**: 5 mi S of Midelt, 2 Sep 1952, *Hams 10* (BM); High Altas, N of Er-Rachidia on rd to Midelt, nr Rich, Gorge du Ziz, 12 Jul 1997, *Jury et al. 17823* (BM, MA). **Fès-Meknès**: Zerhoun, N of Meknès, environs of Souk de Zegota, 23 Oct 1993, *Jury et al. 12606* (BM); Moyen Atlas, Oulad Ali, 3 Aug 1975, *Stanes 28148* (BM). **Guelmim-Oued Noun**: Oasis d'Akka, 1873, *Without Collector s.n.* (K). **Marrakech-Safi**: 87 km NE of Agadir to Imintanoute, 9 May 1982, *Davis* & *King D68139* (E); Marrakech, Sep, *Garnett 1017* (BM); Gt. Atlas

above Marrakech, nr. Oukamaden, Aug 1972, *Grace et al. 6* (E); Above Essaouira, 12 km along rd to Safi, 1 Jun 1974, *Reading University/BM Expedition 160* (BM, E); Marrakech, Mar 1930, *Trethewy 183* (K). **Oriental**: Beni Sidel, Segangan, falaises de Asladen, 17 May 1934, *Sennen* & *Mauricio s.n.* (BM); Riff Oriental, Ulat-Hadu Armar, 18 Sep 1920, *Vicioso s.n.* (MA). **Rabat-Salé-Kénitr**: Temara, 20 Apr 1983, *Lewalle 10673* (MO); **Rabat-Salé-Kénitra**: Rabat, IAV Hassan II campus, 18 Jun 2001, *Jury* & *Young 19031* (MA); Temara, El Harhoura, 10 Nov 1985, *Lewalle 11247* (BM, H, MA, MO); 14 km SE of Tetouan, cap Mazari, 9 Apr 1974, *Miller et al. 873* (MO); Rabat, May 1887, *Without Collector s.n.* (K). **Souss-Massa**: Daraâ, Anezi, Agadir-ogjgal, jbel Imzi, ladera norte, 6 Jun 2009, *Calvo et al. 3846* (MA); Agadir, Aug 1877, *Cosson s.n.* (K); SW 10 km S of Ait Melloul, 21 Mar 1969, *Davis* & *Davis D48539* (BM, E); AA, Above Tafraoute, towards pass to Ait-Baha, 30 Mar 1972, *Davis 53890* (BM, E); on rd from Igherm to Tafraoute, Tagragra, Anti Atlas Mountains, 15 Feb 2007, *Jury* & *Upson 20761* (BM); Tazerwalt, Sidi Ahmed Ou Mousse, 24 Jan 1982, *Lewalle 10085* (BM); 6 km N of Tiznit, nr El-Mader-el-Kebir, 3 Apr 1974, *Miller et al. 555* (MO); 12 km E of Tiznit, Assaka, 3 Apr 1974, *Miller et al. 592* (MO); Tadderte, 29 Jul 1955, *Newbould 83* (BM); Agadir, Dec 1985, *Nicolle s.n.* (BM); Agadir, Feb 1934, *Trethewy 83* (K); High Atlas, Sahara side, coming up from Ouauyugut, 12 Feb 1960, *Whiting* & *Richmond 35-60* (K); Tamri Distr., 40 km NW of Agadir, 25 Apr 1961, *de Wilde et al. 1839* (BM); Oumesnat, Ammeln Valley, nr Tafraoute, 4 Jul 1969, *Williamson 502* (BM); Oumesnat, Tafraoute, Ammeln Valley, 7 Aug 1969, *Williamson* & *Jones 533* (BM). **Tanger-Tetouan-Al Hoceima**: T. Beni Hosmar above Tetuan, 12 May 1972, *Alexander* & *Kupicha 126* (E, MO); Axdir, 23 Jul 1927, *Font Quer 560* (BM, MA); Axdir, 23 Jul 1927, *Font Quer 561* (BM, MA); Al Hocciema, 19 Aug 1965, *Heath 340A* (K); Tangier & Tetuan, Apr 1871, *Hooker s.n.* (K); Targuist 33 km NNW of Ketama, village on coast of El-Jebha, 25 Oct 1993, *Jury* & *Upson 12717* (BM); 20 km W of Tanger, Grottes d'Hercule, 25 Feb 1994, *Jury et al. 13276* (BM); 16 km S of Rabat, Temara-Plage, 28 Mar 1974, *Miller et al. 114* (MO); Nad-Lau, 14 Jan 1921, *Vidal López s.n.* (MA).

MOZAMBIQUE. **Caza**: Canicado, andados entre os km 17-16 de Vila Pinto Teixeiras para Combomuno via entrada ao longo do Rio Limpopo, 21 Aug 1969, *Correia & Marques 1136* (MO). **Lourenco Marques**: Maputo, Ilha de Inhaca, entre a Ponta Ponduini e a Ponta Mandane, costa leste, 10 Jun 1970, *Correia & Marques 1741* (MO). **Maputo**: Goba, 6 Dec 1942, *Mendonça 1646* (MO); **Sabie'**: Moamba, 3 Dec 1940, *Torre 2214* (EA, P).

MYANMAR (BURMA). **Ayeyarwady Region**: Myanaung, Hinzada, Feb 1903, *Mokim 1280* (CAL).

NEPAL. Dhaulagiri Zone, Mustang Distr., Ghasa - Lete - Kalopani - Koketani - Larjung - Tukuche, 29 Jul 1996, *Hoshino et al. 9666069* (E); Kali Gandaki, Tukche - Marpha - Jomson, 19 Jul 1983, *Ohba et al. 83.50528* (BM); Kaki gandaki, Jomson - Kagbeni, 21 Jul 1983, *Ohba et al. 83.50558* (A); Doyan, nr Sinikot, 5 Jun 1952, *Polunin et al. 4281* (BM); Dhaulagiri zone, Mustang Distr., Kalopani - Dhampus - Kokethanti - Larjung - Khanti - Tukuche, 29 Aug 1988, *Suzuki et al. 88-11167* (BM). **Western**: Kullinbee prov., Moyang valley, Oct 1830, *Buchanan-Hamilton s.n.* (K).

NEW ZEALAND. **South Island**: Christchurch, Christchurch, Lake Bryndwr, Christchurch, 2 Apr 1968, *Healy 68/159* (MO).

NIGER. Guelta Killou, 16 Mar 1979, *Newby ZP-51* (K).

NIGERIA. **Bauchi**: Bauchi Plateau, Jul 1930, *Lely 392* (K). **Kano**: Kano, Wudil Distr., 50 km SE of the city of Kano, Northern Nigeria, 16 Mar 1988, *Etkin 63A* (MO); edge of artificial lake Baganda, 40 mi S of Kano, 16 Jul 1972, *van Blom 176* (MO).

OMAN. W. Hajar mountains, Wadi Salahi between Tuwaybah and Al Amirah, 9 Mar 1980, *Edmondson 3311* (E); Batinah, Sohar State Farm, 18 Mar 1980, *Edmondson E3419* (E); Al Hibra, nr Nakhl, 5 Apr 1985, *Gallagher 7487/10* (E); *Jayakar 41* (BM); Arabia: Sultanate of Oman, Jabal al Akhdar, Wadi al Khamr, above Halyaman village, 17 Mar 1972, *Mandaville 3538* (BM); Wadi Sahtan, 6 Apr 1975, *Mandaville 6286* (BM); Jabal al - Akhdar and Adjacent areas, vicinity of an-Nid, 8 Apr 1975, *Mandaville 6358* (BM); Jabal al - Akhdar and Adjacent areas, Vicinity of an-Nid, 9 Apr 1975, *Mandaville 6406* (BM); Jabal al, Akhdar and adjacent areas, Al-Amra, 20 Apr 1975, *Mandaville 6673* (BM); wadi 20 km E of Tagah, 11 Dec 1984, *McLeish 425* (E); Wadi Bani Habib, northern Oman, 15 Jun 1993, *McLeish 2140* (E); Wadi Bani Kharus, northern Oman, 28 Nov 1993, *McLeish 3317* (E); Wadi Qatam, W of Sayq, 17 Oct 1984, *Miller 6632* (E, K); Waliat Kuryaat, Jebel Aswad, Wadi Serin, 19 Mar 1978, *Munton 17* (K); Salalah, 14 Feb 1973, *Parker O 88* (BM); Nizwa

Ziraat, Dept. of Agric. Farm, 10 Mar 1976, *Radcliffe-Smith 3884* (E, K); Al Khaburah, Khabura, 2 Apr 1978, *Whitcombe 183* (E); Ghashab, 14 Feb 1979, *Whitcombe 464* (E); Breik Qotait, 30 Mar 1980, *Whitcombe 815* (E); Sayq, 16 Apr 1980, *Whitcombe 873* (E). **Dhofar**: Wadi Jarsib area, Salaleh, 27 Nov 1981, *Maconochie 2990* (K); Wadi Sarfait, 28 Sep 1976, *Mandaville 6980* (BM); Bandu, 1 Jun 1993, *McLeish 2006* (E); above Ayn Razat, 27 Aug 1993, *McLeish 2257* (E); Wadi Henna, 17 Sep 1993, *McLeish 2510* (E); Jabal al Qara', Thamrait-Salalah rd c. 15 km N of Salalah, 1 Oct 1979, *Miller 2395* (E); Jabal al Qara', nr Kaftwaht, 1 Aug 1985, *Miller 7196* (E). **Musandam**: Wadi Sal al Ala, 14 Mar 1994, *McLeish 3636* (E).

PAKISTAN. sin. loc., W Himalayas, *Duthie s.n.* (K); sin. loc, *Falconer s.n.* (K); Yarkand expedition, 21 Mar 1870, *Henderson 477* (K); NW India, *Herb. Royle, s.n.* (K); in valle inter Kalam et Kolalai, Swat, 6 Jun 1965, *Rechinger 30866* (K); NW India [Pakistan?], *Royle s.n.* (K); rd to Kund, 10 Aug 1975, *Wright 47* (BM); **Azad Jammy & Kashmir**: W. Himalaya, Dachigam, Kashmir, 1960, *Heybroek s.n.* (MO); W. Himalaya, Dachigan, Kashmir, 1960, *Heybroek s.n.* (MO); **Azad Kashmir**: Rainawari, Lolah faresti, Jhelum valley, Kasmir, Jul 1907, *Kesharanandi 720* (DD); **Balochistan**: Loralai, 2 Oct 1903, *Buller REP-19191* (CAL); Quetta, Baluchistan, Queta Brewery, 1 Jun 1952, *Crookshank 98* (K); sin. loc, 1877, *Duke s.n.* (CAL); Beluchistan, Sep 1926, *Forest Office 42514* (DD); Mangli, 8 Mar 1990, *Ghafoor & Goodman 4432* (F); Kagán, 9 Jul 1899, *Ináyat Khan s.n.* (CAL); Sibi, Baluchistan, 1889, *Lace 3394* (E); on the Helmund and Seistan, 18 Jun 1903, *McMahon 47/118* (CAL); coast of Baluchistan, *Pierce s.n.* (K); coast of Baluchistan, *Pierce s.n.* (K); 2 miles from Ziarat, on way to Quetta, 10 Sep 1969, *Qaiser 154* (BM); Makran, ras Nuh prope Gwadar, ad Nagaum, 15 Apr 1965, *Rechinger 27945* (K, W); Baluchistan, Makran, Panjgur, 20 Apr 1965, *Rechinger 28191* (W); Quetta, 38 km E Gumbaz, 17 May 1965, *Rechinger 29683* (W); Quetta, Shingar (31°45'N, 69°50'E), 50 km N Fort Sandeman, 20 May 1965, *Rechinger 29885* (W); **Federally Administered Tribal Areas**: Razmak, Waziristan, N India, Aug 1927, *Herb. Lester-Garland s.n.* (K); **Khyber Pakhtunkhwa**: Hangu, Samana range, 1897, *Captain Hare s.n.* (CAL); Gahirat valley, Chitral Distr., 16 Aug 1999, *Dixon & Expedition to the Hiindu Kush 148/99* (K); Saran range, Hazara, 8 Sep 1899, *Duthie s.n.* (K); W. Himalaya Haz., 19 Sep 1869, *Duthie s.n.* (K); Hazara, Mansehra, 29 Sep 1888, *Duthie 7535* (CAL, DD); Drad, 26 Sep 1848, *Hooker & Thomson s.n.* (K); Hazara, Malkawir, Kagan Hazara, 15 May 1896, *Ináyat Khan 19969* (DD); Chitral, Jarkhun-Tal bei Reshun, 11 Sep 1935, *Kerstan 2016* (W); Khyber Pass, (Peshawar Prov.) Peshawar to Torkham, 29 May 1965, *Lamond 1611* (E); NW Pakistan, Swat, Himalaya bor, W Ushu, 23 Aug 1962, *Rechinger 19498* (MO, W);Peshawar, in jugo Landi Kotal (Khyber Pass), 29 May 1965, *Rechinger 30305* (W); Chitral, Shishi boh, NE of Drosh, 16 Aug 1958, *Stainton 3142* (BM, E); nr Drosh (Chitral Relief Expedition), 1895, *Surgeon Major Hamilton 17900* (CAL, DD); **Punjab**: sin. loc., *Drummond 25867* (K); Multan, *Edgeworth 7009* (K); Kandan, 1888, *Without Collector 128* (CAL); **Sindh**: Sin, 30 Jan 1897, *Lace 4034* (CAL); Sind, inter Amri und Laki, ad ripas Indus fluminis, 4 May 1965, *Rechinger 28669* (W).

PALESTINE. Ramallah, 19 Jun 1902, *American Colony Jerusalem, 102* (K); Wadi Qelt, Dead Sea, Wad-el-Kelt, 24 Nov 1911, *Dinsmore 9151* (K); North Palestine, Mar 1867, *Fox s.n.* (K); Wadi Qelt, 6 Oct 1928, *Gabrielith 121* (K); Wadi Kelt, 1945, *Hardy s.n.* (BM); Sinai & So. Palestine, Wady Ghurundel, Nov 1883, *Hart s.n.* (BM); Dead Sea, Ghor es Safieh, Nov 1883, *Hart s.n.* (K); Lake Hula, 23 Aug 1935, *Jones 48* (BM); Mount Sion, Jun 1889, *Jouannet-Marie s.n.* (W); Kulonieh, American Colony, Jerusalem, 22 Jul 1911, *Meyers 4810* (K); Ramallah, 19 Jun 1902, *Meyers 102 a* (F); South Palestine, Askalon, Aug 1917, *White s.n.* (BM); Deqema, 7 Apr 1945, *Without Collector s.n.* (BM). **West Bank**: Jericho, 28 Mar 1905, *Dinsmore 907* (E).

POLAND. **Greater Poland**: Poznan, pies de Torun, 27 Sep 1891, *Froelich s.n.* (BM, E). **Lower Silesia**: Wroclaw, Breslau, an der Hundsfelder Bruecke, 18 Apr 1894, *Baenitz s.n.* (E);

PORTUGAL. 1918, *Herb. Monteiro de Carvalho s.n.* (BM); sin. loc, *Valorado s.n.* (BM); sin. loc, *Valorado s.n.* (BM). **Alentejo**: 1.75 km (straight line) W of village of NS de Guadalupe, 25 Mar 2010, *Nee 57020* (MO); Beja, Jun 1885, *Daveau 2446* (BM). **Azores**: Pico, ESE von Madelena, von Furma de Frei Matias, bei Dois Caminhos, 2 Jun 1999, *Vitek 99-269* (MA, W). **Centro**: Arredores de Coimbra, Sep 1884, *Lobo Miranda 2444* (BM). **Lisboa**: Cintra, 1928, *Wilmott s.n.* (BM); muralhas do Castello de S, Jorge, Nov 1883, *Zuqte d'Oliveira Simões s.n.* (BM). **Madeira**: Ponta de São Lourenço, Ilhao do Desembarcadoiro, 24 Mar 2007, *Castroviejo et al. 18239* (MA); mouth of Grand Curral, Jul 1862, *Clarke 1827* (K); Monte, 11 Sep 1961, *Coleridge 55* (BM); Funchal, 29 Apr 1861, *Fraser s.n.* (E); between Camacha and Vale de Paraiso on EN102, 23 Aug 1981, *Hampshire 417* (BM); the Mt in

Mr. Grodon's garden, 10 Dec 1832, *Lowe 722[a]* (BM, K); Solanum villosum La var. velutina Lowe, 10 Jun 1828, *Lowe 722[b]* (K); Ri do Vasco Gil, nr FUnchal, Mar 1859, *Mason s.n.* (BM); sin. loc, 1857, *Mason s.n.* (BM); Funchal, Sep 1892, *Murray s.n.* (BM); Levada da Roda, between Boa Morte and Ribeira da Quinta Grande, 19 Mar 1984, *Press* & *Short 169* (BM); Porto Santo, Ilhèu de Cima, between Porta and the Farol, 10 Apr 1986, *Press* & *Short 1166* (BM); Porto Santo, Pico Branco, Faja Pequena, 14 Apr 1986, *Press* & *Short 1285* (BM); Seixal, Chão da Ribeira, Ribeira da Janela, 27 Jun 2000, *Vargas 304* (MA); Encomienda, levada norte, 25 Jun 2000, *Velayos 9695* (MA); Isla Deserta Grande, subida a la meseta, 23 Mar 2007, *Velayos et al. 10916* (MA); zentrales Gebirge, Weg vom Forsthaus beim Pico da Malhada zum Encumeada-Pass, W des Pico Grande, 9 Apr 1997, *Vitek 97-25* (W); Funchal, Sep 1892, *Without Collector s.n.* (BM); Funchal, Ex herb Moriz., Co R. Murray, Naturalised (Lowe), Sep 1892, *Without Collector s.n.* (K). **Norte**: Porto, 1891, *Buchtien s.n.* (MO).

QATAR. Sheikh Khalifa Ibn Ali Al Thani Garden, Doha, 4 Apr 1977, *Boulos 11195* (E). **Al Rayyan**: Dukhan rd sewage pond, 18 Jan 1979, *Batanouy 2281* (K).

ROMANIA. Trans-silvan, Aug, *Schur s.n.* (W); Moldova, Distr. Iasi, in ruderatis oppodi Iasi, 21 Oct 1945, *Topa 2684* (W); Transylvania, Hermanstadt, Lichenbürg, *Without Collector s.n.* (E).

RUSSIAN FEDERATION. **Southern Federal Distr.**: Krasnodar Krai, Distr. Lazarevskoie, in faucibus rivi Dagomys Zapadnyi supra vicum Tretia Rota, 25 Jun 1979, *Vašák s.n.* (W).

SAINT HELENA. *Burchell 78* (K); Cleugh's Plain, 3 Mar 1983, *Cronk 346* (E); Diddly Dight, nr. Prosperous Bay Plain, 1 Sep 1986, *Cronk 488* (E).

SAUDI ARABIA. Riyadh, Mar 1973, *Chaudhary, s.n.* (E); S of Taif nr Wadi Liyyah, 1972, *Collenette 72-146* (K); c. 1/3 of way up escarpment between Jeddah and Taif, 17 Jan 1980, *Collenette 1542* (K); Wadi Kharat, nr the foot of the Taif escarpment, 22 Jan 1980, *Collenette 1592* (E); Wadi Al Ues, base of Jebal Sawdah, 14 May 1981, *Collenette 2696* (E, K); c. 3/4 of way up the escarpment on Jeddah-Taif rd, 15 Nov 1981, *Collenette 3133* (E); nr Jabal Manfah, Abha-Najran rd, nr police post, 21 Feb 1982, *Collenette 3309* (E); Wadi Al Ues, NW of Abha, 2 km below police post, 24 Feb 1982, *Collenette 3332* (E, K); N of Shigri, Taluk rd, 20 Apr 1983, *Collenette 4349* (E, K); Wadi Thenia, 6 Jul 1976, *Dwyer* & *Sheik 13193* (MO); Area of town water station Beesha, 7 Jul 1976, *Dwyer 13147* (MO); Waterfall at Albaha, 30 Jul 1976, *Dwyer 13811* (MO); Al-Baha region, c. 250 km S of Taif, 19 Feb 1987, *Fayed 1295* (E); Bani Malek - Southern Hejaz, 23 Feb 1981, *Grainger 67* (E); In Arabia felici, 1843, *Hohenacker s.n.* (E); Abha area, 8 Sep 1950, *Kercher 137* (BM); Central Prov., al-Majma ah, 28 Feb 1974, *Mandaville 3986* (BM); Eastern Prov., 2 km NW of Dhahran, 12 Apr 1979, *Mandaville 7414* (BM); Subha, Abha City, Ashir pref, 20 Nov 1999, *Miyazaki 99-1120 II/5* (E); Junaina (Bisha), 25 Mar 1932, *Philby s.n.* (BM); nr Hail, 20 May 1972, *Popov GP 72 31* (BM); sin. loc., *Schimper s.n.* (E); Beesha, 6 Jul 1976, *Sheik* & *Dwyer 13176* (MO); Khamis Mshate, 10 Jul 1976, *Sheik* & *Dwyer 13329* (MO); Nr Jibub, 15 Jun 1977, *Turner, DT 113 /77(2)* (BM); Taif highlands, 4 May 1947, *Vesey-Fitzgerald 17068 /3* (BM). **Asir**: 22 km SW of Abha, 8 km NE of Al-Qara'a National Park, 23 May 1980, *Boulos* & *Ads 14223* (K); 22 km SW of Abha, 8 km NE of Al-Qara'a National Park, 23 May 1980, *Boulos* & *Ads 14225* (K); Jabal Sawda, 25 km NWof Abha, 13 May 1981, *Collenette 2675* (E, K); Abha, in the University compound, 22 Feb 1982, *Collenette 3313* (E, K); 15 km NW of Abha in Soudah mountains, 16 Nov 1978, *Fagerström 62* (S); Dalaghan, 33 km SE Abha, 6 Mar 1980, *Nasher IH 126* (E); Ar-Raida escarp, c. 20 km N of Abha, 8 May 1985, *Nasher 26/85* (E); Jabal Sawda, N of Abha, 6 May 1982, *Podzorski 1198* (E); N of Abha, Suda, 4 Jul 1962, *Popov 187 34* (BM); N of Abha, Suda, 4 Jul 1962, *Popov 187 19* (BM); Above Abha, Jun 1946, *Thesiger s.n.* (BM). **Bahah**: Az Zafir, southern Hejaz, 6 Feb 1971, *Wood 71-27* (BM). **Madinah**: Birkah, 22 Mar 1969, *Popov 69.131* (BM). **Makkah**: Al Hadda, nr Taif, 3 Feb 1980, *Collenette 1730* (E, K); Taif, Apr 1950, *Simonds, 32* (K); Taif, 5 Jan 1950, *Trott 1283* (K). **Najran**: Al Mansah, 8 km SW of Abha, 20 May 1980, *Boulos* & *Ads 14102* (K). **Riyadh**: Riyadh, 24 Oct 1937, *Dickson 387* (K); Riyadh, Oct 1939, *Trott 90* (K).

SERBIA. Krivošije, Jul 1906, *Schneider s.n.* (W).

SLOVAKIA. **Košice**: Košice, South, Kukucinova str, N side, c. 30 m E of crossroad with Žižkova str, 1 Nov 2010, *Mikoláš 2541* (W); South, Jantarova str., 29 Aug 2006, *Mikoláš 3928* (W); Alvinczyho str., 20 m W of railway passage, S side, 5 Aug 2008, *Mikoláš 6304* (W); NW, Str. Nemcovej/Letná str. (NW), 22 Aug 2008, *Mikoláš 6359* (W); Košice, NW Memcovej str., at later bus stop op to Technical University, 22 Aug 2008, *Mikoláš 6369* (W); Košice, South, 6 Oct 2008, *Mikoláš 7887* (W); South, Rázusova, NE part of str, 10 Oct 2008, *Mikoláš 8082* (W); South, Palachého str., E

of square Osloboditel'ov, S side of str., edge of later little park, 3 Nov 2008, *Mikoláš 8530* (W); South, Palakehó str., 10 Nov 2008, *Mikoláš 8588* (W); Košice, Skladná 35, N side of str, 11 Nov 2008, *Mikoláš 8619* (W).

SLOVENIA. Carniolia, Prope pagum Vreme in Valle Reka, *Paulin s.n.* (BM).

SOMALIA. Boramo Distr. Brit. Somaliland. Durkaiyagulk, 5 Jan 1945, *Glover* & *Gilliland 494* (BM, EA, K); Somali-land, 1885, *James* & *Thrupp s.n.* (K). **Gobolka Mudug**: Dagah Yado, Daganyado, 27 Jan 1945, *Glover* & *Gilliland 615* (BM, EA, K). **Saaxil**: Sheikh, May 1972, *Wood S/72-84* (K). **Sanaag**: Sugli, Al Hills, 10 Nov 1929, *Collenette 239* (K). **Woqooyi Galbeed**: Hargeisa, Jul 1927, *Burne 86* (K); eastern Al Madu Range, N of Agasur, Sherlani Tug, 12 Oct 1956, *Bally B 11056* (EA, K); Bondary, 2 Oct 1932, *Gillett 4126* (K); Buramo, 3 Feb 1933, *Gillett 4923* (K); Hargeisa, Oct 1961, *Hemming 2226* (EA, K).

SPAIN. Los Barrios, 10 Jan 1956, *DBL 373* (BM); Algeciras, Jul 1924, *Hubbard s.n.* (BM); in cultis regionis caldaes v.c. prope monasterium los Angeles agri Malacitani, 20 Nov 1844, *Willkomm 637* (K). **Andalucia**: Caseres, 26 Mar 1970, *Allen 6505* (BM); in Roque, 17 Jan 1970, *Brinton-Lee 1002* (BM); Cádiz, Grazalema, 7 km S of Zahara, 16 Jul 1981, *Gardner & Gardner 1252* (BM, E); Sevilla, between Moron de La Frontera and Pruna, 23 Apr 1969, *Gibbs et al. 1236.69* (BM); Málaga, Benahavis, 28 Apr 1973, *Jermy 9805* (BM); Málaga, Chorro Schlucht c. 40 km NW der Ortschaft Málaga, and der MA-5403 (bei Strassen km 9), 30 Oct 2011, *Karl s.n.* (W); Tarifa, 11 Apr 2015, *Knapp 10780* (BM); Montes de Málaga, 14 Apr 1980, *Reading MSc Expedition, 227* (BM); Sierra de Mijas, 12 Jun 1888, *Reverchon s.n.* (E); Almeria, Sierra de Almahilla, cima, 27 Apr 1997, *Sequeira 2790* (MA); Granada, nr the Alhambra, garden adjoining Miss Laird's pension, 9 Jun 1926, *Wilmott* & *Lofthouse s.n.* (BM). **Asturias**: Playa de Peñarronda, 27 Aug 1998, *Vitek* & *Dobeš 98-1622* (W). **Baleares**: Ibiza Santa Eulalia del Rio, 14 Jan 1971, *Ferguson 2731* (BM); Mallorca Arta, in Torrent Sa Perada Valley, 9 Feb 1971, *Ferguson & Ferguson 2884* (BM); Mallorca Andratx, 12 Feb 1971, *Ferguson & Ferguson 2927* (BM); Mallorca, Soller, 5 May 1903, *White s.n.* (BM); Mallorca, Sóller, Majorca, 5 May 1903, *White s.n.* (E); Menorca, circa Canisia, San Cristobal et Alazor , 23 Apr 1885, *Porta* & *Frigs s.n.* (BM, E). **Canary Islands**: Tenerife, San Diego del Monte, 21 Jan 1845, *Bourgeau 557* (BM, E, W); La Palma, Insul. Palma, Barranco de la Nieves, 14 Aug 1845, *Bourgeau 934* (BM); Sin. loc., 1801, *Broussonet s.n.* (BM); Gran Canaria, Tejada, 20 Apr 1936, *Brooke 122* (BM); Gran Canaria, *Despréaux Webb-44* (W); Tenerife, Barranco de Torres, 6 km de Carolina, 7 Apr 1880, *Martínez 95* (W); La Palma, SW Kuste, zw. Puerto Naos y El Remo, 18 Feb 2000, *Royl 6826* (B); Gran Canaria, below Vallesco, 19 Apr 1973, *Stearn 1145* (A); Gran Canaria, below Valle Seco, 19 Apr 1973, *Stearn 1174* (BM); Tenerife, Barranco de Ruiz, 5 Jul 1896, *Tullgren 95* (W); Tenerife, sin. loc, *Webb 44* (W). **Castille and León**: Castilla, San Miguel, 14 Oct 1906, *Elías 2954* (BM). **Castille-La Mancha**: Guadalajara, Cercadillo, junto al río Cercadillo, 29 Oct 1987, *Izuzquiza* & *Sánchez 1082AI* (MO). **Catalunya**: Barcelona, Llobregat, 7 May 1919, *Sennen s.n.* (BM); Barcelona, chemins aGervasio, 9 Mar 1913, *Sennen s.n.* (BM); Barcelona, 26 Sep 1913, *Sennen s.n.* (BM); Barcelona, vers Horta, Nov 1918, *Sennen s.n.* (BM). **Ciudad Autónoma de Melilla**: Melilla, coteans, Linares, 9 Jun 1932, *Sennen* & *Mauricio s.n.* (BM). **Galicia**: Pontevedra gallecio, 23 Aug 1851, *Lange s.n.* (K).

SUDAN. Red Sea Hills Distr., in vicinity of Erkowit, 1 Mar 1932, *Aylmer 171* (BM, K); Red Sea Hills Distr., in vicinity of Erkowit, 2 Mar 1936, *Aylmer 517* (BM, K); Aethiopia, 1837, *Kotschy 166 [c]* (K); Aethiopia, 1837, *Kotschy 355* (K); Jebel Marra, Darfur, 1921, *Lynes 37c* (BM); E. Sudan, Erkowit, 2 Apr 1981, *Martin 3243* (BM); Red Sea Prov., 11 May 1928, *Newberry 202* (BM); Jebel Marra Mts 120 km E of Zalingei, 19 Jan 1965, *de Wilde et al. 5377* (MO). **Blue Nile**: Aethiopia, prov. Sennar, 1837, *Kotschy 184[b]* (K); Aethiopia, E regno Sennar, *Kotschy 184 [c]* (K, P); Aethiopia, 1837, *Kotschy 313* (K). **Darfur**: Nyuringya, on W side of Marra Mts, 5 Jan 1934, *Dandy 135* (BM, MO); Darfour, *Expedition Purdy, 109* (K); Jebel Marra, upper Wadi Gindi, 26 Nov 1921, *Lynes 134* (K); Qharb Darfur, Jebel Marra, Dec 1921, *Lynes 132* (K); Qharb Darfur, Jebel Marra, Mar 1930, *MacIntosh, 62* (K); Qharb Darfur, Jebel Marra, Golol, 9 Feb 1964, *Wickens 1191* (K); Qharb Darfur, Jebel Marra, Tora Tonga, 20 May 1964, *Wickens 1711* (K); Qharb Darfur, Jebel Marra, N of Crater, 6 Sep 1964, *Wickens 2391* (K). **Kassala**: Diris Pass, Red Sea Hills, 10 Apr 1953, *Jackson 2887* (K); Red Sea, Ar Kaweit, Erkowit (Jebel Seila), Kilamind, *Andrews, 3590* (K); Red Sea, 1896, *Bent s.n.* (K); Red Sea, Jebel El Sit, 2 km E of Erkowit, 18 Nov 1987, *Carter 1815* (K); Red Sea, Erkowit, Red Sea Hills, 1928, *Maffey 5* (K). **Khartoum**: Khartoum, nr Jebel Bouser forest, 8 May 1961, *Jackson 4187* (K).

SWEDEN. **Götaland**: Kalmar, Kalmar, Smalandrie, Jul 1865, *Ahlberg s.n.* (BM); Västra Götaland, Göteborg, Delsjöupplaget, 1 Oct 1950, *Blom s.n.* (BM); Västra Götaland, Göteborg, Gotebor Backa, 1938, *Blom s.n.* (E); Västra Götaland, Delsjön, 1 Oct 1950, *Blom s.n.* (E); Västra Götaland, Göteborg, Ringon, 15 Sep 1953, *Blom s.n.* (K); Västra Götaland, Göteborg, Ringon, 7 Oct 1951, *Blom s.n.* (K); Västra Götaland, Göteborg, 15 Sep 1953, *Blom s.n.* (W); Västra Götaland, Göteborg, Aug 1938, *Blom s.n.* (W); Västra Götaland, Västergötland, Nodinge Surte, 8 Oct 1950, *Blom 36* (BM, K, W); Västra Götaland, Göteborg, Sep 1938, *Blom s.n.* (MO); Västra Götaland, Nödinge, N Surte, 10 Aug 1950, *Blom 36* (MO); Skåne, Scania, Helsingborg, *Cervin s.n.* (W); Skåne, Höganäs, Aug 1887, *Johansson s.n.* (BM); Skåne, Malmö, 18 Sep 1887, *Johansson s.n.* (BM); Skåne, Höganäs, Sep 1911, *Löfvander s.n.* (BM); Skåne, Lund, Oct 1907, *Påhlman s.n.* (BM); Halland, Õrnevaha, 17 Oct 1913, *Svenson s.n.* (BM); W. Skallsjö s:n, Oskarshöjd, Aug 1895, *Thedenius s.n.* (BM).

SWITZERLAND. **Geneva**: Sin. loc., 1880, *Déséglise s.n.* (BM); a chatelaine pres de Genève, 20 Sep 1878, *Déséglise s.n.* (BM); Genève, Nov 1876, *Déséglise 457* (E). **Ticino**: ad Montem Salvatoris, Lugano, Aug 1842, *Ball s.n.* (E).

SYRIAN ARAB REPUBLIC. Jebel El-Sheikh, Buqush village, c. 40 km W of Damascus, 13 Sep 2001, *Boulos* & *Khatib 19457* (K); Unexplored Syria Coll. c. Burton, *Burton s.n.* (E); Akuba to Petra, 1 Jun 1851, *Marsh s.n.* (GOET); Baniyas, 11 Mar 1945, *Norris s.n.* (BM); Aleppo, 1769, *Without Collector s.n.* (E).

TANZANIA. **Arusha**: Ngorongoro Crater, Soda lake, 10 Sep 1966, *Gilbert E37* (EA); Lake Kasare, 10 Jul 1968, *Gilbert 2655* (EA); Arumeru, Mt. Meru, Arusha, Mt Meru, 5 Jul 1971, *Hedberg 4965* (MO); Arusha National Park, Island, Big Momela Lake, 8 Mar 1973, *Vesey-Fitzgerald 7540* (EA). **Dodoma**: Mpwapwa, 6 Dec 1930, *Hornby 350* (EA, K); Great North rd, 7 mi S of Kondoa, Kondoa Distr., 18 Jan 1962, *Polhill* & *Paulo 1207* (EA, K). **Iringa**: Njombe, Milo, 12 Oct 1978, *Archbold 2545* (K); Mufindi, Ngwazi, 8 May 1987, *Lovett* & *Lovett 2122* (MO); Mt. Image, N of Morogoro rd, 8 Mar 1962, *Polhill* & *Paulo 1686* (EA, K, P); Mufindi, Sao Hill, Mar 1959, *Watermeyer 123* (K). **Kagera**: Ngara, Ngara, Kirushya, Buguji, 23 Nov 1959, *Tanner 4532* (EA, K, MO). **Kilimanjaro**: Mwanga, Nyumba ya Mungu, on the Pangani River between Arusha Chini & same, 36 mi from Moshi, 14 Aug 1968, *Batty 274* (K); Moshi, Engare Nairobi, Block 10, and 11, W slopes of Kilimanjaro, 19 Jun 1944, *Greenway 6882* (EA, K); Mountainside Estate, 24 Sep 1993, *Grimshaw 93-728* (K); Moshi, Kilimanjaro, Mrere, Jun 1927, *Haarer 566* (EA, K); Same, Chome Forest Reserve, Bwambo Gujini-Heigande, 8 Sep 1999, *Mlangwa* & *Masanyika 482* (MA, MO); Kilimanjaro, *Thomson s.n.* (K). **Mara**: Musoma, Banagi Hills, 16 Aug 1952, *Tanner 924* (K). **Masai**: Ardai Plains, Area A, 28 Jun 1944, *Greenway 6997* (EA, K). **Mbeya**: c. 1/2 mi SE of Ilomba Local Court, 20 Feb 1963, *Harwood s.n.* (K); Mbeya, residential area, weed of cultivation, 11 May 1975, *Hepper* & *Field 5500* (EA, K); C. 17.5 km SW of Mbeya, 12 May 1956, *Milne-Redhead* & *Taylor 10186* (EA, K); Poroto Mountains, 17 May 1957, *Richards 9775B* (K). **Mbulu**: Kitingi, 25 Feb 1965, *Hukui 26* (EA, K). **Morogoro**: Bezirk Morogoro, Uluguru-Gebirge, Westseite, Lukwangule-Hochland, Gebirgssavanne uber dem Nebelwald, 22 Feb 1933, *Schlieben 3550* (B, BM). **Mpanda**: Rukwa, Sonta, 30 Oct 1963, *Richards 18304* (EA, K). **Musoma/Maswa**: Seronera, Serengeti, 30 Apr 1962, *Greenway 10635* (EA, K). **Mwanza**: Campus of Butimba Teachers College, Mwanza, 29 Apr 1967, *Humbles 5014* (MO); Inyawenda, Buhumbi, 17 May 1952, *Tanner 829* (K); Magu, T1, 9 Mar 2000, *Kayombo et al. 3225* (MO). **Pangani**: Pangani, Madonga, Dahal, 13 Jul 1956, *Tanner 3020* (EA, K, MO). **Pwani**: Bagamoyo, Matipwili Village, Zaraninge Forest Reserve, N side of railway, 10 Oct 1997, *Abeid 150* (MO); Bagamoyo, Gongo Village, T6 Zaraninge Forest Reserve, Njia Tatu, 6 Apr 1999, *Abeid et al. 533* (MO). **Shinyanga**: Shinyanga, Apr 1932, *Bax s.n.* (K). **Singida**: Iramba Plateau, Mar, *Hammond 172* (EA, K); T5, Mwange Sec. School, Singida, 5 Apr 1974, *Sabaya 34* (MO). **Tabora**: Rd between Kaliua and Urambo about the Rice field nr Urambo, 17 Jun 1980, *Hooper et al. 2014* (EA, K). **Tanga**: Steinbruch Forest Reserve, 31 Dec 1969, *Botany students 1458* (EA); Pongwe, Maweni, 27 Nov 1965, *Faulkner 3746* (K); Lushoto, Poni Usambara Mountains, 10 Jun 1974, *Faulkner 4858* (K). **Ufipa**: Rukwa, Nziga plain, Muse, 18 Mar 1959, *Richards 12233* (EA, K); Kaengesa, 24 May 1967, *Robertson 643* (K).

TUNISIA. 13 Apr 1854, *Kralik s.n.* (BM); Djebel Sidi Khalif, 15 May 1982, *Malicky s.n.* (W); 10.4 km W Matmata, Berberdorf Tamezret, 27 Apr 2001, *Walter 01/0245 A* (W). **Bizerte**: Parque

National d'Ichkeul, E, 1978, *Fay 1012* (K). **Sousse**: Sousse, 5 Jun 1883, *Cosson s.n.* (K); Kantaoui, nr Hammam-Sousse, 3 Jun 1997, *El Assi et al. 523* (K).

TURKEY. Marmara Adasi, southside, 15 Jun 1968, *Baytop 13628* (E); Pl. Anatol. Orient., 1890, *Bornmüller s.n.* (W); Prope Brousseau, Aug 1886, *Bornmüller s.n.* (W); Mont Amanus, Syriae borealis, 1906, *Haradjian 330* (K, W); Albistan, Aug 1865, *Haussknecht s.n.* (K); Thrage, Xanthie, 29 Jul 1931, *HGT 754* (K); Thrace, Gallipoli, Kika, 11 Aug 1923, *Ingoldby 553* (K); sin. loc, *Liston s.n.* (E); Mugla, 13 km S of Fethiye, 16 May 1986, *Polunin 13930* (E); Aintab, Jun 1882, *Post s.n.* (BM); Gozne, 1895, *Siehe 298* (BM, E, K, P); Polathane, Black Sea Coast, c. 20 km W of Trabazon, 25 Aug 1972, *Uotila 19873* (E, H). **Aegean Region**: Mugla, C1, Mugla, 8 km E Resadiye (W Marmaris), 11 Apr 1982, *Sorger & Buchner 82-23-50* (W). **Black Sea Region**: Amasya, Pontus Galaticus, 17 Jul 1890, *Bornmüller 1798* (BM, K, P, W); Kastamonu, Inebolu, 31 Jul 1962, *Davis D 38512* (E); Giresun, (Sandschak) Trapezunti, in ditione vici Eseli prope oppidum Goerele (Elehu), 19 Jul 1907, *Handel-Mazzetti 859* (W); Ordu, c. 20 km W of Ünye, shore of Miliç River, 26 Aug 1972, *Kukkonen 8166* (E, H); Amasya, Merzifon, *Manissadjian 981b* (K, W); Samsun, Kizilirmak, after Asar Köy, 8 Jul 1967, *Tobey 2244* (E); Trabzon, A7 Trabzon, Flughafenbereich, 31 Aug 1980, *Sorger 80-41-4* (W); Çorum, Sungurlu, 27 Aug 1972, *Uotila 19926* (E, H). **Central Anatolia**: Nigde, Göllü, zwischen Merzifoun und Amassia, 20 Aug 1892, *Manissadjian 349b* (E). **East Anatolia Region**: Kars, Aralik, Aras Valley, 21 Jul 1966, *Davis D47039* (E, K); Elazig, Lake Hazar, 28 Jul 1910, *Handel-Mazzetti 2561* (W); Bitlis, Kurdistania media, Taurus Armenius, in valle Sassum, ad vicum Goro, 12 Aug 1910, *Handel-Mazzetti 2923* (W); Bitlis, Dyarbakir-Bitlis, Hazan rd junction c. 5 km from Bitlis, 10 Aug 1956, *McNeill 496* (E, K); Kars, A10, Kars, zwischen Orta Alican u.d. russischen Grenze, 20 Aug 1984, *Sorger 84-72-100* (W). **Marmara**: Kocaeli, prov. Izmit (Kocaeli), Istanbul-Izmit (Kocaeli) rd, c. 55 km from Istanbul, 20 Jul 1956, *McNeill 225* (E, K); Istanbul, Environs de Constantinople, Oct 1840, *Thuret 12* (P). **Mediterranean Region**: Adana, Pozanti, 3 mi N on E slopes of Toros Dagi, 15 Jul 1971, *Aberdeen University Amanus Expedition C1170* (E); Adana, Pozanti, 3 mi N on E slopes opf Toros Dagi, 16 Jul 1971, *Aberdeen University Amanus Expedition D1234* (E); Osmaniye, C2 Othange, Kop, 30 Mar 1983, *Buchner 83-14-8* (W); Maras, Andirin, 8 mi S at Çatak, 17 May 1965, *Coode* & *Jones 1141* (E); Mersin, Silifke, 3 m, 14 Apr 1956, *Davis* & *Polunin 26.067* (BM, E, K); Mersin, C4 Içel, 16 km W Silifke, 9 Apr 1985, *Sorger 85-41-59* (W); Antalya, Antalya, 23 Jun 1935, *Tengwall 40* (K); Hatay, Belen Pass, C6 Amanus Mountains, 13 Apr 1966, *Watson 744* (K).

TURKMENISTAN. Kashgar, over 4000ft, *Creswell 31* (K); Aschabad, Regio transcaspica, 30 Sep 1900, *Sintenis 1126* (W); in ruinas Tiaze Nusai Aschabad, 20 Sep 1976, *Vašák s.n.* (W); montes Kopet Dag, in valle fluminis Firiuzinka, 21 Sep 1976, *Vašák s.n.* (W); Aschgabat, ad marginem boreo-orientalem oppidi Aschabad, 6 Sep 1976, *Vašák s.n.* (W).

UGANDA. **Central**: Buikwe, Bugagali Falls, River Nile, nr Jinga, Jul 1952, *Lind 82* (K). **Northern**: Karamoja, Kidepo Nat. Park, Dododh, 2 Jan 1973, *Synnott 1404* (EA); Karamoja, Kangole, Jul 1957, *Wilson 370* (EA, K); Karamoja, Mt. Moroto, Jan 1959, *Wilson 633* (EA, K). **Western**: Ntungamo, Ruizi River, 3 Apr 1951, *Jarrett 454* (EA, K).

UKRAINE. **Crimea**: Crimea, Eeodosii, 18 Aug 1909, *Sarandinaki s.n.* (LE).

UNITED ARAB EMIRATES. Arabian Peninsula, Ras al Khaima, 2 mi N of Digdaga, Jan 1973, *Tyler s.n.* (BM); Wadi Assimah, nr top, 9 Feb 1973, *Tyler s.n.* (BM). **Abu Dhabi Emirate**: 22 km N of Al-Ain, along the highway to Dubai, 28 Feb 1986, *Boulos* & *Al-Hasan 15839* (E); Al Khubairat and Al Manhal, c. 1 km from coast, Abu Dhabi Island, 2 Apr 1982, *Western 135* (E). **Fujairah**: between Khatt and Dibla, 21 May 1982, *Western 197* (E). **Sharjah**: Wadi Helo, Al-Hussein, 26 Jan 2010, *Heller et al. 289* (K); Sharjah City, close to Emirates rd, 1 Apr 2010, *Heller 340* (K).

UNITED KINGDOM. **Channel Isles**: Jersey, St. Helier, 15 Nov 1929, *Arsène s.n.* (BM); Jersey, St. Helier, 15 Nov 1929, *Arsène s.n.* (BM); Jersey, St. Helier, 15 Nov 1929, *Arsène s.n.* (BM); Jersey, St Helier's, 15 Nov 1929, *Arsène 517/3* (BM); Jersey, Jersey, 8 Sep 1917, *Hunnybun s.n.* (BM); Alderney, Braye Bay, 18 Aug 1932, *Jackson* & *Jackson s.n.* (K); Guernsey, Aug 1841, *Newbould s.n.* (BM); Jersey, St. Peter's, 9 Jul 1842, *Newbould s.n.* (BM). **England**: Greater London, Royal Botanic Gardens Kew, Aug 1877, *Baker s.n.* (BM); Kent Whitstable, Jul 1879, *Bennet s.n.* (BM); Surrey, Croydon, Jul 1879, *Bennet s.n.* (BM); Warwickshire, Milverton, Sep 1897, *Bromwich s.n.* (BM); West Yorkshire, Bradford, 23 Aug 1917, *Cryer s.n.* (BM); Greater London, Enfield, 10 Oct 1989, *Cunnell 167* (K); Oxfordshire, Sep 1886, *Druce s.n.* (BM); Greater London, Mortlake, Thameside, Surrey, 2 Oct 1815, *Groves* & *Groves s.n.* (K); nr Whitstable, Aug 1877, *Hanbury s.n.* (BM); Hertfordshire,

Ware, 30 Sep 1978, *Hanson 329* (BM); Cult. Chelsea Physic Garden, *Herb. Miller s.n.* (BM); Kent Whitstable, 1876, *Jackson s.n.* (BM); West Yorkshire, Meanwood, 1902, *Lees s.n.* (BM); Greater London, Rainham Station, South Essex, 29 Sep 1946, *Sandwith* & *Sandwith 3169a* (K); Surrey, cultivated land at Ripley, 24 Oct 1948, *Sandwith 3413* (K); Kent Whitstable, Beach Head, 15 Sep 1875, *Webb s.n.* (BM); Kent Whitstable, 15 Sep 1875, *Webb s.n.* (BM); Kent Between Whitstable & Sea-salter, 1875, *Webb & Hanbury s.n.* (BM). **Gibraltar**: Catalan Bay, 16 May 1985, *Benusan et al. 51* (MA); The Rock, along King Charles rd, 9 Sep 2009, *Christenhusz et al. 5827* (BM); WDs Distr. 1, Apr 1923, *Paterson s.n.* (BM); El Cobre, 24 Jan 1913, *Wolley-Dod 1542* (K); Middle Gate, 18 Feb 1913, *Wolley-Dod 1595* (K). **Scotland**: Royal Botanic Garden Edinburgh, 27 Jul 1899, *Without Collector s.n.* (E).

WESTERN SAHARA. Grara Talifat, 2 Jun 1946, *Agacino 164934* (MA).

YEMEN. By San`a Hodeida rd side Monaco Village 91 km from San`a, 2 Dec 1971, *Brunt 2490* (BM); km 50 from Sana'a towards Hodeidah, 21 Sep 1962, *Popov PB17* (BM); Al Huseini, N of Lahej, 25 Nov 1937, *Scott* & *Britton 269* (BM); Bau-an, 4 Sep 1975, *Wood Y/75-742* (BM). **Abyan**: 72 km W of Ahwar, along the coastal rd, 15 Mar 1988, *Boulos et al. 17234* (K). **Al Mahwit**: Mandib nr Khamis Beni Sa'ad, 25 Mar 1978, *Wood 2293* (BM); **Amran**: Amran, 4 Oct 1982, *Müller-Hohenstein* & *Deil 544* (E). **Dhamar**: 75 km S of Sanaa on Dhamar rd, nr Mabar Well, 13 Oct 1975, *Hepper 5628* (K). **Hadhramaut**: Shiban 40 km NE of Sanaa, 17 Oct 1975, *Hepper 6311* (K). **Ibb**: 10 km SW of Yerim, 13 Oct 1975, *Hepper 5649 A* (K); Jibla hospital grounds, 27 Oct 1975, *Hepper 6132* (K); Jabal Raymah, Al Jabin, 20 Mar 1984, *Miller* & *King 5292* (E); Ibb Governate, 1.5 mi W of Ibb nr Ibb Secondary Agricultural Institute farm, 12 Jul 1983, *Spellenberg 7258* (K); c. 3 airmi E of Ibb at the pass over Bahdain Mtn, 14 Jul 1983, *Spellenberg 7328* (K). **Sana'a**: N Yemen, along path above Jebel Dawran, 4 Oct 1979, *Gillespie 25* (K); Bani Matar range c. 20 km W of Sanaa on Hodeida rd, 12 Oct 1975, *Hepper 5614* (K); Sanaa, 6 Nov 1975, *Hepper 6285* (K); Central Highlands. Jabal Hadri, 30 May 1972, *Hugh* & *Leach 72/28* (BM); Sana'a to Menacha rd, c. 2 km E of Menacha, 26 Nov 1982, *King 308* (E); Jabal Hadhur an Nabi Shwayib, 13 Sep 1977, *Lavranos* & *Newton 15827* (E, MO); at entrance to Wadi Dhahr, 13 km NNE of Sanaa, 17 Sep 1977, *Lavranos* & *Newton 15875* (E, MO); Jabal an Nabi Shu'ayb, S ridge to summit, Jebal Nabi Schwaib, 20 Sep 1978, *Miller 147* (E, K); Jabel Shibam, above Menacha, 5 Oct 1978, *Miller 374* (E, K); 15 km NE of Sana'a, 10 Sep 1984, *Rex Smith 9* (E); Field of lucerne at Beit-el-Ghofr, N of Haz, 4 Feb 1938, *Scott* & *Britton 491* (BM); 10 Oct 1974, *Wood Y/74-73* (BM); c km 22 between Sanáa and Wálan, 14 Dec 1917, *Wood 2157* (BM, K); betweeb Kankaban and Shibam, 23 Jan 1978, *Wood 2193* (BM, K); between Menackla and Maghrata, 29 Jan 1978, *Wood 2206* (BM, K); **Socotra**: Soqotra, *Hay s.n.* (E); Wadi Deneghan 7 km SE of Hadiboh, 19 Feb 1989, *Miller et al. 8248* (E); Soqotra, 28 Jul 1899, *Without Collector s.n.* (E). **Ta'izz**: Djennat, Nov 1837, *Botta s.n. [30D]* (P); Jebel Sabir above Taiz, 21 Oct 1975, *Hepper 5930* (K); 10 k W. of Taiz on Mocha rd, 14 Sep 1978, *Miller 42* (E); Jebel Sabir, Tita'bad, 15 Nov 1974, *Wood Y/74-265* (BM); 14 Sep 1975, *Wood Y/75-757* (BM).

ZAMBIA. **Central**: Kabwe, 21 Apr 1998, *Leteinturier et al. 145* (K). **Northern**: Prov. N. Rhodesia. Distr. Barotseland. Shangombo, 8 Aug 1952, *Codd 7443* (BM, K); N. Rhodesia. Abercorn, Apr 1954, *Nash 68* (BM); Katema Island, 4 mi N of Kampinda Harbour, Mweru-wa-Ntipa, 25 Jul 1962, *Tyrer 110* (BM).

ZIMBABWE. **Harare**: Distr. Marandellas, Lark Hill Farm, 20 Jan 1933, *Rattray 560* (BM).
